# Serum Metabolite Profile in Progressive Versus Nonprogressive Alcohol‐Related Liver Disease: A Cross‐Sectional Metabolomics Study

**DOI:** 10.1111/liv.70128

**Published:** 2025-05-13

**Authors:** Eemeli Puhakka, Hany Ahmed, Retu Haikonen, Sophie Leclercq, Kati Hanhineva, Luca Maccioni, Camille Amadieu, Marko Lehtonen, Ville Männistö, Jaana Rysä, Peter Stärkel, Olli Kärkkäinen

**Affiliations:** ^1^ School of Pharmacy University of Eastern Finland Kuopio Finland; ^2^ Food Sciences Unit, Department of Life Technologies University of Turku Turku Finland; ^3^ Institute of Public Health and Clinical Nutrition University of Eastern Finland Kuopio Finland; ^4^ Laboratory of Nutritional Psychiatry, Institute of Neuroscience, UCLouvain Université Catholique de Louvain Brussels Belgium; ^5^ National Institute of Alcohol Abuse and Alcoholism Bethesda Maryland USA; ^6^ Université de Bordeaux, NutriNeuro Bordeaux France; ^7^ Institute of Clinical Medicine University of Eastern Finland Kuopio Finland; ^8^ Department of Medicine Kuopio University Hospital Kuopio Finland; ^9^ Department of Hepato‐Gastro‐Enterology Cliniques Universitaires Saint Luc Brussels Belgium; ^10^ Laboratory of Hepato‐Gastroenterology, Institute de Recherche Expérimentale et Clinique Université Catholique de Louvain Brussels Belgium

**Keywords:** bile acids, fibrosis, glutamatic acid, metabolomics, steatosis

## Abstract

**Background and Aims:**

Alcohol‐related liver disease (ALD) is a major cause of mortality and disability‐adjusted life years. It is not fully understood why a small proportion of patients develop progressive forms of ALD (e.g., fibrosis and cirrhosis). Differences in the metabolic processes could be behind the individual progression of ALD. Our aim was to examine differences in serum metabolome between patients with nonprogressive ALD and patients with an early form of progressive ALD.

**Methods:**

The study had three study groups: progressive ALD (alcohol‐related steatohepatitis or early‐stage fibrosis, *n* = 50), nonprogressive ALD (simple steatosis, *n* = 50) and healthy controls (*n* = 32). Both ALD groups took part in a voluntary alcohol rehabilitation programme. A nontargeted metabolomics analysis and targeted analysis of short‐chain fatty acids were done to the serum samples taken on the day of admission.

**Results:**

We found 111 significantly (*p* < 0.0005) altered identified metabolites between the study groups. Our main finding was that levels of glycine‐conjugated bile acids (Cohen's *d* = 0.90–0.91), glutamic acid (*d* = 1.01), 7‐methylguanine (*d* = 0.77) and several phosphatidylcholines (*d* = 0.61–0.85) were elevated in the progressive ALD group in comparison to the nonprogressive ALD group. Glycine‐conjugated bile acids, glutamic acid and 7‐methylguanine also positively correlated with increased levels of aspartate aminotransferase, alanine aminotransferase, gamma‐glutamyl transferase, cell death biomarker M65 and liver stiffness.

**Conclusions:**

Our results indicate that the enterohepatic cycle of glycine‐conjugated bile acids, as well as lipid and energy metabolism, is altered in early forms of progressive ALD. These metabolic processes could be a target for preventing the progression of ALD.

**Abbreviations**
5‐AVAB: 5‐aminovaleric acid betaine
ALD: alcohol‐related liver disease
ALT: alanine aminotransferase
ANOVA: analysis of variance
AST: aspartate aminotransferase
AUD: alcohol use disorder
CAP: controlled attenuation parameter
CAR: acylcarnitine
CYP7A1: 7α‐hydroxylase
ESI: electrospray ionisation
FA: fatty acid
FDR: false discovery rate
FXR: farnesoid X‐receptor
GGT: gammaglutamyltransferase
HCC: hepatocellular carcinoma
HILIC: hydrophilic interactions liquid chromatography
LC–MS: liquid chromatography mass spectrometry
LPC: lysophosphatidylcholines
LPE: lysophosphatidylethanolamine
M65: cell death biomarker
PC: phosphatidylcholine
PCA: principal component analysis
PGRPs: peptidoglycan‐recognition proteins
PLS‐DA: partial least squares discriminant analysis
RP: reverse phase
sCD14: blood‐soluble CD14
SCFA: short‐chain fatty acid
SM: sphingomyelin
SPME‐GC–MS: solid‐phase microextraction coupled to gas chromatography and mass spectrometry
UPLC: ultra‐high performance liquid chromatography
VIP: variable importance in projection
WHO: World Health Organization

**Summary**

- Alcohol‐related liver disease has different stages and the development of the progressive and more serious forms of the disease varies from person to person at similar alcohol consumption levels.
- In this study, it was shown that bile acids and multiple other molecules linked to metabolism had significantly increased levels in early‐stage advanced liver disease.
- These molecular changes could be used for the detection of liver disease progression or treatment development.

## 1 Introduction

Alcohol use is one of the leading causes of disability‐adjusted life years and mortality worldwide [1]. Alcohol‐related liver disease (ALD) is one of the most prevalent liver diseases and the leading cause of liver cirrhosis worldwide [2]. Most (80%–90%) chronic heavy drinkers develop some degree of ALD [3]. ALD‐related mortality and disease burden have been on the rise in both Europe and the United States in recent years [4].

ALD encompasses a spectrum of diseases, ranging from benign isolated steatosis to the more advanced stages steatohepatitis, cirrhosis and eventually liver cancer. The early stage (i.e., steatosis) is mostly asymptomatic and completely reversible with abstinence from alcohol [5]. Although most ALD cases present primarily as steatosis, only 20%–40% of those progress to steatohepatitis and 8%–20% further develop cirrhosis [3] and eventually hepatocellular carcinoma (HCC) [6]. Late stages of ALD (i.e., cirrhosis and HCC) carry a poor prognosis [7] with HCC accounting for around 80% of all primary liver cancer patients [8].

The early detection of ALD and its progression is hindered by the lack of specificity and sensitivity in traditional liver injury and alcohol biomarkers, such as aspartate aminotransferase (AST), alanine aminotransferase (ALT) and gammaglutamyltransferase (GGT). Thus, better biomarkers of progressive ALD are needed. Furthermore, there is a great need for detailed information on the driving factors of the individual differences in the progression of ALD, which has been linked to multiple metabolic processes, including dyslipidemia, altered bile acids, increased gut permeability and dysbiosis of gut microbiota [9, 10, 11]. Because of the multitude of potentially relevant pathways, a global (nontargeted) metabolomics analysis of the circulating metabolome could help to discover metabolic processes related to ALD progression.

Heavy alcohol use has been shown to alter the circulating metabolome and gut permeability as well as diminish the ability of vital nutrients to pass through the gut‐blood barrier [12, 13, 14, 15, 16, 17]. Previous studies have shown that changes in the circulating metabolome are associated with liver cirrhosis in ALD patients [18, 19] and metabolite profiles could be used to predict the development of various alcohol‐associated diseases including ALD [13, 20]. However, the differences in the circulating metabolome between nonprogressive and progressive ALD have not been extensively studied. Because progressive ALD has a significantly worse prognosis and higher mortality rate than nonprogressive ALD, understanding these differences is important.

Our aim was to measure the circulating metabolite profiles associated with progressive ALD when compared to nonprogressive ALD or healthy controls. We used serum samples collected from patients at the start of alcohol detoxification treatment and analysed metabolite profiles using a nontargeted liquid chromatography mass spectrometry (LC–MS) based metabolomics method combined with a targeted measurement of short‐chain fatty acids (SCFAs).

## 2 Materials and Methods

### 2.1 Patients

The study cohort consisted of alcohol use disorder (AUD) patients who were admitted for a standardised and controlled 3‐week detoxification and rehabilitation programme in Cliniques Universitaires Saint Luc, Brussels, Belgium between 2017 and 2019. A healthy volunteer control group (*n* = 32), who socially consumed low amounts of alcohol according to the World Health Organization (WHO) criteria (< 20 g/day), was recruited separately. The patients had a longstanding history of alcohol misuse. Daily alcohol use was inquired from all the patients with the help of the AUDIT‐C questionnaire. All the patients exceeded the WHO criteria for heavy continuous drinking (> 60 g/day) and were actively drinking until the day of admission. Exclusion criteria included antibiotic use during the 2 months preceding enrolment, immunosuppressive medication, diabetes, BMI > 30, inflammatory bowel disease, known liver disease of any aetiology other than ALD and clinically significant cardiovascular, pulmonary or renal co‐morbidities.

The participants were divided into groups depending on their clinical parameters (Table 2): a nonprogressive ALD group (*n* = 50) with controlled attenuation parameter (CAP) results > 250 dB/m, but normal liver enzymes (AST < 40 IU/L, ALT < 40 IU/L) and elastography measured liver stiffness results < 7.6 kPa, and a progressive ALD group (*n* = 50) with CAP > 250 dB/m, increased AST and ALT levels, and liver stiffness > 7.6 kPa. The nonprogressive ALD group included patients with isolated steatosis. The progressive ALD group included patients with possible steatohepatitis or fibrosis.

On the day of hospital admission, fasting serum and plasma EDTA samples were collected from the patients, and Fibroscan (Echosense, Paris, France) measurements were performed (Table 2). The sample collection protocol has been previously described in detail (11). Standard biochemical analyses were obtained from the biochemistry laboratory of the hospital. Blood‐soluble CD14 (sCD14), M65 cell death biomarker (M65) and peptidoglycan‐recognition proteins (PGRPs) were determined using commercially available kits (ELISA kits, Thermo Fisher Scientific, WA, the USA) (Table 2).

### 2.2 Ethical Considerations

The study complies with the Declaration of Helsinki and the Declaration of Istanbul. Written informed consent was obtained from all participants. The study has been approved by the ‘Comité d'éthique Hospitalo‐facultaire Saint Luc UCLouvain’ (B403201422657). The reporting of this study has been done in accordance with the STROBE criteria.

### 2.3 NonTargeted Metabolomics Analysis

The serum samples were sent to the University of Eastern Finland, Kuopio, Finland for nontargeted metabolomics analysis. The samples were stored at −80°C until use. When taken out of storage, they were thawed in ice water, after which they were kept in wet ice until assayed. The samples were then vortexed at the maximum speed with Vortez Genie 2 (Scientific industries, Bohemia, NY, the USA). 400 μL of frigid acetonitrile was added to a 96‐well plate with a filter plate. The vortexed serum samples were then added to the 96‐well plate. Pooled quality control samples were made by collecting 10 μL of each sample and adding them to the same tube and mixing. By pipetting 4 times, the acetonitrile and samples were mixed. After all the samples were ready, the 96‐well plate was centrifuged at 700×*g* for 5 min at 4°C with Heraeus Megafuge 40R (Thermo Fisher Scientific). After centrifuging, the filter plate was removed, and the plate was sealed with the 96‐well cap mat. Samples were prepared separately for reverse phase (RP) and hydrophilic interaction liquid chromatography (HILIC) analyses.

The serum samples were analysed with nontargeted liquid chromatography mass spectrometry metabolomics using ultra‐high performance liquid chromatography (UPLC) combined with Thermo Q Exactive Hybrid Quadrupole‐Orbitrap mass spectrometer (Thermo Scientific). The used RP column was Zorbax Eclipse XDB‐C18, particle size 1.8 μm, 2.1 × 100 mm (Agilent Technologies) and the used HILIC column was Acquity UPLC BEH Amide 1.7 μm, 2.1 × 100 mm (Waters Corporation, Milford, Massachusetts, the USA). The column temperature was 40°C and the flow rate was 0.4 mL/min (mobile phase A: H_2_O + 0.1% HCOOH, B: MeOH +0.1% HCOOH, 16.5 min gradient) for the RP mode and for the HILIC mode, the column temperature was 45°C and the mobile phase flow rate was 0.6 mL/min (mobile phase A: 50% acetonitrile +20 mM ammoniumformate buffer, B: 90% acetonitrile +20 mM ammoniumformate buffer, 12.5 min gradient). Positive and negative electrospray ionisation (ESI) were used for HILIC and RP analytic modes. ESI ray voltage was 3.5 kV for positive and 3.0 kV for negative mode.

Metabolite identification, peak picking and alignment were done with MS‐DIAL version 4.80 [21]. Preprocessing, including drift correction and missing value imputation, was done with ‘notame’ R‐package [22]. Metabolite identifications were ranked according to the community guidelines [23]. Metabolites in level 1 were matched against accurate mass, isotopic pattern, retention time and product ion spectra (MSMS) of fragmented ions from the in‐house library of chemical standards built using the same experimental conditions. Usage of the Level 2 includes metabolites with matching exact mass and MSMS spectra from public libraries, published papers or in the case of lipids, the built‐in MS‐DIAL library. Level 3 identification includes metabolites, whose chemical group has been recognised. All things equal, from multiple different ion forms of a certain compound, the most common product ion form was presented.

### 2.4 Serum Short‐Chain Fatty Acids Analysis

The analysis of serum acetic acid, propionic acid and butyric acid levels was based on a previously published method [24] with modifications. Serum samples stored at −80°C were thawed on wet ice and vortexed before processing in five batches. 150 μL of serum was aliquoted in a 10 mL vial holding a solution consisting of 0.5 g of NaH_2_PO_4_ and 1350 μL of cold Milli‐Q (MQ) water. Analytical blank samples holding only 0.5 g of NaH_2_PO_4_ and 1500 μL of cold MQ water were prepared in an equivalent manner to study samples. Individual stock solutions (500–2500 ppm) of acetic, propionic and butyric acids (Sigma‐Aldrich, Saint Louis, Missouri, the USA) prepared by dissolving standards in MQ water. A pooled analytical standard was prepared by combining 25 μL of each stock solution in a 10 mL vial holding a solution consisting of 0.5 g of NaH_2_PO_4_ and 1500 μL of cold MQ water. Vials were gently whirled and placed on the autosampler until analysis. Three injections consisting of blank, analytical standard and blank were injected at the beginning, end and every 26 injections during the sequence.

Solid‐phase microextraction coupled to gas chromatography and mass spectrometry (SPME‐GC–MS) analysis was carried out on a Thermo Trace 1310—TSQ 8000 Evo instrument holding a TriPlus RSH autosampler (Thermo Scientific) kept at +4°C throughout the analysis. SCFAs were extracted using a 75 μm CAR/PDMS Fused Silica SPME fibre (Supelco, Bellefonte, PA, the USA) that was conditioned according to the manual. Samples were incubated for 10 min followed by an extraction time of 40 min. Incubation and extraction temperatures were kept at 40°C. Five‐minute desorption temperature was 240°C in the GC injector port with a splitless mode. Chromatographic separation was performed by fused silica capillary column (Supelco) SPB‐624 (60 m × 0.25 mm × 1.4 μm) under a carrier gas (helium) 1.40 mL/min. The total GC oven programme time was 48 min where the initial temperature was held at 40°C for 10 min, ramped by 5°C/min to 200°C and then held at 200°C for 10 min. MS was operated at 240°C and an electron ionisation voltage of 70 eV, and ions were scanned in the full scan mode (30–300 amu). The instrument was operated, and data were analysed with Chromeleon 7.2.10 software (Thermo Fisher Scientific) by comparison of retention times and peak intensities against SCFA external analytical standards. Manually integrated spectral areas were exported to spreadsheet format for statistical analysis.

### 2.5 Statistical Analysis

For the statistical analysis, the results from the metabolomics and the SCFA analyses were combined. Welch's one‐way analysis of variance (ANOVA) was used to determine which metabolites had significant differences between the three study groups. For group‐to‐group comparison, Welch's *t*‐test and Cohen's *d* effect sizes (difference between group means divided by standard deviation) were used. Since molecular features in a nontargeted metabolomics analysis are not independent variables, but correlated to each other, we performed a principal component analysis (PCA) to evaluate the number of latent components needed to explain 95% of the variance in the metabolomics data. These components are independent of each other and therefore the number can be used to adjust significance level in a nontargeted metabolomics analysis. Here we needed 106 latent components to explain 95% of the variation in the metabolomics data and therefore adjusted the α level to 0.0005 to account for multiple testing (Bonferroni's method). Multivariate analysis of the differential molecular features, partial least sum of squares discriminant analysis (PLS‐DA) was used, and variable importance for projection (VIP) values are reported. For correlation, the Spearman method was used with false discovery rate (FDR, cutoff 5%) to account for multiple testing. Correlation analysis was done for all identified metabolites of identification level 1 or 2. Logistic regression model to find possible patterns of metabolites separating progressive and nonprogressive ALD patients was done using identified metabolites (level 1 or 2 identification [side chains identified for PCs]) with *p* ≤ 0.0001, *d* ≥ 0.7 and VIP ≥ 2. The data were analysed with R (version 4.2), R‐studio (version 492), JASP (version 0.16.2.0) (University of Amsterdam, Netherlands) and Microsoft Excel (Microsoft, Redmond, Washington, the USA). The pivotal packets in use were: notame, missforest, lme4 and Imertest. Graphs were drawn with JASP (version 0.16.2.0) and GraphPad Prism (version 9) (Graphpad Software, La Jolla, California, the USA).

## 3 Results

### 3.1 Study Population

The demographic, biochemical and clinical data are shown in Tables 1 and 2, respectively. The two ALD groups were similar in terms of age, sex and BMI (Table 1). However, the BMI and age were significantly lower in the control group compared to the ALD groups. According to the selection criteria, liver stiffness, AST and ALT levels were significantly different between the two ALD groups (Table 2). The international normalised ratio (INR), bilirubin and albumin values were within the normal range in the two ALD groups.

**TABLE 1:** Demographical characteristics of the study populations.

	Progressive ALD (*n* = 50)	Nonprogressive ALD (*n* = 50)	Healthy controls (*n* = 32)	*p*
Sex (*n*%)	16 women (32%)	17 women (34%)	16 women (50%)	0.218^a^
Age (years, Mean ± SD)	51.1 ± 9.9*	48.3 ± 11.3*	40.6 ± 14.6	0.001^b^
BMI (kg/m^2^, Mean ± SD)	25.8 ± 3.9*	24.3 ± 3.4	23.7 ± 3.7	0.036^b^

*Note:* There were no significant differences between the progressive ALD group and the nonprogressive ALD group.

Abbreviations: ALD = alcohol‐related liver disease, BMI = body mass index.

^a^ *χ* ^2^ test.

^b^ Welch's ANOVA.

* *p* < 0.05 when compared to the healthy controls.

**TABLE 2:** Baseline biochemical, fibroscan and bacterial translocation markers measurements and inquired daily alcohol intake from the alcohol‐related liver disease (ALD) groups, mean and standard deviation shown.

	Progressive ALD (*n* = 50)	Nonprogressive ALD (*n* = 50)	Welch's *t*‐test	Cohen's effect size
AST (IU/L)	119.5 ± 85.3	26 ± 7.5	*p* < 0.001	*d* = 1.54
ALT (IU/L)	87.2 ± 62.1	23.55 ± 9.82	*p* < 0.001	*d* = 1.43
AST/ALT ratio	1.5 ± 0.71	1.2 ± 0.35	*p* = 0.004	*d* = 0.61
GGT (IU/L)	443.6 ± 477.7	58.10 ± 43.8	*p* < 0.001	*d* = 1.18
M65 (IU/L)	687.5 ± 534.6	155.7 ± 79.1	*p* < 0.001	*d* = 1.50
Bilirubin (mg/dL)	0.78 ± 0.84	0.52 ± 0.26	*p* = 0.041	*d* = 0.42
Albumin (gr/L)	46.1 ± 6.6	47.3 ± 4.1	*p* = 0.290	*d* = 0.22
INR	1.1 ± 0.2	1.0 ± 0.1	*p* = 0.020	*d* = 0.50
CAP (dB/m)	308.5 ± 48.6	255.3 ± 56.9	*p* < 0.001	*d* = 1.01
Liver Stiffness (kPa)	15.9 ± 15.4	4.4 ± 1.1	*p* < 0.001	*d* = 1.05
sCD14 (ng/mL)	2104.7 ± 446.8	1860.4 ± 318.5	*p* = 0.015	*d* = 0.59
PGRPs (ng/mL)	38.4 ± 19.6	43.0 ± 24.7	*p* = 0.387	*d* = 0.21
Alcohol intake (gr/day)	208.3 ± 111.1	174.7 ± 105.1	*p* = 0.125	*d* = 0.311

Abbreviations: ALT = alanine aminotransferase, AST = aspartate aminotransferase, CAP = controlled attenuation parameter, GGT = gamma‐glutamyl aminotransferase, INR = international normalised ratio.

### 3.2 Comparisons Between ALD Groups and Controls

In the nontargeted metabolomics analysis, we collected 5253 molecular features with the two RP modes and 1824 molecular features with the two HILIC modes (Table S1). Levels of acetic acid, propionic acid and butyric acid were detected with the targeted SCFA analysis. In the ANOVA comparison, 3461 molecular features had a *p*‐value < 0.05. Furthermore, 1868 molecular features had a *p*‐value under the corrected α‐level accounting for multiple testing (*p* < 0.0005). Excluding multiple molecular features for a taken compound, 111 identified metabolites had a *p*‐value under the multiple testing corrected α‐level.

**FIGURE 1:**
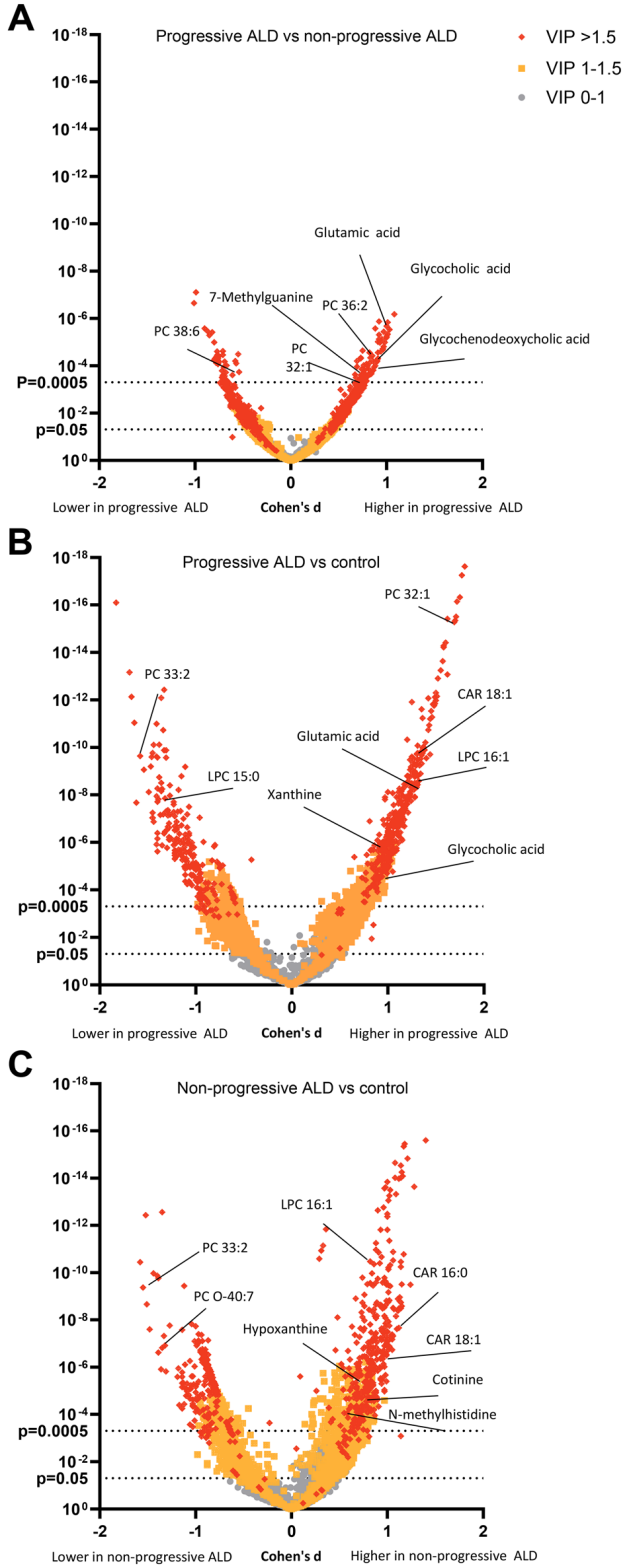
Volcano plot of all molecular features stratified by their significance in group comparison of the three different groups. Cohen's *d* effect sizes (difference between two groups divided by standard deviation) and *p*‐values from Welch's *t*‐test are shown for comparison between progressive alcohol‐related liver disease (ALD) and nonprogressive ALD (A), progressive ALD and healthy controls (B) and nonprogressive ALD and healthy controls (C). CAR = acylcarnitine, Control = control group, LPC = lysophosphatidylcholine, PC = phosphatidylcholine, VIP = variable importance in projection.

**FIGURE 2:**
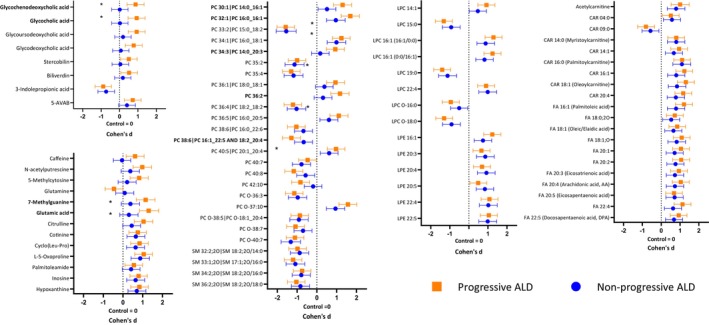
Changes in the significantly differential identified metabolites between alcohol‐related liver disease (ALD) groups and metabolites of interest in comparison to healthy controls. Cohen's *d* effect sizes with 95% confidence intervals are shown for comparisons between the control group and both progressive ALD and nonprogressive ALD groups. Positive Cohen's *d* means higher and negative *d* means lower metabolite levels when compared to the controls. Metabolites which have a significant difference (*p* < 0.0005) between nonprogressive ALD and progressive ALD group are marked with * and bolded. 5‐AVAB = 5‐amino valeric acid betaine, ALD = alcohol‐related liver disease, CAR = acylcarnitine, Control = control group, FA = fatty acid, LPC = lysophosphatidylcholine, LPE = lysophosphatidylethanolamine, PC = phosphatidylcholine, SM = sphingomyelin.

The total 111 identified metabolites and 3 short‐chain fatty acids had significant differences between the control group and the ALD groups (Figures 1 and 2). Glutamic acid, hypoxanthine, lysophosphatidylcholines (LPC), phosphatidylcholines (PC), fatty acids (FA) and acylcarnitines (CAR) all had significant differences between the control group and the two ALD groups (Figures 1 and 2). Furthermore, sphingomyelin (SM) levels were lower in both the nonprogressive ALD and progressive ALD groups when compared to the control group (Figure 2). Lysophosphatidylethanolamine (LPE) levels were higher in the ALD groups when compared to the control group. 5‐aminovaleric acid betaine (5‐AVAB) was significantly increased in the progressive ALD group in comparison to the control group (*p* = 0.0002, *d* = 0.56), but did not reach significance when comparing the nonprogressive ALD group to the control group or in the ALD groups intergroup comparison.

### 3.3 Comparison Between Progressive and Nonprogressive ALD Groups

**FIGURE 3:**
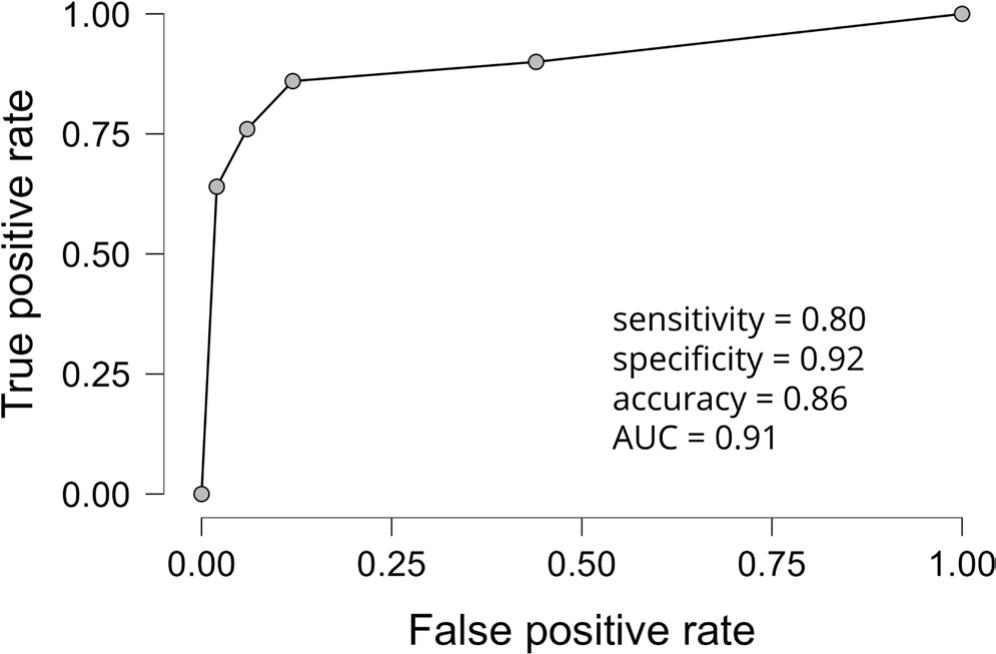
Performance of the logistic regression model with six metabolites to separate progressive ALD and nonprogressive ALD patients. Receiver operating characteristic (ROC) curve is shown. Metabolites included were glycocholic acid, glycochenodeoxycholic acid, glutamic acid, 7‐Methylguanine, PC 14:0_16:1 and PC 16:0_16:1. The logistic regression analysis showed good sensitivity (0.80), specificity (0.92) and accuracy (0.86). AUC, area under the curve.

When comparing the progressive ALD group to the nonprogressive ALD group, the levels of glycocholic acid (*p* < 0.0001, Cohen's *d* = 0.91), glycochenodeoxycholic acid (*p* < 0.0001, *d* = 0.90) (Figures 1 and 2), glutamic acid (*p* < 0.0001, *d* = 1.01) and 7‐methylguanine (*p* = 0.0001, *d* = 0.77) (Figure 2) were increased. Furthermore, PC 14:0_16:1 (*p* = 0.0001, *d* = 0.80), PC 16:0_16:1 (*p* < 0.0001, *d* = 0.73), PC 14:0_20:3 (*p* = 0.0002, *d* = 0.76) and PC 36:2 levels (*p* < 0.0001, *d* = 0.85) were higher in the progressive ALD group when compared to the nonprogressive ALD group (Figure 2). In contrast, PC 38:6 (16:1/22:5) (*p* = 0.0002, *d* = −0.61) levels were lower in the progressive ALD group when compared to the nonprogressive ALD group (Figures 1 and 2). In a logistic regression model with identified metabolites (in PCs, side chains needed to be identified) with *p* ≤ 0.0001, *d* ≥ 0.7 and VIP ≥ 2, we observed good separation between the progressive ALD and nonprogressive ALD groups (sensitivity = 0.80, specificity = 0.92, accuracy = 0.86, AUC = 0.91, Figure 3).

### 3.4 Correlations

**FIGURE 4:**
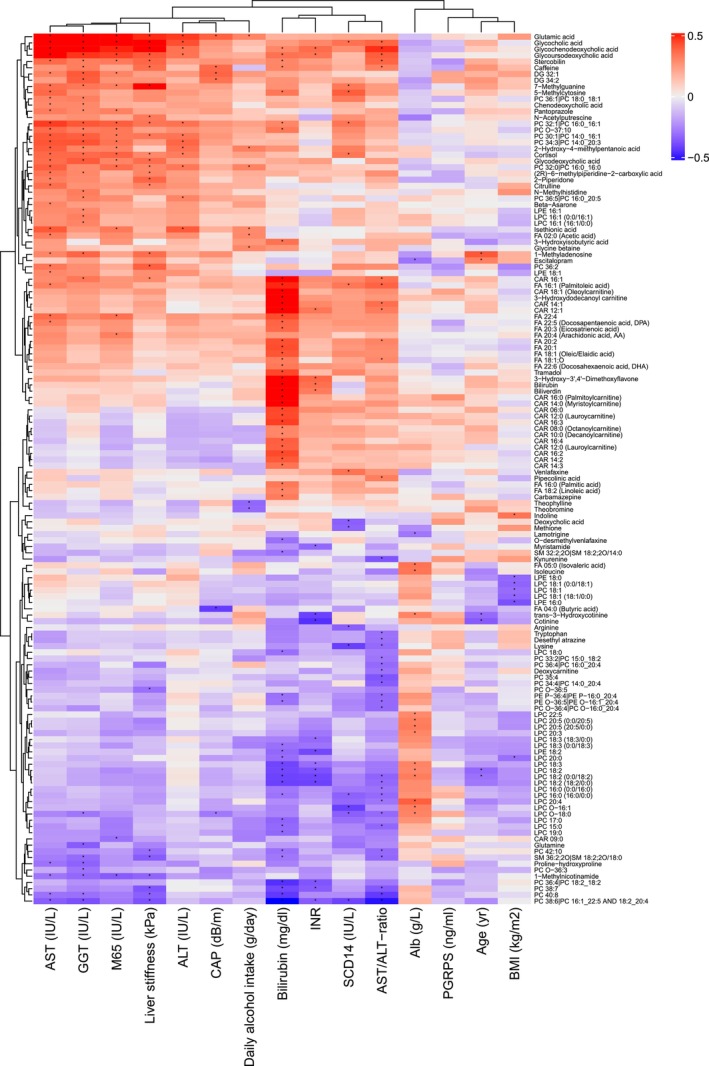
Spearman correlations between identified metabolites and clinical parameters with at least one significant correlation. The colour scale slides with the correlation coefficient, ones with positive correlation become increasingly red according to the correlation coefficient and those that have a negative correlation coefficient become increasingly blue. Alb = albumin, ALT = alanine aminotransferase, AST = aspartate aminotransferase, BMI = body mass index, CAP = controlled attenuation parameter, CAR = acylcarnitine, DG = diglyceride, FA = Fatty acid, GGT = gammaglutamyltransferase, INR = international normalised ratio, LPC = lysophosphatidylcholines, LPE = lysophosphatidylethanolamine, PC = phosphatidylcholine, PE = phosphatidylethanolamine, PGRPS = peptidoglycan‐recognition proteins, sCD14 = blood‐soluble CD14, SM = sphingomyelin. * FDR corrected *p*‐value < 0.05.

Correlation analysis with background variables (Table 1 and Table 2) was done for all identified metabolites and short‐chain fatty acids (Table S1). A total of 139 metabolites of identification levels 1 and 2 and short‐chain fatty acids had significant correlations (FDR corrected *p*‐value < 0.05) with at least one background variable (Figure 4).

Glycine‐conjugated bile acids, multiple phospholipids, 7‐methylguanine, 5‐methylcytosine and stercobilin correlated with AST. Similarly, ALT had a positive correlation with glycine‐conjugated bile acids and phospholipids, but not with 7‐methylguanine, 5‐methylcytosine and stercobilin. Accordingly, the AST/ALT ratio correlated positively with glycine conjugated bile acid levels and negatively with PCs and LPCs. GGT correlated positively with glutamic acid, glycine‐conjugated bile acids, PCs, LPCs and negatively with glutamine. Alcohol use had negative correlations with theophylline and theobromine and positive correlations with PC 32:0|PC 16:0_16:0, glutamic acid, glycine betaine, acetic acid (FA 02:0), isethionic acid and 2‐hydroxy‐4‐methylpentanoic acid.

The international normalised ratio (INR) had significant negative correlations with multiple LPCs and positive correlations with biliverdin, bilirubin, CAR 12:1, glycoursedeoxycholic acid and glycochenodeoxycholic acid. Blood albumin had positive correlations with LPCs, isoleucine, isovaleric acid, trans‐3‐hydroxycotine and negative correlations with lamotrigine and escitalopram. Bilirubin correlated positively with CARs, FAs, biliverdin, stercobilin, glycochenodeoxycholic acid, glycoursedeoxycholic acid, 5‐methylcytosine, caffeine, tramadol, 3‐Hydroxyisobutyric acid and 3‐hydroxy‐3'‐4'‐dimethoxyflavone Bilirubin had negative correlations with multiple LPCs, SMs, PCs, O‐desmethylvenlafaxine and LPE 18:2. sCD14 had a negative correlation with LPC O‐16:1, LPC O‐16:0, LPC O‐18:0 and PC 38:6. Glycocholic acid, 7‐methylguanine, 5‐methylcytosine and PC 32:1 had positive correlations with sCD14. The PGRPs did not have significant correlations with any identified metabolites.

The only metabolites that reached significance in correlation analysis with the CAP values were positive associations with glutamic acid, caffeine, DG 32:1, DG 34:2 and a negative correlation with butyric acid and LPC 0–18:0 (Figure 3). Elastography liver stiffness results correlated positively with glycine‐bound bile acids, glutamic acid, stercobilin, caffeine and 7‐methylguanine among others. Conversely, liver stiffness had a negative correlation with 1‐methylnicotinamide and several PCs. BMI correlated positively with indoline and negatively with LPE 16:0, LPE 18:0, LPC 18:1 (0:0/18:1), LPC 18:1 (18:1/0:0), LPC 20:0. Age correlated positively with escitalopram and 1‐methyladenosine levels and negatively with trans‐3‐hydroxycotinine, LPC 18:2 (18:2/0) and LPC 18:2 (0:0/18:2).

## 4 Discussion

The results of this study demonstrated distinct differences in the serum metabolite profiles between patients with progressive ALD and those with nonprogressive ALD. Increased levels of glycocholic acid, glycochenodeoxycholic acid, glutamic acid, PC 14:0_16:1, PC 16:0_16:1 and 7‐methylguanine demonstrated metabolic and enterohepatic alterations already early in the ALD disease progression, before the patients develop liver decompensation based on clinical and biochemical parameters (normal INR, albumin and bilirubin levels). Furthermore, the logistic regression model with these metabolites was able to separate patients into progressive or nonprogressive forms of ALD.

The primary finding was that circulating levels of glycine‐conjugated bile acids were significantly increased only in the progressive ALD group when compared to the nonprogressive ALD group and controls. Additionally, glycine‐conjugated bile acid levels correlated the strongest with liver stiffness measured with elastography. Glycine‐bound bile acids appearance in the circulating metabolome might be characteristic of the fibrotic process and could potentially be used as an alternate method of screening fibrosis. Increased levels of glycine‐conjugated bile acids have been reported previously in severe forms of ALD such as severe alcohol‐associated hepatitis and cirrhosis [25, 26]. Our results indicate that these circulating bile acids may already contribute to disease progression at much earlier stages and therefore represent a potential target for therapy. However, most bile acids in serum are conjugated with either glycine or taurine [27] and the conjugated bile acids are not directly hepatotoxic, whereas nonconjugated bile acids are [28, 29]. Therefore, the potential role of glycine‐conjugated bile acids as drivers or accelerators of disease progression is more complex. Primary bile acids cholic acid and chenodeoxycholic acid increased in the progressive ALD are produced by the neutral pathway initiated by 7α‐hydroxylase (CYP7A1) in the liver [30]. Secondary bile acids, resulting from transformation of primary bile acids by intestinal bacteria, were also increased, (ursodeoxycholic acid) [31]. This indicates that both the internal production of primary bile acids and the production of secondary bile acids by the gut microbiota are altered in progressive ALD. Alcohol use has been shown to increase bile acid levels in the liver of an early‐stage ALD mice model [32] as well as bile acid formation in hepatocytes in vitro [33]. Heavy alcohol use has previously been shown to increase levels of primary glycine‐conjugated bile acids in plasma [34]. Alcohol use can also alter gut microbiota, further affecting the enterohepatic circulation of bile acids [35]. Bile acids have also been linked to gut permeability, intestinal immunity, glutamine and glutamic acid metabolism, lipid metabolism and more broadly to energy metabolism through the activation of farnesoid X‐receptor (FXR) and secondary pathways [36, 37, 38]. It is conceivable that the high glycine‐conjugated bile acid levels could also be linked to the increased glutamic acid and altered phospholipid levels seen in the progressive ALD group.

7‐methylguanine was significantly higher in the progressive ALD group than in the other two groups, possibly in reaction to liver inflammation. It is a nucleic acid metabolite that inhibits poly(ADP‐ribose)polymerase‐1 and poly(ADP‐ribose)polymerase 2. Increased serum levels of 7‐methylguanine have been previously linked to increased risk of hepatocellular carcinoma [39], but its role in the progression of ALD is unclear. The strong correlation with liver stiffness could indicate 7‐methylguanine to have a role in the individual progression of the disease.

In our study, glutamic acid levels were positively correlated with alcohol consumption, as well as with ALD progression. Previous studies have shown that alcohol use in general increases circulating glutamic acid levels [40]. Glutamic acid is a part of multiple energy metabolism pathways and functions as an excitatory neurotransmitter [41]. Glutamic acid has also been linked to alcohol hepatotoxicity as an enhancer of mitochondrial oxidative stress [42] and therefore high glutamic acid levels could accelerate the progression of liver damage.

The major differences in the serum levels of FAs, CARs, PCs and LPCs observed between the ALD groups and the control group are in line with earlier findings of alterations in lipid profiles in a variety of profiles in steatotic liver diseases [43, 44]. Sphingomyelin levels in the ALD patients were lower than those in the control group, but there was not a significant difference between the two ALD groups. Sphingomyelin depletion has been linked to the progression of liver‐related events and ALD without metabolic syndrome overlap [45]. Overall, our results confirm this association since patients with diabetes and BMI > 30 were excluded from the study, leaving a study population of primarily ALD patients with fewer metabolic risk factors. Of the SMs, only SM 36:2 had a significant correlation with liver stiffness results, indicating that sphingomyelin depletion might be more characteristic of inflammation than fibrosis. Furthermore, both phosphatidylcholines and lysophosphatidylcholines showed significant differences between the study groups. Phosphatidylcholines have been proposed to play a role in the progression and pathogenesis of metabolic dysfunction‐associated steatotic liver disease (MASLD, previously NAFLD) [46]. Our results raise the possibility that this might also be the case in the earlier stages of ALD in a population without features of metabolic syndrome. Alcohol intoxication in patients with ALD has been shown to decrease circulating free FAs and lysophosphatidylcholines and increase circulating triglyceride levels [47]. In this study, fatty acid levels were significantly elevated in the ALD groups in comparison to the healthy control group, suggesting that the baseline levels of FAs are higher in ALD when compared to people without liver disease.

Interestingly, high serum levels of the microbiota‐associated metabolite 5‐aminovaleric acid betaine (5‐AVAB), which has previously been associated with obesity and MASLD in both humans and mouse models [48, 49], were seen in the progressive ALD group when compared to the controls. This provides a potential link between the altered intestinal microbiota observed in the development and progression of AUD and ALD. *Bifidobacteria* and *coriobacteriaceae* have been linked to 5‐AVAB levels [49]. 5‐AVAB influences lipid metabolism by reducing the β‐oxidation of FAs via an inhibition of the cell membrane carnitine transporter [49, 50]. Consequently, reduced β‐oxidation could explain some of the changes observed in lipids.

This study had some limitations. The study was cross‐sectional with a relatively small sample size. Therefore, to validate and increase the generalisability of the results, they should be validated in larger cohorts with a longitudinal design. Alcohol consumption was based on self‐reported daily alcohol use. Likely, using the timeline follow‐back method to assess ethanol consumption would have been more accurate. Furthermore, as in most nontargeted metabolomics studies, many significantly altered molecular features were not matched with known substances (Table S1). In addition, the healthy control group was significantly younger than the two ALD groups and had lower BMI than the progressive ALD group. However, in the present study, age and BMI did not correlate with levels of the key metabolites (glycocholic acid, glycochenodeoxycholic acid, glutamic acid, PC 14:0_16:1, PC 16:0_16:1 and 7‐methylguanine) indicating limited effect to the main findings.

In conclusion, patients with early‐stage progressive ALD with no liver decompensation have altered metabolic and enterohepatic processes when compared to patients with nonprogressive ALD. If the present findings of increased serum levels of glycine‐conjugated bile acids and glutamic acid in progressive ALD can be validated in longitudinal prospective cohorts, these alterations could serve as potential early‐phase biomarkers and/or treatment targets for predicting and preventing the progression of ALD.

## Author Contributions

P.S. and O.K. contributed to conception and design of the work. E.P., H.A., R.H., S.L., K.H., L.M., C.A., M.L., V.M., J.R., P.S. and O.K. contributed to data acquisition, analysis and interpretation. E.P. and O.K. contributed to drafting the manuscript. H.A., R.H., S.L., K.H., L.M., C.A., M.L., V.M., J.R. and P.S. contributed to revising the manuscript for important intellectual content. All authors contributed to final approval of the version to be published.

## Conflicts of Interest

O.K. and K.H. are founders of Afekta Technology Ltd., a company providing metabolomics analysis services. Other authors do not have any conflicts of interest.

## Supporting information


Data S1:

## Data Availability

The data that support the findings of this study are available on request from the corresponding author. The data are not publicly available due to privacy or ethical restrictions.

## References

[1] D. Bryazka , M. B. Reitsma , M. G. Griswold , et al., “Population‐Level Risks of Alcohol Consumption by Amount, Geography, Age, Sex, and Year: A Systematic Analysis for the Global Burden of Disease Study 2020,” Lancet 400, no. 10347 (2022): 185–235, 10.1016/S0140-6736(22)00847-9. 35843246 PMC9289789

[2] H. Devarbhavi , S. K. Asrani , J. P. Arab , Y. A. Nartey , E. Pose , and P. S. Kamath , “Global Burden of Liver Disease: 2023 Update,” Journal of Hepatology 79, no. 2 (2023): 516–537, 10.1016/j.jhep.2023.03.017. 36990226

[3] P. Mathurin and R. Bataller , “Trends in the Management and Burden of Alcoholic Liver Disease,” Journal of Hepatology 62, no. 1 Suppl (2015): S38–S46, 10.1016/j.jhep.2015.03.006. 25920088 PMC5013530

[4] F. Åberg , Z. G. Jiang , H. Cortez‐Pinto , and V. Männistö , “Alcohol‐Associated Liver Disease‐Global Epidemiology,” Hepatology (Baltimore, Md.) 80, no. 6 (2024): 1307–1322, 10.1097/HEP.0000000000000899. 38640041

[5] F. Åberg , P. Puukka , V. Salomaa , et al., “Risks of Light and Moderate Alcohol Use in Fatty Liver Disease: Follow‐Up of Population Cohorts,” Hepatology (Baltimore, Md.) 71, no. 3 (2020): 835–848, 10.1002/hep.30864. 31323122

[6] N. Ganne‐Carrié and P. Nahon , “Hepatocellular Carcinoma in the Setting of Alcohol‐Related Liver Disease,” Journal of Hepatology 70, no. 2 (2019): 284–293, 10.1016/j.jhep.2018.10.008. 30658729

[7] P. Ginès , A. Krag , J. G. Abraldes , E. Solà , N. Fabrellas , and P. S. Kamath , “Liver Cirrhosis,” Lancet (London, England) 398, no. 10308 (2021): 1359–1376, 10.1016/S0140-6736(21)01374-X. 34543610

[8] M. R. Toh , E. Y. T. Wong , S. H. Wong , et al., “Global Epidemiology and Genetics of Hepatocellular Carcinoma,” Gastroenterology 164, no. 5 (2023): 766–782, 10.1053/j.gastro.2023.01.033. 36738977

[9] L. He , V. Vatsalya , X. Ma , et al., “Metabolic Profiling of Bile Acids in the Urine of Patients With Alcohol‐Associated Liver Disease,” Hepatology Communications 5, no. 5 (2021): 798–811, 10.1002/hep4.1671. 34027270 PMC8122376

[10] V. B. Dubinkina , A. V. Tyakht , V. Y. Odintsova , et al., “Links of Gut Microbiota Composition With Alcohol Dependence Syndrome and Alcoholic Liver Disease,” Microbiome 5, no. 1 (2017): 141, 10.1186/s40168-017-0359-2. 29041989 PMC5645934

[11] L. Maccioni , B. Gao , S. Leclercq , et al., “Intestinal Permeability, Microbial Translocation, Changes in Duodenal and Fecal Microbiota, and Their Associations With Alcoholic Liver Disease Progression in Humans,” Gut Microbes 12, no. 1 (2020): 1782157, 10.1080/19490976.2020.1782157. 32588725 PMC7524402

[12] S. M. de la Monte and J. J. Kril , “Human Alcohol‐Related Neuropathology,” Acta Neuropathologica 127, no. 1 (2014): 71–90, 10.1007/s00401-013-1233-3. 24370929 PMC4532397

[13] O. Kärkkäinen , A. Klåvus , A. Voutilainen , et al., “Changes in Circulating Metabolome Precede Alcohol‐Related Diseases in Middle‐Aged Men: A Prospective Population‐Based Study With a 30‐Year Follow‐Up,” Alcoholism, Clinical and Experimental Research 44, no. 12 (2020): 2457–2467, 10.1111/acer.14485. 33067815

[14] M. Jaremek , Z. Yu , M. Mangino , et al., “Alcohol‐Induced Metabolomic Differences in Humans,” Translational Psychiatry 3, no. 7 (2013): e276, 10.1038/tp.2013.55. 23820610 PMC3731787

[15] A. I. Lehikoinen , O. K. Kärkkäinen , M. A. S. Lehtonen , S. O. K. Auriola , K. J. Hanhineva , and S. T. Heinonen , “Alcohol and Substance Use Are Associated With Altered Metabolome in the First Trimester Serum Samples of Pregnant Mothers,” European Journal of Obstetrics, Gynecology, and Reproductive Biology 223 (2018): 79–84, 10.1016/j.ejogrb.2018.02.004. 29500949

[16] S. Leclercq and P. de Timary , “Role of the Microbiome and the Gut‐Brain Axis in Alcohol Use Disorder: Potential Implication for Treatment Development,” Current Topics in Behavioral Neurosciences (2024): 1–18, 10.1007/7854_2024_478. 38914878

[17] O. Kärkkäinen , T. Tolmunen , P. Kivimäki , et al., “Alcohol Use‐Associated Alterations in the Circulating Metabolite Profile in the General Population and in Individuals With Major Depressive Disorder,” Alcohol 120 (2024): 161–167, 10.1016/j.alcohol.2024.01.005. 38278499

[18] J. S. Bajaj , Z. Kassam , A. Fagan , et al., “Fecal Microbiota Transplant From a Rational Stool Donor Improves Hepatic Encephalopathy: A Randomized Clinical Trial,” Hepatology (Baltimore, Md.) 66, no. 6 (2017): 1727–1738, 10.1002/hep.29306. PMC6102730 28586116

[19] Y. F. Xu , Y. X. Hao , L. Ma , et al., “Difference and Clinical Value of Metabolites in Plasma and Feces of Patients With Alcohol‐Related Liver Cirrhosis,” World Journal of Gastroenterology 29, no. 22 (2023): 3534–3547, 10.3748/wjg.v29.i22.3534. 37389241 PMC10303510

[20] Group NHBC , J. C. Barrett , T. Esko , et al., “Metabolomic and Genomic Prediction of Common Diseases in 477,706 Participants in Three National Biobanks.” 2023:2023.06.09.23291213, 10.1101/2023.06.09.23291213. PMC11582662 39572536

[21] H. Tsugawa , T. Cajka , T. Kind , et al., “MS‐DIAL: Data‐Independent MS/MS Deconvolution for Comprehensive Metabolome Analysis,” Nature Methods 12, no. 6 (2015): 523–526, 10.1038/nmeth.3393. 25938372 PMC4449330

[22] A. Klåvus , M. Kokla , S. Noerman , et al., ““Notame”: Workflow for Non‐Targeted LC‐MS Metabolic Profiling,” Metabolites 10, no. 4 (2020): E135, 10.3390/metabo10040135. PMC7240970 32244411

[23] L. W. Sumner , A. Amberg , D. Barrett , et al., “Proposed Minimum Reporting Standards for Chemical Analysis Chemical Analysis Working Group (CAWG) Metabolomics Standards Initiative (MSI),” Metabolomics 3, no. 3 (2007): 211–221, 10.1007/s11306-007-0082-2. 24039616 PMC3772505

[24] L. Nylund , S. Hakkola , L. Lahti , et al., “Diet, Perceived Intestinal Well‐Being and Compositions of Fecal Microbiota and Short Chain Fatty Acids in Oat‐Using Subjects With Celiac Disease or Gluten Sensitivity,” Nutrients 12, no. 9 (2020): 2570, 10.3390/nu12092570. 32854216 PMC7551214

[25] K. Brandl , P. Hartmann , L. J. Jih , et al., “Dysregulation of Serum Bile Acids and FGF19 in Alcoholic Hepatitis,” Journal of Hepatology 69, no. 2 (2018): 396–405, 10.1016/j.jhep.2018.03.031. 29654817 PMC6054564

[26] D. Ciocan , C. S. Voican , L. Wrzosek , et al., “Bile Acid Homeostasis and Intestinal Dysbiosis in Alcoholic Hepatitis,” Alimentary Pharmacology & Therapeutics 48, no. 9 (2018): 961–974, 10.1111/apt.14949. 30144108

[27] A. F. Hofmann , “The Continuing Importance of Bile Acids in Liver and Intestinal Disease,” Archives of Internal Medicine 159, no. 22 (1999): 2647–2658, 10.1001/archinte.159.22.2647. 10597755

[28] S. Kundu , S. Kumar , and A. Bajaj , “Cross‐Talk Between Bile Acids and Gastrointestinal Tract for Progression and Development of Cancer and Its Therapeutic Implications,” IUBMB Life 67, no. 7 (2015): 514–523, 10.1002/iub.1399. 26177921

[29] B. L. Woolbright , K. Dorko , D. J. Antoine , et al., “Bile Acid‐Induced Necrosis in Primary Human Hepatocytes and in Patients With Obstructive Cholestasis,” Toxicology and Applied Pharmacology 283, no. 3 (2015): 168–177, 10.1016/j.taap.2015.01.015. 25636263 PMC4361327

[30] N. Farooqui , A. Elhence , and Shalimar , “A Current Understanding of Bile Acids in Chronic Liver Disease,” Journal of Clinical and Experimental Hepatology 12, no. 1 (2022): 155–173, 10.1016/j.jceh.2021.08.017. 35068796 PMC8766695

[31] M. J. Chen , C. Liu , Y. Wan , et al., “Enterohepatic Circulation of Bile Acids and Their Emerging Roles on Glucolipid Metabolism,” Steroids 165 (2021): 108757, 10.1016/j.steroids.2020.108757. 33161055

[32] G. Charkoftaki , W. Y. Tan , P. Berrios‐Carcamo , et al., “Liver Metabolomics Identifies Bile Acid Profile Changes at Early Stages of Alcoholic Liver Disease in Mice,” Chemico‐Biological Interactions 360 (2022): 109931, 10.1016/j.cbi.2022.109931. 35429548 PMC9364420

[33] L. M. Nilsson , J. Sjövall , S. Strom , et al., “Ethanol Stimulates Bile Acid Formation in Primary Human Hepatocytes,” Biochemical and Biophysical Research Communications 364, no. 4 (2007): 743–747, 10.1016/j.bbrc.2007.10.039. 17976534

[34] M. D. Muthiah , E. Smirnova , P. Puri , et al., “Development of Alcohol‐Associated Hepatitis Is Associated With Specific Changes in Gut‐Modified Bile Acids,” Hepatology Communications 6, no. 5 (2022): 1073–1089, 10.1002/hep4.1885. 34984859 PMC9035568

[35] Y. Liu , T. Liu , X. Zhao , and Y. Gao , “New Insights Into the Bile Acid‐Based Regulatory Mechanisms and Therapeutic Perspectives in Alcohol‐Related Liver Disease,” Cellular and Molecular Life Sciences 79, no. 9 (2022): 486, 10.1007/s00018-022-04509-6. 35978227 PMC11073206

[36] J. Y. L. Chiang and J. M. Ferrell , “Discovery of Farnesoid X Receptor and Its Role in Bile Acid Metabolism,” Molecular and Cellular Endocrinology 548 (2022): 111618, 10.1016/j.mce.2022.111618. 35283218 PMC9038687

[37] V. Keitel , B. Görg , H. J. Bidmon , et al., “The Bile Acid Receptor TGR5 (Gpbar‐1) Acts as a Neurosteroid Receptor in Brain,” Glia 58, no. 15 (2010): 1794–1805, 10.1002/glia.21049. 20665558

[38] B. Renga , A. Mencarelli , S. Cipriani , et al., “The Nuclear Receptor FXR Regulates Hepatic Transport and Metabolism of Glutamine and Glutamate,” Biochimica et Biophysica Acta (BBA) ‐ Molecular Basis of Disease 1812, no. 11 (2011): 1522–1531, 10.1016/j.bbadis.2011.06.009. 21757002

[39] M. Stepien , P. Keski‐Rahkonen , A. Kiss , et al., “Metabolic Perturbations Prior to Hepatocellular Carcinoma Diagnosis: Findings From a Prospective Observational Cohort Study,” International Journal of Cancer 148, no. 3 (2021): 609–625, 10.1002/ijc.33236. 32734650

[40] A. Holmes , R. Spanagel , and J. H. Krystal , “Glutamatergic Targets for New Alcohol Medications,” Psychopharmacology 229, no. 3 (2013): 539–554, 10.1007/s00213-013-3226-2. 23995381 PMC3811052

[41] J. T. Brosnan and M. E. Brosnan , “Glutamate: A Truly Functional Amino Acid,” Amino Acids 45, no. 3 (2013): 413–418, 10.1007/s00726-012-1280-4. 22526238

[42] V. V. Teplova , A. G. Kruglov , L. I. Kovalyov , A. B. Nikiforova , N. I. Fedotcheva , and J. J. Lemasters , “Glutamate Contributes to Alcohol Hepatotoxicity by Enhancing Oxidative Stress in Mitochondria,” Journal of Bioenergetics and Biomembranes 49, no. 3 (2017): 253–264, 10.1007/s10863-017-9713-0. 28478591 PMC5542006

[43] B. Paul , M. Lewinska , and J. B. Andersen , “Lipid Alterations in Chronic Liver Disease and Liver Cancer,” JHEP Reports: Innovation in Hepatology 4, no. 6 (2022): 100479, 10.1016/j.jhepr.2022.100479. 35469167 PMC9034302

[44] K. Enooku , H. Nakagawa , N. Fujiwara , et al., “Altered Serum Acylcarnitine Profile Is Associated With the Status of Nonalcoholic Fatty Liver Disease (NAFLD) and NAFLD‐Related Hepatocellular Carcinoma,” Scientific Reports 9 (2019): 10663, 10.1038/s41598-019-47216-2. 31337855 PMC6650415

[45] M. Thiele , T. Suvitaival , K. Trošt , et al., “Sphingolipids Are Depleted in Alcohol‐Related Liver Fibrosis,” Gastroenterology 164, no. 7 (2023): 1248–1260, 10.1053/j.gastro.2023.02.023. 36849086

[46] J. L. Sherriff , T. A. O'Sullivan , C. Properzi , J. L. Oddo , and L. A. Adams , “Choline, Its Potential Role in Nonalcoholic Fatty Liver Disease, and the Case for Human and Bacterial Genes,” Advances in Nutrition 7, no. 1 (2016): 5–13, 10.3945/an.114.007955. 26773011 PMC4717871

[47] M. Israelsen , M. Kim , T. Suvitaival , et al., “Comprehensive Lipidomics Reveals Phenotypic Differences in Hepatic Lipid Turnover in ALD and NAFLD During Alcohol Intoxication,” JHEP Reports: Innovation in Hepatology 3, no. 5 (2021): 100325, 10.1016/j.jhepr.2021.100325. 34401690 PMC8350545

[48] K. H. Liu , J. A. Owens , B. Saeedi , et al., “Microbial Metabolite Delta‐Valerobetaine is a Diet‐Dependent Obesogen,” Nature Metabolism 3, no. 12 (2021): 1694–1705, 10.1038/s42255-021-00502-8. PMC8711632 34931082

[49] R. Haikonen , O. Kärkkäinen , V. Koistinen , and K. Hanhineva , “Diet‐ and Microbiota‐Related Metabolite, 5‐Aminovaleric Acid Betaine (5‐AVAB), in Health and Disease,” Trends in Endocrinology and Metabolism: TEM 33, no. 7 (2022): 463–480, 10.1016/j.tem.2022.04.004. 35508517

[50] O. Kärkkäinen , T. Tuomainen , V. Koistinen , et al., “Whole Grain Intake Associated Molecule 5‐Aminovaleric Acid Betaine Decreases β‐Oxidation of Fatty Acids in Mouse Cardiomyocytes,” Scientific Reports 8, no. 1 (2018): 13036, 10.1038/s41598-018-31484-5. 30158657 PMC6115339

