# Supplementary material for: Serum Metabolite Profile in Progressive Versus Nonprogressive Alcohol‐Related Liver Disease: A Cross‐Sectional Metabolomics Study
Source: Liver Int. 2025 May 13;45(6):e70128. doi: 10.1111/liv.70128 (PMC12070861; doi:10.1111/liv.70128)
Supplement: Supplementary file 1 — Data S1: [file LIV-45-0-s001.pdf]

**Supplementary table 1: Metabolomics results** (in asceding order according to p-value from progressive ALD vs  non-progressive ALD comparison, with molecular features with ID_level of 1-2 first)

| Feature_ID | RP_HILIC | POS_NEG | Average.Rt | Average.Mz | Metabolite.name | curated_MSMS | ID_level | all_ALD_vs_control t-test p | all_ALD_vs_control Cohen's d | all_ALD_vs_control PLS-DA VIP | Prog_ALD_vs_non-prog_ALD t-test p | Prog_ALD_vs_non-prog_ALD Cohen's d | Prog_ALD_vs_non-prog_ALD PLS-DA VIP | Prog_ALD_vs_control_ALD t-test p | Prog_ALD_vs_control_ALD Cohen's d | Prog_ALD_vs_control_ALD PLS-DA VIP | Non-prog_ALD_vs_control t-test p | Non-prog_ALD_vs_control Cohen's d | Non-prog_ALD_vs_control PLS-DA VIP | Healthy_control Mean | Healthy_control SD | all_ALD Mean | all_ALD SD | Non-prog_ALD Mean | Non-prog_ALD SD | Progressive_ALD Mean | Progressive_ALD SD |
|---|---|---|---|---|---|---|---|---|---|---|---|---|---|---|---|---|---|---|---|---|---|---|---|---|---|---|---|
| HILIC_NEG_146_04582a6_342 | HILIC | NEG | 6.34 | 146.0458 | Glutamic acid | 116.9214(100), 102.0555(65), 128.0349(30), 146.0464(18) | 1 | 9.01E-11 | 0.82 | 1.38 | 1.45E-06 | 1.01 | 2.57 | 2.59E-09 | 1.32 | 1.82 | 2.24E-05 | 0.31 | 1.39 | 490105 | 322685 | 1589809 | 1441092 | 905598 | 512451 | 2274019 | 1725651 |
| HILIC_POS_148_06041a6_375 | HILIC | POS | 6.38 | 148.0604 | Glutamic acid | 84.0452(100), 102.0553(40), 130.056(35), 143.068(25) | 1 | 1.43E-10 | 0.80 | 1.36 | 2.09E-06 | 1.00 | 2.54 | 4.66E-09 | 1.30 | 1.79 | 2.12E-05 | 0.30 | 1.38 | 1454208 | 993547 | 5034590 | 4789916 | 2792354 | 1685027 | 7276825 | 5766720 |
| RP_NEG_464_30231a8_82 | RP | NEG | 8.82 | 464.3023 | Glycocholic acid (BA 24:1;O4;G) | 464.3029(100), 74.0236(30) | 2 | 6.18E-05 | 0.50 | 0.95 | 1.55E-05 | 0.97 | 2.60 | 1.43E-05 | 0.98 | 1.36 | 7.95E-01 | 0.01 | 0.35 | 2923786 | 4426418 | 12023014 | 20479776 | 3163347 | 3358869 | 20882681 | 25998632 |
| RP_POS_786_60083a13_874 | RP | POS | 13.87 | 786.6008 | PC 36:2 | 184.0731(100), 786.5901(3) | 2 | 3.66E-07 | 0.74 | 1.30 | 3.77E-05 | 0.85 | 2.41 | 1.09E-07 | 1.17 | 1.70 | 1.05E-02 | 0.31 | 1.12 | 46148766 | 15528004 | 69551474 | 33501449 | 55991356 | 18012964 | 83111592 | 39596383 |
| RP_POS_754_53552a12_975 | RP | POS | 12.98 | 754.5355 | PC 32:1 [M+Na]+ | 146.9823(100), 86.0965(40), 571.468(35), 754.5403(35), 695.4633(30), 549.4855(15) | 2 | 4.01E-24 | 1.34 | 2.16 | 4.81E-05 | 0.73 | 2.38 | 4.48E-16 | 1.70 | 2.43 | 2.69E-14 | 0.97 | 2.21 | 11679509 | 5507836 | 36320929 | 17075318 | 29557975 | 11566328 | 43083884 | 19025283 |
| RP_POS_732_55353a12_969 | RP | POS | 12.97 | 732.5535 | PC 32:1\|PC 16:0_16:1 | 184.0735(100), 732.5476(3) | 2 | 7.50E-23 | 1.32 | 2.15 | 4.84E-05 | 0.73 | 2.36 | 5.26E-16 | 1.69 | 2.41 | 1.73E-13 | 0.96 | 2.16 | 43071534 | 21118949 | 126931501 | 58675467 | 103711675 | 40266409 | 150151327 | 65070511 |
| RP_POS_466_31628a8_815 | RP | POS | 8.82 | 466.3163 | Glycocholic acid | 412.2819(100), 337.2529(50), 319.2395(25), 430.2958(21), 76.0396(20), | 1 | 1.03E-04 | 0.47 | 0.93 | 4.88E-05 | 0.91 | 2.47 | 4.05E-05 | 0.93 | 1.30 | 7.07E-01 | 0.02 | 0.39 | 2653271 | 4036434 | 12134584 | 22473567 | 2979056 | 3433285 | 21290112 | 28940863 |
| RP_NEG_448_30701a9_311 | RP | NEG | 9.31 | 448.3070 | Glycochenodeoxycholic acid | 448.3033(100), 74.0236(35) | 1 | 1.28E-04 | -0.18 | 0.92 | 5.65E-05 | 0.90 | 2.46 | 4.73E-05 | 0.92 | 1.29 | 7.28E-01 | 0.02 | 0.55 | 11300301 | 13292022 | | | 12301616 | 11584875 | 68349588 | 89400521 |
| RP_POS_852_55029a12_638 | RP | POS | 12.64 | 852.5503 | PC 40:8 [M+Na]+ | 146.9819(100), 669.4792(75), 86.0969(65), 793.4791(30), 852.5507(25) | 2 | 1.24E-05 | -0.95 | 1.70 | 6.64E-05 | -0.69 | 2.30 | 4.41E-08 | -1.29 | 2.12 | 6.68E-03 | -0.60 | 1.12 | 3405928 | 995319 | 2442054 | 910131 | 2793812 | 911172 | 2090295 | 768654 |
| RP_POS_488_29803a8_816 | RP | POS | 8.82 | 488.2980 | Glycocholic acid [M+Na]+ | 488.2962(100) | 2 | 1.18E-04 | 0.47 | 0.93 | 7.09E-05 | 0.90 | 2.44 | 5.66E-05 | 0.91 | 1.29 | 6.56E-01 | 0.02 | 0.39 | 566880 | 900054 | 2916311 | 5670496 | 654497 | 805158 | 5178124 | 7340066 |
| RP_POS_704_52209a12_381 | RP | POS | 12.38 | 704.5221 | PC 30:1\|PC 14:0_16:1 | 184.0732(100), 704.5137(3) | 2 | 1.73E-11 | 0.90 | 1.66 | 1.01E-04 | 0.80 | 2.30 | 3.23E-09 | 1.30 | 1.92 | 2.77E-06 | 0.51 | 1.48 | 2886253 | 2302752 | 8558751 | 6519937 | 6057546 | 3384371 | 11059957 | 7852726 |
| HILIC_POS_166_07256a1_475 | HILIC | POS | 1.48 | 166.0726 | 7-Methylguanine | 166.0732(100) | 2 | 5.36E-07 | 0.76 | 1.31 | 1.50E-04 | 0.77 | 1.97 | 4.48E-08 | 1.15 | 1.96 | 1.13E-02 | 0.38 | 0.88 | 1289673 | 216127 | 1589919 | 410468 | 1437934 | 300377 | 1741903 | 450597 |
| RP_POS_450_32062a9_303 | RP | POS | 9.30 | 450.3206 | Glycochenodeoxycholic acid | 414.2982(100), 339.2652(20), 76.0396(15), 321.2572(15) | 1 | 2.66E-04 | 0.44 | 0.90 | 1.54E-04 | 0.86 | 2.35 | 1.34E-04 | 0.87 | 1.23 | 7.53E-01 | 0.01 | 0.54 | 5923344 | 7431856 | 24069969 | 46374378 | 6431272 | 6548859 | 41708665 | 60557589 |
| RP_POS_432_31018a9_303 | RP | POS | 9.30 | 432.3102 | Glycochenodeoxycholic acid [M-H2O+H]+ | 414.2975(100), 76.0396(30), 339.2647(30), 321.2564(20) | 2 | 2.77E-04 | 0.44 | 0.90 | 1.60E-04 | 0.85 | 2.35 | 1.40E-04 | 0.87 | 1.23 | 7.60E-01 | 0.01 | 0.53 | 5674106 | 7076859 | 23005822 | 44447792 | 6145088 | 6283264 | 39866555 | 58067886 |
| RP_POS_756_55298a12_652 | RP | POS | 12.65 | 756.5530 | PC 34:3\|PC 14:0_20:3 | 184.0732(100), 756.5563(3) | 2 | 1.23E-03 | 0.55 | 1.08 | 1.91E-04 | 0.76 | 2.19 | 2.53E-05 | 0.93 | 1.47 | 2.95E-01 | 0.17 | 0.80 | 39842308 | 13732763 | 50357103 | 19777472 | 43131694 | 13838629 | 57582511 | 22186208 |
| RP_POS_806_56873a12_736 | RP | POS | 12.74 | 806.5687 | PC 38:6\|PC 16:1_22:5 AND 18:2_20:4 | 184.0732(100), 806.5739(3) | 2 | 2.29E-05 | -0.98 | 1.63 | 2.46E-04 | -0.61 | 2.13 | 2.18E-07 | -1.29 | 2.15 | 4.37E-03 | -0.67 | 1.13 | 52627067 | 17662994 | 36403143 | 14184013 | 41476307 | 14784089 | 31329979 | 11635487 |
| RP_POS_726_50385a12_379 | RP | POS | 12.38 | 726.5039 | PC 30:1 [M+Na]+ | 146.9813(100), 86.0966(40), 726.5051(30), 543.436(30), 667.4315(22), 521.457(17) | 2 | 2.35E-10 | 0.87 | 1.62 | 3.56E-04 | 0.74 | 2.10 | 1.78E-08 | 1.24 | 1.85 | 1.03E-05 | 0.51 | 1.42 | 765449 | 779490 | 2430167 | 1981264 | 1730065 | 1069012 | 3130268 | 2405795 |
| RP_NEG_748_51483a12_381 | RP | NEG | 12.38 | 748.5148 | PC 30:1\|PC 14:0_16:1 [M+HCOO]- | 253.2183(100), 227.2001(60), 688.4893(30) | 2 | 3.09E-11 | 0.92 | 1.68 | 3.68E-04 | 0.73 | 2.10 | 4.79E-09 | 1.28 | 1.90 | 2.44E-06 | 0.56 | 1.51 | 902980 | 795130 | 2677702 | 1984571 | 1978917 | 1119472 | 3376488 | 2389074 |
| RP_POS_432_31091a8_33 | RP | POS | 8.33 | 432.3109 | Glycohyodeoxcholic acid [M-H2O+H]+ | 414.2974(100), 76.0394(35), 321.2565(20), 158.0813(20), 339.2697(20) | 2 | 2.34E-06 | 0.57 | 0.96 | 5.69E-04 | 0.76 | 2.02 | 1.74E-05 | 0.95 | 1.32 | 1.26E-02 | 0.19 | 0.96 | 389130 | 330816 | 1829421 | 2825934 | 859405 | 1225059 | 2799437 | 3565633 |
| RP_NEG_850_56183a12_745 | RP | NEG | 12.75 | 850.5618 | PC 38:6\|PC 16:1_22:5 AND 18:2_20:4 [M+HCOO]- | 279.2334(100), 329.2498(85), 253.2187(70), 303.2316(55), 790.5417(50) | 2 | 9.83E-06 | -0.97 | 1.59 | 6.44E-04 | -0.59 | 1.97 | 6.83E-08 | -1.27 | 2.16 | 3.44E-03 | -0.68 | 1.17 | 12955409 | 3729219 | 9306256 | 3315589 | 10416856 | 3633457 | 8195656 | 2547820 |
| RP_POS_770_50861a12_974 | RP | POS | 12.97 | 770.5086 | PC O-37:10 | 184.0728(100), 770.5153(3) | 2 | 5.14E-20 | 1.26 | 2.03 | 7.71E-04 | 0.62 | 1.98 | 2.32E-14 | 1.57 | 2.32 | 2.13E-11 | 0.94 | 2.00 | 697778 | 638379 | 2883117 | 1643966 | 2339832 | 1248765 | 3426401 | 1816213 |
| RP_POS_830_56824a12_639 | RP | POS | 12.64 | 830.5682 | PC 40:8 | 184.0732(100), 830.5507(1) | 2 | 4.90E-05 | -0.87 | 1.59 | 8.46E-04 | -0.60 | 1.96 | 4.34E-07 | -1.17 | 2.01 | 1.21E-02 | -0.57 | 1.04 | 10336707 | 2889994 | 7761593 | 2731898 | 8655792 | 2852282 | 6867393 | 2304672 |
| RP_POS_450_32138a8_33 | RP | POS | 8.33 | 450.3214 | Glycoursodeoxycholic acid | 414.2946(100), 339.2654(25), 76.403(25) | 2 | 3.52E-06 | 0.56 | 0.94 | 8.63E-04 | 0.74 | 1.96 | 2.78E-05 | 0.93 | 1.28 | 1.22E-02 | 0.19 | 0.96 | 335596 | 303140 | 1620487 | 2570585 | 766502 | 1116795 | 2474472 | 3258081 |
| HILIC_POS_450_32153a1_027 | HILIC | POS | 1.03 | 450.3215 | Glycochenodeoxycholic acid | 414.3001(100), 339.268(30), 321.2579(20), 76.0398(20) | 2 | 6.17E-04 | 0.41 | 0.73 | 8.67E-04 | 0.76 | 1.87 | 5.92E-04 | 0.79 | 1.07 | 3.69E-01 | 0.03 | 0.64 | 605931 | 635131 | 2778871 | 6052439 | 749370 | 794600 | 4808371 | 8060669 |
| RP_NEG_540_32928a10_375 | RP | NEG | 10.38 | 540.3293 | LPC 16:0 [M+HCOO]- | 255.2333(100), 480.3058(18), 224.069(1) | 2 | 3.52E-01 | 0.19 | 0.99 | 9.79E-04 | -0.65 | 2.02 | 5.39E-01 | -0.14 | 0.69 | 1.87E-02 | 0.51 | 1.12 | 120531302 | 24796683 | 125297572 | 25699440 | 133607547 | 22438460 | 116987597 | 26267601 |
| RP_POS_810_59705a14_755 | RP | POS | 14.76 | 810.5971 | PC 36:1 [M+Na]+ | 146.9821(100), 86.097(45), 810.6053(35), 627.5277(30), 751.5237(20), 605.5495(15) | 2 | 3.23E-04 | 0.60 | 1.03 | 1.18E-03 | 0.67 | 1.92 | 3.26E-05 | 0.94 | 1.34 | 8.60E-02 | 0.27 | 0.66 | 12664017 | 6545605 | 18384831 | 9877240 | 15216469 | 6343043 | 21553193 | 11678603 |
| RP_POS_854_56775a12_547 | RP | POS | 12.55 | 854.5678 | PC 42:10 | 184.0732(100), 854.5798(1) | 2 | 9.24E-03 | -0.51 | 1.17 | 1.27E-03 | -0.63 | 1.88 | 8.78E-05 | -0.83 | 1.65 | 3.87E-01 | -0.19 | 0.59 | 2631334 | 973529 | 2085035 | 1071379 | 2424548 | 1160733 | 1745522 | 858247 |
| RP_NEG_540_32928a10_504 | RP | NEG | 10.50 | 540.3293 | LPC 16:0 [M+HCOO]- | 255.2331(100), 480.3054(37), 224.0681(9) | 2 | 9.32E-01 | 0.02 | 0.91 | 1.49E-03 | -0.63 | 1.98 | 1.96E-01 | -0.30 | 0.79 | 1.12E-01 | 0.33 | 0.94 | 391151674 | 45168578 | 391947965 | 47529940 | 406804377 | 38834283 | 377091554 | 51046628 |
| RP_POS_734_56848a13_573 | RP | POS | 13.57 | 734.5685 | PC 32:0\|PC 16:0_16:0 | 184.0732(100), 734.5657(3) | 2 | 4.26E-02 | 0.38 | 0.84 | 1.54E-03 | 0.64 | 1.84 | 2.39E-03 | 0.70 | 1.14 | 7.64E-01 | 0.06 | 0.25 | 8567632 | 4045209 | 10361672 | 4894580 | 8832446 | 3626112 | 11890897 | 5521008 |
| RP_POS_450_32156a9_451 | RP | POS | 9.45 | 450.3216 | Glycodeoxycholic acid | 434.2901(100) | 1 | 4.29E-04 | 0.42 | 0.81 | 1.90E-03 | 0.70 | 1.86 | 6.98E-04 | 0.77 | 1.13 | 1.26E-01 | 0.07 | 0.85 | 3383123 | 3241080 | 12727886 | 25074945 | 4896809 | 5608941 | 20558963 | 33372483 |
| RP_POS_480_30869a10_519 | RP | POS | 10.52 | 480.3087 | LPE 18:1 | 339.2899(100), 480.3412(4), 60.0817(22) | 2 | 1.74E-03 | 0.53 | 1.24 | 1.95E-03 | 0.64 | 2.08 | 1.57E-04 | 0.85 | 1.41 | 1.97E-01 | 0.21 | 0.95 | 2301424 | 1002107 | 3050940 | 1482721 | 2596318 | 995581 | 3505562 | 1740348 |
| RP_POS_195_08778a3_862 | RP | POS | 3.86 | 195.0878 | Caffeine | 195.0876(100), 138.0663(25) | 1 | 6.60E-02 | 0.28 | 0.56 | 2.22E-03 | 0.67 | 1.80 | 6.63E-03 | 0.61 | 1.00 | 7.02E-01 | -0.05 | 0.58 | 80499529 | 93647137 | 124844619 | 171725980 | 72507967 | 88808760 | 177181272 | 214719375 |
| HILIC_NEG_131_07137a0_617 | HILIC | NEG | 0.62 | 131.0714 | 2-Hydroxy-4-methylpentanoic acid | 85.0656(100), 131.0721(60) | 2 | 2.43E-08 | 0.68 | 1.42 | 2.28E-03 | 0.67 | 1.80 | 3.62E-06 | 1.01 | 1.42 | 1.73E-04 | 0.35 | 1.25 | 1507805 | 663631 | 4238507 | 4393411 | 2903847 | 2323016 | 5573166 | 5474148 |
| HILIC_POS_144_10197a4_656 | HILIC | POS | 4.66 | 144.1020 | (2R)-6-methylpiperidine-2-carboxylic acid | 85.0289(100), 144.01012(70) | 2 | 2.52E-06 | 0.72 | 1.28 | 2.34E-03 | 0.63 | 1.67 | 1.41E-06 | 1.04 | 1.59 | 6.39E-03 | 0.41 | 0.96 | 1012369 | 371015 | 1487910 | 690091 | 1280169 | 491193 | 1695650 | 795503 |
| RP_NEG_590_34692a10_397 | RP | NEG | 10.40 | 590.3469 | LPC 20:3 [M+HCOO]- | 305.2499(100), 530.3265(20) | 2 | 1.93E-02 | 0.47 | 1.09 | 2.64E-03 | -0.59 | 1.76 | 4.29E-01 | 0.18 | 0.61 | 5.07E-04 | 0.77 | 1.33 | 3884128 | 1496574 | 4625213 | 1562089 | 5088011 | 1371413 | 4162415 | 1616218 |
| RP_POS_496_33841a10_503 | RP | POS | 10.50 | 496.3384 | LPC 16:0 (16:0/0:0) | 184.0733(100), 496.3362(25), 478.3312(5), 104.1067(65) | 2 | 3.52E-01 | 0.18 | 0.99 | 3.21E-03 | -0.59 | 1.90 | 6.04E-01 | -0.12 | 0.69 | 2.36E-02 | 0.48 | 1.09 | 1415784776 | 211859329 | ######### | 233774656 | 1525135336 | 202769164 | 1389224088 | 244697246 |
| HILIC_POS_126_06646a1_936 | HILIC | POS | 1.94 | 126.0665 | 5-Methylcytosine | 126.0661(100), 109.389(3) | 1 | 2.16E-03 | 0.51 | 1.02 | 3.32E-03 | 0.61 | 1.64 | 1.36E-04 | 0.82 | 1.41 | 2.29E-01 | 0.21 | 0.46 | 820442 | 241676 | 998545 | 364606 | 892786 | 293922 | 1104303 | 399215 |
| RP_POS_804_55328a12_408 | RP | POS | 12.41 | 804.5533 | PC 38:7 | 184.0736(100), 804.5588(2), 338.0778(1) | 2 | 3.68E-01 | -0.19 | 0.76 | 3.49E-03 | -0.58 | 1.71 | 2.88E-02 | -0.47 | 0.97 | 6.66E-01 | 0.10 | 0.40 | 7072383 | 3998100 | 6336960 | 3954987 | 7478556 | 4355696 | 5195364 | 3157736 |
| HILIC_POS_131_11803a3_335 | HILIC | POS | 3.34 | 131.1180 | N-Acetylputrescine | 114.0914(100), 86.0606(70), 72.018(45), 131.1177(20) | 1 | 1.19E-06 | 0.67 | 1.14 | 4.43E-03 | 0.61 | 1.63 | 7.16E-06 | 0.98 | 1.47 | 2.82E-03 | 0.37 | 1.09 | 4558200 | 951317 | 6035213 | 2349966 | 5369822 | 1433148 | 6700604 | 2863547 |
| HILIC_POS_176_10301a6_376 | HILIC | POS | 6.38 | 176.1030 | Citrulline | 159.0767(100), 113.0718(50), 70.0658(45) | 1 | 1.47E-07 | 0.74 | 1.19 | 4.54E-03 | 0.60 | 1.67 | 6.44E-07 | 1.04 | 1.55 | 1.78E-03 | 0.44 | 1.03 | 5169686 | 1796996 | 8122856 | 4225487 | 6934780 | 3136671 | 9310933 | 4832648 |
| RP_NEG_832_60986a14_761 | RP | NEG | 14.76 | 832.6099 | PC 36:1\|PC 18:0_18:1 [M+HCOO]- | 281.2503(100), 283.2653(35), 772.5747(20) | 2 | 2.13E-04 | 0.70 | 1.13 | 4.82E-03 | 0.56 | 1.70 | 2.69E-05 | 0.97 | 1.42 | 2.27E-02 | 0.42 | 0.89 | 11420568 | 5936068 | 16412247 | 7132947 | 14418850 | 5204401 | 18405643 | 8221981 |
| RP_NEG_526_35291a10_776 | RP | NEG | 10.78 | 526.3529 | LPC O-16:0 [M+HCOO]- | 466.3308(100), 78.5974(20), 168.0423(12), 377.2458(7) | 2 | 4.63E-03 | -0.64 | 1.23 | 4.90E-03 | -0.51 | 1.82 | 2.34E-04 | -0.90 | 1.55 | 1.06E-01 | -0.39 | 0.85 | 7114304 | 2172510 | 5850938 | 1799569 | 6351186 | 1855810 | 5350691 | 1608803 |
| HILIC_NEG_145_06169a6_093 | HILIC | NEG | 6.09 | 145.0617 | Glutamine | 115.9206(100), 100.9334(30), 127.0514(13), 145.062(12), | 1 | 2.50E-01 | -0.18 | 0.95 | 5.05E-03 | -0.61 | 1.54 | 1.42E-02 | -0.48 | 1.01 | 4.92E-01 | 0.12 | 1.15 | 11895182 | 2377826 | 11209627 | 4169022 | 12364548 | 3780880 | 10054706 | 4254295 |
| RP_POS_496_33997a10_375 | RP | POS | 10.38 | 496.3400 | LPC 16:0 (0:0/16:0) | 184.0733(100), 496.3366(3), 478.3301(1) | 2 | 1.66E-01 | 0.27 | 1.03 | 5.65E-03 | -0.56 | 1.79 | 9.52E-01 | -0.01 | 0.65 | 1.01E-02 | 0.55 | 1.18 | 309977343 | 73888194 | 331680098 | 83257195 | 354456126 | 74704884 | 308904070 | 85822476 |
| RP_POS_828_55005a12_957 | RP | POS | 12.96 | 828.5501 | PC 38:6 [M+Na]+ | 146.9816(100), 645.4805(80), 86.097(70), 769.4818(40), 828.5576(30), 623.5005(10) | 2 | 2.08E-09 | -1.06 | 1.92 | 7.59E-03 | -0.50 | 1.62 | 2.68E-10 | -1.31 | 2.22 | 1.40E-05 | -0.82 | 1.60 | 102717189 | 15155406 | 79505475 | 20611932 | 84951417 | 19324676 | 74059533 | 20601060 |
| RP_POS_788_6153a14_755 | RP | POS | 14.76 | 788.6153 | PC 36:1\|PC 18:0_18:1 | 184.0735(100), 788.6064(3) | 2 | 5.36E-06 | 0.66 | 1.12 | 7.62E-03 | 0.57 | 1.58 | 1.44E-05 | 0.94 | 1.36 | 5.46E-03 | 0.38 | 1.00 | 25267766 | 11915154 | 41120426 | 25636726 | 34311227 | 16707250 | 47929626 | 30889180 |
| RP_POS_184_07434a10_375 | RP | POS | 10.38 | 184.0743 | Phosphocholine | 184.0735(100), 86.097(37), 125.0002(20), 60.0815(7) | 2 | 1.43E-01 | 0.28 | 1.05 | 8.34E-03 | -0.54 | 1.77 | 9.51E-01 | 0.01 | 0.64 | 9.12E-03 | 0.55 | 1.20 | 3474080 | 857506 | 3741706 | 975202 | 3996524 | 861825 | 3486888 | 1022835 |
| HILIC_POS_518_32288a1_172 | HILIC | POS | 1.17 | 518.3229 | LPC 18:3 [M+Na]+ | 146.9822(100), 184.0743(80), 459.2502(55), 313.2715(55) | 2 | 4.71E-03 | 0.52 | 0.81 | 8.56E-03 | -0.53 | 1.43 | 2.27E-01 | 0.25 | 0.50 | 1.35E-04 | 0.79 | 1.37 | 2695604 | 974578 | 3312292 | 1211632 | 3627806 | 1087631 | 2996779 | 1256966 |
| HILIC_POS_100_07619a0_569 | HILIC | POS | 0.57 | 100.0762 | 2-Piperidone | 100.076(100), 56.0504(10), 86.0651(3) | 2 | 1.50E-05 | 0.51 | 0.95 | 8.87E-03 | 0.59 | 1.45 | 1.87E-04 | 0.81 | 1.26 | 1.52E-02 | 0.22 | 0.74 | 4747185 | 6244349 | 42623624 | 82570636 | 21037446 | 45232113 | 64209803 | 103817493 |
| RP_POS_806_56537a13_293 | RP | POS | 13.29 | 806.5654 | PC 36:3 [M+Na]+ | 146.9818(75), 86.0964(50), 623.4926(20), 806.5719(18), 747.4955(13), 601.5135(6), 184.0732 | 2 | 1.34E-02 | -0.43 | 0.85 | 8.90E-03 | 0.54 | 1.58 | 4.39E-01 | -0.16 | 0.64 | 2.60E-04 | -0.71 | 1.25 | 116832105 | 18004191 | 106636825 | 24522895 | 100276745 | 20552468 | 112996904 | 26646979 |
| RP_POS_595_34937a6_222 | RP | POS | 6.22 | 595.3494 | Stercobilin | 595.348(100), 345.1794(90), 470.2667(85), 166.0874(13), 126.0916(7), | 2 | 5.78E-03 | 0.33 | 0.85 | 8.97E-03 | 0.60 | 1.67 | 6.57E-03 | 0.63 | 1.06 | 3.56E-01 | 0.03 | 0.60 | 717235 | 1508155 | 4571468 | 13418985 | 1033677 | 1495221 | 8109259 | 18331250 |
| RP_POS_780_5528a12_577 | RP | POS | 12.58 | 780.5528 | PC 36:5\|PC 16:0_20:5 | 184.0732(100), 780.5327(2) | 2 | 1.09E-06 | 0.86 | 1.48 | 9.82E-03 | 0.51 | 1.61 | 1.04E-06 | 1.11 | 1.68 | 3.04E-04 | 0.61 | 1.41 | 14743398 | 6077631 | 21991760 | 8338970 | 19847748 | 5587874 | 24135772 | 9994522 |
| HILIC_POS_482_323a1_209 | HILIC | POS | 1.21 | 482.3230 | LPC 15:0 | 184.0743(100), 482.3315(5) | 2 | 1.23E-04 | -0.87 | 1.67 | 9.94E-03 | -0.45 | 1.49 | 4.79E-06 | -1.10 | 1.99 | 7.37E-03 | -0.65 | 1.29 | 688418 | 280717 | 458182 | 233062 | 517801 | 257760 | 398564 | 189869 |
| RP_POS_510_39182a11_174 | RP | POS | 11.17 | 510.3918 | LPC O-18:0 | 104.1074(100), 510.3875(25), 184.0727(15), 86.0969(10), 125(6), | 2 | 1.96E-05 | -1.10 | 1.88 | 1.03E-02 | -0.38 | 1.73 | 1.68E-06 | -1.30 | 1.98 | 4.55E-04 | -0.91 | 1.59 | 5748881 | 2225912 | 3722889 | 1378184 | 4073476 | 1474393 | 3372302 | 1188643 |
| HILIC_POS_147_07639a6_13 | HILIC | POS | 6.13 | 147.0764 | Glutamine | 130.0506(100), 84.0453(80), 102.0559(50) | 1 | 2.67E-01 | -0.18 | 0.98 | 1.05E-02 | -0.55 | 1.44 | 2.38E-02 | -0.46 | 0.93 | 6.39E-01 | 0.09 | 1.00 | 105370043 | 27844529 | 98113861 | 42386366 | 108865188 | 39368668 | 87362533 | 42942768 |
| RP_NEG_798_53076a12_467 | RP | NEG | 12.47 | 798.5308 | PC 34:4\|PC 14:0_20:4 [M+HCOO]- | 303.2313(100), 227.1998(60), 738.5117(30) | 2 | 5.35E-01 | 0.14 | 0.71 | 1.14E-02 | -0.47 | 1.50 | 6.98E-01 | -0.09 | 0.96 | 1.34E-01 | 0.38 | 0.74 | 3405520 | 1994949 | 3647527 | 1598823 | 4048698 | 1654765 | 3246356 | 1448428 |
| HILIC_POS_510_35559a1_135 | HILIC | POS | 1.14 | 510.3556 | LPC 17:0 | 184.0741(100), 72.0816(10) | 2 | 5.10E-05 | -0.90 | 1.72 | 1.19E-02 | -0.45 | 1.44 | 2.16E-06 | -1.12 | 2.08 | 3.99E-03 | -0.67 | 1.25 | 1080264 | 392170 | 735382 | 343952 | 821258 | 367755 | 649506 | 297869 |
| RP_POS_482_36081a10_775 | RP | POS | 10.78 | 482.3608 | LPC O-16:0 | 104.1075(100), 482.3635(25), 184.073(15), 86.097(10), 125.0002(6) | 2 | 1.94E-03 | -0.73 | 1.31 | 1.19E-02 | -0.44 | 1.71 | 1.58E-04 | -0.95 | 1.57 | 3.85E-02 | -0.51 | 0.97 | 15170131 | 4818100 | 12103598 | 3721374 | 13032136 | 3820510 | 11175060 | 3409401 |
| RP_NEG_564_33008a10_325 | RP | NEG | 10.33 | 564.3301 | LPC 18:2 [M+HCOO]- | 279.2348(100), 504.3116(17) | 2 | 3.15E-02 | -0.51 | 1.18 | 1.19E-02 | -0.45 | 1.73 | 4.68E-03 | -0.74 | 1.26 | 2.39E-01 | -0.29 | 0.88 | 256643826 | 71808914 | 225968093 | 53942674 | 239417501 | 48735550 | 212518685 | 55988374 |
| RP_NEG_850_5625a12_96 | RP | NEG | 12.96 | 850.5625 | PC 38:6\|PC 16:0_22:6 [M+HCOO]- | 250.2334(100), 327.2307(80), 790.5433(40) | 2 | 1.11E-06 | -0.89 | 1.69 | 1.25E-02 | -0.48 | 1.51 | 9.97E-08 | -1.13 | 1.97 | 9.27E-04 | -0.64 | 1.33 | 62758050 | 10247435 | 50730982 | 13241825 | 54012081 | 12551459 | 47449882 | 13219703 |
| RP_POS_766_57336a13_116 | RP | POS | 13.12 | 766.5734 | PC O-36:5 | 184.0732(100), 766.5707(2) | 2 | 4.21E-01 | -0.18 | 0.53 | 1.27E-02 | -0.48 | 1.48 | 7.38E-02 | -0.42 | 0.73 | 8.07E-01 | 0.06 | 0.34 | 3238011 | 2330194 | 2866150 | 2011983 | 3364208 | 2188689 | 2368092 | 1697692 |
| RP_POS_615_49512a14_633 | RP | POS | 14.63 | 615.4951 | DG 34:2 [M+Na]+ | 615.4916(100) | 2 | 3.44E-05 | 0.55 | 1.00 | 1.35E-02 | 0.54 | 1.46 | 2.72E-04 | 0.82 | 1.16 | 5.48E-03 | 0.28 | 1.00 | 2721780 | 2231429 | 6030132 | 6622314 | 4389734 | 3044304 | 7670530 | 8593350 |
| RP_NEG_331_26471a11_275 | RP | NEG | 11.28 | 331.2647 | FA 22:4 | 331.2646(100) | 2 | 6.88E-11 | 0.88 | 1.75 | 1.63E-02 | 0.50 | 1.53 | 5.45E-08 | 1.13 | 1.83 | 6.91E-06 | 0.63 | 1.68 | 1760523 | 1112056 | 4396658 | 3138992 | 3645830 | 2379393 | 5147487 | 3618788 |
| RP_POS_542_32184a10_198 | RP | POS | 10.20 | 542.3218 | LPC 18:2 [M+Adduct] | 483.2499(50), 542.3265(40) , 337.2713(30), 86.0964(35), 104.107(3), 146.9807(100) | 2 | 5.47E-02 | -0.46 | 1.15 | 1.68E-02 | -0.43 | 1.69 | 9.42E-03 | -0.67 | 1.18 | 3.26E-01 | -0.24 | 0.88 | 30307056 | 13532690 | 25173245 | 10162955 | 27587117 | 9500759 | 22759373 | 10321016 |
| RP_POS_566_32135a10_199 | RP | POS | 10.20 | 566.3214 | LPC 20:4 | 507.2521(60), 86.097(46), 566.3217(35), 361.2737(8), 104.1077(4) | 2 | 2.34E-01 | 0.24 | 0.84 | 1.71E-02 | -0.48 | 1.76 | 9.86E-01 | 0.00 | 0.45 | 2.83E-02 | 0.48 | 1.03 | 4916834 | 1793328 | 5357161 | 1827379 | 5790091 | 1588982 | 4924232 | 1959441 |
| RP_POS_452_27689a10_003 | RP | POS | 10.00 | 452.2769 | LPE 16:1 | 311.2559(100) | 2 | 2.76E-15 | 0.98 | 1.85 | 1.84E-02 | 0.49 | 1.47 | 8.59E-09 | 1.23 | 1.90 | 1.84E-09 | 0.74 | 1.80 | 183839 | 120090 | 1008375 | 871218 | 803497 | 590969 | 1213253 | 1048154 |
| RP_NEG_586_31628a9_909 | RP | NEG | 9.91 | 586.3163 | LPC 20:5 [M+HCOO]- | 301.2173(100), 526.2935(32), 224.0689(3) | 2 | 1.55E-01 | 0.31 | 0.94 | 1.86E-02 | -0.45 | 1.43 | 6.99E-01 | 0.09 | 0.52 | 2.75E-02 | 0.54 | 1.01 | 1805702 | 1441279 | 2216007 | 1249343 | 2508120 | 1258855 | 1923894 | 1180980 |
| RP_POS_520_34045a10_324 | RP | POS | 10.32 | 520.3405 | LPC 18:2 (18:2/0:0) | 184.0736(100), 520.331(25), 502.3302(6), 104.1077(75) | 2 | 8.77E-02 | -0.40 | 1.11 | 1.92E-02 | -0.42 | 1.69 | 1.78E-02 | -0.62 | 1.12 | 4.31E-01 | -0.19 | 0.85 | 905685662 | 316593824 | 799262164 | 239989786 | 855148604 | 213420877 | 743375723 | 253856386 |
| RP_NEG_722_5144a13_381 | RP | NEG | 13.38 | 722.5144 | PE O-36:5\|PE O-16:1_20:4 | 303.2314(100), 436.2796(15), 722.518(13) | 2 | 4.86E-01 | -0.15 | 0.75 | 1.92E-02 | -0.46 | 1.37 | 9.01E-02 | -0.38 | 0.79 | 7.48E-01 | 0.08 | 0.59 | 1964491 | 1073176 | 1814494 | 984331 | 2043959 | 1111626 | 1585029 | 783849 |
| HILIC_POS_282_11938a4_449 | HILIC | POS | 4.45 | 282.1194 | 1-Methyladenosine | 150.078(100), 282.1189(5) | 1 | 2.29E-05 | 0.64 | 0.96 | 1.96E-02 | 0.49 | 1.40 | 5.66E-06 | 0.89 | 1.48 | 2.37E-02 | 0.39 | 0.88 | 2634220 | 319233 | 3004249 | 613961 | 2861835 | 572467 | 3146664 | 626481 |

**Supplementary table 1: Metabolomics results** (in asceding order according to p-value from progressive ALD vs  non-progressive ALD comparison, with molecular features with ID_level of 1-2 first)

| Feature_ID | RP_HILIC | POS_NEG | Average.Rt | Average.Mz | Metabolite.name | curated_MSMS | ID_level |
|---|---|---|---|---|---|---|---|
| HILIC_NEG_124_99135a0_735 | HILIC | NEG | 0.74 | 124.9914 | Isetheonic acid | 96.9596(100), 124.9917(45) | 2 |
| RP_POS_482_32394a10_107 | RP | POS | 10.11 | 482.3239 | LPC 15:0 | 184.0733(100), 482.3246(3) | 2 |
| RP_POS_184_06049a1_807 | RP | POS | 1.81 | 184.0605 | 4-Pyridoxate | 166.0506(100), 184.0614(30), 148.0391(18) | 2 |
| RP_POS_810_59979a13_542 | RP | POS | 13.54 | 810.5998 | PC 38:4\|PC 18:2_20:2 | 184.0732(100), 810.5881(2) | 2 |
| HILIC_POS_170_09241a6_372 | HILIC | POS | 6.37 | 170.0924 | N-Methylhistidine | 124.0868(100), 170.0929(40), 109.0757(10) | 2 |
| RP_NEG_564_33142a10_198 | RP | NEG | 10.20 | 564.3314 | LPC 18:2 [M+HCOO]- | 279.2348(100), 504.3116(17) | 2 |
| RP_NEG_478_29431a10_515 | RP | NEG | 10.52 | 478.2943 | LPE 18:1 | 281.2492(100), 478.2928(6), 214.0502(2), 196.0375(1), 78.9581(1) | 2 |
| RP_POS_504_30548a10_247 | RP | POS | 10.25 | 504.3055 | LPC 15:0 [M+Na]+ | 104.1075(100), 445.2271(60), 504.3061(25), 299.2548(10), 86.097(15) | 2 |
| RP_POS_568_33978a10_192 | RP | POS | 10.19 | 568.3398 | LPC 22:6 (0:0/22:6) | 184.0736(100), 568.3354(3), 550.330(0.6) | 2 |
| RP_NEG_450_26285a10_138 | RP | NEG | 10.14 | 450.2629 | LPE 16:1 | 253.2184(100), 450.2629(18), 196.0373(11), 78.9574(3), 214.0472(2.3) | 2 |
| RP_POS_784_586a13_296 | RP | POS | 13.30 | 784.5860 | PC 36:3 | 184.0732(100), 784.5887(3) | 2 |
| RP_NEG_812_58368a13_592 | RP | NEG | 13.59 | 812.5837 | PC O-36:4\|PC O-16:0_20:4 [M+HCOO]- | 303.235(100), 752.5616(50), 466.33(14), 448.3212(5), 279.2336(90) | 2 |
| RP_POS_520_34009a10_198 | RP | POS | 10.20 | 520.3401 | LPC 18:2 (0:0/18:2) | 184.0733(100), 520.3419(3), 502.3257(1) | 2 |
| HILIC_POS_184_06052a0_827 | HILIC | POS | 0.83 | 184.0605 | 4-Pyridoxic acid | 166.0505(100), 184.0604(25), 148.0393(25) | 1 |
| RP_POS_568_33813a10_52 | RP | POS | 10.52 | 568.3381 | LPC 20:3 [M+Na]+ | 104.1076(100), 509.2663(50), 568.3379(20), 86.0964(17), 363.2902(3) | 2 |
| RP_POS_706_53735a12_843 | RP | POS | 12.84 | 706.5374 | PC 30:0\|PC 14:0_16:0 | 184.0735(100), 706.5386(4), | 2 |
| RP_POS_542_3241a9_91 | RP | POS | 9.91 | 542.3241 | LPC 20:5 (0:0/20:5) | 184.0736(100), 542.3288(5) | 2 |
| RP_POS_247_1441a2_845 | RP | POS | 2.85 | 247.1441 | Lenticin | 188.0712(100), 60.0814(50), 146.0599(20) | 2 |
| RP_POS_398_32635a8_935 | RP | POS | 8.94 | 398.3264 | CAR 16:1 | 85.0288(100), 398.3235(60) | 2 |
| HILIC_POS_496_33926a1_309 | HILIC | POS | 1.31 | 496.3393 | LPC 16:0 | 184.0741(100), 104.1071(70), 496.3452(20), 86.0971(18) | 2 |
| RP_POS_538_38635a11_132 | RP | POS | 11.13 | 538.3864 | LPC 19:0 | 184.0722(100), 538.3342(60), 520.3745(5), 104.107(80), 538.8439(40) | 2 |
| HILIC_POS_137_071a2_193 | HILIC | POS | 2.19 | 137.0710 | 1-Methylnicotinamide | 137.0708(100), 94.0657(5) | 1 |
| RP_NEG_612_33154a10_191 | RP | NEG | 10.19 | 612.3315 | LPC 22:6 [M+HCOO]- | 327.2362(100), 552.3096(40), | 2 |
| RP_POS_724_52667a13_381 | RP | POS | 13.38 | 724.5267 | PE P-36:4\|PE P-16:0_20:4 | 361.2745(100), 266.2842(20), 583.5109(10), 60.0815(30), 81.0704(30), 95.0855(30) | 2 |
| RP_POS_590_32159a10_295 | RP | POS | 10.30 | 590.3216 | LPC 22:6 (22:6/0:0) | 104.1076(100), 531.2495(50), 86.0964(18) | 2 |
| RP_POS_132_07686a0_577 | RP | POS | 0.58 | 132.0769 | Creatine | 90.0559(100), 132.0767(90), 114.067(4) | 2 |
| HILIC_POS_520_34039a1_279 | HILIC | POS | 1.28 | 520.3404 | LPC 18:2 | 184.0745(100), 104.1073(70), 520.3422(40), 86.0972(20) | 2 |
| RP_POS_544_33948a10_199 | RP | POS | 10.20 | 544.3395 | LPC 20:4 | 184.0734(100), 544.333(3), 526.3271(0.8) | 2 |
| RP_POS_744_58899a14_128 | RP | POS | 14.13 | 744.5890 | PC O-34:2 | 184.0732(100), 744.5767(3) | 2 |
| RP_POS_776_51837a12_466 | RP | POS | 12.47 | 776.5184 | PC 34:4 [M+Na]+ | 146.9817(100), 593.4515(75), 86.0968(65), 776.52(40), 717.4384(38) | 2 |
| RP_NEG_253_21748a10_827 | RP | NEG | 10.83 | 253.2175 | FA 16:1 (Palmitoleic acid) | 253.2186(100) | 2 |
| RP_POS_426_35754a9_373 | RP | POS | 9.37 | 426.3575 | CAR 18:1 (Oleoylcarnitine) | 85.0288(100), 426.3528(60) | 1 |
| HILIC_POS_595_34918a1_11 | HILIC | POS | 1.11 | 595.3492 | Stercobilin | 345.182(100), 470.2632(95), 595.3523(75) | 2 |
| RP_POS_288_25229a9_361 | RP | POS | 9.36 | 288.2523 | Lauryl diethanolamide | 106.0876(100), 88.0762(55), 288.2547(20), 227.1991(20) | 2 |
| HILIC_POS_482_32376a1_354 | HILIC | POS | 1.35 | 482.3238 | LPC 15:0 | 104.1073(100), 184.0728(80), 482.3219(10) | 2 |
| RP_POS_247_14406a3_062 | RP | POS | 3.06 | 247.1441 | Lenticin | 188.0716(100), 60.0815(65), 146.0603(25) | 2 |
| RP_POS_564_30579a10_032 | RP | POS | 10.03 | 564.3058 | LPC 20:5 (20:5/0:0) | 104.1076(100), 505.2331(55), 564.306(15), 86.0964 | 2 |
| RP_POS_494_32391a9_984 | RP | POS | 9.98 | 494.3239 | LPC 16:1 (0:0/16:1) | 184.0735(100), 494.3196(3) | 1 |
| RP_POS_140_06783a0_552 | RP | POS | 0.55 | 140.0678 | Glycine betaine [M+Na]+ | 140.0682(100), 74.0968(50), 114.0889(4), 96.0793(1) | 2 |
| RP_POS_466_29263a9_501 | RP | POS | 9.50 | 466.2926 | LPC 14:1 | 184.0729(100), 466.2884(25), 448.2802(7), 104.1073(85) | 2 |
| RP_POS_494_32336a10_125 | RP | POS | 10.13 | 494.3234 | LPC 16:1 (16:1/0:0) | 184.073(100), 494.3183(25), 476.3127(8) , 104.1075(90) | 1 |
| RP_POS_832_5813a13_541 | RP | POS | 13.54 | 832.5813 | PC 38:4\|PC 18:2_20:2 [M+Na]+ | 146.9818(100), 86.0968(50), 649.5152(40), 832.5806(30), 773.5119(25), 627.5269(8) | 2 |
| RP_POS_728_5191a12_844 | RP | POS | 12.84 | 728.5191 | PC 30:0 [M+Na]+ | 146.982(100), 86.0964(40), 728.5206(35), 545.4542(28), 669.4459(25), 523.475(20) | 2 |
| HILIC_POS_520_34064a1_15 | HILIC | POS | 1.15 | 520.3406 | LPC 18:2 | 184.0742(100), 520.3424(3) | 2 |
| HILIC_POS_118_08664a3_699 | HILIC | POS | 3.70 | 118.0866 | Glycine betaine | 118.0868(100), 59.0739(3) | 1 |
| RP_POS_285_07895a8_02 | RP | POS | 8.02 | 285.0790 | Diazepam | 285.0785(100), 257.084(6), 154.0414(5) | 2 |
| HILIC_POS_132_0768a5_591 | HILIC | POS | 5.59 | 132.0768 | Creatine | 90.0554(100), 132.0771(80) | 1 |
| RP_POS_790_57318a13_242 | RP | POS | 13.24 | 790.5732 | PC O-38:7 | 184.0732(100), 790.5635(2) | 2 |
| RP_POS_746_56818a13_32 | RP | POS | 13.32 | 746.5682 | PC 33:1 | 184.0732(100), 746.5707(2) | 2 |
| RP_NEG_588_33154a10_199 | RP | NEG | 10.20 | 588.3315 | LPC 20:4 [M+HCOO]- | 303.2313(100), 528.3049(20) | 2 |
| RP_NEG_538_31592a9_984 | RP | NEG | 9.98 | 538.3159 | LPC 16:1 [M+HCOO]- | 253.2186(100), 478.2944(19) | 2 |
| RP_NEG_750_53046a12_847 | RP | NEG | 12.85 | 750.5305 | PC 30:0\|PC 14:0_16:0 [M+HCOO]- | 227.2(100), 255.2332(90), 690.5033(30) | 2 |
| RP_NEG_824_54694a12_579 | RP | NEG | 12.58 | 824.5469 | PC 36:5\|PC 16:0_20:5 [M+HCOO]- | 279.235(100), 303.2319(45), 764.5127(35), 253.2187(30) | 2 |
| RP_POS_448_34177a9_121 | RP | POS | 9.12 | 448.3418 | CAR 20:4 | 448.3451(100), 85.0292(75) | 2 |
| RP_POS_146_11719a0_566 | RP | POS | 0.57 | 146.1172 | Deoxycarnitine | 146.1178(100), 87.0447(48), 60.0814(25), 130.0506(5) | 1 |
| HILIC_POS_229_11833a6_131 | HILIC | POS | 6.13 | 229.1183 | Proline-hydroxyproline | 70.0659(100), 229.1171(35), 132.0657(20) | 2 |
| RP_NEG_824_54712a12_695 | RP | NEG | 12.70 | 824.5471 | PC 36:5\|PC 16:0_20:5 [M+HCOO]- | 301.2184(100), 255.2334(90), 764.5135(35) | 2 |
| RP_NEG_826_56226a13_071 | RP | NEG | 13.07 | 826.5623 | PC 36:4\|PC 16:0_20:4 [M+HCOO]- | 303.2315(100), 255.233(60), 766.5427(25) | 2 |
| RP_POS_480_34473a10_699 | RP | POS | 10.70 | 480.3447 | LPC O-16:1 | 104.1076(100), 480.3414(100), 86.0964(45), 184.0733(35), 124.9998(22) | 2 |
| RP_POS_802_53503a12_693 | RP | POS | 12.69 | 802.5350 | PC 36:5 [M+Na]+ | 146.9814(100), 619.4614(65), 86.0966(61), 743.4567(35), 802.5381(30), 597.4855(7) | 2 |
| RP_NEG_538_31543a10_125 | RP | NEG | 10.13 | 538.3154 | LPC 16:1 [M+HCOO]- | 253.2184(100), 478.2941(35), 224.0698(9) | 2 |
| RP_POS_782_56714a13_72 | RP | POS | 13.72 | 782.5671 | PC 34:1 [M+Na]+ | 146.9823(100), 86.0965(45), 782.5644(35), 599.5015(30), 723.4892(22), 577.5145(15) | 2 |
| RP_POS_363_21646a7_048 | RP | POS | 7.05 | 363.2165 | Cortisol | 363.217(100), 121.0645(50), 327.1921(20), 309.1825(15), 267.1724(13), 345.2052(10) | 1 |
| SCFA_Acetic acid | SCFA | SCFA | | | FA 02:0 (Acetic acid) | targeted_SCFA_analysis | 1 |
| RP_POS_160_13326a2_925 | RP | POS | 2.93 | 160.1333 | Pregabalin | 160.1332(30), 142.1218(100), 97.103(40) | 2 |
| RP_POS_752_55762a14_271 | RP | POS | 14.27 | 752.5576 | PE P-38:4\|PE P-18:0_20:4 | 361.2746(100), 392.2911(45), 294.313(22), 611.5332(7), 752.5183(2), 81.0704(30), 95.0856(28), 67.055(25), 119.0853(20) | 2 |
| RP_POS_384_08264a6_157 | RP | POS | 6.16 | 384.0826 | Pantoprazole | 138.0552(100), 200.038(90), 153.0791(38), 122.0599(8), 107.073(2) | 2 |
| RP_POS_806_56885a12_958 | RP | POS | 12.96 | 806.5689 | PC 38:6\|PC 16:0_22:6 | 184.0735(100), 806.5713(3) | 2 |
| RP_POS_152_07077a2_588 | RP | POS | 2.59 | 152.0708 | Paracetamol | 152.0712(100), 110.0598(60), 134.0595(2) | 2 |
| RP_POS_225_19606a7_999 | RP | POS | 8.00 | 225.1961 | Dicyclohexylurea | 225.1955(100), 100.1126(25), 143.1169(2) | 2 |

| Feature_ID | all_ALD_vs_control t-test p | all_ALD_vs_control Cohen's d | all_ALD_vs_control PLS-DA VIP | Prog_ALD_vs_non-prog_ALD t-test p | Prog_ALD_vs_non-prog_ALD Cohen's d | Prog_ALD_vs_non-prog_ALD PLS-DA VIP | Prog_ALD_vs_control_ALD t-test p | Prog_ALD_vs_control_ALD Cohen's d | Prog_ALD_vs_control_ALD PLS-DA VIP | Non-prog_ALD_vs_control t-test p | Non-prog_ALD_vs_control Cohen's d | Non-prog_ALD_vs_control PLS-DA VIP | Healthy_control Mean | Healthy_control SD | all_ALD Mean | all_ALD SD | Non-prog_ALD Mean | Non-prog_ALD SD | Progressive_ALD Mean | Progressive_ALD SD |
|---|---|---|---|---|---|---|---|---|---|---|---|---|---|---|---|---|---|---|---|---|
| HILIC_NEG_124_99135a0_735 | 8.43E-08 | 0.64 | 1.21 | 2.06E-02 | 0.51 | 1.27 | 1.00E-05 | 0.90 | 1.38 | 1.81E-03 | 0.39 | 0.94 | 95435 | 189266 | 2301078 | 3801349 | 1423664 | 2838097 | 3178492 | 4423741 |
| RP_POS_482_32394a10_107 | 3.44E-07 | -1.12 | 1.90 | 2.18E-02 | -0.39 | 1.61 | 1.38E-08 | -1.32 | 2.15 | 6.25E-05 | -0.93 | 1.52 | 2774748 | 866525 | 1760499 | 770473 | 1936498 | 865034 | 1584501 | 622794 |
| RP_POS_184_06049a1_807 | 6.03E-03 | 0.32 | 1.08 | 2.24E-02 | 0.53 | 1.63 | 1.15E-02 | 0.58 | 1.34 | 1.16E-02 | 0.06 | 1.07 | 40703 | 26516 | 1510674 | 5237528 | 303387 | 707589 | 2717960 | 7207494 |
| RP_POS_810_59979a13_542 | 3.65E-01 | 0.19 | 0.48 | 2.26E-02 | 0.45 | 1.34 | 8.16E-02 | 0.42 | 0.66 | 8.95E-01 | -0.03 | 0.70 | 31027692 | 10610512 | 32967835 | 9883772 | 30726981 | 9150471 | 35208689 | 10168216 |
| HILIC_POS_170_09241a6_372 | 6.45E-07 | 0.76 | 1.16 | 2.30E-02 | 0.47 | 1.35 | 9.45E-06 | 1.00 | 1.39 | 1.63E-04 | 0.52 | 1.34 | 5370796 | 4455880 | 11504195 | 8441803 | 9583610 | 4997719 | 13424781 | 10558285 |
| RP_NEG_564_33142a10_198 | 5.30E-02 | -0.48 | 1.17 | 2.31E-02 | -0.39 | 1.61 | 1.14E-02 | -0.68 | 1.17 | 2.67E-01 | -0.28 | 0.90 | 64774366 | 28531293 | 53992029 | 19593327 | 58418752 | 18191566 | 49565307 | 20117329 |
| RP_NEG_478_29431a10_515 | 2.80E-03 | 0.53 | 1.30 | 2.34E-02 | 0.47 | 1.68 | 9.85E-04 | 0.77 | 1.30 | 8.13E-02 | 0.30 | 1.07 | 2085171 | 1078202 | 2824416 | 1433442 | 2499772 | 957877 | 3149060 | 1737477 |
| RP_POS_504_30548a10_247 | 1.28E-06 | -1.10 | 1.90 | 2.40E-02 | -0.38 | 1.61 | 7.27E-08 | -1.29 | 2.08 | 1.17E-04 | -0.91 | 1.53 | 4357765 | 1468554 | 2757217 | 1227729 | 3033028 | 1347350 | 2481406 | 1036725 |
| RP_POS_568_33978a10_192 | 6.75E-01 | -0.08 | 0.99 | 2.44E-02 | -0.47 | 1.39 | 1.37E-01 | -0.31 | 0.77 | 4.77E-01 | 0.15 | 0.90 | 7255418 | 3056201 | 6981217 | 3627388 | 7793263 | 3703003 | 6169170 | 3395060 |
| RP_NEG_450_26285a10_138 | 7.52E-18 | 1.08 | 1.92 | 2.47E-02 | 0.45 | 1.41 | 3.20E-10 | 1.31 | 2.02 | 3.47E-10 | 0.85 | 1.86 | 1080988 | 619883 | 5125148 | 3791214 | 4276218 | 2867799 | 5974078 | 4398282 |
| RP_POS_784_586a13_296 | 3.03E-02 | -0.47 | 0.88 | 2.49E-02 | 0.43 | 1.36 | 2.54E-01 | -0.26 | 0.59 | 4.92E-03 | -0.69 | 1.23 | 217912908 | 57811395 | 192380842 | 51364781 | 180917591 | 53133676 | 203844094 | 47319784 |
| RP_NEG_812_58368a13_592 | 2.01E-02 | -0.45 | 0.82 | 2.50E-02 | -0.45 | 1.32 | 1.06E-03 | -0.67 | 1.21 | 3.28E-01 | -0.22 | 0.47 | 10478619 | 2552581 | 9196806 | 2904004 | 9845160 | 3250182 | 8548453 | 2370026 |
| RP_POS_520_34009a10_198 | 6.57E-02 | -0.45 | 1.14 | 2.67E-02 | -0.39 | 1.63 | 1.44E-02 | -0.64 | 1.13 | 3.17E-01 | -0.25 | 0.88 | 161435926 | 78088733 | 133294869 | 55757578 | 145587092 | 52383302 | 121002647 | 56823297 |
| HILIC_POS_184_06052a0_827 | 1.77E-03 | 0.37 | 1.08 | 2.72E-02 | 0.51 | 1.34 | 6.69E-03 | 0.62 | 1.13 | 1.40E-02 | 0.11 | 1.23 | 214175 | 122474 | 5624468 | 16834392 | 1878162 | 4613807 | 9370774 | 22861483 |
| RP_POS_568_33813a10_52 | 1.15E-02 | 0.50 | 1.06 | 2.81E-02 | -0.44 | 1.30 | 1.98E-01 | 0.28 | 0.71 | 1.06E-03 | 0.72 | 1.24 | 5574627 | 1682654 | 6483998 | 1804641 | 6878362 | 1680026 | 6089634 | 1854735 |
| RP_POS_706_53735a12_843 | 5.45E-03 | 0.54 | 1.00 | 2.86E-02 | 0.44 | 1.31 | 1.16E-03 | 0.76 | 1.21 | 1.03E-01 | 0.32 | 0.59 | 6773192 | 4093088 | 9245456 | 4574934 | 8247036 | 3684313 | 10243876 | 5165138 |
| RP_POS_542_3241a9_91 | 1.27E-01 | 0.32 | 0.96 | 2.89E-02 | -0.43 | 1.35 | 6.36E-01 | 0.11 | 0.55 | 2.37E-02 | 0.53 | 1.02 | 3305299 | 2601355 | 4117951 | 2498097 | 4661356 | 2563504 | 3574547 | 2331128 |
| RP_POS_247_1441a2_845 | 7.41E-02 | -0.50 | 0.92 | 2.92E-02 | -0.33 | 1.45 | 1.91E-02 | -0.67 | 1.16 | 2.56E-01 | -0.34 | 0.45 | 10136039 | 13105818 | 5702513 | 6678016 | 7158351 | 8149974 | 4246674 | 4399364 |
| RP_POS_398_32635a8_935 | 6.46E-11 | 1.01 | 1.76 | 2.92E-02 | 0.43 | 1.56 | 3.30E-10 | 1.23 | 2.01 | 6.32E-06 | 0.80 | 1.57 | 2802052 | 1777237 | 6117985 | 3248397 | 5412966 | 3101562 | 6823003 | 3268859 |
| HILIC_POS_496_33926a1_309 | 2.91E-02 | 0.40 | 1.00 | 2.96E-02 | -0.45 | 1.30 | 4.05E-01 | 0.17 | 0.30 | 3.12E-03 | 0.62 | 1.10 | 690900610 | 188706862 | 782707945 | 240981525 | 834910418 | 235722738 | 730505471 | 237071090 |
| RP_POS_538_38635a11_132 | 3.92E-06 | -1.26 | 2.08 | 3.04E-02 | -0.28 | 1.47 | 6.97E-07 | -1.40 | 2.04 | 3.68E-05 | -1.12 | 1.72 | 2323884 | 1141243 | 1182873 | 596840 | 1311577 | 617025 | 1054169 | 552431 |
| HILIC_POS_137_071a2_193 | 3.85E-01 | -0.15 | 0.46 | 3.06E-02 | -0.46 | 1.21 | 8.71E-02 | -0.38 | 0.59 | 6.76E-01 | 0.08 | 0.25 | 24372493 | 13299135 | 21783032 | 18036759 | 25672336 | 14259557 | 17893727 | 20568978 |
| RP_NEG_612_33154a10_191 | 6.49E-01 | -0.08 | 0.98 | 3.09E-02 | -0.45 | 1.30 | 1.36E-01 | -0.31 | 0.75 | 5.05E-01 | 0.14 | 0.90 | 3124791 | 1176762 | 3008417 | 1461373 | 3322467 | 1482672 | 2694368 | 1383912 |
| RP_POS_724_52667a13_381 | 5.16E-01 | -0.14 | 0.87 | 3.17E-02 | -0.43 | 1.26 | 9.93E-02 | -0.35 | 0.80 | 7.59E-01 | 0.08 | 0.67 | 5471775 | 2674330 | 5119989 | 2559848 | 5669397 | 3069128 | 4570581 | 1789918 |
| RP_POS_590_32159a10_295 | 6.92E-01 | -0.07 | 0.95 | 3.21E-02 | -0.45 | 1.30 | 1.50E-01 | -0.30 | 0.74 | 4.76E-01 | 0.15 | 0.90 | 8724385 | 3197915 | 8448679 | 4004895 | 9303466 | 4089330 | 7593891 | 3767217 |
| RP_POS_132_07686a0_577 | 8.25E-02 | 0.32 | 0.51 | 3.26E-02 | -0.44 | 1.45 | 6.36E-01 | 0.10 | 0.57 | 1.05E-02 | 0.54 | 0.88 | 6109140 | 3726232 | 7526688 | 4601188 | 8505782 | 4466996 | 6547594 | 4567658 |
| HILIC_POS_520_34039a1_279 | 1.66E-01 | -0.33 | 0.88 | 3.34E-02 | -0.39 | 1.24 | 4.05E-02 | -0.52 | 0.92 | 5.96E-01 | -0.13 | 0.34 | 285871340 | 140419696 | 247808939 | 106870268 | 270452220 | 105279845 | 225165658 | 104620197 |
| RP_POS_544_33948a10_199 | 2.73E-01 | 0.23 | 0.81 | 3.36E-02 | -0.42 | 1.67 | 9.19E-01 | 0.02 | 0.44 | 5.16E-02 | 0.44 | 0.96 | 26610415 | 10934134 | 29038706 | 10271102 | 31213360 | 9012565 | 26864052 | 11057416 |
| RP_POS_744_58899a14_128 | 4.27E-01 | -0.15 | 0.37 | 3.40E-02 | 0.44 | 1.28 | 7.59E-01 | 0.07 | 0.28 | 6.12E-02 | -0.37 | 0.69 | 4631243 | 1474922 | 4380447 | 1743056 | 4011603 | 1372412 | 4749291 | 1994326 |
| RP_POS_776_51837a12_466 | 6.71E-01 | 0.10 | 0.67 | 3.42E-02 | -0.40 | 1.26 | 6.74E-01 | -0.10 | 0.92 | 2.40E-01 | 0.30 | 0.57 | 3652681 | 2316282 | 3844969 | 1864747 | 4238527 | 1940924 | 3451411 | 1715296 |
| RP_NEG_253_21748a10_827 | 2.78E-11 | 1.00 | 1.88 | 3.53E-02 | 0.42 | 1.48 | 1.55E-09 | 1.21 | 2.02 | 2.11E-06 | 0.79 | 1.73 | 1274513 | 1078853 | 3403496 | 2130919 | 2956491 | 1892457 | 3850502 | 2277088 |
| RP_POS_426_35754a9_373 | 2.87E-11 | 1.11 | 1.78 | 3.79E-02 | 0.47 | 1.26 | 1.71E-10 | 1.31 | 1.98 | 2.73E-07 | 0.84 | 1.75 | 12749926 | 5215526 | 22162205 | 8020179 | 19880440 | 7244677 | 23821726 | 8476643 |
| HILIC_POS_595_34918a1_11 | 5.87E-02 | 0.27 | 0.76 | 3.81E-02 | 0.46 | 1.19 | 2.56E-02 | 0.50 | 0.85 | 7.16E-01 | 0.05 | 0.22 | 1811623 | 5587625 | 4755204 | 11826975 | 2294365 | 6220026 | 7216044 | 15217161 |
| RP_POS_288_25229a9_361 | 1.23E-01 | -0.30 | 0.93 | 3.84E-02 | 0.42 | 1.23 | 7.04E-01 | -0.09 | 0.59 | 9.98E-03 | -0.51 | 0.95 | 5894523 | 336613 | 5784749 | 370759 | 5707977 | 258685 | 5861521 | 445851 |
| HILIC_POS_482_32376a1_354 | 2.00E-04 | -0.77 | 1.59 | 3.88E-02 | -0.39 | 1.26 | 9.94E-06 | -0.97 | 1.88 | 1.33E-02 | -0.58 | 1.24 | 7418106 | 2569083 | 5333328 | 2552824 | 5860067 | 2918578 | 4806590 | 2020517 |
| RP_POS_247_14406a3_062 | 7.97E-02 | -0.48 | 0.89 | 3.91E-02 | -0.33 | 1.37 | 2.01E-02 | -0.64 | 1.14 | 2.80E-01 | -0.31 | 0.42 | 15471256 | 18466578 | 9308453 | 10197530 | 11413623 | 12475016 | 7203284 | 6740258 |
| RP_POS_564_30579a10_032 | 9.73E-02 | 0.33 | 0.97 | 3.94E-02 | -0.41 | 1.26 | 5.45E-01 | 0.13 | 0.57 | 2.14E-02 | 0.54 | 1.06 | 4287268 | 4057287 | 5690204 | 4202907 | 6553623 | 4546296 | 4826784 | 3674116 |
| RP_POS_494_32391a9_984 | 4.78E-16 | 1.03 | 1.87 | 3.99E-02 | 0.42 | 1.30 | 7.97E-09 | 1.24 | 1.90 | 3.32E-11 | 0.82 | 1.95 | 7841732 | 3270524 | 22226873 | 14253052 | 19295243 | 9331322 | 25158502 | 17488042 |
| RP_POS_140_06783a0_552 | 1.40E-04 | 0.53 | 0.90 | 4.05E-02 | 0.45 | 1.41 | 1.74E-04 | 0.76 | 1.23 | 4.22E-02 | 0.31 | 0.97 | 4371724 | 747724 | 5240577 | 1769607 | 4878721 | 1461769 | 5602433 | 1980639 |
| RP_POS_466_29263a9_501 | 4.38E-08 | 0.69 | 1.41 | 4.06E-02 | 0.45 | 1.33 | 3.42E-05 | 0.92 | 1.44 | 4.98E-06 | 0.47 | 1.38 | 231674 | 192158 | 717867 | 761817 | 561373 | 408196 | 874361 | 977738 |
| RP_POS_494_32336a10_125 | 2.34E-17 | 1.09 | 1.92 | 4.08E-02 | 0.41 | 1.30 | 1.55E-09 | 1.29 | 1.97 | 5.13E-12 | 0.88 | 2.01 | 68531727 | 27857219 | 186186962 | 108750645 | 163926128 | 72991453 | 208447795 | 132497921 |
| RP_POS_832_5813a13_541 | 5.66E-01 | 0.12 | 0.41 | 4.20E-02 | 0.40 | 1.19 | 1.78E-01 | 0.32 | 0.51 | 7.40E-01 | -0.08 | 0.58 | 11126733 | 3779757 | 11561935 | 3479644 | 10856349 | 3264812 | 12267522 | 3576020 |
| RP_POS_728_5191a12_844 | 5.85E-03 | 0.54 | 1.00 | 4.24E-02 | 0.40 | 1.25 | 1.47E-03 | 0.74 | 1.19 | 9.17E-02 | 0.34 | 0.61 | 2059118 | 1339049 | 2856377 | 1464392 | 2559677 | 1210547 | 3153077 | 1639382 |
| HILIC_POS_520_34064a1_15 | 1.52E-01 | -0.35 | 0.91 | 4.29E-02 | -0.36 | 1.19 | 4.32E-02 | -0.53 | 0.92 | 5.01E-01 | -0.17 | 0.40 | 33603138 | 21239096 | 27713055 | 14783984 | 30697598 | 14622840 | 24728511 | 14477115 |
| HILIC_POS_118_08664a3_699 | 3.95E-03 | 0.40 | 0.92 | 4.30E-02 | 0.45 | 1.28 | 3.61E-03 | 0.63 | 1.09 | 1.78E-01 | 0.18 | 0.46 | 1273942568 | 475380563 | ######### | ######### | 1459132488 | 758423818 | 1921096284 | ######### |
| RP_POS_285_07895a8_02 | 1.11E-03 | 0.43 | 1.09 | 4.35E-02 | 0.45 | 1.60 | 2.27E-03 | 0.66 | 1.41 | 8.86E-02 | 0.21 | 0.66 | 11402119 | 32370412 | 48986848 | 97019506 | 29371300 | 61578263 | 68602397 | 120168798 |
| HILIC_POS_132_0768a5_591 | 1.24E-01 | 0.29 | 0.55 | 4.36E-02 | -0.41 | 1.41 | 6.89E-01 | 0.09 | 0.46 | 1.90E-02 | 0.50 | 0.91 | 70737110 | 43324146 | 85010278 | 49894417 | 95051813 | 46875568 | 74968743 | 51250478 |
| RP_POS_790_57318a13_242 | 1.12E-05 | -0.89 | 1.58 | 4.37E-02 | -0.38 | 1.27 | 9.99E-07 | -1.08 | 1.82 | 1.36E-03 | -0.70 | 1.21 | 6139808 | 1553615 | 4596776 | 1618897 | 4922485 | 1700229 | 4271068 | 1479142 |
| RP_POS_746_56818a13_32 | 3.13E-01 | 0.19 | 0.62 | 4.38E-02 | 0.41 | 1.19 | 7.09E-02 | 0.40 | 0.78 | 9.50E-01 | -0.01 | 0.14 | 7128338 | 2260132 | 7611328 | 2567856 | 7094923 | 2426857 | 8127734 | 2624589 |
| RP_NEG_588_33154a10_199 | 2.10E-01 | 0.27 | 0.76 | 4.52E-02 | -0.39 | 1.57 | 7.69E-01 | 0.07 | 0.40 | 4.06E-02 | 0.46 | 0.94 | 10870500 | 3867576 | 11852951 | 3614053 | 12575463 | 3118238 | 11130440 | 3949753 |
| RP_NEG_538_31592a9_984 | 1.96E-17 | 1.08 | 1.92 | 4.60E-02 | 0.40 | 1.26 | 1.69E-09 | 1.28 | 1.97 | 1.07E-11 | 0.88 | 1.98 | 3969272 | 1490259 | 10898932 | 6465544 | 9607670 | 4454467 | 12190194 | 7823976 |
| RP_NEG_750_53046a12_847 | 1.56E-03 | 0.63 | 1.11 | 4.76E-02 | 0.39 | 1.24 | 4.62E-04 | 0.83 | 1.30 | 3.10E-02 | 0.44 | 0.79 | 1799787 | 1106129 | 2559304 | 1176475 | 2326573 | 965534 | 2792034 | 1324251 |
| RP_NEG_824_54694a12_579 | 8.72E-07 | 0.90 | 1.59 | 4.78E-02 | 0.39 | 1.20 | 2.19E-06 | 1.09 | 1.67 | 6.69E-05 | 0.70 | 1.51 | 4284188 | 1700215 | 6301584 | 2193104 | 5867254 | 1522576 | 6735914 | 2648461 |
| RP_POS_448_34177a9_121 | 5.96E-11 | 0.97 | 1.63 | 4.81E-02 | 0.40 | 1.62 | 3.57E-09 | 1.17 | 1.86 | 3.59E-06 | 0.78 | 1.59 | 362310 | 219099 | 789495 | 441156 | 702478 | 397058 | 876512 | 469149 |
| RP_POS_146_11719a0_566 | 8.53E-01 | 0.03 | 0.68 | 4.81E-02 | -0.42 | 1.34 | 4.01E-01 | -0.18 | 0.83 | 1.97E-01 | 0.24 | 0.90 | 4981390 | 1143859 | 5029489 | 1623826 | 5349850 | 1402946 | 4709128 | 1774746 |
| HILIC_POS_229_11833a6_131 | 6.49E-01 | -0.09 | 0.79 | 4.86E-02 | -0.41 | 1.14 | 1.63E-01 | -0.29 | 1.02 | 6.04E-01 | 0.12 | 0.67 | 1254657 | 714354 | 1185899 | 816997 | 1346739 | 876914 | 1025058 | 725822 |
| RP_NEG_824_54712a12_695 | 2.08E-03 | 0.61 | 1.21 | 4.94E-02 | -0.39 | 1.22 | 5.43E-02 | 0.41 | 0.85 | 3.24E-04 | 0.80 | 1.31 | 32791967 | 13192168 | 41645017 | 14464217 | 44481186 | 14340857 | 38808848 | 14164059 |
| RP_NEG_826_56226a13_071 | 3.04E-01 | 0.22 | 0.51 | 5.64E-02 | -0.38 | 1.15 | 9.03E-01 | 0.03 | 0.62 | 6.65E-02 | 0.41 | 0.83 | 97630208 | 17676945 | 101308268 | 16658455 | 104485244 | 13470099 | 98131293 | 18937522 |
| RP_POS_480_34473a10_699 | 7.83E-02 | -0.40 | 1.01 | 5.84E-02 | -0.36 | 1.71 | 2.00E-02 | -0.58 | 1.07 | 3.58E-01 | -0.22 | 0.80 | 24664467 | 8272414 | 21752908 | 6887758 | 23054488 | 6652345 | 20451328 | 6938266 |
| RP_POS_802_53503a12_693 | 3.75E-03 | 0.58 | 1.22 | 5.86E-02 | -0.37 | 1.18 | 6.92E-02 | 0.40 | 0.85 | 6.45E-04 | 0.77 | 1.29 | 50431049 | 22187141 | 64247045 | 23305019 | 68648086 | 22959866 | 59846004 | 23035790 |
| RP_NEG_538_31543a10_125 | 3.43E-19 | 1.18 | 2.00 | 5.89E-02 | 0.37 | 1.22 | 9.36E-11 | 1.37 | 2.07 | 4.20E-13 | 1.00 | 2.09 | 33339917 | 11811190 | 78152681 | 36966331 | 71162082 | 26517576 | 85143280 | 44249223 |
| RP_POS_782_56714a13_72 | 4.35E-08 | 0.94 | 1.54 | 5.91E-02 | 0.37 | 1.13 | 5.43E-09 | 1.12 | 1.90 | 1.75E-04 | 0.75 | 1.38 | 137918081 | 23555194 | 170752270 | 34350960 | 164274492 | 37061767 | 177230047 | 30411312 |
| RP_POS_363_21646a7_048 | 8.94E-02 | -0.43 | 2.00 | 6.02E-02 | 0.32 | 1.13 | 3.02E-01 | -0.27 | 1.18 | 2.87E-02 | -0.59 | 1.53 | 11348473 | 5945582 | 9399844 | 3840044 | 8679111 | 3848208 | 10120577 | 3731487 |
| SCFA_Acetic acid | 8.11E-05 | 0.47 | 0.93 | 6.27E-02 | 0.42 | 1.37 | 6.83E-04 | 0.68 | 1.08 | 4.29E-02 | 0.26 | 0.69 | 683499 | 304851 | 3458037 | 6692952 | 2213096 | 5191490 | 4728386 | 7789915 |
| RP_POS_160_13326a2_925 | 1.30E-01 | 0.18 | 0.65 | 6.32E-02 | 0.43 | 1.25 | 8.77E-02 | 0.39 | 0.69 | 3.53E-01 | -0.03 | 0.57 | 2839223 | 3829715 | 6912062 | 25812476 | 2070246 | 3289444 | 11753879 | 35881776 |
| RP_POS_752_55762a14_271 | 9.28E-01 | -0.02 | 0.35 | 6.37E-02 | -0.39 | 1.11 | 2.73E-01 | -0.21 | 0.59 | 4.51E-01 | 0.18 | 0.43 | 7183164 | 3729739 | 7111071 | 4433800 | 7933899 | 5229530 | 6288244 | 3314913 |
| RP_POS_384_08264a6_157 | 2.97E-03 | 0.46 | 0.74 | 6.51E-02 | 0.40 | 1.15 | 3.49E-03 | 0.66 | 1.00 | 6.58E-02 | 0.26 | 0.85 | 20031232 | 10627979 | 28144547 | 18827616 | 24661018 | 11410497 | 31628075 | 23690247 |
| RP_POS_806_56885a12_958 | 2.18E-06 | -0.86 | 1.68 | 6.51E-02 | -0.36 | 1.16 | 1.01E-06 | -1.04 | 1.87 | 5.14E-04 | -0.68 | 1.35 | 318435243 | 57973748 | 252580794 | 75168567 | 266430165 | 70770947 | 238731424 | 77562707 |
| RP_POS_152_07077a2_588 | 4.01E-02 | 0.24 | 0.44 | 6.73E-02 | -0.42 | 1.23 | 2.92E-01 | 0.03 | 0.36 | 5.07E-02 | 0.45 | 0.55 | 364824 | 1076657 | 3175612 | 13386174 | 5648548 | 18609003 | 702676 | 1805763 |
| RP_POS_225_19606a7_999 | 5.79E-01 | 0.12 | 0.79 | 6.90E-02 | -0.35 | 1.21 | 8.10E-01 | -0.06 | 0.59 | 2.27E-01 | 0.30 | 0.68 | 3974241 | 1891928 | 4182909 | 1673631 | 4487083 | 1800031 | 3878736 | 1493423 |

**Supplementary table 1: Metabolomics results** (in asceding order according to p-value from progressive ALD vs  non-progressive ALD comparison, with molecular features with ID_level of 1-2 first)

| Feature_ID | RP_HILIC | POS_NEG | Average.Rt | Average.Mz | Metabolite.name | curated_MSMS | ID_level | all_ALD_vs_control t-test p | all_ALD_vs_control Cohen's d | all_ALD_vs_control PLS-DA VIP | Prog_ALD_vs_non-prog_ALD t-test p | Prog_ALD_vs_non-prog_ALD Cohen's d | Prog_ALD_vs_non-prog_ALD PLS-DA VIP | Prog_ALD_vs_control_ALD t-test p | Prog_ALD_vs_control_ALD Cohen's d | Prog_ALD_vs_control_ALD PLS-DA VIP | Non-prog_ALD_vs_control t-test p | Non-prog_ALD_vs_control Cohen's d | Non-prog_ALD_vs_control PLS-DA VIP | Healthy_control Mean | Healthy_control SD | all_ALD Mean | all_ALD SD | Non-prog_ALD Mean | Non-prog_ALD SD | Progressive_ALD Mean | Progressive_ALD SD |
|---|---|---|---|---|---|---|---|---|---|---|---|---|---|---|---|---|---|---|---|---|---|---|---|---|---|---|---|
| RP_POS_808_58398a13_996 | RP | POS | 14.00 | 808.5840 | PC 36:2 [M+Na]+ | 146.9816(100), 86.0968(50), 625.5158(35), 808.5881(30), 749.5114(20), 603.5292(12) | 2 | 2.75E-02 | -0.49 | 0.93 | 7.30E-02 | 0.34 | 1.16 | 1.66E-01 | -0.32 | 0.78 | 8.24E-03 | -0.66 | 1.05 | 144384054 | 35035668 | 128654819 | 30721124 | 123147605 | 33371798 | 134162034 | 27041860 |
| RP_POS_786_5993a13_998 | RP | POS | 14.00 | 786.5993 | PC 36:2\|PC 18:0_18:2 | 184.0732(100), 786.5901(2) | 2 | 6.72E-02 | -0.44 | 0.88 | 7.43E-02 | 0.32 | 1.17 | 2.68E-01 | -0.28 | 0.72 | 1.95E-02 | -0.60 | 0.94 | 351706188 | 103721029 | 314465290 | 75224639 | 301046862 | 72260900 | 327883718 | 76445287 |
| RP_POS_768_58923a13_584 | RP | POS | 13.58 | 768.5892 | PC O-36:4\|PC O-16:0_20:4 | 184.0735(100), 768.5784(3) | 2 | 3.81E-02 | -0.40 | 0.72 | 7.61E-02 | -0.36 | 1.05 | 3.86E-03 | -0.58 | 1.10 | 3.49E-01 | -0.22 | 0.44 | 48776751 | 12803373 | 43061071 | 14623388 | 45658311 | 17089862 | 40463831 | 11234814 |
| RP_NEG_854_59454a13_547 | RP | NEG | 13.55 | 854.5945 | PC 38:4\|PC 18:2_20:2 [M+HCOO]- | 305.2496(100), 279.2349(56), 794.5665(27), 112.9847(50) | 2 | 3.81E-01 | 0.19 | 0.50 | 7.73E-02 | 0.35 | 1.05 | 1.34E-01 | 0.36 | 0.57 | 9.57E-01 | 0.01 | 0.62 | 8015170 | 2517849 | 8460242 | 2352571 | 8044654 | 2155254 | 8875830 | 2486872 |
| RP_POS_396_31055a8_634 | RP | POS | 8.63 | 396.3106 | CAR 16:2 | 85.0287(100), 396.3098(75) | 1 | 8.44E-01 | -0.04 | 0.88 | 7.91E-02 | -0.37 | 1.81 | 2.16E-01 | -0.22 | 0.87 | 5.37E-01 | 0.15 | 0.81 | 623133 | 365171 | 607586 | 448948 | 686685 | 562543 | 528486 | 279282 |
| RP_POS_836_61517a14_022 | RP | POS | 14.02 | 836.6152 | PC 40:5\|PC 20:1_20:4 | 184.0732(100), 836.6042(2) | 2 | 4.88E-06 | 0.80 | 1.35 | 7.98E-02 | 0.35 | 1.30 | 1.03E-05 | 0.97 | 1.53 | 4.89E-04 | 0.62 | 1.25 | 12909805 | 3994816 | 17361747 | 5616739 | 16376770 | 4482236 | 18346723 | 6455150 |
| RP_POS_752_52142a12_164 | RP | POS | 12.16 | 752.5214 | PC 34:5 | 184.0732(100), 752.5291(3) | 2 | 6.78E-02 | 0.29 | 0.96 | 8.03E-02 | -0.38 | 1.05 | 5.79E-01 | 0.10 | 0.70 | 2.00E-02 | 0.48 | 0.79 | 1912517 | 995420 | 2367099 | 1718067 | 2667636 | 1872980 | 2066563 | 1507103 |
| RP_POS_589_48016a14_415 | RP | POS | 14.42 | 589.4802 | DG 32:1 [M+Na]+ | 589.4122(100) | 2 | 2.30E-03 | 0.36 | 0.81 | 8.26E-02 | 0.40 | 1.03 | 1.38E-02 | 0.56 | 0.82 | 4.69E-03 | 0.17 | 0.86 | 1053388 | 805056 | 2870563 | 5646389 | 1882594 | 1741226 | 3858532 | 7706503 |
| RP_POS_437_323a11_2 | RP | POS | 11.20 | 437.3230 | DG 21:0 [M+Na]+ | 437.3226(100), 96.9217(6), 232.8979(3) | 2 | 2.16E-06 | 0.56 | 1.22 | 8.73E-02 | -0.38 | 1.41 | 9.45E-04 | 0.37 | 1.37 | 3.14E-04 | 0.75 | 1.34 | 186157 | 84912 | 1967283 | 3535936 | 2573735 | 4352974 | 1360832 | 2358366 |
| RP_POS_218_13847a0_71 | RP | POS | 0.71 | 218.1385 | CAR 03:0 (Propionylcarnitine) | 85.0288(100), 218.1379(60), 159.0644(15), 60.0816(13), | 1 | 9.97E-01 | 0.00 | 0.76 | 8.81E-02 | -0.36 | 1.10 | 3.78E-01 | -0.18 | 0.48 | 3.72E-01 | 0.18 | 0.74 | 4835877 | 1610774 | 4834366 | 2190131 | 5207936 | 2126468 | 4460796 | 2210105 |
| RP_POS_147_0766a0_525 | RP | POS | 0.53 | 147.0766 | Glutamine | 130.0501(100), 84.0446(80), 147.0766(8), 101.0714(8), 102.055(5) | 1 | 7.19E-01 | 0.05 | 0.69 | 8.91E-02 | -0.38 | 1.09 | 4.89E-01 | -0.13 | 0.48 | 1.66E-01 | 0.24 | 0.82 | 8636320 | 1416022 | 8771704 | 2803172 | 9248586 | 2544964 | 8294823 | 2989139 |
| RP_POS_279_17035a4_44 | RP | POS | 4.44 | 279.1704 | Phenylalanylisoleucine | 120.0814(100), 279.1688(5) | 2 | 1.34E-04 | 0.45 | 1.19 | 9.06E-02 | 0.38 | 1.38 | 2.58E-03 | 0.64 | 1.30 | 1.06E-02 | 0.26 | 1.21 | 309627 | 56920 | 3092948 | 7001992 | 1903257 | 4239362 | 4282640 | 8842831 |
| RP_NEG_498_26358a10_049 | RP | NEG | 10.05 | 498.2636 | LPE 20:5 | 301.2183(100), 196.0374(33), 498.2665(25), 78.9574(18), 214.0472(9) | 2 | 1.51E-05 | 0.69 | 1.30 | 9.27E-02 | -0.35 | 0.99 | 2.74E-03 | 0.52 | 1.19 | 3.23E-05 | 0.87 | 1.43 | 2757964 | 1983553 | 4970776 | 3327237 | 5530700 | 3686656 | 4410852 | 2852311 |
| RP_NEG_748_53003a13_52 | RP | NEG | 13.52 | 748.5300 | PE O-38:6\|PE O-16:1_22:5 | 303.2315(100), 748.5336(17), 462.298(13) | 2 | 9.29E-01 | -0.02 | 0.67 | 9.33E-02 | -0.35 | 0.99 | 3.60E-01 | -0.19 | 0.41 | 4.70E-01 | 0.16 | 0.74 | 3724098 | 1252949 | 3700310 | 1488718 | 3950268 | 1547911 | 3450352 | 1397947 |
| RP_POS_290_15967a2_242 | RP | POS | 2.24 | 290.1597 | Adipoyl-L-carnitine | 290.1582(100), 85.0287(75), 129.0542(35), | 2 | 7.56E-04 | 0.39 | 0.91 | 9.40E-02 | 0.38 | 1.13 | 6.14E-03 | 0.59 | 0.90 | 3.36E-02 | 0.20 | 0.69 | 144185 | 263519 | 2252546 | 6049336 | 1234762 | 3514193 | 3270330 | 7711819 |
| RP_POS_800_51837a12_377 | RP | POS | 12.38 | 800.5184 | PC 36:6 | 146.9818(100), 86.0968(85), 617.453(80), 741.4485(50), 800.5135(40), 595.4698(13) | 2 | 6.99E-02 | -0.37 | 1.15 | 9.52E-02 | -0.33 | 1.02 | 1.41E-02 | -0.54 | 1.31 | 3.81E-01 | -0.21 | 0.51 | 1734074 | 918196 | 1388579 | 923368 | 1542813 | 1019718 | 1234345 | 796394 |
| RP_POS_307_11127a4_916 | RP | POS | 4.92 | 307.1113 | Fluconazole | 220.0664(100), 238.0788(95), 73.0652(50), 307.1103(50), 169.046(40) | 2 | 5.03E-01 | 0.10 | 1.12 | 9.69E-02 | -0.37 | 1.25 | 4.85E-01 | -0.09 | 0.58 | 2.13E-01 | 0.28 | 1.10 | 8241731 | 6150336 | 9366239 | 12746109 | 11494257 | 16618577 | 7238222 | 6543953 |
| RP_NEG_309_28049a11_691 | RP | NEG | 11.69 | 309.2805 | FA 20:1 | 309.2805(100) | 2 | 1.08E-08 | 0.88 | 1.72 | 9.78E-02 | 0.34 | 1.20 | 3.73E-07 | 1.05 | 1.76 | 1.62E-05 | 0.71 | 1.66 | 610296 | 367575 | 1194941 | 673196 | 1083337 | 565645 | 1306544 | 755157 |
| RP_POS_806_56622a13_401 | RP | POS | 13.40 | 806.5662 | PC 36:3 [M+Na]+ | 146.9818(100), 86.0969(55), 623.5042(40), 806.5659(30), 747.4948(20), 601.5248(12) | 2 | 8.74E-01 | 0.04 | 0.45 | 9.79E-02 | -0.30 | 1.11 | 6.57E-01 | -0.11 | 0.47 | 4.54E-01 | 0.19 | 0.61 | 88907602 | 22752977 | 89606398 | 16904216 | 92406024 | 16315726 | 86806772 | 17179583 |
| RP_POS_500_27722a10_049 | RP | POS | 10.05 | 500.2772 | LPE 20:5 | 359.2576(100), 62.0608(80), 500.2825(7) | 2 | 3.24E-05 | 0.67 | 1.28 | 9.80E-02 | -0.35 | 0.98 | 4.50E-03 | 0.49 | 1.16 | 5.62E-05 | 0.84 | 1.40 | 4463281 | 3374428 | 8053286 | 5620557 | 8984339 | 6204516 | 7122233 | 4853958 |
| SCFA_Propionic acid | SCFA | SCFA | | | FA 03:0 (Propionic acid) | targeted_SCFA_analysis | 1 | 5.25E-04 | 0.82 | 1.34 | 1.03E-01 | -0.61 | 2.12 | 6.95E-02 | 0.52 | 1.16 | 8.22E-04 | 1.14 | 1.81 | 64210 | 19974 | 102246 | 49802 | 116877 | 49285 | 88476 | 47620 |
| RP_NEG_773_58307a12_915 | RP | NEG | 12.92 | 773.5831 | SM 36:2;2O [M+HCOO]- | 713.558(100), 168.042(80), 78.9573(65) | 2 | 5.29E-05 | -0.98 | 1.66 | 1.05E-01 | -0.27 | 1.04 | 2.31E-05 | -1.11 | 1.62 | 4.38E-04 | -0.84 | 1.61 | 3993036 | 1320025 | 2857256 | 961379 | 3013473 | 779715 | 2701039 | 1099819 |
| RP_NEG_498_29181a9_012 | RP | NEG | 9.01 | 498.2918 | BA 24:1;O3;T | 498.2869(100) | 2 | 2.29E-01 | 0.15 | 1.00 | 1.06E-01 | 0.37 | 1.11 | 1.37E-01 | 0.34 | 0.82 | 7.25E-01 | -0.03 | 0.52 | 1005164 | 725715 | 1338747 | 2440956 | 941266 | 904064 | 1736229 | 3301267 |
| RP_POS_760_58386a14_257 | RP | POS | 14.26 | 760.5839 | PC 34:1 | 184.0729(100), 760.5709(3) | 2 | 2.46E-04 | 0.56 | 0.94 | 1.08E-01 | 0.35 | 0.97 | 7.22E-04 | 0.73 | 1.11 | 1.14E-02 | 0.39 | 0.95 | 4780197 | 3049904 | 7759241 | 5681084 | 6842537 | 4140477 | 8675946 | 6808052 |
| HILIC_NEG_151_02615a1_655 | HILIC | NEG | 1.66 | 151.0262 | Xanthine | 151.0262(100), 108.0202(35) | 2 | 4.61E-11 | 0.81 | 1.25 | 1.09E-01 | 0.35 | 1.18 | 9.90E-07 | 0.98 | 1.50 | 4.81E-06 | 0.64 | 1.51 | 162912 | 52861 | 443926 | 373197 | 383947 | 300214 | 503905 | 428863 |
| HILIC_POS_177_10217a1_624 | HILIC | POS | 1.624 | 177.1022 | Serotonin | 159.95.10(100) | 1 | 9.65E-03 | 0.31 | 0.80 | 1.11E-01 | -0.36 | 1.04 | 1.53E-01 | 0.13 | 1.02 | 2.40E-02 | 0.49 | 0.95 | 88995 | 91959 | 246301 | 574894 | 338491 | 749945 | 154111 | 296627 |
| RP_NEG_733_55182a12_477 | RP | NEG | 12.48 | 733.5518 | SM 33:1;2O [M+HCOO]- | 673.5261(100), 168.042(80), 78.9574(60) | 2 | 4.02E-07 | -1.21 | 2.08 | 1.13E-01 | -0.25 | 0.93 | 6.56E-08 | -1.34 | 2.18 | 1.03E-05 | -1.09 | 1.77 | 2724529 | 900097 | 1692107 | 664593 | 1797552 | 758457 | 1586663 | 542619 |
| RP_POS_271_06332a7_721 | RP | POS | 7.72 | 271.0633 | Nordiazepam | 271.0631(100) | 2 | 2.83E-01 | 0.14 | 0.71 | 1.13E-01 | 0.36 | 1.17 | 1.62E-01 | 0.32 | 0.95 | 5.95E-01 | -0.04 | 0.74 | 56642121 | 51101091 | 78224786 | 178879406 | 49685995 | 66279471 | 106763577 | 242061076 |
| RP_POS_583_25439a8_27 | RP | POS | 8.27 | 583.2544 | Biliverdin | 297.1203(100), 583.2581(85) | 2 | 2.00E-02 | 0.35 | 0.92 | 1.16E-01 | 0.34 | 1.48 | 1.71E-02 | 0.52 | 0.90 | 2.30E-01 | 0.18 | 0.79 | 1087878 | 781914 | 1574386 | 1526217 | 1333894 | 1056215 | 1814879 | 1863489 |
| HILIC_NEG_103_03985a0_701 | HILIC | NEG | 0.70 | 103.0399 | 3-Hydroxyisobutyric acid | 73.0286(100), 59.0131(30), 101.9237(25), 103.0403(10) | 2 | 5.86E-04 | 0.41 | 0.84 | 1.18E-01 | 0.36 | 1.28 | 1.05E-02 | 0.59 | 0.83 | 3.15E-05 | 0.23 | 1.33 | 3215470 | 3581770 | 15549168 | 34209436 | 10153756 | 10025035 | 20944581 | 46952572 |
| RP_NEG_717_51996a11_856 | RP | NEG | 11.86 | 717.5200 | SM 32:2;2O [M+HCOO]- | 657.504(100), 168.0422(85), 78.9574(70) | 2 | 2.19E-05 | -1.01 | 1.85 | 1.18E-01 | -0.26 | 0.93 | 5.47E-06 | -1.14 | 1.84 | 3.28E-04 | -0.88 | 1.56 | 2759531 | 911828 | 1924897 | 683868 | 2031919 | 707632 | 1817876 | 648674 |
| RP_POS_804_54791a13_069 | RP | POS | 13.07 | 804.5479 | PC 36:4 [M+Na]+ | 146.9817(100), 86.0968(65), 621.4869(60), 745.4814(30), 804.5549(30), | 2 | 9.19E-01 | -0.02 | 0.49 | 1.19E-01 | -0.32 | 0.94 | 4.38E-01 | -0.18 | 0.81 | 5.29E-01 | 0.14 | 0.45 | 146049367 | 21525980 | 145597052 | 22313269 | 149085560 | 20688795 | 142108544 | 23517281 |
| RP_POS_182_08159a0_709 | RP | POS | 0.71 | 182.0816 | Tyrosine | 136.0752(100), 165.0546(50), 123.0443(30), 147.0445(17), 119.0495(15), 182.081(8) | 2 | 3.33E-01 | 0.17 | 0.92 | 1.20E-01 | 0.33 | 1.07 | 1.30E-01 | 0.33 | 1.18 | 9.99E-01 | 0.00 | 0.55 | 75858953 | 16786033 | 79563368 | 23718637 | 75862569 | 19460052 | 83264166 | 27018219 |
| RP_NEG_303_23337a10_942 | RP | NEG | 10.94 | 303.2334 | FA 20:4 (Arachidonic acid, AA) | 303.2318(100) | 2 | 9.14E-08 | 0.88 | 1.70 | 1.21E-01 | 0.31 | 1.01 | 6.13E-07 | 1.04 | 1.74 | 4.43E-05 | 0.73 | 1.61 | 16296802 | 7773446 | 27066049 | 12218835 | 25167919 | 10767073 | 28964179 | 13355301 |
| HILIC_NEG_89_02393a1_301 | HILIC | NEG | 1.30 | 89.0239 | D-Lactic acid | 89.024(100) | 2 | 5.25E-11 | 0.91 | 1.54 | 1.22E-01 | 0.32 | 0.97 | 5.97E-08 | 1.07 | 1.67 | 3.24E-06 | 0.75 | 1.49 | 152280210 | 52610387 | 268871847 | 132788175 | 248289869 | 117509763 | 289453825 | 144748676 |
| RP_POS_325_17111a5_597 | RP | POS | 5.60 | 325.1711 | Escitalopram | 325.1696(100), 109.045(50), 262.1021(20) | 2 | 7.02E-02 | 0.31 | 0.79 | 1.23E-01 | 0.33 | 1.08 | 3.00E-02 | 0.47 | 1.22 | 4.23E-01 | 0.15 | 0.44 | 3727720 | 2896165 | 4935992 | 4130825 | 4296639 | 3432489 | 5575345 | 4675411 |
| RP_POS_290_15964a2_595 | RP | POS | 2.60 | 290.1596 | Adipoyl-L-carnitine | 290.1571(100), 85.0291(85), 129.0557(40), 101.0242(30), 60.0808(18) | 1 | 1.34E-03 | 0.38 | 0.82 | 1.24E-01 | 0.35 | 1.04 | 7.23E-03 | 0.55 | 0.87 | 7.57E-02 | 0.20 | 0.63 | 195902 | 326727 | 1915502 | 5182397 | 1114908 | 3560055 | 2716097 | 6346695 |
| RP_POS_812_61475a14_294 | RP | POS | 14.29 | 812.6148 | PC 38:3\|PC 18:0_20:3 | 184.0732(100), 812.6267(2) | 2 | 2.31E-01 | 0.23 | 0.60 | 1.25E-01 | 0.31 | 0.93 | 1.01E-01 | 0.39 | 0.70 | 7.01E-01 | 0.08 | 0.59 | 71201816 | 26309920 | 77847060 | 29097579 | 73375800 | 22584513 | 82318321 | 34055199 |
| RP_POS_368_27936a8_076 | RP | POS | 8.08 | 368.2794 | CAR 14:2 | 85.0288(100), 368.2795(65) | 2 | 2.69E-01 | -0.20 | 0.93 | 1.26E-01 | -0.32 | 1.63 | 3.55E-02 | -0.36 | 1.09 | 8.78E-01 | -0.04 | 0.75 | 4088666 | 2344123 | 3515617 | 3038680 | 3982876 | 3896342 | 3048357 | 1740271 |
| RP_POS_136_07573a0_71 | RP | POS | 0.71 | 136.0757 | fragment of tyrosine | 136.0752(100), 119.0495(55), 91.0544(70) | 2 | 4.49E-01 | 0.13 | 0.92 | 1.27E-01 | 0.33 | 1.02 | 1.80E-01 | 0.29 | 1.14 | 8.50E-01 | -0.03 | 0.57 | 8547988 | 1843248 | 8867594 | 2651282 | 8462445 | 2190009 | 9272743 | 3011797 |
| RP_POS_832_58313a13_081 | RP | POS | 13.08 | 832.5831 | PC 40:7 | 184.0732(100), 832.5811(3) | 2 | 5.66E-04 | -0.63 | 1.22 | 1.28E-01 | 0.31 | 0.97 | 3.37E-02 | -0.47 | 0.83 | 4.48E-05 | -0.78 | 1.54 | 17554628 | 4846216 | 13730096 | 6164731 | 12789913 | 4755761 | 14670279 | 7236171 |
| RP_POS_751_5708a12_913 | RP | POS | 12.91 | 751.5708 | SM 36:2;2O [M+Na]+ | 568.4996(100), 692.4962(60), 751.5734(40), 86.0967(50), 146.9814(42) | 2 | 2.63E-05 | -0.98 | 1.62 | 1.30E-01 | -0.26 | 0.98 | 1.28E-05 | -1.11 | 1.65 | 2.69E-04 | -0.85 | 1.53 | 3784177 | 1274375 | 2626183 | 1006566 | 2779094 | 859235 | 2473271 | 1122953 |
| RP_NEG_568_3634a10_934 | RP | NEG | 10.93 | 568.3634 | LPC 18:0 | 283.2653(100), 508.3423(30), 224.0689(5) | 2 | 7.69E-02 | -0.42 | 1.11 | 1.32E-01 | -0.27 | 1.62 | 3.29E-02 | -0.56 | 1.06 | 2.44E-01 | -0.29 | 0.91 | 171698079 | 45305571 | 155959303 | 33449538 | 161008780 | 30036375 | 150909825 | 36143579 |
| RP_POS_754_53674a12_465 | RP | POS | 12.47 | 754.5367 | PC 34:4\|PC 14:0_20:4 | 184.0732(100), 754.5454(2), | 2 | 4.88E-01 | 0.15 | 0.64 | 1.32E-01 | -0.30 | 0.91 | 9.98E-01 | 0.00 | 0.86 | 2.04E-01 | 0.30 | 0.58 | 12099324 | 6464103 | 13001357 | 5999655 | 13906761 | 5810408 | 12095953 | 6106760 |
| RP_POS_144_1019a0_615 | RP | POS | 0.62 | 144.1019 | Proline betaine | 144.1012(100) | 1 | 3.96E-01 | 0.15 | 1.18 | 1.34E-01 | 0.32 | 1.22 | 1.53E-01 | 0.31 | 1.27 | 9.41E-01 | -0.01 | 0.69 | 30747980 | 24502014 | 35473216 | 34475586 | 30292836 | 30526211 | 40653595 | 37613441 |
| RP_POS_746_604a14_36 | RP | POS | 14.36 | 746.6040 | PC O-34:1 | 184.0732(100), 746.6037(3) | 2 | 3.99E-02 | -0.38 | 0.78 | 1.37E-01 | 0.31 | 0.97 | 3.14E-01 | -0.22 | 0.52 | 7.32E-03 | -0.53 | 1.08 | 5499385 | 2075304 | 4560352 | 2566848 | 4177269 | 2169531 | 4943436 | 2881929 |
| RP_NEG_307_2652a11_385 | RP | NEG | 11.39 | 307.2652 | FA 20:2 | 307.263(100) | 2 | 3.79E-10 | 0.91 | 1.76 | 1.40E-01 | 0.30 | 1.15 | 1.09E-07 | 1.06 | 1.81 | 4.23E-06 | 0.76 | 1.66 | 721152 | 378162 | 1446416 | 818870 | 1325126 | 721777 | 1567705 | 896546 |
| HILIC_POS_160_13289a2_098 | HILIC | POS | 2.10 | 160.1329 | 5-AVAB | 160.1328(100), 101.0603(30), 60.0815(20) | 1 | 2.29E-04 | 0.56 | 0.99 | 1.44E-01 | 0.31 | 0.92 | 3.83E-04 | 0.72 | 1.20 | 1.88E-02 | 0.40 | 0.80 | 66495525 | 21776096 | 88074191 | 41316200 | 82022309 | 36803323 | 94126074 | 44940672 |
| RP_POS_730_53711a12_533 | RP | POS | 12.53 | 730.5371 | PC 32:2\|PC 16:1_16:1 | 184.0732(100), 730.5381(3) | 2 | 3.41E-01 | 0.19 | 0.74 | 1.45E-01 | 0.29 | 0.86 | 1.53E-01 | 0.34 | 0.80 | 8.34E-01 | 0.04 | 0.18 | 30342401 | 13380986 | 32974845 | 13840360 | 30951529 | 11810148 | 34998161 | 15465644 |
| RP_POS_810_59827a13_871 | RP | POS | 13.87 | 810.5983 | PC 38:4 + PC 36:1 [M+Na]+ | 146.9819(100), 86.0969(65), 649.5148(55), 773.5109(32), 832.5852(30), 627.5255(5) | 2 | 6.61E-01 | -0.09 | 0.51 | 1.48E-01 | 0.28 | 1.12 | 8.48E-01 | 0.05 | 0.49 | 2.90E-01 | -0.24 | 0.44 | 192144519 | 59791051 | 186902926 | 54375499 | 179019144 | 44288490 | 194786708 | 62333099 |
| RP_POS_786_59991a14_252 | RP | POS | 14.25 | 786.5999 | PC 36:2 | 184.0732(100), 786.5897(2) | 2 | 7.19E-01 | -0.08 | 0.35 | 1.51E-01 | 0.28 | 0.99 | 7.80E-01 | 0.07 | 0.18 | 3.49E-01 | -0.22 | 0.67 | 22208884 | 13236654 | 21250963 | 12360617 | 19471742 | 12137919 | 23030184 | 12445812 |
| RP_POS_299_13901a5_809 | RP | POS | 5.81 | 299.1390 | 3-Hydroxy-3',4'-Dimethoxyflavone | 299.1375(100) | 2 | 5.42E-01 | -0.08 | 0.61 | 1.51E-01 | 0.32 | 1.28 | 7.36E-01 | 0.08 | 0.39 | 1.27E-02 | -0.25 | 1.15 | 634447 | 356246 | 567875 | 887307 | 439404 | 298073 | 696347 | 1211678 |
| HILIC_POS_170_08121a0_885 | HILIC | POS | 0.89 | 170.0812 | Pyridoxine (Vitamin B6) | 152.0701(100), 170.0821(35), 134.0606(20) | 1 | 1.52E-01 | 0.17 | 0.77 | 1.53E-01 | 0.33 | 0.94 | 1.53E-01 | 0.33 | 0.76 | 5.98E-01 | 0.00 | 0.67 | 12697 | 27027 | 7231305 | 49965431 | 15694 | 21414 | 14446916 | 70269360 |
| RP_POS_256_01541a4_299 | RP | POS | 4.30 | 256.0154 | Lamotrigine | 256.0161(100) | 2 | 1.31E-02 | -0.43 | 0.70 | 1.54E-01 | 0.30 | 0.86 | 2.45E-01 | -0.28 | 0.43 | 2.60E-04 | -0.58 | 1.47 | 78735090 | 35582967 | 58391180 | 49334636 | 51301543 | 20112611 | 65480816 | 66410764 |
| RP_NEG_810_56763a13_375 | RP | NEG | 13.38 | 810.5676 | PC O-36:5\|PC O-16:1_20:4 [M+HCOO]- | 303.2352(100), 750.5476(28), 464.3197(16), 446.3023(5) | 2 | 9.07E-01 | -0.02 | 0.60 | 1.56E-01 | -0.30 | 0.89 | 4.14E-01 | -0.17 | 0.38 | 5.58E-01 | 0.13 | 0.48 | 6749295 | 1730732 | 6705597 | 2097411 | 7004093 | 2166605 | 6407102 | 2003004 |
| SCFA_Isovaleric acid | SCFA | SCFA | | | FA 05:0 (Isovaleric acid) | targeted_SCFA_analysis | 1 | 2.54E-02 | 0.31 | 0.57 | 1.58E-01 | -0.34 | 1.00 | 1.84E-01 | 0.13 | 0.64 | 4.69E-02 | 0.47 | 0.74 | 85573 | 88255 | 179884 | 342948 | 230153 | 449977 | 125846 | 153145 |
| RP_POS_804_55029a12_821 | RP | POS | 12.82 | 804.5503 | PC 36:4 [M+Na]+ | 146.9823(100), 621.487(45), 804.5485(30), 745.4802(25), 599.5013(12) | 2 | 2.00E-06 | -1.15 | 1.84 | 1.58E-01 | -0.23 | 0.94 | 6.68E-07 | -1.26 | 1.93 | 2.36E-05 | -1.03 | 1.60 | 57199483 | 20287995 | 35818086 | 14838236 | 37918326 | 14777569 | 33717846 | 14746565 |
| HILIC_POS_130_1089a1_485 | HILIC | POS | 1.49 | 130.1089 | Metformin | 60.0567(100), 130.1095(100), 71.0604(40), 85.0518(25), 88.0869(25) | 2 | 8.20E-01 | -0.08 | 0.77 | 1.59E-01 | 0.14 | 0.98 | 9.81E-01 | -0.01 | 0.11 | 6.67E-01 | -0.14 | 0.53 | 34934212 | 114634732 | 30227260 | 29737331 | 26021555 | 23467144 | 34432966 | 34638996 |
| RP_POS_369_35065a13_296 | RP | POS | 13.30 | 369.3507 | Cholesterol | 369.3474(100),  161.1315(45), 147.1173(40), 135.1167(30), 95.0857(30), 109.1011(28), 81.0705(26), 175.1472(18), 121.1013(13), 215.1801(10), 203.1797(8), 243.2101(8), | 2 | 1.56E-01 | -0.26 | 0.73 | 1.60E-01 | 0.30 | 0.88 | 6.24E-01 | -0.11 | 0.47 | 3.95E-02 | -0.40 | 0.79 | 45463844 | 7507204 | 43107547 | 9661312 | 41746480 | 8303662 | 44468614 | 10763615 |
| HILIC_POS_143_08162a0_723 | HILIC | POS | 0.72 | 143.0816 | Piracetam | 98.0603(100), 126.055(45), 143.0815(5) | 2 | 1.61E-01 | 0.16 | 0.46 | 1.63E-01 | -0.32 | 0.83 | 7.27E-01 | 0.00 | 0.27 | 1.63E-01 | 0.32 | 0.42 | 298831 | 106519 | 5591106 | 37510234 | 10874710 | 52780343 | 307503 | 113783 |
| HILIC_POS_250_18013a0_867 | HILIC | POS | 0.87 | 250.1801 | N,O-Didesmethylvenlafaxine | 232.17(100), 250.18(70), 202.127(50), 133.0645(40), 107.0498(40) | 2 | 4.23E-02 | 0.24 | 0.76 | 1.64E-01 | -0.32 | 1.18 | 8.30E-03 | 0.08 | 0.86 | 8.59E-02 | 0.40 | 0.78 | 212291 | 829562 | 4820054 | 22349360 | 7958255 | 31236633 | 1681854 | 3654550 |
| RP_POS_524_37085a10_932 | RP | POS | 10.93 | 524.3709 | LPC 18:0 | 184.0724(100), 524.3712(33), 506.3652(8), 104.107(85) | 2 | 1.61E-01 | -0.34 | 1.01 | 1.66E-01 | -0.25 | 1.52 | 7.70E-02 | -0.46 | 0.89 | 3.96E-01 | -0.21 | 0.84 | 501365860 | 156623930 | 458681396 | 113229873 | 474399783 | 106128920 | 442963008 | 118895307 |
| RP_NEG_826_56268a12_823 | RP | NEG | 12.82 | 826.5627 | PC 36:4\|PC 18:2_18:2 [M+HCOO]- | 303.2315(100), 255.2332(60), 766.5427(25) | 2 | 5.95E-06 | -1.10 | 1.78 | 1.67E-01 | -0.22 | 0.92 | 2.26E-06 | -1.21 | 1.86 | 5.87E-05 | -0.99 | 1.55 | 37010478 | 12647643 | 24497051 | 9124625 | 25760597 | 8884156 | 23233505 | 9275249 |
| RP_POS_808_58411a13_2 | RP | POS | 13.20 | 808.5841 | PC 38:5\|PC 16:0_22:5 AND PC 18:1_20:4 | 184.0731(100), 808.5745(2) | 2 | 2.82E-03 | 0.58 | 1.03 | 1.70E-01 | 0.27 | 1.08 | 2.63E-03 | 0.72 | 1.11 | 2.19E-02 | 0.44 | 0.93 | 103754159 | 21961484 | 118086635 | 24579272 | 114697631 | 18012137 | 121475639 | 29542072 |
| RP_NEG_614_34747a10_447 | RP | NEG | 10.45 | 614.3475 | LPC 22:5 [M+HCOO]- | 329.2495(100), 554.3241(60), 224.0686(20) | 2 | 8.20E-04 | 0.64 | 1.29 | 1.70E-01 | -0.27 | 1.03 | 2.25E-02 | 0.50 | 1.00 | 2.00E-04 | 0.78 | 1.46 | 5655722 | 2979846 | 7878775 | 3473388 | 8356658 | 3119221 | 7400891 | 3765561 |
| RP_POS_143_08138a0_71 | RP | POS | 0.71 | 143.0814 | Piracetam | 98.0602(100), 126.0553(48), 70.0657(4), 143.0808(2) | 2 | 1.64E-01 | 0.16 | 0.58 | 1.73E-01 | -0.32 | 0.84 | 1.54E-01 | 0.00 | 0.65 | 1.69E-01 | 0.32 | 0.58 | 15124 | 8615 | 2131963 | 15092452 | 4210996 | 21245182 | 52930 | 184106 |
| RP_POS_818_60437a13_565 | RP | POS | 13.57 | 818.6044 | PC O-40:7 | 184.0732(100), 818.6068(2) | 2 | 4.01E-07 | -1.21 | 2.15 | 1.73E-01 | 0.21 | 1.12 | 5.57E-06 | -1.11 | 1.77 | 1.20E-07 | -1.32 | 2.07 | 10007238 | 2902091 | 6677769 | 2147171 | 6384658 | 2035009 | 6970881 | 2235660 |
| HILIC_POS_344_27966a0_66 | HILIC | POS | 0.66 | 344.2797 | AC 12:0 (Lauroylcarnitine) | 85.0287(100), 344.2782(50) | 1 | 8.50E-01 | 0.03 | 0.49 | 1.79E-01 | -0.28 | 1.13 | 5.56E-01 | -0.11 | 0.23 | 4.47E-01 | 0.18 | 0.35 | 7382375 | 4208704 | 7556936 | 5364383 | 8281883 | 6447651 | 6831990 | 3936463 |
| RP_POS_370_29504a8_374 | RP | POS | 8.37 | 370.2950 | CAR 14:1 | 85.0288(100), 370.2921(50) | 2 | 1.10E-08 | 0.81 | 1.50 | 1.80E-01 | 0.28 | 1.38 | 1.13E-07 | 0.95 | 1.77 | 2.46E-04 | 0.67 | 1.32 | 908317 | 739419 | 2226936 | 1701308 | 1997877 | 1768193 | 2455995 | 1616905 |

**Supplementary table 1: Metabolomics results** (in asceding order according to p-value from progressive ALD vs non-progressive ALD comparison, with molecular features with ID_level of 1-2 first)

| Feature_ID | RP_HILIC | POS_NEG | Average.Rt | Average.Mz | Metabolite.name | curated_MSMS | ID_level |
|---|---|---|---|---|---|---|---|
| RP_POS_810_59894a14_255 | RP | POS | 14.26 | 810.5989 | PC 38:4\|PC 18:0_20:4 | 184.0732(100), 810.5998(2) | 2 |
| RP_POS_130_08647a0_708 | RP | POS | 0.71 | 130.0865 | Pipecolinic acid | 84.0811(100), 130.0865(45) | 2 |
| RP_POS_150_0582a0_709 | RP | POS | 0.71 | 150.0582 | Methione | 104.053(100), 56.0503(55), 133.0317(55), 61.0114(22), 102.0557(22), 150.084(18) | 2 |
| RP_POS_760_58508a13_717 | RP | POS | 13.72 | 760.5851 | PC 34:1\|PC 16:0_18:1 | 184.0735(100), 760.5764(3) | 2 |
| RP_POS_346_1221a5_854 | RP | POS | 5.85 | 346.1221 | Omeprazole | 198.0595(100), 151.0994(55), 136.0752(20), 168.1014(20), 180.047(18), 149.0714(17), 179.0275(7( | 1 |
| RP_POS_633_45544a10_872 | RP | POS | 10.87 | 633.4554 | DG 36:7 | 633.457(100) | 2 |
| RP_POS_858_59827a14_026 | RP | POS | 14.03 | 858.5983 | PC 40:5 [M+Na]+ | 146.9818(100), 86.0969(60), 675.5334(40), 858.6024(30), 799.5298(23), | 2 |
| RP_POS_675_54248a12_21 | RP | POS | 12.21 | 675.5425 | SM 32:1;2O\|SM 16:1;2O/16:0 | 184.0731(100), 675.5374(1) | 2 |
| RP_POS_678_50568a12_274 | RP | POS | 12.27 | 678.5057 | PC 28:0 | 184.0732(100), 678.4957(4) | 2 |
| RP_POS_231_08333a0_709 | RP | POS | 0.71 | 231.0833 | Beta-Asarone | 231.0833(100) | 2 |
| RP_NEG_297_24417a9_953 | RP | NEG | 9.95 | 297.2442 | FA 18:1;O | 297.2419(100) | 2 |
| RP_POS_246_16994a3_08 | RP | POS | 3.08 | 246.1699 | Isovaleryl-L-carnitine | 85.0292(100), 246.1712(70), 187.0963(30), 60.0806(15) | 1 |
| RP_POS_570_35535a10_446 | RP | POS | 10.45 | 570.3554 | LPC 22:5 | 184.0735(100), 570.3572(25), 552.3459(6), 104.1067(75) | 2 |
| RP_NEG_566_34454a10_502 | RP | NEG | 10.50 | 566.3445 | LPC 18:1 [M+HCOO]- | 281.2505(100), 506.3257(18) | 2 |
| RP_POS_518_3241a9_905 | RP | POS | 9.91 | 518.3241 | LPC 18:3 (0:0/18:3) | 184.0733(100), 518.3215(5) | 2 |
| RP_NEG_786_56799a13_501 | RP | NEG | 13.50 | 786.5680 | PC O-34:3\|PC O-16:1_18:2 [M+HCOO]- | 279.2345(100), 762.5512(18), 464.3112(7), 446.3023(2) | 2 |
| RP_POS_134_06029a3_283 | RP | POS | 3.28 | 134.0603 | Indoxyl sulfate | 134.0598(100), 106.0653(13) | 2 |
| HILIC_NEG_151_02606a1_2 | HILIC | NEG | 1.20 | 151.0261 | Oxypurinol | 151.0262(100), 108.0202(3) | 2 |
| RP_POS_729_59027a12_915 | RP | POS | 12.92 | 729.5903 | SM 36:2;2O\|SM 18:2;2O/18:0 | 184.0735(100), 729.5819(1) | 2 |
| RP_POS_357_27863a9_858 | RP | POS | 9.86 | 357.2786 | Chenodeoxycholic acid | 357.2766(100), 135.1163(15), 161.1328(16), 147.1169(13) | 1 |
| RP_POS_344_2793a8_114 | RP | POS | 8.11 | 344.2793 | CAR 12:0 (Lauroycarnitine) | 85.0288(100), 344.2816(50) | 1 |
| RP_POS_391_28372a11_391 | RP | POS | 11.39 | 391.2837 | Dioctyl phthalate | 149.0228(100), 71.0858(15), 167.0348(13), 57.0706(11) | 2 |
| RP_POS_834_59705a14_323 | RP | POS | 14.32 | 834.5971 | PC 38:3 [M+Na]+ | 146.9816(100), 86.0967(60), 651.5278(45), 834.5889(35), 775.5125(27), 629.5432(8), 184.0726(60) | 2 |
| RP_POS_302_23242a6_351 | RP | POS | 6.35 | 302.2324 | CAR 09:0 | 85.0288(100), 302.2323(60) | 2 |
| RP_POS_782_56873a12_82 | RP | POS | 12.82 | 782.5687 | PC 36:4\|PC 18:2_18:2 | 146.9817(100), 86.0968(55), 595.4709(40), 778.5302(30), 719.4662(25), 573.4848(13), 184.0727(70) | 2 |
| RP_NEG_329_24921a11_049 | RP | NEG | 11.05 | 329.2492 | FA 22:5 (Docosapentaenoic acid) | 329.2497(100) | 2 |
| RP_POS_308_12555a8_419 | RP | POS | 8.42 | 308.1256 | Piperine [M+Na]+ | 308.124(100) | 2 |
| RP_POS_502_29254a10_216 | RP | POS | 10.22 | 502.2925 | LPE 20:4 | 361.2755(100), 502.4443(4), 81.0705(18) | 2 |
| RP_POS_766_57379a13_372 | RP | POS | 13.37 | 766.5738 | PC O-36:5\|PC O-16:1_20:4 | 184.0732(100), 766.5822(2) | 2 |
| RP_POS_583_25464a11_804 | RP | POS | 11.80 | 583.2546 | Biliverdin | 297.1208(100), 583.2571(70) | 2 |
| RP_POS_342_26376a7_71 | RP | POS | 7.71 | 342.2638 | CAR 12:1 | 85.0286(100), 342.2594(50) | 2 |
| RP_POS_287_05817a7_376 | RP | POS | 7.38 | 287.0582 | Oxazepam | 269.0463(100), 241.0513(90), 287.0583(85), 231.0684(10) | 2 |
| RP_NEG_498_29214a8_173 | RP | NEG | 8.17 | 498.2921 | BA 24:1;O3;T | 498.2869(100) | 2 |
| RP_NEG_856_60919a14_325 | RP | NEG | 14.33 | 856.6092 | PC 38:3\|PC 18:0_20:3 [M+HCOO]- | 305.2499(100), 283.2655(40), 796.5891(20) | 2 |
| RP_POS_130_06532a6_095 | RP | POS | 6.10 | 130.0653 | Fragment of 3-indolepropionic acid | 130.0655(100) | 2 |
| RP_NEG_774_53088a12_535 | RP | NEG | 12.54 | 774.5309 | PC 32:2\|PC 16:1_16:1 [M+HCOO]- | 253.2185(100), 714.511(10) | 2 |
| RP_POS_190_08635a6_096 | RP | POS | 6.10 | 190.0864 | 3-Indolepropionic acid | 130.0656(100), 172.0753(20), 190.0865(15), 55.0188(10) | 1 |
| RP_POS_780_55267a13_158 | RP | POS | 13.16 | 780.5527 | PC 34:2 [M+Na]+ | 146.9818(100), 86.0968(50), 597.4879(35), 780.5561(33), 721.4839(25), 575.5069(10) | 2 |
| RP_POS_286_14368a8_418 | RP | POS | 8.42 | 286.1437 | Piperine | 201.0543(100), 286.1456(100), 135.0441(10) | 2 |
| RP_POS_147_11279a0_465 | RP | POS | 0.47 | 147.1128 | Lysine | 84.0811(100), 130.0865(50), 147.1123(8), 129.1016(3), 101.0598(2) | 1 |
| RP_POS_366_26373a7_689 | RP | POS | 7.69 | 366.2637 | CAR 14:3 | 85.0287(100), 366.2627(70) | 2 |
| RP_POS_373_27377a9_323 | RP | POS | 9.32 | 373.2738 | Cholic acid [M+H-H2O]+ | 355.2615(100), 373.2716(13), 245.1533(10), 337.2521(8), 319.2402(6), | 1 |
| RP_POS_358_29498a8_357 | RP | POS | 8.36 | 358.2950 | CAR 13:0 | 85.0289(100), 358.2919(55) | 2 |
| RP_NEG_498_29016a8_562 | RP | NEG | 8.56 | 498.2902 | BA 24:1;O3;T | 498.2869(100) | 2 |
| RP_NEG_500_27905a10_216 | RP | NEG | 10.22 | 500.2791 | LPE 20:4 | 303.2313(100), 500.2761(6), 214.0469(6), 78.9574(3), 196.0375(2) | 2 |
| RP_POS_689_51117a14_537 | RP | POS | 14.54 | 689.5112 | DG 40:7 [M+Na]+ | 689.5001(100) | 2 |
| RP_NEG_305_2496a11_128 | RP | NEG | 11.13 | 305.2496 | FA 20:3 (Eicosatrienoic acid) | 305.2499(100) | 2 |
| RP_POS_540_30707a10_04 | RP | POS | 10.04 | 540.3071 | LPC 18:3 (18:3/0:0) | 104.1076(100), 481.2313(55), 540.306(20), 84.0964(17), 335.2595(3) | 2 |
| RP_POS_504_30762a10_523 | RP | POS | 10.52 | 504.3076 | LPE 20:3 | 363.2895(100), 504.3085(10), 62.0608(50) | 2 |
| HILIC_POS_204_123a2_968 | HILIC | POS | 2.97 | 204.1230 | Acetylcarnitine | 85.0289(100), 204.1229(50), 145.0495(20), 60.0802(10) | 1 |
| RP_POS_752_51874a12_533 | RP | POS | 12.53 | 752.5187 | PC 32:2 [M+Na]+ | 146.9818(100), 86.0969(40), 752.5212(36), 569.4515(35), 693.454(24), 547.484(13) | 2 |
| RP_POS_264_19583a5_311 | RP | POS | 5.31 | 264.1958 | Fragment of N-Desmethylvenlafaxine | 246.1861(100), 264.1958(75), 215.1436(60), 121.0643(40), 147.0801(30) | 2 |
| RP_POS_253_09724a5_873 | RP | POS | 5.87 | 253.0972 | Carbamazepine-10,11-epoxide | 180.0813(100), 210.092(95), 182.0969(18), 208.0767(9) 236.0683(4) | 2 |
| RP_POS_271_1076a5_576 | RP | POS | 5.58 | 271.1076 | 10,11-Dihydro-10,11-dihydroxycarbamazepine | 180.0809(100), 210.0915(95), 254.0795(80), 253.0953(25), 182.0963(18), 208.0767(10) | 2 |
| RP_POS_320_25583a9_477 | RP | POS | 9.48 | 320.2558 | NAE 18:4 | 320.2545(100) | 2 |
| RP_POS_394_29514a8_446 | RP | POS | 8.45 | 394.2951 | CAR 16:3 | 394.2965(100), 85.0285(80) | 2 |
| RP_POS_770_60498a13_833 | RP | POS | 13.83 | 770.6050 | PC O-36:3 | 184.0732(100), 770.611(2) | 2 |
| RP_POS_237_10228a6_888 | RP | POS | 6.89 | 237.1023 | Carbamazepine | 194.0963(100), 237.1004(90), 192.081(15), 220.0761(3) | 2 |
| HILIC_NEG_117_01951a1_1 | HILIC | NEG | 1.10 | 117.0195 | Succinic acid | 73.0287(100), 116.9285(60), 99.925(15), 99.0092(10) | 2 |
| RP_POS_782_56836a13_069 | RP | POS | 13.07 | 782.5684 | PC 36:4\|PC 16:0_20:4 | 184.0731(100), 782.5666(3) | 2 |
| RP_POS_550_38635a11_005 | RP | POS | 11.01 | 550.3864 | LPC 20:1 | 184.0733(100), 550.3918(30), 532.3806(6), 104.1077(85) | 2 |
| RP_NEG_281_24884a11_254 | RP | NEG | 11.25 | 281.2488 | FA 18:1 (Oleic/Elaidic acid) | 281.2505(100) | 2 |
| RP_POS_742_57379a13_498 | RP | POS | 13.50 | 742.5738 | PC O-34:3\|PC O-16:1_18:2 | 184.0731(100), 742.5775(3) | 2 |
| RP_POS_211_1442a4_68 | RP | POS | 4.68 | 211.1442 | Cyclo(Leu-Pro) | 211.1448(100), 86.097(20), 70.0659(15), 183.1483(10) | 2 |
| RP_POS_774_59814a14_235 | RP | POS | 14.24 | 774.5981 | PC 35:1 | 184.0732(100), 774.5886(3) | 2 |
| RP_POS_146_06006a2_489 | RP | POS | 2.49 | 146.0601 | 3-Formylindole | 118.065(100), 146.06(70), 100.1126(25) | 2 |
| RP_POS_552_40198a11_315 | RP | POS | 11.32 | 552.4020 | LPC 20:0 | 184.0728(100), 552.4073(32), 534.3874(6), 104.1073(90), 86.0969(15) | 2 |
| HILIC_NEG_154_06221a6_747 | HILIC | NEG | 6.75 | 154.0622 | Histidine | 154.062(100), 93.0454(60), 137.0364(40), 110.0723(15) | 2 |
| RP_POS_330_26373a7_561 | RP | POS | 7.56 | 330.2637 | CAR 11:0 | 85.0287(100), 330.2617(45) | 2 |
| RP_POS_290_1387a4_214 | RP | POS | 4.21 | 290.1387 | Benzoylecgonine | 168.1011(100), 290.1392(90), 105.0335(20) | 2 |
| RP_NEG_762_50958a12_956 | RP | NEG | 12.96 | 762.5096 | PE 38:6\|PE 16:0_22:6 | 255.2321(100), 327.2347(65), 762.5117(18) | 2 |

| Feature_ID | all_ALD_vs_control t-test p | all_ALD_vs_control Cohen's d | all_ALD_vs_control PLS-DA VIP | Prog_ALD_vs_non-prog_ALD t-test p | Prog_ALD_vs_non-prog_ALD Cohen's d | Prog_ALD_vs_non-prog_ALD PLS-DA VIP | Prog_ALD_vs_control_ALD t-test p | Prog_ALD_vs_control_ALD Cohen's d | Prog_ALD_vs_control_ALD PLS-DA VIP | Non-prog_ALD_vs_control t-test p | Non-prog_ALD_vs_control Cohen's d | Non-prog_ALD_vs_control PLS-DA VIP | Healthy_control Mean | Healthy_control SD | all_ALD Mean | all_ALD SD | Non-prog_ALD Mean | Non-prog_ALD SD | Progressive_ALD Mean | Progressive_ALD SD |
|---|---|---|---|---|---|---|---|---|---|---|---|---|---|---|---|---|---|---|---|---|
| RP_POS_810_59894a14_255 | 6.54E-01 | 0.08 | 0.49 | 1.80E-01 | 0.28 | 0.97 | 3.07E-01 | 0.22 | 0.43 | 7.60E-01 | -0.06 | 0.73 | 4183248 | 2772279 | 4456079 | 3551367 | 3978017 | 3218610 | 4934141 | 3828309 |
| RP_POS_130_08647a0_708 | 5.56E-01 | -0.15 | 0.43 | 1.85E-01 | 0.23 | 0.88 | 8.85E-01 | -0.04 | 0.17 | 3.08E-01 | -0.27 | 0.58 | 10620882 | 6984902 | 9844225 | 4260787 | 9276387 | 2879758 | 10412062 | 5265744 |
| RP_POS_150_0582a0_709 | 4.36E-01 | -0.10 | 0.47 | 1.85E-01 | 0.30 | 0.91 | 8.27E-01 | 0.05 | 0.27 | 4.80E-03 | -0.25 | 1.35 | 24047617 | 3339034 | 23108607 | 10480655 | 21708594 | 3860482 | 24508621 | 14248739 |
| RP_POS_760_58508a13_717 | 2.71E-10 | 1.12 | 1.77 | 1.89E-01 | 0.25 | 0.95 | 3.28E-09 | 1.24 | 1.88 | 1.20E-07 | 0.99 | 1.76 | 373955781 | 79835411 | 505158758 | 109955123 | 490665858 | 99949055 | 519651658 | 118357596 |
| RP_POS_346_1221a5_854 | 8.40E-04 | 0.42 | 0.80 | 1.90E-01 | 0.30 | 1.00 | 1.27E-02 | 0.56 | 0.84 | 1.36E-03 | 0.27 | 1.12 | 8531556 | 4524457 | 15718151 | 19413277 | 13156028 | 8059284 | 18280274 | 26136049 |
| RP_POS_633_45544a10_872 | 3.08E-01 | -0.17 | 0.61 | 1.93E-01 | 0.28 | 0.89 | 8.78E-01 | -0.03 | 0.37 | 1.09E-01 | -0.31 | 0.61 | 11935654 | 587890 | 11796531 | 870424 | 11682903 | 822046 | 11910160 | 910292 |
| RP_POS_858_59827a14_026 | 5.73E-07 | 0.94 | 1.61 | 1.99E-01 | 0.25 | 1.00 | 2.12E-06 | 1.07 | 1.67 | 1.98E-05 | 0.82 | 1.58 | 4212446 | 1594384 | 6112688 | 1918470 | 5865129 | 1575601 | 6360247 | 2197402 |
| RP_POS_675_54248a12_21 | 2.72E-02 | -0.45 | 0.89 | 2.01E-01 | 0.25 | 0.89 | 1.49E-01 | -0.32 | 0.70 | 1.11E-02 | -0.58 | 1.02 | 25226498 | 7692844 | 21663631 | 7815220 | 20660596 | 7761927 | 22666665 | 7816718 |
| RP_POS_678_50568a12_274 | 1.32E-01 | 0.28 | 0.69 | 2.03E-01 | 0.26 | 0.88 | 8.14E-02 | 0.41 | 0.75 | 4.23E-01 | 0.15 | 0.36 | 1591769 | 1892679 | 2208760 | 2268407 | 1918358 | 1614868 | 2499163 | 2759791 |
| RP_POS_231_08333a0_709 | 6.43E-16 | 1.00 | 1.69 | 2.03E-01 | 0.27 | 0.92 | 1.04E-08 | 1.14 | 1.82 | 6.89E-09 | 0.87 | 1.69 | 224878 | 245550 | 3142391 | 3015038 | 2756909 | 2557434 | 3527872 | 3394510 |
| RP_NEG_297_24417a9_953 | 5.36E-13 | 0.96 | 1.66 | 2.07E-01 | 0.26 | 1.37 | 1.22E-08 | 1.09 | 1.78 | 5.14E-07 | 0.82 | 1.71 | 979616 | 535994 | 2499039 | 1640250 | 2291411 | 1525638 | 2706667 | 1737872 |
| RP_POS_246_16994a3_08 | 3.14E-01 | 0.17 | 0.34 | 2.09E-01 | -0.27 | 0.92 | 8.55E-01 | 0.03 | 0.23 | 1.49E-01 | 0.30 | 0.50 | 2634111 | 1169158 | 2907202 | 1727959 | 3125028 | 1877701 | 2689375 | 1552425 |
| RP_POS_570_35535a10_446 | 1.93E-03 | 0.59 | 1.24 | 2.15E-01 | -0.25 | 0.96 | 3.47E-02 | 0.47 | 0.96 | 5.86E-04 | 0.72 | 1.38 | 11812767 | 6682991 | 16404156 | 7803260 | 17374876 | 7013006 | 15433437 | 8480512 |
| RP_NEG_566_34454a10_502 | 1.47E-03 | 0.63 | 1.34 | 2.16E-01 | 0.24 | 0.99 | 1.49E-03 | 0.76 | 1.34 | 1.12E-02 | 0.51 | 1.20 | 15587672 | 4647147 | 18803051 | 4978707 | 18184530 | 3925515 | 19421573 | 5821555 |
| RP_POS_518_3241a9_905 | 4.57E-01 | 0.16 | 0.65 | 2.17E-01 | -0.24 | 0.82 | 8.76E-01 | 0.04 | 0.53 | 2.26E-01 | 0.28 | 0.79 | 1924825 | 1645354 | 2170945 | 1523536 | 2359599 | 1448777 | 1982291 | 1586854 |
| RP_NEG_786_56799a13_501 | 3.73E-03 | -0.67 | 1.07 | 2.22E-01 | -0.22 | 0.78 | 1.89E-03 | -0.78 | 1.21 | 1.97E-02 | -0.56 | 0.87 | 6044899 | 2175038 | 4754311 | 1723762 | 4965562 | 1639086 | 4543060 | 1796015 |
| RP_POS_134_06029a3_283 | 7.64E-02 | -0.43 | 0.82 | 2.22E-01 | -0.22 | 0.72 | 4.06E-02 | -0.54 | 0.94 | 2.05E-01 | -0.32 | 0.58 | 1275863 | 915445 | 957888 | 667633 | 1039688 | 616401 | 876088 | 712010 |
| HILIC_NEG_151_02606a1_2 | 3.95E-04 | 0.46 | 0.72 | 2.23E-01 | 0.27 | 0.79 | 1.13E-03 | 0.59 | 1.01 | 5.20E-02 | 0.32 | 0.80 | 3445908 | 1931733 | 6169168 | 6660539 | 5355075 | 6370805 | 6983260 | 6905883 |
| RP_POS_729_59027a12_915 | 2.67E-05 | -0.94 | 1.55 | 2.24E-01 | -0.22 | 0.85 | 2.47E-05 | -1.04 | 1.57 | 2.22E-04 | -0.83 | 1.51 | 13766857 | 4428619 | 9705370 | 3845272 | 10174993 | 3198862 | 9235746 | 4380774 |
| RP_POS_357_27863a9_858 | 4.78E-01 | 0.13 | 0.33 | 2.29E-01 | 0.25 | 0.73 | 2.47E-01 | 0.26 | 0.46 | 9.85E-01 | 0.00 | 0.64 | 3507680 | 5500233 | 4354029 | 6792344 | 3532830 | 6084803 | 5175229 | 7403536 |
| RP_POS_344_2793a8_114 | 5.04E-01 | 0.11 | 0.94 | 2.30E-01 | -0.26 | 1.59 | 9.31E-01 | -0.01 | 0.69 | 2.88E-01 | 0.24 | 0.87 | 8978813 | 4734950 | 9702562 | 6774667 | 10519459 | 8280170 | 8885665 | 4775540 |
| RP_POS_391_28372a11_391 | 1.49E-01 | -0.30 | 0.58 | 2.34E-01 | 0.24 | 1.01 | 4.62E-01 | -0.18 | 0.38 | 5.32E-02 | -0.41 | 0.82 | 7606524 | 745971 | 7384608 | 746943 | 7295379 | 610186 | 7473837 | 859452 |
| RP_POS_834_59705a14_323 | 5.45E-01 | 0.14 | 0.61 | 2.35E-01 | 0.22 | 0.77 | 3.38E-01 | 0.25 | 0.60 | 9.01E-01 | 0.03 | 0.39 | 16605442 | 7519662 | 17492702 | 5880126 | 16790860 | 4668751 | 18194543 | 6859656 |
| RP_POS_302_23242a6_351 | 2.06E-04 | -0.69 | 1.20 | 2.40E-01 | -0.24 | 0.72 | 5.47E-05 | -0.81 | 1.53 | 9.23E-03 | -0.57 | 0.96 | 1506985 | 770353 | 853707 | 946260 | 965426 | 1063567 | 741988 | 807740 |
| RP_POS_782_56873a12_82 | 5.79E-06 | -1.12 | 1.81 | 2.40E-01 | -0.19 | 0.86 | 2.51E-06 | -1.21 | 1.88 | 4.50E-05 | -1.03 | 1.56 | 154503389 | 57779276 | 97390529 | 40074976 | 102116720 | 40851958 | 92664338 | 39119242 |
| RP_NEG_329_24921a11_049 | 3.30E-09 | 0.81 | 1.76 | 2.48E-01 | 0.25 | 0.90 | 2.01E-06 | 0.93 | 1.68 | 2.12E-05 | 0.68 | 1.70 | 4401558 | 2414017 | 9306262 | 6419022 | 8561027 | 5710115 | 10051497 | 7036316 |
| RP_POS_308_12555a8_419 | 6.71E-01 | 0.09 | 0.28 | 2.52E-01 | 0.23 | 0.83 | 4.05E-01 | 0.20 | 0.45 | 8.97E-01 | -0.03 | 0.43 | 5936843 | 7951066 | 6625229 | 7870886 | 5719508 | 6421431 | 7530950 | 9069568 |
| RP_POS_502_29254a10_216 | 4.31E-06 | 0.80 | 1.52 | 2.54E-01 | -0.23 | 0.87 | 1.31E-03 | 0.69 | 1.22 | 9.97E-07 | 0.92 | 1.76 | 7062091 | 2213564 | 9539887 | 3090810 | 9893882 | 2523294 | 9185891 | 3560677 |
| RP_POS_766_57379a13_372 | 7.50E-01 | -0.06 | 0.58 | 2.57E-01 | -0.24 | 0.74 | 3.91E-01 | -0.18 | 0.34 | 7.97E-01 | 0.06 | 0.52 | 34965066 | 9385991 | 34330258 | 10910026 | 35572430 | 11789755 | 33088086 | 9916613 |
| RP_POS_583_25464a11_804 | 2.56E-02 | -0.44 | 1.10 | 2.61E-01 | 0.23 | 1.31 | 1.88E-01 | -0.32 | 0.68 | 4.15E-03 | -0.55 | 1.32 | 25029179 | 13655694 | 18520394 | 14957052 | 16829226 | 8919142 | 20211562 | 19146917 |
| RP_POS_342_26376a7_71 | 1.44E-06 | 0.67 | 1.26 | 2.67E-01 | 0.24 | 1.19 | 7.80E-06 | 0.79 | 1.52 | 2.83E-03 | 0.55 | 1.15 | 4801668 | 3230454 | 9831589 | 8124748 | 8925156 | 8500035 | 10738022 | 7709919 |
| RP_POS_287_05817a7_376 | 6.57E-01 | -0.06 | 0.61 | 2.67E-01 | 0.25 | 0.84 | 7.60E-01 | 0.07 | 0.60 | 5.89E-03 | -0.18 | 1.41 | 3346044 | 2483439 | 2898735 | 9034551 | 1885755 | 1814095 | 3911715 | 12630397 |
| RP_NEG_498_29214a8_173 | 9.99E-01 | 0.00 | 0.46 | 2.74E-01 | 0.25 | 1.25 | 5.93E-01 | 0.12 | 0.75 | 8.23E-02 | -0.13 | 0.74 | 14893306 | 15324236 | 14885460 | 49768241 | 9392660 | 10696649 | 20378260 | 69486061 |
| RP_NEG_856_60919a14_325 | 4.30E-01 | 0.19 | 0.66 | 2.77E-01 | 0.20 | 0.70 | 2.76E-01 | 0.29 | 0.66 | 7.22E-01 | 0.09 | 0.43 | 14780571 | 6258658 | 15739205 | 4701016 | 15225118 | 3968675 | 16253292 | 5325445 |
| RP_POS_130_06532a6_095 | 1.97E-03 | -0.83 | 1.45 | 2.78E-01 | -0.17 | 0.64 | 8.06E-04 | -0.92 | 1.48 | 8.01E-03 | -0.75 | 1.12 | 2248453 | 1963140 | 1036074 | 1110250 | 1157263 | 1330082 | 914886 | 831489 |
| RP_NEG_774_53088a12_535 | 1.86E-01 | 0.28 | 0.92 | 2.82E-01 | 0.21 | 0.66 | 1.10E-01 | 0.39 | 0.92 | 4.35E-01 | 0.18 | 0.36 | 8769586 | 3809302 | 9790903 | 3557157 | 9406090 | 3184891 | 10175716 | 3888346 |
| RP_POS_190_08635a6_096 | 2.95E-03 | -0.81 | 1.41 | 2.86E-01 | -0.16 | 0.63 | 1.24E-03 | -0.89 | 1.43 | 1.10E-02 | -0.73 | 1.08 | 10103276 | 9163283 | 4702484 | 5069929 | 5246524 | 6148672 | 4158444 | 3677327 |
| RP_POS_780_55267a13_158 | 5.42E-01 | 0.15 | 0.42 | 2.88E-01 | 0.19 | 0.88 | 3.34E-01 | 0.24 | 0.60 | 8.48E-01 | 0.05 | 0.24 | 212402209 | 43212062 | 217478013 | 31237381 | 214140699 | 33936293 | 220815327 | 28232246 |
| RP_POS_286_14368a8_418 | 6.28E-01 | 0.10 | 0.31 | 2.90E-01 | 0.21 | 0.77 | 4.03E-01 | 0.21 | 0.45 | 9.81E-01 | -0.01 | 0.42 | 38318645 | 56925768 | 43907018 | 55221302 | 38036736 | 43533241 | 49777300 | 64773041 |
| RP_POS_147_11279a0_465 | 8.43E-03 | 0.35 | 1.03 | 2.92E-01 | -0.24 | 1.12 | 1.97E-01 | 0.23 | 0.95 | 6.83E-03 | 0.47 | 1.13 | 1149023 | 254914 | 1385970 | 762513 | 1466742 | 738331 | 1305198 | 785029 |
| RP_POS_366_26373a7_689 | 3.79E-01 | -0.19 | 0.93 | 2.95E-01 | -0.21 | 1.12 | 1.96E-01 | -0.29 | 0.67 | 7.26E-01 | -0.09 | 0.96 | 571309 | 431563 | 495026 | 393812 | 536501 | 443611 | 453550 | 336215 |
| RP_POS_373_27377a9_323 | 2.71E-01 | -0.32 | 0.63 | 2.98E-01 | 0.16 | 0.64 | 4.24E-01 | -0.24 | 0.40 | 1.81E-01 | -0.39 | 0.60 | 2689676 | 5464725 | 1570772 | 2620436 | 1296899 | 2358803 | 1844645 | 2855954 |
| RP_POS_358_29498a8_357 | 1.15E-01 | -0.34 | 0.88 | 3.01E-01 | -0.20 | 0.66 | 7.43E-02 | -0.44 | 0.71 | 2.91E-01 | -0.23 | 0.75 | 2300896 | 1413134 | 1847213 | 1319802 | 1984423 | 1133961 | 1710002 | 1481557 |
| RP_NEG_498_29016a8_562 | 5.21E-01 | 0.09 | 0.41 | 3.01E-01 | 0.23 | 1.03 | 3.83E-01 | 0.21 | 0.66 | 8.06E-01 | -0.02 | 0.40 | 9414707 | 24186815 | 13799276 | 52907046 | 8276804 | 12014207 | 19321748 | 73816363 |
| RP_NEG_500_27905a10_216 | 1.51E-06 | 0.84 | 1.57 | 3.02E-01 | -0.21 | 0.79 | 5.62E-04 | 0.74 | 1.28 | 4.43E-07 | 0.95 | 1.79 | 4205188 | 1288791 | 5723519 | 1788406 | 5909055 | 1452290 | 5537983 | 2069461 |
| RP_POS_689_51117a14_537 | 5.33E-01 | 0.07 | 0.76 | 3.09E-01 | 0.24 | 0.62 | 4.08E-01 | 0.19 | 0.52 | 2.58E-01 | -0.04 | 0.73 | 944636 | 848892 | 1293001 | 5358099 | 742363 | 663947 | 1843640 | 7546174 |
| RP_NEG_305_2496a11_128 | 6.54E-08 | 0.84 | 1.76 | 3.09E-01 | 0.21 | 1.01 | 3.99E-06 | 0.94 | 1.69 | 2.02E-05 | 0.73 | 1.69 | 1631321 | 792325 | 2808177 | 1443286 | 2660584 | 1264053 | 2955770 | 1601991 |
| RP_POS_540_30707a10_04 | 2.70E-02 | 0.42 | 0.97 | 3.10E-01 | -0.21 | 0.89 | 1.44E-01 | 0.31 | 0.90 | 1.53E-02 | 0.52 | 1.17 | 2692383 | 2242350 | 3774612 | 2664821 | 4046420 | 2647464 | 3502805 | 2680976 |
| RP_POS_504_30762a10_523 | 3.14E-05 | 0.76 | 1.30 | 3.12E-01 | -0.20 | 0.66 | 1.38E-03 | 0.66 | 1.21 | 3.34E-05 | 0.86 | 1.44 | 2862796 | 1063758 | 3898839 | 1353557 | 4036340 | 1330341 | 3761338 | 1375949 |
| HILIC_POS_204_123a2_968 | 6.62E-12 | 0.90 | 1.47 | 3.14E-01 | 0.21 | 1.03 | 7.57E-09 | 1.00 | 1.77 | 1.11E-05 | 0.79 | 1.46 | 279367494 | 133412491 | 653939970 | 436106531 | 609788076 | 458550962 | 698091863 | 412324978 |
| RP_POS_752_51874a12_533 | 5.34E-01 | 0.13 | 0.82 | 3.16E-01 | 0.20 | 0.60 | 3.41E-01 | 0.23 | 0.82 | 8.76E-01 | 0.04 | 0.14 | 10363194 | 4957354 | 10980674 | 4502024 | 10527000 | 4078847 | 11434348 | 4888051 |
| RP_POS_264_19583a5_311 | 5.35E-01 | 0.09 | 0.71 | 3.18E-01 | 0.22 | 0.62 | 2.13E-01 | 0.20 | 0.80 | 9.26E-01 | -0.02 | 0.59 | 8225379 | 5839685 | 9195840 | 11679837 | 8022627 | 13433240 | 10369053 | 9610425 |
| RP_POS_253_09724a5_873 | 3.21E-01 | 0.11 | 0.75 | 3.22E-01 | -0.23 | 0.78 | 1.93E-01 | 0.00 | 0.66 | 3.23E-01 | 0.23 | 0.83 | 33211 | 13675 | 2261995 | 22338745 | 4494705 | 31591922 | 29284 | 12365 |
| RP_POS_271_1076a5_576 | 3.20E-01 | 0.11 | 0.75 | 3.23E-01 | -0.23 | 0.78 | 8.98E-01 | 0.00 | 0.53 | 3.22E-01 | 0.23 | 0.83 | 19866 | 9507 | 1415342 | 13960788 | 2810528 | 19743661 | 20155 | 10576 |
| RP_POS_320_25583a9_477 | 3.74E-01 | -0.24 | 0.83 | 3.24E-01 | -0.16 | 0.67 | 2.47E-01 | -0.32 | 0.72 | 5.82E-01 | -0.16 | 0.49 | 26608500 | 36810984 | 20439017 | 21512887 | 22570750 | 23289768 | 18307285 | 19579742 |
| RP_POS_394_29514a8_446 | 2.92E-03 | 0.46 | 0.88 | 3.26E-01 | -0.21 | 1.30 | 4.82E-02 | 0.36 | 0.81 | 4.25E-03 | 0.57 | 1.04 | 455024 | 211038 | 616365 | 373821 | 653287 | 396118 | 579443 | 350207 |
| RP_POS_770_60498a13_833 | 3.28E-06 | -1.06 | 1.64 | 3.26E-01 | -0.17 | 0.67 | 3.92E-06 | -1.14 | 1.67 | 2.10E-05 | -0.97 | 1.58 | 6952886 | 2331850 | 4538736 | 1939134 | 4730172 | 1657171 | 4347299 | 2185456 |
| RP_POS_237_10228a6_888 | 3.13E-01 | 0.12 | 0.75 | 3.27E-01 | -0.23 | 0.78 | 7.01E-02 | 0.00 | 0.77 | 3.22E-01 | 0.23 | 0.83 | 476208 | 102899 | 9625906 | 90314430 | 18566062 | 127734550 | 685749 | 790714 |
| HILIC_NEG_117_01951a1_1 | 8.82E-01 | -0.03 | 0.74 | 3.29E-01 | 0.20 | 0.95 | 7.33E-01 | 0.07 | 0.17 | 5.52E-01 | -0.13 | 0.69 | 1475727 | 366126 | 1464168 | 422123 | 1422728 | 429229 | 1505609 | 415054 |
| RP_POS_782_56836a13_069 | 3.60E-01 | 0.19 | 0.54 | 3.35E-01 | -0.19 | 0.63 | 6.92E-01 | 0.09 | 0.75 | 1.96E-01 | 0.29 | 0.68 | 511754194 | 119073348 | 533931989 | 115178224 | 545091779 | 101352611 | 522772199 | 127578465 |
| RP_POS_550_38635a11_005 | 5.43E-01 | -0.14 | 0.68 | 3.36E-01 | 0.18 | 1.22 | 8.57E-01 | -0.05 | 0.43 | 3.32E-01 | -0.23 | 0.79 | 6653730 | 2890816 | 6308041 | 2380998 | 6077450 | 2056184 | 6538633 | 2667882 |
| RP_NEG_281_24884a11_254 | 6.60E-07 | 0.75 | 1.64 | 3.38E-01 | 0.20 | 1.00 | 1.33E-05 | 0.85 | 1.62 | 2.57E-04 | 0.65 | 1.56 | 22010014 | 11741821 | 38363493 | 22846617 | 36160817 | 21614214 | 40566170 | 24031488 |
| RP_POS_742_57379a13_498 | 4.56E-03 | -0.67 | 1.08 | 3.46E-01 | -0.17 | 0.64 | 2.70E-03 | -0.76 | 1.21 | 1.85E-02 | -0.59 | 0.87 | 27509150 | 10782359 | 21305605 | 8127407 | 22076231 | 8286829 | 20534980 | 7973334 |
| RP_POS_211_1442a4_68 | 1.14E-06 | 0.74 | 1.23 | 3.47E-01 | 0.20 | 0.60 | 5.74E-05 | 0.84 | 1.41 | 9.83E-05 | 0.64 | 1.28 | 4120346 | 2757407 | 7838989 | 5280349 | 7339098 | 4350402 | 8338879 | 6074308 |
| RP_POS_774_59814a14_235 | 3.13E-02 | -0.47 | 0.89 | 3.48E-01 | 0.18 | 0.63 | 1.13E-01 | -0.38 | 0.68 | 1.69E-02 | -0.56 | 0.97 | 5594907 | 2723488 | 4399800 | 2417749 | 4171632 | 2269888 | 4627969 | 2559625 |
| RP_POS_146_06006a2_489 | 1.71E-03 | -0.55 | 1.15 | 3.48E-01 | -0.20 | 1.02 | 3.52E-03 | -0.65 | 1.20 | 1.34E-02 | -0.45 | 1.06 | 4099296 | 731387 | 3567188 | 996310 | 3661257 | 810023 | 3473119 | 1153832 |
| RP_POS_552_40198a11_315 | 6.53E-04 | -0.90 | 1.58 | 3.48E-01 | -0.14 | 0.96 | 3.90E-04 | -0.98 | 1.50 | 2.19E-03 | -0.83 | 1.34 | 1978283 | 868918 | 1377404 | 508550 | 1425353 | 519035 | 1329455 | 498433 |
| HILIC_NEG_154_06221a6_747 | 6.65E-01 | -0.07 | 0.95 | 3.54E-01 | -0.20 | 0.84 | 3.66E-01 | -0.17 | 0.99 | 9.08E-01 | 0.02 | 0.96 | 2658371 | 423550 | 2616877 | 592206 | 2672017 | 646174 | 2561737 | 533692 |
| RP_POS_330_26373a7_561 | 4.65E-03 | -0.66 | 1.24 | 3.66E-01 | -0.16 | 0.55 | 3.15E-03 | -0.74 | 1.23 | 1.83E-02 | -0.58 | 1.10 | 1410131 | 667980 | 1024556 | 526669 | 1072474 | 516049 | 976638 | 537985 |
| RP_POS_290_1387a4_214 | 2.60E-01 | -0.16 | 0.48 | 3.66E-01 | -0.20 | 0.58 | 1.57E-02 | -0.26 | 0.96 | 8.05E-01 | -0.06 | 0.32 | 33304786 | 19176695 | 27011705 | 44093191 | 31030982 | 60079390 | 22992428 | 16899557 |
| RP_NEG_762_50958a12_956 | 8.83E-01 | 0.03 | 0.70 | 3.68E-01 | 0.19 | 0.57 | 6.15E-01 | 0.12 | 0.57 | 7.23E-01 | -0.07 | 0.34 | 2221924 | 1394956 | 2265595 | 1621868 | 2118604 | 1078662 | 2412586 | 2026571 |

**Supplementary table 1: Metabolomics results** (in asceding order according to p-value from progressive ALD vs  non-progressive ALD comparison, with molecular features with ID_level of 1-2 first)

| | | | | | | | | all_ALD_vs_control | | | Prog_ALD_vs_non-prog_ALD | | | Prog_ALD_vs_control_ALD | | | Non-prog_ALD_vs_control | | | Healthy_control | | all_ALD | | Non-prog_ALD | | Progressive_ALD | |
|---|---|---|---|---|---|---|---|---|---|---|---|---|---|---|---|---|---|---|---|---|---|---|---|---|---|---|---|
| | | | | | | | | t-test | Cohen's | PLS-DA | t-test | Cohen's | PLS-DA | t-test | Cohen's | PLS-DA | t-test | Cohen's | PLS-DA | | | | | | | | |
| Feature_ID | RP_HILIC | POS_NEG | Average.Rt | Average.Mz | Metabolite.name | curated_MSMS | ID_level | p | d | VIP | p | d | VIP | p | d | VIP | p | d | VIP | Mean | SD | Mean | SD | Mean | SD | Mean | SD |
| RP_NEG_502_29468a10_519 | RP | NEG | 10.52 | 502.2947 | LPE 20:3 | 305.2496(100), 502.299(15), 196.0373(10), 78.9581(5), 314.0472(3) | 2 | 1.57E-05 | 0.76 | 1.33 | 3.71E-01 | -0.18 | 0.54 | 1.05E-03 | 0.67 | 1.22 | 2.26E-05 | 0.85 | 1.44 | 2189551 | 785199 | 3004883 | 1081984 | 3102270 | 1041137 | 2907496 | 1123365 |
| RP_NEG_315_25494a9_606 | RP | NEG | 9.61 | 315.2549 | FA 18:0;2O | 315.2517(100) | 2 | 1.07E-05 | 0.63 | 1.31 | 3.71E-01 | 0.19 | 1.15 | 4.22E-04 | 0.72 | 1.30 | 6.28E-04 | 0.53 | 1.34 | 1101285 | 174914 | 1332073 | 394960 | 1296549 | 319982 | 1367598 | 458483 |
| RP_NEG_452_27863a10_388 | RP | NEG | 10.39 | 452.2786 | LPE 16:0 | 255.2332(100), 452.2818(5) | 2 | 4.27E-01 | 0.14 | 0.98 | 3.71E-01 | -0.19 | 0.85 | 8.35E-01 | 0.05 | 0.70 | 2.23E-01 | 0.24 | 0.83 | 2050234 | 648451 | 2164708 | 860126 | 2242051 | 748494 | 2087365 | 960355 |
| RP_POS_261_13089a2_881 | RP | POS | 2.88 | 261.1309 | 2-(4-oxoquinazolin-3(4H)-yl)ethyl isobutyrate [M+Na]+ Fragment | 261.1308(100) | 2 | 3.25E-02 | 0.32 | 1.11 | 3.74E-01 | -0.20 | 1.10 | 1.83E-01 | 0.23 | 1.13 | 3.85E-02 | 0.42 | 1.12 | 752016 | 837212 | 1220018 | 1571878 | 1360692 | 1754650 | 1079344 | 1368518 |
| RP_POS_228_2321a10_19 | RP | POS | 10.19 | 228.2321 | Myristamide | 228.2326(100) | 2 | 7.08E-03 | 0.33 | 0.70 | 3.75E-01 | 0.20 | 0.92 | 6.19E-02 | 0.43 | 0.79 | 2.52E-04 | 0.23 | 1.26 | 5671005 | 1585307 | 7899559 | 7635445 | 7215211 | 2040035 | 8583907 | 10614721 |
| RP_POS_522_3551a10_503 | RP | POS | 10.50 | 522.3551 | LPC 18:1 (0:0/18:1) | 184.0735(100), 522.3544(3.5), 504.3395(0.8) | 2 | 4.28E-04 | 0.70 | 1.45 | 3.77E-01 | 0.17 | 0.94 | 1.00E-03 | 0.78 | 1.36 | 2.13E-03 | 0.61 | 1.39 | 56638742 | 19053947 | 71440087 | 20707991 | 69597550 | 15586719 | 73282625 | 24829799 |
| RP_POS_264_19577a4_03 | RP | POS | 4.03 | 264.1958 | O-desmethylvenlafaxine | 58.066(100), 264.1947(35), 246.183(30), 107.0489(3), 201.1271(5) | 2 | 9.26E-01 | -0.01 | 0.49 | 3.77E-01 | -0.20 | 0.93 | 2.77E-01 | -0.11 | 0.80 | 6.93E-01 | 0.09 | 0.67 | 36252955 | 10352400 | 35915512 | 31033306 | 38678759 | 41189861 | 33152266 | 15283953 |
| RP_POS_130_04987a0_526 | RP | POS | 0.53 | 130.0499 | L-5-Oxoproline | 84.0445(100), 130.0499(35) | 2 | 1.46E-01 | 0.22 | 0.77 | 3.78E-01 | -0.20 | 0.83 | 5.38E-01 | 0.12 | 0.53 | 8.08E-02 | 0.31 | 0.86 | 3962044 | 638049 | 4210061 | 1265301 | 4322335 | 1199232 | 4097787 | 1330700 |
| RP_NEG_283_26535a11_611 | RP | NEG | 11.61 | 283.2654 | FA 18:0 (Stearic acid) | 283.2652(100) | 2 | 6.84E-02 | -0.34 | 0.87 | 3.79E-01 | -0.18 | 1.00 | 3.04E-02 | -0.43 | 0.92 | 2.71E-01 | -0.25 | 0.80 | 35684798 | 9731904 | 31832770 | 11639142 | 32862751 | 13268862 | 30802788 | 9771194 |
| RP_POS_205_09669a2_489 | RP | POS | 2.49 | 205.0967 | Tryptophan | 188.0713(100), 146.06(50), 144.0813(8), 159.0923(6) | 2 | 1.95E-03 | -0.53 | 1.14 | 3.79E-01 | -0.18 | 1.04 | 4.61E-03 | -0.62 | 1.18 | 1.47E-02 | -0.44 | 1.05 | 214906042 | 36924841 | 187939500 | 52878667 | 192619124 | 42932474 | 183259877 | 61330924 |
| RP_POS_188_07024a2_489 | RP | POS | 2.49 | 188.0702 | Desethyl atrazine | 146.06(100), 188.0713(45) | 2 | 2.03E-03 | -0.54 | 1.14 | 3.80E-01 | -0.18 | 1.03 | 4.68E-03 | -0.63 | 1.18 | 1.44E-02 | -0.44 | 1.05 | 140834135 | 24720587 | 122982071 | 34681411 | 126042652 | 27984289 | 119921489 | 40347371 |
| RP_NEG_464_31503a11_092 | RP | NEG | 11.09 | 464.3150 | LPE O-18:1 | 464.3122(100), 78.9574(37), 140.011(28), 196.0376(42), 267.2686(25) | 2 | 2.43E-01 | -0.25 | 0.84 | 3.84E-01 | -0.17 | 1.11 | 1.58E-01 | -0.34 | 0.96 | 4.78E-01 | -0.17 | 0.81 | 1383779 | 509829 | 1263932 | 465748 | 1304678 | 455965 | 1223187 | 476420 |
| RP_POS_748_52667a13_248 | RP | POS | 13.25 | 748.5267 | PE P-38:6\|PE P-16:0_22:6 | 385.2754(100), 364.262(30), 266.283(15), 607.5018(5) | 2 | 5.87E-01 | -0.09 | 0.89 | 3.86E-01 | 0.19 | 0.57 | 9.69E-01 | 0.01 | 0.67 | 2.30E-01 | -0.18 | 0.54 | 4062683 | 1229143 | 3899582 | 2059318 | 3719612 | 1285516 | 4079553 | 2617150 |
| RP_POS_283_26343a10_223 | RP | POS | 10.22 | 283.2634 | FA 18:1 (Petroselinic acid) | 57.0706(100), 69.0707(75), 71.0851(73), 83.0861(70), 97.1011(60), 247.2419(57), 265.253(40), 283.2611(13) | 2 | 4.39E-01 | 0.14 | 0.87 | 3.86E-01 | 0.18 | 0.81 | 2.75E-01 | 0.23 | 0.76 | 8.28E-01 | 0.04 | 0.80 | 13532845 | 1569967 | 13803898 | 2105300 | 13620425 | 2049973 | 13987371 | 2164242 |
| RP_POS_830_56531a13_206 | RP | POS | 13.21 | 830.5653 | PC 38:5\|PC 16:0_22:5 AND PC 18:1_20:4 | 146.9817(100), 86.0968(60), 647.4933(60), 771.4937(38), 830.5549(31), 625.5184(8) | 2 | 2.86E-04 | 0.66 | 1.16 | 3.89E-01 | 0.18 | 0.92 | 1.16E-03 | 0.75 | 1.14 | 1.76E-03 | 0.57 | 1.22 | 28128006 | 5046313 | 32350098 | 6474490 | 31789156 | 4800968 | 32911039 | 7810383 |
| RP_POS_796_62048a14_574 | RP | POS | 14.57 | 796.6205 | PC O-38:4 | 184.0735(100), 796.6392(3) | 2 | 1.21E-03 | -0.76 | 1.24 | 3.90E-01 | -0.15 | 0.51 | 6.60E-04 | -0.83 | 1.36 | 6.71E-03 | -0.68 | 1.12 | 7402895 | 3209518 | 5255040 | 2508968 | 5472167 | 2736897 | 5037913 | 2265279 |
| RP_NEG_745_552a12_329 | RP | NEG | 12.33 | 745.5520 | SM 34:2;2O\|SM 18:2;2O/16:0 [M+HCOO]- | 685.5215(100), 168.0421(90), 78.9574(70) | 2 | 2.23E-05 | -0.95 | 1.66 | 3.94E-01 | -0.15 | 0.55 | 2.55E-05 | -1.02 | 1.61 | 1.46E-04 | -0.87 | 1.63 | 18773203 | 3901374 | 15157995 | 3350073 | 15445213 | 3080113 | 14870777 | 3608263 |
| RP_POS_132_10172a1_457 | RP | POS | 1.46 | 132.1017 | Leucine | 86.097(100), 132.1024(4), 69.0707(2) | 2 | 3.99E-01 | 0.14 | 0.65 | 3.97E-01 | -0.18 | 0.58 | 8.20E-01 | 0.05 | 0.50 | 2.17E-01 | 0.23 | 0.46 | 48056445 | 9970087 | 50038594 | 15372099 | 51347192 | 13942726 | 48729996 | 16719180 |
| RP_POS_130_04988a1_13 | RP | POS | 1.13 | 130.0499 | L-5-Oxoproline | 84.045(100), 130.0495(25) | 2 | 1.49E-12 | 0.96 | 1.58 | 3.98E-01 | 0.17 | 0.98 | 1.25E-07 | 1.05 | 1.66 | 4.09E-08 | 0.87 | 1.76 | 1318412 | 247380 | 1950368 | 676330 | 1892827 | 584263 | 2007910 | 758986 |
| RP_POS_758_5683a14_259 | RP | POS | 14.26 | 758.5683 | PC 34:2 | 184.0732(100), 758.5762(3) | 2 | 9.45E-01 | -0.01 | 0.33 | 4.03E-01 | 0.18 | 0.57 | 7.34E-01 | 0.08 | 0.19 | 6.17E-01 | -0.10 | 0.76 | 3714471 | 1846936 | 3686687 | 2270330 | 3495610 | 2042959 | 3877763 | 2483102 |
| RP_POS_314_23242a6_778 | RP | POS | 6.78 | 314.2324 | CAR 10:1 | 85.0287(100), 314.2295(50) | 2 | 8.13E-02 | -0.28 | 0.76 | 4.07E-01 | -0.18 | 1.10 | 2.50E-02 | -0.37 | 0.89 | 3.89E-01 | -0.19 | 0.61 | 12811109 | 8019099 | 9389338 | 13248868 | 10495408 | 16019797 | 8283267 | 9773264 |
| RP_POS_327_07709a9_047 | RP | POS | 9.05 | 327.0771 | Triphenylphosphate | 327.0796(100), 233.0359(8), 153.0699(3) | 2 | 4.99E-03 | -0.61 | 0.97 | 4.08E-01 | -0.16 | 0.72 | 2.49E-03 | -0.69 | 1.17 | 2.96E-02 | -0.53 | 0.80 | 7013783 | 434494 | 6759470 | 396830 | 6792496 | 447817 | 6726444 | 339700 |
| RP_POS_665_51068a14_775 | RP | POS | 14.78 | 665.5107 | DG 38:5 | 665.5103(100) | 2 | 3.53E-01 | 0.11 | 0.69 | 4.09E-01 | 0.19 | 0.50 | 3.73E-01 | 0.21 | 0.50 | 7.05E-01 | 0.02 | 0.70 | 2035861 | 1147348 | 2654761 | 6315848 | 2128198 | 944917 | 3181324 | 8895798 |
| RP_POS_301_17847a9_141 | RP | POS | 9.14 | 301.1785 | Adrenosterone | 301.1745(100) | 2 | 3.27E-04 | -0.62 | 1.04 | 4.09E-01 | -0.17 | 0.68 | 4.74E-04 | -0.71 | 1.19 | 7.63E-03 | -0.53 | 1.10 | 6143161 | 238114 | 5939639 | 338394 | 5967726 | 341576 | 5911553 | 336264 |
| RP_NEG_279_23267a10_979 | RP | NEG | 10.98 | 279.2327 | FA 18:2 (Linoleic acid) | 279.2349(100) | 2 | 1.96E-02 | 0.41 | 1.43 | 4.11E-01 | -0.17 | 1.32 | 1.20E-01 | 0.32 | 1.18 | 1.40E-02 | 0.50 | 1.36 | 12552057 | 4667778 | 15046064 | 6404274 | 15576262 | 6183556 | 14515865 | 6637501 |
| RP_POS_326_7095a5_652 | RP | POS | 5.65 | 326.7095 | Midazolam | 326.0836(100), 291.1169(12.5) | 2 | 5.52E-07 | 0.60 | 1.30 | 4.12E-01 | -0.18 | 1.20 | 9.44E-04 | 0.51 | 1.37 | 1.90E-04 | 0.69 | 1.40 | 16097 | 28028 | 333009 | 590096 | 381740 | 639785 | 284279 | 537924 |
| RP_POS_166_08618a2_202 | RP | POS | 2.20 | 166.0862 | Phenylalanine | 120.0814(100), 166.0852(3) | 1 | 2.93E-01 | 0.16 | 0.69 | 4.16E-01 | 0.18 | 0.79 | 2.44E-01 | 0.25 | 0.71 | 6.39E-01 | 0.08 | 0.88 | 136273962 | 20951880 | 141771171 | 36442441 | 138786555 | 27143424 | 144755788 | 43911971 |
| HILIC_NEG_267_07355a2_387 | HILIC | NEG | 2.39 | 267.0736 | Inosine | 135.0308(100), 267.0735(20) | 2 | 7.28E-07 | 0.61 | 1.09 | 4.18E-01 | 0.18 | 0.73 | 4.25E-04 | 0.70 | 1.10 | 1.63E-04 | 0.52 | 1.41 | 832259 | 706355 | 2721892 | 3391594 | 2445132 | 2690788 | 2998652 | 3980441 |
| RP_NEG_552_36853a10_868 | RP | NEG | 10.87 | 552.3685 | LPC O-18:1 [M+HCOO]- | 492.3474(100), 78.9574(23), 168.0421(15), 403.2585(12) | 2 | 1.18E-02 | -0.62 | 1.11 | 4.20E-01 | -0.14 | 1.01 | 7.08E-03 | -0.69 | 1.24 | 3.69E-02 | -0.55 | 1.02 | 5279083 | 1958924 | 4296888 | 1380586 | 4408823 | 1507995 | 4184953 | 1245519 |
| HILIC_POS_585_27112a0_629 | HILIC | POS | 0.63 | 585.2711 | Bilirubin | 299.137(100), 585.2726(15) | 2 | 8.95E-01 | -0.03 | 0.61 | 4.21E-01 | 0.16 | 0.87 | 8.28E-01 | 0.05 | 0.11 | 6.18E-01 | -0.11 | 0.26 | 5149821 | 3229515 | 5063813 | 3134634 | 4810107 | 2571050 | 5317518 | 3620874 |
| RP_POS_478_29291a10_214 | RP | POS | 10.21 | 478.2929 | LPE 18:2 | 337.2718(100), 81.075(11), 60.0813(10) | 2 | 1.31E-01 | 0.30 | 1.05 | 4.26E-01 | -0.16 | 0.80 | 3.59E-01 | 0.22 | 0.75 | 6.58E-02 | 0.38 | 1.04 | 10213853 | 4844408 | 11753397 | 5229000 | 12172810 | 4236385 | 11333983 | 6077577 |
| RP_POS_360_27429a7_451 | RP | POS | 7.45 | 360.2743 | 3-Hydroxydodecanoyl carnitine | 85.0287(100), 360.274(75), 60.0815(22), 145.0492(7) | 1 | 6.31E-03 | 0.47 | 1.20 | 4.28E-01 | 0.17 | 0.98 | 6.98E-03 | 0.55 | 1.13 | 5.22E-02 | 0.38 | 1.13 | 366625 | 189970 | 488198 | 272773 | 466468 | 268116 | 509928 | 278350 |
| RP_POS_349_18338a3_84 | RP | POS | 3.84 | 349.1834 | Hydroquinide | 349.1806(100) | 2 | 9.85E-01 | -0.01 | 1.17 | 4.29E-01 | -0.12 | 1.13 | 8.15E-01 | -0.07 | 0.92 | 8.47E-01 | 0.06 | 0.96 | 1042639 | 3307351 | 1031017 | 1703008 | 1166495 | 1826131 | 895539 | 1577165 |
| RP_NEG_277_21768a10_735 | RP | NEG | 10.74 | 277.2177 | FA 18:3 (alfa-Linolenic acid) | 277.2188(100) | 2 | 1.71E-04 | 0.56 | 1.44 | 4.30E-01 | -0.17 | 1.08 | 5.42E-03 | 0.47 | 1.27 | 1.26E-03 | 0.65 | 1.47 | 1728649 | 856666 | 2618103 | 1707427 | 2753729 | 1877494 | 2482477 | 1525642 |
| RP_POS_357_27878a9_306 | RP | POS | 9.31 | 357.2788 | Ursodeoxycholic acid | 357.279(100) | 1 | 2.67E-02 | 0.35 | 1.34 | 4.36E-01 | -0.17 | 0.58 | 1.17E-01 | 0.27 | 0.89 | 3.95E-02 | 0.44 | 1.34 | 915513 | 682452 | 1288614 | 1134211 | 1377452 | 1305738 | 1199776 | 937051 |
| RP_POS_605_42249a10_446 | RP | POS | 10.45 | 605.4225 | DG 34:7 | 605.4208(100) | 2 | 5.45E-02 | -0.39 | 0.74 | 4.41E-01 | -0.15 | 0.47 | 3.03E-02 | -0.47 | 0.92 | 1.83E-01 | -0.31 | 0.62 | 10260635 | 2544909 | 9239521 | 2596078 | 9441009 | 2911231 | 9038033 | 2249148 |
| SCFA_Butyric acid | SCFA | SCFA | | | FA 04:0 (Butyric acid) | targeted_SCFA_analysis | 1 | 1.06E-04 | 0.68 | 1.46 | 4.41E-01 | 0.26 | 0.86 | 1.13E-02 | 0.83 | 1.65 | 9.16E-04 | 0.57 | 1.26 | 58021 | 16579 | 95665 | 60482 | 89560 | 43544 | 103628 | 77652 |
| RP_POS_548_37103a10_752 | RP | POS | 10.75 | 548.3710 | LPC 20:2 | 184.0733(100), 548.3658(40), 530.3571(8), 104.1076(85) | 2 | 1.66E-01 | 0.29 | 1.02 | 4.42E-01 | -0.15 | 0.86 | 3.76E-01 | 0.22 | 0.71 | 9.51E-02 | 0.37 | 0.99 | 8480351 | 3845579 | 9564763 | 3651667 | 9847076 | 3046809 | 9282449 | 4182795 |
| RP_NEG_854_59344a13_875 | RP | NEG | 13.88 | 854.5934 | PC 38:4\|PC 18:0_20:4 [M+HCOO]- | 303.2314(100), 283.2654(60), 794.5798(35) | 2 | 6.43E-01 | -0.10 | 0.51 | 4.43E-01 | 0.15 | 0.80 | 9.10E-01 | -0.03 | 0.49 | 4.40E-01 | -0.18 | 0.37 | 47166926 | 13343749 | 45946327 | 11385913 | 45066393 | 9225503 | 46826260 | 13237584 |
| RP_POS_772_58472a13_42 | RP | POS | 13.42 | 772.5847 | PC 35:2 | 184.0375(100), 772.5782(3) | 2 | 5.78E-06 | -1.07 | 1.81 | 4.44E-01 | 0.13 | 0.62 | 3.81E-05 | -1.00 | 1.70 | 4.52E-06 | -1.13 | 1.66 | 12361109 | 4429692 | 7949428 | 3420103 | 7685940 | 3360279 | 8212916 | 3492817 |
| RP_POS_768_55292a12_749 | RP | POS | 12.75 | 768.5529 | PC 35:4 | 184.0732(100), 768.5555(2) | 2 | 2.81E-07 | -1.24 | 2.28 | 4.52E-01 | -0.12 | 0.94 | 2.92E-07 | -1.30 | 2.15 | 1.12E-06 | -1.18 | 2.06 | 6690765 | 2363296 | 3933603 | 1713930 | 4063282 | 1545202 | 3803923 | 1874324 |
| RP_POS_393_20926a4_2 | RP | POS | 4.20 | 393.2093 | Octaethylene glycol | 393.2081(100) | 2 | 9.80E-01 | -0.01 | 1.20 | 4.53E-01 | -0.12 | 1.11 | 8.20E-01 | -0.07 | 0.93 | 8.61E-01 | 0.05 | 1.00 | 858259 | 2781646 | 845127 | 1423761 | 952673 | 1527502 | 737582 | 1318595 |
| RP_POS_778_53705a12_264 | RP | POS | 12.26 | 778.5371 | PC 36:6 | 184.0732(100), 778.5347(2) | 2 | 9.73E-08 | 0.80 | 1.42 | 4.55E-01 | 0.16 | 0.49 | 6.90E-06 | 0.88 | 1.53 | 3.99E-05 | 0.73 | 1.36 | 2076262 | 1164581 | 3832540 | 2268510 | 3661847 | 2123924 | 4003233 | 2413874 |
| RP_POS_482_32401a10_932 | RP | POS | 10.93 | 482.3240 | LPE 18:0 | 341.3073(100), 482.3236(3), 62.0607(25) | 2 | 4.86E-02 | 0.35 | 1.09 | 4.60E-01 | 0.16 | 1.03 | 5.73E-02 | 0.43 | 0.90 | 1.44E-01 | 0.27 | 0.97 | 11637003 | 3349306 | 13126806 | 4474901 | 12794057 | 3623624 | 13459555 | 5205913 |
| RP_POS_271_1156a4_569 | RP | POS | 4.57 | 271.1156 | 7-Prenyl-theophylline [M+Na]+ | 271.1138(100) | 2 | 1.38E-01 | 0.21 | 0.45 | 4.61E-01 | -0.16 | 0.57 | 3.59E-01 | 0.13 | 0.48 | 1.72E-01 | 0.29 | 0.78 | 5017487 | 299581 | 5145602 | 673770 | 5195594 | 832572 | 5095609 | 467889 |
| RP_POS_241_06731a4_851 | RP | POS | 4.85 | 241.0673 | Triacetin [M+Na]+ | 241.0692(100), 181.0459(55), 159.0647(15) | 2 | 5.71E-01 | 0.10 | 0.53 | 4.66E-01 | -0.15 | 0.54 | 8.97E-01 | 0.03 | 0.46 | 4.09E-01 | 0.18 | 0.55 | 127014501 | 37011469 | 131591705 | 46574044 | 135009565 | 49969992 | 128173845 | 43147548 |
| HILIC_NEG_243_06212a1_415 | HILIC | NEG | 1.42 | 243.0621 | Uridine | 99.9255(100), 110.025(60), 200.0576(12), 140.035(8) | 2 | 7.77E-01 | 0.05 | 0.85 | 4.67E-01 | -0.16 | 0.66 | 8.89E-01 | -0.03 | 0.62 | 5.40E-01 | 0.13 | 0.90 | 973097 | 315535 | 993117 | 429201 | 1024523 | 440275 | 961711 | 419918 |
| RP_POS_526_29272a10_209 | RP | POS | 10.21 | 526.2927 | LPE 22:6 | 385.2761(100), 93.0701(20) | 2 | 2.82E-02 | 0.35 | 1.13 | 4.70E-01 | -0.16 | 0.48 | 1.55E-01 | 0.27 | 0.79 | 3.05E-02 | 0.42 | 1.02 | 5383130 | 1794742 | 6372059 | 3098304 | 6597502 | 3186539 | 6146617 | 3022768 |
| RP_NEG_462_2998a10_799 | RP | NEG | 10.80 | 462.2998 | LPE O-18:2 | 462.2972(100), 78.9573(50), 140.0109(32), 401.2463(8) | 2 | 2.20E-02 | 0.36 | 0.69 | 4.71E-01 | 0.16 | 0.74 | 1.75E-02 | 0.44 | 1.04 | 1.56E-01 | 0.28 | 0.52 | 876008 | 285856 | 1040460 | 492855 | 1004744 | 525076 | 1076177 | 460936 |
| RP_POS_521_41681a13_623 | RP | POS | 13.62 | 521.4168 | DG 27:0 [M+Na]+ | 521.4131(100) | 2 | 8.98E-01 | 0.02 | 0.37 | 4.72E-01 | 0.16 | 0.51 | 6.60E-01 | 0.10 | 0.29 | 6.01E-01 | -0.06 | 0.20 | 7970697 | 876494 | 8003579 | 2039391 | 7855666 | 1097773 | 8151491 | 2674576 |
| RP_NEG_512_30035a9_796 | RP | NEG | 9.80 | 512.3004 | LPC 14:0 [M+HCOO]- | 227.1999(100), 452.2813(20) | 2 | 1.08E-03 | 0.49 | 0.99 | 4.75E-01 | -0.16 | 0.52 | 3.00E-02 | 0.42 | 0.88 | 1.82E-03 | 0.57 | 1.01 | 2813005 | 951238 | 3649756 | 1832976 | 3781555 | 1756298 | 3517958 | 1915238 |
| HILIC_POS_269_08807a2_457 | HILIC | POS | 2.46 | 269.0881 | Inosine | 137.0452(100) | 1 | 1.27E-08 | 0.69 | 1.17 | 4.77E-01 | 0.16 | 0.68 | 1.23E-04 | 0.76 | 1.19 | 9.46E-06 | 0.61 | 1.61 | 616313 | 556925 | 4680200 | 6498685 | 4215373 | 5123935 | 5145027 | 7657157 |
| RP_POS_205_09686a2_893 | RP | POS | 2.89 | 205.0969 | Tryptophan | 188.0694(100), 146.0603(50) | 2 | 1.27E-03 | -0.55 | 1.19 | 4.78E-01 | -0.15 | 1.06 | 4.17E-03 | -0.63 | 1.19 | 8.30E-03 | -0.48 | 1.14 | 195531238 | 33166336 | 170266727 | 47475128 | 173656253 | 39143306 | 166877201 | 54755166 |
| RP_POS_280_26343a9_476 | RP | POS | 9.48 | 280.2634 | Isomer of Linoleamide | 95.0861(100), 263.2354(85), 81.0701(80), 245.2251(78), 280.2631(77( | 1 | 3.51E-01 | -0.26 | 0.87 | 4.78E-01 | -0.11 | 0.49 | 2.74E-01 | -0.32 | 0.69 | 4.81E-01 | -0.21 | 0.56 | 51073097 | 93184363 | 34857843 | 48288913 | 38302393 | 50397795 | 31413294 | 46336534 |
| RP_POS_508_37546a10_864 | RP | POS | 10.86 | 508.3755 | LPC O-18:1 | 104.1074(100), 508.3809(20), 184.0729(15), 86.0969(11), 125.0001(5) | 2 | 2.16E-01 | -0.29 | 0.80 | 4.79E-01 | -0.13 | 1.07 | 1.55E-01 | -0.36 | 0.85 | 3.75E-01 | -0.22 | 0.72 | 13762126 | 5211367 | 12500057 | 4011929 | 12786176 | 4147950 | 12213938 | 3891924 |
| RP_POS_794_60358a14_273 | RP | POS | 14.27 | 794.6036 | PC O-38:5 | 184.0732(100), 794.6079(2) | 2 | 1.84E-01 | -0.25 | 0.45 | 4.79E-01 | -0.15 | 0.45 | 1.14E-01 | -0.32 | 0.77 | 4.34E-01 | -0.17 | 0.36 | 9099459 | 3799126 | 8009789 | 4537374 | 8332886 | 4997720 | 7686692 | 4050362 |
| RP_POS_327_00729a8_121 | RP | POS | 8.12 | 327.0073 | Tris(1-chloro-2-propyl)phosphate | 98.9848(100), 174.9912(15), 251.0011(3), 327.0083(1) | 2 | 8.82E-01 | -0.03 | 0.47 | 4.81E-01 | 0.14 | 0.54 | 8.46E-01 | 0.04 | 0.45 | 6.68E-01 | -0.10 | 0.26 | 2697591 | 549557 | 2680901 | 564614 | 2640853 | 630144 | 2720949 | 493688 |
| RP_POS_833_64868a14_816 | RP | POS | 14.82 | 833.6487 | SM 42:3;2O [M+Na]+ | 650.5846(100), 774.5735(60), 833.6593(45) | 2 | 6.84E-03 | -0.62 | 1.12 | 4.81E-01 | -0.13 | 0.43 | 5.93E-03 | -0.69 | 1.15 | 2.12E-02 | -0.56 | 0.98 | 3700607 | 1729577 | 2747557 | 1393262 | 2846328 | 1341976 | 2648785 | 1449540 |
| HILIC_NEG_128_03552a4_083 | HILIC | NEG | 4.08 | 128.0355 | Pyroglutamic acid | 128.0348(100), 99.9254(70) | 2 | 1.61E-06 | 0.75 | 1.31 | 4.85E-01 | 0.15 | 0.75 | 7.34E-05 | 0.82 | 1.29 | 7.19E-05 | 0.68 | 1.36 | 3026358 | 935988 | 4225100 | 1656981 | 4108585 | 1403447 | 4341614 | 1884102 |
| RP_POS_697_52405a12_209 | RP | POS | 12.21 | 697.5241 | SM 32:1;2O\|SM 16:1;2O/16:0 | 514.4564(100), 638.4449(75), 697.5195(45), 86.0968(55), 146.9816(50) | 2 | 1.70E-02 | -0.53 | 1.07 | 4.87E-01 | 0.13 | 0.51 | 4.59E-02 | -0.46 | 0.96 | 1.60E-02 | -0.60 | 0.99 | 6677836 | 2350756 | 5533015 | 2030298 | 5390854 | 2207390 | 5675177 | 1847845 |
| RP_NEG_498_29034a9_329 | RP | NEG | 9.33 | 498.2903 | BA 24:1;O3;T | 498.2869(100) | 2 | 3.58E-01 | 0.14 | 0.37 | 4.88E-01 | -0.15 | 0.57 | 7.42E-01 | 0.06 | 0.25 | 2.46E-01 | 0.22 | 0.57 | 763407 | 930596 | 985977 | 1761165 | 1108915 | 1738962 | 863039 | 1792170 |
| RP_POS_689_55872a12_472 | RP | POS | 12.47 | 689.5587 | SM 33:1;2O\|SM 17:1;2O/16:0 | 184.0732(100), 689.5507(1) | 2 | 4.03E-07 | -1.14 | 1.94 | 5.00E-01 | -0.11 | 0.45 | 2.87E-07 | -1.20 | 1.99 | 4.87E-06 | -1.09 | 1.73 | 7770546 | 2542305 | 4830612 | 2141337 | 4975905 | 2343282 | 4685318 | 1931365 |
| RP_POS_269_08749a0_71 | RP | POS | 0.71 | 269.0875 | Inosine | 137.0454(100) | 2 | 5.73E-09 | 0.72 | 1.33 | 5.01E-01 | 0.15 | 0.82 | 5.32E-05 | 0.79 | 1.42 | 1.13E-05 | 0.64 | 1.44 | 1813541 | 2525401 | 11765200 | 15151157 | 10737561 | 12675315 | 12792838 | 17348809 |
| HILIC_POS_316_24817a0_739 | HILIC | POS | 0.74 | 316.2482 | AC 10:0 (Decanoylcarnitine) | 85.0288(100), 316.2486(40) | 1 | 3.38E-01 | -0.16 | 0.43 | 5.01E-01 | -0.14 | 0.74 | 1.91E-01 | -0.24 | 0.49 | 6.82E-01 | -0.09 | 0.40 | 40185602 | 26760107 | 34311483 | 38476920 | 36916730 | 45113134 | 31706236 | 30693876 |
| RP_NEG_566_34497a10_62 | RP | NEG | 10.62 | 566.3450 | LPC 18:1 [M+HCOO]- | 281.2505(100), 506.3257(15) | 2 | 5.42E-03 | 0.61 | 1.35 | 5.03E-01 | -0.13 | 0.95 | 2.66E-02 | 0.55 | 1.10 | 3.18E-03 | 0.67 | 1.38 | 221223504 | 60554526 | 256125688 | 53763647 | 259755428 | 45130398 | 252495948 | 61452496 |
| RP_POS_286_20123a5_18 | RP | POS | 5.18 | 286.2012 | CAR 08:1 | 85.0287(100), 286.2001(60), 227.1275(15), 60.0815(10), 144.1012(2) | 2 | 9.80E-01 | 0.00 | 0.50 | 5.03E-01 | -0.14 | 0.52 | 7.14E-01 | -0.08 | 0.47 | 7.45E-01 | 0.07 | 0.28 | 8390101 | 5705101 | 8358390 | 7606952 | 8871245 | 7591542 | 7845535 | 7664468 |
| RP_NEG_476_27872a10_214 | RP | NEG | 10.21 | 476.2787 | LPE 18:2 | 279.2348(100), 476.2775(5), 214.0468(2) | 2 | 1.17E-01 | 0.31 | 1.06 | 5.04E-01 | -0.14 | 0.74 | 3.04E-01 | 0.24 | 0.77 | 6.77E-02 | 0.38 | 1.04 | 6116442 | 2901782 | 7067016 | 3061822 | 7272964 | 2479517 | 6861068 | 3564585 |
| RP_POS_811_66663a14_816 | RP | POS | 14.82 | 811.6666 | SM 42:3;2O | 184.0735(100), 811.5959(5) | 2 | 5.48E-03 | -0.65 | 1.16 | 5.08E-01 | -0.12 | 0.41 | 5.05E-03 | -0.71 | 1.23 | 1.57E-02 | -0.59 | 0.96 | 10023574 | 4435843 | 7520757 | 3441475 | 7750180 | 3274154 | 7291335 | 3619629 |
| RP_POS_228_19582a7_801 | RP | POS | 7.80 | 228.1958 | Isomer of myristic acid | 95.0857(100), 175.1473(70), 109.1013(60), 193.1584(44), 83.086(38), 81.0705(37) | 2 | 1.40E-01 | 0.27 | 0.61 | 5.11E-01 | -0.14 | 0.41 | 3.14E-01 | 0.20 | 0.48 | 1.31E-01 | 0.33 | 0.57 | 1696132 | 1203389 | 2089921 | 1558830 | 2193009 | 1746071 | 1986833 | 1356111 |
| RP_POS_792_58734a13_501 | RP | POS | 13.50 | 792.5873 | PC O-38:6\|PC O-18:2_20:4 | 184.0732(100), 792.5884(2) | 2 | 3.83E-01 | 0.16 | 0.41 | 5.11E-01 | 0.14 | 0.66 | 2.76E-01 | 0.23 | 0.51 | 6.63E-01 | 0.09 | 0.46 | 14305680 | 5008874 | 15247256 | 6026510 | 14848928 | 6152628 | 15645585 | 5932977 |
| RP_NEG_592_36322a10_754 | RP | NEG | 10.75 | 592.3632 | LPC 20:2 [M+HCOO]- | 307.2629(100), 532.3364(35), 224.0686(10) | 2 | 5.48E-02 | 0.40 | 1.08 | 5.13E-01 | -0.13 | 0.85 | 1.68E-01 | 0.33 | 0.81 | 3.24E-02 | 0.46 | 1.08 | 3933779 | 1783412 | 4642689 | 1753524 | 4758257 | 1446189 | 4527121 | 2023295 |
| RP_POS_703_57452a12_767 | RP | POS | 12.77 | 703.5745 | SM 34:1;2O\|SM 18:1;2O/16:0 | 184.0731(100), 703.5678(1) | 2 | 5.04E-02 | -0.40 | 0.78 | 5.13E-01 | 0.13 | 0.46 | 1.39E-01 | -0.34 | 0.63 | 4.02E-02 | -0.47 | 0.79 | 65151616 | 20742593 | 56707781 | 20821581 | 55336844 | 20669004 | 58078718 | 21092198 |
| RP_POS_280_26337a9_618 | RP | POS | 9.62 | 280.2634 | Isomer of Linoleamide | 95.0856(100), 263.2366(95), 81.0705(85), 280.2633(83), 245.2274(81) | 2 | 3.81E-01 | -0.24 | 0.85 | 5.13E-01 | -0.10 | 0.44 | 3.06E-01 | -0.30 | 0.67 | 5.07E-01 | -0.19 | 0.56 | 44356405 | 80386491 | 31211959 | 42202231 | 33989529 | 43397983 | 28434388 | 41222256 |
| RP_POS_224_12646a0_621 | RP | POS | 0.62 | 224.1265 | 4-Hydroxybenzoylcholine | 224.1285(100), 165.0546(35) | 2 | 1.44E-04 | -0.71 | 1.45 | 5.16E-01 | -0.13 | 0.96 | 2.69E-04 | -0.78 | 1.53 | 1.93E-03 | -0.65 | 1.25 | 3256972 | 1031004 | 2362769 | 1246849 | 2444270 | 1238463 | 2281268 | 1262397 |

**Supplementary table 1: Metabolomics results** (in asceding order according to p-value from progressive ALD vs  non-progressive ALD comparison, with molecular features with ID_level of 1-2 first)

| | | | | | | | | all_ALD_vs_control | | | Prog_ALD_vs_non-prog_ALD | | | Prog_ALD_vs_control_ALD | | | Non-prog_ALD_vs_control | | | Healthy_control | | all_ALD | | Non-prog_ALD | | Progressive_ALD | |
|---|---|---|---|---|---|---|---|---|---|---|---|---|---|---|---|---|---|---|---|---|---|---|---|---|---|---|---|
| | | | | | | | | t-test | Cohen's | PLS-DA | t-test | Cohen's | PLS-DA | t-test | Cohen's | PLS-DA | t-test | Cohen's | PLS-DA | | | | | | | | |
| Feature_ID | RP_HILIC | POS_NEG | Average.Rt | Average.Mz | Metabolite.name | curated_MSMS | ID_level | p | d | VIP | p | d | VIP | p | d | VIP | p | d | VIP | Mean | SD | Mean | SD | Mean | SD | Mean | SD |
| RP_POS_372_15863a4_998 | RP | POS | 5.00 | 372.1586 | Trazodone | 176.0813(100), 372.1578(95), 148.051(20) | 2 | 1.79E-02 | 0.33 | 0.87 | 5.17E-01 | 0.14 | 0.61 | 4.08E-02 | 0.41 | 0.98 | 1.06E-01 | 0.26 | 0.58 | 16272740 | 27347849 | 35474317 | 63734652 | 31314203 | 55372877 | 39634432 | 71453740 |
| RP_POS_206_08124a4_894 | RP | POS | 4.89 | 206.0812 | Indolelactic acid | 160.076(100), 188.0697(95), 206.0813(80), 118.0655(65), 130.0658(55), 146.0604(50), 170.0596(25) | 1 | 2.88E-02 | 0.32 | 0.70 | 5.22E-01 | -0.14 | 0.46 | 1.64E-01 | 0.25 | 0.58 | 4.11E-02 | 0.39 | 0.77 | 4956957 | 1583391 | 5926814 | 3368122 | 6144005 | 3518983 | 5709624 | 3231175 |
| RP_POS_468_30829a9_797 | RP | POS | 9.80 | 468.3083 | isomer of LPC 14:0 | 184.0735(100), 468.3025(5) | 1 | 2.07E-03 | 0.47 | 0.96 | 5.24E-01 | -0.14 | 0.46 | 4.01E-02 | 0.40 | 0.86 | 3.20E-03 | 0.54 | 0.97 | 5328266 | 1816484 | 6821623 | 3463392 | 7043898 | 3280413 | 6599348 | 3656914 |
| RP_POS_641_51044a14_987 | RP | POS | 14.99 | 641.5104 | DG 36:3 [M+Na]+ | 641.5031(100) | 2 | 1.60E-02 | -0.36 | 0.88 | 5.27E-01 | 0.14 | 0.40 | 2.11E-01 | -0.29 | 0.60 | 7.28E-04 | -0.43 | 1.22 | 13052776 | 6599062 | 8773886 | 13011373 | 7944179 | 5911730 | 9603593 | 17484085 |
| RP_POS_619_43799a10_66 | RP | POS | 10.66 | 619.4380 | DG 35:7 | 619.4352(100) | 2 | 5.86E-01 | -0.13 | 0.35 | 5.27E-01 | 0.12 | 0.38 | 7.81E-01 | -0.07 | 0.65 | 4.72E-01 | -0.19 | 0.33 | 17563798 | 1216923 | 17434626 | 952030 | 17374055 | 1060831 | 17495197 | 835684 |
| RP_POS_316_24829a7_242 | RP | POS | 7.24 | 316.2483 | CAR 10:0 (Decanoylcarnitine) | 85.0288(100), 316.2482(60), 257.173(12) | 1 | 4.31E-01 | -0.13 | 0.82 | 5.29E-01 | -0.14 | 1.12 | 2.49E-01 | -0.20 | 0.65 | 7.80E-01 | -0.06 | 0.72 | 37688743 | 24173527 | 33212645 | 37097010 | 35565302 | 44188674 | 30859988 | 28574935 |
| RP_NEG_524_27887a10_209 | RP | NEG | 10.21 | 524.2789 | LPE 22:6 | 327.2359(100), 214.0472(17), 524.2744(13), 78.9581(6) | 2 | 3.26E-02 | 0.34 | 1.11 | 5.29E-01 | -0.14 | 0.40 | 1.51E-01 | 0.27 | 0.76 | 3.79E-02 | 0.41 | 1.01 | 3233949 | 1093302 | 3811236 | 1826935 | 3926993 | 1876613 | 3695480 | 1787309 |
| RP_POS_468_30856a9_952 | RP | POS | 9.95 | 468.3086 | LPC 14:0 | 184.0731(100), 468.3103 | 1 | 1.81E-03 | 0.47 | 0.97 | 5.29E-01 | -0.14 | 0.46 | 3.65E-02 | 0.40 | 0.86 | 3.00E-03 | 0.54 | 0.98 | 45344790 | 15652041 | 58388159 | 29867795 | 60280060 | 28389760 | 56496259 | 31449849 |
| RP_NEG_804_57751a13_722 | RP | NEG | 13.72 | 804.5775 | PC 34:1\|PC 16:0_18:1 [M+HCOO]- | 281.2505(100), 255.2333(40), 744.5633(20) | 2 | 5.35E-10 | 1.18 | 1.89 | 5.32E-01 | 0.11 | 0.82 | 7.32E-09 | 1.23 | 1.91 | 2.15E-08 | 1.12 | 1.91 | 87874739 | 17784332 | 115791326 | 21319888 | 114449050 | 19892324 | 117133601 | 22780913 |
| RP_POS_725_5556a12_767 | RP | POS | 12.77 | 725.5556 | SM 34:1;2O\|SM 18:1;2O/16:0 | 542.4913(100), 666.4796(65), 725.5598(45), 502.4989(5), 86.0967(55), 146.9816(40) | 2 | 3.07E-02 | -0.44 | 0.86 | 5.34E-01 | 0.12 | 0.40 | 9.36E-02 | -0.38 | 0.71 | 2.70E-02 | -0.50 | 0.86 | 20749412 | 6408338 | 17839364 | 6594740 | 17426994 | 6621366 | 18251734 | 6609025 |
| RP_POS_217_14339a6_035 | RP | POS | 6.04 | 217.1434 | (2R)-2-[(2R,5S)-5-[(2S)-2-hydroxybutyl]oxolan-2-yl]propanoic acid | 125.0957(100), 97.1012(65), 217.105(50), 107.0857(25), 135.0811(20), 83.086(17), 203.1276(80) | 2 | 5.99E-01 | 0.08 | 0.72 | 5.38E-01 | -0.14 | 0.71 | 9.34E-01 | 0.02 | 0.75 | 4.33E-01 | 0.15 | 0.66 | 987881 | 129009 | 1004473 | 216055 | 1017872 | 215788 | 991073 | 217673 |
| RP_POS_254_24776a10_363 | RP | POS | 10.36 | 254.2478 | Palmitoleamide | 254.246(100), 83.0862(35), 219.2116(31), 97.1012(30), 69.0703(28), 237.2218(25) | 1 | 3.23E-04 | 0.48 | 0.87 | 5.39E-01 | 0.14 | 0.77 | 1.60E-02 | 0.55 | 0.87 | 7.08E-05 | 0.41 | 1.36 | 20340429 | 7779324 | 30005944 | 22246795 | 28628435 | 9999780 | 31383453 | 29934463 |
| RP_POS_176_07071a5_352 | RP | POS | 5.35 | 176.0707 | 3-Indoleacetic acid | 130.0655(100), 176.07(15) | 1 | 5.82E-01 | -0.12 | 0.39 | 5.48E-01 | 0.12 | 0.55 | 8.13E-01 | -0.06 | 0.40 | 4.29E-01 | -0.18 | 0.64 | 11947198 | 6025911 | 11280733 | 5557720 | 10944113 | 4747168 | 11617354 | 6296032 |
| RP_POS_137_04588a1_786 | RP | POS | 1.79 | 137.0459 | Hypoxanthine | 137.0456(100), 90.9482(30), 104.9641(28) | 1 | 2.08E-10 | 0.77 | 1.44 | 5.49E-01 | 0.13 | 0.78 | 2.29E-05 | 0.83 | 1.49 | 8.95E-07 | 0.70 | 1.64 | 18962 | 15981 | 5941841 | 8361684 | 5437496 | 6817378 | 6446187 | 9709102 |
| RP_POS_370_29498a8_445 | RP | POS | 8.45 | 370.2950 | CAR 14:1 | 85.029(100), 370.2926(65) | 2 | 6.13E-02 | 0.32 | 1.04 | 5.50E-01 | -0.13 | 1.47 | 1.52E-01 | 0.25 | 0.88 | 8.56E-02 | 0.38 | 0.98 | 9632693 | 5652766 | 12088475 | 8206654 | 12583107 | 9680527 | 11593843 | 6470001 |
| RP_POS_223_09589a7_494 | RP | POS | 7.49 | 223.0959 | Diethyl-phthalate | 149.0227(100), 177.0534(35), 223.0945(30) | 2 | 7.36E-01 | 0.07 | 0.40 | 5.54E-01 | 0.12 | 0.40 | 5.89E-01 | 0.13 | 0.38 | 9.47E-01 | 0.02 | 0.38 | 14571521 | 2424680 | 14733926 | 2144592 | 14606123 | 2049113 | 14861728 | 2249493 |
| RP_POS_338_34174a11_668 | RP | POS | 11.67 | 338.3417 | 13-Docosenamide | 338.3423(100), 83.086(50), 97.1011(48), 69.0707(45), 321.3165(32), 95.0856(30), 81.0704(28), 71.0881(25), | 2 | 9.34E-01 | -0.02 | 0.69 | 5.62E-01 | -0.12 | 0.34 | 7.36E-01 | -0.08 | 0.39 | 8.53E-01 | 0.04 | 0.45 | 300408471 | 155545546 | 297747518 | 159339634 | 307053555 | 162156516 | 288441480 | 157559220 |
| RP_POS_181_07202a2_801 | RP | POS | 2.80 | 181.0720 | Paraxanthine | 181.0714(100), 124.05(10) | 1 | 6.79E-01 | -0.08 | 0.44 | 5.63E-01 | -0.12 | 0.34 | 5.35E-01 | -0.14 | 0.44 | 9.28E-01 | -0.02 | 0.45 | 22872361 | 22954693 | 20868889 | 26076092 | 22386445 | 24443731 | 19351332 | 27777796 |
| HILIC_NEG_204_06667a0_701 | HILIC | NEG | 0.70 | 204.0667 | Indolelactic acid | 99.9254(100), 117.929(80), 158.061(35), 204.0672(30), 186.0561(15) | 2 | 1.83E-03 | 0.44 | 0.71 | 5.63E-01 | -0.13 | 0.55 | 3.42E-02 | 0.37 | 0.69 | 6.69E-03 | 0.50 | 0.88 | 401583 | 123922 | 518135 | 292733 | 535164 | 300445 | 501106 | 286837 |
| RP_NEG_852_57751a13_206 | RP | NEG | 13.21 | 852.5775 | PC 38:5\|PC 16:0_22:5 AND PC 18:1 20:4 [M+HCOO]- | 303.2311(100), 329.2491(85), 281.25(62), 255.2327(60), 792.5487(47) | 2 | 6.08E-05 | 0.75 | 1.29 | 5.64E-01 | 0.11 | 0.59 | 5.13E-04 | 0.81 | 1.25 | 2.05E-04 | 0.70 | 1.44 | 18740458 | 3477253 | 21940162 | 4186753 | 21696824 | 2999299 | 22183500 | 5128245 |
| RP_POS_264_19583a4_245 | RP | POS | 4.25 | 264.1958 | Tramadol | 58.0657(100), 264.1949(15) | 2 | 7.50E-02 | 0.24 | 0.64 | 5.66E-01 | -0.13 | 0.80 | 9.29E-02 | 0.17 | 0.99 | 1.83E-01 | 0.30 | 0.62 | 8515547 | 6466223 | 12401100 | 18383475 | 13466898 | 24667614 | 11335303 | 8484803 |
| RP_POS_673_52698a11_854 | RP | POS | 11.85 | 673.5270 | SM 32:2;2O\|SM 18:2;2O/14:0 | 184.0735(100), 673.5216(1) | 2 | 2.07E-05 | -0.92 | 1.68 | 5.67E-01 | -0.10 | 0.35 | 3.36E-05 | -0.98 | 1.62 | 1.29E-04 | -0.87 | 1.51 | 8740001 | 2638617 | 6265067 | 2408024 | 6403758 | 2327003 | 6126376 | 2502259 |
| RP_POS_476_27728a10_053 | RP | POS | 10.05 | 476.2773 | LPE 18:3 | 335.2592(100), 475.3276(45), 62.0608(40) | 2 | 8.14E-03 | 0.44 | 0.97 | 5.69E-01 | 0.12 | 0.92 | 2.20E-02 | 0.50 | 1.03 | 3.17E-02 | 0.37 | 1.04 | 2551975 | 1609965 | 3581253 | 2510618 | 3437109 | 2033129 | 3725397 | 2925582 |
| RP_POS_758_57062a13_16 | RP | POS | 13.16 | 758.5706 | PC 34:2\|PC 16:1_18:1 | 184.0731(100), 758.5703(3) | 2 | 5.08E-02 | -0.39 | 0.71 | 5.74E-01 | 0.11 | 0.45 | 1.31E-01 | -0.33 | 0.70 | 4.88E-02 | -0.44 | 0.79 | 883410931 | 108139484 | 838762607 | 116184921 | 832183905 | 119875550 | 845341309 | 113202860 |
| HILIC_NEG_137_02422a0_6 | HILIC | NEG | 0.60 | 137.0242 | Salicylic acid | 93.0344(100), 137.0243(35) | 2 | 9.12E-02 | 0.20 | 1.01 | 5.74E-01 | -0.13 | 0.55 | 4.16E-01 | 0.13 | 0.58 | 1.22E-01 | 0.26 | 0.46 | 519567 | 497048 | 2456543 | 11324196 | 3097669 | 11569911 | 1815417 | 11153036 |
| HILIC_NEG_131_04617a6_199 | HILIC | NEG | 6.20 | 131.0462 | L-Asparagine | 114.0195(100), 113.0327(60), 70.0292(45), 95.0236(25) | 2 | 5.75E-01 | -0.09 | 1.23 | 5.75E-01 | 0.12 | 0.77 | 9.04E-01 | -0.02 | 1.09 | 3.98E-01 | -0.15 | 1.17 | 624896 | 118232 | 608091 | 213206 | 596048 | 189079 | 620135 | 236209 |
| RP_NEG_311_29678a12_128 | RP | NEG | 12.13 | 311.2968 | FA 20:0 (Arachidic acid) | 311.297(100) | 2 | 3.85E-02 | -0.44 | 0.82 | 5.80E-01 | -0.11 | 0.77 | 2.83E-02 | -0.49 | 0.86 | 1.09E-01 | -0.39 | 0.73 | 1126993 | 283106 | 1006545 | 265703 | 1021340 | 294739 | 991750 | 235200 |
| RP_POS_250_18005a4_057 | RP | POS | 4.06 | 250.1801 | N,O-Didesmethylvenlafaxine | 250.181(100), 232.1693(90), 201.1271(35) | 2 | 6.69E-01 | 0.06 | 0.82 | 5.82E-01 | -0.12 | 0.51 | 9.90E-01 | 0.00 | 0.71 | 5.95E-01 | 0.12 | 0.92 | 12712287 | 4620417 | 13245028 | 9360119 | 13765324 | 12681721 | 12724732 | 3953874 |
| RP_POS_778_53485a12_761 | RP | POS | 12.76 | 778.5349 | PC 34:3 [M+Na]+ | 146.9817(100), 86.0968(55), 595.4709(45), 719.4662(25), 573.4848(13), 184.0727(65) | 2 | 2.26E-03 | 0.53 | 0.91 | 5.84E-01 | 0.11 | 0.42 | 4.74E-03 | 0.58 | 1.05 | 1.74E-02 | 0.47 | 0.87 | 15464081 | 7387793 | 20773023 | 10549162 | 20191767 | 10199307 | 21354279 | 10960173 |
| RP_POS_135_04413a4_201 | RP | POS | 4.20 | 135.0441 | Caffeic acid | 135.0439(100), 107.0502(1) | 2 | 5.41E-01 | -0.12 | 0.41 | 5.87E-01 | 0.11 | 0.42 | 7.72E-01 | -0.07 | 0.27 | 4.21E-01 | -0.18 | 0.42 | 4418869 | 878341 | 4307432 | 934303 | 4256382 | 898592 | 4358483 | 975127 |
| RP_NEG_616_36328a10_679 | RP | NEG | 10.68 | 616.3633 | LPC 22:4 [M+HCOO]- | 331.2642(100), 556.3359(40), 224.0689(15) | 2 | 3.07E-08 | 1.01 | 1.81 | 5.90E-01 | -0.10 | 0.69 | 8.41E-06 | 0.95 | 1.65 | 4.73E-08 | 1.06 | 1.91 | 1466780 | 684728 | 2407240 | 890388 | 2455594 | 758768 | 2358886 | 1010575 |
| RP_NEG_830_59357a14 | RP | NEG | 14.00 | 830.5936 | PC 36:2\|PC 18:0_18:2 [M+HCOO]- | 279.2348(100), 283.2654(35), 770.5653(22) | 2 | 1.62E-02 | -0.53 | 0.95 | 5.91E-01 | 0.10 | 0.44 | 4.59E-02 | -0.48 | 0.90 | 1.46E-02 | -0.58 | 0.95 | 92634970 | 30139630 | 77804413 | 26451668 | 76371481 | 25825761 | 79237346 | 27248864 |
| RP_POS_280_26428a10_59 | RP | POS | 10.59 | 280.2643 | Linoleamide | 95.0861(100), 263.2354(85), 81.0701(80), 245.2251(78), 280.2631(77) | 1 | 2.48E-04 | 0.63 | 1.13 | 5.96E-01 | -0.11 | 0.37 | 4.91E-03 | 0.57 | 0.98 | 5.14E-04 | 0.68 | 1.42 | 267038186 | 110285312 | 363730128 | 158490893 | 372192861 | 152033407 | 355267396 | 165804254 |
| RP_NEG_327_23331a10_908 | RP | NEG | 10.91 | 327.2333 | FA 22:6 (Docosahexaenoic acid) | 327.2354(100), 283.2422(90), 59.0125(60) | 2 | 8.19E-04 | 0.46 | 1.51 | 5.97E-01 | 0.12 | 0.65 | 8.36E-03 | 0.52 | 1.19 | 1.16E-02 | 0.40 | 1.41 | 34948557 | 14373270 | 50139335 | 36270685 | 48207895 | 31469336 | 52070774 | 40743602 |
| RP_POS_319_07315a8_805 | RP | POS | 8.81 | 319.0732 | Fenofibric acid | 233.036(100), 138.994(20), 121.028(10) | 2 | 7.67E-02 | 0.22 | 0.55 | 5.98E-01 | -0.12 | 0.89 | 3.39E-02 | 0.16 | 0.96 | 2.22E-01 | 0.28 | 0.42 | 26937156 | 30571846 | 53423095 | 138156730 | 60771980 | 189699101 | 46074211 | 49680328 |
| RP_POS_524_37073a10_823 | RP | POS | 10.82 | 524.3707 | LPC 18:0 | 184.0733(100), 524.373(4), 506.3576(1) | 2 | 8.68E-01 | -0.04 | 0.81 | 6.07E-01 | -0.10 | 1.02 | 7.35E-01 | -0.09 | 0.47 | 9.70E-01 | 0.01 | 0.76 | 76633060 | 27043115 | 75762690 | 20826831 | 76840482 | 19377230 | 74684897 | 22327522 |
| RP_POS_832_58173a13_652 | RP | POS | 13.65 | 832.5817 | PC 38:4 [M+Na]+ | 146.9822(100), 86.0971(50), 649.5156(45), 832.5833(30), 773.5108(25), 627.528(10) | 2 | 1.30E-05 | 0.93 | 1.62 | 6.09E-01 | -0.09 | 0.47 | 9.10E-05 | 0.88 | 1.61 | 2.52E-05 | 0.97 | 1.45 | 4576992 | 2097768 | 6614203 | 2001430 | 6717349 | 2064161 | 6511056 | 1952106 |
| HILIC_NEG_104_03526a6_129 | HILIC | NEG | 6.13 | 104.0353 | Serine | 74.0242(100), 72.0078(85), 104.0355(40) | 2 | 4.48E-01 | -0.11 | 1.17 | 6.09E-01 | -0.11 | 0.78 | 4.10E-01 | -0.17 | 1.20 | 7.39E-01 | -0.05 | 1.23 | 434664 | 100427 | 414031 | 204989 | 424593 | 172207 | 403470 | 234554 |
| RP_POS_181_07196a3_128 | RP | POS | 3.13 | 181.0720 | Theophylline | 181.0714(100), 124.05(10) | 1 | 5.99E-01 | -0.11 | 0.42 | 6.21E-01 | -0.10 | 0.30 | 4.99E-01 | -0.16 | 0.41 | 8.02E-01 | -0.06 | 0.46 | 24004588 | 25066833 | 21287511 | 26081356 | 22586103 | 24554396 | 19988919 | 27712756 |
| RP_POS_301_14124a9_441 | RP | POS | 9.44 | 301.1412 | Enterodiol | 301.1396(100) | 2 | 9.55E-01 | -0.01 | 0.20 | 6.21E-01 | -0.10 | 0.81 | 7.89E-01 | -0.06 | 0.37 | 8.50E-01 | 0.04 | 0.32 | 23196939 | 1508878 | 23179068 | 1642412 | 23260808 | 1448457 | 23097328 | 1827134 |
| RP_POS_723_53973a12_326 | RP | POS | 12.33 | 723.5397 | SM 34:2;2O\|SM 18:2;2O/16:0 | 540.4738(100), 664.4639(60), 723.552(35), 500.4831(5), 60.0815(85), 86.0968(55), 146.9818(45) | 2 | 2.70E-05 | -0.93 | 1.58 | 6.25E-01 | -0.09 | 0.38 | 4.70E-05 | -0.98 | 1.55 | 1.23E-04 | -0.89 | 1.52 | 17957657 | 4538358 | 13797057 | 3950766 | 13991695 | 3761828 | 13602418 | 4160167 |
| RP_POS_784_58533a13_398 | RP | POS | 13.40 | 784.5853 | PC 36:3 | 184.0735(100), 784.587(2) | 2 | 5.40E-01 | 0.14 | 0.48 | 6.25E-01 | -0.09 | 0.34 | 7.08E-01 | 0.09 | 0.46 | 4.47E-01 | 0.19 | 0.62 | 230433572 | 66764797 | 238443952 | 54138646 | 241106816 | 52587814 | 235781088 | 56052233 |
| HILIC_NEG_173_10446a6_934 | HILIC | NEG | 6.93 | 173.1045 | Arginine | 131.0821(100), 173.1049(3) | 2 | 4.80E-02 | 0.26 | 1.10 | 6.32E-01 | -0.11 | 0.79 | 2.38E-01 | 0.21 | 0.95 | 7.07E-02 | 0.32 | 1.17 | 1034788 | 283150 | 1227747 | 826753 | 1267611 | 823202 | 1187882 | 836710 |
| RP_POS_293_09753a0_622 | RP | POS | 0.62 | 293.0975 | Ethylenediaminetetraacetic acid EDTA | 160.0599(100), 132.0647(40), 293.0956(20) | 2 | 1.30E-04 | -0.54 | 1.19 | 6.37E-01 | 0.10 | 1.09 | 7.01E-03 | -0.48 | 1.18 | 1.25E-03 | -0.59 | 1.31 | 344120143 | 76564293 | 253987961 | 183489614 | 245274050 | 184551764 | 262701873 | 183874061 |
| RP_POS_466_32883a11_091 | RP | POS | 11.09 | 466.3288 | LPE O-18:1 | 293.3134(100), 466.3249(50), 312.3251(40), 448.3138(30), 86.0604(17) | 2 | 2.48E-01 | -0.26 | 0.81 | 6.42E-01 | -0.09 | 1.17 | 2.09E-01 | -0.30 | 0.87 | 3.86E-01 | -0.21 | 0.75 | 1729807 | 680119 | 1573120 | 589020 | 1600719 | 606868 | 1545520 | 575436 |
| RP_POS_832_58038a13_871 | RP | POS | 13.87 | 832.5804 | PC 38:4 [M+Na]+ | 184.0732(100), 810.5858(10), 146.982(50), 86.0969(30) | 2 | 4.04E-01 | -0.18 | 0.56 | 6.47E-01 | 0.09 | 0.71 | 5.79E-01 | -0.14 | 0.55 | 3.18E-01 | -0.23 | 0.43 | 72485785 | 20863863 | 69027981 | 18023057 | 68197476 | 14970895 | 69858486 | 20754639 |
| RP_POS_177_10231a1_38 | RP | POS | 1.38 | 177.1023 | Cotinine | 177.1026(100), 80.05(7), 98.0601(4), 105.9347(25), 102.0558(21) | 2 | 2.99E-05 | 0.70 | 1.22 | 6.49E-01 | 0.09 | 0.64 | 5.38E-04 | 0.75 | 1.16 | 2.46E-04 | 0.65 | 1.32 | 1548913 | 1218458 | 2806941 | 1854286 | 2721814 | 1524741 | 2892069 | 2146462 |
| RP_NEG_788_54688a12_835 | RP | NEG | 12.84 | 788.5469 | PC 33:2\|PC 15:0_18:2 [M+HCOO]- | 279.2347(100), 241.2187(40), 728.5233(20) | 2 | 1.53E-11 | -1.61 | 2.70 | 6.50E-01 | -0.06 | 0.84 | 8.98E-12 | -1.64 | 2.57 | 3.57E-11 | -1.58 | 2.33 | 4142222 | 1106158 | 2207653 | 780576 | 2243306 | 848076 | 2172000 | 713587 |
| RP_POS_314_23245a6_93 | RP | POS | 6.93 | 314.2325 | CAR 10:1 | 85.0287(100), 314.2295(55) | 2 | 1.01E-01 | -0.32 | 0.78 | 6.53E-01 | -0.09 | 0.55 | 1.13E-01 | -0.37 | 0.63 | 1.84E-01 | -0.28 | 0.86 | 4264808 | 1712469 | 3673122 | 1845631 | 3756655 | 1608975 | 3589589 | 2068619 |
| RP_POS_175_11873a0_512 | RP | POS | 0.51 | 175.1187 | Arginine | 175.1188(100), 60.0564(58), 70.0657(50), 116.071(45), 130.0979(20), 158.0928(20) | 1 | 3.93E-04 | 0.43 | 1.15 | 6.55E-01 | -0.10 | 1.11 | 1.90E-02 | 0.38 | 1.12 | 5.99E-03 | 0.48 | 1.23 | 2599035 | 574173 | 3712283 | 2877674 | 3841634 | 2986902 | 3582932 | 2788421 |
| RP_POS_572_37091a10_678 | RP | POS | 10.68 | 572.3709 | LPC 22:4 | 184.0733(100), 572.3646(25), 554.3591(5), 104.1076(90) | 2 | 2.71E-07 | 0.95 | 1.73 | 6.60E-01 | -0.09 | 0.65 | 3.78E-05 | 0.90 | 1.55 | 3.17E-07 | 0.99 | 1.87 | 3113946 | 1601976 | 5111163 | 2028865 | 5200994 | 1662158 | 5021331 | 2353158 |
| RP_NEG_255_23318a11_162 | RP | NEG | 11.16 | 255.2332 | FA 16:0 (Palmitic acid) | 255.2333(100) | 2 | 1.37E-01 | 0.26 | 1.05 | 6.62E-01 | -0.09 | 1.01 | 2.90E-01 | 0.21 | 0.91 | 1.39E-01 | 0.31 | 1.04 | 10526365 | 2451524 | 11350402 | 3344242 | 11497585 | 3423933 | 11203219 | 3290689 |
| RP_NEG_474_26349a10_052 | RP | NEG | 10.05 | 474.2635 | LPE 18:3 | 277.2187(100), 474.2629(17), 196.0374(13), 78.9574(5), 214.0472(3) | 2 | 7.18E-03 | 0.44 | 0.98 | 6.62E-01 | 0.09 | 0.73 | 2.33E-02 | 0.49 | 0.97 | 2.57E-02 | 0.39 | 1.01 | 1613306 | 1025964 | 2285009 | 1628497 | 2213316 | 1354091 | 2356702 | 1874586 |
| RP_POS_361_19992a6_798 | RP | POS | 6.80 | 361.1999 | Cortisone | 361.2028(100), 163.1108(50) | 1 | 1.22E-01 | 0.28 | 1.39 | 6.67E-01 | 0.09 | 0.38 | 1.06E-01 | 0.32 | 0.92 | 2.87E-01 | 0.23 | 1.27 | 1663070 | 350644 | 1784397 | 464106 | 1764314 | 503061 | 1804479 | 425784 |
| RP_POS_643_52563a11_838 | RP | POS | 11.84 | 643.5256 | DG 36:2 | 643.5267(100) | 2 | 3.11E-01 | -0.35 | 0.85 | 6.68E-01 | -0.03 | 0.38 | 2.90E-01 | -0.37 | 0.63 | 3.39E-01 | -0.33 | 0.71 | 3725506 | 9070868 | 2063702 | 1886235 | 2145228 | 2059932 | 1982176 | 1712159 |
| RP_NEG_233_15483a9_107 | RP | NEG | 9.11 | 233.1548 | FA 15:4 | 233.1547(100) | 2 | 1.20E-01 | 0.31 | 0.62 | 6.71E-01 | -0.09 | 0.57 | 2.13E-01 | 0.27 | 0.52 | 1.27E-01 | 0.36 | 0.66 | 1375937 | 80108 | 1401903 | 83554 | 1405475 | 90835 | 1398330 | 76340 |
| RP_POS_356_27927a8_084 | RP | POS | 8.08 | 356.2793 | CAR 13:1 | 85.0289(100), 356.2804(75) | 2 | 4.91E-02 | -0.37 | 0.73 | 6.80E-01 | -0.09 | 0.26 | 8.02E-02 | -0.41 | 0.63 | 8.66E-02 | -0.33 | 0.85 | 2805312 | 1246859 | 2271661 | 1486055 | 2333462 | 1113532 | 2209860 | 1792774 |
| RP_NEG_747_56763a12_77 | RP | NEG | 12.77 | 747.5676 | SM 34:1;2O [M+HCOO]- | 687.5526(100), 168.0422(90), 78.9574(70) | 2 | 6.60E-02 | -0.38 | 0.78 | 6.82E-01 | 0.08 | 0.27 | 1.39E-01 | -0.34 | 0.66 | 6.60E-02 | -0.42 | 0.73 | 19181533 | 5643149 | 17031042 | 5622379 | 16798901 | 5606800 | 17263183 | 5685176 |
| RP_NEG_552_33093a10_39 | RP | NEG | 10.39 | 552.3309 | LPC 17:1 [M+HCOO]- | 267.2311(100), 492.3061(40), 224.0688(10) | 2 | 8.45E-04 | 0.58 | 1.20 | 6.86E-01 | 0.08 | 0.86 | 3.76E-03 | 0.62 | 1.15 | 4.48E-03 | 0.54 | 1.13 | 2527326 | 936737 | 3261859 | 1306827 | 3208562 | 1154120 | 3315156 | 1453497 |
| RP_POS_522_35614a10_619 | RP | POS | 10.62 | 522.3561 | LPC 18:1 (18:1/0:0) | 184.0733(100), 522.3521(30), 504.3383(6), 104.1077(80) | 2 | 1.88E-03 | 0.64 | 1.41 | 6.90E-01 | -0.08 | 0.90 | 1.20E-02 | 0.60 | 1.17 | 1.37E-03 | 0.68 | 1.45 | 642570021 | 211946767 | 783412433 | 210869915 | 791883872 | 166949297 | 774940994 | 248638666 |
| RP_NEG_719_53613a12_212 | RP | NEG | 12.21 | 719.5361 | SM 32:1;2O [M+HCOO]- | 659.5084(100), 168.0419(70), 78.9579(60) | 2 | 1.57E-02 | -0.55 | 1.08 | 6.92E-01 | 0.07 | 0.36 | 3.10E-02 | -0.51 | 1.02 | 1.96E-02 | -0.59 | 1.02 | 9356178 | 2901327 | 7935443 | 2395881 | 7839715 | 2617002 | 8031172 | 2174909 |
| HILIC_POS_162_11221a4_832 | HILIC | POS | 4.83 | 162.1122 | Carnitine | 162.1135(100), 103.0395(25), 60.0815(20) | 1 | 1.27E-02 | 0.43 | 1.02 | 6.92E-01 | -0.08 | 0.60 | 7.43E-02 | 0.38 | 0.65 | 1.21E-02 | 0.47 | 0.90 | 1634011394 | 449777697 | ######### | 648622698 | 1922026229 | 558316812 | 1870206254 | 732748959 |
| HILIC_POS_290_13861a0_815 | HILIC | POS | 0.82 | 290.1386 | Benzoylecgonine | 168.101(100), 290.1383(80), 105.0342(10) | 2 | 2.55E-01 | 0.15 | 0.53 | 6.95E-01 | -0.09 | 0.32 | 2.24E-01 | 0.10 | 0.65 | 4.02E-01 | 0.19 | 0.48 | 25629873 | 23169226 | 34892575 | 69925189 | 37660295 | 96455687 | 32124855 | 23654125 |
| RP_POS_834_59906a13_712 | RP | POS | 13.71 | 834.5991 | PC 40:6\|PC 18:0_22:6 | 184.0735(100), 834.5901(2) | 2 | 2.55E-02 | -0.47 | 1.06 | 7.03E-01 | 0.08 | 0.62 | 7.03E-02 | -0.43 | 0.82 | 2.40E-02 | -0.50 | 1.08 | 52296554 | 18601535 | 43650946 | 18179607 | 42951716 | 16536842 | 44350177 | 19831191 |
| HILIC_POS_193_09724a0_67 | HILIC | POS | 0.67 | 193.0972 | trans-3-Hydroxycotinine | 193.0967(100), 134.0603(10) | 2 | 3.81E-10 | 0.87 | 1.55 | 7.03E-01 | 0.08 | 0.47 | 1.69E-06 | 0.91 | 1.56 | 1.38E-06 | 0.83 | 1.83 | 2373358 | 3323212 | 9315459 | 8338632 | 8994888 | 7850491 | 9636030 | 8868183 |
| RP_POS_295_1889a9_271 | RP | POS | 9.27 | 295.1889 | Cinchonine | 295.1871(100), 277.2146(5) | 2 | 6.77E-01 | -0.09 | 0.62 | 7.05E-01 | -0.07 | 0.37 | 5.91E-01 | -0.13 | 0.27 | 8.26E-01 | -0.05 | 0.54 | 2354727 | 367035 | 2324170 | 334602 | 2336923 | 337973 | 2311418 | 334133 |
| RP_POS_278_21146a5_275 | RP | POS | 5.28 | 278.2115 | Venlafaxine | 278.113(25), 58.0657(100), 121.0646(10) | 2 | 6.95E-01 | 0.06 | 0.98 | 7.09E-01 | 0.08 | 0.54 | 5.95E-01 | 0.10 | 0.64 | 9.32E-01 | 0.02 | 0.86 | 51434856 | 21637735 | 53745595 | 44598410 | 52066106 | 44134648 | 55425083 | 45442326 |
| HILIC_POS_264_19559a0_623 | HILIC | POS | 0.62 | 264.1956 | Tramadol | 58.0658(100), 264.1966(15) | 2 | 2.75E-02 | 0.26 | 0.92 | 7.10E-01 | -0.09 | 0.51 | 1.34E-01 | 0.21 | 0.72 | 1.07E-01 | 0.30 | 0.81 | 50682 | 255102 | 1847580 | 8018984 | 2147794 | 9033989 | 1547366 | 6937121 |
| RP_POS_428_37332a9_66 | RP | POS | 9.66 | 428.3733 | CAR 18:0 (Octadecanoylcarnitine) | 85.0289(100), 428.3723(55) | 1 | 1.49E-02 | 0.47 | 0.79 | 7.20E-01 | -0.07 | 0.54 | 5.00E-02 | 0.43 | 0.83 | 1.87E-02 | 0.50 | 0.95 | 2169652 | 687330 | 2533545 | 793045 | 2562199 | 769360 | 2504891 | 822853 |
| RP_NEG_562_31641a9_896 | RP | NEG | 9.90 | 562.3164 | LPC 18:3 [M+HCOO]- | 277.2187(100), 502.2986(16) | 2 | 1.77E-01 | 0.25 | 0.71 | 7.23E-01 | -0.07 | 0.68 | 3.26E-01 | 0.21 | 0.78 | 1.71E-01 | 0.29 | 0.75 | 922447 | 678064 | 1121691 | 835280 | 1151515 | 809490 | 1091867 | 867488 |
| RP_POS_259_19034a9_529 | RP | POS | 9.53 | 259.1903 | Dibutyladipate | 129.054(100), 111.044(95), 101.0597(62), 185.1172(50) | 2 | 1.87E-02 | -0.45 | 0.76 | 7.33E-01 | -0.07 | 0.36 | 2.10E-02 | -0.49 | 0.90 | 6.13E-02 | -0.42 | 0.63 | 14822955 | 438317 | 14599883 | 500056 | 14617058 | 536052 | 14582707 | 466113 |

**Supplementary table 1: Metabolomics results** (in asceding order according to p-value from progressive ALD vs non-progressive ALD comparison, with molecular features with ID_level of 1-2 first)

| Feature_ID | RP_HILIC | POS_NEG | Average.Rt | Average.Mz | Metabolite.name | curated_MSMS | ID_level | all_ALD_vs_control t-test p | all_ALD_vs_control Cohen's d | all_ALD_vs_control PLS-DA VIP | Prog_ALD_vs_non-prog_ALD t-test p | Prog_ALD_vs_non-prog_ALD Cohen's d | Prog_ALD_vs_non-prog_ALD PLS-DA VIP | Prog_ALD_vs_control_ALD t-test p | Prog_ALD_vs_control_ALD Cohen's d | Prog_ALD_vs_control_ALD PLS-DA VIP | Non-prog_ALD_vs_control t-test p | Non-prog_ALD_vs_control Cohen's d | Non-prog_ALD_vs_control PLS-DA VIP | Healthy_control Mean | Healthy_control SD | all_ALD Mean | all_ALD SD | Non-prog_ALD Mean | Non-prog_ALD SD | Progressive_ALD Mean | Progressive_ALD SD |
|---|---|---|---|---|---|---|---|---|---|---|---|---|---|---|---|---|---|---|---|---|---|---|---|---|---|---|---|
| RP_POS_137_04588a1_043 | RP | POS | 1.04 | 137.0459 | Hypoxanthine | 137.0455(100), 118.9426(10), 110.0345(1), 90.9483(35) | 2 | 1.92E-12 | 0.86 | 1.65 | 7.36E-01 | 0.07 | 0.80 | 2.32E-06 | 0.90 | 1.66 | 1.39E-07 | 0.82 | 1.80 | 439489 | 248933 | 5144824 | 5840917 | 4946302 | 5181871 | 5343347 | 6480483 |
| RP_POS_392_27905a8_113 | RP | POS | 8.11 | 392.2791 | CAR 16:4 | 392.2112(100), 85.0287(55) | 2 | 7.15E-01 | 0.06 | 0.93 | 7.40E-01 | -0.07 | 0.83 | 9.15E-01 | 0.02 | 0.54 | 5.66E-01 | 0.10 | 1.07 | 646880 | 330891 | 675482 | 515795 | 692767 | 382496 | 658196 | 624984 |
| RP_NEG_528_31067a10_684 | RP | NEG | 10.68 | 528.3107 | LPE 22:4 | 331.2644(100), 196.0375(20), 528.3052(19), 78.9581(7), 213.6675(5) | 2 | 4.31E-15 | 1.05 | 1.86 | 7.43E-01 | 0.07 | 0.24 | 1.47E-07 | 1.08 | 1.76 | 4.39E-12 | 1.02 | 2.09 | 528511 | 249574 | 1279950 | 719757 | 1256173 | 540359 | 1303726 | 868061 |
| RP_POS_162_11258a0_532 | RP | POS | 0.53 | 162.1126 | Carnitine | 162.112(100), 103.0398(17), 60.0815(14) | 1 | 4.28E-03 | 0.43 | 0.75 | 7.44E-01 | -0.07 | 0.58 | 5.98E-02 | 0.39 | 0.68 | 4.65E-03 | 0.46 | 1.22 | 57941749 | 9420896 | 65258198 | 18722540 | 65875206 | 15243603 | 64641190 | 21796247 |
| RP_POS_508_33942a10_389 | RP | POS | 10.39 | 508.3394 | LPC 17:1 | 184.0736(100), 508.3387(30), 490.3309(8), 104.1069(80) | 2 | 4.84E-04 | 0.59 | 1.22 | 7.47E-01 | 0.07 | 0.80 | 3.77E-03 | 0.62 | 1.15 | 2.35E-03 | 0.55 | 1.18 | 6163829 | 2422772 | 8208925 | 3634819 | 8090973 | 3095735 | 8326878 | 4132975 |
| RP_NEG_480_31073a10_825 | RP | NEG | 10.83 | 480.3107 | LPE 18:0 | 283.2652(100), 480.3055(4), 214.0472(2), 196.0375(1.5) | 2 | 3.58E-01 | 0.17 | 0.99 | 7.50E-01 | -0.07 | 0.94 | 5.36E-01 | 0.14 | 0.67 | 3.12E-01 | 0.21 | 0.92 | 1466967 | 525073 | 1570032 | 614795 | 1589799 | 543705 | 1550266 | 683554 |
| RP_POS_138_05482a0_59 | RP | POS | 0.59 | 138.0548 | Trigonelline | 138.0541(100), 94.066(2) | 2 | 3.43E-01 | 0.16 | 0.41 | 7.55E-01 | 0.07 | 0.41 | 3.61E-01 | 0.19 | 0.49 | 5.07E-01 | 0.12 | 0.57 | 3272951 | 2314067 | 3784983 | 3479730 | 3675755 | 3149115 | 3894211 | 3810891 |
| RP_POS_202_18037a0_709 | RP | POS | 0.71 | 202.1804 | 4-Hydroxy-1-(2-hydroxyethyl)-2,2,6,6-tetramethylpiperidine | 202.1808(100), 102.092(25), 62.067(10) | 2 | 7.86E-01 | -0.05 | 0.52 | 7.58E-01 | -0.06 | 0.39 | 6.90E-01 | -0.09 | 0.40 | 9.24E-01 | -0.02 | 0.53 | 2517635 | 1089386 | 2455993 | 1172567 | 2492434 | 1274507 | 2419553 | 1072759 |
| RP_POS_232_15433a1_769 | RP | POS | 1.77 | 232.1543 | CAR 04:0 | 85.0288(100), 232.1552(55), 173.0817(20), 60.0815(6) | 2 | 2.16E-05 | 0.54 | 0.89 | 7.58E-01 | -0.07 | 0.80 | 7.08E-03 | 0.51 | 0.92 | 2.68E-04 | 0.57 | 1.12 | 2064631 | 829454 | 3570267 | 3082336 | 3665876 | 2739915 | 3474658 | 3416093 |
| RP_POS_235_18044a3_588 | RP | POS | 3.59 | 235.1804 | Lidocaine | 86.0966(100), 235.1806(30) | 2 | 5.15E-03 | -0.98 | 1.94 | 7.59E-01 | 0.01 | 0.29 | 5.43E-03 | -0.97 | 1.47 | 4.96E-03 | -0.98 | 1.43 | 20065082 | 33880888 | 2014217 | 3331720 | 1911146 | 3189974 | 2117288 | 3497090 |
| HILIC_POS_104_10756a1_472 | HILIC | POS | 1.47 | 104.1076 | Choline | 104.1071(100), 60.0815(10) | 1 | 1.83E-01 | 0.22 | 0.77 | 7.59E-01 | -0.07 | 0.76 | 3.65E-01 | 0.19 | 0.31 | 1.79E-01 | 0.25 | 0.48 | 493867570 | 124397242 | 532838667 | 189251515 | 538681304 | 174174145 | 526996031 | 204833870 |
| RP_POS_348_28735a10_06 | RP | POS | 10.06 | 348.2874 | NAE 20:4 | 348.2853(100), 60.0816(7), 229.3589(3) | 2 | 2.96E-01 | -0.29 | 0.97 | 7.61E-01 | -0.05 | 0.36 | 2.76E-01 | -0.32 | 0.72 | 3.54E-01 | -0.27 | 0.62 | 6273951 | 9626486 | 4390550 | 5036950 | 4544924 | 5072329 | 4236175 | 5048004 |
| RP_NEG_526_29449a10_455 | RP | NEG | 10.46 | 526.2945 | LPE 22:5 | 329.2492(100), 196.0373(24), 526.298(24), 78.9574(9), 214.0502(5) | 2 | 1.55E-14 | 1.04 | 1.81 | 7.63E-01 | 0.06 | 0.46 | 1.16E-07 | 1.07 | 1.74 | 6.06E-11 | 1.01 | 1.97 | 2340255 | 877228 | 4833635 | 2417566 | 4760098 | 1947424 | 4907173 | 2829317 |
| RP_POS_120_0807a1_451 | RP | POS | 1.45 | 120.0807 | Indoline | 120.0814(100), 103.0546(16) | 2 | 2.11E-01 | 0.20 | 0.64 | 7.64E-01 | 0.07 | 0.37 | 2.79E-01 | 0.24 | 0.61 | 3.18E-01 | 0.17 | 0.77 | 11711349 | 2585834 | 12488764 | 4132979 | 12363926 | 3258360 | 12613601 | 4884968 |
| RP_POS_286_20123a5_543 | RP | POS | 5.54 | 286.2012 | CAR 08:1 (Octenoyl-L-carnitine) | 85.287(100), 286.2004(55), 60.0815(14), 227.128(11) | 1 | 3.33E-01 | 0.17 | 0.75 | 7.72E-01 | -0.06 | 0.73 | 5.07E-01 | 0.14 | 0.49 | 3.27E-01 | 0.20 | 0.90 | 1063170 | 444287 | 1160851 | 622774 | 1179040 | 617832 | 1142662 | 633419 |
| RP_POS_766_53497a12_834 | RP | POS | 12.83 | 766.5350 | PC 33:2 [M+Na]+ | 146.982(100), 766.5388(45), 86.097(40), 583.4727(35), 707.4636(25), 561.4822(13), 184.0732(16) | 2 | 1.08E-09 | -1.53 | 2.55 | 7.72E-01 | -0.04 | 0.65 | 8.69E-10 | -1.54 | 2.42 | 2.18E-09 | -1.51 | 2.18 | 5598485 | 1653919 | 3132337 | 1055330 | 3163116 | 1153644 | 3101559 | 957782 |
| RP_POS_207_09889a5_341 | RP | POS | 5.34 | 207.0989 | p-methoxycinnamic acid ethyl ester | 207.978(100) | 2 | 8.74E-01 | 0.03 | 0.36 | 7.74E-01 | 0.06 | 0.22 | 7.82E-01 | 0.06 | 0.18 | 9.96E-01 | 0.00 | 0.23 | 503348 | 168743 | 508964 | 186619 | 503573 | 189878 | 514355 | 185071 |
| RP_POS_528_30847a10_456 | RP | POS | 10.46 | 528.3085 | LPE 22:5 | 387.2857(100), 528.3135(6), 62.0608(50) | 2 | 1.82E-13 | 1.02 | 1.78 | 7.85E-01 | 0.06 | 0.48 | 2.05E-07 | 1.05 | 1.72 | 3.14E-10 | 0.99 | 1.92 | 3929779 | 1554351 | 7880602 | 3913990 | 7773118 | 3209279 | 7988086 | 4541830 |
| HILIC_NEG_124_00751a5_262 | HILIC | NEG | 5.26 | 124.0075 | Taurine | 124.0074(100), 79.9569(60) | 2 | 1.26E-04 | 0.48 | 1.11 | 7.90E-01 | 0.06 | 0.76 | 4.99E-03 | 0.51 | 1.16 | 5.96E-03 | 0.45 | 1.26 | 1428645 | 392639 | 2173114 | 1747362 | 2126127 | 1655556 | 2220102 | 1850267 |
| RP_POS_337_23535a10_16 | RP | POS | 10.16 | 337.2354 | 8-hydroxy-8-(3-octyloxiran-2-yl)octanoic acid | 337.236(100) | 2 | 4.76E-02 | -0.44 | 0.79 | 7.97E-01 | 0.05 | 0.45 | 7.95E-02 | -0.42 | 0.70 | 5.64E-02 | -0.47 | 0.85 | 2303486 | 426936 | 2133210 | 360784 | 2123865 | 376286 | 2142555 | 348164 |
| RP_POS_701_55817a12_326 | RP | POS | 12.33 | 701.5582 | SM 34:2;2O\|SM 18:2;2O/16:0 | 184.0732(100), 701.5657(1) | 2 | 1.09E-04 | -0.77 | 1.30 | 8.05E-01 | 0.05 | 0.46 | 8.84E-04 | -0.75 | 1.24 | 2.14E-04 | -0.79 | 1.36 | 54398915 | 12108276 | 43952049 | 13091718 | 43627078 | 12210424 | 44277020 | 14034743 |
| RP_POS_132_10216a0_712 | RP | POS | 0.71 | 132.1022 | Isoleucine | 86.0971(100), 131.9745(10), 113.9642(30) | 1 | 6.06E-01 | 0.09 | 0.65 | 8.06E-01 | -0.05 | 0.62 | 7.71E-01 | 0.06 | 0.46 | 5.47E-01 | 0.12 | 0.31 | 160933478 | 28469498 | 164269687 | 40055047 | 165262736 | 36055529 | 163276639 | 44040243 |
| RP_POS_180_06563a3_625 | RP | POS | 3.63 | 180.0656 | Hippuric acid | 105.0335(100), 95.0497(1), 77.0394(0.5) | 1 | 2.15E-02 | -0.50 | 0.88 | 8.09E-01 | 0.05 | 0.78 | 5.35E-02 | -0.47 | 0.76 | 2.14E-02 | -0.52 | 1.08 | 14629099 | 12569778 | 8682900 | 11514323 | 8402188 | 9951182 | 8963611 | 12987616 |
| RP_POS_424_34186a9_11 | RP | POS | 9.11 | 424.3419 | CAR 18:2 | 85.0289(100), 424.3397(70) | 2 | 1.14E-04 | 0.64 | 1.05 | 8.09E-01 | 0.05 | 0.16 | 9.12E-04 | 0.67 | 1.13 | 1.39E-03 | 0.62 | 1.16 | 5288946 | 1931920 | 7124655 | 2971845 | 7052386 | 2886054 | 7196923 | 3082857 |
| HILIC_NEG_118_05093a5_743 | HILIC | NEG | 5.74 | 118.0509 | Threonine | 74.0243(100), 72.0078(60), 117.9291(45), | 2 | 2.85E-01 | -0.20 | 1.01 | 8.09E-01 | 0.05 | 0.68 | 4.67E-01 | -0.17 | 0.87 | 2.37E-01 | -0.22 | 0.88 | 1367000 | 383063 | 1277592 | 479347 | 1265930 | 358191 | 1289253 | 579360 |
| RP_POS_737_50073a10_467 | RP | POS | 10.47 | 737.5007 | DG 44:11 | 737.4964(100) | 2 | 1.59E-01 | -0.30 | 0.99 | 8.12E-01 | -0.05 | 0.68 | 1.89E-01 | -0.32 | 0.81 | 2.16E-01 | -0.27 | 0.81 | 3189125 | 469781 | 3054471 | 444621 | 3065133 | 381121 | 3043808 | 503909 |
| RP_POS_794_6048a13_71 | RP | POS | 13.71 | 794.6048 | PC O-38:5\|PC O-18:1_20:4 | 184.0735(100), 794.6074(3) | 2 | 3.01E-05 | -0.89 | 1.38 | 8.13E-01 | 0.04 | 0.51 | 1.11E-04 | -0.86 | 1.41 | 9.85E-05 | -0.91 | 1.38 | 41457806 | 9241474 | 32944335 | 8804584 | 32734176 | 9398958 | 33154494 | 8257898 |
| HILIC_POS_137_04573a1_385 | HILIC | POS | 1.39 | 137.0457 | Hypoxanthine | 137.0464(100) | 1 | 1.85E-15 | 0.99 | 1.68 | 8.15E-01 | -0.05 | 0.63 | 1.76E-07 | 0.96 | 1.63 | 1.71E-09 | 1.01 | 2.13 | 13278742 | 6945242 | 73969884 | 63907985 | 75479832 | 59482359 | 72459935 | 68622217 |
| RP_POS_287_10013a3_743 | RP | POS | 3.74 | 287.1001 | (4R)-3-methylidene-4-[(E)-3-methyl-4-(4-methyl-5-oxooxolan-2-yl)but-2-enyl]oxolan-2-one | 287.0972(100) | 2 | 7.37E-02 | -0.37 | 0.66 | 8.29E-01 | -0.04 | 0.26 | 1.06E-01 | -0.39 | 0.63 | 1.08E-01 | -0.34 | 0.73 | 2670017 | 1728836 | 2028013 | 1742726 | 2066033 | 1479249 | 1989993 | 1986213 |
| RP_POS_279_09348a7_822 | RP | POS | 7.82 | 279.0935 | Triphenylphosphine oxide | 279.0936(100), 219.0576(10), 201.046(3) | 2 | 8.16E-02 | -0.36 | 0.68 | 8.37E-01 | -0.04 | 0.53 | 9.47E-02 | -0.38 | 0.74 | 1.39E-01 | -0.34 | 0.69 | 2323279 | 102584 | 2286458 | 100409 | 2288542 | 102412 | 2284373 | 99361 |
| RP_POS_315_07913a0_62 | RP | POS | 0.62 | 315.0791 | Altenuene [M+Na]+ | 315.0771(100) | 2 | 5.19E-08 | -0.74 | 1.36 | 8.39E-01 | 0.04 | 1.10 | 3.54E-05 | -0.72 | 1.46 | 3.77E-05 | -0.76 | 1.43 | 38518270 | 6155492 | 26992060 | 16672075 | 26650092 | 17360393 | 27334027 | 16123489 |
| RP_POS_744_55316a12_832 | RP | POS | 12.83 | 744.5532 | PC 33:2\|PC 15:0_18:2 | 184.0735(100), 744.5581(3) | 2 | 2.61E-10 | -1.57 | 2.61 | 8.55E-01 | -0.02 | 0.84 | 2.27E-10 | -1.58 | 2.46 | 4.21E-10 | -1.55 | 2.26 | 16783209 | 5094784 | 8746595 | 3304584 | 8807646 | 3560758 | 8685545 | 3062158 |
| RP_POS_153_05492a4_29 | RP | POS | 4.29 | 153.0549 | Vanillin | 111.0443(100), 125.0599(90), 153.0543(60), 93.0339(35), 65.0394(20) | 2 | 7.84E-01 | 0.05 | 0.36 | 8.60E-01 | -0.04 | 0.35 | 8.74E-01 | 0.03 | 0.20 | 7.49E-01 | 0.07 | 0.37 | 1142092 | 402451 | 1165528 | 464208 | 1173771 | 485133 | 1157285 | 447083 |
| RP_POS_288_21689a5_995 | RP | POS | 6.00 | 288.2169 | CAR 08:0 (Octanoylcarnitine) | 85.0289(100), 288.2181(65), 229.1446(15), 60.087(10) | 1 | 7.19E-01 | -0.06 | 0.55 | 8.61E-01 | -0.04 | 0.87 | 6.99E-01 | -0.07 | 0.33 | 8.48E-01 | -0.04 | 0.62 | 21320913 | 16124078 | 19849249 | 29217768 | 20365653 | 28634752 | 19332845 | 30071327 |
| RP_POS_357_27878a9_935 | RP | POS | 9.94 | 357.2788 | Deoxycholic acid | 357.2788(100) | 2 | 6.63E-02 | 0.28 | 1.07 | 8.64E-01 | -0.04 | 0.36 | 1.31E-01 | 0.26 | 0.64 | 1.38E-01 | 0.30 | 1.21 | 5847692 | 5418303 | 8392201 | 9792397 | 8561535 | 10876975 | 8222866 | 8681701 |
| RP_POS_647_45959a13_458 | RP | POS | 13.46 | 647.4596 | DG 37:7 | 647.4604(100) | 2 | 4.63E-01 | -0.16 | 0.72 | 8.67E-01 | 0.03 | 0.12 | 5.47E-01 | -0.14 | 0.51 | 4.63E-01 | -0.17 | 0.49 | 18032271 | 15822888 | 15704894 | 14362217 | 15462123 | 14656257 | 15947665 | 14206691 |
| RP_NEG_802_56183a13_163 | RP | NEG | 13.16 | 802.5618 | PC 34:2\|PC 16:1_18:1 [M+HCOO]- | 279.2347(100), 255.2332(40), 742.5426(20) | 2 | 1.97E-01 | -0.27 | 0.51 | 8.68E-01 | 0.03 | 0.12 | 2.67E-01 | -0.25 | 0.50 | 2.21E-01 | -0.29 | 0.54 | 154183146 | 15830139 | 150015761 | 15234768 | 149760145 | 15820413 | 150271377 | 14782226 |
| RP_POS_171_11302a2_883 | RP | POS | 2.88 | 171.1130 | fragment of Levetiracetam | 126.0915(100), 154.0866(10), 171.1126(2) | 2 | 1.62E-01 | 0.16 | 0.73 | 8.72E-01 | -0.04 | 0.21 | 3.23E-01 | 0.14 | 0.86 | 3.23E-01 | 0.18 | 0.45 | 16475 | 26850 | 4226907 | 29857357 | 4712029 | 33239450 | 3741785 | 26377551 |
| RP_NEG_838_59821a13_713 | RP | NEG | 13.71 | 838.5982 | PC O-38:5\|PC O-18:1_20:4 [M+HCOO]- | 303.2313(100), 778.5822(32), 492.3481(14), 474.341(6) | 2 | 3.75E-06 | -0.97 | 1.51 | 8.73E-01 | -0.03 | 0.45 | 9.50E-06 | -0.98 | 1.59 | 2.86E-05 | -0.95 | 1.46 | 8330119 | 1790423 | 6455357 | 1758573 | 6483591 | 1859715 | 6427123 | 1669762 |
| HILIC_POS_288_21689a0_864 | HILIC | POS | 0.86 | 288.2169 | AC 08:0 (Octanoylcarnitine) | 85.0289(100), 288.217(40), 229.1458(10), 60.0815(8) | 2 | 5.26E-01 | -0.10 | 0.23 | 8.82E-01 | -0.03 | 0.53 | 5.52E-01 | -0.12 | 0.21 | 6.51E-01 | -0.09 | 0.42 | 30269778 | 24328954 | 26588313 | 38714828 | 27166255 | 37577880 | 26010372 | 40193103 |
| RP_NEG_301_21786a10_704 | RP | NEG | 10.70 | 301.2179 | FA 20:5 (Eicosapentaenoic acid) | 301.2179(100), 257.228(35), 59.0125(25) | 2 | 2.55E-07 | 0.70 | 1.55 | 8.85E-01 | -0.03 | 0.46 | 1.17E-04 | 0.68 | 1.42 | 8.10E-05 | 0.71 | 1.56 | 3033004 | 1632654 | 5947949 | 4508959 | 6013395 | 4582137 | 5882502 | 4480136 |
| RP_POS_128_10689a5_058 | RP | POS | 5.06 | 128.1069 | N-Cyclohexylformahide | 83.0857(100), 128.1064(90), 55.055(45) | 2 | 1.22E-02 | -0.38 | 1.11 | 8.86E-01 | 0.03 | 1.08 | 5.09E-02 | -0.37 | 1.08 | 3.65E-02 | -0.40 | 1.16 | 1131158 | 263199 | 957633 | 495400 | 950475 | 502092 | 964791 | 493608 |
| RP_NEG_271_22827a9_674 | RP | NEG | 9.67 | 271.2283 | FA 16:0;O | 271.2284(100) | 2 | 3.04E-02 | 0.40 | 0.91 | 8.89E-01 | -0.03 | 0.65 | 7.75E-02 | 0.39 | 0.78 | 4.24E-02 | 0.42 | 1.01 | 4892522 | 679177 | 5215608 | 828369 | 5227275 | 769506 | 5203940 | 891053 |
| RP_POS_312_21686a6_182 | RP | POS | 6.18 | 312.2169 | CAR 10:2 | 85.0287(100), 312.2154(60) | 2 | 3.99E-01 | -0.14 | 0.63 | 8.89E-01 | 0.03 | 0.66 | 5.72E-01 | -0.12 | 0.46 | 3.89E-01 | -0.15 | 0.76 | 966026 | 603165 | 842337 | 994441 | 828383 | 834103 | 856292 | 1141001 |
| RP_POS_834_60022a13_433 | RP | POS | 13.43 | 834.6002 | PC 40:6 | 184.0732(100), 834.5837(2) | 2 | 5.46E-01 | 0.13 | 0.71 | 8.94E-01 | 0.03 | 0.08 | 5.53E-01 | 0.15 | 0.42 | 6.08E-01 | 0.12 | 0.56 | 3311247 | 2245781 | 3579447 | 1918492 | 3553717 | 1785956 | 3605176 | 2060424 |
| RP_NEG_436_28381a10_706 | RP | NEG | 10.71 | 436.2838 | LPE O-16:1 | 436.2795(100), 78.9574(40), 40.0107(35), 375.2247(7) | 2 | 2.26E-01 | 0.21 | 0.74 | 9.00E-01 | 0.03 | 1.05 | 2.77E-01 | 0.23 | 1.02 | 3.28E-01 | 0.20 | 0.68 | 1888692 | 568081 | 2043162 | 770161 | 2033457 | 760557 | 2052866 | 787250 |
| RP_POS_265_11829a3_744 | RP | POS | 3.74 | 265.1183 | Phenylacetylglutamine | 130.0502(100), 84.0447(15), 91.0546(14), 136.0754(14), 147.0768(13) | 2 | 6.04E-02 | -0.39 | 0.68 | 9.16E-01 | -0.02 | 0.26 | 1.02E-01 | -0.40 | 0.63 | 7.55E-02 | -0.38 | 0.79 | 15156074 | 10009048 | 11272449 | 9795410 | 11376815 | 7812471 | 11168083 | 11523946 |
| HILIC_POS_329_15283a0_665 | HILIC | POS | 0.67 | 329.1528 | Tiapride | 256.0634(100), 329.1509(20) | 2 | 4.92E-01 | 0.09 | 0.33 | 9.17E-01 | -0.02 | 0.81 | 4.60E-01 | 0.08 | 0.35 | 6.40E-01 | 0.11 | 0.41 | 32998445 | 12021384 | 35606228 | 31273429 | 35936748 | 41612805 | 35275709 | 15625812 |
| RP_POS_171_11292a3_143 | RP | POS | 3.14 | 171.1129 | fragment of Levetiracetam | 126.0916(100), 154.0867(15), 171.1128(2) | 2 | 1.60E-01 | 0.16 | 0.74 | 9.23E-01 | -0.02 | 0.19 | 3.22E-01 | 0.15 | 0.86 | 3.25E-01 | 0.17 | 0.45 | 112525 | 127697 | 5310251 | 36748375 | 5666173 | 39465870 | 4954328 | 34214512 |
| HILIC_NEG_135_03123a1_359 | HILIC | NEG | 1.36 | 135.0312 | Hypoxanthine | 134.8954(100), 92.0248(40), | 1 | 6.84E-12 | 0.84 | 1.41 | 9.29E-01 | 0.02 | 0.63 | 6.71E-06 | 0.85 | 1.39 | 1.67E-07 | 0.83 | 1.93 | 399889 | 202553 | 4344143 | 5059150 | 4298956 | 4520376 | 4389331 | 5592350 |
| RP_POS_856_58093a13_71 | RP | POS | 13.71 | 856.5809 | PC 40:6 [M+Na]+ | 146.9816(100), 86.0967(75), 673.5201(65), 797.5168(35), 856.577(30), 651.5269(7) | 2 | 2.08E-03 | -0.63 | 1.23 | 9.34E-01 | -0.02 | 0.68 | 5.46E-03 | -0.64 | 1.10 | 4.90E-03 | -0.62 | 1.26 | 17370979 | 5516717 | 13724735 | 5632727 | 13772096 | 5359139 | 13677375 | 5947922 |
| RP_NEG_391_28635a9_932 | RP | NEG | 9.93 | 391.2864 | ST 24:1;O4 | 391.2819(100), 345.2812(4) | 2 | 7.31E-02 | 0.28 | 1.12 | 9.39E-01 | -0.02 | 0.32 | 1.24E-01 | 0.28 | 0.68 | 1.56E-01 | 0.29 | 1.23 | 2783687 | 2643468 | 3959113 | 4494025 | 3993984 | 4976631 | 3924242 | 4004416 |
| HILIC_POS_181_07201a0_589 | HILIC | POS | 0.59 | 181.0720 | Theobromine | 181.0721(100) | 1 | 4.06E-01 | -0.16 | 0.41 | 9.42E-01 | -0.02 | 0.78 | 4.80E-01 | -0.17 | 0.25 | 4.46E-01 | -0.15 | 0.40 | 1106868 | 1011130 | 928487 | 1161614 | 936978 | 924536 | 919996 | 1367961 |
| RP_POS_400_3421a9_256 | RP | POS | 9.26 | 400.3421 | CAR 16:0 (Palmitoylcarnitine) | 85.0289(100), 400.3445(60), 341.2653(3) | 1 | 5.15E-11 | 1.10 | 1.74 | 9.42E-01 | 0.01 | 0.52 | 7.54E-09 | 1.11 | 1.85 | 2.41E-08 | 1.10 | 1.79 | 8173107 | 2510079 | 12619895 | 3837374 | 12591921 | 3952736 | 12647869 | 3758414 |
| RP_POS_279_15955a9_441 | RP | POS | 9.44 | 279.1596 | Di-n-butyl phthalate | 149.0228(100), 57.0705(30) | 2 | 2.42E-01 | 0.22 | 0.37 | 9.46E-01 | -0.01 | 0.79 | 3.38E-01 | 0.22 | 0.56 | 2.70E-01 | 0.23 | 0.44 | 13701354 | 827864 | 13907389 | 950514 | 13913898 | 867211 | 13900881 | 1035979 |
| RP_NEG_836_58185a13_511 | RP | NEG | 13.51 | 836.5819 | PC O-38:6\|PC O-18:2_20:4 [M+HCOO]- | 303.2352(100), 776.5573(40), 490.333(12), 224.0689(6), 472.514(4) | 2 | 9.67E-02 | 0.30 | 1.04 | 9.52E-01 | 0.01 | 0.32 | 1.47E-01 | 0.30 | 0.77 | 1.58E-01 | 0.29 | 1.19 | 3286584 | 846390 | 3599760 | 1104045 | 3593026 | 1092166 | 3606494 | 1126848 |
| HILIC_POS_177_10228a0_539 | HILIC | POS | 0.54 | 177.1023 | Cotinine | 130.0504(100), 72.0816(10) | 2 | 6.12E-06 | 0.77 | 1.47 | 9.52E-01 | 0.01 | 0.77 | 2.17E-04 | 0.78 | 1.24 | 4.24E-05 | 0.77 | 1.78 | 15551735 | 42281610 | 62686699 | 61845171 | 62312468 | 54823660 | 63060931 | 68715271 |
| HILIC_POS_522_35626a1_248 | HILIC | POS | 1.25 | 522.3563 | LPC 18:1 | 184.0744(100), 104.1074(70), 522.3516(25) | 2 | 4.36E-05 | 0.72 | 1.34 | 9.56E-01 | 0.01 | 0.74 | 8.20E-04 | 0.73 | 1.23 | 1.48E-04 | 0.72 | 1.47 | 128351678 | 57139722 | 183922757 | 77856149 | 183489140 | 66200812 | 184356375 | 88678827 |
| HILIC_POS_286_20114a0_973 | HILIC | POS | 0.97 | 286.2011 | AC 08:1 (Octenoylcarnitine) | 85.0288(100), 286.1991(40), 227.1266(10) | 2 | 9.37E-01 | -0.01 | 0.42 | 9.60E-01 | -0.01 | 0.32 | 9.29E-01 | -0.02 | 0.19 | 9.63E-01 | -0.01 | 0.31 | 17516222 | 12770829 | 17296540 | 15794118 | 17375515 | 13935305 | 17217565 | 17600959 |
| RP_POS_160_13316a0_62 | RP | POS | 0.62 | 160.1332 | NAE 6:0 | 160.1331(100), 101.0597(30), 132.0647(25), 60.0811(20) | 2 | 4.97E-02 | 0.33 | 0.60 | 9.69E-01 | -0.01 | 0.41 | 1.10E-01 | 0.32 | 0.57 | 8.93E-02 | 0.33 | 0.66 | 7676440 | 2388600 | 8781542 | 3595630 | 8795479 | 3500403 | 8767606 | 3723943 |
| RP_POS_209_09201a1_959 | RP | POS | 1.96 | 209.0920 | Kynurenine | 94.066(100), 192.0655(70), 146.0603(50), 136.0753(40), 150.0549(22), 174.0555(21), 209.1368(17), 120.044(16), 74.0241(15) | 1 | 9.00E-03 | -0.45 | 1.32 | 9.69E-01 | -0.01 | 1.05 | 4.05E-02 | -0.45 | 1.41 | 1.47E-02 | -0.44 | 1.09 | 4261031 | 927291 | 3693314 | 1339991 | 3698496 | 1090184 | 3688132 | 1561807 |
| RP_POS_209_0919a1_27 | RP | POS | 1.27 | 209.0919 | Kynurenine | 94.0658(100), 192.065(80), 146.0603(50), 136.0754(45), 174.0556(20), 120.044(20) | 2 | 1.17E-02 | -0.42 | 1.31 | 9.69E-01 | 0.01 | 0.98 | 5.69E-02 | -0.42 | 1.37 | 1.49E-02 | -0.43 | 1.07 | 6543839 | 1394390 | 5711957 | 2073637 | 5703938 | 1624119 | 5719976 | 2459634 |
| RP_POS_260_18573a4_181 | RP | POS | 4.18 | 260.1857 | CAR 06:0 | 85.0289(100), 201.111(15), 260.1852(65) | 1 | 1.46E-01 | 0.19 | 0.54 | 9.71E-01 | -0.01 | 0.89 | 2.97E-01 | 0.19 | 0.46 | 2.44E-01 | 0.20 | 0.60 | 4479197 | 3117743 | 5991977 | 8766753 | 6024268 | 8434849 | 5959685 | 9172328 |
| RP_POS_639_49524a14_158 | RP | POS | 14.16 | 639.4952 | DG 36:4 [M+Na]+ | 639.4892(100) | 2 | 4.71E-05 | -0.78 | 1.41 | 9.74E-01 | 0.01 | 0.13 | 1.18E-03 | -0.78 | 1.20 | 3.01E-05 | -0.79 | 1.56 | 4358980 | 2523025 | 2020048 | 2916362 | 2010306 | 1784217 | 2029790 | 3741696 |
| RP_NEG_878_59351a13_715 | RP | NEG | 13.72 | 878.5935 | PC 40:6\|PC 18:0_22:6 [M+HCOO]- | 283.2655(100), 327.2355(82), 818.5672(42) | 2 | 2.83E-03 | -0.64 | 1.26 | 9.78E-01 | -0.01 | 0.61 | 6.93E-03 | -0.64 | 1.11 | 5.32E-03 | -0.63 | 1.28 | 12537726 | 4211780 | 9888858 | 3962111 | 9900055 | 3726800 | 9877662 | 4222289 |
| RP_POS_647_51154a11_755 | RP | POS | 11.76 | 647.5115 | SM 30:1;2O | 184.0732(100), 647.438(3) | 2 | 1.05E-03 | -0.68 | 1.28 | 9.83E-01 | 0.00 | 0.08 | 2.10E-03 | -0.68 | 1.23 | 3.42E-03 | -0.68 | 1.11 | 3008272 | 1095287 | 2238521 | 1078025 | 2236187 | 1169611 | 2240855 | 989944 |
| HILIC_NEG_178_05095a0_972 | HILIC | NEG | 0.97 | 178.0510 | Hippuric acid | 134.0613(100), 178.0513(35) | 2 | 2.74E-02 | -0.48 | 1.20 | 9.84E-01 | 0.00 | 0.35 | 4.79E-02 | -0.48 | 0.86 | 3.74E-02 | -0.48 | 1.28 | 3228058 | 3024747 | 1864608 | 2702452 | 1869985 | 2449670 | 1859230 | 2958817 |
| RP_POS_183_08109a8_099 | RP | POS | 8.10 | 183.0811 | Benzophenone | 105.0334(100), 183.0797(30) | 2 | 3.66E-01 | 0.16 | 0.52 | 9.90E-01 | 0.00 | 0.43 | 4.29E-01 | 0.16 | 0.39 | 4.53E-01 | 0.16 | 0.44 | 953261 | 335737 | 1021796 | 462784 | 1021219 | 480373 | 1022373 | 449388 |
| RP_POS_372_31073a8_758 | RP | POS | 8.76 | 372.3107 | CAR 14:0 (Myristoylcarnitine) | 85.0289(100), 372.3084(55) | 1 | 8.20E-07 | 0.80 | 1.42 | 9.96E-01 | 0.00 | 1.23 | 1.79E-05 | 0.80 | 1.49 | 7.04E-05 | 0.80 | 1.39 | 2668655 | 1220323 | 4236890 | 2018220 | 4235953 | 2155767 | 4237827 | 1892674 |
| RP_POS_454_29248a10_516 | RP | POS | 10.52 | 454.2925 | LPE 16:0 | 313.2715(100), 454.2939(3), 62.0608(22) | 2 | 3.06E-01 | 0.17 | 1.01 | 1.00E+00 | 0.00 | 0.64 | 4.12E-01 | 0.17 | 0.79 | 3.67E-01 | 0.17 | 0.73 | 13923437 | 2981231 | 14631808 | 4424308 | 14631687 | 4072751 | 14631929 | 4791783 |
| HILIC_NEG_185_96243a0_927 | HILIC | NEG | 0.927 | 185.9624 | | | | 2.51E-02 | -0.46 | 0.90 | 7.75E-08 | -0.99 | 2.78 | 4.05E-05 | -0.96 | 1.73 | 8.91E-01 | 0.03 | 0.25 | 9259307 | 2722957 | 7988111 | 2679166 | 9338422 | 2196344 | 6637801 | 2440764 |
| RP_POS_815_54254a12_719 | RP | POS | 12.72 | 815.5425 | | | | 3.20E-01 | -0.19 | 0.90 | 2.23E-07 | -1.01 | 2.89 | 9.39E-04 | -0.69 | 1.39 | 1.33E-01 | 0.31 | 0.89 | 1262636 | 347916 | 1189013 | 401195 | 1384856 | 366288 | 993169 | 335794 |

**Supplementary table 1: Metabolomics results** (in asceding order according to p-value from progressive ALD vs non-progressive ALD comparison, with molecular features with ID_level of 1-2 first)

| | | | | | | | | all_ALD_vs_control | | | Prog_ALD_vs_non-prog_ALD | | | Prog_ALD_vs_control_ALD | | | Non-prog_ALD_vs_control | | | Healthy_control | | all_ALD | | Non-prog_ALD | | Progressive_ALD | |
|---|---|---|---|---|---|---|---|---|---|---|---|---|---|---|---|---|---|---|---|---|---|---|---|---|---|---|---|
| | | | | | | | | t-test | Cohen's | PLS-DA | t-test | Cohen's | PLS-DA | t-test | Cohen's | PLS-DA | t-test | Cohen's | PLS-DA | | | | | | | | |
| Feature_ID | RP_HILIC | POS_NEG | Average.Rt | Average.Mz | Metabolite.name | curated_MSMS | ID_level | p | d | VIP | p | d | VIP | p | d | VIP | p | d | VIP | Mean | SD | Mean | SD | Mean | SD | Mean | SD |
| RP_NEG_516_29498a9_311 | RP | NEG | 9.31 | 516.2950 | adduct of glycochenodeoxycholic acid | 448.3027(100), 516.295(30), 74.0234(30) | | 1.26E-05 | 0.57 | 1.01 | 6.56E-07 | 1.08 | 2.91 | 4.78E-07 | 1.11 | 1.58 | 6.60E-01 | 0.03 | 0.55 | 1023753 | 1184072 | 2948146 | 3683681 | 1136232 | 1026734 | 4760059 | 4434152 |
| RP_NEG_241_08723a7_928 | RP | NEG | 7.93 | 241.0872 | Unknown | 59.0125(100), 167.0854(70), 220.9839(45), 241.0872(40) | 4 | 1.16E-02 | 0.51 | 1.34 | 1.33E-06 | 0.92 | 2.77 | 6.98E-05 | 0.97 | 1.46 | 7.84E-01 | 0.05 | 1.01 | 2687704 | 7194284 | 6535019 | 7391871 | 3073661 | 4088760 | 9996378 | 8320194 |
| RP_POS_792_55078a13_116 | RP | POS | 13.12 | 792.5508 | | | | 3.86E-17 | 1.18 | 2.03 | 2.61E-06 | 0.88 | 2.71 | 8.34E-14 | 1.62 | 2.30 | 1.62E-10 | 0.74 | 2.06 | 3832488 | 454985 | 5165148 | 1090586 | 4665638 | 557138 | 5664658 | 1258341 |
| HILIC_NEG_167_00179a0_923 | HILIC | NEG | 0.923 | 167.0018 | | | | 1.02E-03 | -0.62 | 1.18 | 2.62E-06 | -0.90 | 2.41 | 4.84E-07 | -1.07 | 1.93 | 3.99E-01 | -0.17 | 0.41 | 313210 | 84045 | 251396 | 100168 | 296276 | 94141 | 206516 | 85434 |
| RP_POS_506_2402a9_303 | RP | POS | 9.30 | 506.2402 | | | | 2.19E-05 | 0.54 | 0.98 | 2.91E-06 | 1.03 | 2.77 | 2.13E-06 | 1.06 | 1.47 | 6.73E-01 | 0.03 | 0.53 | 293007 | 349076 | 911661 | 1261071 | 325235 | 313182 | 1498088 | 1553430 |
| HILIC_NEG_205_02945a0_927 | HILIC | NEG | 0.927 | 205.0295 | | | | 1.50E-03 | -0.64 | 1.20 | 3.43E-06 | -0.87 | 2.39 | 2.57E-06 | -1.07 | 1.80 | 3.17E-01 | -0.21 | 0.43 | 264104 | 63272 | 220607 | 66527 | 250092 | 58383 | 191121 | 61312 |
| HILIC_POS_130_04999a6_374 | HILIC | POS | 6.374 | 130.0500 | | | | 8.26E-11 | 0.82 | 1.37 | 3.58E-06 | 0.97 | 2.49 | 3.69E-09 | 1.31 | 1.80 | 2.13E-05 | 0.33 | 1.38 | 401113 | 164754 | 940433 | 701025 | 620370 | 274350 | 1260497 | 841767 |
| HILIC_NEG_109_01061a0_921 | HILIC | NEG | 0.921 | 109.0106 | | | | 3.24E-03 | -0.65 | 1.26 | 3.80E-06 | -0.82 | 2.38 | 1.13E-05 | -1.06 | 1.86 | 2.93E-01 | -0.24 | 0.62 | 298106 | 105924 | 233259 | 92928 | 274292 | 87716 | 192226 | 79481 |
| RP_POS_508_23563a9_303 | RP | POS | 9.30 | 508.2356 | | | | 1.22E-05 | 0.56 | 1.00 | 4.64E-06 | 1.01 | 2.72 | 2.02E-06 | 1.06 | 1.48 | 3.90E-01 | 0.06 | 0.58 | 89891 | 125455 | 315955 | 444483 | 113332 | 110013 | 518577 | 550702 |
| RP_POS_810_09253a13_873 | RP | POS | 13.87 | 810.0925 | | | | 8.85E-05 | 0.63 | 1.13 | 4.84E-06 | 0.92 | 2.65 | 1.31E-07 | 1.09 | 1.70 | 2.78E-01 | 0.18 | 0.66 | 2396410 | 752065 | 3141164 | 1229377 | 2601917 | 942420 | 3680411 | 1253788 |
| HILIC_NEG_127_00108a0_926 | HILIC | NEG | 0.926 | 127.0011 | | | | 5.11E-03 | -0.58 | 1.17 | 5.15E-06 | -0.85 | 2.37 | 1.42E-05 | -1.00 | 1.75 | 4.72E-01 | -0.15 | 0.37 | 494093 | 119592 | 423088 | 119614 | 475228 | 107881 | 370947 | 108261 |
| RP_POS_535_258a9_302 | RP | POS | 9.30 | 535.2580 | | | | 3.40E-05 | 0.53 | 0.96 | 5.56E-06 | 1.00 | 2.70 | 4.22E-06 | 1.03 | 1.44 | 6.77E-01 | 0.03 | 0.51 | 166959 | 215276 | 543132 | 788501 | 186280 | 185088 | 899984 | 980823 |
| RP_POS_503_23114a9_301 | RP | POS | 9.30 | 503.2311 | | | | 2.59E-05 | 0.53 | 0.98 | 6.36E-06 | 1.00 | 2.69 | 4.15E-06 | 1.03 | 1.44 | 5.90E-01 | 0.03 | 0.50 | 105160 | 130333 | 346583 | 503211 | 120489 | 115974 | 572676 | 626431 |
| RP_POS_148_06046a0_543 | RP | POS | 0.54 | 148.0605 | | | | 1.12E-10 | 0.80 | 1.25 | 7.33E-06 | 0.95 | 2.98 | 1.18E-08 | 1.28 | 1.73 | 1.78E-06 | 0.33 | 1.48 | 442788 | 274402 | 1503924 | 1418589 | 873715 | 480492 | 2134134 | 1739094 |
| RP_POS_743_54504a12_973 | RP | POS | 12.97 | 743.5450 | | | | 8.51E-14 | 0.92 | 1.90 | 7.35E-06 | 0.92 | 2.60 | 1.26E-10 | 1.38 | 2.11 | 2.42E-06 | 0.46 | 1.48 | 231434 | 157109 | 1395079 | 1329183 | 814825 | 759671 | 1975333 | 1518315 |
| RP_POS_748_54736a12_19 | RP | POS | 12.19 | 748.5474 | | | | 2.58E-14 | 0.94 | 1.69 | 9.68E-06 | 0.91 | 2.59 | 2.81E-10 | 1.40 | 1.98 | 7.78E-09 | 0.48 | 1.73 | 164728 | 86144 | 1101743 | 1047318 | 646412 | 486456 | 1557074 | 1247549 |
| RP_NEG_532_29065a8_82 | RP | NEG | 8.82 | 532.2907 | | | | 7.89E-05 | 0.49 | 0.94 | 1.01E-05 | 0.98 | 2.64 | 1.16E-05 | 0.99 | 1.38 | 9.53E-01 | 0.00 | 0.29 | 261277 | 381513 | 957120 | 1565533 | 265863 | 271446 | 1648378 | 1975588 |
| HILIC_NEG_132_92355a0_926 | HILIC | NEG | 0.926 | 132.9236 | | | | 4.05E-05 | -0.83 | 1.48 | 1.02E-05 | -0.80 | 2.25 | 5.19E-08 | -1.23 | 2.04 | 3.98E-02 | -0.42 | 0.82 | 1452156 | 334333 | 1144807 | 353426 | 1294417 | 328563 | 995197 | 314221 |
| RP_NEG_146_04506a0_542 | RP | NEG | 0.54 | 146.0451 | | | | 1.37E-10 | 0.81 | 1.25 | 1.05E-05 | 0.93 | 2.95 | 1.26E-08 | 1.28 | 1.73 | 3.92E-06 | 0.34 | 1.47 | 448241 | 310244 | 1464168 | 1341837 | 878493 | 475526 | 2049843 | 1646708 |
| RP_POS_519_22754a8_815 | RP | POS | 8.82 | 519.2275 | | | | 4.63E-04 | 0.43 | 0.88 | 1.87E-05 | 0.96 | 2.58 | 4.79E-05 | 0.92 | 1.30 | 3.69E-01 | -0.05 | 0.55 | 183958 | 241713 | 594741 | 1058797 | 140628 | 150552 | 1048855 | 1349614 |
| RP_POS_522_23578a8_815 | RP | POS | 8.82 | 522.2358 | | | | 3.44E-04 | 0.44 | 0.89 | 2.06E-05 | 0.96 | 2.57 | 4.55E-05 | 0.92 | 1.30 | 4.75E-01 | -0.04 | 0.36 | 138029 | 163785 | 436771 | 757500 | 113727 | 122651 | 759814 | 965043 |
| RP_POS_393_73969a12_975 | RP | POS | 12.98 | 393.7397 | Unknown | 86.0969(95), 309.1511(90), 327.1637(75), 393.2073(70), 163.0143(20) | 4 | 2.47E-24 | 1.37 | 2.21 | 2.28E-05 | 0.75 | 2.46 | 4.73E-17 | 1.75 | 2.48 | 1.44E-14 | 1.00 | 2.25 | 1267184 | 719372 | 4244481 | 1976616 | 3431543 | 1346413 | 5057419 | 2175366 |
| HILIC_NEG_355_15808a0_927 | HILIC | NEG | 0.927 | 355.1581 | | | | 1.47E-07 | -1.11 | 1.85 | 2.38E-05 | -0.71 | 2.18 | 2.48E-10 | -1.46 | 2.39 | 3.83E-04 | -0.75 | 1.37 | 313057 | 100781 | 189232 | 97980 | 229140 | 95683 | 149325 | 83611 |
| HILIC_NEG_84_00858a0_926 | HILIC | NEG | 0.926 | 84.0086 | | | | 7.09E-05 | -0.81 | 1.46 | 2.46E-05 | -0.77 | 2.56 | 1.34E-07 | -1.20 | 2.10 | 4.36E-02 | -0.43 | 0.87 | 351236 | 72327 | 287548 | 74254 | 317737 | 71216 | 257359 | 64871 |
| RP_POS_472_30325a9_303 | RP | POS | 9.30 | 472.3033 | Glycodeoxycholic acid [M+Na]+ | 472.3047(100), 98.0215(1) | | 7.20E-05 | 0.49 | 0.94 | 2.47E-05 | 0.94 | 2.56 | 2.06E-05 | 0.96 | 1.35 | 7.39E-01 | 0.02 | 0.55 | 3039859 | 3923409 | 11472633 | 19295939 | 3325857 | 3513354 | 19619408 | 24586024 |
| RP_POS_405_74146a12_647 | RP | POS | 12.65 | 405.7415 | Unknown | 335.1653(95), 86.0969(60), 212.1168(65), 406.2035(35) | | 4.05E-04 | 0.65 | 1.16 | 2.76E-05 | 0.82 | 2.43 | 2.68E-06 | 1.06 | 1.64 | 1.70E-01 | 0.24 | 1.04 | 1588618 | 644500 | 2108058 | 799301 | 1783163 | 578446 | 2432953 | 860687 |
| RP_NEG_613_36023a8_937 | RP | NEG | 8.94 | 613.3602 | Unknown | 613.3566(100), 75.0076(7) | | 5.14E-09 | 0.72 | 1.26 | 2.76E-05 | 0.91 | 2.45 | 2.06E-07 | 1.17 | 1.69 | 1.00E-05 | 0.26 | 1.53 | 684912 | 303750 | 1839248 | 1746302 | 1109717 | 511122 | 2568780 | 2194077 |
| HILIC_NEG_255_08762a0_613 | HILIC | NEG | 0.613 | 255.0876 | | | | 3.93E-05 | -1.13 | 2.19 | 3.21E-05 | -0.55 | 2.20 | 1.21E-06 | -1.41 | 2.27 | 1.59E-03 | -0.86 | 1.64 | 256694 | 191502 | 91704 | 99880 | 131940 | 105780 | 51469 | 75247 |
| HILIC_NEG_383_19a0_927 | HILIC | NEG | 0.927 | 383.1900 | | | | 8.33E-08 | -1.10 | 1.82 | 3.47E-05 | -0.71 | 2.14 | 1.70E-10 | -1.45 | 2.33 | 2.91E-04 | -0.74 | 1.38 | 204496 | 62766 | 124853 | 64888 | 150777 | 60573 | 98929 | 58875 |
| HILIC_NEG_94_99465a0_926 | HILIC | NEG | 0.926 | 94.9947 | | | | 1.38E-02 | -0.52 | 1.05 | 3.56E-05 | -0.77 | 2.18 | 9.54E-05 | -0.90 | 1.58 | 5.58E-01 | -0.13 | 0.26 | 849094 | 217120 | 737505 | 209539 | 821116 | 198591 | 653893 | 187093 |
| HILIC_NEG_61_98754a0_925 | HILIC | NEG | 0.925 | 61.9875 | | | | 1.63E-03 | -0.65 | 1.22 | 3.61E-05 | -0.77 | 2.14 | 7.63E-06 | -1.03 | 1.77 | 2.14E-01 | -0.26 | 0.57 | 3812079 | 1338937 | 2907530 | 1344058 | 3443372 | 1228029 | 2371669 | 1247326 |
| RP_POS_787_60437a13_874 | RP | POS | 13.87 | 787.6044 | | | | 1.17E-06 | 0.72 | 1.26 | 3.75E-05 | 0.85 | 2.41 | 1.88E-07 | 1.15 | 1.68 | 1.92E-02 | 0.29 | 1.07 | 21837007 | 8202893 | 33231848 | 16708783 | 26468603 | 9039657 | 39995093 | 19723061 |
| HILIC_NEG_141_01675a0_926 | HILIC | NEG | 0.926 | 141.0168 | | | | 5.88E-04 | -0.73 | 1.32 | 3.91E-05 | -0.75 | 2.14 | 2.65E-06 | -1.10 | 1.87 | 1.04E-01 | -0.35 | 0.74 | 258484 | 67263 | 208930 | 64041 | 234362 | 60245 | 183499 | 57766 |
| HILIC_NEG_159_00731a0_927 | HILIC | NEG | 0.927 | 159.0073 | | | | 3.46E-03 | -0.58 | 1.17 | 4.24E-05 | -0.78 | 2.19 | 1.31E-05 | -0.97 | 1.71 | 3.67E-01 | -0.19 | 0.48 | 639113 | 134674 | 554021 | 144871 | 611305 | 136067 | 496738 | 131120 |
| RP_POS_755_53815a12_975 | RP | POS | 12.98 | 755.5382 | | | | 2.88E-24 | 1.34 | 2.17 | 4.53E-05 | 0.73 | 2.39 | 3.11E-16 | 1.71 | 2.43 | 2.41E-14 | 0.98 | 2.21 | 5023013 | 2407678 | 15767393 | 7392969 | 12830614 | 5033708 | 18704173 | 8215098 |
| HILIC_POS_214_05875a6_377 | HILIC | POS | 6.377 | 214.0588 | | | | 6.51E-11 | 0.88 | 1.35 | 4.57E-05 | 0.83 | 2.18 | 1.36E-09 | 1.29 | 1.85 | 9.20E-05 | 0.46 | 1.26 | 367415 | 172177 | 780459 | 493218 | 583793 | 302456 | 977085 | 566653 |
| HILIC_NEG_145_05052a0_926 | HILIC | NEG | 0.926 | 145.0505 | | | | 1.36E-02 | -0.47 | 0.97 | 4.83E-05 | -0.79 | 2.17 | 5.06E-05 | -0.87 | 1.61 | 7.13E-01 | -0.08 | 0.41 | 196559 | 55365 | 166919 | 63217 | 191752 | 60877 | 142085 | 55764 |
| HILIC_NEG_158_95026a0_926 | HILIC | NEG | 0.926 | 158.9503 | | | | 2.16E-02 | -0.48 | 0.90 | 4.92E-05 | -0.76 | 2.21 | 2.49E-04 | -0.86 | 1.48 | 6.48E-01 | -0.10 | 0.36 | 2162393 | 1116689 | 1629263 | 1079519 | 2052934 | 950857 | 1205593 | 1041181 |
| RP_POS_467_34717a9_304 | RP | POS | 9.30 | 467.3472 | | | | 8.56E-05 | 0.48 | 0.92 | 4.94E-05 | 0.91 | 2.48 | 3.54E-05 | 0.94 | 1.31 | 5.77E-01 | 0.03 | 0.54 | 182339 | 268499 | 775993 | 1379751 | 214477 | 227719 | 1337510 | 1775092 |
| HILIC_NEG_223_01962a0_927 | HILIC | NEG | 0.927 | 223.0196 | | | | 5.20E-02 | -0.38 | 0.98 | 5.44E-05 | -0.79 | 2.14 | 3.81E-04 | -0.78 | 1.38 | 9.58E-01 | 0.01 | 0.21 | 647979 | 136157 | 591909 | 147839 | 649623 | 142516 | 534196 | 130576 |
| HILIC_NEG_117_01929a0_926 | HILIC | NEG | 0.926 | 117.0193 | | | | 2.48E-01 | -0.23 | 0.64 | 5.53E-05 | -0.80 | 2.24 | 4.93E-03 | -0.62 | 1.17 | 4.07E-01 | 0.17 | 0.38 | 247414 | 87927 | 226010 | 97091 | 263879 | 85936 | 188141 | 93456 |
| HILIC_POS_64_01659a0_936 | HILIC | POS | 0.936 | 64.0166 | | | | 4.39E-03 | -0.57 | 1.15 | 5.58E-05 | -0.77 | 2.13 | 2.21E-05 | -0.96 | 1.65 | 3.76E-01 | -0.19 | 0.39 | 3177636 | 698188 | 2750524 | 736751 | 3037672 | 686939 | 2463377 | 675666 |
| HILIC_POS_102_06667a0_938 | HILIC | POS | 0.938 | 102.0667 | | | | 1.50E-02 | -0.50 | 0.98 | 5.82E-05 | -0.76 | 2.18 | 1.51E-04 | -0.88 | 1.50 | 5.72E-01 | -0.12 | 0.29 | 3065697 | 1045798 | 2531749 | 1042261 | 2937144 | 921817 | 2126353 | 1004810 |
| RP_NEG_564_22815a8_82 | RP | NEG | 8.82 | 564.2282 | | | | 5.03E-04 | 0.43 | 0.98 | 6.02E-05 | 0.90 | 2.47 | 8.67E-05 | 0.88 | 1.29 | 7.82E-01 | -0.02 | 1.09 | 219164 | 247914 | 626997 | 1053873 | 203800 | 239292 | 1050193 | 1349519 |
| RP_NEG_844_53448a12_981 | RP | NEG | 12.98 | 844.5345 | Unknown | 112.9846(100) | 4 | 1.05E-25 | 1.42 | 2.28 | 6.23E-05 | 0.69 | 2.35 | 5.61E-18 | 1.77 | 2.43 | 2.24E-15 | 1.08 | 2.29 | 747812 | 410539 | 2467535 | 1073008 | 2049459 | 777563 | 2885612 | 1168278 |
| RP_NEG_255_08781a5_615 | RP | NEG | 5.62 | 255.0878 | Unknown | 211.0959(100), 255.0858(15) | 4 | 5.45E-06 | -1.16 | 2.14 | 6.27E-05 | -0.58 | 2.29 | 8.89E-08 | -1.45 | 2.26 | 5.49E-04 | -0.87 | 1.63 | 3717300 | 2357448 | 1389079 | 1506265 | 1973950 | 1552018 | 804454 | 1215603 |
| RP_POS_430_29501a8_815 | RP | POS | 8.82 | 430.2950 | | | | 2.08E-04 | 0.45 | 0.92 | 6.52E-05 | 0.90 | 2.45 | 7.14E-05 | 0.90 | 1.28 | 9.81E-01 | 0.00 | 0.27 | 235779 | 341379 | 1041980 | 2015119 | 234118 | 267148 | 1849842 | 2607872 |
| RP_POS_626_35291a8_932 | RP | POS | 8.93 | 626.3529 | | | | 5.44E-05 | 0.57 | 1.05 | 6.54E-05 | 0.86 | 2.69 | 8.10E-06 | 1.00 | 1.54 | 1.84E-01 | 0.14 | 0.69 | 208361 | 263063 | 530472 | 612873 | 287983 | 260459 | 772961 | 755679 |
| RP_POS_448_30566a8_816 | RP | POS | 8.82 | 448.3057 | | | | 1.91E-04 | 0.45 | 0.92 | 6.63E-05 | 0.90 | 2.44 | 6.84E-05 | 0.90 | 1.28 | 9.50E-01 | 0.00 | 0.32 | 453378 | 671473 | 2040002 | 3939699 | 462339 | 549753 | 3617664 | 5096799 |
| HILIC_POS_61_04051a0_938 | HILIC | POS | 0.938 | 61.0405 | | | | 1.85E-02 | -0.48 | 0.96 | 6.65E-05 | -0.76 | 2.17 | 1.85E-04 | -0.86 | 1.46 | 6.35E-01 | -0.10 | 0.25 | 48360374 | 16964561 | 39957372 | 17176130 | 46588760 | 15428018 | 33325983 | 16379573 |
| HILIC_POS_105_04271a0_936 | HILIC | POS | 0.936 | 105.0427 | | | | 5.27E-03 | -0.57 | 1.13 | 6.88E-05 | -0.75 | 2.11 | 3.10E-05 | -0.95 | 1.63 | 3.64E-01 | -0.20 | 0.36 | 943328 | 207092 | 820709 | 208396 | 901021 | 199961 | 740396 | 186001 |
| HILIC_NEG_241_01036a0_928 | HILIC | NEG | 0.928 | 241.0104 | | | | 1.34E-01 | -0.31 | 0.93 | 6.96E-05 | -0.76 | 2.11 | 2.48E-03 | -0.69 | 1.21 | 7.70E-01 | 0.07 | 0.16 | 1162314 | 236917 | 1089660 | 228286 | 1177590 | 219549 | 1001730 | 203253 |
| HILIC_NEG_255_02582a0_928 | HILIC | NEG | 0.928 | 255.0258 | | | | 2.95E-02 | -0.42 | 1.00 | 7.42E-05 | -0.78 | 2.08 | 1.77E-04 | -0.81 | 1.49 | 8.77E-01 | -0.03 | 0.23 | 447394 | 92356 | 404226 | 103693 | 444026 | 100222 | 364426 | 91907 |
| RP_POS_834_59906a13_327 | RP | POS | 13.33 | 834.5991 | | | | 1.54E-02 | 0.41 | 0.83 | 7.59E-05 | 0.82 | 2.42 | 2.37E-04 | 0.82 | 1.33 | 9.69E-01 | 0.01 | 0.31 | 4447135 | 1688604 | 5400352 | 2425313 | 4461753 | 1571902 | 6338951 | 2759631 |
| RP_POS_239_09129a5_617 | RP | POS | 5.62 | 239.0913 | Unknown | 193.0846(100), 179.0706(95), 239.0901(65) | 4 | 6.94E-06 | -1.15 | 2.11 | 7.91E-05 | -0.57 | 2.27 | 1.33E-07 | -1.44 | 2.22 | 5.81E-04 | -0.87 | 1.61 | 5286117 | 3341434 | 2036617 | 2096400 | 2839424 | 2133182 | 1233809 | 1736004 |
| RP_NEG_180_10265a7_559 | RP | NEG | 7.56 | 180.1027 | Unknown | 180.1009(100), 92.9186(55), 136.9082(25) | 4 | 4.13E-02 | -0.41 | 1.12 | 8.07E-05 | -0.76 | 2.35 | 6.36E-04 | -0.79 | 1.72 | 8.72E-01 | -0.03 | 0.42 | 842149 | 386189 | 677054 | 396573 | 828665 | 336401 | 525443 | 397083 |
| RP_POS_776_57916a12_716 | RP | POS | 12.72 | 776.5792 | | | | 7.71E-14 | 1.00 | 1.63 | 8.25E-05 | 0.79 | 2.31 | 6.20E-11 | 1.39 | 2.00 | 4.70E-07 | 0.61 | 1.62 | 981672 | 306115 | 1852670 | 887386 | 1510594 | 562867 | 2194746 | 1017522 |
| RP_POS_832_58417a12_925 | RP | POS | 12.93 | 832.5842 | | | | 1.51E-01 | 0.29 | 0.73 | 8.46E-05 | -0.76 | 2.26 | 6.84E-01 | -0.09 | 0.49 | 3.05E-03 | 0.67 | 1.23 | 2526547 | 917362 | 2799285 | 933725 | 3154964 | 875532 | 2443606 | 858338 |
| RP_POS_377_76468a12_972 | RP | POS | 12.97 | 377.7647 | | | | 1.35E-17 | 1.12 | 1.94 | 9.53E-05 | 0.76 | 2.27 | 7.59E-13 | 1.49 | 2.24 | 7.51E-09 | 0.74 | 1.82 | 121994 | 149629 | 685905 | 503328 | 494228 | 355322 | 877582 | 557329 |
| RP_POS_828_54968a12_737 | RP | POS | 12.74 | 828.5497 | | | | 2.01E-06 | -1.02 | 1.66 | 9.69E-05 | -0.67 | 2.25 | 5.55E-09 | -1.35 | 2.25 | 2.09E-03 | -0.68 | 1.21 | 18742550 | 5726388 | 12571720 | 5414190 | 14622435 | 5590100 | 10521005 | 4392346 |
| RP_NEG_528_26459a8_664 | RP | NEG | 8.66 | 528.2646 | | | | 9.06E-06 | 0.53 | 1.01 | 9.90E-05 | 0.88 | 2.31 | 2.41E-05 | 0.96 | 1.35 | 2.44E-06 | 0.09 | 1.68 | 327506 | 309342 | 4978169 | 9922496 | 1095360 | 973626 | 8860978 | 12930794 |
| RP_NEG_776_54681a12_974 | RP | NEG | 12.97 | 776.5468 | Unknown | 253.2186(100), 716.5328(20), 281.2506(18), 227.2003(7), 224.0687(3) | 4 | 1.63E-24 | 1.37 | 2.21 | 1.07E-04 | 0.69 | 2.27 | 7.17E-17 | 1.72 | 2.47 | 3.16E-14 | 1.03 | 2.20 | 8943970 | 4249507 | 26763794 | 11708275 | 22302798 | 8620947 | 31224791 | 12912577 |
| RP_NEG_874_56213a12_637 | RP | NEG | 12.64 | 874.5621 | | | | 5.91E-06 | -0.92 | 1.67 | 1.13E-04 | -0.70 | 2.24 | 1.06E-08 | -1.27 | 2.16 | 7.83E-03 | -0.57 | 1.12 | 2687971 | 682241 | 1985533 | 708348 | 2251524 | 736898 | 1719542 | 571267 |
| RP_POS_412_28439a8_816 | RP | POS | 8.82 | 412.2844 | | | | 3.03E-04 | 0.44 | 0.91 | 1.13E-04 | 0.87 | 2.38 | 1.22E-04 | 0.87 | 1.26 | 9.95E-01 | 0.00 | 0.38 | 99305 | 134181 | 420868 | 829435 | 99121 | 101273 | 742615 | 1080944 |
| RP_NEG_254_62338a10_423 | RP | NEG | 10.42 | 254.6234 | | | | 3.70E-05 | 0.61 | 1.06 | 1.33E-04 | 0.81 | 2.22 | 4.66E-06 | 1.02 | 1.49 | 9.27E-02 | 0.21 | 0.80 | 249755 | 221211 | 504285 | 443159 | 337199 | 234578 | 671370 | 533654 |
| RP_POS_718_53705a12_652 | RP | POS | 12.65 | 718.5371 | | | | 1.26E-04 | 0.72 | 1.21 | 1.38E-04 | 0.74 | 2.21 | 1.74E-06 | 1.09 | 1.67 | 5.60E-02 | 0.35 | 0.75 | 548603 | 478139 | 967574 | 579158 | 752191 | 434670 | 1182957 | 627760 |
| RP_POS_846_52197a12_648 | RP | POS | 12.65 | 846.5220 | | | | 9.49E-04 | 0.62 | 1.18 | 1.49E-04 | 0.75 | 2.20 | 2.01E-05 | 0.99 | 1.51 | 1.68E-01 | 0.24 | 0.79 | 1191758 | 466556 | 1538544 | 564457 | 1328789 | 376855 | 1748298 | 641807 |
| HILIC_NEG_191_01344a0_927 | HILIC | NEG | 0.927 | 191.0134 | | | | 1.01E-02 | -0.52 | 1.11 | 1.61E-04 | -0.72 | 2.01 | 8.57E-05 | -0.88 | 1.53 | 4.68E-01 | -0.16 | 0.30 | 572474 | 123346 | 504997 | 128295 | 552052 | 123712 | 457942 | 115896 |
| RP_POS_414_29956a9_303 | RP | POS | 9.30 | 414.2996 | fragment of glycochenodeoxycholic acid | 414.2977(100), 76.0397(50), 339.2651(35), 201.1645(30) | | 2.69E-04 | 0.44 | 0.90 | 1.63E-04 | 0.85 | 2.35 | 1.41E-04 | 0.87 | 1.23 | 7.40E-01 | 0.01 | 0.54 | 2485881 | 3215531 | 10568920 | 20719997 | 2717953 | 2837691 | 18419887 | 27084210 |
| RP_POS_279_08383a5_618 | RP | POS | 5.62 | 279.0838 | | | | 1.41E-05 | -1.13 | 2.07 | 1.82E-04 | -0.54 | 2.16 | 4.01E-07 | -1.40 | 2.14 | 7.60E-04 | -0.87 | 1.60 | 1026156 | 691983 | 383394 | 417597 | 535534 | 419581 | 231254 | 359233 |
| HILIC_NEG_115_92058a0_924 | HILIC | NEG | 0.924 | 115.9206 | | | | 4.48E-05 | -0.85 | 1.54 | 1.90E-04 | -0.68 | 1.96 | 2.31E-07 | -1.19 | 1.99 | 1.80E-02 | -0.51 | 0.92 | 57826180 | 11206515 | 47703324 | 11125467 | 51743341 | 10862038 | 43663308 | 9939041 |
| RP_NEG_548_23315a9_311 | RP | NEG | 9.31 | 548.2332 | adduct of glycochenodeoxycholic acid | 548.2355(100), 115.9192(40) | | 2.93E-04 | 0.44 | 0.90 | 1.94E-04 | 0.84 | 2.32 | 1.62E-04 | 0.86 | 1.21 | 6.84E-01 | 0.02 | 0.55 | 512585 | 661267 | 2109091 | 4108122 | 569937 | 547386 | 3648246 | 5381769 |
| RP_NEG_528_26501a11_393 | RP | NEG | 11.39 | 528.2650 | Glycine conjugated sterol | 448.3024(100), 528.269(75), 74.0234(25) | 3 | 2.19E-04 | 0.44 | 1.03 | 2.17E-04 | 0.84 | 2.21 | 1.66E-04 | 0.86 | 1.25 | 4.50E-01 | 0.02 | 0.75 | 50156 | 69295 | 332054 | 725718 | 62060 | 69064 | 602048 | 954224 |
| HILIC_POS_198_08466a6_377 | HILIC | POS | 6.377 | 198.0847 | | | | 2.10E-10 | 0.87 | 1.31 | 2.22E-04 | 0.76 | 2.01 | 3.25E-09 | 1.25 | 1.81 | 1.24E-04 | 0.49 | 1.25 | 1234923 | 489420 | 2302965 | 1279859 | 1839248 | 864099 | 2766523 | 1457496 |
| RP_POS_483_34271a8_815 | RP | POS | 8.82 | 483.3427 | | | | 1.26E-04 | 0.46 | 0.97 | 2.23E-04 | 0.83 | 2.31 | 1.12E-04 | 0.88 | 1.28 | 2.63E-01 | 0.05 | 0.80 | 99316 | 153654 | 510887 | 999704 | 140189 | 169477 | 881586 | 1307688 |
| RP_NEG_385_16937a6_699 | RP | NEG | 6.70 | 385.1694 | | | | 4.49E-07 | 0.62 | 1.10 | 2.28E-04 | 0.81 | 2.17 | 6.42E-06 | 1.02 | 1.49 | 3.66E-04 | 0.21 | 1.14 | 1444696 | 1270914 | 5805050 | 7823798 | 2920126 | 2306315 | 8689973 | 10068192 |
| RP_POS_809_58661a13_886 | RP | POS | 13.89 | 809.5866 | | | | 2.99E-07 | 0.75 | 1.37 | 2.31E-04 | 0.77 | 2.18 | 3.76E-07 | 1.13 | 1.70 | 2.84E-03 | 0.36 | 1.30 | 9589129 | 3210521 | 14441415 | 6856301 | 11944424 | 3596252 | 16938407 | 8325841 |
| HILIC_POS_171_18552a6_378 | HILIC | POS | 6.378 | 171.1855 | | | | 4.43E-03 | 0.62 | 0.93 | 2.31E-04 | 0.67 | 1.97 | 1.30E-04 | 0.96 | 1.45 | 1.81E-01 | 0.29 | 0.56 | 6074760 | 1578314 | 7007302 | 1399381 | 6503534 | 1052875 | 7511071 | 1526444 |
| RP_POS_435_1987a9_274 | RP | POS | 9.27 | 435.1987 | | | | 6.83E-02 | 0.35 | 0.68 | 2.35E-04 | 0.74 | 2.14 | 3.11E-03 | 0.72 | 1.10 | 9.13E-01 | -0.02 | 0.19 | 348381 | 254333 | 447476 | 287412 | 342927 | 151439 | 552026 | 348792 |
| HILIC_NEG_528_26385a2_819 | HILIC | NEG | 2.819 | 528.2639 | | | | 2.10E-04 | 0.46 | 1.06 | 2.36E-04 | 0.82 | 2.03 | 1.06E-04 | 0.88 | 1.19 | 4.15E-01 | 0.05 | 0.41 | 60552 | 146026 | 307985 | 594446 | 89185 | 166443 | 526786 | 767163 |
| RP_NEG_800_54694a12_651 | RP | NEG | 12.65 | 800.5469 | | | | 3.48E-03 | 0.54 | 1.11 | 2.44E-04 | 0.73 | 2.15 | 9.96E-05 | 0.91 | 1.40 | 3.18E-01 | 0.18 | 0.68 | 11624528 | 3751941 | 14074309 | 4598473 | 12416658 | 2985719 | 15731961 | 5310477 |
| HILIC_POS_466_31595a2_257 | HILIC | POS | 2.257 | 466.3160 | | | | 2.42E-03 | 0.41 | 0.93 | 2.47E-04 | 0.81 | 2.06 | 2.89E-04 | 0.82 | 1.12 | 9.45E-01 | 0.01 | 0.07 | 182307 | 409213 | 587528 | 1090851 | 188648 | 392400 | 986408 | 1387630 |
| RP_NEG_530_3028a10_376 | RP | NEG | 10.38 | 530.3028 | Unknown | 255.2334(100), 480.3058(20) | | 3.99E-01 | 0.17 | 1.04 | 2.51E-04 | -0.71 | 2.18 | 4.30E-01 | -0.18 | 0.71 | 1.53E-02 | 0.53 | 1.22 | 3064658 | 488288 | 3148915 | 485155 | 3322258 | 397595 | 2975572 | 506114 |

**Supplementary table 1: Metabolomics results** (in asceding order according to p-value from progressive ALD vs non-progressive ALD comparison, with molecular features with ID_level of 1-2 first)

| Feature_ID | RP_HILIC | POS_NEG | Average.Rt | Average.Mz | Metabolite.name | curated_MSMS | ID_level | all_ALD_vs_control | | | Prog_ALD_vs_non-prog_ALD | | | Prog_ALD_vs_control_ALD | | | Non-prog_ALD_vs_control | | | Healthy_control | | all_ALD | | Non-prog_ALD | | Progressive_ALD | |
|---|---|---|---|---|---|---|---|---|---|---|---|---|---|---|---|---|---|---|---|---|---|---|---|---|---|---|---|
| | | | | | | | | t-test p | Cohen's d | PLS-DA VIP | t-test p | Cohen's d | PLS-DA VIP | t-test p | Cohen's d | PLS-DA VIP | t-test p | Cohen's d | PLS-DA VIP | Mean | SD | Mean | SD | Mean | SD | Mean | SD |
| RP_POS_182_11761a7_562 | RP | POS | 7.56 | 182.1176 | Unknown | 90.9482(100), 104.964(45), 107.9506(40), 102.9475(35), 71.93(28), 72.9374(26) | | 4.73E-02 | -0.40 | 1.09 | 2.63E-04 | -0.71 | 2.18 | 1.30E-03 | -0.76 | 1.63 | 8.21E-01 | -0.05 | 0.42 | 1445426 | 654003 | 1173807 | 673508 | 1413744 | 549200 | 933871 | 705247 |
| HILIC_NEG_85_0038a0_926 | HILIC | NEG | 0.926 | 85.0038 | | | | 6.69E-02 | -0.39 | 0.83 | 2.66E-04 | -0.69 | 1.91 | 1.36E-03 | -0.74 | 1.28 | 8.53E-01 | -0.04 | 0.08 | 14414556 | 3461971 | 13116626 | 3248509 | 14271632 | 3295951 | 11961620 | 2781217 |
| HILIC_NEG_464_30185a2_143 | HILIC | NEG | 2.143 | 464.3019 | | | | 1.82E-03 | 0.42 | 0.93 | 2.73E-04 | 0.80 | 2.05 | 2.36E-04 | 0.82 | 1.14 | 8.45E-01 | 0.02 | 0.14 | 108827 | 228407 | 345527 | 623963 | 119353 | 249549 | 571702 | 787359 |
| HILIC_NEG_298_03687a0_725 | HILIC | NEG | 0.725 | 298.0369 | | | | 1.80E-10 | 0.78 | 1.45 | 2.80E-04 | 0.77 | 2.07 | 4.65E-08 | 1.17 | 1.69 | 1.41E-04 | 0.39 | 1.23 | 35479 | 57383 | 385613 | 486036 | 211647 | 295734 | 559580 | 572766 |
| RP_POS_562_29688a10_264 | RP | POS | 10.26 | 562.2969 | | | | 2.76E-05 | 0.68 | 1.45 | 2.88E-04 | 0.74 | 2.26 | 1.95E-06 | 1.05 | 1.84 | 4.02E-02 | 0.31 | 1.22 | 297990 | 187058 | 496985 | 304157 | 388553 | 197665 | 605417 | 351915 |
| RP_NEG_608_31805a10_376 | RP | NEG | 10.38 | 608.3181 | Unknown | 112.9847(100) | | 7.49E-01 | 0.06 | 0.96 | 3.06E-04 | -0.72 | 2.14 | 1.99E-01 | -0.29 | 0.76 | 4.65E-02 | 0.42 | 1.05 | 9504143 | 1152015 | 9580218 | 1211955 | 10007566 | 996033 | 9152870 | 1266040 |
| RP_POS_738_53973a13_289 | RP | POS | 13.29 | 738.5397 | | | | 1.17E-07 | 0.78 | 1.37 | 3.07E-04 | 0.74 | 2.09 | 1.45E-07 | 1.15 | 1.78 | 1.66E-03 | 0.41 | 1.08 | 141482 | 203069 | 454568 | 419477 | 305222 | 247911 | 603915 | 498531 |
| RP_POS_822_52136a12_982 | RP | POS | 12.98 | 822.5214 | | | | 2.21E-21 | 1.24 | 2.02 | 3.15E-04 | 0.68 | 2.13 | 5.21E-15 | 1.58 | 2.39 | 1.80E-10 | 0.90 | 1.88 | 683150 | 431863 | 2563282 | 1455401 | 2050835 | 1169285 | 3075729 | 1541578 |
| RP_NEG_199_80447a0_564 | RP | NEG | 0.56 | 199.8045 | | | | 4.18E-06 | -0.81 | 1.27 | 3.29E-04 | -0.70 | 2.32 | 4.82E-08 | -1.16 | 1.84 | 1.07E-02 | -0.46 | 0.94 | 53195314 | 7249007 | 45122695 | 9889787 | 48589585 | 8524547 | 41655804 | 10020713 |
| RP_NEG_627_37616a9_253 | RP | NEG | 9.25 | 627.3762 | Unknown | 627.3838(100) | | 5.45E-10 | 0.75 | 1.25 | 3.47E-04 | 0.78 | 2.11 | 6.91E-07 | 1.14 | 1.58 | 1.44E-12 | 0.36 | 2.09 | 407494 | 250478 | 3197672 | 4040634 | 1748149 | 1008643 | 4647196 | 5261301 |
| HILIC_NEG_172_99106a0_723 | HILIC | NEG | 0.723 | 172.9911 | | | | 3.55E-10 | 0.76 | 1.44 | 3.58E-04 | 0.76 | 2.06 | 5.78E-08 | 1.15 | 1.69 | 3.52E-04 | 0.38 | 1.15 | 14217 | 14587 | 109301 | 134925 | 61888 | 86367 | 156714 | 157269 |
| RP_POS_1002_66528a10_502 | RP | POS | 10.50 | 1002.6653 | | | | 5.50E-01 | -0.12 | 0.93 | 3.61E-04 | -0.71 | 2.10 | 3.44E-02 | -0.47 | 0.97 | 2.73E-01 | 0.24 | 0.80 | 2123731 | 415395 | 2072071 | 443630 | 2226477 | 403725 | 1917665 | 431256 |
| RP_POS_220_9955a3_001 | RP | POS | 3.00 | 220.9955 | | | | 6.61E-11 | 0.80 | 1.43 | 3.67E-04 | 0.76 | 2.09 | 3.01E-08 | 1.18 | 1.74 | 7.79E-05 | 0.42 | 1.19 | 161212 | 104970 | 800833 | 862231 | 498168 | 541997 | 1103497 | 1010617 |
| HILIC_NEG_300_03079a0_724 | HILIC | NEG | 0.724 | 300.0308 | | | | 3.40E-10 | 0.77 | 1.44 | 3.69E-04 | 0.76 | 2.04 | 7.93E-08 | 1.15 | 1.66 | 1.68E-04 | 0.39 | 1.21 | 810269 | 1252405 | 8228931 | 10487691 | 4545116 | 6348068 | 11912745 | 12419147 |
| RP_POS_778_53461a12_651 | RP | POS | 12.65 | 778.5346 | | | | 9.94E-04 | 0.57 | 1.14 | 3.82E-04 | 0.72 | 2.07 | 3.90E-05 | 0.93 | 1.45 | 1.98E-01 | 0.21 | 0.85 | 12954055 | 4683357 | 16574971 | 6545505 | 14286434 | 4256438 | 18863507 | 7599723 |
| RP_POS_222_01918a3_001 | RP | POS | 3.00 | 222.0192 | Unknown | 90.948(100), 222.1122(70), 171.9812(70), 105.9347(65) 55.935(65), 189.9921(60), 72.938(55), 149.9609(50) | | 1.08E-11 | 0.85 | 1.49 | 3.85E-04 | 0.75 | 2.08 | 1.22E-08 | 1.22 | 1.78 | 1.13E-05 | 0.48 | 1.34 | 402132 | 224170 | 1325081 | 1159257 | 919259 | 713248 | 1730903 | 1367608 |
| RP_NEG_480_30746a10_376 | RP | NEG | 10.38 | 480.3075 | | | | 2.63E-01 | 0.23 | 1.00 | 3.95E-04 | -0.69 | 2.12 | 5.97E-01 | -0.12 | 0.66 | 1.05E-02 | 0.57 | 1.16 | 1764882 | 392126 | 1855143 | 396324 | 1992236 | 361203 | 1718050 | 385392 |
| HILIC_POS_352_24622a0_936 | HILIC | POS | 0.936 | 352.2462 | | | | 9.80E-04 | -0.66 | 1.23 | 4.09E-04 | -0.67 | 1.88 | 7.23E-06 | -1.00 | 1.67 | 1.30E-01 | -0.33 | 0.64 | 608585 | 127853 | 516889 | 134904 | 563452 | 133922 | 470325 | 120040 |
| HILIC_NEG_183_9826a0_725 | HILIC | NEG | 0.725 | 183.9826 | | | | 1.03E-10 | 0.79 | 1.47 | 4.18E-04 | 0.75 | 2.03 | 4.24E-08 | 1.17 | 1.70 | 7.25E-05 | 0.42 | 1.28 | 18455 | 23648 | 136307 | 159765 | 80708 | 98594 | 191905 | 188525 |
| RP_POS_218_99495a3 | RP | POS | 3.00 | 218.9950 | Unknown | 219.1728(100), 190.9995(80), 129.0543(60), 111.0443(55), 101.0599(50) | | 1.44E-11 | 0.83 | 1.46 | 4.20E-04 | 0.75 | 2.07 | 1.67E-08 | 1.20 | 1.77 | 2.11E-05 | 0.46 | 1.29 | 296811 | 188519 | 1414904 | 1443415 | 913041 | 907290 | 1916768 | 1694763 |
| RP_NEG_241_92035a2_997 | RP | NEG | 3.00 | 241.9204 | Unknown | 157.862(95), 125.8727(50) | 4 | 1.94E-13 | 0.96 | 1.61 | 4.32E-04 | 0.72 | 2.05 | 4.66E-10 | 1.31 | 1.92 | 1.28E-06 | 0.60 | 1.50 | 862039 | 853625 | 3578876 | 2944397 | 2559721 | 2003509 | 4598031 | 3373733 |
| RP_POS_506_24136a9_451 | RP | POS | 9.45 | 506.2414 | | | | 3.91E-05 | 0.50 | 1.02 | 4.34E-04 | 0.79 | 2.08 | 7.74E-05 | 0.89 | 1.37 | 6.16E-02 | 0.11 | 0.87 | 134835 | 128793 | 468470 | 745833 | 205505 | 208739 | 731435 | 969119 |
| HILIC_NEG_216_98085a0_724 | HILIC | NEG | 0.724 | 216.9809 | | | | 3.73E-10 | 0.77 | 1.45 | 4.34E-04 | 0.75 | 2.03 | 9.74E-08 | 1.14 | 1.65 | 1.30E-04 | 0.39 | 1.24 | 377041 | 332270 | 2143037 | 2494638 | 1276469 | 1490697 | 3009605 | 2969636 |
| RP_NEG_448_30771a8_331 | RP | NEG | 8.33 | 448.3077 | | | | 1.22E-06 | 0.59 | 0.98 | 4.41E-04 | 0.78 | 2.06 | 1.03E-05 | 0.97 | 1.35 | 9.52E-03 | 0.20 | 1.00 | 799056 | 708084 | 3608792 | 5323080 | 1747080 | 2343609 | 5470503 | 6684513 |
| HILIC_NEG_98_93896a0_927 | HILIC | NEG | 0.927 | 98.9390 | | | | 9.23E-05 | -0.78 | 1.46 | 4.56E-04 | -0.66 | 1.88 | 6.44E-07 | -1.11 | 1.87 | 3.18E-02 | -0.45 | 0.87 | 2122456 | 397045 | 1776453 | 425420 | 1922112 | 413851 | 1630793 | 388697 |
| RP_POS_798_56085a12_715 | RP | POS | 12.72 | 798.5609 | | | | 5.67E-16 | 1.10 | 1.76 | 4.92E-04 | 0.68 | 2.05 | 6.31E-12 | 1.44 | 2.08 | 1.04E-08 | 0.76 | 1.82 | 270448 | 147515 | 711669 | 397443 | 575555 | 281504 | 847782 | 449546 |
| HILIC_NEG_115_00371a0_927 | HILIC | NEG | 0.927 | 115.0037 | | | | 9.18E-01 | -0.02 | 0.61 | 4.96E-04 | -0.70 | 2.01 | 9.21E-02 | -0.37 | 0.65 | 1.08E-01 | 0.33 | 0.64 | 247127 | 65172 | 245713 | 74718 | 271159 | 64904 | 220267 | 75800 |
| HILIC_NEG_100_93339a0_928 | HILIC | NEG | 0.928 | 100.9334 | | | | 3.53E-04 | -0.72 | 1.38 | 4.97E-04 | -0.66 | 1.88 | 3.21E-06 | -1.05 | 1.75 | 6.63E-02 | -0.39 | 0.73 | 45556039 | 8563219 | 38837189 | 9044061 | 41914842 | 8695947 | 35759536 | 8385069 |
| HILIC_NEG_383_15366a0_603 | HILIC | NEG | 0.603 | 383.1537 | | | | 5.95E-03 | 0.42 | 0.92 | 5.01E-04 | 0.75 | 1.86 | 4.33E-04 | 0.79 | 1.34 | 7.46E-01 | 0.04 | 0.18 | 77734 | 62543 | 123369 | 119009 | 82277 | 60618 | 164461 | 146610 |
| RP_POS_275_63443a10_375 | RP | POS | 10.38 | 275.6344 | | | | 3.60E-01 | 0.17 | 0.95 | 5.12E-04 | -0.71 | 2.02 | 3.81E-01 | -0.19 | 0.64 | 8.60E-03 | 0.52 | 1.12 | 485526 | 77870 | 501043 | 97062 | 534043 | 81241 | 468042 | 101054 |
| RP_NEG_411_20331a9_303 | RP | NEG | 9.30 | 411.2033 | | | | 1.22E-01 | 0.32 | 0.63 | 5.19E-04 | 0.68 | 2.02 | 8.67E-03 | 0.66 | 1.04 | 9.07E-01 | -0.02 | 0.19 | 778189 | 580071 | 963354 | 577588 | 765014 | 328405 | 1161694 | 697059 |
| RP_POS_342_24582a11_299 | RP | POS | 11.30 | 342.2458 | | | | 9.95E-01 | 0.00 | 0.67 | 5.19E-04 | -0.74 | 2.34 | 1.44E-02 | -0.37 | 1.31 | 1.21E-01 | 0.37 | 0.78 | 289324 | 292268 | 289727 | 407224 | 431116 | 525344 | 148338 | 135143 |
| RP_NEG_243_91692a2_995 | RP | NEG | 3.00 | 243.9169 | | | | 1.28E-13 | 0.96 | 1.61 | 5.22E-04 | 0.71 | 2.02 | 4.43E-10 | 1.31 | 1.93 | 1.11E-06 | 0.60 | 1.51 | 540405 | 516524 | 2245942 | 1843162 | 1616912 | 1272426 | 2874971 | 2106478 |
| RP_POS_367_31839a11_61 | RP | POS | 11.61 | 367.3184 | | | | 5.80E-02 | 0.32 | 0.72 | 5.42E-04 | 0.74 | 2.07 | 3.55E-03 | 0.69 | 0.97 | 7.25E-01 | -0.05 | 0.54 | 499671 | 217387 | 596632 | 326248 | 484272 | 144364 | 708992 | 410418 |
| RP_POS_385_75302a12_976 | RP | POS | 12.98 | 385.7530 | | | | 1.73E-18 | 1.15 | 1.87 | 5.70E-04 | 0.67 | 2.04 | 1.43E-12 | 1.49 | 2.20 | 2.75E-10 | 0.82 | 1.91 | 184832 | 244282 | 1104870 | 784611 | 838989 | 553713 | 1370752 | 890463 |
| HILIC_POS_495_37915a1_021 | HILIC | POS | 1.021 | 495.3792 | | | | 2.06E-03 | 0.42 | 0.87 | 5.80E-04 | 0.76 | 1.93 | 4.00E-04 | 0.80 | 1.10 | 7.01E-01 | 0.04 | 0.19 | 283824 | 746777 | 1040172 | 2009284 | 349975 | 775796 | 1730370 | 2565694 |
| RP_NEG_626_27393a10_376 | RP | NEG | 10.38 | 626.2739 | | | | 3.84E-01 | 0.17 | 0.92 | 5.97E-04 | -0.68 | 2.05 | 4.54E-01 | -0.17 | 0.65 | 1.82E-02 | 0.51 | 1.07 | 1354570 | 231936 | 1396465 | 245359 | 1478868 | 217765 | 1314062 | 245665 |
| HILIC_NEG_152_99689a0_927 | HILIC | NEG | 0.927 | 152.9969 | | | | 4.13E-01 | -0.16 | 0.78 | 6.05E-04 | -0.68 | 1.87 | 2.60E-02 | -0.50 | 0.87 | 4.27E-01 | 0.17 | 0.30 | 398173 | 82847 | 384190 | 85583 | 412897 | 79174 | 355483 | 82754 |
| RP_NEG_510_25403a10_414 | RP | NEG | 10.41 | 510.2540 | | | | 4.85E-05 | 0.59 | 1.04 | 6.08E-04 | 0.72 | 2.01 | 1.73E-06 | 0.95 | 1.64 | 1.44E-01 | 0.23 | 0.66 | 568601 | 415037 | 1056605 | 891879 | 757109 | 740228 | 1356101 | 936022 |
| HILIC_POS_371_24084a0_936 | HILIC | POS | 0.936 | 371.2408 | | | | 9.07E-05 | -0.82 | 1.49 | 6.14E-04 | -0.63 | 1.82 | 1.06E-06 | -1.13 | 1.83 | 1.91E-02 | -0.51 | 1.10 | 2776997 | 492128 | 2354785 | 480256 | 2515688 | 460360 | 2193882 | 448589 |
| HILIC_NEG_209_00429a0_927 | HILIC | NEG | 0.927 | 209.0043 | | | | 2.81E-01 | -0.22 | 0.86 | 6.20E-04 | -0.67 | 1.91 | 1.49E-02 | -0.56 | 0.96 | 6.08E-01 | 0.11 | 0.22 | 1213334 | 249895 | 1158007 | 250308 | 1241809 | 235283 | 1074206 | 238530 |
| HILIC_NEG_130_99248a0_927 | HILIC | NEG | 0.927 | 130.9925 | | | | 1.54E-01 | -0.30 | 0.92 | 6.40E-04 | -0.66 | 1.85 | 6.50E-03 | -0.63 | 1.09 | 8.97E-01 | 0.03 | 0.05 | 1611391 | 326988 | 1516337 | 312637 | 1620760 | 303412 | 1411914 | 288386 |
| RP_POS_414_30035a8_329 | RP | POS | 8.33 | 414.3004 | | | | 2.71E-06 | 0.56 | 0.95 | 6.44E-04 | 0.76 | 2.00 | 2.05E-05 | 0.94 | 1.30 | 1.24E-02 | 0.18 | 0.96 | 214377 | 183485 | 1018923 | 1590341 | 478097 | 685774 | 1559749 | 2010665 |
| RP_POS_389_26825a9_044 | RP | POS | 9.04 | 389.2683 | | | | 6.64E-06 | 0.56 | 0.99 | 6.44E-04 | 0.76 | 2.02 | 3.20E-05 | 0.93 | 1.37 | 1.35E-02 | 0.18 | 1.04 | 364733 | 158150 | 755104 | 779625 | 489251 | 286645 | 1020957 | 1000815 |
| HILIC_POS_315_12683a6_128 | HILIC | POS | 6.128 | 315.1268 | | | | 4.06E-02 | -0.34 | 1.01 | 6.57E-04 | -0.72 | 1.77 | 1.35E-03 | -0.70 | 1.18 | 9.09E-01 | 0.02 | 0.97 | 399302 | 68907 | 365739 | 105778 | 401176 | 76719 | 330302 | 118985 |
| RP_NEG_516_29504a9_46 | RP | NEG | 9.46 | 516.2950 | | | | 1.45E-04 | 0.46 | 0.88 | 6.64E-04 | 0.77 | 2.03 | 1.91E-04 | 0.84 | 1.23 | 1.60E-01 | 0.08 | 0.83 | 365871 | 332333 | 1144291 | 1891527 | 498523 | 515139 | 1790058 | 2472316 |
| RP_POS_779_53833a12_647 | RP | POS | 12.65 | 779.5383 | | | | 2.57E-03 | 0.56 | 1.09 | 6.72E-04 | 0.68 | 1.99 | 1.16E-04 | 0.90 | 1.39 | 2.14E-01 | 0.22 | 0.83 | 6783807 | 2687701 | 8596759 | 3282253 | 7496400 | 2187098 | 9697119 | 3809439 |
| HILIC_NEG_288_61975a4_126 | HILIC | NEG | 4.126 | 288.6198 | | | | 1.11E-03 | 0.42 | 0.97 | 6.75E-04 | 0.76 | 1.96 | 3.75E-04 | 0.80 | 1.09 | 5.98E-01 | 0.04 | 0.21 | 80120 | 180523 | 304315 | 591824 | 103084 | 206957 | 505546 | 763013 |
| RP_POS_586_3075a10_376 | RP | POS | 10.38 | 586.3075 | | | | 3.76E-01 | 0.18 | 0.87 | 6.86E-04 | -0.66 | 2.03 | 5.22E-01 | -0.15 | 0.58 | 2.09E-02 | 0.51 | 1.10 | 1089998 | 179409 | 1122441 | 176434 | 1181102 | 152869 | 1063779 | 180281 |
| HILIC_POS_371_24008a1_843 | HILIC | POS | 1.843 | 371.2401 | | | | 1.95E-05 | 0.66 | 1.19 | 6.98E-04 | 0.70 | 1.83 | 2.04E-06 | 1.01 | 1.54 | 4.40E-02 | 0.30 | 0.70 | 2150315 | 798824 | 3066540 | 1474275 | 2575011 | 1074901 | 3558069 | 1656156 |
| HILIC_NEG_305_02322a0_928 | HILIC | NEG | 0.928 | 305.0232 | | | | 3.20E-02 | -0.43 | 1.04 | 7.23E-04 | -0.66 | 1.83 | 8.68E-04 | -0.76 | 1.28 | 6.27E-01 | -0.10 | 0.36 | 445975 | 88080 | 406257 | 91197 | 436438 | 83524 | 376075 | 89266 |
| HILIC_NEG_201_994a0_726 | HILIC | NEG | 0.726 | 201.9940 | | | | 2.21E-11 | 0.83 | 1.50 | 7.25E-04 | 0.71 | 1.96 | 2.28E-08 | 1.19 | 1.74 | 2.07E-05 | 0.48 | 1.39 | 54840 | 42524 | 233481 | 229301 | 156975 | 147159 | 309988 | 269499 |
| RP_NEG_131_0704a4_436 | RP | NEG | 4.44 | 131.0704 | | | | 2.46E-08 | 0.68 | 1.27 | 7.32E-04 | 0.74 | 2.01 | 2.63E-06 | 1.05 | 1.49 | 8.17E-05 | 0.31 | 1.23 | 811071 | 430232 | 3046880 | 3634213 | 1823951 | 1605233 | 4269810 | 4588641 |
| RP_NEG_532_30115a10_378 | RP | NEG | 10.38 | 532.3012 | | | | 4.01E-01 | 0.17 | 0.93 | 7.33E-04 | -0.67 | 2.00 | 4.71E-01 | -0.16 | 0.66 | 2.19E-02 | 0.50 | 1.07 | 852969 | 141567 | 877491 | 145546 | 925628 | 127933 | 829353 | 147322 |
| RP_POS_211_14409a4_835 | RP | POS | 4.84 | 211.1441 | | | | 3.44E-07 | 0.63 | 1.13 | 7.59E-04 | 0.74 | 1.98 | 6.79E-06 | 1.00 | 1.51 | 1.71E-03 | 0.26 | 1.08 | 1367255 | 1164140 | 4757753 | 5929564 | 2768850 | 2671212 | 6746655 | 7471862 |
| RP_NEG_624_34003a8_857 | RP | NEG | 8.86 | 624.3400 | | | | 1.27E-02 | 0.41 | 0.72 | 7.75E-04 | 0.71 | 1.97 | 6.51E-04 | 0.76 | 1.13 | 7.31E-01 | 0.05 | 0.44 | 524964 | 896002 | 1066763 | 1420121 | 595205 | 898698 | 1538322 | 1677252 |
| RP_NEG_297_24396a10_659 | RP | NEG | 10.66 | 297.2440 | | | | 6.89E-11 | 0.89 | 1.74 | 7.79E-04 | 0.69 | 2.03 | 1.15E-08 | 1.23 | 1.88 | 8.95E-06 | 0.54 | 1.61 | 5770117 | 1265716 | 8678148 | 3414243 | 7544191 | 2116667 | 9812104 | 4055556 |
| RP_NEG_383_15396a7_368 | RP | NEG | 7.37 | 383.1540 | | | | 5.17E-02 | 0.29 | 0.95 | 7.80E-04 | 0.73 | 2.18 | 3.80E-03 | 0.66 | 1.48 | 5.55E-01 | -0.07 | 0.28 | 5602839 | 4490025 | 7897884 | 8537671 | 5038301 | 3713773 | 10757467 | 10806986 |
| HILIC_NEG_291_00748a0_929 | HILIC | NEG | 0.929 | 291.0075 | | | | 2.26E-01 | -0.25 | 0.90 | 8.08E-04 | -0.65 | 1.86 | 9.84E-03 | -0.58 | 1.04 | 7.69E-01 | 0.07 | 0.11 | 913875 | 181706 | 869111 | 173148 | 926005 | 182432 | 812217 | 143824 |
| RP_NEG_480_30966a10_504 | RP | NEG | 10.50 | 480.3097 | | | | 8.89E-01 | 0.03 | 0.89 | 8.08E-04 | -0.67 | 2.03 | 1.82E-01 | -0.31 | 0.79 | 8.40E-02 | 0.36 | 0.96 | 6934896 | 806044 | 6958292 | 859606 | 7240791 | 707175 | 6675793 | 911022 |
| HILIC_POS_185_03258a6_129 | HILIC | POS | 6.129 | 185.0326 | | | | 1.26E-02 | -0.41 | 1.10 | 8.32E-04 | -0.70 | 1.75 | 4.92E-04 | -0.76 | 1.31 | 7.41E-01 | -0.05 | 1.00 | 5139389 | 723681 | 4698313 | 1164971 | 5081673 | 831144 | 4314953 | 1323360 |
| RP_POS_250_01358a3_003 | RP | POS | 3.00 | 250.0136 | | | | 1.60E-12 | 0.88 | 1.53 | 8.40E-04 | 0.70 | 1.97 | 8.44E-09 | 1.23 | 1.81 | 2.17E-06 | 0.53 | 1.44 | 1559964 | 1048159 | 6278501 | 5669747 | 4408300 | 3619544 | 8148702 | 6686474 |
| RP_NEG_221_15494a9_398 | RP | NEG | 9.40 | 221.1549 | | | | 8.47E-02 | -0.42 | 0.86 | 8.44E-04 | -0.58 | 2.04 | 5.93E-03 | -0.71 | 1.34 | 6.13E-01 | -0.13 | 0.48 | 847604 | 73403 | 822907 | 52120 | 839960 | 53285 | 805853 | 45338 |
| RP_POS_1019_63129a10_502 | RP | POS | 10.50 | 1019.6313 | | | | 2.86E-01 | -0.20 | 0.87 | 8.72E-04 | -0.68 | 1.97 | 1.41E-02 | -0.54 | 1.07 | 4.79E-01 | 0.14 | 0.81 | 3051675 | 469185 | 2943643 | 567430 | 3128817 | 494148 | 2758468 | 579969 |
| HILIC_NEG_143_05215a0_724 | HILIC | NEG | 0.724 | 143.0522 | | | | 2.07E-10 | 0.78 | 1.45 | 9.04E-04 | 0.71 | 1.97 | 1.25E-07 | 1.13 | 1.63 | 5.02E-05 | 0.42 | 1.30 | 79301 | 55874 | 385121 | 424833 | 245616 | 258373 | 524625 | 508109 |
| RP_POS_284_67719a10_375 | RP | POS | 10.38 | 284.6772 | | | | 3.70E-01 | 0.17 | 1.04 | 9.12E-04 | -0.68 | 2.03 | 4.25E-01 | -0.17 | 0.70 | 1.34E-02 | 0.51 | 1.29 | 593571 | 107321 | 614337 | 129680 | 656510 | 112890 | 572164 | 132677 |
| RP_NEG_269_21277a10_123 | RP | NEG | 10.12 | 269.2128 | Unknown | 269.2101(100), 59.0125(85), 197.1898(30) | 4 | 1.76E-12 | 0.89 | 1.67 | 9.19E-04 | 0.69 | 1.98 | 9.55E-09 | 1.23 | 1.84 | 1.09E-06 | 0.54 | 1.65 | 1329804 | 188739 | 2053331 | 858818 | 1771815 | 531965 | 2334846 | 1022480 |
| RP_NEG_365_23138a10_656 | RP | NEG | 10.66 | 365.2314 | | | | 1.12E-09 | 0.83 | 1.70 | 9.38E-04 | 0.69 | 2.06 | 3.65E-08 | 1.18 | 1.85 | 1.05E-04 | 0.49 | 1.48 | 663049 | 152157 | 979653 | 398715 | 849372 | 260698 | 1109934 | 467535 |
| RP_POS_253_02148a3 | RP | POS | 3.00 | 253.0215 | | | | 1.25E-11 | 0.84 | 1.52 | 9.43E-04 | 0.70 | 1.95 | 1.58E-08 | 1.19 | 1.79 | 2.69E-05 | 0.49 | 1.27 | 234888 | 186259 | 1120010 | 1127375 | 752221 | 767146 | 1487799 | 1305111 |
| RP_POS_507_32281a10_374 | RP | POS | 10.37 | 507.3228 | | | | 2.18E-01 | 0.23 | 1.00 | 9.47E-04 | -0.67 | 1.96 | 5.98E-01 | -0.11 | 0.64 | 7.90E-03 | 0.57 | 1.18 | 393680 | 103990 | 421446 | 126411 | 462403 | 121512 | 380489 | 118732 |
| HILIC_NEG_117_05568a0_723 | HILIC | NEG | 0.723 | 117.0557 | | | | 1.70E-08 | 0.68 | 1.39 | 9.54E-04 | 0.72 | 2.01 | 1.61E-06 | 1.04 | 1.47 | 5.10E-04 | 0.32 | 1.09 | 10534389 | 8458682 | 67421089 | 91803222 | 37321873 | 50080241 | 97520304 | 112564318 |
| RP_NEG_160_8412a0_548 | RP | NEG | 0.55 | 160.8412 | | | | 4.13E-01 | -0.15 | 0.44 | 9.86E-04 | -0.68 | 2.13 | 2.11E-02 | -0.49 | 0.83 | 3.41E-01 | 0.19 | 0.61 | 6197568 | 1561827 | 5917296 | 1984653 | 6558322 | 1809212 | 5276269 | 1961179 |
| RP_NEG_546_28064a10_635 | RP | NEG | 10.64 | 546.2806 | | | | 1.32E-01 | 0.27 | 0.80 | 9.90E-04 | 0.68 | 2.18 | 5.62E-03 | 0.61 | 1.07 | 7.25E-01 | -0.07 | 0.66 | 971949 | 261512 | 1058899 | 334642 | 950656 | 272516 | 1167142 | 357883 |
| RP_POS_472_30341a8_33 | RP | POS | 8.33 | 472.3034 | | | | 2.69E-06 | 0.56 | 0.96 | 9.97E-04 | 0.73 | 1.94 | 2.68E-05 | 0.93 | 1.29 | 8.38E-03 | 0.20 | 1.03 | 118185 | 112431 | 601560 | 954603 | 288194 | 417802 | 914925 | 1210855 |
| HILIC_NEG_189_05757a0_723 | HILIC | NEG | 0.723 | 189.0576 | | | | 6.37E-11 | 0.80 | 1.47 | 9.98E-04 | 0.70 | 1.98 | 6.41E-08 | 1.15 | 1.67 | 2.89E-05 | 0.45 | 1.35 | 21875 | 18454 | 118511 | 129452 | 76383 | 81515 | 160639 | 153593 |
| HILIC_NEG_71_04948a0_723 | HILIC | NEG | 0.723 | 71.0495 | | | | 2.63E-08 | 0.67 | 1.39 | 1.02E-03 | 0.72 | 2.01 | 2.07E-06 | 1.03 | 1.45 | 6.87E-04 | 0.31 | 1.07 | 89489 | 76170 | 609432 | 852757 | 331267 | 464399 | 887597 | 1046734 |
| RP_NEG_164_83536a0_55 | RP | NEG | 0.55 | 164.8354 | | | | 9.64E-02 | -0.29 | 0.80 | 1.02E-03 | -0.68 | 2.15 | 2.18E-03 | -0.63 | 1.07 | 7.81E-01 | 0.05 | 0.55 | 2275394 | 506644 | 2083067 | 708305 | 2311260 | 654083 | 1854875 | 692486 |
| RP_POS_791_54138a12_971 | RP | POS | 12.97 | 791.5414 | | | | 3.27E-19 | 1.54 | 2.45 | 1.02E-03 | 0.52 | 1.92 | 2.36E-18 | 1.80 | 2.59 | 2.29E-14 | 1.28 | 2.38 | 2210821 | 1086136 | 5188988 | 1561266 | 4684876 | 1244102 | 5693100 | 1690688 |
| RP_POS_240_09941a10_375 | RP | POS | 10.38 | 240.0994 | | | | 5.11E-01 | 0.12 | 0.96 | 1.03E-03 | -0.68 | 2.00 | 2.91E-01 | -0.22 | 0.68 | 2.10E-02 | 0.46 | 1.16 | 1104302 | 146820 | 1125630 | 191373 | 1187254 | 167623 | 1064005 | 195312 |
| HILIC_POS_243_13365a1_72 | HILIC | POS | 1.72 | 243.1337 | | | | 1.40E-03 | 0.58 | 1.15 | 1.04E-03 | 0.66 | 1.84 | 1.03E-04 | 0.91 | 1.35 | 1.37E-01 | 0.25 | 0.61 | 159304 | 307571 | 382595 | 390406 | 255883 | 237404 | 509306 | 467779 |
| RP_POS_659_4754a10_377 | RP | POS | 10.38 | 659.4754 | | | | 5.93E-01 | 0.12 | 0.97 | 1.05E-03 | 0.62 | 1.96 | 8.70E-02 | 0.43 | 0.95 | 4.15E-01 | -0.19 | 0.84 | 987790 | 212496 | 1010208 | 179968 | 952303 | 150178 | 1068112 | 189847 |
| RP_POS_716_55865a13_271 | RP | POS | 13.27 | 716.5587 | | | | 1.15E-09 | 0.78 | 1.33 | 1.06E-03 | 0.70 | 1.91 | 2.02E-07 | 1.12 | 1.66 | 8.10E-05 | 0.43 | 1.30 | 279333 | 201596 | 844896 | 782911 | 590871 | 463025 | 1098921 | 944630 |
| RP_POS_414_0452a0_553 | RP | POS | 0.55 | 414.0452 | | | | 6.13E-03 | 0.37 | 0.70 | 1.07E-03 | 0.73 | 2.00 | 1.14E-03 | 0.73 | 1.11 | 9.70E-01 | 0.00 | 0.62 | 472573 | 85830 | 551494 | 239082 | 473328 | 94120 | 629660 | 306855 |
| RP_NEG_1035_66504a10_375 | RP | NEG | 10.38 | 1035.6650 | | | | 1.27E-01 | 0.30 | 1.03 | 1.08E-03 | -0.65 | 1.97 | 8.90E-01 | -0.03 | 0.66 | 4.45E-03 | 0.62 | 1.21 | 5930021 | 2029266 | 6584946 | 2246372 | 7305096 | 2118652 | 5864795 | 2155985 |
| RP_NEG_185_04305a2_988 | RP | NEG | 2.99 | 185.0431 | | | | 2.68E-13 | 0.92 | 1.56 | 1.10E-03 | 0.67 | 1.90 | 1.19E-09 | 1.26 | 1.89 | 3.82E-06 | 0.59 | 1.40 | 264106 | 181588 | 981611 | 813713 | 719940 | 593791 | 1243282 | 919480 |

**Supplementary table 1: Metabolomics results** (in asceding order according to p-value from progressive ALD vs  non-progressive ALD comparison, with molecular features with ID_level of 1-2 first)

| | | | | | | | | all_ALD_vs_control | | | Prog_ALD_vs_non-prog_ALD | | | Prog_ALD_vs_control_ALD | | | Non-prog_ALD_vs_control | | | Healthy_control | | all_ALD | | Non-prog_ALD | | Progressive_ALD | |
|---|---|---|---|---|---|---|---|---|---|---|---|---|---|---|---|---|---|---|---|---|---|---|---|---|---|---|---|
| | | | | | | | | t-test | Cohen's | PLS-DA | t-test | Cohen's | PLS-DA | t-test | Cohen's | PLS-DA | t-test | Cohen's | PLS-DA | | | | | | | | |
| Feature_ID | RP_HILIC | POS_NEG | Average.Rt | Average.Mz | Metabolite.name | curated_MSMS | ID_level | p | d | VIP | p | d | VIP | p | d | VIP | p | d | VIP | Mean | SD | Mean | SD | Mean | SD | Mean | SD |
| RP_POS_322_07068a3_003 | RP | POS | 3.00 | 322.0707 | | | | 4.71E-06 | 0.55 | 1.08 | 1.11E-03 | 0.72 | 1.94 | 1.27E-05 | 0.91 | 1.39 | 7.69E-02 | 0.19 | 0.57 | 122447 | 230748 | 1275938 | 2353867 | 517138 | 1519406 | 2034737 | 2776833 |
| RP_POS_1013_65375a10_502 | RP | POS | 10.50 | 1013.6538 | | | | 6.47E-01 | -0.09 | 0.92 | 1.12E-03 | -0.66 | 2.02 | 5.96E-02 | -0.42 | 0.94 | 2.37E-01 | 0.24 | 0.83 | 15567390 | 1775098 | 15395180 | 2038673 | 16047342 | 1779909 | 14743018 | 2088332 |
| RP_NEG_612_2644a10_376 | RP | NEG | 10.38 | 612.2644 | | | | 2.90E-01 | 0.20 | 1.00 | 1.13E-03 | -0.66 | 1.91 | 5.65E-01 | -0.13 | 0.62 | 1.02E-02 | 0.53 | 1.25 | 2124985 | 260514 | 2183622 | 298861 | 2279207 | 252874 | 2088036 | 312837 |
| HILIC_NEG_68_995a0_927 | HILIC | NEG | 0.927 | 68.9950 | | | | 1.25E-02 | -0.50 | 1.10 | 1.14E-03 | -0.63 | 1.77 | 2.01E-04 | -0.82 | 1.48 | 3.97E-01 | -0.19 | 0.40 | 400606 | 75412 | 360635 | 78303 | 385650 | 80633 | 335620 | 67891 |
| RP_NEG_1035_66479a10_503 | RP | NEG | 10.50 | 1035.6648 | | | | 8.20E-01 | 0.05 | 0.92 | 1.15E-03 | -0.65 | 2.02 | 2.21E-01 | -0.28 | 0.78 | 8.23E-02 | 0.37 | 0.97 | 44540337 | 7360813 | 44887900 | 7796077 | 47377385 | 6659921 | 42398416 | 8111439 |
| HILIC_NEG_448_3074a0_992 | HILIC | NEG | 0.992 | 448.3074 | | | | 2.39E-03 | 0.41 | 0.85 | 1.17E-03 | 0.72 | 1.84 | 5.83E-04 | 0.77 | 1.07 | 5.99E-01 | 0.05 | 0.27 | 580400 | 1290916 | 1861764 | 3448593 | 744874 | 1495022 | 2978654 | 4387186 |
| HILIC_NEG_243_06223a3_277 | HILIC | NEG | 3.277 | 243.0622 | | | | 9.82E-05 | 0.58 | 0.92 | 1.17E-03 | 0.69 | 1.74 | 2.45E-05 | 0.93 | 1.39 | 7.66E-02 | 0.24 | 0.71 | 458687 | 67461 | 530832 | 132276 | 488326 | 80705 | 573339 | 158589 |
| HILIC_POS_295_18622a1_04 | HILIC | POS | 1.04 | 295.1862 | | | | 5.84E-06 | 0.58 | 1.27 | 1.18E-03 | 0.71 | 1.76 | 1.53E-05 | 0.94 | 1.37 | 3.00E-02 | 0.23 | 0.70 | 147240 | 233214 | 580136 | 817444 | 316890 | 457731 | 843381 | 999563 |
| RP_POS_302_21466a8_097 | RP | POS | 8.10 | 302.2147 | | | | 7.71E-13 | 0.96 | 1.72 | 1.21E-03 | 0.66 | 2.00 | 5.75E-10 | 1.29 | 1.96 | 2.41E-06 | 0.63 | 1.54 | 207485 | 144350 | 595420 | 415234 | 462991 | 304260 | 727849 | 469034 |
| RP_POS_417_33597a9_229 | RP | POS | 9.23 | 417.3360 | | | | 5.31E-08 | 0.65 | 1.11 | 1.24E-03 | 0.72 | 1.91 | 1.17E-05 | 1.01 | 1.41 | 2.58E-11 | 0.29 | 1.98 | 73350 | 47426 | 648299 | 973572 | 332265 | 213063 | 964332 | 1290659 |
| RP_POS_472_30341a9_451 | RP | POS | 9.45 | 472.3034 | | | | 2.64E-04 | 0.44 | 0.85 | 1.25E-03 | 0.73 | 1.93 | 4.25E-04 | 0.80 | 1.18 | 1.24E-01 | 0.07 | 0.85 | 892341 | 873526 | 3442648 | 6583863 | 1307855 | 1539860 | 5577440 | 8712478 |
| HILIC_NEG_242_10832a0_723 | HILIC | NEG | 0.723 | 242.1083 | | | | 6.83E-07 | 0.60 | 1.28 | 1.28E-03 | 0.71 | 1.93 | 1.14E-05 | 0.95 | 1.35 | 6.81E-03 | 0.24 | 0.84 | 17527 | 33201 | 191829 | 324507 | 87960 | 172106 | 295698 | 401398 |
| RP_POS_365_30182a11_257 | RP | POS | 11.26 | 365.3018 | | | | 5.45E-05 | 0.59 | 1.34 | 1.30E-03 | 0.69 | 1.90 | 3.35E-05 | 0.93 | 1.46 | 4.47E-02 | 0.24 | 1.00 | 854327 | 546071 | 1489876 | 1167318 | 1116306 | 596498 | 1863445 | 1453429 |
| RP_POS_399_32559a9_234 | RP | POS | 9.23 | 399.3256 | | | | 1.10E-08 | 0.69 | 1.16 | 1.31E-03 | 0.71 | 1.90 | 6.10E-06 | 1.04 | 1.45 | 7.12E-12 | 0.33 | 2.05 | 90388 | 59071 | 683149 | 946471 | 377438 | 226284 | 988860 | 1252182 |
| RP_NEG_448_30728a9_46 | RP | NEG | 9.46 | 448.3073 | | | | 3.06E-04 | 0.43 | 0.82 | 1.33E-03 | 0.73 | 1.92 | 4.69E-04 | 0.80 | 1.16 | 1.36E-01 | 0.07 | 0.86 | 3574796 | 3385350 | 12681256 | 23682691 | 5040534 | 5427395 | 20321977 | 31378102 |
| RP_POS_876_54926a12_545 | RP | POS | 12.55 | 876.5493 | | | | 1.35E-02 | -0.49 | 1.19 | 1.36E-03 | -0.63 | 1.88 | 1.60E-04 | -0.80 | 1.59 | 4.38E-01 | -0.17 | 0.62 | 728227 | 330540 | 553553 | 356718 | 665978 | 385380 | 441127 | 287714 |
| RP_POS_413_78531a14_754 | RP | POS | 14.75 | 413.7853 | | | | 3.38E-03 | 0.51 | 0.87 | 1.42E-03 | 0.66 | 1.88 | 1.89E-04 | 0.84 | 1.23 | 2.83E-01 | 0.18 | 0.48 | 913521 | 749439 | 1423960 | 1039752 | 1096601 | 743973 | 1751318 | 1188291 |
| RP_POS_537_30505a10_499 | RP | POS | 10.50 | 537.3051 | | | | 1.16E-04 | 0.61 | 1.19 | 1.43E-03 | 0.66 | 1.89 | 1.25E-05 | 0.94 | 1.72 | 7.51E-02 | 0.28 | 0.81 | 316996 | 164891 | 481245 | 285278 | 391500 | 206724 | 570990 | 324431 |
| RP_POS_337_27118a11_097 | RP | POS | 11.10 | 337.2712 | | | | 1.11E-01 | 0.28 | 1.07 | 1.54E-03 | 0.67 | 1.85 | 1.02E-02 | 0.61 | 1.02 | 7.45E-01 | -0.05 | 0.77 | 981814 | 469592 | 1149732 | 629208 | 951147 | 305557 | 1348316 | 791226 |
| RP_POS_568_33685a10_396 | RP | POS | 10.40 | 568.3369 | | | | 4.33E-02 | 0.41 | 1.06 | 1.54E-03 | -0.62 | 1.86 | 6.72E-01 | 0.09 | 0.54 | 1.14E-03 | 0.72 | 1.30 | 2229139 | 856328 | 2593312 | 898262 | 2872870 | 797186 | 2313753 | 913901 |
| RP_POS_826_53473a12_406 | RP | POS | 12.41 | 826.5347 | | | | 3.20E-01 | -0.21 | 0.81 | 1.59E-03 | -0.61 | 1.84 | 2.28E-02 | -0.52 | 1.00 | 7.08E-01 | 0.09 | 0.43 | 2062574 | 1323996 | 1797651 | 1212506 | 2174478 | 1292387 | 1420823 | 1005130 |
| RP_POS_1020_13263a10_499 | RP | POS | 10.50 | 1020.1326 | | | | 7.69E-01 | -0.06 | 0.95 | 1.59E-03 | -0.64 | 1.95 | 9.21E-02 | -0.38 | 0.88 | 1.96E-01 | 0.26 | 0.90 | 2904945 | 455803 | 2876664 | 521787 | 3038690 | 446422 | 2714638 | 545170 |
| RP_NEG_288_03061a3_001 | RP | NEG | 3.00 | 288.0306 | | | | 1.80E-06 | 0.57 | 1.11 | 1.61E-03 | 0.70 | 1.89 | 1.52E-05 | 0.92 | 1.38 | 2.52E-02 | 0.22 | 0.68 | 80078 | 184533 | 1911647 | 3596447 | 786571 | 2152095 | 3036724 | 4349489 |
| RP_POS_290_0444a3_002 | RP | POS | 3.00 | 290.0444 | | | | 3.33E-06 | 0.55 | 1.14 | 1.62E-03 | 0.70 | 1.90 | 2.76E-05 | 0.90 | 1.37 | 2.44E-02 | 0.20 | 0.68 | 63190 | 136752 | 1985962 | 3895484 | 765059 | 2130437 | 3206865 | 4804005 |
| RP_NEG_205_83928a15_552 | RP | NEG | 15.55 | 205.8393 | | | | 1.48E-01 | -0.31 | 0.54 | 1.62E-03 | -0.60 | 2.14 | 5.95E-03 | -0.62 | 1.13 | 9.60E-01 | -0.01 | 0.15 | 5058702 | 267611 | 4980620 | 240918 | 5055620 | 276509 | 4905620 | 171263 |
| RP_POS_651_37079a9_234 | RP | POS | 9.23 | 651.3708 | | | | 4.33E-08 | 0.66 | 1.12 | 1.62E-03 | 0.70 | 1.86 | 1.29E-05 | 1.01 | 1.41 | 1.16E-11 | 0.31 | 2.03 | 104865 | 67376 | 908674 | 1350555 | 480632 | 301485 | 1336715 | 1794550 |
| HILIC_NEG_224_06192a1_193 | HILIC | NEG | 1.193 | 224.0619 | | | | 2.31E-08 | 0.75 | 1.35 | 1.64E-03 | 0.67 | 1.70 | 8.50E-07 | 1.08 | 1.65 | 2.11E-04 | 0.41 | 1.58 | 1253002 | 3477946 | 8213789 | 9940646 | 5102618 | 5500076 | 11324960 | 12232943 |
| RP_POS_797_02637a12_96 | RP | POS | 12.96 | 797.0264 | | | | 1.48E-19 | 1.34 | 2.13 | 1.66E-03 | 0.56 | 1.86 | 3.83E-16 | 1.62 | 2.41 | 1.31E-11 | 1.06 | 2.05 | 186766 | 215239 | 826252 | 430331 | 693114 | 364745 | 959390 | 452697 |
| HILIC_NEG_185_04303a0_722 | HILIC | NEG | 0.722 | 185.0430 | | | | 2.71E-08 | 0.67 | 1.35 | 1.70E-03 | 0.69 | 1.98 | 3.22E-06 | 1.02 | 1.43 | 3.08E-04 | 0.33 | 1.14 | 53344 | 44073 | 291211 | 388482 | 169971 | 207043 | 412452 | 481717 |
| RP_POS_765_98688a10_375 | RP | POS | 10.38 | 765.9869 | | | | 2.54E-01 | 0.21 | 0.95 | 1.72E-03 | -0.65 | 1.90 | 5.69E-01 | -0.12 | 0.65 | 1.03E-02 | 0.53 | 1.11 | 1495725 | 485156 | 1617508 | 619771 | 1808402 | 581784 | 1426614 | 602686 |
| RP_POS_771_96429a10_374 | RP | POS | 10.37 | 771.9643 | | | | 2.83E-01 | 0.21 | 0.96 | 1.74E-03 | -0.63 | 1.90 | 6.19E-01 | -0.11 | 0.65 | 1.86E-02 | 0.52 | 1.07 | 445443 | 198297 | 490261 | 219012 | 557679 | 216769 | 422843 | 201612 |
| HILIC_POS_659_41449a1_228 | HILIC | POS | 1.228 | 659.4145 | | | | 1.20E-02 | 0.50 | 0.90 | 1.74E-03 | -0.61 | 1.67 | 3.69E-01 | 0.19 | 0.35 | 3.35E-04 | 0.80 | 1.39 | 343402 | 186154 | 443474 | 200427 | 505163 | 193698 | 381785 | 189412 |
| RP_POS_524_29938a10_375 | RP | POS | 10.38 | 524.2994 | | | | 2.10E-01 | 0.24 | 0.97 | 1.74E-03 | -0.64 | 1.93 | 7.03E-01 | -0.08 | 0.66 | 8.91E-03 | 0.55 | 1.15 | 2757585 | 711987 | 2948869 | 835158 | 3205893 | 771949 | 2691844 | 823704 |
| RP_POS_1013_65259a10_375 | RP | POS | 10.38 | 1013.6526 | | | | 1.07E-01 | 0.29 | 1.03 | 1.75E-03 | -0.64 | 1.90 | 9.00E-01 | -0.03 | 0.65 | 3.61E-03 | 0.62 | 1.22 | 2046055 | 872038 | 2355023 | 1091226 | 2690773 | 1054637 | 2019273 | 1031277 |
| RP_POS_1041_68689a10_506 | RP | POS | 10.51 | 1041.6869 | | | | 1.81E-02 | 0.47 | 1.17 | 1.76E-03 | -0.61 | 1.85 | 4.37E-01 | 0.17 | 0.65 | 4.99E-04 | 0.78 | 1.36 | 11670630 | 5298174 | 14322989 | 5548621 | 16029294 | 5184188 | 12616685 | 5420804 |
| RP_POS_430_74789a12_734 | RP | POS | 12.73 | 430.7479 | | | | 1.25E-04 | -0.84 | 1.36 | 1.76E-03 | -0.56 | 1.81 | 1.57E-06 | -1.12 | 1.91 | 1.72E-02 | -0.56 | 0.99 | 1959496 | 702878 | 1377666 | 632567 | 1572625 | 693219 | 1182707 | 500325 |
| HILIC_NEG_224_06194a1_296 | HILIC | NEG | 1.296 | 224.0619 | | | | 5.19E-05 | 0.58 | 1.10 | 1.77E-03 | 0.67 | 1.67 | 2.79E-05 | 0.92 | 1.45 | 5.52E-02 | 0.25 | 0.82 | 486846 | 287329 | 829783 | 638271 | 631817 | 385147 | 1027750 | 771217 |
| HILIC_NEG_244_00462a0_616 | HILIC | NEG | 0.616 | 244.0046 | | | | 3.25E-08 | 0.69 | 1.44 | 1.77E-03 | 0.68 | 1.81 | 1.93E-06 | 1.03 | 1.48 | 7.61E-04 | 0.35 | 1.21 | 148102 | 66288 | 346542 | 314722 | 248887 | 184125 | 444197 | 383089 |
| RP_NEG_201_80153a0_564 | RP | NEG | 0.56 | 201.8015 | | | | 1.63E-03 | -0.56 | 0.98 | 1.78E-03 | -0.63 | 2.11 | 5.11E-05 | -0.88 | 1.44 | 1.89E-01 | -0.25 | 0.76 | 11844908 | 1676013 | 10628734 | 2228451 | 11313818 | 1903205 | 9943650 | 2335231 |
| HILIC_NEG_213_04958a6_093 | HILIC | NEG | 6.093 | 213.0496 | | | | 2.30E-01 | -0.18 | 0.94 | 1.78E-03 | -0.67 | 1.67 | 9.07E-03 | -0.52 | 1.06 | 3.65E-01 | 0.15 | 1.04 | 214932 | 30923 | 205404 | 56871 | 222889 | 48094 | 187918 | 59981 |
| HILIC_NEG_243_10449a0_723 | HILIC | NEG | 0.723 | 243.1045 | | | | 4.62E-07 | 0.60 | 1.30 | 1.79E-03 | 0.69 | 1.92 | 1.06E-05 | 0.95 | 1.35 | 5.26E-03 | 0.26 | 0.86 | 47159 | 120545 | 760516 | 1308746 | 354195 | 730487 | 1166837 | 1609362 |
| RP_POS_326_03061a3_86 | RP | POS | 3.86 | 326.0306 | | | | 1.64E-02 | 0.36 | 0.67 | 1.79E-03 | 0.68 | 1.83 | 2.16E-03 | 0.70 | 1.12 | 9.07E-01 | 0.01 | 0.48 | 625418 | 810330 | 1154263 | 1629465 | 646506 | 758070 | 1662020 | 2064866 |
| HILIC_NEG_144_92342a0_926 | HILIC | NEG | 0.926 | 144.9234 | | | | 5.12E-07 | -0.87 | 1.56 | 1.84E-03 | -0.61 | 1.70 | 6.11E-09 | -1.18 | 2.00 | 2.91E-03 | -0.57 | 1.08 | 47275634 | 6409501 | 39280130 | 9102254 | 42068052 | 8913495 | 36492207 | 8490008 |
| HILIC_POS_432_31088a1_021 | HILIC | POS | 1.021 | 432.3109 | | | | 4.37E-03 | 0.38 | 0.81 | 1.86E-03 | 0.70 | 1.80 | 1.44E-03 | 0.72 | 0.98 | 7.41E-01 | 0.03 | 0.17 | 486976 | 1157615 | 1651439 | 3453224 | 574226 | 1163911 | 2728651 | 4513319 |
| RP_POS_432_31104a9_451 | RP | POS | 9.45 | 432.3110 | | | | 4.24E-04 | 0.42 | 0.80 | 1.86E-03 | 0.71 | 1.87 | 7.01E-04 | 0.77 | 1.12 | 1.12E-01 | 0.07 | 0.87 | 582966 | 585906 | 2370446 | 4803000 | 866931 | 1012553 | 3873961 | 6400763 |
| RP_POS_236_03358a3_003 | RP | POS | 3.00 | 236.0336 | | | | 7.82E-12 | 0.87 | 1.52 | 1.88E-03 | 0.65 | 1.87 | 1.25E-08 | 1.20 | 1.80 | 8.29E-06 | 0.55 | 1.37 | 1005533 | 742972 | 3425360 | 2926629 | 2525579 | 2022876 | 4325142 | 3400123 |
| RP_POS_794_50818a12_646 | RP | POS | 12.65 | 794.5082 | | | | 2.57E-04 | 0.61 | 1.26 | 1.88E-03 | 0.64 | 1.82 | 3.08E-05 | 0.93 | 1.48 | 7.30E-02 | 0.28 | 0.83 | 775270 | 373643 | 1111056 | 579342 | 932891 | 395796 | 1289222 | 675787 |
| HILIC_NEG_540_33002a1_126 | HILIC | NEG | 1.126 | 540.3300 | | | | 2.38E-02 | 0.41 | 0.64 | 1.96E-03 | -0.63 | 1.65 | 6.33E-01 | 0.10 | 0.35 | 5.43E-04 | 0.73 | 1.26 | 1648139 | 382164 | 1840306 | 482803 | 1987332 | 460426 | 1693279 | 463515 |
| RP_POS_172_09648a0_708 | RP | POS | 0.71 | 172.0965 | | | | 1.01E-04 | 0.54 | 1.03 | 1.99E-03 | 0.67 | 1.83 | 3.36E-05 | 0.88 | 1.40 | 1.16E-01 | 0.21 | 0.55 | 189664 | 134814 | 351990 | 325898 | 252287 | 222381 | 451693 | 380586 |
| RP_POS_991_67139a10_502 | RP | POS | 10.50 | 991.6714 | | | | 1.98E-01 | 0.25 | 1.02 | 2.02E-03 | -0.62 | 1.93 | 7.58E-01 | -0.07 | 0.66 | 9.38E-03 | 0.56 | 1.17 | 228750475 | 65772381 | 246769168 | 75203124 | 269608672 | 70190754 | 223929665 | 73723259 |
| HILIC_NEG_279_61429a1_729 | HILIC | NEG | 1.729 | 279.6143 | | | | 1.00E-03 | 0.45 | 0.97 | 2.02E-03 | 0.68 | 1.70 | 4.01E-04 | 0.79 | 1.10 | 3.07E-01 | 0.11 | 0.36 | 131784 | 96513 | 234281 | 251937 | 156701 | 121425 | 311862 | 318139 |
| HILIC_NEG_176_99821a0_927 | HILIC | NEG | 0.927 | 176.9982 | | | | 7.23E-02 | -0.36 | 0.97 | 2.07E-03 | -0.61 | 1.75 | 3.25E-03 | -0.66 | 1.14 | 7.97E-01 | -0.06 | 0.13 | 1558665 | 294342 | 1447694 | 309582 | 1541497 | 293335 | 1353892 | 299392 |
| HILIC_POS_250_1284a1_039 | HILIC | POS | 1.039 | 250.1284 | | | | 3.55E-05 | 0.53 | 1.24 | 2.10E-03 | 0.68 | 1.68 | 6.77E-05 | 0.86 | 1.25 | 6.80E-02 | 0.19 | 0.60 | 284522 | 383194 | 964872 | 1435512 | 525667 | 786630 | 1404077 | 1775101 |
| HILIC_NEG_213_99434a0_618 | HILIC | NEG | 0.618 | 213.9943 | | | | 1.20E-08 | 0.70 | 1.45 | 2.14E-03 | 0.66 | 1.79 | 1.55E-06 | 1.04 | 1.48 | 2.97E-04 | 0.37 | 1.25 | 181313 | 76987 | 445647 | 408975 | 320941 | 239208 | 570353 | 498976 |
| RP_POS_791_54236a12_813 | RP | POS | 12.81 | 791.5424 | | | | 2.68E-02 | 0.41 | 0.80 | 2.14E-03 | -0.62 | 1.92 | 5.95E-01 | 0.10 | 0.60 | 1.33E-03 | 0.72 | 1.24 | 1256153 | 604568 | 1549740 | 730228 | 1770643 | 788391 | 1328836 | 596825 |
| HILIC_NEG_234_8201a0_996 | HILIC | NEG | 0.996 | 234.8201 | | | | 5.66E-03 | -0.42 | 0.69 | 2.16E-03 | -0.66 | 1.64 | 4.09E-04 | -0.75 | 1.14 | 5.40E-01 | -0.09 | 0.43 | 645745 | 99859 | 573068 | 186784 | 629669 | 136129 | 516466 | 213118 |
| RP_NEG_145_06091a0_529 | RP | NEG | 0.53 | 145.0609 | | | | 3.66E-01 | -0.14 | 0.64 | 2.17E-03 | -0.66 | 1.78 | 1.70E-02 | -0.47 | 0.87 | 2.89E-01 | 0.19 | 0.64 | 6863256 | 1183331 | 6596615 | 2056239 | 7217289 | 1821287 | 5975942 | 2106952 |
| RP_NEG_429_30151a9_971 | RP | NEG | 9.97 | 429.3015 | | | | 4.32E-04 | 0.49 | 0.89 | 2.17E-03 | 0.67 | 1.89 | 2.67E-04 | 0.82 | 1.18 | 1.51E-01 | 0.15 | 0.76 | 1047233 | 337282 | 1419439 | 838601 | 1162565 | 371516 | 1676313 | 1071527 |
| HILIC_NEG_315_04205a6_344 | HILIC | NEG | 6.344 | 315.0421 | | | | 1.73E-02 | 0.46 | 0.94 | 2.19E-03 | 0.61 | 1.75 | 9.32E-04 | 0.77 | 1.17 | 4.25E-01 | 0.16 | 0.40 | 2897535 | 627441 | 3219190 | 701708 | 3007294 | 566231 | 3431086 | 763274 |
| RP_POS_453_29739a9_972 | RP | POS | 9.97 | 453.2974 | Steroid, unknown | 453.3004(100) | 3 | 8.95E-05 | 0.53 | 0.96 | 2.21E-03 | 0.67 | 1.87 | 1.09E-04 | 0.87 | 1.25 | 5.90E-02 | 0.20 | 0.87 | 1990529 | 643141 | 2823285 | 1714992 | 2299506 | 808104 | 3347064 | 2174742 |
| HILIC_NEG_540_32996a1_253 | HILIC | NEG | 1.253 | 540.3300 | | | | 7.52E-02 | 0.32 | 0.72 | 2.22E-03 | -0.63 | 1.66 | 9.96E-01 | 0.00 | 0.31 | 3.17E-03 | 0.63 | 1.05 | 11139541 | 2611250 | 12180229 | 3445264 | 13217880 | 3556227 | 11142578 | 3022877 |
| RP_POS_431_31564a9_972 | RP | POS | 9.97 | 431.3156 | | | | 8.75E-05 | 0.53 | 0.95 | 2.23E-03 | 0.67 | 1.87 | 1.14E-04 | 0.87 | 1.24 | 5.48E-02 | 0.20 | 0.87 | 4861497 | 1546398 | 6876501 | 4152438 | 5608824 | 1898068 | 8144179 | 5287260 |
| RP_POS_414_30026a9_451 | RP | POS | 9.45 | 414.3003 | | | | 3.35E-04 | 0.43 | 0.81 | 2.24E-03 | 0.69 | 1.85 | 7.03E-04 | 0.77 | 1.12 | 5.41E-02 | 0.08 | 0.94 | 263600 | 261072 | 1086130 | 2171077 | 418502 | 455358 | 1753757 | 2899371 |
| RP_NEG_465_25021a8_564 | RP | NEG | 8.56 | 465.2502 | | | | 1.76E-02 | -0.59 | 1.10 | 2.26E-03 | -0.53 | 1.81 | 7.23E-04 | -0.85 | 1.67 | 2.31E-01 | -0.33 | 0.75 | 1633145 | 1356266 | 996713 | 932408 | 1280537 | 1170635 | 712889 | 470876 |
| HILIC_NEG_259_00095a0_929 | HILIC | NEG | 0.929 | 259.0010 | | | | 2.72E-01 | -0.22 | 0.89 | 2.29E-03 | -0.61 | 1.70 | 1.62E-02 | -0.52 | 0.94 | 6.95E-01 | 0.09 | 0.15 | 1184436 | 211155 | 1136007 | 226139 | 1203891 | 229700 | 1068122 | 202866 |
| HILIC_POS_191_04037a6_128 | HILIC | POS | 6.128 | 191.0404 | | | | 4.89E-02 | -0.34 | 1.00 | 2.30E-03 | -0.64 | 1.60 | 2.87E-03 | -0.66 | 1.17 | 9.13E-01 | -0.02 | 0.98 | 869309 | 86989 | 829771 | 123949 | 867088 | 93857 | 792454 | 139244 |
| RP_NEG_397_22391a9_544 | RP | NEG | 9.54 | 397.2239 | | | | 2.35E-08 | 0.70 | 1.23 | 2.33E-03 | 0.66 | 1.83 | 5.50E-07 | 1.02 | 1.59 | 3.34E-03 | 0.37 | 0.95 | 1205686 | 1049919 | 4391292 | 4994879 | 2888286 | 3667015 | 5894297 | 5687664 |
| RP_POS_434_32614a9_857 | RP | POS | 9.86 | 434.3261 | | | | 3.18E-02 | 0.31 | 0.66 | 2.34E-03 | 0.68 | 1.85 | 5.77E-03 | 0.64 | 1.03 | 7.26E-01 | -0.03 | 0.62 | 150717 | 265407 | 316247 | 599918 | 132643 | 145703 | 499852 | 798200 |
| RP_POS_522_79785a10_375 | RP | POS | 10.38 | 522.7979 | | | | 3.90E-01 | 0.17 | 0.95 | 2.39E-03 | -0.62 | 1.89 | 5.08E-01 | -0.14 | 0.70 | 2.85E-02 | 0.47 | 1.03 | 1400718 | 384027 | 1470529 | 434773 | 1600502 | 410056 | 1340555 | 423427 |
| RP_NEG_475_3071a9_971 | RP | NEG | 9.97 | 475.3071 | | | | 2.47E-04 | 0.50 | 0.91 | 2.39E-03 | 0.67 | 1.88 | 2.14E-04 | 0.83 | 1.19 | 1.03E-01 | 0.17 | 0.79 | 1960317 | 603619 | 2675172 | 1565223 | 2200296 | 695672 | 3150048 | 2001416 |
| HILIC_POS_414_30017a1_021 | HILIC | POS | 1.021 | 414.3002 | | | | 6.90E-03 | 0.37 | 0.83 | 2.45E-03 | 0.67 | 1.76 | 1.86E-03 | 0.71 | 0.97 | 7.17E-01 | 0.04 | 0.16 | 250176 | 669210 | 815963 | 1685709 | 304463 | 644689 | 1327464 | 2188984 |
| RP_POS_518_31934a10_376 | RP | POS | 10.38 | 518.3193 | LPC 16:0 [M+Na]+ | 459.2449(60), 313.2737(50), 518.3217(50), 86.0972(40), 104.107(2), 146.9823(100) | | 1.85E-01 | 0.26 | 1.00 | 2.49E-03 | -0.61 | 1.89 | 8.31E-01 | -0.05 | 0.63 | 7.92E-03 | 0.56 | 1.20 | 58036062 | 10919761 | 61101830 | 12185999 | 64733609 | 10597371 | 57470050 | 12680909 |
| HILIC_POS_534_29486a1_171 | HILIC | POS | 1.171 | 534.2949 | | | | 6.23E-02 | 0.35 | 0.59 | 2.49E-03 | -0.61 | 1.62 | 8.32E-01 | 0.04 | 0.36 | 2.20E-03 | 0.66 | 1.13 | 544283 | 206835 | 627901 | 245450 | 700983 | 234417 | 554818 | 236367 |
| RP_POS_828_55182a12_321 | RP | POS | 12.32 | 828.5518 | | | | 3.13E-01 | 0.17 | 0.84 | 2.56E-03 | -0.63 | 1.78 | 4.27E-01 | -0.14 | 0.60 | 2.40E-02 | 0.49 | 0.83 | 677131 | 378634 | 763959 | 532115 | 922383 | 586514 | 605536 | 420536 |
| RP_POS_205_19521a9_512 | RP | POS | 9.51 | 205.1952 | | | | 2.46E-08 | 0.70 | 1.23 | 2.57E-03 | 0.65 | 1.81 | 4.08E-07 | 1.02 | 1.62 | 4.38E-03 | 0.37 | 0.93 | 261559 | 215626 | 900596 | 1001085 | 602468 | 768192 | 1198723 | 1119504 |
| RP_POS_421_21954a9_513 | RP | POS | 9.51 | 421.2195 | | | | 2.82E-08 | 0.69 | 1.23 | 2.63E-03 | 0.65 | 1.80 | 5.37E-07 | 1.01 | 1.61 | 4.46E-03 | 0.36 | 0.94 | 553025 | 484860 | 2073082 | 2409421 | 1357073 | 1824721 | 2789091 | 2711684 |
| RP_NEG_778_56183a13_58 | RP | NEG | 13.58 | 778.5618 | | | | 2.99E-02 | 0.42 | 0.89 | 2.64E-03 | 0.60 | 1.76 | 2.03E-03 | 0.72 | 1.17 | 5.46E-01 | 0.12 | 0.33 | 2277814 | 1030658 | 2759665 | 1168437 | 2412638 | 899843 | 3106692 | 1304939 |
| HILIC_POS_204_08662a1_039 | HILIC | POS | 1.039 | 204.0866 | | | | 4.38E-07 | 0.61 | 1.31 | 2.65E-03 | 0.66 | 1.66 | 1.83E-05 | 0.94 | 1.36 | 1.47E-03 | 0.27 | 0.96 | 44742 | 55603 | 378306 | 610708 | 195272 | 309802 | 561340 | 767588 |
| RP_POS_195_08783a3_778 | RP | POS | 3.78 | 195.0878 | | | | 7.42E-02 | 0.28 | 0.56 | 2.68E-03 | 0.65 | 1.76 | 7.43E-03 | 0.60 | 0.99 | 7.19E-01 | -0.05 | 0.58 | 41485600 | 48375992 | 63494421 | 86917083 | 37524135 | 48618924 | 89464707 | 107345673 |
| RP_POS_756_54956a13_575 | RP | POS | 13.58 | 756.5496 | | | | 1.30E-02 | 0.48 | 0.95 | 2.69E-03 | 0.60 | 1.75 | 7.78E-04 | 0.78 | 1.26 | 3.46E-01 | 0.19 | 0.40 | 2972271 | 1422189 | 3733412 | 1579670 | 3265444 | 1265407 | 4201380 | 1730162 |
| RP_POS_339_28687a11_173 | RP | POS | 11.17 | 339.2869 | | | | 3.15E-04 | 0.56 | 1.09 | 2.73E-03 | 0.64 | 1.77 | 1.66E-04 | 0.88 | 1.25 | 5.21E-02 | 0.24 | 0.89 | 945223 | 396109 | 1316039 | 701971 | 1105456 | 281711 | 1526621 | 908692 |
| RP_POS_754_99414a10_375 | RP | POS | 10.38 | 754.9941 | | | | 8.44E-02 | 0.29 | 0.94 | 2.74E-03 | -0.63 | 1.78 | 9.11E-01 | -0.02 | 0.58 | 3.06E-03 | 0.61 | 1.19 | 267455 | 112245 | 312404 | 163416 | 360619 | 163747 | 264189 | 149658 |
| RP_POS_770_46606a10_374 | RP | POS | 10.37 | 770.4661 | | | | 4.28E-02 | 0.37 | 1.07 | 2.77E-03 | -0.61 | 1.82 | 7.28E-01 | 0.07 | 0.67 | 1.82E-03 | 0.68 | 1.24 | 261912 | 119775 | 315221 | 147342 | 358657 | 149603 | 271784 | 132780 |
| RP_POS_416_26428a9_512 | RP | POS | 9.51 | 416.2643 | | | | 5.16E-08 | 0.68 | 1.21 | 2.78E-03 | 0.65 | 1.79 | 7.72E-07 | 1.00 | 1.58 | 5.87E-03 | 0.35 | 0.91 | 169565 | 168515 | 680571 | 826906 | 436136 | 625073 | 925006 | 932121 |
| HILIC_NEG_134_89479a6_412 | HILIC | NEG | 6.412 | 134.8948 | | | | 6.02E-01 | 0.10 | 0.36 | 2.83E-03 | -0.60 | 1.79 | 3.58E-01 | -0.20 | 0.36 | 7.26E-02 | 0.40 | 0.77 | 11966943 | 7140530 | 12743475 | 7709107 | 15010762 | 7730484 | 10476188 | 7058480 |
| HILIC_NEG_99_92568a0_725 | HILIC | NEG | 0.725 | 99.9257 | | | | 6.63E-11 | 0.86 | 1.54 | 2.84E-03 | 0.62 | 1.73 | 2.74E-08 | 1.17 | 1.74 | 1.79E-05 | 0.55 | 1.47 | 628620 | 154343 | 1025211 | 485795 | 881617 | 338760 | 1168806 | 565661 |
| RP_POS_445_3674a12_358 | RP | POS | 12.36 | 445.3674 | | | | 1.42E-01 | -0.26 | 1.15 | 2.84E-03 | -0.62 | 1.77 | 9.59E-03 | -0.57 | 1.33 | 8.05E-01 | 0.05 | 0.42 | 3600099 | 568800 | 3414303 | 741474 | 3632702 | 599912 | 3195903 | 808425 |

**Supplementary table 1: Metabolomics results** (in asceding order according to p-value from progressive ALD vs  non-progressive ALD comparison, with molecular features with ID_level of 1-2 first)

| Feature_ID | RP_HILIC | POS_NEG | Average.Rt | Average.Mz | Metabolite.name | curated_MSMS | ID_level | all_ALD_vs_control t-test p | all_ALD_vs_control Cohen's d | all_ALD_vs_control PLS-DA VIP | Prog_ALD_vs_non-prog_ALD t-test p | Prog_ALD_vs_non-prog_ALD Cohen's d | Prog_ALD_vs_non-prog_ALD PLS-DA VIP | Prog_ALD_vs_control_ALD t-test p | Prog_ALD_vs_control_ALD Cohen's d | Prog_ALD_vs_control_ALD PLS-DA VIP | Non-prog_ALD_vs_control t-test p | Non-prog_ALD_vs_control Cohen's d | Non-prog_ALD_vs_control PLS-DA VIP |
|---|---|---|---|---|---|---|---|---|---|---|---|---|---|---|---|---|---|---|---|
| RP_POS_519_32245a10_375 | RP | POS | 10.38 | 519.3225 | | | | 2.01E-01 | 0.25 | 1.00 | 2.86E-03 | -0.60 | 1.86 | 8.18E-01 | -0.05 | 0.63 | 9.66E-03 | 0.55 | 1.19 |
| RP_POS_445_36655a12_428 | RP | POS | 12.43 | 445.3666 | | | | 1.73E-01 | -0.27 | 1.02 | 2.87E-03 | -0.59 | 1.79 | 1.45E-02 | -0.57 | 1.19 | 9.12E-01 | 0.02 | 0.38 |
| RP_POS_478_32712a10_505 | RP | POS | 10.51 | 478.3271 | | | | 5.72E-01 | -0.11 | 0.55 | 2.92E-03 | -0.60 | 1.72 | 5.72E-02 | -0.41 | 0.86 | 4.04E-01 | 0.19 | 0.47 |
| RP_NEG_261_0748a7_839 | RP | NEG | 7.84 | 261.0748 | | | | 1.85E-02 | -0.50 | 1.17 | 2.94E-03 | -0.56 | 1.75 | 6.18E-04 | -0.79 | 1.43 | 3.44E-01 | -0.22 | 0.84 |
| HILIC_POS_151_04778a6_129 | HILIC | POS | 6.129 | 151.0478 | | | | 3.64E-02 | -0.35 | 1.08 | 2.99E-03 | -0.63 | 1.60 | 1.97E-03 | -0.66 | 1.18 | 8.54E-01 | -0.03 | 1.03 |
| HILIC_NEG_181_05098a1_076 | HILIC | NEG | 1.076 | 181.0510 | | | | 2.15E-03 | 0.49 | 0.95 | 3.03E-03 | 0.63 | 1.74 | 3.07E-04 | 0.80 | 1.18 | 2.42E-01 | 0.18 | 0.49 |
| RP_NEG_195_81055a0_565 | RP | NEG | 0.57 | 195.8106 | | | | 7.64E-06 | -0.77 | 1.21 | 3.08E-03 | -0.59 | 1.98 | 4.00E-07 | -1.07 | 1.68 | 9.09E-03 | -0.48 | 0.97 |
| RP_POS_497_39624a13_923 | RP | POS | 13.92 | 497.3962 | | | | 1.87E-01 | 0.31 | 0.51 | 3.09E-03 | -0.54 | 1.80 | 8.83E-01 | 0.04 | 0.38 | 1.72E-02 | 0.58 | 0.97 |
| HILIC_POS_152_03162a6_129 | HILIC | POS | 6.129 | 152.0316 | | | | 2.61E-02 | -0.36 | 1.10 | 3.17E-03 | -0.63 | 1.59 | 1.58E-03 | -0.67 | 1.20 | 7.87E-01 | -0.05 | 1.09 |
| HILIC_NEG_157_90067a0_943 | HILIC | NEG | 0.943 | 157.9007 | | | | 4.02E-08 | 0.82 | 1.41 | 3.19E-03 | 0.60 | 1.59 | 8.89E-08 | 1.12 | 1.77 | 5.99E-04 | 0.52 | 1.48 |
| RP_POS_267_19305a9_053 | RP | POS | 9.05 | 267.1931 | | | | 9.00E-08 | 0.70 | 1.30 | 3.20E-03 | 0.64 | 1.74 | 5.78E-06 | 1.02 | 1.49 | 1.04E-04 | 0.38 | 1.45 |
| RP_POS_329_16251a10_822 | RP | POS | 10.82 | 329.1625 | | | | 2.12E-07 | 0.93 | 1.78 | 3.22E-03 | 0.57 | 1.86 | 5.48E-08 | 1.21 | 1.94 | 2.31E-04 | 0.64 | 1.60 |
| HILIC_NEG_118_92419a0_925 | HILIC | NEG | 0.925 | 118.9242 | | | | 2.89E-06 | -0.95 | 1.68 | 3.22E-03 | -0.54 | 1.66 | 7.73E-08 | -1.22 | 1.95 | 1.11E-03 | -0.68 | 1.33 |
| RP_POS_222_01859a1_361 | RP | POS | 1.36 | 222.0186 | | | | 8.84E-09 | 0.69 | 1.26 | 3.23E-03 | 0.64 | 1.80 | 1.50E-06 | 1.01 | 1.50 | 6.87E-04 | 0.37 | 1.05 |
| RP_POS_593_33289a8_601 | RP | POS | 8.60 | 593.3329 | | | | 4.59E-04 | 0.43 | 0.79 | 3.27E-03 | 0.66 | 1.86 | 7.51E-04 | 0.76 | 1.06 | 1.48E-01 | 0.10 | 0.67 |
| RP_POS_781_05286a12_965 | RP | POS | 12.97 | 781.0529 | | | | 9.92E-18 | 1.32 | 2.11 | 3.31E-03 | 0.53 | 1.71 | 5.98E-15 | 1.58 | 2.35 | 2.97E-11 | 1.06 | 2.01 |
| RP_POS_550_39172a11_282 | RP | POS | 11.28 | 550.3917 | | | | 2.09E-04 | 0.50 | 1.12 | 3.33E-03 | 0.64 | 1.72 | 1.60E-04 | 0.82 | 1.35 | 1.25E-01 | 0.18 | 0.70 |
| RP_NEG_650_35107a13_182 | RP | NEG | 13.18 | 650.3511 | | | | 6.39E-01 | -0.10 | 0.31 | 3.35E-03 | -0.56 | 2.01 | 1.03E-01 | -0.38 | 0.83 | 4.43E-01 | 0.18 | 0.49 |
| HILIC_NEG_273_97845a0_729 | HILIC | NEG | 0.729 | 273.9785 | | | | 8.16E-14 | 0.98 | 1.67 | 3.38E-03 | 0.60 | 1.72 | 4.93E-10 | 1.28 | 1.97 | 7.19E-07 | 0.68 | 1.64 |
| HILIC_NEG_117_05566a0_925 | HILIC | NEG | 0.925 | 117.0557 | | | | 1.07E-06 | 0.58 | 1.30 | 3.42E-03 | 0.65 | 1.90 | 3.30E-05 | 0.91 | 1.27 | 2.47E-03 | 0.26 | 0.94 |
| HILIC_NEG_165_0192a0_927 | HILIC | NEG | 0.927 | 165.0192 | | | | 3.58E-01 | 0.16 | 0.47 | 3.45E-03 | -0.61 | 1.86 | 4.97E-01 | -0.14 | 0.34 | 1.95E-02 | 0.47 | 0.82 |
| HILIC_NEG_199_978a0_726 | HILIC | NEG | 0.726 | 199.9780 | | | | 5.23E-15 | 1.04 | 1.75 | 3.48E-03 | 0.58 | 1.75 | 1.58E-10 | 1.34 | 2.04 | 2.82E-08 | 0.75 | 1.85 |
| RP_NEG_952_79413a0_49 | RP | NEG | 0.49 | 952.7941 | | | | 2.19E-05 | -0.64 | 1.09 | 3.52E-03 | 0.61 | 1.74 | 7.07E-02 | -0.33 | 0.65 | 1.91E-07 | -0.95 | 1.59 |
| HILIC_POS_104_57923a1_471 | HILIC | POS | 1.471 | 104.5792 | | | | 6.05E-01 | 0.10 | 0.37 | 3.53E-03 | -0.59 | 1.69 | 3.33E-01 | -0.19 | 0.52 | 1.02E-01 | 0.40 | 0.66 |
| RP_POS_676_48987a11_909 | RP | POS | 11.91 | 676.4899 | | | | 1.50E-05 | 0.54 | 1.36 | 3.55E-03 | 0.65 | 1.90 | 1.24E-04 | 0.87 | 1.33 | 5.69E-03 | 0.22 | 1.00 |
| HILIC_NEG_128_92851a0_927 | HILIC | NEG | 0.927 | 128.9285 | | | | 1.03E-03 | -0.68 | 1.32 | 3.56E-03 | -0.55 | 1.62 | 3.93E-05 | -0.95 | 1.56 | 6.35E-02 | -0.40 | 0.75 |
| RP_POS_252_06531a0_782 | RP | POS | 0.78 | 252.0653 | | | | 2.03E-03 | 0.38 | 1.07 | 3.64E-03 | 0.65 | 1.78 | 5.55E-04 | 0.71 | 1.11 | 6.19E-01 | 0.06 | 0.44 |
| RP_POS_1032_76379a0_491 | RP | POS | 0.49 | 1032.7638 | | | | 6.81E-02 | -0.30 | 0.59 | 3.67E-03 | 0.62 | 1.74 | 9.78E-01 | 0.01 | 0.15 | 7.44E-04 | -0.61 | 1.21 |
| HILIC_NEG_323_01358a0_929 | HILIC | NEG | 0.929 | 323.0136 | | | | 2.38E-02 | -0.43 | 1.09 | 3.68E-03 | -0.58 | 1.64 | 1.01E-03 | -0.72 | 1.31 | 5.05E-01 | -0.14 | 0.42 |
| HILIC_POS_467_3479a1_021 | HILIC | POS | 1.021 | 467.3479 | | | | 1.02E-02 | 0.35 | 0.83 | 3.70E-03 | 0.65 | 1.73 | 2.87E-03 | 0.68 | 0.93 | 7.58E-01 | 0.03 | 0.14 |
| RP_POS_798_02966a13_107 | RP | POS | 13.11 | 798.0297 | | | | 2.94E-03 | 0.41 | 0.98 | 3.71E-03 | 0.63 | 1.76 | 8.38E-05 | 0.73 | 1.36 | 5.58E-01 | 0.10 | 0.78 |
| RP_POS_754_53717a12_325 | RP | POS | 12.33 | 754.5372 | | | | 6.14E-04 | 0.50 | 1.07 | 3.81E-03 | 0.63 | 1.76 | 2.51E-04 | 0.81 | 1.24 | 1.45E-01 | 0.19 | 0.70 |
| HILIC_NEG_257_10077a0_721 | HILIC | NEG | 0.721 | 257.1008 | | | | 2.24E-05 | 0.50 | 1.21 | 3.81E-03 | 0.65 | 1.87 | 1.68E-04 | 0.83 | 1.16 | 2.43E-02 | 0.18 | 0.68 |
| HILIC_NEG_206_9729a5_429 | HILIC | NEG | 5.429 | 206.9729 | | | | 8.81E-02 | -0.27 | 0.55 | 3.82E-03 | -0.62 | 1.61 | 4.75E-03 | -0.58 | 0.96 | 8.02E-01 | 0.04 | 0.53 |
| RP_NEG_181_05008a3_115 | RP | NEG | 3.12 | 181.0501 | | | | 3.98E-03 | 0.50 | 0.80 | 3.91E-03 | 0.59 | 1.70 | 3.26E-04 | 0.80 | 1.20 | 2.39E-01 | 0.21 | 0.51 |
| RP_NEG_195_1024a7_839 | RP | NEG | 7.84 | 195.1024 | | | | 2.07E-02 | -0.50 | 1.20 | 4.00E-03 | -0.54 | 1.71 | 8.46E-04 | -0.77 | 1.42 | 3.31E-01 | -0.23 | 0.89 |
| RP_POS_168_98039a11_211 | RP | POS | 11.21 | 168.9804 | | | | 4.89E-02 | -0.40 | 0.81 | 4.00E-03 | -0.57 | 1.71 | 4.46E-03 | -0.68 | 1.09 | 5.82E-01 | -0.11 | 0.78 |
| RP_POS_770_60443a13_97 | RP | POS | 13.97 | 770.6044 | | | | 4.62E-01 | -0.15 | 0.53 | 4.01E-03 | -0.58 | 1.75 | 4.43E-02 | -0.43 | 0.81 | 5.34E-01 | 0.14 | 0.43 |
| RP_POS_626_35352a8_826 | RP | POS | 8.83 | 626.3535 | | | | 4.20E-02 | 0.34 | 0.64 | 4.02E-03 | 0.61 | 1.69 | 5.06E-03 | 0.65 | 0.98 | 7.96E-01 | 0.04 | 0.50 |
| RP_POS_350_30484a11_274 | RP | POS | 11.27 | 350.3048 | | | | 1.68E-10 | 0.90 | 1.77 | 4.09E-03 | 0.59 | 1.73 | 1.19E-08 | 1.19 | 1.90 | 2.12E-05 | 0.60 | 1.60 |
| RP_POS_245_07646a0_708 | RP | POS | 0.71 | 245.0765 | | | | 5.85E-02 | 0.29 | 0.51 | 4.17E-03 | 0.62 | 1.70 | 4.29E-03 | 0.60 | 1.00 | 8.96E-01 | -0.02 | 0.69 |
| RP_NEG_918_5495a12_958 | RP | NEG | 12.96 | 918.5495 | | | | 3.30E-07 | -0.96 | 1.65 | 4.18E-03 | -0.54 | 1.71 | 1.96E-08 | -1.23 | 2.02 | 3.30E-04 | -0.69 | 1.38 |
| RP_POS_351_25034a10_53 | RP | POS | 10.53 | 351.2503 | | | | 4.15E-06 | 0.56 | 1.12 | 4.22E-03 | 0.63 | 1.84 | 3.03E-05 | 0.88 | 1.38 | 2.43E-02 | 0.25 | 0.95 |
| HILIC_POS_159_07652a6_376 | HILIC | POS | 6.376 | 159.0765 | | | | 1.00E-07 | 0.74 | 1.19 | 4.28E-03 | 0.60 | 1.68 | 5.69E-07 | 1.04 | 1.55 | 1.58E-03 | 0.44 | 1.04 |
| RP_NEG_162_8383a0_549 | RP | NEG | 0.55 | 162.8383 | | | | 1.50E-01 | -0.27 | 0.53 | 4.32E-03 | -0.58 | 1.82 | 9.29E-03 | -0.56 | 0.98 | 9.14E-01 | 0.02 | 0.47 |
| RP_POS_222_01857a1_586 | RP | POS | 1.59 | 222.0186 | | | | 5.23E-08 | 0.65 | 1.22 | 4.34E-03 | 0.63 | 1.73 | 3.73E-06 | 0.96 | 1.43 | 2.30E-03 | 0.34 | 0.97 |
| HILIC_NEG_341_00436a0_931 | HILIC | NEG | 0.931 | 341.0044 | | | | 2.45E-02 | -0.46 | 1.06 | 4.38E-03 | -0.55 | 1.62 | 1.05E-03 | -0.74 | 1.30 | 4.07E-01 | -0.19 | 0.50 |
| RP_POS_856_58228a13_294 | RP | POS | 13.29 | 856.5823 | | | | 2.86E-04 | 0.60 | 1.11 | 4.40E-03 | 0.59 | 1.67 | 1.00E-04 | 0.90 | 1.37 | 4.15E-02 | 0.30 | 0.84 |
| HILIC_NEG_142_92879a0_925 | HILIC | NEG | 0.925 | 142.9288 | | | | 1.25E-06 | -0.97 | 1.75 | 4.46E-03 | -0.52 | 1.58 | 2.49E-08 | -1.23 | 2.07 | 7.62E-04 | -0.71 | 1.42 |
| RP_POS_422_76144a12_957 | RP | POS | 12.96 | 422.7614 | | | | 1.17E-09 | -1.04 | 1.82 | 4.51E-03 | -0.54 | 1.72 | 1.30E-10 | -1.31 | 2.18 | 2.68E-05 | -0.77 | 1.54 |
| RP_NEG_1085_68042a10_512 | RP | NEG | 10.51 | 1085.6804 | | | | 1.60E-02 | 0.47 | 1.11 | 4.55E-03 | -0.57 | 1.66 | 3.96E-01 | 0.18 | 0.61 | 4.94E-04 | 0.75 | 1.36 |
| RP_POS_833_52869a12_953 | RP | POS | 12.95 | 833.5287 | | | | 1.93E-10 | -1.16 | 2.00 | 4.55E-03 | -0.51 | 1.73 | 1.00E-11 | -1.41 | 2.34 | 2.47E-06 | -0.90 | 1.66 |
| HILIC_NEG_141_92377a0_925 | HILIC | NEG | 0.925 | 141.9238 | | | | 1.83E-07 | -1.12 | 1.88 | 4.56E-03 | -0.49 | 1.66 | 5.89E-09 | -1.36 | 2.18 | 5.24E-05 | -0.88 | 1.54 |
| RP_POS_780_04431a13_025 | RP | POS | 13.03 | 780.0443 | | | | 1.54E-15 | 1.08 | 1.76 | 4.57E-03 | 0.56 | 1.69 | 5.72E-12 | 1.36 | 2.10 | 1.49E-07 | 0.80 | 1.57 |
| HILIC_NEG_174_83954a3_03 | HILIC | NEG | 3.03 | 174.8395 | | | | 6.48E-02 | -0.24 | 1.19 | 4.60E-03 | -0.63 | 1.54 | 3.45E-03 | -0.56 | 1.36 | 6.43E-01 | 0.07 | 1.00 |
| RP_NEG_383_15402a7_66 | RP | NEG | 7.66 | 383.1540 | | | | 1.70E-05 | 0.57 | 1.08 | 4.61E-03 | 0.62 | 1.78 | 6.21E-05 | 0.88 | 1.25 | 1.66E-02 | 0.26 | 0.82 |
| RP_POS_351_24969a10_403 | RP | POS | 10.40 | 351.2497 | | | | 1.51E-01 | 0.27 | 0.69 | 4.64E-03 | 0.58 | 1.74 | 1.44E-02 | 0.56 | 0.91 | 8.96E-01 | -0.02 | 0.56 |
| HILIC_NEG_134_89471a1_21 | HILIC | NEG | 1.21 | 134.8947 | | | | 1.42E-04 | 0.55 | 1.04 | 4.65E-03 | 0.61 | 1.60 | 6.31E-05 | 0.85 | 1.39 | 7.68E-02 | 0.25 | 0.83 |
| RP_POS_916_65485a11_15 | RP | POS | 11.15 | 916.6549 | | | | 7.69E-04 | 0.44 | 1.14 | 4.78E-03 | -0.62 | 1.81 | 2.89E-01 | 0.13 | 0.62 | 3.65E-04 | 0.75 | 1.35 |
| RP_NEG_239_09262a7_84 | RP | NEG | 7.84 | 239.0926 | | | | 1.99E-02 | -0.50 | 1.19 | 4.85E-03 | -0.53 | 1.68 | 8.56E-04 | -0.77 | 1.41 | 3.20E-01 | -0.24 | 0.89 |
| RP_POS_532_3736a11_171 | RP | POS | 11.17 | 532.3736 | | | | 8.59E-06 | -1.11 | 1.80 | 4.88E-03 | -0.44 | 1.78 | 3.51E-07 | -1.32 | 2.05 | 4.84E-04 | -0.89 | 1.48 |
| RP_POS_257_14951a5_025 | RP | POS | 5.03 | 257.1495 | | | | 4.43E-04 | 0.59 | 1.24 | 4.89E-03 | 0.58 | 1.74 | 3.63E-05 | 0.88 | 1.48 | 9.16E-02 | 0.30 | 0.84 |
| RP_NEG_193_81514a0_564 | RP | NEG | 0.56 | 193.8151 | | | | 2.84E-02 | -0.35 | 0.77 | 4.94E-03 | -0.60 | 1.83 | 1.01E-03 | -0.65 | 1.23 | 8.01E-01 | -0.05 | 0.72 |
| RP_POS_395_2417a12_981 | RP | POS | 12.98 | 395.2417 | | | | 5.53E-17 | 1.15 | 1.89 | 4.94E-03 | 0.54 | 1.65 | 8.43E-13 | 1.42 | 2.18 | 8.32E-09 | 0.88 | 1.84 |
| HILIC_NEG_286_01492a0_928 | HILIC | NEG | 0.928 | 286.0149 | | | | 1.30E-08 | 0.69 | 1.35 | 5.06E-03 | 0.61 | 1.85 | 1.80E-06 | 1.00 | 1.46 | 7.73E-04 | 0.39 | 1.05 |
| RP_POS_764_55731a12_946 | RP | POS | 12.95 | 764.5573 | | | | 2.13E-01 | 0.23 | 1.14 | 5.07E-03 | -0.58 | 1.64 | 7.73E-01 | -0.06 | 0.52 | 2.01E-02 | 0.52 | 1.13 |
| RP_POS_878_58411a14_752 | RP | POS | 14.75 | 878.5841 | | | | 3.69E-03 | 0.50 | 0.98 | 5.14E-03 | 0.58 | 1.68 | 4.64E-04 | 0.79 | 1.15 | 2.20E-01 | 0.20 | 0.52 |
| RP_POS_490_15332a4_587 | RP | POS | 4.59 | 490.1533 | | | | 8.01E-01 | -0.04 | 0.69 | 5.16E-03 | 0.61 | 1.64 | 2.48E-01 | 0.26 | 0.63 | 1.56E-02 | -0.34 | 1.16 |
| RP_POS_292_64673a10_374 | RP | POS | 10.37 | 292.6467 | | | | 5.71E-01 | 0.11 | 0.97 | 5.18E-03 | -0.57 | 1.83 | 4.15E-01 | -0.18 | 0.75 | 6.19E-02 | 0.39 | 0.99 |
| RP_POS_381_27661a11_41 | RP | POS | 11.41 | 381.2766 | | | | 6.74E-08 | 0.69 | 1.49 | 5.20E-03 | 0.61 | 1.64 | 8.65E-06 | 0.99 | 1.51 | 5.20E-05 | 0.39 | 1.53 |
| RP_NEG_203_0202a0_525 | RP | NEG | 0.53 | 203.0202 | | | | 5.86E-01 | -0.08 | 0.65 | 5.22E-03 | -0.61 | 1.64 | 4.35E-02 | -0.39 | 0.77 | 2.15E-01 | 0.22 | 0.78 |
| RP_NEG_307_0806a7_839 | RP | NEG | 7.84 | 307.0806 | | | | 1.77E-02 | -0.51 | 1.19 | 5.24E-03 | -0.53 | 1.65 | 7.40E-04 | -0.78 | 1.42 | 2.99E-01 | -0.25 | 0.87 |
| RP_POS_546_35522a10_396 | RP | POS | 10.40 | 546.3552 | | | | 7.28E-03 | 0.52 | 1.16 | 5.27E-03 | -0.55 | 1.65 | 2.57E-01 | 0.25 | 0.71 | 2.07E-04 | 0.80 | 1.40 |
| HILIC_NEG_315_04214a5_429 | HILIC | NEG | 5.429 | 315.0421 | | | | 5.05E-01 | 0.15 | 0.72 | 5.32E-03 | 0.53 | 1.75 | 9.46E-02 | 0.41 | 0.67 | 6.19E-01 | -0.12 | 0.33 |
| RP_POS_219_17447a10_407 | RP | POS | 10.41 | 219.1745 | | | | 2.66E-01 | -0.23 | 0.61 | 5.35E-03 | 0.55 | 1.61 | 8.43E-01 | 0.05 | 0.33 | 2.55E-02 | -0.50 | 0.82 |
| RP_NEG_556_29852a10_328 | RP | NEG | 10.33 | 556.2985 | | | | 9.07E-03 | -0.62 | 1.25 | 5.37E-03 | -0.49 | 1.70 | 9.59E-04 | -0.86 | 1.37 | 1.19E-01 | -0.37 | 0.92 |
| RP_POS_616_85449a0_493 | RP | POS | 0.49 | 616.8545 | | | | 1.91E-01 | -0.21 | 0.58 | 5.39E-03 | 0.60 | 1.71 | 6.54E-01 | 0.09 | 0.38 | 2.04E-03 | -0.51 | 1.13 |
| RP_POS_811_57617a11_162 | RP | POS | 11.16 | 811.5762 | | | | 9.33E-03 | 0.31 | 0.95 | 5.41E-03 | -0.63 | 1.75 | 9.48E-01 | -0.01 | 0.70 | 1.96E-03 | 0.62 | 1.25 |
| RP_POS_742_53693a13_288 | RP | POS | 13.29 | 742.5369 | | | | 5.71E-01 | 0.10 | 0.30 | 5.47E-03 | 0.58 | 1.68 | 1.01E-01 | 0.39 | 0.60 | 2.75E-01 | -0.19 | 0.53 |
| RP_POS_478_38901a15_435 | RP | POS | 15.44 | 478.3890 | | | | 7.09E-01 | -0.09 | 0.39 | 5.53E-03 | -0.51 | 1.77 | 1.55E-01 | -0.34 | 0.65 | 5.32E-01 | 0.17 | 0.40 |
| HILIC_POS_202_08626a0_5 | HILIC | POS | 0.5 | 202.0863 | | | | 1.32E-01 | 0.25 | 0.52 | 5.55E-03 | -0.59 | 1.48 | 7.70E-01 | -0.05 | 0.11 | 9.94E-03 | 0.54 | 0.85 |
| HILIC_NEG_127_05118a6_093 | HILIC | NEG | 6.093 | 127.0512 | | | | 3.12E-01 | -0.16 | 0.93 | 5.56E-03 | -0.60 | 1.52 | 2.07E-02 | -0.46 | 0.95 | 4.52E-01 | 0.14 | 1.11 |
| HILIC_NEG_337_02905a0_929 | HILIC | NEG | 0.929 | 337.0291 | | | | 1.18E-01 | -0.31 | 0.91 | 5.64E-03 | -0.55 | 1.60 | 9.32E-03 | -0.58 | 1.04 | 8.87E-01 | -0.03 | 0.27 |
| RP_POS_208_00293a1_583 | RP | POS | 1.58 | 208.0029 | | | | 1.12E-07 | 0.63 | 1.22 | 5.65E-03 | 0.61 | 1.68 | 7.63E-06 | 0.94 | 1.40 | 2.40E-03 | 0.33 | 0.97 |
| RP_POS_801_98657a10_326 | RP | POS | 10.33 | 801.9866 | | | | 5.29E-04 | -0.84 | 1.50 | 5.76E-03 | -0.46 | 1.72 | 4.31E-05 | -1.07 | 1.69 | 1.25E-02 | -0.61 | 1.17 |
| RP_POS_584_29944a10_296 | RP | POS | 10.30 | 584.2994 | | | | 5.12E-01 | 0.13 | 0.78 | 5.78E-03 | -0.56 | 1.72 | 4.86E-01 | -0.15 | 0.51 | 6.35E-02 | 0.41 | 0.98 |
| RP_NEG_261_97763a2_998 | RP | NEG | 3.00 | 261.9776 | | | | 6.62E-14 | 0.94 | 1.53 | 5.91E-03 | 0.57 | 1.62 | 1.71E-09 | 1.23 | 1.86 | 1.21E-06 | 0.66 | 1.47 |
| HILIC_NEG_157_03674a1_818 | HILIC | NEG | 1.818 | 157.0367 | | | | 3.86E-03 | 0.46 | 0.85 | 5.95E-03 | 0.59 | 1.66 | 4.96E-04 | 0.75 | 1.19 | 3.06E-01 | 0.16 | 0.60 |

| Feature_ID | Healthy_control Mean | Healthy_control SD | all_ALD Mean | all_ALD SD | Non-prog_ALD Mean | Non-prog_ALD SD | Progressive_ALD Mean | Progressive_ALD SD |
|---|---|---|---|---|---|---|---|---|
| RP_POS_519_32245a10_375 | 15052620 | 2867150 | 15827101 | 3177835 | 16761276 | 2770446 | 14892926 | 3308626 |
| RP_POS_445_36655a12_428 | 6252891 | 1338067 | 5872964 | 1402339 | 6285207 | 1194348 | 5460722 | 1483224 |
| RP_POS_478_32712a10_505 | 1257216 | 402610 | 1209919 | 432013 | 1336626 | 440428 | 1083213 | 387735 |
| RP_NEG_261_0748a7_839 | 730735 | 530472 | 472931 | 490652 | 616863 | 522241 | 328999 | 413978 |
| HILIC_POS_151_04778a6_129 | 1476248 | 281309 | 1335620 | 435374 | 1463259 | 349479 | 1207981 | 477060 |
| HILIC_NEG_181_05098a1_076 | 284045 | 113125 | 371364 | 190577 | 315309 | 123009 | 427419 | 227677 |
| RP_NEG_195_81055a0_565 | 81377646 | 10831710 | 69507558 | 15589848 | 74056318 | 13810867 | 64958799 | 16064863 |
| RP_POS_497_39624a13_923 | 4398990 | 1346618 | 4748010 | 1048139 | 5054376 | 825854 | 4441643 | 1160183 |
| HILIC_POS_152_03162a6_129 | 1030442 | 183954 | 930172 | 300620 | 1017822 | 234591 | 842523 | 334482 |
| HILIC_NEG_157_90067a0_943 | 825296 | 171984 | 1096923 | 343854 | 996678 | 262174 | 1197168 | 386834 |
| RP_POS_267_19305a9_053 | 884569 | 112865 | 1119503 | 363510 | 1012463 | 170541 | 1226543 | 463150 |
| RP_POS_329_16251a10_822 | 227644 | 219817 | 508566 | 295522 | 422462 | 222979 | 594671 | 334064 |
| HILIC_NEG_118_92419a0_925 | 3313377 | 629625 | 2638906 | 655681 | 2829272 | 622494 | 2448541 | 638085 |
| RP_POS_222_01859a1_361 | 75641 | 42281 | 1250019 | 1867437 | 704335 | 1225569 | 1795703 | 2221732 |
| RP_POS_593_33289a8_601 | 162134 | 215465 | 573996 | 1077675 | 255979 | 366150 | 892014 | 1416335 |
| RP_POS_781_05286a12_965 | 1621498 | 848078 | 3888936 | 1553701 | 3438314 | 1284876 | 4339557 | 1676880 |
| RP_POS_550_39172a11_282 | 257110 | 142225 | 435340 | 393409 | 320417 | 227777 | 550264 | 483595 |
| RP_NEG_650_35107a13_182 | 4254238 | 643931 | 4193936 | 580613 | 4361873 | 567666 | 4025999 | 548908 |
| HILIC_NEG_273_97845a0_729 | 150221 | 76587 | 386199 | 247511 | 314464 | 190459 | 457935 | 277483 |
| HILIC_NEG_117_05566a0_925 | 272594 | 233814 | 1627924 | 2580653 | 874285 | 1307252 | 2381563 | 3253832 |
| HILIC_NEG_165_0192a0_927 | 1069857 | 399687 | 1151425 | 527574 | 1303567 | 479433 | 999284 | 534092 |
| HILIC_NEG_199_978a0_726 | 1030219 | 502997 | 2560853 | 1480413 | 2132664 | 1087188 | 2989042 | 1694644 |
| RP_NEG_952_79413a0_49 | 941871 | 210808 | 695864 | 408747 | 578182 | 365490 | 813545 | 419180 |
| HILIC_POS_104_57923a1_471 | 564304 | 324001 | 599016 | 343129 | 698296 | 404800 | 499736 | 232164 |
| RP_POS_676_48987a11_909 | 211310 | 252051 | 725270 | 1050239 | 418468 | 407943 | 1032071 | 1367480 |
| HILIC_NEG_128_92851a0_927 | 1037518 | 210685 | 888576 | 211988 | 949511 | 198031 | 827641 | 209765 |
| RP_POS_252_06531a0_782 | 364301 | 165279 | 606749 | 711470 | 401544 | 484255 | 811954 | 838016 |
| RP_POS_1032_76379a0_491 | 357915 | 147948 | 293850 | 227296 | 228587 | 182849 | 359113 | 249510 |
| HILIC_NEG_323_01358a0_929 | 635202 | 99098 | 586561 | 115425 | 619627 | 107738 | 553494 | 114362 |
| HILIC_POS_467_3479a1_021 | 351827 | 959461 | 1121531 | 2414269 | 419772 | 985723 | 1823290 | 3130441 |
| RP_POS_798_02966a13_107 | 543187 | 128338 | 660822 | 314230 | 570890 | 291850 | 750754 | 312753 |
| RP_POS_754_53717a12_325 | 1292226 | 861356 | 2129362 | 1822053 | 1604456 | 1044737 | 2654269 | 2248050 |
| HILIC_NEG_257_10077a0_721 | 5944 | 10017 | 170102 | 368459 | 63595 | 175029 | 276610 | 469583 |
| HILIC_NEG_206_9729a5_429 | 517310 | 29052 | 504923 | 50262 | 519283 | 42066 | 490564 | 53979 |
| RP_NEG_181_05008a3_115 | 2355798 | 978333 | 3007592 | 1344196 | 2623783 | 1022398 | 3391402 | 1518114 |
| RP_NEG_195_1024a7_839 | 1073999 | 831040 | 679780 | 746434 | 892011 | 802558 | 467549 | 624240 |
| RP_POS_168_98039a11_211 | 5412281 | 476684 | 5215146 | 497485 | 5356803 | 384195 | 5073488 | 558100 |
| RP_POS_770_60443a13_97 | 3067138 | 1518755 | 2835429 | 1602384 | 3290541 | 1664396 | 2380318 | 1412355 |
| RP_POS_626_35352a8_826 | 257845 | 493427 | 491989 | 721805 | 285300 | 421940 | 698677 | 887367 |
| RP_POS_350_30484a11_274 | 200983 | 125844 | 463033 | 302433 | 377045 | 225835 | 549021 | 344540 |
| RP_POS_245_07646a0_708 | 2310812 | 358320 | 2486438 | 663593 | 2298268 | 505412 | 2674607 | 749664 |
| RP_NEG_918_5495a12_958 | 6035673 | 948468 | 4875347 | 1144921 | 5199124 | 1023132 | 4551570 | 1178007 |
| RP_POS_351_25034a10_53 | 369196 | 193089 | 943823 | 1138125 | 620276 | 728895 | 1267371 | 1368271 |
| HILIC_POS_159_07652a6_376 | 1951951 | 647040 | 3056579 | 1579998 | 2609450 | 1167039 | 3503708 | 1809349 |
| RP_NEG_162_8383a0_549 | 6731821 | 1741835 | 6189161 | 2086685 | 6776860 | 1970218 | 5601463 | 2052007 |
| RP_POS_222_01857a1_586 | 212016 | 100851 | 1977322 | 2991534 | 1130279 | 2015379 | 2824365 | 3543330 |
| HILIC_NEG_341_00436a0_931 | 632147 | 120282 | 575657 | 119534 | 609284 | 122115 | 542030 | 107929 |
| RP_POS_856_58228a13_294 | 1110551 | 731112 | 1763781 | 1140613 | 1440114 | 645243 | 2087448 | 1413652 |
| HILIC_NEG_142_92879a0_925 | 2186885 | 392643 | 1745530 | 420971 | 1863730 | 424704 | 1627330 | 386217 |
| RP_POS_422_76144a12_957 | 3701185 | 531714 | 2854025 | 785606 | 3074307 | 738813 | 2633743 | 775936 |
| RP_NEG_1085_68042a10_512 | 2246321 | 737805 | 2629715 | 829102 | 2862031 | 750156 | 2397400 | 846150 |
| RP_POS_833_52869a12_953 | 2085860 | 485333 | 1287635 | 633515 | 1465069 | 612002 | 1110200 | 609980 |
| HILIC_NEG_141_92377a0_925 | 187964 | 63884 | 110535 | 59941 | 127322 | 57821 | 93749 | 57800 |
| RP_POS_780_04431a13_025 | 244604 | 192900 | 813603 | 524076 | 666763 | 452092 | 960444 | 553647 |
| HILIC_NEG_174_83954a3_03 | 2363532 | 84990 | 2309727 | 246752 | 2378845 | 206722 | 2240609 | 265579 |
| RP_NEG_383_15402a7_66 | 524006 | 379819 | 1104922 | 1113005 | 790447 | 606040 | 1419398 | 1390587 |
| RP_POS_351_24969a10_403 | 1284557 | 329885 | 1387469 | 401831 | 1274885 | 316258 | 1500053 | 447600 |
| HILIC_NEG_134_89471a1_21 | 4220295 | 835953 | 5138692 | 1803532 | 4632059 | 1243516 | 5645326 | 2121715 |
| RP_POS_916_65485a11_15 | 373636 | 73050 | 464321 | 229365 | 528635 | 275093 | 400008 | 148905 |
| RP_NEG_239_09262a7_84 | 23778995 | 18113508 | 15105789 | 16505336 | 19702639 | 17714357 | 10508939 | 13910031 |
| RP_POS_532_3736a11_171 | 1316804 | 535851 | 800106 | 365087 | 901968 | 419670 | 698245 | 268326 |
| RP_POS_257_14951a5_025 | 299340 | 423653 | 662000 | 646998 | 481966 | 539383 | 842033 | 699053 |
| RP_NEG_193_81514a0_564 | 4455357 | 582734 | 4135566 | 999917 | 4413182 | 929064 | 3857950 | 999812 |
| RP_POS_395_2417a12_981 | 193302 | 180387 | 738757 | 461585 | 610500 | 393816 | 867013 | 491739 |
| HILIC_NEG_286_01492a0_928 | 56984 | 61914 | 346391 | 457750 | 218990 | 312290 | 473792 | 541014 |
| RP_POS_764_55731a12_946 | 2677401 | 1334458 | 3037331 | 1616750 | 3485363 | 1734997 | 2589299 | 1364271 |
| RP_POS_878_58411a14_752 | 1244171 | 993906 | 1925077 | 1441047 | 1524917 | 1015700 | 2325237 | 1684391 |
| RP_POS_490_15332a4_587 | 19661702 | 5764448 | 19307739 | 9627107 | 16617066 | 4753230 | 21998413 | 12242782 |
| RP_POS_292_64673a10_374 | 2365060 | 345746 | 2406598 | 398889 | 2516775 | 364113 | 2296421 | 405118 |
| RP_POS_381_27661a11_41 | 356792 | 272434 | 994485 | 1002828 | 714362 | 482707 | 1274609 | 1280119 |
| RP_NEG_203_0202a0_525 | 1205407 | 199618 | 1177313 | 374050 | 1280530 | 344217 | 1074095 | 377431 |
| RP_NEG_307_0806a7_839 | 1051182 | 758842 | 680287 | 693980 | 871955 | 750305 | 488618 | 578891 |
| RP_POS_546_35522a10_396 | 8829750 | 3648121 | 10950161 | 4059378 | 12069473 | 3632932 | 9830848 | 4187880 |
| HILIC_NEG_315_04214a5_429 | 9734853 | 1826115 | 9975672 | 1562687 | 9545245 | 1398780 | 10406098 | 1612184 |
| RP_POS_219_17447a10_407 | 1496251 | 119509 | 1469006 | 118531 | 1436385 | 109009 | 1501627 | 119713 |
| RP_NEG_556_29852a10_328 | 1244444 | 231933 | 1122368 | 177407 | 1171221 | 149654 | 1073514 | 190582 |
| RP_POS_616_85449a0_493 | 1719770 | 321552 | 1615507 | 551467 | 1463283 | 400719 | 1767731 | 637630 |
| RP_POS_811_57617a11_162 | 617096 | 44400 | 729402 | 416763 | 844466 | 488818 | 614337 | 291487 |
| RP_POS_742_53693a13_288 | 912198 | 858089 | 1017837 | 1069867 | 721351 | 585178 | 1314323 | 1338194 |
| RP_POS_478_38901a15_435 | 10341445 | 4736219 | 9999366 | 3571702 | 10982121 | 4105146 | 9016611 | 2636513 |
| HILIC_POS_202_08626a0_5 | 616719 | 403976 | 762711 | 642885 | 939132 | 699986 | 586289 | 530599 |
| HILIC_NEG_127_05118a6_093 | 426936 | 92607 | 404063 | 154517 | 446404 | 140817 | 361722 | 157322 |
| HILIC_NEG_337_02905a0_929 | 356204 | 71708 | 332601 | 77819 | 353887 | 72476 | 311315 | 77831 |
| RP_POS_208_00293a1_583 | 169726 | 55927 | 1289309 | 1954602 | 751492 | 1283086 | 1827125 | 2341430 |
| RP_POS_801_98657a10_326 | 2365960 | 787885 | 1800905 | 569895 | 1956455 | 525249 | 1645355 | 575260 |
| RP_POS_584_29944a10_296 | 323110 | 149876 | 343679 | 164884 | 388652 | 158997 | 298706 | 159751 |
| RP_NEG_261_97763a2_998 | 608091 | 386751 | 2290937 | 1862742 | 1782852 | 1458767 | 2799022 | 2087004 |
| HILIC_NEG_157_03674a1_818 | 308954 | 106512 | 386984 | 183896 | 336822 | 137257 | 437145 | 210597 |

**Supplementary table 1: Metabolomics results** (in asceding order according to p-value from progressive ALD vs  non-progressive ALD comparison, with molecular features with ID_level of 1-2 first)

| | | | | | | | | all_ALD_vs_control | | | Prog_ALD_vs_non-prog_ALD | | | Prog_ALD_vs_control_ALD | | | Non-prog_ALD_vs_control | | | Healthy_control | | all_ALD | | Non-prog_ALD | | Progressive_ALD | |
|---|---|---|---|---|---|---|---|---|---|---|---|---|---|---|---|---|---|---|---|---|---|---|---|---|---|---|---|
| | | | | | | | | t-test | Cohen's | PLS-DA | t-test | Cohen's | PLS-DA | t-test | Cohen's | PLS-DA | t-test | Cohen's | PLS-DA | | | | | | | | |
| Feature_ID | RP_HILIC | POS_NEG | Average.Rt | Average.Mz | Metabolite.name | curated_MSMS | ID_level | p | d | VIP | p | d | VIP | p | d | VIP | p | d | VIP | Mean | SD | Mean | SD | Mean | SD | Mean | SD |
| HILIC_NEG_226_99448a0_929 | HILIC | NEG | 0.929 | 226.9945 | | | | 6.45E-02 | -0.38 | 1.02 | 6.02E-03 | -0.54 | 1.60 | 3.84E-03 | -0.65 | 1.14 | 6.27E-01 | -0.11 | 0.38 | 1323802 | 252943 | 1226761 | 253279 | 1295550 | 260254 | 1157972 | 228517 |
| RP_POS_345_17273a10_821 | RP | POS | 10.82 | 345.1727 | | | | 3.83E-09 | 1.02 | 1.89 | 6.04E-03 | 0.52 | 1.85 | 4.06E-09 | 1.28 | 2.03 | 9.94E-06 | 0.76 | 1.76 | 1422353 | 1076885 | 3058951 | 1550650 | 2637006 | 1203466 | 3480895 | 1745403 |
| RP_POS_191_0004a1_358 | RP | POS | 1.36 | 191.0004 | | | | 4.71E-08 | 0.65 | 1.20 | 6.08E-03 | 0.60 | 1.68 | 5.70E-06 | 0.95 | 1.42 | 1.13E-03 | 0.35 | 1.05 | 38571 | 15964 | 1100056 | 1793701 | 610655 | 1169403 | 1589457 | 2155012 |
| RP_POS_930_78479a0_49 | RP | POS | 0.49 | 930.7848 | | | | 8.15E-06 | -0.71 | 1.15 | 6.13E-03 | 0.57 | 1.65 | 2.56E-02 | -0.42 | 0.77 | 1.20E-07 | -0.99 | 1.62 | 820052 | 199780 | 586825 | 344433 | 493461 | 308938 | 680190 | 355609 |
| RP_NEG_197_80746a0_564 | RP | NEG | 0.56 | 197.8075 | | | | 8.72E-06 | -0.74 | 1.16 | 6.17E-03 | -0.56 | 1.78 | 1.61E-06 | -1.02 | 1.57 | 7.33E-03 | -0.46 | 0.99 | 105387688 | 13311902 | 90586550 | 20438971 | 96128107 | 16969485 | 85044993 | 22212474 |
| RP_POS_566_41052a11_538 | RP | POS | 11.54 | 566.4105 | | | | 1.57E-07 | -1.46 | 2.54 | 6.19E-03 | -0.31 | 1.62 | 2.09E-08 | -1.62 | 2.36 | 1.50E-06 | -1.31 | 2.17 | 1419913 | 724154 | 558200 | 335164 | 648910 | 328360 | 467489 | 319921 |
| HILIC_NEG_394_05457a6_093 | HILIC | NEG | 6.093 | 394.0546 | | | | 1.51E-01 | -0.22 | 0.96 | 6.20E-03 | -0.59 | 1.49 | 1.12E-02 | -0.52 | 0.99 | 6.61E-01 | 0.07 | 1.09 | 147755 | 31628 | 136250 | 56617 | 151591 | 47155 | 120909 | 61421 |
| RP_POS_340_16956a10_822 | RP | POS | 10.82 | 340.1696 | | | | 1.44E-09 | 1.05 | 1.93 | 6.20E-03 | 0.52 | 1.83 | 1.46E-09 | 1.31 | 2.07 | 4.37E-06 | 0.79 | 1.78 | 1476986 | 1112626 | 3219745 | 1579912 | 2791269 | 1248613 | 3648220 | 1763371 |
| RP_POS_190_99989a1_584 | RP | POS | 1.58 | 190.9999 | | | | 1.40E-07 | 0.63 | 1.18 | 6.25E-03 | 0.60 | 1.66 | 6.01E-06 | 0.93 | 1.40 | 4.89E-03 | 0.33 | 0.93 | 53318 | 29491 | 1313627 | 2221543 | 710404 | 1575756 | 1916850 | 2597230 |
| RP_POS_389_33591a10_383 | RP | POS | 10.38 | 389.3359 | | | | 6.79E-02 | -0.35 | 0.95 | 6.26E-03 | 0.56 | 1.65 | 7.57E-01 | -0.07 | 0.59 | 2.63E-03 | -0.62 | 1.20 | 2083456 | 315313 | 1959140 | 369287 | 1859239 | 319501 | 2059040 | 391255 |
| RP_POS_343_1777a10_821 | RP | POS | 10.82 | 343.1777 | | | | 8.06E-09 | 1.01 | 1.88 | 6.30E-03 | 0.52 | 1.83 | 6.03E-09 | 1.27 | 2.02 | 1.66E-05 | 0.75 | 1.73 | 4061285 | 3094324 | 8613808 | 4352747 | 7435457 | 3418597 | 9792158 | 4874318 |
| HILIC_POS_597_36407a0_504 | HILIC | POS | 0.504 | 597.3641 | | | | 4.02E-02 | 0.31 | 0.83 | 6.31E-03 | 0.60 | 1.47 | 6.66E-03 | 0.61 | 1.06 | 9.24E-01 | 0.01 | 0.05 | 342972 | 803770 | 776281 | 1525046 | 360858 | 863452 | 1191705 | 1897696 |
| RP_POS_621_44031a10_832 | RP | POS | 10.83 | 621.4403 | | | | 3.64E-10 | 1.20 | 2.07 | 6.33E-03 | 0.48 | 1.73 | 1.97E-10 | 1.44 | 2.13 | 1.95E-07 | 0.96 | 2.00 | 299039 | 162129 | 556278 | 190825 | 504591 | 141139 | 607965 | 219543 |
| RP_POS_355_26041a11_273 | RP | POS | 11.27 | 355.2604 | | | | 7.98E-10 | 0.86 | 1.75 | 6.40E-03 | 0.56 | 1.70 | 8.68E-08 | 1.14 | 1.83 | 2.15E-05 | 0.57 | 1.67 | 1978773 | 1381941 | 4760048 | 3382804 | 3843849 | 2353950 | 5676246 | 3983225 |
| RP_POS_829_55212a12_957 | RP | POS | 12.96 | 829.5521 | | | | 1.29E-09 | -1.08 | 1.94 | 6.44E-03 | -0.51 | 1.65 | 1.31E-10 | -1.33 | 2.24 | 1.09E-05 | -0.83 | 1.61 | 52700672 | 7731029 | 40672440 | 10473719 | 43494239 | 9886666 | 37850641 | 10374972 |
| HILIC_NEG_229_98845a0_727 | HILIC | NEG | 0.727 | 229.9885 | | | | 1.36E-13 | 0.98 | 1.68 | 6.54E-03 | 0.55 | 1.62 | 9.26E-10 | 1.26 | 1.96 | 4.12E-07 | 0.71 | 1.69 | 842204 | 423032 | 2059390 | 1267019 | 1717789 | 977495 | 2400992 | 1431709 |
| RP_POS_251_04604a10_284 | RP | POS | 10.28 | 251.0460 | | | | 3.37E-01 | -0.20 | 0.68 | 6.59E-03 | 0.54 | 1.60 | 7.66E-01 | 0.07 | 0.46 | 3.09E-02 | -0.47 | 0.95 | 1003287 | 64667 | 990578 | 64090 | 973325 | 51143 | 1007832 | 71245 |
| RP_POS_223_09645a7_844 | RP | POS | 7.84 | 223.0965 | Unknown | 223.0948(100) | 4 | 6.23E-03 | -0.60 | 1.30 | 6.66E-03 | -0.50 | 1.61 | 3.12E-04 | -0.85 | 1.49 | 1.40E-01 | -0.35 | 0.99 | 27130613 | 12457396 | 20082777 | 11010406 | 23038904 | 11479800 | 17126649 | 9762800 |
| HILIC_NEG_162_98238a0_927 | HILIC | NEG | 0.927 | 162.9824 | | | | 9.20E-02 | -0.34 | 0.97 | 6.70E-03 | -0.54 | 1.56 | 5.68E-03 | -0.61 | 1.10 | 7.66E-01 | -0.07 | 0.41 | 657789 | 123524 | 614173 | 130398 | 649153 | 134198 | 579194 | 117679 |
| RP_POS_311_15125a10_822 | RP | POS | 10.82 | 311.1513 | | | | 2.32E-09 | 1.03 | 1.89 | 6.75E-03 | 0.52 | 1.83 | 3.07E-09 | 1.28 | 2.04 | 6.94E-06 | 0.77 | 1.74 | 1254997 | 969944 | 2759894 | 1414479 | 2380046 | 1106103 | 3139742 | 1588839 |
| HILIC_POS_169_05852a6_129 | HILIC | POS | 6.129 | 169.0585 | | | | 7.90E-02 | -0.30 | 1.04 | 6.78E-03 | -0.57 | 1.49 | 5.91E-03 | -0.58 | 1.05 | 9.52E-01 | -0.01 | 0.86 | 21744044 | 4745749 | 19801250 | 6981927 | 21672636 | 5965768 | 17929865 | 7466723 |
| HILIC_POS_103_63889a1_471 | HILIC | POS | 1.471 | 103.6389 | | | | 6.10E-01 | 0.10 | 0.29 | 6.78E-03 | -0.54 | 1.58 | 3.95E-01 | -0.17 | 0.55 | 1.24E-01 | 0.37 | 0.61 | 585514 | 326288 | 619833 | 339986 | 711302 | 401431 | 528363 | 235200 |
| HILIC_POS_326_03983a2_981 | HILIC | POS | 2.981 | 326.0398 | | | | 7.87E-10 | 0.93 | 1.60 | 6.80E-03 | 0.54 | 1.67 | 2.43E-08 | 1.20 | 1.78 | 6.77E-06 | 0.66 | 1.50 | 841652 | 380565 | 1515751 | 732649 | 1318734 | 512202 | 1712767 | 861938 |
| RP_POS_122_05782a1_855 | RP | POS | 1.86 | 122.0578 | | | | 8.44E-07 | 0.59 | 0.97 | 6.86E-03 | 0.60 | 1.60 | 1.43E-05 | 0.89 | 1.44 | 1.26E-02 | 0.29 | 0.75 | 111468 | 177108 | 1047211 | 1759163 | 574797 | 1248680 | 1519626 | 2058592 |
| RP_POS_1061_65332a10_324 | RP | POS | 10.32 | 1061.6533 | | | | 3.96E-03 | -0.71 | 1.37 | 6.88E-03 | -0.46 | 1.79 | 3.81E-04 | -0.94 | 1.52 | 5.70E-02 | -0.48 | 1.02 | 5892145 | 2406965 | 4492884 | 1706694 | 4949078 | 1628166 | 4036689 | 1676130 |
| RP_POS_389_19781a11_277 | RP | POS | 11.28 | 389.1978 | | | | 2.36E-11 | 0.97 | 1.84 | 6.92E-03 | 0.54 | 1.67 | 4.81E-09 | 1.24 | 1.95 | 1.15E-06 | 0.70 | 1.74 | 418925 | 223942 | 882294 | 483392 | 752673 | 349986 | 1011915 | 561539 |
| RP_POS_760_82007a0_495 | RP | POS | 0.50 | 760.8201 | | | | 5.66E-04 | -0.56 | 0.97 | 6.94E-03 | 0.56 | 1.58 | 1.25E-01 | -0.28 | 0.55 | 2.42E-05 | -0.85 | 1.34 | 2262916 | 358104 | 1958016 | 568117 | 1806298 | 563931 | 2109734 | 535824 |
| RP_POS_998_76965a0_49 | RP | POS | 0.49 | 998.7697 | | | | 1.65E-02 | -0.40 | 0.76 | 6.95E-03 | 0.57 | 1.61 | 5.75E-01 | -0.11 | 0.27 | 2.56E-04 | -0.68 | 1.28 | 469064 | 161000 | 376926 | 246098 | 311163 | 210564 | 442688 | 263062 |
| RP_POS_421_255a13_292 | RP | POS | 13.29 | 421.2550 | | | | 2.18E-01 | -0.26 | 0.67 | 6.96E-03 | 0.52 | 1.78 | 9.98E-01 | 0.00 | 0.91 | 2.91E-02 | -0.52 | 0.79 | 1578407 | 594610 | 1430089 | 554062 | 1282128 | 573585 | 1578051 | 496544 |
| RP_POS_313_14688a10_822 | RP | POS | 10.82 | 313.1469 | | | | 2.44E-09 | 1.06 | 1.93 | 7.02E-03 | 0.51 | 1.80 | 1.60E-09 | 1.31 | 2.07 | 5.41E-06 | 0.81 | 1.78 | 429146 | 351453 | 964846 | 479550 | 836724 | 387346 | 1092969 | 530180 |
| RP_POS_502_29034a10_63 | RP | POS | 10.63 | 502.2903 | | | | 1.87E-02 | 0.42 | 1.06 | 7.02E-03 | 0.56 | 1.93 | 1.78E-03 | 0.70 | 1.22 | 4.36E-01 | 0.14 | 0.86 | 4630983 | 1347422 | 5344726 | 1763579 | 4873445 | 1396151 | 5816006 | 1970123 |
| HILIC_NEG_243_96837a0_93 | HILIC | NEG | 0.93 | 243.9684 | | | | 1.88E-02 | 0.43 | 0.76 | 7.05E-03 | -0.55 | 1.88 | 4.91E-01 | 0.15 | 0.26 | 2.91E-04 | 0.70 | 1.36 | 218331 | 59457 | 249494 | 75323 | 269621 | 58768 | 229368 | 84751 |
| RP_POS_402_77249a13_069 | RP | POS | 13.07 | 402.7725 | | | | 9.86E-01 | 0.00 | 0.55 | 7.14E-03 | -0.54 | 1.66 | 2.03E-01 | -0.28 | 0.94 | 2.28E-01 | 0.27 | 0.76 | 3618290 | 886716 | 3615013 | 961331 | 3870893 | 963118 | 3359133 | 897752 |
| RP_POS_854_81024a0_494 | RP | POS | 0.49 | 854.8102 | | | | 8.98E-03 | -0.39 | 0.73 | 7.16E-03 | 0.58 | 1.63 | 6.13E-01 | -0.10 | 0.24 | 3.99E-05 | -0.68 | 1.36 | 6761107 | 759689 | 6228004 | 1484795 | 5832148 | 1174380 | 6623860 | 1659897 |
| HILIC_NEG_257_98428a0_929 | HILIC | NEG | 0.929 | 257.9843 | | | | 5.11E-01 | 0.13 | 0.70 | 7.25E-03 | -0.55 | 1.72 | 4.98E-01 | -0.15 | 0.31 | 5.64E-02 | 0.40 | 0.70 | 243775 | 76059 | 254370 | 87491 | 277621 | 78458 | 231119 | 90592 |
| HILIC_NEG_353_99582a7_369 | HILIC | NEG | 7.369 | 353.9958 | | | | 9.39E-01 | -0.02 | 0.44 | 7.27E-03 | -0.54 | 1.76 | 1.97E-01 | -0.29 | 0.61 | 2.54E-01 | 0.25 | 0.45 | 445145 | 17313 | 444870 | 18254 | 449718 | 17954 | 440022 | 17404 |
| HILIC_POS_368_21997a2_225 | HILIC | POS | 2.225 | 368.2200 | | | | 1.74E-01 | -0.28 | 0.88 | 7.34E-03 | -0.53 | 1.49 | 1.67E-02 | -0.55 | 1.04 | 9.32E-01 | -0.02 | 0.50 | 1251979 | 352776 | 1153749 | 344692 | 1245192 | 346981 | 1062305 | 320295 |
| HILIC_POS_566_32043a1_198 | HILIC | POS | 1.198 | 566.3204 | | | | 1.80E-01 | 0.29 | 0.56 | 7.40E-03 | -0.52 | 1.43 | 8.96E-01 | 0.03 | 0.06 | 1.79E-02 | 0.55 | 1.04 | 1276025 | 549130 | 1424713 | 500132 | 1557333 | 439468 | 1292093 | 525684 |
| RP_POS_416_36465a12_743 | RP | POS | 12.74 | 416.3647 | | | | 8.05E-01 | 0.04 | 0.80 | 7.48E-03 | 0.59 | 1.59 | 1.39E-01 | 0.33 | 1.21 | 7.71E-02 | -0.25 | 0.74 | 898169 | 337595 | 919019 | 597079 | 759701 | 345473 | 1078337 | 741033 |
| RP_POS_372_1951a10_821 | RP | POS | 10.82 | 372.1951 | | | | 2.51E-09 | 1.03 | 1.89 | 7.53E-03 | 0.51 | 1.84 | 4.99E-09 | 1.28 | 2.02 | 4.53E-06 | 0.77 | 1.78 | 808057 | 599953 | 1733922 | 865889 | 1504196 | 645753 | 1963648 | 995052 |
| RP_POS_512_2674a9_944 | RP | POS | 9.94 | 512.2674 | | | | 4.72E-04 | 0.46 | 0.83 | 7.59E-03 | 0.59 | 1.58 | 5.54E-04 | 0.76 | 1.12 | 1.30E-01 | 0.16 | 0.61 | 537953 | 340144 | 966674 | 1032831 | 691333 | 567994 | 1242016 | 1295342 |
| RP_NEG_865_09442a9_304 | RP | NEG | 9.30 | 865.0944 | | | | 1.86E-02 | -0.41 | 0.73 | 7.60E-03 | -0.55 | 1.60 | 6.22E-04 | -0.69 | 1.24 | 5.00E-01 | -0.14 | 0.31 | 1738628 | 153735 | 1655938 | 209519 | 1711286 | 211195 | 1600589 | 194517 |
| HILIC_POS_123_04765a0_765 | HILIC | POS | 0.765 | 123.0477 | | | | 3.35E-05 | 0.49 | 1.17 | 7.64E-03 | 0.60 | 1.45 | 3.44E-04 | 0.79 | 1.12 | 1.26E-02 | 0.19 | 0.78 | 49340 | 34065 | 393518 | 789918 | 182531 | 361318 | 604506 | 1019448 |
| RP_POS_255_12279a7_844 | RP | POS | 7.84 | 255.1228 | | | | 1.75E-02 | -0.52 | 1.21 | 7.79E-03 | -0.50 | 1.60 | 1.04E-03 | -0.77 | 1.39 | 2.57E-01 | -0.27 | 0.91 | 13791479 | 10696638 | 8582118 | 9502410 | 11086091 | 10028518 | 6078145 | 8310687 |
| RP_POS_358_07092a7_844 | RP | POS | 7.84 | 358.0709 | | | | 1.45E-02 | -0.53 | 1.21 | 7.81E-03 | -0.50 | 1.59 | 8.17E-04 | -0.78 | 1.40 | 2.37E-01 | -0.28 | 0.92 | 1433427 | 1067723 | 896030 | 964124 | 1150010 | 1015386 | 642050 | 845805 |
| RP_POS_241_10696a7_844 | RP | POS | 7.84 | 241.1070 | | | | 2.09E-02 | -0.51 | 1.19 | 7.82E-03 | -0.50 | 1.60 | 1.31E-03 | -0.76 | 1.37 | 2.79E-01 | -0.26 | 0.90 | 3059994 | 2444822 | 1907469 | 2144035 | 2472214 | 2269377 | 1342724 | 1867251 |
| RP_POS_429_37338a12_232 | RP | POS | 12.23 | 429.3734 | | | | 6.74E-02 | -0.33 | 0.87 | 7.82E-03 | -0.55 | 1.58 | 4.02E-03 | -0.61 | 1.19 | 7.75E-01 | -0.06 | 0.24 | 1375291 | 280918 | 1261596 | 356474 | 1355419 | 342829 | 1167772 | 348201 |
| HILIC_NEG_180_97351a5_428 | HILIC | NEG | 5.428 | 180.9735 | | | | 1.39E-01 | -0.32 | 0.83 | 7.83E-03 | -0.51 | 1.43 | 1.66E-02 | -0.57 | 0.92 | 7.96E-01 | -0.06 | 0.43 | 397133 | 28017 | 388728 | 26003 | 395572 | 24049 | 381884 | 26308 |
| RP_POS_122_05779a2_25 | RP | POS | 2.25 | 122.0578 | | | | 2.00E-06 | 0.57 | 0.96 | 8.05E-03 | 0.59 | 1.56 | 3.17E-05 | 0.86 | 1.40 | 1.42E-02 | 0.28 | 0.76 | 105695 | 153097 | 921324 | 1596913 | 500370 | 1083523 | 1342279 | 1901747 |
| RP_POS_856_58337a12_751 | RP | POS | 12.75 | 856.5834 | | | | 3.49E-01 | 0.19 | 0.72 | 8.08E-03 | -0.52 | 1.55 | 7.63E-01 | -0.07 | 0.46 | 5.44E-02 | 0.46 | 0.84 | 764211 | 496092 | 858860 | 483762 | 985761 | 505825 | 731960 | 429072 |
| RP_POS_325_1124a0_548 | RP | POS | 0.55 | 325.1124 | | | | 3.32E-03 | 0.37 | 0.69 | 8.08E-03 | 0.60 | 1.68 | 3.13E-03 | 0.67 | 1.02 | 3.76E-01 | 0.07 | 0.80 | 3373037 | 586612 | 4151935 | 2386023 | 3517504 | 883092 | 4786366 | 3146604 |
| RP_POS_825_5556a10_463 | RP | POS | 10.46 | 825.5556 | | | | 6.07E-01 | -0.11 | 0.63 | 8.19E-03 | -0.51 | 1.64 | 1.02E-01 | -0.37 | 0.83 | 5.49E-01 | 0.15 | 0.64 | 783140 | 241292 | 758303 | 220643 | 816152 | 243789 | 700455 | 179158 |
| RP_POS_830_5556a12_954 | RP | POS | 12.95 | 830.5556 | | | | 1.08E-09 | -1.14 | 2.03 | 8.19E-03 | -0.48 | 1.63 | 7.62E-11 | -1.38 | 2.29 | 3.89E-06 | -0.90 | 1.71 | 15484561 | 2704194 | 11323878 | 3333223 | 12196241 | 3208100 | 10451516 | 3256147 |
| RP_POS_263_08887a7_844 | RP | POS | 7.84 | 263.0889 | C14H14O5 similar to methyl 2-((4-methyl-2-oxo-2H-chromen-7-yl)oxy)propanoate (ALDH2 inhibitor) | 263.0868(100) | 3 | 1.77E-02 | -0.53 | 1.21 | 8.22E-03 | -0.49 | 1.60 | 1.16E-03 | -0.77 | 1.38 | 2.44E-01 | -0.28 | 0.91 | 15432619 | 12264530 | 9497393 | 10625315 | 12278943 | 11183806 | 6715843 | 9339770 |
| HILIC_POS_172_09676a3_999 | HILIC | POS | 3.999 | 172.0968 | | | | 1.13E-05 | 0.61 | 1.05 | 8.24E-03 | 0.57 | 1.67 | 2.46E-05 | 0.89 | 1.40 | 1.63E-02 | 0.32 | 0.79 | 424844 | 310795 | 859597 | 774932 | 656020 | 541288 | 1063174 | 914180 |
| RP_POS_790_53442a12_671 | RP | POS | 12.67 | 790.5344 | | | | 3.41E-04 | 0.60 | 1.07 | 8.25E-03 | 0.55 | 1.58 | 6.48E-05 | 0.87 | 1.37 | 5.33E-02 | 0.33 | 1.18 | 1500895 | 490846 | 1928520 | 742981 | 1733708 | 570748 | 2123332 | 843862 |
| RP_POS_532_33722a10_73 | RP | POS | 10.73 | 532.3372 | | | | 1.35E-06 | -1.16 | 1.95 | 8.27E-03 | -0.41 | 1.79 | 6.84E-08 | -1.37 | 2.11 | 8.24E-05 | -0.96 | 1.58 | 6027189 | 2219095 | 3638876 | 1620976 | 4062930 | 1716580 | 3214822 | 1412488 |
| RP_NEG_197_80789a1_453 | RP | NEG | 1.45 | 197.8079 | | | | 4.19E-01 | 0.15 | 0.55 | 8.32E-03 | -0.54 | 1.79 | 5.80E-01 | -0.12 | 0.62 | 4.89E-02 | 0.42 | 0.75 | 18767676 | 6084779 | 19817703 | 7113028 | 21675891 | 6894686 | 17959516 | 6902140 |
| RP_POS_811_54388a13_276 | RP | POS | 13.28 | 811.5439 | | | | 4.18E-01 | -0.18 | 0.47 | 8.38E-03 | 0.50 | 1.63 | 7.75E-01 | 0.07 | 0.89 | 8.10E-02 | -0.43 | 0.76 | 953737 | 558046 | 864430 | 470143 | 741716 | 475939 | 987144 | 435119 |
| HILIC_POS_593_33301a0_5 | HILIC | POS | 0.5 | 593.3330 | | | | 3.73E-03 | 0.40 | 0.99 | 8.39E-03 | 0.58 | 1.65 | 2.02E-03 | 0.69 | 0.96 | 3.10E-01 | 0.11 | 0.43 | 224077 | 373892 | 556021 | 907557 | 316595 | 437631 | 795447 | 1164310 |
| RP_POS_352_24496a10_385 | RP | POS | 10.39 | 352.2450 | | | | 5.57E-01 | -0.12 | 0.98 | 8.46E-03 | 0.53 | 1.74 | 5.24E-01 | 0.15 | 0.69 | 5.83E-02 | -0.38 | 1.05 | 11848734 | 1900462 | 11615940 | 2053213 | 11078861 | 1510429 | 12153019 | 2376409 |
| RP_POS_485_35934a12_428 | RP | POS | 12.43 | 485.3593 | | | | 4.62E-02 | -0.38 | 1.29 | 8.47E-03 | -0.54 | 1.57 | 4.58E-03 | -0.64 | 1.29 | 5.83E-01 | -0.11 | 0.63 | 2492355 | 467240 | 2290171 | 552181 | 2434290 | 460159 | 2146051 | 601577 |
| HILIC_NEG_312_83673a3_956 | HILIC | NEG | 3.956 | 312.8367 | | | | 2.53E-01 | 0.20 | 0.94 | 8.51E-03 | -0.55 | 1.45 | 7.33E-01 | -0.07 | 0.22 | 2.17E-02 | 0.48 | 1.04 | 1218792 | 430618 | 1327690 | 559361 | 1473406 | 547942 | 1181973 | 537197 |
| RP_POS_181_08603a7_843 | RP | POS | 7.84 | 181.0860 | | | | 1.82E-02 | -0.52 | 1.20 | 8.53E-03 | -0.49 | 1.59 | 1.20E-03 | -0.77 | 1.37 | 2.51E-01 | -0.27 | 0.91 | 11490341 | 8999932 | 7147131 | 7884944 | 9201531 | 8267206 | 5092732 | 6975202 |
| RP_POS_820_58307a13_876 | RP | POS | 13.88 | 820.5831 | | | | 1.30E-02 | 0.50 | 0.97 | 8.62E-03 | 0.52 | 1.55 | 6.23E-04 | 0.76 | 1.31 | 2.79E-01 | 0.24 | 0.69 | 4685755 | 1686392 | 5575777 | 1770476 | 5115199 | 1817582 | 6036356 | 1611402 |
| RP_POS_792_04407a12_816 | RP | POS | 12.82 | 792.0441 | | | | 3.02E-02 | 0.40 | 0.81 | 8.69E-03 | -0.54 | 1.68 | 4.77E-01 | 0.14 | 0.52 | 3.26E-03 | 0.67 | 1.12 | 1033104 | 582754 | 1310117 | 706649 | 1494092 | 789152 | 1126143 | 563081 |
| HILIC_NEG_276_992a0_93 | HILIC | NEG | 0.93 | 276.9920 | | | | 5.04E-01 | -0.13 | 0.83 | 8.74E-03 | -0.52 | 1.47 | 7.67E-02 | -0.40 | 0.67 | 5.68E-01 | 0.13 | 0.27 | 702126 | 129778 | 684271 | 134039 | 719071 | 131495 | 649471 | 128597 |
| RP_POS_991_67065a10_375 | RP | POS | 10.38 | 991.6707 | | | | 8.67E-02 | 0.32 | 1.03 | 8.83E-03 | -0.53 | 1.65 | 7.95E-01 | 0.05 | 0.63 | 6.38E-03 | 0.59 | 1.20 | 7817987 | 4633030 | 9536605 | 5513072 | 10965990 | 5412943 | 8107221 | 5285698 |
| RP_POS_421_7709a14_746 | RP | POS | 14.75 | 421.7709 | | | | 3.42E-05 | 0.60 | 1.04 | 8.84E-03 | 0.56 | 1.57 | 4.61E-05 | 0.88 | 1.30 | 1.94E-02 | 0.32 | 0.89 | 978925 | 658453 | 1768356 | 1410504 | 1400742 | 940435 | 2135970 | 1690986 |
| RP_POS_817_55908a12_956 | RP | POS | 12.96 | 817.5591 | | | | 7.36E-08 | -1.03 | 1.88 | 8.87E-03 | -0.48 | 1.60 | 3.95E-09 | -1.27 | 2.15 | 8.03E-05 | -0.79 | 1.52 | 8043150 | 1994345 | 5454021 | 2328051 | 6057308 | 2248583 | 4850735 | 2269662 |
| HILIC_NEG_117_91902a0_924 | HILIC | NEG | 0.924 | 117.9190 | | | | 2.32E-04 | -0.75 | 1.41 | 8.93E-03 | -0.49 | 1.48 | 1.26E-05 | -1.00 | 1.65 | 1.88E-02 | -0.50 | 0.92 | 1656600 | 327707 | 1392275 | 335727 | 1479204 | 320908 | 1305346 | 330637 |
| RP_POS_504_34174a10_775 | RP | POS | 10.78 | 504.3417 | | | | 3.52E-03 | -0.67 | 1.21 | 8.95E-03 | -0.47 | 1.70 | 2.09E-04 | -0.91 | 1.53 | 7.48E-02 | -0.44 | 0.88 | 3698728 | 1003344 | 3097949 | 808718 | 3307492 | 868131 | 2888406 | 691474 |
| RP_POS_245_12845a5_215 | RP | POS | 5.22 | 245.1285 | | | | 2.22E-04 | 0.44 | 0.81 | 9.05E-03 | 0.59 | 1.54 | 6.87E-04 | 0.73 | 1.14 | 1.16E-01 | 0.14 | 0.61 | 157986 | 154646 | 1106217 | 2460768 | 464458 | 1341734 | 1747976 | 3097349 |
| RP_POS_287_13309a10_198 | RP | POS | 10.20 | 287.1331 | | | | 1.28E-02 | -0.60 | 1.29 | 9.06E-03 | -0.46 | 1.76 | 1.59E-03 | -0.83 | 1.36 | 1.30E-01 | -0.37 | 0.95 | 769626 | 303558 | 618719 | 221131 | 675882 | 202101 | 561557 | 226457 |
| RP_POS_374_32639a10_959 | RP | POS | 10.96 | 374.3264 | | | | 1.24E-05 | 0.53 | 0.98 | 9.27E-03 | 0.58 | 1.65 | 1.51E-04 | 0.82 | 1.20 | 1.05E-02 | 0.25 | 0.94 | 199382 | 189097 | 693245 | 1028633 | 425801 | 559295 | 960690 | 1295755 |
| HILIC_POS_141_10233a0_559 | HILIC | POS | 0.559 | 141.1023 | | | | 4.57E-10 | 0.79 | 1.26 | 9.27E-03 | 0.56 | 1.51 | 1.23E-06 | 1.07 | 1.48 | 7.31E-07 | 0.52 | 1.56 | 338553 | 349501 | 1356452 | 1373491 | 999350 | 745002 | 1713553 | 1730947 |
| RP_POS_279_23154a11_259 | RP | POS | 11.26 | 279.2315 | | | | 8.01E-02 | -0.39 | 0.63 | 9.36E-03 | -0.50 | 1.65 | 3.69E-03 | -0.64 | 1.37 | 5.92E-01 | -0.14 | 0.60 | 1123613 | 1124438 | 727867 | 971283 | 980540 | 1247628 | 475194 | 468151 |
| RP_POS_321_23984a10_662 | RP | POS | 10.66 | 321.2398 | | | | 9.85E-03 | 0.44 | 1.28 | 9.48E-03 | 0.54 | 1.68 | 1.10E-03 | 0.71 | 1.40 | 3.36E-01 | 0.17 | 0.96 | 8743920 | 1481309 | 9638183 | 2132306 | 9089245 | 1709395 | 10187120 | 2376817 |
| RP_NEG_357_28125a11_419 | RP | NEG | 11.42 | 357.2813 | FA 24:5 (Tetracosapentaenoic acid) | 357.2808(100) | 3 | 4.77E-08 | 0.72 | 1.55 | 9.61E-03 | 0.56 | 1.53 | 4.95E-06 | 1.00 | 1.56 | 9.12E-05 | 0.45 | 1.49 | 386592 | 325185 | 1039423 | 967431 | 789303 | 558338 | 1289544 | 1204795 |
| RP_POS_964_77942a0_491 | RP | POS | 0.49 | 964.7794 | | | | 6.65E-03 | -0.44 | 0.80 | 9.73E-03 | 0.55 | 1.52 | 3.92E-01 | -0.17 | 0.34 | 1.19E-04 | -0.72 | 1.27 | 643614 | 185086 | 521413 | 292732 | 446385 | 255118 | 596440 | 310741 |
| HILIC_POS_358_25897a1_046 | HILIC | POS | 1.046 | 358.2590 | | | | 1.43E-06 | 0.65 | 1.21 | 9.78E-03 | 0.56 | 1.59 | 1.64E-05 | 0.93 | 1.49 | 2.63E-03 | 0.37 | 0.99 | 398886 | 172632 | 684959 | 476146 | 562350 | 303224 | 807567 | 579165 |
| RP_POS_377_24228a11_272 | RP | POS | 11.27 | 377.2423 | | | | 1.45E-08 | 0.82 | 1.73 | 9.79E-03 | 0.54 | 1.64 | 3.83E-07 | 1.09 | 1.79 | 8.59E-05 | 0.55 | 1.63 | 422708 | 343226 | 1010315 | 747035 | 818331 | 522367 | 1202298 | 882850 |
| RP_POS_788_55511a13_105 | RP | POS | 13.11 | 788.5551 | | | | 4.92E-01 | -0.17 | 0.52 | 9.86E-03 | -0.46 | 1.56 | 1.17E-01 | -0.40 | 0.65 | 8.07E-01 | 0.06 | 0.40 | 503879 | 562890 | 429443 | 402829 | 532508 | 428605 | 326379 | 349962 |
| RP_POS_828_80743a0_494 | RP | POS | 0.49 | 828.8074 | | | | 1.19E-02 | -0.41 | 0.91 | 9.90E-03 | 0.55 | 1.57 | 4.97E-01 | -0.14 | 0.28 | 2.18E-04 | -0.68 | 1.32 | 1576561 | 268671 | 1412015 | 429112 | 1302271 | 370813 | 1521760 | 458203 |

**Supplementary table 1: Metabolomics results** (in asceding order according to p-value from progressive ALD vs  non-progressive ALD comparison, with molecular features with ID_level of 1-2 first)

| | | | | | | | | all_ALD_vs_control | | | Prog_ALD_vs_non-prog_ALD | | | Prog_ALD_vs_control_ALD | | | Non-prog_ALD_vs_control | | | Healthy_control | | all_ALD | | Non-prog_ALD | | Progressive_ALD | |
|---|---|---|---|---|---|---|---|---|---|---|---|---|---|---|---|---|---|---|---|---|---|---|---|---|---|---|---|
| | | | | | | | | t-test | Cohen's | PLS-DA | t-test | Cohen's | PLS-DA | t-test | Cohen's | PLS-DA | t-test | Cohen's | PLS-DA | | | | | | | | |
| Feature_ID | RP_HILIC | POS_NEG | Average.Rt | Average.Mz | Metabolite.name | curated_MSMS | ID_level | p | d | VIP | p | d | VIP | p | d | VIP | p | d | VIP | Mean | SD | Mean | SD | Mean | SD | Mean | SD |
| HILIC_NEG_286_01495a0_712 | HILIC | NEG | 0.712 | 286.0150 | | | | 2.19E-09 | 0.73 | 1.31 | 9.92E-03 | 0.56 | 1.73 | 3.79E-06 | 1.01 | 1.49 | 4.01E-06 | 0.45 | 1.46 | 67573 | 95983 | 487214 | 621912 | 326971 | 341138 | 647456 | 782728 |
| HILIC_POS_357_68613a1_919 | HILIC | POS | 1.919 | 357.6861 | | | | 1.16E-01 | -0.24 | 1.01 | 1.01E-02 | -0.56 | 1.40 | 7.90E-03 | -0.52 | 1.05 | 8.51E-01 | 0.03 | 1.24 | 674113 | 137125 | 619730 | 242775 | 681603 | 222969 | 557857 | 248102 |
| HILIC_POS_657_39923a1_223 | HILIC | POS | 1.223 | 657.3992 | | | | 1.80E-01 | 0.29 | 0.54 | 1.01E-02 | -0.50 | 1.38 | 8.61E-01 | 0.04 | 0.07 | 2.06E-02 | 0.54 | 0.98 | 764956 | 398620 | 872863 | 362371 | 965223 | 324472 | 780503 | 377642 |
| RP_POS_605_27783a10_198 | RP | POS | 10.20 | 605.2778 | | | | 2.39E-02 | -0.53 | 1.23 | 1.01E-02 | -0.46 | 1.81 | 2.95E-03 | -0.76 | 1.32 | 2.14E-01 | -0.30 | 0.91 | 560656 | 199637 | 470669 | 152062 | 509410 | 143307 | 431929 | 152046 |
| RP_POS_529_29218a10_629 | RP | POS | 10.63 | 529.2922 | | | | 1.57E-03 | 0.54 | 1.15 | 1.02E-02 | 0.53 | 1.84 | 1.89E-04 | 0.81 | 1.40 | 1.24E-01 | 0.27 | 1.01 | 433848 | 184004 | 571604 | 265739 | 503862 | 220050 | 639345 | 291356 |
| RP_POS_375_32016a10_385 | RP | POS | 10.39 | 375.3202 | | | | 4.94E-02 | -0.42 | 1.08 | 1.03E-02 | 0.50 | 1.61 | 4.96E-01 | -0.17 | 0.63 | 2.31E-03 | -0.66 | 1.35 | 1115210 | 301995 | 993161 | 286575 | 919981 | 204774 | 1066342 | 336252 |
| RP_POS_786_82269a0_494 | RP | POS | 0.49 | 786.8227 | | | | 2.47E-03 | -0.45 | 0.80 | 1.03E-02 | 0.55 | 1.56 | 3.58E-01 | -0.18 | 0.32 | 2.35E-05 | -0.73 | 1.36 | 8508001 | 904735 | 7770039 | 1759821 | 7321965 | 1485152 | 8218112 | 1908332 |
| RP_NEG_606_36053a10_827 | RP | NEG | 10.83 | 606.3605 | | | | 4.96E-11 | 0.85 | 1.78 | 1.03E-02 | 0.54 | 1.65 | 1.23E-08 | 1.12 | 1.92 | 8.77E-05 | 0.58 | 1.59 | 146785 | 157786 | 592951 | 555445 | 451617 | 475526 | 734286 | 597028 |
| RP_POS_429_37189a13_84 | RP | POS | 13.84 | 429.3719 | | | | 7.32E-03 | -0.56 | 0.95 | 1.03E-02 | -0.49 | 1.51 | 5.42E-04 | -0.81 | 1.36 | 1.54E-01 | -0.32 | 0.57 | 2904698 | 979237 | 2353945 | 942502 | 2593530 | 908124 | 2114360 | 923534 |
| RP_NEG_384_9368a12_889 | RP | NEG | 12.89 | 384.9368 | | | | 3.09E-02 | 0.40 | 0.84 | 1.03E-02 | 0.53 | 1.52 | 1.91E-03 | 0.66 | 1.30 | 5.16E-01 | 0.13 | 0.54 | 2095809 | 417054 | 2294913 | 520184 | 2162687 | 502779 | 2427139 | 508171 |
| RP_POS_922_79694a0_493 | RP | POS | 0.49 | 922.7969 | | | | 3.61E-01 | -0.14 | 0.59 | 1.03E-02 | 0.56 | 1.63 | 4.77E-01 | 0.15 | 0.45 | 7.68E-03 | -0.42 | 1.04 | 3611814 | 489690 | 3492026 | 978803 | 3242512 | 732662 | 3741541 | 1127746 |
| RP_POS_300_21698a7_727 | RP | POS | 7.73 | 300.2170 | | | | 2.18E-01 | 0.21 | 0.82 | 1.03E-02 | 0.54 | 1.68 | 3.31E-02 | 0.48 | 1.20 | 7.19E-01 | -0.06 | 0.27 | 1031289 | 72992 | 1051844 | 103769 | 1025357 | 71608 | 1078332 | 123272 |
| RP_POS_718_57379a13_467 | RP | POS | 13.47 | 718.5738 | | | | 6.72E-05 | 0.50 | 0.95 | 1.04E-02 | 0.57 | 1.59 | 3.97E-04 | 0.78 | 1.17 | 2.04E-02 | 0.21 | 0.76 | 625450 | 258237 | 1112125 | 1087828 | 832877 | 528516 | 1391374 | 1397290 |
| HILIC_NEG_113_92606a0_924 | HILIC | NEG | 0.924 | 113.9261 | | | | 2.50E-04 | -0.78 | 1.49 | 1.04E-02 | -0.47 | 1.47 | 1.21E-05 | -1.02 | 1.71 | 1.60E-02 | -0.54 | 1.03 | 2577393 | 539993 | 2150718 | 508830 | 2279949 | 516294 | 2021488 | 471655 |
| RP_POS_817_05078a12_806 | RP | POS | 12.81 | 817.0508 | | | | 5.06E-02 | -0.44 | 1.01 | 1.04E-02 | -0.47 | 1.51 | 5.52E-03 | -0.68 | 1.19 | 3.94E-01 | -0.21 | 0.63 | 1016058 | 484762 | 826158 | 400247 | 927764 | 401353 | 724552 | 376181 |
| RP_NEG_622_33478a10_73 | RP | NEG | 10.73 | 622.3348 | | | | 1.26E-07 | -1.19 | 1.99 | 1.04E-02 | -0.42 | 1.80 | 5.26E-09 | -1.40 | 2.19 | 1.81E-05 | -0.98 | 1.65 | 1500743 | 464155 | 933148 | 393992 | 1033187 | 408622 | 833108 | 355291 |
| RP_POS_808_58264a13_877 | RP | POS | 13.88 | 808.5826 | | | | 1.97E-01 | 0.33 | 0.81 | 1.05E-02 | 0.44 | 1.56 | 4.68E-02 | 0.55 | 0.95 | 6.75E-01 | 0.11 | 0.22 | 20554492 | 22497510 | 26116782 | 14670131 | 22393948 | 12635815 | 29839616 | 15712069 |
| HILIC_NEG_564_33032a1_229 | HILIC | NEG | 1.229 | 564.3303 | | | | 1.18E-01 | -0.39 | 0.86 | 1.07E-02 | -0.45 | 1.39 | 2.14E-02 | -0.61 | 1.12 | 5.24E-01 | -0.16 | 0.41 | 5193958 | 2418451 | 4459746 | 1676328 | 4883720 | 1601085 | 4035773 | 1657463 |
| RP_POS_705_59076a13_073 | RP | POS | 13.07 | 705.5908 | | | | 2.55E-01 | -0.23 | 0.49 | 1.08E-02 | 0.51 | 1.74 | 9.18E-01 | 0.02 | 0.76 | 2.73E-02 | -0.48 | 0.89 | 640714 | 450264 | 534740 | 462151 | 417868 | 411096 | 651612 | 484407 |
| HILIC_POS_171_07648a6_13 | HILIC | POS | 6.13 | 171.0765 | | | | 5.93E-01 | -0.09 | 0.92 | 1.08E-02 | -0.54 | 1.43 | 7.35E-02 | -0.36 | 0.78 | 3.56E-01 | 0.18 | 0.89 | 2907094 | 799166 | 2808925 | 1161560 | 3102532 | 1102176 | 2515318 | 1155535 |
| RP_POS_205_98705a15_422 | RP | POS | 15.42 | 205.9871 | | | | 8.83E-01 | 0.03 | 0.65 | 1.09E-02 | -0.53 | 1.52 | 3.44E-01 | -0.24 | 0.55 | 7.80E-02 | 0.29 | 0.77 | 86730667 | 7368657 | 86961317 | 8715523 | 89192473 | 2701596 | 84730161 | 11662460 |
| RP_POS_546_35413a10_512 | RP | POS | 10.51 | 546.3541 | | | | 8.37E-03 | 0.52 | 1.12 | 1.09E-02 | -0.50 | 1.51 | 2.19E-01 | 0.27 | 0.70 | 4.06E-04 | 0.77 | 1.32 | 42862096 | 14547795 | 51104394 | 15747076 | 55077608 | 14371216 | 47131180 | 16194207 |
| HILIC_NEG_121_02934a0_927 | HILIC | NEG | 0.927 | 121.0293 | | | | 2.87E-01 | 0.19 | 0.49 | 1.12E-02 | -0.53 | 1.71 | 7.49E-01 | -0.07 | 0.18 | 2.33E-02 | 0.46 | 0.82 | 253159 | 93551 | 274926 | 117154 | 304412 | 103700 | 245441 | 123298 |
| RP_POS_591_31775a6_112 | RP | POS | 6.11 | 591.3178 | | | | 4.45E-04 | 0.42 | 0.81 | 1.12E-02 | 0.58 | 1.66 | 1.81E-03 | 0.71 | 1.04 | 2.51E-02 | 0.13 | 0.91 | 293920 | 424660 | 1497224 | 3235926 | 671461 | 1038701 | 2322987 | 4322687 |
| RP_NEG_554_30267a10_198 | RP | NEG | 10.20 | 554.3027 | | | | 4.31E-02 | -0.49 | 1.16 | 1.12E-02 | -0.45 | 1.68 | 6.34E-03 | -0.71 | 1.23 | 2.93E-01 | -0.26 | 0.87 | 1835798 | 695153 | 1558225 | 510563 | 1686615 | 478624 | 1429835 | 513758 |
| RP_NEG_654_30365a10_031 | RP | NEG | 10.03 | 654.3037 | | | | 8.40E-02 | 0.38 | 1.02 | 1.12E-02 | -0.48 | 1.54 | 5.44E-01 | 0.14 | 0.55 | 1.05E-02 | 0.62 | 1.11 | 1418433 | 970958 | 1755926 | 841306 | 1967389 | 826508 | 1544464 | 809729 |
| HILIC_NEG_590_34821a1_17 | HILIC | NEG | 1.17 | 590.3482 | | | | 2.59E-04 | 0.72 | 1.35 | 1.15E-02 | -0.49 | 1.40 | 2.73E-02 | 0.48 | 0.84 | 9.14E-06 | 0.97 | 1.75 | 277587 | 117237 | 372449 | 127453 | 404405 | 114995 | 340494 | 132333 |
| HILIC_POS_496_33929a1_171 | HILIC | POS | 1.171 | 496.3393 | | | | 9.01E-02 | 0.32 | 0.83 | 1.15E-02 | -0.52 | 1.43 | 7.85E-01 | 0.06 | 0.15 | 7.51E-03 | 0.57 | 1.00 | 81227841 | 29966228 | 92273103 | 36179543 | 101340749 | 35687315 | 83205456 | 34687854 |
| RP_POS_299_62543a10_032 | RP | POS | 10.03 | 299.6254 | | | | 1.02E-01 | 0.35 | 1.00 | 1.15E-02 | -0.48 | 1.54 | 6.24E-01 | 0.11 | 0.56 | 1.37E-02 | 0.60 | 1.07 | 1368712 | 973408 | 1691229 | 880624 | 1911794 | 901968 | 1470664 | 808699 |
| RP_POS_401_26575a10_667 | RP | POS | 10.67 | 401.2658 | | | | 8.01E-05 | 0.52 | 1.09 | 1.16E-02 | 0.56 | 1.58 | 2.46E-04 | 0.79 | 1.24 | 3.70E-02 | 0.24 | 0.94 | 392513 | 193471 | 678434 | 612980 | 523985 | 365338 | 832883 | 759607 |
| HILIC_NEG_221_06674a5_435 | HILIC | NEG | 5.435 | 221.0667 | | | | 1.05E-03 | 0.42 | 0.95 | 1.16E-02 | 0.56 | 1.42 | 8.94E-04 | 0.70 | 1.02 | 2.35E-01 | 0.14 | 0.55 | 191922 | 157974 | 397667 | 546304 | 260211 | 352086 | 535123 | 663674 |
| RP_POS_779_54333a13_018 | RP | POS | 13.02 | 779.5433 | | | | 8.37E-14 | 1.05 | 1.72 | 1.17E-02 | 0.50 | 1.55 | 1.88E-10 | 1.30 | 1.99 | 1.39E-07 | 0.79 | 1.71 | 381909 | 270570 | 1064214 | 653433 | 900582 | 533909 | 1227846 | 723161 |
| HILIC_POS_633_39838a1_259 | HILIC | POS | 1.259 | 633.3984 | | | | 1.27E-01 | -0.35 | 0.72 | 1.17E-02 | -0.46 | 1.40 | 1.77E-02 | -0.59 | 1.17 | 6.26E-01 | -0.12 | 0.31 | 1367101 | 634023 | 1176146 | 501128 | 1301477 | 520219 | 1050814 | 452435 |
| HILIC_POS_130_05009a6_129 | HILIC | POS | 6.129 | 130.0501 | | | | 2.60E-01 | -0.19 | 0.98 | 1.19E-02 | -0.53 | 1.42 | 2.51E-02 | -0.46 | 0.91 | 6.90E-01 | 0.08 | 1.00 | 45020294 | 12496810 | 41767036 | 18302088 | 46331319 | 17087860 | 37202753 | 18500224 |
| RP_POS_338_16592a7_822 | RP | POS | 7.82 | 338.1659 | | | | 8.38E-01 | 0.05 | 0.35 | 1.19E-02 | 0.48 | 1.62 | 2.41E-01 | 0.28 | 0.84 | 4.28E-01 | -0.19 | 0.55 | 936943 | 56035 | 939193 | 47096 | 927454 | 46358 | 950932 | 45288 |
| HILIC_POS_84_04503a6_129 | HILIC | POS | 6.129 | 84.0450 | | | | 3.02E-01 | -0.17 | 0.98 | 1.20E-02 | -0.53 | 1.42 | 2.93E-02 | -0.44 | 0.91 | 6.29E-01 | 0.09 | 0.98 | 1261139 | 343234 | 1178976 | 506712 | 1305234 | 478912 | 1052719 | 506818 |
| HILIC_POS_590_32074a1_185 | HILIC | POS | 1.185 | 590.3207 | | | | 9.39E-01 | -0.01 | 0.39 | 1.21E-02 | -0.51 | 1.41 | 2.00E-01 | -0.27 | 0.55 | 2.67E-01 | 0.24 | 0.41 | 442316 | 211805 | 438883 | 243306 | 499425 | 244919 | 378342 | 228338 |
| RP_POS_202_14374a6_99 | RP | POS | 6.99 | 202.1437 | | | | 5.15E-01 | 0.13 | 0.59 | 1.21E-02 | 0.50 | 1.65 | 1.11E-01 | 0.38 | 0.89 | 5.70E-01 | -0.12 | 0.29 | 476864 | 321035 | 519818 | 326611 | 438386 | 257023 | 601250 | 368691 |
| HILIC_NEG_355_01974a0_93 | HILIC | NEG | 0.93 | 355.0197 | | | | 4.27E-01 | -0.16 | 0.85 | 1.22E-02 | -0.50 | 1.53 | 5.69E-02 | -0.41 | 0.77 | 6.80E-01 | 0.09 | 0.15 | 475158 | 94299 | 459609 | 99803 | 484421 | 105381 | 434796 | 88108 |
| RP_NEG_672_85962a0_493 | RP | NEG | 0.49 | 672.8596 | | | | 2.05E-03 | -0.47 | 0.85 | 1.23E-02 | 0.54 | 1.55 | 3.06E-01 | -0.20 | 0.39 | 1.75E-05 | -0.74 | 1.38 | 11552465 | 1058021 | 10693228 | 1965822 | 10204352 | 1612408 | 11182104 | 2172594 |
| RP_POS_288_6337a10_198 | RP | POS | 10.20 | 288.6337 | | | | 2.13E-02 | -0.55 | 1.23 | 1.23E-02 | -0.44 | 1.73 | 3.03E-03 | -0.77 | 1.31 | 1.79E-01 | -0.33 | 0.93 | 5388529 | 1962276 | 4488949 | 1453389 | 4849754 | 1335871 | 4128145 | 1489149 |
| HILIC_NEG_564_33057a1_105 | HILIC | NEG | 1.105 | 564.3306 | | | | 1.63E-01 | -0.34 | 0.76 | 1.24E-02 | -0.44 | 1.37 | 3.30E-02 | -0.56 | 1.03 | 6.32E-01 | -0.12 | 0.33 | 798559 | 398912 | 690996 | 278353 | 760050 | 266700 | 621941 | 275103 |
| RP_POS_510_35535a10_729 | RP | POS | 10.73 | 510.3554 | | | | 3.32E-07 | -1.20 | 2.01 | 1.24E-02 | -0.39 | 1.79 | 1.92E-08 | -1.40 | 2.16 | 2.23E-05 | -1.00 | 1.66 | 26266052 | 8770785 | 16086955 | 6740759 | 17758978 | 6952004 | 14414931 | 6145500 |
| RP_POS_513_84949a11_128 | RP | POS | 11.13 | 513.8495 | | | | 1.45E-01 | 0.30 | 0.87 | 1.25E-02 | -0.50 | 1.69 | 7.95E-01 | 0.05 | 0.56 | 2.78E-02 | 0.55 | 1.06 | 292229 | 730229 | 511529 | 727408 | 692558 | 870820 | 330499 | 493797 |
| RP_POS_718_83563a0_495 | RP | POS | 0.50 | 718.8356 | | | | 4.83E-05 | -0.59 | 0.99 | 1.25E-02 | 0.53 | 1.53 | 8.06E-02 | -0.33 | 0.59 | 1.03E-06 | -0.86 | 1.51 | 11527421 | 1112660 | 10228347 | 2360347 | 9643415 | 2090628 | 10813280 | 2487406 |
| HILIC_POS_101_07129a6_129 | HILIC | POS | 6.129 | 101.0713 | | | | 3.42E-01 | -0.17 | 0.97 | 1.25E-02 | -0.53 | 1.42 | 3.75E-02 | -0.43 | 0.86 | 6.29E-01 | 0.10 | 0.91 | 869321 | 220337 | 822289 | 299879 | 896549 | 286359 | 748029 | 297379 |
| RP_POS_534_29584a10_375 | RP | POS | 10.38 | 534.2958 | | | | 3.35E-01 | 0.20 | 0.99 | 1.26E-02 | -0.49 | 1.70 | 8.67E-01 | -0.04 | 0.58 | 4.94E-02 | 0.45 | 1.13 | 1200930 | 372112 | 1273501 | 350100 | 1360173 | 313469 | 1186829 | 366126 |
| RP_POS_496_33994a11_254 | RP | POS | 11.25 | 496.3399 | | | | 6.64E-01 | 0.08 | 1.11 | 1.26E-02 | -0.52 | 1.61 | 3.52E-01 | -0.18 | 0.68 | 1.19E-01 | 0.34 | 1.02 | 1713920 | 639759 | 1774909 | 821480 | 1978237 | 873877 | 1571582 | 717911 |
| RP_POS_1009_66394a11_135 | RP | POS | 11.14 | 1009.6639 | | | | 2.19E-02 | 0.28 | 0.94 | 1.27E-02 | -0.56 | 1.58 | 9.64E-01 | 0.00 | 0.57 | 6.86E-03 | 0.56 | 1.17 | 691598 | 78167 | 795544 | 425699 | 901332 | 517780 | 689755 | 274231 |
| RP_POS_254_95401a15_48 | RP | POS | 15.48 | 254.9540 | | | | 6.21E-01 | -0.12 | 0.51 | 1.27E-02 | -0.45 | 1.46 | 1.82E-01 | -0.34 | 0.94 | 6.80E-01 | 0.11 | 0.26 | 1984815 | 114299 | 1973925 | 83050 | 1994447 | 80774 | 1953402 | 80940 |
| HILIC_NEG_373_01025a0_931 | HILIC | NEG | 0.931 | 373.0103 | | | | 6.66E-01 | -0.09 | 0.77 | 1.28E-02 | -0.50 | 1.52 | 1.45E-01 | -0.34 | 0.56 | 4.75E-01 | 0.16 | 0.27 | 496185 | 98882 | 487472 | 98707 | 511853 | 92293 | 463091 | 99769 |
| RP_NEG_554_34717a10_73 | RP | NEG | 10.73 | 554.3472 | | | | 2.83E-07 | -1.19 | 1.98 | 1.30E-02 | -0.40 | 1.79 | 1.51E-08 | -1.39 | 2.16 | 2.40E-05 | -0.99 | 1.64 | 12017512 | 3966194 | 7367836 | 3165794 | 8147958 | 3291071 | 6587714 | 2859347 |
| HILIC_POS_204_12309a3_117 | HILIC | POS | 3.117 | 204.1231 | | | | 1.17E-08 | 0.85 | 1.54 | 1.31E-02 | 0.51 | 1.53 | 1.12E-07 | 1.11 | 1.79 | 1.09E-04 | 0.60 | 1.27 | 17133221 | 9170763 | 32241256 | 18255451 | 27738896 | 14422153 | 36743617 | 20590189 |
| HILIC_NEG_128_03555a6_093 | HILIC | NEG | 6.093 | 128.0356 | | | | 4.04E-01 | -0.14 | 0.91 | 1.31E-02 | -0.52 | 1.41 | 4.36E-02 | -0.41 | 0.80 | 5.55E-01 | 0.12 | 0.91 | 210067 | 54397 | 199746 | 76445 | 218558 | 74967 | 180934 | 73925 |
| RP_POS_320_90628a15_426 | RP | POS | 15.43 | 320.9063 | | | | 2.01E-03 | -0.40 | 0.75 | 1.32E-02 | -0.55 | 1.92 | 1.31E-03 | -0.68 | 1.24 | 2.90E-01 | -0.13 | 0.49 | 5096230 | 186995 | 4885002 | 582617 | 5028935 | 380167 | 4741068 | 706404 |
| RP_POS_548_30023a10_327 | RP | POS | 10.33 | 548.3002 | | | | 1.57E-03 | -0.77 | 1.45 | 1.32E-02 | -0.42 | 1.70 | 1.87E-04 | -0.98 | 1.59 | 2.40E-02 | -0.56 | 1.12 | 6780706 | 1949891 | 5520994 | 1410516 | 5867655 | 1320358 | 5174332 | 1425172 |
| RP_POS_496_33984a11_163 | RP | POS | 11.16 | 496.3398 | | | | 5.09E-01 | -0.12 | 0.99 | 1.33E-02 | -0.51 | 1.52 | 8.20E-02 | -0.38 | 0.90 | 5.25E-01 | 0.13 | 0.87 | 2538286 | 759994 | 2430684 | 904127 | 2652812 | 839499 | 2208557 | 919848 |
| HILIC_POS_360_27429a1_026 | HILIC | POS | 1.026 | 360.2743 | | | | 1.82E-05 | 0.63 | 1.19 | 1.33E-02 | 0.53 | 1.55 | 3.37E-05 | 0.89 | 1.43 | 1.08E-02 | 0.36 | 0.87 | 650784 | 303763 | 1024262 | 637692 | 867016 | 445871 | 1181509 | 756531 |
| RP_POS_299_19543a10_824 | RP | POS | 10.82 | 299.1954 | | | | 1.13E-10 | 0.91 | 1.77 | 1.33E-02 | 0.51 | 1.66 | 1.11E-08 | 1.16 | 1.94 | 1.39E-05 | 0.66 | 1.66 | 469689 | 451908 | 1406933 | 1058385 | 1146737 | 863153 | 1667130 | 1174742 |
| RP_POS_692_83264a0_496 | RP | POS | 0.50 | 692.8326 | | | | 6.73E-05 | -0.63 | 1.17 | 1.34E-02 | 0.52 | 1.49 | 4.90E-02 | -0.37 | 0.72 | 2.59E-06 | -0.89 | 1.62 | 2917216 | 400511 | 2504537 | 689115 | 2335374 | 640144 | 2673700 | 700904 |
| HILIC_POS_595_34863a0_504 | HILIC | POS | 0.504 | 595.3486 | | | | 2.84E-04 | 0.54 | 1.09 | 1.34E-02 | 0.53 | 1.32 | 3.22E-04 | 0.80 | 1.40 | 4.08E-02 | 0.27 | 0.87 | 214152 | 420124 | 639228 | 855498 | 427766 | 500336 | 850690 | 1066347 |
| RP_NEG_195_8111a1_454 | RP | NEG | 1.45 | 195.8111 | | | | 7.14E-01 | -0.07 | 0.40 | 1.34E-02 | -0.50 | 1.65 | 1.41E-01 | -0.32 | 0.72 | 4.09E-01 | 0.18 | 0.57 | 16952238 | 5443179 | 16535445 | 5986379 | 18004212 | 5826225 | 15066679 | 5835866 |
| HILIC_NEG_241_9742a1_3 | HILIC | NEG | 1.3 | 241.9742 | | | | 4.22E-07 | 0.86 | 1.47 | 1.34E-02 | 0.49 | 1.38 | 5.20E-07 | 1.11 | 1.61 | 2.53E-04 | 0.61 | 1.34 | 1866818 | 289905 | 2236146 | 429177 | 2130571 | 320805 | 2341722 | 496469 |
| RP_POS_145_04935a0_549 | RP | POS | 0.55 | 145.0494 | | | | 2.88E-03 | 0.37 | 0.71 | 1.35E-02 | 0.56 | 1.60 | 3.59E-03 | 0.65 | 1.01 | 2.62E-01 | 0.09 | 0.90 | 1605154 | 236596 | 1924550 | 963723 | 1685606 | 407198 | 2163493 | 1262597 |
| HILIC_NEG_229_08878a0_713 | HILIC | NEG | 0.713 | 229.0888 | | | | 3.48E-05 | 0.49 | 1.00 | 1.36E-02 | 0.56 | 1.68 | 7.04E-04 | 0.77 | 1.04 | 1.47E-04 | 0.22 | 1.19 | 24422 | 54477 | 238681 | 486165 | 117976 | 149521 | 359386 | 652262 |
| RP_POS_807_96405a10_325 | RP | POS | 10.33 | 807.9641 | | | | 4.47E-04 | -0.86 | 1.54 | 1.36E-02 | -0.41 | 1.72 | 5.03E-05 | -1.06 | 1.69 | 8.26E-03 | -0.65 | 1.22 | 1693898 | 722945 | 1168580 | 513329 | 1294240 | 486841 | 1042921 | 512983 |
| RP_NEG_986_7937a0_49 | RP | NEG | 0.49 | 986.7937 | | | | 3.43E-03 | -0.51 | 0.92 | 1.39E-02 | 0.51 | 1.53 | 2.26E-01 | -0.25 | 0.56 | 6.67E-05 | -0.76 | 1.37 | 724298 | 237632 | 562409 | 331397 | 481426 | 276788 | 643393 | 363172 |
| RP_POS_411_759a13_588 | RP | POS | 13.59 | 411.7590 | | | | 4.88E-02 | -0.37 | 0.78 | 1.40E-02 | -0.50 | 1.44 | 2.66E-03 | -0.62 | 1.10 | 5.89E-01 | -0.12 | 0.43 | 1705112 | 566100 | 1462007 | 678868 | 1627634 | 718441 | 1296380 | 599156 |
| RP_POS_1087_67004a10_31 | RP | POS | 10.31 | 1087.6700 | | | | 6.45E-01 | 0.10 | 0.80 | 1.40E-02 | -0.48 | 1.76 | 5.70E-01 | -0.14 | 0.50 | 1.42E-01 | 0.34 | 0.87 | 4268638 | 2726805 | 4519171 | 2448794 | 5116549 | 2144773 | 3921792 | 2605230 |
| RP_NEG_182_04524a0_714 | RP | NEG | 0.71 | 182.0452 | | | | 1.59E-03 | 0.37 | 1.11 | 1.40E-02 | 0.56 | 1.70 | 4.49E-03 | 0.65 | 1.40 | 1.74E-02 | 0.09 | 0.96 | 124676 | 85193 | 2643680 | 7753882 | 724200 | 1718948 | 4563161 | 10535577 |
| RP_NEG_590_34711a10_518 | RP | NEG | 10.52 | 590.3471 | | | | 1.39E-02 | 0.48 | 1.00 | 1.42E-02 | -0.49 | 1.44 | 2.79E-01 | 0.23 | 0.63 | 8.68E-04 | 0.72 | 1.22 | 11703401 | 3465987 | 13540214 | 3850114 | 14477072 | 3593747 | 12603356 | 3904231 |
| RP_NEG_504_31015a10_325 | RP | NEG | 10.33 | 504.3102 | | | | 2.34E-02 | -0.52 | 1.17 | 1.44E-02 | -0.45 | 1.68 | 3.49E-03 | -0.74 | 1.26 | 2.07E-01 | -0.30 | 0.86 | 3609918 | 988166 | 3159305 | 796778 | 3352953 | 702122 | 2965657 | 844490 |
| RP_NEG_213_14952a8_782 | RP | NEG | 8.78 | 213.1495 | | | | 3.66E-07 | 0.75 | 1.69 | 1.44E-02 | 0.51 | 1.82 | 9.79E-07 | 1.00 | 1.74 | 1.67E-03 | 0.49 | 1.51 | 539212 | 506378 | 1285921 | 1049162 | 1030761 | 860016 | 1541080 | 1162519 |
| HILIC_NEG_132_92365a6_093 | HILIC | NEG | 6.093 | 132.9237 | | | | 2.40E-01 | -0.22 | 0.79 | 1.44E-02 | -0.50 | 1.35 | 3.08E-02 | -0.47 | 0.78 | 8.74E-01 | 0.03 | 0.73 | 1416004 | 325695 | 1333540 | 390005 | 1428229 | 361782 | 1238852 | 397654 |
| RP_POS_472_94687a10_708 | RP | POS | 10.71 | 472.9469 | | | | 2.11E-01 | -0.25 | 0.79 | 1.46E-02 | 0.49 | 1.43 | 9.85E-01 | 0.00 | 0.54 | 2.27E-02 | -0.49 | 1.23 | 503925 | 162199 | 461663 | 171313 | 420104 | 152564 | 503221 | 180262 |
| HILIC_POS_414_32086a0_857 | HILIC | POS | 0.857 | 414.3209 | | | | 4.05E-10 | 0.90 | 1.57 | 1.47E-02 | 0.50 | 1.50 | 5.87E-09 | 1.15 | 1.96 | 5.16E-05 | 0.66 | 1.34 | 244481 | 126916 | 488234 | 276574 | 421215 | 244502 | 555253 | 292575 |
| RP_POS_269_22601a11_598 | RP | POS | 11.60 | 269.2260 | | | | 5.01E-01 | -0.10 | 0.57 | 1.47E-02 | -0.54 | 1.60 | 7.46E-02 | -0.37 | 0.74 | 2.75E-01 | 0.17 | 0.64 | 1597896 | 303105 | 1542851 | 615572 | 1692287 | 474199 | 1393416 | 703671 |
| HILIC_POS_184_0737a1_303 | HILIC | POS | 1.303 | 184.0737 | | | | 4.02E-01 | 0.17 | 0.40 | 1.49E-02 | -0.49 | 1.31 | 7.46E-01 | -0.07 | 0.22 | 6.67E-02 | 0.41 | 0.68 | 968652 | 300698 | 1020461 | 306171 | 1094467 | 293825 | 946455 | 303124 |
| RP_POS_204_12276a0_644 | RP | POS | 0.64 | 204.1228 | | | | 2.19E-09 | 0.91 | 1.60 | 1.51E-02 | 0.49 | 1.43 | 2.95E-08 | 1.16 | 1.82 | 2.65E-05 | 0.66 | 1.43 | 100345344 | 29038109 | 149933949 | 55369613 | 136551356 | 44467282 | 163316543 | 62058992 |
| RP_POS_559_51782a14_005 | RP | POS | 14.01 | 559.5178 | | | | 3.14E-01 | 0.24 | 0.60 | 1.51E-02 | -0.44 | 1.67 | 9.17E-01 | 0.03 | 0.59 | 7.86E-02 | 0.46 | 0.77 | 15999089 | 3163370 | 16613861 | 2273137 | 17162338 | 2321170 | 16065384 | 2106655 |
| HILIC_NEG_175_04192a0_927 | HILIC | NEG | 0.927 | 175.0419 | | | | 3.59E-01 | 0.13 | 0.22 | 1.52E-02 | -0.53 | 1.45 | 4.21E-01 | -0.13 | 0.67 | 4.26E-02 | 0.40 | 0.80 | 307984 | 97457 | 332334 | 200544 | 380688 | 217359 | 283980 | 170997 |
| RP_NEG_394_01221a2_884 | RP | NEG | 2.88 | 394.0122 | | | | 6.66E-07 | -0.79 | 1.50 | 1.53E-02 | -0.50 | 1.64 | 1.54E-06 | -1.04 | 1.70 | 5.78E-04 | -0.55 | 1.39 | 825744 | 129518 | 656146 | 219409 | 709119 | 162740 | 603172 | 255053 |
| HILIC_NEG_231_93166a6_094 | HILIC | NEG | 6.094 | 231.9317 | | | | 6.91E-01 | -0.06 | 0.88 | 1.53E-02 | -0.52 | 1.38 | 1.05E-01 | -0.33 | 0.65 | 2.96E-01 | 0.20 | 0.78 | 1249825 | 253674 | 1225911 | 399129 | 1321988 | 367463 | 1129834 | 409867 |
| RP_POS_417_33597a10_092 | RP | POS | 10.09 | 417.3360 | | | | 9.42E-07 | 0.69 | 1.13 | 1.54E-02 | 0.51 | 1.52 | 3.84E-06 | 0.95 | 1.46 | 3.21E-03 | 0.44 | 0.98 | 610042 | 175708 | 874640 | 406273 | 776688 | 319384 | 972591 | 460323 |
| RP_POS_282_11905a0_709 | RP | POS | 0.71 | 282.1191 | | | | 1.24E-02 | 0.39 | 0.88 | 1.54E-02 | 0.52 | 1.46 | 1.44E-03 | 0.65 | 1.27 | 4.50E-01 | 0.13 | 0.67 | 616712 | 80199 | 667772 | 141545 | 633698 | 122388 | 701845 | 152087 |
| RP_NEG_526_31641a10_112 | RP | NEG | 10.11 | 526.3164 | | | | 9.99E-09 | -1.21 | 2.07 | 1.55E-02 | -0.41 | 1.62 | 2.43E-10 | -1.41 | 2.32 | 6.66E-06 | -1.00 | 1.71 | 1402667 | 409573 | 838207 | 399054 | 934291 | 448100 | 742123 | 319537 |
| RP_NEG_221_06644a0_71 | RP | NEG | 0.71 | 221.0664 | | | | 3.35E-06 | 0.56 | 1.04 | 1.55E-02 | 0.54 | 1.70 | 6.52E-05 | 0.83 | 1.29 | 1.07E-02 | 0.29 | 0.85 | 575985 | 551993 | 2625310 | 4067432 | 1644030 | 2771691 | 3606591 | 4876294 |
| RP_NEG_586_31592a10_032 | RP | NEG | 10.03 | 586.3159 | | | | 1.06E-01 | 0.35 | 0.99 | 1.56E-02 | -0.46 | 1.47 | 6.02E-01 | 0.12 | 0.54 | 1.55E-02 | 0.58 | 1.08 | 13386445 | 10091489 | 16675127 | 8988052 | 18832829 | 8923701 | 14517424 | 8607264 |

**Supplementary table 1: Metabolomics results** (in asceding order according to p-value from progressive ALD vs non-progressive ALD comparison, with molecular features with ID_level of 1-2 first)

| | | | | | | | | all_ALD_vs_control | | | Prog_ALD_vs_non-prog_ALD | | | Prog_ALD_vs_control_ALD | | | Non-prog_ALD_vs_control | | | Healthy_control | | all_ALD | | Non-prog_ALD | | Progressive_ALD | |
|---|---|---|---|---|---|---|---|---|---|---|---|---|---|---|---|---|---|---|---|---|---|---|---|---|---|---|---|
| | | | | | | | | t-test | Cohen's | PLS-DA | t-test | Cohen's | PLS-DA | t-test | Cohen's | PLS-DA | t-test | Cohen's | PLS-DA | | | | | | | | |
| Feature_ID | RP_HILIC | POS_NEG | Average.Rt | Average.Mz | Metabolite.name | curated_MSMS | ID_level | p | d | VIP | p | d | VIP | p | d | VIP | p | d | VIP | Mean | SD | Mean | SD | Mean | SD | Mean | SD |
| RP_POS_480_30835a10_627 | RP | POS | 10.63 | 480.3084 | | | | 6.43E-03 | 0.49 | 1.22 | 1.57E-02 | 0.50 | 1.83 | 1.24E-03 | 0.74 | 1.28 | 1.71E-01 | 0.25 | 1.00 | 29830240 | 11465595 | 36870138 | 14662252 | 33342993 | 10781898 | 40397282 | 17108830 |
| RP_POS_430_7482a12_957 | RP | POS | 12.96 | 430.7482 | | | | 2.68E-11 | -1.11 | 2.00 | 1.57E-02 | -0.46 | 1.50 | 1.84E-11 | -1.34 | 2.29 | 1.32E-06 | -0.88 | 1.68 | 11923276 | 1518641 | 9173081 | 2349414 | 9736431 | 2264644 | 8609732 | 2318588 |
| RP_POS_367_18271a10_823 | RP | POS | 10.82 | 367.1827 | | | | 2.23E-12 | 0.95 | 1.79 | 1.58E-02 | 0.49 | 1.62 | 2.87E-09 | 1.20 | 1.98 | 2.12E-06 | 0.71 | 1.72 | 150682 | 133317 | 486811 | 362609 | 399802 | 298677 | 573820 | 401243 |
| RP_POS_289_63089a10_198 | RP | POS | 10.20 | 289.6309 | | | | 2.07E-02 | -0.55 | 1.23 | 1.59E-02 | -0.43 | 1.69 | 3.06E-03 | -0.76 | 1.30 | 1.74E-01 | -0.33 | 0.92 | 1886027 | 694246 | 1565262 | 527219 | 1691527 | 493525 | 1438997 | 534312 |
| HILIC_NEG_157_90068a3_677 | HILIC | NEG | 3.677 | 157.9007 | | | | 4.61E-01 | 0.16 | 0.35 | 1.59E-02 | 0.46 | 1.77 | 1.02E-01 | 0.39 | 0.83 | 7.90E-01 | -0.06 | 0.25 | 1597135 | 930605 | 1732611 | 788028 | 1543919 | 793543 | 1921303 | 743152 |
| HILIC_NEG_194_9884a0_928 | HILIC | NEG | 0.928 | 194.9884 | | | | 3.59E-02 | -0.45 | 1.08 | 1.59E-02 | -0.46 | 1.40 | 3.42E-03 | -0.68 | 1.19 | 3.44E-01 | -0.22 | 0.42 | 1214232 | 236095 | 1112983 | 214440 | 1164310 | 223459 | 1061655 | 193899 |
| RP_NEG_348_00562a2_887 | RP | NEG | 2.89 | 348.0056 | | | | 2.05E-07 | -0.83 | 1.55 | 1.60E-02 | -0.49 | 1.62 | 7.57E-07 | -1.08 | 1.72 | 2.01E-04 | -0.59 | 1.48 | 897664 | 139029 | 706531 | 234388 | 762786 | 171258 | 650277 | 274242 |
| RP_POS_218_13808a1_423 | RP | POS | 1.42 | 218.1381 | | | | 2.43E-02 | -0.43 | 0.97 | 1.60E-02 | -0.49 | 1.45 | 1.93E-03 | -0.67 | 1.14 | 3.77E-01 | -0.19 | 0.84 | 4362831 | 1053082 | 3848999 | 1215115 | 4139576 | 1192316 | 3558422 | 1178646 |
| RP_POS_757_6217a13_673 | RP | POS | 13.67 | 757.6217 | | | | 8.54E-08 | -1.32 | 2.24 | 1.60E-02 | -0.35 | 1.41 | 7.52E-09 | -1.49 | 2.29 | 2.55E-06 | -1.14 | 1.92 | 2383610 | 924688 | 1243088 | 637628 | 1395564 | 622725 | 1090612 | 621453 |
| RP_POS_307_09714a7_667 | RP | POS | 7.67 | 307.0971 | Unknown | 307.0947(100) | 4 | 8.70E-05 | -0.74 | 1.15 | 1.61E-02 | -0.47 | 1.57 | 9.51E-06 | -0.98 | 1.55 | 1.07E-02 | -0.51 | 0.90 | 1230642 | 72540 | 1165790 | 85756 | 1186293 | 77968 | 1145288 | 89003 |
| RP_POS_1009_62012a10_123 | RP | POS | 10.12 | 1009.6201 | | | | 5.32E-05 | 0.48 | 1.73 | 1.61E-02 | 0.54 | 1.49 | 5.10E-04 | 0.75 | 1.44 | 2.50E-02 | 0.21 | 1.39 | 115460 | 45936 | 478792 | 856775 | 272480 | 476976 | 685104 | 1081080 |
| RP_NEG_884_80939a0_489 | RP | NEG | 0.49 | 884.8094 | | | | 9.35E-07 | -0.69 | 1.20 | 1.63E-02 | 0.51 | 1.55 | 1.18E-02 | -0.44 | 0.92 | 2.27E-07 | -0.95 | 1.59 | 1453809 | 259217 | 1062856 | 600582 | 919546 | 576905 | 1206165 | 595001 |
| RP_POS_379_28143a10_961 | RP | POS | 10.96 | 379.2814 | | | | 2.21E-05 | 0.51 | 1.00 | 1.63E-02 | 0.54 | 1.51 | 3.17E-04 | 0.78 | 1.15 | 9.68E-03 | 0.24 | 0.95 | 3485387 | 2357394 | 10391855 | 15029442 | 6778423 | 8203313 | 14005286 | 19037781 |
| RP_POS_532_27985a10_124 | RP | POS | 10.12 | 532.2799 | | | | 9.24E-19 | 1.12 | 2.01 | 1.63E-02 | 0.48 | 1.53 | 1.72E-10 | 1.36 | 2.07 | 5.56E-12 | 0.88 | 2.03 | 126360 | 86253 | 584081 | 407825 | 486405 | 280211 | 681757 | 487902 |
| HILIC_NEG_100_93347a4_019 | HILIC | NEG | 4.019 | 100.9335 | | | | 7.90E-02 | -0.33 | 0.79 | 1.64E-02 | -0.49 | 1.41 | 1.07E-02 | -0.57 | 1.01 | 6.79E-01 | -0.08 | 0.24 | 24259981 | 3966759 | 22745385 | 4768721 | 23882824 | 4062166 | 21607947 | 5177255 |
| RP_POS_410_76151a13_071 | RP | POS | 13.07 | 410.7615 | | | | 7.82E-01 | -0.05 | 0.54 | 1.64E-02 | -0.49 | 1.41 | 1.86E-01 | -0.30 | 1.14 | 3.82E-01 | 0.19 | 0.41 | 4932500 | 1044681 | 4872339 | 1127397 | 5140926 | 1047783 | 4603751 | 1150181 |
| RP_POS_807_56879a13_293 | RP | POS | 13.29 | 807.5688 | | | | 1.96E-02 | -0.41 | 0.85 | 1.65E-02 | 0.50 | 1.46 | 4.55E-01 | -0.16 | 0.64 | 5.80E-04 | -0.66 | 1.18 | 55868771 | 8636414 | 51257142 | 11816567 | 48441352 | 9813642 | 54072933 | 13023961 |
| RP_POS_762_96265a10_123 | RP | POS | 10.12 | 762.9627 | | | | 1.88E-07 | 0.62 | 1.80 | 1.66E-02 | 0.53 | 1.49 | 2.77E-05 | 0.89 | 1.59 | 7.82E-04 | 0.36 | 1.44 | 66604 | 30317 | 366804 | 533696 | 239153 | 339033 | 494456 | 653666 |
| HILIC_NEG_129_03648a0_927 | HILIC | NEG | 0.927 | 129.0365 | | | | 6.67E-02 | 0.28 | 0.47 | 1.66E-02 | -0.52 | 1.52 | 9.29E-01 | 0.02 | 0.44 | 4.10E-03 | 0.54 | 1.08 | 1911195 | 706391 | 2251628 | 1346523 | 2571924 | 1309680 | 1931332 | 1318569 |
| RP_POS_572_29913a10_303 | RP | POS | 10.30 | 572.2991 | | | | 1.79E-01 | 0.28 | 0.69 | 1.66E-02 | -0.47 | 1.69 | 8.58E-01 | 0.04 | 0.31 | 1.91E-02 | 0.51 | 0.95 | 655585 | 214216 | 714526 | 209768 | 764468 | 173457 | 664584 | 231788 |
| HILIC_POS_199_14436a0_568 | HILIC | POS | 0.568 | 199.1444 | | | | 8.90E-05 | 0.46 | 0.89 | 1.70E-02 | 0.54 | 1.37 | 7.86E-04 | 0.73 | 1.14 | 2.43E-02 | 0.19 | 0.68 | 66652 | 113661 | 2200428 | 5217859 | 952649 | 2692520 | 3448207 | 6676873 |
| HILIC_POS_591_31738a1_146 | HILIC | POS | 1.146 | 591.3174 | | | | 2.81E-03 | 0.41 | 0.92 | 1.71E-02 | 0.53 | 1.56 | 3.06E-03 | 0.68 | 0.93 | 1.56E-01 | 0.15 | 0.74 | 524860 | 1216000 | 1672139 | 3088461 | 933195 | 1322152 | 2411083 | 4050852 |
| HILIC_POS_510_35641a1_264 | HILIC | POS | 1.264 | 510.3564 | | | | 4.76E-06 | -0.93 | 1.76 | 1.72E-02 | -0.44 | 1.37 | 1.89E-07 | -1.15 | 2.18 | 1.13E-03 | -0.71 | 1.37 | 9213077 | 2616829 | 6488554 | 2701715 | 7128146 | 2855946 | 5848961 | 2399210 |
| HILIC_POS_568_33966a1_187 | HILIC | POS | 1.187 | 568.3397 | | | | 9.14E-01 | 0.02 | 0.39 | 1.72E-02 | -0.49 | 1.35 | 2.90E-01 | -0.22 | 0.45 | 2.30E-01 | 0.26 | 0.44 | 13815626 | 6513071 | 13963799 | 7413361 | 15718052 | 7577023 | 12209545 | 6880824 |
| RP_POS_862_79767a0_491 | RP | POS | 0.49 | 862.7977 | | | | 6.00E-06 | -0.69 | 1.17 | 1.73E-02 | 0.50 | 1.52 | 1.72E-02 | -0.44 | 0.84 | 4.76E-07 | -0.94 | 1.55 | 1226839 | 265305 | 897852 | 504240 | 778602 | 472738 | 1017102 | 511080 |
| RP_NEG_636_2608a10_199 | RP | NEG | 10.20 | 636.2608 | | | | 5.08E-02 | -0.46 | 1.15 | 1.74E-02 | -0.43 | 1.63 | 8.56E-03 | -0.68 | 1.19 | 3.15E-01 | -0.25 | 0.87 | 1413032 | 495882 | 1221227 | 377966 | 1310486 | 355001 | 1131968 | 382553 |
| RP_POS_363_16211a10_943 | RP | POS | 10.94 | 363.1621 | | | | 9.63E-06 | 0.78 | 1.59 | 1.75E-02 | 0.47 | 1.48 | 3.60E-06 | 1.02 | 1.67 | 2.38E-03 | 0.55 | 1.44 | 722797 | 443530 | 1194137 | 601497 | 1051952 | 487944 | 1336321 | 672036 |
| RP_POS_271_65808a10_198 | RP | POS | 10.20 | 271.6581 | | | | 3.74E-02 | -0.49 | 1.19 | 1.77E-02 | -0.43 | 1.70 | 6.14E-03 | -0.71 | 1.24 | 2.54E-01 | -0.28 | 0.89 | 997022 | 378804 | 840484 | 287290 | 908147 | 269612 | 772820 | 291069 |
| RP_POS_161_98155a15_628 | RP | POS | 15.63 | 161.9816 | | | | 3.66E-01 | 0.18 | 0.65 | 1.78E-02 | -0.48 | 1.39 | 7.75E-01 | -0.06 | 0.42 | 5.83E-02 | 0.42 | 0.70 | 9725622 | 3654698 | 10416137 | 3969731 | 11350905 | 3840516 | 9481369 | 3912330 |
| HILIC_POS_154_05872a5_591 | HILIC | POS | 5.591 | 154.0587 | | | | 1.54E-01 | 0.29 | 0.51 | 1.78E-02 | -0.47 | 1.58 | 8.20E-01 | 0.05 | 0.54 | 1.86E-02 | 0.52 | 0.93 | 2996643 | 1784974 | 3525560 | 1846893 | 3960301 | 1726038 | 3090818 | 1877957 |
| RP_POS_185_11481a13_641 | RP | POS | 13.64 | 185.1148 | | | | 1.19E-01 | 0.33 | 0.71 | 1.79E-02 | -0.46 | 1.68 | 6.60E-01 | 0.10 | 0.20 | 1.79E-02 | 0.56 | 1.09 | 7496618 | 3807039 | 8706083 | 3588338 | 9550079 | 3610625 | 7862086 | 3394816 |
| RP_NEG_554_29889a10_328 | RP | NEG | 10.33 | 554.2989 | | | | 1.14E-03 | -0.75 | 1.38 | 1.79E-02 | -0.42 | 1.55 | 1.36E-04 | -0.96 | 1.57 | 2.17E-02 | -0.54 | 1.07 | 4591511 | 844805 | 4021669 | 673029 | 4179942 | 632089 | 3863396 | 681557 |
| RP_POS_546_79639a10_326 | RP | POS | 10.33 | 546.7964 | | | | 1.63E-03 | -0.76 | 1.45 | 1.79E-02 | -0.41 | 1.69 | 2.48E-04 | -0.96 | 1.57 | 2.19E-02 | -0.56 | 1.14 | 3531944 | 1049565 | 2855455 | 770763 | 3036788 | 679841 | 2674121 | 819136 |
| RP_POS_508_37546a11_094 | RP | POS | 11.09 | 508.3755 | | | | 3.21E-04 | -0.93 | 1.65 | 1.79E-02 | -0.37 | 1.84 | 4.33E-05 | -1.11 | 1.70 | 4.35E-03 | -0.74 | 1.33 | 2040421 | 850368 | 1410925 | 535326 | 1536799 | 530388 | 1285051 | 515122 |
| HILIC_POS_532_2807a1_342 | HILIC | POS | 1.342 | 532.2807 | | | | 2.39E-17 | 1.14 | 1.88 | 1.80E-02 | 0.46 | 1.28 | 1.01E-10 | 1.37 | 2.12 | 1.39E-11 | 0.91 | 2.23 | 151712 | 94243 | 457828 | 263045 | 395757 | 183011 | 519899 | 313753 |
| RP_NEG_706_85394a0_49 | RP | NEG | 0.49 | 706.8539 | | | | 1.07E-02 | -0.39 | 0.73 | 1.80E-02 | 0.51 | 1.48 | 5.25E-01 | -0.13 | 0.28 | 9.17E-05 | -0.64 | 1.28 | 1743780 | 286050 | 1550949 | 543024 | 1423025 | 418377 | 1678874 | 622359 |
| RP_POS_163_05981a0_551 | RP | POS | 0.55 | 163.0598 | | | | 1.55E-03 | 0.39 | 0.72 | 1.80E-02 | 0.54 | 1.55 | 3.45E-03 | 0.66 | 1.03 | 1.15E-01 | 0.12 | 0.94 | 648072 | 106533 | 821736 | 502072 | 702429 | 200821 | 941043 | 663263 |
| RP_POS_184_06007a0_716 | RP | POS | 0.72 | 184.0601 | | | | 5.70E-03 | 0.32 | 1.14 | 1.81E-02 | 0.54 | 1.69 | 1.01E-02 | 0.59 | 1.38 | 2.78E-03 | 0.05 | 1.57 | 26765 | 35659 | 1862303 | 6494508 | 312721 | 640757 | 3411886 | 8939076 |
| RP_POS_276_63242a10_375 | RP | POS | 10.38 | 276.6324 | | | | 9.55E-01 | 0.01 | 0.97 | 1.81E-02 | -0.48 | 1.68 | 3.09E-01 | -0.23 | 0.76 | 2.45E-01 | 0.25 | 0.93 | 1912647 | 272097 | 1915815 | 294328 | 1984923 | 272204 | 1846707 | 301966 |
| RP_NEG_740_84741a0_49 | RP | NEG | 0.49 | 740.8474 | | | | 5.14E-01 | -0.10 | 0.67 | 1.83E-02 | 0.52 | 1.58 | 4.38E-01 | 0.16 | 0.52 | 3.12E-02 | -0.36 | 1.02 | 8161315 | 952215 | 8001460 | 1769497 | 7586161 | 1421491 | 8416759 | 1988345 |
| HILIC_NEG_200_85857a1_004 | HILIC | NEG | 1.004 | 200.8586 | | | | 1.01E-01 | -0.27 | 0.68 | 1.84E-02 | -0.50 | 1.48 | 1.07E-02 | -0.52 | 0.87 | 9.26E-01 | -0.02 | 0.07 | 1285731 | 216293 | 1200873 | 339163 | 1280319 | 308332 | 1121428 | 352791 |
| HILIC_POS_202_84023a2_967 | HILIC | POS | 2.967 | 202.8402 | | | | 4.81E-05 | 0.63 | 1.18 | 1.85E-02 | 0.50 | 1.36 | 5.70E-05 | 0.88 | 1.29 | 1.12E-02 | 0.38 | 0.95 | 274285 | 312619 | 609284 | 559847 | 477793 | 392173 | 740774 | 666460 |
| RP_NEG_1083_66467a10_324 | RP | NEG | 10.32 | 1083.6647 | | | | 1.35E-02 | -0.60 | 1.26 | 1.86E-02 | -0.41 | 1.66 | 2.06E-03 | -0.80 | 1.34 | 1.17E-01 | -0.39 | 0.96 | 19920394 | 8503167 | 15726486 | 6198648 | 17176086 | 5923237 | 14276886 | 6185260 |
| HILIC_NEG_182_04585a0_926 | HILIC | NEG | 0.926 | 182.0459 | | | | 3.28E-02 | 0.26 | 0.73 | 1.86E-02 | 0.54 | 1.42 | 2.11E-02 | 0.53 | 0.95 | 9.09E-01 | -0.01 | 0.19 | 121312 | 227316 | 359209 | 1026051 | 115748 | 192948 | 602669 | 1403158 |
| RP_NEG_632_32227a10_198 | RP | NEG | 10.20 | 632.3223 | | | | 3.56E-02 | -0.50 | 1.18 | 1.91E-02 | -0.42 | 1.62 | 6.18E-03 | -0.71 | 1.23 | 2.36E-01 | -0.29 | 0.88 | 5545369 | 1888650 | 4758550 | 1410750 | 5087212 | 1310090 | 4429888 | 1443699 |
| RP_POS_237_14616a8_784 | RP | POS | 8.78 | 237.1462 | | | | 1.26E-07 | 0.75 | 1.63 | 1.91E-02 | 0.49 | 1.82 | 9.20E-07 | 1.00 | 1.72 | 9.80E-04 | 0.51 | 1.48 | 980108 | 275336 | 1427961 | 629886 | 1281076 | 517062 | 1574846 | 700161 |
| HILIC_POS_593_33301a1_134 | HILIC | POS | 1.134 | 593.3330 | | | | 3.60E-03 | 0.48 | 0.98 | 1.91E-02 | 0.50 | 1.36 | 1.43E-03 | 0.73 | 1.22 | 1.34E-01 | 0.23 | 0.78 | 340456 | 974409 | 1030188 | 1526430 | 672860 | 959246 | 1387516 | 1877977 |
| HILIC_POS_58_06563a3_7 | HILIC | POS | 3.7 | 58.0656 | | | | 8.28E-04 | 0.53 | 1.12 | 1.92E-02 | 0.50 | 1.38 | 2.61E-04 | 0.78 | 1.43 | 8.90E-02 | 0.28 | 0.71 | 1944544 | 929545 | 2739627 | 1609222 | 2364605 | 1275032 | 3114648 | 1821898 |
| HILIC_POS_542_3233a1_263 | HILIC | POS | 1.263 | 542.3233 | | | | 1.74E-01 | 0.28 | 0.54 | 1.93E-02 | -0.46 | 1.26 | 8.15E-01 | 0.05 | 0.15 | 2.83E-02 | 0.51 | 0.90 | 13062776 | 5571602 | 14614839 | 5441321 | 15880012 | 5513595 | 13349667 | 5114078 |
| RP_POS_806_46277a10_324 | RP | POS | 10.32 | 806.4628 | | | | 4.58E-04 | -0.85 | 1.48 | 1.95E-02 | -0.39 | 1.41 | 6.30E-05 | -1.04 | 1.63 | 7.75E-03 | -0.65 | 1.18 | 814484 | 402782 | 521630 | 294102 | 589889 | 271117 | 453371 | 302892 |
| HILIC_POS_114_06653a1_344 | HILIC | POS | 1.344 | 114.0665 | | | | 1.61E-01 | -0.33 | 0.82 | 1.96E-02 | -0.43 | 1.39 | 3.77E-02 | -0.54 | 0.83 | 6.42E-01 | -0.11 | 0.44 | 6817202 | 3115266 | 5959056 | 2443572 | 6526648 | 2019746 | 5391465 | 2706862 |
| RP_POS_590_32166a10_193 | RP | POS | 10.19 | 590.3217 | | | | 6.63E-01 | -0.08 | 0.99 | 1.96E-02 | -0.49 | 1.43 | 1.19E-01 | -0.32 | 0.79 | 4.41E-01 | 0.16 | 0.93 | 1350391 | 543586 | 1298940 | 675358 | 1455540 | 679091 | 1142340 | 640557 |
| RP_POS_278_88852a15_459 | RP | POS | 15.46 | 278.8885 | | | | 8.62E-01 | 0.04 | 0.37 | 1.96E-02 | -0.42 | 1.64 | 5.08E-01 | -0.17 | 0.48 | 3.20E-01 | 0.25 | 0.47 | 24383787 | 7495871 | 24634018 | 5492278 | 25907411 | 5231846 | 23360625 | 5501307 |
| RP_POS_314_2327a7_727 | RP | POS | 7.73 | 314.2327 | | | | 5.87E-02 | 0.33 | 0.84 | 1.97E-02 | 0.49 | 1.48 | 9.98E-03 | 0.57 | 1.30 | 6.51E-01 | 0.08 | 0.32 | 3214346 | 258802 | 3326676 | 364537 | 3241989 | 282663 | 3411364 | 417075 |
| RP_POS_390_17505a0_706 | RP | POS | 0.71 | 390.1751 | | | | 1.02E-12 | 0.89 | 1.51 | 1.98E-02 | 0.49 | 1.44 | 2.45E-08 | 1.14 | 1.75 | 2.22E-06 | 0.64 | 1.48 | 50640 | 46277 | 259490 | 248097 | 201916 | 194929 | 317064 | 282132 |
| RP_POS_298_12088a10_031 | RP | POS | 10.03 | 298.1209 | | | | 1.34E-01 | 0.34 | 0.99 | 1.98E-02 | -0.44 | 1.46 | 6.27E-01 | 0.12 | 0.56 | 2.35E-02 | 0.56 | 1.02 | 373660 | 288403 | 459889 | 243719 | 516321 | 240715 | 403458 | 235728 |
| HILIC_POS_116_10729a0_871 | HILIC | POS | 0.871 | 116.1073 | | | | 9.58E-02 | -0.37 | 0.71 | 2.00E-02 | -0.44 | 1.28 | 1.16E-02 | -0.59 | 1.10 | 5.39E-01 | -0.15 | 0.23 | 2270863 | 1302090 | 1836760 | 1104647 | 2092594 | 1231815 | 1580925 | 902469 |
| RP_POS_279_64697a10_198 | RP | POS | 10.20 | 279.6470 | | | | 1.72E-02 | -0.58 | 1.26 | 2.00E-02 | -0.41 | 1.64 | 2.93E-03 | -0.78 | 1.31 | 1.31E-01 | -0.38 | 0.96 | 1133728 | 447796 | 921918 | 319615 | 995820 | 300549 | 848017 | 323908 |
| RP_POS_394_2081a9_967 | RP | POS | 9.97 | 394.2081 | | | | 7.82E-07 | 0.73 | 1.17 | 2.01E-02 | 0.49 | 1.44 | 1.55E-08 | 0.97 | 1.93 | 1.16E-02 | 0.49 | 0.76 | 729119 | 693513 | 1714948 | 1425807 | 1385321 | 1567390 | 2044575 | 1195360 |
| RP_POS_127_0389a0_55 | RP | POS | 0.55 | 127.0389 | | | | 1.78E-03 | 0.39 | 0.72 | 2.02E-02 | 0.53 | 1.53 | 3.76E-03 | 0.65 | 0.99 | 1.31E-01 | 0.12 | 0.95 | 902430 | 131937 | 1110958 | 609467 | 968789 | 259166 | 1253128 | 801288 |
| HILIC_NEG_588_33142a1_16 | HILIC | NEG | 1.16 | 588.3314 | | | | 1.05E-01 | 0.36 | 0.65 | 2.02E-02 | -0.44 | 1.23 | 5.75E-01 | 0.14 | 0.23 | 1.36E-02 | 0.58 | 1.08 | 859795 | 344226 | 971899 | 299864 | 1041158 | 259596 | 902640 | 323252 |
| RP_NEG_593_47961a11_359 | RP | NEG | 11.36 | 593.4796 | | | | 9.39E-02 | -0.31 | 0.57 | 2.03E-02 | -0.48 | 1.44 | 3.58E-03 | -0.55 | 1.23 | 7.63E-01 | -0.07 | 0.65 | 3199275 | 1972330 | 2478041 | 2414732 | 3037225 | 2885144 | 1918857 | 1678890 |
| RP_POS_766_57135a13_684 | RP | POS | 13.68 | 766.5714 | | | | 6.10E-07 | -1.11 | 1.78 | 2.03E-02 | -0.39 | 1.35 | 4.84E-08 | -1.31 | 2.01 | 5.22E-05 | -0.92 | 1.48 | 3012720 | 1256802 | 1585597 | 1089250 | 1836858 | 1077260 | 1334337 | 1052527 |
| RP_POS_746_50861a13_381 | RP | POS | 13.38 | 746.5086 | | | | 5.34E-01 | -0.13 | 0.94 | 2.03E-02 | -0.45 | 1.36 | 9.61E-02 | -0.36 | 0.83 | 7.06E-01 | 0.09 | 0.71 | 1456151 | 816567 | 1354181 | 754434 | 1528820 | 895349 | 1179541 | 534850 |
| RP_NEG_305_24933a11_199 | RP | NEG | 11.20 | 305.2493 | | | | 2.53E-12 | 0.92 | 1.70 | 2.03E-02 | 0.48 | 1.58 | 5.56E-08 | 1.16 | 1.81 | 9.12E-08 | 0.68 | 1.80 | 825345 | 510884 | 2260259 | 1620623 | 1884946 | 1092201 | 2635572 | 1956034 |
| RP_NEG_224_97958a0_713 | RP | NEG | 0.71 | 224.9796 | | | | 9.56E-01 | 0.01 | 0.91 | 2.07E-02 | -0.52 | 1.46 | 1.70E-01 | -0.25 | 0.88 | 1.03E-01 | 0.27 | 1.19 | 2479574 | 228868 | 2483676 | 632129 | 2629100 | 572254 | 2338253 | 660829 |
| HILIC_POS_127_08677a1_355 | HILIC | POS | 1.355 | 127.0868 | | | | 2.63E-06 | 0.56 | 1.30 | 2.07E-02 | 0.52 | 1.28 | 1.27E-04 | 0.82 | 1.17 | 3.18E-03 | 0.30 | 0.89 | 141168 | 29471 | 583358 | 885765 | 378492 | 539870 | 788223 | 1099117 |
| RP_NEG_541_26648a7_01 | RP | NEG | 7.01 | 541.2665 | | | | 2.77E-02 | -0.49 | 0.92 | 2.07E-02 | -0.44 | 1.36 | 2.68E-03 | -0.70 | 1.39 | 2.68E-01 | -0.27 | 0.54 | 1508728 | 951920 | 1082527 | 827343 | 1272903 | 900425 | 892150 | 705856 |
| HILIC_POS_242_07932a2_974 | HILIC | POS | 2.974 | 242.0793 | | | | 5.08E-12 | 1.01 | 1.65 | 2.07E-02 | 0.46 | 1.54 | 6.61E-10 | 1.24 | 1.91 | 1.15E-06 | 0.78 | 1.55 | 13012609 | 6284734 | 26356616 | 13195004 | 23321082 | 11376540 | 29392150 | 14266702 |
| RP_POS_280_0603a0_78 | RP | POS | 0.78 | 280.0603 | | | | 1.48E-02 | 0.30 | 0.94 | 2.08E-02 | 0.52 | 1.43 | 7.40E-03 | 0.56 | 0.88 | 7.56E-01 | 0.03 | 0.59 | 1128873 | 509010 | 1823358 | 2659246 | 1209063 | 1699435 | 2437652 | 3260261 |
| RP_POS_271_22675a10_292 | RP | POS | 10.29 | 271.2268 | | | | 1.90E-01 | -0.22 | 0.87 | 2.08E-02 | 0.49 | 1.41 | 8.88E-01 | 0.03 | 0.64 | 1.50E-02 | -0.47 | 0.99 | 657776 | 63311 | 638272 | 95959 | 616225 | 87709 | 660320 | 99597 |
| HILIC_NEG_222_83035a0_881 | HILIC | NEG | 0.881 | 222.8304 | | | | 1.87E-01 | 0.26 | 0.77 | 2.09E-02 | 0.47 | 1.41 | 3.18E-02 | 0.49 | 0.90 | 9.13E-01 | 0.02 | 0.12 | 204842 | 66642 | 223386 | 73541 | 206485 | 64546 | 240287 | 78598 |
| HILIC_NEG_158_84668a3_003 | HILIC | NEG | 3.003 | 158.8467 | | | | 2.23E-01 | 0.22 | 0.72 | 2.10E-02 | -0.49 | 1.37 | 8.86E-01 | -0.02 | 0.26 | 5.14E-02 | 0.46 | 1.05 | 2541994 | 482108 | 2672013 | 626574 | 2816577 | 774765 | 2527450 | 387674 |
| HILIC_POS_136_0482a5_591 | HILIC | POS | 5.591 | 136.0482 | | | | 1.66E-01 | 0.28 | 0.52 | 2.10E-02 | -0.46 | 1.54 | 8.24E-01 | 0.05 | 0.52 | 2.18E-02 | 0.51 | 0.91 | 1028804 | 597723 | 1200614 | 614598 | 1341589 | 571506 | 1059638 | 629279 |
| RP_POS_300_6228a10_032 | RP | POS | 10.03 | 300.6228 | | | | 8.79E-02 | 0.37 | 1.02 | 2.12E-02 | -0.44 | 1.43 | 5.25E-01 | 0.15 | 0.58 | 1.35E-02 | 0.59 | 1.07 | 463867 | 338763 | 581159 | 307790 | 651683 | 304946 | 510636 | 297086 |
| RP_POS_542_31909a10_325 | RP | POS | 10.33 | 542.3191 | | | | 3.21E-02 | -0.51 | 1.18 | 2.13E-02 | -0.41 | 1.62 | 6.09E-03 | -0.72 | 1.25 | 2.11E-01 | -0.31 | 0.91 | 122428496 | 28944418 | 110124597 | 21608700 | 115072398 | 19351032 | 105176796 | 22781089 |
| RP_POS_255_23143a10_824 | RP | POS | 10.82 | 255.2314 | | | | 5.90E-09 | 0.83 | 1.68 | 2.17E-02 | 0.48 | 1.63 | 1.22E-07 | 1.07 | 1.84 | 1.36E-04 | 0.59 | 1.56 | 583800 | 491153 | 1467012 | 1105527 | 1214351 | 925667 | 1719672 | 1217449 |
| RP_POS_367_21362a11_689 | RP | POS | 11.69 | 367.2136 | | | | 1.06E-09 | 0.90 | 1.78 | 2.17E-02 | 0.47 | 1.49 | 4.29E-08 | 1.14 | 1.87 | 1.55E-05 | 0.67 | 1.66 | 272445 | 203203 | 638820 | 414345 | 544106 | 331219 | 733534 | 467819 |
| HILIC_POS_517_33057a1_407 | HILIC | POS | 1.407 | 517.3306 | | | | 1.84E-02 | 0.29 | 0.56 | 2.17E-02 | 0.53 | 1.51 | 1.68E-02 | 0.55 | 0.75 | 6.54E-01 | 0.02 | 0.18 | 142817 | 420565 | 694011 | 2183364 | 188929 | 498824 | 1199094 | 2976909 |
| HILIC_POS_494_3241a1_339 | HILIC | POS | 1.339 | 494.3241 | | | | 8.85E-17 | 1.09 | 1.78 | 2.18E-02 | 0.46 | 1.24 | 5.55E-10 | 1.32 | 2.01 | 4.32E-11 | 0.86 | 2.13 | 24459837 | 11924044 | 66481482 | 38451949 | 57675560 | 26457791 | 75287405 | 46141207 |
| RP_POS_227_12498a0_552 | RP | POS | 0.55 | 227.1250 | | | | 1.09E-04 | -0.80 | 1.68 | 2.18E-02 | -0.43 | 1.35 | 1.01E-05 | -1.02 | 1.91 | 6.91E-03 | -0.59 | 1.21 | 5565916 | 1646756 | 4167517 | 1636386 | 4540738 | 1584205 | 3794296 | 1617463 |
| HILIC_NEG_128_0401a0_927 | HILIC | NEG | 0.927 | 128.0401 | | | | 7.80E-02 | 0.27 | 0.52 | 2.18E-02 | -0.50 | 1.49 | 9.11E-01 | 0.02 | 0.39 | 5.23E-03 | 0.52 | 1.02 | 462394 | 176986 | 542760 | 325069 | 616894 | 309427 | 468626 | 326397 |
| RP_POS_156_04227a15_444 | RP | POS | 15.44 | 156.0423 | | | | 3.13E-01 | -0.20 | 1.42 | 2.19E-02 | 0.47 | 1.56 | 8.75E-01 | 0.04 | 0.75 | 4.55E-02 | -0.43 | 1.44 | 2563572 | 776428 | 2399141 | 850300 | 2205321 | 775779 | 2592962 | 884463 |
| HILIC_POS_100_05094a1_727 | HILIC | POS | 1.727 | 100.0509 | | | | 6.14E-01 | 0.07 | 0.99 | 2.20E-02 | -0.51 | 1.26 | 3.61E-01 | -0.18 | 0.43 | 3.77E-02 | 0.33 | 0.79 | 441032 | 164193 | 464643 | 365974 | 548148 | 293846 | 381138 | 412348 |
| RP_POS_781_55603a12_575 | RP | POS | 12.58 | 781.5560 | | | | 2.14E-06 | 0.80 | 1.42 | 2.22E-02 | 0.46 | 1.40 | 5.78E-06 | 1.03 | 1.59 | 3.90E-04 | 0.56 | 1.35 | 7227800 | 2713316 | 10459729 | 4122764 | 9518110 | 2694839 | 11401349 | 5026888 |
| HILIC_NEG_229_08868a0_927 | HILIC | NEG | 0.927 | 229.0887 | | | | 3.76E-07 | 0.61 | 1.20 | 2.23E-02 | 0.50 | 1.51 | 2.57E-05 | 0.86 | 1.30 | 2.74E-03 | 0.36 | 0.97 | 33402 | 39738 | 220128 | 337507 | 143210 | 242048 | 297046 | 399357 |
| RP_POS_518_31989a11_609 | RP | POS | 11.61 | 518.3199 | | | | 6.49E-01 | 0.10 | 0.93 | 2.24E-02 | -0.45 | 1.60 | 5.81E-01 | -0.13 | 0.61 | 1.63E-01 | 0.32 | 0.98 | 1096879 | 346190 | 1128687 | 328437 | 1203263 | 310193 | 1054111 | 332224 |
| RP_POS_417_74237a12_566 | RP | POS | 12.57 | 417.7424 | | | | 5.99E-07 | 0.93 | 1.56 | 2.25E-02 | 0.44 | 1.44 | 7.99E-07 | 1.15 | 1.71 | 6.38E-05 | 0.71 | 1.62 | 655273 | 369197 | 1096831 | 456237 | 992855 | 304634 | 1200806 | 552890 |

**Supplementary table 1: Metabolomics results** (in asceding order according to p-value from progressive ALD vs non-progressive ALD comparison, with molecular features with ID_level of 1-2 first)

| Feature_ID | RP_HILIC | POS_NEG | Average.Rt | Average.Mz | Metabolite.name | curated_MSMS | ID_level | all_ALD_vs_control t-test p | all_ALD_vs_control Cohen's d | all_ALD_vs_control PLS-DA VIP | Prog_ALD_vs_non-prog_ALD t-test p | Prog_ALD_vs_non-prog_ALD Cohen's d | Prog_ALD_vs_non-prog_ALD PLS-DA VIP | Prog_ALD_vs_control_ALD t-test p | Prog_ALD_vs_control_ALD Cohen's d | Prog_ALD_vs_control_ALD PLS-DA VIP | Non-prog_ALD_vs_control t-test p | Non-prog_ALD_vs_control Cohen's d | Non-prog_ALD_vs_control PLS-DA VIP | Healthy_control Mean | Healthy_control SD | all_ALD Mean | all_ALD SD | Non-prog_ALD Mean | Non-prog_ALD SD | Progressive_ALD Mean | Progressive_ALD SD |
|---|---|---|---|---|---|---|---|---|---|---|---|---|---|---|---|---|---|---|---|---|---|---|---|---|---|---|---|
| HILIC_NEG_165_05551a0_687 | HILIC | NEG | 0.687 | 165.0555 | | | | 1.70E-04 | 0.47 | 1.12 | 2.25E-02 | 0.51 | 1.64 | 1.45E-03 | 0.72 | 0.97 | 5.80E-03 | 0.21 | 0.93 | 228271 | 96386 | 410438 | 437591 | 310042 | 164524 | 510835 | 582444 |
| RP_POS_312_63242a10_292 | RP | POS | 10.29 | 312.6324 | | | | 4.67E-01 | -0.14 | 0.98 | 2.26E-02 | -0.47 | 1.38 | 8.04E-02 | -0.37 | 0.84 | 6.45E-01 | 0.10 | 0.87 | 1626653 | 526136 | 1544743 | 621416 | 1685683 | 618602 | 1403803 | 597486 |
| RP_POS_587_46381a13_624 | RP | POS | 13.62 | 587.4638 | | | | 1.87E-04 | 0.45 | 0.86 | 2.30E-02 | 0.51 | 1.35 | 1.67E-03 | 0.71 | 1.00 | 5.74E-03 | 0.20 | 0.85 | 276242 | 314007 | 997574 | 1783982 | 589674 | 673009 | 1405474 | 2374371 |
| RP_POS_315_13431a7_11 | RP | POS | 7.11 | 315.1343 | | | | 1.87E-02 | 0.33 | 0.91 | 2.31E-02 | 0.50 | 1.63 | 8.50E-03 | 0.58 | 1.01 | 5.33E-01 | 0.08 | 0.79 | 535358 | 297195 | 746211 | 712083 | 584249 | 407611 | 908173 | 897101 |
| RP_POS_822_53815a13_295 | RP | POS | 13.30 | 822.5382 | | | | 6.07E-02 | -0.37 | 0.73 | 2.31E-02 | 0.45 | 1.38 | 5.00E-01 | -0.15 | 0.55 | 7.80E-03 | -0.60 | 1.04 | 5848419 | 1363150 | 5309959 | 1448696 | 4982680 | 1444454 | 5637238 | 1391126 |
| RP_POS_828_60211a11_162 | RP | POS | 11.16 | 828.6021 | | | | 3.08E-06 | 0.56 | 1.27 | 2.31E-02 | -0.51 | 1.60 | 5.18E-03 | 0.31 | 1.16 | 1.03E-04 | 0.82 | 1.41 | 139888 | 54339 | 340391 | 396185 | 430297 | 482782 | 250485 | 259910 |
| HILIC_NEG_195_81082a0_733 | HILIC | NEG | 0.733 | 195.8108 | | | | 2.59E-06 | 0.57 | 1.10 | 2.32E-02 | 0.50 | 1.24 | 6.24E-05 | 0.82 | 1.24 | 8.07E-03 | 0.32 | 0.82 | 200324 | 98987 | 518528 | 618374 | 378493 | 441679 | 658564 | 733128 |
| RP_NEG_526_31622a10_247 | RP | NEG | 10.25 | 526.3162 | | | | 4.52E-08 | -1.19 | 2.02 | 2.32E-02 | -0.38 | 1.61 | 1.79E-09 | -1.38 | 2.25 | 1.14E-05 | -1.00 | 1.67 | 11615785 | 3391681 | 7251229 | 3082997 | 7948054 | 3451109 | 6554404 | 2510502 |
| RP_NEG_676_30615a10_378 | RP | NEG | 10.38 | 676.3062 | | | | 2.89E-01 | -0.21 | 1.23 | 2.33E-02 | -0.46 | 1.35 | 4.78E-02 | -0.44 | 0.93 | 9.28E-01 | 0.02 | 1.36 | 1752571 | 184937 | 1711592 | 198847 | 1756466 | 196224 | 1666718 | 193064 |
| RP_NEG_267_12448a8_867 | RP | NEG | 8.87 | 267.1245 | | | | 2.37E-05 | -1.04 | 1.77 | 2.33E-02 | -0.36 | 1.42 | 2.14E-06 | -1.22 | 1.92 | 6.80E-04 | -0.86 | 1.44 | 10910017 | 5513259 | 5913757 | 3882256 | 6790296 | 4212465 | 5037218 | 3337448 |
| RP_POS_440_81302a15_449 | RP | POS | 15.45 | 440.8130 | | | | 7.19E-01 | 0.07 | 0.35 | 2.34E-02 | -0.47 | 1.37 | 4.60E-01 | -0.17 | 0.71 | 1.38E-01 | 0.30 | 0.55 | 1987133 | 191419 | 2001802 | 223634 | 2052254 | 192397 | 1951351 | 242556 |
| HILIC_POS_203_13907a4_671 | HILIC | POS | 4.671 | 203.1391 | | | | 1.61E-07 | 0.70 | 1.16 | 2.34E-02 | 0.49 | 1.48 | 5.96E-06 | 0.95 | 1.48 | 7.00E-04 | 0.46 | 1.14 | 2144190 | 978032 | 3957162 | 2776375 | 3329461 | 2031072 | 4584863 | 3262588 |
| RP_POS_289_63144a10_328 | RP | POS | 10.33 | 289.6314 | | | | 6.06E-03 | -0.67 | 1.34 | 2.34E-02 | -0.39 | 1.52 | 1.20E-03 | -0.87 | 1.41 | 5.20E-02 | -0.48 | 1.08 | 5146292 | 953692 | 4621067 | 673195 | 4772912 | 574557 | 4469222 | 733799 |
| RP_POS_522_28308a10_124 | RP | POS | 10.12 | 522.2831 | | | | 3.85E-16 | 1.02 | 1.89 | 2.35E-02 | 0.47 | 1.41 | 4.51E-09 | 1.25 | 1.94 | 3.04E-10 | 0.79 | 1.88 | 285968 | 238587 | 1779141 | 1509670 | 1437569 | 1027260 | 2120712 | 1819712 |
| HILIC_NEG_134_89471a5_468 | HILIC | NEG | 5.468 | 134.8947 | | | | 3.37E-01 | 0.22 | 0.55 | 2.35E-02 | -0.42 | 1.42 | 9.69E-01 | 0.01 | 0.09 | 9.66E-02 | 0.43 | 0.90 | 10051414 | 6126976 | 11204501 | 4898084 | 12310201 | 5564632 | 10098800 | 3874518 |
| RP_POS_284_09827a0_709 | RP | POS | 0.71 | 284.0983 | | | | 1.11E-03 | 0.39 | 1.21 | 2.35E-02 | -0.52 | 1.45 | 1.89E-02 | 0.13 | 1.42 | 4.57E-03 | 0.65 | 1.16 | 162343 | 130502 | 550401 | 1134713 | 809242 | 1531972 | 291560 | 343152 |
| RP_POS_295_22372a10_514 | RP | POS | 10.51 | 295.2237 | | | | 4.65E-01 | -0.15 | 0.92 | 2.35E-02 | 0.45 | 1.49 | 7.72E-01 | 0.07 | 0.64 | 9.31E-02 | -0.38 | 0.89 | 1038140 | 683263 | 937153 | 649160 | 790828 | 564922 | 1083478 | 698989 |
| RP_POS_317_24707a10_853 | RP | POS | 10.85 | 317.2471 | | | | 5.41E-02 | -0.30 | 0.77 | 2.35E-02 | -0.49 | 1.49 | 1.01E-02 | -0.54 | 0.97 | 7.41E-01 | -0.05 | 0.44 | 2033080 | 454559 | 1810830 | 807993 | 1993181 | 631069 | 1628480 | 923536 |
| RP_POS_315_13382a7_304 | RP | POS | 7.30 | 315.1338 | | | | 9.47E-02 | 0.22 | 0.79 | 2.37E-02 | 0.51 | 1.65 | 3.21E-02 | 0.48 | 0.85 | 7.35E-01 | -0.03 | 0.63 | 805619 | 411456 | 1045593 | 1225756 | 767583 | 600312 | 1323603 | 1586666 |
| RP_POS_482_32376a10_248 | RP | POS | 10.25 | 482.3238 | | | | 1.47E-07 | -1.17 | 1.99 | 2.39E-02 | -0.38 | 1.60 | 6.93E-09 | -1.36 | 2.20 | 2.29E-05 | -0.98 | 1.62 | 24276555 | 7456937 | 15206503 | 6490092 | 16666818 | 7211732 | 13746187 | 5361068 |
| RP_POS_454_38867a9_734 | RP | POS | 9.73 | 454.3887 | | | | 1.25E-05 | 0.79 | 1.31 | 2.39E-02 | 0.45 | 1.35 | 4.35E-06 | 1.01 | 1.52 | 2.45E-03 | 0.56 | 1.22 | 387081 | 176106 | 570030 | 232144 | 517822 | 195387 | 622238 | 255231 |
| RP_POS_237_22096a10_823 | RP | POS | 10.82 | 237.2210 | | | | 4.24E-08 | 0.78 | 1.64 | 2.39E-02 | 0.47 | 1.58 | 4.22E-07 | 1.02 | 1.77 | 4.77E-04 | 0.55 | 1.53 | 148502 | 116268 | 343614 | 261457 | 284821 | 220670 | 402406 | 286994 |
| RP_POS_1063_67041a10_316 | RP | POS | 10.32 | 1063.6704 | | | | 8.55E-01 | -0.04 | 0.87 | 2.41E-02 | -0.42 | 1.75 | 3.18E-01 | -0.25 | 0.68 | 4.79E-01 | 0.17 | 0.82 | 20223956 | 13289417 | 19750544 | 10686727 | 22150051 | 9392845 | 17351038 | 11435348 |
| RP_POS_804_54102a12_692 | RP | POS | 12.69 | 804.5410 | | | | 5.52E-03 | 0.55 | 1.22 | 2.42E-02 | -0.45 | 1.38 | 1.32E-01 | 0.32 | 0.79 | 5.51E-04 | 0.77 | 1.32 | 6442410 | 2481055 | 7930790 | 2715326 | 8539472 | 2673493 | 7322107 | 2644388 |
| RP_POS_520_77899a10_124 | RP | POS | 10.12 | 520.7790 | | | | 1.72E-15 | 0.99 | 1.85 | 2.44E-02 | 0.47 | 1.40 | 9.42E-09 | 1.23 | 1.91 | 8.47E-10 | 0.76 | 1.83 | 135178 | 119494 | 932866 | 833792 | 745385 | 565349 | 1120347 | 1006605 |
| RP_POS_814_53406a12_648 | RP | POS | 12.65 | 814.5341 | | | | 1.93E-15 | -1.67 | 2.94 | 2.44E-02 | -0.31 | 1.36 | 7.88E-17 | -1.83 | 2.97 | 3.66E-13 | -1.52 | 2.43 | 1349572 | 390791 | 459513 | 364978 | 541347 | 409448 | 377679 | 296349 |
| RP_POS_287_634a10_198 | RP | POS | 10.20 | 287.6340 | | | | 4.23E-02 | -0.49 | 1.18 | 2.44E-02 | -0.40 | 1.68 | 8.68E-03 | -0.69 | 1.20 | 2.46E-01 | -0.28 | 0.89 | 999399 | 412337 | 833884 | 306064 | 902425 | 275252 | 765343 | 322353 |
| RP_POS_360_16348a5_551 | RP | POS | 5.55 | 360.1635 | | | | 8.90E-02 | -0.26 | 0.74 | 2.45E-02 | -0.49 | 1.34 | 1.35E-02 | -0.51 | 0.88 | 9.16E-01 | -0.02 | 0.72 | 1087392 | 184277 | 1007385 | 332886 | 1081930 | 280984 | 932839 | 365507 |
| RP_NEG_124_99035a0_739 | RP | NEG | 0.74 | 124.9904 | | | | 8.77E-08 | 0.65 | 1.14 | 2.46E-02 | 0.49 | 1.60 | 1.05E-05 | 0.89 | 1.44 | 1.62E-03 | 0.40 | 0.99 | 252080 | 387812 | 2386566 | 3648682 | 1568528 | 2756125 | 3204604 | 4235038 |
| RP_POS_223_09445a5_877 | RP | POS | 5.88 | 223.0945 | Unknown | 223.0948(100) | 4 | 7.71E-01 | -0.06 | 0.58 | 2.46E-02 | 0.45 | 1.54 | 4.64E-01 | 0.17 | 0.70 | 2.05E-01 | -0.28 | 0.64 | 11326891 | 2539576 | 11175096 | 2586137 | 10596907 | 2482307 | 11753284 | 2582365 |
| RP_NEG_612_3316a10_294 | RP | NEG | 10.29 | 612.3316 | | | | 7.30E-01 | -0.06 | 0.96 | 2.47E-02 | -0.47 | 1.36 | 1.49E-01 | -0.30 | 0.74 | 4.04E-01 | 0.17 | 0.91 | 19029455 | 6441113 | 18540961 | 8318020 | 20399365 | 8260115 | 16682558 | 8032007 |
| HILIC_POS_546_35638a1_092 | HILIC | POS | 1.092 | 546.3564 | | | | 5.92E-03 | 0.52 | 1.06 | 2.49E-02 | -0.45 | 1.27 | 1.67E-01 | 0.29 | 0.61 | 4.31E-04 | 0.75 | 1.51 | 2049668 | 842224 | 2559975 | 992293 | 2781401 | 923433 | 2338550 | 1018134 |
| RP_NEG_478_29431a10_626 | RP | NEG | 10.63 | 478.2943 | | | | 5.17E-03 | 0.51 | 1.28 | 2.50E-02 | 0.46 | 1.74 | 1.50E-03 | 0.74 | 1.28 | 1.12E-01 | 0.29 | 1.04 | 19231009 | 8969929 | 24844035 | 11158045 | 22347445 | 7772318 | 27340625 | 13357227 |
| RP_POS_581_27795a10_375 | RP | POS | 10.38 | 581.2780 | | | | 6.30E-01 | 0.09 | 0.94 | 2.51E-02 | -0.46 | 1.61 | 5.40E-01 | -0.14 | 0.69 | 1.27E-01 | 0.32 | 0.96 | 893265 | 127391 | 906205 | 143655 | 938224 | 130183 | 874185 | 150515 |
| RP_NEG_528_26471a10_882 | RP | NEG | 10.88 | 528.2647 | | | | 3.25E-03 | 0.35 | 1.06 | 2.51E-02 | 0.51 | 1.50 | 7.99E-03 | 0.60 | 1.09 | 6.83E-02 | 0.09 | 0.89 | 235778 | 187335 | 727609 | 1599669 | 366662 | 441803 | 1088556 | 2170031 |
| HILIC_POS_341_24319a0_568 | HILIC | POS | 0.568 | 341.2432 | | | | 9.62E-01 | -0.01 | 1.17 | 2.51E-02 | -0.47 | 1.30 | 1.99E-01 | -0.25 | 1.01 | 2.89E-01 | 0.23 | 1.02 | 221564 | 108785 | 220410 | 147544 | 253309 | 160577 | 187511 | 126447 |
| RP_POS_546_7962a10_198 | RP | POS | 10.20 | 546.7962 | | | | 1.93E-02 | -0.60 | 1.26 | 2.53E-02 | -0.37 | 1.58 | 3.66E-03 | -0.78 | 1.31 | 1.21E-01 | -0.41 | 0.97 | 584423 | 399433 | 401121 | 258252 | 458604 | 261206 | 343637 | 244494 |
| HILIC_NEG_197_80795a0_733 | HILIC | NEG | 0.733 | 197.8080 | | | | 3.93E-05 | 0.50 | 1.15 | 2.54E-02 | 0.50 | 1.23 | 2.86E-04 | 0.75 | 1.11 | 3.70E-02 | 0.25 | 0.69 | 299382 | 120075 | 665755 | 828477 | 480831 | 581134 | 850678 | 989571 |
| RP_POS_275_62561a9_984 | RP | POS | 9.98 | 275.6256 | | | | 1.67E-18 | 1.13 | 1.97 | 2.54E-02 | 0.45 | 1.41 | 4.74E-10 | 1.35 | 2.02 | 2.30E-13 | 0.90 | 2.12 | 273590 | 111714 | 771300 | 439714 | 673039 | 279408 | 869560 | 541175 |
| RP_POS_452_27649a10_139 | RP | POS | 10.14 | 452.2765 | | | | 3.24E-17 | 1.06 | 1.90 | 2.56E-02 | 0.45 | 1.40 | 1.01E-09 | 1.28 | 1.98 | 2.98E-10 | 0.83 | 1.86 | 1825393 | 1069967 | 9096250 | 7018789 | 7533307 | 5098534 | 10659194 | 8279603 |
| RP_POS_790_57117a13_587 | RP | POS | 13.59 | 790.5712 | | | | 1.04E-02 | -0.50 | 0.86 | 2.59E-02 | -0.45 | 1.31 | 4.65E-04 | -0.72 | 1.31 | 2.30E-01 | -0.27 | 0.49 | 14985345 | 3939096 | 12802383 | 4414727 | 13782429 | 5015223 | 11822338 | 3502105 |
| RP_NEG_165_0551a4_826 | RP | NEG | 4.83 | 165.0551 | | | | 2.73E-02 | 0.33 | 0.72 | 2.61E-02 | 0.49 | 1.40 | 1.17E-02 | 0.58 | 0.86 | 4.86E-01 | 0.09 | 0.41 | 1343041 | 781307 | 1797444 | 1486157 | 1465929 | 762080 | 2128960 | 1912417 |
| HILIC_NEG_224_82733a0_881 | HILIC | NEG | 0.881 | 224.8273 | | | | 1.99E-01 | 0.25 | 0.77 | 2.62E-02 | 0.45 | 1.36 | 3.95E-02 | 0.48 | 0.88 | 8.98E-01 | 0.03 | 0.06 | 192113 | 64202 | 209463 | 70110 | 193940 | 59788 | 224985 | 76582 |
| HILIC_POS_170_03275a5_591 | HILIC | POS | 5.591 | 170.0328 | | | | 2.39E-01 | 0.24 | 0.54 | 2.62E-02 | -0.44 | 1.42 | 9.41E-01 | 0.02 | 0.35 | 4.05E-02 | 0.46 | 0.82 | 787179 | 444720 | 895292 | 454795 | 995889 | 435132 | 794696 | 455966 |
| RP_POS_631_14099a9_21 | RP | POS | 9.21 | 631.1410 | | | | 4.00E-01 | 0.15 | 0.55 | 2.65E-02 | 0.47 | 1.32 | 9.54E-02 | 0.38 | 0.87 | 6.42E-01 | -0.08 | 0.43 | 1790624 | 139955 | 1816686 | 182630 | 1776228 | 130178 | 1857145 | 217029 |
| RP_POS_269_13846a8_87 | RP | POS | 8.87 | 269.1385 | (E)-2-decylpent-2-enedioic acid | 209.1155(100) | 3 | 3.91E-05 | -1.04 | 1.76 | 2.65E-02 | -0.35 | 1.40 | 4.28E-06 | -1.21 | 1.89 | 8.03E-04 | -0.87 | 1.42 | 1786187 | 942248 | 964415 | 620816 | 1101591 | 677473 | 827239 | 530395 |
| RP_NEG_850_81525a0_49 | RP | NEG | 0.49 | 850.8153 | | | | 3.80E-07 | -0.74 | 1.24 | 2.67E-02 | 0.47 | 1.45 | 6.72E-03 | -0.50 | 0.95 | 3.98E-08 | -0.97 | 1.66 | 1829811 | 287241 | 1397405 | 620210 | 1260629 | 553905 | 1534181 | 657404 |
| RP_POS_794_81079a0_492 | RP | POS | 0.49 | 794.8108 | | | | 1.72E-06 | -0.69 | 1.20 | 2.67E-02 | 0.47 | 1.48 | 1.03E-02 | -0.46 | 0.93 | 4.86E-07 | -0.93 | 1.58 | 1759521 | 315182 | 1313332 | 686513 | 1161994 | 658237 | 1464670 | 687170 |
| RP_POS_568_33972a10_296 | RP | POS | 10.30 | 568.3397 | | | | 8.48E-01 | -0.04 | 0.95 | 2.69E-02 | -0.46 | 1.35 | 1.95E-01 | -0.27 | 0.70 | 3.63E-01 | 0.20 | 0.92 | 51410557 | 20286973 | 50567238 | 25095737 | 56095989 | 25825990 | 45038487 | 23304108 |
| HILIC_NEG_113_92614a1_049 | HILIC | NEG | 1.049 | 113.9261 | | | | 8.60E-02 | 0.33 | 0.95 | 2.69E-02 | 0.45 | 1.32 | 1.25E-02 | 0.55 | 1.10 | 6.19E-01 | 0.10 | 0.22 | 1078956 | 262966 | 1175561 | 299950 | 1109499 | 281155 | 1241623 | 306304 |
| RP_NEG_892_53381a12_696 | RP | NEG | 12.70 | 892.5338 | | | | 2.13E-03 | 0.64 | 1.24 | 2.69E-02 | -0.42 | 1.35 | 5.80E-02 | 0.42 | 0.85 | 2.01E-04 | 0.85 | 1.41 | 3653351 | 1272825 | 4487344 | 1262027 | 4765260 | 1198979 | 4209427 | 1273872 |
| HILIC_NEG_144_92348a0_678 | HILIC | NEG | 0.678 | 144.9235 | | | | 2.65E-01 | -0.21 | 0.67 | 2.71E-02 | 0.46 | 1.36 | 9.29E-01 | 0.02 | 0.30 | 6.23E-02 | -0.44 | 0.72 | 13107793 | 2007011 | 12629485 | 2342611 | 12112863 | 2746583 | 13146107 | 1731830 |
| HILIC_POS_546_35394a1_211 | HILIC | POS | 1.211 | 546.3539 | | | | 1.71E-03 | 0.58 | 1.14 | 2.72E-02 | -0.45 | 1.28 | 8.73E-02 | 0.35 | 0.67 | 1.36E-04 | 0.80 | 1.46 | 16270131 | 6546929 | 20907216 | 8168864 | 22702529 | 7787593 | 19111902 | 8221804 |
| RP_POS_148_09671a0_708 | RP | POS | 0.71 | 148.0967 | | | | 1.92E-06 | 0.66 | 1.09 | 2.73E-02 | 0.48 | 1.33 | 6.21E-05 | 0.90 | 1.38 | 3.10E-04 | 0.42 | 1.19 | 2264174 | 569940 | 3137022 | 1425955 | 2821536 | 762910 | 3452509 | 1822920 |
| RP_POS_519_32556a10_502 | RP | POS | 10.50 | 519.3256 | | | | 9.77E-01 | 0.01 | 1.06 | 2.75E-02 | -0.45 | 1.58 | 3.15E-01 | -0.22 | 0.76 | 2.90E-01 | 0.23 | 1.22 | 50288492 | 4739960 | 50316548 | 5264765 | 51471597 | 5139288 | 49161500 | 5183257 |
| RP_POS_266_97427a15_48 | RP | POS | 15.48 | 266.9743 | | | | 4.70E-01 | -0.16 | 0.66 | 2.76E-02 | 0.43 | 1.39 | 7.98E-01 | 0.06 | 0.17 | 1.27E-01 | -0.37 | 1.01 | 1671039 | 85670 | 1658626 | 77875 | 1641550 | 81833 | 1675702 | 70434 |
| RP_POS_597_44775a11_158 | RP | POS | 11.16 | 597.4478 | | | | 3.90E-02 | -0.50 | 0.77 | 2.77E-02 | -0.39 | 1.40 | 5.92E-03 | -0.69 | 1.22 | 2.46E-01 | -0.30 | 0.58 | 945898 | 830760 | 607052 | 610004 | 740866 | 672288 | 473239 | 513118 |
| RP_POS_975_63776a10_515 | RP | POS | 10.52 | 975.6378 | | | | 7.08E-04 | 0.54 | 1.34 | 2.78E-02 | 0.47 | 1.74 | 5.97E-04 | 0.77 | 1.27 | 4.32E-02 | 0.30 | 1.04 | 241533 | 198718 | 413072 | 340693 | 338119 | 219926 | 488025 | 417946 |
| RP_POS_744_58899a13_689 | RP | POS | 13.69 | 744.5890 | | | | 9.42E-06 | -0.99 | 1.56 | 2.78E-02 | -0.38 | 1.31 | 7.66E-07 | -1.18 | 1.87 | 6.01E-04 | -0.80 | 1.24 | 10265504 | 3865631 | 6483914 | 3352037 | 7217901 | 3478204 | 5749927 | 3082896 |
| RP_NEG_389_19415a10_824 | RP | NEG | 10.82 | 389.1942 | | | | 1.06E-10 | 1.09 | 1.97 | 2.80E-02 | 0.42 | 1.57 | 5.36E-10 | 1.30 | 2.09 | 4.69E-07 | 0.89 | 1.83 | 243784 | 214253 | 613400 | 321123 | 543100 | 275872 | 683699 | 349504 |
| RP_POS_1085_65271a10_312 | RP | POS | 10.31 | 1085.6527 | | | | 8.33E-01 | 0.05 | 0.82 | 2.81E-02 | -0.42 | 1.80 | 5.03E-01 | -0.16 | 0.55 | 2.76E-01 | 0.26 | 0.75 | 1140567 | 448463 | 1159311 | 386445 | 1243812 | 354059 | 1074810 | 402240 |
| RP_POS_564_30579a9_91 | RP | POS | 9.91 | 564.3058 | | | | 1.25E-01 | 0.32 | 0.96 | 2.81E-02 | -0.43 | 1.36 | 6.43E-01 | 0.10 | 0.55 | 2.36E-02 | 0.53 | 1.03 | 401329 | 334843 | 506879 | 327657 | 578522 | 342765 | 435237 | 298240 |
| RP_POS_650_84888a0_495 | RP | POS | 0.50 | 650.8489 | | | | 1.53E-06 | -0.66 | 1.12 | 2.81E-02 | 0.47 | 1.43 | 1.57E-02 | -0.43 | 0.83 | 4.86E-07 | -0.90 | 1.54 | 11750463 | 969169 | 10228209 | 2480633 | 9686037 | 2340022 | 10770380 | 2521322 |
| RP_POS_781_05304a12_808 | RP | POS | 12.81 | 781.0530 | | | | 1.56E-01 | 0.24 | 0.55 | 2.82E-02 | -0.47 | 1.33 | 9.71E-01 | 0.01 | 0.44 | 3.36E-02 | 0.47 | 0.92 | 516331 | 299271 | 614752 | 437680 | 710618 | 513727 | 518887 | 323055 |
| RP_POS_507_32272a10_504 | RP | POS | 10.50 | 507.3227 | | | | 1.02E-02 | -0.50 | 0.92 | 2.82E-02 | 0.44 | 1.30 | 1.92E-01 | -0.28 | 0.59 | 1.03E-03 | -0.72 | 1.21 | 1538376 | 392681 | 1320610 | 436715 | 1225226 | 420173 | 1415995 | 436140 |
| RP_POS_349_21094a10_945 | RP | POS | 10.95 | 349.2109 | | | | 4.79E-05 | 0.72 | 1.61 | 2.84E-02 | 0.44 | 1.51 | 2.73E-05 | 0.94 | 1.62 | 5.22E-03 | 0.49 | 1.45 | 546584 | 449981 | 982666 | 618138 | 847550 | 477374 | 1117783 | 711930 |
| RP_POS_542_32404a10_032 | RP | POS | 10.03 | 542.3240 | | | | 1.04E-01 | 0.34 | 0.97 | 2.85E-02 | -0.43 | 1.35 | 5.84E-01 | 0.12 | 0.56 | 1.90E-02 | 0.55 | 1.06 | 27258980 | 21983598 | 34612769 | 21621952 | 39328331 | 22471509 | 29897207 | 19854703 |
| RP_POS_281_11304a3_034 | RP | POS | 3.03 | 281.1130 | | | | 6.61E-05 | 0.47 | 1.14 | 2.87E-02 | 0.49 | 1.66 | 1.17E-03 | 0.72 | 1.30 | 4.49E-03 | 0.22 | 1.19 | 7313 | 8471 | 399094 | 940058 | 192645 | 439829 | 605543 | 1226794 |
| RP_POS_283_15411a8_87 | RP | POS | 8.87 | 283.1541 | | | | 2.59E-05 | -1.05 | 1.77 | 2.87E-02 | -0.35 | 1.38 | 2.78E-06 | -1.22 | 1.91 | 5.98E-04 | -0.87 | 1.44 | 7362510 | 3816148 | 3932648 | 2600230 | 4499162 | 2820508 | 3366135 | 2247250 |
| RP_POS_551_4245a11_441 | RP | POS | 11.44 | 551.4245 | | | | 9.86E-06 | -1.27 | 2.19 | 2.88E-02 | -0.26 | 1.29 | 2.40E-06 | -1.40 | 2.00 | 5.61E-05 | -1.14 | 1.90 | 1347790 | 774217 | 619653 | 339480 | 693528 | 322268 | 545778 | 343294 |
| RP_NEG_1031_63354a10_124 | RP | NEG | 10.12 | 1031.6335 | | | | 1.70E-12 | 0.87 | 1.79 | 2.89E-02 | 0.47 | 1.37 | 3.57E-07 | 1.10 | 1.75 | 1.89E-08 | 0.63 | 1.75 | 344741 | 299459 | 2617870 | 2792673 | 2007255 | 1740572 | 3228485 | 3459292 |
| RP_POS_390_18442a10_943 | RP | POS | 10.94 | 390.1844 | | | | 3.68E-05 | 0.76 | 1.56 | 2.91E-02 | 0.43 | 1.46 | 1.50E-05 | 0.98 | 1.61 | 3.47E-03 | 0.54 | 1.41 | 409343 | 354916 | 750205 | 444838 | 653419 | 357045 | 846992 | 503192 |
| RP_NEG_153_01851a4_093 | RP | NEG | 4.09 | 153.0185 | | | | 3.13E-04 | 0.48 | 0.85 | 2.91E-02 | 0.48 | 1.31 | 8.41E-04 | 0.72 | 1.16 | 4.38E-02 | 0.24 | 0.91 | 852418 | 608633 | 1599172 | 1704290 | 1227022 | 1045763 | 1971321 | 2119486 |
| RP_POS_918_7074a0_5 | RP | POS | 0.50 | 918.7074 | | | | 1.39E-02 | 0.42 | 0.96 | 2.91E-02 | -0.46 | 1.49 | 2.96E-01 | 0.19 | 0.56 | 2.39E-03 | 0.65 | 1.33 | 406842 | 214936 | 530040 | 307838 | 596951 | 333931 | 463129 | 266111 |
| RP_POS_291_1203a8_87 | RP | POS | 8.87 | 291.1203 | | | | 3.04E-05 | -1.05 | 1.78 | 2.91E-02 | -0.34 | 1.37 | 3.48E-06 | -1.22 | 1.90 | 6.23E-04 | -0.88 | 1.44 | 7325158 | 3904812 | 3858874 | 2593978 | 4422638 | 2805889 | 3295109 | 2252358 |
| RP_POS_279_64703a10_328 | RP | POS | 10.33 | 279.6470 | | | | 2.76E-02 | -0.54 | 1.07 | 2.92E-02 | -0.38 | 1.27 | 6.43E-03 | -0.73 | 1.16 | 1.61E-01 | -0.35 | 0.74 | 2007611 | 386785 | 1839312 | 275887 | 1899255 | 236630 | 1779369 | 300755 |
| RP_POS_1025_45825a0_487 | RP | POS | 0.49 | 1025.4583 | | | | 2.27E-04 | 0.47 | 0.89 | 2.93E-02 | -0.48 | 1.38 | 1.52E-01 | 0.23 | 0.68 | 1.10E-04 | 0.71 | 1.27 | 508178 | 43684 | 577430 | 165311 | 613312 | 170778 | 541547 | 153037 |
| RP_POS_357_29956a10_959 | RP | POS | 10.96 | 357.2996 | | | | 1.14E-04 | 0.46 | 0.95 | 2.94E-02 | 0.49 | 1.37 | 1.30E-03 | 0.71 | 1.04 | 1.03E-02 | 0.22 | 0.95 | 393813 | 317672 | 1336464 | 2286557 | 836534 | 1114307 | 1836394 | 2968441 |
| RP_POS_569_4325a13_63 | RP | POS | 13.63 | 569.4325 | | | | 1.93E-01 | 0.29 | 0.58 | 2.94E-02 | 0.42 | 1.28 | 4.49E-02 | 0.50 | 0.83 | 7.35E-01 | 0.08 | 0.66 | 2630507 | 1417336 | 2999414 | 1236786 | 2731176 | 1113156 | 3267651 | 1305579 |
| RP_NEG_968_76837a0_493 | RP | NEG | 0.49 | 968.7684 | | | | 9.61E-03 | -0.50 | 0.92 | 2.94E-02 | 0.44 | 1.39 | 2.13E-01 | -0.28 | 0.54 | 4.89E-04 | -0.72 | 1.28 | 1300024 | 116387 | 1234633 | 131547 | 1206074 | 106825 | 1263192 | 147940 |
| HILIC_NEG_182_04587a0_816 | HILIC | NEG | 0.816 | 182.0459 | | | | 3.07E-03 | 0.35 | 1.03 | 2.94E-02 | 0.50 | 1.34 | 9.50E-03 | 0.60 | 1.09 | 6.77E-03 | 0.10 | 1.38 | 178189 | 101149 | 4142765 | 13059668 | 1274677 | 2739582 | 7010852 | 17896862 |
| RP_POS_269_2265a10_854 | RP | POS | 10.85 | 269.2265 | | | | 1.76E-02 | -0.38 | 0.82 | 2.95E-02 | -0.47 | 1.46 | 5.54E-03 | -0.61 | 1.02 | 3.54E-01 | -0.15 | 0.51 | 46851869 | 9408187 | 41328079 | 15645082 | 44729075 | 10980182 | 37927083 | 18717969 |
| RP_POS_361_16687a10_943 | RP | POS | 10.94 | 361.1669 | | | | 6.92E-06 | 0.79 | 1.62 | 2.95E-02 | 0.44 | 1.43 | 4.99E-06 | 1.01 | 1.68 | 1.36E-03 | 0.57 | 1.45 | 1968194 | 1149207 | 3220047 | 1593986 | 2874180 | 1276464 | 3565915 | 1805531 |
| RP_NEG_511_29214a8_899 | RP | NEG | 8.90 | 511.2921 | | | | 1.36E-01 | -0.48 | 0.96 | 2.96E-02 | -0.24 | 1.39 | 6.84E-02 | -0.60 | 0.90 | 2.64E-01 | -0.36 | 0.62 | 2552435 | 3317866 | 1641928 | 1033409 | 1865796 | 1038892 | 1418061 | 987970 |
| RP_POS_684_84216a0_492 | RP | POS | 0.49 | 684.8422 | | | | 4.92E-01 | 0.10 | 0.69 | 2.96E-02 | 0.48 | 1.53 | 1.02E-01 | 0.34 | 0.74 | 3.35E-01 | -0.14 | 0.94 | 1805565 | 364187 | 1876195 | 797360 | 1703066 | 592626 | 2049324 | 933898 |
| RP_POS_581_45367a12_257 | RP | POS | 12.26 | 581.4537 | | | | 9.28E-02 | -0.41 | 0.69 | 2.98E-02 | -0.38 | 1.39 | 1.76E-02 | -0.61 | 1.24 | 4.03E-01 | -0.22 | 0.55 | 3251949 | 2859844 | 2316691 | 1992872 | 2748510 | 2249719 | 1884873 | 1606968 |
| RP_POS_184_07294a10_198 | RP | POS | 10.20 | 184.0729 | | | | 1.25E-01 | -0.36 | 1.07 | 2.98E-02 | -0.39 | 1.65 | 3.09E-02 | -0.56 | 1.02 | 4.97E-01 | -0.17 | 0.84 | 1693757 | 771366 | 1462097 | 578935 | 1587364 | 526701 | 1336831 | 606412 |
| RP_NEG_680_31909a10_291 | RP | NEG | 10.29 | 680.3191 | | | | 6.60E-01 | -0.08 | 0.93 | 2.98E-02 | -0.46 | 1.30 | 1.39E-01 | -0.31 | 0.74 | 4.56E-01 | 0.15 | 0.86 | 1412029 | 403318 | 1372605 | 536337 | 1488597 | 517069 | 1256613 | 535132 |

**Supplementary table 1: Metabolomics results** (in asceding order according to p-value from progressive ALD vs  non-progressive ALD comparison, with molecular features with ID_level of 1-2 first)

| | | | | | | | | all_ALD_vs_control | | | Prog_ALD_vs_non-prog_ALD | | | Prog_ALD_vs_control_ALD | | | Non-prog_ALD_vs_control | | | Healthy_control | | all_ALD | | Non-prog_ALD | | Progressive_ALD | |
|---|---|---|---|---|---|---|---|---|---|---|---|---|---|---|---|---|---|---|---|---|---|---|---|---|---|---|---|
| | | | | | | | | t-test | Cohen's | PLS-DA | t-test | Cohen's | PLS-DA | t-test | Cohen's | PLS-DA | t-test | Cohen's | PLS-DA | | | | | | | | |
| Feature_ID | RP_HILIC | POS_NEG | Average.Rt | Average.Mz | Metabolite.name | curated_MSMS | ID_level | p | d | VIP | p | d | VIP | p | d | VIP | p | d | VIP | Mean | SD | Mean | SD | Mean | SD | Mean | SD |
| HILIC_NEG_498_29004a1_356 | HILIC | NEG | 1.356 | 498.2900 | | | | 3.82E-02 | 0.29 | 0.74 | 2.99E-02 | 0.48 | 1.44 | 1.81E-02 | 0.53 | 0.75 | 6.80E-01 | 0.05 | 0.15 | 188589 | 677994 | 612428 | 1629046 | 257489 | 818152 | 967367 | 2106006 |
| HILIC_NEG_61_98754a0_881 | HILIC | NEG | 0.881 | 61.9875 | | | | 1.44E-01 | 0.29 | 0.70 | 2.99E-02 | 0.44 | 1.39 | 3.33E-02 | 0.51 | 0.83 | 7.10E-01 | 0.08 | 0.15 | 3768634 | 1690791 | 4283537 | 1760918 | 3902156 | 1378379 | 4664919 | 2016971 |
| RP_NEG_650_28131a10_328 | RP | NEG | 10.33 | 650.2813 | | | | 1.46E-03 | -0.74 | 1.35 | 3.01E-02 | -0.39 | 1.52 | 2.30E-04 | -0.93 | 1.51 | 2.23E-02 | -0.54 | 1.07 | 2077171 | 373426 | 1831119 | 299668 | 1895824 | 280607 | 1766414 | 306838 |
| RP_POS_248_14882a0_71 | RP | POS | 0.71 | 248.1488 | | | | 2.00E-08 | 0.69 | 1.17 | 3.03E-02 | 0.48 | 1.34 | 2.93E-05 | 0.93 | 1.31 | 1.71E-06 | 0.45 | 1.46 | 366090 | 354495 | 1701514 | 2121730 | 1240363 | 1085998 | 2162666 | 2735316 |
| RP_POS_1053_6908a11_129 | RP | POS | 11.13 | 1053.6908 | | | | 6.36E-04 | 0.41 | 1.08 | 3.04E-02 | -0.49 | 1.52 | 1.49E-01 | 0.16 | 0.91 | 1.52E-03 | 0.65 | 1.28 | 246954 | 40944 | 354385 | 296766 | 418609 | 358525 | 290161 | 202432 |
| RP_POS_353_2449a11_382 | RP | POS | 11.38 | 353.2449 | | | | 9.80E-09 | 0.73 | 1.33 | 3.04E-02 | 0.47 | 1.27 | 4.78E-06 | 0.96 | 1.51 | 7.97E-05 | 0.49 | 1.31 | 356541 | 186390 | 857839 | 746599 | 696383 | 521665 | 1019296 | 894909 |
| RP_POS_186_18484a9_04 | RP | POS | 9.04 | 186.1848 | | | | 6.40E-02 | 0.44 | 0.74 | 3.04E-02 | -0.39 | 1.29 | 3.45E-01 | 0.24 | 0.41 | 1.14E-02 | 0.63 | 1.04 | 864705 | 334210 | 987179 | 257305 | 1042647 | 234030 | 931711 | 269654 |
| RP_POS_535_86243a11_127 | RP | POS | 11.13 | 535.8624 | | | | 4.38E-06 | 0.55 | 1.24 | 3.05E-02 | -0.48 | 1.52 | 7.58E-03 | 0.31 | 1.09 | 1.21E-04 | 0.79 | 1.40 | 289588 | 58204 | 501016 | 425078 | 592887 | 510093 | 409145 | 296058 |
| RP_POS_313_63058a10_291 | RP | POS | 10.29 | 313.6306 | | | | 4.79E-01 | -0.13 | 1.01 | 3.06E-02 | -0.46 | 1.31 | 8.18E-02 | -0.35 | 0.84 | 6.24E-01 | 0.10 | 0.92 | 541813 | 168249 | 515416 | 221968 | 563218 | 224999 | 467614 | 210373 |
| RP_POS_339_28903a10_962 | RP | POS | 10.96 | 339.2890 | | | | 7.04E-05 | 0.48 | 0.98 | 3.07E-02 | 0.49 | 1.35 | 1.07E-03 | 0.72 | 1.05 | 6.17E-03 | 0.24 | 0.98 | 129859 | 109676 | 452797 | 757372 | 288590 | 371193 | 617003 | 982913 |
| RP_POS_770_46136a10_501 | RP | POS | 10.50 | 770.4614 | | | | 2.77E-02 | -0.44 | 0.90 | 3.09E-02 | -0.43 | 1.27 | 5.21E-03 | -0.66 | 1.21 | 2.78E-01 | -0.23 | 0.96 | 2080412 | 343263 | 1920615 | 361923 | 1998453 | 309966 | 1842776 | 395230 |
| RP_POS_896_53857a12_74 | RP | POS | 12.74 | 896.5386 | | | | 1.38E-04 | -0.88 | 1.48 | 3.09E-02 | -0.38 | 1.26 | 9.92E-06 | -1.07 | 1.86 | 5.04E-03 | -0.69 | 1.19 | 1202946 | 469271 | 822758 | 378102 | 904130 | 428476 | 741386 | 302879 |
| RP_POS_679_49835a11_178 | RP | POS | 11.18 | 679.4984 | | | | 1.02E-05 | 0.52 | 1.18 | 3.10E-02 | -0.48 | 1.55 | 6.05E-03 | 0.28 | 1.20 | 3.06E-04 | 0.77 | 1.34 | 56770 | 29676 | 235153 | 380564 | 317317 | 473035 | 152989 | 234679 |
| RP_POS_558_29468a10_324 | RP | POS | 10.32 | 558.2947 | | | | 5.81E-02 | -0.45 | 1.15 | 3.10E-02 | -0.39 | 1.52 | 1.32E-02 | -0.64 | 1.11 | 2.98E-01 | -0.25 | 0.87 | 4107124 | 1583102 | 3514524 | 1197416 | 3771774 | 1084646 | 3257274 | 1259111 |
| RP_POS_1039_67102a10_323 | RP | POS | 10.32 | 1039.6710 | | | | 5.08E-02 | -0.50 | 1.16 | 3.11E-02 | -0.37 | 1.57 | 1.19E-02 | -0.68 | 1.16 | 2.35E-01 | -0.31 | 0.92 | 88142777 | 62285056 | 64495114 | 40806988 | 73255313 | 40046000 | 55734916 | 40051315 |
| RP_POS_448_30518a8_668 | RP | POS | 8.67 | 448.3052 | | | | 1.92E-01 | 0.18 | 0.64 | 3.12E-02 | 0.48 | 1.27 | 5.98E-02 | 0.42 | 0.88 | 5.87E-01 | -0.06 | 0.34 | 519233 | 497498 | 716524 | 1220400 | 452630 | 598895 | 980419 | 1583778 |
| HILIC_POS_183_00583a2_975 | HILIC | POS | 2.975 | 183.0058 | | | | 3.87E-11 | 0.98 | 1.59 | 3.13E-02 | 0.43 | 1.50 | 1.58E-09 | 1.20 | 1.87 | 4.22E-06 | 0.77 | 1.49 | 1362144 | 647109 | 2641321 | 1308320 | 2360624 | 1176326 | 2922018 | 1383401 |
| RP_NEG_558_31378a10_623 | RP | NEG | 10.62 | 558.3138 | | | | 6.38E-02 | 0.37 | 1.22 | 3.14E-02 | -0.43 | 1.29 | 4.92E-01 | 0.16 | 0.49 | 6.40E-03 | 0.59 | 1.60 | 1339595 | 243146 | 1433949 | 252541 | 1488119 | 213895 | 1379779 | 277699 |
| RP_POS_257_22629a8_886 | RP | POS | 8.89 | 257.2263 | | | | 1.18E-01 | -0.51 | 0.98 | 3.14E-02 | -0.23 | 1.39 | 5.96E-02 | -0.62 | 0.93 | 2.30E-01 | -0.39 | 0.65 | 1324370 | 1708487 | 832467 | 522689 | 944484 | 529596 | 720449 | 495886 |
| RP_POS_766_53638a13_197 | RP | POS | 13.20 | 766.5364 | | | | 1.00E-02 | 0.43 | 0.76 | 3.15E-02 | 0.46 | 1.30 | 3.65E-03 | 0.66 | 1.01 | 2.15E-01 | 0.20 | 0.68 | 1221102 | 668948 | 1633375 | 1021618 | 1413769 | 697618 | 1852981 | 1234347 |
| RP_POS_593_33282a6_145 | RP | POS | 6.15 | 593.3328 | | | | 3.18E-03 | 0.42 | 0.78 | 3.16E-02 | 0.47 | 1.40 | 3.85E-03 | 0.66 | 0.99 | 1.10E-01 | 0.19 | 0.69 | 403991 | 1194141 | 1423391 | 2643931 | 852784 | 1269611 | 1993997 | 3441949 |
| HILIC_NEG_497_04599a7_623 | HILIC | NEG | 7.623 | 497.0460 | | | | 6.29E-01 | -0.10 | 0.23 | 3.16E-02 | -0.43 | 1.21 | 1.57E-01 | -0.31 | 0.60 | 6.27E-01 | 0.11 | 0.36 | 349835 | 16483 | 348216 | 16231 | 351691 | 17323 | 344741 | 14407 |
| RP_NEG_594_30389a10_248 | RP | NEG | 10.25 | 594.3039 | | | | 5.44E-09 | -1.22 | 2.07 | 3.17E-02 | -0.37 | 1.59 | 2.63E-10 | -1.40 | 2.27 | 2.42E-06 | -1.04 | 1.75 | 1198821 | 314909 | 753682 | 311559 | 820385 | 341364 | 686978 | 265536 |
| RP_POS_287_13022a10_329 | RP | POS | 10.33 | 287.1302 | | | | 3.32E-05 | -0.89 | 1.65 | 3.18E-02 | -0.39 | 1.36 | 6.30E-06 | -1.09 | 1.74 | 1.31E-03 | -0.70 | 1.43 | 3452946 | 613547 | 2893530 | 568841 | 3015251 | 499022 | 2771809 | 611968 |
| HILIC_POS_148_09682a4_203 | HILIC | POS | 4.203 | 148.0968 | | | | 2.55E-07 | 0.71 | 1.24 | 3.20E-02 | 0.46 | 1.41 | 2.07E-05 | 0.94 | 1.49 | 6.94E-05 | 0.49 | 1.44 | 3744443 | 1333315 | 5963412 | 3314847 | 5251464 | 1913528 | 6675360 | 4183857 |
| RP_POS_284_63034a10_127 | RP | POS | 10.13 | 284.6303 | | | | 1.86E-21 | 1.23 | 2.05 | 3.22E-02 | 0.41 | 1.35 | 1.15E-11 | 1.43 | 2.16 | 5.54E-14 | 1.02 | 2.15 | 238699 | 122467 | 832429 | 468101 | 732295 | 335190 | 932563 | 556680 |
| RP_POS_896_78961a0_493 | RP | POS | 0.49 | 896.7896 | | | | 7.93E-03 | -0.48 | 0.92 | 3.25E-02 | 0.44 | 1.39 | 2.39E-01 | -0.26 | 0.43 | 3.28E-04 | -0.70 | 1.27 | 1041863 | 280570 | 873122 | 366570 | 794940 | 300676 | 951304 | 410620 |
| RP_POS_748_52576a13_075 | RP | POS | 13.08 | 748.5258 | | | | 5.98E-01 | -0.11 | 0.64 | 3.27E-02 | -0.41 | 1.31 | 1.54E-01 | -0.32 | 0.61 | 7.09E-01 | 0.09 | 0.57 | 719780 | 853250 | 630179 | 761562 | 792441 | 859592 | 467917 | 615758 |
| RP_POS_518_32239a10_503 | RP | POS | 10.50 | 518.3224 | | | | 7.83E-01 | 0.05 | 1.10 | 3.27E-02 | -0.44 | 1.57 | 4.48E-01 | -0.17 | 0.73 | 2.09E-01 | 0.27 | 1.28 | 192184039 | 17857084 | 193219389 | 20085850 | 197492503 | 19397921 | 188946276 | 20039278 |
| RP_POS_1015_66241a10_501 | RP | POS | 10.50 | 1015.6624 | | | | 3.35E-01 | -0.20 | 0.98 | 3.28E-02 | -0.42 | 1.54 | 7.50E-02 | -0.41 | 0.95 | 9.55E-01 | 0.01 | 0.75 | 2930353 | 528036 | 2826388 | 520120 | 2936941 | 504287 | 2715835 | 517033 |
| HILIC_NEG_243_99753a0_882 | HILIC | NEG | 0.882 | 243.9975 | | | | 2.06E-01 | 0.25 | 0.66 | 3.29E-02 | 0.43 | 1.39 | 4.88E-02 | 0.47 | 0.77 | 8.60E-01 | 0.04 | 0.07 | 139491 | 66586 | 157079 | 70774 | 142019 | 57477 | 172139 | 79710 |
| RP_POS_300_63226a10_199 | RP | POS | 10.20 | 300.6323 | | | | 2.62E-01 | 0.23 | 0.69 | 3.30E-02 | -0.42 | 1.61 | 9.20E-01 | 0.02 | 0.33 | 4.83E-02 | 0.44 | 0.86 | 938712 | 301636 | 1007651 | 291840 | 1069664 | 262296 | 945637 | 308920 |
| RP_POS_598_26404a10_376 | RP | POS | 10.38 | 598.2640 | | | | 9.08E-01 | -0.02 | 0.74 | 3.30E-02 | -0.44 | 1.31 | 2.87E-01 | -0.24 | 0.63 | 3.50E-01 | 0.20 | 0.69 | 614058 | 87987 | 611914 | 98323 | 632802 | 87798 | 591026 | 104562 |
| HILIC_NEG_195_81104a0_882 | HILIC | NEG | 0.882 | 195.8110 | | | | 4.17E-01 | 0.16 | 0.76 | 3.30E-02 | 0.43 | 1.34 | 8.89E-02 | 0.38 | 0.87 | 7.95E-01 | -0.06 | 0.33 | 761696 | 139690 | 785420 | 151756 | 753199 | 149404 | 817642 | 148625 |
| RP_POS_548_30035a10_198 | RP | POS | 10.20 | 548.3004 | | | | 2.01E-02 | -0.59 | 1.25 | 3.31E-02 | -0.36 | 1.51 | 3.97E-03 | -0.77 | 1.30 | 1.22E-01 | -0.41 | 0.98 | 1011981 | 670989 | 705459 | 442062 | 799291 | 457903 | 611628 | 408879 |
| RP_POS_516_30518a10_125 | RP | POS | 10.13 | 516.3052 | | | | 6.28E-21 | 1.21 | 2.06 | 3.31E-02 | 0.41 | 1.33 | 1.81E-11 | 1.42 | 2.14 | 8.77E-14 | 1.01 | 2.14 | 11019593 | 5664615 | 36994391 | 20734025 | 32580684 | 14763110 | 41408098 | 24715721 |
| HILIC_POS_290_15289a0_899 | HILIC | POS | 0.899 | 290.1529 | | | | 3.29E-04 | 0.53 | 1.08 | 3.32E-02 | 0.46 | 1.26 | 5.34E-04 | 0.76 | 1.19 | 3.30E-02 | 0.30 | 0.73 | 218834 | 64202 | 283792 | 133432 | 255372 | 87928 | 312212 | 163070 |
| RP_POS_274_17554a0_71 | RP | POS | 0.71 | 274.1755 | | | | 7.72E-02 | 0.24 | 0.81 | 3.32E-02 | -0.48 | 1.46 | 9.86E-01 | 0.00 | 0.90 | 2.08E-02 | 0.47 | 0.94 | 7271348 | 1644988 | 8243457 | 4617712 | 9225380 | 5451785 | 7261534 | 3375208 |
| HILIC_POS_273_12112a3_697 | HILIC | POS | 3.697 | 273.1211 | | | | 1.01E-02 | 0.31 | 0.84 | 3.34E-02 | 0.48 | 1.41 | 1.19E-02 | 0.56 | 0.90 | 3.93E-01 | 0.07 | 0.59 | 1315856 | 929016 | 2579272 | 4547946 | 1607544 | 2101612 | 3551000 | 5953648 |
| HILIC_NEG_177_86018a2_978 | HILIC | NEG | 2.978 | 177.8602 | | | | 5.72E-01 | 0.10 | 0.28 | 3.34E-02 | 0.45 | 1.29 | 1.31E-01 | 0.32 | 0.53 | 5.00E-01 | -0.13 | 0.40 | 592945 | 168118 | 614389 | 233088 | 564969 | 202512 | 663808 | 252535 |
| HILIC_POS_516_30615a1_34 | HILIC | POS | 1.34 | 516.3062 | | | | 1.55E-17 | 1.13 | 1.84 | 3.35E-02 | 0.42 | 1.16 | 1.76E-10 | 1.34 | 2.07 | 3.95E-11 | 0.92 | 2.14 | 825900 | 402384 | 2251819 | 1254292 | 1985496 | 926838 | 2518142 | 1474728 |
| HILIC_POS_245_0954a0_899 | HILIC | POS | 0.899 | 245.0954 | | | | 9.41E-06 | 0.62 | 1.15 | 3.35E-02 | 0.46 | 1.23 | 7.63E-05 | 0.85 | 1.31 | 3.35E-03 | 0.40 | 0.99 | 380806 | 99252 | 515822 | 233069 | 466277 | 156629 | 565367 | 283212 |
| RP_POS_310_20111a5_604 | RP | POS | 5.60 | 310.2011 | | | | 6.59E-02 | 0.33 | 0.71 | 3.37E-02 | 0.44 | 1.27 | 1.64E-02 | 0.55 | 1.02 | 5.40E-01 | 0.11 | 0.50 | 471556 | 402822 | 636091 | 516802 | 526505 | 379234 | 745677 | 609338 |
| HILIC_POS_480_30936a1_859 | HILIC | POS | 1.859 | 480.3094 | | | | 4.26E-04 | 0.61 | 1.01 | 3.39E-02 | 0.44 | 1.33 | 3.40E-04 | 0.83 | 1.19 | 1.73E-02 | 0.39 | 0.98 | 1595496 | 830746 | 2289755 | 1176453 | 2040006 | 756047 | 2539504 | 1448250 |
| RP_POS_430_24893a12_953 | RP | POS | 12.95 | 430.2489 | | | | 1.49E-06 | -0.91 | 1.56 | 3.41E-02 | -0.41 | 1.41 | 4.65E-07 | -1.11 | 1.82 | 3.28E-04 | -0.70 | 1.48 | 1299367 | 311820 | 944185 | 376603 | 1023716 | 337840 | 864655 | 399387 |
| RP_POS_209_11739a8_87 | RP | POS | 8.87 | 209.1174 | Primin | 209.1166(100), 153.0541(28) | 3 | 3.39E-05 | -1.04 | 1.78 | 3.42E-02 | -0.33 | 1.34 | 4.21E-06 | -1.21 | 1.89 | 6.41E-04 | -0.88 | 1.45 | 2915744 | 1530214 | 1567512 | 1013763 | 1781489 | 1098093 | 1353535 | 881565 |
| RP_POS_385_29306a10_443 | RP | POS | 10.44 | 385.2931 | | | | 6.03E-01 | -0.10 | 0.57 | 3.43E-02 | 0.43 | 1.33 | 6.04E-01 | 0.11 | 0.71 | 1.63E-01 | -0.32 | 0.62 | 19844061 | 873106 | 19749688 | 934081 | 19552731 | 970699 | 19946645 | 861043 |
| RP_POS_698_78564a6_136 | RP | POS | 6.14 | 698.7856 | | | | 1.07E-05 | 0.52 | 1.16 | 3.48E-02 | -0.47 | 1.45 | 1.47E-03 | 0.28 | 1.21 | 5.08E-04 | 0.76 | 1.22 | 72690 | 57807 | 802714 | 1570996 | 1135324 | 2017359 | 470105 | 831212 |
| RP_NEG_294_98227a7_902 | RP | NEG | 7.90 | 294.9823 | | | | 9.18E-01 | -0.02 | 0.64 | 3.49E-02 | -0.44 | 1.26 | 2.15E-01 | -0.24 | 0.58 | 3.73E-01 | 0.20 | 0.74 | 14079954 | 794856 | 14062291 | 982896 | 14269226 | 1116071 | 13855356 | 786687 |
| RP_POS_375_32004a10_27 | RP | POS | 10.27 | 375.3200 | | | | 1.41E-01 | -0.25 | 0.91 | 3.50E-02 | 0.45 | 1.24 | 8.93E-01 | -0.03 | 0.77 | 1.01E-02 | -0.48 | 0.94 | 1118803 | 78940 | 1092184 | 112034 | 1068616 | 91313 | 1115752 | 126046 |
| RP_NEG_353_14334a10_835 | RP | NEG | 10.84 | 353.1433 | | | | 1.52E-15 | 1.14 | 1.99 | 3.51E-02 | 0.41 | 1.44 | 2.46E-12 | 1.35 | 2.19 | 2.28E-08 | 0.94 | 1.82 | 1538010 | 1468708 | 5394155 | 3274327 | 4706513 | 3079468 | 6081797 | 3348599 |
| RP_POS_287_07544a8_02 | RP | POS | 8.02 | 287.0754 | | | | 1.08E-05 | 0.52 | 1.24 | 3.51E-02 | 0.47 | 1.62 | 3.23E-04 | 0.76 | 1.54 | 7.31E-03 | 0.28 | 0.86 | 186227 | 937617 | 14701569 | 31272380 | 8105440 | 19975734 | 21297699 | 38575358 |
| RP_POS_274_18338a7_445 | RP | POS | 7.45 | 274.1834 | | | | 3.88E-13 | 1.04 | 1.80 | 3.51E-02 | 0.42 | 1.59 | 2.45E-10 | 1.25 | 2.00 | 2.88E-07 | 0.83 | 1.66 | 702083 | 492212 | 1846040 | 1092260 | 1616626 | 970931 | 2075454 | 1166316 |
| RP_POS_284_27005a7_999 | RP | POS | 8.00 | 284.2701 | | | | 9.68E-01 | 0.01 | 0.87 | 3.52E-02 | -0.41 | 1.39 | 4.01E-01 | -0.20 | 0.77 | 3.77E-01 | 0.21 | 0.65 | 480561 | 240206 | 482459 | 214727 | 527525 | 221612 | 437393 | 199750 |
| RP_POS_223_16919a11_151 | RP | POS | 11.15 | 223.1692 | | | | 1.12E-02 | -0.56 | 1.28 | 3.53E-02 | -0.39 | 1.79 | 1.40E-03 | -0.76 | 1.74 | 1.26E-01 | -0.37 | 0.77 | 1276563 | 624143 | 952249 | 537599 | 1065053 | 568799 | 839445 | 484173 |
| RP_POS_445_36612a13_123 | RP | POS | 13.12 | 445.3661 | | | | 3.05E-01 | -0.19 | 0.86 | 3.53E-02 | -0.43 | 1.23 | 6.17E-02 | -0.41 | 1.00 | 9.06E-01 | 0.02 | 0.78 | 916522 | 194094 | 873784 | 229693 | 921963 | 214202 | 825605 | 236589 |
| RP_POS_538_34998a10_163 | RP | POS | 10.16 | 538.3500 | | | | 1.96E-11 | 0.82 | 1.58 | 3.54E-02 | 0.45 | 1.30 | 1.08E-06 | 1.05 | 1.62 | 2.05E-07 | 0.59 | 1.63 | 59887 | 47414 | 522250 | 607607 | 394302 | 389935 | 650197 | 748631 |
| RP_POS_990_7854a0_492 | RP | POS | 0.49 | 990.7854 | | | | 2.75E-01 | 0.15 | 0.74 | 3.55E-02 | 0.47 | 1.51 | 5.99E-02 | 0.39 | 0.84 | 5.58E-01 | -0.08 | 0.89 | 1776600 | 274706 | 1868323 | 681111 | 1725336 | 512019 | 2011311 | 795866 |
| RP_NEG_1096_76135a0_49 | RP | NEG | 0.49 | 1096.7614 | | | | 6.20E-07 | -0.76 | 1.24 | 3.56E-02 | 0.44 | 1.44 | 4.54E-03 | -0.54 | 1.02 | 7.50E-08 | -0.97 | 1.61 | 1015500 | 208258 | 726750 | 399145 | 643147 | 359696 | 810353 | 422185 |
| RP_POS_511_32394a9_957 | RP | POS | 9.96 | 511.3239 | | | | 2.07E-05 | 0.58 | 0.94 | 3.56E-02 | 0.46 | 1.25 | 2.87E-05 | 0.81 | 1.31 | 2.79E-02 | 0.35 | 0.71 | 224566 | 154410 | 444439 | 415492 | 357416 | 371161 | 531463 | 442238 |
| RP_POS_311_21945a10_579 | RP | POS | 10.58 | 311.2195 | | | | 8.67E-01 | 0.04 | 0.44 | 3.58E-02 | -0.40 | 1.30 | 5.07E-01 | -0.16 | 0.34 | 3.41E-01 | 0.24 | 0.60 | 4020325 | 1528536 | 4070588 | 1254040 | 4332888 | 1285221 | 3808289 | 1176912 |
| RP_NEG_195_8111a4_571 | RP | NEG | 4.57 | 195.8111 | | | | 2.64E-02 | -0.47 | 0.78 | 3.58E-02 | -0.40 | 1.36 | 4.39E-03 | -0.67 | 1.08 | 2.35E-01 | -0.27 | 0.61 | 13196716 | 5245401 | 10794239 | 4926578 | 11824599 | 4728955 | 9763879 | 4950559 |
| RP_POS_798_56366a12_279 | RP | POS | 12.28 | 798.5637 | | | | 3.37E-02 | 0.37 | 1.04 | 3.59E-02 | 0.44 | 1.52 | 4.48E-03 | 0.59 | 1.20 | 4.45E-01 | 0.15 | 0.85 | 478732 | 265431 | 607510 | 365546 | 531075 | 349663 | 683945 | 368495 |
| RP_POS_568_19092a11_014 | RP | POS | 11.01 | 568.1909 | | | | 4.49E-01 | 0.16 | 0.54 | 3.61E-02 | -0.42 | 1.28 | 8.07E-01 | -0.05 | 0.32 | 1.18E-01 | 0.36 | 0.77 | 478772 | 142342 | 500801 | 142305 | 530523 | 147085 | 471080 | 132203 |
| RP_POS_147_04408a0_71 | RP | POS | 0.71 | 147.0441 | | | | 3.08E-01 | 0.18 | 0.89 | 3.62E-02 | 0.44 | 1.32 | 6.74E-02 | 0.40 | 1.22 | 8.07E-01 | -0.05 | 0.58 | 1910041 | 400808 | 2002393 | 555593 | 1886342 | 464246 | 2118444 | 616973 |
| HILIC_POS_120_0658a1_03 | HILIC | POS | 1.03 | 120.0658 | | | | 1.27E-01 | 0.23 | 0.48 | 3.63E-02 | 0.46 | 1.19 | 3.96E-02 | 0.46 | 0.74 | 9.94E-01 | 0.00 | 0.68 | 3081967 | 1807344 | 3800257 | 3409523 | 3085062 | 2039424 | 4515453 | 4275956 |
| RP_NEG_1059_66504a10_368 | RP | NEG | 10.37 | 1059.6650 | | | | 8.07E-01 | -0.06 | 0.98 | 3.63E-02 | -0.39 | 1.52 | 3.19E-01 | -0.25 | 0.75 | 5.59E-01 | 0.14 | 0.87 | 2073493 | 1077916 | 2022036 | 865222 | 2202644 | 768297 | 1841427 | 925011 |
| HILIC_NEG_172_84219a3_03 | HILIC | NEG | 3.03 | 172.8422 | | | | 7.93E-03 | -0.37 | 1.33 | 3.63E-02 | -0.46 | 1.16 | 2.22E-03 | -0.60 | 1.45 | 3.86E-01 | -0.14 | 1.28 | 2381887 | 103218 | 2297296 | 254843 | 2350491 | 219606 | 2244101 | 277874 |
| RP_POS_241_01546a15_438 | RP | POS | 15.44 | 241.0155 | | | | 3.80E-01 | 0.18 | 0.61 | 3.64E-02 | -0.41 | 1.39 | 9.19E-01 | -0.02 | 0.64 | 8.38E-02 | 0.39 | 0.84 | 2106331 | 292062 | 2158451 | 282484 | 2217353 | 257154 | 2099548 | 296671 |
| RP_NEG_606_30359a9_984 | RP | NEG | 9.98 | 606.3036 | | | | 8.45E-19 | 1.13 | 1.96 | 3.67E-02 | 0.42 | 1.31 | 2.97E-10 | 1.34 | 2.04 | 1.53E-12 | 0.93 | 2.04 | 377552 | 149172 | 1074613 | 611824 | 946802 | 423701 | 1202424 | 737180 |
| HILIC_NEG_142_92889a1_049 | HILIC | NEG | 1.049 | 142.9289 | | | | 2.00E-01 | 0.26 | 0.68 | 3.68E-02 | 0.42 | 1.45 | 4.24E-02 | 0.47 | 0.97 | 8.10E-01 | 0.05 | 0.15 | 1007029 | 352279 | 1100083 | 356543 | 1025912 | 335926 | 1174255 | 364371 |
| RP_NEG_604_87225a0_495 | RP | NEG | 0.50 | 604.8723 | | | | 1.01E-06 | -0.69 | 1.16 | 3.74E-02 | 0.44 | 1.39 | 1.09E-02 | -0.46 | 0.87 | 2.33E-07 | -0.91 | 1.56 | 17203519 | 1182379 | 15390013 | 2831145 | 14802526 | 2586841 | 15977500 | 2966180 |
| RP_NEG_206_78928a15_581 | RP | NEG | 15.58 | 206.7893 | | | | 1.05E-01 | -0.31 | 0.70 | 3.74E-02 | -0.43 | 1.72 | 9.62E-03 | -0.52 | 1.20 | 6.84E-01 | -0.09 | 0.43 | 1030807 | 35831 | 1018292 | 42089 | 1027032 | 47536 | 1009552 | 34111 |
| RP_POS_422_24435a10_284 | RP | POS | 10.28 | 422.2444 | | | | 1.31E-01 | -0.31 | 0.85 | 3.75E-02 | 0.41 | 1.39 | 6.35E-01 | -0.11 | 0.46 | 2.22E-02 | -0.52 | 1.02 | 1167086 | 103982 | 1134841 | 101185 | 1113857 | 93880 | 1155824 | 104756 |
| RP_POS_965_63696a11_142 | RP | POS | 11.14 | 965.6370 | | | | 1.20E-07 | 0.64 | 1.31 | 3.76E-02 | -0.46 | 1.51 | 5.20E-04 | 0.41 | 1.29 | 3.02E-05 | 0.87 | 1.45 | 191368 | 96300 | 714996 | 906136 | 903350 | 1090904 | 526641 | 629641 |
| RP_POS_369_29736a10_947 | RP | POS | 10.95 | 369.2974 | | | | 4.58E-02 | -0.44 | 1.04 | 3.80E-02 | 0.39 | 1.22 | 2.98E-01 | -0.25 | 0.54 | 8.07E-03 | -0.64 | 1.18 | 15700362 | 3328372 | 14358120 | 2858056 | 13767230 | 2733798 | 14949011 | 2884001 |
| RP_POS_557_87524a11_121 | RP | POS | 11.12 | 557.8752 | | | | 1.11E-05 | 0.54 | 1.23 | 3.81E-02 | -0.46 | 1.46 | 9.30E-03 | 0.31 | 1.06 | 2.02E-04 | 0.77 | 1.37 | 334832 | 67842 | 496728 | 331816 | 565550 | 399889 | 427907 | 229942 |
| RP_POS_831_49713a10_872 | RP | POS | 10.87 | 831.4971 | | | | 7.88E-07 | 0.59 | 1.22 | 3.81E-02 | -0.46 | 1.64 | 8.64E-04 | 0.36 | 1.32 | 1.10E-04 | 0.82 | 1.39 | 44786 | 21915 | 1008884 | 1827945 | 1388320 | 2258482 | 629448 | 1164646 |
| HILIC_POS_257_14713a3_699 | HILIC | POS | 3.699 | 257.1471 | | | | 1.85E-03 | 0.41 | 0.90 | 3.84E-02 | 0.46 | 1.37 | 2.55E-03 | 0.64 | 1.07 | 1.41E-01 | 0.18 | 0.57 | 14847614 | 10836656 | 26628262 | 31714963 | 20055208 | 20699326 | 33201316 | 38930315 |
| RP_NEG_573_45459a11_525 | RP | NEG | 11.53 | 573.4546 | | | | 1.50E-01 | -0.38 | 0.63 | 3.84E-02 | -0.34 | 1.25 | 3.75E-02 | -0.55 | 1.02 | 4.69E-01 | -0.21 | 0.53 | 2256990 | 2946636 | 1450318 | 1757971 | 1814967 | 2196066 | 1085669 | 1072346 |
| HILIC_POS_140_06841a3_699 | HILIC | POS | 3.699 | 140.0684 | | | | 3.49E-01 | 0.18 | 0.74 | 3.84E-02 | 0.42 | 1.26 | 8.30E-02 | 0.39 | 0.96 | 8.80E-01 | -0.03 | 0.20 | 31950221 | 12177754 | 34361140 | 13702882 | 31532484 | 12259221 | 37189796 | 14586030 |
| RP_POS_267_6478a10_375 | RP | POS | 10.38 | 267.6478 | | | | 8.11E-01 | 0.05 | 0.85 | 3.85E-02 | -0.42 | 1.47 | 4.91E-01 | -0.16 | 0.70 | 2.49E-01 | 0.26 | 0.75 | 738873 | 117114 | 744590 | 118266 | 768993 | 109951 | 720187 | 122289 |
| RP_POS_740_54968a11_175 | RP | POS | 11.18 | 740.5497 | | | | 1.46E-06 | 0.57 | 1.24 | 3.85E-02 | -0.46 | 1.53 | 1.22E-03 | 0.34 | 1.28 | 1.51E-04 | 0.80 | 1.39 | 34308 | 13074 | 241925 | 404480 | 325729 | 501419 | 158121 | 254545 |
| RP_NEG_755_13904a9_304 | RP | NEG | 9.30 | 755.1390 | | | | 1.35E-01 | 0.25 | 0.63 | 3.86E-02 | 0.44 | 1.21 | 2.61E-02 | 0.47 | 0.80 | 8.99E-01 | 0.02 | 0.27 | 1104657 | 79832 | 1133173 | 125396 | 1107296 | 107559 | 1159050 | 137235 |
| HILIC_POS_352_24496a2_224 | HILIC | POS | 2.224 | 352.2450 | | | | 9.27E-01 | -0.02 | 0.65 | 3.87E-02 | -0.42 | 1.25 | 2.90E-01 | -0.23 | 0.55 | 3.88E-01 | 0.19 | 0.40 | 1904810 | 577872 | 1893832 | 631442 | 2023983 | 646537 | 1763681 | 594133 |
| RP_POS_383_11526a0_548 | RP | POS | 0.55 | 383.1153 | | | | 1.24E-04 | 0.49 | 0.92 | 3.90E-02 | 0.46 | 1.48 | 5.75E-04 | 0.72 | 1.11 | 3.40E-02 | 0.26 | 1.15 | 6825056 | 1185216 | 8669199 | 4160106 | 7810166 | 2860639 | 9528231 | 5027615 |
| HILIC_POS_184_07355a1_167 | HILIC | POS | 1.167 | 184.0736 | | | | 2.02E-01 | 0.28 | 0.66 | 3.90E-02 | -0.40 | 1.24 | 7.42E-01 | 0.08 | 0.17 | 4.39E-02 | 0.48 | 0.84 | 1382050 | 627378 | 1542952 | 561628 | 1658496 | 534969 | 1427409 | 569079 |
| RP_POS_813_55853a13_996 | RP | POS | 14.00 | 813.5585 | | | | 2.27E-01 | -0.27 | 0.71 | 3.91E-02 | 0.39 | 1.41 | 7.61E-01 | -0.07 | 0.70 | 5.51E-02 | -0.47 | 0.85 | 2218615 | 957654 | 1988620 | 815079 | 1821040 | 792634 | 2156201 | 810361 |

**Supplementary table 1: Metabolomics results** (in asceding order according to p-value from progressive ALD vs  non-progressive ALD comparison, with molecular features with ID_level of 1-2 first)

| Feature_ID | RP_HILIC | POS_NEG | Average.Rt | Average.Mz | Metabolite.name | curated_MSMS | ID_level | all_ALD_vs_control t-test p | all_ALD_vs_control Cohen's d | all_ALD_vs_control PLS-DA VIP | Prog_ALD_vs_non-prog_ALD t-test p | Prog_ALD_vs_non-prog_ALD Cohen's d | Prog_ALD_vs_non-prog_ALD PLS-DA VIP | Prog_ALD_vs_control_ALD t-test p | Prog_ALD_vs_control_ALD Cohen's d | Prog_ALD_vs_control_ALD PLS-DA VIP | Non-prog_ALD_vs_control t-test p | Non-prog_ALD_vs_control Cohen's d | Non-prog_ALD_vs_control PLS-DA VIP | Healthy_control Mean | Healthy_control SD | all_ALD Mean | all_ALD SD | Non-prog_ALD Mean | Non-prog_ALD SD | Progressive_ALD Mean | Progressive_ALD SD |
|---|---|---|---|---|---|---|---|---|---|---|---|---|---|---|---|---|---|---|---|---|---|---|---|---|---|---|---|
| RP_POS_936_72302a0_498 | RP | POS | 0.50 | 936.7230 | | | | 1.61E-01 | 0.25 | 0.69 | 3.93E-02 | -0.43 | 1.40 | 8.83E-01 | 0.03 | 0.44 | 2.57E-02 | 0.46 | 1.12 | 367269 | 129429 | 407998 | 174447 | 443841 | 174627 | 372155 | 168428 |
| RP_POS_795_57465a13_297 | RP | POS | 13.30 | 795.5747 | | | | 1.38E-01 | -0.32 | 0.66 | 3.93E-02 | 0.40 | 1.26 | 6.13E-01 | -0.12 | 0.59 | 2.83E-02 | -0.52 | 0.95 | 3347652 | 1305871 | 2954391 | 1208627 | 2706148 | 1188797 | 3202635 | 1188430 |
| HILIC_POS_166_08627a4_496 | HILIC | POS | 4.496 | 166.0863 | | | | 1.74E-01 | 0.24 | 0.64 | 3.94E-02 | -0.44 | 1.37 | 9.21E-01 | 0.02 | 0.26 | 5.32E-02 | 0.45 | 0.76 | 1387734 | 849620 | 1650735 | 1187151 | 1895763 | 1490279 | 1405708 | 709900 |
| RP_POS_429_37354a13_124 | RP | POS | 13.12 | 429.3735 | | | | 1.30E-01 | -0.27 | 1.07 | 3.94E-02 | -0.43 | 1.23 | 2.87E-02 | -0.49 | 1.22 | 7.55E-01 | -0.06 | 0.79 | 4382885 | 726392 | 4140437 | 923096 | 4330177 | 769850 | 3950697 | 1027356 |
| RP_POS_330_24615a8_699 | RP | POS | 8.70 | 330.2462 | | | | 2.16E-02 | 0.41 | 1.06 | 3.95E-02 | 0.43 | 1.25 | 4.75E-03 | 0.62 | 1.15 | 3.01E-01 | 0.19 | 0.69 | 1979724 | 911192 | 2452917 | 1207959 | 2204727 | 1018313 | 2701107 | 1336203 |
| HILIC_NEG_198_90411a0_881 | HILIC | NEG | 0.881 | 198.9041 | | | | 1.27E-01 | 0.30 | 0.71 | 3.96E-02 | 0.42 | 1.36 | 3.17E-02 | 0.51 | 0.83 | 6.41E-01 | 0.09 | 0.23 | 1051623 | 579657 | 1237288 | 620579 | 1109793 | 497150 | 1364783 | 705523 |
| HILIC_NEG_187_94209a6_093 | HILIC | NEG | 6.093 | 187.9421 | | | | 8.09E-02 | -0.31 | 1.04 | 3.96E-02 | -0.43 | 1.21 | 1.40E-02 | -0.52 | 0.97 | 6.37E-01 | -0.09 | 1.16 | 446442 | 102569 | 406108 | 137843 | 434389 | 126315 | 377827 | 144227 |
| RP_POS_793_55548a12_651 | RP | POS | 12.65 | 793.5555 | | | | 1.17E-12 | -1.54 | 2.67 | 3.97E-02 | -0.30 | 1.28 | 6.87E-14 | -1.69 | 2.73 | 1.71E-10 | -1.39 | 2.21 | 2216920 | 706675 | 881470 | 636997 | 1012197 | 701976 | 750744 | 540523 |
| RP_POS_491_83658a11_136 | RP | POS | 11.14 | 491.8366 | | | | 2.36E-05 | 0.51 | 1.16 | 3.99E-02 | -0.46 | 1.48 | 2.55E-02 | 0.28 | 0.99 | 2.42E-04 | 0.74 | 1.35 | 222819 | 42729 | 354400 | 288383 | 413602 | 337674 | 295198 | 216450 |
| RP_POS_535_2876a8_888 | RP | POS | 8.89 | 535.2876 | | | | 1.24E-01 | -0.50 | 0.98 | 4.00E-02 | -0.22 | 1.37 | 6.49E-02 | -0.61 | 0.91 | 2.35E-01 | -0.39 | 0.64 | 708624 | 911127 | 450514 | 281049 | 508062 | 283152 | 392966 | 269548 |
| RP_POS_597_44818a11_52 | RP | POS | 11.52 | 597.4482 | | | | 1.29E-01 | -0.42 | 0.67 | 4.00E-02 | -0.33 | 1.25 | 3.75E-02 | -0.58 | 1.00 | 3.85E-01 | -0.25 | 0.53 | 2276137 | 3016327 | 1409356 | 1652917 | 1748672 | 1969530 | 1070040 | 1185754 |
| RP_NEG_1028_77051a0_489 | RP | NEG | 0.49 | 1028.7705 | | | | 8.75E-09 | -0.76 | 1.33 | 4.01E-02 | 0.44 | 1.46 | 1.31E-03 | -0.54 | 1.17 | 8.21E-08 | -0.98 | 1.63 | 1954526 | 249738 | 1407197 | 772255 | 1249151 | 762459 | 1565243 | 756708 |
| RP_POS_552_33093a10_822 | RP | POS | 10.82 | 552.3309 | | | | 2.78E-03 | -0.80 | 1.46 | 4.04E-02 | -0.32 | 1.62 | 6.12E-04 | -0.96 | 1.52 | 1.85E-02 | -0.64 | 1.15 | 385231 | 243793 | 240205 | 139752 | 268767 | 143665 | 211643 | 130979 |
| RP_POS_251_12784a8_87 | RP | POS | 8.87 | 251.1278 | | | | 3.15E-05 | -1.05 | 1.79 | 4.04E-02 | -0.32 | 1.32 | 4.35E-06 | -1.21 | 1.88 | 5.33E-04 | -0.90 | 1.48 | 5502110 | 2664173 | 3145189 | 1740685 | 3501013 | 1881800 | 2789364 | 1523892 |
| RP_POS_490_20676a7_364 | RP | POS | 7.36 | 490.2068 | | | | 1.66E-04 | 0.53 | 1.74 | 4.05E-02 | -0.45 | 1.21 | 5.48E-02 | 0.31 | 1.54 | 1.33E-04 | 0.75 | 1.31 | 341750 | 254947 | 633527 | 600065 | 756168 | 650046 | 510885 | 523688 |
| RP_POS_199_14418a1_858 | RP | POS | 1.86 | 199.1442 | | | | 4.86E-03 | 0.34 | 0.64 | 4.05E-02 | 0.47 | 1.26 | 1.03E-02 | 0.57 | 0.91 | 1.74E-01 | 0.10 | 0.42 | 591880 | 1074970 | 2713055 | 7133930 | 1244037 | 3075111 | 4182073 | 9432079 |
| HILIC_POS_144_10202a5_185 | HILIC | POS | 5.185 | 144.1020 | | | | 8.07E-05 | 0.53 | 1.10 | 4.07E-02 | 0.45 | 1.52 | 5.89E-04 | 0.76 | 1.07 | 8.30E-03 | 0.31 | 0.89 | 1629933 | 553087 | 2358253 | 1496654 | 2051182 | 856140 | 2665323 | 1897432 |
| RP_NEG_636_3504a10_822 | RP | NEG | 10.82 | 636.3504 | | | | 1.19E-02 | -0.61 | 1.30 | 4.10E-02 | -0.36 | 1.73 | 2.43E-03 | -0.79 | 1.34 | 8.80E-02 | -0.43 | 1.02 | 4182070 | 1290472 | 3532737 | 945521 | 3725379 | 919482 | 3340095 | 940787 |
| RP_POS_534_29608a10_503 | RP | POS | 10.50 | 534.2961 | | | | 1.24E-01 | 0.33 | 1.04 | 4.10E-02 | -0.40 | 1.32 | 5.94E-01 | 0.13 | 0.65 | 2.17E-02 | 0.53 | 1.19 | 8285248 | 2263256 | 8995603 | 2140981 | 9431851 | 1953252 | 8559355 | 2248903 |
| RP_POS_550_63068a10_818 | RP | POS | 10.82 | 550.6307 | | | | 9.21E-02 | -0.34 | 0.73 | 4.12E-02 | 0.41 | 1.50 | 5.68E-01 | -0.14 | 0.49 | 8.14E-03 | -0.55 | 1.11 | 1558656 | 267312 | 1465164 | 271475 | 1409715 | 185311 | 1520612 | 329069 |
| HILIC_POS_494_32355a1_198 | HILIC | POS | 1.198 | 494.3236 | | | | 1.24E-15 | 1.05 | 1.72 | 4.12E-02 | 0.41 | 1.12 | 4.01E-09 | 1.26 | 1.92 | 1.05E-10 | 0.84 | 2.09 | 2372110 | 1123370 | 6145572 | 3631955 | 5403920 | 2478686 | 6887224 | 4402836 |
| RP_POS_723_52484a11_174 | RP | POS | 11.17 | 723.5248 | | | | 5.74E-05 | 0.50 | 1.15 | 4.13E-02 | -0.45 | 1.50 | 1.81E-02 | 0.28 | 1.19 | 5.35E-04 | 0.73 | 1.32 | 238561 | 125701 | 477403 | 528483 | 585329 | 647490 | 369476 | 348243 |
| RP_POS_414_77185a12_955 | RP | POS | 12.96 | 414.7719 | | | | 8.76E-06 | -0.86 | 1.41 | 4.14E-02 | -0.39 | 1.27 | 1.35E-06 | -1.05 | 1.74 | 1.40E-03 | -0.66 | 1.12 | 2094125 | 427587 | 1655020 | 493706 | 1755418 | 483200 | 1554623 | 488275 |
| RP_POS_231_08386a1_43 | RP | POS | 1.43 | 231.0839 | | | | 8.21E-13 | 0.88 | 1.56 | 4.15E-02 | 0.43 | 1.28 | 2.45E-07 | 1.10 | 1.63 | 4.24E-08 | 0.66 | 1.65 | 76097 | 90089 | 852557 | 938703 | 661061 | 635017 | 1044053 | 1141151 |
| HILIC_POS_104_08233a2_484 | HILIC | POS | 2.484 | 104.0823 | | | | 6.61E-05 | 0.56 | 1.07 | 4.15E-02 | 0.44 | 1.17 | 1.32E-04 | 0.78 | 1.38 | 2.48E-02 | 0.34 | 1.03 | 642753 | 174487 | 857588 | 418345 | 772441 | 336164 | 942735 | 475179 |
| RP_POS_123_0441a0_71 | RP | POS | 0.71 | 123.0441 | | | | 1.79E-01 | 0.22 | 0.82 | 4.16E-02 | 0.44 | 1.38 | 4.02E-02 | 0.44 | 1.22 | 9.74E-01 | 0.01 | 0.52 | 1195690 | 259947 | 1277543 | 391960 | 1197809 | 320720 | 1357278 | 441095 |
| RP_POS_441_29593a12_887 | RP | POS | 12.89 | 441.2959 | | | | 9.98E-01 | 0.00 | 0.45 | 4.16E-02 | -0.40 | 1.40 | 3.92E-01 | -0.20 | 0.78 | 4.05E-01 | 0.20 | 0.37 | 19554750 | 7288184 | 19559087 | 6636335 | 20907444 | 6853475 | 18210731 | 6188718 |
| RP_NEG_918_80426a0_489 | RP | NEG | 0.49 | 918.8043 | | | | 2.25E-04 | -0.55 | 1.01 | 4.17E-02 | 0.44 | 1.33 | 8.55E-02 | -0.33 | 0.62 | 1.11E-05 | -0.77 | 1.47 | 1188291 | 242137 | 942580 | 479896 | 845085 | 419434 | 1040075 | 519594 |
| HILIC_POS_568_33844a1_072 | HILIC | POS | 1.072 | 568.3384 | | | | 6.14E-01 | -0.10 | 0.49 | 4.18E-02 | -0.42 | 1.13 | 1.51E-01 | -0.31 | 0.57 | 5.96E-01 | 0.12 | 0.20 | 1470946 | 659376 | 1400214 | 769107 | 1556333 | 778575 | 1244095 | 734305 |
| RP_POS_162_05811a0_731 | RP | POS | 0.73 | 162.0581 | | | | 1.31E-03 | 0.38 | 0.77 | 4.18E-02 | 0.47 | 1.21 | 8.01E-03 | 0.61 | 0.92 | 2.58E-04 | 0.14 | 1.33 | 107005 | 177398 | 1086906 | 2947989 | 481616 | 644838 | 1692196 | 4049089 |
| HILIC_POS_160_13315a1_954 | HILIC | POS | 1.954 | 160.1332 | | | | 9.03E-01 | -0.02 | 0.27 | 4.19E-02 | 0.45 | 1.31 | 3.77E-01 | 0.20 | 0.38 | 8.72E-02 | -0.24 | 0.66 | 784716 | 589883 | 767086 | 1001097 | 562635 | 521625 | 971538 | 1291295 |
| RP_NEG_766_56116a10_509 | RP | NEG | 10.51 | 766.5612 | | | | 4.51E-02 | -0.36 | 1.10 | 4.19E-02 | 0.42 | 1.41 | 4.68E-01 | -0.15 | 0.81 | 5.76E-03 | -0.57 | 1.15 | 1144457 | 204496 | 1053135 | 263350 | 999717 | 253667 | 1106553 | 264483 |
| RP_NEG_822_53113a12_382 | RP | NEG | 12.38 | 822.5311 | | | | 6.46E-02 | -0.39 | 1.21 | 4.19E-02 | -0.40 | 1.23 | 8.66E-03 | -0.59 | 1.42 | 4.25E-01 | -0.19 | 0.54 | 1699252 | 843006 | 1378987 | 807324 | 1542864 | 887664 | 1215110 | 688544 |
| RP_POS_744_55261a13_974 | RP | POS | 13.97 | 744.5526 | | | | 6.32E-01 | 0.10 | 0.45 | 4.20E-02 | 0.41 | 1.26 | 2.19E-01 | 0.30 | 0.51 | 6.08E-01 | -0.11 | 0.44 | 856650 | 868244 | 941734 | 872124 | 764504 | 643938 | 1118963 | 1028574 |
| RP_POS_331_26227a11_38 | RP | POS | 11.38 | 331.2623 | | | | 5.17E-10 | 0.85 | 1.61 | 4.21E-02 | 0.43 | 1.30 | 2.25E-07 | 1.06 | 1.73 | 1.39E-05 | 0.64 | 1.50 | 606427 | 288676 | 1223631 | 761165 | 1069160 | 604131 | 1378103 | 869998 |
| RP_POS_327_2291a10_943 | RP | POS | 10.94 | 327.2291 | | | | 1.92E-06 | 0.81 | 1.66 | 4.24E-02 | 0.41 | 1.44 | 2.91E-06 | 1.01 | 1.71 | 5.23E-04 | 0.61 | 1.53 | 5124255 | 2723070 | 8354123 | 4018715 | 7539690 | 3254628 | 9168556 | 4547906 |
| RP_POS_584_2937a10_125 | RP | POS | 10.13 | 584.2937 | | | | 4.55E-24 | 1.37 | 2.24 | 4.27E-02 | 0.37 | 1.28 | 5.66E-14 | 1.55 | 2.32 | 3.53E-16 | 1.18 | 2.32 | 225257 | 92965 | 601726 | 250240 | 551057 | 189529 | 652396 | 292159 |
| HILIC_NEG_247_89224a0_881 | HILIC | NEG | 0.881 | 247.8922 | | | | 1.99E-01 | 0.26 | 0.68 | 4.29E-02 | 0.41 | 1.35 | 5.34E-02 | 0.46 | 0.77 | 7.88E-01 | 0.06 | 0.12 | 1628262 | 825192 | 1847928 | 852199 | 1675614 | 691321 | 2020243 | 963736 |
| HILIC_POS_140_06827a3_921 | HILIC | POS | 3.921 | 140.0683 | | | | 2.13E-03 | 0.38 | 0.84 | 4.29E-02 | 0.46 | 1.34 | 6.59E-03 | 0.61 | 0.85 | 7.18E-02 | 0.15 | 0.58 | 891014 | 388651 | 1495315 | 1798964 | 1129084 | 783773 | 1861546 | 2377091 |
| RP_POS_235_16524a0_551 | RP | POS | 0.55 | 235.1652 | | | | 4.05E-03 | 0.34 | 0.78 | 4.30E-02 | 0.46 | 1.28 | 1.13E-02 | 0.58 | 0.95 | 6.66E-02 | 0.11 | 0.62 | 334098 | 176420 | 694460 | 1187544 | 452470 | 391612 | 936450 | 1605127 |
| RP_POS_469_32837a11_155 | RP | POS | 11.16 | 469.3284 | | | | 8.31E-02 | -0.39 | 1.10 | 4.31E-02 | -0.38 | 1.83 | 1.50E-02 | -0.58 | 1.43 | 4.27E-01 | -0.19 | 0.83 | 3884576 | 2200326 | 3119213 | 1882686 | 3499018 | 2010163 | 2739409 | 1681131 |
| RP_POS_223_09438a13_013 | RP | POS | 13.01 | 223.0944 | | | | 5.05E-01 | 0.14 | 0.55 | 4.33E-02 | -0.40 | 1.21 | 7.78E-01 | -0.07 | 0.26 | 1.20E-01 | 0.34 | 0.91 | 12698281 | 3226739 | 13139189 | 3249648 | 13794407 | 2817040 | 12483970 | 3538937 |
| RP_POS_772_54767a12_45 | RP | POS | 12.45 | 772.5477 | | | | 2.03E-03 | 0.60 | 1.01 | 4.34E-02 | 0.40 | 1.32 | 3.88E-04 | 0.80 | 1.26 | 5.31E-02 | 0.40 | 0.77 | 412209 | 340023 | 642359 | 381906 | 565427 | 349711 | 719291 | 400381 |
| RP_POS_921_61694a9_303 | RP | POS | 9.30 | 921.6169 | | | | 5.07E-02 | 0.24 | 0.84 | 4.37E-02 | 0.46 | 1.63 | 4.30E-02 | 0.47 | 0.93 | 9.42E-01 | 0.00 | 1.02 | 882210 | 769750 | 1807167 | 4481033 | 895019 | 785694 | 2719315 | 6184966 |
| RP_POS_1049_65857a11_113 | RP | POS | 11.11 | 1049.6586 | | | | 1.71E-02 | 0.29 | 0.97 | 4.38E-02 | -0.46 | 1.31 | 6.50E-01 | 0.06 | 0.66 | 1.00E-02 | 0.51 | 1.14 | 948041 | 77421 | 1076299 | 511843 | 1179440 | 604279 | 973158 | 377426 |
| RP_POS_315_16895a10_823 | RP | POS | 10.82 | 315.1690 | | | | 4.91E-10 | 0.83 | 1.69 | 4.39E-02 | 0.42 | 1.46 | 1.51E-07 | 1.04 | 1.83 | 4.27E-05 | 0.62 | 1.59 | 126986 | 142226 | 451163 | 410592 | 368574 | 348572 | 533752 | 452978 |
| HILIC_NEG_243_79901a0_881 | HILIC | NEG | 0.881 | 243.7990 | | | | 1.56E-01 | 0.28 | 0.70 | 4.39E-02 | 0.41 | 1.33 | 3.92E-02 | 0.48 | 0.81 | 7.14E-01 | 0.08 | 0.16 | 134552 | 65109 | 153926 | 70340 | 139776 | 58840 | 168076 | 78266 |
| RP_POS_553_4588a13_479 | RP | POS | 13.48 | 553.4588 | | | | 7.57E-03 | 0.52 | 0.93 | 4.40E-02 | 0.40 | 1.38 | 1.25E-03 | 0.72 | 1.35 | 1.29E-01 | 0.32 | 0.56 | 8800593 | 3891771 | 11049185 | 4318066 | 10181880 | 4102598 | 11916491 | 4393797 |
| HILIC_NEG_130_06213a5_548 | HILIC | NEG | 5.548 | 130.0621 | | | | 1.25E-01 | 0.29 | 0.53 | 4.41E-02 | -0.41 | 1.47 | 7.10E-01 | 0.08 | 0.51 | 1.75E-02 | 0.50 | 0.94 | 198786 | 127005 | 240761 | 150138 | 270916 | 136662 | 210606 | 158147 |
| HILIC_NEG_538_31622a1_289 | HILIC | NEG | 1.289 | 538.3162 | | | | 2.33E-15 | 1.08 | 1.76 | 4.41E-02 | 0.40 | 1.09 | 1.29E-09 | 1.28 | 1.99 | 2.57E-10 | 0.88 | 2.08 | 387829 | 200977 | 966570 | 533022 | 859276 | 384232 | 1073864 | 634740 |
| RP_POS_741_47552a11_146 | RP | POS | 11.15 | 741.4755 | | | | 3.70E-05 | 0.49 | 1.12 | 4.41E-02 | -0.45 | 1.46 | 4.65E-03 | 0.26 | 1.25 | 9.62E-04 | 0.72 | 1.24 | 84352 | 44318 | 379995 | 680564 | 517422 | 870323 | 242568 | 373875 |
| RP_NEG_774_84094a0_49 | RP | NEG | 0.49 | 774.8409 | | | | 9.82E-01 | 0.00 | 0.68 | 4.42E-02 | 0.44 | 1.45 | 2.92E-01 | 0.22 | 0.62 | 1.65E-01 | -0.23 | 0.93 | 1551262 | 342707 | 1549232 | 653331 | 1417896 | 518018 | 1680568 | 747562 |
| HILIC_NEG_200_8587a4_848 | HILIC | NEG | 4.848 | 200.8587 | | | | 1.51E-01 | -0.24 | 0.48 | 4.43E-02 | -0.43 | 1.12 | 2.36E-02 | -0.46 | 0.83 | 8.97E-01 | -0.03 | 0.23 | 4021484 | 754296 | 3767444 | 1133482 | 3994838 | 1096649 | 3540050 | 1134731 |
| RP_NEG_999_64355a10_625 | RP | NEG | 10.63 | 999.6436 | | | | 2.38E-03 | 0.57 | 1.30 | 4.44E-02 | 0.41 | 1.64 | 9.79E-04 | 0.77 | 1.31 | 4.92E-02 | 0.36 | 1.13 | 2177106 | 1136305 | 2949016 | 1381033 | 2671438 | 1007899 | 3226594 | 1637169 |
| RP_POS_358_27725a9_131 | RP | POS | 9.13 | 358.2773 | | | | 4.34E-01 | 0.14 | 0.61 | 4.46E-02 | 0.42 | 1.21 | 1.14E-01 | 0.35 | 0.74 | 7.34E-01 | -0.07 | 0.43 | 1623685 | 760791 | 1752987 | 943864 | 1563778 | 800438 | 1942196 | 1042199 |
| RP_POS_489_28226a10_565 | RP | POS | 10.57 | 489.2823 | | | | 5.01E-01 | 0.13 | 0.63 | 4.47E-02 | 0.41 | 1.24 | 1.71E-01 | 0.34 | 0.61 | 7.01E-01 | -0.07 | 0.23 | 572334 | 867839 | 694287 | 942409 | 504820 | 594539 | 883754 | 1169469 |
| HILIC_NEG_308_9978a0_931 | HILIC | NEG | 0.931 | 308.9978 | | | | 5.64E-01 | -0.11 | 0.82 | 4.49E-02 | -0.41 | 1.32 | 1.50E-01 | -0.32 | 0.52 | 6.57E-01 | 0.10 | 0.18 | 700931 | 123639 | 685886 | 139636 | 713822 | 133968 | 657950 | 140905 |
| RP_NEG_246_98051a6_83 | RP | NEG | 6.83 | 246.9805 | | | | 4.97E-01 | 0.12 | 0.72 | 4.49E-02 | 0.42 | 1.41 | 1.38E-01 | 0.33 | 0.64 | 6.55E-01 | -0.09 | 0.45 | 4294135 | 1925662 | 4578647 | 2407897 | 4096518 | 1979262 | 5060777 | 2705994 |
| RP_POS_536_29614a10_367 | RP | POS | 10.37 | 536.2961 | | | | 5.63E-01 | -0.13 | 0.98 | 4.50E-02 | -0.39 | 1.53 | 1.96E-01 | -0.32 | 0.80 | 7.72E-01 | 0.07 | 0.83 | 1110529 | 442301 | 1059675 | 387559 | 1137208 | 339390 | 982142 | 419542 |
| RP_POS_391_28421a9_293 | RP | POS | 9.29 | 391.2842 | | | | 9.94E-01 | 0.00 | 0.37 | 4.50E-02 | 0.40 | 1.19 | 4.46E-01 | 0.20 | 0.43 | 3.20E-01 | -0.20 | 0.52 | 471058 | 804165 | 469871 | 761792 | 316633 | 406839 | 623108 | 979315 |
| RP_POS_548_50317a12_891 | RP | POS | 12.89 | 548.5032 | | | | 7.66E-01 | -0.05 | 0.70 | 4.51E-02 | -0.43 | 1.50 | 2.34E-01 | -0.27 | 0.53 | 3.39E-01 | 0.16 | 0.65 | 3531011 | 777136 | 3477673 | 1139478 | 3705908 | 838756 | 3249438 | 1346662 |
| RP_NEG_557_45856a12_261 | RP | NEG | 12.26 | 557.4586 | | | | 7.94E-02 | -0.41 | 0.66 | 4.53E-02 | -0.36 | 1.27 | 1.55E-02 | -0.60 | 1.18 | 3.69E-01 | -0.23 | 0.50 | 2740385 | 2203828 | 1977958 | 1682154 | 2314110 | 1874055 | 1641806 | 1405112 |
| RP_POS_516_30591a9_985 | RP | POS | 9.99 | 516.3059 | LPC 16:1 [M+Na]+ | 457.2347(55), 516.3019(45), 311.2558(40), 86.097(37), 104.107(3) | | 3.23E-13 | 0.94 | 1.77 | 4.54E-02 | 0.42 | 1.26 | 9.58E-08 | 1.15 | 1.79 | 4.78E-09 | 0.73 | 1.77 | 1135834 | 634429 | 3260197 | 2360778 | 2787368 | 1559118 | 3733026 | 2893645 |
| RP_POS_274_1228a10_123 | RP | POS | 10.12 | 274.1228 | | | | 2.37E-20 | 1.27 | 2.10 | 4.54E-02 | 0.38 | 1.29 | 5.24E-12 | 1.46 | 2.18 | 9.58E-15 | 1.08 | 2.24 | 769941 | 278813 | 1728703 | 708633 | 1586882 | 496795 | 1870524 | 852477 |
| RP_NEG_181_08611a6_273 | RP | NEG | 6.27 | 181.0861 | | | | 2.27E-01 | -0.18 | 1.00 | 4.56E-02 | -0.44 | 1.37 | 3.60E-02 | -0.40 | 1.20 | 8.27E-01 | 0.04 | 0.96 | 1536345 | 57198 | 1518064 | 111578 | 1540313 | 105933 | 1495815 | 113672 |
| HILIC_NEG_214_07687a0_699 | HILIC | NEG | 0.699 | 214.0769 | | | | 4.98E-07 | 0.61 | 1.11 | 4.57E-02 | 0.44 | 1.43 | 1.40E-04 | 0.83 | 1.28 | 6.46E-05 | 0.39 | 1.26 | 65562 | 122187 | 508310 | 800930 | 347706 | 438663 | 668914 | 1025184 |
| RP_POS_802_53448a12_578 | RP | POS | 12.58 | 802.5345 | | | | 6.71E-06 | 0.81 | 1.46 | 4.57E-02 | 0.40 | 1.22 | 1.19E-05 | 1.00 | 1.57 | 4.70E-04 | 0.61 | 1.35 | 4843585 | 2120134 | 7130692 | 2825358 | 6565803 | 1964569 | 7695582 | 3408439 |
| HILIC_POS_156_04214a3_921 | HILIC | POS | 3.921 | 156.0421 | | | | 1.69E-02 | 0.30 | 0.77 | 4.57E-02 | 0.45 | 1.28 | 1.77E-02 | 0.53 | 0.82 | 4.08E-01 | 0.08 | 0.39 | 839320 | 359945 | 1200057 | 1347015 | 929552 | 621522 | 1470563 | 1769267 |
| RP_POS_592_42346a10_833 | RP | POS | 10.83 | 592.4235 | | | | 8.77E-10 | 1.16 | 2.03 | 4.58E-02 | 0.36 | 1.59 | 1.26E-09 | 1.34 | 2.10 | 2.68E-07 | 0.98 | 1.91 | 744335 | 540028 | 1578634 | 652176 | 1448572 | 549239 | 1708696 | 723298 |
| RP_POS_774_56299a12_345 | RP | POS | 12.35 | 774.5630 | | | | 6.19E-02 | 0.35 | 0.82 | 4.58E-02 | 0.41 | 1.36 | 1.30E-02 | 0.56 | 1.12 | 4.66E-01 | 0.15 | 0.67 | 1523951 | 607201 | 1769212 | 714931 | 1626749 | 636375 | 1911675 | 765696 |
| RP_NEG_203_00162a3_93 | RP | NEG | 3.93 | 203.0016 | | | | 2.54E-04 | 0.44 | 0.73 | 4.59E-02 | -0.45 | 1.20 | 7.38E-02 | 0.22 | 0.68 | 1.01E-03 | 0.67 | 1.00 | 901946 | 2227810 | 5951207 | 12779967 | 8503014 | 15180168 | 3399399 | 9287711 |
| RP_POS_300_19925a7_851 | RP | POS | 7.85 | 300.1993 | | | | 3.50E-01 | -0.20 | 1.02 | 4.60E-02 | -0.39 | 1.70 | 8.01E-02 | -0.40 | 1.03 | 9.83E-01 | -0.01 | 0.70 | 1349084 | 686844 | 1220023 | 630859 | 1345709 | 699064 | 1094336 | 532126 |
| RP_POS_351_28671a11_342 | RP | POS | 11.34 | 351.2867 | | | | 1.63E-01 | 0.21 | 0.73 | 4.60E-02 | 0.44 | 1.28 | 4.26E-02 | 0.43 | 0.85 | 9.81E-01 | 0.00 | 0.65 | 1132632 | 117478 | 1174526 | 213733 | 1131894 | 161937 | 1217158 | 249727 |
| RP_POS_215_16412a8_932 | RP | POS | 8.93 | 215.1641 | | | | 1.24E-08 | 0.71 | 1.33 | 4.61E-02 | 0.43 | 1.23 | 2.64E-05 | 0.93 | 1.39 | 8.85E-07 | 0.50 | 1.50 | 370357 | 151221 | 812894 | 673830 | 678021 | 357147 | 947767 | 867572 |
| HILIC_NEG_199_80501a0_733 | HILIC | NEG | 0.733 | 199.8050 | | | | 5.17E-05 | 0.49 | 1.09 | 4.64E-02 | 0.45 | 1.13 | 5.46E-04 | 0.71 | 1.06 | 2.44E-02 | 0.27 | 0.70 | 133497 | 59187 | 297276 | 374769 | 222598 | 262081 | 371954 | 451334 |
| HILIC_NEG_163_89758a6_276 | HILIC | NEG | 6.276 | 163.8976 | | | | 1.08E-02 | -0.51 | 0.82 | 4.65E-02 | -0.39 | 1.23 | 1.73E-03 | -0.71 | 1.14 | 1.49E-01 | -0.32 | 0.65 | 4236979 | 1599499 | 3369633 | 1677389 | 3702704 | 1638847 | 3036562 | 1665074 |
| RP_POS_565_56689a15_222 | RP | POS | 15.22 | 565.5669 | | | | 6.46E-01 | -0.09 | 0.74 | 4.66E-02 | -0.41 | 1.33 | 2.34E-01 | -0.29 | 1.01 | 5.63E-01 | 0.11 | 0.44 | 8173379 | 780260 | 8099004 | 833145 | 8265045 | 537434 | 7932962 | 1028264 |
| RP_POS_277_14078a8_586 | RP | POS | 8.59 | 277.1408 | | | | 1.64E-01 | 0.27 | 0.87 | 4.66E-02 | 0.40 | 1.21 | 5.85E-02 | 0.48 | 0.75 | 6.95E-01 | 0.07 | 0.42 | 910814 | 473606 | 1049020 | 510037 | 947146 | 269719 | 1150894 | 657005 |
| RP_POS_644_49329a10_55 | RP | POS | 10.55 | 644.4933 | | | | 4.80E-01 | -0.15 | 0.42 | 4.66E-02 | 0.39 | 1.16 | 8.42E-01 | 0.05 | 0.45 | 1.24E-01 | -0.35 | 0.64 | 869129 | 178168 | 843658 | 169386 | 810025 | 148996 | 877291 | 182922 |
| RP_POS_987_63837a10_123 | RP | POS | 10.12 | 987.6384 | | | | 8.07E-05 | 0.47 | 1.51 | 4.67E-02 | 0.45 | 1.27 | 1.80E-03 | 0.69 | 1.25 | 2.53E-03 | 0.24 | 1.39 | 170066 | 249591 | 2079144 | 4623686 | 1154826 | 2169707 | 3003461 | 6061519 |
| RP_POS_365_19815a11_38 | RP | POS | 11.38 | 365.1982 | | | | 8.84E-10 | 0.89 | 1.78 | 4.68E-02 | 0.41 | 1.36 | 1.60E-07 | 1.09 | 1.81 | 7.02E-06 | 0.68 | 1.69 | 750136 | 401668 | 1504687 | 877386 | 1330423 | 689426 | 1678952 | 1008979 |
| RP_NEG_363_9559a4_567 | RP | NEG | 4.57 | 363.9559 | | | | 3.90E-03 | -0.65 | 1.02 | 4.68E-02 | -0.36 | 1.29 | 5.45E-04 | -0.83 | 1.44 | 5.26E-02 | -0.47 | 0.74 | 997449 | 594221 | 643891 | 498812 | 742823 | 527082 | 544959 | 452676 |
| RP_NEG_1083_66528a10_198 | RP | NEG | 10.20 | 1083.6653 | | | | 3.80E-02 | -0.55 | 1.23 | 4.72E-02 | -0.32 | 1.45 | 1.05E-02 | -0.71 | 1.19 | 1.58E-01 | -0.38 | 0.99 | 1850320 | 1430116 | 1276245 | 863382 | 1447148 | 867676 | 1105342 | 832826 |
| RP_POS_819_96564a10_313 | RP | POS | 10.31 | 819.9656 | | | | 6.89E-01 | -0.08 | 1.07 | 4.73E-02 | -0.39 | 1.60 | 2.42E-01 | -0.28 | 0.96 | 6.09E-01 | 0.11 | 0.84 | 402258 | 179535 | 387724 | 172935 | 421967 | 152161 | 353482 | 186754 |
| RP_POS_544_33655a10_308 | RP | POS | 10.31 | 544.3366 | | | | 2.39E-02 | 0.47 | 0.99 | 4.73E-02 | -0.39 | 1.57 | 2.42E-01 | 0.28 | 0.59 | 2.64E-03 | 0.66 | 1.22 | 158221120 | 51840915 | 182585937 | 50592522 | 192605904 | 42100955 | 172565970 | 56515976 |
| RP_POS_377_12958a0_707 | RP | POS | 0.71 | 377.1296 | | | | 5.33E-13 | 0.89 | 1.55 | 4.75E-02 | 0.42 | 1.18 | 4.77E-07 | 1.10 | 1.66 | 1.23E-09 | 0.67 | 1.79 | 76112 | 70171 | 870085 | 952592 | 680855 | 571124 | 1059314 | 1197549 |
| RP_POS_199_14407a2_25 | RP | POS | 2.25 | 199.1441 | | | | 2.49E-02 | 0.27 | 0.54 | 4.75E-02 | 0.45 | 1.22 | 2.55E-02 | 0.50 | 0.83 | 5.53E-01 | 0.05 | 0.38 | 986352 | 1371339 | 2712688 | 7200352 | 1277566 | 3000166 | 4147809 | 9567862 |

**Supplementary table 1: Metabolomics results** (in asceding order according to p-value from progressive ALD vs  non-progressive ALD comparison, with molecular features with ID_level of 1-2 first)

| | | | | | | | | all_ALD_vs_control | | | Prog_ALD_vs_non-prog_ALD | | | Prog_ALD_vs_control_ALD | | | Non-prog_ALD_vs_control | | | Healthy_control | | all_ALD | | Non-prog_ALD | | Progressive_ALD | |
|---|---|---|---|---|---|---|---|---|---|---|---|---|---|---|---|---|---|---|---|---|---|---|---|---|---|---|---|
| | | | | | | | | t-test | Cohen's | PLS-DA | t-test | Cohen's | PLS-DA | t-test | Cohen's | PLS-DA | t-test | Cohen's | PLS-DA | | | | | | | | |
| Feature_ID | RP_HILIC | POS_NEG | Average.Rt | Average.Mz | Metabolite.name | curated_MSMS | ID_level | p | d | VIP | p | d | VIP | p | d | VIP | p | d | VIP | Mean | SD | Mean | SD | Mean | SD | Mean | SD |
| HILIC_NEG_245_89449a0_881 | HILIC | NEG | 0.881 | 245.8945 | | | | 1.88E-01 | 0.26 | 0.69 | 4.76E-02 | 0.40 | 1.33 | 5.18E-02 | 0.46 | 0.77 | 7.56E-01 | 0.06 | 0.14 | 11374423 | 5598566 | 12908407 | 5864448 | 11748343 | 4779718 | 14068472 | 6625197 |
| RP_POS_331_28378a10_864 | RP | POS | 10.86 | 331.2838 | | | | 3.37E-01 | -0.21 | 0.55 | 4.77E-02 | 0.38 | 1.18 | 9.29E-01 | -0.02 | 0.31 | 6.03E-02 | -0.40 | 0.87 | 484997 | 746086 | 343187 | 632718 | 217247 | 291331 | 469127 | 831620 |
| RP_NEG_203_0817a2_891 | RP | NEG | 2.89 | 203.0817 | | | | 2.22E-05 | -0.86 | 1.55 | 4.77E-02 | -0.37 | 1.40 | 9.41E-06 | -1.05 | 1.65 | 8.92E-04 | -0.67 | 1.40 | 4378090 | 1074878 | 3353498 | 1122315 | 3575432 | 905086 | 3131563 | 1274828 |
| HILIC_NEG_478_29453a1_767 | HILIC | NEG | 1.767 | 478.2945 | | | | 4.98E-04 | 0.62 | 1.01 | 4.78E-02 | 0.40 | 1.25 | 3.57E-04 | 0.82 | 1.19 | 1.61E-02 | 0.42 | 1.00 | 337427 | 183649 | 485851 | 244025 | 437530 | 170654 | 534171 | 293980 |
| HILIC_NEG_243_89899a0_881 | HILIC | NEG | 0.881 | 243.8990 | | | | 1.92E-01 | 0.26 | 0.69 | 4.79E-02 | 0.40 | 1.33 | 5.27E-02 | 0.46 | 0.77 | 7.67E-01 | 0.06 | 0.13 | 29330435 | 14205185 | 33189065 | 14930726 | 30238458 | 12239770 | 36139672 | 16817349 |
| HILIC_NEG_197_90608a0_881 | HILIC | NEG | 0.881 | 197.9061 | | | | 1.83E-01 | 0.27 | 0.68 | 4.81E-02 | 0.40 | 1.32 | 5.06E-02 | 0.47 | 0.77 | 7.35E-01 | 0.07 | 0.14 | 148239 | 74363 | 168788 | 76773 | 153633 | 62786 | 183944 | 86588 |
| RP_POS_732_54834a10_459 | RP | POS | 10.46 | 732.5483 | | | | 3.37E-01 | -0.16 | 0.36 | 4.82E-02 | -0.43 | 1.34 | 1.53E-02 | -0.37 | 1.00 | 8.28E-01 | 0.05 | 0.41 | 1132489 | 186025 | 1091111 | 274588 | 1145582 | 356675 | 1036640 | 138070 |
| RP_POS_363_1962a11_38 | RP | POS | 11.38 | 363.1962 | | | | 1.02E-09 | 0.88 | 1.72 | 4.83E-02 | 0.41 | 1.31 | 1.86E-07 | 1.08 | 1.78 | 8.24E-06 | 0.68 | 1.62 | 215099 | 122724 | 446048 | 271126 | 392543 | 213664 | 499553 | 311488 |
| HILIC_POS_375_32129a1_117 | HILIC | POS | 1.117 | 375.3213 | | | | 9.19E-03 | -0.57 | 1.03 | 4.83E-02 | -0.37 | 1.22 | 1.89E-03 | -0.76 | 1.29 | 9.29E-02 | -0.39 | 0.69 | 5460934 | 1630654 | 4585155 | 1446034 | 4870088 | 1349828 | 4300222 | 1495653 |
| RP_POS_617_47498a11_603 | RP | POS | 11.60 | 617.4750 | | | | 4.48E-02 | -0.40 | 0.66 | 4.84E-02 | -0.40 | 1.23 | 3.49E-03 | -0.60 | 1.17 | 4.01E-01 | -0.20 | 0.58 | 1254352 | 809480 | 911074 | 865776 | 1081996 | 1030388 | 740151 | 626985 |
| HILIC_POS_189_0731a2_225 | HILIC | POS | 2.225 | 189.0731 | | | | 5.10E-01 | -0.13 | 0.75 | 4.85E-02 | -0.40 | 1.20 | 1.42E-01 | -0.33 | 0.64 | 7.56E-01 | 0.07 | 0.31 | 4388199 | 1111648 | 4236353 | 1172885 | 4467226 | 1130527 | 4005481 | 1180052 |
| RP_POS_526_43097a10_66 | RP | POS | 10.66 | 526.4310 | | | | 8.05E-01 | -0.05 | 0.35 | 4.87E-02 | 0.41 | 1.22 | 4.52E-01 | 0.15 | 0.36 | 2.75E-01 | -0.25 | 0.60 | 11990732 | 757972 | 11951370 | 849916 | 11784081 | 933712 | 12118660 | 728371 |
| RP_NEG_554_34747a10_668 | RP | NEG | 10.67 | 554.3475 | | | | 1.18E-05 | -0.91 | 1.59 | 4.89E-02 | -0.36 | 1.42 | 1.06E-06 | -1.09 | 1.86 | 1.14E-03 | -0.73 | 1.23 | 8821216 | 2859192 | 6013256 | 2835115 | 6570577 | 3030050 | 5455935 | 2534698 |
| RP_POS_599_44495a11_441 | RP | POS | 11.44 | 599.4450 | | | | 8.44E-06 | -1.29 | 2.22 | 4.89E-02 | -0.23 | 1.17 | 2.47E-06 | -1.40 | 2.00 | 3.96E-05 | -1.17 | 1.96 | 1330305 | 784564 | 585428 | 336559 | 651558 | 319258 | 519298 | 343518 |
| HILIC_NEG_157_90102a0_708 | HILIC | NEG | 0.708 | 157.9010 | | | | 6.48E-06 | 0.72 | 1.44 | 4.91E-02 | -0.41 | 1.09 | 8.85E-03 | 0.52 | 0.93 | 4.24E-07 | 0.93 | 1.92 | 798803 | 228315 | 1065177 | 381683 | 1140156 | 332030 | 990198 | 415475 |
| RP_NEG_207_83682a15_552 | RP | NEG | 15.55 | 207.8368 | | | | 7.35E-01 | 0.07 | 0.50 | 4.92E-02 | -0.39 | 1.18 | 5.85E-01 | -0.13 | 0.31 | 2.34E-01 | 0.27 | 0.68 | 5226441 | 321516 | 5248665 | 319407 | 5311350 | 296754 | 5185981 | 331725 |
| HILIC_POS_398_32581a0_618 | HILIC | POS | 0.618 | 398.3258 | | | | 2.93E-07 | 0.86 | 1.59 | 4.93E-02 | 0.39 | 1.20 | 1.35E-07 | 1.06 | 2.00 | 4.14E-04 | 0.67 | 1.28 | 682912 | 476072 | 1302599 | 717443 | 1161864 | 700133 | 1443333 | 713685 |
| RP_POS_192_13853a15_382 | RP | POS | 15.38 | 192.1385 | | | | 2.83E-01 | -0.24 | 0.45 | 4.93E-02 | -0.37 | 1.76 | 7.87E-02 | -0.43 | 0.85 | 8.10E-01 | -0.06 | 0.44 | 3617860 | 2024828 | 3189510 | 1651717 | 3513504 | 1701490 | 2865516 | 1550070 |
| HILIC_NEG_161_91367a0_881 | HILIC | NEG | 0.881 | 161.9137 | | | | 2.54E-01 | 0.23 | 0.77 | 4.94E-02 | 0.40 | 1.35 | 7.23E-02 | 0.43 | 0.76 | 8.82E-01 | 0.03 | 0.15 | 1395926 | 420861 | 1495483 | 438617 | 1409410 | 363372 | 1581556 | 491464 |
| RP_POS_144_1387a7_136 | RP | POS | 7.14 | 144.1387 | | | | 7.51E-01 | 0.07 | 0.39 | 4.95E-02 | -0.38 | 1.23 | 6.28E-01 | -0.12 | 0.24 | 2.92E-01 | 0.26 | 0.60 | 1574072 | 739419 | 1620257 | 621924 | 1742173 | 629939 | 1498341 | 595244 |
| HILIC_POS_249_10707a1_232 | HILIC | POS | 1.232 | 249.1071 | | | | 7.70E-10 | -1.31 | 2.41 | 4.95E-02 | -0.32 | 1.06 | 6.29E-10 | -1.47 | 2.27 | 2.60E-08 | -1.14 | 2.64 | 5870275 | 1507443 | 3580988 | 1440539 | 3864114 | 1031732 | 3297862 | 1721799 |
| HILIC_NEG_249_89191a0_881 | HILIC | NEG | 0.881 | 249.8919 | | | | 1.87E-01 | 0.27 | 0.72 | 4.96E-02 | 0.39 | 1.33 | 5.34E-02 | 0.47 | 0.77 | 7.26E-01 | 0.07 | 0.17 | 347764 | 172301 | 394555 | 173087 | 360604 | 141479 | 428505 | 195347 |
| RP_POS_510_35522a10_547 | RP | POS | 10.55 | 510.3552 | | | | 2.56E-05 | -0.88 | 1.56 | 4.96E-02 | -0.36 | 1.39 | 2.19E-06 | -1.06 | 1.82 | 2.11E-03 | -0.70 | 1.20 | 2355282 | 828960 | 1579457 | 818474 | 1739862 | 883962 | 1419053 | 720813 |
| HILIC_POS_152_02191a1_232 | HILIC | POS | 1.232 | 152.0219 | | | | 9.83E-10 | -1.18 | 2.09 | 4.97E-02 | -0.35 | 1.08 | 3.11E-09 | -1.36 | 2.09 | 7.89E-08 | -1.01 | 2.40 | 2557537 | 339977 | 2037877 | 390277 | 2114515 | 279021 | 1961240 | 466800 |
| RP_POS_236_87883a0_252 | RP | POS | 0.25 | 236.8788 | | | | 7.91E-02 | 0.33 | 0.97 | 4.97E-02 | 0.40 | 1.67 | 1.68E-02 | 0.53 | 1.36 | 5.37E-01 | 0.12 | 0.58 | 696532 | 420549 | 857227 | 507772 | 757780 | 459248 | 956675 | 538364 |
| RP_POS_652_49902a11_189 | RP | POS | 11.19 | 652.4990 | | | | 4.45E-05 | 0.49 | 1.14 | 4.97E-02 | -0.44 | 1.46 | 6.31E-03 | 0.26 | 1.22 | 1.03E-03 | 0.71 | 1.26 | 73724 | 41259 | 261265 | 433909 | 346666 | 551080 | 175864 | 248642 |
| HILIC_POS_346_0094a6_3 | HILIC | POS | 6.3 | 346.0094 | | | | 6.20E-01 | -0.10 | 0.35 | 4.98E-02 | -0.39 | 1.08 | 1.92E-01 | -0.30 | 0.51 | 6.67E-01 | 0.10 | 0.19 | 28718187 | 8635720 | 27840788 | 8771834 | 29557603 | 8510152 | 26123973 | 8776249 |
| RP_POS_237_14597a8_931 | RP | POS | 8.93 | 237.1460 | | | | 7.15E-09 | 0.72 | 1.35 | 5.01E-02 | 0.43 | 1.23 | 2.01E-05 | 0.94 | 1.40 | 1.06E-06 | 0.51 | 1.48 | 1338999 | 471394 | 2769033 | 2142210 | 2347904 | 1195436 | 3190162 | 2735100 |
| HILIC_POS_248_14923a4_251 | HILIC | POS | 4.251 | 248.1492 | | | | 5.28E-05 | 0.54 | 1.09 | 5.02E-02 | 0.43 | 1.50 | 7.62E-04 | 0.76 | 1.06 | 2.12E-03 | 0.33 | 1.05 | 2266903 | 4112794 | 7956865 | 11493475 | 5695452 | 5635168 | 10218279 | 14990131 |
| HILIC_NEG_253_79378a3_05 | HILIC | NEG | 3.05 | 253.7938 | | | | 1.06E-01 | 0.25 | 0.95 | 5.03E-02 | -0.43 | 1.20 | 8.62E-01 | 0.03 | 0.51 | 1.83E-02 | 0.46 | 0.97 | 452871 | 88315 | 490078 | 166319 | 522568 | 171971 | 457587 | 155435 |
| RP_POS_188_07062a3_06 | RP | POS | 3.06 | 188.0706 | | | | 4.05E-02 | -0.55 | 1.01 | 5.03E-02 | -0.31 | 1.24 | 9.83E-03 | -0.71 | 1.23 | 1.69E-01 | -0.39 | 0.55 | 2111283 | 1813637 | 1395644 | 1049477 | 1601075 | 1237811 | 1190214 | 779091 |
| RP_POS_986_69482a0_5 | RP | POS | 0.50 | 986.6948 | | | | 9.69E-03 | 0.46 | 0.99 | 5.04E-02 | -0.41 | 1.44 | 1.87E-01 | 0.26 | 0.64 | 1.91E-03 | 0.66 | 1.33 | 283461 | 160665 | 377875 | 212627 | 419399 | 221905 | 336352 | 196423 |
| HILIC_NEG_216_9808a0_826 | HILIC | NEG | 0.826 | 216.9808 | | | | 1.12E-05 | 0.58 | 1.25 | 5.04E-02 | 0.43 | 1.40 | 2.09E-04 | 0.80 | 1.15 | 3.10E-03 | 0.37 | 0.95 | 226989 | 61863 | 325747 | 186342 | 289229 | 121610 | 362266 | 229444 |
| RP_NEG_897_62317a9_312 | RP | NEG | 9.31 | 897.6232 | | | | 2.16E-02 | 0.27 | 0.91 | 5.05E-02 | 0.45 | 1.61 | 3.07E-02 | 0.50 | 0.95 | 2.82E-01 | 0.05 | 1.31 | 1077585 | 1570065 | 4580179 | 14760036 | 1668946 | 3320133 | 7491412 | 20293931 |
| RP_NEG_704_3385a10_824 | RP | NEG | 10.82 | 704.3385 | | | | 1.06E-02 | -0.62 | 1.32 | 5.08E-02 | -0.34 | 1.76 | 2.34E-03 | -0.79 | 1.36 | 7.63E-02 | -0.44 | 1.03 | 1135528 | 340896 | 961147 | 249598 | 1009796 | 244933 | 912497 | 247078 |
| RP_POS_558_29565a10_198 | RP | POS | 10.20 | 558.2957 | | | | 6.52E-02 | -0.49 | 1.19 | 5.08E-02 | -0.32 | 1.55 | 2.06E-02 | -0.65 | 1.10 | 2.29E-01 | -0.32 | 0.97 | 522956 | 320175 | 409446 | 192915 | 447041 | 184122 | 371851 | 195975 |
| HILIC_POS_371_24026a1_353 | HILIC | POS | 1.353 | 371.2403 | | | | 3.24E-03 | -0.54 | 0.92 | 5.11E-02 | 0.40 | 1.14 | 1.22E-01 | -0.35 | 0.62 | 1.51E-04 | -0.74 | 1.53 | 1808928 | 280352 | 1623895 | 346767 | 1556297 | 272214 | 1691492 | 399403 |
| HILIC_POS_160_09683a0_769 | HILIC | POS | 0.769 | 160.0968 | | | | 2.57E-02 | 0.37 | 0.71 | 5.12E-02 | 0.41 | 1.12 | 5.94E-03 | 0.58 | 0.99 | 3.63E-01 | 0.17 | 0.33 | 1253999 | 1025805 | 1794718 | 1539028 | 1495067 | 1351848 | 2094370 | 1665777 |
| RP_POS_785_50189a11_143 | RP | POS | 11.14 | 785.5019 | | | | 8.71E-06 | 0.53 | 1.19 | 5.13E-02 | -0.44 | 1.46 | 3.05E-03 | 0.31 | 1.26 | 4.40E-04 | 0.75 | 1.32 | 79395 | 44669 | 567382 | 1037796 | 770086 | 1294605 | 364679 | 645106 |
| HILIC_NEG_134_89474a4_522 | HILIC | NEG | 4.522 | 134.8947 | | | | 4.43E-01 | -0.16 | 0.38 | 5.16E-02 | 0.38 | 1.13 | 9.01E-01 | 0.03 | 0.25 | 1.21E-01 | -0.35 | 0.97 | 24930269 | 16895409 | 22311997 | 15926559 | 19217443 | 14639066 | 25406551 | 16692561 |
| RP_POS_619_33777a14_261 | RP | POS | 14.26 | 619.3378 | | | | 5.98E-01 | -0.12 | 0.36 | 5.16E-02 | 0.37 | 1.42 | 8.03E-01 | 0.06 | 0.36 | 2.15E-01 | -0.30 | 0.80 | 3330284 | 1363498 | 3189682 | 1096611 | 2976696 | 1033453 | 3402668 | 1126522 |
| RP_POS_518_37616a11_485 | RP | POS | 11.49 | 518.3762 | | | | 1.96E-02 | -0.48 | 0.77 | 5.17E-02 | 0.38 | 1.41 | 2.13E-01 | -0.29 | 0.69 | 2.23E-03 | -0.67 | 1.19 | 1461657 | 219739 | 1354463 | 217374 | 1312229 | 183038 | 1396697 | 241503 |
| RP_NEG_251_78772a15_552 | RP | NEG | 15.55 | 251.7877 | | | | 4.13E-02 | -0.49 | 0.91 | 5.18E-02 | -0.35 | 1.22 | 9.63E-03 | -0.66 | 1.06 | 2.14E-01 | -0.31 | 0.62 | 1902535 | 102200 | 1861289 | 75890 | 1876019 | 76561 | 1846559 | 73019 |
| HILIC_POS_260_00885a0_541 | HILIC | POS | 0.541 | 260.0089 | | | | 7.58E-03 | 0.69 | 1.89 | 5.18E-02 | 0.32 | 1.18 | 2.94E-03 | 0.85 | 1.84 | 3.48E-02 | 0.53 | 1.47 | 23451136 | 4586599 | 25876205 | 2910089 | 25308764 | 1745286 | 26443645 | 3661546 |
| RP_POS_617_47375a11_16 | RP | POS | 11.16 | 617.4738 | | | | 1.43E-01 | -0.28 | 0.56 | 5.20E-02 | -0.40 | 1.21 | 1.19E-02 | -0.48 | 1.05 | 7.23E-01 | -0.09 | 0.63 | 1602950 | 1330143 | 1191054 | 1473878 | 1478104 | 1846388 | 904004 | 900920 |
| RP_POS_241_15385a0_71 | RP | POS | 0.71 | 241.1539 | | | | 5.03E-04 | 0.54 | 0.91 | 5.21E-02 | 0.42 | 1.29 | 4.25E-04 | 0.74 | 1.25 | 4.26E-02 | 0.33 | 0.84 | 781772 | 124474 | 895852 | 228131 | 851561 | 182117 | 940143 | 260728 |
| RP_POS_423_7695a13_208 | RP | POS | 13.21 | 423.7695 | | | | 8.87E-04 | 0.72 | 1.25 | 5.22E-02 | 0.36 | 1.40 | 2.31E-04 | 0.90 | 1.38 | 1.47E-02 | 0.54 | 1.02 | 787853 | 321410 | 1014648 | 295707 | 957312 | 255133 | 1071983 | 323833 |
| RP_NEG_568_36279a10_822 | RP | NEG | 10.82 | 568.3628 | | | | 2.37E-02 | -0.55 | 1.23 | 5.23E-02 | -0.34 | 1.68 | 5.93E-03 | -0.72 | 1.24 | 1.31E-01 | -0.38 | 0.97 | 34231813 | 11701699 | 28998895 | 8304197 | 30607399 | 8016792 | 27390392 | 8353298 |
| RP_NEG_299_25983a10_358 | RP | NEG | 10.36 | 299.2598 | | | | 2.53E-02 | 0.44 | 1.05 | 5.24E-02 | 0.39 | 1.24 | 6.32E-03 | 0.63 | 1.10 | 2.36E-01 | 0.24 | 0.76 | 2362333 | 867710 | 2777197 | 954258 | 2592341 | 818013 | 2962054 | 1049246 |
| RP_POS_524_37048a11_249 | RP | POS | 11.25 | 524.3705 | | | | 1.65E-01 | -0.30 | 1.07 | 5.26E-02 | -0.38 | 1.38 | 4.12E-02 | -0.49 | 0.94 | 6.38E-01 | -0.11 | 0.98 | 2737035 | 1122670 | 2421221 | 1037825 | 2621960 | 996118 | 2220482 | 1049597 |
| RP_POS_179_10696a6_861 | RP | POS | 6.86 | 179.1070 | | | | 4.43E-01 | 0.09 | 0.57 | 5.27E-02 | 0.44 | 1.17 | 1.46E-01 | 0.32 | 0.67 | 1.60E-01 | -0.13 | 0.80 | 1326507 | 53753 | 1348722 | 272445 | 1295694 | 137739 | 1401750 | 353916 |
| RP_POS_537_39233a15_125 | RP | POS | 15.13 | 537.3923 | | | | 8.24E-01 | 0.05 | 0.44 | 5.33E-02 | 0.37 | 1.33 | 3.62E-01 | 0.23 | 0.58 | 5.62E-01 | -0.13 | 0.31 | 4851852 | 3085912 | 4986367 | 2561444 | 4491538 | 2036993 | 5481196 | 2933742 |
| HILIC_POS_544_33899a1_204 | HILIC | POS | 1.204 | 544.3390 | | | | 9.01E-02 | 0.36 | 0.83 | 5.35E-02 | -0.38 | 1.11 | 4.69E-01 | 0.17 | 0.33 | 1.68E-02 | 0.55 | 1.04 | 45722724 | 22141836 | 53391417 | 20888975 | 57417848 | 19072510 | 49364987 | 22017197 |
| RP_POS_872_62769a11_156 | RP | POS | 11.16 | 872.6277 | | | | 1.32E-06 | 0.58 | 1.26 | 5.37E-02 | -0.43 | 1.47 | 2.13E-03 | 0.36 | 1.23 | 1.27E-04 | 0.79 | 1.41 | 62127 | 18859 | 281056 | 424005 | 362921 | 510600 | 199192 | 298063 |
| RP_POS_151_00517a11_211 | RP | POS | 11.21 | 151.0052 | | | | 6.13E-02 | -0.36 | 0.96 | 5.39E-02 | -0.39 | 1.22 | 1.24E-02 | -0.56 | 0.93 | 4.26E-01 | -0.17 | 1.04 | 2376564 | 358144 | 2233883 | 397200 | 2310294 | 377585 | 2157471 | 405302 |
| RP_NEG_199_8047a4_571 | RP | NEG | 4.57 | 199.8047 | | | | 5.00E-03 | -0.60 | 0.97 | 5.40E-02 | -0.37 | 1.18 | 1.03E-03 | -0.78 | 1.22 | 6.41E-02 | -0.42 | 0.76 | 7683948 | 2748856 | 6068352 | 2572582 | 6563105 | 2419658 | 5573599 | 2648984 |
| RP_POS_899_62939a11_151 | RP | POS | 11.15 | 899.6294 | | | | 5.77E-07 | 0.59 | 1.28 | 5.42E-02 | -0.43 | 1.48 | 1.25E-03 | 0.38 | 1.28 | 8.69E-05 | 0.81 | 1.42 | 29761 | 11847 | 257141 | 424917 | 339026 | 510946 | 175256 | 300074 |
| HILIC_POS_264_19574a0_714 | HILIC | POS | 0.714 | 264.1957 | | | | 2.62E-02 | 0.26 | 0.86 | 5.42E-02 | -0.44 | 1.45 | 1.52E-01 | 0.04 | 0.65 | 3.71E-02 | 0.48 | 0.89 | 10237 | 5284 | 14911428 | 66027855 | 27742602 | 91494960 | 2080255 | 10050005 |
| RP_POS_397_3107a11_609 | RP | POS | 11.61 | 397.3107 | | | | 3.18E-02 | -0.41 | 0.95 | 5.43E-02 | 0.39 | 1.16 | 2.81E-01 | -0.22 | 0.49 | 7.71E-03 | -0.61 | 1.23 | 4730507 | 775484 | 4372798 | 874913 | 4204661 | 951161 | 4540935 | 764307 |
| HILIC_NEG_112_98573a0_927 | HILIC | NEG | 0.927 | 112.9857 | | | | 3.87E-02 | -0.45 | 1.05 | 5.45E-02 | -0.37 | 1.16 | 7.79E-03 | -0.63 | 1.13 | 2.54E-01 | -0.27 | 0.49 | 5336710 | 1004404 | 4913978 | 894320 | 5085624 | 896930 | 4742333 | 866791 |
| RP_POS_726_82373a0_493 | RP | POS | 0.49 | 726.8237 | | | | 2.42E-08 | -0.78 | 1.33 | 5.47E-02 | 0.40 | 1.39 | 9.78E-04 | -0.58 | 1.15 | 8.13E-08 | -0.98 | 1.63 | 2440629 | 368837 | 1781009 | 892328 | 1609886 | 868100 | 1952132 | 891846 |
| RP_POS_293_09735a0_753 | RP | POS | 0.75 | 293.0974 | | | | 4.86E-02 | -0.38 | 0.71 | 5.48E-02 | -0.39 | 1.39 | 2.82E-03 | -0.57 | 1.22 | 4.46E-01 | -0.18 | 0.70 | 49024943 | 34473671 | 34424276 | 39282819 | 41983813 | 48585924 | 26864740 | 25309184 |
| RP_POS_327_33542a10_849 | RP | POS | 10.85 | 327.3354 | | | | 3.20E-04 | -0.77 | 1.29 | 5.49E-02 | -0.36 | 1.27 | 1.90E-05 | -0.95 | 1.64 | 1.43E-02 | -0.59 | 1.14 | 3143446 | 619571 | 2663865 | 580924 | 2775446 | 689157 | 2552285 | 426013 |
| RP_POS_716_52216a13_151 | RP | POS | 13.15 | 716.5222 | | | | 3.42E-01 | 0.18 | 0.50 | 5.50E-02 | 0.40 | 1.26 | 1.02E-01 | 0.38 | 0.61 | 9.00E-01 | -0.02 | 0.42 | 672354 | 801247 | 837223 | 977276 | 649738 | 771626 | 1024707 | 1123604 |
| RP_POS_257_14709a0_55 | RP | POS | 0.55 | 257.1471 | | | | 4.75E-04 | 0.44 | 0.90 | 5.50E-02 | 0.43 | 1.26 | 2.78E-03 | 0.66 | 1.10 | 2.65E-02 | 0.23 | 0.71 | 2310479 | 1285827 | 4274252 | 4979586 | 3315025 | 2696096 | 5233480 | 6399365 |
| RP_POS_241_10295a0_553 | RP | POS | 0.55 | 241.1030 | | | | 2.74E-01 | -0.18 | 0.88 | 5.51E-02 | -0.41 | 1.30 | 7.46E-02 | -0.39 | 0.75 | 8.82E-01 | 0.03 | 0.90 | 1963475 | 402458 | 1859800 | 617422 | 1978198 | 488983 | 1741402 | 708862 |
| RP_NEG_221_94638a15_474 | RP | NEG | 15.47 | 221.9464 | | | | 2.93E-01 | 0.21 | 1.06 | 5.52E-02 | -0.38 | 1.38 | 9.28E-01 | 0.02 | 0.54 | 7.08E-02 | 0.41 | 0.91 | 21968071 | 4622053 | 22970414 | 4734053 | 23876349 | 4539639 | 22064478 | 4795480 |
| RP_POS_275_62604a10_124 | RP | POS | 10.12 | 275.6260 | | | | 2.36E-20 | 1.30 | 2.14 | 5.53E-02 | 0.36 | 1.23 | 1.73E-12 | 1.48 | 2.22 | 1.05E-14 | 1.12 | 2.25 | 2532616 | 822626 | 5235509 | 1926556 | 4866038 | 1403643 | 5604979 | 2291328 |
| RP_POS_696_52594a11_183 | RP | POS | 11.18 | 696.5259 | | | | 3.39E-05 | 0.49 | 1.15 | 5.58E-02 | -0.43 | 1.44 | 5.91E-03 | 0.28 | 1.21 | 9.23E-04 | 0.71 | 1.29 | 63953 | 34640 | 243055 | 408548 | 321428 | 514792 | 164682 | 244299 |
| RP_POS_620_69714a10_841 | RP | POS | 10.84 | 620.6971 | | | | 7.73E-01 | -0.04 | 1.32 | 5.58E-02 | -0.43 | 1.26 | 6.89E-02 | -0.25 | 1.06 | 4.22E-01 | 0.17 | 1.10 | 751655 | 174631 | 737442 | 383323 | 810881 | 469586 | 664002 | 255646 |
| RP_POS_379_28134a10_874 | RP | POS | 10.87 | 379.2813 | | | | 4.41E-04 | 0.45 | 0.83 | 5.58E-02 | 0.43 | 1.18 | 2.64E-03 | 0.67 | 0.95 | 1.85E-02 | 0.24 | 0.87 | 258207 | 328753 | 705894 | 1096742 | 495193 | 562732 | 916595 | 1422309 |
| RP_POS_276_62238a10_123 | RP | POS | 10.12 | 276.6224 | | | | 8.12E-21 | 1.32 | 2.17 | 5.59E-02 | 0.35 | 1.22 | 1.03E-12 | 1.50 | 2.25 | 2.90E-15 | 1.14 | 2.29 | 859651 | 296687 | 1846709 | 688474 | 1714957 | 496157 | 1978460 | 822234 |
| RP_POS_767_55023a11_168 | RP | POS | 11.17 | 767.5502 | | | | 8.51E-07 | 0.59 | 1.27 | 5.59E-02 | -0.43 | 1.47 | 1.04E-03 | 0.37 | 1.29 | 1.28E-04 | 0.80 | 1.40 | 37443 | 17183 | 333290 | 562367 | 440959 | 685687 | 225620 | 380975 |
| RP_POS_333_14444a6_868 | RP | POS | 6.87 | 333.1444 | | | | 6.70E-01 | 0.06 | 0.64 | 5.61E-02 | 0.43 | 1.45 | 2.33E-01 | 0.27 | 0.59 | 1.25E-01 | -0.16 | 0.82 | 486003 | 328105 | 528051 | 794636 | 375183 | 292627 | 680920 | 1068859 |
| RP_NEG_624_26141a10_123 | RP | NEG | 10.12 | 624.2614 | | | | 1.82E-20 | 1.27 | 2.10 | 5.61E-02 | 0.36 | 1.21 | 2.92E-12 | 1.45 | 2.20 | 4.71E-14 | 1.09 | 2.17 | 404636 | 160544 | 961173 | 410675 | 882683 | 307758 | 1039662 | 483179 |
| RP_POS_273_121a0_55 | RP | POS | 0.55 | 273.1210 | | | | 1.15E-01 | 0.21 | 0.88 | 5.62E-02 | 0.43 | 1.23 | 4.38E-02 | 0.43 | 1.03 | 9.89E-01 | 0.00 | 0.85 | 296276 | 157877 | 377186 | 426603 | 295597 | 285405 | 458775 | 522162 |
| HILIC_NEG_343_72137a1_364 | HILIC | NEG | 1.364 | 343.7214 | | | | 2.56E-08 | 0.97 | 1.67 | 5.64E-02 | -0.37 | 1.02 | 1.09E-04 | 0.78 | 1.39 | 2.58E-09 | 1.15 | 2.18 | 159708 | 42398 | 219556 | 60064 | 231003 | 52553 | 208110 | 65268 |
| HILIC_NEG_88_0399a5_548 | HILIC | NEG | 5.548 | 88.0399 | | | | 1.02E-01 | 0.31 | 0.54 | 5.64E-02 | -0.39 | 1.40 | 5.94E-01 | 0.12 | 0.41 | 1.51E-02 | 0.51 | 1.00 | 183672 | 128015 | 228328 | 145209 | 255999 | 128013 | 200657 | 157009 |
| RP_POS_608_47235a11_194 | RP | POS | 11.19 | 608.4724 | | | | 1.12E-05 | 0.52 | 1.17 | 5.65E-02 | -0.43 | 1.44 | 1.95E-03 | 0.31 | 1.29 | 6.10E-04 | 0.74 | 1.29 | 44031 | 25819 | 240675 | 422840 | 321569 | 534947 | 159781 | 248421 |
| HILIC_NEG_160_93544a0_926 | HILIC | NEG | 0.926 | 160.9354 | | | | 3.17E-02 | -0.33 | 0.72 | 5.69E-02 | -0.42 | 1.07 | 3.01E-04 | -0.54 | 1.38 | 5.82E-01 | -0.12 | 0.30 | 763336 | 174947 | 666150 | 321434 | 727516 | 402226 | 604784 | 198185 |
| RP_POS_637_49054a10_376 | RP | POS | 10.38 | 637.4905 | | | | 2.24E-02 | -0.48 | 1.07 | 5.71E-02 | 0.37 | 1.30 | 2.04E-01 | -0.29 | 0.65 | 3.80E-03 | -0.66 | 1.21 | 1161860 | 98343 | 1115126 | 95808 | 1096926 | 90575 | 1133325 | 98315 |
| RP_NEG_465_24997a8_447 | RP | NEG | 8.45 | 465.2500 | | | | 2.83E-01 | -0.22 | 0.51 | 5.72E-02 | -0.38 | 1.13 | 6.23E-02 | -0.41 | 0.85 | 8.94E-01 | -0.03 | 0.56 | 1043844 | 724592 | 884359 | 720595 | 1021256 | 783776 | 747463 | 629731 |
| RP_POS_634_53656a10_861 | RP | POS | 10.86 | 634.5366 | | | | 8.47E-01 | -0.03 | 0.58 | 5.73E-02 | 0.43 | 1.13 | 3.77E-01 | 0.19 | 0.54 | 9.17E-02 | -0.24 | 0.90 | 455497 | 116318 | 448920 | 269712 | 397597 | 190705 | 500243 | 324392 |
| RP_NEG_231_93193a14_001 | RP | NEG | 14.00 | 231.9319 | | | | 4.03E-01 | 0.18 | 0.58 | 5.74E-02 | -0.37 | 1.31 | 1.00E+00 | 0.00 | 0.59 | 1.41E-01 | 0.37 | 0.69 | 6234570 | 3421522 | 6804060 | 2999727 | 7373581 | 3298685 | 6234539 | 2576256 |
| HILIC_POS_171_18553a5_413 | HILIC | POS | 5.413 | 171.1855 | | | | 3.86E-01 | 0.16 | 0.97 | 5.77E-02 | -0.39 | 1.15 | 8.68E-01 | -0.04 | 0.35 | 9.24E-02 | 0.36 | 0.78 | 66984485 | 17648892 | 70274956 | 21163711 | 74286151 | 20741394 | 66263761 | 21021529 |
| HILIC_NEG_215_07324a0_699 | HILIC | NEG | 0.699 | 215.0732 | | | | 4.15E-07 | 0.62 | 1.11 | 5.77E-02 | 0.42 | 1.40 | 1.62E-04 | 0.83 | 1.27 | 3.23E-05 | 0.41 | 1.31 | 274919 | 514135 | 2149472 | 3364401 | 1508342 | 1831548 | 2790602 | 4321568 |
| RP_POS_579_26361a10_123 | RP | POS | 10.12 | 579.2636 | | | | 2.73E-21 | 1.34 | 2.21 | 5.77E-02 | 0.35 | 1.24 | 6.98E-13 | 1.51 | 2.27 | 4.49E-16 | 1.17 | 2.38 | 283675 | 106431 | 641349 | 243414 | 595102 | 170787 | 687595 | 293559 |
| RP_POS_828_55084a12_796 | RP | POS | 12.80 | 828.5508 | | | | 2.04E-04 | -0.86 | 1.40 | 5.79E-02 | -0.33 | 1.14 | 2.34E-05 | -1.03 | 1.84 | 4.91E-03 | -0.70 | 1.10 | 16989497 | 7477301 | 11121571 | 5939678 | 12247261 | 6674083 | 9995881 | 4914274 |

**Supplementary table 1: Metabolomics results** (in asceding order according to p-value from progressive ALD vs  non-progressive ALD comparison, with molecular features with ID_level of 1-2 first)

| | | | | | | | | all_ALD_vs_control | | | Prog_ALD_vs_non-prog_ALD | | | Prog_ALD_vs_control_ALD | | | Non-prog_ALD_vs_control | | | Healthy_control | | all_ALD | | Non-prog_ALD | | Progressive_ALD | |
|---|---|---|---|---|---|---|---|---|---|---|---|---|---|---|---|---|---|---|---|---|---|---|---|---|---|---|---|
| | | | | | | | | t-test | Cohen's | PLS-DA | t-test | Cohen's | PLS-DA | t-test | Cohen's | PLS-DA | t-test | Cohen's | PLS-DA | | | | | | | | |
| Feature_ID | RP_HILIC | POS_NEG | Average.Rt | Average.Mz | Metabolite.name | curated_MSMS | ID_level | p | d | VIP | p | d | VIP | p | d | VIP | p | d | VIP | Mean | SD | Mean | SD | Mean | SD | Mean | SD |
| RP_POS_380_33856a10_535 | RP | POS | 10.54 | 380.3386 | | | | 1.00E-01 | -0.33 | 0.91 | 5.81E-02 | 0.38 | 1.13 | 5.23E-01 | -0.14 | 0.53 | 2.14E-02 | -0.52 | 1.02 | 652608 | 167884 | 595118 | 173814 | 562239 | 172008 | 627997 | 171015 |
| RP_POS_789_53241a11_168 | RP | POS | 11.17 | 789.5324 | | | | 2.29E-06 | 0.56 | 1.24 | 5.82E-02 | -0.42 | 1.48 | 2.46E-03 | 0.35 | 1.28 | 1.87E-04 | 0.77 | 1.39 | 127732 | 73654 | 982438 | 1698910 | 1304689 | 2058161 | 660188 | 1176239 |
| RP_POS_347_22156a7_611 | RP | POS | 7.61 | 347.2216 | | | | 1.23E-01 | -0.40 | 1.43 | 5.84E-02 | 0.32 | 1.12 | 3.77E-01 | -0.24 | 0.70 | 3.99E-02 | -0.56 | 1.17 | 576093 | 555890 | 411448 | 349906 | 345328 | 334610 | 477568 | 355641 |
| RP_POS_242_92522a15_413 | RP | POS | 15.41 | 242.9252 | | | | 9.88E-01 | 0.00 | 0.38 | 5.85E-02 | 0.39 | 1.23 | 4.01E-01 | 0.19 | 0.51 | 3.49E-01 | -0.20 | 0.55 | 2965113 | 1150078 | 2961459 | 1273785 | 2720718 | 1136976 | 3202199 | 1366447 |
| RP_POS_593_47668a11_153 | RP | POS | 11.15 | 593.4767 | | | | 1.91E-01 | -0.36 | 0.65 | 5.85E-02 | -0.30 | 1.16 | 6.27E-02 | -0.51 | 0.89 | 4.87E-01 | -0.21 | 0.52 | 1429434 | 2270937 | 869713 | 1251108 | 1107151 | 1584112 | 632276 | 733500 |
| HILIC_POS_87_09225a0_898 | HILIC | POS | 0.898 | 87.0923 | | | | 4.94E-01 | -0.15 | 0.61 | 5.85E-02 | -0.36 | 1.08 | 1.84E-01 | -0.33 | 0.51 | 8.93E-01 | 0.03 | 0.18 | 6138735 | 1069744 | 5993781 | 923280 | 6168290 | 789556 | 5819271 | 1018205 |
| RP_POS_771_96576a10_502 | RP | POS | 10.50 | 771.9658 | | | | 2.54E-01 | -0.22 | 1.14 | 5.89E-02 | -0.39 | 1.44 | 5.31E-02 | -0.41 | 0.97 | 9.23E-01 | -0.02 | 1.24 | 4268334 | 511295 | 4143393 | 598054 | 4256201 | 612273 | 4030585 | 567263 |
| RP_POS_248_99841a0_523 | RP | POS | 0.52 | 248.9984 | | | | 1.21E-03 | 0.40 | 1.06 | 5.91E-02 | -0.43 | 1.41 | 1.87E-01 | 0.18 | 0.85 | 1.64E-03 | 0.61 | 1.26 | 426648 | 58635 | 530759 | 296268 | 586656 | 333246 | 474862 | 244768 |
| RP_POS_1004_71155a0_496 | RP | POS | 0.50 | 1004.7116 | | | | 9.10E-01 | -0.02 | 0.91 | 5.94E-02 | -0.40 | 1.52 | 2.66E-01 | -0.22 | 0.78 | 4.07E-01 | 0.18 | 1.10 | 298608 | 139349 | 295139 | 182517 | 329510 | 195792 | 260768 | 162971 |
| RP_POS_482_32413a10_167 | RP | POS | 10.17 | 482.3241 | | | | 8.03E-02 | -0.35 | 0.96 | 5.95E-02 | -0.38 | 1.26 | 1.13E-02 | -0.54 | 1.06 | 4.97E-01 | -0.16 | 0.53 | 2394071 | 1051536 | 2006880 | 1126259 | 2218987 | 1251193 | 1794773 | 951580 |
| RP_POS_796_54486a12_342 | RP | POS | 12.34 | 796.5449 | | | | 3.47E-02 | 0.36 | 0.75 | 5.95E-02 | 0.40 | 1.17 | 8.95E-03 | 0.56 | 1.03 | 3.90E-01 | 0.16 | 0.59 | 682197 | 177595 | 769739 | 259368 | 720906 | 225389 | 818572 | 283283 |
| RP_POS_1015_67017a10_371 | RP | POS | 10.37 | 1015.6702 | | | | 8.13E-01 | 0.05 | 0.98 | 5.97E-02 | -0.37 | 1.41 | 5.90E-01 | -0.13 | 0.65 | 3.06E-01 | 0.23 | 0.96 | 2504489 | 1952488 | 2596652 | 1748512 | 2925567 | 1532536 | 2267737 | 1899349 |
| HILIC_POS_175_10789a3_134 | HILIC | POS | 3.134 | 175.1079 | | | | 3.90E-01 | -0.14 | 0.69 | 5.99E-02 | 0.40 | 1.19 | 7.72E-01 | 0.06 | 0.10 | 8.02E-02 | -0.35 | 0.61 | 2140820 | 402394 | 2060402 | 599230 | 1947801 | 583087 | 2173003 | 599679 |
| RP_POS_621_89874a11_09 | RP | POS | 11.09 | 621.8987 | | | | 2.08E-05 | 0.57 | 1.27 | 6.00E-02 | -0.41 | 1.32 | 3.71E-03 | 0.37 | 1.15 | 2.96E-04 | 0.78 | 1.30 | 404739 | 111734 | 566807 | 308956 | 625035 | 381053 | 508579 | 201828 |
| RP_NEG_874_77637a0_496 | RP | NEG | 0.50 | 874.7764 | | | | 7.24E-01 | -0.07 | 0.80 | 6.02E-02 | -0.38 | 1.41 | 2.28E-01 | -0.26 | 0.67 | 6.00E-01 | 0.12 | 0.89 | 1287192 | 180456 | 1274008 | 190220 | 1309721 | 201667 | 1238296 | 172723 |
| RP_POS_768_54993a13_308 | RP | POS | 13.31 | 768.5499 | | | | 1.83E-01 | 0.24 | 0.84 | 6.02E-02 | 0.39 | 1.10 | 5.15E-02 | 0.44 | 0.94 | 8.17E-01 | 0.04 | 0.51 | 2716296 | 1067286 | 3029342 | 1361675 | 2773502 | 1119846 | 3285182 | 1535749 |
| RP_POS_266_6391a10_121 | RP | POS | 10.12 | 266.6391 | | | | 2.09E-20 | 1.32 | 2.16 | 6.04E-02 | 0.35 | 1.18 | 1.02E-12 | 1.49 | 2.24 | 5.53E-15 | 1.15 | 2.27 | 333956 | 117824 | 713647 | 264313 | 663965 | 192882 | 763330 | 314496 |
| RP_POS_387_25375a10_659 | RP | POS | 10.66 | 387.2538 | | | | 9.10E-01 | 0.02 | 0.47 | 6.07E-02 | 0.37 | 1.13 | 3.95E-01 | 0.21 | 0.67 | 4.87E-01 | -0.16 | 0.75 | 1961926 | 881891 | 1981802 | 797228 | 1832381 | 704625 | 2131222 | 861427 |
| RP_POS_333_14429a6_705 | RP | POS | 6.71 | 333.1443 | Unknown | 333.1439(100), 315.134(45) | 4 | 9.82E-01 | 0.00 | 0.65 | 6.07E-02 | 0.42 | 1.45 | 3.64E-01 | 0.21 | 0.57 | 1.14E-01 | -0.20 | 0.82 | 757982 | 510586 | 760984 | 941279 | 583417 | 428536 | 938550 | 1241815 |
| RP_POS_308_65976a10_932 | RP | POS | 10.93 | 308.6598 | | | | 4.11E-04 | -0.83 | 1.59 | 6.07E-02 | -0.33 | 1.46 | 8.01E-05 | -0.99 | 1.65 | 6.18E-03 | -0.66 | 1.32 | 1265296 | 337546 | 1015702 | 264375 | 1065224 | 261892 | 966179 | 260042 |
| RP_NEG_291_16168a9_607 | RP | NEG | 9.61 | 291.1617 | | | | 1.14E-01 | -0.39 | 0.86 | 6.08E-02 | -0.33 | 1.13 | 3.85E-02 | -0.56 | 0.83 | 3.75E-01 | -0.22 | 0.68 | 2932690 | 129843 | 2892881 | 90050 | 2909748 | 80586 | 2876014 | 96482 |
| HILIC_NEG_246_07503a1_544 | HILIC | NEG | 1.544 | 246.0750 | | | | 1.81E-05 | 0.56 | 1.03 | 6.09E-02 | 0.41 | 1.20 | 5.03E-04 | 0.77 | 1.14 | 1.34E-03 | 0.35 | 1.28 | 288046 | 292114 | 760051 | 925970 | 585844 | 517019 | 934259 | 1184523 |
| RP_POS_796_07617a13_294 | RP | POS | 13.29 | 796.0762 | | | | 7.62E-02 | -0.42 | 0.79 | 6.12E-02 | 0.34 | 1.13 | 3.16E-01 | -0.25 | 0.71 | 2.10E-02 | -0.59 | 1.01 | 3302706 | 1514969 | 2773633 | 1143081 | 2559928 | 1136224 | 2987339 | 1120591 |
| RP_NEG_401_1474a10_827 | RP | NEG | 10.83 | 401.1474 | | | | 1.74E-10 | 1.19 | 2.05 | 6.15E-02 | 0.34 | 1.36 | 2.10E-10 | 1.35 | 2.16 | 1.28E-07 | 1.02 | 1.90 | 753530 | 416005 | 1435167 | 519019 | 1338211 | 477514 | 1532123 | 545028 |
| RP_POS_367_19345a11_38 | RP | POS | 11.38 | 367.1935 | | | | 3.38E-10 | 0.90 | 1.79 | 6.16E-02 | 0.38 | 1.29 | 1.65E-07 | 1.10 | 1.81 | 1.98E-06 | 0.71 | 1.73 | 237632 | 140267 | 510584 | 310138 | 452595 | 238683 | 568573 | 361250 |
| HILIC_NEG_247_96234a0_699 | HILIC | NEG | 0.699 | 247.9623 | | | | 5.45E-08 | 0.69 | 1.22 | 6.17E-02 | 0.41 | 1.41 | 3.14E-05 | 0.89 | 1.43 | 3.28E-05 | 0.49 | 1.33 | 28917 | 32988 | 109921 | 127422 | 86051 | 81133 | 133791 | 158300 |
| HILIC_POS_304_10083a4_448 | HILIC | POS | 4.448 | 304.1008 | | | | 9.17E-06 | 0.66 | 0.99 | 6.18E-02 | 0.40 | 1.23 | 6.35E-06 | 0.85 | 1.44 | 1.03E-02 | 0.46 | 0.97 | 684790 | 80275 | 785754 | 163940 | 755175 | 160397 | 816332 | 163296 |
| RP_POS_568_42688a11_441 | RP | POS | 11.44 | 568.4269 | | | | 2.55E-07 | -1.32 | 2.21 | 6.19E-02 | -0.26 | 1.14 | 5.63E-08 | -1.45 | 2.10 | 2.53E-06 | -1.19 | 2.07 | 6517965 | 2644632 | 3433754 | 1654024 | 3742276 | 1508151 | 3125232 | 1748899 |
| RP_POS_384_20297a10_855 | RP | POS | 10.86 | 384.2030 | | | | 4.03E-01 | 0.16 | 0.73 | 6.20E-02 | -0.39 | 1.16 | 8.58E-01 | -0.04 | 0.71 | 8.96E-02 | 0.35 | 0.83 | 6568608 | 1807594 | 6895082 | 2190417 | 7303456 | 2002430 | 6486707 | 2311644 |
| RP_POS_191_03995a0_523 | RP | POS | 0.52 | 191.0400 | | | | 6.25E-03 | 0.35 | 0.98 | 6.20E-02 | -0.42 | 1.36 | 3.60E-01 | 0.14 | 0.74 | 3.08E-03 | 0.56 | 1.19 | 3008910 | 460252 | 3519155 | 1645068 | 3825905 | 1780730 | 3212405 | 1450756 |
| HILIC_NEG_429_58261a1_365 | HILIC | NEG | 1.365 | 429.5826 | | | | 1.76E-10 | -0.96 | 1.59 | 6.21E-02 | -0.37 | 1.16 | 1.62E-09 | -1.15 | 1.96 | 1.36E-05 | -0.78 | 1.57 | 218056 | 36103 | 150977 | 69974 | 164013 | 68762 | 137941 | 69409 |
| HILIC_POS_315_08023a7_326 | HILIC | POS | 7.326 | 315.0802 | | | | 2.15E-02 | 0.38 | 0.79 | 6.22E-02 | -0.40 | 1.22 | 3.39E-01 | 0.19 | 0.61 | 3.54E-03 | 0.58 | 1.01 | 1238545 | 164479 | 1328137 | 247507 | 1374226 | 244208 | 1282047 | 244533 |
| HILIC_POS_562_32739a1_203 | HILIC | POS | 1.203 | 562.3274 | | | | 1.45E-01 | -0.33 | 0.56 | 6.23E-02 | -0.35 | 1.27 | 3.86E-02 | -0.50 | 0.92 | 5.37E-01 | -0.15 | 0.24 | 565077 | 250696 | 492201 | 212621 | 531792 | 212801 | 452610 | 207014 |
| HILIC_POS_255_09749a4_568 | HILIC | POS | 4.568 | 255.0975 | | | | 6.63E-08 | 0.68 | 1.15 | 6.27E-02 | 0.41 | 1.11 | 4.20E-05 | 0.88 | 1.33 | 2.68E-05 | 0.48 | 1.37 | 363100 | 436712 | 1490574 | 1804726 | 1153722 | 1115836 | 1827426 | 2259177 |
| RP_NEG_782_82819a0_493 | RP | NEG | 0.49 | 782.8282 | | | | 5.87E-08 | -0.73 | 1.25 | 6.28E-02 | 0.40 | 1.36 | 3.10E-03 | -0.53 | 1.08 | 7.71E-08 | -0.93 | 1.62 | 2389300 | 259022 | 1898984 | 717329 | 1765655 | 658176 | 2032312 | 755083 |
| HILIC_NEG_345_71869a1_363 | HILIC | NEG | 1.363 | 345.7187 | | | | 1.81E-08 | 0.96 | 1.64 | 6.29E-02 | -0.36 | 0.99 | 1.29E-04 | 0.77 | 1.35 | 1.82E-09 | 1.14 | 2.19 | 354705 | 88411 | 482964 | 131821 | 507466 | 113374 | 458462 | 145015 |
| RP_POS_419_31543a11_966 | RP | POS | 11.97 | 419.3154 | | | | 7.73E-01 | 0.05 | 0.74 | 6.30E-02 | 0.40 | 1.34 | 2.05E-01 | 0.25 | 0.73 | 4.78E-01 | -0.15 | 0.81 | 68494194 | 3776336 | 68740134 | 5260673 | 67762927 | 5513907 | 69717340 | 4854086 |
| HILIC_NEG_132_92355a4_983 | HILIC | NEG | 4.983 | 132.9236 | | | | 5.06E-01 | 0.13 | 0.47 | 6.30E-02 | 0.38 | 1.02 | 1.67E-01 | 0.32 | 0.50 | 7.57E-01 | -0.06 | 0.24 | 5066364 | 3350846 | 5536757 | 3773199 | 4835419 | 3181574 | 6238094 | 4199807 |
| RP_POS_333_14444a6_414 | RP | POS | 6.41 | 333.1444 | | | | 9.75E-01 | 0.00 | 0.74 | 6.31E-02 | 0.41 | 1.47 | 3.83E-01 | 0.20 | 0.58 | 1.20E-01 | -0.21 | 0.89 | 826388 | 502492 | 822445 | 913832 | 651831 | 468238 | 993060 | 1186831 |
| RP_POS_411_77005a13_402 | RP | POS | 13.40 | 411.7701 | | | | 5.42E-02 | 0.45 | 0.89 | 6.32E-02 | -0.34 | 1.13 | 2.78E-01 | 0.28 | 0.51 | 1.04E-02 | 0.62 | 1.22 | 3793066 | 1237132 | 4265686 | 959989 | 4444105 | 757357 | 4087268 | 1106078 |
| RP_POS_855_60291a11_156 | RP | POS | 11.16 | 855.6029 | | | | 3.39E-07 | 0.61 | 1.28 | 6.32E-02 | -0.41 | 1.46 | 6.20E-04 | 0.40 | 1.31 | 8.45E-05 | 0.81 | 1.42 | 39569 | 15323 | 349284 | 565774 | 454565 | 684289 | 244003 | 394792 |
| RP_POS_829_52753a11_138 | RP | POS | 11.14 | 829.5275 | | | | 2.62E-06 | 0.56 | 1.22 | 6.32E-02 | -0.42 | 1.42 | 1.72E-03 | 0.35 | 1.28 | 2.51E-04 | 0.77 | 1.35 | 58270 | 36613 | 757252 | 1400493 | 1017990 | 1718321 | 496513 | 933498 |
| HILIC_POS_202_1185a4_014 | HILIC | POS | 4.014 | 202.1185 | | | | 1.58E-01 | 0.21 | 0.76 | 6.32E-02 | 0.41 | 1.09 | 4.58E-02 | 0.41 | 0.83 | 9.82E-01 | 0.00 | 0.25 | 1257320 | 662376 | 1510033 | 1336905 | 1261645 | 1063211 | 1758422 | 1534528 |
| HILIC_POS_117_52146a3_723 | HILIC | POS | 3.723 | 117.5215 | | | | 1.19E-03 | 0.54 | 1.12 | 6.32E-02 | 0.39 | 1.14 | 1.37E-03 | 0.74 | 1.26 | 2.64E-02 | 0.35 | 1.31 | 732024 | 697947 | 1276472 | 1047484 | 1081325 | 646916 | 1471620 | 1311723 |
| RP_POS_276_11893a0_604 | RP | POS | 0.60 | 276.1189 | | | | 2.25E-03 | 0.39 | 0.92 | 6.32E-02 | -0.42 | 1.23 | 2.14E-01 | 0.18 | 0.72 | 1.96E-03 | 0.60 | 1.04 | 443827 | 129872 | 603680 | 458602 | 688828 | 508976 | 518532 | 388679 |
| RP_POS_316_66666a10_932 | RP | POS | 10.93 | 316.6667 | | | | 4.67E-04 | -0.79 | 1.61 | 6.33E-02 | -0.34 | 1.58 | 9.31E-05 | -0.95 | 1.67 | 8.27E-03 | -0.62 | 1.27 | 1150347 | 280615 | 943017 | 238696 | 987287 | 233651 | 898747 | 237743 |
| RP_POS_833_55853a11_162 | RP | POS | 11.16 | 833.5585 | | | | 7.32E-07 | 0.59 | 1.27 | 6.35E-02 | -0.41 | 1.47 | 1.27E-03 | 0.38 | 1.30 | 1.10E-04 | 0.80 | 1.42 | 62701 | 46091 | 951111 | 1677753 | 1262885 | 2017441 | 639337 | 1191096 |
| HILIC_POS_247_144a1_433 | HILIC | POS | 1.433 | 247.1440 | | | | 1.50E-01 | -0.40 | 0.71 | 6.41E-02 | -0.28 | 1.04 | 5.41E-02 | -0.55 | 0.97 | 3.79E-01 | -0.26 | 0.37 | 39290360 | 50029043 | 25735571 | 25757970 | 30511711 | 31056829 | 20959430 | 18149532 |
| RP_NEG_132_86658a15_51 | RP | NEG | 15.51 | 132.8666 | | | | 9.90E-01 | 0.00 | 0.54 | 6.42E-02 | -0.38 | 1.12 | 3.71E-01 | -0.19 | 0.52 | 4.23E-01 | 0.19 | 0.56 | 2237273 | 85580 | 2237058 | 90766 | 2253847 | 98584 | 2220269 | 79694 |
| RP_NEG_610_24341a10_124 | RP | NEG | 10.12 | 610.2434 | | | | 6.13E-21 | 1.33 | 2.17 | 6.44E-02 | 0.34 | 1.19 | 4.97E-13 | 1.50 | 2.26 | 8.19E-15 | 1.16 | 2.23 | 827862 | 287582 | 1785537 | 662667 | 1662905 | 503322 | 1908169 | 776655 |
| HILIC_POS_421_37119a0_89 | HILIC | POS | 0.89 | 421.3712 | | | | 9.10E-03 | 0.44 | 0.79 | 6.45E-02 | 0.39 | 1.09 | 3.84E-03 | 0.64 | 1.10 | 1.56E-01 | 0.25 | 0.54 | 727625 | 138575 | 812614 | 202059 | 775244 | 158884 | 849983 | 233227 |
| RP_POS_805_57501a13_995 | RP | POS | 14.00 | 805.5750 | | | | 2.84E-01 | -0.24 | 0.49 | 6.47E-02 | 0.36 | 1.37 | 8.16E-01 | -0.06 | 0.52 | 7.22E-02 | -0.41 | 0.79 | 626393 | 372608 | 546833 | 324656 | 486856 | 268344 | 606811 | 365520 |
| RP_POS_196_00293a3_79 | RP | POS | 3.79 | 196.0029 | | | | 5.57E-01 | 0.12 | 0.99 | 6.47E-02 | -0.36 | 1.11 | 8.08E-01 | -0.06 | 0.70 | 1.86E-01 | 0.31 | 0.78 | 11040837 | 4848104 | 11614200 | 4530564 | 12450377 | 4322910 | 10778022 | 4621302 |
| RP_POS_257_17468a9_576 | RP | POS | 9.58 | 257.1747 | | | | 3.28E-04 | 0.43 | 0.85 | 6.47E-02 | -0.42 | 1.29 | 1.80E-02 | 0.22 | 0.96 | 3.37E-03 | 0.64 | 1.00 | 456572 | 267921 | 1414397 | 2535437 | 1884745 | 3263452 | 944048 | 1373490 |
| RP_POS_1040_75098a0_49 | RP | POS | 0.49 | 1040.7510 | | | | 2.65E-03 | -0.52 | 1.07 | 6.49E-02 | 0.39 | 1.32 | 1.20E-01 | -0.33 | 0.77 | 2.17E-04 | -0.71 | 1.31 | 535439 | 160107 | 422409 | 227897 | 380367 | 198964 | 464451 | 248476 |
| RP_POS_921_61121a11_148 | RP | POS | 11.15 | 921.6112 | | | | 8.61E-07 | 0.59 | 1.26 | 6.49E-02 | -0.41 | 1.46 | 1.01E-03 | 0.38 | 1.30 | 1.38E-04 | 0.79 | 1.40 | 90946 | 56769 | 812220 | 1370514 | 1065626 | 1665150 | 558814 | 944023 |
| RP_POS_277_21378a10_823 | RP | POS | 10.82 | 277.2138 | | | | 4.94E-10 | 0.85 | 1.67 | 6.50E-02 | 0.39 | 1.36 | 5.62E-08 | 1.05 | 1.87 | 5.19E-05 | 0.66 | 1.57 | 437605 | 304645 | 1083328 | 791206 | 937483 | 726797 | 1229172 | 832554 |
| RP_POS_332_26157a8_999 | RP | POS | 9.00 | 332.2616 | | | | 1.20E-01 | 0.25 | 0.62 | 6.51E-02 | 0.40 | 1.23 | 4.04E-02 | 0.45 | 0.90 | 7.37E-01 | 0.06 | 0.34 | 805494 | 341608 | 932760 | 538991 | 833221 | 396225 | 1032298 | 640106 |
| RP_POS_343_33771a11_161 | RP | POS | 11.16 | 343.3377 | | | | 1.43E-02 | -0.51 | 0.97 | 6.52E-02 | 0.36 | 1.28 | 1.43E-01 | -0.34 | 0.64 | 2.67E-03 | -0.69 | 1.24 | 19221840 | 5502939 | 16410671 | 5309801 | 15432748 | 5104689 | 17388593 | 5380876 |
| RP_NEG_588_3324a10_306 | RP | NEG | 10.31 | 588.3324 | | | | 1.40E-02 | 0.51 | 0.98 | 6.53E-02 | -0.36 | 1.45 | 1.63E-01 | 0.33 | 0.61 | 1.74E-03 | 0.69 | 1.21 | 48299798 | 13257064 | 55132319 | 13147158 | 57554772 | 11051833 | 52709866 | 14666422 |
| HILIC_NEG_171_02744a0_7 | HILIC | NEG | 0.7 | 171.0274 | | | | 9.31E-08 | 0.68 | 1.21 | 6.56E-02 | 0.40 | 1.37 | 4.25E-05 | 0.88 | 1.41 | 5.55E-05 | 0.47 | 1.29 | 54853 | 56394 | 195161 | 226541 | 153341 | 145845 | 236980 | 280801 |
| RP_POS_385_15353a10_283 | RP | POS | 10.28 | 385.1535 | | | | 1.38E-01 | -0.32 | 0.83 | 6.56E-02 | 0.36 | 1.19 | 5.64E-01 | -0.14 | 0.51 | 2.59E-02 | -0.50 | 1.00 | 948169 | 81212 | 923879 | 73001 | 910432 | 56522 | 937327 | 84871 |
| RP_NEG_199_80472a2_464 | RP | NEG | 2.46 | 199.8047 | | | | 6.92E-01 | 0.08 | 0.98 | 6.56E-02 | 0.36 | 1.18 | 2.64E-01 | 0.27 | 0.44 | 6.68E-01 | -0.10 | 0.82 | 4617424 | 2461103 | 4814041 | 2314670 | 4388331 | 2157365 | 5239750 | 2408454 |
| HILIC_POS_123_0555a4_568 | HILIC | POS | 4.568 | 123.0555 | | | | 2.00E-08 | 0.68 | 1.18 | 6.60E-02 | 0.41 | 1.13 | 4.88E-05 | 0.88 | 1.35 | 4.56E-06 | 0.47 | 1.48 | 219282 | 58190 | 607402 | 629904 | 491164 | 369171 | 723639 | 798621 |
| HILIC_POS_246_18526a0_741 | HILIC | POS | 0.741 | 246.1853 | | | | 1.10E-02 | 0.30 | 0.86 | 6.64E-02 | -0.42 | 1.41 | 1.78E-02 | 0.09 | 1.07 | 2.68E-02 | 0.51 | 0.92 | 185497 | 100460 | 438834 | 962413 | 617158 | 1332012 | 260510 | 179524 |
| RP_NEG_391_20853a11_162 | RP | NEG | 11.16 | 391.2085 | | | | 1.35E-01 | -0.30 | 0.64 | 6.65E-02 | -0.37 | 1.35 | 3.67E-02 | -0.48 | 0.77 | 5.91E-01 | -0.12 | 0.41 | 1367701 | 150895 | 1320609 | 158142 | 1349609 | 143191 | 1291610 | 168251 |
| RP_POS_223_09242a0_553 | RP | POS | 0.55 | 223.0924 | | | | 2.88E-01 | -0.18 | 0.88 | 6.65E-02 | -0.40 | 1.25 | 8.58E-02 | -0.37 | 0.74 | 8.98E-01 | 0.02 | 0.89 | 1987297 | 400517 | 1886897 | 616646 | 2000084 | 489084 | 1773711 | 709167 |
| RP_POS_582_86255a0_496 | RP | POS | 0.50 | 582.8626 | | | | 1.63E-08 | -0.73 | 1.28 | 6.68E-02 | 0.39 | 1.34 | 1.41E-03 | -0.54 | 1.11 | 2.52E-07 | -0.93 | 1.59 | 15110061 | 983619 | 12881136 | 3265426 | 12283114 | 3227675 | 13479158 | 3224264 |
| HILIC_NEG_130_05096a1_536 | HILIC | NEG | 1.536 | 130.0510 | | | | 6.98E-02 | 0.31 | 0.81 | 6.69E-02 | 0.39 | 1.22 | 2.19E-02 | 0.51 | 0.86 | 5.13E-01 | 0.12 | 0.46 | 545576 | 132043 | 600637 | 187247 | 566312 | 150154 | 634963 | 214214 |
| HILIC_POS_637_42957a1_23 | HILIC | POS | 1.23 | 637.4296 | | | | 5.77E-01 | -0.13 | 0.49 | 6.69E-02 | -0.35 | 1.20 | 2.25E-01 | -0.30 | 0.64 | 8.40E-01 | 0.05 | 0.15 | 2493702 | 1003738 | 2383535 | 834735 | 2536334 | 798251 | 2230736 | 850254 |
| HILIC_POS_154_15907a4_852 | HILIC | POS | 4.852 | 154.1591 | | | | 5.91E-02 | -0.45 | 0.83 | 6.69E-02 | -0.33 | 1.11 | 1.45E-02 | -0.62 | 1.03 | 2.62E-01 | -0.29 | 0.44 | 7754925 | 2025713 | 7002690 | 1486179 | 7274834 | 1604868 | 6730546 | 1317491 |
| RP_POS_154_05864a0_574 | RP | POS | 0.57 | 154.0586 | | | | 1.13E-02 | 0.41 | 0.77 | 6.71E-02 | -0.39 | 1.32 | 2.49E-01 | 0.22 | 0.65 | 2.19E-03 | 0.61 | 1.04 | 336581 | 208952 | 465090 | 330673 | 525572 | 331753 | 404608 | 321551 |
| RP_POS_888_5127a13_068 | RP | POS | 13.07 | 888.5127 | | | | 9.29E-01 | 0.02 | 0.35 | 6.72E-02 | -0.36 | 1.14 | 5.22E-01 | -0.16 | 0.49 | 3.77E-01 | 0.20 | 0.58 | 1638045 | 588214 | 1648476 | 528694 | 1745275 | 429669 | 1551677 | 600833 |
| RP_POS_899_63373a9_302 | RP | POS | 9.30 | 899.6337 | | | | 5.15E-02 | 0.23 | 0.87 | 6.72E-02 | 0.42 | 1.54 | 5.53E-02 | 0.44 | 0.91 | 5.88E-01 | 0.02 | 1.25 | 1101460 | 1802203 | 3550648 | 12024919 | 1328775 | 1911318 | 5772520 | 16685931 |
| RP_POS_362_11719a6_373 | RP | POS | 6.37 | 362.1172 | | | | 7.91E-06 | 0.64 | 1.15 | 6.72E-02 | 0.39 | 1.18 | 1.79E-04 | 0.84 | 1.22 | 5.48E-04 | 0.44 | 1.12 | 31454926 | 9721488 | 44462279 | 21871509 | 40444684 | 12770887 | 48479874 | 27757014 |
| RP_NEG_219_9514a15_478 | RP | NEG | 15.48 | 219.9514 | | | | 5.99E-01 | 0.11 | 0.40 | 6.78E-02 | -0.37 | 1.15 | 7.34E-01 | -0.08 | 0.56 | 2.01E-01 | 0.29 | 0.67 | 1042429 | 548039 | 1101503 | 556178 | 1202972 | 549826 | 1000033 | 549236 |
| RP_NEG_427_16379a10_973 | RP | NEG | 10.97 | 427.1638 | | | | 7.65E-01 | 0.05 | 0.90 | 6.79E-02 | -0.39 | 1.18 | 4.88E-01 | -0.15 | 0.82 | 1.86E-01 | 0.25 | 0.86 | 1654872 | 273028 | 1673737 | 404536 | 1747565 | 352907 | 1599909 | 441556 |
| RP_POS_877_58472a11_155 | RP | POS | 11.16 | 877.5847 | | | | 5.79E-07 | 0.59 | 1.28 | 6.80E-02 | -0.41 | 1.46 | 1.16E-03 | 0.39 | 1.30 | 9.86E-05 | 0.80 | 1.42 | 54643 | 44190 | 919476 | 1616512 | 1214963 | 1934561 | 623990 | 1165692 |
| RP_POS_777_9873a10_372 | RP | POS | 10.37 | 777.9873 | | | | 8.54E-01 | -0.04 | 0.97 | 6.80E-02 | -0.35 | 1.48 | 3.70E-01 | -0.22 | 0.72 | 5.64E-01 | 0.14 | 0.87 | 492728 | 272663 | 482745 | 239360 | 526389 | 227814 | 439101 | 244886 |
| RP_POS_265_17737a9_75 | RP | POS | 9.75 | 265.1774 | | | | 4.95E-03 | 0.37 | 0.84 | 6.81E-02 | 0.41 | 1.19 | 1.09E-02 | 0.57 | 0.89 | 9.60E-02 | 0.16 | 0.63 | 1356770 | 512405 | 1875736 | 1573087 | 1587061 | 723791 | 2164411 | 2075034 |
| RP_POS_770_56854a12_95 | RP | POS | 12.95 | 770.5685 | | | | 4.72E-03 | -0.64 | 1.04 | 6.81E-02 | 0.33 | 1.24 | 4.21E-02 | -0.48 | 0.86 | 1.25E-03 | -0.81 | 1.20 | 2560170 | 978750 | 1993060 | 803734 | 1846525 | 848628 | 2139596 | 735641 |
| RP_POS_400_07724a6_323 | RP | POS | 6.32 | 400.0772 | | | | 9.88E-01 | 0.00 | 0.69 | 6.81E-02 | 0.38 | 1.44 | 4.04E-01 | 0.19 | 0.64 | 2.98E-01 | -0.19 | 0.64 | 18856227 | 11492230 | 18894985 | 14509263 | 16243418 | 10147375 | 21546552 | 17550383 |
| RP_NEG_478_29346a10_124 | RP | NEG | 10.12 | 478.2935 | | | | 1.79E-19 | 1.18 | 2.01 | 6.82E-02 | 0.36 | 1.16 | 1.03E-10 | 1.36 | 2.07 | 3.22E-13 | 1.00 | 2.10 | 537717 | 206588 | 1360716 | 679316 | 1236642 | 490316 | 1484790 | 812731 |
| RP_POS_617_47461a11_361 | RP | POS | 11.36 | 617.4746 | | | | 4.27E-02 | -0.36 | 0.72 | 6.84E-02 | -0.38 | 1.15 | 2.85E-03 | -0.55 | 1.13 | 4.44E-01 | -0.17 | 0.75 | 4006667 | 2334138 | 2949523 | 3028869 | 3502143 | 3601674 | 2396904 | 2222616 |
| RP_POS_193_15468a0_542 | RP | POS | 0.54 | 193.1547 | | | | 1.26E-02 | 0.38 | 1.00 | 6.87E-02 | 0.40 | 1.08 | 8.52E-03 | 0.58 | 1.14 | 2.26E-01 | 0.18 | 0.82 | 5542981 | 2581613 | 7250441 | 4934873 | 6350255 | 3390308 | 8150627 | 6004582 |
| RP_POS_303_07074a7_589 | RP | POS | 7.59 | 303.0707 | | | | 2.60E-02 | 0.26 | 0.74 | 6.88E-02 | 0.42 | 1.33 | 4.13E-02 | 0.47 | 1.00 | 1.29E-01 | 0.05 | 0.56 | 75539 | 128502 | 549128 | 2083106 | 166553 | 386480 | 931702 | 2884295 |
| RP_NEG_816_8219a0_49 | RP | NEG | 0.49 | 816.8219 | | | | 1.03E-07 | -0.72 | 1.29 | 6.88E-02 | 0.39 | 1.38 | 2.03E-03 | -0.52 | 1.13 | 7.15E-07 | -0.91 | 1.56 | 2033513 | 290689 | 1495216 | 804093 | 1349035 | 805802 | 1641396 | 783198 |
| RP_POS_153_06593a1_744 | RP | POS | 1.74 | 153.0659 | | | | 1.24E-01 | -0.28 | 0.50 | 6.89E-02 | -0.38 | 1.14 | 2.92E-02 | -0.47 | 0.77 | 6.47E-01 | -0.09 | 0.70 | 1671775 | 772828 | 1411086 | 961058 | 1585753 | 904619 | 1236419 | 992732 |

**Supplementary table 1: Metabolomics results** (in asceding order according to p-value from progressive ALD vs  non-progressive ALD comparison, with molecular features with ID_level of 1-2 first)

| | | | | | | | | all_ALD_vs_control | | | Prog_ALD_vs_non-prog_ALD | | | Prog_ALD_vs_control_ALD | | | Non-prog_ALD_vs_control | | | Healthy_control | | all_ALD | | Non-prog_ALD | | Progressive_ALD | |
|---|---|---|---|---|---|---|---|---|---|---|---|---|---|---|---|---|---|---|---|---|---|---|---|---|---|---|---|
| | | | | | | | | t-test | Cohen's | PLS-DA | t-test | Cohen's | PLS-DA | t-test | Cohen's | PLS-DA | t-test | Cohen's | PLS-DA | | | | | | | | |
| Feature_ID | RP_HILIC | POS_NEG | Average.Rt | Average.Mz | Metabolite.name | curated_MSMS | ID_level | p | d | VIP | p | d | VIP | p | d | VIP | p | d | VIP | Mean | SD | Mean | SD | Mean | SD | Mean | SD |
| HILIC_POS_151_09929a0_512 | HILIC | POS | 0.512 | 151.0993 | | | | 5.54E-02 | 0.22 | 0.69 | 6.94E-02 | 0.42 | 1.05 | 6.03E-02 | 0.43 | 0.69 | 6.21E-01 | 0.01 | 0.18 | 434041 | 231395 | 1067541 | 3243123 | 473130 | 475488 | 1661952 | 4505905 |
| RP_POS_714_74377a0_501 | RP | POS | 0.50 | 714.7438 | | | | 2.76E-02 | 0.40 | 0.87 | 6.95E-02 | -0.38 | 1.31 | 2.84E-01 | 0.21 | 0.53 | 7.12E-03 | 0.59 | 1.13 | 285058 | 174296 | 370757 | 222957 | 411201 | 238126 | 330314 | 200977 |
| HILIC_POS_104_07101a0_683 | HILIC | POS | 0.683 | 104.0710 | | | | 2.24E-07 | 0.72 | 1.34 | 6.99E-02 | 0.39 | 1.13 | 1.59E-05 | 0.92 | 1.38 | 1.48E-04 | 0.53 | 1.39 | 234087 | 162928 | 502132 | 394401 | 430522 | 282727 | 573742 | 473159 |
| RP_POS_873_55383a11_134 | RP | POS | 11.13 | 873.5538 | | | | 1.78E-06 | 0.57 | 1.24 | 6.99E-02 | -0.41 | 1.42 | 1.53E-03 | 0.36 | 1.29 | 2.08E-04 | 0.77 | 1.37 | 53607 | 31677 | 898974 | 1663473 | 1201127 | 2023832 | 596820 | 1143915 |
| RP_POS_419_75659a13_296 | RP | POS | 13.30 | 419.7566 | | | | 1.74E-01 | -0.31 | 0.77 | 6.99E-02 | 0.34 | 1.11 | 5.72E-01 | -0.13 | 0.55 | 5.52E-02 | -0.48 | 1.00 | 6731631 | 1832634 | 6236600 | 1534011 | 5958769 | 1600380 | 6514430 | 1426765 |
| RP_POS_597_44849a11_609 | RP | POS | 11.61 | 597.4485 | | | | 1.12E-01 | -0.47 | 0.81 | 7.00E-02 | -0.26 | 1.11 | 4.50E-02 | -0.59 | 0.95 | 2.70E-01 | -0.34 | 0.61 | 3808511 | 5631000 | 2133268 | 2550529 | 2596174 | 3054486 | 1670363 | 1837380 |
| RP_POS_510_35541a10_666 | RP | POS | 10.67 | 510.3554 | | | | 4.76E-05 | -0.86 | 1.54 | 7.00E-02 | -0.33 | 1.37 | 7.24E-06 | -1.03 | 1.73 | 2.09E-03 | -0.70 | 1.21 | 21200865 | 7365824 | 14619029 | 7003516 | 15887131 | 7263367 | 13350928 | 6562102 |
| RP_POS_577_48181a11_152 | RP | POS | 11.15 | 577.4818 | | | | 1.29E-01 | -0.29 | 0.57 | 7.00E-02 | -0.37 | 1.13 | 1.01E-02 | -0.48 | 1.09 | 6.63E-01 | -0.11 | 0.60 | 1366979 | 1203665 | 978200 | 1352542 | 1224361 | 1743613 | 732038 | 729536 |
| HILIC_NEG_200_99734a6_093 | HILIC | NEG | 6.093 | 200.9973 | | | | 8.76E-01 | 0.03 | 0.80 | 7.00E-02 | -0.39 | 1.16 | 3.69E-01 | -0.17 | 0.53 | 2.75E-01 | 0.22 | 1.06 | 351188 | 140490 | 356347 | 217711 | 395763 | 226782 | 316931 | 202885 |
| RP_POS_814_57062a13_247 | RP | POS | 13.25 | 814.5706 | | | | 4.22E-01 | -0.20 | 0.72 | 7.01E-02 | -0.32 | 1.10 | 1.58E-01 | -0.36 | 0.61 | 8.92E-01 | -0.04 | 0.68 | 1127082 | 1036025 | 967630 | 717590 | 1097617 | 808938 | 837643 | 592887 |
| RP_POS_774_5036a12_16 | RP | POS | 12.16 | 774.5036 | | | | 1.49E-01 | 0.22 | 0.92 | 7.01E-02 | -0.40 | 1.10 | 9.19E-01 | 0.02 | 0.66 | 4.02E-02 | 0.42 | 0.70 | 361717 | 237106 | 452385 | 461172 | 535894 | 509784 | 368876 | 394447 |
| RP_POS_1061_65186a10_197 | RP | POS | 10.20 | 1061.6519 | | | | 3.39E-02 | -0.55 | 1.21 | 7.07E-02 | -0.30 | 1.23 | 8.83E-03 | -0.70 | 1.23 | 1.47E-01 | -0.40 | 0.99 | 380362 | 376747 | 224938 | 236959 | 267774 | 266400 | 182102 | 196803 |
| RP_NEG_269_05478a8_651 | RP | NEG | 8.65 | 269.0548 | | | | 8.13E-01 | -0.03 | 0.29 | 7.13E-02 | 0.41 | 1.18 | 4.62E-01 | 0.17 | 0.64 | 1.98E-02 | -0.24 | 1.14 | 26366034 | 20412521 | 24934952 | 48475435 | 16123875 | 16128824 | 33746029 | 65796004 |
| HILIC_POS_235_16545a3_699 | HILIC | POS | 3.699 | 235.1655 | | | | 1.00E-02 | 0.30 | 0.73 | 7.14E-02 | 0.41 | 1.24 | 2.59E-02 | 0.51 | 0.74 | 3.90E-02 | 0.10 | 0.67 | 7767394 | 5751322 | 25415164 | 66476325 | 13328002 | 17219053 | 37502326 | 91289272 |
| HILIC_POS_166_05316a6_092 | HILIC | POS | 6.092 | 166.0532 | | | | 9.78E-01 | 0.00 | 0.56 | 7.17E-02 | 0.39 | 1.05 | 4.00E-01 | 0.19 | 0.31 | 1.85E-01 | -0.20 | 0.85 | 683295 | 302053 | 681288 | 492054 | 592366 | 295795 | 770209 | 620922 |
| RP_POS_779_55688a13_72 | RP | POS | 13.72 | 779.5569 | | | | 9.37E-09 | 0.97 | 1.66 | 7.18E-02 | 0.35 | 1.09 | 4.14E-08 | 1.15 | 1.76 | 9.84E-06 | 0.80 | 1.57 | 579419 | 350619 | 1101334 | 525801 | 1006700 | 464603 | 1195968 | 569598 |
| RP_POS_221_07846a5_517 | RP | POS | 5.52 | 221.0785 | | | | 5.81E-03 | 0.39 | 0.80 | 7.19E-02 | 0.40 | 1.09 | 1.06E-02 | 0.59 | 0.91 | 8.72E-02 | 0.19 | 0.80 | 241884 | 291864 | 485013 | 695190 | 359147 | 308662 | 610880 | 921324 |
| RP_POS_917_57996a11_13 | RP | POS | 11.13 | 917.5800 | | | | 9.89E-07 | 0.58 | 1.26 | 7.21E-02 | -0.40 | 1.41 | 9.41E-04 | 0.38 | 1.32 | 1.66E-04 | 0.78 | 1.38 | 43484 | 45979 | 950251 | 1735806 | 1263099 | 2113637 | 637403 | 1191775 |
| RP_POS_624_84137a0_497 | RP | POS | 0.50 | 624.8414 | | | | 4.14E-07 | -0.70 | 1.30 | 7.22E-02 | 0.38 | 1.24 | 5.44E-03 | -0.51 | 1.03 | 2.40E-07 | -0.90 | 1.57 | 2571626 | 248628 | 2167554 | 614306 | 2057163 | 557700 | 2277945 | 653104 |
| RP_POS_808_03345a10_932 | RP | POS | 10.93 | 808.0335 | | | | 5.79E-02 | -0.45 | 1.23 | 7.24E-02 | -0.33 | 1.65 | 1.64E-02 | -0.61 | 1.17 | 2.51E-01 | -0.29 | 1.00 | 3699869 | 1436419 | 3161782 | 1085531 | 3356718 | 1074188 | 2966846 | 1072088 |
| RP_NEG_475_32828a9_718 | RP | NEG | 9.72 | 475.3283 | | | | 8.51E-01 | -0.04 | 0.31 | 7.24E-02 | 0.34 | 1.69 | 6.40E-01 | 0.13 | 0.38 | 3.18E-01 | -0.21 | 0.71 | 1140149 | 2573658 | 1045184 | 2097824 | 665583 | 782394 | 1424784 | 2825832 |
| RP_POS_377_26608a10_69 | RP | POS | 10.69 | 377.2661 | | | | 3.98E-03 | 0.37 | 0.87 | 7.24E-02 | 0.40 | 1.19 | 6.35E-03 | 0.57 | 0.95 | 1.87E-01 | 0.16 | 0.78 | 2192337 | 1685562 | 4198932 | 6160144 | 3089881 | 4266345 | 5307984 | 7480460 |
| RP_POS_368_21887a10_382 | RP | POS | 10.38 | 368.2189 | | | | 1.08E-01 | -0.33 | 0.92 | 7.27E-02 | 0.36 | 1.27 | 5.31E-01 | -0.15 | 0.61 | 1.80E-02 | -0.51 | 1.07 | 850444 | 191320 | 786757 | 193371 | 752029 | 156216 | 821484 | 220643 |
| RP_NEG_369_17465a8_165 | RP | NEG | 8.17 | 369.1747 | | | | 2.55E-12 | 0.88 | 1.48 | 7.28E-02 | 0.38 | 1.18 | 5.70E-08 | 1.07 | 1.69 | 2.84E-06 | 0.69 | 1.71 | 697945 | 416140 | 2276833 | 1890633 | 1937631 | 1610316 | 2616035 | 2096189 |
| RP_POS_524_37067a11_154 | RP | POS | 11.15 | 524.3707 | | | | 1.58E-01 | -0.33 | 1.03 | 7.28E-02 | -0.33 | 1.42 | 5.07E-02 | -0.49 | 0.97 | 5.08E-01 | -0.16 | 0.87 | 5812881 | 2008095 | 5254372 | 1575476 | 5536821 | 1513499 | 4971924 | 1600442 |
| RP_NEG_323_22241a11_162 | RP | NEG | 11.16 | 323.2224 | | | | 3.87E-01 | -0.18 | 0.53 | 7.33E-02 | -0.35 | 1.27 | 1.21E-01 | -0.36 | 0.67 | 9.69E-01 | -0.01 | 0.26 | 1518442 | 244751 | 1475814 | 226449 | 1516355 | 231726 | 1435273 | 215763 |
| RP_NEG_243_06261a0_708 | RP | NEG | 0.71 | 243.0626 | | | | 1.80E-01 | 0.20 | 0.79 | 7.36E-02 | 0.40 | 1.23 | 4.09E-02 | 0.39 | 0.76 | 9.95E-01 | 0.00 | 0.87 | 3244670 | 420429 | 3398698 | 865787 | 3243840 | 799687 | 3553556 | 908864 |
| RP_POS_188_12802a10_38 | RP | POS | 10.38 | 188.1280 | | | | 4.39E-01 | -0.14 | 0.78 | 7.36E-02 | 0.38 | 1.15 | 8.23E-01 | 0.05 | 0.58 | 7.70E-02 | -0.33 | 0.88 | 2914948 | 224925 | 2876442 | 293969 | 2823786 | 222896 | 2929098 | 345337 |
| RP_POS_203_05922a15_342 | RP | POS | 15.34 | 203.0592 | | | | 5.45E-01 | 0.13 | 0.42 | 7.37E-02 | 0.35 | 1.05 | 1.82E-01 | 0.30 | 0.55 | 8.40E-01 | -0.05 | 0.61 | 9071097 | 2420367 | 9366089 | 2269142 | 8960419 | 2395817 | 9771758 | 2080216 |
| HILIC_POS_583_255a1_293 | HILIC | POS | 1.293 | 583.2550 | | | | 3.53E-02 | 0.32 | 0.73 | 7.37E-02 | 0.39 | 1.23 | 1.96E-02 | 0.52 | 0.78 | 3.90E-01 | 0.13 | 0.42 | 571129 | 455421 | 820288 | 845547 | 668680 | 559293 | 971897 | 1041525 |
| RP_POS_766_57104a14_127 | RP | POS | 14.13 | 766.5710 | | | | 2.10E-01 | -0.23 | 0.50 | 7.38E-02 | 0.37 | 1.10 | 8.56E-01 | -0.04 | 0.31 | 3.73E-02 | -0.41 | 0.80 | 1618618 | 581686 | 1458414 | 739907 | 1326157 | 648467 | 1590671 | 806158 |
| RP_NEG_310_86508a15_501 | RP | NEG | 15.50 | 310.8651 | | | | 1.35E-01 | -0.32 | 0.70 | 7.42E-02 | -0.35 | 1.05 | 3.28E-02 | -0.49 | 0.88 | 5.48E-01 | -0.14 | 0.44 | 840550 | 174884 | 787383 | 164831 | 816804 | 171769 | 757963 | 153694 |
| RP_POS_784_77875a0_497 | RP | POS | 0.50 | 784.7788 | | | | 7.90E-01 | 0.05 | 0.83 | 7.43E-02 | -0.37 | 1.38 | 5.40E-01 | -0.13 | 0.57 | 3.00E-01 | 0.23 | 0.96 | 1552251 | 194225 | 1563069 | 212942 | 1601058 | 224430 | 1525081 | 195704 |
| RP_POS_511_83414a11_118 | RP | POS | 11.12 | 511.8341 | | | | 3.48E-07 | 0.61 | 1.29 | 7.43E-02 | -0.40 | 1.40 | 6.31E-04 | 0.41 | 1.31 | 8.81E-05 | 0.81 | 1.40 | 101588 | 40301 | 414576 | 569645 | 516421 | 685013 | 312730 | 406450 |
| RP_POS_569_40137a11_2 | RP | POS | 11.20 | 569.4014 | | | | 1.56E-05 | 0.51 | 1.15 | 7.44E-02 | -0.40 | 1.38 | 1.14E-03 | 0.31 | 1.35 | 9.96E-04 | 0.71 | 1.25 | 263157 | 106160 | 2124265 | 4092685 | 2857634 | 5237669 | 1390896 | 2304601 |
| RP_POS_1018_12756a10_5 | RP | POS | 10.50 | 1018.1276 | | | | 7.93E-01 | -0.05 | 0.59 | 7.45E-02 | -0.37 | 1.12 | 2.96E-01 | -0.23 | 0.56 | 5.34E-01 | 0.13 | 0.73 | 1724825 | 347487 | 1705633 | 388102 | 1774819 | 362454 | 1636447 | 403955 |
| RP_POS_1074_74927a0_492 | RP | POS | 0.49 | 1074.7493 | | | | 4.11E-07 | -0.69 | 1.27 | 7.48E-02 | 0.38 | 1.37 | 5.39E-03 | -0.50 | 1.11 | 5.10E-07 | -0.88 | 1.55 | 1218529 | 169062 | 930860 | 447839 | 851086 | 419213 | 1010633 | 465280 |
| RP_POS_184_09444a0_539 | RP | POS | 0.54 | 184.0944 | | | | 8.57E-02 | 0.26 | 0.73 | 7.48E-02 | -0.39 | 1.30 | 7.29E-01 | 0.07 | 0.68 | 1.14E-02 | 0.46 | 1.12 | 426939 | 103502 | 473030 | 192484 | 507313 | 177294 | 438748 | 202546 |
| RP_NEG_494_95419a10_967 | RP | NEG | 10.97 | 494.9542 | | | | 6.84E-01 | -0.10 | 0.75 | 7.49E-02 | 0.33 | 1.05 | 8.16E-01 | 0.06 | 0.40 | 2.69E-01 | -0.26 | 1.12 | 308371 | 443841 | 273639 | 322250 | 215977 | 176634 | 331300 | 414518 |
| HILIC_NEG_271_99979a0_699 | HILIC | NEG | 0.699 | 271.9998 | | | | 1.07E-09 | 0.79 | 1.38 | 7.49E-02 | 0.38 | 1.32 | 1.38E-06 | 0.98 | 1.70 | 1.90E-05 | 0.60 | 1.37 | 193404 | 290469 | 909793 | 960563 | 738583 | 755876 | 1081004 | 1110414 |
| RP_NEG_188_89304a15_406 | RP | NEG | 15.41 | 188.8930 | | | | 1.52E-01 | 0.31 | 1.03 | 7.52E-02 | -0.34 | 1.09 | 5.67E-01 | 0.14 | 0.48 | 3.86E-02 | 0.48 | 1.06 | 859625 | 263270 | 935649 | 238101 | 977999 | 218986 | 893298 | 250849 |
| RP_POS_1014_74402a0_493 | RP | POS | 0.49 | 1014.7440 | | | | 4.30E-02 | -0.36 | 0.84 | 7.53E-02 | 0.37 | 1.28 | 3.99E-01 | -0.17 | 0.48 | 8.12E-03 | -0.54 | 1.11 | 824083 | 111210 | 773303 | 148421 | 746916 | 144913 | 799690 | 148615 |
| RP_NEG_445_33279a11_155 | RP | NEG | 11.16 | 445.3328 | | | | 1.49E-01 | -0.34 | 1.01 | 7.53E-02 | -0.33 | 1.72 | 4.67E-02 | -0.50 | 1.35 | 4.83E-01 | -0.18 | 0.79 | 1381978 | 850694 | 1141405 | 651651 | 1257239 | 655588 | 1025572 | 633077 |
| HILIC_POS_205_41711a2_972 | HILIC | POS | 2.972 | 205.4171 | | | | 6.78E-05 | 0.62 | 1.21 | 7.55E-02 | 0.38 | 1.11 | 1.31E-04 | 0.80 | 1.27 | 8.95E-03 | 0.43 | 0.98 | 267928 | 347371 | 634486 | 630870 | 522294 | 511830 | 746678 | 718650 |
| RP_POS_166_98495a11_552 | RP | POS | 11.55 | 166.9850 | | | | 2.23E-01 | -0.22 | 0.52 | 7.56E-02 | 0.37 | 1.26 | 8.68E-01 | -0.04 | 0.35 | 3.61E-02 | -0.41 | 0.91 | 13190882 | 2689657 | 12475568 | 3349355 | 11880028 | 2733593 | 13071107 | 3803809 |
| RP_NEG_394_97168a8_709 | RP | NEG | 8.71 | 394.9717 | | | | 4.08E-01 | -0.19 | 0.36 | 7.59E-02 | -0.34 | 1.35 | 1.60E-01 | -0.36 | 0.65 | 9.40E-01 | -0.02 | 0.36 | 1882916 | 323664 | 1830095 | 269364 | 1877917 | 234706 | 1782273 | 294688 |
| HILIC_NEG_144_06659a0_747 | HILIC | NEG | 0.747 | 144.0666 | | | | 3.47E-03 | 0.35 | 0.68 | 7.59E-02 | 0.41 | 1.25 | 1.59E-02 | 0.56 | 0.75 | 4.80E-03 | 0.15 | 1.08 | 89592 | 34614 | 149069 | 189731 | 115089 | 44499 | 183049 | 261522 |
| HILIC_POS_104_07106a4_678 | HILIC | POS | 4.678 | 104.0711 | | | | 1.11E-01 | 0.26 | 0.56 | 7.60E-02 | 0.38 | 1.06 | 4.86E-02 | 0.45 | 0.69 | 6.51E-01 | 0.07 | 0.44 | 5133754 | 2460024 | 6071361 | 3860353 | 5384026 | 2391313 | 6758696 | 4840068 |
| RP_POS_381_20718a10_864 | RP | POS | 10.86 | 381.2072 | | | | 8.31E-02 | -0.36 | 0.68 | 7.61E-02 | -0.35 | 1.25 | 1.52E-02 | -0.53 | 0.98 | 4.45E-01 | -0.18 | 0.44 | 740044 | 248877 | 650728 | 249103 | 694916 | 275053 | 606540 | 213850 |
| RP_POS_200_20068a9_286 | RP | POS | 9.29 | 200.2007 | | | | 5.88E-01 | -0.11 | 0.86 | 7.62E-02 | 0.36 | 1.10 | 7.56E-01 | 0.07 | 0.86 | 1.76E-01 | -0.29 | 0.93 | 1259499 | 44389 | 1254513 | 46921 | 1246191 | 40635 | 1262834 | 51532 |
| RP_NEG_154_9733a15_453 | RP | NEG | 15.45 | 154.9733 | | | | 8.42E-01 | 0.04 | 0.62 | 7.62E-02 | -0.34 | 1.07 | 5.94E-01 | -0.12 | 0.32 | 4.01E-01 | 0.21 | 0.58 | 2888700 | 110100 | 2893013 | 91703 | 2909281 | 103046 | 2876744 | 76368 |
| RP_NEG_331_17712a8_142 | RP | NEG | 8.14 | 331.1771 | | | | 1.80E-01 | -0.39 | 0.72 | 7.63E-02 | 0.25 | 1.13 | 3.93E-01 | -0.27 | 0.39 | 7.39E-02 | -0.52 | 1.04 | 1956520 | 3881425 | 988065 | 1747795 | 676153 | 698192 | 1299976 | 2342198 |
| RP_NEG_271_22839a10_135 | RP | NEG | 10.14 | 271.2284 | | | | 6.77E-01 | 0.07 | 0.43 | 7.63E-02 | -0.37 | 1.18 | 5.95E-01 | -0.11 | 0.48 | 1.90E-01 | 0.26 | 0.81 | 3109776 | 315433 | 3138853 | 415331 | 3212447 | 381869 | 3065258 | 437770 |
| RP_POS_1082_73108a0_493 | RP | POS | 0.49 | 1082.7311 | | | | 1.35E-03 | -0.53 | 0.92 | 7.63E-02 | 0.37 | 1.22 | 1.01E-01 | -0.34 | 0.78 | 8.97E-05 | -0.71 | 1.27 | 531181 | 113552 | 442039 | 178597 | 410373 | 150254 | 473704 | 199556 |
| RP_POS_385_1983a7_048 | RP | POS | 7.05 | 385.1983 | | | | 7.25E-02 | -0.46 | 2.02 | 7.64E-02 | 0.30 | 1.05 | 2.50E-01 | -0.30 | 1.22 | 2.42E-02 | -0.61 | 1.52 | 2494138 | 1227796 | 2067108 | 803740 | 1924727 | 797038 | 2209488 | 792805 |
| RP_POS_340_19611a0_707 | RP | POS | 0.71 | 340.1961 | | | | 2.44E-14 | 0.95 | 1.64 | 7.64E-02 | 0.37 | 1.09 | 7.64E-08 | 1.13 | 1.72 | 2.59E-09 | 0.76 | 1.74 | 103349 | 107706 | 741116 | 703310 | 616226 | 494573 | 866005 | 850265 |
| RP_POS_489_82062a11_124 | RP | POS | 11.12 | 489.8206 | | | | 7.38E-07 | 0.59 | 1.28 | 7.65E-02 | -0.39 | 1.39 | 6.14E-04 | 0.39 | 1.32 | 1.54E-04 | 0.79 | 1.38 | 62814 | 30260 | 263298 | 376272 | 330113 | 459742 | 196483 | 256080 |
| RP_NEG_748_83472a0_492 | RP | NEG | 0.49 | 748.8347 | | | | 2.06E-09 | -0.78 | 1.38 | 7.65E-02 | 0.38 | 1.38 | 4.21E-04 | -0.59 | 1.28 | 9.22E-08 | -0.97 | 1.64 | 2482221 | 297678 | 1771182 | 969435 | 1599534 | 963694 | 1942831 | 953915 |
| RP_POS_548_5036a13_933 | RP | POS | 13.93 | 548.5036 | | | | 4.67E-01 | 0.16 | 0.59 | 7.66E-02 | -0.34 | 1.10 | 9.89E-01 | 0.00 | 0.42 | 1.87E-01 | 0.33 | 0.61 | 3147266 | 1018252 | 3293091 | 840484 | 3441922 | 902634 | 3144259 | 753186 |
| RP_POS_409_75241a12_694 | RP | POS | 12.69 | 409.7524 | | | | 4.00E-03 | 0.60 | 1.22 | 7.66E-02 | -0.34 | 1.08 | 5.97E-02 | 0.43 | 0.85 | 7.51E-04 | 0.77 | 1.33 | 1556869 | 627980 | 1940164 | 621431 | 2050159 | 594918 | 1830170 | 633731 |
| RP_POS_497_39594a13_803 | RP | POS | 13.80 | 497.3959 | | | | 3.69E-04 | -0.75 | 1.25 | 7.68E-02 | -0.33 | 1.07 | 6.60E-05 | -0.92 | 1.49 | 9.80E-03 | -0.59 | 1.00 | 6363907 | 971711 | 5617965 | 931404 | 5782717 | 953943 | 5453214 | 887316 |
| RP_POS_961_6059a11_125 | RP | POS | 11.13 | 961.6059 | | | | 9.36E-07 | 0.58 | 1.26 | 7.69E-02 | -0.39 | 1.41 | 1.18E-03 | 0.39 | 1.31 | 1.48E-04 | 0.78 | 1.39 | 53672 | 51956 | 832038 | 1485415 | 1095343 | 1789643 | 568733 | 1055290 |
| RP_POS_502_3266a10_7 | RP | POS | 10.70 | 502.3266 | | | | 7.64E-02 | -0.40 | 0.99 | 7.71E-02 | -0.33 | 1.62 | 2.18E-02 | -0.57 | 1.02 | 3.27E-01 | -0.24 | 0.86 | 5158175 | 1845957 | 4505505 | 1519401 | 4774009 | 1487238 | 4237002 | 1518310 |
| RP_POS_594_16241a9_667 | RP | POS | 9.67 | 594.1624 | | | | 6.46E-01 | -0.09 | 0.63 | 7.73E-02 | 0.37 | 1.07 | 6.42E-01 | 0.10 | 0.54 | 2.02E-01 | -0.27 | 0.73 | 1356488 | 65705 | 1349998 | 79608 | 1335939 | 77546 | 1364057 | 79922 |
| RP_POS_745_50641a11_176 | RP | POS | 11.18 | 745.5064 | | | | 1.85E-05 | 0.51 | 1.19 | 7.74E-02 | -0.40 | 1.42 | 5.80E-03 | 0.31 | 1.25 | 6.90E-04 | 0.71 | 1.34 | 274842 | 116791 | 1011960 | 1627328 | 1300105 | 1996944 | 723816 | 1092342 |
| RP_POS_393_29688a11_173 | RP | POS | 11.17 | 393.2969 | | | | 2.46E-05 | 0.53 | 1.11 | 7.75E-02 | -0.39 | 1.29 | 1.97E-03 | 0.34 | 1.21 | 7.83E-04 | 0.73 | 1.18 | 1771621 | 251853 | 2240775 | 974408 | 2413461 | 1237150 | 2068088 | 571769 |
| RP_POS_546_35242a11_158 | RP | POS | 11.16 | 546.3524 | | | | 1.29E-01 | -0.36 | 1.02 | 7.76E-02 | -0.32 | 1.49 | 4.10E-02 | -0.52 | 0.92 | 4.33E-01 | -0.20 | 0.89 | 1675107 | 599231 | 1496281 | 459583 | 1577361 | 452101 | 1415202 | 457090 |
| HILIC_NEG_161_04555a4_993 | HILIC | NEG | 4.993 | 161.0456 | | | | 8.01E-01 | -0.04 | 0.91 | 7.78E-02 | 0.37 | 1.01 | 5.19E-01 | 0.14 | 0.23 | 2.15E-01 | -0.23 | 0.94 | 1870955 | 965005 | 1816641 | 1299992 | 1587167 | 1055206 | 2046115 | 1481054 |
| RP_POS_690_78375a0_499 | RP | POS | 0.50 | 690.7838 | | | | 4.22E-01 | 0.14 | 0.82 | 7.78E-02 | -0.37 | 1.46 | 8.35E-01 | -0.04 | 0.56 | 1.19E-01 | 0.33 | 1.04 | 730643 | 210802 | 768024 | 274313 | 816392 | 280704 | 719655 | 261651 |
| RP_POS_1037_65222a10_37 | RP | POS | 10.37 | 1037.6522 | | | | 5.96E-01 | -0.12 | 0.98 | 7.80E-02 | -0.34 | 1.44 | 2.43E-01 | -0.29 | 0.76 | 8.22E-01 | 0.05 | 0.87 | 507376 | 358330 | 469695 | 310034 | 524333 | 285470 | 415057 | 326525 |
| RP_POS_539_31317a10_325 | RP | POS | 10.33 | 539.3132 | | | | 3.23E-03 | -0.74 | 1.36 | 7.81E-02 | -0.29 | 1.05 | 9.01E-04 | -0.89 | 1.51 | 2.14E-02 | -0.59 | 1.14 | 700776 | 310247 | 517079 | 207949 | 553699 | 201983 | 480459 | 209366 |
| RP_POS_441_29688a10_579 | RP | POS | 10.58 | 441.2969 | | | | 6.84E-01 | 0.09 | 0.84 | 7.83E-02 | 0.35 | 1.08 | 2.59E-01 | 0.26 | 0.64 | 7.14E-01 | -0.09 | 0.78 | 1807379 | 408965 | 1840857 | 380929 | 1773813 | 394571 | 1907902 | 358212 |
| RP_POS_353_26578a10_863 | RP | POS | 10.86 | 353.2658 | | | | 5.90E-01 | 0.10 | 0.43 | 7.84E-02 | 0.37 | 1.05 | 2.20E-01 | 0.28 | 0.62 | 6.31E-01 | -0.09 | 0.43 | 5804738 | 4206849 | 6301571 | 5369217 | 5354988 | 3980055 | 7248155 | 6369920 |
| RP_POS_934_81274a0_487 | RP | POS | 0.49 | 934.8127 | | | | 1.16E-04 | 0.49 | 0.91 | 7.85E-02 | -0.39 | 1.17 | 8.46E-02 | 0.30 | 0.58 | 6.56E-05 | 0.69 | 1.33 | 382465 | 31773 | 433328 | 114932 | 453543 | 110301 | 413113 | 116997 |
| HILIC_NEG_211_97729a1_213 | HILIC | NEG | 1.213 | 211.9773 | | | | 9.55E-04 | -0.62 | 1.27 | 7.87E-02 | -0.35 | 0.97 | 4.13E-04 | -0.80 | 1.37 | 2.40E-02 | -0.44 | 1.27 | 416779 | 93744 | 347156 | 113319 | 367087 | 97063 | 327225 | 125349 |
| HILIC_POS_188_17578a6_233 | HILIC | POS | 6.233 | 188.1758 | | | | 1.59E-05 | 0.57 | 0.97 | 7.89E-02 | 0.39 | 1.45 | 1.22E-04 | 0.76 | 1.19 | 1.13E-02 | 0.38 | 1.06 | 1028036 | 534047 | 1873932 | 1633152 | 1586786 | 1363092 | 2161077 | 1833716 |
| HILIC_POS_227_17538a1_111 | HILIC | POS | 1.111 | 227.1754 | | | | 6.57E-02 | 0.23 | 0.79 | 7.89E-02 | 0.40 | 1.43 | 6.04E-02 | 0.43 | 0.61 | 6.35E-01 | 0.03 | 0.26 | 662032 | 467339 | 1011206 | 1689633 | 711764 | 450347 | 1310648 | 2319952 |
| RP_NEG_197_08168a5_516 | RP | NEG | 5.52 | 197.0817 | | | | 6.85E-03 | 0.38 | 0.81 | 7.92E-02 | 0.39 | 1.06 | 1.21E-02 | 0.58 | 0.91 | 9.02E-02 | 0.19 | 0.82 | 378535 | 494352 | 774153 | 1141270 | 572542 | 504816 | 975765 | 1514524 |
| RP_POS_253_21368a10_464 | RP | POS | 10.46 | 253.2137 | | | | 6.30E-02 | -0.34 | 0.73 | 7.94E-02 | 0.37 | 1.06 | 4.63E-01 | -0.16 | 0.53 | 1.11E-02 | -0.52 | 0.91 | 716152 | 46447 | 697056 | 58736 | 686752 | 54819 | 707359 | 61224 |
| HILIC_POS_188_07068a1_433 | HILIC | POS | 1.433 | 188.0707 | | | | 1.21E-01 | -0.43 | 0.86 | 7.96E-02 | -0.27 | 1.04 | 4.47E-02 | -0.57 | 1.06 | 3.15E-01 | -0.30 | 0.44 | 3684979 | 4586897 | 2341858 | 2408648 | 2764854 | 2848096 | 1918863 | 1801323 |
| HILIC_POS_171_18535a6_125 | HILIC | POS | 6.125 | 171.1854 | | | | 9.78E-01 | -0.01 | 0.87 | 7.96E-02 | -0.35 | 1.05 | 4.36E-01 | -0.18 | 0.76 | 4.58E-01 | 0.17 | 0.73 | 18762610 | 5969663 | 18728678 | 5823113 | 19749352 | 5633550 | 17708003 | 5886114 |
| HILIC_NEG_202_96533a0_7 | HILIC | NEG | 0.7 | 202.9653 | | | | 3.04E-09 | 0.83 | 1.43 | 7.98E-02 | 0.37 | 1.38 | 1.01E-06 | 1.02 | 1.69 | 1.21E-05 | 0.65 | 1.45 | 2541706 | 2447602 | 7210167 | 5871936 | 6180198 | 4574351 | 8240135 | 6824462 |
| RP_POS_525_37543a11_202 | RP | POS | 11.20 | 525.3754 | | | | 9.34E-05 | 0.47 | 1.15 | 7.99E-02 | -0.39 | 1.40 | 1.88E-02 | 0.28 | 1.20 | 1.21E-03 | 0.67 | 1.28 | 1144151 | 862475 | 3321345 | 5154243 | 4226239 | 6271432 | 2416452 | 3559869 |
| RP_NEG_528_2868a10_124 | RP | NEG | 10.12 | 528.2868 | | | | 4.96E-21 | 1.30 | 2.14 | 8.02E-02 | 0.33 | 1.11 | 1.49E-12 | 1.47 | 2.22 | 9.58E-15 | 1.14 | 2.22 | 1039824 | 371518 | 2326576 | 915975 | 2166090 | 693400 | 2487061 | 1077858 |
| RP_POS_265_0835a7_928 | RP | POS | 7.93 | 265.0835 | | | | 9.31E-01 | -0.02 | 0.43 | 8.03E-02 | 0.35 | 1.21 | 5.72E-01 | 0.15 | 0.23 | 3.24E-01 | -0.19 | 0.77 | 1942467 | 10247731 | 1765838 | 9286729 | 125212 | 42746 | 3406465 | 12990448 |
| RP_POS_657_45441a11_188 | RP | POS | 11.19 | 657.4544 | | | | 2.27E-04 | 0.44 | 1.11 | 8.04E-02 | -0.40 | 1.38 | 1.32E-02 | 0.24 | 1.24 | 3.21E-03 | 0.63 | 1.23 | 580729 | 158004 | 1418666 | 2173745 | 1800309 | 2776456 | 1037024 | 1241295 |
| RP_POS_470_28525a11_129 | RP | POS | 11.13 | 470.2853 | | | | 4.59E-07 | 0.60 | 1.28 | 8.06E-02 | -0.39 | 1.41 | 6.52E-04 | 0.41 | 1.33 | 1.16E-04 | 0.79 | 1.40 | 21906 | 12754 | 452492 | 797201 | 592110 | 961911 | 312873 | 564777 |
| RP_POS_200_20108a9_477 | RP | POS | 9.48 | 200.2011 | | | | 1.37E-02 | 0.37 | 0.92 | 8.06E-02 | 0.38 | 1.41 | 1.50E-02 | 0.56 | 0.92 | 1.58E-01 | 0.18 | 0.59 | 10800726 | 703654 | 11263016 | 1360422 | 11024025 | 669107 | 11502008 | 1781852 |

**Supplementary table 1: Metabolomics results** (in asceding order according to p-value from progressive ALD vs non-progressive ALD comparison, with molecular features with ID_level of 1-2 first)

| | | | | | | | | all_ALD_vs_control | | | Prog_ALD_vs_non-prog_ALD | | | Prog_ALD_vs_control_ALD | | | Non-prog_ALD_vs_control | | | Healthy_control | | all_ALD | | Non-prog_ALD | | Progressive_ALD | |
|---|---|---|---|---|---|---|---|---|---|---|---|---|---|---|---|---|---|---|---|---|---|---|---|---|---|---|---|
| | | | | | | | | t-test | Cohen's | PLS-DA | t-test | Cohen's | PLS-DA | t-test | Cohen's | PLS-DA | t-test | Cohen's | PLS-DA | | | | | | | | |
| Feature_ID | RP_HILIC | POS_NEG | Average.Rt | Average.Mz | Metabolite.name | curated_MSMS | ID_level | p | d | VIP | p | d | VIP | p | d | VIP | p | d | VIP | Mean | SD | Mean | SD | Mean | SD | Mean | SD |
| RP_NEG_529_46448a15_056 | RP | NEG | 15.06 | 529.4645 | | | | 5.08E-01 | -0.11 | 0.57 | 8.07E-02 | 0.38 | 1.34 | 5.27E-01 | 0.08 | 0.34 | 2.14E-01 | -0.30 | 0.68 | 15922791 | 2111703 | 15585269 | 3441701 | 14979839 | 4615663 | 16190699 | 1371115 |
| RP_POS_172_9769a0_837 | RP | POS | 0.84 | 172.9769 | | | | 5.22E-02 | -0.44 | 0.82 | 8.07E-02 | -0.33 | 1.12 | 1.30E-02 | -0.60 | 1.00 | 2.59E-01 | -0.28 | 0.55 | 32021417 | 14721781 | 26294979 | 12141811 | 28415873 | 12701466 | 24174085 | 11284882 |
| RP_POS_874_55469a13_296 | RP | POS | 13.30 | 874.5547 | | | | 7.42E-01 | 0.07 | 0.30 | 8.07E-02 | 0.33 | 1.07 | 3.32E-01 | 0.24 | 0.66 | 7.09E-01 | -0.09 | 0.25 | 2992721 | 1097026 | 3063520 | 898780 | 2906566 | 864899 | 3220474 | 913112 |
| RP_POS_310_87701a15_419 | RP | POS | 15.42 | 310.8770 | | | | 4.17E-01 | -0.17 | 0.40 | 8.12E-02 | -0.34 | 1.08 | 1.61E-01 | -0.34 | 0.57 | 9.92E-01 | 0.00 | 0.51 | 1681058 | 401613 | 1615418 | 371411 | 1680229 | 312862 | 1550607 | 415033 |
| RP_POS_570_79486a10_303 | RP | POS | 10.30 | 570.7949 | | | | 3.77E-01 | 0.18 | 0.56 | 8.15E-02 | -0.35 | 1.35 | 9.97E-01 | 0.00 | 0.32 | 9.26E-02 | 0.35 | 0.74 | 320093 | 112492 | 340638 | 117191 | 361077 | 94574 | 320200 | 133982 |
| HILIC_POS_118_08655a0_627 | HILIC | POS | 0.627 | 118.0866 | | | | 3.42E-07 | 0.62 | 1.20 | 8.15E-02 | 0.39 | 0.99 | 1.28E-07 | 0.81 | 1.70 | 2.07E-02 | 0.42 | 0.73 | 765584 | 156339 | 1417318 | 1167673 | 1213737 | 1312974 | 1620900 | 972761 |
| RP_POS_481_81656a14_965 | RP | POS | 14.97 | 481.8166 | | | | 5.48E-01 | -0.11 | 0.42 | 8.15E-02 | -0.36 | 1.21 | 8.17E-02 | -0.30 | 0.77 | 7.94E-01 | 0.07 | 0.43 | 122924 | 150593 | 103720 | 173184 | 134135 | 236146 | 73306 | 54266 |
| RP_POS_701_48053a11_182 | RP | POS | 11.18 | 701.4805 | | | | 1.70E-04 | 0.44 | 1.13 | 8.18E-02 | -0.39 | 1.39 | 1.57E-02 | 0.25 | 1.23 | 2.49E-03 | 0.64 | 1.26 | 434478 | 133864 | 1118914 | 1737521 | 1422416 | 2185810 | 815411 | 1064808 |
| RP_POS_785_52875a13_157 | RP | POS | 13.16 | 785.5288 | | | | 5.12E-01 | -0.15 | 0.36 | 8.20E-02 | 0.33 | 1.14 | 9.52E-01 | 0.02 | 0.21 | 1.99E-01 | -0.31 | 0.65 | 5388122 | 1177010 | 5236912 | 956984 | 5070483 | 907129 | 5403341 | 985350 |
| HILIC_POS_112_03961a1_809 | HILIC | POS | 1.809 | 112.0396 | | | | 2.32E-02 | 0.36 | 0.64 | 8.24E-02 | -0.38 | 1.30 | 3.31E-01 | 0.17 | 0.36 | 7.61E-03 | 0.55 | 0.87 | 379487 | 391457 | 601420 | 666622 | 717230 | 721316 | 485610 | 591753 |
| RP_POS_797_5899a13_998 | RP | POS | 14.00 | 797.5899 | | | | 6.52E-02 | -0.46 | 0.90 | 8.26E-02 | 0.30 | 1.12 | 2.33E-01 | -0.31 | 0.74 | 2.12E-02 | -0.61 | 0.95 | 11950780 | 4856658 | 10210969 | 3239191 | 9648714 | 3132394 | 10773223 | 3277467 |
| RP_POS_957_47552a0_488 | RP | POS | 0.49 | 957.4755 | | | | 4.50E-07 | 0.61 | 1.20 | 8.30E-02 | -0.38 | 1.34 | 4.67E-03 | 0.42 | 1.09 | 2.03E-05 | 0.80 | 1.45 | 218229 | 30449 | 339987 | 220400 | 378200 | 237989 | 301773 | 196278 |
| RP_POS_613_42731a11_194 | RP | POS | 11.19 | 613.4273 | | | | 4.78E-05 | 0.48 | 1.13 | 8.33E-02 | -0.39 | 1.36 | 3.01E-03 | 0.28 | 1.30 | 1.70E-03 | 0.68 | 1.24 | 482047 | 159156 | 1803892 | 3097140 | 2342728 | 3959021 | 1265056 | 1764633 |
| RP_POS_583_2547a9_295 | RP | POS | 9.30 | 583.2547 | | | | 2.25E-01 | -0.21 | 0.74 | 8.35E-02 | 0.37 | 1.38 | 8.99E-01 | -0.03 | 0.36 | 1.32E-02 | -0.40 | 1.23 | 779207 | 463757 | 653560 | 617177 | 546032 | 267775 | 761088 | 821151 |
| RP_POS_308_88013a15_42 | RP | POS | 15.42 | 308.8801 | | | | 3.68E-01 | -0.19 | 1.13 | 8.35E-02 | -0.34 | 1.17 | 1.31E-01 | -0.36 | 0.68 | 9.46E-01 | -0.01 | 1.17 | 2124089 | 533804 | 2026206 | 520188 | 2116264 | 466647 | 1936149 | 558929 |
| RP_POS_279_15674a9_575 | RP | POS | 9.58 | 279.1567 | | | | 3.46E-04 | 0.47 | 0.86 | 8.38E-02 | -0.38 | 1.21 | 1.58E-02 | 0.28 | 0.87 | 2.27E-03 | 0.67 | 1.00 | 3784654 | 1888277 | 6160164 | 5533610 | 7120765 | 7007941 | 5199562 | 3297301 |
| HILIC_POS_104_07104a5_179 | HILIC | POS | 5.179 | 104.0710 | | | | 6.86E-03 | 0.44 | 0.98 | 8.41E-02 | 0.37 | 1.07 | 3.06E-03 | 0.63 | 0.98 | 1.42E-01 | 0.26 | 0.74 | 580257 | 201771 | 712612 | 317184 | 657779 | 270352 | 767445 | 352192 |
| RP_POS_167_08563a7_928 | RP | POS | 7.93 | 167.0856 | | | | 9.37E-01 | -0.02 | 0.43 | 8.41E-02 | 0.34 | 1.19 | 5.70E-01 | 0.15 | 0.23 | 3.25E-01 | -0.19 | 0.76 | 3685948 | 20693138 | 3358873 | 19075025 | 30340 | 20179 | 6687405 | 26693219 |
| RP_POS_501_3754a10_937 | RP | POS | 10.94 | 501.3754 | | | | 7.99E-01 | 0.05 | 0.57 | 8.43E-02 | -0.34 | 1.38 | 6.02E-01 | -0.12 | 0.27 | 3.57E-01 | 0.22 | 0.60 | 28134054 | 4703438 | 28375274 | 4391611 | 29133747 | 4861145 | 27616801 | 3763153 |
| HILIC_POS_224_12804a6_135 | HILIC | POS | 6.135 | 224.1280 | | | | 1.18E-01 | 0.31 | 1.06 | 8.44E-02 | -0.35 | 0.96 | 5.45E-01 | 0.13 | 0.54 | 3.08E-02 | 0.48 | 1.26 | 5845766 | 2295491 | 6604717 | 2518585 | 7039373 | 2535909 | 6170061 | 2449408 |
| RP_POS_309_05768a8_02 | RP | POS | 8.02 | 309.0577 | | | | 2.49E-02 | 0.33 | 1.10 | 8.46E-02 | 0.38 | 1.32 | 8.82E-03 | 0.52 | 1.41 | 4.06E-01 | 0.14 | 0.59 | 369221 | 466603 | 657823 | 963930 | 491531 | 855901 | 824114 | 1043208 |
| RP_POS_550_82623a10_932 | RP | POS | 10.93 | 550.8262 | | | | 3.41E-02 | -0.50 | 1.26 | 8.50E-02 | -0.31 | 1.60 | 9.89E-03 | -0.66 | 1.22 | 1.64E-01 | -0.35 | 1.07 | 1877941 | 605390 | 1623143 | 457626 | 1701961 | 454179 | 1544325 | 451842 |
| HILIC_POS_104_07104a5_353 | HILIC | POS | 5.353 | 104.0710 | | | | 8.91E-02 | 0.26 | 0.53 | 8.51E-02 | -0.38 | 1.39 | 7.03E-01 | 0.07 | 0.23 | 1.63E-02 | 0.45 | 0.79 | 1635393 | 562905 | 1881997 | 1035357 | 2060300 | 1001527 | 1703693 | 1047792 |
| RP_POS_838_63074a14_677 | RP | POS | 14.68 | 838.6307 | | | | 1.42E-04 | 0.73 | 1.26 | 8.51E-02 | 0.34 | 1.10 | 7.27E-05 | 0.90 | 1.39 | 4.84E-03 | 0.56 | 1.10 | 2148271 | 1410804 | 3359854 | 1629417 | 3079123 | 1409613 | 3640586 | 1793415 |
| RP_POS_304_21173a4_828 | RP | POS | 4.83 | 304.2117 | | | | 3.83E-02 | 0.35 | 0.67 | 8.51E-02 | -0.37 | 1.47 | 3.71E-01 | 0.17 | 0.47 | 1.09E-02 | 0.53 | 1.00 | 1057668 | 524818 | 1311042 | 764050 | 1442617 | 812576 | 1179467 | 695592 |
| RP_POS_646_75616a0_501 | RP | POS | 0.50 | 646.7562 | | | | 7.20E-04 | 0.60 | 1.16 | 8.57E-02 | -0.35 | 1.26 | 2.67E-02 | 0.42 | 0.97 | 3.39E-04 | 0.77 | 1.35 | 195842 | 154151 | 317307 | 208991 | 353234 | 226165 | 281381 | 185636 |
| RP_POS_805_01013a10_618 | RP | POS | 10.62 | 805.0101 | | | | 4.79E-03 | 0.66 | 1.45 | 8.58E-02 | -0.31 | 1.15 | 4.69E-02 | 0.50 | 1.06 | 8.56E-04 | 0.81 | 1.60 | 3458593 | 1113995 | 4099265 | 878600 | 4250357 | 735111 | 3948172 | 986231 |
| HILIC_NEG_144_92345a1_342 | HILIC | NEG | 1.342 | 144.9235 | | | | 4.60E-02 | -0.43 | 0.97 | 8.61E-02 | -0.33 | 1.16 | 7.88E-03 | -0.60 | 1.11 | 2.78E-01 | -0.27 | 0.44 | 7505589 | 4061152 | 5857205 | 3635120 | 6482148 | 4236943 | 5232262 | 2819420 |
| RP_POS_153_06555a0_711 | RP | POS | 0.71 | 153.0656 | | | | 4.47E-02 | -0.37 | 0.66 | 8.64E-02 | -0.35 | 1.13 | 1.19E-02 | -0.55 | 0.87 | 3.40E-01 | -0.19 | 0.82 | 3004804 | 1336225 | 2416569 | 1634567 | 2696921 | 1532988 | 2136217 | 1699322 |
| RP_POS_120_97954a11_209 | RP | POS | 11.21 | 120.9795 | | | | 6.71E-01 | -0.10 | 0.87 | 8.64E-02 | -0.33 | 1.20 | 2.90E-01 | -0.26 | 0.47 | 7.87E-01 | 0.07 | 1.20 | 2596196 | 529228 | 2552146 | 434242 | 2626614 | 437835 | 2477677 | 421857 |
| RP_POS_870_52057a12_693 | RP | POS | 12.69 | 870.5206 | | | | 1.29E-04 | 0.73 | 1.38 | 8.66E-02 | -0.34 | 1.05 | 8.54E-03 | 0.56 | 1.08 | 2.26E-05 | 0.90 | 1.52 | 3639968 | 1118158 | 4610607 | 1311907 | 4835494 | 1231142 | 4385721 | 1363232 |
| HILIC_NEG_347_71643a1_363 | HILIC | NEG | 1.363 | 347.7164 | | | | 1.89E-08 | 0.95 | 1.65 | 8.66E-02 | -0.34 | 0.91 | 1.04E-04 | 0.78 | 1.37 | 3.26E-09 | 1.12 | 2.19 | 252489 | 61938 | 342325 | 92850 | 358247 | 80810 | 326404 | 101835 |
| RP_POS_292_04221a6_862 | RP | POS | 6.86 | 292.0422 | | | | 2.32E-02 | -0.38 | 0.90 | 8.69E-02 | 0.36 | 1.22 | 3.19E-01 | -0.20 | 0.71 | 4.64E-03 | -0.56 | 1.14 | 1761529 | 129528 | 1691592 | 197044 | 1657857 | 192604 | 1725327 | 197550 |
| HILIC_POS_544_33893a1_088 | HILIC | POS | 1.088 | 544.3389 | | | | 9.72E-02 | 0.36 | 0.76 | 8.74E-02 | -0.33 | 0.97 | 4.31E-01 | 0.19 | 0.39 | 2.12E-02 | 0.52 | 1.13 | 5440593 | 2488724 | 6277111 | 2259214 | 6663689 | 1895118 | 5890532 | 2532943 |
| RP_POS_560_30206a10_312 | RP | POS | 10.31 | 560.3021 | | | | 1.74E-01 | 0.27 | 0.93 | 8.75E-02 | -0.35 | 1.63 | 6.64E-01 | 0.10 | 0.66 | 4.48E-02 | 0.44 | 0.91 | 1904195 | 432249 | 2027100 | 460530 | 2105805 | 440715 | 1948394 | 470778 |
| RP_POS_1005_63196a11_119 | RP | POS | 11.12 | 1005.6320 | | | | 7.68E-07 | 0.59 | 1.25 | 8.76E-02 | -0.38 | 1.38 | 1.92E-03 | 0.40 | 1.26 | 9.85E-05 | 0.78 | 1.41 | 122355 | 73634 | 708585 | 1104741 | 897605 | 1290666 | 519565 | 852690 |
| RP_POS_809_48456a10_881 | RP | POS | 10.88 | 809.4846 | | | | 1.20E-02 | 0.30 | 0.93 | 8.77E-02 | -0.39 | 1.28 | 4.34E-01 | 0.10 | 0.72 | 1.08E-02 | 0.49 | 1.09 | 2158745 | 167898 | 2491021 | 1266071 | 2707512 | 1452093 | 2274529 | 1017036 |
| RP_POS_538_34021a11_127 | RP | POS | 11.13 | 538.3402 | | | | 2.52E-07 | 0.61 | 1.31 | 8.79E-02 | -0.38 | 1.40 | 5.71E-04 | 0.42 | 1.33 | 8.10E-05 | 0.80 | 1.43 | 39745 | 27115 | 810379 | 1390966 | 1048291 | 1658174 | 572467 | 1021764 |
| RP_POS_1071_724a10_932 | RP | POS | 10.93 | 1071.7240 | | | | 1.47E-02 | -0.58 | 1.25 | 8.80E-02 | -0.31 | 1.55 | 3.51E-03 | -0.73 | 1.29 | 9.40E-02 | -0.42 | 1.02 | 875852 | 456926 | 652149 | 349288 | 711760 | 371215 | 592538 | 318499 |
| RP_NEG_701_1557a9_766 | RP | NEG | 9.77 | 701.1557 | | | | 5.86E-01 | -0.12 | 0.39 | 8.81E-02 | -0.33 | 1.05 | 2.31E-01 | -0.29 | 0.53 | 8.63E-01 | 0.04 | 0.26 | 4683758 | 311079 | 4650269 | 264808 | 4695450 | 278717 | 4605089 | 244599 |
| RP_POS_353_26627a10_768 | RP | POS | 10.77 | 353.2663 | | | | 5.22E-02 | 0.32 | 0.56 | 8.81E-02 | 0.37 | 1.02 | 2.20E-02 | 0.50 | 0.83 | 4.33E-01 | 0.13 | 0.44 | 913161 | 505500 | 1150628 | 809442 | 1012345 | 625331 | 1288912 | 945357 |
| HILIC_POS_202_1548a3_509 | HILIC | POS | 3.509 | 202.1548 | | | | 3.68E-01 | 0.16 | 0.47 | 8.82E-02 | 0.36 | 1.16 | 1.30E-01 | 0.34 | 0.54 | 9.23E-01 | -0.02 | 0.04 | 1500770 | 1471027 | 1792969 | 1902777 | 1468073 | 1526989 | 2117864 | 2183550 |
| RP_NEG_275_82349a15_561 | RP | NEG | 15.56 | 275.8235 | | | | 3.81E-02 | -0.44 | 0.89 | 8.82E-02 | -0.33 | 1.46 | 9.29E-03 | -0.60 | 1.03 | 2.40E-01 | -0.27 | 0.71 | 1056667 | 45225 | 1037297 | 43363 | 1044693 | 43712 | 1029902 | 42151 |
| RP_NEG_407_20792a7_044 | RP | NEG | 7.04 | 407.2079 | | | | 9.81E-02 | -0.42 | 2.02 | 8.84E-02 | 0.29 | 1.05 | 3.00E-01 | -0.27 | 1.20 | 3.63E-02 | -0.57 | 1.55 | 5616556 | 2989103 | 4661924 | 1960347 | 4327875 | 1986742 | 4995973 | 1894589 |
| HILIC_POS_156_10188a0_49 | HILIC | POS | 0.49 | 156.1019 | | | | 1.19E-01 | 0.29 | 0.48 | 8.85E-02 | 0.35 | 1.17 | 4.71E-02 | 0.46 | 0.72 | 5.52E-01 | 0.11 | 0.28 | 528619 | 127857 | 572140 | 157355 | 545274 | 114774 | 599005 | 188097 |
| RP_POS_768_58582a14_362 | RP | POS | 14.36 | 768.5858 | | | | 2.12E-02 | -0.49 | 0.83 | 8.86E-02 | 0.33 | 1.01 | 1.76E-01 | -0.33 | 0.61 | 3.39E-03 | -0.66 | 1.12 | 1741433 | 1035422 | 1249133 | 963785 | 1084804 | 785731 | 1413461 | 1097377 |
| RP_NEG_606_30365a10_124 | RP | NEG | 10.12 | 606.3037 | | | | 4.60E-22 | 1.37 | 2.22 | 8.86E-02 | 0.31 | 1.10 | 1.23E-13 | 1.52 | 2.30 | 1.47E-15 | 1.21 | 2.28 | 3398824 | 1076448 | 7120045 | 2464790 | 6699708 | 1924161 | 7540383 | 2865552 |
| RP_POS_163_97665a10_741 | RP | POS | 10.74 | 163.9767 | | | | 9.70E-01 | 0.01 | 0.33 | 8.90E-02 | 0.33 | 1.03 | 4.82E-01 | 0.17 | 0.41 | 5.26E-01 | -0.15 | 0.46 | 2190533 | 1028818 | 2198259 | 863505 | 2051385 | 851686 | 2345133 | 858547 |
| RP_POS_494_31418a11_136 | RP | POS | 11.14 | 494.3142 | | | | 3.85E-07 | 0.60 | 1.29 | 8.91E-02 | -0.38 | 1.38 | 7.98E-04 | 0.42 | 1.31 | 9.57E-05 | 0.79 | 1.41 | 44114 | 32642 | 636059 | 1086994 | 821233 | 1292782 | 450885 | 803717 |
| RP_POS_478_38855a13_932 | RP | POS | 13.93 | 478.3886 | | | | 8.09E-02 | 0.42 | 0.75 | 8.93E-02 | -0.30 | 1.15 | 2.91E-01 | 0.27 | 0.51 | 2.62E-02 | 0.57 | 0.95 | 6393135 | 1722790 | 6982007 | 1250840 | 7194603 | 1217934 | 6769412 | 1259178 |
| RP_POS_535_35516a10_932 | RP | POS | 10.93 | 535.3552 | | | | 8.57E-02 | -0.41 | 1.05 | 8.95E-02 | -0.31 | 1.53 | 2.90E-02 | -0.56 | 1.02 | 3.04E-01 | -0.26 | 0.86 | 795582 | 273871 | 703196 | 203134 | 737695 | 195542 | 668698 | 206648 |
| RP_POS_593_26196a8_418 | RP | POS | 8.42 | 593.2620 | | | | 5.37E-01 | 0.14 | 0.33 | 8.98E-02 | 0.33 | 1.09 | 2.49E-01 | 0.30 | 0.45 | 8.99E-01 | -0.03 | 0.43 | 1153136 | 2777754 | 1493022 | 2404738 | 1083845 | 1614486 | 1902199 | 2955553 |
| HILIC_POS_208_1333a1_357 | HILIC | POS | 1.357 | 208.1333 | | | | 2.67E-01 | 0.17 | 0.43 | 9.03E-02 | 0.37 | 0.92 | 1.32E-01 | 0.36 | 0.77 | 9.05E-01 | -0.01 | 0.15 | 721565 | 1160473 | 1046506 | 2067064 | 693714 | 756217 | 1399298 | 2794073 |
| RP_NEG_254_99406a4_57 | RP | NEG | 4.57 | 254.9941 | | | | 3.13E-03 | -0.64 | 0.99 | 9.03E-02 | -0.32 | 1.11 | 8.23E-04 | -0.80 | 1.27 | 3.69E-02 | -0.47 | 0.81 | 2173386 | 1163525 | 1452037 | 1075006 | 1634176 | 1037930 | 1269898 | 1090806 |
| RP_POS_514_31171a11_118 | RP | POS | 11.12 | 514.3117 | | | | 5.46E-07 | 0.60 | 1.29 | 9.03E-02 | -0.38 | 1.39 | 7.20E-04 | 0.41 | 1.34 | 1.32E-04 | 0.78 | 1.40 | 76144 | 41159 | 1177066 | 2053308 | 1525699 | 2469809 | 828434 | 1473154 |
| RP_POS_357_19315a11_043 | RP | POS | 11.04 | 357.1932 | | | | 1.35E-04 | 0.72 | 1.65 | 9.04E-02 | 0.34 | 1.30 | 1.20E-04 | 0.89 | 1.59 | 3.43E-03 | 0.56 | 1.50 | 465293 | 320048 | 742677 | 378687 | 678432 | 291868 | 806922 | 442859 |
| RP_POS_500_27686a9_93 | RP | POS | 9.93 | 500.2769 | | | | 1.23E-04 | 0.62 | 1.23 | 9.04E-02 | -0.36 | 1.00 | 9.96E-03 | 0.44 | 1.09 | 1.62E-04 | 0.80 | 1.34 | 578426 | 422589 | 987119 | 689081 | 1103917 | 774557 | 870322 | 575826 |
| RP_POS_522_25916a10_049 | RP | POS | 10.05 | 522.2592 | | | | 2.17E-05 | 0.68 | 1.29 | 9.06E-02 | -0.35 | 1.00 | 3.51E-03 | 0.50 | 1.18 | 4.26E-05 | 0.86 | 1.42 | 608248 | 479693 | 1132464 | 804916 | 1268791 | 894338 | 996136 | 686464 |
| RP_POS_499_82727a4_156 | RP | POS | 4.16 | 499.8273 | | | | 1.55E-02 | 0.38 | 0.89 | 9.07E-02 | -0.37 | 1.01 | 2.65E-01 | 0.20 | 0.56 | 5.74E-03 | 0.56 | 0.93 | 1609409 | 1786082 | 2702113 | 3111956 | 3228836 | 3356299 | 2175390 | 2781230 |
| RP_NEG_278_88992a15_47 | RP | NEG | 15.47 | 278.8899 | | | | 3.30E-01 | 0.15 | 0.72 | 9.09E-02 | -0.37 | 1.09 | 8.75E-01 | -0.04 | 0.24 | 1.62E-02 | 0.34 | 1.37 | 898552 | 38100 | 907949 | 68464 | 919584 | 36916 | 896314 | 88494 |
| RP_POS_778_53662a12_381 | RP | POS | 12.38 | 778.5366 | | | | 5.65E-02 | -0.36 | 1.12 | 9.10E-02 | -0.35 | 1.03 | 1.02E-02 | -0.54 | 1.32 | 3.88E-01 | -0.19 | 0.48 | 5878083 | 2472188 | 4864485 | 2837148 | 5344350 | 3061605 | 4384620 | 2533718 |
| HILIC_NEG_217_0298a4_977 | HILIC | NEG | 4.977 | 217.0298 | | | | 6.87E-01 | -0.08 | 0.37 | 9.12E-02 | 0.34 | 1.00 | 7.07E-01 | 0.09 | 0.23 | 2.09E-01 | -0.25 | 0.92 | 3758067 | 2719373 | 3531511 | 2865342 | 3046303 | 2022840 | 4016718 | 3466349 |
| HILIC_POS_203_05269a5_167 | HILIC | POS | 5.167 | 203.0527 | | | | 1.45E-01 | 0.27 | 0.83 | 9.13E-02 | 0.35 | 0.97 | 4.06E-02 | 0.45 | 0.86 | 6.46E-01 | 0.09 | 0.48 | 16771643 | 6077637 | 18695681 | 7402200 | 17445069 | 7004303 | 19946293 | 7645338 |
| RP_NEG_212_00209a3_784 | RP | NEG | 3.78 | 212.0021 | | | | 2.64E-02 | -0.52 | 1.00 | 9.13E-02 | -0.31 | 1.03 | 9.53E-03 | -0.68 | 1.11 | 1.24E-01 | -0.37 | 0.67 | 1669298 | 1101716 | 1180972 | 846786 | 1324090 | 727370 | 1037855 | 936941 |
| RP_POS_553_43188a10_709 | RP | POS | 10.71 | 553.4319 | | | | 4.35E-01 | -0.16 | 0.80 | 9.14E-02 | -0.34 | 1.60 | 1.39E-01 | -0.33 | 1.47 | 9.48E-01 | 0.01 | 0.33 | 3465177 | 256672 | 3423693 | 268870 | 3469103 | 276161 | 3378282 | 256095 |
| HILIC_NEG_312_83633a6_125 | HILIC | NEG | 6.125 | 312.8363 | | | | 3.15E-01 | 0.18 | 0.74 | 9.16E-02 | 0.36 | 1.26 | 7.72E-02 | 0.36 | 0.99 | 9.93E-01 | 0.00 | 0.03 | 789119 | 151785 | 823265 | 204355 | 788770 | 211020 | 857759 | 193433 |
| RP_POS_404_73193a12_458 | RP | POS | 12.46 | 404.7319 | | | | 4.47E-01 | 0.16 | 0.60 | 9.17E-02 | -0.34 | 1.02 | 9.58E-01 | -0.01 | 0.80 | 1.45E-01 | 0.33 | 0.66 | 474715 | 295751 | 520642 | 293613 | 570184 | 269720 | 471100 | 310514 |
| RP_POS_264_95346a15_333 | RP | POS | 15.33 | 264.9535 | | | | 2.02E-01 | -0.22 | 0.67 | 9.17E-02 | -0.36 | 1.00 | 4.43E-02 | -0.40 | 0.93 | 8.53E-01 | -0.04 | 0.30 | 2817908 | 91116 | 2791161 | 131106 | 2813280 | 134670 | 2769042 | 124876 |
| RP_NEG_808_83472a0_487 | RP | NEG | 0.49 | 808.8347 | | | | 2.49E-01 | 0.16 | 0.88 | 9.17E-02 | 0.38 | 1.42 | 7.02E-02 | 0.35 | 0.89 | 8.79E-01 | -0.03 | 1.01 | 5174334 | 735365 | 5421386 | 1690450 | 5136113 | 1503522 | 5706658 | 1829454 |
| HILIC_POS_371_23959a1_133 | HILIC | POS | 1.133 | 371.2396 | | | | 1.40E-02 | -0.52 | 0.94 | 9.18E-02 | -0.33 | 1.01 | 4.08E-03 | -0.68 | 1.10 | 1.12E-01 | -0.35 | 0.77 | 3218811 | 1145571 | 2632368 | 1095582 | 2817195 | 1023292 | 2447541 | 1143788 |
| RP_POS_463_3028a11_147 | RP | POS | 11.15 | 463.3028 | | | | 1.75E-05 | 0.57 | 1.04 | 9.18E-02 | 0.37 | 1.34 | 1.53E-04 | 0.76 | 1.31 | 8.52E-03 | 0.39 | 1.10 | 441138 | 196963 | 736541 | 563063 | 641489 | 462154 | 831593 | 639159 |
| RP_POS_264_87231a15_465 | RP | POS | 15.47 | 264.8723 | | | | 1.29E-01 | 0.31 | 1.00 | 9.19E-02 | -0.34 | 0.99 | 5.32E-01 | 0.14 | 0.85 | 3.74E-02 | 0.48 | 1.04 | 1749643 | 539673 | 1919931 | 555517 | 2013597 | 563781 | 1826264 | 536417 |
| RP_POS_615_45837a11_158 | RP | POS | 11.16 | 615.4584 | | | | 2.00E-01 | -0.34 | 0.60 | 9.22E-02 | -0.28 | 1.04 | 7.19E-02 | -0.48 | 0.83 | 4.87E-01 | -0.20 | 0.55 | 3629636 | 5269124 | 2349028 | 3105455 | 2873820 | 3852973 | 1824236 | 2019174 |
| RP_POS_275_63217a10_497 | RP | POS | 10.50 | 275.6322 | | | | 3.80E-01 | -0.19 | 0.60 | 9.22E-02 | 0.33 | 1.00 | 9.10E-01 | -0.03 | 0.41 | 1.11E-01 | -0.36 | 0.73 | 469562 | 173592 | 439198 | 153022 | 413372 | 113870 | 465024 | 181610 |
| HILIC_NEG_182_98662a6_093 | HILIC | NEG | 6.093 | 182.9866 | | | | 8.95E-01 | 0.02 | 0.84 | 9.22E-02 | -0.37 | 1.10 | 3.86E-01 | -0.16 | 0.65 | 3.16E-01 | 0.20 | 1.10 | 325639 | 154604 | 330531 | 246477 | 372058 | 261466 | 289003 | 225517 |
| HILIC_POS_141_0659a5_265 | HILIC | POS | 5.265 | 141.0659 | | | | 7.30E-01 | 0.06 | 0.69 | 9.32E-02 | -0.36 | 0.96 | 4.82E-01 | -0.12 | 0.26 | 2.78E-01 | 0.24 | 0.38 | 1278183 | 1125699 | 1368240 | 1675323 | 1650002 | 1950270 | 1086478 | 1305805 |
| HILIC_POS_139_05026a1_057 | HILIC | POS | 1.057 | 139.0503 | | | | 5.28E-01 | -0.11 | 0.40 | 9.32E-02 | -0.35 | 1.12 | 8.79E-02 | -0.29 | 0.75 | 7.96E-01 | 0.06 | 0.16 | 816983 | 453331 | 754426 | 573435 | 851169 | 739962 | 657683 | 312604 |
| HILIC_POS_290_1601a1_51 | HILIC | POS | 1.51 | 290.1601 | | | | 2.28E-04 | 0.43 | 1.04 | 9.38E-02 | 0.38 | 1.00 | 2.67E-03 | 0.62 | 0.92 | 2.78E-02 | 0.24 | 0.69 | 240239 | 398839 | 9040791 | 22991172 | 5175244 | 15385807 | 12906338 | 28297663 |
| HILIC_NEG_278_98141a5_102 | HILIC | NEG | 5.102 | 278.9814 | | | | 1.76E-01 | 0.19 | 0.44 | 9.39E-02 | 0.37 | 1.10 | 8.40E-02 | 0.38 | 0.66 | 9.70E-01 | 0.00 | 0.60 | 4394235 | 1465995 | 4967619 | 3321013 | 4408998 | 2045595 | 5526240 | 4178759 |
| RP_POS_1023_64001a10_625 | RP | POS | 10.63 | 1023.6400 | | | | 7.93E-03 | 0.51 | 1.24 | 9.39E-02 | 0.34 | 1.46 | 3.59E-03 | 0.68 | 1.23 | 8.21E-02 | 0.34 | 1.10 | 859719 | 487880 | 1141817 | 556319 | 1048474 | 445327 | 1235161 | 639686 |
| RP_POS_194_15164a7_794 | RP | POS | 7.79 | 194.1516 | | | | 3.09E-04 | 0.42 | 0.87 | 9.42E-02 | -0.38 | 1.16 | 1.08E-02 | 0.23 | 0.92 | 4.51E-03 | 0.61 | 0.97 | 21552 | 58563 | 537741 | 1377015 | 769334 | 1774883 | 306148 | 755891 |
| RP_POS_308_97095a15_44 | RP | POS | 15.44 | 308.9710 | | | | 3.29E-01 | 0.18 | 0.41 | 9.43E-02 | -0.35 | 1.03 | 9.57E-01 | 0.01 | 0.36 | 1.09E-01 | 0.36 | 0.70 | 2681649 | 360381 | 2756854 | 421947 | 2827505 | 449907 | 2686203 | 383547 |
| RP_NEG_473_40143a13_181 | RP | NEG | 13.18 | 473.4014 | | | | 2.45E-02 | -0.48 | 0.84 | 9.49E-02 | -0.32 | 1.00 | 5.65E-03 | -0.65 | 1.11 | 1.70E-01 | -0.32 | 0.56 | 14546471 | 1083618 | 14046054 | 990511 | 14211570 | 1037258 | 13880538 | 922189 |
| RP_POS_379_2608a11_202 | RP | POS | 11.20 | 379.2608 | | | | 6.30E-07 | 0.63 | 1.47 | 9.50E-02 | 0.37 | 1.01 | 1.43E-04 | 0.82 | 1.34 | 1.31E-04 | 0.45 | 1.50 | 503374 | 393236 | 1379515 | 1518400 | 1125073 | 968371 | 1633957 | 1894271 |
| RP_POS_516_32715a11_131 | RP | POS | 11.13 | 516.3272 | | | | 2.80E-07 | 0.61 | 1.30 | 9.51E-02 | -0.37 | 1.38 | 6.45E-04 | 0.43 | 1.31 | 8.56E-05 | 0.80 | 1.42 | 42846 | 23626 | 825909 | 1419682 | 1063441 | 1684591 | 588377 | 1057892 |
| HILIC_NEG_201_95786a1_374 | HILIC | NEG | 1.374 | 201.9579 | | | | 4.28E-02 | -0.37 | 0.67 | 9.52E-02 | -0.35 | 0.96 | 6.27E-03 | -0.54 | 1.00 | 3.68E-01 | -0.20 | 0.31 | 3169332 | 1119011 | 2668963 | 1395602 | 2902065 | 1548198 | 2435862 | 1194718 |
| RP_POS_308_66025a10_502 | RP | POS | 10.50 | 308.6603 | | | | 3.01E-01 | -0.21 | 0.61 | 9.53E-02 | 0.33 | 0.99 | 8.48E-01 | -0.04 | 0.32 | 8.73E-02 | -0.38 | 0.79 | 1947756 | 677855 | 1803778 | 680880 | 1690124 | 617475 | 1917433 | 727336 |
| RP_POS_1070_7865a0_488 | RP | POS | 0.49 | 1070.7865 | | | | 3.28E-05 | 0.50 | 0.95 | 9.56E-02 | -0.37 | 1.13 | 5.51E-02 | 0.31 | 0.70 | 6.73E-05 | 0.69 | 1.32 | 442418 | 25141 | 517518 | 167685 | 545475 | 165323 | 489562 | 166983 |

**Supplementary table 1: Metabolomics results** (in asceding order according to p-value from progressive ALD vs  non-progressive ALD comparison, with molecular features with ID_level of 1-2 first)

| | | | | | | | | all_ALD_vs_control | | | Prog_ALD_vs_non-prog_ALD | | | Prog_ALD_vs_control_ALD | | | Non-prog_ALD_vs_control | | | Healthy_control | | all_ALD | | Non-prog_ALD | | Progressive_ALD | |
|---|---|---|---|---|---|---|---|---|---|---|---|---|---|---|---|---|---|---|---|---|---|---|---|---|---|---|---|
| | | | | | | | | t-test | Cohen's | PLS-DA | t-test | Cohen's | PLS-DA | t-test | Cohen's | PLS-DA | t-test | Cohen's | PLS-DA | | | | | | | | |
| Feature_ID | RP_HILIC | POS_NEG | Average.Rt | Average.Mz | Metabolite.name | curated_MSMS | ID_level | p | d | VIP | p | d | VIP | p | d | VIP | p | d | VIP | Mean | SD | Mean | SD | Mean | SD | Mean | SD |
| HILIC_NEG_568_36401a1_057 | HILIC | NEG | 1.057 | 568.3640 | | | | 6.28E-01 | -0.10 | 0.43 | 9.57E-02 | -0.33 | 1.13 | 2.63E-01 | -0.27 | 0.62 | 7.93E-01 | 0.06 | 0.10 | 352257 | 118385 | 340739 | 109532 | 359000 | 103481 | 322477 | 113361 |
| RP_POS_481_34918a11_205 | RP | POS | 11.21 | 481.3492 | | | | 2.92E-06 | 0.55 | 1.22 | 9.57E-02 | -0.37 | 1.38 | 9.88E-04 | 0.37 | 1.36 | 3.99E-04 | 0.74 | 1.32 | 247656 | 107301 | 2743263 | 5028205 | 3583895 | 6204967 | 1902632 | 3337348 |
| RP_POS_492_29816a11_123 | RP | POS | 11.12 | 492.2982 | | | | 5.09E-07 | 0.60 | 1.29 | 9.60E-02 | -0.37 | 1.38 | 7.94E-04 | 0.41 | 1.33 | 1.23E-04 | 0.78 | 1.41 | 39874 | 28148 | 815364 | 1441947 | 1055964 | 1722077 | 574763 | 1056961 |
| RP_POS_233_0696a15_34 | RP | POS | 15.34 | 233.0696 | | | | 4.52E-01 | -0.15 | 0.76 | 9.63E-02 | -0.34 | 1.10 | 1.66E-01 | -0.32 | 0.56 | 9.37E-01 | 0.02 | 0.88 | 1645799 | 444469 | 1576661 | 462648 | 1653650 | 427520 | 1499672 | 487426 |
| HILIC_NEG_200_9707a0_701 | HILIC | NEG | 0.701 | 200.9707 | | | | 1.27E-12 | 1.04 | 1.78 | 9.65E-02 | 0.33 | 1.37 | 2.70E-09 | 1.21 | 2.10 | 5.68E-08 | 0.88 | 1.80 | 196515 | 144410 | 513589 | 302727 | 463211 | 257337 | 563967 | 337277 |
| HILIC_POS_199_1806a5_648 | HILIC | POS | 5.648 | 199.1806 | | | | 5.10E-01 | 0.12 | 0.61 | 9.65E-02 | -0.35 | 1.38 | 8.23E-01 | -0.05 | 0.29 | 1.59E-01 | 0.30 | 0.51 | 3979574 | 775841 | 4089019 | 916384 | 4241424 | 866562 | 3936614 | 947800 |
| RP_NEG_828_57788a13_385 | RP | NEG | 13.39 | 828.5779 | | | | 3.36E-01 | -0.20 | 0.50 | 9.67E-02 | -0.33 | 1.05 | 1.08E-01 | -0.36 | 0.71 | 8.95E-01 | -0.03 | 0.40 | 53582748 | 17347193 | 50157983 | 17452304 | 53058440 | 17709062 | 47257526 | 16870160 |
| RP_POS_172_16959a7_794 | RP | POS | 7.79 | 172.1696 | | | | 3.06E-04 | 0.42 | 0.87 | 9.68E-02 | -0.38 | 1.15 | 1.08E-02 | 0.24 | 0.92 | 4.52E-03 | 0.61 | 0.97 | 136260 | 304853 | 2490731 | 6272121 | 3537570 | 8073657 | 1443891 | 3472958 |
| RP_POS_732_76514a0_499 | RP | POS | 0.50 | 732.7651 | | | | 5.41E-02 | 0.35 | 0.97 | 9.71E-02 | -0.34 | 1.26 | 3.71E-01 | 0.18 | 0.69 | 1.50E-02 | 0.53 | 1.14 | 285490 | 184634 | 363592 | 227798 | 401412 | 234886 | 325772 | 216223 |
| RP_POS_752_82928a0_493 | RP | POS | 0.49 | 752.8293 | | | | 6.78E-02 | 0.25 | 0.89 | 9.71E-02 | 0.37 | 1.42 | 2.84E-02 | 0.44 | 0.96 | 6.68E-01 | 0.07 | 0.99 | 1307664 | 311631 | 1484595 | 786466 | 1353940 | 651311 | 1615250 | 889177 |
| HILIC_POS_184_09425a5_028 | HILIC | POS | 5.028 | 184.0943 | | | | 2.26E-03 | 0.46 | 0.83 | 9.76E-02 | 0.36 | 1.08 | 1.06E-03 | 0.64 | 1.13 | 1.10E-01 | 0.28 | 0.53 | 2908893 | 1327422 | 3990248 | 2531814 | 3570471 | 2372103 | 4410025 | 2639033 |
| RP_POS_1021_62463a10_324 | RP | POS | 10.32 | 1021.6246 | | | | 1.25E-02 | 0.47 | 1.16 | 9.76E-02 | -0.34 | 1.34 | 1.92E-01 | 0.30 | 0.78 | 1.22E-03 | 0.64 | 1.37 | 4509958 | 1992144 | 5608661 | 2415725 | 6009627 | 1899975 | 5207696 | 2802238 |
| RP_POS_389_17798a11_048 | RP | POS | 11.05 | 389.1780 | | | | 2.50E-09 | 0.84 | 1.74 | 9.78E-02 | 0.35 | 1.10 | 2.46E-07 | 1.01 | 1.77 | 5.15E-05 | 0.66 | 1.61 | 409647 | 224317 | 840766 | 539402 | 751385 | 487084 | 930146 | 578180 |
| RP_POS_536_40613a11_244 | RP | POS | 11.24 | 536.4061 | | | | 1.62E-05 | -1.02 | 1.62 | 9.78E-02 | -0.28 | 1.03 | 2.12E-06 | -1.16 | 1.90 | 4.49E-04 | -0.88 | 1.39 | 959518 | 449392 | 539461 | 343305 | 596417 | 404109 | 482505 | 261146 |
| RP_POS_352_24509a11_253 | RP | POS | 11.25 | 352.2451 | | | | 1.38E-03 | 0.65 | 1.29 | 9.79E-02 | 0.32 | 1.17 | 3.43E-04 | 0.81 | 1.50 | 2.58E-02 | 0.49 | 0.98 | 3244651 | 777386 | 3781073 | 798438 | 3648837 | 791965 | 3913310 | 790646 |
| RP_POS_1067_62854a10_324 | RP | POS | 10.32 | 1067.6285 | | | | 2.55E-05 | -1.10 | 1.83 | 9.79E-02 | -0.25 | 1.09 | 4.92E-06 | -1.22 | 1.94 | 2.96E-04 | -0.97 | 1.49 | 766080 | 483137 | 334317 | 293887 | 383075 | 349138 | 285559 | 218523 |
| RP_POS_338_34259a11_063 | RP | POS | 11.06 | 338.3426 | | | | 1.02E-02 | -0.50 | 0.91 | 9.80E-02 | 0.33 | 1.06 | 1.18E-01 | -0.33 | 0.74 | 2.85E-03 | -0.66 | 1.11 | 1897569 | 112849 | 1834689 | 127662 | 1813549 | 130812 | 1855829 | 122082 |
| RP_POS_1048_12097a0_487 | RP | POS | 0.49 | 1048.1210 | | | | 3.18E-07 | 0.62 | 1.17 | 9.82E-02 | -0.37 | 1.26 | 6.57E-03 | 0.44 | 1.02 | 7.18E-06 | 0.80 | 1.48 | 282265 | 25916 | 395840 | 203266 | 429476 | 205892 | 362204 | 196918 |
| RP_POS_537_16754a0_709 | RP | POS | 0.71 | 537.1675 | | | | 4.65E-07 | 0.65 | 1.03 | 9.83E-02 | 0.36 | 1.16 | 7.70E-05 | 0.83 | 1.22 | 2.76E-04 | 0.47 | 1.11 | 575782 | 267972 | 1136044 | 942763 | 979749 | 666298 | 1292339 | 1141024 |
| RP_POS_447_3461a11_152 | RP | POS | 11.15 | 447.3461 | | | | 5.51E-02 | -0.43 | 1.13 | 9.84E-02 | -0.31 | 1.66 | 1.70E-02 | -0.59 | 1.41 | 2.51E-01 | -0.27 | 0.82 | 1381057 | 682575 | 1117776 | 578036 | 1213355 | 562668 | 1022197 | 582949 |
| RP_POS_355_17245a8_141 | RP | POS | 8.14 | 355.1725 | | | | 9.49E-02 | -0.49 | 0.92 | 9.86E-02 | 0.23 | 1.14 | 2.30E-01 | -0.38 | 0.52 | 3.76E-02 | -0.61 | 1.21 | 681293 | 1252834 | 289267 | 558340 | 196361 | 219139 | 382173 | 751141 |
| RP_POS_499_32358a9_702 | RP | POS | 9.70 | 499.3236 | | | | 8.80E-01 | -0.03 | 0.28 | 9.90E-02 | 0.32 | 1.69 | 6.41E-01 | 0.13 | 0.45 | 3.50E-01 | -0.19 | 0.61 | 489040 | 1187984 | 453632 | 1021112 | 283896 | 378288 | 623368 | 1380116 |
| RP_NEG_470_34955a9_367 | RP | NEG | 9.37 | 470.3496 | | | | 4.66E-11 | 1.10 | 1.76 | 9.91E-02 | 0.31 | 1.00 | 4.66E-10 | 1.26 | 1.94 | 2.31E-07 | 0.95 | 1.75 | 652950 | 279096 | 1148765 | 426475 | 1078367 | 400780 | 1219164 | 443548 |
| RP_POS_499_8269a6_196 | RP | POS | 6.20 | 499.8269 | | | | 9.29E-01 | 0.02 | 0.41 | 9.96E-02 | -0.32 | 1.07 | 5.19E-01 | -0.14 | 0.41 | 4.99E-01 | 0.18 | 0.51 | 1262497 | 1725025 | 1292962 | 1476404 | 1537188 | 1879232 | 1048735 | 866476 |
| RP_POS_562_32642a10_933 | RP | POS | 10.93 | 562.3264 | | | | 1.21E-01 | -0.37 | 0.99 | 9.97E-02 | -0.30 | 1.32 | 4.16E-02 | -0.52 | 0.93 | 3.91E-01 | -0.21 | 0.84 | 4349838 | 1554838 | 3876040 | 1186819 | 4071575 | 1187185 | 3680505 | 1165499 |
| RP_POS_219_02661a0_547 | RP | POS | 0.55 | 219.0266 | | | | 2.20E-03 | -0.37 | 1.05 | 9.99E-02 | 0.37 | 1.31 | 3.22E-01 | -0.18 | 0.94 | 1.41E-04 | -0.56 | 1.43 | 12925509 | 1030841 | 11018993 | 5807784 | 10061834 | 4783539 | 11976153 | 6587650 |
| HILIC_NEG_144_92345a1_485 | HILIC | NEG | 1.485 | 144.9235 | | | | 8.59E-01 | -0.03 | 0.25 | 9.99E-02 | 0.36 | 1.07 | 4.74E-01 | 0.15 | 0.31 | 2.47E-01 | -0.21 | 0.46 | 18145647 | 3487551 | 17997640 | 5575120 | 17079232 | 4772640 | 18916049 | 6188601 |
| RP_POS_211_09677a6_516 | RP | POS | 6.52 | 211.0968 | | | | 9.24E-03 | 0.34 | 0.78 | 1.00E-01 | -0.37 | 0.99 | 2.11E-01 | 0.16 | 0.54 | 1.31E-02 | 0.53 | 0.87 | 4199930 | 13881679 | 17284017 | 43008226 | 24383751 | 52993873 | 10184283 | 28739237 |
| RP_POS_533_84686a11_113 | RP | POS | 11.11 | 533.8469 | | | | 4.48E-07 | 0.60 | 1.29 | 1.00E-01 | -0.37 | 1.36 | 5.90E-04 | 0.42 | 1.33 | 1.28E-04 | 0.78 | 1.40 | 50847 | 33133 | 468637 | 771327 | 595857 | 926067 | 341416 | 558035 |
| RP_POS_257_16812a6_67 | RP | POS | 6.67 | 257.1681 | Unknown | 257.1661(100) | 4 | 2.19E-01 | 0.21 | 0.50 | 1.00E-01 | -0.35 | 1.14 | 8.30E-01 | 0.04 | 0.26 | 9.33E-02 | 0.39 | 0.71 | 2686221 | 2460023 | 3374861 | 3460234 | 3945589 | 4245737 | 2804134 | 2345155 |
| RP_POS_560_35254a11_121 | RP | POS | 11.12 | 560.3525 | | | | 2.23E-07 | 0.62 | 1.29 | 1.00E-01 | -0.37 | 1.37 | 5.09E-04 | 0.44 | 1.32 | 7.99E-05 | 0.80 | 1.42 | 111353 | 61839 | 710734 | 1072891 | 887531 | 1273531 | 533937 | 800006 |
| RP_NEG_440_88892a15_335 | RP | NEG | 15.34 | 440.8889 | | | | 7.47E-01 | -0.06 | 0.61 | 1.01E-01 | -0.33 | 0.99 | 2.96E-01 | -0.23 | 0.62 | 6.55E-01 | 0.10 | 0.42 | 1878810 | 68277 | 1874271 | 71042 | 1885947 | 73324 | 1862595 | 67397 |
| RP_POS_595_2561a10_375 | RP | POS | 10.38 | 595.2561 | | | | 7.03E-01 | 0.07 | 0.97 | 1.01E-01 | -0.34 | 1.49 | 6.41E-01 | -0.10 | 0.91 | 2.47E-01 | 0.24 | 0.78 | 576681 | 85164 | 583681 | 103155 | 600617 | 98531 | 566744 | 105855 |
| RP_POS_309_17834a0_607 | RP | POS | 0.61 | 309.1783 | | | | 6.75E-03 | 0.39 | 0.99 | 1.01E-01 | 0.36 | 1.29 | 4.11E-03 | 0.57 | 1.34 | 2.02E-01 | 0.21 | 0.48 | 210545 | 207686 | 372321 | 457298 | 297220 | 399386 | 447422 | 501488 |
| RP_POS_277_07236a15_433 | RP | POS | 15.43 | 277.0724 | | | | 8.30E-01 | -0.04 | 0.55 | 1.01E-01 | -0.33 | 1.10 | 3.61E-01 | -0.21 | 0.41 | 5.64E-01 | 0.12 | 0.52 | 5943725 | 244425 | 5932812 | 260434 | 5975535 | 238306 | 5890088 | 276625 |
| RP_POS_553_45911a10_577 | RP | POS | 10.58 | 553.4591 | | | | 1.02E-01 | -0.32 | 0.87 | 1.01E-01 | 0.33 | 1.11 | 4.97E-01 | -0.16 | 0.47 | 1.92E-02 | -0.49 | 1.10 | 1560804 | 279158 | 1465059 | 297033 | 1416295 | 241660 | 1513823 | 339125 |
| RP_POS_884_4939a13_062 | RP | POS | 13.06 | 884.4939 | | | | 9.25E-02 | -0.45 | 0.71 | 1.01E-01 | -0.27 | 1.03 | 3.81E-02 | -0.58 | 0.89 | 2.50E-01 | -0.32 | 0.51 | 740787 | 358023 | 625907 | 205954 | 659677 | 204726 | 592136 | 203616 |
| HILIC_POS_149_01862a0_708 | HILIC | POS | 0.708 | 149.0186 | | | | 1.12E-09 | 0.88 | 1.52 | 1.01E-01 | 0.34 | 1.29 | 1.97E-07 | 1.05 | 1.85 | 1.01E-05 | 0.71 | 1.48 | 168077 | 175923 | 496660 | 385436 | 433449 | 330940 | 559872 | 427175 |
| HILIC_NEG_312_83636a4_84 | HILIC | NEG | 4.84 | 312.8364 | | | | 5.23E-01 | -0.14 | 0.42 | 1.02E-01 | -0.32 | 1.20 | 2.11E-01 | -0.30 | 0.52 | 9.13E-01 | 0.03 | 0.45 | 1373003 | 442584 | 1316145 | 411078 | 1383473 | 392695 | 1248818 | 421852 |
| HILIC_NEG_295_73611a3_059 | HILIC | NEG | 3.059 | 295.7361 | | | | 2.57E-02 | -0.27 | 0.86 | 1.02E-01 | 0.37 | 1.09 | 7.03E-01 | -0.08 | 0.58 | 5.54E-05 | -0.45 | 1.42 | 1763429 | 112411 | 1577792 | 796445 | 1446971 | 493583 | 1708613 | 1001521 |
| HILIC_POS_358_29495a0_629 | HILIC | POS | 0.629 | 358.2950 | | | | 5.97E-02 | -0.42 | 0.84 | 1.02E-01 | -0.31 | 0.98 | 1.88E-02 | -0.57 | 0.95 | 2.68E-01 | -0.26 | 0.49 | 1315297 | 848306 | 992911 | 736227 | 1113381 | 712388 | 872441 | 746998 |
| HILIC_NEG_223_00764a1_3 | HILIC | NEG | 1.3 | 223.0076 | | | | 2.86E-10 | 1.04 | 1.73 | 1.02E-01 | 0.32 | 1.00 | 3.42E-09 | 1.20 | 1.89 | 1.02E-06 | 0.88 | 1.68 | 339253 | 135993 | 570088 | 214630 | 534966 | 198391 | 605210 | 226264 |
| RP_NEG_204_98103a2_206 | RP | NEG | 2.21 | 204.9810 | | | | 8.05E-01 | -0.04 | 0.46 | 1.02E-01 | 0.35 | 1.46 | 5.73E-01 | 0.14 | 0.49 | 1.29E-01 | -0.22 | 0.76 | 966479 | 1811524 | 862437 | 2716994 | 415346 | 1109660 | 1309527 | 3643553 |
| RP_POS_193_08624a6_516 | RP | POS | 6.52 | 193.0862 | | | | 8.85E-03 | 0.34 | 0.77 | 1.03E-01 | -0.37 | 0.98 | 2.05E-01 | 0.16 | 0.54 | 1.31E-02 | 0.52 | 0.87 | 568833 | 1943520 | 2441623 | 6152363 | 3449604 | 7577355 | 1433641 | 4121382 |
| HILIC_NEG_119_03493a4_985 | HILIC | NEG | 4.985 | 119.0349 | | | | 4.58E-01 | 0.11 | 0.33 | 1.03E-01 | 0.36 | 1.01 | 1.98E-01 | 0.29 | 0.60 | 6.00E-01 | -0.07 | 0.60 | 1931805 | 1064591 | 2139580 | 2058928 | 1801700 | 1129053 | 2477461 | 2656527 |
| RP_NEG_842_82892a0_49 | RP | NEG | 0.49 | 842.8289 | | | | 1.14E-01 | 0.21 | 0.89 | 1.03E-01 | 0.37 | 1.40 | 4.62E-02 | 0.40 | 0.96 | 8.37E-01 | 0.03 | 0.97 | 1075969 | 256577 | 1205917 | 680062 | 1094717 | 555294 | 1317116 | 775126 |
| RP_POS_1039_67114a10_197 | RP | POS | 10.20 | 1039.6711 | | | | 9.42E-02 | -0.48 | 1.14 | 1.03E-01 | -0.24 | 1.28 | 4.37E-02 | -0.60 | 1.00 | 2.18E-01 | -0.36 | 0.97 | 1717962 | 2297462 | 994950 | 1089207 | 1172793 | 1112752 | 817107 | 1046036 |
| RP_POS_338_23251a9_524 | RP | POS | 9.52 | 338.2325 | | | | 3.16E-01 | -0.16 | 0.49 | 1.03E-01 | 0.35 | 1.23 | 9.43E-01 | 0.02 | 0.51 | 4.98E-02 | -0.34 | 0.77 | 2543030 | 156953 | 2505154 | 253568 | 2463709 | 201711 | 2546600 | 292767 |
| RP_POS_288_87952a15_428 | RP | POS | 15.43 | 288.8795 | | | | 5.50E-01 | -0.08 | 0.39 | 1.04E-01 | -0.36 | 1.08 | 1.98E-01 | -0.27 | 0.41 | 5.04E-01 | 0.10 | 0.35 | 2587882 | 121208 | 2566648 | 282442 | 2612739 | 213424 | 2520557 | 333602 |
| RP_POS_286_23605a10_142 | RP | POS | 10.14 | 286.2361 | | | | 2.55E-02 | 0.42 | 0.88 | 1.04E-01 | 0.33 | 1.18 | 8.24E-03 | 0.59 | 1.02 | 2.17E-01 | 0.25 | 0.83 | 643619 | 192449 | 737686 | 230734 | 700092 | 211536 | 775280 | 244808 |
| RP_POS_333_14423a6_19 | RP | POS | 6.19 | 333.1442 | | | | 7.41E-01 | 0.05 | 0.70 | 1.04E-01 | 0.36 | 1.39 | 3.13E-01 | 0.23 | 0.59 | 3.17E-01 | -0.13 | 0.74 | 945333 | 552303 | 993381 | 1071448 | 818031 | 567310 | 1168732 | 1390984 |
| RP_POS_694_49353a13_57 | RP | POS | 13.57 | 694.4935 | | | | 5.17E-01 | 0.14 | 1.17 | 1.04E-01 | 0.32 | 0.99 | 2.05E-01 | 0.30 | 0.72 | 9.40E-01 | -0.02 | 0.71 | 1387736 | 211445 | 1415073 | 189212 | 1384258 | 193195 | 1445888 | 181853 |
| RP_POS_1050_65881a10_324 | RP | POS | 10.32 | 1050.6588 | | | | 2.39E-03 | -0.78 | 1.35 | 1.04E-01 | -0.27 | 1.12 | 5.12E-04 | -0.91 | 1.52 | 1.79E-02 | -0.65 | 1.02 | 482603 | 348899 | 269753 | 222895 | 306113 | 265845 | 233393 | 164336 |
| RP_NEG_994_7774a0_491 | RP | NEG | 0.49 | 994.7774 | | | | 7.86E-09 | -0.83 | 1.38 | 1.04E-01 | 0.34 | 1.28 | 4.09E-04 | -0.66 | 1.24 | 1.83E-08 | -1.00 | 1.67 | 1021842 | 167425 | 723632 | 376874 | 662271 | 345345 | 784993 | 400024 |
| RP_POS_300_28967a8_869 | RP | POS | 8.87 | 300.2897 | | | | 1.64E-12 | 0.88 | 1.53 | 1.04E-01 | 0.35 | 1.10 | 4.77E-07 | 1.05 | 1.63 | 5.16E-08 | 0.71 | 1.81 | 243135 | 55802 | 495961 | 304128 | 446396 | 219232 | 545525 | 365786 |
| RP_POS_169_05809a0_524 | RP | POS | 0.52 | 169.0581 | | | | 3.40E-02 | 0.28 | 0.91 | 1.04E-01 | -0.37 | 1.20 | 5.71E-01 | 0.09 | 0.65 | 1.14E-02 | 0.46 | 1.10 | 4346235 | 651454 | 4854378 | 2072598 | 5191406 | 2142727 | 4517350 | 1963644 |
| RP_NEG_195_81113a2_201 | RP | NEG | 2.20 | 195.8111 | | | | 6.20E-02 | 0.38 | 1.07 | 1.04E-01 | -0.32 | 1.12 | 2.80E-01 | 0.22 | 0.97 | 2.83E-02 | 0.54 | 0.74 | 10121673 | 1868255 | 10848404 | 1904649 | 11158620 | 2302944 | 10538188 | 1352577 |
| RP_POS_288_64056a10_619 | RP | POS | 10.62 | 288.6406 | | | | 3.11E-01 | 0.23 | 0.90 | 1.04E-01 | -0.31 | 0.96 | 7.60E-01 | 0.08 | 0.60 | 1.05E-01 | 0.38 | 1.03 | 2326242 | 494742 | 2424778 | 401313 | 2490051 | 331927 | 2359504 | 454446 |
| RP_POS_813_98932a10_313 | RP | POS | 10.31 | 813.9893 | | | | 4.74E-01 | -0.13 | 0.77 | 1.05E-01 | -0.34 | 1.18 | 1.47E-01 | -0.30 | 0.73 | 8.33E-01 | 0.04 | 0.37 | 602634 | 138878 | 580614 | 182083 | 610168 | 183042 | 551060 | 178039 |
| RP_POS_770_60406a14_273 | RP | POS | 14.27 | 770.6041 | | | | 1.88E-01 | 0.22 | 0.43 | 1.05E-01 | 0.35 | 0.99 | 7.46E-02 | 0.40 | 0.64 | 7.87E-01 | 0.05 | 0.28 | 1197112 | 1237844 | 1576147 | 1836566 | 1277649 | 1412594 | 1874646 | 2153491 |
| HILIC_NEG_197_80779a0_882 | HILIC | NEG | 0.882 | 197.8078 | | | | 3.01E-01 | 0.20 | 0.82 | 1.05E-01 | 0.33 | 1.11 | 1.03E-01 | 0.37 | 0.89 | 8.59E-01 | 0.04 | 0.09 | 969561 | 178726 | 1008257 | 193836 | 976823 | 183195 | 1039692 | 200835 |
| HILIC_NEG_200_85893a7_101 | HILIC | NEG | 7.101 | 200.8589 | | | | 5.04E-02 | -0.33 | 0.75 | 1.05E-01 | 0.35 | 1.42 | 4.54E-01 | -0.15 | 0.54 | 8.40E-03 | -0.50 | 0.90 | 1819826 | 207535 | 1724135 | 312052 | 1673521 | 281201 | 1774749 | 335317 |
| RP_POS_544_33746a10_501 | RP | POS | 10.50 | 544.3375 | | | | 4.79E-03 | 0.54 | 1.04 | 1.05E-01 | 0.33 | 1.05 | 1.57E-03 | 0.71 | 1.21 | 6.66E-02 | 0.38 | 0.85 | 5894190 | 1936410 | 7086906 | 2196091 | 6730846 | 2052775 | 7442966 | 2295962 |
| RP_POS_1001_65619a10_623 | RP | POS | 10.62 | 1001.6562 | | | | 2.67E-03 | 0.56 | 1.39 | 1.05E-01 | 0.33 | 1.45 | 2.39E-03 | 0.72 | 1.27 | 2.91E-02 | 0.39 | 1.21 | 2806705 | 2110853 | 4229091 | 2605290 | 3805581 | 1730869 | 4652601 | 3217397 |
| HILIC_NEG_188_94977a1_307 | HILIC | NEG | 1.307 | 188.9498 | | | | 5.47E-04 | 0.59 | 1.18 | 1.05E-01 | 0.34 | 1.11 | 3.71E-04 | 0.76 | 1.21 | 2.18E-02 | 0.42 | 0.82 | 15857381 | 4316428 | 19414131 | 6230295 | 18404043 | 5479503 | 20424219 | 6805746 |
| RP_POS_450_16068a0_708 | RP | POS | 0.71 | 450.1607 | | | | 1.73E-01 | 0.18 | 0.49 | 1.05E-01 | 0.37 | 1.06 | 7.13E-02 | 0.36 | 0.68 | 9.84E-01 | 0.00 | 0.52 | 987667 | 263061 | 1111617 | 775887 | 985734 | 604909 | 1237500 | 904452 |
| HILIC_NEG_153_86874a7_679 | HILIC | NEG | 7.679 | 153.8687 | | | | 7.13E-01 | 0.07 | 0.18 | 1.06E-01 | 0.33 | 0.91 | 3.08E-01 | 0.24 | 0.43 | 6.89E-01 | -0.09 | 0.15 | 1666598 | 445767 | 1700152 | 450375 | 1627169 | 414733 | 1773135 | 476383 |
| RP_POS_233_07869a6_516 | RP | POS | 6.52 | 233.0787 | Sinapyl alcohol [M+Na]+ | 233.0768(100) | 3 | 1.01E-02 | 0.34 | 0.78 | 1.06E-01 | -0.36 | 0.97 | 2.17E-01 | 0.16 | 0.54 | 1.33E-02 | 0.52 | 0.88 | 1156812 | 3953516 | 4709821 | 11671789 | 6604771 | 14265482 | 2814870 | 8025542 |
| RP_POS_448_88812a0_496 | RP | POS | 0.50 | 448.8881 | | | | 4.52E-09 | -0.74 | 1.33 | 1.06E-01 | 0.35 | 1.31 | 4.60E-04 | -0.56 | 1.25 | 7.49E-07 | -0.91 | 1.58 | 2127059 | 113853 | 1797298 | 482494 | 1719158 | 498606 | 1875439 | 457475 |
| RP_POS_280_06039a1_587 | RP | POS | 1.59 | 280.0604 | | | | 7.20E-02 | 0.22 | 0.77 | 1.06E-01 | 0.37 | 1.06 | 5.99E-02 | 0.40 | 0.72 | 7.35E-01 | 0.03 | 0.44 | 1448099 | 511614 | 1943140 | 2573050 | 1525229 | 1469067 | 2361050 | 3295710 |
| RP_POS_221_0247a0_547 | RP | POS | 0.55 | 221.0247 | | | | 1.56E-03 | -0.38 | 1.07 | 1.06E-01 | 0.37 | 1.31 | 2.76E-01 | -0.20 | 0.96 | 1.51E-04 | -0.57 | 1.43 | 1028607 | 88103 | 860930 | 493541 | 781053 | 415955 | 940806 | 553261 |
| RP_POS_658_83557a0_494 | RP | POS | 0.49 | 658.8356 | | | | 2.42E-09 | -0.77 | 1.36 | 1.06E-01 | 0.35 | 1.32 | 3.55E-04 | -0.60 | 1.26 | 2.40E-07 | -0.94 | 1.60 | 2734629 | 288329 | 2000374 | 1024258 | 1834729 | 1031718 | 2166018 | 999590 |
| RP_NEG_539_25092a7_183 | RP | NEG | 7.18 | 539.2509 | | | | 4.76E-01 | -0.14 | 0.59 | 1.06E-01 | -0.33 | 1.09 | 1.94E-01 | -0.30 | 0.63 | 8.70E-01 | 0.03 | 0.43 | 1569761 | 641549 | 1472518 | 743839 | 1592938 | 590813 | 1352098 | 859785 |
| HILIC_NEG_186_04558a1_298 | HILIC | NEG | 1.298 | 186.0456 | | | | 1.71E-13 | 1.01 | 1.63 | 1.06E-01 | 0.33 | 0.96 | 4.70E-09 | 1.17 | 1.79 | 6.92E-08 | 0.84 | 1.71 | 2383530 | 984801 | 4979137 | 2618933 | 4555534 | 2241558 | 5402739 | 2909778 |
| RP_POS_385_29352a10_537 | RP | POS | 10.54 | 385.2935 | | | | 8.98E-01 | -0.03 | 0.55 | 1.07E-01 | 0.32 | 0.99 | 5.60E-01 | 0.13 | 0.54 | 4.44E-01 | -0.19 | 0.61 | 8370610 | 1555655 | 8330589 | 1437563 | 8098235 | 1569547 | 8562943 | 1265579 |
| RP_POS_555_85974a11_107 | RP | POS | 11.11 | 555.8597 | | | | 6.78E-08 | 0.65 | 1.33 | 1.07E-01 | -0.36 | 1.35 | 1.90E-04 | 0.47 | 1.39 | 5.36E-05 | 0.82 | 1.43 | 51670 | 32160 | 518235 | 798103 | 647338 | 951124 | 389133 | 590157 |
| RP_POS_295_22424a10_136 | RP | POS | 10.14 | 295.2242 | | | | 7.68E-02 | -0.28 | 0.68 | 1.07E-01 | -0.35 | 1.29 | 2.56E-02 | -0.46 | 0.98 | 5.57E-01 | -0.10 | 0.71 | 3852876 | 341199 | 3703042 | 580264 | 3796762 | 521240 | 3609323 | 625035 |
| RP_POS_536_32458a11_113 | RP | POS | 11.11 | 536.3246 | | | | 3.44E-07 | 0.61 | 1.30 | 1.07E-01 | -0.36 | 1.36 | 5.68E-04 | 0.43 | 1.35 | 1.12E-04 | 0.79 | 1.41 | 72393 | 46211 | 1392837 | 2414639 | 1783024 | 2878926 | 1002650 | 1783544 |
| RP_POS_338_198a10_867 | RP | POS | 10.87 | 338.1980 | | | | 4.87E-02 | 0.32 | 1.26 | 1.07E-01 | -0.35 | 0.99 | 4.65E-01 | 0.14 | 0.87 | 9.79E-03 | 0.49 | 1.29 | 1785414 | 370137 | 1963391 | 603515 | 2060715 | 571791 | 1866066 | 624194 |
| RP_POS_517_37042a10_461 | RP | POS | 10.46 | 517.3704 | | | | 9.09E-01 | 0.02 | 0.49 | 1.07E-01 | 0.34 | 0.96 | 3.78E-01 | 0.19 | 0.49 | 4.88E-01 | -0.15 | 0.50 | 19450963 | 1038683 | 19476328 | 1245877 | 19275538 | 1218762 | 19677118 | 1252373 |
| HILIC_POS_171_18547a5_647 | HILIC | POS | 5.647 | 171.1855 | | | | 4.26E-02 | -0.37 | 1.07 | 1.08E-01 | 0.33 | 1.52 | 3.56E-01 | -0.21 | 0.70 | 5.86E-03 | -0.54 | 1.60 | 224184131 | 17263737 | 216488245 | 21250822 | 213061843 | 17229178 | 219914647 | 24322971 |
| RP_POS_496_33978a10_646 | RP | POS | 10.65 | 496.3398 | | | | 4.50E-05 | 0.65 | 1.31 | 1.08E-01 | -0.34 | 1.00 | 7.30E-03 | 0.48 | 1.08 | 5.21E-05 | 0.82 | 1.50 | 10234301 | 11289764 | 22075214 | 19074233 | 25147330 | 20196079 | 19003097 | 17548081 |
| RP_POS_582_36597a11_116 | RP | POS | 11.12 | 582.3660 | | | | 5.11E-07 | 0.60 | 1.28 | 1.08E-01 | -0.36 | 1.35 | 1.15E-03 | 0.42 | 1.30 | 1.01E-04 | 0.78 | 1.41 | 127691 | 58370 | 554534 | 789218 | 681787 | 923794 | 427281 | 609917 |
| RP_POS_472_30124a11_141 | RP | POS | 11.14 | 472.3012 | | | | 1.76E-06 | 0.57 | 1.21 | 1.08E-01 | -0.36 | 1.31 | 4.28E-03 | 0.39 | 1.16 | 1.28E-04 | 0.75 | 1.39 | 43624 | 20328 | 405614 | 711619 | 520262 | 809964 | 290966 | 583329 |
| HILIC_POS_305_19666a1_431 | HILIC | POS | 1.431 | 305.1967 | | | | 8.32E-02 | -0.42 | 0.82 | 1.08E-01 | -0.29 | 0.92 | 2.60E-02 | -0.57 | 1.05 | 2.88E-01 | -0.28 | 0.44 | 510884 | 391506 | 378473 | 277795 | 423191 | 308009 | 333754 | 238671 |
| RP_POS_1069_71533a10_932 | RP | POS | 10.93 | 1069.7153 | | | | 2.82E-02 | -0.52 | 1.25 | 1.08E-01 | -0.29 | 1.60 | 8.89E-03 | -0.67 | 1.22 | 1.34E-01 | -0.38 | 1.03 | 5500305 | 2642197 | 4345828 | 2001128 | 4667661 | 2007644 | 4023995 | 1961819 |
| RP_POS_168_00821a15_616 | RP | POS | 15.62 | 168.0082 | | | | 2.38E-01 | 0.27 | 0.64 | 1.08E-01 | 0.30 | 1.05 | 9.51E-02 | 0.42 | 0.66 | 6.15E-01 | 0.12 | 0.70 | 13702671 | 2494703 | 14281008 | 1979330 | 13962829 | 1865326 | 14599188 | 2056549 |
| RP_POS_677_90826a10_921 | RP | POS | 10.92 | 677.9083 | | | | 1.43E-01 | 0.19 | 0.89 | 1.08E-01 | -0.37 | 1.22 | 9.74E-01 | 0.00 | 0.73 | 8.40E-02 | 0.37 | 0.98 | 814160 | 133662 | 892479 | 476036 | 969238 | 600522 | 815721 | 291880 |

**Supplementary table 1: Metabolomics results** (in asceding order according to p-value from progressive ALD vs  non-progressive ALD comparison, with molecular features with ID_level of 1-2 first)

| | | | | | | | | all_ALD_vs_control | | | Prog_ALD_vs_non-prog_ALD | | | Prog_ALD_vs_control_ALD | | | Non-prog_ALD_vs_control | | | Healthy_control | | all_ALD | | Non-prog_ALD | | Progressive_ALD | |
|---|---|---|---|---|---|---|---|---|---|---|---|---|---|---|---|---|---|---|---|---|---|---|---|---|---|---|---|
| | | | | | | | | t-test | Cohen's | PLS-DA | t-test | Cohen's | PLS-DA | t-test | Cohen's | PLS-DA | t-test | Cohen's | PLS-DA | | | | | | | | |
| Feature_ID | RP_HILIC | POS_NEG | Average.Rt | Average.Mz | Metabolite.name | curated_MSMS | ID_level | p | d | VIP | p | d | VIP | p | d | VIP | p | d | VIP | Mean | SD | Mean | SD | Mean | SD | Mean | SD |
| RP_NEG_524_33661a10_699 | RP | NEG | 10.70 | 524.3366 | | | | 3.91E-02 | -0.47 | 1.06 | 1.08E-01 | -0.30 | 1.71 | 1.31E-02 | -0.62 | 1.14 | 1.82E-01 | -0.32 | 0.86 | 9585661 | 3132750 | 8287877 | 2573437 | 8701429 | 2455992 | 7874325 | 2645634 |
| HILIC_POS_207_14926a0_708 | HILIC | POS | 0.708 | 207.1493 | | | | 1.58E-02 | -0.84 | 1.77 | 1.08E-01 | 0.08 | 1.24 | 2.19E-02 | -0.80 | 1.21 | 1.17E-02 | -0.88 | 1.50 | 2872705 | 5587394 | 345738 | 772785 | 221118 | 466862 | 470358 | 978226 |
| RP_POS_375_22675a11_049 | RP | POS | 11.05 | 375.2268 | | | | 3.13E-09 | 0.85 | 1.81 | 1.08E-01 | 0.33 | 1.11 | 4.00E-07 | 1.02 | 1.78 | 2.24E-05 | 0.68 | 1.70 | 625611 | 347128 | 1259876 | 774749 | 1135323 | 670245 | 1384429 | 855473 |
| HILIC_POS_118_65576a3_718 | HILIC | POS | 3.718 | 118.6558 | | | | 8.36E-02 | 0.29 | 0.62 | 1.09E-01 | 0.34 | 1.00 | 4.80E-02 | 0.46 | 0.81 | 4.48E-01 | 0.12 | 0.57 | 911593 | 833449 | 1249259 | 1241385 | 1049098 | 730900 | 1449419 | 1580362 |
| HILIC_POS_675_54272a0_912 | HILIC | POS | 0.912 | 675.5427 | | | | 7.31E-01 | -0.06 | 0.28 | 1.09E-01 | 0.34 | 0.97 | 6.14E-01 | 0.11 | 0.27 | 2.76E-01 | -0.23 | 0.47 | 891096 | 318800 | 867345 | 395587 | 803806 | 397662 | 930884 | 387037 |
| RP_POS_360_08688a6_516 | RP | POS | 6.52 | 360.0869 | | | | 8.56E-03 | 0.35 | 0.80 | 1.09E-01 | -0.36 | 0.97 | 1.94E-01 | 0.17 | 0.56 | 1.18E-02 | 0.53 | 0.90 | 119387 | 404535 | 483633 | 1161703 | 670561 | 1412623 | 296704 | 812302 |
| RP_POS_545_40076a10_878 | RP | POS | 10.88 | 545.4008 | | | | 4.82E-01 | -0.16 | 0.70 | 1.09E-01 | 0.30 | 1.45 | 9.74E-01 | -0.01 | 0.67 | 2.23E-01 | -0.31 | 0.73 | 23173273 | 1449003 | 22972852 | 1188787 | 22781908 | 1331003 | 23163795 | 1004636 |
| HILIC_POS_147_14906a5_856 | HILIC | POS | 5.856 | 147.1491 | | | | 6.37E-01 | 0.10 | 0.39 | 1.09E-01 | 0.32 | 1.05 | 2.92E-01 | 0.26 | 0.57 | 7.48E-01 | -0.07 | 0.35 | 55612486 | 53867460 | 60828521 | 54875956 | 52007982 | 41437779 | 69649061 | 64871633 |
| HILIC_NEG_197_80795a4_003 | HILIC | NEG | 4.003 | 197.8080 | | | | 2.24E-01 | 0.21 | 0.77 | 1.09E-01 | -0.34 | 1.16 | 8.54E-01 | 0.04 | 0.27 | 5.39E-02 | 0.38 | 1.05 | 726060 | 660353 | 909259 | 933375 | 1059040 | 875229 | 759478 | 973832 |
| RP_NEG_251_092a7_386 | RP | NEG | 7.39 | 251.0920 | | | | 8.74E-01 | -0.03 | 0.39 | 1.09E-01 | -0.33 | 0.98 | 3.77E-01 | -0.19 | 0.38 | 5.58E-01 | 0.13 | 0.41 | 607271 | 30059 | 606280 | 31971 | 611408 | 32480 | 601152 | 30929 |
| RP_POS_346_00806a0_743 | RP | POS | 0.74 | 346.0081 | | | | 2.90E-02 | -0.38 | 1.00 | 1.10E-01 | 0.34 | 1.39 | 3.01E-01 | -0.21 | 0.94 | 6.29E-03 | -0.55 | 1.09 | 23739514 | 10575179 | 18458768 | 14572699 | 16124518 | 13885693 | 20793018 | 15004291 |
| HILIC_NEG_134_89471a4_07 | HILIC | NEG | 4.07 | 134.8947 | | | | 1.54E-01 | -0.32 | 0.97 | 1.10E-01 | 0.30 | 1.31 | 4.78E-01 | -0.17 | 0.35 | 5.27E-02 | -0.48 | 1.13 | 21863024 | 10881315 | 18788428 | 8897383 | 17364497 | 8551892 | 20212358 | 9092285 |
| RP_POS_919_54797a10_838 | RP | POS | 10.84 | 919.5480 | | | | 3.47E-06 | 0.56 | 1.18 | 1.10E-01 | -0.36 | 1.31 | 1.44E-03 | 0.38 | 1.25 | 3.76E-04 | 0.74 | 1.33 | 529451 | 110023 | 1000509 | 942061 | 1151586 | 1145566 | 849432 | 658914 |
| RP_POS_594_41913a10_832 | RP | POS | 10.83 | 594.4191 | | | | 1.20E-10 | 1.18 | 2.02 | 1.10E-01 | 0.29 | 1.51 | 5.71E-10 | 1.33 | 2.10 | 4.38E-08 | 1.04 | 1.94 | 189834 | 151912 | 442709 | 194335 | 411607 | 171903 | 473810 | 211608 |
| RP_POS_633_40338a15_1 | RP | POS | 15.10 | 633.4034 | | | | 1.73E-01 | 0.28 | 0.65 | 1.10E-01 | -0.32 | 0.96 | 6.19E-01 | 0.12 | 0.26 | 4.40E-02 | 0.44 | 0.82 | 4350292 | 1305794 | 4718589 | 1332497 | 4931861 | 1153766 | 4505316 | 1470827 |
| HILIC_NEG_199_80501a4_007 | HILIC | NEG | 4.007 | 199.8050 | | | | 1.81E-01 | 0.23 | 0.91 | 1.10E-01 | -0.34 | 1.10 | 7.68E-01 | 0.06 | 0.27 | 4.81E-02 | 0.40 | 1.16 | 329270 | 319805 | 427261 | 454481 | 499937 | 448955 | 354585 | 452750 |
| RP_POS_1076_79224a0_49 | RP | POS | 0.49 | 1076.7922 | | | | 2.66E-06 | 0.57 | 1.11 | 1.10E-01 | -0.35 | 1.19 | 1.66E-02 | 0.40 | 0.92 | 1.63E-05 | 0.75 | 1.47 | 2214488 | 120126 | 2572487 | 692726 | 2683240 | 683352 | 2461734 | 691035 |
| RP_NEG_481_28101a9_356 | RP | NEG | 9.36 | 481.2810 | | | | 8.06E-03 | 0.31 | 0.52 | 1.11E-01 | 0.37 | 0.96 | 2.87E-02 | 0.49 | 0.74 | 3.70E-02 | 0.13 | 0.66 | 1174364 | 1128661 | 5484751 | 15823248 | 2941011 | 5671407 | 8028491 | 21459028 |
| RP_POS_281_24756a9_997 | RP | POS | 10.00 | 281.2476 | | | | 3.62E-07 | 0.77 | 1.46 | 1.11E-01 | 0.33 | 1.45 | 1.72E-05 | 0.93 | 1.46 | 5.88E-05 | 0.60 | 1.58 | 991069 | 494184 | 1698590 | 964960 | 1544211 | 682916 | 1852969 | 1168883 |
| RP_POS_553_45825a13_806 | RP | POS | 13.81 | 553.4583 | | | | 6.89E-03 | -0.60 | 1.09 | 1.11E-01 | -0.30 | 0.94 | 1.95E-03 | -0.75 | 1.20 | 5.60E-02 | -0.45 | 1.05 | 14623239 | 2555892 | 13201447 | 2207487 | 13553771 | 2201177 | 12849123 | 2178747 |
| RP_POS_577_87262a11_102 | RP | POS | 11.10 | 577.8726 | | | | 7.25E-07 | 0.59 | 1.28 | 1.11E-01 | -0.36 | 1.34 | 9.99E-04 | 0.41 | 1.32 | 1.50E-04 | 0.77 | 1.39 | 81595 | 39735 | 478161 | 746831 | 597426 | 886582 | 358895 | 558426 |
| RP_POS_273_05969a7_721 | RP | POS | 7.72 | 273.0597 | | | | 6.78E-01 | 0.06 | 0.65 | 1.11E-01 | 0.36 | 1.17 | 3.00E-01 | 0.24 | 0.86 | 1.92E-01 | -0.13 | 0.90 | 23348290 | 20731570 | 26197901 | 57813256 | 16922804 | 22734198 | 35472997 | 77849514 |
| RP_POS_715_5744a12_604 | RP | POS | 12.60 | 715.5744 | | | | 4.99E-12 | -1.56 | 2.66 | 1.12E-01 | -0.22 | 0.94 | 7.31E-13 | -1.67 | 2.62 | 1.05E-10 | -1.44 | 2.32 | 869544 | 290071 | 344980 | 237896 | 382877 | 246229 | 307082 | 225353 |
| RP_POS_468_35709a11_238 | RP | POS | 11.24 | 468.3571 | | | | 3.52E-01 | -0.17 | 0.69 | 1.12E-01 | -0.33 | 1.08 | 1.24E-01 | -0.34 | 0.73 | 9.73E-01 | -0.01 | 0.18 | 2114142 | 801859 | 1952829 | 972405 | 2107725 | 893951 | 1797932 | 1030693 |
| RP_NEG_929_10693a9_374 | RP | NEG | 9.37 | 929.1069 | | | | 4.98E-01 | -0.14 | 0.39 | 1.12E-01 | -0.32 | 1.07 | 1.82E-01 | -0.30 | 0.59 | 9.14E-01 | 0.02 | 0.28 | 1469982 | 124205 | 1452620 | 128641 | 1473105 | 131823 | 1432134 | 123293 |
| RP_POS_599_46405a11_609 | RP | POS | 11.61 | 599.4641 | | | | 4.88E-01 | -0.11 | 0.48 | 1.12E-01 | -0.35 | 0.98 | 3.57E-02 | -0.28 | 0.84 | 7.75E-01 | 0.07 | 0.53 | 1379211 | 1092386 | 1189560 | 1918036 | 1496370 | 2538261 | 882750 | 893269 |
| RP_POS_293_20859a10_29 | RP | POS | 10.29 | 293.2086 | | | | 6.26E-01 | -0.09 | 0.81 | 1.12E-01 | 0.34 | 1.28 | 6.98E-01 | 0.08 | 0.66 | 1.93E-01 | -0.25 | 0.76 | 2670722 | 335517 | 2634167 | 452111 | 2562247 | 406014 | 2706087 | 487419 |
| RP_POS_237_14825a8_618 | RP | POS | 8.62 | 237.1483 | | | | 2.32E-01 | 0.24 | 1.00 | 1.12E-01 | 0.32 | 1.08 | 1.09E-01 | 0.40 | 0.77 | 6.97E-01 | 0.07 | 0.61 | 742645 | 648056 | 904376 | 693667 | 793805 | 443506 | 1014947 | 866325 |
| RP_POS_609_15997a9_21 | RP | POS | 9.21 | 609.1600 | | | | 1.63E-03 | -0.63 | 1.03 | 1.13E-01 | -0.31 | 1.18 | 2.86E-04 | -0.78 | 1.32 | 3.58E-02 | -0.47 | 1.14 | 2116117 | 125005 | 2030622 | 133281 | 2051805 | 144157 | 2009439 | 119136 |
| RP_POS_307_06067a8_02 | RP | POS | 8.02 | 307.0607 | Unknown | 73.0653(100), 307.1872(30) | 4 | 7.53E-02 | -0.28 | 0.79 | 1.13E-01 | 0.35 | 1.26 | 6.09E-01 | -0.10 | 0.83 | 1.00E-02 | -0.45 | 1.02 | 3431527 | 1709006 | 2665704 | 3001237 | 2188569 | 2558127 | 3142838 | 3345154 |
| RP_POS_420_7645a13_998 | RP | POS | 14.00 | 420.7645 | | | | 3.13E-01 | -0.22 | 0.71 | 1.13E-01 | 0.31 | 1.06 | 7.85E-01 | -0.06 | 0.77 | 1.18E-01 | -0.37 | 0.69 | 15027476 | 3766680 | 14264563 | 3401040 | 13724864 | 3409893 | 14804261 | 3338922 |
| RP_POS_632_37695a14_266 | RP | POS | 14.27 | 632.3770 | | | | 5.17E-01 | 0.13 | 0.63 | 1.13E-01 | -0.32 | 0.96 | 9.15E-01 | -0.02 | 0.24 | 1.92E-01 | 0.29 | 0.51 | 1530722 | 470635 | 1593000 | 465719 | 1666899 | 432133 | 1519100 | 490235 |
| HILIC_NEG_312_83682a5_419 | HILIC | NEG | 5.419 | 312.8368 | | | | 9.68E-01 | -0.01 | 0.15 | 1.13E-01 | -0.32 | 1.30 | 4.46E-01 | -0.17 | 0.41 | 5.15E-01 | 0.15 | 0.27 | 3370157 | 1061547 | 3361551 | 1077176 | 3532325 | 1146807 | 3190777 | 984689 |
| RP_POS_304_23038a8_476 | RP | POS | 8.48 | 304.2304 | | | | 2.43E-04 | 0.58 | 1.28 | 1.13E-01 | 0.34 | 0.96 | 5.03E-04 | 0.75 | 1.31 | 1.12E-02 | 0.42 | 0.99 | 685633 | 313528 | 979596 | 533337 | 894964 | 413455 | 1064229 | 623810 |
| RP_NEG_630_3031a10_042 | RP | NEG | 10.04 | 630.3031 | | | | 1.88E-02 | 0.40 | 0.91 | 1.14E-01 | -0.34 | 0.95 | 2.53E-01 | 0.23 | 0.73 | 4.21E-03 | 0.57 | 1.17 | 815795 | 504043 | 1093945 | 740679 | 1211315 | 709317 | 976576 | 759712 |
| HILIC_NEG_217_87839a0_882 | HILIC | NEG | 0.882 | 217.8784 | | | | 9.42E-02 | 0.32 | 1.00 | 1.14E-01 | 0.32 | 1.15 | 3.28E-02 | 0.49 | 0.93 | 4.34E-01 | 0.16 | 0.35 | 649739 | 147253 | 702153 | 164602 | 676118 | 148983 | 728188 | 176525 |
| RP_POS_788_60583a14_241 | RP | POS | 14.24 | 788.6058 | | | | 2.45E-01 | -0.24 | 0.60 | 1.14E-01 | 0.31 | 0.97 | 7.06E-01 | -0.09 | 0.44 | 8.13E-02 | -0.40 | 0.76 | 2232088 | 1528152 | 1870725 | 1460991 | 1639691 | 1392513 | 2101759 | 1504835 |
| RP_POS_245_14952a4_597 | RP | POS | 4.60 | 245.1495 | | | | 4.62E-06 | 0.55 | 1.13 | 1.14E-01 | 0.35 | 1.00 | 9.90E-04 | 0.73 | 1.14 | 3.05E-05 | 0.37 | 1.34 | 233210 | 180532 | 946115 | 1442586 | 716965 | 721719 | 1175265 | 1891174 |
| RP_POS_417_24396a12_686 | RP | POS | 12.69 | 417.2440 | | | | 2.76E-03 | 0.58 | 1.19 | 1.14E-01 | -0.32 | 0.98 | 4.90E-02 | 0.42 | 0.88 | 7.36E-04 | 0.74 | 1.26 | 712952 | 304575 | 912725 | 344069 | 967104 | 338289 | 858345 | 344540 |
| RP_POS_125_03907a15_514 | RP | POS | 15.51 | 125.0391 | | | | 4.61E-01 | -0.14 | 0.44 | 1.15E-01 | 0.33 | 0.94 | 8.98E-01 | 0.03 | 0.34 | 1.89E-01 | -0.30 | 0.54 | 14902573 | 1863810 | 14608517 | 2200533 | 14260788 | 2514291 | 14956245 | 1793066 |
| HILIC_NEG_112_9857a2_171 | HILIC | NEG | 2.171 | 112.9857 | | | | 6.12E-01 | 0.10 | 0.38 | 1.15E-01 | 0.32 | 1.17 | 2.43E-01 | 0.26 | 0.48 | 8.03E-01 | -0.06 | 0.48 | 3930556 | 1198134 | 4055348 | 1228689 | 3861443 | 1248000 | 4249253 | 1189928 |
| RP_NEG_714_84039a0_495 | RP | NEG | 0.50 | 714.8404 | | | | 1.01E-07 | -0.73 | 1.30 | 1.15E-01 | 0.34 | 1.30 | 2.15E-03 | -0.57 | 1.14 | 1.77E-07 | -0.90 | 1.58 | 2380546 | 229585 | 1977972 | 584967 | 1885661 | 532876 | 2070282 | 624506 |
| HILIC_POS_235_18036a0_535 | HILIC | POS | 0.535 | 235.1804 | | | | 1.39E-02 | -0.88 | 1.77 | 1.15E-01 | 0.00 | 0.92 | 1.41E-02 | -0.88 | 1.32 | 1.37E-02 | -0.88 | 1.41 | 33006849 | 71409518 | 84725 | 459935 | 11593 | 35248 | 157857 | 644392 |
| RP_NEG_240_97899a6_484 | RP | NEG | 6.48 | 240.9790 | | | | 1.13E-01 | 0.18 | 0.73 | 1.16E-01 | -0.36 | 0.99 | 1.81E-01 | 0.00 | 0.80 | 1.15E-01 | 0.37 | 0.72 | 44592 | 19592 | 2831971 | 17444377 | 5604450 | 24477141 | 59493 | 73861 |
| RP_NEG_637_14557a9_481 | RP | NEG | 9.48 | 637.1456 | | | | 8.37E-01 | -0.04 | 0.50 | 1.16E-01 | -0.31 | 1.02 | 3.71E-01 | -0.20 | 0.45 | 6.25E-01 | 0.11 | 0.53 | 6927991 | 420896 | 6910437 | 412869 | 6975487 | 438645 | 6845386 | 378623 |
| RP_NEG_371_22076a10_941 | RP | NEG | 10.94 | 371.2208 | | | | 2.34E-07 | 0.88 | 1.69 | 1.16E-01 | 0.31 | 1.00 | 7.85E-07 | 1.04 | 1.74 | 7.65E-05 | 0.72 | 1.57 | 1673088 | 762817 | 2669430 | 1126729 | 2491884 | 1005640 | 2846976 | 1220369 |
| HILIC_NEG_113_9261a5_424 | HILIC | NEG | 5.424 | 113.9261 | | | | 1.51E-01 | 0.27 | 0.77 | 1.16E-01 | -0.32 | 1.01 | 6.10E-01 | 0.11 | 0.18 | 4.31E-02 | 0.43 | 1.10 | 3303838 | 702460 | 3520038 | 817824 | 3648744 | 794744 | 3391332 | 828190 |
| RP_POS_166_98488a10_776 | RP | POS | 10.78 | 166.9849 | | | | 8.30E-01 | -0.05 | 0.55 | 1.16E-01 | 0.30 | 1.00 | 6.98E-01 | 0.10 | 0.50 | 4.15E-01 | -0.20 | 0.56 | 14631387 | 6529640 | 14358015 | 5206467 | 13538658 | 4683002 | 15177372 | 5609564 |
| RP_POS_617_47437a11_256 | RP | POS | 11.26 | 617.4744 | | | | 8.55E-01 | 0.03 | 0.46 | 1.16E-01 | -0.34 | 1.05 | 4.27E-01 | -0.14 | 0.51 | 3.80E-01 | 0.20 | 0.74 | 1450735 | 1401467 | 1509102 | 1984468 | 1821856 | 2398162 | 1196348 | 1416261 |
| RP_POS_387_18262a11_049 | RP | POS | 11.05 | 387.1826 | | | | 3.84E-09 | 0.83 | 1.77 | 1.16E-01 | 0.33 | 1.08 | 3.74E-07 | 1.00 | 1.77 | 4.69E-05 | 0.67 | 1.65 | 1161082 | 652114 | 2376282 | 1523692 | 2136606 | 1372079 | 2615959 | 1640372 |
| HILIC_POS_240_10188a1_756 | HILIC | POS | 1.756 | 240.1019 | | | | 4.75E-01 | -0.16 | 0.26 | 1.17E-01 | -0.30 | 0.89 | 1.85E-01 | -0.31 | 0.53 | 9.82E-01 | -0.01 | 0.01 | 1986892 | 1045798 | 1838364 | 910772 | 1981549 | 990753 | 1695179 | 807779 |
| RP_NEG_199_80457a1_457 | RP | NEG | 1.46 | 199.8046 | | | | 4.93E-01 | 0.13 | 0.47 | 1.17E-01 | -0.33 | 0.98 | 8.53E-01 | -0.04 | 0.40 | 1.66E-01 | 0.29 | 0.60 | 8816352 | 2741794 | 9225380 | 3415146 | 9762049 | 3325647 | 8688710 | 3452066 |
| RP_POS_149_01227a15_342 | RP | POS | 15.34 | 149.0123 | | | | 8.61E-01 | -0.04 | 0.65 | 1.17E-01 | -0.31 | 0.96 | 3.86E-01 | -0.19 | 0.67 | 6.16E-01 | 0.12 | 0.90 | 190942748 | 71086866 | 188413996 | 68666645 | 199205112 | 74656486 | 177622879 | 60952461 |
| RP_POS_780_58868a13_637 | RP | POS | 13.64 | 780.5887 | | | | 1.15E-02 | -0.52 | 0.86 | 1.17E-01 | -0.31 | 0.96 | 2.45E-03 | -0.67 | 1.20 | 1.14E-01 | -0.36 | 0.58 | 1714592 | 675559 | 1354250 | 683792 | 1461615 | 733546 | 1246885 | 618928 |
| RP_POS_121_0172a15_338 | RP | POS | 15.34 | 121.0172 | | | | 2.21E-01 | -0.29 | 1.15 | 1.17E-01 | 0.29 | 0.94 | 5.63E-01 | -0.15 | 0.87 | 8.68E-02 | -0.43 | 0.94 | 2907504 | 1260011 | 2606612 | 948462 | 2457805 | 914453 | 2755419 | 967526 |
| RP_POS_365_23203a7_183 | RP | POS | 7.18 | 365.2320 | | | | 4.86E-01 | -0.13 | 0.51 | 1.17E-01 | -0.33 | 1.06 | 2.04E-01 | -0.29 | 0.58 | 8.65E-01 | 0.03 | 0.33 | 721991 | 285143 | 679410 | 339944 | 732784 | 271696 | 626037 | 392238 |
| RP_POS_165_05441a0_709 | RP | POS | 0.71 | 165.0544 | | | | 3.77E-01 | 0.15 | 0.91 | 1.18E-01 | 0.34 | 1.07 | 1.47E-01 | 0.32 | 1.16 | 9.29E-01 | -0.02 | 0.56 | 12752949 | 2829112 | 13323843 | 4025904 | 12692267 | 3273356 | 13955419 | 4606264 |
| RP_POS_505_27695a9_335 | RP | POS | 9.34 | 505.2770 | | | | 1.36E-02 | 0.29 | 0.49 | 1.18E-01 | 0.36 | 0.94 | 3.93E-02 | 0.47 | 0.70 | 4.18E-02 | 0.11 | 0.65 | 481550 | 495811 | 2646755 | 8576514 | 1293059 | 2681531 | 4000452 | 11733880 |
| RP_POS_366_06021a0_734 | RP | POS | 0.73 | 366.0602 | | | | 1.44E-06 | 0.59 | 1.00 | 1.18E-01 | 0.35 | 0.94 | 1.44E-04 | 0.76 | 1.22 | 1.83E-03 | 0.41 | 1.02 | 321268 | 194086 | 929062 | 1144731 | 749488 | 893058 | 1108636 | 1335739 |
| RP_POS_512_33429a10_088 | RP | POS | 10.09 | 512.3343 | | | | 6.01E-01 | 0.10 | 1.43 | 1.18E-01 | -0.32 | 1.07 | 7.77E-01 | -0.06 | 0.85 | 2.32E-01 | 0.26 | 1.51 | 5258756 | 1793077 | 5456838 | 2038202 | 5775968 | 2040387 | 5137708 | 2005565 |
| RP_POS_348_28976a11_049 | RP | POS | 11.05 | 348.2898 | | | | 9.74E-10 | 0.84 | 1.75 | 1.18E-01 | 0.33 | 1.08 | 4.87E-07 | 1.01 | 1.74 | 1.45E-05 | 0.68 | 1.64 | 1151556 | 608729 | 2408167 | 1565802 | 2163000 | 1338185 | 2653335 | 1743589 |
| RP_POS_122_97486a15_402 | RP | POS | 15.40 | 122.9749 | | | | 3.08E-01 | -0.24 | 0.56 | 1.18E-01 | 0.29 | 1.47 | 7.25E-01 | -0.09 | 0.63 | 1.19E-01 | -0.38 | 0.80 | 10217472 | 5164521 | 9184835 | 4114393 | 8541253 | 3786455 | 9828417 | 4361122 |
| RP_POS_318_909a15_426 | RP | POS | 15.43 | 318.9090 | | | | 7.60E-01 | 0.07 | 0.55 | 1.19E-01 | -0.31 | 0.95 | 7.28E-01 | -0.09 | 0.43 | 3.25E-01 | 0.22 | 0.44 | 9452675 | 1066990 | 9517832 | 965840 | 9668971 | 771234 | 9366693 | 1115039 |
| RP_POS_301_07391a7_589 | RP | POS | 7.59 | 301.0739 | | | | 2.69E-01 | 0.15 | 0.62 | 1.19E-01 | 0.36 | 1.14 | 1.64E-01 | 0.32 | 0.82 | 6.94E-01 | -0.03 | 0.65 | 1632445 | 2180805 | 2470193 | 6494096 | 1447682 | 1872862 | 3492703 | 8919972 |
| RP_POS_478_3884a13_641 | RP | POS | 13.64 | 478.3884 | | | | 2.12E-03 | -0.60 | 1.34 | 1.19E-01 | 0.31 | 1.59 | 2.19E-02 | -0.44 | 1.03 | 1.55E-03 | -0.76 | 1.46 | 7344531 | 996452 | 6675027 | 1106985 | 6501729 | 1322560 | 6848325 | 815710 |
| HILIC_POS_176_12823a4_066 | HILIC | POS | 4.066 | 176.1282 | | | | 6.94E-05 | 0.60 | 0.98 | 1.19E-01 | 0.33 | 1.08 | 1.65E-04 | 0.77 | 1.25 | 9.72E-03 | 0.43 | 0.88 | 1748379 | 682772 | 2490651 | 1322275 | 2283937 | 1146636 | 2697365 | 1459633 |
| RP_NEG_311_16968a10_423 | RP | NEG | 10.42 | 311.1697 | | | | 8.94E-01 | -0.03 | 0.39 | 1.19E-01 | 0.30 | 0.94 | 6.22E-01 | 0.12 | 0.23 | 4.64E-01 | -0.18 | 0.39 | 11022779 | 5893225 | 10867879 | 4948067 | 10094915 | 4982408 | 11640842 | 4839698 |
| RP_NEG_1091_72742a10_933 | RP | NEG | 10.93 | 1091.7274 | | | | 4.82E-02 | -0.48 | 1.18 | 1.19E-01 | -0.28 | 1.58 | 1.89E-02 | -0.62 | 1.13 | 1.73E-01 | -0.34 | 0.96 | 10212491 | 4328049 | 8532887 | 3087939 | 9014539 | 2866276 | 8051236 | 3252131 |
| RP_POS_583_38617a14_266 | RP | POS | 14.27 | 583.3862 | | | | 3.83E-02 | -0.46 | 0.96 | 1.20E-01 | -0.29 | 1.24 | 1.22E-02 | -0.61 | 1.28 | 1.89E-01 | -0.31 | 0.62 | 3164496 | 924592 | 2776776 | 798133 | 2901156 | 794995 | 2652396 | 789585 |
| HILIC_POS_229_155a4_222 | HILIC | POS | 4.222 | 229.1550 | | | | 3.78E-01 | 0.14 | 0.82 | 1.20E-01 | 0.34 | 1.19 | 1.67E-01 | 0.31 | 0.52 | 8.40E-01 | -0.03 | 0.74 | 8512185 | 2388731 | 9031782 | 4063218 | 8397133 | 2690035 | 9666430 | 5029724 |
| RP_POS_793_55914a12_82 | RP | POS | 12.82 | 793.5591 | | | | 1.57E-06 | -1.21 | 1.97 | 1.20E-01 | -0.23 | 0.96 | 4.15E-07 | -1.33 | 2.02 | 1.59E-05 | -1.10 | 1.65 | 2398098 | 1185076 | 1139850 | 772923 | 1260191 | 790652 | 1019509 | 743187 |
| RP_POS_534_31653a10_088 | RP | POS | 10.09 | 534.3165 | | | | 4.38E-01 | 0.15 | 1.42 | 1.20E-01 | -0.32 | 1.03 | 9.51E-01 | -0.01 | 0.84 | 1.55E-01 | 0.31 | 1.52 | 819560 | 295308 | 868253 | 341197 | 921326 | 338570 | 815179 | 338863 |
| RP_POS_322_67517a10_931 | RP | POS | 10.93 | 322.6752 | | | | 3.69E-03 | -0.67 | 1.59 | 1.21E-01 | -0.28 | 1.62 | 1.22E-03 | -0.81 | 1.56 | 2.76E-02 | -0.53 | 1.27 | 1242357 | 335847 | 1042594 | 268755 | 1084355 | 259156 | 1000834 | 274248 |
| HILIC_POS_162_05009a4_852 | HILIC | POS | 4.852 | 162.0501 | | | | 8.17E-01 | -0.04 | 0.91 | 1.21E-01 | -0.33 | 1.05 | 3.25E-01 | -0.21 | 0.79 | 5.73E-01 | 0.12 | 0.59 | 2543862 | 547556 | 2516412 | 676848 | 2621552 | 689320 | 2411272 | 654121 |
| RP_POS_884_16089a0_484 | RP | POS | 0.48 | 884.1609 | | | | 4.93E-05 | 0.49 | 0.95 | 1.21E-01 | -0.35 | 1.10 | 5.78E-02 | 0.32 | 0.75 | 9.07E-05 | 0.67 | 1.34 | 278444 | 28901 | 357030 | 179046 | 384840 | 173883 | 329220 | 181535 |
| HILIC_POS_480_34457a1_11 | HILIC | POS | 1.11 | 480.3446 | | | | 2.47E-01 | -0.26 | 0.72 | 1.21E-01 | -0.29 | 1.12 | 1.01E-01 | -0.41 | 0.64 | 6.28E-01 | -0.12 | 0.19 | 4720347 | 1961947 | 4272079 | 1601963 | 4520890 | 1529435 | 4023268 | 1649070 |
| RP_POS_172_13307a10_38 | RP | POS | 10.38 | 172.1331 | | | | 3.45E-01 | -0.19 | 0.81 | 1.21E-01 | 0.31 | 0.96 | 8.92E-01 | -0.03 | 0.59 | 9.92E-02 | -0.35 | 0.93 | 11801318 | 799621 | 11645030 | 833786 | 11515451 | 675844 | 11774608 | 955799 |
| HILIC_NEG_244_98708a6_093 | HILIC | NEG | 6.093 | 244.9871 | | | | 9.49E-01 | 0.01 | 0.77 | 1.22E-01 | -0.33 | 1.03 | 4.21E-01 | -0.16 | 0.48 | 3.80E-01 | 0.18 | 1.01 | 222012 | 80076 | 223201 | 120656 | 241879 | 123767 | 204523 | 115682 |
| RP_POS_816_42133a7_549 | RP | POS | 7.55 | 816.4213 | | | | 5.52E-01 | -0.13 | 0.77 | 1.22E-01 | 0.30 | 1.04 | 9.35E-01 | 0.02 | 0.63 | 2.47E-01 | -0.28 | 0.77 | 919781 | 71081 | 911377 | 62409 | 901722 | 63378 | 921033 | 60515 |
| RP_POS_513_84448a14_671 | RP | POS | 14.67 | 513.8445 | | | | 5.27E-01 | 0.10 | 0.81 | 1.23E-01 | 0.34 | 1.02 | 2.58E-01 | 0.27 | 0.66 | 6.29E-01 | -0.07 | 0.49 | 223890 | 340002 | 275301 | 542523 | 190911 | 223492 | 359690 | 728140 |
| RP_POS_585_27002a11_94 | RP | POS | 11.94 | 585.2700 | | | | 3.33E-01 | -0.18 | 0.71 | 1.23E-01 | 0.32 | 1.30 | 9.25E-01 | -0.02 | 0.43 | 4.78E-02 | -0.34 | 0.98 | 1167939 | 1471567 | 864469 | 1699525 | 600136 | 696101 | 1128801 | 2282227 |
| RP_POS_352_24469a10_271 | RP | POS | 10.27 | 352.2447 | | | | 7.06E-01 | -0.07 | 1.00 | 1.23E-01 | 0.33 | 1.01 | 6.87E-01 | 0.09 | 0.85 | 2.10E-01 | -0.23 | 0.76 | 12371775 | 874152 | 12299938 | 1098890 | 12129848 | 791998 | 12470027 | 1324182 |
| RP_POS_166_98485a11_212 | RP | POS | 11.21 | 166.9849 | | | | 5.17E-03 | -0.52 | 1.10 | 1.23E-01 | -0.31 | 0.99 | 2.29E-03 | -0.68 | 1.20 | 6.90E-02 | -0.36 | 1.29 | 14125127 | 1025448 | 13486813 | 1252658 | 13680293 | 1122159 | 13293332 | 1354508 |
| HILIC_NEG_161_98665a0_546 | HILIC | NEG | 0.546 | 161.9867 | | | | 4.37E-01 | 0.13 | 0.32 | 1.23E-01 | 0.34 | 1.25 | 2.18E-01 | 0.30 | 0.52 | 7.81E-01 | -0.04 | 0.12 | 709323 | 660310 | 830391 | 1019729 | 672101 | 454179 | 988681 | 1357756 |
| HILIC_POS_64_01662a0_709 | HILIC | POS | 0.709 | 64.0166 | | | | 1.07E-06 | 0.74 | 1.30 | 1.23E-01 | 0.32 | 1.39 | 1.38E-05 | 0.91 | 1.52 | 3.53E-04 | 0.58 | 1.27 | 670463 | 324186 | 1105177 | 609687 | 1010972 | 501843 | 1199382 | 693589 |
| RP_NEG_676_30573a10_537 | RP | NEG | 10.54 | 676.3057 | | | | 3.16E-02 | -0.40 | 0.82 | 1.23E-01 | -0.32 | 0.95 | 9.02E-03 | -0.55 | 1.04 | 2.57E-01 | -0.24 | 0.53 | 1906713 | 111092 | 1853961 | 137975 | 1875260 | 136620 | 1832662 | 137374 |
| RP_POS_206_89355a0_519 | RP | POS | 0.52 | 206.8936 | | | | 8.63E-01 | 0.03 | 0.30 | 1.23E-01 | -0.31 | 1.04 | 5.78E-01 | -0.12 | 0.34 | 4.01E-01 | 0.19 | 0.48 | 1297026 | 612106 | 1318822 | 643141 | 1418095 | 662787 | 1219549 | 613435 |
| RP_POS_547_36115a10_678 | RP | POS | 10.68 | 547.3612 | | | | 4.71E-01 | -0.16 | 0.73 | 1.23E-01 | 0.30 | 1.12 | 9.84E-01 | -0.01 | 0.15 | 1.62E-01 | -0.31 | 1.09 | 3689349 | 1482321 | 3475262 | 1340362 | 3268043 | 978255 | 3682481 | 1607853 |

**Supplementary table 1: Metabolomics results** (in asceding order according to p-value from progressive ALD vs  non-progressive ALD comparison, with molecular features with ID_level of 1-2 first)

| | | | | | | | | all_ALD_vs_control | | | Prog_ALD_vs_non-prog_ALD | | | Prog_ALD_vs_control_ALD | | | Non-prog_ALD_vs_control | | | Healthy_control | | all_ALD | | Non-prog_ALD | | Progressive_ALD | |
|---|---|---|---|---|---|---|---|---|---|---|---|---|---|---|---|---|---|---|---|---|---|---|---|---|---|---|---|
| | | | | | | | | t-test | Cohen's | PLS-DA | t-test | Cohen's | PLS-DA | t-test | Cohen's | PLS-DA | t-test | Cohen's | PLS-DA | | | | | | | | |
| Feature_ID | RP_HILIC | POS_NEG | Average.Rt | Average.Mz | Metabolite.name | curated_MSMS | ID_level | p | d | VIP | p | d | VIP | p | d | VIP | p | d | VIP | Mean | SD | Mean | SD | Mean | SD | Mean | SD |
| HILIC_POS_229_15433a4_787 | HILIC | POS | 4.787 | 229.1543 | | | | 7.88E-01 | 0.05 | 0.81 | 1.24E-01 | 0.33 | 1.18 | 3.67E-01 | 0.21 | 0.32 | 4.47E-01 | -0.12 | 1.05 | 5307916 | 1541436 | 5403112 | 2247739 | 5055329 | 1316525 | 5750894 | 2868395 |
| RP_POS_558_33771a11_107 | RP | POS | 11.11 | 558.3377 | | | | 2.83E-07 | 0.61 | 1.31 | 1.24E-01 | -0.34 | 1.34 | 4.69E-04 | 0.44 | 1.36 | 1.12E-04 | 0.78 | 1.41 | 75788 | 58254 | 1440719 | 2474884 | 1822793 | 2940285 | 1058646 | 1852541 |
| HILIC_POS_258_0119a0_541 | HILIC | POS | 0.541 | 258.0119 | | | | 4.15E-01 | -0.11 | 0.77 | 1.24E-01 | 0.35 | 0.98 | 7.57E-01 | 0.06 | 0.33 | 5.41E-02 | -0.28 | 1.56 | 98698064 | 12746501 | 95423695 | 33132773 | 90312409 | 25787837 | 100534981 | 38725130 |
| RP_POS_586_30743a10_504 | RP | POS | 10.50 | 586.3074 | | | | 1.58E-01 | 0.29 | 0.72 | 1.24E-01 | -0.31 | 0.93 | 5.33E-01 | 0.14 | 0.57 | 5.77E-02 | 0.44 | 0.82 | 3867085 | 1008154 | 4159591 | 994886 | 4312863 | 1038330 | 4006319 | 934733 |
| HILIC_NEG_59_01299a0_701 | HILIC | NEG | 0.701 | 59.0130 | | | | 4.93E-04 | 0.41 | 0.84 | 1.24E-01 | 0.35 | 1.26 | 1.01E-02 | 0.59 | 0.84 | 2.58E-05 | 0.24 | 1.35 | 468062 | 246056 | 1316304 | 2318313 | 956685 | 698787 | 1675923 | 3179089 |
| HILIC_NEG_100_93345a1_051 | HILIC | NEG | 1.051 | 100.9335 | | | | 2.31E-02 | 0.42 | 1.02 | 1.24E-01 | 0.32 | 1.12 | 6.53E-03 | 0.58 | 1.32 | 2.16E-01 | 0.26 | 0.44 | 13905230 | 4853127 | 16352044 | 6075711 | 15416294 | 6040369 | 17287794 | 6025681 |
| RP_POS_272_18582a7_728 | RP | POS | 7.73 | 272.1858 | | | | 1.77E-05 | -0.65 | 1.17 | 1.24E-01 | -0.33 | 1.21 | 1.60E-04 | -0.82 | 1.43 | 1.37E-03 | -0.49 | 1.03 | 6052530 | 359574 | 5632824 | 680543 | 5737810 | 497772 | 5527839 | 815757 |
| RP_NEG_193_81508a15_583 | RP | NEG | 15.58 | 193.8151 | | | | 6.90E-02 | -0.37 | 0.70 | 1.24E-01 | -0.31 | 0.99 | 1.71E-02 | -0.52 | 1.02 | 3.54E-01 | -0.21 | 0.38 | 1702188 | 67110 | 1676708 | 69120 | 1687354 | 74714 | 1666062 | 61962 |
| RP_POS_633_25494a8_499 | RP | POS | 8.50 | 633.2549 | | | | 8.86E-01 | -0.02 | 0.65 | 1.24E-01 | 0.34 | 1.30 | 5.24E-01 | 0.15 | 0.54 | 2.10E-01 | -0.19 | 0.74 | 2576715 | 1411292 | 2527597 | 2321786 | 2168983 | 1439462 | 2886212 | 2925216 |
| HILIC_POS_154_09747a1_969 | HILIC | POS | 1.969 | 154.0975 | | | | 3.22E-02 | 0.29 | 0.84 | 1.24E-01 | -0.35 | 0.88 | 2.34E-01 | 0.12 | 0.65 | 4.44E-02 | 0.46 | 0.94 | 361051 | 665532 | 828810 | 1811711 | 1109276 | 2438220 | 548343 | 725347 |
| RP_POS_728_52191a12_215 | RP | POS | 12.22 | 728.5219 | | | | 6.32E-02 | 0.33 | 0.82 | 1.25E-01 | 0.32 | 1.10 | 2.80E-02 | 0.49 | 0.89 | 3.77E-01 | 0.16 | 0.39 | 1023614 | 830164 | 1373968 | 1136400 | 1199035 | 932801 | 1548902 | 1294834 |
| RP_POS_421_35245a10_708 | RP | POS | 10.71 | 421.3525 | | | | 8.69E-01 | -0.03 | 0.56 | 1.25E-01 | -0.32 | 0.97 | 3.81E-01 | -0.19 | 0.50 | 5.71E-01 | 0.13 | 0.44 | 7300226 | 596824 | 7279543 | 661257 | 7381212 | 675565 | 7177875 | 637152 |
| RP_POS_485_81409a2_762 | RP | POS | 2.76 | 485.8141 | | | | 3.16E-01 | -0.24 | 0.47 | 1.25E-01 | -0.28 | 1.24 | 1.19E-01 | -0.38 | 0.68 | 7.01E-01 | -0.10 | 0.39 | 1140209 | 1217081 | 904674 | 873789 | 1039266 | 1056722 | 770082 | 623669 |
| RP_POS_271_24191a9_334 | RP | POS | 9.33 | 271.2419 | | | | 1.29E-02 | 0.29 | 0.49 | 1.25E-01 | 0.35 | 0.92 | 3.95E-02 | 0.47 | 0.69 | 3.71E-02 | 0.11 | 0.66 | 420791 | 438798 | 2292526 | 7350750 | 1155584 | 2368438 | 3429467 | 10046006 |
| RP_POS_210_01862a15_425 | RP | POS | 15.43 | 210.0186 | | | | 7.64E-01 | 0.06 | 0.51 | 1.25E-01 | -0.30 | 1.08 | 7.08E-01 | -0.09 | 0.37 | 3.61E-01 | 0.22 | 0.82 | 1956754 | 523354 | 1988230 | 485504 | 2062811 | 486076 | 1913650 | 478139 |
| HILIC_NEG_219_8756a0_881 | HILIC | NEG | 0.881 | 219.8756 | | | | 1.15E-01 | 0.31 | 0.99 | 1.26E-01 | 0.31 | 1.14 | 4.10E-02 | 0.47 | 0.90 | 4.61E-01 | 0.16 | 0.34 | 205690 | 50564 | 222415 | 53870 | 214147 | 50099 | 230682 | 56690 |
| HILIC_POS_146_11757a4_67 | HILIC | POS | 4.67 | 146.1176 | | | | 5.67E-03 | 0.37 | 0.78 | 1.26E-01 | 0.34 | 1.19 | 9.84E-03 | 0.54 | 0.90 | 1.38E-01 | 0.20 | 0.56 | 700690 | 812802 | 1466362 | 2311427 | 1110999 | 1649394 | 1821725 | 2795748 |
| HILIC_POS_178_05862a6_779 | HILIC | POS | 6.779 | 178.0586 | | | | 4.96E-02 | 0.28 | 1.03 | 1.26E-01 | -0.34 | 1.11 | 4.71E-01 | 0.11 | 1.02 | 2.80E-02 | 0.45 | 1.19 | 1085864 | 263719 | 1233826 | 582137 | 1323215 | 670997 | 1144438 | 467055 |
| HILIC_POS_230_0956a5_436 | HILIC | POS | 5.436 | 230.0956 | | | | 2.40E-02 | 0.40 | 0.91 | 1.26E-01 | 0.32 | 0.94 | 8.47E-03 | 0.56 | 1.12 | 2.27E-01 | 0.24 | 0.49 | 316289 | 439415 | 541762 | 592735 | 450834 | 555147 | 632691 | 620306 |
| HILIC_POS_140_06812a5_459 | HILIC | POS | 5.459 | 140.0681 | | | | 3.57E-01 | 0.17 | 0.38 | 1.26E-01 | 0.32 | 0.92 | 1.30E-01 | 0.33 | 0.72 | 9.41E-01 | 0.02 | 0.32 | 988014 | 94228 | 1006566 | 110211 | 989672 | 105024 | 1023461 | 113702 |
| RP_POS_1074_69202a10_931 | RP | POS | 10.93 | 1074.6920 | | | | 5.22E-03 | -0.65 | 1.28 | 1.26E-01 | -0.28 | 1.46 | 1.51E-03 | -0.79 | 1.35 | 3.87E-02 | -0.51 | 1.09 | 585895 | 321719 | 402635 | 255884 | 441859 | 264305 | 363411 | 243501 |
| RP_POS_295_11246a7_093 | RP | POS | 7.09 | 295.1125 | | | | 4.14E-01 | 0.16 | 0.64 | 1.26E-01 | 0.32 | 1.03 | 1.57E-01 | 0.31 | 0.65 | 9.89E-01 | 0.00 | 0.96 | 770225 | 57144 | 780147 | 65938 | 770045 | 62404 | 790248 | 68425 |
| RP_POS_167_02266a15_338 | RP | POS | 15.34 | 167.0227 | | | | 1.53E-01 | -0.32 | 1.15 | 1.26E-01 | 0.29 | 1.01 | 4.69E-01 | -0.18 | 0.89 | 5.55E-02 | -0.47 | 0.99 | 23081470 | 9369949 | 20420012 | 7837976 | 19219431 | 7609152 | 21620592 | 7954915 |
| HILIC_NEG_187_04176a1_299 | HILIC | NEG | 1.299 | 187.0418 | | | | 4.30E-12 | 0.95 | 1.60 | 1.27E-01 | 0.31 | 0.96 | 2.28E-08 | 1.11 | 1.72 | 5.22E-07 | 0.80 | 1.60 | 9651460 | 4503715 | 20458260 | 11641575 | 18676425 | 10131887 | 22240095 | 12832918 |
| RP_POS_353_24484a11_049 | RP | POS | 11.05 | 353.2448 | | | | 7.29E-10 | 0.85 | 1.79 | 1.27E-01 | 0.32 | 1.08 | 4.38E-07 | 1.01 | 1.75 | 1.16E-05 | 0.69 | 1.70 | 6349153 | 3285919 | 13196411 | 8461521 | 11902115 | 7264203 | 14490708 | 9405765 |
| RP_POS_614_33923a10_934 | RP | POS | 10.93 | 614.3392 | | | | 3.18E-02 | -0.48 | 1.19 | 1.27E-01 | -0.29 | 1.49 | 1.05E-02 | -0.62 | 1.16 | 1.60E-01 | -0.33 | 0.99 | 2891669 | 728104 | 2575052 | 623451 | 2670335 | 617151 | 2479768 | 621217 |
| HILIC_NEG_165_04167a0_799 | HILIC | NEG | 0.799 | 165.0417 | | | | 1.59E-01 | 0.23 | 0.53 | 1.27E-01 | -0.33 | 1.11 | 7.29E-01 | 0.07 | 0.12 | 5.07E-02 | 0.40 | 0.67 | 145353 | 149570 | 195685 | 235721 | 231697 | 244513 | 159673 | 223226 |
| RP_NEG_355_26492a11_203 | RP | NEG | 11.20 | 355.2649 | FA 24:6 (Tetracosahexaenoic acid) | 355.2663(100), 116.9271(28), 99.9246(16) | 3 | 8.63E-07 | 0.63 | 1.46 | 1.27E-01 | 0.34 | 0.94 | 1.95E-04 | 0.79 | 1.32 | 1.57E-04 | 0.46 | 1.45 | 538187 | 459315 | 1539125 | 1755425 | 1270313 | 1160426 | 1807936 | 2175285 |
| RP_NEG_217_86539a15_575 | RP | NEG | 15.58 | 217.8654 | | | | 3.47E-01 | -0.20 | 0.47 | 1.27E-01 | -0.30 | 1.27 | 1.36E-01 | -0.35 | 0.68 | 8.38E-01 | -0.05 | 0.35 | 1066200 | 269724 | 1014761 | 256532 | 1053938 | 252602 | 975584 | 256944 |
| RP_POS_279_07339a15_387 | RP | POS | 15.39 | 279.0734 | | | | 6.37E-01 | 0.09 | 0.36 | 1.27E-01 | -0.31 | 1.02 | 7.41E-01 | -0.07 | 0.16 | 2.86E-01 | 0.25 | 0.59 | 1930372 | 1035806 | 2033341 | 1170381 | 2212200 | 1326526 | 1854482 | 970866 |
| RP_POS_1075_69495a10_932 | RP | POS | 10.93 | 1075.6950 | | | | 1.50E-02 | -0.61 | 1.27 | 1.28E-01 | -0.26 | 1.47 | 5.29E-03 | -0.74 | 1.27 | 6.57E-02 | -0.48 | 1.03 | 660578 | 434923 | 451824 | 289571 | 496023 | 294199 | 407625 | 280844 |
| HILIC_POS_203_1212a0_524 | HILIC | POS | 0.524 | 203.1212 | | | | 2.85E-01 | 0.21 | 0.55 | 1.28E-01 | -0.31 | 0.92 | 7.65E-01 | 0.06 | 0.14 | 1.39E-01 | 0.37 | 0.68 | 255082 | 222763 | 304542 | 234889 | 340481 | 292600 | 268602 | 152386 |
| RP_POS_1017_68555a10_616 | RP | POS | 10.62 | 1017.6856 | | | | 6.57E-03 | 0.53 | 1.29 | 1.28E-01 | -0.30 | 1.25 | 8.98E-02 | 0.38 | 0.94 | 1.30E-03 | 0.69 | 1.39 | 1455219 | 883875 | 1974126 | 969663 | 2122096 | 864493 | 1826156 | 1052450 |
| RP_POS_1045_62329a10_315 | RP | POS | 10.32 | 1045.6233 | | | | 4.73E-02 | 0.36 | 0.99 | 1.28E-01 | -0.32 | 1.14 | 3.51E-01 | 0.20 | 0.58 | 1.14E-02 | 0.51 | 1.07 | 3075367 | 1295286 | 3650171 | 1689215 | 3907572 | 1590415 | 3392770 | 1760822 |
| RP_POS_499_43591a13_618 | RP | POS | 13.62 | 499.4359 | | | | 3.39E-01 | 0.14 | 0.40 | 1.28E-01 | 0.34 | 1.01 | 1.60E-01 | 0.31 | 0.53 | 8.01E-01 | -0.03 | 0.35 | 855914 | 117600 | 888125 | 263017 | 847939 | 168256 | 928311 | 328879 |
| RP_POS_1046_84192a14_258 | RP | POS | 14.26 | 1046.8419 | | | | 4.70E-01 | 0.14 | 0.35 | 1.28E-01 | 0.31 | 0.96 | 1.92E-01 | 0.30 | 0.75 | 9.59E-01 | -0.01 | 0.33 | 496434 | 409593 | 557803 | 434292 | 491640 | 407617 | 623965 | 453854 |
| HILIC_POS_394_31644a0_891 | HILIC | POS | 0.891 | 394.3164 | | | | 8.42E-01 | -0.04 | 0.62 | 1.29E-01 | 0.31 | 1.23 | 6.28E-01 | 0.12 | 0.19 | 3.53E-01 | -0.19 | 0.38 | 2933879 | 679093 | 2905943 | 714667 | 2797041 | 588117 | 3014844 | 813534 |
| RP_POS_329_24478a11_128 | RP | POS | 11.13 | 329.2448 | | | | 1.66E-07 | 0.81 | 1.82 | 1.29E-01 | 0.31 | 1.16 | 1.83E-06 | 0.96 | 1.76 | 1.62E-04 | 0.65 | 1.69 | 1591385 | 849142 | 2812502 | 1561865 | 2574684 | 1398571 | 3050320 | 1690328 |
| RP_NEG_426_96826a11_162 | RP | NEG | 11.16 | 426.9683 | | | | 1.18E-01 | 0.26 | 0.92 | 1.29E-01 | -0.32 | 0.93 | 5.97E-01 | 0.10 | 0.30 | 3.90E-02 | 0.43 | 1.21 | 21321867 | 6944365 | 23839041 | 10161224 | 25385449 | 10591367 | 22292633 | 9568211 |
| RP_NEG_339_21582a10_713 | RP | NEG | 10.71 | 339.2158 | | | | 2.23E-02 | 0.37 | 1.17 | 1.29E-01 | 0.33 | 1.15 | 1.40E-02 | 0.53 | 1.15 | 2.29E-01 | 0.20 | 1.09 | 1237623 | 165399 | 1331079 | 275503 | 1289114 | 217579 | 1373045 | 320028 |
| RP_POS_416_26096a10_543 | RP | POS | 10.54 | 416.2610 | | | | 1.09E-05 | 0.75 | 1.27 | 1.29E-01 | -0.31 | 1.05 | 4.49E-03 | 0.59 | 1.02 | 1.41E-06 | 0.90 | 1.68 | 418321 | 210159 | 646114 | 310637 | 693407 | 262621 | 598822 | 348463 |
| RP_NEG_177_95622a13_287 | RP | NEG | 13.29 | 177.9562 | | | | 2.94E-01 | -0.25 | 0.66 | 1.29E-01 | 0.27 | 1.04 | 6.61E-01 | -0.12 | 0.37 | 1.19E-01 | -0.39 | 0.90 | 926450 | 202821 | 885340 | 146140 | 863092 | 122741 | 907589 | 164543 |
| RP_POS_1048_74414a0_493 | RP | POS | 0.49 | 1048.7441 | | | | 4.24E-09 | -0.78 | 1.33 | 1.29E-01 | 0.32 | 1.19 | 6.89E-04 | -0.61 | 1.24 | 4.24E-08 | -0.94 | 1.64 | 710637 | 87349 | 516158 | 267266 | 475501 | 245290 | 556816 | 284218 |
| RP_NEG_556_32709a10_087 | RP | NEG | 10.09 | 556.3271 | | | | 9.93E-01 | 0.00 | 1.31 | 1.29E-01 | -0.31 | 1.07 | 4.71E-01 | -0.16 | 0.87 | 4.91E-01 | 0.15 | 1.33 | 2768476 | 938137 | 2766769 | 1020949 | 2921972 | 1042704 | 2611567 | 984659 |
| RP_POS_970_72034a0_498 | RP | POS | 0.50 | 970.7203 | | | | 4.65E-01 | 0.14 | 0.92 | 1.29E-01 | -0.31 | 1.34 | 9.28E-01 | -0.02 | 0.67 | 1.86E-01 | 0.30 | 1.06 | 1325167 | 288488 | 1370043 | 335171 | 1420988 | 357800 | 1319099 | 306029 |
| RP_POS_246_17001a2_848 | RP | POS | 2.85 | 246.1700 | | | | 8.95E-02 | 0.31 | 0.62 | 1.29E-01 | -0.32 | 1.16 | 4.60E-01 | 0.15 | 0.34 | 3.09E-02 | 0.47 | 0.84 | 1447610 | 760609 | 1733680 | 973682 | 1881614 | 1022371 | 1585747 | 908601 |
| RP_POS_262_01337a1_462 | RP | POS | 1.46 | 262.0134 | | | | 5.39E-04 | 0.41 | 0.75 | 1.30E-01 | 0.35 | 0.95 | 1.10E-02 | 0.58 | 0.85 | 3.87E-05 | 0.23 | 1.23 | 229287 | 418272 | 3313392 | 8593492 | 2000071 | 2736697 | 4626713 | 11755585 |
| RP_POS_800_74994a0_499 | RP | POS | 0.50 | 800.7499 | | | | 7.50E-01 | 0.06 | 0.57 | 1.30E-01 | -0.31 | 1.16 | 7.04E-01 | -0.09 | 0.28 | 3.26E-01 | 0.22 | 0.66 | 414499 | 212785 | 428399 | 216145 | 461185 | 201197 | 395613 | 227413 |
| RP_NEG_613_40125a11_163 | RP | NEG | 11.16 | 613.4013 | | | | 9.22E-03 | 0.43 | 1.07 | 1.30E-01 | -0.32 | 1.40 | 1.63E-01 | 0.27 | 0.85 | 3.33E-03 | 0.59 | 1.30 | 1093480 | 84131 | 1146526 | 132166 | 1166566 | 134518 | 1126485 | 127975 |
| RP_POS_388_71155a0_522 | RP | POS | 0.52 | 388.7116 | | | | 9.56E-03 | -0.43 | 0.89 | 1.30E-01 | 0.32 | 1.05 | 1.78E-01 | -0.27 | 0.68 | 2.41E-03 | -0.59 | 1.10 | 407463 | 78962 | 358259 | 122001 | 339760 | 116534 | 376758 | 125673 |
| RP_POS_559_1311a10_529 | RP | POS | 10.53 | 559.1311 | | | | 2.40E-01 | -0.28 | 1.05 | 1.30E-01 | 0.28 | 0.90 | 5.82E-01 | -0.14 | 0.64 | 9.30E-02 | -0.42 | 0.99 | 375142 | 266829 | 314194 | 196330 | 284416 | 169550 | 343973 | 217534 |
| HILIC_NEG_200_85863a4_682 | HILIC | NEG | 4.682 | 200.8586 | | | | 5.67E-01 | 0.11 | 0.25 | 1.30E-01 | 0.32 | 1.09 | 2.47E-01 | 0.26 | 0.42 | 7.81E-01 | -0.05 | 0.22 | 5627065 | 2575602 | 5946341 | 3179350 | 5464066 | 2596578 | 6428616 | 3633998 |
| RP_POS_246_98045a15_564 | RP | POS | 15.56 | 246.9805 | | | | 1.93E-01 | -0.28 | 0.55 | 1.30E-01 | 0.30 | 1.10 | 5.90E-01 | -0.13 | 0.30 | 5.99E-02 | -0.43 | 0.92 | 2676959 | 105980 | 2649067 | 97698 | 2634254 | 84658 | 2663880 | 108026 |
| RP_NEG_372_67331a15_575 | RP | NEG | 15.58 | 372.6733 | | | | 2.24E-01 | -0.26 | 0.70 | 1.30E-01 | -0.30 | 1.08 | 8.32E-02 | -0.41 | 0.84 | 6.44E-01 | -0.11 | 0.40 | 620723 | 39988 | 610831 | 38186 | 616617 | 37525 | 605045 | 38336 |
| RP_NEG_562_31647a10_041 | RP | NEG | 10.04 | 562.3165 | | | | 2.25E-02 | 0.42 | 0.94 | 1.31E-01 | -0.31 | 0.95 | 2.23E-01 | 0.26 | 0.71 | 4.67E-03 | 0.58 | 1.16 | 8359068 | 5906177 | 11336904 | 7294916 | 12442143 | 6573188 | 10231665 | 7862435 |
| RP_NEG_631_24188a8_496 | RP | NEG | 8.50 | 631.2419 | | | | 7.74E-01 | -0.05 | 0.62 | 1.31E-01 | 0.33 | 1.30 | 6.14E-01 | 0.12 | 0.50 | 1.74E-01 | -0.21 | 0.71 | 1332881 | 756377 | 1281655 | 1166249 | 1104450 | 694581 | 1458860 | 1483746 |
| RP_POS_164_9845a11_213 | RP | POS | 11.21 | 164.9845 | | | | 2.46E-04 | -0.67 | 1.20 | 1.31E-01 | -0.31 | 0.92 | 1.14E-04 | -0.82 | 1.53 | 1.08E-02 | -0.51 | 1.10 | 3476190 | 282111 | 3237044 | 363527 | 3292086 | 351473 | 3182002 | 370520 |
| RP_POS_347_18799a11_159 | RP | POS | 11.16 | 347.1880 | | | | 1.50E-01 | -0.26 | 0.82 | 1.31E-01 | 0.32 | 1.16 | 6.29E-01 | -0.10 | 0.61 | 4.98E-02 | -0.42 | 0.87 | 1745887 | 118612 | 1707746 | 156683 | 1684031 | 161885 | 1731461 | 149149 |
| HILIC_NEG_112_98563a1_307 | HILIC | NEG | 1.307 | 112.9856 | | | | 1.11E-01 | 0.32 | 0.56 | 1.31E-01 | -0.30 | 1.08 | 4.41E-01 | 0.17 | 0.39 | 3.98E-02 | 0.47 | 0.76 | 3189332 | 1177491 | 3581605 | 1234355 | 3768267 | 1286048 | 3394944 | 1163328 |
| RP_NEG_284_22363a10_16 | RP | NEG | 10.16 | 284.2236 | | | | 1.31E-02 | 0.47 | 0.94 | 1.31E-01 | 0.31 | 1.17 | 3.60E-03 | 0.62 | 1.13 | 1.40E-01 | 0.32 | 1.00 | 515587 | 144190 | 593840 | 169712 | 568179 | 172374 | 619501 | 164729 |
| HILIC_POS_143_11786a2_425 | HILIC | POS | 2.425 | 143.1179 | | | | 5.77E-04 | 0.50 | 1.05 | 1.31E-01 | 0.33 | 0.95 | 1.17E-03 | 0.67 | 1.25 | 3.33E-02 | 0.34 | 1.03 | 462650 | 353885 | 807816 | 746433 | 694890 | 615799 | 920743 | 848799 |
| RP_NEG_293_87778a15_515 | RP | NEG | 15.52 | 293.8778 | | | | 7.64E-01 | -0.07 | 0.28 | 1.32E-01 | 0.28 | 1.07 | 7.79E-01 | 0.07 | 0.36 | 4.56E-01 | -0.21 | 0.46 | 1227832 | 145831 | 1219416 | 105114 | 1203459 | 139818 | 1235374 | 47482 |
| RP_NEG_896_565a13_427 | RP | NEG | 13.43 | 896.5650 | | | | 7.53E-02 | 0.38 | 0.63 | 1.32E-01 | -0.29 | 0.96 | 3.45E-01 | 0.23 | 0.59 | 1.75E-02 | 0.53 | 1.03 | 6074136 | 1379227 | 6574957 | 1286197 | 6769363 | 1020097 | 6380551 | 1491522 |
| RP_NEG_378_77911a15_629 | RP | NEG | 15.63 | 378.7791 | | | | 1.11E-01 | -0.32 | 0.58 | 1.32E-01 | -0.30 | 1.14 | 3.80E-02 | -0.47 | 0.80 | 4.48E-01 | -0.17 | 0.65 | 1734099 | 66606 | 1711889 | 70199 | 1722488 | 68025 | 1701290 | 71413 |
| RP_NEG_251_09369a7_77 | RP | NEG | 7.77 | 251.0937 | | | | 1.93E-02 | -0.52 | 0.99 | 1.32E-01 | 0.28 | 1.14 | 1.09E-01 | -0.38 | 0.71 | 6.44E-03 | -0.66 | 1.07 | 2467366 | 126274 | 2407242 | 107946 | 2390945 | 107999 | 2423538 | 106474 |
| RP_POS_183_0993a6_567 | RP | POS | 6.57 | 183.0993 | | | | 4.52E-02 | 0.30 | 0.93 | 1.32E-01 | 0.33 | 1.13 | 4.39E-02 | 0.46 | 0.85 | 2.99E-01 | 0.13 | 1.14 | 580356 | 272102 | 727918 | 546662 | 645042 | 274943 | 810795 | 717053 |
| RP_POS_387_25259a8_497 | RP | POS | 8.50 | 387.2526 | | | | 9.28E-02 | 0.28 | 0.75 | 1.32E-01 | 0.32 | 1.02 | 4.45E-02 | 0.44 | 0.76 | 4.92E-01 | 0.12 | 0.60 | 512211 | 461159 | 694418 | 694307 | 589575 | 543678 | 799262 | 809903 |
| RP_POS_547_38403a14_946 | RP | POS | 14.95 | 547.3840 | | | | 8.98E-02 | 0.25 | 0.66 | 1.32E-01 | 0.33 | 0.95 | 3.74E-02 | 0.42 | 0.79 | 6.05E-01 | 0.09 | 0.30 | 2227926 | 355957 | 2389061 | 699869 | 2283458 | 610832 | 2494663 | 770556 |
| HILIC_NEG_100_93343a6_101 | HILIC | NEG | 6.101 | 100.9334 | | | | 6.37E-02 | -0.29 | 0.94 | 1.32E-01 | -0.33 | 1.08 | 2.70E-02 | -0.45 | 0.72 | 4.75E-01 | -0.12 | 0.42 | 34205396 | 3443073 | 32589458 | 6093402 | 33508651 | 5349414 | 31670264 | 6684032 |
| RP_NEG_926_79022a0_491 | RP | NEG | 0.49 | 926.7902 | | | | 6.24E-08 | -0.71 | 1.33 | 1.32E-01 | 0.33 | 1.31 | 2.34E-03 | -0.55 | 1.22 | 2.96E-07 | -0.87 | 1.61 | 1483913 | 187898 | 1096807 | 586446 | 1008363 | 539978 | 1185251 | 622348 |
| HILIC_POS_542_32349a1_137 | HILIC | POS | 1.137 | 542.3235 | | | | 3.54E-01 | -0.21 | 0.68 | 1.32E-01 | -0.29 | 0.93 | 1.56E-01 | -0.35 | 0.55 | 7.83E-01 | -0.07 | 0.49 | 1840377 | 1228056 | 1616030 | 1015934 | 1769201 | 975625 | 1462860 | 1041913 |
| RP_NEG_536_88458a0_496 | RP | NEG | 0.50 | 536.8846 | | | | 3.13E-09 | -0.77 | 1.34 | 1.33E-01 | 0.32 | 1.27 | 3.36E-04 | -0.61 | 1.24 | 2.82E-07 | -0.93 | 1.60 | 21619709 | 1266417 | 18530635 | 4307574 | 17881622 | 4301141 | 19179648 | 4257889 |
| RP_NEG_321_20529a10_826 | RP | NEG | 10.83 | 321.2053 | | | | 2.30E-11 | 1.13 | 1.98 | 1.33E-01 | 0.28 | 1.22 | 2.91E-10 | 1.27 | 2.11 | 9.85E-08 | 0.99 | 1.83 | 304365 | 270663 | 793639 | 408583 | 732157 | 389295 | 855122 | 421926 |
| RP_POS_850_55292a13_727 | RP | POS | 13.73 | 850.5529 | | | | 3.36E-01 | 0.18 | 0.36 | 1.33E-01 | -0.31 | 1.18 | 9.06E-01 | 0.03 | 0.50 | 1.17E-01 | 0.34 | 0.61 | 7557736 | 2507809 | 8073505 | 2946860 | 8516877 | 2907967 | 7630132 | 2947497 |
| HILIC_NEG_343_99533a6_273 | HILIC | NEG | 6.273 | 343.9953 | | | | 5.42E-01 | 0.13 | 0.51 | 1.33E-01 | -0.30 | 0.82 | 9.35E-01 | -0.02 | 0.19 | 2.47E-01 | 0.28 | 0.47 | 19527385 | 6795514 | 20361220 | 6344628 | 21315235 | 6704218 | 19407205 | 5875896 |
| HILIC_NEG_201_03835a1_301 | HILIC | NEG | 1.301 | 201.0384 | | | | 9.51E-12 | 0.99 | 1.59 | 1.33E-01 | 0.30 | 0.93 | 7.15E-09 | 1.14 | 1.78 | 6.01E-07 | 0.83 | 1.61 | 714978 | 225516 | 1194583 | 490317 | 1120860 | 445328 | 1268306 | 525659 |
| RP_POS_566_31891a10_305 | RP | POS | 10.31 | 566.3189 | | | | 8.32E-03 | 0.57 | 1.04 | 1.34E-01 | -0.29 | 1.24 | 7.76E-02 | 0.42 | 0.72 | 1.84E-03 | 0.71 | 1.21 | 22560481 | 6169032 | 25947312 | 5726917 | 26808114 | 4990243 | 25086510 | 6312666 |
| RP_POS_409_26532a10_659 | RP | POS | 10.66 | 409.2653 | | | | 5.94E-01 | 0.10 | 0.75 | 1.34E-01 | -0.31 | 1.38 | 8.13E-01 | -0.05 | 0.81 | 2.45E-01 | 0.26 | 0.67 | 1922537 | 752266 | 2006833 | 840175 | 2132950 | 853187 | 1880717 | 815969 |
| RP_POS_451_3627a10_453 | RP | POS | 10.45 | 451.3627 | | | | 1.81E-02 | -0.48 | 0.85 | 1.34E-01 | 0.30 | 0.89 | 1.32E-01 | -0.33 | 0.71 | 6.00E-03 | -0.63 | 1.09 | 3482802 | 256641 | 3355091 | 261928 | 3315778 | 265313 | 3394403 | 255083 |
| RP_POS_353_24472a11_129 | RP | POS | 11.13 | 353.2447 | | | | 6.68E-05 | 0.63 | 1.68 | 1.34E-01 | 0.32 | 1.27 | 1.75E-04 | 0.79 | 1.58 | 5.58E-03 | 0.47 | 1.50 | 2862381 | 1472413 | 4387517 | 2561343 | 4002890 | 2150770 | 4772144 | 2885674 |
| RP_POS_606_02289a10_867 | RP | POS | 10.87 | 606.0229 | | | | 1.41E-03 | 0.40 | 1.05 | 1.34E-01 | -0.34 | 1.12 | 5.80E-02 | 0.23 | 1.01 | 6.77E-03 | 0.56 | 1.08 | 425893 | 92838 | 570911 | 412374 | 632933 | 506737 | 508889 | 280966 |
| RP_NEG_247_96269a1_462 | RP | NEG | 1.46 | 247.9627 | | | | 3.89E-08 | 0.68 | 1.15 | 1.34E-01 | 0.33 | 0.95 | 1.14E-04 | 0.84 | 1.24 | 1.16E-06 | 0.51 | 1.46 | 567604 | 545511 | 2373037 | 2913451 | 1934370 | 1664869 | 2811705 | 3739669 |
| HILIC_POS_132_10213a3_04 | HILIC | POS | 3.04 | 132.1021 | | 132.1017(100) | 4 | 6.03E-07 | 0.66 | 1.09 | 1.34E-01 | 0.32 | 1.03 | 1.41E-05 | 0.83 | 1.50 | 1.93E-03 | 0.50 | 0.97 | 620558 | 195059 | 973589 | 577148 | 887026 | 530466 | 1060152 | 613442 |
| RP_POS_382_2478a12_208 | RP | POS | 12.21 | 382.2478 | | | | 1.92E-02 | -0.51 | 0.97 | 1.35E-01 | 0.29 | 1.08 | 1.19E-01 | -0.36 | 0.77 | 6.27E-03 | -0.65 | 1.16 | 602340 | 220714 | 495938 | 201714 | 465708 | 201033 | 526167 | 199821 |
| RP_NEG_682_33496a10_446 | RP | NEG | 10.45 | 682.3350 | | | | 3.00E-04 | 0.68 | 1.34 | 1.35E-01 | -0.30 | 1.08 | 1.41E-02 | 0.53 | 1.03 | 5.97E-05 | 0.83 | 1.53 | 675173 | 335881 | 949931 | 402117 | 1010203 | 361922 | 889659 | 433930 |
| HILIC_POS_139_12309a2_936 | HILIC | POS | 2.936 | 139.1231 | | | | 4.61E-01 | 0.14 | 0.79 | 1.35E-01 | 0.31 | 0.94 | 1.69E-01 | 0.29 | 0.89 | 9.32E-01 | -0.02 | 0.46 | 12184668 | 3108604 | 12677019 | 3719418 | 12119794 | 3717552 | 13234244 | 3673771 |
| RP_POS_256_10986a4_551 | RP | POS | 4.55 | 256.1099 | | | | 5.82E-01 | 0.08 | 0.48 | 1.35E-01 | -0.33 | 0.89 | 4.81E-01 | -0.09 | 0.51 | 2.78E-01 | 0.25 | 0.58 | 21059053 | 5461982 | 21886641 | 11490614 | 23615607 | 15041024 | 20157675 | 5867700 |

**Supplementary table 1: Metabolomics results** (in asceding order according to p-value from progressive ALD vs non-progressive ALD comparison, with molecular features with ID_level of 1-2 first)

| | | | | | | | | all_ALD_vs_control | | | Prog_ALD_vs_non-prog_ALD | | | Prog_ALD_vs_control_ALD | | | Non-prog_ALD_vs_control | | | Healthy_control | | all_ALD | | Non-prog_ALD | | Progressive_ALD | |
|---|---|---|---|---|---|---|---|---|---|---|---|---|---|---|---|---|---|---|---|---|---|---|---|---|---|---|---|
| | | | | | | | | t-test | Cohen's | PLS-DA | t-test | Cohen's | PLS-DA | t-test | Cohen's | PLS-DA | t-test | Cohen's | PLS-DA | | | | | | | | |
| Feature_ID | RP_HILIC | POS_NEG | Average.Rt | Average.Mz | Metabolite.name | curated_MSMS | ID_level | p | d | VIP | p | d | VIP | p | d | VIP | p | d | VIP | Mean | SD | Mean | SD | Mean | SD | Mean | SD |
| RP_POS_199_13042a13_013 | RP | POS | 13.01 | 199.1304 | | | | 8.07E-01 | -0.05 | 0.67 | 1.35E-01 | -0.29 | 0.96 | 4.35E-01 | -0.20 | 0.48 | 6.78E-01 | 0.09 | 0.58 | 23766970 | 4179545 | 23564568 | 3687359 | 24117475 | 2800387 | 23011660 | 4359433 |
| RP_POS_205_98676a0_254 | RP | POS | 0.25 | 205.9868 | | | | 8.59E-01 | -0.04 | 0.31 | 1.36E-01 | -0.29 | 0.99 | 4.38E-01 | -0.18 | 0.40 | 6.47E-01 | 0.11 | 0.56 | 3732910 | 588080 | 3712065 | 531987 | 3791622 | 520707 | 3632508 | 536417 |
| RP_NEG_851_11426a9_304 | RP | NEG | 9.30 | 851.1143 | | | | 2.13E-01 | -0.22 | 0.57 | 1.36E-01 | -0.32 | 0.96 | 6.32E-02 | -0.38 | 0.72 | 7.63E-01 | -0.06 | 0.32 | 4250577 | 267621 | 4176267 | 355324 | 4229360 | 364491 | 4123174 | 341293 |
| RP_NEG_307_85608a15_525 | RP | NEG | 15.53 | 307.8561 | | | | 2.29E-01 | -0.24 | 0.64 | 1.36E-01 | -0.30 | 0.88 | 7.74E-02 | -0.39 | 0.70 | 6.91E-01 | -0.09 | 0.29 | 11001180 | 302033 | 10925684 | 316718 | 10972976 | 326205 | 10878393 | 302809 |
| RP_NEG_519_40668a13_181 | RP | NEG | 13.18 | 519.4067 | | | | 1.83E-02 | -0.52 | 0.89 | 1.36E-01 | -0.28 | 0.88 | 5.88E-03 | -0.67 | 1.09 | 1.07E-01 | -0.38 | 0.64 | 17417368 | 1181192 | 16849735 | 1015589 | 17001332 | 1027745 | 16698138 | 990319 |
| RP_POS_140_06827a0_694 | RP | POS | 0.69 | 140.0683 | | | | 4.94E-02 | 0.45 | 1.24 | 1.37E-01 | -0.33 | 1.38 | 5.43E-01 | 0.10 | 1.31 | 1.96E-02 | 0.44 | 1.22 | 298939 | 53319 | 332060 | 137731 | 352607 | 144023 | 311513 | 129317 |
| RP_POS_289_64111a10_617 | RP | POS | 10.62 | 289.6411 | | | | 3.35E-01 | 0.22 | 1.04 | 1.37E-01 | -0.28 | 1.27 | 7.50E-01 | 0.08 | 0.74 | 1.31E-01 | 0.36 | 1.07 | 10846992 | 1972883 | 11220567 | 1583926 | 11456848 | 1350458 | 10984287 | 1769512 |
| HILIC_NEG_217_02998a4_805 | HILIC | NEG | 4.805 | 217.0300 | | | | 1.27E-01 | 0.23 | 0.50 | 1.37E-01 | 0.33 | 1.02 | 7.56E-02 | 0.39 | 0.66 | 6.52E-01 | 0.06 | 0.51 | 768804 | 199952 | 849296 | 386426 | 791542 | 251790 | 907050 | 481136 |
| RP_POS_392_20059a11_125 | RP | POS | 11.13 | 392.2006 | | | | 1.20E-06 | 0.81 | 1.72 | 1.37E-01 | 0.30 | 1.06 | 3.70E-06 | 0.96 | 1.70 | 3.12E-04 | 0.66 | 1.54 | 251602 | 130431 | 412344 | 201812 | 382258 | 182814 | 442429 | 216839 |
| RP_POS_416_20053a11_048 | RP | POS | 11.05 | 416.2005 | | | | 4.79E-09 | 0.83 | 1.77 | 1.37E-01 | 0.31 | 1.01 | 4.80E-07 | 0.99 | 1.75 | 4.29E-05 | 0.68 | 1.64 | 301938 | 172012 | 617145 | 395371 | 558230 | 357344 | 676060 | 425497 |
| RP_POS_326_18066a0_702 | RP | POS | 0.70 | 326.1807 | | | | 7.35E-15 | 0.97 | 1.67 | 1.37E-01 | 0.31 | 0.92 | 5.27E-08 | 1.12 | 1.75 | 3.67E-09 | 0.81 | 1.73 | 59256 | 59112 | 476643 | 450763 | 409419 | 343635 | 543867 | 532179 |
| RP_NEG_271_22806a10_509 | RP | NEG | 10.51 | 271.2281 | | | | 1.24E-01 | -0.26 | 0.94 | 1.37E-01 | 0.32 | 1.21 | 6.02E-01 | -0.10 | 0.79 | 3.73E-02 | -0.42 | 0.94 | 8471504 | 1064542 | 8094778 | 1526367 | 7867476 | 1515622 | 8322080 | 1518087 |
| RP_POS_592_46838a12_111 | RP | POS | 12.11 | 592.4684 | | | | 9.10E-02 | -0.35 | 0.67 | 1.37E-01 | 0.29 | 1.18 | 3.75E-01 | -0.20 | 0.47 | 3.01E-02 | -0.50 | 0.83 | 951905 | 461109 | 791107 | 455124 | 723331 | 445696 | 858882 | 458784 |
| RP_POS_265_14349a7_276 | RP | POS | 7.28 | 265.1435 | | | | 1.61E-06 | -1.09 | 1.71 | 1.37E-01 | -0.25 | 0.94 | 3.02E-07 | -1.22 | 1.98 | 5.34E-05 | -0.97 | 1.42 | 778225 | 358811 | 392188 | 297234 | 436453 | 330056 | 347924 | 256053 |
| RP_POS_465_28467a9_334 | RP | POS | 9.33 | 465.2847 | | | | 2.24E-02 | 0.27 | 0.45 | 1.37E-01 | 0.34 | 0.89 | 5.51E-02 | 0.44 | 0.65 | 4.96E-02 | 0.10 | 0.62 | 247279 | 258809 | 1347392 | 4720964 | 639985 | 1344716 | 2054799 | 6496177 |
| RP_POS_136_04803a0_55 | RP | POS | 0.55 | 136.0480 | | | | 5.13E-01 | -0.10 | 1.18 | 1.37E-01 | -0.33 | 1.20 | 1.51E-01 | -0.26 | 1.29 | 7.50E-01 | 0.06 | 1.09 | 2395584 | 430825 | 2324370 | 771942 | 2439252 | 800872 | 2209488 | 731861 |
| RP_POS_176_07059a5_864 | RP | POS | 5.86 | 176.0706 | | | | 5.96E-02 | -0.25 | 0.64 | 1.38E-01 | -0.34 | 0.96 | 5.35E-06 | -0.42 | 1.76 | 7.14E-01 | -0.08 | 0.61 | 648779 | 388471 | 406626 | 1073451 | 567450 | 1481882 | 245801 | 281691 |
| HILIC_NEG_98_93893a3_015 | HILIC | NEG | 3.015 | 98.9389 | | | | 3.16E-01 | -0.21 | 0.70 | 1.38E-01 | 0.29 | 0.96 | 7.70E-01 | -0.07 | 0.43 | 1.26E-01 | -0.36 | 0.74 | 689535 | 106541 | 668023 | 98355 | 653395 | 96951 | 682651 | 98532 |
| RP_POS_473_32288a10_897 | RP | POS | 10.90 | 473.3229 | | | | 1.68E-02 | 0.43 | 0.80 | 1.38E-01 | 0.31 | 0.98 | 5.06E-03 | 0.58 | 1.14 | 1.81E-01 | 0.28 | 0.61 | 1386160 | 504699 | 1657719 | 656392 | 1560163 | 656671 | 1755274 | 647962 |
| RP_POS_170_09219a0_514 | RP | POS | 0.51 | 170.0922 | | | | 1.91E-01 | 0.24 | 0.68 | 1.39E-01 | -0.31 | 0.94 | 6.69E-01 | 0.08 | 0.43 | 8.13E-02 | 0.39 | 0.74 | 644020 | 481786 | 782269 | 608879 | 872695 | 690039 | 691844 | 506154 |
| RP_POS_619_49023a11_61 | RP | POS | 11.61 | 619.4902 | | | | 1.20E-01 | -0.28 | 0.50 | 1.39E-01 | -0.31 | 0.94 | 2.50E-02 | -0.44 | 0.84 | 5.74E-01 | -0.12 | 0.29 | 6285422 | 4056826 | 4886926 | 5239927 | 5665002 | 5897654 | 4108851 | 4410915 |
| RP_POS_535_32354a10_857 | RP | POS | 10.86 | 535.3236 | | | | 8.19E-06 | 0.58 | 1.10 | 1.39E-01 | 0.33 | 1.26 | 2.12E-04 | 0.74 | 1.21 | 4.00E-03 | 0.41 | 1.18 | 533735 | 207668 | 899764 | 697012 | 796274 | 566082 | 1003255 | 799538 |
| RP_POS_266_84134a0_515 | RP | POS | 0.52 | 266.8413 | | | | 1.00E-01 | -0.29 | 0.63 | 1.39E-01 | -0.31 | 0.90 | 2.67E-02 | -0.45 | 0.94 | 5.18E-01 | -0.14 | 0.41 | 346766 | 89098 | 313878 | 118775 | 331496 | 123698 | 296259 | 112107 |
| RP_POS_367_22693a10_884 | RP | POS | 10.88 | 367.2269 | | | | 1.02E-02 | -0.34 | 0.86 | 1.39E-01 | 0.33 | 1.09 | 3.91E-01 | -0.18 | 0.66 | 2.35E-04 | -0.51 | 1.19 | 1405832 | 167326 | 1258237 | 482480 | 1186622 | 342460 | 1329852 | 585303 |
| HILIC_POS_518_32294a1_32 | HILIC | POS | 1.32 | 518.3229 | | | | 6.76E-09 | 0.99 | 1.56 | 1.39E-01 | -0.29 | 1.00 | 4.57E-05 | 0.84 | 1.33 | 1.10E-09 | 1.13 | 2.16 | 20107604 | 3233652 | 24963221 | 4798116 | 25674511 | 3958334 | 24251931 | 5460105 |
| HILIC_POS_263_14612a5_591 | HILIC | POS | 5.591 | 263.1461 | | | | 1.34E-01 | 0.27 | 0.64 | 1.40E-01 | -0.31 | 1.35 | 6.03E-01 | 0.11 | 0.56 | 3.13E-02 | 0.42 | 0.84 | 797885 | 863261 | 1084893 | 1121586 | 1251002 | 981462 | 918783 | 1233696 |
| RP_POS_873_69244a10_839 | RP | POS | 10.84 | 873.6924 | | | | 1.98E-08 | 0.84 | 1.51 | 1.40E-01 | 0.31 | 0.90 | 5.38E-07 | 1.00 | 1.61 | 5.56E-05 | 0.69 | 1.47 | 164813 | 121319 | 359787 | 238449 | 324484 | 217186 | 395090 | 255271 |
| RP_POS_191_97209a15_548 | RP | POS | 15.55 | 191.9721 | | | | 1.98E-01 | -0.25 | 0.55 | 1.40E-01 | 0.30 | 0.89 | 6.33E-01 | -0.10 | 0.37 | 8.53E-02 | -0.40 | 0.70 | 4432994 | 949783 | 4173737 | 1069283 | 4015398 | 1208045 | 4332076 | 894162 |
| HILIC_POS_229_15492a5_011 | HILIC | POS | 5.011 | 229.1549 | | | | 4.93E-01 | 0.11 | 0.79 | 1.40E-01 | 0.32 | 1.10 | 2.39E-01 | 0.27 | 0.73 | 7.47E-01 | -0.05 | 1.03 | 4995610 | 1255181 | 5200959 | 1989530 | 4905647 | 1177657 | 5496271 | 2536215 |
| RP_POS_808_07574a13_841 | RP | POS | 13.84 | 808.0757 | | | | 2.54E-02 | 0.42 | 0.98 | 1.40E-01 | -0.30 | 0.90 | 2.16E-01 | 0.26 | 0.62 | 6.96E-03 | 0.57 | 1.21 | 1508360 | 623195 | 1814318 | 755320 | 1926055 | 724143 | 1702581 | 776400 |
| RP_POS_514_87274a0_497 | RP | POS | 0.50 | 514.8727 | | | | 1.97E-09 | -0.75 | 1.34 | 1.40E-01 | 0.32 | 1.27 | 2.71E-04 | -0.59 | 1.27 | 7.61E-07 | -0.91 | 1.57 | 18460071 | 856095 | 15602162 | 4132746 | 14991179 | 4264123 | 16213146 | 3945017 |
| RP_NEG_205_83949a1_319 | RP | NEG | 1.32 | 205.8395 | | | | 1.20E-03 | 0.53 | 0.93 | 1.40E-01 | -0.31 | 1.02 | 2.51E-02 | 0.37 | 1.00 | 1.81E-03 | 0.69 | 1.10 | 3008105 | 1452580 | 4166524 | 2312134 | 4508808 | 2736663 | 3824240 | 1752884 |
| RP_NEG_160_84106a15_582 | RP | NEG | 15.58 | 160.8411 | | | | 8.13E-03 | -0.54 | 0.96 | 1.40E-01 | -0.29 | 1.11 | 2.09E-03 | -0.68 | 1.23 | 8.45E-02 | -0.39 | 0.65 | 3221376 | 106326 | 3161539 | 109859 | 3177777 | 115605 | 3145301 | 102380 |
| RP_POS_144_10176a0_705 | RP | POS | 0.71 | 144.1018 | | | | 1.34E-01 | 0.17 | 0.73 | 1.40E-01 | 0.34 | 0.99 | 1.36E-01 | 0.35 | 0.91 | 8.58E-01 | 0.00 | 0.36 | 1524045 | 992700 | 3451390 | 12650490 | 1567434 | 1176766 | 5335346 | 17740000 |
| RP_POS_203_05185a0_547 | RP | POS | 0.55 | 203.0519 | Unknown | 203.05185(100) | 4 | 4.85E-06 | 0.58 | 1.13 | 1.40E-01 | 0.33 | 1.37 | 9.98E-05 | 0.74 | 1.33 | 6.79E-03 | 0.41 | 1.28 | 83353072 | 8064914 | 100303264 | 32488511 | 95502642 | 28937323 | 105103886 | 35329114 |
| RP_NEG_316_9491a14_258 | RP | NEG | 14.26 | 316.9491 | | | | 5.08E-01 | -0.13 | 0.60 | 1.41E-01 | 0.31 | 1.09 | 8.98E-01 | 0.03 | 0.29 | 2.01E-01 | -0.28 | 0.69 | 5636413 | 2100743 | 5340151 | 2443425 | 4979301 | 2469077 | 5701000 | 2387554 |
| HILIC_NEG_142_92892a7_352 | HILIC | NEG | 7.352 | 142.9289 | | | | 2.66E-01 | -0.16 | 0.27 | 1.41E-01 | -0.33 | 0.84 | 8.61E-02 | -0.32 | 0.63 | 9.86E-01 | 0.00 | 0.32 | 3048480 | 222365 | 2979507 | 475937 | 3049794 | 452227 | 2909220 | 493013 |
| RP_POS_809_58551a13_021 | RP | POS | 13.02 | 809.5855 | | | | 4.39E-04 | -0.78 | 1.33 | 1.41E-01 | -0.27 | 0.89 | 1.11E-04 | -0.92 | 1.54 | 6.66E-03 | -0.65 | 1.11 | 7134577 | 3430902 | 4578288 | 2981452 | 5018595 | 3164687 | 4137981 | 2747477 |
| RP_POS_285_12387a11_804 | RP | POS | 11.80 | 285.1239 | | | | 1.41E-02 | -0.49 | 1.21 | 1.41E-01 | 0.29 | 1.33 | 1.58E-01 | -0.35 | 0.69 | 1.40E-03 | -0.64 | 1.46 | 1316415 | 698209 | 952841 | 730095 | 844750 | 453307 | 1060932 | 920665 |
| RP_POS_335_25513a10_902 | RP | POS | 10.90 | 335.2551 | | | | 5.20E-02 | -0.32 | 0.92 | 1.41E-01 | 0.32 | 1.15 | 4.66E-01 | -0.16 | 0.79 | 6.18E-03 | -0.47 | 1.08 | 748850 | 127432 | 688816 | 205126 | 658520 | 161473 | 719112 | 238884 |
| HILIC_NEG_199_80507a0_882 | HILIC | NEG | 0.882 | 199.8051 | | | | 1.80E-01 | 0.27 | 0.93 | 1.41E-01 | 0.30 | 1.16 | 6.71E-02 | 0.42 | 0.92 | 5.76E-01 | 0.12 | 0.21 | 449829 | 84304 | 473205 | 86058 | 460512 | 83341 | 485898 | 87684 |
| RP_NEG_197_80789a2_201 | RP | NEG | 2.20 | 197.8079 | | | | 5.64E-01 | 0.11 | 0.66 | 1.41E-01 | -0.30 | 1.14 | 8.46E-01 | -0.04 | 0.42 | 2.61E-01 | 0.26 | 0.45 | 12722088 | 2243615 | 12993666 | 2491815 | 13361459 | 2836382 | 12625872 | 2055206 |
| HILIC_POS_171_18575a7_097 | HILIC | POS | 7.097 | 171.1858 | | | | 7.03E-01 | 0.08 | 0.99 | 1.41E-01 | -0.30 | 1.02 | 7.30E-01 | -0.08 | 0.94 | 3.18E-01 | 0.23 | 0.85 | 6763088 | 1682337 | 6896385 | 1808724 | 7163084 | 1863968 | 6629686 | 1729217 |
| RP_POS_580_35052a11_101 | RP | POS | 11.10 | 580.3505 | | | | 1.94E-07 | 0.62 | 1.30 | 1.41E-01 | -0.33 | 1.32 | 4.59E-04 | 0.46 | 1.36 | 8.99E-05 | 0.78 | 1.41 | 41953 | 33462 | 1273144 | 2198700 | 1597707 | 2576949 | 948582 | 1706333 |
| HILIC_NEG_568_36389a1_173 | HILIC | NEG | 1.173 | 568.3639 | | | | 3.02E-01 | -0.24 | 0.61 | 1.41E-01 | -0.28 | 1.12 | 1.33E-01 | -0.38 | 0.81 | 6.84E-01 | -0.10 | 0.24 | 2439190 | 786250 | 2280189 | 623861 | 2372156 | 615135 | 2188223 | 625056 |
| RP_POS_298_69098a10_932 | RP | POS | 10.93 | 298.6910 | | | | 2.73E-04 | -0.84 | 1.63 | 1.41E-01 | -0.26 | 1.45 | 8.87E-05 | -0.97 | 1.66 | 3.20E-03 | -0.71 | 1.39 | 993262 | 267308 | 787846 | 217040 | 819825 | 216685 | 755866 | 214789 |
| RP_POS_232_05246a8_574 | RP | POS | 8.57 | 232.0525 | | | | 6.07E-01 | 0.07 | 0.63 | 1.42E-01 | 0.33 | 1.02 | 3.05E-01 | 0.24 | 0.87 | 2.67E-01 | -0.10 | 0.77 | 1688458 | 1472943 | 1949898 | 4350538 | 1305633 | 1570231 | 2594162 | 5909983 |
| RP_NEG_197_80798a4_573 | RP | NEG | 4.57 | 197.8080 | | | | 1.61E-03 | -0.69 | 1.08 | 1.42E-01 | -0.27 | 0.95 | 4.86E-04 | -0.83 | 1.30 | 1.76E-02 | -0.56 | 0.95 | 17501279 | 6400276 | 13268280 | 5654156 | 14100365 | 5762865 | 12436195 | 5474285 |
| RP_POS_321_12079a11_804 | RP | POS | 11.80 | 321.1208 | | | | 2.98E-02 | -0.43 | 1.11 | 1.42E-01 | 0.30 | 1.32 | 2.56E-01 | -0.28 | 0.66 | 2.83E-03 | -0.58 | 1.37 | 595043 | 339380 | 438276 | 367080 | 384095 | 208978 | 492458 | 471786 |
| RP_POS_441_297a13_095 | RP | POS | 13.10 | 441.2970 | | | | 1.01E-01 | 0.24 | 0.46 | 1.43E-01 | 0.33 | 0.98 | 4.92E-02 | 0.40 | 0.76 | 6.35E-01 | 0.08 | 0.51 | 8745438 | 1352792 | 9363468 | 2868695 | 8941687 | 2372839 | 9785249 | 3260882 |
| RP_NEG_894_54907a12_821 | RP | NEG | 12.82 | 894.5491 | | | | 8.47E-06 | -1.03 | 1.68 | 1.43E-01 | -0.25 | 0.98 | 3.22E-06 | -1.16 | 1.80 | 1.20E-04 | -0.91 | 1.47 | 4296869 | 1294610 | 3032425 | 1036427 | 3184703 | 986719 | 2880146 | 1072076 |
| RP_NEG_211_91728a13_292 | RP | NEG | 13.29 | 211.9173 | | | | 1.80E-01 | 0.25 | 0.42 | 1.43E-01 | -0.30 | 1.09 | 6.42E-01 | 0.10 | 0.62 | 5.49E-02 | 0.40 | 0.79 | 2096482 | 650828 | 2283789 | 763237 | 2395909 | 717657 | 2171670 | 797660 |
| HILIC_NEG_253_0929a1_279 | HILIC | NEG | 1.279 | 253.0929 | | | | 1.69E-12 | 0.86 | 1.44 | 1.43E-01 | 0.31 | 1.03 | 2.01E-07 | 1.02 | 1.62 | 1.39E-06 | 0.71 | 1.73 | 20850 | 18297 | 219596 | 245262 | 183556 | 208812 | 255636 | 274374 |
| RP_NEG_235_92616a13_287 | RP | NEG | 13.29 | 235.9262 | | | | 5.49E-02 | -0.43 | 0.79 | 1.43E-01 | 0.28 | 0.87 | 2.20E-01 | -0.29 | 0.50 | 2.12E-02 | -0.57 | 1.00 | 947363 | 218991 | 862785 | 185990 | 835487 | 191645 | 890083 | 177882 |
| RP_POS_331_26315a11_049 | RP | POS | 11.05 | 331.2632 | | | | 4.66E-10 | 0.83 | 1.74 | 1.43E-01 | 0.31 | 1.02 | 5.99E-07 | 0.98 | 1.71 | 1.53E-05 | 0.67 | 1.67 | 1456373 | 767708 | 3263301 | 2311536 | 2924267 | 2011848 | 3602334 | 2552123 |
| RP_POS_305_09622a7_093 | RP | POS | 7.09 | 305.0962 | | | | 3.26E-01 | -0.19 | 0.74 | 1.44E-01 | 0.30 | 1.01 | 8.46E-01 | -0.04 | 0.35 | 1.21E-01 | -0.34 | 0.76 | 988168 | 68366 | 974079 | 74751 | 963122 | 73624 | 985037 | 74994 |
| HILIC_NEG_113_92623a4_793 | HILIC | NEG | 4.793 | 113.9262 | | | | 9.86E-01 | 0.00 | 0.48 | 1.44E-01 | 0.29 | 1.70 | 5.51E-01 | 0.14 | 0.48 | 5.43E-01 | -0.15 | 0.27 | 3293876 | 1066123 | 3290115 | 958221 | 3149703 | 999087 | 3430527 | 903716 |
| RP_POS_421_32828a11_341 | RP | POS | 11.34 | 421.3283 | | | | 2.58E-01 | -0.21 | 0.70 | 1.44E-01 | 0.30 | 1.20 | 7.83E-01 | -0.06 | 0.75 | 8.91E-02 | -0.36 | 0.69 | 1931086 | 256976 | 1868275 | 310090 | 1822894 | 306547 | 1913655 | 310000 |
| RP_POS_229_15436a0_709 | RP | POS | 0.71 | 229.1544 | | | | 2.79E-02 | 0.32 | 0.55 | 1.45E-01 | 0.32 | 0.93 | 2.46E-02 | 0.48 | 0.79 | 2.69E-01 | 0.16 | 0.71 | 6107647 | 1167572 | 6813615 | 2403024 | 6461705 | 1713566 | 7165525 | 2911675 |
| HILIC_POS_130_08643a4_986 | HILIC | POS | 4.986 | 130.0864 | | | | 2.77E-01 | 0.28 | 0.68 | 1.45E-01 | 0.25 | 1.25 | 1.57E-01 | 0.40 | 0.58 | 5.51E-01 | 0.15 | 0.33 | 5941064 | 11697005 | 8373014 | 7652726 | 7252323 | 4817322 | 9493706 | 9620492 |
| RP_POS_194_11528a10_386 | RP | POS | 10.39 | 194.1153 | | | | 9.00E-01 | 0.02 | 0.88 | 1.45E-01 | 0.30 | 1.30 | 4.59E-01 | 0.17 | 0.66 | 5.44E-01 | -0.12 | 0.72 | 1314057 | 269408 | 1321091 | 294017 | 1278159 | 243783 | 1364023 | 333862 |
| RP_POS_261_98135a15_59 | RP | POS | 15.59 | 261.9814 | | | | 5.81E-02 | -0.36 | 0.74 | 1.46E-01 | 0.30 | 1.02 | 3.31E-01 | -0.22 | 0.39 | 1.55E-02 | -0.51 | 1.20 | 7407599 | 249288 | 7306366 | 282989 | 7265098 | 260318 | 7347635 | 300930 |
| RP_POS_165_09105a11_237 | RP | POS | 11.24 | 165.0911 | | | | 1.71E-01 | -0.25 | 0.72 | 1.46E-01 | -0.30 | 1.04 | 8.63E-02 | -0.41 | 0.77 | 5.81E-01 | -0.10 | 0.40 | 704942 | 243568 | 632503 | 296740 | 675841 | 211207 | 589165 | 359812 |
| RP_NEG_317_11475a5_92 | RP | NEG | 5.92 | 317.1148 | | | | 7.35E-01 | -0.05 | 0.67 | 1.46E-01 | 0.32 | 1.34 | 6.27E-01 | 0.11 | 0.46 | 7.71E-02 | -0.21 | 0.90 | 1703300 | 959012 | 1615473 | 1956667 | 1328850 | 854588 | 1902096 | 2614812 |
| HILIC_POS_421_37061a1_388 | HILIC | POS | 1.388 | 421.3706 | | | | 3.44E-01 | -0.15 | 0.66 | 1.46E-01 | -0.32 | 0.93 | 1.35E-01 | -0.31 | 0.50 | 9.66E-01 | 0.01 | 0.46 | 1141351 | 238735 | 1086236 | 397023 | 1144114 | 346581 | 1028359 | 437626 |
| HILIC_NEG_148_04633a0_927 | HILIC | NEG | 0.927 | 148.0463 | | | | 1.66E-04 | 0.54 | 0.89 | 1.46E-01 | -0.32 | 1.34 | 4.75E-02 | 0.39 | 0.65 | 3.36E-05 | 0.70 | 1.41 | 103308 | 50140 | 158094 | 109256 | 174015 | 94679 | 142172 | 120979 |
| RP_POS_319_12875a5_922 | RP | POS | 5.92 | 319.1288 | | | | 7.91E-01 | -0.04 | 0.68 | 1.46E-01 | 0.32 | 1.33 | 5.94E-01 | 0.12 | 0.49 | 1.17E-01 | -0.20 | 0.86 | 1506157 | 834487 | 1447434 | 1639213 | 1207640 | 822438 | 1687227 | 2152933 |
| RP_POS_820_81677a0_493 | RP | POS | 0.49 | 820.8168 | | | | 7.80E-03 | 0.35 | 1.00 | 1.46E-01 | 0.33 | 1.36 | 8.71E-03 | 0.51 | 1.08 | 2.08E-01 | 0.19 | 1.08 | 851327 | 230827 | 1065796 | 680656 | 966678 | 573897 | 1164914 | 765920 |
| RP_POS_1066_12915a10_324 | RP | POS | 10.32 | 1066.1292 | | | | 1.00E-02 | -0.71 | 1.27 | 1.47E-01 | -0.22 | 1.03 | 4.33E-03 | -0.82 | 1.29 | 3.32E-02 | -0.60 | 1.00 | 653758 | 461258 | 422243 | 249673 | 458562 | 246260 | 385925 | 250219 |
| RP_POS_421_35123a10_657 | RP | POS | 10.66 | 421.3512 | | | | 7.20E-01 | 0.07 | 0.31 | 1.47E-01 | 0.30 | 0.86 | 3.00E-01 | 0.22 | 0.45 | 7.10E-01 | -0.08 | 0.60 | 5195327 | 382090 | 5224382 | 441453 | 5160167 | 463927 | 5288596 | 412425 |
| RP_POS_801_98761a10_197 | RP | POS | 10.20 | 801.9876 | | | | 3.00E-02 | -0.54 | 1.19 | 1.47E-01 | -0.25 | 1.17 | 9.75E-03 | -0.67 | 1.21 | 1.20E-01 | -0.42 | 0.96 | 311599 | 275639 | 194286 | 186371 | 221403 | 211237 | 167169 | 155097 |
| RP_POS_187_13335a10_271 | RP | POS | 10.27 | 187.1334 | | | | 9.17E-01 | -0.02 | 0.62 | 1.47E-01 | 0.29 | 1.10 | 6.26E-01 | 0.12 | 0.58 | 4.65E-01 | -0.17 | 0.90 | 1916090 | 149095 | 1912975 | 135799 | 1893220 | 116309 | 1932729 | 151443 |
| RP_POS_164_98398a15_442 | RP | POS | 15.44 | 164.9840 | | | | 5.01E-02 | -0.45 | 0.85 | 1.47E-01 | -0.27 | 0.98 | 1.39E-02 | -0.59 | 1.18 | 2.15E-01 | -0.32 | 0.55 | 586869991 | 164880262 | 522519956 | 130726028 | 541535137 | 151769786 | 503504774 | 103708096 |
| HILIC_NEG_136_89165a4_071 | HILIC | NEG | 4.071 | 136.8917 | | | | 5.37E-01 | -0.14 | 0.86 | 1.47E-01 | 0.28 | 1.09 | 9.93E-01 | 0.00 | 0.20 | 2.52E-01 | -0.28 | 0.86 | 6380996 | 3283410 | 5979664 | 2805151 | 5572304 | 2762547 | 6387024 | 2815673 |
| HILIC_NEG_312_83673a4_757 | HILIC | NEG | 4.757 | 312.8367 | | | | 1.01E-02 | -0.42 | 0.70 | 1.47E-01 | -0.31 | 1.01 | 1.12E-03 | -0.58 | 1.16 | 2.10E-01 | -0.27 | 0.53 | 1139969 | 151097 | 1045632 | 238680 | 1080318 | 275121 | 1010946 | 192229 |
| RP_POS_173_07838a0_772 | RP | POS | 0.77 | 173.0784 | | | | 9.99E-03 | 0.32 | 1.10 | 1.48E-01 | -0.33 | 1.28 | 3.27E-01 | 0.15 | 1.09 | 8.23E-03 | 0.48 | 1.20 | 2943178 | 362064 | 3427187 | 1734824 | 3679057 | 1842780 | 3175316 | 1598544 |
| HILIC_NEG_179_05621a4_984 | HILIC | NEG | 4.984 | 179.0562 | | | | 3.04E-02 | 0.33 | 0.85 | 1.48E-01 | 0.32 | 0.87 | 3.40E-02 | 0.49 | 0.84 | 2.07E-01 | 0.17 | 0.50 | 18710063 | 7997907 | 23292364 | 15365282 | 21053375 | 8319931 | 25531352 | 19938655 |
| RP_POS_252_15695a7_139 | RP | POS | 7.14 | 252.1570 | | | | 7.45E-01 | 0.08 | 0.59 | 1.48E-01 | -0.27 | 0.90 | 8.29E-01 | -0.06 | 0.53 | 4.06E-01 | 0.21 | 0.51 | 761027 | 600346 | 798708 | 445539 | 863333 | 425495 | 734082 | 459883 |
| RP_NEG_134_8934a11_209 | RP | NEG | 11.21 | 134.8934 | | | | 1.11E-01 | 0.29 | 0.54 | 1.48E-01 | -0.30 | 1.05 | 5.28E-01 | 0.14 | 0.85 | 2.76E-02 | 0.44 | 0.92 | 449949 | 109170 | 488287 | 138448 | 508372 | 123058 | 468203 | 150866 |
| RP_NEG_334_78738a15_4 | RP | NEG | 15.40 | 334.7874 | | | | 9.41E-01 | -0.02 | 0.76 | 1.48E-01 | -0.25 | 0.87 | 5.85E-01 | -0.14 | 0.33 | 7.02E-01 | 0.11 | 0.62 | 1316043 | 103016 | 1314607 | 65410 | 1324098 | 73123 | 1305116 | 55798 |
| RP_NEG_960_78442a0_491 | RP | NEG | 0.49 | 960.7844 | | | | 7.45E-09 | -0.75 | 1.35 | 1.48E-01 | 0.31 | 1.27 | 3.75E-04 | -0.59 | 1.28 | 8.08E-07 | -0.90 | 1.57 | 3134606 | 348975 | 2301916 | 1195968 | 2128568 | 1218292 | 2475264 | 1159444 |
| HILIC_POS_524_37189a1_096 | HILIC | POS | 1.096 | 524.3719 | | | | 3.32E-01 | -0.22 | 0.57 | 1.48E-01 | -0.28 | 1.08 | 1.48E-01 | -0.36 | 0.63 | 7.36E-01 | -0.08 | 0.15 | 12475540 | 4810459 | 11553990 | 3990076 | 12132252 | 3908844 | 10975729 | 4025523 |
| RP_POS_777_69281a7_775 | RP | POS | 7.78 | 777.6928 | | | | 9.45E-08 | -1.38 | 2.61 | 1.49E-01 | 0.20 | 0.99 | 7.49E-07 | -1.28 | 2.11 | 2.47E-08 | -1.48 | 2.35 | 1537510 | 420323 | 1023164 | 252565 | 986604 | 219727 | 1059724 | 279056 |
| RP_POS_810_98871a10_618 | RP | POS | 10.62 | 810.9887 | | | | 4.68E-03 | 0.63 | 1.36 | 1.49E-01 | -0.27 | 1.08 | 4.59E-02 | 0.49 | 1.27 | 1.01E-03 | 0.76 | 1.48 | 1862684 | 790621 | 2325495 | 689636 | 2425322 | 570551 | 2225668 | 784243 |
| HILIC_POS_285_09973a1_437 | HILIC | POS | 1.437 | 285.0997 | | | | 8.50E-02 | -0.46 | 0.98 | 1.49E-01 | -0.24 | 0.86 | 3.58E-02 | -0.58 | 1.09 | 2.26E-01 | -0.34 | 0.55 | 405963 | 412128 | 270175 | 242307 | 305196 | 267586 | 235153 | 210993 |
| RP_POS_223_09402a10_796 | RP | POS | 10.80 | 223.0940 | | | | 6.26E-02 | -0.44 | 0.74 | 1.50E-01 | 0.26 | 0.86 | 2.26E-01 | -0.31 | 0.56 | 2.26E-02 | -0.57 | 1.00 | 9049853 | 4180533 | 7509819 | 3213530 | 7045578 | 3009992 | 7974059 | 3371115 |
| RP_POS_419_35126a12_236 | RP | POS | 12.24 | 419.3513 | | | | 4.34E-01 | -0.14 | 0.42 | 1.50E-01 | 0.31 | 0.91 | 9.36E-01 | 0.02 | 0.30 | 1.54E-01 | -0.29 | 0.72 | 1956914 | 280295 | 1907605 | 382892 | 1852390 | 374459 | 1962819 | 386993 |
| RP_POS_319_12878a6_048 | RP | POS | 6.05 | 319.1288 | | | | 8.33E-01 | -0.03 | 0.69 | 1.50E-01 | 0.32 | 1.33 | 5.76E-01 | 0.13 | 0.51 | 1.42E-01 | -0.19 | 0.81 | 925065 | 528735 | 895883 | 1019480 | 748342 | 520936 | 1043425 | 1335698 |

**Supplementary table 1: Metabolomics results** (in asceding order according to p-value from progressive ALD vs  non-progressive ALD comparison, with molecular features with ID_level of 1-2 first)

| | | | | | | | | all_ALD_vs_control | | | Prog_ALD_vs_non-prog_ALD | | | Prog_ALD_vs_control_ALD | | | Non-prog_ALD_vs_control | | | Healthy_control | | all_ALD | | Non-prog_ALD | | Progressive_ALD | |
|---|---|---|---|---|---|---|---|---|---|---|---|---|---|---|---|---|---|---|---|---|---|---|---|---|---|---|---|
| | | | | | | | | t-test | Cohen's | PLS-DA | t-test | Cohen's | PLS-DA | t-test | Cohen's | PLS-DA | t-test | Cohen's | PLS-DA | | | | | | | | |
| Feature_ID | RP_HILIC | POS_NEG | Average.Rt | Average.Mz | Metabolite.name | curated_MSMS | ID_level | p | d | VIP | p | d | VIP | p | d | VIP | p | d | VIP | Mean | SD | Mean | SD | Mean | SD | Mean | SD |
| RP_POS_997_47577a0_483 | RP | POS | 0.48 | 997.4758 | | | | 1.16E-07 | 0.68 | 1.16 | 1.51E-01 | -0.31 | 1.10 | 2.09E-03 | 0.52 | 1.02 | 2.72E-06 | 0.84 | 1.49 | 233262 | 58196 | 365484 | 211799 | 395995 | 209911 | 334972 | 211354 |
| RP_POS_425_33893a12_021 | RP | POS | 12.02 | 425.3389 | | | | 8.91E-02 | -0.46 | 0.73 | 1.51E-01 | -0.23 | 1.06 | 3.75E-02 | -0.57 | 0.95 | 2.30E-01 | -0.34 | 0.52 | 477230 | 521633 | 308196 | 296669 | 350959 | 339158 | 265432 | 243025 |
| HILIC_NEG_200_85873a5_355 | HILIC | NEG | 5.355 | 200.8587 | | | | 7.79E-01 | 0.06 | 0.41 | 1.51E-01 | -0.29 | 0.92 | 6.96E-01 | -0.09 | 0.18 | 3.73E-01 | 0.20 | 0.55 | 4842801 | 2460778 | 4984123 | 2506535 | 5345061 | 2497694 | 4623185 | 2487832 |
| RP_NEG_204_79147a15_58 | RP | NEG | 15.58 | 204.7915 | | | | 2.59E-01 | -0.23 | 0.50 | 1.51E-01 | -0.29 | 0.92 | 1.05E-01 | -0.38 | 0.69 | 6.91E-01 | -0.09 | 0.36 | 676245 | 31641 | 668934 | 31257 | 673434 | 30211 | 664434 | 31935 |
| RP_POS_685_43463a11_915 | RP | POS | 11.92 | 685.4346 | | | | 1.28E-01 | -0.22 | 0.43 | 1.51E-01 | 0.32 | 1.24 | 6.88E-01 | -0.06 | 0.71 | 6.39E-02 | -0.38 | 0.70 | 4994679 | 491873 | 4788360 | 1024411 | 4640769 | 1178785 | 4935951 | 828410 |
| HILIC_POS_558_29517a1_275 | HILIC | POS | 1.275 | 558.2952 | | | | 4.79E-02 | -0.43 | 1.00 | 1.51E-01 | -0.28 | 0.97 | 1.98E-02 | -0.57 | 0.95 | 2.02E-01 | -0.29 | 0.62 | 1862508 | 547242 | 1642199 | 491605 | 1712925 | 451645 | 1571472 | 523537 |
| RP_POS_872_54028a13_071 | RP | POS | 13.07 | 872.5403 | | | | 6.36E-01 | 0.11 | 0.44 | 1.51E-01 | -0.27 | 1.10 | 9.07E-01 | -0.03 | 0.27 | 3.00E-01 | 0.24 | 0.69 | 6994618 | 2118469 | 7191503 | 1747907 | 7443072 | 1470788 | 6939934 | 1969856 |
| RP_NEG_317_11478a6_043 | RP | NEG | 6.04 | 317.1148 | | | | 8.59E-01 | -0.03 | 0.68 | 1.51E-01 | 0.32 | 1.35 | 5.63E-01 | 0.13 | 0.47 | 1.09E-01 | -0.19 | 0.89 | 940175 | 553187 | 913328 | 1146756 | 747524 | 469793 | 1079132 | 1542767 |
| RP_POS_812_034a13_195 | RP | POS | 13.20 | 812.0340 | | | | 3.51E-03 | 0.61 | 0.98 | 1.52E-01 | 0.28 | 1.08 | 1.17E-03 | 0.75 | 1.31 | 3.78E-02 | 0.47 | 0.79 | 697797 | 379979 | 933031 | 374321 | 879222 | 375133 | 986839 | 369403 |
| RP_POS_323_25537a10_556 | RP | POS | 10.56 | 323.2554 | | | | 4.37E-03 | -0.53 | 1.06 | 1.52E-01 | 0.29 | 1.09 | 6.96E-02 | -0.39 | 0.88 | 1.28E-03 | -0.68 | 1.17 | 5143254 | 574885 | 4778883 | 696453 | 4678773 | 667331 | 4878992 | 717084 |
| RP_NEG_231_93213a15_412 | RP | NEG | 15.41 | 231.9321 | | | | 1.25E-01 | 0.32 | 0.63 | 1.52E-01 | 0.28 | 1.05 | 3.52E-02 | 0.46 | 0.94 | 4.56E-01 | 0.18 | 0.40 | 14971957 | 1914211 | 15572846 | 1836751 | 15309196 | 2094463 | 15836496 | 1512457 |
| RP_POS_293_9454a15_349 | RP | POS | 15.35 | 293.9454 | | | | 7.93E-01 | -0.05 | 0.43 | 1.53E-01 | 0.31 | 1.29 | 5.83E-01 | 0.11 | 0.33 | 3.37E-01 | -0.20 | 0.92 | 2580541 | 516600 | 2549660 | 731713 | 2444729 | 756507 | 2654590 | 697826 |
| HILIC_POS_130_08646a4_124 | HILIC | POS | 4.124 | 130.0865 | | | | 1.82E-02 | -0.47 | 1.44 | 1.53E-01 | 0.29 | 0.94 | 1.34E-01 | -0.33 | 0.69 | 6.10E-03 | -0.62 | 1.46 | 2300848 | 671882 | 1964142 | 708225 | 1862589 | 702534 | 2065694 | 706258 |
| RP_NEG_291_00827a7_31 | RP | NEG | 7.31 | 291.0083 | | | | 5.96E-01 | -0.10 | 0.55 | 1.53E-01 | -0.30 | 0.90 | 2.34E-01 | -0.25 | 0.70 | 8.25E-01 | 0.05 | 0.48 | 2621281 | 537758 | 2560488 | 630535 | 2650905 | 666665 | 2470070 | 584924 |
| RP_POS_331_26279a11_132 | RP | POS | 11.13 | 331.2628 | | | | 1.84E-05 | 0.67 | 1.73 | 1.53E-01 | 0.30 | 1.21 | 7.69E-05 | 0.82 | 1.62 | 2.46E-03 | 0.51 | 1.58 | 530274 | 345943 | 923591 | 621948 | 834437 | 534948 | 1012745 | 692200 |
| HILIC_POS_242_07883a3_016 | HILIC | POS | 3.016 | 242.0788 | | | | 4.25E-10 | 0.81 | 1.48 | 1.53E-01 | 0.31 | 1.24 | 9.52E-07 | 0.96 | 1.56 | 1.95E-05 | 0.65 | 1.51 | 5401856 | 4725854 | 18058296 | 16746692 | 15657985 | 14559132 | 20458607 | 18517571 |
| RP_POS_351_22708a11_132 | RP | POS | 11.13 | 351.2271 | | | | 3.06E-06 | 0.70 | 1.65 | 1.53E-01 | 0.30 | 1.09 | 3.21E-05 | 0.85 | 1.57 | 8.24E-04 | 0.55 | 1.51 | 777081 | 330242 | 1205805 | 643382 | 1113631 | 545991 | 1297978 | 721723 |
| HILIC_POS_167_09274a1_16 | HILIC | POS | 1.16 | 167.0927 | | | | 4.86E-02 | 0.23 | 0.66 | 1.53E-01 | 0.33 | 0.86 | 8.42E-02 | 0.40 | 0.76 | 1.28E-01 | 0.07 | 0.87 | 606439 | 342722 | 1303517 | 3440045 | 807602 | 818952 | 1799433 | 4768306 |
| RP_POS_385_23456a11_095 | RP | POS | 11.10 | 385.2346 | | | | 1.92E-03 | -0.60 | 1.01 | 1.53E-01 | 0.29 | 0.89 | 3.75E-02 | -0.45 | 0.81 | 5.21E-04 | -0.74 | 1.21 | 2123090 | 209835 | 1979507 | 240848 | 1945036 | 225620 | 2013977 | 252724 |
| HILIC_POS_130_09758a1_559 | HILIC | POS | 1.559 | 130.0976 | | | | 4.64E-02 | -0.47 | 1.06 | 1.53E-01 | -0.26 | 0.92 | 1.66E-02 | -0.60 | 1.26 | 1.78E-01 | -0.34 | 0.76 | 795743 | 663206 | 534145 | 502378 | 606039 | 529223 | 462250 | 468287 |
| RP_NEG_351_25143a11_611 | RP | NEG | 11.61 | 351.2514 | | | | 1.62E-01 | -0.25 | 0.63 | 1.54E-01 | -0.30 | 1.09 | 4.41E-02 | -0.40 | 0.77 | 6.51E-01 | -0.10 | 0.58 | 2249002 | 368970 | 2133644 | 491512 | 2203974 | 528673 | 2063314 | 445543 |
| RP_NEG_188_93326a15_487 | RP | NEG | 15.49 | 188.9333 | | | | 4.84E-01 | -0.12 | 0.51 | 1.54E-01 | 0.31 | 1.13 | 8.16E-01 | 0.04 | 0.40 | 2.47E-01 | -0.27 | 0.59 | 2188902 | 166109 | 2161962 | 245687 | 2126696 | 315207 | 2197228 | 141643 |
| RP_NEG_285_16486a8_337 | RP | NEG | 8.34 | 285.1649 | | | | 8.49E-01 | 0.04 | 0.36 | 1.54E-01 | -0.29 | 0.85 | 6.45E-01 | -0.10 | 0.31 | 4.34E-01 | 0.18 | 0.40 | 647741 | 431084 | 664468 | 425565 | 725309 | 442500 | 603627 | 403170 |
| RP_POS_328_06067a6_515 | RP | POS | 6.52 | 328.0607 | | | | 1.44E-03 | 0.42 | 0.86 | 1.54E-01 | -0.32 | 0.87 | 4.46E-02 | 0.26 | 0.73 | 5.61E-03 | 0.58 | 0.96 | 176630 | 648482 | 892125 | 1874218 | 1160560 | 2282676 | 623690 | 1318866 |
| RP_POS_615_4585a11_255 | RP | POS | 11.26 | 615.4585 | | | | 8.11E-01 | 0.04 | 0.41 | 1.54E-01 | -0.32 | 0.98 | 3.26E-01 | -0.12 | 0.53 | 4.13E-01 | 0.19 | 0.63 | 1941857 | 2276345 | 2079963 | 4130193 | 2673570 | 5596257 | 1486356 | 1558173 |
| RP_POS_516_3078a9_748 | RP | POS | 9.75 | 516.3078 | | | | 5.02E-07 | 0.70 | 1.28 | 1.54E-01 | -0.31 | 0.85 | 1.45E-03 | 0.55 | 1.19 | 5.40E-06 | 0.85 | 1.51 | 123353 | 159421 | 379108 | 390904 | 434940 | 400157 | 323277 | 377152 |
| RP_NEG_185_11795a8_007 | RP | NEG | 8.01 | 185.1180 | | | | 9.78E-02 | -0.39 | 0.65 | 1.54E-01 | -0.26 | 0.87 | 2.95E-02 | -0.52 | 0.92 | 3.24E-01 | -0.26 | 0.47 | 1569160 | 1341635 | 1133109 | 1005129 | 1276916 | 1224283 | 989302 | 707187 |
| RP_NEG_253_78329a15_551 | RP | NEG | 15.55 | 253.7833 | | | | 2.23E-01 | -0.25 | 0.65 | 1.54E-01 | -0.28 | 1.15 | 8.83E-02 | -0.40 | 0.68 | 6.22E-01 | -0.11 | 0.59 | 842458 | 54025 | 829040 | 52231 | 836497 | 51796 | 821582 | 52110 |
| HILIC_POS_72_08159a4_858 | HILIC | POS | 4.858 | 72.0816 | | | | 8.15E-01 | -0.04 | 1.02 | 1.54E-01 | -0.30 | 1.13 | 3.70E-01 | -0.19 | 0.66 | 5.93E-01 | 0.11 | 0.70 | 22557803 | 5485903 | 22275804 | 7021675 | 23278430 | 6557301 | 21273179 | 7386764 |
| RP_POS_569_4342a11_165 | RP | POS | 11.17 | 569.4342 | | | | 9.22E-01 | 0.02 | 0.76 | 1.55E-01 | 0.29 | 0.99 | 4.92E-01 | 0.16 | 0.70 | 5.80E-01 | -0.12 | 0.66 | 2199058 | 516368 | 2209338 | 507927 | 2136833 | 455645 | 2281843 | 550367 |
| RP_POS_277_2139a10_463 | RP | POS | 10.46 | 277.2139 | | | | 3.56E-10 | 0.80 | 1.44 | 1.55E-01 | -0.31 | 1.21 | 4.12E-06 | 0.65 | 1.56 | 2.84E-06 | 0.95 | 1.65 | 918918 | 206041 | 1516995 | 799637 | 1631164 | 932808 | 1402826 | 628616 |
| RP_POS_212_16457a7_139 | RP | POS | 7.14 | 212.1646 | | | | 8.30E-01 | 0.05 | 0.61 | 1.55E-01 | -0.26 | 0.88 | 7.67E-01 | -0.08 | 0.54 | 4.76E-01 | 0.18 | 0.48 | 1105347 | 858327 | 1140710 | 619009 | 1228948 | 572420 | 1052473 | 656209 |
| RP_POS_338_01166a4_555 | RP | POS | 4.56 | 338.0117 | | | | 5.78E-03 | -0.60 | 0.94 | 1.55E-01 | -0.27 | 0.97 | 2.48E-03 | -0.73 | 1.14 | 4.17E-02 | -0.46 | 0.79 | 2630212 | 1630410 | 1693252 | 1492169 | 1905918 | 1376289 | 1480586 | 1584962 |
| RP_NEG_317_11484a5_729 | RP | NEG | 5.73 | 317.1148 | | | | 6.61E-01 | -0.06 | 0.65 | 1.55E-01 | 0.32 | 1.31 | 6.84E-01 | 0.09 | 0.44 | 6.55E-02 | -0.22 | 0.90 | 956558 | 544325 | 892014 | 1109203 | 733249 | 496156 | 1050779 | 1479243 |
| RP_NEG_367_24704a10_224 | RP | NEG | 10.22 | 367.2470 | | | | 2.09E-01 | 0.22 | 0.92 | 1.55E-01 | 0.30 | 1.00 | 7.85E-02 | 0.37 | 0.93 | 7.25E-01 | 0.07 | 0.69 | 1485892 | 201898 | 1542855 | 273163 | 1503954 | 258340 | 1581756 | 284485 |
| RP_POS_208_13345a0_714 | RP | POS | 0.71 | 208.1335 | | | | 1.74E-01 | 0.24 | 0.74 | 1.56E-01 | 0.30 | 0.91 | 6.58E-02 | 0.39 | 0.81 | 6.51E-01 | 0.09 | 0.56 | 789328 | 70300 | 810773 | 94351 | 797344 | 88682 | 824201 | 98758 |
| HILIC_POS_306_08121a0_555 | HILIC | POS | 0.555 | 306.0812 | | | | 9.46E-01 | -0.01 | 0.93 | 1.56E-01 | 0.31 | 0.93 | 5.05E-01 | 0.14 | 0.76 | 3.11E-01 | -0.17 | 1.31 | 7817801 | 1263947 | 7796754 | 2132966 | 7492852 | 1604626 | 8100656 | 2535473 |
| RP_POS_446_88773a0_497 | RP | POS | 0.50 | 446.8877 | | | | 2.51E-09 | -0.74 | 1.34 | 1.56E-01 | 0.31 | 1.26 | 3.00E-04 | -0.58 | 1.28 | 1.17E-06 | -0.89 | 1.57 | 32393030 | 1247569 | 27649770 | 6999660 | 26653847 | 7236824 | 28645692 | 6677917 |
| RP_POS_596_60864a14_01 | RP | POS | 14.01 | 596.6086 | | | | 4.75E-01 | 0.17 | 0.69 | 1.56E-01 | -0.27 | 1.21 | 8.96E-01 | 0.03 | 0.66 | 2.36E-01 | 0.30 | 0.84 | 5295419 | 2519773 | 5646662 | 1987503 | 5929168 | 2022223 | 5364156 | 1930982 |
| RP_NEG_289_93567a15_678 | RP | NEG | 15.68 | 289.9357 | | | | 3.86E-02 | -0.42 | 0.77 | 1.56E-01 | 0.28 | 0.87 | 2.20E-01 | -0.27 | 0.50 | 1.26E-02 | -0.56 | 0.96 | 1022668 | 301946 | 891337 | 314251 | 846684 | 304585 | 935989 | 320439 |
| RP_POS_401_76367a12_691 | RP | POS | 12.69 | 401.7637 | | | | 1.71E-03 | 0.60 | 1.17 | 1.56E-01 | -0.29 | 0.87 | 3.61E-02 | 0.45 | 0.89 | 4.69E-04 | 0.74 | 1.28 | 1186786 | 443789 | 1495660 | 519769 | 1569508 | 486037 | 1421812 | 546325 |
| RP_POS_305_9855a4_554 | RP | POS | 4.55 | 305.9855 | | | | 4.94E-03 | -0.61 | 0.95 | 1.56E-01 | -0.27 | 0.97 | 2.14E-03 | -0.74 | 1.17 | 3.69E-02 | -0.47 | 0.81 | 2978357 | 1809835 | 1916808 | 1662254 | 2152981 | 1532957 | 1680636 | 1766006 |
| HILIC_NEG_100_93342a7_429 | HILIC | NEG | 7.429 | 100.9334 | | | | 8.62E-01 | 0.03 | 0.41 | 1.57E-01 | 0.31 | 0.95 | 3.64E-01 | 0.18 | 0.56 | 5.32E-01 | -0.12 | 0.34 | 27008617 | 2529352 | 27110833 | 3762373 | 26576616 | 3699651 | 27645051 | 3785491 |
| RP_POS_363_18268a11_126 | RP | POS | 11.13 | 363.1827 | | | | 2.82E-07 | 0.84 | 1.75 | 1.57E-01 | 0.29 | 1.07 | 2.12E-06 | 0.98 | 1.73 | 9.29E-05 | 0.70 | 1.59 | 910211 | 456214 | 1518638 | 734753 | 1414312 | 651089 | 1622963 | 802878 |
| RP_POS_904_77698a0_493 | RP | POS | 0.49 | 904.7770 | | | | 3.68E-08 | -0.76 | 1.34 | 1.57E-01 | 0.30 | 1.24 | 6.89E-04 | -0.61 | 1.25 | 4.02E-07 | -0.91 | 1.60 | 1254036 | 184552 | 916208 | 474210 | 848899 | 457198 | 983517 | 485863 |
| RP_POS_780_55298a12_693 | RP | POS | 12.69 | 780.5530 | | | | 2.24E-03 | 0.57 | 1.18 | 1.57E-01 | -0.29 | 0.89 | 4.26E-02 | 0.43 | 0.89 | 7.58E-04 | 0.71 | 1.22 | 146960222 | 62414057 | 189621998 | 75740793 | 200363481 | 73908700 | 178880514 | 76762269 |
| RP_POS_258_01196a4_299 | RP | POS | 4.30 | 258.0120 | | | | 9.56E-01 | 0.01 | 0.30 | 1.57E-01 | 0.30 | 0.90 | 5.38E-01 | 0.16 | 0.41 | 3.76E-01 | -0.14 | 0.47 | 5837503 | 27150359 | 6161778 | 32596141 | 1507919 | 2923851 | 10815637 | 45759709 |
| HILIC_NEG_200_94971a0_857 | HILIC | NEG | 0.857 | 200.9497 | | | | 2.24E-01 | 0.15 | 0.61 | 1.58E-01 | 0.32 | 1.12 | 1.73E-01 | 0.31 | 0.46 | 8.78E-01 | -0.01 | 0.18 | 386869 | 496077 | 653757 | 2001877 | 368366 | 575687 | 939149 | 2756660 |
| RP_POS_499_82721a3_138 | RP | POS | 3.14 | 499.8272 | | | | 1.83E-01 | 0.17 | 0.76 | 1.58E-01 | -0.32 | 0.97 | 9.00E-01 | 0.01 | 0.42 | 1.53E-01 | 0.33 | 1.10 | 1589941 | 1420189 | 2326586 | 4894634 | 3024106 | 6763376 | 1629067 | 1291371 |
| RP_POS_136_98967a15_5 | RP | POS | 15.50 | 136.9897 | | | | 8.36E-01 | 0.05 | 0.37 | 1.58E-01 | -0.28 | 0.84 | 7.03E-01 | -0.09 | 0.28 | 4.33E-01 | 0.18 | 0.65 | 62337503 | 24167987 | 63334769 | 21539968 | 66383246 | 19971647 | 60286291 | 22794228 |
| RP_NEG_215_03256a0_549 | RP | NEG | 0.55 | 215.0326 | | | | 1.07E-01 | 0.22 | 0.54 | 1.58E-01 | 0.32 | 0.95 | 8.40E-02 | 0.38 | 0.63 | 6.01E-01 | 0.06 | 0.81 | 205851707 | 21216687 | 216327629 | 52543077 | 208870957 | 30841764 | 223784301 | 67180388 |
| RP_POS_465_37885a10_709 | RP | POS | 10.71 | 465.3789 | | | | 7.89E-01 | -0.06 | 0.94 | 1.58E-01 | -0.28 | 1.19 | 3.77E-01 | -0.20 | 1.23 | 7.30E-01 | 0.08 | 0.60 | 6726027 | 511213 | 6698395 | 485292 | 6767048 | 539260 | 6629743 | 418812 |
| RP_POS_750_54266a13_523 | RP | POS | 13.52 | 750.5427 | | | | 9.03E-01 | -0.02 | 0.69 | 1.59E-01 | -0.29 | 0.84 | 4.31E-01 | -0.17 | 0.43 | 5.91E-01 | 0.12 | 0.75 | 12456323 | 3578688 | 12364587 | 3949827 | 12922488 | 4163701 | 11806687 | 3680904 |
| HILIC_POS_162_1125a5_026 | HILIC | POS | 5.026 | 162.1125 | | | | 4.88E-02 | 0.34 | 0.61 | 1.59E-01 | 0.30 | 0.90 | 1.97E-02 | 0.49 | 0.85 | 3.34E-01 | 0.19 | 0.34 | 14643392 | 11133116 | 19729614 | 16052760 | 17462427 | 15036473 | 21996802 | 16853949 |
| HILIC_POS_585_27039a1_29 | HILIC | POS | 1.29 | 585.2704 | | | | 1.44E-01 | 0.24 | 0.65 | 1.59E-01 | 0.30 | 1.11 | 8.41E-02 | 0.39 | 0.57 | 5.74E-01 | 0.09 | 0.24 | 1589284 | 1087586 | 1962531 | 1648114 | 1729352 | 1103770 | 2195709 | 2039294 |
| RP_POS_972_76611a0_491 | RP | POS | 0.49 | 972.7661 | | | | 3.42E-07 | -0.75 | 1.25 | 1.59E-01 | 0.30 | 1.16 | 1.76E-03 | -0.60 | 1.11 | 2.57E-07 | -0.90 | 1.57 | 899220 | 140451 | 691952 | 289879 | 651033 | 256830 | 732871 | 316853 |
| HILIC_NEG_117_05573a0_603 | HILIC | NEG | 0.603 | 117.0557 | | | | 1.02E-04 | 0.50 | 0.98 | 1.60E-01 | 0.31 | 1.20 | 2.81E-03 | 0.66 | 0.92 | 1.39E-03 | 0.35 | 1.07 | 497864 | 159215 | 739007 | 531299 | 663853 | 292900 | 814162 | 687750 |
| RP_POS_156_0416a0_549 | RP | POS | 0.55 | 156.0416 | | | | 1.47E-03 | -0.45 | 1.09 | 1.60E-01 | 0.31 | 1.22 | 1.16E-01 | -0.30 | 1.01 | 4.03E-04 | -0.61 | 1.31 | 2455138 | 477376 | 2009846 | 1074879 | 1858350 | 971161 | 2161342 | 1159446 |
| HILIC_NEG_215_03285a4_804 | HILIC | NEG | 4.804 | 215.0329 | | | | 7.90E-02 | 0.27 | 0.54 | 1.60E-01 | 0.31 | 0.98 | 5.75E-02 | 0.42 | 0.71 | 4.44E-01 | 0.11 | 0.53 | 2307616 | 589745 | 2578347 | 1114134 | 2421042 | 736324 | 2735651 | 1383926 |
| RP_POS_345_25681a10_65 | RP | POS | 10.65 | 345.2568 | | | | 1.97E-01 | -0.19 | 0.64 | 1.60E-01 | 0.31 | 0.86 | 8.92E-01 | -0.03 | 0.45 | 4.88E-03 | -0.34 | 1.09 | 2235273 | 322682 | 2119992 | 680406 | 2023737 | 317588 | 2216246 | 903096 |
| RP_POS_441_29816a15_229 | RP | POS | 15.23 | 441.2982 | | | | 8.86E-01 | 0.03 | 0.31 | 1.60E-01 | 0.29 | 1.28 | 4.45E-01 | 0.17 | 0.41 | 5.95E-01 | -0.12 | 0.62 | 13246780 | 6222905 | 13432950 | 6745482 | 12483793 | 6431850 | 14382107 | 6980286 |
| RP_POS_293_04486a7_72 | RP | POS | 7.72 | 293.0449 | | | | 5.85E-01 | -0.07 | 0.56 | 1.61E-01 | 0.32 | 0.99 | 6.81E-01 | 0.09 | 0.65 | 2.27E-04 | -0.23 | 1.55 | 1742638 | 807313 | 1528381 | 3641284 | 1012699 | 861662 | 2044063 | 5050081 |
| RP_POS_787_47137a10_886 | RP | POS | 10.89 | 787.4714 | | | | 9.15E-02 | 0.20 | 0.79 | 1.61E-01 | -0.32 | 1.03 | 7.51E-01 | 0.04 | 0.62 | 5.94E-02 | 0.36 | 0.97 | 2550097 | 213983 | 2763019 | 1192542 | 2930751 | 1370945 | 2595286 | 967693 |
| HILIC_POS_325_17117a0_523 | HILIC | POS | 0.523 | 325.1712 | | | | 3.94E-02 | 0.35 | 0.89 | 1.61E-01 | 0.30 | 0.97 | 2.24E-02 | 0.50 | 0.85 | 2.61E-01 | 0.20 | 0.45 | 5436278 | 3400020 | 7060765 | 4889523 | 6373313 | 4014410 | 7748218 | 5587739 |
| RP_POS_610_30902a10_325 | RP | POS | 10.33 | 610.3090 | | | | 2.17E-01 | -0.29 | 0.82 | 1.61E-01 | -0.26 | 1.01 | 9.52E-02 | -0.42 | 0.83 | 5.20E-01 | -0.16 | 0.63 | 1580294 | 546406 | 1448787 | 410911 | 1506516 | 425888 | 1391057 | 391100 |
| RP_POS_260_00888a4_299 | RP | POS | 4.30 | 260.0089 | | | | 9.65E-01 | -0.01 | 0.25 | 1.61E-01 | 0.29 | 0.89 | 5.92E-01 | 0.14 | 0.35 | 3.22E-01 | -0.16 | 0.52 | 840129 | 4435938 | 798020 | 5279948 | 50806 | 22666 | 1545234 | 7428638 |
| HILIC_POS_176_12799a5_341 | HILIC | POS | 5.341 | 176.1280 | | | | 5.82E-01 | 0.11 | 0.27 | 1.61E-01 | 0.29 | 0.98 | 2.72E-01 | 0.25 | 0.44 | 8.62E-01 | -0.04 | 0.48 | 454864 | 481643 | 510399 | 530374 | 435894 | 479513 | 584904 | 571903 |
| HILIC_POS_288_28952a0_524 | HILIC | POS | 0.524 | 288.2895 | | | | 3.64E-01 | -0.19 | 0.47 | 1.61E-01 | 0.28 | 0.90 | 8.18E-01 | -0.06 | 0.33 | 1.45E-01 | -0.33 | 0.57 | 644365 | 122486 | 621923 | 114067 | 605899 | 102281 | 637947 | 123704 |
| RP_POS_386_19916a10_857 | RP | POS | 10.86 | 386.1992 | | | | 1.37E-01 | -0.31 | 0.94 | 1.61E-01 | -0.28 | 0.91 | 5.79E-02 | -0.45 | 0.92 | 4.37E-01 | -0.17 | 0.65 | 2253674 | 672614 | 2049122 | 643407 | 2139492 | 598620 | 1958752 | 679249 |
| RP_POS_334_30774a11_603 | RP | POS | 11.60 | 334.3077 | | | | 5.08E-08 | 0.77 | 1.32 | 1.62E-01 | 0.30 | 1.32 | 2.21E-05 | 0.92 | 1.43 | 6.92E-06 | 0.62 | 1.44 | 2851277 | 1036079 | 4626927 | 2442451 | 4283473 | 1658611 | 4970381 | 3010176 |
| RP_POS_165_05449a1_268 | RP | POS | 1.27 | 165.0545 | | | | 2.42E-01 | 0.20 | 0.95 | 1.62E-01 | 0.30 | 1.07 | 1.12E-01 | 0.35 | 1.13 | 7.76E-01 | 0.05 | 0.60 | 3227659 | 788060 | 3437923 | 1112138 | 3281778 | 905553 | 3594067 | 1276385 |
| RP_NEG_403_16251a11_163 | RP | NEG | 11.16 | 403.1625 | | | | 9.55E-01 | -0.01 | 0.59 | 1.62E-01 | -0.30 | 1.00 | 4.49E-01 | -0.16 | 0.39 | 4.71E-01 | 0.14 | 0.64 | 2357467 | 113791 | 2356012 | 159738 | 2378418 | 147195 | 2333606 | 169889 |
| RP_POS_301_1181a5_921 | RP | POS | 5.92 | 301.1181 | | | | 7.61E-01 | -0.05 | 0.68 | 1.62E-01 | 0.31 | 1.34 | 6.34E-01 | 0.11 | 0.48 | 1.24E-01 | -0.20 | 0.85 | 650769 | 372918 | 621110 | 713319 | 520670 | 361932 | 721550 | 936189 |
| RP_POS_301_87332a15_557 | RP | POS | 15.56 | 301.8733 | | | | 5.89E-01 | 0.12 | 0.57 | 1.62E-01 | -0.27 | 0.88 | 9.33E-01 | -0.02 | 0.13 | 2.96E-01 | 0.25 | 0.47 | 1494936 | 77663 | 1503314 | 69714 | 1513085 | 73514 | 1493544 | 64963 |
| HILIC_POS_256_01511a0_541 | HILIC | POS | 0.541 | 256.0151 | | | | 9.02E-01 | 0.02 | 0.67 | 1.62E-01 | 0.31 | 0.93 | 3.55E-01 | 0.17 | 0.33 | 4.20E-01 | -0.14 | 0.93 | 187422625 | 20814794 | 188168218 | 47559358 | 181504710 | 44545529 | 194831726 | 49950225 |
| HILIC_POS_583_25488a0_525 | HILIC | POS | 0.525 | 583.2549 | | | | 2.87E-03 | 0.43 | 0.93 | 1.62E-01 | 0.31 | 1.22 | 7.40E-03 | 0.59 | 0.97 | 4.48E-02 | 0.28 | 1.04 | 644068 | 429832 | 1008329 | 922186 | 878856 | 611726 | 1137803 | 1144460 |
| RP_POS_619_61267a11_246 | RP | POS | 11.25 | 619.6127 | | | | 4.65E-06 | 0.57 | 1.03 | 1.63E-01 | 0.31 | 1.32 | 1.14E-03 | 0.73 | 1.13 | 8.98E-06 | 0.42 | 1.33 | 2987950 | 4282327 | 12561046 | 18490219 | 9960349 | 8855381 | 15161742 | 24464926 |
| RP_POS_150_96902a15_487 | RP | POS | 15.49 | 150.9690 | | | | 3.28E-01 | 0.19 | 0.93 | 1.63E-01 | 0.29 | 0.95 | 1.61E-01 | 0.33 | 0.86 | 8.17E-01 | 0.05 | 0.67 | 50466974 | 7916390 | 52099992 | 8829065 | 50862219 | 6790187 | 53337765 | 10404917 |
| HILIC_POS_266_16507a0_637 | HILIC | POS | 0.637 | 266.1651 | | | | 1.19E-01 | 0.18 | 0.42 | 1.63E-01 | 0.32 | 0.85 | 1.40E-01 | 0.34 | 0.64 | 1.50E-02 | 0.02 | 0.82 | 4639 | 3167 | 1057505 | 6696614 | 113057 | 304182 | 2001952 | 9417620 |
| RP_POS_223_09453a10_282 | RP | POS | 10.28 | 223.0945 | | | | 7.32E-01 | 0.07 | 0.25 | 1.63E-01 | -0.27 | 0.83 | 7.82E-01 | -0.06 | 0.20 | 3.84E-01 | 0.21 | 0.41 | 6497565 | 2221869 | 6649841 | 2029878 | 6933769 | 2160278 | 6365913 | 1869112 |
| RP_POS_721_43457a10_915 | RP | POS | 10.92 | 721.4346 | | | | 1.87E-05 | 0.58 | 1.25 | 1.63E-01 | -0.31 | 1.06 | 6.96E-04 | 0.43 | 1.29 | 7.14E-04 | 0.74 | 1.19 | 1200400 | 393360 | 1754359 | 1032941 | 1899137 | 1297067 | 1609580 | 656162 |
| RP_NEG_199_80464a15_584 | RP | NEG | 15.58 | 199.8046 | | | | 1.21E-01 | -0.32 | 0.64 | 1.63E-01 | -0.28 | 1.00 | 4.05E-02 | -0.46 | 0.82 | 4.31E-01 | -0.18 | 0.39 | 16560734 | 491215 | 16404346 | 478404 | 16471231 | 511206 | 16337461 | 438121 |
| RP_NEG_243_75459a15_587 | RP | NEG | 15.59 | 243.7546 | | | | 1.47E-01 | -0.28 | 0.59 | 1.63E-01 | -0.29 | 0.98 | 4.40E-02 | -0.42 | 0.80 | 5.55E-01 | -0.13 | 0.26 | 10131479 | 318933 | 10032416 | 369832 | 10084119 | 399962 | 9980713 | 333042 |
| RP_POS_184_0732a10_501 | RP | POS | 10.50 | 184.0732 | | | | 6.23E-02 | 0.37 | 1.11 | 1.64E-01 | -0.28 | 1.23 | 3.11E-01 | 0.23 | 0.80 | 1.74E-02 | 0.51 | 1.16 | 2283940 | 470429 | 2467879 | 492154 | 2536654 | 434881 | 2399105 | 539074 |
| RP_POS_941_55951a10_836 | RP | POS | 10.84 | 941.5595 | | | | 6.65E-07 | 0.61 | 1.19 | 1.64E-01 | -0.31 | 1.13 | 1.72E-04 | 0.45 | 1.39 | 2.66E-04 | 0.76 | 1.37 | 381955 | 116904 | 765330 | 698205 | 863062 | 856828 | 667599 | 480920 |
| HILIC_POS_188_13931a0_727 | HILIC | POS | 0.727 | 188.1393 | | | | 1.68E-01 | 0.16 | 0.46 | 1.64E-01 | -0.32 | 0.83 | 5.64E-01 | 0.00 | 0.21 | 1.66E-01 | 0.32 | 0.42 | 16143 | 9892 | 118373 | 736245 | 221949 | 1035936 | 14798 | 10798 |
| RP_POS_133_03468a10_571 | RP | POS | 10.57 | 133.0347 | | | | 4.15E-02 | 0.38 | 0.69 | 1.64E-01 | -0.29 | 0.84 | 2.90E-01 | 0.24 | 0.55 | 8.68E-03 | 0.53 | 1.16 | 1707188 | 632514 | 1987960 | 753313 | 2093222 | 626853 | 1882698 | 854976 |
| RP_POS_230_17509a7_139 | RP | POS | 7.14 | 230.1751 | | | | 7.77E-01 | 0.07 | 0.59 | 1.64E-01 | -0.26 | 0.87 | 8.18E-01 | -0.06 | 0.53 | 4.39E-01 | 0.20 | 0.48 | 1018731 | 798320 | 1062227 | 586667 | 1144130 | 544827 | 980324 | 620366 |

**Supplementary table 1: Metabolomics results** (in asceding order according to p-value from progressive ALD vs  non-progressive ALD comparison, with molecular features with ID_level of 1-2 first)

| Feature_ID | RP_HILIC | POS_NEG | Average.Rt | Average.Mz | Metabolite.name | curated_MSMS | ID_level | all_ALD_vs_control | | | Prog_ALD_vs_non-prog_ALD | | | Prog_ALD_vs_control_ALD | | | Non-prog_ALD_vs_control | | | Healthy_control | | all_ALD | | Non-prog_ALD | | Progressive_ALD | |
|---|---|---|---|---|---|---|---|---|---|---|---|---|---|---|---|---|---|---|---|---|---|---|---|---|---|---|---|
| | | | | | | | | t-test p | Cohen's d | PLS-DA VIP | t-test p | Cohen's d | PLS-DA VIP | t-test p | Cohen's d | PLS-DA VIP | t-test p | Cohen's d | PLS-DA VIP | Mean | SD | Mean | SD | Mean | SD | Mean | SD |
| RP_POS_225_11047a6_819 | RP | POS | 6.82 | 225.1105 | | | | 2.55E-01 | 0.25 | 0.61 | 1.64E-01 | 0.27 | 0.84 | 1.18E-01 | 0.38 | 0.67 | 6.24E-01 | 0.11 | 0.48 | 1574800 | 307718 | 1644996 | 273618 | 1606798 | 250629 | 1683195 | 292350 |
| RP_POS_323_99603a4_552 | RP | POS | 4.55 | 323.9960 | | | | 2.21E-03 | -0.65 | 1.01 | 1.64E-01 | -0.27 | 0.96 | 1.04E-03 | -0.78 | 1.23 | 2.06E-02 | -0.51 | 0.87 | 853143 | 496689 | 530872 | 476704 | 597337 | 439412 | 464407 | 506984 |
| HILIC_POS_375_32153a4_054 | HILIC | POS | 4.054 | 375.3215 | | | | 9.18E-01 | 0.02 | 0.43 | 1.65E-01 | -0.28 | 0.81 | 5.97E-01 | -0.12 | 0.30 | 4.51E-01 | 0.16 | 0.42 | 4427326 | 1226150 | 4453644 | 1306040 | 4635644 | 1196885 | 4271643 | 1395047 |
| RP_POS_799_39441a7_549 | RP | POS | 7.55 | 799.3944 | | | | 8.42E-01 | -0.04 | 0.85 | 1.65E-01 | 0.28 | 0.98 | 6.79E-01 | 0.10 | 0.74 | 4.46E-01 | -0.18 | 0.88 | 645409 | 47669 | 643491 | 44797 | 637250 | 45897 | 649733 | 43225 |
| RP_POS_575_49164a14_002 | RP | POS | 14.00 | 575.4916 | | | | 9.53E-01 | 0.01 | 0.28 | 1.65E-01 | -0.28 | 0.93 | 5.94E-01 | -0.13 | 0.38 | 4.90E-01 | 0.15 | 0.48 | 2447747 | 822727 | 2457674 | 810601 | 2570634 | 712737 | 2344714 | 890799 |
| RP_POS_600_46643a10_463 | RP | POS | 10.46 | 600.4664 | | | | 5.87E-02 | -0.36 | 0.95 | 1.65E-01 | 0.28 | 0.91 | 3.40E-01 | -0.22 | 0.68 | 1.21E-02 | -0.51 | 1.13 | 4037282 | 398280 | 3875942 | 452356 | 3812896 | 357894 | 3938987 | 526525 |
| RP_POS_805_55402a12_82 | RP | POS | 12.82 | 805.5540 | | | | 2.16E-06 | -1.16 | 1.87 | 1.65E-01 | -0.22 | 0.93 | 7.51E-07 | -1.27 | 1.94 | 2.12E-05 | -1.06 | 1.62 | 27415116 | 9954002 | 16997826 | 6961583 | 17966118 | 6915136 | 16029533 | 6941453 |
| RP_POS_685_4342a11_61 | RP | POS | 11.61 | 685.4342 | | | | 8.81E-01 | -0.02 | 0.61 | 1.65E-01 | 0.30 | 0.92 | 4.55E-01 | 0.13 | 0.57 | 4.07E-01 | -0.18 | 0.71 | 2785540 | 283920 | 2775134 | 476404 | 2708821 | 545671 | 2841447 | 389643 |
| HILIC_NEG_247_95482a0_833 | HILIC | NEG | 0.833 | 247.9548 | | | | 8.30E-01 | -0.04 | 0.79 | 1.65E-01 | -0.29 | 1.15 | 3.88E-01 | -0.19 | 0.56 | 5.83E-01 | 0.11 | 0.57 | 407610 | 104615 | 402643 | 137825 | 421813 | 126818 | 383472 | 146787 |
| RP_POS_474_25967a10_139 | RP | POS | 10.14 | 474.2597 | | | | 2.89E-04 | 0.55 | 1.10 | 1.66E-01 | 0.30 | 0.91 | 3.51E-04 | 0.70 | 1.32 | 2.29E-02 | 0.41 | 0.82 | 1217650 | 962887 | 2145328 | 1788023 | 1896753 | 1684468 | 2393902 | 1869696 |
| HILIC_POS_269_12582a1_435 | HILIC | POS | 1.435 | 269.1258 | | | | 9.67E-02 | -0.46 | 0.94 | 1.66E-01 | -0.22 | 0.83 | 4.47E-02 | -0.57 | 1.06 | 2.27E-01 | -0.35 | 0.55 | 747879 | 861230 | 476905 | 457195 | 540485 | 523318 | 413325 | 374446 |
| RP_NEG_217_02945a0_549 | RP | NEG | 0.55 | 217.0295 | | | | 1.23E-01 | 0.21 | 0.53 | 1.66E-01 | 0.31 | 0.93 | 9.48E-02 | 0.37 | 0.62 | 6.46E-01 | 0.06 | 0.82 | 69921387 | 7077748 | 73311269 | 17888681 | 70818459 | 10520143 | 75804078 | 22873245 |
| HILIC_POS_400_34122a0_615 | HILIC | POS | 0.615 | 400.3412 | | | | 1.40E-04 | 0.73 | 1.52 | 1.66E-01 | -0.27 | 0.87 | 3.37E-03 | 0.60 | 1.33 | 1.08E-04 | 0.87 | 1.61 | 1330227 | 534583 | 1789514 | 615992 | 1875101 | 664877 | 1703926 | 556439 |
| RP_NEG_493_24509a9_147 | RP | NEG | 9.15 | 493.2451 | | | | 4.62E-02 | 0.25 | 0.80 | 1.66E-01 | 0.31 | 1.37 | 6.35E-02 | 0.41 | 0.95 | 3.27E-01 | 0.10 | 0.96 | 1836321 | 1358599 | 2915871 | 4794336 | 2247510 | 2406866 | 3584232 | 6303631 |
| HILIC_NEG_230_0128a0_626 | HILIC | NEG | 0.626 | 230.0128 | | | | 8.23E-02 | 0.20 | 0.46 | 1.66E-01 | -0.32 | 0.82 | 2.24E-01 | 0.04 | 0.44 | 1.15E-01 | 0.36 | 0.47 | 22437 | 50786 | 191569 | 959462 | 325680 | 1336630 | 57459 | 191409 |
| HILIC_NEG_200_85867a7_017 | HILIC | NEG | 7.017 | 200.8587 | | | | 2.27E-01 | -0.26 | 0.47 | 1.66E-01 | -0.27 | 0.97 | 8.70E-02 | -0.39 | 0.84 | 6.05E-01 | -0.12 | 0.20 | 2892691 | 1271902 | 2582429 | 1167419 | 2744413 | 1244461 | 2420444 | 1072993 |
| RP_NEG_870_54944a13_159 | RP | NEG | 13.16 | 870.5494 | | | | 4.29E-01 | 0.16 | 0.34 | 1.67E-01 | 0.28 | 0.88 | 1.77E-01 | 0.30 | 0.63 | 9.39E-01 | 0.02 | 0.42 | 7658154 | 2653201 | 8093184 | 2795157 | 7705533 | 2876391 | 8480835 | 2684202 |
| RP_NEG_385_04672a8_81 | RP | NEG | 8.81 | 385.0467 | | | | 4.05E-03 | 0.38 | 0.94 | 1.67E-01 | 0.31 | 0.87 | 1.29E-02 | 0.54 | 0.92 | 5.96E-02 | 0.23 | 0.91 | 988374 | 407958 | 1384525 | 1145546 | 1225234 | 712839 | 1543817 | 1446170 |
| RP_POS_194_00798a15_625 | RP | POS | 15.63 | 194.0080 | | | | 5.13E-01 | -0.13 | 0.46 | 1.67E-01 | -0.28 | 1.02 | 2.24E-01 | -0.27 | 0.47 | 9.59E-01 | 0.01 | 0.52 | 5846218 | 2034819 | 5569586 | 2171051 | 5870617 | 2171676 | 5268554 | 2149884 |
| RP_POS_1058_77356a0_491 | RP | POS | 0.49 | 1058.7736 | | | | 6.53E-03 | 0.35 | 0.99 | 1.67E-01 | 0.31 | 1.33 | 8.68E-03 | 0.51 | 1.09 | 1.87E-01 | 0.20 | 1.05 | 747080 | 175229 | 924404 | 561695 | 846518 | 480154 | 1002289 | 628103 |
| HILIC_POS_126_05524a0_723 | HILIC | POS | 0.723 | 126.0552 | | | | 1.66E-01 | 0.16 | 0.46 | 1.67E-01 | -0.32 | 0.82 | 3.91E-01 | 0.00 | 0.35 | 1.67E-01 | 0.32 | 0.41 | 24219 | 15198 | 4185100 | 29837579 | 8348766 | 41992276 | 21434 | 12572 |
| RP_NEG_230_01279a2_224 | RP | NEG | 2.22 | 230.0128 | | | | 1.50E-02 | 0.30 | 0.48 | 1.67E-01 | -0.32 | 0.88 | 2.65E-02 | 0.14 | 0.86 | 4.80E-02 | 0.45 | 0.58 | 1266175 | 1635806 | 3451643 | 8370366 | 4617755 | 11523825 | 2285530 | 2445860 |
| RP_POS_429_37219a11_237 | RP | POS | 11.24 | 429.3722 | | | | 1.66E-01 | -0.25 | 0.72 | 1.67E-01 | -0.29 | 0.98 | 8.52E-02 | -0.40 | 0.77 | 5.64E-01 | -0.11 | 0.30 | 890416 | 353876 | 782776 | 446011 | 844582 | 342446 | 720969 | 526161 |
| HILIC_POS_98_06068a0_723 | HILIC | POS | 0.723 | 98.0607 | | | | 1.65E-01 | 0.16 | 0.46 | 1.68E-01 | -0.32 | 0.82 | 1.42E-01 | 0.00 | 0.53 | 1.67E-01 | 0.32 | 0.41 | 15897 | 16307 | 1420764 | 10031711 | 2819357 | 14118491 | 22171 | 21882 |
| RP_POS_182_02361a11_209 | RP | POS | 11.21 | 182.0236 | | | | 4.93E-03 | -0.59 | 1.33 | 1.68E-01 | -0.27 | 0.94 | 2.09E-03 | -0.73 | 1.24 | 3.97E-02 | -0.46 | 1.26 | 1420880 | 147070 | 1333986 | 140353 | 1353411 | 133238 | 1314561 | 145869 |
| HILIC_NEG_243_96844a1_3 | HILIC | NEG | 1.3 | 243.9684 | | | | 2.11E-07 | 0.87 | 1.42 | 1.68E-01 | 0.28 | 1.25 | 5.65E-06 | 1.01 | 1.46 | 1.08E-05 | 0.73 | 1.71 | 40971977 | 7133853 | 50428955 | 10888877 | 48919651 | 7819885 | 51938260 | 13181633 |
| RP_POS_665_47351a10_851 | RP | POS | 10.85 | 665.4735 | | | | 8.25E-01 | 0.04 | 0.87 | 1.68E-01 | -0.28 | 1.12 | 6.87E-01 | -0.10 | 0.77 | 3.83E-01 | 0.18 | 0.92 | 1478982 | 733259 | 1512267 | 759171 | 1617375 | 634040 | 1407159 | 860169 |
| RP_NEG_297_15439a12_418 | RP | NEG | 12.42 | 297.1544 | | | | 7.79E-01 | -0.06 | 0.63 | 1.68E-01 | 0.28 | 0.90 | 7.14E-01 | 0.09 | 0.54 | 3.40E-01 | -0.20 | 0.62 | 1554214 | 967022 | 1497569 | 1048175 | 1352443 | 861813 | 1642694 | 1197521 |
| RP_POS_425_78445a14_328 | RP | POS | 14.33 | 425.7845 | | | | 8.43E-01 | -0.04 | 0.44 | 1.68E-01 | 0.26 | 0.88 | 7.29E-01 | 0.09 | 0.52 | 4.50E-01 | -0.18 | 0.42 | 1072578 | 728450 | 1044123 | 610391 | 959642 | 520251 | 1128603 | 683763 |
| RP_POS_850_71875a0_5 | RP | POS | 0.50 | 850.7188 | | | | 1.98E-01 | 0.25 | 0.78 | 1.68E-01 | -0.28 | 1.28 | 6.32E-01 | 0.10 | 0.46 | 7.51E-02 | 0.39 | 0.92 | 511732 | 249373 | 580186 | 287131 | 619904 | 286767 | 540467 | 284809 |
| RP_POS_428_80298a10_653 | RP | POS | 10.65 | 428.8030 | | | | 9.65E-01 | -0.01 | 0.25 | 1.68E-01 | 0.27 | 0.90 | 5.86E-01 | 0.13 | 0.71 | 5.22E-01 | -0.15 | 0.40 | 775652 | 295648 | 773000 | 282311 | 733953 | 270768 | 812046 | 290858 |
| RP_NEG_195_81117a15_584 | RP | NEG | 15.58 | 195.8112 | | | | 1.66E-01 | -0.29 | 0.66 | 1.68E-01 | -0.28 | 0.98 | 5.71E-02 | -0.42 | 0.78 | 5.29E-01 | -0.15 | 0.45 | 28277556 | 844486 | 28037297 | 837922 | 28153186 | 904741 | 27921407 | 756697 |
| RP_POS_287_19672a0_612 | RP | POS | 0.61 | 287.1967 | | | | 2.28E-01 | 0.19 | 1.07 | 1.68E-01 | 0.30 | 1.18 | 1.05E-01 | 0.34 | 1.34 | 8.35E-01 | 0.04 | 0.55 | 2991040 | 4044770 | 4226401 | 7244475 | 3224740 | 6052174 | 5228062 | 8207284 |
| HILIC_POS_62_06092a3_773 | HILIC | POS | 3.773 | 62.0609 | | | | 1.62E-05 | 0.59 | 1.11 | 1.68E-01 | 0.30 | 1.00 | 2.97E-04 | 0.74 | 1.25 | 2.65E-03 | 0.44 | 1.12 | 2316430 | 616615 | 3173685 | 1568935 | 2956800 | 1236462 | 3390570 | 1829894 |
| HILIC_POS_177_07468a1_233 | HILIC | POS | 1.233 | 177.0747 | | | | 1.71E-06 | -1.07 | 1.94 | 1.69E-01 | -0.24 | 0.88 | 2.02E-06 | -1.18 | 1.84 | 1.49E-05 | -0.95 | 2.16 | 721217 | 154255 | 555096 | 133862 | 573609 | 97407 | 536583 | 161296 |
| RP_NEG_262_99371a8_012 | RP | NEG | 8.01 | 262.9937 | | | | 1.00E-01 | -0.32 | 0.54 | 1.69E-01 | -0.28 | 0.94 | 3.14E-02 | -0.46 | 0.86 | 4.31E-01 | -0.17 | 0.48 | 4592413 | 224835 | 4513442 | 255473 | 4548721 | 270385 | 4478164 | 237100 |
| RP_POS_133_03165a0_71 | RP | POS | 0.71 | 133.0317 | | | | 4.20E-01 | -0.10 | 0.46 | 1.69E-01 | 0.32 | 0.94 | 8.09E-01 | 0.05 | 0.28 | 1.95E-03 | -0.26 | 1.41 | 4671177 | 616754 | 4475827 | 2156950 | 4176397 | 770357 | 4775256 | 2936561 |
| RP_POS_172_13304a10_271 | RP | POS | 10.27 | 172.1330 | | | | 7.53E-01 | -0.06 | 0.81 | 1.69E-01 | 0.28 | 0.83 | 7.53E-01 | 0.08 | 0.78 | 3.25E-01 | -0.20 | 0.62 | 8178793 | 461579 | 8148923 | 476707 | 8083054 | 363830 | 8214791 | 563835 |
| RP_POS_341_26624a10_536 | RP | POS | 10.54 | 341.2662 | | | | 6.38E-01 | -0.09 | 0.53 | 1.69E-01 | 0.28 | 0.88 | 8.16E-01 | 0.05 | 0.44 | 2.90E-01 | -0.23 | 0.53 | 3010690 | 694676 | 2941921 | 774595 | 2835034 | 777868 | 3048807 | 764098 |
| RP_POS_185_03024a0_713 | RP | POS | 0.71 | 185.0302 | | | | 9.42E-05 | 0.62 | 1.05 | 1.69E-01 | 0.29 | 0.85 | 3.66E-04 | 0.76 | 1.25 | 4.32E-03 | 0.47 | 0.98 | 968251 | 171541 | 1141404 | 296262 | 1100506 | 235021 | 1182301 | 344507 |
| RP_POS_1047_7334a10_932 | RP | POS | 10.93 | 1047.7334 | | | | 1.02E-01 | -0.41 | 1.04 | 1.69E-01 | -0.24 | 1.43 | 4.98E-02 | -0.53 | 0.93 | 2.65E-01 | -0.29 | 0.85 | 32602795 | 19053242 | 26571692 | 12936956 | 28356065 | 11914605 | 24787319 | 13772782 |
| RP_POS_199_13045a12_888 | RP | POS | 12.89 | 199.1305 | | | | 7.10E-01 | -0.08 | 0.51 | 1.69E-01 | -0.27 | 0.93 | 3.81E-01 | -0.22 | 0.63 | 8.03E-01 | 0.06 | 0.18 | 20966252 | 9335289 | 20269680 | 8595685 | 21455345 | 7422830 | 19084015 | 9555736 |
| RP_POS_577_00537a10_874 | RP | POS | 10.87 | 577.0054 | | | | 2.74E-01 | 0.14 | 0.75 | 1.69E-01 | -0.31 | 0.90 | 8.76E-01 | -0.02 | 0.45 | 1.50E-01 | 0.29 | 0.82 | 548038 | 87192 | 589035 | 339662 | 635930 | 411262 | 542140 | 243865 |
| RP_POS_319_12885a5_731 | RP | POS | 5.73 | 319.1289 | | | | 7.07E-01 | -0.06 | 0.66 | 1.69E-01 | 0.31 | 1.30 | 6.69E-01 | 0.10 | 0.47 | 1.02E-01 | -0.21 | 0.86 | 885988 | 499525 | 835619 | 1004256 | 696657 | 510625 | 974582 | 1318141 |
| RP_POS_604_37885a11_108 | RP | POS | 11.11 | 604.3789 | | | | 2.07E-07 | 0.62 | 1.29 | 1.69E-01 | -0.31 | 1.29 | 6.29E-04 | 0.47 | 1.33 | 8.34E-05 | 0.77 | 1.41 | 22774 | 21988 | 328199 | 546039 | 403510 | 626940 | 252888 | 444718 |
| RP_POS_126_05495a0_71 | RP | POS | 0.71 | 126.0550 | | | | 1.68E-01 | 0.16 | 0.58 | 1.69E-01 | -0.32 | 0.85 | 5.22E-01 | 0.00 | 0.77 | 1.69E-01 | 0.32 | 0.58 | 3907 | 2878 | 717946 | 5141090 | 1431444 | 7236164 | 4448 | 4731 |
| RP_POS_511_29587a11_476 | RP | POS | 11.48 | 511.2959 | | | | 2.41E-01 | -0.22 | 0.57 | 1.70E-01 | 0.29 | 1.01 | 7.30E-01 | -0.07 | 0.50 | 8.43E-02 | -0.36 | 0.74 | 2038793 | 110965 | 2010653 | 134297 | 1992151 | 127595 | 2029154 | 139501 |
| HILIC_NEG_234_82022a6_157 | HILIC | NEG | 6.157 | 234.8202 | | | | 6.02E-02 | 0.35 | 1.55 | 1.70E-01 | 0.28 | 1.56 | 2.86E-02 | 0.50 | 1.57 | 2.91E-01 | 0.21 | 1.15 | 872943 | 145001 | 932034 | 171916 | 908345 | 150261 | 955723 | 189710 |
| RP_POS_429_31924a10_451 | RP | POS | 10.45 | 429.3192 | | | | 9.59E-02 | -0.32 | 0.75 | 1.70E-01 | 0.28 | 1.11 | 4.34E-01 | -0.17 | 0.51 | 2.80E-02 | -0.46 | 0.93 | 31599547 | 1934915 | 30906368 | 2257176 | 30595517 | 2036160 | 31217220 | 2439367 |
| HILIC_POS_64_01661a2_223 | HILIC | POS | 2.223 | 64.0166 | | | | 9.33E-01 | -0.02 | 0.61 | 1.70E-01 | -0.28 | 0.93 | 4.80E-01 | -0.16 | 0.34 | 5.67E-01 | 0.12 | 0.35 | 3125229 | 765039 | 3111858 | 841014 | 3227605 | 816131 | 2996112 | 857690 |
| RP_POS_818_60266a14_573 | RP | POS | 14.57 | 818.6027 | | | | 4.68E-05 | -0.87 | 1.49 | 1.70E-01 | -0.25 | 0.81 | 8.66E-06 | -1.00 | 1.71 | 1.58E-03 | -0.75 | 1.35 | 2538753 | 1052624 | 1599666 | 982327 | 1734938 | 1119460 | 1464394 | 811844 |
| RP_POS_408_16104a4_275 | RP | POS | 4.28 | 408.1610 | | | | 5.67E-02 | 0.27 | 0.70 | 1.70E-01 | 0.31 | 0.97 | 5.12E-02 | 0.42 | 0.91 | 3.91E-01 | 0.12 | 0.49 | 1679095 | 628857 | 2030786 | 1450562 | 1830812 | 963486 | 2230760 | 1800357 |
| HILIC_NEG_161_02644a1_297 | HILIC | NEG | 1.297 | 161.0264 | | | | 4.02E-06 | 0.76 | 1.32 | 1.70E-01 | 0.28 | 0.93 | 5.30E-06 | 0.90 | 1.56 | 1.28E-03 | 0.61 | 1.06 | 341130 | 113651 | 474558 | 181160 | 449650 | 180580 | 499466 | 180090 |
| RP_POS_230_96573a15_325 | RP | POS | 15.33 | 230.9657 | | | | 1.84E-01 | -0.20 | 0.44 | 1.70E-01 | -0.30 | 0.98 | 6.55E-02 | -0.35 | 0.74 | 7.80E-01 | -0.05 | 0.37 | 1020679 | 51485 | 1002938 | 96320 | 1016181 | 93698 | 989695 | 98017 |
| RP_POS_599_88562a11_096 | RP | POS | 11.10 | 599.8856 | | | | 4.81E-07 | 0.60 | 1.29 | 1.71E-01 | -0.31 | 1.26 | 4.76E-04 | 0.45 | 1.34 | 1.88E-04 | 0.75 | 1.38 | 82135 | 32927 | 414734 | 615228 | 499384 | 729422 | 330085 | 466965 |
| RP_NEG_159_10199a6_563 | RP | NEG | 6.56 | 159.1020 | | | | 2.32E-02 | 0.34 | 0.94 | 1.71E-01 | 0.30 | 1.06 | 3.37E-02 | 0.49 | 0.87 | 1.42E-01 | 0.19 | 1.13 | 1059093 | 548626 | 1396149 | 1096229 | 1244937 | 559413 | 1547362 | 1438177 |
| RP_POS_1058_72009a10_931 | RP | POS | 10.93 | 1058.7201 | | | | 2.28E-02 | -0.52 | 1.14 | 1.71E-01 | -0.25 | 1.42 | 7.61E-03 | -0.65 | 1.21 | 1.11E-01 | -0.40 | 0.93 | 427719 | 257719 | 309689 | 207142 | 338154 | 222903 | 281224 | 188021 |
| HILIC_NEG_202_92886a4_213 | HILIC | NEG | 4.213 | 202.9289 | | | | 1.16E-01 | -0.32 | 0.69 | 1.71E-01 | 0.28 | 0.99 | 4.28E-01 | -0.18 | 0.49 | 4.20E-02 | -0.45 | 0.86 | 3453653 | 1015203 | 3121565 | 1050764 | 2977207 | 1014806 | 3265923 | 1076282 |
| HILIC_NEG_500_27789a1_711 | HILIC | NEG | 1.711 | 500.2779 | | | | 2.15E-06 | 0.86 | 1.45 | 1.71E-01 | -0.27 | 0.77 | 8.43E-04 | 0.72 | 1.17 | 2.86E-07 | 0.99 | 2.05 | 468475 | 165350 | 656859 | 216217 | 686574 | 175548 | 627144 | 248665 |
| HILIC_POS_144_10185a3_51 | HILIC | POS | 3.51 | 144.1019 | | | | 1.98E-01 | 0.22 | 0.51 | 1.71E-01 | 0.29 | 0.95 | 8.10E-02 | 0.36 | 0.66 | 7.14E-01 | 0.07 | 0.28 | 493483418 | 565301033 | 662929831 | 837530419 | 547907021 | 770563639 | 777952640 | 892446655 |
| RP_POS_782_73035a0_501 | RP | POS | 0.50 | 782.7304 | | | | 9.30E-01 | 0.02 | 0.83 | 1.71E-01 | -0.29 | 1.25 | 4.87E-01 | -0.13 | 0.58 | 4.43E-01 | 0.16 | 0.96 | 479628 | 227993 | 484244 | 328700 | 529389 | 355872 | 439099 | 295785 |
| HILIC_NEG_262_98703a2_172 | HILIC | NEG | 2.172 | 262.9870 | | | | 4.58E-01 | 0.14 | 0.53 | 1.71E-01 | -0.28 | 1.00 | 9.95E-01 | 0.00 | 0.24 | 2.19E-01 | 0.28 | 0.50 | 838920 | 385688 | 899352 | 436410 | 959291 | 488334 | 839413 | 372817 |
| RP_NEG_462_17764a6_604 | RP | NEG | 6.60 | 462.1776 | | | | 3.67E-03 | 0.46 | 1.55 | 1.71E-01 | -0.29 | 0.83 | 7.74E-02 | 0.31 | 1.33 | 3.21E-03 | 0.61 | 1.12 | 1031435 | 1219816 | 1929704 | 2103558 | 2218471 | 2299887 | 1640937 | 1865627 |
| RP_POS_549_30853a10_522 | RP | POS | 10.52 | 549.3085 | | | | 4.71E-01 | -0.21 | 0.57 | 1.71E-01 | -0.21 | 0.84 | 2.92E-01 | -0.31 | 0.54 | 7.30E-01 | -0.10 | 0.57 | 552513 | 600949 | 472310 | 292985 | 512505 | 313169 | 432115 | 268440 |
| RP_POS_537_39258a13_626 | RP | POS | 13.63 | 537.3926 | | | | 4.86E-01 | -0.16 | 0.50 | 1.71E-01 | 0.26 | 0.80 | 9.20E-01 | -0.02 | 0.27 | 2.28E-01 | -0.29 | 0.68 | 798377 | 340526 | 751481 | 287480 | 712049 | 262975 | 790913 | 307648 |
| HILIC_POS_156_07684a7_096 | HILIC | POS | 7.096 | 156.0768 | | | | 7.70E-01 | -0.05 | 1.04 | 1.72E-01 | -0.29 | 1.04 | 3.12E-01 | -0.20 | 1.08 | 6.54E-01 | 0.10 | 0.90 | 1374039 | 508316 | 1340516 | 702216 | 1436796 | 752535 | 1244236 | 641132 |
| HILIC_NEG_149_04254a0_928 | HILIC | NEG | 0.928 | 149.0425 | | | | 5.71E-05 | 0.57 | 0.94 | 1.72E-01 | -0.30 | 1.27 | 3.00E-02 | 0.42 | 0.70 | 1.37E-05 | 0.72 | 1.47 | 426044 | 208065 | 677206 | 475846 | 742439 | 405240 | 611974 | 533457 |
| RP_POS_365_17801a11_125 | RP | POS | 11.13 | 365.1780 | | | | 5.18E-07 | 0.81 | 1.70 | 1.72E-01 | 0.28 | 1.03 | 3.94E-06 | 0.95 | 1.67 | 1.59E-04 | 0.67 | 1.55 | 316404 | 157031 | 522450 | 259909 | 486868 | 232168 | 558033 | 282840 |
| RP_POS_321_23993a10_005 | RP | POS | 10.01 | 321.2399 | | | | 7.30E-11 | 0.86 | 1.50 | 1.73E-01 | 0.29 | 1.45 | 3.03E-06 | 1.01 | 1.52 | 2.24E-08 | 0.72 | 1.78 | 1092323 | 552069 | 2481547 | 1695925 | 2249082 | 1113593 | 2714012 | 2112024 |
| RP_POS_161_13258a8_589 | RP | POS | 8.59 | 161.1326 | | | | 8.33E-01 | 0.05 | 0.32 | 1.73E-01 | 0.26 | 0.83 | 4.68E-01 | 0.18 | 0.60 | 7.22E-01 | -0.08 | 0.40 | 13132306 | 1646167 | 13200655 | 1408601 | 13008017 | 1340423 | 13393292 | 1461622 |
| RP_POS_267_19308a9_919 | RP | POS | 9.92 | 267.1931 | | | | 1.85E-04 | 0.58 | 1.55 | 1.73E-01 | 0.29 | 1.25 | 2.21E-04 | 0.73 | 1.49 | 1.62E-02 | 0.44 | 1.29 | 916257 | 222758 | 1133556 | 395983 | 1079427 | 378065 | 1187686 | 409747 |
| HILIC_POS_249_06113a4_123 | HILIC | POS | 4.123 | 249.0611 | | | | 5.15E-03 | -0.46 | 1.20 | 1.73E-01 | -0.29 | 1.43 | 5.00E-03 | -0.61 | 1.29 | 7.06E-02 | -0.31 | 1.18 | 1328035 | 195408 | 1195388 | 306152 | 1237263 | 250732 | 1153513 | 350607 |
| RP_POS_602_36322a11_096 | RP | POS | 11.10 | 602.3632 | | | | 3.40E-07 | 0.61 | 1.30 | 1.73E-01 | -0.30 | 1.29 | 5.39E-04 | 0.46 | 1.36 | 1.37E-04 | 0.76 | 1.40 | 76902 | 90868 | 1040894 | 1757408 | 1281053 | 2054795 | 800735 | 1378442 |
| RP_POS_266_15903a4_569 | RP | POS | 4.57 | 266.1590 | | | | 1.96E-01 | 0.19 | 0.37 | 1.73E-01 | 0.30 | 0.95 | 1.45E-01 | 0.34 | 0.57 | 7.67E-01 | 0.03 | 0.33 | 1086787 | 72923 | 1113445 | 159480 | 1091569 | 67635 | 1135322 | 214093 |
| HILIC_POS_133_98238a5_19 | HILIC | POS | 5.19 | 133.9824 | | | | 5.30E-02 | 0.30 | 0.59 | 1.74E-01 | 0.30 | 0.82 | 4.44E-02 | 0.44 | 0.67 | 3.25E-01 | 0.15 | 0.38 | 23585 | 31114 | 39209 | 57809 | 31302 | 39044 | 47117 | 71414 |
| RP_POS_816_55237a13_022 | RP | POS | 13.02 | 816.5524 | | | | 3.73E-05 | -0.84 | 1.65 | 1.74E-01 | -0.26 | 0.87 | 1.34E-05 | -0.97 | 1.74 | 1.28E-03 | -0.71 | 1.43 | 1997026 | 680718 | 1370638 | 702063 | 1466420 | 723971 | 1274856 | 673043 |
| RP_POS_774_54211a13_381 | RP | POS | 13.38 | 774.5421 | | | | 4.16E-03 | -0.53 | 1.21 | 1.74E-01 | 0.28 | 0.87 | 9.68E-02 | -0.39 | 0.79 | 3.32E-04 | -0.67 | 1.37 | 3030068 | 991309 | 2391841 | 1242230 | 2222112 | 841726 | 2561569 | 1533123 |
| RP_POS_187_1321a10_38 | RP | POS | 10.38 | 187.1321 | | | | 7.66E-01 | 0.07 | 0.69 | 1.74E-01 | 0.26 | 1.09 | 4.38E-01 | 0.20 | 0.55 | 7.88E-01 | -0.06 | 0.57 | 2531039 | 297325 | 2548445 | 247326 | 2514717 | 210801 | 2582173 | 277182 |
| HILIC_POS_283_17523a0_615 | HILIC | POS | 0.615 | 283.1752 | | | | 9.01E-01 | 0.03 | 1.05 | 1.74E-01 | -0.24 | 1.04 | 7.41E-01 | -0.09 | 0.85 | 5.98E-01 | 0.15 | 1.05 | 589173 | 1539473 | 625452 | 972776 | 758197 | 1172356 | 492706 | 708180 |
| RP_NEG_380_92154a15_428 | RP | NEG | 15.43 | 380.9215 | | | | 6.43E-01 | -0.09 | 0.29 | 1.74E-01 | -0.27 | 0.96 | 2.88E-01 | -0.23 | 0.66 | 8.57E-01 | 0.04 | 0.44 | 1092494 | 54410 | 1087336 | 54918 | 1094818 | 60336 | 1079854 | 48360 |
| RP_POS_548_38831a11_476 | RP | POS | 11.48 | 548.3883 | | | | 5.84E-01 | -0.09 | 0.47 | 1.75E-01 | 0.29 | 0.96 | 8.01E-01 | 0.05 | 0.42 | 2.45E-01 | -0.24 | 0.54 | 2990458 | 175914 | 2968705 | 244873 | 2935368 | 249208 | 3002042 | 238278 |
| RP_POS_553_45856a12_423 | RP | POS | 12.42 | 553.4586 | | | | 6.19E-01 | 0.10 | 0.51 | 1.75E-01 | 0.27 | 0.84 | 3.00E-01 | 0.24 | 0.58 | 8.73E-01 | -0.04 | 0.42 | 10727554 | 4710575 | 11207343 | 4776066 | 10557218 | 4611831 | 11857468 | 4894445 |
| HILIC_POS_118_08667a4_858 | HILIC | POS | 4.858 | 118.0867 | | | | 7.91E-01 | -0.05 | 1.02 | 1.75E-01 | -0.29 | 1.11 | 3.75E-01 | -0.19 | 0.67 | 6.51E-01 | 0.09 | 0.75 | 83253906 | 22891366 | 81943148 | 28227241 | 85784576 | 27013463 | 78101721 | 29154286 |
| RP_POS_1063_67004a10_198 | RP | POS | 10.20 | 1063.6700 | | | | 3.35E-01 | -0.25 | 0.91 | 1.75E-01 | -0.23 | 1.26 | 1.88E-01 | -0.37 | 0.75 | 6.06E-01 | -0.14 | 0.84 | 512716 | 656901 | 393088 | 390809 | 446269 | 376412 | 339907 | 401399 |
| RP_POS_552_33136a10_932 | RP | POS | 10.93 | 552.3314 | | | | 4.02E-02 | -0.48 | 1.21 | 1.75E-01 | -0.25 | 1.63 | 1.72E-02 | -0.61 | 1.16 | 1.47E-01 | -0.36 | 0.99 | 6301263 | 1888046 | 5531327 | 1452587 | 5728977 | 1412790 | 5333677 | 1478980 |
| RP_POS_798_06024a13_378 | RP | POS | 13.38 | 798.0602 | | | | 8.68E-01 | -0.03 | 0.55 | 1.75E-01 | -0.28 | 0.80 | 4.32E-01 | -0.17 | 0.45 | 6.32E-01 | 0.11 | 0.36 | 991194 | 441380 | 975862 | 481730 | 1041388 | 491585 | 910336 | 467381 |
| RP_NEG_508_34042a10_934 | RP | NEG | 10.93 | 508.3404 | | | | 1.53E-01 | -0.34 | 1.06 | 1.75E-01 | -0.25 | 1.60 | 7.49E-02 | -0.46 | 0.98 | 3.88E-01 | -0.21 | 0.87 | 2268941 | 625659 | 2093781 | 477997 | 2158795 | 434316 | 2028768 | 514169 |
| RP_POS_749_3819a4_792 | RP | POS | 4.79 | 749.3819 | | | | 8.44E-08 | 0.66 | 1.17 | 1.75E-01 | 0.30 | 1.25 | 8.45E-06 | 0.81 | 1.46 | 1.48E-03 | 0.51 | 1.14 | 365258 | 156029 | 869007 | 837005 | 755246 | 800190 | 982767 | 865295 |

**Supplementary table 1: Metabolomics results** (in asceding order according to p-value from progressive ALD vs  non-progressive ALD comparison, with molecular features with ID_level of 1-2 first)

| | | | | | | | | all_ALD_vs_control | | | Prog_ALD_vs_non-prog_ALD | | | Prog_ALD_vs_control_ALD | | | Non-prog_ALD_vs_control | | | Healthy_control | | all_ALD | | Non-prog_ALD | | Progressive_ALD | |
|---|---|---|---|---|---|---|---|---|---|---|---|---|---|---|---|---|---|---|---|---|---|---|---|---|---|---|---|
| | | | | | | | | t-test | Cohen's | PLS-DA | t-test | Cohen's | PLS-DA | t-test | Cohen's | PLS-DA | t-test | Cohen's | PLS-DA | | | | | | | | |
| Feature_ID | RP_HILIC | POS_NEG | Average.Rt | Average.Mz | Metabolite.name | curated_MSMS | ID_level | p | d | VIP | p | d | VIP | p | d | VIP | p | d | VIP | Mean | SD | Mean | SD | Mean | SD | Mean | SD |
| RP_POS_379_25018a12_766 | RP | POS | 12.77 | 379.2502 | | | | 6.77E-02 | -0.35 | 0.76 | 1.76E-01 | 0.28 | 0.89 | 3.43E-01 | -0.21 | 0.50 | 2.20E-02 | -0.49 | 0.90 | 2423625 | 633437 | 2173852 | 740459 | 2073372 | 701357 | 2274333 | 771520 |
| RP_POS_591_68073a10_88 | RP | POS | 10.88 | 591.6807 | | | | 9.09E-01 | -0.01 | 0.87 | 1.76E-01 | -0.31 | 0.87 | 2.55E-01 | -0.17 | 0.65 | 4.43E-01 | 0.14 | 0.83 | 774488 | 70567 | 770186 | 354229 | 818248 | 390844 | 722124 | 309910 |
| RP_POS_317_05591a0_587 | RP | POS | 0.59 | 317.0559 | | | | 8.12E-01 | -0.05 | 0.60 | 1.77E-01 | -0.27 | 1.13 | 3.80E-01 | -0.19 | 0.48 | 7.06E-01 | 0.09 | 0.75 | 697280 | 662408 | 664808 | 689351 | 758292 | 782290 | 571324 | 574713 |
| RP_POS_441_29712a11_645 | RP | POS | 11.65 | 441.2971 | | | | 6.04E-01 | -0.11 | 0.38 | 1.77E-01 | -0.27 | 1.09 | 1.97E-01 | -0.24 | 0.76 | 9.22E-01 | 0.03 | 0.32 | 28012144 | 22101145 | 25698701 | 21049259 | 28570373 | 29476974 | 22827030 | 3076249 |
| HILIC_POS_371_2406a7_098 | HILIC | POS | 7.098 | 371.2406 | | | | 9.81E-01 | 0.00 | 1.03 | 1.77E-01 | -0.28 | 1.06 | 5.32E-01 | -0.13 | 0.97 | 5.18E-01 | 0.14 | 0.91 | 2053342 | 529347 | 2055996 | 590585 | 2136021 | 609764 | 1975971 | 565524 |
| RP_POS_424_36362a10_539 | RP | POS | 10.54 | 424.3636 | | | | 1.19E-01 | -0.29 | 0.72 | 1.77E-01 | 0.28 | 0.83 | 4.94E-01 | -0.15 | 0.46 | 3.24E-02 | -0.43 | 0.92 | 1067382 | 192972 | 1002734 | 226051 | 972101 | 191915 | 1033367 | 253957 |
| RP_POS_824_55511a13_999 | RP | POS | 14.00 | 824.5551 | | | | 2.85E-02 | -0.51 | 0.98 | 1.77E-01 | 0.25 | 0.89 | 1.33E-01 | -0.38 | 0.82 | 9.04E-03 | -0.63 | 1.11 | 7734896 | 2730693 | 6534776 | 2186904 | 6238461 | 1874140 | 6831091 | 2443585 |
| RP_POS_319_2244a9_925 | RP | POS | 9.93 | 319.2244 | | | | 1.39E-11 | 0.97 | 1.61 | 1.77E-01 | -0.27 | 1.37 | 2.02E-06 | 0.83 | 1.57 | 4.06E-09 | 1.11 | 1.87 | 1349306 | 304650 | 1997967 | 676367 | 2089536 | 684754 | 1906397 | 662028 |
| RP_POS_807_51276a12_688 | RP | POS | 12.69 | 807.5128 | | | | 1.11E-03 | 0.57 | 1.22 | 1.77E-01 | -0.28 | 0.87 | 3.56E-02 | 0.43 | 0.93 | 4.01E-04 | 0.71 | 1.23 | 615885 | 472835 | 976477 | 653956 | 1064980 | 622312 | 887974 | 678813 |
| HILIC_NEG_238_07747a0_929 | HILIC | NEG | 0.929 | 238.0775 | | | | 1.45E-05 | 0.57 | 1.04 | 1.78E-01 | 0.30 | 1.02 | 1.13E-03 | 0.72 | 1.10 | 1.58E-04 | 0.43 | 1.55 | 3182108 | 2710066 | 7408821 | 8062619 | 6314836 | 4438364 | 8502806 | 10449726 |
| HILIC_POS_174_11252a4_87 | HILIC | POS | 4.87 | 174.1125 | | | | 4.59E-01 | -0.18 | 0.75 | 1.78E-01 | 0.25 | 0.98 | 8.35E-01 | -0.05 | 0.13 | 2.35E-01 | -0.30 | 0.78 | 561191 | 675352 | 464399 | 498743 | 396968 | 468488 | 531831 | 523264 |
| HILIC_POS_179_00494a3_757 | HILIC | POS | 3.757 | 179.0049 | | | | 2.66E-01 | 0.13 | 0.47 | 1.78E-01 | 0.31 | 1.38 | 2.13E-01 | 0.29 | 0.58 | 5.27E-01 | -0.02 | 0.35 | 1220806 | 1234515 | 2294202 | 9354636 | 1021257 | 1599416 | 3567148 | 13074375 |
| RP_POS_792_5882a13_244 | RP | POS | 13.24 | 792.5882 | | | | 6.18E-02 | -0.46 | 0.95 | 1.78E-01 | -0.24 | 0.84 | 2.53E-02 | -0.58 | 0.98 | 1.92E-01 | -0.34 | 0.69 | 4831882 | 2507183 | 3918516 | 1724637 | 4151623 | 1847866 | 3685408 | 1576053 |
| RP_POS_418_74777a12_821 | RP | POS | 12.82 | 418.7478 | | | | 8.87E-06 | -1.03 | 1.67 | 1.78E-01 | -0.23 | 0.89 | 3.10E-06 | -1.15 | 1.81 | 1.30E-04 | -0.92 | 1.44 | 6350818 | 2159410 | 4248887 | 1716772 | 4480778 | 1752264 | 4016995 | 1665710 |
| RP_POS_582_59283a14_006 | RP | POS | 14.01 | 582.5928 | | | | 9.09E-01 | 0.03 | 0.61 | 1.78E-01 | -0.25 | 1.16 | 7.07E-01 | -0.10 | 0.58 | 5.65E-01 | 0.15 | 0.67 | 1121603 | 905105 | 1141390 | 656209 | 1230026 | 688698 | 1052754 | 616175 |
| RP_POS_465_29639a11_153 | RP | POS | 11.15 | 465.2964 | | | | 2.29E-03 | -0.47 | 0.99 | 1.78E-01 | 0.29 | 1.00 | 5.28E-02 | -0.32 | 0.97 | 3.36E-03 | -0.61 | 1.11 | 1314838 | 49010 | 1275375 | 91541 | 1263003 | 104285 | 1287746 | 75782 |
| RP_NEG_915_12708a9_653 | RP | NEG | 9.65 | 915.1271 | | | | 2.80E-01 | -0.21 | 0.47 | 1.78E-01 | -0.28 | 0.82 | 1.01E-01 | -0.34 | 0.62 | 7.65E-01 | -0.07 | 0.36 | 5384238 | 323321 | 5309877 | 372870 | 5360232 | 396890 | 5259522 | 343811 |
| HILIC_POS_310_14163a0_518 | HILIC | POS | 0.518 | 310.1416 | | | | 2.77E-01 | 0.14 | 0.88 | 1.78E-01 | -0.31 | 0.81 | 7.94E-01 | -0.02 | 0.29 | 2.10E-01 | 0.29 | 0.82 | 905592 | 1431079 | 1546998 | 5298530 | 2266798 | 7374933 | 827198 | 1129064 |
| RP_POS_300_28876a10_691 | RP | POS | 10.69 | 300.2888 | | | | 1.27E-13 | 1.11 | 1.88 | 1.79E-01 | -0.26 | 0.97 | 1.24E-08 | 0.97 | 1.80 | 3.21E-10 | 1.24 | 2.07 | 726690 | 160296 | 1090410 | 320735 | 1133681 | 342501 | 1047139 | 294470 |
| RP_POS_433_7717a14_327 | RP | POS | 14.33 | 433.7717 | | | | 4.27E-01 | 0.18 | 0.43 | 1.79E-01 | 0.25 | 0.82 | 2.27E-01 | 0.31 | 0.60 | 8.12E-01 | 0.06 | 0.25 | 1667200 | 981162 | 1819784 | 778798 | 1714832 | 695878 | 1924736 | 847765 |
| RP_NEG_339_23337a10_658 | RP | NEG | 10.66 | 339.2334 | | | | 6.72E-05 | 0.63 | 1.00 | 1.79E-01 | -0.28 | 0.87 | 2.97E-03 | 0.49 | 1.04 | 3.13E-04 | 0.77 | 1.13 | 5258817 | 4343421 | 9724865 | 7436043 | 10727053 | 8681017 | 8722676 | 5857313 |
| RP_POS_420_24786a12_822 | RP | POS | 12.82 | 420.2479 | | | | 2.68E-05 | -1.01 | 1.67 | 1.80E-01 | -0.22 | 1.03 | 1.04E-05 | -1.12 | 1.74 | 2.66E-04 | -0.89 | 1.46 | 1980344 | 845086 | 1217416 | 628454 | 1301948 | 623241 | 1132884 | 628456 |
| HILIC_NEG_234_82027a3_049 | HILIC | NEG | 3.049 | 234.8203 | | | | 2.64E-01 | 0.20 | 0.90 | 1.80E-01 | -0.29 | 0.97 | 7.81E-01 | 0.05 | 0.26 | 1.21E-01 | 0.34 | 0.59 | 604284 | 89294 | 626725 | 121591 | 643084 | 134573 | 610367 | 105899 |
| RP_POS_390_7088a0_519 | RP | POS | 0.52 | 390.7088 | | | | 9.84E-03 | -0.40 | 1.00 | 1.80E-01 | 0.29 | 0.99 | 1.73E-01 | -0.26 | 0.74 | 4.20E-03 | -0.55 | 1.15 | 342713 | 61366 | 302337 | 108226 | 287783 | 107103 | 316891 | 108451 |
| HILIC_POS_153_06589a0_74 | HILIC | POS | 0.74 | 153.0659 | | | | 2.56E-01 | -0.20 | 0.47 | 1.80E-01 | -0.28 | 0.82 | 1.11E-01 | -0.34 | 0.55 | 7.59E-01 | -0.06 | 0.25 | 8817576 | 4410032 | 7705061 | 5795001 | 8484445 | 5290885 | 6925678 | 6214200 |
| RP_POS_246_86243a15_458 | RP | POS | 15.46 | 246.8624 | | | | 2.75E-01 | -0.26 | 0.43 | 1.80E-01 | -0.25 | 1.10 | 1.39E-01 | -0.38 | 0.61 | 5.92E-01 | -0.13 | 0.41 | 2571960 | 798882 | 2401495 | 615175 | 2484182 | 573324 | 2318808 | 649578 |
| RP_NEG_307_85663a1_013 | RP | NEG | 1.01 | 307.8566 | | | | 3.25E-01 | 0.19 | 0.57 | 1.80E-01 | -0.28 | 0.82 | 8.28E-01 | 0.05 | 0.27 | 1.11E-01 | 0.33 | 0.64 | 31847 | 12854 | 34537 | 14744 | 36519 | 12658 | 32555 | 16462 |
| HILIC_NEG_134_89482a4_126 | HILIC | NEG | 4.126 | 134.8948 | | | | 8.98E-01 | -0.03 | 0.39 | 1.81E-01 | 0.26 | 1.12 | 6.76E-01 | 0.10 | 0.25 | 5.03E-01 | -0.16 | 0.35 | 19144809 | 9893394 | 18894370 | 8639757 | 17734205 | 8143183 | 20054535 | 9041894 |
| RP_POS_315_66678a10_932 | RP | POS | 10.93 | 315.6668 | | | | 4.56E-04 | -0.82 | 1.66 | 1.81E-01 | -0.24 | 1.58 | 1.81E-04 | -0.93 | 1.64 | 3.98E-03 | -0.70 | 1.42 | 3465003 | 878006 | 2820778 | 694671 | 2914041 | 688806 | 2727515 | 694823 |
| RP_NEG_135_02882a0_569 | RP | NEG | 0.57 | 135.0288 | | | | 8.86E-01 | 0.02 | 0.45 | 1.81E-01 | -0.29 | 0.86 | 5.38E-01 | -0.12 | 0.52 | 4.05E-01 | 0.17 | 0.53 | 1561395 | 505114 | 1578263 | 754207 | 1679515 | 772328 | 1477011 | 729281 |
| RP_POS_384_34857a11_063 | RP | POS | 11.06 | 384.3486 | | | | 2.30E-05 | -0.67 | 1.05 | 1.81E-01 | 0.28 | 0.86 | 3.68E-03 | -0.53 | 1.02 | 4.60E-05 | -0.81 | 1.25 | 2474809 | 63424 | 2405056 | 108907 | 2390435 | 113309 | 2419677 | 103384 |
| RP_POS_406_7872a10_655 | RP | POS | 10.66 | 406.7872 | | | | 1.06E-01 | -0.34 | 1.21 | 1.81E-01 | 0.27 | 0.83 | 3.85E-01 | -0.20 | 0.63 | 3.82E-02 | -0.47 | 1.13 | 792145 | 307490 | 690060 | 300964 | 649680 | 280310 | 730439 | 317972 |
| RP_POS_166_98486a10_888 | RP | POS | 10.89 | 166.9849 | | | | 3.25E-05 | -0.78 | 1.29 | 1.82E-01 | 0.26 | 0.91 | 3.37E-03 | -0.64 | 1.05 | 5.62E-06 | -0.91 | 1.55 | 13990508 | 3627641 | 10501106 | 4432818 | 9906749 | 3703388 | 11095464 | 5026404 |
| HILIC_POS_375_32175a0_528 | HILIC | POS | 0.528 | 375.3218 | | | | 3.30E-03 | -0.59 | 1.42 | 1.82E-01 | -0.26 | 0.79 | 2.48E-03 | -0.72 | 1.29 | 2.42E-02 | -0.46 | 1.14 | 3150912 | 765528 | 2668069 | 796764 | 2774873 | 635088 | 2561265 | 925205 |
| RP_POS_583_25421a11_668 | RP | POS | 11.67 | 583.2542 | | | | 4.05E-01 | -0.17 | 0.57 | 1.82E-01 | 0.27 | 1.10 | 8.87E-01 | -0.04 | 0.30 | 1.09E-01 | -0.31 | 0.88 | 1041983 | 2038570 | 695473 | 2012338 | 424573 | 756545 | 966373 | 2731212 |
| RP_NEG_388_87476a15_403 | RP | NEG | 15.40 | 388.8748 | | | | 3.44E-01 | -0.19 | 0.65 | 1.82E-01 | 0.27 | 0.84 | 7.82E-01 | -0.06 | 0.65 | 1.75E-01 | -0.33 | 0.53 | 1417133 | 221765 | 1374047 | 223872 | 1344052 | 255806 | 1404042 | 184357 |
| HILIC_NEG_431_58038a1_366 | HILIC | NEG | 1.366 | 431.5804 | | | | 2.99E-10 | -0.93 | 1.53 | 1.82E-01 | -0.27 | 0.89 | 3.14E-08 | -1.07 | 1.79 | 5.03E-06 | -0.80 | 1.61 | 244530 | 39509 | 170633 | 80318 | 181384 | 76428 | 159882 | 83407 |
| HILIC_NEG_136_89172a3_131 | HILIC | NEG | 3.131 | 136.8917 | | | | 1.51E-04 | -0.64 | 1.10 | 1.82E-01 | 0.28 | 0.95 | 9.20E-03 | -0.50 | 1.02 | 1.20E-04 | -0.78 | 1.30 | 34391022 | 7579479 | 27436035 | 11177159 | 25940334 | 11337452 | 28931736 | 10922636 |
| RP_POS_343_29544a8_988 | RP | POS | 8.99 | 343.2954 | | | | 9.91E-02 | -0.39 | 0.84 | 1.82E-01 | -0.25 | 0.89 | 2.49E-02 | -0.51 | 1.11 | 3.38E-01 | -0.26 | 0.67 | 1124509 | 271590 | 1036194 | 210070 | 1064426 | 280872 | 1007962 | 92972 |
| RP_POS_199_13034a13_497 | RP | POS | 13.50 | 199.1303 | | | | 5.61E-01 | 0.10 | 0.60 | 1.82E-01 | -0.29 | 1.04 | 8.27E-01 | -0.04 | 0.58 | 2.56E-01 | 0.24 | 0.43 | 20255200 | 7215005 | 21204602 | 10054230 | 22549718 | 10941085 | 19859486 | 8991008 |
| RP_NEG_207_83698a1_323 | RP | NEG | 1.32 | 207.8370 | | | | 2.89E-02 | 0.39 | 0.67 | 1.83E-01 | -0.28 | 0.91 | 2.08E-01 | 0.25 | 0.74 | 1.40E-02 | 0.53 | 0.96 | 2902755 | 1191524 | 3492762 | 1598703 | 3706460 | 1701039 | 3279063 | 1475511 |
| RP_POS_776_57794a11_889 | RP | POS | 11.89 | 776.5779 | | | | 1.88E-09 | 0.73 | 1.52 | 1.83E-01 | 0.29 | 0.92 | 2.31E-05 | 0.88 | 1.52 | 3.04E-06 | 0.59 | 1.45 | 161069 | 114815 | 736247 | 853416 | 622003 | 609394 | 850491 | 1036101 |
| HILIC_POS_256_16983a0_517 | HILIC | POS | 0.517 | 256.1698 | | | | 2.10E-01 | 0.14 | 0.38 | 1.83E-01 | 0.31 | 0.77 | 1.96E-01 | 0.30 | 0.53 | 1.24E-01 | -0.01 | 0.92 | 143389 | 129162 | 713324 | 4513211 | 105902 | 48241 | 1320747 | 6355984 |
| RP_POS_203_99384a15_42 | RP | POS | 15.42 | 203.9938 | | | | 3.67E-01 | 0.19 | 0.65 | 1.83E-01 | -0.26 | 0.84 | 7.92E-01 | 0.06 | 0.46 | 1.58E-01 | 0.32 | 0.77 | 3298769 | 811592 | 3445781 | 738799 | 3544530 | 669370 | 3347033 | 796763 |
| HILIC_NEG_183_93427a2_945 | HILIC | NEG | 2.945 | 183.9343 | | | | 2.49E-03 | -0.54 | 0.86 | 1.83E-01 | 0.28 | 0.93 | 5.60E-02 | -0.40 | 0.73 | 8.16E-04 | -0.67 | 1.17 | 483736 | 82192 | 426416 | 110728 | 411619 | 104129 | 441212 | 116111 |
| RP_NEG_892_79675a0_492 | RP | NEG | 0.49 | 892.7968 | | | | 6.62E-10 | -0.77 | 1.39 | 1.84E-01 | 0.29 | 1.28 | 1.63E-04 | -0.63 | 1.36 | 3.72E-07 | -0.92 | 1.60 | 4830368 | 380114 | 3537066 | 1806210 | 3296090 | 1820198 | 3778041 | 1777565 |
| RP_POS_343_19147a11_16 | RP | POS | 11.16 | 343.1915 | | | | 4.11E-01 | -0.14 | 0.68 | 1.84E-01 | 0.28 | 0.99 | 9.88E-01 | 0.00 | 0.55 | 1.63E-01 | -0.29 | 0.80 | 1349606 | 101492 | 1330907 | 137220 | 1312615 | 135445 | 1349200 | 137894 |
| RP_POS_391_3399a10_88 | RP | POS | 10.88 | 391.3399 | | | | 5.36E-01 | 0.10 | 0.66 | 1.84E-01 | -0.29 | 0.98 | 8.46E-01 | -0.04 | 0.41 | 1.99E-01 | 0.25 | 0.99 | 4594302 | 278277 | 4634387 | 416075 | 4689846 | 388897 | 4578928 | 438466 |
| RP_POS_1020_1355a0_483 | RP | POS | 0.48 | 1020.1355 | | | | 5.11E-05 | 0.48 | 1.07 | 1.84E-01 | -0.30 | 1.11 | 3.66E-02 | 0.33 | 0.95 | 3.41E-04 | 0.63 | 1.35 | 238091 | 19870 | 329438 | 213277 | 357854 | 218883 | 301023 | 205779 |
| RP_NEG_197_80806a7_099 | RP | NEG | 7.10 | 197.8081 | | | | 7.64E-01 | 0.06 | 0.45 | 1.84E-01 | -0.26 | 0.80 | 7.68E-01 | -0.07 | 0.29 | 3.97E-01 | 0.20 | 0.53 | 4495348 | 470017 | 4523833 | 448082 | 4583522 | 436094 | 4464144 | 456293 |
| HILIC_POS_300_2016a0_616 | HILIC | POS | 0.616 | 300.2016 | | | | 8.54E-01 | 0.05 | 1.07 | 1.84E-01 | -0.23 | 1.03 | 7.83E-01 | -0.07 | 0.88 | 5.60E-01 | 0.16 | 1.07 | 1949142 | 4890717 | 2119878 | 3213353 | 2548423 | 3850447 | 1691333 | 2379340 |
| RP_POS_315_19296a9_988 | RP | POS | 9.99 | 315.1930 | | | | 8.21E-04 | 0.49 | 1.21 | 1.84E-01 | 0.29 | 0.96 | 3.25E-03 | 0.64 | 1.15 | 1.72E-02 | 0.35 | 1.24 | 1361033 | 403873 | 1738030 | 830776 | 1627199 | 586230 | 1848862 | 1012783 |
| RP_POS_183_98911a6_427 | RP | POS | 6.43 | 183.9891 | | | | 3.42E-02 | 0.35 | 0.89 | 1.84E-01 | -0.29 | 0.92 | 2.29E-01 | 0.20 | 0.61 | 2.54E-02 | 0.49 | 1.02 | 692578 | 729348 | 1064659 | 1150193 | 1217997 | 1351961 | 911320 | 892805 |
| HILIC_POS_176_06577a4_248 | HILIC | POS | 4.248 | 176.0658 | | | | 4.55E-01 | -0.13 | 1.18 | 1.85E-01 | 0.28 | 1.10 | 9.55E-01 | 0.01 | 0.28 | 1.51E-01 | -0.27 | 0.82 | 943392 | 272524 | 897383 | 378093 | 847040 | 323120 | 947725 | 423376 |
| RP_POS_199_13052a11_58 | RP | POS | 11.58 | 199.1305 | | | | 5.75E-01 | -0.12 | 0.43 | 1.85E-01 | 0.26 | 0.87 | 9.70E-01 | 0.01 | 0.14 | 2.76E-01 | -0.25 | 0.64 | 5395134 | 1854680 | 5187356 | 1680905 | 4963723 | 1526050 | 5410989 | 1810430 |
| HILIC_NEG_117_9191a1_049 | HILIC | NEG | 1.049 | 117.9191 | | | | 4.27E-03 | 0.49 | 0.98 | 1.85E-01 | 0.28 | 0.92 | 2.24E-03 | 0.63 | 1.19 | 7.29E-02 | 0.35 | 0.70 | 683418 | 148553 | 783157 | 213429 | 754786 | 206413 | 811528 | 218596 |
| RP_NEG_276_99142a6_926 | RP | NEG | 6.93 | 276.9914 | | | | 5.10E-01 | 0.12 | 0.50 | 1.85E-01 | -0.28 | 0.79 | 9.00E-01 | -0.03 | 0.34 | 2.13E-01 | 0.26 | 0.55 | 5286050 | 1483837 | 5505183 | 2014798 | 5772986 | 2021606 | 5237381 | 1992087 |
| RP_POS_173_02892a15_735 | RP | POS | 15.74 | 173.0289 | | | | 4.76E-01 | -0.16 | 0.58 | 1.85E-01 | -0.25 | 0.90 | 2.24E-01 | -0.29 | 0.54 | 8.88E-01 | -0.04 | 0.36 | 65846146 | 44711629 | 59578402 | 36688716 | 64457298 | 41188359 | 54699506 | 31217538 |
| RP_POS_569_43103a14_513 | RP | POS | 14.51 | 569.4310 | | | | 4.84E-01 | -0.15 | 0.59 | 1.85E-01 | -0.26 | 0.96 | 2.23E-01 | -0.28 | 0.48 | 9.39E-01 | -0.02 | 0.59 | 4481785 | 2342799 | 4152086 | 2175304 | 4441131 | 2308157 | 3863041 | 2015533 |
| RP_POS_276_89249a15_46 | RP | POS | 15.46 | 276.8925 | | | | 5.20E-01 | 0.15 | 0.54 | 1.86E-01 | -0.25 | 0.78 | 9.09E-01 | 0.03 | 0.24 | 2.73E-01 | 0.27 | 0.56 | 2717069 | 906847 | 2830168 | 687187 | 2921440 | 646690 | 2738897 | 720333 |
| RP_POS_807_57269a13_859 | RP | POS | 13.86 | 807.5727 | | | | 2.76E-03 | 0.61 | 1.47 | 1.86E-01 | -0.26 | 0.82 | 2.69E-02 | 0.48 | 1.12 | 1.41E-03 | 0.74 | 1.40 | 2103397 | 1032908 | 2766486 | 1063722 | 2907678 | 1121193 | 2625295 | 994155 |
| RP_POS_307_16873a4_555 | RP | POS | 4.56 | 307.1687 | | | | 4.42E-03 | -0.60 | 0.95 | 1.86E-01 | -0.26 | 0.94 | 2.13E-03 | -0.72 | 1.18 | 3.48E-02 | -0.47 | 0.84 | 3631682 | 2209911 | 2303992 | 2145746 | 2588800 | 2009328 | 2019184 | 2258201 |
| RP_POS_605_23621a11_803 | RP | POS | 11.80 | 605.2362 | | | | 4.35E-02 | -0.40 | 1.02 | 1.86E-01 | 0.27 | 1.29 | 2.85E-01 | -0.27 | 0.59 | 5.46E-03 | -0.53 | 1.31 | 1677146 | 1030946 | 1237417 | 1099659 | 1091033 | 592540 | 1383801 | 1431204 |
| RP_NEG_130_94281a15_605 | RP | NEG | 15.61 | 130.9428 | | | | 1.73E-01 | -0.24 | 0.59 | 1.86E-01 | 0.28 | 0.91 | 6.14E-01 | -0.10 | 0.59 | 7.02E-02 | -0.38 | 0.85 | 2923561 | 67105 | 2903082 | 89484 | 2891215 | 92141 | 2914948 | 86027 |
| RP_POS_182_08177a1_268 | RP | POS | 1.27 | 182.0818 | | | | 2.57E-01 | 0.19 | 0.94 | 1.87E-01 | 0.28 | 1.03 | 1.27E-01 | 0.34 | 1.13 | 7.73E-01 | 0.05 | 0.60 | 19995370 | 4852856 | 21257029 | 6946531 | 20335867 | 5712090 | 22178192 | 7945671 |
| HILIC_NEG_100_93342a1_331 | HILIC | NEG | 1.331 | 100.9334 | | | | 1.34E-02 | -0.43 | 1.06 | 1.87E-01 | -0.28 | 1.05 | 6.66E-03 | -0.57 | 0.93 | 1.38E-01 | -0.29 | 0.52 | 15134894 | 2390874 | 13770681 | 3334026 | 14212486 | 3160712 | 13328877 | 3474169 |
| RP_POS_189_07846a6_096 | RP | POS | 6.10 | 189.0785 | | | | 3.08E-05 | -1.00 | 1.74 | 1.87E-01 | -0.22 | 0.78 | 1.06E-05 | -1.11 | 1.73 | 3.21E-04 | -0.90 | 1.47 | 2685762 | 1377318 | 1455329 | 1009567 | 1589026 | 1049557 | 1321633 | 959794 |
| RP_NEG_636_26288a10_331 | RP | NEG | 10.33 | 636.2629 | | | | 4.83E-02 | -0.49 | 1.13 | 1.87E-01 | -0.23 | 1.13 | 2.44E-02 | -0.61 | 1.15 | 1.39E-01 | -0.38 | 0.88 | 2606534 | 434645 | 2439049 | 293042 | 2477838 | 263867 | 2400260 | 317496 |
| RP_POS_366_06067a1_463 | RP | POS | 1.46 | 366.0607 | | | | 4.61E-01 | 0.09 | 0.37 | 1.87E-01 | 0.30 | 0.85 | 2.99E-01 | 0.24 | 0.43 | 1.26E-01 | -0.06 | 0.66 | 830256 | 797491 | 1186989 | 4613878 | 572423 | 620054 | 1801555 | 6469538 |
| RP_POS_606_35541a10_868 | RP | POS | 10.87 | 606.3554 | | | | 2.56E-01 | 0.15 | 0.62 | 1.88E-01 | -0.30 | 0.90 | 9.74E-01 | 0.00 | 0.50 | 1.36E-01 | 0.30 | 0.78 | 644561 | 114681 | 695258 | 395347 | 747552 | 459818 | 642965 | 314285 |
| HILIC_POS_388_30545a0_917 | HILIC | POS | 0.917 | 388.3055 | | | | 2.71E-06 | 0.81 | 1.43 | 1.88E-01 | 0.27 | 1.15 | 5.09E-06 | 0.94 | 1.68 | 4.33E-04 | 0.67 | 1.26 | 596979 | 281914 | 923497 | 407298 | 869722 | 388954 | 977272 | 421879 |
| RP_POS_553_45923a13_288 | RP | POS | 13.29 | 553.4592 | | | | 1.27E-01 | 0.32 | 0.67 | 1.88E-01 | -0.26 | 0.98 | 4.30E-01 | 0.18 | 0.65 | 4.55E-02 | 0.45 | 0.86 | 13139959 | 4637484 | 14598556 | 4583620 | 15203720 | 4180956 | 13993392 | 4921394 |
| HILIC_POS_161_01843a1_017 | HILIC | POS | 1.017 | 161.0184 | | | | 1.64E-01 | 0.21 | 0.46 | 1.88E-01 | -0.29 | 1.11 | 7.23E-01 | 0.07 | 0.19 | 6.15E-02 | 0.36 | 0.72 | 370873 | 113145 | 412152 | 215642 | 440611 | 218039 | 383692 | 211560 |
| RP_POS_573_14655a11_014 | RP | POS | 11.01 | 573.1466 | | | | 3.18E-01 | 0.21 | 0.60 | 1.89E-01 | -0.26 | 0.86 | 7.29E-01 | 0.08 | 0.38 | 1.41E-01 | 0.34 | 0.77 | 979404 | 300797 | 1040108 | 283137 | 1077454 | 274044 | 1002761 | 289867 |
| RP_NEG_269_21237a10_292 | RP | NEG | 10.29 | 269.2124 | | | | 5.50E-01 | -0.12 | 0.77 | 1.89E-01 | 0.27 | 1.02 | 9.41E-01 | 0.02 | 0.70 | 2.58E-01 | -0.25 | 0.61 | 1370361 | 201720 | 1345298 | 216339 | 1316783 | 216246 | 1373812 | 214798 |
| HILIC_NEG_195_81093a4_011 | HILIC | NEG | 4.011 | 195.8109 | | | | 8.31E-02 | 0.30 | 0.98 | 1.89E-01 | -0.28 | 0.78 | 4.28E-01 | 0.16 | 0.28 | 2.82E-02 | 0.44 | 1.15 | 515470 | 489793 | 707847 | 669781 | 796100 | 641877 | 619595 | 691713 |
| RP_NEG_162_83859a15_582 | RP | NEG | 15.58 | 162.8386 | | | | 1.38E-01 | -0.30 | 0.59 | 1.89E-01 | -0.26 | 0.89 | 5.14E-02 | -0.43 | 0.81 | 4.59E-01 | -0.17 | 0.29 | 3173231 | 110840 | 3139180 | 112603 | 3154014 | 118499 | 3124346 | 105482 |
| HILIC_POS_132_10204a2_639 | HILIC | POS | 2.639 | 132.1020 | | | | 1.31E-01 | -0.54 | 1.00 | 1.89E-01 | 0.03 | 0.87 | 1.44E-01 | -0.53 | 0.76 | 1.20E-01 | -0.56 | 0.85 | 874391 | 2627822 | 154184 | 170723 | 131669 | 124231 | 176698 | 205960 |
| RP_POS_345_66989a5_958 | RP | POS | 5.96 | 345.6699 | | | | 1.16E-01 | -0.29 | 1.02 | 1.89E-01 | -0.27 | 0.85 | 5.08E-02 | -0.43 | 0.97 | 4.57E-01 | -0.15 | 0.77 | 729401 | 149522 | 677837 | 186308 | 702365 | 174028 | 653308 | 196509 |
| RP_POS_301_03546a15_445 | RP | POS | 15.45 | 301.0355 | | | | 2.33E-01 | -0.28 | 0.67 | 1.89E-01 | 0.24 | 0.81 | 5.43E-01 | -0.16 | 0.46 | 1.02E-01 | -0.40 | 0.69 | 3835286 | 1059637 | 3587383 | 829657 | 3478134 | 739629 | 3696632 | 905152 |
| RP_POS_583_25513a7_159 | RP | POS | 7.16 | 583.2551 | | | | 9.38E-01 | -0.01 | 0.67 | 1.90E-01 | 0.28 | 1.18 | 5.80E-01 | 0.13 | 0.40 | 3.55E-01 | -0.15 | 0.86 | 894156 | 526468 | 884786 | 753201 | 785431 | 498539 | 984142 | 936756 |
| RP_POS_287_23691a9_749 | RP | POS | 9.75 | 287.2369 | | | | 5.70E-04 | 0.47 | 0.86 | 1.90E-01 | 0.29 | 0.84 | 1.54E-03 | 0.62 | 1.02 | 3.96E-02 | 0.33 | 0.76 | 277615 | 158760 | 451353 | 403230 | 398265 | 355483 | 504440 | 443157 |
| RP_NEG_228_16061a7_134 | RP | NEG | 7.13 | 228.1606 | | | | 7.91E-01 | 0.06 | 0.60 | 1.90E-01 | -0.24 | 0.82 | 8.16E-01 | -0.06 | 0.55 | 4.54E-01 | 0.18 | 0.49 | 1211570 | 835948 | 1254451 | 641087 | 1338809 | 575572 | 1170094 | 696109 |
| RP_NEG_308_9993a7_742 | RP | NEG | 7.74 | 308.9993 | | | | 2.11E-01 | 0.21 | 0.54 | 1.90E-01 | -0.28 | 0.80 | 7.13E-01 | 0.07 | 0.54 | 7.79E-02 | 0.36 | 0.71 | 6974315 | 774595 | 7195462 | 1095967 | 7339626 | 1074103 | 7051298 | 1109364 |
| HILIC_POS_218_1385a2_109 | HILIC | POS | 2.109 | 218.1385 | | | | 7.37E-01 | -0.06 | 0.45 | 1.90E-01 | -0.28 | 0.84 | 3.48E-01 | -0.20 | 0.40 | 6.97E-01 | 0.08 | 0.18 | 42944640 | 17843589 | 41623717 | 23344796 | 44692443 | 22362543 | 38554991 | 24120099 |
| RP_NEG_198_93556a15_778 | RP | NEG | 15.78 | 198.9356 | | | | 9.35E-01 | -0.02 | 0.65 | 1.90E-01 | -0.27 | 0.78 | 5.02E-01 | -0.15 | 0.58 | 6.06E-01 | 0.12 | 0.43 | 12023156 | 5599422 | 11929652 | 5839713 | 12696646 | 5961138 | 11162657 | 5671395 |
| RP_POS_929_48486a0_484 | RP | POS | 0.48 | 929.4849 | | | | 7.67E-07 | 0.59 | 1.19 | 1.91E-01 | -0.29 | 1.18 | 3.87E-03 | 0.44 | 1.11 | 5.94E-05 | 0.73 | 1.42 | 120408 | 13129 | 286597 | 314144 | 327817 | 333477 | 245377 | 291054 |
| RP_NEG_595_49506a11_477 | RP | NEG | 11.48 | 595.4951 | | | | 2.15E-01 | -0.22 | 0.36 | 1.91E-01 | -0.28 | 0.82 | 5.78E-02 | -0.35 | 0.70 | 7.29E-01 | -0.08 | 0.25 | 1751151 | 1150870 | 1427990 | 1593155 | 1637057 | 1815377 | 1218923 | 1320337 |

**Supplementary table 1: Metabolomics results** (in asceding order according to p-value from progressive ALD vs non-progressive ALD comparison, with molecular features with ID_level of 1-2 first)

| | | | | | | | | all_ALD_vs_control | | | Prog_ALD_vs_non-prog_ALD | | | Prog_ALD_vs_control_ALD | | | Non-prog_ALD_vs_control | | | Healthy_control | | all_ALD | | Non-prog_ALD | | Progressive_ALD | |
|---|---|---|---|---|---|---|---|---|---|---|---|---|---|---|---|---|---|---|---|---|---|---|---|---|---|---|---|
| | | | | | | | | t-test | Cohen's | PLS-DA | t-test | Cohen's | PLS-DA | t-test | Cohen's | PLS-DA | t-test | Cohen's | PLS-DA | | | | | | | | |
| Feature_ID | RP_HILIC | POS_NEG | Average.Rt | Average.Mz | Metabolite.name | curated_MSMS | ID_level | p | d | VIP | p | d | VIP | p | d | VIP | p | d | VIP | Mean | SD | Mean | SD | Mean | SD | Mean | SD |
| RP_POS_419_31485a13_932 | RP | POS | 13.93 | 419.3149 | | | | 2.67E-01 | 0.24 | 0.48 | 1.91E-01 | 0.25 | 0.84 | 1.25E-01 | 0.37 | 0.92 | 6.19E-01 | 0.12 | 0.38 | 4803716 | 2353785 | 5325363 | 2053119 | 5056147 | 2026823 | 5594579 | 2064144 |
| RP_POS_327_22635a11_254 | RP | POS | 11.25 | 327.2264 | | | | 3.01E-05 | 0.74 | 1.71 | 1.91E-01 | 0.26 | 1.06 | 6.89E-05 | 0.87 | 1.62 | 1.10E-03 | 0.61 | 1.62 | 7223335 | 2794452 | 10000543 | 3787071 | 9503860 | 3207620 | 10497225 | 4264297 |
| RP_POS_368_27933a8_328 | RP | POS | 8.33 | 368.2793 | | | | 7.15E-07 | 0.67 | 1.08 | 1.91E-01 | 0.28 | 0.77 | 1.03E-04 | 0.81 | 1.30 | 1.66E-04 | 0.53 | 1.37 | 430421 | 184638 | 745507 | 508284 | 678806 | 378252 | 812208 | 608131 |
| RP_NEG_239_75981a15_587 | RP | NEG | 15.59 | 239.7598 | | | | 4.94E-02 | -0.38 | 0.73 | 1.92E-01 | -0.27 | 0.84 | 1.62E-02 | -0.51 | 0.92 | 2.64E-01 | -0.25 | 0.45 | 8507052 | 286248 | 8387034 | 318536 | 8428772 | 338005 | 8345295 | 295295 |
| RP_POS_190_98807a0_843 | RP | POS | 0.84 | 190.9881 | | | | 6.33E-01 | -0.09 | 0.42 | 1.92E-01 | -0.27 | 0.77 | 2.85E-01 | -0.23 | 0.43 | 8.35E-01 | 0.05 | 0.44 | 7058826 | 2985078 | 6755180 | 3498005 | 7213083 | 3634198 | 6297277 | 3329637 |
| RP_POS_166_98491a11_09 | RP | POS | 11.09 | 166.9849 | | | | 2.41E-02 | 0.44 | 0.95 | 1.93E-01 | 0.26 | 0.79 | 9.99E-03 | 0.57 | 1.03 | 1.51E-01 | 0.31 | 0.60 | 10184800 | 4764597 | 12480918 | 5222671 | 11798132 | 5130133 | 13163704 | 5276320 |
| RP_POS_414_3002a8_564 | RP | POS | 8.56 | 414.3002 | | | | 1.24E-01 | 0.18 | 0.87 | 1.93E-01 | 0.30 | 0.94 | 1.56E-01 | 0.33 | 0.90 | 5.40E-02 | 0.03 | 0.85 | 295758 | 624499 | 3546133 | 20916760 | 797616 | 1633361 | 6294650 | 29425595 |
| RP_POS_1006_75824a0_493 | RP | POS | 0.49 | 1006.7582 | | | | 2.17E-09 | -0.76 | 1.38 | 1.93E-01 | 0.28 | 1.26 | 2.57E-04 | -0.62 | 1.34 | 5.42E-07 | -0.90 | 1.59 | 2314230 | 223222 | 1671254 | 914941 | 1551858 | 914728 | 1790651 | 908569 |
| RP_POS_607_25189a11_805 | RP | POS | 11.81 | 607.2519 | | | | 1.66E-02 | -0.49 | 1.24 | 1.93E-01 | 0.26 | 1.33 | 1.42E-01 | -0.36 | 0.83 | 2.74E-03 | -0.62 | 1.31 | 4483035 | 1781114 | 3584450 | 1810563 | 3347741 | 1236484 | 3821159 | 2231577 |
| RP_POS_279_22971a11_161 | RP | POS | 11.16 | 279.2297 | | | | 2.70E-01 | -0.25 | 0.46 | 1.93E-01 | -0.24 | 0.92 | 1.09E-01 | -0.38 | 0.82 | 6.14E-01 | -0.13 | 0.45 | 1708748 | 803635 | 1534754 | 640691 | 1618448 | 761767 | 1451060 | 484524 |
| RP_POS_401_19681a10_143 | RP | POS | 10.14 | 401.1968 | | 401.1967(100) | 4 | 4.30E-01 | -0.22 | 0.73 | 1.94E-01 | -0.20 | 0.90 | 2.68E-01 | -0.32 | 0.59 | 6.71E-01 | -0.13 | 0.70 | 5149212 | 9458242 | 3763698 | 4665015 | 4372157 | 5062013 | 3155239 | 4193964 |
| RP_POS_371_25653a10_854 | RP | POS | 10.85 | 371.2565 | | | | 5.39E-06 | -0.75 | 1.25 | 1.94E-01 | -0.27 | 1.02 | 2.25E-05 | -0.89 | 1.41 | 5.85E-04 | -0.62 | 1.12 | 1772475 | 445181 | 1261422 | 695550 | 1352124 | 614934 | 1170720 | 763229 |
| RP_POS_647_45807a13_32 | RP | POS | 13.32 | 647.4581 | | | | 2.37E-01 | -0.26 | 0.52 | 1.94E-01 | -0.25 | 0.90 | 9.83E-02 | -0.38 | 0.85 | 5.91E-01 | -0.13 | 0.34 | 7571027 | 8297615 | 5606422 | 7384736 | 6568452 | 8021352 | 4644391 | 6629535 |
| RP_POS_299_1387a11_805 | RP | POS | 11.81 | 299.1387 | | | | 2.90E-02 | -0.44 | 1.11 | 1.94E-01 | 0.26 | 1.32 | 2.15E-01 | -0.31 | 0.67 | 3.93E-03 | -0.57 | 1.34 | 4409086 | 2356395 | 3323738 | 2460892 | 3002299 | 1429581 | 3645177 | 3159273 |
| RP_NEG_723_08887a9_357 | RP | NEG | 9.36 | 723.0889 | | | | 3.13E-03 | -0.60 | 1.06 | 1.95E-01 | -0.26 | 0.91 | 1.17E-03 | -0.72 | 1.24 | 3.57E-02 | -0.47 | 0.84 | 3197036 | 192044 | 3075017 | 201014 | 3101181 | 206149 | 3048852 | 194276 |
| RP_POS_524_27441a10_216 | RP | POS | 10.22 | 524.2744 | | | | 3.29E-06 | 0.82 | 1.53 | 1.95E-01 | -0.26 | 0.94 | 1.54E-03 | 0.69 | 1.20 | 3.39E-07 | 0.95 | 1.84 | 1103681 | 341027 | 1489493 | 470688 | 1550779 | 363633 | 1428206 | 554727 |
| RP_POS_290_91843a15_37 | RP | POS | 15.37 | 290.9184 | | | | 1.01E-02 | -0.52 | 0.87 | 1.95E-01 | -0.26 | 0.97 | 3.21E-03 | -0.64 | 1.26 | 8.74E-02 | -0.39 | 0.61 | 2187754 | 464441 | 1932877 | 489679 | 1996534 | 521230 | 1869220 | 452232 |
| RP_NEG_394_97629a10_261 | RP | NEG | 10.26 | 394.9763 | | | | 9.08E-01 | 0.02 | 0.38 | 1.96E-01 | 0.26 | 1.09 | 5.21E-01 | 0.16 | 0.62 | 5.92E-01 | -0.11 | 0.46 | 1124642 | 803367 | 1143753 | 848876 | 1033425 | 651971 | 1254080 | 1002985 |
| HILIC_NEG_234_82019a7_019 | HILIC | NEG | 7.019 | 234.8202 | | | | 7.84E-01 | -0.06 | 0.17 | 1.96E-01 | -0.25 | 1.55 | 4.24E-01 | -0.19 | 0.36 | 7.79E-01 | 0.07 | 0.20 | 1143872 | 467273 | 1118342 | 422079 | 1173123 | 444229 | 1063562 | 395567 |
| RP_POS_700_52673a13_495 | RP | POS | 13.50 | 700.5267 | | | | 2.74E-02 | -0.54 | 1.03 | 1.96E-01 | -0.23 | 0.76 | 1.29E-02 | -0.66 | 1.09 | 9.34E-02 | -0.43 | 0.75 | 1296561 | 796251 | 950125 | 559396 | 1022715 | 538810 | 877536 | 575469 |
| RP_POS_587_54944a15_22 | RP | POS | 15.22 | 587.5494 | | | | 1.15E-01 | 0.37 | 0.72 | 1.96E-01 | -0.24 | 0.83 | 2.98E-01 | 0.26 | 0.49 | 5.88E-02 | 0.49 | 0.88 | 15278841 | 2423546 | 16027798 | 1817804 | 16263672 | 1963255 | 15791923 | 1645706 |
| RP_POS_872_5401a12_82 | RP | POS | 12.82 | 872.5401 | | | | 2.18E-05 | -0.97 | 1.61 | 1.96E-01 | -0.22 | 0.92 | 1.10E-05 | -1.09 | 1.71 | 2.43E-04 | -0.86 | 1.42 | 4580873 | 1419892 | 3270339 | 1160089 | 3420778 | 1099395 | 3119900 | 1210051 |
| HILIC_POS_166_08385a3_509 | HILIC | POS | 3.509 | 166.0839 | | | | 2.42E-01 | 0.20 | 0.54 | 1.96E-01 | 0.28 | 0.90 | 1.08E-01 | 0.34 | 0.67 | 7.65E-01 | 0.06 | 0.36 | 3138139 | 3846289 | 4189360 | 5761799 | 3442125 | 5293075 | 4936595 | 6157761 |
| RP_POS_185_11484a13_809 | RP | POS | 13.81 | 185.1148 | | | | 1.74E-01 | -0.31 | 0.72 | 1.96E-01 | 0.25 | 0.88 | 4.64E-01 | -0.18 | 0.51 | 7.14E-02 | -0.43 | 1.11 | 8111944 | 4197718 | 6976507 | 3526365 | 6519320 | 3150764 | 7433694 | 3843228 |
| RP_POS_195_12225a10_652 | RP | POS | 10.65 | 195.1223 | | | | 5.17E-01 | 0.13 | 0.73 | 1.97E-01 | -0.26 | 0.98 | 9.85E-01 | 0.00 | 0.35 | 2.44E-01 | 0.26 | 0.70 | 1629694 | 468267 | 1692939 | 504453 | 1758275 | 505720 | 1627603 | 499674 |
| RP_POS_417_73984a12_693 | RP | POS | 12.69 | 417.7398 | | | | 1.62E-03 | 0.59 | 1.21 | 1.97E-01 | -0.26 | 0.82 | 3.22E-02 | 0.46 | 0.94 | 5.61E-04 | 0.72 | 1.27 | 5865386 | 2195446 | 7413794 | 2647386 | 7756580 | 2487690 | 7071007 | 2780637 |
| RP_POS_624_3764a11_089 | RP | POS | 11.09 | 624.3764 | | | | 1.27E-07 | 0.63 | 1.31 | 1.97E-01 | -0.29 | 1.27 | 3.36E-04 | 0.49 | 1.37 | 8.68E-05 | 0.77 | 1.41 | 46841 | 35060 | 775709 | 1279003 | 941322 | 1477673 | 610097 | 1032273 |
| RP_POS_548_80963a10_618 | RP | POS | 10.62 | 548.8096 | | | | 6.29E-03 | 0.62 | 1.37 | 1.97E-01 | -0.24 | 1.03 | 4.56E-02 | 0.50 | 1.04 | 1.73E-03 | 0.74 | 1.47 | 3023515 | 875438 | 3512934 | 722115 | 3606429 | 589740 | 3419439 | 829402 |
| RP_NEG_335_04788a0_62 | RP | NEG | 0.62 | 335.0479 | | | | 1.64E-05 | 0.51 | 1.09 | 1.97E-01 | -0.29 | 1.16 | 6.52E-03 | 0.36 | 1.08 | 8.23E-04 | 0.65 | 1.27 | 4309636 | 755772 | 16029926 | 25836481 | 19375305 | 29870716 | 12684548 | 20822364 |
| RP_NEG_412_62927a15_583 | RP | NEG | 15.58 | 412.6293 | | | | 2.69E-01 | -0.24 | 0.60 | 1.98E-01 | -0.25 | 0.86 | 1.16E-01 | -0.37 | 0.66 | 6.38E-01 | -0.11 | 0.42 | 1669239 | 72635 | 1653109 | 65450 | 1661573 | 69876 | 1644645 | 60216 |
| RP_NEG_269_24805a11_381 | RP | NEG | 11.38 | 269.2481 | | | | 1.32E-03 | -0.67 | 1.11 | 1.98E-01 | -0.25 | 1.05 | 2.79E-04 | -0.79 | 1.36 | 2.14E-02 | -0.55 | 0.85 | 943868 | 218470 | 793793 | 214296 | 821523 | 246529 | 766064 | 174460 |
| RP_POS_677_47821a10_868 | RP | POS | 10.87 | 677.4782 | | | | 2.29E-01 | -0.22 | 0.49 | 1.98E-01 | 0.27 | 0.93 | 6.86E-01 | -0.08 | 0.32 | 9.35E-02 | -0.35 | 0.69 | 8111819 | 549114 | 7967094 | 689373 | 7877973 | 690569 | 8056216 | 683427 |
| RP_POS_376_31848a10_829 | RP | POS | 10.83 | 376.3185 | | | | 6.49E-01 | 0.12 | 0.87 | 1.98E-01 | 0.22 | 0.94 | 4.08E-01 | 0.23 | 0.71 | 9.84E-01 | 0.01 | 0.61 | 646739 | 584322 | 697155 | 371956 | 649074 | 334628 | 745235 | 403524 |
| HILIC_POS_426_35788a0_608 | HILIC | POS | 0.608 | 426.3579 | | | | 2.94E-07 | 0.95 | 1.76 | 1.98E-01 | 0.25 | 0.76 | 1.48E-07 | 1.08 | 1.99 | 7.19E-05 | 0.83 | 1.69 | 1159881 | 519060 | 1801439 | 642286 | 1718438 | 682203 | 1884440 | 594997 |
| HILIC_NEG_134_89473a4_009 | HILIC | NEG | 4.009 | 134.8947 | | | | 1.20E-01 | -0.32 | 0.59 | 1.98E-01 | -0.26 | 0.88 | 5.34E-02 | -0.45 | 0.72 | 3.88E-01 | -0.19 | 0.80 | 18077081 | 8213843 | 15447430 | 8086014 | 16491356 | 7797773 | 14403504 | 8310975 |
| HILIC_POS_478_32684a1_309 | HILIC | POS | 1.309 | 478.3268 | | | | 5.31E-03 | 0.56 | 0.88 | 1.98E-01 | -0.25 | 0.71 | 4.76E-02 | 0.44 | 0.74 | 2.28E-03 | 0.69 | 1.18 | 448805 | 193842 | 564238 | 201144 | 590198 | 201783 | 538278 | 199128 |
| RP_NEG_237_93413a1_459 | RP | NEG | 1.46 | 237.9341 | | | | 5.20E-12 | 0.91 | 1.48 | 1.98E-01 | 0.27 | 0.78 | 1.18E-07 | 1.04 | 1.69 | 6.15E-07 | 0.77 | 1.50 | 303919 | 279482 | 1067423 | 874770 | 954483 | 758425 | 1180363 | 972019 |
| RP_NEG_187_86122a15_536 | RP | NEG | 15.54 | 187.8612 | | | | 1.09E-01 | -0.36 | 0.85 | 1.98E-01 | -0.24 | 0.99 | 4.77E-02 | -0.48 | 0.81 | 3.26E-01 | -0.24 | 0.69 | 579426 | 36455 | 567751 | 30650 | 571706 | 30957 | 563796 | 30130 |
| HILIC_POS_570_35577a1_192 | HILIC | POS | 1.192 | 570.3558 | | | | 2.70E-04 | 0.67 | 1.17 | 1.98E-01 | -0.26 | 0.79 | 1.16E-02 | 0.54 | 0.97 | 7.76E-05 | 0.80 | 1.54 | 3012022 | 1612105 | 4353905 | 1998646 | 4611860 | 1792536 | 4095949 | 2172950 |
| RP_POS_814_01208a10_932 | RP | POS | 10.93 | 814.0121 | | | | 4.42E-02 | -0.48 | 1.18 | 1.99E-01 | -0.23 | 1.61 | 1.97E-02 | -0.60 | 1.14 | 1.49E-01 | -0.36 | 0.96 | 1406903 | 655902 | 1145720 | 491771 | 1209148 | 491861 | 1082292 | 488338 |
| HILIC_NEG_221_01254a1_297 | HILIC | NEG | 1.297 | 221.0125 | | | | 1.09E-12 | 0.99 | 1.64 | 1.99E-01 | 0.26 | 0.89 | 1.80E-08 | 1.12 | 1.73 | 7.15E-08 | 0.86 | 1.73 | 332142 | 190297 | 789804 | 468250 | 729437 | 405133 | 850171 | 520980 |
| RP_NEG_215_86778a3_128 | RP | NEG | 3.13 | 215.8678 | | | | 1.61E-01 | -0.25 | 0.89 | 1.99E-01 | -0.27 | 0.78 | 4.67E-02 | -0.39 | 0.85 | 6.01E-01 | -0.11 | 0.53 | 1504842 | 784568 | 1260339 | 1027199 | 1392752 | 1150261 | 1127925 | 879162 |
| HILIC_NEG_124_04037a0_692 | HILIC | NEG | 0.692 | 124.0404 | | | | 1.18E-01 | -0.39 | 0.74 | 1.99E-01 | -0.22 | 1.08 | 5.46E-02 | -0.50 | 0.82 | 2.96E-01 | -0.28 | 0.48 | 140060 | 110292 | 106779 | 74037 | 116316 | 79591 | 97242 | 67487 |
| RP_POS_1070_66125a10_617 | RP | POS | 10.62 | 1070.6613 | | | | 1.47E-03 | 0.66 | 1.39 | 2.00E-01 | -0.25 | 0.84 | 2.24E-02 | 0.53 | 1.09 | 3.66E-04 | 0.78 | 1.50 | 866367 | 524017 | 1223689 | 523182 | 1291020 | 448487 | 1156358 | 585348 |
| RP_NEG_235_92589a0_104 | RP | NEG | 0.10 | 235.9259 | | | | 7.84E-01 | 0.06 | 0.52 | 2.00E-01 | 0.25 | 0.89 | 4.35E-01 | 0.18 | 0.54 | 7.65E-01 | -0.07 | 0.52 | 5256595 | 2234785 | 5379715 | 2092169 | 5110859 | 2006330 | 5648571 | 2161202 |
| RP_NEG_314_84808a15_494 | RP | NEG | 15.49 | 314.8481 | | | | 5.41E-01 | 0.17 | 0.53 | 2.00E-01 | -0.21 | 0.78 | 8.26E-01 | 0.06 | 0.28 | 3.34E-01 | 0.27 | 0.68 | 967483 | 108689 | 979906 | 59870 | 987602 | 51773 | 972210 | 66638 |
| RP_POS_590_85297a0_494 | RP | POS | 0.49 | 590.8530 | | | | 2.85E-09 | -0.76 | 1.38 | 2.01E-01 | 0.28 | 1.26 | 1.78E-04 | -0.62 | 1.35 | 1.08E-06 | -0.89 | 1.56 | 1548707 | 157838 | 1119599 | 610669 | 1041208 | 628738 | 1197991 | 587871 |
| RP_NEG_458_57303a15_583 | RP | NEG | 15.58 | 458.5730 | | | | 6.57E-02 | -0.33 | 0.55 | 2.01E-01 | -0.27 | 0.80 | 2.58E-02 | -0.47 | 0.81 | 3.40E-01 | -0.20 | 0.34 | 678781 | 28609 | 667062 | 36932 | 671802 | 36975 | 662321 | 36643 |
| RP_POS_365_26575a10_744 | RP | POS | 10.74 | 365.2658 | | | | 1.79E-02 | -0.52 | 1.37 | 2.01E-01 | 0.24 | 0.79 | 1.08E-01 | -0.39 | 0.94 | 5.16E-03 | -0.64 | 1.20 | 1055046 | 295988 | 911285 | 264298 | 877340 | 220252 | 945231 | 300450 |
| RP_POS_409_24481a11_17 | RP | POS | 11.17 | 409.2448 | | | | 1.70E-02 | 0.43 | 1.00 | 2.01E-01 | -0.27 | 0.91 | 1.43E-01 | 0.29 | 0.71 | 8.64E-03 | 0.56 | 1.11 | 2054514 | 155676 | 2138587 | 205781 | 2165001 | 215092 | 2112173 | 194595 |
| RP_POS_450_35699a11_394 | RP | POS | 11.39 | 450.3570 | | | | 3.32E-02 | -0.42 | 0.75 | 2.01E-01 | 0.26 | 1.03 | 2.11E-01 | -0.29 | 0.59 | 8.35E-03 | -0.55 | 0.97 | 7235630 | 705232 | 6916694 | 760527 | 6819029 | 624068 | 7014358 | 871596 |
| RP_NEG_266_01834a6_762 | RP | NEG | 6.76 | 266.0183 | | | | 1.77E-02 | -0.75 | 1.48 | 2.01E-01 | -0.14 | 1.21 | 1.03E-02 | -0.82 | 1.25 | 3.41E-02 | -0.68 | 1.07 | 7365956 | 12068540 | 1946572 | 3998438 | 2459959 | 4548044 | 1433186 | 3328466 |
| RP_POS_518_32135a11_255 | RP | POS | 11.26 | 518.3214 | | | | 4.01E-04 | 0.56 | 1.35 | 2.01E-01 | -0.27 | 0.97 | 2.42E-02 | 0.42 | 1.07 | 3.68E-04 | 0.69 | 1.58 | 582718 | 272065 | 830087 | 471363 | 890523 | 475669 | 769651 | 463881 |
| RP_NEG_297_15375a15_468 | RP | NEG | 15.47 | 297.1538 | | | | 7.98E-01 | 0.05 | 1.06 | 2.01E-01 | -0.26 | 0.93 | 7.05E-01 | -0.08 | 0.32 | 4.10E-01 | 0.18 | 1.17 | 1651117 | 534763 | 1679809 | 597762 | 1756438 | 599935 | 1603179 | 591629 |
| RP_NEG_437_29184a9_858 | RP | NEG | 9.86 | 437.2918 | | | | 4.64E-01 | 0.13 | 0.32 | 2.02E-01 | 0.27 | 0.77 | 2.30E-01 | 0.27 | 0.47 | 9.95E-01 | 0.00 | 0.64 | 2460987 | 3727894 | 3057626 | 4693723 | 2455985 | 4058881 | 3659267 | 5224791 |
| HILIC_POS_64_01665a4_007 | HILIC | POS | 4.007 | 64.0167 | | | | 8.51E-02 | 0.30 | 0.74 | 2.02E-01 | -0.27 | 1.00 | 4.29E-01 | 0.16 | 0.53 | 2.90E-02 | 0.44 | 0.77 | 3112343 | 792386 | 3422617 | 1093384 | 3562711 | 1033621 | 3282524 | 1143219 |
| RP_POS_1067_77185a14_271 | RP | POS | 14.27 | 1067.7719 | | | | 2.90E-01 | 0.17 | 0.51 | 2.02E-01 | 0.28 | 0.81 | 1.75E-01 | 0.31 | 0.64 | 8.37E-01 | 0.03 | 0.35 | 4222147 | 1295339 | 4552987 | 2100062 | 4283392 | 1330302 | 4822582 | 2644332 |
| RP_POS_677_47968a10_921 | RP | POS | 10.92 | 677.4797 | | | | 2.58E-01 | -0.27 | 0.50 | 2.02E-01 | -0.23 | 1.09 | 1.34E-01 | -0.39 | 0.67 | 5.36E-01 | -0.16 | 0.46 | 7128840 | 1217687 | 6862186 | 876571 | 6974420 | 864191 | 6749953 | 883121 |
| RP_POS_393_19058a8_034 | RP | POS | 8.03 | 393.1906 | | | | 2.11E-07 | 0.62 | 1.08 | 2.02E-01 | 0.28 | 1.16 | 3.65E-04 | 0.76 | 1.24 | 9.79E-06 | 0.48 | 1.33 | 36498 | 64104 | 1106609 | 1915709 | 860718 | 1180374 | 1352501 | 2428602 |
| RP_POS_774_60046a14_012 | RP | POS | 14.01 | 774.6005 | | | | 5.12E-01 | 0.12 | 0.59 | 2.02E-01 | 0.27 | 0.77 | 2.53E-01 | 0.26 | 0.59 | 9.61E-01 | -0.01 | 0.43 | 1683912 | 934412 | 1815255 | 1108646 | 1673286 | 995638 | 1957223 | 1204511 |
| HILIC_POS_386_28961a0_925 | HILIC | POS | 0.925 | 386.2896 | | | | 1.92E-03 | 0.50 | 1.04 | 2.03E-01 | 0.27 | 1.29 | 2.14E-03 | 0.64 | 1.12 | 3.97E-02 | 0.37 | 0.70 | 1205244 | 533675 | 1613547 | 857582 | 1503840 | 758094 | 1723253 | 941612 |
| RP_POS_1065_68384a10_618 | RP | POS | 10.62 | 1065.6838 | | | | 1.96E-03 | 0.67 | 1.46 | 2.03E-01 | -0.24 | 1.00 | 2.34E-02 | 0.55 | 1.14 | 4.90E-04 | 0.79 | 1.58 | 6267823 | 2682101 | 8016845 | 2468955 | 8332776 | 2059172 | 7700913 | 2805708 |
| RP_POS_315_13321a11_803 | RP | POS | 11.80 | 315.1332 | | | | 5.49E-02 | -0.37 | 0.98 | 2.03E-01 | 0.26 | 1.30 | 3.28E-01 | -0.24 | 0.54 | 7.05E-03 | -0.50 | 1.31 | 992820 | 705125 | 704225 | 783930 | 603679 | 415356 | 804770 | 1023954 |
| RP_POS_772_5274a13_52 | RP | POS | 13.52 | 772.5274 | | | | 9.64E-01 | 0.01 | 0.61 | 2.03E-01 | -0.26 | 0.76 | 5.68E-01 | -0.12 | 0.34 | 5.33E-01 | 0.14 | 0.65 | 2880242 | 1054490 | 2890130 | 1170949 | 3039760 | 1224661 | 2740500 | 1106680 |
| RP_POS_136_07588a1_268 | RP | POS | 1.27 | 136.0759 | | | | 3.87E-01 | 0.15 | 0.99 | 2.03E-01 | 0.27 | 1.05 | 1.99E-01 | 0.28 | 1.15 | 9.49E-01 | 0.01 | 0.63 | 2521372 | 566912 | 2633386 | 808449 | 2530096 | 648304 | 2736676 | 937254 |
| RP_POS_229_95404a15_592 | RP | POS | 15.59 | 229.9540 | | | | 3.34E-01 | -0.22 | 0.70 | 2.03E-01 | 0.24 | 0.94 | 6.91E-01 | -0.10 | 0.46 | 1.66E-01 | -0.34 | 0.58 | 3685775 | 113564 | 3664136 | 94065 | 3652130 | 92849 | 3676142 | 94667 |
| HILIC_NEG_452_2778a1_867 | HILIC | NEG | 1.867 | 452.2778 | | | | 8.44E-02 | 0.28 | 0.73 | 2.03E-01 | -0.27 | 0.84 | 4.86E-01 | 0.15 | 0.26 | 2.18E-02 | 0.42 | 0.80 | 259847 | 82123 | 293810 | 129337 | 310322 | 112748 | 277298 | 143279 |
| RP_POS_667_27289a11_802 | RP | POS | 11.80 | 667.2729 | | | | 6.15E-02 | -0.34 | 0.92 | 2.03E-01 | 0.27 | 1.28 | 3.99E-01 | -0.21 | 0.52 | 5.01E-03 | -0.47 | 1.32 | 968194 | 729198 | 666822 | 920681 | 548837 | 420634 | 784806 | 1227707 |
| RP_POS_693_47516a10_468 | RP | POS | 10.47 | 693.4752 | | | | 9.23E-01 | 0.01 | 0.35 | 2.04E-01 | -0.28 | 1.13 | 5.12E-01 | -0.13 | 0.48 | 3.93E-01 | 0.16 | 0.35 | 5365747 | 430610 | 5376548 | 816676 | 5480756 | 780098 | 5272339 | 846649 |
| RP_POS_784_57642a11_168 | RP | POS | 11.17 | 784.5764 | | | | 2.45E-04 | 0.44 | 1.15 | 2.04E-01 | -0.29 | 1.25 | 2.22E-02 | 0.30 | 1.14 | 3.35E-03 | 0.58 | 1.30 | 238755 | 77048 | 440128 | 513627 | 505721 | 605882 | 374534 | 396404 |
| HILIC_POS_143_99702a7_961 | HILIC | POS | 7.961 | 143.9970 | | | | 5.51E-05 | -0.63 | 1.27 | 2.04E-01 | 0.27 | 0.99 | 9.23E-03 | -0.50 | 1.24 | 4.94E-05 | -0.77 | 1.39 | 1794943 | 466107 | 1304187 | 818146 | 1199825 | 788523 | 1408550 | 841660 |
| RP_NEG_396_97314a7_568 | RP | NEG | 7.57 | 396.9731 | | | | 8.91E-01 | 0.03 | 0.21 | 2.04E-01 | 0.26 | 1.03 | 4.79E-01 | 0.16 | 0.79 | 6.38E-01 | -0.10 | 0.34 | 2671788 | 668376 | 2690825 | 726972 | 2598119 | 719698 | 2783531 | 729559 |
| RP_POS_568_29468a7_093 | RP | POS | 7.09 | 568.2947 | | | | 3.65E-01 | -0.17 | 0.56 | 2.04E-01 | 0.27 | 0.86 | 8.74E-01 | -0.03 | 0.41 | 1.55E-01 | -0.30 | 0.73 | 51251006 | 2342517 | 50790666 | 2881645 | 50423266 | 2836754 | 51158067 | 2907675 |
| HILIC_NEG_89_02402a4_985 | HILIC | NEG | 4.985 | 89.0240 | | | | 9.59E-01 | 0.01 | 0.32 | 2.04E-01 | 0.27 | 0.76 | 5.21E-01 | 0.14 | 0.56 | 5.11E-01 | -0.13 | 0.82 | 7298098 | 4248728 | 7345981 | 5503611 | 6644136 | 4550474 | 8047827 | 6283752 |
| RP_NEG_274_97604a7_752 | RP | NEG | 7.75 | 274.9760 | | | | 5.43E-02 | -0.30 | 0.53 | 2.04E-01 | 0.28 | 0.88 | 2.78E-01 | -0.17 | 0.47 | 4.89E-02 | -0.44 | 0.69 | 8712257 | 394854 | 8522960 | 674701 | 8436804 | 838186 | 8609115 | 449476 |
| RP_POS_768_55292a13_859 | RP | POS | 13.86 | 768.5529 | | | | 3.19E-01 | 0.21 | 0.53 | 2.05E-01 | 0.25 | 0.83 | 1.71E-01 | 0.34 | 0.65 | 6.94E-01 | 0.09 | 0.45 | 3102310 | 1880280 | 3480409 | 1751519 | 3257297 | 1472156 | 3703521 | 1982275 |
| HILIC_POS_168_10193a1_104 | HILIC | POS | 1.104 | 168.1019 | | | | 2.15E-02 | 0.27 | 0.45 | 2.05E-01 | 0.29 | 0.87 | 6.37E-02 | 0.41 | 0.56 | 7.35E-02 | 0.12 | 0.56 | 729431 | 1200314 | 6261183 | 23585316 | 3245327 | 9620090 | 9277039 | 31824182 |
| RP_NEG_322_80655a15_382 | RP | NEG | 15.38 | 322.8066 | | | | 6.53E-01 | 0.10 | 0.48 | 2.05E-01 | -0.24 | 0.81 | 9.46E-01 | -0.02 | 0.24 | 3.84E-01 | 0.22 | 0.46 | 781568 | 61810 | 787003 | 49371 | 793288 | 54477 | 780717 | 43317 |
| RP_POS_1019_60614a10_335 | RP | POS | 10.34 | 1019.6061 | | | | 5.87E-01 | 0.11 | 0.96 | 2.05E-01 | -0.25 | 1.02 | 9.61E-01 | -0.01 | 0.74 | 2.73E-01 | 0.24 | 0.91 | 1138207 | 591857 | 1203270 | 565173 | 1275224 | 468422 | 1131316 | 644495 |
| RP_POS_625_38861a13_501 | RP | POS | 13.50 | 625.3886 | | | | 6.06E-01 | 0.07 | 0.56 | 2.05E-01 | 0.29 | 0.78 | 3.56E-01 | 0.21 | 0.50 | 4.42E-01 | -0.07 | 0.30 | 1828044 | 301973 | 1877161 | 787205 | 1776535 | 282008 | 1977787 | 1073237 |
| RP_NEG_348_00574a2_476 | RP | NEG | 2.48 | 348.0057 | | | | 3.28E-03 | -0.50 | 1.15 | 2.05E-01 | -0.27 | 1.17 | 3.86E-03 | -0.63 | 1.23 | 4.18E-02 | -0.36 | 1.03 | 1121284 | 178949 | 995920 | 265961 | 1029770 | 218076 | 962070 | 304989 |
| RP_POS_263_12778a7_279 | RP | POS | 7.28 | 263.1278 | | | | 3.31E-06 | -1.05 | 1.65 | 2.05E-01 | -0.22 | 0.84 | 1.04E-06 | -1.16 | 1.88 | 7.06E-05 | -0.95 | 1.40 | 810980 | 367774 | 430219 | 307982 | 469378 | 330794 | 391061 | 281226 |
| RP_NEG_245_75206a15_587 | RP | NEG | 15.59 | 245.7521 | | | | 3.61E-01 | -0.18 | 0.59 | 2.05E-01 | -0.26 | 0.95 | 1.58E-01 | -0.31 | 0.64 | 8.18E-01 | -0.05 | 0.32 | 2634893 | 98121 | 2616201 | 104946 | 2629539 | 108487 | 2602863 | 100596 |
| RP_POS_537_53571a14_003 | RP | POS | 14.00 | 537.5357 | | | | 5.81E-01 | 0.13 | 0.46 | 2.05E-01 | -0.23 | 0.82 | 9.46E-01 | 0.02 | 0.51 | 3.47E-01 | 0.25 | 0.60 | 13748150 | 2191223 | 13981002 | 1596945 | 14183974 | 1750937 | 13778030 | 1415153 |
| RP_POS_553_45807a10_888 | RP | POS | 10.89 | 553.4581 | | | | 8.03E-03 | -0.48 | 0.92 | 2.06E-01 | 0.26 | 1.02 | 8.14E-02 | -0.35 | 0.87 | 4.32E-03 | -0.61 | 1.04 | 3460905 | 1011900 | 2859159 | 1282277 | 2696239 | 1334855 | 3022078 | 1219025 |
| RP_POS_771_57568a13_718 | RP | POS | 13.72 | 771.5757 | | | | 7.85E-13 | 1.19 | 1.89 | 2.06E-01 | 0.24 | 1.00 | 8.24E-11 | 1.31 | 2.01 | 3.46E-09 | 1.07 | 1.88 | 11681897 | 3500093 | 18672309 | 5461878 | 17978542 | 5074868 | 19366077 | 5791069 |
| RP_POS_788_55487a13_372 | RP | POS | 13.37 | 788.5549 | PC compound | 184.0733(75), 788.5593(30), 605.4916(22), 146.9818(100), 86.0969(55), 729.4908(45), | 3 | 7.95E-01 | -0.05 | 0.53 | 2.06E-01 | -0.27 | 0.80 | 3.81E-01 | -0.18 | 0.34 | 6.99E-01 | 0.08 | 0.45 | 10523805 | 2674579 | 10373959 | 3257620 | 10787500 | 3445280 | 9960419 | 3036738 |

**Supplementary table 1: Metabolomics results** (in asceding order according to p-value from progressive ALD vs  non-progressive ALD comparison, with molecular features with ID_level of 1-2 first)

| | | | | | | | | all_ALD_vs_control | | | Prog_ALD_vs_non-prog_ALD | | | Prog_ALD_vs_control_ALD | | | Non-prog_ALD_vs_control | | | Healthy_control | | all_ALD | | Non-prog_ALD | | Progressive_ALD | |
|---|---|---|---|---|---|---|---|---|---|---|---|---|---|---|---|---|---|---|---|---|---|---|---|---|---|---|---|
| | | | | | | | | t-test | Cohen's | PLS-DA | t-test | Cohen's | PLS-DA | t-test | Cohen's | PLS-DA | t-test | Cohen's | PLS-DA | | | | | | | | |
| Feature_ID | RP_HILIC | POS_NEG | Average.Rt | Average.Mz | Metabolite.name | curated_MSMS | ID_level | p | d | VIP | p | d | VIP | p | d | VIP | p | d | VIP | Mean | SD | Mean | SD | Mean | SD | Mean | SD |
| RP_NEG_367_15927a9_588 | RP | NEG | 9.59 | 367.1593 | | | | 5.72E-05 | 0.55 | 0.96 | 2.06E-01 | 0.28 | 1.06 | 1.76E-03 | 0.69 | 1.01 | 9.74E-04 | 0.41 | 1.28 | 1861874 | 1338817 | 3646030 | 3571083 | 3192098 | 2177704 | 4099962 | 4539010 |
| HILIC_POS_157_07605a4_124 | HILIC | POS | 4.124 | 157.0761 | | | | 5.91E-03 | -0.45 | 1.08 | 2.07E-01 | -0.27 | 1.44 | 7.24E-03 | -0.59 | 1.18 | 6.42E-02 | -0.32 | 1.07 | 715220 | 116638 | 636956 | 185063 | 660438 | 145888 | 613475 | 216302 |
| HILIC_POS_497_0578a7_643 | HILIC | POS | 7.643 | 497.0578 | | | | 8.45E-01 | 0.03 | 0.85 | 2.07E-01 | -0.27 | 0.76 | 6.18E-01 | -0.10 | 0.47 | 4.19E-01 | 0.17 | 0.37 | 434665 | 16888 | 435401 | 22864 | 438297 | 23559 | 432506 | 22001 |
| RP_POS_205_01593a15_441 | RP | POS | 15.44 | 205.0159 | | | | 1.93E-01 | -0.28 | 0.62 | 2.07E-01 | 0.24 | 1.03 | 5.18E-01 | -0.16 | 0.55 | 7.15E-02 | -0.41 | 0.69 | 4203962 | 1625051 | 3780073 | 1435149 | 3598170 | 1133628 | 3961976 | 1675917 |
| HILIC_POS_267_17044a1_387 | HILIC | POS | 1.387 | 267.1704 | | | | 3.96E-02 | 0.24 | 0.89 | 2.07E-01 | 0.29 | 0.87 | 9.46E-02 | 0.38 | 0.83 | 8.86E-03 | 0.09 | 0.96 | 322880 | 910412 | 5346614 | 24042220 | 2283848 | 4977266 | 8409379 | 33525155 |
| RP_POS_176_10295a0_548 | RP | POS | 0.55 | 176.1030 | | | | 3.69E-07 | 0.82 | 1.57 | 2.07E-01 | 0.26 | 1.44 | 1.63E-06 | 0.95 | 1.76 | 2.09E-04 | 0.69 | 1.41 | 1036077 | 253198 | 1373903 | 421190 | 1320575 | 409528 | 1427231 | 430007 |
| RP_POS_699_41901a10_918 | RP | POS | 10.92 | 699.4190 | | | | 3.92E-01 | 0.10 | 0.79 | 2.07E-01 | -0.29 | 0.82 | 7.31E-01 | -0.04 | 0.52 | 2.13E-01 | 0.25 | 0.82 | 2117834 | 122655 | 2189416 | 804513 | 2291381 | 961112 | 2087451 | 602277 |
| RP_POS_585_27063a7_157 | RP | POS | 7.16 | 585.2706 | | | | 9.13E-01 | -0.02 | 0.68 | 2.07E-01 | 0.27 | 1.17 | 6.13E-01 | 0.12 | 0.39 | 3.65E-01 | -0.15 | 0.86 | 8774942 | 4906381 | 8652983 | 6905772 | 7777405 | 4720625 | 9528561 | 8514914 |
| RP_NEG_514_28595a12_402 | RP | NEG | 12.40 | 514.2860 | Likely taurine conjugated bile acid (BA 24:1;O4;T) | 514.285(100) | 3 | 6.33E-01 | 0.06 | 0.48 | 2.07E-01 | 0.29 | 1.05 | 3.80E-01 | 0.21 | 0.39 | 1.71E-01 | -0.08 | 1.10 | 410688 | 433757 | 495583 | 1598146 | 292114 | 262870 | 699053 | 2237561 |
| HILIC_NEG_212_0023a0_554 | HILIC | NEG | 0.554 | 212.0023 | | | | 7.14E-02 | -0.41 | 0.72 | 2.08E-01 | -0.24 | 0.91 | 3.56E-02 | -0.53 | 0.81 | 2.23E-01 | -0.29 | 0.54 | 2185814 | 1435136 | 1668595 | 1189645 | 1819023 | 1100349 | 1518167 | 1265877 |
| RP_POS_214_0034a0_787 | RP | POS | 0.79 | 214.0034 | | | | 2.97E-02 | -0.46 | 0.86 | 2.08E-01 | -0.25 | 0.88 | 1.27E-02 | -0.58 | 0.99 | 1.42E-01 | -0.34 | 0.68 | 2717961 | 1461342 | 2062101 | 1383254 | 2236963 | 1376280 | 1887238 | 1381767 |
| RP_POS_946_75916a0_494 | RP | POS | 0.49 | 946.7592 | | | | 1.65E-08 | -0.74 | 1.31 | 2.08E-01 | 0.27 | 1.17 | 1.34E-03 | -0.60 | 1.24 | 7.26E-08 | -0.87 | 1.62 | 1111661 | 96026 | 898889 | 309852 | 859704 | 268529 | 938074 | 344579 |
| RP_POS_162_9892a15_442 | RP | POS | 15.44 | 162.9892 | | | | 1.10E-01 | -0.37 | 0.61 | 2.08E-01 | -0.24 | 0.87 | 4.04E-02 | -0.49 | 0.93 | 3.33E-01 | -0.25 | 0.47 | 35906220 | 10535426 | 32567472 | 8465441 | 33638263 | 9843038 | 31496680 | 6750149 |
| HILIC_POS_239_14893a0_616 | HILIC | POS | 0.616 | 239.1489 | | | | 1.76E-01 | 0.23 | 1.09 | 2.08E-01 | -0.27 | 0.98 | 5.83E-01 | 0.09 | 1.11 | 1.06E-01 | 0.37 | 1.18 | 1056797 | 1245393 | 1445614 | 1806395 | 1674188 | 2166225 | 1217041 | 1339240 |
| HILIC_POS_224_12801a5_328 | HILIC | POS | 5.328 | 224.1280 | | | | 8.84E-04 | 0.57 | 1.05 | 2.08E-01 | -0.26 | 0.83 | 2.25E-02 | 0.44 | 0.79 | 6.87E-04 | 0.71 | 1.14 | 5707342 | 1634549 | 6990364 | 2314514 | 7282841 | 2401742 | 6697887 | 2209148 |
| RP_POS_787_54297a13_718 | RP | POS | 13.72 | 787.5430 | | | | 2.54E-11 | 1.11 | 1.79 | 2.08E-01 | 0.24 | 0.91 | 8.69E-10 | 1.23 | 1.93 | 8.20E-08 | 0.99 | 1.74 | 2230309 | 698614 | 3500326 | 1089856 | 3362607 | 1036952 | 3638045 | 1133948 |
| HILIC_NEG_186_95505a1_307 | HILIC | NEG | 1.307 | 186.9551 | | | | 4.32E-05 | 0.78 | 1.27 | 2.08E-01 | 0.25 | 0.93 | 5.34E-05 | 0.91 | 1.39 | 1.01E-03 | 0.66 | 1.28 | 686986 | 177640 | 852734 | 206068 | 826701 | 182660 | 878766 | 225937 |
| RP_NEG_225_06982a7_63 | RP | NEG | 7.63 | 225.0698 | | | | 1.56E-01 | 0.17 | 0.42 | 2.08E-01 | 0.29 | 1.00 | 1.63E-01 | 0.32 | 0.53 | 6.85E-01 | 0.03 | 0.89 | 654220 | 509542 | 1043924 | 2577608 | 716730 | 881092 | 1371118 | 3525467 |
| RP_POS_559_80048a10_617 | RP | POS | 10.62 | 559.8005 | | | | 2.62E-02 | -0.49 | 0.99 | 2.08E-01 | -0.24 | 1.06 | 1.23E-02 | -0.61 | 1.14 | 1.12E-01 | -0.37 | 0.71 | 642944 | 242101 | 533417 | 210680 | 560021 | 201095 | 506812 | 218620 |
| RP_NEG_221_94623a15_646 | RP | NEG | 15.65 | 221.9462 | | | | 6.02E-01 | -0.10 | 0.25 | 2.09E-01 | 0.26 | 1.15 | 8.71E-01 | 0.03 | 0.15 | 3.12E-01 | -0.23 | 0.56 | 33397366 | 2034346 | 33170807 | 2396997 | 32868295 | 2654986 | 33473319 | 2091108 |
| HILIC_POS_148_09686a4_858 | HILIC | POS | 4.858 | 148.0969 | | | | 1.12E-01 | -0.28 | 0.81 | 2.09E-01 | -0.27 | 0.84 | 4.01E-02 | -0.41 | 0.73 | 4.85E-01 | -0.15 | 0.46 | 3407048 | 544927 | 3211542 | 740216 | 3304929 | 771868 | 3118155 | 702455 |
| RP_POS_318_24008a9_299 | RP | POS | 9.30 | 318.2401 | | | | 7.12E-01 | -0.09 | 0.62 | 2.09E-01 | -0.22 | 0.75 | 4.44E-01 | -0.20 | 0.56 | 9.41E-01 | 0.02 | 0.28 | 6577106 | 5598434 | 6182797 | 3784492 | 6659951 | 3692853 | 5705644 | 3851654 |
| HILIC_NEG_129_93636a6_272 | HILIC | NEG | 6.272 | 129.9364 | | | | 7.02E-01 | -0.08 | 0.41 | 2.09E-01 | -0.25 | 0.75 | 3.61E-01 | -0.20 | 0.35 | 8.24E-01 | 0.05 | 0.25 | 2702801 | 931145 | 2629584 | 962331 | 2750915 | 980188 | 2508254 | 938217 |
| RP_POS_569_43268a11_256 | RP | POS | 11.26 | 569.4327 | | | | 7.25E-02 | 0.37 | 1.17 | 2.10E-01 | 0.25 | 0.98 | 3.63E-02 | 0.49 | 1.12 | 2.68E-01 | 0.24 | 1.01 | 2248037 | 767262 | 2534722 | 779847 | 2436531 | 709447 | 2632912 | 840086 |
| RP_POS_241_17329a8_341 | RP | POS | 8.34 | 241.1733 | | | | 7.97E-01 | 0.05 | 0.37 | 2.10E-01 | -0.25 | 0.75 | 7.52E-01 | -0.07 | 0.28 | 4.44E-01 | 0.18 | 0.37 | 12870906 | 8188799 | 13298634 | 7964307 | 14300823 | 8213746 | 12296444 | 7657642 |
| RP_NEG_880_60944a14_034 | RP | NEG | 14.03 | 880.6094 | PC 40:5\|PC 20:1_20:4 [M+HCOO]- | 329.2493(100), 283.2648(68), 820.593(30) | 3 | 4.10E-07 | 0.91 | 1.50 | 2.10E-01 | 0.25 | 0.95 | 3.07E-06 | 1.03 | 1.58 | 1.85E-05 | 0.79 | 1.52 | 3334482 | 1064243 | 4650659 | 1410005 | 4473231 | 1138265 | 4828087 | 1630002 |
| RP_POS_245_17464a8_028 | RP | POS | 8.03 | 245.1746 | | | | 1.41E-01 | 0.30 | 0.67 | 2.10E-01 | 0.25 | 0.79 | 6.59E-02 | 0.43 | 0.74 | 4.25E-01 | 0.18 | 0.60 | 587242 | 115046 | 622142 | 114388 | 607764 | 109274 | 636519 | 118632 |
| RP_NEG_197_80794a15_584 | RP | NEG | 15.58 | 197.8079 | | | | 4.09E-02 | -0.41 | 0.74 | 2.11E-01 | -0.25 | 0.87 | 1.28E-02 | -0.54 | 0.98 | 2.17E-01 | -0.29 | 0.48 | 34814028 | 964983 | 34400078 | 997849 | 34525508 | 1106393 | 34274648 | 869203 |
| RP_POS_573_25446a7_093 | RP | POS | 7.09 | 573.2545 | | | | 6.58E-01 | -0.08 | 0.50 | 2.11E-01 | 0.26 | 0.83 | 8.36E-01 | 0.05 | 0.33 | 3.05E-01 | -0.21 | 0.68 | 216332975 | 9776884 | 215414825 | 11326672 | 213991754 | 10373727 | 216837897 | 12143233 |
| RP_POS_165_09056a13_129 | RP | POS | 13.13 | 165.0906 | | | | 1.50E-01 | -0.29 | 1.02 | 2.11E-01 | -0.25 | 0.85 | 8.44E-02 | -0.42 | 0.97 | 4.22E-01 | -0.17 | 0.66 | 2849557 | 661368 | 2652638 | 670588 | 2736871 | 534706 | 2568405 | 779849 |
| RP_POS_792_55261a13_7 | RP | POS | 13.70 | 792.5526 | | | | 7.35E-01 | 0.05 | 0.78 | 2.12E-01 | 0.28 | 0.74 | 4.17E-01 | 0.19 | 0.61 | 4.73E-01 | -0.09 | 0.47 | 1028108 | 833982 | 1102186 | 1609523 | 899557 | 706444 | 1304814 | 2156650 |
| HILIC_POS_209_20125a0_511 | HILIC | POS | 0.511 | 209.2013 | | | | 1.15E-06 | 0.58 | 1.12 | 2.12E-01 | -0.28 | 0.99 | 3.16E-03 | 0.44 | 1.24 | 1.07E-04 | 0.72 | 1.34 | 106247 | 73898 | 521517 | 792913 | 620928 | 859999 | 422107 | 714484 |
| RP_POS_227_20041a8_322 | RP | POS | 8.32 | 227.2004 | | | | 8.10E-02 | -0.37 | 0.74 | 2.12E-01 | 0.24 | 0.77 | 2.89E-01 | -0.25 | 0.43 | 3.34E-02 | -0.49 | 0.94 | 993413 | 46309 | 976906 | 43430 | 971463 | 41669 | 982350 | 44879 |
| RP_NEG_271_22839a10_711 | RP | NEG | 10.71 | 271.2284 | | | | 1.09E-01 | 0.27 | 1.00 | 2.12E-01 | 0.27 | 1.01 | 6.59E-02 | 0.40 | 0.95 | 4.55E-01 | 0.13 | 0.96 | 10965169 | 1480211 | 11525977 | 2265365 | 11242177 | 1836112 | 11809776 | 2613964 |
| HILIC_NEG_115_9206a4_794 | HILIC | NEG | 4.794 | 115.9206 | | | | 8.84E-01 | 0.03 | 0.88 | 2.12E-01 | 0.25 | 1.33 | 4.88E-01 | 0.16 | 0.94 | 6.71E-01 | -0.10 | 0.17 | 71819852 | 17537991 | 72342983 | 17915795 | 70100757 | 18243246 | 74585209 | 17476429 |
| RP_POS_571_28003a8_418 | RP | POS | 8.42 | 571.2800 | | | | 9.06E-01 | 0.03 | 0.36 | 2.12E-01 | 0.24 | 0.85 | 5.84E-01 | 0.15 | 0.29 | 6.85E-01 | -0.09 | 0.53 | 780471 | 1616986 | 817982 | 1315080 | 653091 | 881001 | 982873 | 1631722 |
| HILIC_POS_138_05504a2_081 | HILIC | POS | 2.081 | 138.0550 | | | | 1.25E-01 | 0.18 | 0.33 | 2.13E-01 | 0.29 | 0.80 | 1.57E-01 | 0.32 | 0.55 | 4.57E-01 | 0.04 | 0.26 | 2681848 | 4948011 | 11527254 | 56482899 | 4422389 | 15245879 | 18632119 | 78168337 |
| RP_POS_430_04108a0_726 | RP | POS | 0.73 | 430.0411 | | | | 5.10E-09 | 0.73 | 1.19 | 2.13E-01 | 0.27 | 0.80 | 1.07E-05 | 0.87 | 1.43 | 4.01E-05 | 0.60 | 1.27 | 121693 | 92042 | 394263 | 401916 | 343960 | 335254 | 444565 | 456957 |
| HILIC_NEG_98_93883a2_941 | HILIC | NEG | 2.941 | 98.9388 | | | | 3.75E-03 | 0.48 | 1.04 | 2.13E-01 | -0.26 | 1.00 | 8.72E-02 | 0.35 | 0.76 | 1.14E-03 | 0.62 | 1.13 | 683323 | 99074 | 752290 | 150529 | 771117 | 135899 | 733464 | 163059 |
| RP_POS_288_63388a10_328 | RP | POS | 10.33 | 288.6339 | | | | 4.75E-04 | -0.77 | 1.42 | 2.13E-01 | -0.23 | 1.32 | 2.88E-04 | -0.88 | 1.51 | 4.03E-03 | -0.65 | 1.23 | 14952294 | 2242866 | 13288122 | 1986492 | 13536524 | 1811517 | 13039719 | 2136660 |
| RP_NEG_459_27383a9_902 | RP | NEG | 9.90 | 459.2738 | | | | 1.14E-01 | 0.29 | 1.10 | 2.13E-01 | 0.26 | 0.73 | 4.90E-02 | 0.42 | 0.84 | 4.38E-01 | 0.16 | 1.11 | 683649 | 726493 | 933794 | 889420 | 822598 | 875413 | 1044991 | 898163 |
| RP_POS_791_54297a12_686 | RP | POS | 12.69 | 791.5430 | | | | 1.62E-03 | 0.59 | 1.24 | 2.13E-01 | -0.25 | 0.83 | 2.84E-02 | 0.46 | 0.95 | 7.15E-04 | 0.72 | 1.25 | 3559505 | 2048272 | 5005234 | 2473538 | 5314267 | 2404579 | 4696201 | 2526804 |
| HILIC_POS_394_29507a0_626 | HILIC | POS | 0.626 | 394.2951 | | | | 1.62E-01 | 0.25 | 0.54 | 2.14E-01 | -0.26 | 1.02 | 5.48E-01 | 0.12 | 0.37 | 7.52E-02 | 0.38 | 0.63 | 436056 | 186796 | 493930 | 241396 | 524065 | 254193 | 463795 | 226419 |
| RP_NEG_378_92041a15_317 | RP | NEG | 15.32 | 378.9204 | | | | 9.21E-01 | -0.02 | 0.81 | 2.14E-01 | -0.25 | 0.77 | 4.96E-01 | -0.15 | 0.68 | 6.43E-01 | 0.11 | 0.36 | 1320532 | 67487 | 1319142 | 71456 | 1328053 | 77066 | 1310230 | 64922 |
| RP_POS_397_41544a11_669 | RP | POS | 11.67 | 397.4154 | | | | 9.59E-01 | -0.01 | 0.65 | 2.15E-01 | -0.25 | 0.74 | 5.50E-01 | -0.14 | 0.49 | 6.20E-01 | 0.11 | 0.39 | 4236235 | 1859052 | 4216946 | 1850640 | 4447510 | 1892785 | 3986382 | 1796805 |
| RP_NEG_529_46478a14_275 | RP | NEG | 14.28 | 529.4648 | | | | 3.82E-01 | -0.18 | 0.56 | 2.15E-01 | 0.25 | 0.77 | 8.03E-01 | -0.06 | 0.25 | 1.79E-01 | -0.31 | 0.62 | 986456 | 748157 | 853587 | 724827 | 763352 | 688360 | 943821 | 755654 |
| HILIC_NEG_254_97211a4_79 | HILIC | NEG | 4.79 | 254.9721 | | | | 9.83E-01 | 0.00 | 1.07 | 2.15E-01 | -0.26 | 1.10 | 5.67E-01 | -0.13 | 0.82 | 5.22E-01 | 0.13 | 1.05 | 315494 | 97725 | 315947 | 116642 | 330465 | 110164 | 301429 | 122156 |
| RP_POS_980_13605a0_488 | RP | POS | 0.49 | 980.1361 | | | | 3.21E-08 | 0.67 | 1.28 | 2.15E-01 | -0.27 | 1.21 | 3.61E-04 | 0.54 | 1.25 | 1.99E-05 | 0.81 | 1.48 | 194949 | 34825 | 352290 | 256977 | 384276 | 281462 | 320304 | 228284 |
| RP_POS_516_87354a0_496 | RP | POS | 0.50 | 516.8735 | | | | 4.45E-09 | -0.74 | 1.33 | 2.15E-01 | 0.27 | 1.19 | 2.47E-04 | -0.60 | 1.29 | 1.77E-06 | -0.87 | 1.56 | 1349347 | 76135 | 1121536 | 334692 | 1079917 | 344211 | 1163156 | 322967 |
| RP_NEG_209_83333a15_553 | RP | NEG | 15.55 | 209.8333 | | | | 6.56E-01 | -0.09 | 0.32 | 2.15E-01 | -0.26 | 0.88 | 3.38E-01 | -0.21 | 0.52 | 8.37E-01 | 0.04 | 0.44 | 1583531 | 112083 | 1572952 | 128436 | 1588922 | 120687 | 1556981 | 135065 |
| RP_NEG_680_84668a0_493 | RP | NEG | 0.49 | 680.8467 | | | | 1.30E-08 | -0.71 | 1.35 | 2.16E-01 | 0.27 | 1.26 | 5.15E-04 | -0.58 | 1.32 | 2.54E-06 | -0.85 | 1.55 | 1610026 | 143250 | 1185542 | 648511 | 1104942 | 657949 | 1266143 | 635264 |
| RP_POS_665_42072a10_463 | RP | POS | 10.46 | 665.4207 | | | | 4.10E-03 | 0.54 | 0.97 | 2.16E-01 | 0.25 | 0.75 | 2.24E-03 | 0.67 | 1.10 | 4.49E-02 | 0.42 | 0.79 | 818172 | 292724 | 1003734 | 345271 | 960834 | 332241 | 1046634 | 355973 |
| RP_NEG_636_35291a10_936 | RP | NEG | 10.94 | 636.3529 | | | | 2.32E-02 | -0.51 | 1.33 | 2.16E-01 | -0.23 | 1.73 | 1.28E-02 | -0.63 | 1.26 | 8.94E-02 | -0.39 | 1.11 | 13616235 | 2356932 | 12532009 | 1986598 | 12778755 | 1740679 | 12285264 | 2195329 |
| HILIC_POS_159_11298a4_857 | HILIC | POS | 4.857 | 159.1130 | | | | 4.18E-01 | -0.15 | 1.00 | 2.16E-01 | -0.26 | 1.08 | 2.03E-01 | -0.28 | 0.70 | 9.09E-01 | -0.02 | 0.79 | 1127392 | 317755 | 1072307 | 374242 | 1118765 | 351288 | 1025849 | 393908 |
| RP_NEG_317_05917a8_812 | RP | NEG | 8.81 | 317.0592 | | | | 2.68E-01 | -0.14 | 0.75 | 2.16E-01 | 0.28 | 0.82 | 9.69E-01 | 0.00 | 0.55 | 1.91E-01 | -0.29 | 0.69 | 74721975 | 12657642 | 69711746 | 39057783 | 64850547 | 50365916 | 74572945 | 22298006 |
| RP_NEG_351_90536a15_419 | RP | NEG | 15.42 | 351.9054 | | | | 1.50E-01 | -0.29 | 0.48 | 2.16E-01 | -0.25 | 0.95 | 5.00E-02 | -0.41 | 1.01 | 4.89E-01 | -0.16 | 0.38 | 850474 | 209130 | 787660 | 219280 | 814880 | 250398 | 760439 | 181488 |
| RP_NEG_212_00212a3_298 | RP | NEG | 3.30 | 212.0021 | | | | 6.64E-02 | -0.42 | 0.81 | 2.17E-01 | -0.23 | 0.75 | 3.27E-02 | -0.53 | 0.94 | 2.09E-01 | -0.30 | 0.54 | 27436432 | 17667973 | 20960000 | 14479430 | 22756219 | 13786944 | 19163781 | 15063935 |
| RP_POS_146_06013a4_314 | RP | POS | 4.31 | 146.0601 | | | | 2.13E-02 | -0.55 | 0.85 | 2.17E-01 | -0.22 | 0.77 | 4.38E-03 | -0.66 | 1.47 | 1.13E-01 | -0.44 | 0.56 | 1154540 | 1577083 | 432271 | 1164438 | 577414 | 1600249 | 287128 | 368393 |
| HILIC_NEG_113_92614a7_352 | HILIC | NEG | 7.352 | 113.9261 | | | | 8.51E-01 | -0.04 | 0.75 | 2.17E-01 | -0.26 | 0.92 | 4.23E-01 | -0.16 | 0.39 | 6.78E-01 | 0.09 | 0.55 | 3926277 | 476171 | 3907195 | 559809 | 3976589 | 611504 | 3837802 | 499403 |
| RP_POS_132_95853a15_618 | RP | POS | 15.62 | 132.9585 | | | | 2.17E-01 | 0.28 | 0.46 | 2.17E-01 | 0.24 | 0.82 | 1.13E-01 | 0.39 | 0.63 | 5.04E-01 | 0.16 | 0.65 | 74063130 | 12593235 | 77147414 | 10624334 | 75830925 | 9848394 | 78463903 | 11292858 |
| RP_POS_267_89459a0_252 | RP | POS | 0.25 | 267.8946 | | | | 2.62E-01 | 0.23 | 0.88 | 2.17E-01 | -0.25 | 0.95 | 6.36E-01 | 0.10 | 0.40 | 1.30E-01 | 0.35 | 0.95 | 3267807 | 319135 | 3341635 | 324286 | 3381811 | 342691 | 3301458 | 302883 |
| HILIC_NEG_133_05061a1_39 | HILIC | NEG | 1.39 | 133.0506 | | | | 1.08E-02 | 0.30 | 0.86 | 2.17E-01 | 0.28 | 0.80 | 5.08E-02 | 0.44 | 0.67 | 1.51E-02 | 0.16 | 0.84 | 54566 | 80906 | 593062 | 2068252 | 335667 | 783408 | 850456 | 2809573 |
| RP_POS_1030_72058a0_496 | RP | POS | 0.50 | 1030.7206 | | | | 4.02E-01 | -0.19 | 0.43 | 2.17E-01 | -0.24 | 0.77 | 2.14E-01 | -0.31 | 0.60 | 7.73E-01 | -0.07 | 0.45 | 566679 | 142504 | 543072 | 120116 | 557951 | 116258 | 528194 | 123217 |
| RP_POS_281_20792a10_403 | RP | POS | 10.40 | 281.2079 | | | | 9.71E-01 | 0.00 | 0.63 | 2.17E-01 | 0.28 | 0.77 | 5.45E-01 | 0.14 | 0.55 | 1.83E-01 | -0.14 | 0.59 | 3021615 | 234222 | 3018862 | 645386 | 2938673 | 323514 | 3099051 | 850743 |
| RP_POS_146_06009a5_026 | RP | POS | 5.03 | 146.0601 | | | | 9.36E-01 | -0.02 | 0.36 | 2.18E-01 | -0.24 | 0.77 | 5.66E-01 | -0.14 | 0.51 | 6.75E-01 | 0.10 | 0.42 | 425825 | 295632 | 421130 | 255934 | 452804 | 261480 | 389456 | 248840 |
| RP_POS_591_26056a6_357 | RP | POS | 6.36 | 591.2606 | | | | 7.71E-01 | 0.05 | 0.41 | 2.18E-01 | 0.26 | 0.74 | 4.13E-01 | 0.18 | 0.41 | 7.10E-01 | -0.08 | 0.59 | 1070362 | 64381 | 1074393 | 77700 | 1064775 | 68729 | 1084010 | 85356 |
| HILIC_POS_123_05552a0_611 | HILIC | POS | 0.611 | 123.0555 | | | | 6.55E-01 | -0.08 | 0.64 | 2.18E-01 | -0.26 | 0.92 | 2.80E-01 | -0.21 | 0.40 | 8.10E-01 | 0.05 | 0.64 | 385485 | 178020 | 367777 | 237858 | 397206 | 260899 | 338347 | 210883 |
| RP_POS_417_22815a7_549 | RP | POS | 7.55 | 417.2282 | | | | 2.23E-01 | -0.25 | 0.84 | 2.18E-01 | 0.25 | 0.93 | 5.80E-01 | -0.12 | 0.76 | 1.09E-01 | -0.37 | 0.80 | 613158 | 43412 | 602180 | 45020 | 596612 | 47507 | 607749 | 42128 |
| RP_POS_587_43268a13_497 | RP | POS | 13.50 | 587.4327 | | | | 4.22E-01 | 0.11 | 0.42 | 2.18E-01 | 0.28 | 0.83 | 2.60E-01 | 0.25 | 0.50 | 7.95E-01 | -0.03 | 0.31 | 770424 | 111351 | 799056 | 296007 | 762345 | 168621 | 835768 | 381896 |
| RP_POS_332_96414a6_184 | RP | POS | 6.18 | 332.9641 | | | | 1.21E-01 | 0.29 | 1.07 | 2.18E-01 | -0.26 | 0.88 | 4.16E-01 | 0.16 | 0.77 | 6.62E-02 | 0.42 | 1.01 | 175165 | 210269 | 245741 | 252406 | 276964 | 283119 | 214518 | 215806 |
| RP_NEG_207_93074a1_582 | RP | NEG | 1.58 | 207.9307 | | | | 3.57E-01 | 0.18 | 0.55 | 2.18E-01 | 0.25 | 1.00 | 1.76E-01 | 0.30 | 0.64 | 8.09E-01 | 0.05 | 0.71 | 1419562 | 674532 | 1550356 | 752281 | 1457362 | 705251 | 1643349 | 792701 |
| RP_POS_140_95232a15_38 | RP | POS | 15.38 | 140.9523 | | | | 6.91E-01 | -0.08 | 0.20 | 2.19E-01 | -0.26 | 0.83 | 3.37E-01 | -0.20 | 0.44 | 8.09E-01 | 0.05 | 0.39 | 26402086 | 753830 | 26338350 | 880113 | 26447043 | 913982 | 26229658 | 839961 |
| HILIC_NEG_200_85866a3_965 | HILIC | NEG | 3.965 | 200.8587 | | | | 9.61E-01 | -0.01 | 0.30 | 2.19E-01 | -0.25 | 0.88 | 5.50E-01 | -0.13 | 0.45 | 6.11E-01 | 0.11 | 0.23 | 2439508 | 825925 | 2431113 | 851817 | 2536260 | 852545 | 2325967 | 846520 |
| RP_POS_321_24002a10_333 | RP | POS | 10.33 | 321.2400 | | | | 1.67E-01 | 0.22 | 0.66 | 2.19E-01 | -0.27 | 0.77 | 5.95E-01 | 0.09 | 0.48 | 1.03E-01 | 0.36 | 0.66 | 4196248 | 1241855 | 4613339 | 2031477 | 4864266 | 2405114 | 4362413 | 1557226 |
| RP_POS_260_1601a0_705 | RP | POS | 0.71 | 260.1601 | | | | 8.56E-01 | 0.02 | 0.58 | 2.19E-01 | -0.28 | 0.78 | 7.07E-02 | -0.12 | 0.73 | 4.79E-01 | 0.16 | 0.45 | 514878 | 472793 | 551188 | 1810720 | 775831 | 2519296 | 326545 | 417715 |
| RP_POS_912_51154a12_958 | RP | POS | 12.96 | 912.5115 | | | | 4.31E-04 | -0.76 | 1.36 | 2.19E-01 | -0.23 | 0.87 | 2.05E-04 | -0.88 | 1.45 | 4.74E-03 | -0.65 | 1.13 | 819201 | 433028 | 493932 | 393420 | 542425 | 390869 | 445439 | 393878 |
| HILIC_NEG_164_83583a3_005 | HILIC | NEG | 3.005 | 164.8358 | | | | 7.94E-01 | 0.06 | 0.82 | 2.20E-01 | -0.24 | 0.84 | 7.78E-01 | -0.07 | 0.38 | 4.51E-01 | 0.18 | 0.78 | 23271513 | 6819146 | 23628849 | 6354283 | 24411580 | 6332665 | 22846118 | 6342306 |
| RP_POS_313_64136a10_446 | RP | POS | 10.45 | 313.6414 | | | | 1.71E-03 | 0.60 | 1.23 | 2.20E-01 | -0.25 | 1.00 | 2.85E-02 | 0.48 | 0.98 | 6.16E-04 | 0.73 | 1.37 | 630981 | 333215 | 861770 | 382519 | 908864 | 355520 | 814676 | 405841 |
| HILIC_POS_117_52026a3_689 | HILIC | POS | 3.689 | 117.5203 | | | | 9.21E-02 | 0.29 | 0.58 | 2.20E-01 | 0.26 | 1.03 | 6.16E-02 | 0.42 | 0.74 | 3.59E-01 | 0.16 | 0.39 | 1031878 | 725386 | 1309578 | 1000904 | 1186342 | 760389 | 1432813 | 1189485 |
| RP_NEG_201_03471a0_611 | RP | NEG | 0.61 | 201.0347 | | | | 2.48E-01 | 0.23 | 0.43 | 2.21E-01 | -0.25 | 0.81 | 6.38E-01 | 0.10 | 0.31 | 1.18E-01 | 0.35 | 0.72 | 2100111 | 718454 | 2274309 | 781256 | 2370372 | 807067 | 2178247 | 750326 |
| RP_POS_997_62421a10_332 | RP | POS | 10.33 | 997.6242 | | | | 9.68E-01 | 0.01 | 0.96 | 2.21E-01 | -0.24 | 1.12 | 6.62E-01 | -0.11 | 0.69 | 5.78E-01 | 0.13 | 0.87 | 5830274 | 3911893 | 5860936 | 3391412 | 6278125 | 2855457 | 5443747 | 3837870 |
| RP_POS_199_13046a13_638 | RP | POS | 13.64 | 199.1305 | | | | 2.25E-01 | 0.25 | 0.87 | 2.21E-01 | -0.24 | 0.94 | 5.65E-01 | 0.13 | 0.54 | 1.06E-01 | 0.38 | 0.76 | 27479560 | 11982813 | 30441508 | 11515577 | 31855906 | 11448164 | 29027111 | 11523029 |
| RP_POS_366_18594a10_65 | RP | POS | 10.65 | 366.1859 | | | | 8.76E-01 | -0.02 | 0.57 | 2.21E-01 | 0.28 | 0.77 | 6.04E-01 | 0.12 | 0.51 | 1.36E-01 | -0.16 | 0.63 | 431239 | 97700 | 426442 | 254415 | 395078 | 118043 | 457807 | 338871 |
| HILIC_POS_363_03571a6_299 | HILIC | POS | 6.299 | 363.0357 | | | | 3.19E-01 | -0.21 | 0.48 | 2.21E-01 | -0.24 | 0.72 | 1.58E-01 | -0.33 | 0.55 | 6.98E-01 | -0.09 | 0.23 | 1694644 | 653201 | 1562923 | 615778 | 1638507 | 607185 | 1487340 | 621105 |
| RP_POS_301_21057a11_161 | RP | POS | 11.16 | 301.2106 | | | | 7.06E-01 | 0.07 | 0.84 | 2.21E-01 | 0.26 | 0.97 | 3.86E-01 | 0.20 | 0.73 | 7.70E-01 | -0.06 | 0.85 | 3205362 | 690468 | 3261338 | 829306 | 3159496 | 690182 | 3363180 | 944466 |
| RP_POS_301_63004a10_303 | RP | POS | 10.30 | 301.6300 | | | | 3.53E-03 | 0.68 | 1.14 | 2.21E-01 | -0.22 | 0.85 | 2.33E-02 | 0.56 | 0.90 | 1.29E-03 | 0.79 | 1.22 | 936604 | 313233 | 1123845 | 249847 | 1154513 | 227350 | 1093177 | 269283 |
| RP_POS_477_10568a7_035 | RP | POS | 7.04 | 477.1057 | | | | 2.79E-01 | -0.17 | 0.78 | 2.22E-01 | 0.27 | 1.21 | 8.72E-01 | -0.04 | 0.81 | 3.00E-02 | -0.31 | 1.07 | 2084716 | 1586723 | 1662318 | 2680531 | 1332388 | 1335092 | 1992248 | 3537302 |

**Supplementary table 1: Metabolomics results** (in asceding order according to p-value from progressive ALD vs  non-progressive ALD comparison, with molecular features with ID_level of 1-2 first)

| | | | | | | | | all_ALD_vs_control | | | Prog_ALD_vs_non-prog_ALD | | | Prog_ALD_vs_control_ALD | | | Non-prog_ALD_vs_control | | | Healthy_control | | all_ALD | | Non-prog_ALD | | Progressive_ALD | |
|---|---|---|---|---|---|---|---|---|---|---|---|---|---|---|---|---|---|---|---|---|---|---|---|---|---|---|---|
| | | | | | | | | t-test | Cohen's | PLS-DA | t-test | Cohen's | PLS-DA | t-test | Cohen's | PLS-DA | t-test | Cohen's | PLS-DA | | | | | | | | |
| Feature_ID | RP_HILIC | POS_NEG | Average.Rt | Average.Mz | Metabolite.name | curated_MSMS | ID_level | p | d | VIP | p | d | VIP | p | d | VIP | p | d | VIP | Mean | SD | Mean | SD | Mean | SD | Mean | SD |
| RP_POS_818_50824a12_693 | RP | POS | 12.69 | 818.5082 | | | | 1.13E-03 | 0.57 | 1.20 | 2.22E-01 | -0.25 | 0.83 | 3.00E-02 | 0.44 | 0.97 | 5.96E-04 | 0.69 | 1.24 | 3794310 | 1700462 | 5094619 | 2380408 | 5386310 | 2318592 | 4802927 | 2428747 |
| RP_POS_341_17776a11_248 | RP | POS | 11.25 | 341.1778 | | | | 3.21E-05 | 0.78 | 1.63 | 2.22E-01 | 0.24 | 1.14 | 4.87E-05 | 0.90 | 1.61 | 8.75E-04 | 0.66 | 1.45 | 2748401 | 600906 | 3325716 | 728757 | 3236438 | 648492 | 3414995 | 797624 |
| RP_POS_312_32599a11_601 | RP | POS | 11.60 | 312.3260 | | | | 3.61E-08 | 0.74 | 1.30 | 2.22E-01 | 0.26 | 1.28 | 4.92E-05 | 0.87 | 1.39 | 2.86E-06 | 0.61 | 1.47 | 1772391 | 631771 | 2992028 | 1754592 | 2776718 | 1164801 | 3207338 | 2183722 |
| HILIC_NEG_203_08269a4_014 | HILIC | NEG | 4.014 | 203.0827 | | | | 5.77E-03 | -0.44 | 1.06 | 2.23E-01 | -0.26 | 1.43 | 7.06E-03 | -0.58 | 1.18 | 6.81E-02 | -0.31 | 1.05 | 315254 | 55550 | 277295 | 91344 | 288484 | 75167 | 266106 | 104652 |
| RP_POS_853_50909a10_868 | RP | POS | 10.87 | 853.5091 | | | | 2.27E-05 | 0.51 | 1.16 | 2.23E-01 | -0.27 | 1.15 | 6.56E-03 | 0.37 | 1.20 | 9.66E-04 | 0.65 | 1.25 | 612222 | 201903 | 1320410 | 1558868 | 1511228 | 1796847 | 1129592 | 1267582 |
| HILIC_NEG_157_01199a1_302 | HILIC | NEG | 1.302 | 157.0120 | | | | 9.33E-08 | 0.84 | 1.49 | 2.23E-01 | 0.25 | 0.94 | 1.29E-06 | 0.96 | 1.57 | 7.05E-05 | 0.71 | 1.34 | 1194897 | 328628 | 1669407 | 578424 | 1598655 | 542885 | 1740158 | 609127 |
| RP_POS_441_29495a14_166 | RP | POS | 14.17 | 441.2950 | | | | 3.88E-01 | 0.17 | 0.53 | 2.24E-01 | -0.25 | 0.72 | 8.32E-01 | 0.05 | 0.65 | 1.82E-01 | 0.30 | 0.72 | 15441881 | 7287601 | 16747578 | 7719740 | 17690098 | 7496886 | 15805057 | 7898654 |
| RP_POS_864_51117a13_159 | RP | POS | 13.16 | 864.5112 | | | | 1.97E-01 | -0.28 | 0.73 | 2.24E-01 | -0.24 | 0.83 | 9.45E-02 | -0.40 | 0.73 | 5.01E-01 | -0.16 | 0.54 | 2666110 | 878897 | 2436680 | 812092 | 2535817 | 804109 | 2337544 | 815964 |
| RP_NEG_674_29102a10_124 | RP | NEG | 10.12 | 674.2910 | | | | 1.22E-19 | 1.50 | 2.44 | 2.24E-01 | 0.20 | 0.77 | 3.85E-15 | 1.60 | 2.42 | 2.47E-16 | 1.40 | 2.45 | 696608 | 233799 | 1353795 | 362938 | 1309472 | 295518 | 1398118 | 418086 |
| RP_NEG_170_94081a15_774 | RP | NEG | 15.77 | 170.9408 | | | | 8.71E-01 | -0.04 | 0.34 | 2.24E-01 | -0.22 | 0.73 | 5.68E-01 | -0.15 | 0.48 | 7.83E-01 | 0.07 | 0.31 | 1135898 | 507780 | 1120036 | 362145 | 1164210 | 345581 | 1075862 | 376252 |
| RP_NEG_626_2807a10_508 | RP | NEG | 10.51 | 626.2807 | | | | 9.94E-02 | -0.36 | 0.97 | 2.25E-01 | 0.23 | 0.89 | 3.07E-01 | -0.25 | 0.69 | 4.44E-02 | -0.48 | 1.05 | 2522830 | 804271 | 2256073 | 705026 | 2170170 | 679615 | 2341977 | 726176 |
| RP_POS_259_08414a6_888 | RP | POS | 6.89 | 259.0841 | | | | 9.41E-01 | 0.01 | 0.64 | 2.25E-01 | -0.28 | 0.92 | 1.01E-01 | -0.13 | 0.68 | 5.24E-01 | 0.15 | 0.72 | 5524597 | 4114941 | 5632336 | 12569046 | 7172072 | 17432948 | 4092600 | 3231082 |
| RP_POS_807_56995a13_801 | RP | POS | 13.80 | 807.5700 | | | | 1.51E-01 | 0.25 | 0.92 | 2.25E-01 | -0.26 | 0.89 | 5.29E-01 | 0.12 | 0.71 | 7.58E-02 | 0.38 | 0.99 | 1278008 | 1034414 | 1613001 | 1410486 | 1784777 | 1515527 | 1441224 | 1289380 |
| RP_POS_306_95697a0_522 | RP | POS | 0.52 | 306.9570 | | | | 1.20E-04 | 0.47 | 1.10 | 2.25E-01 | -0.27 | 1.14 | 2.64E-02 | 0.33 | 0.95 | 1.19E-03 | 0.61 | 1.28 | 200538 | 37492 | 284237 | 199593 | 308544 | 218091 | 259930 | 178097 |
| RP_POS_550_38647a10_9 | RP | POS | 10.90 | 550.3865 | | | | 2.82E-01 | -0.23 | 0.74 | 2.25E-01 | 0.24 | 1.42 | 6.52E-01 | -0.11 | 0.50 | 1.22E-01 | -0.35 | 0.87 | 698778 | 351722 | 622349 | 327598 | 582469 | 284745 | 662228 | 364014 |
| RP_POS_194_11497a10_271 | RP | POS | 10.27 | 194.1150 | | | | 2.37E-01 | -0.24 | 0.92 | 2.26E-01 | 0.24 | 0.85 | 6.22E-01 | -0.12 | 0.77 | 9.14E-02 | -0.36 | 0.81 | 895453 | 68820 | 878582 | 71158 | 869929 | 60580 | 887236 | 80047 |
| HILIC_POS_203_15009a6_433 | HILIC | POS | 6.433 | 203.1501 | | | | 6.34E-03 | 0.43 | 0.95 | 2.26E-01 | 0.26 | 0.96 | 7.01E-03 | 0.56 | 0.89 | 8.72E-02 | 0.30 | 1.02 | 3939384 | 1169131 | 4753071 | 2053969 | 4503511 | 1780476 | 5002631 | 2286149 |
| RP_POS_307_22443a10_564 | RP | POS | 10.56 | 307.2244 | | | | 4.18E-03 | -0.55 | 1.15 | 2.26E-01 | 0.24 | 1.08 | 5.58E-02 | -0.43 | 1.09 | 1.25E-03 | -0.67 | 1.16 | 758803 | 83431 | 706378 | 96008 | 694717 | 84799 | 718039 | 105616 |
| RP_NEG_411_89331a15_708 | RP | NEG | 15.71 | 411.8933 | | | | 9.84E-02 | -0.30 | 0.71 | 2.27E-01 | -0.25 | 0.84 | 5.53E-02 | -0.43 | 0.75 | 3.77E-01 | -0.17 | 0.65 | 811237 | 94385 | 776685 | 120986 | 791375 | 104901 | 761996 | 134646 |
| RP_POS_518_3244a10_041 | RP | POS | 10.04 | 518.3244 | | | | 4.39E-02 | 0.38 | 0.93 | 2.27E-01 | -0.25 | 0.86 | 2.41E-01 | 0.26 | 0.72 | 1.67E-02 | 0.50 | 1.10 | 18174279 | 13457563 | 24064618 | 15923980 | 25996872 | 15051085 | 22132365 | 16678323 |
| RP_POS_194_00746a15_547 | RP | POS | 15.55 | 194.0075 | | | | 1.59E-02 | -0.38 | 1.08 | 2.27E-01 | -0.26 | 1.14 | 1.75E-02 | -0.51 | 1.06 | 1.36E-01 | -0.25 | 0.97 | 5224113 | 407888 | 4974382 | 717098 | 5061455 | 567288 | 4887309 | 837654 |
| RP_NEG_410_6319a15_584 | RP | NEG | 15.58 | 410.6319 | | | | 4.60E-01 | -0.15 | 0.48 | 2.27E-01 | -0.24 | 0.72 | 2.08E-01 | -0.27 | 0.53 | 9.09E-01 | -0.03 | 0.25 | 724360 | 36541 | 718807 | 37508 | 723358 | 41696 | 714257 | 32583 |
| RP_NEG_155_86446a15_588 | RP | NEG | 15.59 | 155.8645 | | | | 4.16E-01 | -0.15 | 0.33 | 2.27E-01 | -0.25 | 0.76 | 1.99E-01 | -0.28 | 0.65 | 9.02E-01 | -0.03 | 0.31 | 495853 | 23439 | 491758 | 28000 | 495153 | 27310 | 488362 | 28541 |
| RP_POS_378_90021a0_497 | RP | POS | 0.50 | 378.9002 | | | | 3.25E-09 | -0.72 | 1.35 | 2.27E-01 | 0.26 | 1.23 | 2.24E-04 | -0.59 | 1.33 | 3.29E-06 | -0.85 | 1.55 | 34962004 | 933651 | 29694375 | 7992550 | 28725180 | 8362671 | 30663570 | 7564001 |
| RP_POS_585_27014a11_669 | RP | POS | 11.67 | 585.2701 | | | | 6.79E-01 | -0.09 | 0.54 | 2.27E-01 | 0.24 | 1.19 | 9.15E-01 | 0.03 | 0.36 | 3.07E-01 | -0.21 | 0.73 | 952703 | 1693505 | 812942 | 1511699 | 628900 | 637327 | 996984 | 2035140 |
| RP_POS_179_10689a7_866 | RP | POS | 7.87 | 179.1069 | | | | 7.66E-02 | -0.39 | 0.61 | 2.27E-01 | -0.23 | 0.76 | 3.89E-02 | -0.50 | 0.80 | 2.43E-01 | -0.27 | 0.49 | 1932199 | 112461 | 1891954 | 99489 | 1904016 | 93373 | 1879893 | 104798 |
| RP_NEG_414_62457a15_583 | RP | NEG | 15.58 | 414.6246 | | | | 5.04E-01 | -0.13 | 0.51 | 2.27E-01 | -0.25 | 0.86 | 2.45E-01 | -0.26 | 0.60 | 9.68E-01 | -0.01 | 0.30 | 1586780 | 77393 | 1576022 | 82711 | 1586045 | 85115 | 1565999 | 79827 |
| RP_NEG_259_85715a15_626 | RP | NEG | 15.63 | 259.8572 | | | | 1.71E-01 | 0.28 | 0.80 | 2.27E-01 | -0.24 | 0.84 | 5.08E-01 | 0.15 | 0.31 | 6.70E-02 | 0.40 | 0.90 | 795583 | 52070 | 810382 | 53896 | 816914 | 48071 | 803851 | 58915 |
| RP_POS_163_97653a10_57 | RP | POS | 10.57 | 163.9765 | | | | 2.83E-01 | -0.22 | 0.57 | 2.28E-01 | 0.24 | 0.95 | 6.41E-01 | -0.10 | 0.37 | 1.49E-01 | -0.34 | 0.61 | 2572239 | 802366 | 2396811 | 776276 | 2302785 | 834455 | 2490837 | 709329 |
| RP_POS_479_39282a10_88 | RP | POS | 10.88 | 479.3928 | | | | 9.72E-01 | -0.01 | 0.73 | 2.28E-01 | 0.25 | 1.33 | 6.06E-01 | 0.12 | 0.55 | 5.63E-01 | -0.13 | 0.62 | 3625973 | 367293 | 3623335 | 383021 | 3576951 | 381545 | 3669719 | 382671 |
| HILIC_NEG_114_02457a1_298 | HILIC | NEG | 1.298 | 114.0246 | | | | 1.18E-07 | 0.82 | 1.44 | 2.28E-01 | 0.25 | 0.94 | 9.06E-07 | 0.95 | 1.58 | 1.46E-04 | 0.70 | 1.26 | 496518 | 149012 | 711755 | 268370 | 679264 | 264847 | 744247 | 270585 |
| HILIC_POS_659_76855a0_575 | HILIC | POS | 0.575 | 659.7686 | | | | 3.46E-06 | 0.60 | 1.28 | 2.29E-01 | -0.27 | 0.96 | 1.13E-03 | 0.46 | 1.43 | 2.81E-04 | 0.73 | 1.45 | 56820 | 213423 | 463959 | 749494 | 554601 | 870058 | 373317 | 600982 |
| RP_NEG_178_81479a15_52 | RP | NEG | 15.52 | 178.8148 | | | | 7.14E-01 | -0.07 | 0.60 | 2.29E-01 | -0.25 | 0.79 | 3.33E-01 | -0.19 | 0.57 | 7.89E-01 | 0.06 | 0.37 | 2617674 | 100360 | 2609715 | 124191 | 2624722 | 136522 | 2594709 | 109836 |
| HILIC_NEG_134_89464a4_771 | HILIC | NEG | 4.771 | 134.8946 | | | | 1.68E-01 | -0.24 | 0.48 | 2.29E-01 | 0.26 | 0.82 | 5.51E-01 | -0.11 | 0.63 | 9.01E-02 | -0.37 | 0.58 | 15896576 | 3317467 | 14865077 | 4520044 | 14318884 | 5009648 | 15411270 | 3946283 |
| RP_NEG_387_18552a8_139 | RP | NEG | 8.14 | 387.1855 | | | | 1.27E-01 | 0.24 | 0.58 | 2.29E-01 | 0.26 | 0.76 | 9.55E-02 | 0.37 | 0.65 | 4.82E-01 | 0.11 | 0.59 | 1107788 | 1196572 | 1560863 | 2048384 | 1313107 | 1405662 | 1808620 | 2525119 |
| HILIC_NEG_136_89163a0_824 | HILIC | NEG | 0.824 | 136.8916 | | | | 7.46E-01 | -0.06 | 0.75 | 2.29E-01 | -0.25 | 0.93 | 4.04E-01 | -0.19 | 0.30 | 7.77E-01 | 0.06 | 0.15 | 6046764 | 973275 | 5980712 | 1078174 | 6110875 | 1032668 | 5850550 | 1116989 |
| HILIC_NEG_132_90002a0_93 | HILIC | NEG | 0.93 | 132.9000 | | | | 4.86E-02 | 0.38 | 1.14 | 2.30E-01 | 0.25 | 0.94 | 1.52E-02 | 0.50 | 1.21 | 2.60E-01 | 0.26 | 0.88 | 1475714 | 368429 | 1631796 | 420282 | 1581091 | 468805 | 1682501 | 363118 |
| RP_NEG_213_02251a6_647 | RP | NEG | 6.65 | 213.0225 | | | | 1.25E-01 | 0.18 | 0.54 | 2.30E-01 | 0.28 | 0.81 | 1.60E-01 | 0.32 | 0.72 | 4.47E-01 | 0.04 | 0.45 | 7958113 | 14952849 | 26709835 | 118521368 | 12349551 | 36080281 | 41070118 | 163274830 |
| RP_NEG_299_73135a15_599 | RP | NEG | 15.60 | 299.7314 | | | | 1.60E-01 | -0.30 | 0.81 | 2.30E-01 | -0.23 | 0.71 | 8.34E-02 | -0.42 | 0.73 | 4.18E-01 | -0.19 | 0.72 | 1152212 | 60998 | 1134907 | 55559 | 1141600 | 51443 | 1128214 | 59150 |
| RP_POS_323_25568a10_287 | RP | POS | 10.29 | 323.2557 | | | | 1.48E-01 | 0.25 | 0.91 | 2.30E-01 | 0.26 | 1.10 | 9.19E-02 | 0.37 | 0.84 | 5.07E-01 | 0.12 | 0.75 | 4280610 | 600166 | 4481689 | 873941 | 4376340 | 685492 | 4587038 | 1024979 |
| HILIC_NEG_199_03789a2_172 | HILIC | NEG | 2.172 | 199.0379 | | | | 3.57E-01 | 0.18 | 0.51 | 2.30E-01 | -0.24 | 0.95 | 7.72E-01 | 0.06 | 0.24 | 1.88E-01 | 0.31 | 0.53 | 411095 | 176967 | 444895 | 185590 | 467251 | 201199 | 422539 | 167598 |
| RP_NEG_244_90852a8_371 | RP | NEG | 8.37 | 244.9085 | | | | 6.79E-02 | -0.40 | 1.16 | 2.30E-01 | -0.23 | 0.74 | 2.42E-02 | -0.51 | 1.14 | 2.54E-01 | -0.28 | 0.50 | 1672792 | 949551 | 1321315 | 850126 | 1423752 | 967465 | 1218878 | 709067 |
| RP_POS_192_13838a0_883 | RP | POS | 0.88 | 192.1384 | Unknown | 192.1387(100), 133.0642(40), 58.0657(10) | 4 | 9.29E-02 | 0.34 | 0.69 | 2.30E-01 | -0.24 | 0.74 | 3.24E-01 | 0.22 | 0.49 | 4.47E-02 | 0.46 | 0.86 | 27168929 | 4705524 | 28821319 | 4910252 | 29412523 | 5062664 | 28230115 | 4729621 |
| RP_NEG_215_01816a6_646 | RP | NEG | 6.65 | 215.0182 | | | | 1.30E-01 | 0.18 | 0.54 | 2.30E-01 | 0.28 | 0.82 | 1.64E-01 | 0.32 | 0.72 | 4.68E-01 | 0.04 | 0.44 | 371455 | 681797 | 1196531 | 5275781 | 558103 | 1597242 | 1834958 | 7269990 |
| RP_POS_181_07205a2_679 | RP | POS | 2.68 | 181.0721 | | | | 7.97E-04 | -0.95 | 1.87 | 2.31E-01 | 0.17 | 0.73 | 2.99E-03 | -0.86 | 1.38 | 3.31E-04 | -1.03 | 1.92 | 21902375 | 20327949 | 8234285 | 10076949 | 7020208 | 7833367 | 9448362 | 11865597 |
| RP_POS_299_25787a10_54 | RP | POS | 10.54 | 299.2579 | | | | 1.15E-01 | -0.30 | 0.86 | 2.31E-01 | 0.25 | 0.96 | 4.01E-01 | -0.18 | 0.74 | 5.28E-02 | -0.43 | 0.86 | 2762769 | 405179 | 2626992 | 456247 | 2572114 | 460666 | 2681871 | 449680 |
| RP_NEG_261_85083a15_621 | RP | NEG | 15.62 | 261.8508 | | | | 2.07E-01 | -0.24 | 0.78 | 2.31E-01 | 0.25 | 0.90 | 5.96E-01 | -0.11 | 0.51 | 9.33E-02 | -0.36 | 1.15 | 8695299 | 452421 | 8572680 | 533501 | 8508517 | 532502 | 8636844 | 532067 |
| RP_POS_949_6264a10_511 | RP | POS | 10.51 | 949.6264 | | | | 3.51E-01 | 0.16 | 1.00 | 2.31E-01 | -0.26 | 0.97 | 8.67E-01 | 0.04 | 0.72 | 1.33E-01 | 0.29 | 0.90 | 2619775 | 853824 | 2797934 | 1152925 | 2936621 | 1017898 | 2659246 | 1268948 |
| RP_POS_143_01675a14_268 | RP | POS | 14.27 | 143.0168 | | | | 6.38E-01 | 0.07 | 0.40 | 2.31E-01 | 0.26 | 0.76 | 3.51E-01 | 0.21 | 0.46 | 7.02E-01 | -0.06 | 0.39 | 5271333 | 588315 | 5341153 | 1051071 | 5214577 | 744483 | 5467728 | 1282612 |
| HILIC_NEG_99_92564a1_294 | HILIC | NEG | 1.294 | 99.9256 | | | | 8.09E-01 | 0.05 | 0.63 | 2.32E-01 | 0.24 | 0.87 | 4.66E-01 | 0.17 | 0.33 | 7.64E-01 | -0.07 | 0.13 | 5585270 | 1631754 | 5665128 | 1574947 | 5476069 | 1541452 | 5854187 | 1600787 |
| RP_POS_888_80432a0_493 | RP | POS | 0.49 | 888.8043 | | | | 3.30E-03 | 0.39 | 1.03 | 2.32E-01 | 0.27 | 1.30 | 6.83E-03 | 0.53 | 1.12 | 9.44E-02 | 0.26 | 1.09 | 526672 | 193584 | 718831 | 542915 | 653640 | 471080 | 784022 | 604102 |
| RP_NEG_704_33734a10_94 | RP | NEG | 10.94 | 704.3373 | | | | 2.00E-01 | -0.29 | 1.33 | 2.32E-01 | -0.23 | 1.14 | 1.08E-01 | -0.40 | 1.07 | 4.64E-01 | -0.17 | 1.05 | 2439655 | 525664 | 2305678 | 444341 | 2359016 | 405992 | 2252340 | 477777 |
| RP_POS_514_03711a0_711 | RP | POS | 0.71 | 514.0371 | | | | 3.81E-01 | -0.13 | 0.85 | 2.32E-01 | 0.27 | 1.01 | 9.94E-01 | 0.00 | 0.77 | 1.26E-01 | -0.26 | 0.94 | 589623 | 120982 | 561549 | 236534 | 533158 | 209378 | 589941 | 259913 |
| HILIC_POS_143_11786a1_548 | HILIC | POS | 1.548 | 143.1179 | | | | 4.05E-02 | 0.37 | 0.78 | 2.33E-01 | 0.25 | 0.88 | 2.59E-02 | 0.49 | 0.86 | 2.05E-01 | 0.25 | 0.46 | 2530779 | 1019716 | 2996301 | 1309547 | 2839272 | 1132276 | 3153329 | 1460292 |
| RP_POS_311_1553a5_624 | RP | POS | 5.62 | 311.1553 | | | | 1.91E-01 | 0.24 | 0.70 | 2.33E-01 | 0.25 | 0.91 | 1.15E-01 | 0.36 | 1.13 | 5.44E-01 | 0.11 | 0.63 | 694830 | 1010906 | 983381 | 1257963 | 832494 | 975250 | 1134268 | 1483125 |
| RP_NEG_230_0128a2_409 | RP | NEG | 2.41 | 230.0128 | | | | 1.17E-01 | 0.21 | 0.43 | 2.33E-01 | -0.27 | 0.76 | 4.03E-01 | 0.07 | 0.74 | 1.40E-01 | 0.34 | 0.47 | 1685829 | 2311289 | 2935010 | 6786491 | 3752163 | 9321297 | 2117856 | 2191700 |
| RP_POS_320_25604a9_979 | RP | POS | 9.98 | 320.2560 | | | | 2.80E-01 | -0.30 | 0.97 | 2.33E-01 | -0.19 | 0.88 | 1.66E-01 | -0.39 | 0.87 | 4.81E-01 | -0.21 | 0.64 | 5788274 | 7735964 | 4224143 | 4066837 | 4711610 | 4585506 | 3736676 | 3450223 |
| RP_NEG_256_98642a7_425 | RP | NEG | 7.43 | 256.9864 | | | | 1.20E-01 | -0.26 | 0.58 | 2.33E-01 | -0.26 | 0.77 | 8.46E-02 | -0.39 | 0.69 | 4.23E-01 | -0.14 | 0.42 | 6416076 | 346380 | 6291596 | 502190 | 6351843 | 360944 | 6231350 | 609794 |
| RP_NEG_855_66193a14_829 | RP | NEG | 14.83 | 855.6619 | | | | 5.89E-03 | -0.68 | 1.20 | 2.33E-01 | -0.21 | 0.72 | 2.76E-03 | -0.78 | 1.30 | 2.53E-02 | -0.57 | 0.96 | 4536821 | 2008977 | 3426845 | 1411482 | 3595753 | 1424785 | 3257938 | 1391758 |
| HILIC_POS_663_45471a10_694 | HILIC | POS | 10.694 | 663.4547 | | | | 3.51E-02 | -0.39 | 0.83 | 2.33E-01 | -0.25 | 0.88 | 1.73E-02 | -0.51 | 1.01 | 1.97E-01 | -0.27 | 0.65 | 1769875 | 46512 | 1748393 | 56501 | 1755152 | 54783 | 1741634 | 57926 |
| RP_NEG_529_46478a13_289 | RP | NEG | 13.29 | 529.4648 | | | | 2.88E-01 | -0.20 | 0.72 | 2.34E-01 | 0.25 | 0.76 | 7.58E-01 | -0.07 | 0.58 | 9.59E-02 | -0.32 | 0.66 | 3777434 | 1347366 | 3466350 | 1659272 | 3267714 | 1308958 | 3664987 | 1941304 |
| RP_POS_208_89061a0_518 | RP | POS | 0.52 | 208.8906 | | | | 1.58E-02 | 0.36 | 1.01 | 2.34E-01 | -0.26 | 0.94 | 1.91E-01 | 0.23 | 0.85 | 1.24E-02 | 0.49 | 1.06 | 506590 | 101162 | 572446 | 200272 | 596380 | 213080 | 548511 | 185645 |
| HILIC_NEG_119_90759a3_046 | HILIC | NEG | 3.046 | 119.9076 | | | | 4.41E-02 | -0.42 | 0.88 | 2.34E-01 | -0.23 | 0.94 | 2.15E-02 | -0.54 | 0.87 | 1.79E-01 | -0.30 | 0.65 | 722829 | 213892 | 633876 | 206813 | 658588 | 200573 | 609164 | 211992 |
| HILIC_NEG_209_0665a2_173 | HILIC | NEG | 2.173 | 209.0665 | | | | 4.60E-01 | 0.14 | 0.53 | 2.34E-01 | -0.25 | 0.98 | 9.32E-01 | 0.02 | 0.32 | 2.31E-01 | 0.26 | 0.50 | 4633117 | 1649047 | 4890716 | 1875834 | 5114819 | 1926498 | 4666613 | 1815333 |
| RP_POS_235_99799a1_464 | RP | POS | 1.46 | 235.9980 | | | | 4.99E-11 | 0.90 | 1.47 | 2.34E-01 | 0.25 | 0.73 | 1.96E-07 | 1.02 | 1.68 | 1.56E-06 | 0.77 | 1.48 | 964210 | 904133 | 3040685 | 2405948 | 2753297 | 2133914 | 3328073 | 2640675 |
| HILIC_POS_144_10202a5_119 | HILIC | POS | 5.119 | 144.1020 | | | | 3.15E-01 | 0.12 | 0.48 | 2.35E-01 | 0.28 | 1.28 | 2.71E-01 | 0.26 | 0.60 | 4.22E-01 | -0.02 | 0.61 | 1150308 | 1434814 | 2623958 | 14364719 | 899845 | 1261447 | 4348070 | 20229773 |
| RP_NEG_189_93207a15_38 | RP | NEG | 15.38 | 189.9321 | | | | 1.97E-01 | 0.28 | 1.16 | 2.35E-01 | -0.23 | 0.71 | 4.87E-01 | 0.16 | 0.84 | 9.88E-02 | 0.39 | 0.88 | 2261367 | 789015 | 2466396 | 715208 | 2551721 | 726706 | 2381071 | 700374 |
| RP_POS_525_28772a5_108 | RP | POS | 5.11 | 525.2877 | | | | 4.05E-02 | 0.36 | 1.20 | 2.35E-01 | -0.25 | 1.28 | 2.05E-01 | 0.23 | 1.13 | 3.08E-02 | 0.48 | 1.22 | 620076 | 1790628 | 1461522 | 2499683 | 1759917 | 2904052 | 1163128 | 2002277 |
| RP_POS_548_50372a11_253 | RP | POS | 11.25 | 548.5037 | | | | 4.75E-01 | 0.13 | 0.86 | 2.35E-01 | 0.25 | 1.17 | 2.37E-01 | 0.25 | 0.73 | 9.98E-01 | 0.00 | 0.75 | 1395007 | 439186 | 1464584 | 580757 | 1395311 | 543396 | 1533857 | 613489 |
| RP_POS_413_32349a10_944 | RP | POS | 10.94 | 413.3235 | | | | 3.17E-01 | -0.22 | 0.62 | 2.35E-01 | 0.23 | 0.76 | 6.74E-01 | -0.10 | 0.30 | 1.51E-01 | -0.33 | 0.82 | 27893608 | 5250725 | 26843323 | 4649969 | 26288774 | 4186462 | 27397872 | 5052883 |
| RP_POS_336_93079a15_423 | RP | POS | 15.42 | 336.9308 | | | | 1.60E-02 | -0.53 | 1.03 | 2.35E-01 | -0.22 | 0.87 | 5.93E-03 | -0.64 | 1.18 | 8.56E-02 | -0.42 | 0.86 | 4926836 | 1511044 | 4182687 | 1319453 | 4339986 | 1442538 | 4025388 | 1177299 |
| RP_POS_431_38806a13_125 | RP | POS | 13.13 | 431.3881 | | | | 1.55E-01 | -0.28 | 1.05 | 2.36E-01 | -0.24 | 0.84 | 8.86E-02 | -0.40 | 0.98 | 4.39E-01 | -0.16 | 0.68 | 2816481 | 628884 | 2628350 | 681117 | 2709498 | 569033 | 2547203 | 774643 |
| HILIC_NEG_289_70312a1_362 | HILIC | NEG | 1.362 | 289.7031 | | | | 1.66E-10 | 0.87 | 1.64 | 2.36E-01 | -0.25 | 0.68 | 9.84E-05 | 0.75 | 1.37 | 1.55E-09 | 0.99 | 2.18 | 462031 | 66844 | 611227 | 179047 | 632549 | 153065 | 589906 | 201031 |
| RP_POS_404_15005a7_727 | RP | POS | 7.73 | 404.1501 | | | | 9.57E-01 | 0.01 | 0.24 | 2.36E-01 | -0.25 | 0.81 | 6.12E-01 | -0.12 | 0.26 | 4.20E-01 | 0.14 | 0.61 | 782288 | 52781 | 782936 | 73690 | 791722 | 49100 | 774150 | 91667 |
| HILIC_POS_256_17532a0_615 | HILIC | POS | 0.615 | 256.1753 | | | | 2.30E-01 | 0.21 | 1.09 | 2.36E-01 | -0.25 | 0.95 | 6.53E-01 | 0.08 | 1.11 | 1.40E-01 | 0.33 | 1.17 | 611647 | 718336 | 809020 | 1021791 | 930748 | 1217919 | 687291 | 771916 |
| HILIC_NEG_136_89168a0_709 | HILIC | NEG | 0.709 | 136.8917 | | | | 2.51E-01 | 0.23 | 0.73 | 2.36E-01 | -0.24 | 0.75 | 6.24E-01 | 0.11 | 0.62 | 1.16E-01 | 0.35 | 0.62 | 4706761 | 1423117 | 5045782 | 1481347 | 5221966 | 1435539 | 4869599 | 1519684 |
| RP_NEG_787_10193a9_437 | RP | NEG | 9.44 | 787.1019 | | | | 2.08E-01 | -0.29 | 0.63 | 2.36E-01 | -0.22 | 0.72 | 1.05E-01 | -0.40 | 0.77 | 4.79E-01 | -0.17 | 0.37 | 4110549 | 290490 | 4038335 | 237810 | 4066615 | 241560 | 4010056 | 232976 |
| RP_NEG_199_03769a0_611 | RP | NEG | 0.61 | 199.0377 | | | | 2.78E-01 | 0.22 | 0.40 | 2.36E-01 | -0.24 | 0.78 | 6.66E-01 | 0.09 | 0.28 | 1.37E-01 | 0.34 | 0.68 | 6254940 | 2077733 | 6725464 | 2220463 | 6989471 | 2280126 | 6461456 | 2149491 |
| RP_NEG_246_90561a8_372 | RP | NEG | 8.37 | 246.9056 | | | | 5.31E-02 | -0.42 | 1.18 | 2.37E-01 | -0.23 | 0.74 | 1.90E-02 | -0.54 | 1.18 | 2.12E-01 | -0.31 | 0.52 | 1623331 | 908436 | 1266622 | 809679 | 1362895 | 917339 | 1170348 | 681259 |
| HILIC_POS_394_31537a7_665 | HILIC | POS | 7.665 | 394.3154 | | | | 9.25E-01 | -0.02 | 0.38 | 2.37E-01 | -0.25 | 0.71 | 4.95E-01 | -0.14 | 0.50 | 5.77E-01 | 0.11 | 0.79 | 2484869 | 297311 | 2478600 | 412169 | 2527581 | 390105 | 2429618 | 431458 |
| RP_NEG_1002_76892a0_493 | RP | NEG | 0.49 | 1002.7689 | | | | 3.58E-10 | -0.81 | 1.43 | 2.37E-01 | 0.25 | 1.23 | 7.85E-05 | -0.68 | 1.41 | 1.70E-07 | -0.93 | 1.63 | 1171355 | 131594 | 812798 | 473430 | 756555 | 468166 | 869041 | 476666 |
| RP_POS_316_11783a7_526 | RP | POS | 7.53 | 316.1178 | | | | 9.54E-05 | 0.47 | 0.92 | 2.37E-01 | -0.27 | 0.71 | 1.43E-02 | 0.34 | 0.86 | 1.89E-03 | 0.61 | 1.02 | 155300 | 137585 | 507668 | 836374 | 607062 | 960598 | 408274 | 685863 |
| RP_NEG_608_31793a10_528 | RP | NEG | 10.53 | 608.3179 | | | | 6.34E-02 | -0.46 | 0.80 | 2.37E-01 | -0.21 | 0.85 | 3.62E-02 | -0.56 | 0.89 | 1.62E-01 | -0.35 | 0.76 | 11571255 | 1007433 | 11206309 | 697214 | 11289128 | 619038 | 11123490 | 764807 |
| RP_POS_187_12619a6_499 | RP | POS | 6.50 | 187.1262 | | | | 1.07E-01 | 0.34 | 0.67 | 2.37E-01 | -0.23 | 0.76 | 3.41E-01 | 0.22 | 0.51 | 4.82E-02 | 0.45 | 0.84 | 39201571 | 21071918 | 46182603 | 20446164 | 48609359 | 19925511 | 43755847 | 20870508 |
| RP_NEG_134_86436a15_504 | RP | NEG | 15.50 | 134.8644 | | | | 4.59E-01 | 0.14 | 0.85 | 2.37E-01 | -0.25 | 0.75 | 9.53E-01 | 0.01 | 0.54 | 2.35E-01 | 0.26 | 0.55 | 1504422 | 60991 | 1514211 | 75147 | 1523130 | 80070 | 1505292 | 69540 |
| HILIC_NEG_343_99524a7_355 | HILIC | NEG | 7.355 | 343.9952 | | | | 1.79E-01 | 0.20 | 0.43 | 2.37E-01 | 0.26 | 0.71 | 1.40E-01 | 0.33 | 0.47 | 6.28E-01 | 0.07 | 0.34 | 1084930 | 73824 | 1111948 | 151391 | 1093946 | 93278 | 1129951 | 192209 |
| RP_POS_569_43201a15_102 | RP | POS | 15.10 | 569.4320 | | | | 1.04E-01 | -0.36 | 0.66 | 2.37E-01 | 0.23 | 0.70 | 3.12E-01 | -0.25 | 0.40 | 4.66E-02 | -0.47 | 0.80 | 109564536 | 40731885 | 96260419 | 35419218 | 92058396 | 33313781 | 100462442 | 37266631 |
| RP_POS_387_16187a10_908 | RP | POS | 10.91 | 387.1619 | | | | 6.10E-02 | 0.27 | 1.39 | 2.38E-01 | 0.26 | 0.92 | 4.22E-02 | 0.40 | 1.11 | 3.97E-01 | 0.14 | 1.20 | 965468 | 413168 | 1180677 | 871682 | 1077266 | 770691 | 1284087 | 958841 |

**Supplementary table 1: Metabolomics results** (in asceding order according to p-value from progressive ALD vs non-progressive ALD comparison, with molecular features with ID_level of 1-2 first)

| | | | | | | | | all_ALD_vs_control | | | Prog_ALD_vs_non-prog_ALD | | | Prog_ALD_vs_control_ALD | | | Non-prog_ALD_vs_control | | | Healthy_control | | all_ALD | | Non-prog_ALD | | Progressive_ALD | |
|---|---|---|---|---|---|---|---|---|---|---|---|---|---|---|---|---|---|---|---|---|---|---|---|---|---|---|---|
| | | | | | | | | t-test | Cohen's | PLS-DA | t-test | Cohen's | PLS-DA | t-test | Cohen's | PLS-DA | t-test | Cohen's | PLS-DA | | | | | | | | |
| Feature_ID | RP_HILIC | POS_NEG | Average.Rt | Average.Mz | Metabolite.name | curated_MSMS | ID_level | p | d | VIP | p | d | VIP | p | d | VIP | p | d | VIP | Mean | SD | Mean | SD | Mean | SD | Mean | SD |
| RP_POS_230_8889a15_43 | RP | POS | 15.43 | 230.8889 | | | | 6.31E-01 | -0.07 | 1.05 | 2.38E-01 | 0.26 | 0.75 | 6.96E-01 | 0.06 | 0.69 | 3.50E-01 | -0.20 | 0.93 | 3342703 | 229732 | 3313778 | 441751 | 3261308 | 539611 | 3366248 | 312204 |
| RP_POS_614_48492a10_659 | RP | POS | 10.66 | 614.4849 | | | | 8.18E-01 | 0.05 | 0.60 | 2.38E-01 | 0.23 | 1.02 | 4.93E-01 | 0.17 | 0.82 | 7.57E-01 | -0.07 | 0.54 | 6627647 | 502882 | 6650919 | 476252 | 6594444 | 421299 | 6707394 | 523697 |
| RP_POS_189_12163a6_499 | RP | POS | 6.50 | 189.1216 | | | | 1.10E-01 | 0.33 | 0.66 | 2.38E-01 | -0.23 | 0.76 | 3.50E-01 | 0.21 | 0.51 | 4.99E-02 | 0.45 | 0.83 | 1697232 | 938163 | 2005418 | 916934 | 2114116 | 894338 | 1896720 | 935275 |
| HILIC_POS_244_12756a0_571 | HILIC | POS | 0.571 | 244.1276 | | | | 7.36E-01 | 0.04 | 0.19 | 2.38E-01 | -0.27 | 0.75 | 3.13E-01 | -0.09 | 0.44 | 4.37E-01 | 0.18 | 0.31 | 4565576 | 2711789 | 4876457 | 7844702 | 5810524 | 10737576 | 3942391 | 2694159 |
| RP_POS_497_39606a13_281 | RP | POS | 13.28 | 497.3961 | | | | 9.35E-01 | 0.02 | 0.72 | 2.38E-01 | 0.23 | 0.75 | 5.84E-01 | 0.13 | 0.74 | 6.67E-01 | -0.10 | 0.33 | 21185069 | 14202174 | 21414891 | 12908289 | 19885444 | 11691699 | 22944338 | 13970634 |
| RP_POS_812_55548a13_266 | RP | POS | 13.27 | 812.5555 | | | | 5.20E-02 | -0.45 | 0.95 | 2.38E-01 | -0.22 | 0.88 | 2.28E-02 | -0.56 | 0.98 | 1.75E-01 | -0.34 | 0.84 | 2101814 | 1203311 | 1636032 | 955679 | 1749205 | 1017735 | 1522859 | 885076 |
| HILIC_POS_102_1282a0_828 | HILIC | POS | 0.828 | 102.1282 | | | | 5.24E-01 | -0.13 | 0.82 | 2.39E-01 | 0.24 | 0.67 | 9.73E-01 | -0.01 | 0.32 | 2.54E-01 | -0.25 | 0.43 | 3530965 | 922851 | 3409239 | 966931 | 3294892 | 879508 | 3523586 | 1043443 |
| HILIC_POS_162_11266a1_873 | HILIC | POS | 1.873 | 162.1127 | | | | 2.10E-01 | 0.16 | 0.55 | 2.39E-01 | 0.27 | 1.16 | 2.00E-01 | 0.29 | 0.72 | 7.76E-01 | 0.02 | 0.16 | 47294 | 127707 | 116276 | 498443 | 57100 | 182720 | 175451 | 679285 |
| HILIC_POS_173_12842a0_629 | HILIC | POS | 0.629 | 173.1284 | | | | 2.95E-04 | 0.50 | 0.99 | 2.39E-01 | 0.26 | 0.82 | 1.58E-03 | 0.63 | 1.02 | 1.70E-02 | 0.37 | 1.01 | 241498 | 162652 | 425118 | 400129 | 377826 | 338528 | 472410 | 452005 |
| RP_POS_531_40851a12_461 | RP | POS | 12.46 | 531.4085 | Unknown | 531.3886(70), 105.0006(100), 57.0705(28), 71.0863(20) | 4 | 1.26E-01 | 0.23 | 0.43 | 2.39E-01 | 0.26 | 0.82 | 7.83E-02 | 0.36 | 0.60 | 5.60E-01 | 0.10 | 0.34 | 2753075 | 281674 | 2869264 | 564292 | 2802575 | 483584 | 2935953 | 632788 |
| HILIC_POS_106_08674a1_87 | HILIC | POS | 1.87 | 106.0867 | | | | 2.67E-01 | 0.22 | 0.62 | 2.39E-01 | 0.24 | 0.91 | 1.58E-01 | 0.34 | 0.52 | 6.24E-01 | 0.10 | 0.19 | 4232850 | 1690255 | 4624173 | 1810383 | 4410142 | 1412517 | 4838205 | 2129124 |
| RP_NEG_556_31799a10_621 | RP | NEG | 10.62 | 556.3180 | | | | 1.82E-02 | 0.54 | 1.30 | 2.39E-01 | -0.22 | 1.03 | 8.43E-02 | 0.43 | 0.97 | 6.99E-03 | 0.65 | 1.38 | 4679536 | 888650 | 5102748 | 717081 | 5187446 | 631236 | 5018050 | 791077 |
| HILIC_POS_189_12328a5_681 | HILIC | POS | 5.681 | 189.1233 | | | | 1.02E-01 | 0.29 | 0.96 | 2.40E-01 | 0.25 | 0.94 | 5.68E-02 | 0.41 | 0.66 | 3.96E-01 | 0.16 | 0.60 | 423245 | 115056 | 465673 | 155071 | 447369 | 138640 | 483976 | 169352 |
| RP_POS_324_32504a11_45 | RP | POS | 11.45 | 324.3250 | | | | 4.50E-02 | 0.33 | 0.93 | 2.40E-01 | 0.25 | 1.19 | 3.91E-02 | 0.46 | 0.92 | 2.29E-01 | 0.20 | 0.60 | 230173 | 140117 | 297436 | 218741 | 271609 | 166046 | 323263 | 260268 |
| RP_POS_570_35498a10_341 | RP | POS | 10.34 | 570.3550 | | | | 6.85E-06 | 0.82 | 1.49 | 2.40E-01 | -0.23 | 0.85 | 8.76E-04 | 0.70 | 1.27 | 3.39E-06 | 0.93 | 1.66 | 1110526 | 541401 | 1689533 | 701288 | 1772238 | 639231 | 1606827 | 755692 |
| RP_NEG_332_87173a15_437 | RP | NEG | 15.44 | 332.8717 | | | | 7.20E-01 | 0.07 | 0.65 | 2.41E-01 | -0.25 | 0.95 | 7.95E-01 | -0.06 | 0.24 | 3.72E-01 | 0.19 | 0.71 | 2576481 | 263833 | 2596740 | 315661 | 2633941 | 310223 | 2559539 | 319773 |
| RP_NEG_583_25708a7_16 | RP | NEG | 7.16 | 583.2571 | | | | 3.16E-01 | -0.18 | 0.87 | 2.41E-01 | 0.25 | 1.15 | 7.92E-01 | -0.06 | 0.42 | 9.93E-02 | -0.31 | 1.10 | 2211856 | 1146126 | 1962320 | 1408621 | 1796239 | 1014288 | 2128402 | 1709927 |
| RP_POS_299_25781a10_663 | RP | POS | 10.66 | 299.2578 | | | | 4.37E-01 | -0.14 | 0.80 | 2.41E-01 | 0.25 | 0.93 | 9.52E-01 | -0.01 | 0.71 | 1.93E-01 | -0.26 | 0.77 | 3698131 | 494219 | 3612462 | 660709 | 3534684 | 627318 | 3690241 | 690007 |
| HILIC_POS_226_95102a5_461 | HILIC | POS | 5.461 | 226.9510 | | | | 5.08E-01 | -0.12 | 0.35 | 2.41E-01 | 0.25 | 0.92 | 9.93E-01 | 0.00 | 0.37 | 1.95E-01 | -0.24 | 0.64 | 1682050 | 228486 | 1649186 | 283753 | 1615759 | 215645 | 1682613 | 337479 |
| RP_NEG_1060_7196a0_497 | RP | NEG | 0.50 | 1060.7196 | | | | 8.50E-01 | -0.04 | 0.85 | 2.42E-01 | -0.24 | 1.16 | 4.79E-01 | -0.16 | 0.49 | 7.23E-01 | 0.08 | 0.86 | 1285010 | 202310 | 1277123 | 209634 | 1301776 | 217141 | 1252471 | 200996 |
| RP_POS_584_47095a10_875 | RP | POS | 10.88 | 584.4710 | | | | 1.54E-02 | -0.43 | 0.77 | 2.42E-01 | 0.24 | 0.76 | 1.06E-01 | -0.31 | 0.77 | 1.14E-02 | -0.56 | 0.83 | 5143547 | 522669 | 4858227 | 681840 | 4778027 | 754857 | 4938426 | 596962 |
| RP_POS_255_23192a10_462 | RP | POS | 10.46 | 255.2319 | | | | 7.08E-09 | 0.74 | 1.36 | 2.42E-01 | -0.25 | 0.97 | 1.60E-05 | 0.61 | 1.47 | 1.82E-05 | 0.87 | 1.55 | 821794 | 153262 | 1216317 | 575345 | 1284017 | 671322 | 1148616 | 456916 |
| RP_POS_695_50903a11_854 | RP | POS | 11.85 | 695.5090 | | | | 1.68E-05 | -0.95 | 1.71 | 2.42E-01 | -0.21 | 0.70 | 1.03E-05 | -1.06 | 1.71 | 2.20E-04 | -0.85 | 1.46 | 1498814 | 511495 | 1014937 | 450593 | 1067913 | 435104 | 961960 | 463862 |
| RP_POS_213_15973a5_858 | RP | POS | 5.86 | 213.1597 | | | | 7.61E-01 | -0.09 | 1.25 | 2.42E-01 | 0.18 | 0.88 | 9.82E-01 | 0.01 | 0.66 | 5.43E-01 | -0.18 | 1.08 | 656116 | 717742 | 615702 | 369705 | 572237 | 357371 | 659166 | 380241 |
| RP_POS_592_33716a10_446 | RP | POS | 10.45 | 592.3372 | | | | 3.37E-03 | 0.56 | 1.22 | 2.42E-01 | -0.24 | 0.93 | 4.64E-02 | 0.44 | 0.93 | 1.17E-03 | 0.68 | 1.35 | 2131693 | 1324395 | 2988419 | 1548170 | 3170342 | 1400211 | 2806497 | 1677630 |
| HILIC_NEG_136_89163a5_138 | HILIC | NEG | 5.138 | 136.8916 | | | | 2.51E-01 | -0.25 | 0.64 | 2.42E-01 | 0.23 | 1.01 | 5.57E-01 | -0.14 | 0.25 | 1.32E-01 | -0.37 | 0.71 | 6820133 | 1905880 | 6382606 | 1674662 | 6185903 | 1719925 | 6579309 | 1621456 |
| RP_POS_166_98492a15_486 | RP | POS | 15.49 | 166.9849 | | | | 6.38E-01 | -0.10 | 0.84 | 2.42E-01 | 0.24 | 0.76 | 9.27E-01 | 0.02 | 0.57 | 2.85E-01 | -0.21 | 0.83 | 54805382 | 10565094 | 53787075 | 10729990 | 52524405 | 6945898 | 55049745 | 13457926 |
| RP_POS_211_13052a8_135 | RP | POS | 8.14 | 211.1305 | | | | 4.31E-03 | 0.46 | 1.44 | 2.42E-01 | 0.25 | 1.42 | 3.11E-03 | 0.58 | 1.33 | 7.79E-02 | 0.33 | 1.20 | 992293 | 576043 | 1399915 | 947328 | 1288680 | 926612 | 1511151 | 964010 |
| HILIC_NEG_119_90741a2_939 | HILIC | NEG | 2.939 | 119.9074 | | | | 1.30E-01 | 0.29 | 0.90 | 2.42E-01 | -0.24 | 0.89 | 4.25E-01 | 0.16 | 0.38 | 6.85E-02 | 0.41 | 0.67 | 149681 | 33158 | 160477 | 38984 | 165055 | 41762 | 155900 | 35829 |
| RP_POS_279_9552a15_411 | RP | POS | 15.41 | 279.9552 | | | | 7.57E-01 | 0.07 | 0.83 | 2.43E-01 | -0.22 | 0.78 | 8.79E-01 | -0.04 | 0.79 | 4.48E-01 | 0.18 | 0.58 | 3510713 | 952593 | 3568343 | 760765 | 3657645 | 654153 | 3479040 | 851556 |
| HILIC_POS_154_15903a5_179 | HILIC | POS | 5.179 | 154.1590 | | | | 4.20E-01 | 0.17 | 0.69 | 2.43E-01 | -0.23 | 1.02 | 8.35E-01 | 0.05 | 0.20 | 2.13E-01 | 0.28 | 0.51 | 14756448 | 3223609 | 15288488 | 3221632 | 15666438 | 3146561 | 14910538 | 3282877 |
| RP_POS_359_17429a11_252 | RP | POS | 11.25 | 359.1743 | | | | 2.59E-05 | 0.66 | 1.51 | 2.43E-01 | 0.25 | 0.96 | 1.83E-04 | 0.78 | 1.48 | 1.53E-03 | 0.54 | 1.51 | 229756 | 154564 | 400174 | 271329 | 368361 | 227572 | 431987 | 308038 |
| RP_POS_548_50415a11_16 | RP | POS | 11.16 | 548.5042 | | | | 9.72E-02 | 0.26 | 1.06 | 2.43E-01 | 0.26 | 1.06 | 4.94E-02 | 0.38 | 1.10 | 4.85E-01 | 0.13 | 0.94 | 1650089 | 260515 | 1760205 | 469251 | 1705221 | 450254 | 1815188 | 485787 |
| HILIC_POS_520_33252a0_618 | HILIC | POS | 0.618 | 520.3325 | | | | 5.08E-02 | 0.34 | 1.21 | 2.43E-01 | -0.25 | 1.00 | 2.47E-01 | 0.21 | 1.17 | 3.61E-02 | 0.46 | 1.36 | 899830 | 2215870 | 1896573 | 3140808 | 2264755 | 3582004 | 1528390 | 2612138 |
| RP_POS_568_42694a11_645 | RP | POS | 11.65 | 568.4269 | | | | 8.17E-03 | -0.77 | 1.39 | 2.43E-01 | -0.16 | 0.80 | 4.61E-03 | -0.85 | 1.28 | 1.95E-02 | -0.69 | 1.19 | 619339 | 590944 | 316874 | 270186 | 348520 | 269916 | 285228 | 269430 |
| RP_POS_347_17166a7_093 | RP | POS | 7.09 | 347.1717 | | | | 3.90E-01 | -0.16 | 0.64 | 2.44E-01 | 0.24 | 0.78 | 8.65E-01 | -0.04 | 0.32 | 1.85E-01 | -0.28 | 0.66 | 1388644 | 79748 | 1373765 | 98274 | 1362261 | 97565 | 1385268 | 98609 |
| RP_NEG_180_89876a15_544 | RP | NEG | 15.54 | 180.8988 | | | | 2.35E-01 | -0.19 | 0.66 | 2.44E-01 | 0.25 | 0.70 | 6.46E-01 | -0.07 | 0.74 | 1.37E-01 | -0.32 | 0.63 | 5076127 | 540448 | 4923644 | 843958 | 4824789 | 972364 | 5022498 | 688209 |
| RP_POS_177_95604a0_25 | RP | POS | 0.25 | 177.9560 | | | | 1.51E-01 | 0.29 | 1.09 | 2.44E-01 | 0.24 | 0.71 | 7.37E-02 | 0.40 | 1.05 | 4.43E-01 | 0.17 | 0.61 | 9492543 | 2836461 | 10343347 | 2990250 | 9993419 | 2920593 | 10693275 | 3047279 |
| RP_POS_335_22052a9_056 | RP | POS | 9.06 | 335.2205 | | | | 1.80E-01 | 0.27 | 0.57 | 2.44E-01 | -0.23 | 0.74 | 4.82E-01 | 0.16 | 0.53 | 9.01E-02 | 0.39 | 0.69 | 1627377 | 482641 | 1761115 | 488488 | 1818277 | 502594 | 1703952 | 472061 |
| RP_POS_233_05968a0_734 | RP | POS | 0.73 | 233.0597 | | | | 8.71E-11 | 0.83 | 1.37 | 2.44E-01 | 0.25 | 0.73 | 1.61E-06 | 0.95 | 1.57 | 1.94E-06 | 0.71 | 1.45 | 84382 | 118540 | 434092 | 447482 | 381713 | 372585 | 486470 | 510047 |
| HILIC_POS_154_15926a5_334 | HILIC | POS | 5.334 | 154.1593 | | | | 3.64E-01 | 0.18 | 0.61 | 2.45E-01 | -0.23 | 0.79 | 7.63E-01 | 0.07 | 0.31 | 1.89E-01 | 0.30 | 0.56 | 21062936 | 7844474 | 22524826 | 7942996 | 23453106 | 8144960 | 21596546 | 7705259 |
| RP_POS_323_198a10_122 | RP | POS | 10.12 | 323.1980 | | | | 3.18E-01 | 0.14 | 0.45 | 2.45E-01 | -0.26 | 0.90 | 9.23E-01 | 0.01 | 0.26 | 1.98E-01 | 0.27 | 0.62 | 319825 | 452656 | 446074 | 972486 | 559850 | 1175206 | 332299 | 709388 |
| RP_POS_348_28726a10_432 | RP | POS | 10.43 | 348.2873 | | | | 4.15E-01 | -0.23 | 0.88 | 2.45E-01 | -0.17 | 0.88 | 2.69E-01 | -0.32 | 0.77 | 6.28E-01 | -0.15 | 0.52 | 2135880 | 4157794 | 1507960 | 2005692 | 1742492 | 2343414 | 1273428 | 1588664 |
| HILIC_NEG_261_81305a3_028 | HILIC | NEG | 3.028 | 261.8131 | | | | 1.77E-02 | -0.45 | 0.98 | 2.45E-01 | 0.24 | 0.82 | 1.19E-01 | -0.34 | 0.68 | 8.21E-03 | -0.57 | 0.97 | 537838 | 51177 | 511518 | 58772 | 504655 | 57943 | 518381 | 59375 |
| HILIC_POS_476_30682a0_616 | HILIC | POS | 0.616 | 476.3068 | | | | 5.84E-01 | 0.13 | 1.13 | 2.45E-01 | -0.22 | 0.98 | 9.31E-01 | 0.02 | 0.93 | 3.69E-01 | 0.24 | 1.18 | 1800604 | 4919927 | 2323468 | 3755880 | 2762236 | 4320997 | 1884700 | 3072011 |
| HILIC_POS_64_01666a5_049 | HILIC | POS | 5.049 | 64.0167 | | | | 6.95E-01 | 0.07 | 0.56 | 2.45E-01 | 0.24 | 0.95 | 4.00E-01 | 0.19 | 0.48 | 8.03E-01 | -0.05 | 0.11 | 9393052 | 5165125 | 9826844 | 6185534 | 9104451 | 4956944 | 10549236 | 7187919 |
| HILIC_POS_680_48022a10_693 | HILIC | POS | 10.693 | 680.4802 | | | | 2.51E-01 | -0.16 | 0.36 | 2.46E-01 | -0.26 | 0.69 | 1.88E-01 | -0.29 | 0.52 | 7.93E-01 | -0.03 | 0.18 | 2780146 | 66012 | 2758375 | 148248 | 2775706 | 85940 | 2741045 | 190801 |
| RP_NEG_329_24902a11_13 | RP | NEG | 11.13 | 329.2490 | | | | 1.08E-05 | 0.68 | 1.68 | 2.46E-01 | 0.25 | 1.16 | 8.62E-05 | 0.80 | 1.60 | 1.14E-03 | 0.56 | 1.54 | 3168587 | 1906996 | 5409258 | 3452716 | 5006567 | 3022042 | 5811949 | 3823887 |
| HILIC_POS_170_09235a6_886 | HILIC | POS | 6.886 | 170.0924 | | | | 9.54E-01 | -0.01 | 0.22 | 2.46E-01 | -0.23 | 0.67 | 5.85E-01 | -0.13 | 0.30 | 6.79E-01 | 0.10 | 0.23 | 16668274 | 22091074 | 16415593 | 19778756 | 18722001 | 21379442 | 14109184 | 17956762 |
| RP_POS_392_2576a12_908 | RP | POS | 12.91 | 392.2576 | | | | 8.31E-06 | -0.96 | 1.65 | 2.46E-01 | -0.21 | 0.80 | 9.66E-06 | -1.07 | 1.61 | 8.72E-05 | -0.86 | 1.62 | 852593 | 278401 | 576063 | 258016 | 606138 | 219407 | 545987 | 290719 |
| HILIC_NEG_136_89165a6_093 | HILIC | NEG | 6.093 | 136.8917 | | | | 3.77E-01 | -0.18 | 0.84 | 2.46E-01 | -0.23 | 0.93 | 1.81E-01 | -0.30 | 0.69 | 7.89E-01 | -0.06 | 0.94 | 4761238 | 2142099 | 4370906 | 2197159 | 4626916 | 2311587 | 4114896 | 2067933 |
| HILIC_POS_645_09351a0_569 | HILIC | POS | 0.569 | 645.0935 | | | | 2.55E-06 | 0.60 | 1.29 | 2.46E-01 | -0.26 | 0.95 | 9.13E-04 | 0.48 | 1.45 | 2.58E-04 | 0.73 | 1.45 | 54162 | 214163 | 474074 | 764197 | 563138 | 884338 | 385010 | 617794 |
| RP_POS_464_31369a10_799 | RP | POS | 10.80 | 464.3137 | | | | 4.14E-02 | 0.33 | 0.61 | 2.46E-01 | 0.25 | 0.90 | 1.27E-02 | 0.45 | 0.82 | 3.26E-01 | 0.20 | 0.44 | 1152578 | 397653 | 1351698 | 656989 | 1275164 | 723067 | 1428233 | 580774 |
| HILIC_POS_184_07335a0_604 | HILIC | POS | 0.604 | 184.0734 | | | | 1.91E-02 | 0.51 | 1.05 | 2.47E-01 | 0.22 | 0.83 | 9.04E-03 | 0.62 | 1.11 | 9.13E-02 | 0.39 | 1.09 | 759716 | 107450 | 811565 | 98253 | 800137 | 98646 | 822993 | 97502 |
| RP_NEG_201_8889a14_27 | RP | NEG | 14.27 | 201.8889 | | | | 4.59E-01 | 0.14 | 0.35 | 2.47E-01 | -0.24 | 0.93 | 9.40E-01 | 0.02 | 0.42 | 1.31E-01 | 0.26 | 0.69 | 1208422 | 337452 | 1261784 | 395162 | 1307921 | 176120 | 1215647 | 529274 |
| RP_POS_195_10159a7_699 | RP | POS | 7.70 | 195.1016 | | | | 4.59E-01 | -0.12 | 0.49 | 2.47E-01 | 0.26 | 0.74 | 9.56E-01 | 0.01 | 0.32 | 1.10E-01 | -0.24 | 0.64 | 2162989 | 63306 | 2151212 | 111868 | 2138177 | 74066 | 2164248 | 139469 |
| RP_POS_185_12843a2_82 | RP | POS | 2.82 | 185.1284 | | | | 1.26E-02 | 0.43 | 0.74 | 2.47E-01 | 0.24 | 0.69 | 1.57E-02 | 0.56 | 0.98 | 7.08E-02 | 0.31 | 0.98 | 3388066 | 1530559 | 4269609 | 2136652 | 4020735 | 1507352 | 4518482 | 2612511 |
| RP_NEG_393_80124a15_618 | RP | NEG | 15.62 | 393.8012 | | | | 5.87E-02 | -0.41 | 0.85 | 2.47E-01 | -0.22 | 0.69 | 2.93E-02 | -0.52 | 0.89 | 2.01E-01 | -0.30 | 0.70 | 850727 | 42997 | 834216 | 38609 | 838706 | 37820 | 829726 | 39247 |
| RP_POS_874_5542a13_404 | RP | POS | 13.40 | 874.5542 | | | | 8.70E-01 | 0.03 | 0.51 | 2.47E-01 | -0.23 | 0.83 | 7.17E-01 | -0.08 | 0.34 | 4.95E-01 | 0.15 | 0.78 | 6250556 | 1203586 | 6290870 | 1230012 | 6433917 | 1140159 | 6147824 | 1309585 |
| RP_POS_286_8829a15_427 | RP | POS | 15.43 | 286.8829 | | | | 1.86E-02 | -0.32 | 0.60 | 2.47E-01 | 0.26 | 1.15 | 6.75E-02 | -0.19 | 0.71 | 5.03E-02 | -0.45 | 0.74 | 5298615 | 140652 | 5188490 | 389470 | 5143043 | 521893 | 5233936 | 172866 |
| RP_POS_963_57471a10_834 | RP | POS | 10.83 | 963.5747 | | | | 9.37E-06 | 0.53 | 1.08 | 2.47E-01 | -0.26 | 1.04 | 2.08E-03 | 0.40 | 1.16 | 9.48E-04 | 0.66 | 1.23 | 459776 | 60797 | 696695 | 497745 | 754597 | 588852 | 638794 | 383363 |
| RP_POS_429_31921a10_54 | RP | POS | 10.54 | 429.3192 | | | | 7.95E-01 | -0.05 | 0.58 | 2.47E-01 | 0.23 | 0.77 | 7.61E-01 | 0.07 | 0.37 | 4.73E-01 | -0.17 | 0.66 | 12876368 | 1614987 | 12790137 | 1662930 | 12596832 | 1856941 | 12983441 | 1436183 |
| RP_POS_441_29739a12_619 | RP | POS | 12.62 | 441.2974 | | | | 4.44E-02 | 0.36 | 0.88 | 2.47E-01 | -0.24 | 0.74 | 2.16E-01 | 0.23 | 0.73 | 3.04E-02 | 0.48 | 0.82 | 15990137 | 6331633 | 18861339 | 8442679 | 19842454 | 9488101 | 17880224 | 7212752 |
| HILIC_POS_159_09143a4_124 | HILIC | POS | 4.124 | 159.0914 | | | | 2.88E-02 | -0.40 | 1.05 | 2.47E-01 | -0.24 | 1.34 | 2.07E-02 | -0.52 | 0.97 | 1.46E-01 | -0.28 | 0.82 | 2846778 | 679094 | 2519308 | 833810 | 2616718 | 709229 | 2421899 | 947364 |
| RP_POS_499_82715a5_878 | RP | POS | 5.88 | 499.8272 | | | | 3.22E-01 | 0.17 | 0.57 | 2.48E-01 | 0.25 | 0.70 | 2.27E-01 | 0.29 | 0.61 | 7.58E-01 | 0.05 | 0.42 | 1322169 | 2225985 | 1823072 | 3126841 | 1458698 | 1397114 | 2187446 | 4186993 |
| RP_POS_259_19043a8_028 | RP | POS | 8.03 | 259.1904 | | | | 1.24E-01 | 0.32 | 0.79 | 2.48E-01 | 0.23 | 0.77 | 6.16E-02 | 0.44 | 0.78 | 3.65E-01 | 0.21 | 0.70 | 1947856 | 320051 | 2048673 | 309877 | 2012715 | 304823 | 2084631 | 313769 |
| RP_POS_402_77362a12_817 | RP | POS | 12.82 | 402.7736 | | | | 8.81E-06 | -1.10 | 1.80 | 2.48E-01 | -0.18 | 1.08 | 3.39E-06 | -1.19 | 1.86 | 7.66E-05 | -1.00 | 1.56 | 1093558 | 512382 | 599624 | 356596 | 641005 | 382581 | 558243 | 327190 |
| RP_POS_617_26111a11_803 | RP | POS | 11.80 | 617.2611 | | | | 1.14E-02 | -0.52 | 1.07 | 2.48E-01 | 0.23 | 1.26 | 9.77E-02 | -0.41 | 0.68 | 2.34E-03 | -0.63 | 1.31 | 416713 | 239823 | 288943 | 240611 | 260987 | 167677 | 316898 | 295396 |
| RP_NEG_364_86996a0_5 | RP | NEG | 0.50 | 364.8700 | | | | 7.83E-01 | 0.04 | 0.97 | 2.49E-01 | -0.26 | 1.43 | 6.41E-01 | -0.09 | 0.69 | 3.77E-01 | 0.17 | 1.00 | 872163 | 201296 | 886514 | 377874 | 930272 | 388236 | 842755 | 365865 |
| HILIC_POS_645_4281a0_569 | HILIC | POS | 0.569 | 645.4281 | | | | 1.87E-06 | 0.60 | 1.29 | 2.49E-01 | -0.26 | 0.94 | 8.13E-04 | 0.47 | 1.46 | 2.63E-04 | 0.73 | 1.46 | 59257 | 191538 | 484809 | 780577 | 575255 | 904770 | 394364 | 629072 |
| RP_NEG_191_86061a15_551 | RP | NEG | 15.55 | 191.8606 | | | | 5.94E-01 | -0.11 | 0.47 | 2.49E-01 | -0.23 | 1.30 | 3.19E-01 | -0.23 | 0.67 | 9.73E-01 | 0.01 | 0.29 | 972073 | 61126 | 965392 | 61780 | 972544 | 62759 | 958240 | 60567 |
| RP_POS_431_27911a10_659 | RP | POS | 10.66 | 431.2791 | | | | 5.44E-01 | 0.10 | 0.59 | 2.49E-01 | 0.25 | 0.73 | 2.83E-01 | 0.23 | 0.55 | 9.10E-01 | -0.02 | 0.69 | 1729361 | 548141 | 1803216 | 776263 | 1713372 | 722437 | 1893060 | 824063 |
| RP_POS_768_80524a0_494 | RP | POS | 0.49 | 768.8052 | | | | 2.59E-09 | -0.74 | 1.39 | 2.49E-01 | 0.25 | 1.25 | 2.11E-04 | -0.62 | 1.37 | 1.32E-06 | -0.87 | 1.57 | 2369056 | 208803 | 1690411 | 987988 | 1576128 | 1000310 | 1804694 | 972053 |
| RP_POS_312_90988a0_497 | RP | POS | 0.50 | 312.9099 | | | | 4.04E-08 | -0.67 | 1.32 | 2.49E-01 | 0.25 | 1.25 | 5.77E-04 | -0.54 | 1.31 | 1.62E-05 | -0.79 | 1.50 | 1444182 | 54277 | 1198188 | 405382 | 1151294 | 429796 | 1245082 | 377903 |
| RP_POS_613_26489a11_803 | RP | POS | 11.80 | 613.2649 | | | | 4.11E-02 | -0.40 | 1.06 | 2.49E-01 | 0.23 | 1.28 | 2.48E-01 | -0.29 | 0.63 | 6.97E-03 | -0.52 | 1.28 | 2157361 | 1335383 | 1579014 | 1443418 | 1411636 | 838306 | 1746393 | 1857283 |
| RP_POS_358_83374a0_5 | RP | POS | 0.50 | 358.8337 | | | | 7.33E-01 | 0.05 | 1.12 | 2.50E-01 | -0.26 | 1.31 | 6.54E-01 | -0.08 | 1.01 | 3.65E-01 | 0.18 | 1.23 | 480603 | 173982 | 496492 | 347364 | 536670 | 376469 | 456314 | 314265 |
| RP_POS_163_98746a15_397 | RP | POS | 15.40 | 163.9875 | | | | 7.26E-01 | 0.07 | 0.29 | 2.50E-01 | 0.23 | 0.74 | 4.17E-01 | 0.19 | 0.71 | 8.55E-01 | -0.04 | 0.47 | 55952682 | 22272184 | 57533652 | 21532317 | 55043694 | 21371335 | 60023611 | 21618432 |
| HILIC_NEG_202_92896a2_998 | HILIC | NEG | 2.998 | 202.9290 | | | | 2.03E-01 | -0.24 | 0.83 | 2.50E-01 | 0.24 | 0.95 | 5.72E-01 | -0.12 | 0.42 | 9.19E-02 | -0.36 | 0.70 | 4993066 | 1043013 | 4709302 | 1205446 | 4569934 | 1169429 | 4848670 | 1236387 |
| RP_NEG_573_12891a9_262 | RP | NEG | 9.26 | 573.1289 | | | | 2.66E-01 | -0.26 | 0.59 | 2.50E-01 | 0.21 | 0.73 | 5.41E-01 | -0.16 | 0.30 | 1.42E-01 | -0.37 | 0.64 | 5069564 | 627036 | 4933655 | 477411 | 4878481 | 455019 | 4988830 | 497232 |
| RP_NEG_765_16815a9_304 | RP | NEG | 9.30 | 765.1682 | | | | 7.04E-01 | 0.06 | 0.38 | 2.50E-01 | -0.25 | 1.05 | 7.53E-01 | -0.07 | 0.19 | 2.97E-01 | 0.19 | 0.49 | 8167541 | 451306 | 8209309 | 750344 | 8296029 | 655470 | 8122589 | 832186 |
| RP_POS_339_1824a11_247 | RP | POS | 11.25 | 339.1824 | | | | 3.80E-05 | 0.76 | 1.61 | 2.50E-01 | 0.23 | 1.12 | 7.89E-05 | 0.87 | 1.58 | 9.17E-04 | 0.64 | 1.45 | 7870370 | 1655923 | 9459414 | 2085626 | 9218404 | 1816950 | 9700425 | 2317023 |
| RP_POS_308_89911a0_252 | RP | POS | 0.25 | 308.8991 | | | | 6.80E-01 | -0.09 | 0.64 | 2.51E-01 | -0.23 | 0.86 | 3.93E-01 | -0.20 | 0.41 | 9.10E-01 | 0.03 | 0.92 | 1176439 | 233886 | 1157064 | 218460 | 1182268 | 215579 | 1131860 | 220574 |
| RP_POS_339_25064a9_607 | RP | POS | 9.61 | 339.2506 | | | | 1.18E-05 | 0.68 | 1.27 | 2.51E-01 | 0.24 | 1.24 | 1.38E-04 | 0.80 | 1.31 | 8.44E-04 | 0.55 | 1.29 | 1147313 | 149132 | 1322635 | 273291 | 1291090 | 226108 | 1354180 | 312640 |
| RP_POS_818_60303a14_119 | RP | POS | 14.12 | 818.6030 | | | | 5.59E-03 | -0.70 | 1.18 | 2.51E-01 | -0.19 | 0.75 | 2.65E-03 | -0.79 | 1.25 | 2.25E-02 | -0.60 | 0.94 | 1707885 | 969144 | 1171393 | 650790 | 1246414 | 673737 | 1096373 | 624732 |
| RP_POS_169_03589a0_71 | RP | POS | 0.71 | 169.0359 | Uric acid | 169.0353(100), 141.04(15), 126.0299(10), 152.01(3) | 3 | 6.21E-04 | 0.54 | 0.98 | 2.51E-01 | 0.25 | 0.68 | 2.23E-03 | 0.66 | 1.15 | 1.07E-02 | 0.41 | 0.92 | 69416225 | 13329244 | 81151959 | 23285333 | 78466733 | 17909985 | 83837185 | 27568027 |
| RP_POS_812_54028a11_613 | RP | POS | 11.61 | 812.5403 | | | | 7.08E-01 | 0.06 | 0.31 | 2.51E-01 | -0.25 | 0.83 | 6.53E-01 | -0.07 | 0.23 | 4.16E-01 | 0.19 | 0.46 | 5347137 | 9610014 | 6234777 | 16362270 | 8123930 | 20800797 | 4345624 | 10047633 |
| HILIC_POS_327_20132a0_616 | HILIC | POS | 0.616 | 327.2013 | | | | 7.29E-01 | -0.11 | 1.02 | 2.52E-01 | -0.16 | 0.96 | 5.49E-01 | -0.18 | 0.76 | 9.32E-01 | -0.03 | 0.94 | 331983 | 1038463 | 266042 | 425685 | 315085 | 493852 | 216998 | 342512 |
| RP_POS_360_27438a7_568 | RP | POS | 7.57 | 360.2744 | | | | 1.39E-04 | 0.62 | 1.14 | 2.52E-01 | 0.24 | 0.89 | 5.09E-04 | 0.74 | 1.22 | 4.01E-03 | 0.50 | 1.17 | 456844 | 201283 | 648879 | 324146 | 611547 | 269786 | 686210 | 369672 |

**Supplementary table 1: Metabolomics results** (in asceding order according to p-value from progressive ALD vs  non-progressive ALD comparison, with molecular features with ID_level of 1-2 first)

| | | | | | | | | all_ALD_vs_control | | | Prog_ALD_vs_non-prog_ALD | | | Prog_ALD_vs_control_ALD | | | Non-prog_ALD_vs_control | | | Healthy_control | | all_ALD | | Non-prog_ALD | | Progressive_ALD | |
|---|---|---|---|---|---|---|---|---|---|---|---|---|---|---|---|---|---|---|---|---|---|---|---|---|---|---|---|
| | | | | | | | | t-test | Cohen's | PLS-DA | t-test | Cohen's | PLS-DA | t-test | Cohen's | PLS-DA | t-test | Cohen's | PLS-DA | | | | | | | | |
| Feature_ID | RP_HILIC | POS_NEG | Average.Rt | Average.Mz | Metabolite.name | curated_MSMS | ID_level | p | d | VIP | p | d | VIP | p | d | VIP | p | d | VIP | Mean | SD | Mean | SD | Mean | SD | Mean | SD |
| RP_POS_274_7912a0_522 | RP | POS | 0.52 | 274.7912 | | | | 1.66E-05 | -0.60 | 1.20 | 2.52E-01 | 0.25 | 1.14 | 5.98E-03 | -0.47 | 1.10 | 1.05E-04 | -0.72 | 1.36 | 470994 | 59141 | 390955 | 144635 | 374320 | 148776 | 407591 | 139881 |
| HILIC_POS_344_22815a0_617 | HILIC | POS | 0.617 | 344.2282 | | | | 7.46E-01 | -0.10 | 1.02 | 2.52E-01 | -0.16 | 0.96 | 5.59E-01 | -0.18 | 0.77 | 9.55E-01 | -0.02 | 0.95 | 3783743 | 11415471 | 3105876 | 4825716 | 3661116 | 5586963 | 2550635 | 3899616 |
| RP_POS_1054_69275a10_617 | RP | POS | 10.62 | 1054.6928 | | | | 7.49E-03 | 0.57 | 1.33 | 2.52E-01 | -0.22 | 0.99 | 5.60E-02 | 0.46 | 1.12 | 2.36E-03 | 0.68 | 1.37 | 669156 | 445794 | 917473 | 414101 | 965104 | 345798 | 869842 | 471437 |
| RP_POS_792_58698a13_829 | RP | POS | 13.83 | 792.5870 | | | | 1.17E-05 | -0.99 | 1.57 | 2.52E-01 | -0.20 | 0.72 | 8.90E-06 | -1.09 | 1.63 | 1.09E-04 | -0.89 | 1.48 | 2391807 | 974215 | 1453829 | 820700 | 1548173 | 745374 | 1359486 | 887184 |
| RP_POS_273_20667a8_908 | RP | POS | 8.91 | 273.2067 | | | | 1.80E-01 | 0.28 | 0.62 | 2.53E-01 | 0.23 | 0.69 | 1.07E-01 | 0.39 | 0.72 | 4.43E-01 | 0.17 | 0.41 | 595989 | 116969 | 628132 | 114161 | 615007 | 94411 | 641256 | 130639 |
| RP_POS_415_28159a9_859 | RP | POS | 9.86 | 415.2816 | Chenodeoxycholic acid [M+Na]+ | 415.278(100) | | 4.56E-01 | 0.14 | 0.36 | 2.53E-01 | 0.24 | 0.69 | 2.43E-01 | 0.26 | 0.46 | 9.32E-01 | 0.02 | 0.67 | 1394188 | 1929712 | 1706227 | 2375270 | 1433314 | 2171062 | 1979141 | 2556060 |
| HILIC_NEG_303_68158a1_369 | HILIC | NEG | 1.369 | 303.6816 | | | | 2.98E-18 | -1.23 | 2.13 | 2.53E-01 | -0.22 | 0.94 | 3.72E-13 | -1.33 | 2.31 | 3.63E-10 | -1.12 | 2.22 | 296056 | 35483 | 188198 | 83680 | 197807 | 84574 | 178588 | 82501 |
| RP_POS_844_5213a12_959 | RP | POS | 12.96 | 844.5213 | | | | 1.74E-03 | -0.66 | 1.37 | 2.53E-01 | -0.22 | 0.77 | 9.57E-04 | -0.77 | 1.48 | 1.50E-02 | -0.55 | 1.09 | 9100067 | 3040423 | 7075190 | 2937766 | 7412519 | 2889163 | 6737861 | 2976170 |
| RP_NEG_397_23651a11_049 | RP | NEG | 11.05 | 397.2365 | | | | 2.08E-09 | 0.82 | 1.77 | 2.53E-01 | 0.24 | 0.89 | 1.82E-06 | 0.94 | 1.68 | 1.32E-05 | 0.70 | 1.72 | 436757 | 251128 | 954425 | 667765 | 877730 | 587747 | 1031121 | 737204 |
| RP_POS_386_28986a7_98 | RP | POS | 7.98 | 386.2899 | | | | 7.30E-04 | 0.55 | 1.09 | 2.53E-01 | 0.24 | 0.96 | 1.34E-03 | 0.67 | 1.18 | 1.73E-02 | 0.43 | 1.09 | 888319 | 429118 | 1249803 | 699544 | 1169471 | 617559 | 1330134 | 770818 |
| RP_POS_668_40118a11_078 | RP | POS | 11.08 | 668.4012 | | | | 2.53E-07 | 0.62 | 1.28 | 2.53E-01 | -0.25 | 1.21 | 6.17E-04 | 0.49 | 1.34 | 1.12E-04 | 0.74 | 1.39 | 43663 | 29141 | 319054 | 495464 | 375928 | 558675 | 262181 | 421023 |
| RP_POS_499_8273a2_965 | RP | POS | 2.97 | 499.8273 | | | | 5.09E-01 | -0.16 | 0.52 | 2.54E-01 | -0.21 | 0.77 | 3.00E-01 | -0.26 | 0.56 | 8.43E-01 | -0.05 | 0.50 | 2125252 | 2027399 | 1866057 | 1528716 | 2041350 | 1568879 | 1690764 | 1482413 |
| RP_POS_321_23962a10_542 | RP | POS | 10.54 | 321.2396 | | | | 1.62E-01 | -0.26 | 0.84 | 2.54E-01 | 0.24 | 0.98 | 5.13E-01 | -0.14 | 0.70 | 7.72E-02 | -0.38 | 0.84 | 5622176 | 773837 | 5385431 | 963398 | 5274966 | 970870 | 5495896 | 952744 |
| RP_POS_308_92014a15_458 | RP | POS | 15.46 | 308.9201 | | | | 5.46E-01 | 0.13 | 0.57 | 2.54E-01 | -0.22 | 0.92 | 9.38E-01 | 0.02 | 0.45 | 3.00E-01 | 0.24 | 0.88 | 3061337 | 784830 | 3156085 | 707200 | 3237165 | 670079 | 3075005 | 740303 |
| RP_POS_192_03242a0_706 | RP | POS | 0.71 | 192.0324 | | | | 5.40E-04 | 0.47 | 0.99 | 2.54E-01 | 0.25 | 0.71 | 5.55E-03 | 0.60 | 1.03 | 7.92E-03 | 0.35 | 1.11 | 538500 | 473705 | 1070813 | 1243220 | 928062 | 819440 | 1213564 | 1552311 |
| HILIC_POS_564_35913a0_619 | HILIC | POS | 0.619 | 564.3591 | | | | 2.27E-04 | 0.48 | 1.25 | 2.54E-01 | -0.25 | 1.00 | 1.78E-02 | 0.36 | 1.34 | 1.92E-03 | 0.61 | 1.46 | 561275 | 1230574 | 2235970 | 3842112 | 2676491 | 4345243 | 1795449 | 3247729 |
| RP_POS_292_63455a7_158 | RP | POS | 7.16 | 292.6346 | | | | 5.31E-01 | -0.12 | 0.86 | 2.54E-01 | 0.24 | 1.14 | 9.98E-01 | 0.00 | 0.42 | 2.31E-01 | -0.24 | 0.94 | 492376 | 215536 | 463552 | 252842 | 434557 | 203598 | 492547 | 293249 |
| RP_NEG_203_08163a2_484 | RP | NEG | 2.48 | 203.0816 | | | | 5.91E-04 | -0.72 | 1.38 | 2.54E-01 | -0.22 | 1.10 | 5.56E-04 | -0.83 | 1.39 | 4.44E-03 | -0.61 | 1.25 | 4703550 | 1164379 | 3842879 | 1132140 | 3972552 | 961974 | 3713206 | 1276686 |
| RP_POS_646_38892a11_085 | RP | POS | 11.09 | 646.3889 | | | | 1.73E-07 | 0.62 | 1.30 | 2.54E-01 | -0.25 | 1.22 | 3.91E-04 | 0.50 | 1.37 | 1.11E-04 | 0.75 | 1.40 | 54323 | 40274 | 521823 | 829077 | 616784 | 945278 | 426862 | 690517 |
| RP_POS_164_98444a13_12 | RP | POS | 13.12 | 164.9844 | | | | 7.22E-01 | -0.08 | 0.30 | 2.54E-01 | -0.23 | 0.81 | 4.24E-01 | -0.19 | 0.62 | 8.72E-01 | 0.04 | 0.26 | 13751674 | 4630987 | 13419807 | 4334875 | 13915929 | 4243333 | 12923685 | 4411074 |
| RP_POS_246_09058a0_614 | RP | POS | 0.61 | 246.0906 | | | | 1.55E-02 | 0.45 | 0.97 | 2.54E-01 | 0.23 | 0.95 | 9.07E-03 | 0.57 | 1.19 | 1.07E-01 | 0.33 | 0.76 | 244721 | 299360 | 404699 | 365250 | 362898 | 349441 | 446501 | 379292 |
| RP_POS_560_46979a14_267 | RP | POS | 14.27 | 560.4698 | | | | 6.96E-01 | -0.08 | 0.37 | 2.55E-01 | 0.23 | 1.15 | 8.69E-01 | 0.04 | 0.21 | 3.94E-01 | -0.19 | 0.46 | 31619872 | 9456632 | 30860195 | 9749966 | 29744454 | 9976136 | 31975935 | 9486826 |
| HILIC_POS_285_12802a5_592 | HILIC | POS | 5.592 | 285.1280 | | | | 2.10E-01 | 0.22 | 0.70 | 2.55E-01 | -0.24 | 1.18 | 6.58E-01 | 0.10 | 0.58 | 6.94E-02 | 0.34 | 0.71 | 199282 | 234559 | 265718 | 321758 | 302555 | 266429 | 228880 | 367989 |
| RP_POS_293_17477a8_696 | RP | POS | 8.70 | 293.1748 | | | | 2.40E-01 | -0.24 | 0.51 | 2.55E-01 | 0.23 | 0.94 | 5.83E-01 | -0.13 | 0.45 | 1.11E-01 | -0.36 | 0.66 | 1078566 | 59784 | 1064179 | 59029 | 1057425 | 54387 | 1070933 | 63158 |
| RP_POS_661_52698a11_968 | RP | POS | 11.97 | 661.5270 | | | | 3.12E-06 | -1.12 | 1.96 | 2.55E-01 | -0.18 | 0.67 | 1.11E-06 | -1.21 | 2.01 | 3.55E-05 | -1.03 | 1.63 | 1011159 | 451817 | 546621 | 331119 | 584469 | 362544 | 508774 | 295221 |
| RP_POS_463_12979a10_133 | RP | POS | 10.13 | 463.1298 | | | | 6.86E-01 | 0.09 | 0.40 | 2.55E-01 | -0.22 | 0.75 | 9.33E-01 | -0.02 | 0.36 | 4.08E-01 | 0.20 | 0.49 | 629631 | 125260 | 639638 | 107441 | 651908 | 106092 | 627368 | 108444 |
| RP_POS_743_44574a10_909 | RP | POS | 10.91 | 743.4457 | | | | 1.26E-04 | 0.50 | 1.14 | 2.56E-01 | -0.25 | 1.11 | 4.32E-03 | 0.38 | 1.12 | 2.64E-03 | 0.63 | 1.20 | 1200580 | 363521 | 1709368 | 1115308 | 1836842 | 1359533 | 1581894 | 794806 |
| RP_NEG_394_01221a2_473 | RP | NEG | 2.47 | 394.0122 | | | | 7.44E-03 | -0.45 | 1.10 | 2.56E-01 | -0.24 | 1.17 | 8.76E-03 | -0.57 | 1.17 | 6.55E-02 | -0.33 | 0.97 | 1043108 | 168181 | 935791 | 253407 | 964725 | 209132 | 906857 | 290337 |
| HILIC_POS_694_46509a0_51 | HILIC | POS | 0.51 | 694.4651 | | | | 7.98E-07 | 0.61 | 1.33 | 2.56E-01 | -0.25 | 1.00 | 8.22E-04 | 0.49 | 1.50 | 1.46E-04 | 0.74 | 1.51 | 59347 | 173388 | 506781 | 803104 | 598467 | 908058 | 415096 | 679258 |
| RP_POS_309_9057a0_253 | RP | POS | 0.25 | 309.9057 | | | | 7.61E-01 | 0.06 | 0.38 | 2.56E-01 | -0.23 | 0.78 | 7.99E-01 | -0.06 | 0.24 | 4.10E-01 | 0.18 | 0.43 | 1088351 | 143497 | 1097499 | 158022 | 1115529 | 146493 | 1079469 | 168308 |
| RP_POS_515_41296a14_267 | RP | POS | 14.27 | 515.4130 | | | | 3.72E-01 | 0.13 | 0.43 | 2.56E-01 | 0.25 | 0.72 | 2.34E-01 | 0.26 | 0.54 | 9.53E-01 | 0.01 | 0.39 | 485619044 | 66178432 | 501016525 | 125935231 | 486636302 | 87589932 | 515396749 | 154754689 |
| RP_POS_464_19171a6_602 | RP | POS | 6.60 | 464.1917 | | | | 3.27E-03 | 0.46 | 1.58 | 2.56E-01 | -0.24 | 0.72 | 5.83E-02 | 0.34 | 1.40 | 3.78E-03 | 0.59 | 1.12 | 1941198 | 2313122 | 3669858 | 4006053 | 4126544 | 4293252 | 3213171 | 3683311 |
| RP_POS_550_31537a10_618 | RP | POS | 10.62 | 550.3154 | | | | 6.32E-03 | 0.62 | 1.40 | 2.57E-01 | -0.21 | 1.03 | 4.15E-02 | 0.52 | 1.07 | 1.94E-03 | 0.73 | 1.50 | 7689201 | 2111914 | 8868144 | 1731935 | 9065620 | 1348709 | 8670668 | 2040052 |
| RP_POS_392_22147a11_167 | RP | POS | 11.17 | 392.2215 | | | | 1.19E-02 | 0.45 | 0.95 | 2.57E-01 | -0.24 | 0.82 | 1.04E-01 | 0.33 | 0.73 | 6.64E-03 | 0.57 | 1.07 | 1087954 | 70509 | 1128069 | 92535 | 1138610 | 93406 | 1117529 | 91372 |
| RP_POS_764_52203a12_954 | RP | POS | 12.95 | 764.5220 | PE 38:6 + PC 35:6 | 623.509(55), 313.2715(3), 764.547(2), 184.0735(100) | 3 | 9.75E-01 | 0.01 | 0.68 | 2.57E-01 | 0.24 | 0.68 | 6.09E-01 | 0.12 | 0.57 | 5.45E-01 | -0.11 | 0.36 | 5568929 | 3322848 | 5591415 | 3855989 | 5151356 | 2497880 | 6031473 | 4837989 |
| RP_POS_621_30939a11_426 | RP | POS | 11.43 | 621.3094 | | | | 6.64E-01 | -0.09 | 0.22 | 2.57E-01 | 0.22 | 0.98 | 9.46E-01 | 0.02 | 0.37 | 3.83E-01 | -0.21 | 0.35 | 505844 | 104455 | 496809 | 92995 | 486219 | 88872 | 507399 | 96672 |
| RP_POS_700_48743a12_272 | RP | POS | 12.27 | 700.4874 | | | | 1.48E-01 | 0.29 | 0.71 | 2.57E-01 | 0.23 | 0.74 | 8.63E-02 | 0.41 | 0.79 | 4.05E-01 | 0.18 | 0.36 | 388422 | 504003 | 539868 | 522368 | 480371 | 452759 | 599365 | 582316 |
| RP_POS_318_24036a9_213 | RP | POS | 9.21 | 318.2404 | | 318.2419(100) | 4 | 1.97E-01 | -0.34 | 0.96 | 2.57E-01 | -0.19 | 0.83 | 1.05E-01 | -0.44 | 0.87 | 3.87E-01 | -0.25 | 0.58 | 5534517 | 8913967 | 3347573 | 5342771 | 3956180 | 6255843 | 2738966 | 4216808 |
| RP_POS_583_25452a11_94 | RP | POS | 11.94 | 583.2545 | | | | 5.77E-01 | -0.08 | 0.49 | 2.57E-01 | 0.26 | 1.06 | 8.41E-01 | 0.05 | 0.30 | 3.52E-02 | -0.21 | 1.04 | 1185935 | 1548260 | 939877 | 3437640 | 546620 | 746205 | 1333135 | 4796193 |
| HILIC_POS_454_29251a1_965 | HILIC | POS | 1.965 | 454.2925 | | | | 8.94E-02 | 0.29 | 0.81 | 2.58E-01 | -0.24 | 0.82 | 4.24E-01 | 0.17 | 0.27 | 3.35E-02 | 0.41 | 0.75 | 1130621 | 418593 | 1296045 | 613913 | 1365866 | 562919 | 1226225 | 659276 |
| RP_POS_294_11243a8_289 | RP | POS | 8.29 | 294.1124 | | | | 1.55E-06 | 0.66 | 1.09 | 2.58E-01 | 0.24 | 0.90 | 8.89E-05 | 0.78 | 1.25 | 5.72E-04 | 0.54 | 1.11 | 680661 | 129808 | 885150 | 334117 | 847185 | 283924 | 923115 | 376818 |
| RP_POS_645_29108a11_804 | RP | POS | 11.80 | 645.2911 | | | | 3.25E-02 | -0.42 | 1.07 | 2.58E-01 | 0.23 | 1.30 | 2.16E-01 | -0.31 | 0.65 | 5.41E-03 | -0.53 | 1.28 | 8679052 | 4985849 | 6407333 | 5450413 | 5786933 | 3222100 | 7027733 | 6989481 |
| RP_NEG_171_02673a0_737 | RP | NEG | 0.74 | 171.0267 | | | | 7.00E-08 | 0.71 | 1.21 | 2.58E-01 | 0.25 | 0.77 | 9.24E-05 | 0.83 | 1.29 | 6.22E-06 | 0.59 | 1.40 | 235736 | 235556 | 716265 | 729785 | 633291 | 497423 | 799238 | 902530 |
| RP_NEG_172_99072a3_098 | RP | NEG | 3.10 | 172.9907 | | | | 1.17E-01 | 0.24 | 0.86 | 2.59E-01 | 0.25 | 0.96 | 8.01E-02 | 0.36 | 1.20 | 5.04E-01 | 0.11 | 0.36 | 19370169 | 18820322 | 27155778 | 36343322 | 23030223 | 30573276 | 31281333 | 41220985 |
| RP_POS_297_24182a9_925 | RP | POS | 9.93 | 297.2418 | | | | 5.93E-09 | 0.89 | 1.49 | 2.59E-01 | -0.23 | 1.19 | 1.75E-05 | 0.78 | 1.42 | 1.82E-07 | 1.01 | 1.72 | 1343469 | 234152 | 1726173 | 435555 | 1775591 | 445712 | 1676755 | 423844 |
| HILIC_POS_59_06126a0_541 | HILIC | POS | 0.541 | 59.0613 | | | | 5.18E-02 | -0.30 | 0.45 | 2.59E-01 | -0.25 | 0.72 | 1.47E-02 | -0.42 | 0.83 | 3.88E-01 | -0.18 | 0.29 | 2464994 | 351413 | 2289796 | 636717 | 2362041 | 713580 | 2217552 | 547032 |
| RP_POS_350_98441a15_406 | RP | POS | 15.41 | 350.9844 | | | | 2.49E-01 | -0.19 | 0.81 | 2.59E-01 | 0.24 | 0.74 | 6.74E-01 | -0.07 | 0.67 | 1.61E-01 | -0.32 | 0.89 | 2921257 | 191458 | 2870004 | 283780 | 2837783 | 341463 | 2902226 | 209737 |
| RP_POS_203_97173a1_459 | RP | POS | 1.46 | 203.9717 | | | | 3.40E-11 | 0.88 | 1.44 | 2.59E-01 | 0.24 | 0.78 | 3.26E-07 | 1.00 | 1.65 | 1.73E-06 | 0.76 | 1.45 | 471618 | 474284 | 1686738 | 1452384 | 1521998 | 1286753 | 1851477 | 1597113 |
| RP_POS_396_26321a12_768 | RP | POS | 12.77 | 396.2632 | | | | 2.11E-02 | -0.45 | 0.93 | 2.59E-01 | 0.23 | 0.72 | 1.26E-01 | -0.33 | 0.65 | 1.04E-02 | -0.56 | 1.02 | 1057336 | 318914 | 898761 | 359969 | 857974 | 357536 | 939548 | 361349 |
| RP_POS_298_27408a9_958 | RP | POS | 9.96 | 298.2741 | Unknown | 298.274(45), 95.0858(100), 263.2368(65) | 4 | 2.60E-01 | -0.34 | 0.99 | 2.59E-01 | -0.15 | 0.79 | 1.76E-01 | -0.42 | 0.84 | 3.94E-01 | -0.27 | 0.66 | 5390696 | 9506860 | 3416100 | 3828321 | 3849881 | 4289498 | 2982319 | 3290499 |
| HILIC_POS_176_13916a3_702 | HILIC | POS | 3.702 | 176.1392 | | | | 8.95E-01 | 0.02 | 0.73 | 2.59E-01 | 0.24 | 0.89 | 5.08E-01 | 0.14 | 0.25 | 6.64E-01 | -0.09 | 0.63 | 4526557 | 1325125 | 4563835 | 1568277 | 4386207 | 1558362 | 4741463 | 1573625 |
| HILIC_NEG_144_92352a7_948 | HILIC | NEG | 7.948 | 144.9235 | | | | 8.47E-03 | -0.50 | 0.77 | 2.60E-01 | 0.23 | 1.26 | 6.78E-02 | -0.39 | 0.80 | 4.54E-03 | -0.62 | 1.03 | 62038350 | 18012883 | 51693530 | 20714350 | 49348858 | 20778098 | 54038202 | 20590694 |
| RP_POS_400_1817a0_71 | RP | POS | 0.71 | 400.1817 | | | | 1.52E-01 | 0.19 | 0.48 | 2.60E-01 | 0.26 | 0.83 | 9.55E-02 | 0.31 | 0.56 | 7.05E-01 | 0.06 | 0.79 | 314483 | 71080 | 351073 | 220712 | 326099 | 197306 | 376048 | 241286 |
| RP_POS_293_93311a15_509 | RP | POS | 15.51 | 293.9331 | | | | 6.37E-01 | 0.10 | 0.34 | 2.60E-01 | -0.22 | 0.70 | 9.87E-01 | 0.00 | 0.52 | 3.66E-01 | 0.21 | 0.36 | 3127954 | 441742 | 3169127 | 378416 | 3211937 | 345379 | 3126316 | 407790 |
| RP_POS_790_55884a11_613 | RP | POS | 11.61 | 790.5588 | | | | 7.31E-01 | 0.05 | 0.31 | 2.60E-01 | -0.25 | 0.81 | 6.52E-01 | -0.07 | 0.22 | 4.34E-01 | 0.18 | 0.46 | 3124805 | 5562923 | 3589643 | 9219699 | 4634299 | 11667465 | 2544987 | 5777850 |
| RP_POS_583_25494a5_914 | RP | POS | 5.91 | 583.2549 | | | | 1.67E-01 | 0.20 | 0.72 | 2.61E-01 | 0.25 | 1.12 | 1.32E-01 | 0.33 | 0.59 | 5.91E-01 | 0.08 | 0.91 | 381747 | 275701 | 486198 | 571660 | 421575 | 392295 | 550822 | 705578 |
| RP_NEG_455_60333a15_584 | RP | NEG | 15.58 | 455.6033 | | | | 2.07E-01 | -0.26 | 0.59 | 2.61E-01 | 0.22 | 0.97 | 5.17E-01 | -0.15 | 0.33 | 9.92E-02 | -0.37 | 0.69 | 722661 | 39310 | 712516 | 38225 | 708202 | 36290 | 716831 | 39964 |
| RP_POS_330_75305a0_522 | RP | POS | 0.52 | 330.7531 | | | | 6.20E-05 | -0.62 | 1.12 | 2.61E-01 | 0.24 | 1.01 | 8.22E-03 | -0.50 | 1.01 | 9.87E-05 | -0.74 | 1.31 | 423085 | 69666 | 348791 | 127810 | 334373 | 125654 | 363209 | 129580 |
| HILIC_POS_226_04488a5_119 | HILIC | POS | 5.119 | 226.0449 | | | | 7.54E-01 | 0.05 | 0.89 | 2.61E-01 | 0.24 | 1.00 | 4.03E-01 | 0.17 | 0.29 | 7.42E-01 | -0.07 | 0.66 | 1039444 | 234645 | 1055962 | 323158 | 1019511 | 309372 | 1092414 | 335517 |
| HILIC_POS_111_05559a6_789 | HILIC | POS | 6.789 | 111.0556 | | | | 8.10E-01 | -0.05 | 0.73 | 2.62E-01 | -0.23 | 0.84 | 4.72E-01 | -0.16 | 0.45 | 7.80E-01 | 0.06 | 0.40 | 1095740 | 345806 | 1078767 | 347752 | 1117977 | 355851 | 1039558 | 338472 |
| RP_NEG_193_08643a7_695 | RP | NEG | 7.70 | 193.0864 | | | | 2.28E-02 | -0.45 | 0.83 | 2.62E-01 | -0.23 | 0.70 | 1.00E-02 | -0.56 | 0.96 | 1.33E-01 | -0.33 | 0.65 | 3357203 | 137205 | 3290412 | 150202 | 3307336 | 156374 | 3273487 | 143333 |
| RP_POS_383_39987a11_668 | RP | POS | 11.67 | 383.3999 | | | | 7.83E-01 | -0.06 | 0.71 | 2.62E-01 | -0.23 | 0.66 | 4.52E-01 | -0.17 | 0.50 | 8.08E-01 | 0.06 | 0.46 | 3350525 | 1530608 | 3264448 | 1523008 | 3435966 | 1576985 | 3092929 | 1462713 |
| HILIC_NEG_125_87303a3_06 | HILIC | NEG | 3.06 | 125.8730 | | | | 5.30E-01 | 0.10 | 1.01 | 2.62E-01 | 0.25 | 1.02 | 2.00E-01 | 0.22 | 0.69 | 9.02E-01 | -0.03 | 1.14 | 801742 | 117257 | 820187 | 206309 | 796945 | 232911 | 843429 | 175058 |
| RP_POS_280_93927a15_444 | RP | POS | 15.44 | 280.9393 | | | | 9.61E-01 | -0.01 | 0.46 | 2.62E-01 | -0.23 | 0.68 | 5.81E-01 | -0.13 | 0.32 | 5.96E-01 | 0.11 | 0.34 | 6614987 | 292829 | 6611959 | 341894 | 6650458 | 295224 | 6573460 | 382082 |
| RP_POS_173_96126a15_422 | RP | POS | 15.42 | 173.9613 | | | | 8.83E-02 | 0.35 | 0.59 | 2.63E-01 | 0.22 | 0.68 | 2.92E-02 | 0.46 | 0.94 | 3.25E-01 | 0.24 | 0.42 | 4631038 | 485715 | 4801959 | 480751 | 4747803 | 570002 | 4856114 | 368879 |
| HILIC_POS_133_03194a4_625 | HILIC | POS | 4.625 | 133.0319 | | | | 7.83E-01 | -0.04 | 0.52 | 2.63E-01 | 0.26 | 0.68 | 6.93E-01 | 0.09 | 0.21 | 7.42E-02 | -0.16 | 1.02 | 2668298 | 520529 | 2620479 | 1472587 | 2454070 | 523631 | 2786887 | 2012604 |
| RP_NEG_326_98926a8_592 | RP | NEG | 8.59 | 326.9893 | | | | 1.33E-01 | -0.30 | 0.89 | 2.63E-01 | -0.23 | 0.85 | 5.76E-02 | -0.42 | 1.01 | 4.08E-01 | -0.19 | 0.55 | 6474006 | 1728619 | 5935025 | 1772397 | 6134313 | 1911110 | 5735737 | 1616633 |
| RP_NEG_207_93219a1_036 | RP | NEG | 1.04 | 207.9322 | | | | 7.20E-01 | 0.06 | 0.56 | 2.63E-01 | -0.24 | 0.87 | 7.88E-01 | -0.05 | 0.35 | 3.90E-01 | 0.18 | 0.80 | 2170278 | 1001855 | 2249427 | 1303700 | 2395986 | 1355735 | 2102869 | 1245833 |
| RP_POS_585_27057a5_914 | RP | POS | 5.91 | 585.2706 | | | | 1.91E-01 | 0.20 | 0.75 | 2.63E-01 | 0.25 | 1.10 | 1.36E-01 | 0.32 | 0.60 | 6.27E-01 | 0.08 | 0.93 | 2444194 | 1670059 | 3010744 | 3129859 | 2658598 | 2298833 | 3362890 | 3775479 |
| RP_POS_431_75452a13_207 | RP | POS | 13.21 | 431.7545 | | | | 4.83E-04 | 0.64 | 1.12 | 2.64E-01 | 0.23 | 0.89 | 1.09E-03 | 0.76 | 1.18 | 4.01E-03 | 0.53 | 1.11 | 2788969 | 578699 | 3248629 | 718185 | 3167970 | 529934 | 3329288 | 864869 |
| RP_NEG_357_0318a0_708 | RP | NEG | 0.71 | 357.0318 | | | | 8.54E-02 | 0.28 | 0.64 | 2.64E-01 | -0.24 | 0.71 | 3.95E-01 | 0.16 | 0.50 | 4.83E-02 | 0.40 | 0.78 | 5319215 | 1389998 | 5893464 | 2201430 | 6140392 | 2315286 | 5646536 | 2075212 |
| HILIC_POS_292_20633a0_508 | HILIC | POS | 0.508 | 292.2063 | | | | 1.23E-01 | 0.18 | 0.38 | 2.64E-01 | 0.26 | 0.67 | 1.83E-01 | 0.31 | 0.50 | 3.44E-02 | 0.05 | 1.15 | 78903 | 215404 | 871858 | 5078404 | 299205 | 668581 | 1444511 | 7140760 |
| RP_POS_287_22226a8_909 | RP | POS | 8.91 | 287.2223 | | | | 1.68E-01 | 0.28 | 0.65 | 2.64E-01 | 0.23 | 0.66 | 1.06E-01 | 0.39 | 0.73 | 4.25E-01 | 0.16 | 0.38 | 1681769 | 308549 | 1770290 | 321651 | 1734205 | 253881 | 1806375 | 376719 |
| RP_NEG_634_33282a10_626 | RP | NEG | 10.63 | 634.3328 | | | | 9.92E-02 | -0.34 | 0.81 | 2.65E-01 | -0.22 | 0.83 | 4.43E-02 | -0.45 | 0.88 | 3.28E-01 | -0.23 | 0.61 | 15087024 | 2679130 | 14172284 | 2695114 | 14474277 | 2851434 | 13870291 | 2521650 |
| RP_NEG_187_13365a8_132 | RP | NEG | 8.13 | 187.1337 | | | | 7.09E-03 | 0.45 | 1.45 | 2.65E-01 | 0.24 | 1.38 | 5.32E-03 | 0.57 | 1.31 | 8.50E-02 | 0.33 | 1.22 | 2180827 | 1385859 | 3073870 | 2107294 | 2837800 | 2023221 | 3309940 | 2182852 |
| RP_POS_323_25583a10_415 | RP | POS | 10.42 | 323.2558 | | | | 3.48E-02 | -0.42 | 1.03 | 2.65E-01 | 0.22 | 0.97 | 1.56E-01 | -0.31 | 0.84 | 1.83E-02 | -0.54 | 1.00 | 3480368 | 429226 | 3289655 | 447741 | 3239519 | 456587 | 3339791 | 437531 |
| RP_NEG_351_84293a15_713 | RP | NEG | 15.71 | 351.8429 | | | | 1.22E-01 | -0.30 | 0.54 | 2.65E-01 | -0.23 | 0.81 | 6.46E-02 | -0.41 | 0.71 | 3.83E-01 | -0.19 | 0.45 | 1074609 | 176264 | 1016737 | 196415 | 1038728 | 186886 | 994746 | 205016 |
| HILIC_POS_610_62384a10_707 | HILIC | POS | 10.707 | 610.6238 | | | | 3.32E-01 | -0.18 | 0.67 | 2.65E-01 | -0.23 | 0.88 | 1.37E-01 | -0.30 | 0.79 | 7.81E-01 | -0.06 | 0.28 | 3868058 | 146995 | 3837196 | 179567 | 3857306 | 201569 | 3817086 | 153919 |
| RP_POS_1069_62231a10_308 | RP | POS | 10.31 | 1069.6223 | | | | 3.21E-03 | 0.48 | 1.10 | 2.65E-01 | -0.24 | 0.86 | 5.76E-02 | 0.37 | 0.81 | 2.47E-03 | 0.60 | 1.23 | 828425 | 420060 | 1129732 | 658922 | 1203491 | 666458 | 1055972 | 649575 |
| HILIC_POS_199_18056a4_009 | HILIC | POS | 4.009 | 199.1806 | | | | 7.16E-01 | 0.07 | 0.72 | 2.65E-01 | -0.23 | 0.81 | 8.44E-01 | -0.04 | 0.08 | 4.13E-01 | 0.19 | 0.31 | 8065767 | 1702863 | 8194635 | 1838654 | 8400399 | 1931294 | 7988872 | 1736084 |
| RP_POS_836_79102a0_494 | RP | POS | 0.49 | 836.7910 | | | | 5.23E-09 | -0.75 | 1.38 | 2.65E-01 | 0.24 | 1.22 | 2.58E-04 | -0.63 | 1.34 | 8.21E-07 | -0.87 | 1.58 | 1764025 | 201215 | 1270575 | 703344 | 1191878 | 692666 | 1349272 | 712078 |
| RP_POS_283_26282a11_254 | RP | POS | 11.25 | 283.2628 | | | | 8.17E-06 | 0.81 | 1.76 | 2.65E-01 | 0.22 | 1.16 | 2.61E-05 | 0.92 | 1.70 | 2.77E-04 | 0.70 | 1.66 | 6031591 | 2732758 | 8930803 | 3562920 | 8532112 | 3118667 | 9329495 | 3949352 |
| RP_POS_711_54047a12_471 | RP | POS | 12.47 | 711.5405 | | | | 5.08E-07 | -1.19 | 2.03 | 2.66E-01 | -0.18 | 0.66 | 1.57E-07 | -1.28 | 2.11 | 7.89E-06 | -1.10 | 1.73 | 2030299 | 731219 | 1200458 | 551453 | 1262152 | 627406 | 1138765 | 461519 |
| RP_POS_271_16595a9_207 | RP | POS | 9.21 | 271.1660 | | | | 6.31E-02 | -0.33 | 0.62 | 2.66E-01 | -0.23 | 0.82 | 3.48E-02 | -0.45 | 0.76 | 2.83E-01 | -0.22 | 0.53 | 1373061 | 45154 | 1354329 | 58778 | 1360900 | 56007 | 1347758 | 61279 |
| RP_POS_281_91141a0_253 | RP | POS | 0.25 | 281.9114 | | | | 6.52E-01 | 0.09 | 0.59 | 2.66E-01 | -0.23 | 0.88 | 9.00E-01 | -0.03 | 0.29 | 3.58E-01 | 0.20 | 0.78 | 1811780 | 215454 | 1832159 | 239594 | 1858943 | 239118 | 1805376 | 239460 |
| RP_POS_615_42841a15_099 | RP | POS | 15.10 | 615.4284 | | | | 3.34E-01 | 0.21 | 0.48 | 2.66E-01 | -0.22 | 0.68 | 6.67E-01 | 0.10 | 0.32 | 1.82E-01 | 0.32 | 0.71 | 4218669 | 1647306 | 4536179 | 1456546 | 4698970 | 1442208 | 4373387 | 1467072 |
| RP_POS_655_39435a10_925 | RP | POS | 10.93 | 655.3944 | | | | 1.31E-02 | 0.29 | 0.88 | 2.66E-01 | -0.25 | 1.09 | 2.68E-01 | 0.17 | 1.04 | 1.99E-02 | 0.42 | 1.03 | 745382 | 76957 | 902929 | 609792 | 971078 | 657001 | 834781 | 556912 |
| RP_POS_141_05025a15_447 | RP | POS | 15.45 | 141.0503 | | | | 1.85E-01 | 0.27 | 0.71 | 2.66E-01 | -0.22 | 0.66 | 4.96E-01 | 0.16 | 0.52 | 8.61E-02 | 0.38 | 0.69 | 8891991 | 268572 | 8965651 | 275075 | 8996390 | 258239 | 8934912 | 290278 |

**Supplementary table 1: Metabolomics results** (in asceding order according to p-value from progressive ALD vs non-progressive ALD comparison, with molecular features with ID_level of 1-2 first)

| | | | | | | | | all_ALD_vs_control | | | Prog_ALD_vs_non-prog_ALD | | | Prog_ALD_vs_control_ALD | | | Non-prog_ALD_vs_control | | | Healthy_control | | all_ALD | | Non-prog_ALD | | Progressive_ALD | |
|---|---|---|---|---|---|---|---|---|---|---|---|---|---|---|---|---|---|---|---|---|---|---|---|---|---|---|---|
| | | | | | | | | t-test | Cohen's | PLS-DA | t-test | Cohen's | PLS-DA | t-test | Cohen's | PLS-DA | t-test | Cohen's | PLS-DA | | | | | | | | |
| Feature_ID | RP_HILIC | POS_NEG | Average.Rt | Average.Mz | Metabolite.name | curated_MSMS | ID_level | p | d | VIP | p | d | VIP | p | d | VIP | p | d | VIP | Mean | SD | Mean | SD | Mean | SD | Mean | SD |
| RP_NEG_174_9864a3_09 | RP | NEG | 3.09 | 174.9864 | | | | 1.06E-01 | 0.24 | 0.87 | 2.66E-01 | 0.25 | 0.96 | 7.86E-02 | 0.36 | 1.20 | 4.74E-01 | 0.12 | 0.38 | 856421 | 865133 | 1235205 | 1747948 | 1039933 | 1445005 | 1430476 | 2001784 |
| HILIC_POS_165_05475a5_119 | HILIC | POS | 5.119 | 165.0548 | | | | 3.06E-01 | 0.18 | 0.97 | 2.66E-01 | 0.24 | 1.14 | 1.81E-01 | 0.30 | 0.47 | 7.47E-01 | 0.06 | 0.47 | 1246033 | 326974 | 1321362 | 448033 | 1271318 | 369643 | 1371405 | 513630 |
| RP_NEG_180_97296a15_463 | RP | NEG | 15.46 | 180.9730 | | | | 3.21E-01 | -0.23 | 0.53 | 2.67E-01 | -0.21 | 0.67 | 1.68E-01 | -0.33 | 0.57 | 6.24E-01 | -0.13 | 0.40 | 4284418 | 196097 | 4246388 | 153781 | 4263556 | 171886 | 4229221 | 132791 |
| RP_POS_470_13562a6_499 | RP | POS | 6.50 | 470.1356 | | | | 6.25E-02 | 0.39 | 0.71 | 2.67E-01 | -0.22 | 0.70 | 2.25E-01 | 0.28 | 0.61 | 3.08E-02 | 0.50 | 0.88 | 382730 | 282988 | 491591 | 276863 | 522464 | 274147 | 460718 | 278870 |
| HILIC_POS_660_10303a0_575 | HILIC | POS | 0.575 | 660.1030 | | | | 1.96E-06 | 0.60 | 1.28 | 2.67E-01 | -0.25 | 0.93 | 8.13E-04 | 0.48 | 1.46 | 2.79E-04 | 0.72 | 1.45 | 57679 | 194484 | 487677 | 790007 | 575772 | 912530 | 399582 | 642192 |
| RP_POS_210_08932a6_519 | RP | POS | 6.52 | 210.0893 | | | | 7.84E-03 | 0.40 | 0.85 | 2.67E-01 | -0.24 | 0.72 | 8.40E-02 | 0.28 | 0.65 | 1.29E-02 | 0.52 | 0.96 | 97861 | 267865 | 288650 | 520458 | 346661 | 603876 | 230639 | 419221 |
| RP_NEG_343_99527a1_093 | RP | NEG | 1.09 | 343.9953 | | | | 3.04E-01 | -0.19 | 0.78 | 2.68E-01 | -0.23 | 1.05 | 1.05E-01 | -0.31 | 0.84 | 7.52E-01 | -0.07 | 0.79 | 15146013 | 11612296 | 12558533 | 14199740 | 14141661 | 17052710 | 10975405 | 10557953 |
| RP_POS_238_88387a15_367 | RP | POS | 15.37 | 238.8839 | | | | 7.63E-01 | -0.07 | 0.38 | 2.68E-01 | 0.22 | 0.72 | 8.41E-01 | 0.04 | 0.29 | 4.96E-01 | -0.17 | 0.47 | 1515347 | 230563 | 1501452 | 207579 | 1478308 | 251903 | 1524596 | 150031 |
| RP_POS_459_27994a4_885 | RP | POS | 4.89 | 459.2799 | | | | 4.00E-01 | 0.19 | 1.19 | 2.68E-01 | -0.21 | 1.24 | 7.14E-01 | 0.09 | 0.99 | 2.48E-01 | 0.30 | 1.13 | 499367 | 1608924 | 765571 | 1316260 | 912051 | 1490523 | 619090 | 1111300 |
| RP_POS_209_1076a6_497 | RP | POS | 6.50 | 209.1076 | Unknown | 209.111(100) | 4 | 2.26E-01 | 0.26 | 0.65 | 2.68E-01 | -0.22 | 0.78 | 5.33E-01 | 0.15 | 0.48 | 1.15E-01 | 0.37 | 0.81 | 1770097 | 840520 | 1976419 | 793957 | 2064721 | 768444 | 1888118 | 816812 |
| HILIC_NEG_356_99289a7_309 | HILIC | NEG | 7.309 | 356.9929 | | | | 4.21E-01 | 0.15 | 0.50 | 2.68E-01 | -0.23 | 0.68 | 8.53E-01 | 0.04 | 0.42 | 2.23E-01 | 0.27 | 0.55 | 616879 | 26265 | 621356 | 29885 | 624680 | 30657 | 618033 | 29019 |
| RP_POS_393_2959a11_436 | RP | POS | 11.44 | 393.2959 | | | | 3.58E-01 | 0.17 | 0.65 | 2.68E-01 | -0.23 | 0.71 | 8.00E-01 | 0.05 | 0.24 | 2.00E-01 | 0.28 | 0.86 | 6947019 | 1073177 | 7162704 | 1356684 | 7313549 | 1488688 | 7011859 | 1206698 |
| RP_POS_191_97168a0_254 | RP | POS | 0.25 | 191.9717 | | | | 4.86E-01 | 0.15 | 0.57 | 2.69E-01 | -0.22 | 0.75 | 8.62E-01 | 0.04 | 0.17 | 2.69E-01 | 0.26 | 0.85 | 18616359 | 3009671 | 19035946 | 2733330 | 19339585 | 2618356 | 18732306 | 2837379 |
| HILIC_POS_110_07167a6_784 | HILIC | POS | 6.784 | 110.0717 | | | | 6.42E-01 | -0.09 | 0.89 | 2.69E-01 | -0.23 | 0.98 | 3.22E-01 | -0.20 | 0.66 | 8.93E-01 | 0.03 | 0.70 | 1791982 | 476991 | 1744238 | 579343 | 1808555 | 626783 | 1679921 | 526142 |
| HILIC_NEG_391_66599a1_364 | HILIC | NEG | 1.364 | 391.6660 | | | | 9.24E-06 | 0.72 | 1.13 | 2.69E-01 | -0.23 | 0.70 | 3.24E-03 | 0.60 | 0.99 | 4.27E-06 | 0.83 | 1.56 | 394551 | 159460 | 575750 | 261645 | 604790 | 225457 | 546710 | 292851 |
| RP_POS_533_84021a10_617 | RP | POS | 10.62 | 533.8402 | | | | 1.67E-01 | 0.31 | 1.20 | 2.70E-01 | -0.21 | 0.90 | 4.09E-01 | 0.21 | 0.78 | 8.03E-02 | 0.42 | 1.15 | 639490 | 216576 | 698944 | 180569 | 718975 | 161461 | 678913 | 197451 |
| RP_POS_503_30569a5_107 | RP | POS | 5.11 | 503.3057 | | | | 3.15E-02 | 0.37 | 1.20 | 2.70E-01 | -0.23 | 1.26 | 1.68E-01 | 0.25 | 1.12 | 2.57E-02 | 0.49 | 1.24 | 339501 | 1052840 | 861264 | 1482542 | 1025712 | 1680229 | 696816 | 1249944 |
| HILIC_NEG_178_85893a2_974 | HILIC | NEG | 2.974 | 178.8589 | | | | 7.83E-01 | 0.06 | 0.29 | 2.70E-01 | 0.22 | 0.68 | 4.84E-01 | 0.17 | 0.28 | 8.17E-01 | -0.05 | 0.54 | 620354 | 202228 | 631623 | 192779 | 610251 | 173978 | 652995 | 209488 |
| HILIC_NEG_192_89981a2_966 | HILIC | NEG | 2.966 | 192.8998 | | | | 4.54E-01 | -0.17 | 0.52 | 2.70E-01 | 0.21 | 0.85 | 7.91E-01 | -0.07 | 0.11 | 2.63E-01 | -0.28 | 0.77 | 439253 | 209193 | 408504 | 170698 | 389604 | 166870 | 427404 | 174055 |
| RP_POS_1008_80438a0_491 | RP | POS | 0.49 | 1008.8044 | | | | 2.33E-09 | 0.73 | 1.36 | 2.70E-01 | -0.24 | 1.22 | 1.25E-04 | 0.60 | 1.36 | 4.51E-06 | 0.85 | 1.53 | 547092 | 186360 | 1657454 | 1668729 | 1842205 | 1767774 | 1472703 | 1559314 |
| RP_POS_623_29059a7_257 | RP | POS | 7.26 | 623.2906 | | | | 1.68E-01 | -0.25 | 0.76 | 2.71E-01 | 0.23 | 0.90 | 5.07E-01 | -0.14 | 0.56 | 8.50E-02 | -0.37 | 0.82 | 2063109 | 113019 | 2029037 | 140215 | 2013527 | 142845 | 2044548 | 137209 |
| RP_POS_120_08019a4_72 | RP | POS | 4.72 | 120.0802 | | | | 7.37E-06 | 0.53 | 1.30 | 2.71E-01 | 0.25 | 1.20 | 8.62E-04 | 0.65 | 1.36 | 2.41E-03 | 0.41 | 1.38 | 3388 | 4081 | 789833 | 1661609 | 605954 | 1331665 | 973711 | 1932847 |
| RP_POS_938_77222a0_495 | RP | POS | 0.50 | 938.7722 | | | | 7.05E-10 | -0.76 | 1.40 | 2.71E-01 | 0.24 | 1.24 | 1.05E-04 | -0.64 | 1.41 | 1.24E-06 | -0.88 | 1.57 | 4202748 | 250427 | 3013693 | 1705357 | 2825149 | 1749198 | 3202238 | 1656379 |
| RP_NEG_507_22452a9_349 | RP | NEG | 9.35 | 507.2245 | | | | 5.00E-03 | -0.76 | 1.29 | 2.71E-01 | -0.17 | 0.72 | 2.26E-03 | -0.85 | 1.38 | 1.68E-02 | -0.68 | 0.97 | 1641740 | 1715614 | 693874 | 943453 | 798249 | 1093038 | 589499 | 762500 |
| RP_NEG_199_00674a5_419 | RP | NEG | 5.42 | 199.0067 | | | | 4.69E-03 | 0.51 | 0.88 | 2.71E-01 | 0.23 | 1.07 | 8.07E-03 | 0.63 | 1.01 | 2.35E-02 | 0.40 | 0.81 | 1967143 | 2655747 | 3666127 | 3416050 | 3287786 | 2254870 | 4044468 | 4266190 |
| RP_POS_297_92844a15_499 | RP | POS | 15.50 | 297.9284 | | | | 5.05E-03 | -0.42 | 0.76 | 2.71E-01 | -0.24 | 0.74 | 9.02E-03 | -0.54 | 0.85 | 6.58E-02 | -0.30 | 0.72 | 2953485 | 231693 | 2779186 | 449880 | 2828948 | 372801 | 2729425 | 514666 |
| RP_POS_285_24213a10_12 | RP | POS | 10.12 | 285.2421 | | | | 4.01E-03 | -0.57 | 0.97 | 2.71E-01 | -0.22 | 0.92 | 1.57E-03 | -0.68 | 1.23 | 4.27E-02 | -0.46 | 0.83 | 1377884 | 171498 | 1270861 | 188075 | 1291644 | 203724 | 1250078 | 170532 |
| RP_POS_420_24771a13_061 | RP | POS | 13.06 | 420.2477 | | | | 4.71E-01 | 0.17 | 0.70 | 2.72E-01 | -0.20 | 0.70 | 7.81E-01 | 0.07 | 0.34 | 2.77E-01 | 0.27 | 0.65 | 4379667 | 1772236 | 4626103 | 1291043 | 4768736 | 1165230 | 4483470 | 1402968 |
| RP_NEG_416_14499a11_256 | RP | NEG | 11.26 | 416.1450 | | | | 1.43E-04 | 0.81 | 1.36 | 2.72E-01 | -0.21 | 1.10 | 2.82E-03 | 0.70 | 1.31 | 4.63E-05 | 0.91 | 1.62 | 540108 | 213214 | 716482 | 203927 | 739015 | 174884 | 693948 | 228911 |
| RP_POS_308_17563a4_697 | RP | POS | 4.70 | 308.1756 | | | | 6.52E-03 | 0.37 | 0.69 | 2.72E-01 | 0.25 | 0.89 | 2.64E-02 | 0.50 | 0.75 | 3.27E-02 | 0.25 | 0.74 | 11972831 | 6988308 | 17952318 | 17731914 | 15990030 | 9721410 | 19914607 | 23084501 |
| RP_POS_395_2146a11_254 | RP | POS | 11.25 | 395.2146 | | | | 3.19E-05 | 0.77 | 1.72 | 2.72E-01 | 0.22 | 1.01 | 8.03E-05 | 0.88 | 1.62 | 6.61E-04 | 0.66 | 1.64 | 2149093 | 706864 | 2833955 | 885271 | 2736255 | 758303 | 2931656 | 994434 |
| HILIC_POS_154_15887a0_658 | HILIC | POS | 0.658 | 154.1589 | | | | 3.94E-04 | -0.60 | 1.22 | 2.72E-01 | -0.23 | 0.98 | 1.34E-03 | -0.71 | 1.06 | 4.79E-03 | -0.49 | 1.09 | 899175 | 77273 | 833181 | 114318 | 845805 | 86416 | 820558 | 136422 |
| HILIC_POS_150_05835a0_846 | HILIC | POS | 0.846 | 150.0584 | | | | 7.59E-02 | 0.34 | 0.76 | 2.72E-01 | 0.23 | 0.88 | 4.49E-02 | 0.45 | 0.82 | 2.79E-01 | 0.22 | 0.40 | 4900611 | 2100500 | 5704548 | 2455651 | 5433757 | 2244568 | 5975338 | 2644954 |
| HILIC_NEG_200_85875a5_463 | HILIC | NEG | 5.463 | 200.8588 | | | | 9.92E-01 | 0.00 | 0.31 | 2.72E-01 | -0.22 | 0.90 | 6.31E-01 | -0.11 | 0.21 | 6.40E-01 | 0.11 | 0.25 | 9341136 | 2227543 | 9336491 | 2144341 | 9572902 | 2105754 | 9100080 | 2177608 |
| RP_POS_217_98738a0_734 | RP | POS | 0.73 | 217.9874 | | | | 4.66E-10 | 0.79 | 1.30 | 2.73E-01 | 0.24 | 0.78 | 3.53E-06 | 0.91 | 1.48 | 8.63E-06 | 0.67 | 1.35 | 66391 | 105843 | 385856 | 433949 | 338037 | 372364 | 433676 | 486974 |
| HILIC_POS_608_38519a0_621 | HILIC | POS | 0.621 | 608.3852 | | | | 4.99E-06 | 0.55 | 1.27 | 2.73E-01 | -0.25 | 0.98 | 3.00E-03 | 0.43 | 1.41 | 4.52E-04 | 0.67 | 1.48 | 283796 | 488878 | 2165207 | 3818752 | 2585986 | 4296828 | 1744427 | 3261790 |
| HILIC_NEG_134_89474a5_507 | HILIC | NEG | 5.507 | 134.8947 | | | | 3.57E-01 | -0.18 | 0.37 | 2.73E-01 | -0.23 | 0.59 | 1.80E-01 | -0.29 | 0.61 | 7.76E-01 | -0.06 | 0.11 | 13045942 | 4652863 | 12134719 | 5357519 | 12724209 | 5441938 | 11545229 | 5260030 |
| HILIC_POS_154_04987a0_742 | HILIC | POS | 0.742 | 154.0499 | | | | 5.81E-01 | -0.10 | 0.39 | 2.74E-01 | -0.23 | 0.69 | 3.22E-01 | -0.21 | 0.35 | 9.12E-01 | 0.02 | 0.21 | 476541 | 233840 | 447472 | 323033 | 483014 | 289613 | 411930 | 352670 |
| RP_NEG_1019_61267a10_324 | RP | NEG | 10.32 | 1019.6127 | | | | 6.99E-08 | 0.91 | 1.61 | 2.74E-01 | -0.22 | 0.94 | 1.57E-04 | 0.80 | 1.34 | 1.74E-08 | 1.02 | 1.90 | 2423631 | 623097 | 3285880 | 944260 | 3389819 | 750040 | 3181941 | 1103106 |
| HILIC_POS_672_45221a0_505 | HILIC | POS | 0.505 | 672.4522 | | | | 1.40E-06 | 0.60 | 1.32 | 2.74E-01 | -0.24 | 1.00 | 9.46E-04 | 0.48 | 1.51 | 2.13E-04 | 0.72 | 1.49 | 68952 | 211629 | 580788 | 936796 | 683749 | 1063524 | 477826 | 787633 |
| RP_POS_279_23181a9_786 | RP | POS | 9.79 | 279.2318 | | | | 9.77E-01 | 0.00 | 0.58 | 2.74E-01 | -0.24 | 1.01 | 5.34E-01 | -0.11 | 0.28 | 5.62E-01 | 0.12 | 0.93 | 1361888 | 489485 | 1365144 | 744657 | 1446947 | 830725 | 1283342 | 645434 |
| RP_POS_249_18524a9_207 | RP | POS | 9.21 | 249.1852 | Unknown | 249.1854(100), 193.1226(40) | 4 | 7.82E-01 | -0.06 | 0.52 | 2.74E-01 | -0.22 | 0.82 | 4.64E-01 | -0.17 | 0.40 | 8.27E-01 | 0.05 | 0.53 | 5435485 | 386150 | 5413824 | 371955 | 5454668 | 386246 | 5372979 | 356277 |
| RP_POS_313_15506a4_72 | RP | POS | 4.72 | 313.1551 | | | | 4.51E-06 | 0.54 | 1.30 | 2.75E-01 | 0.25 | 1.21 | 6.87E-04 | 0.67 | 1.37 | 1.80E-03 | 0.42 | 1.39 | 523713 | 187327 | 48808277 | 99464825 | 37883019 | 80018941 | 59733536 | 115506626 |
| HILIC_POS_232_15456a1_608 | HILIC | POS | 1.608 | 232.1546 | | | | 4.64E-01 | 0.11 | 0.41 | 2.75E-01 | -0.24 | 0.87 | 9.62E-01 | -0.01 | 0.25 | 1.97E-01 | 0.23 | 0.43 | 23363804 | 9482970 | 25157365 | 17700321 | 27100050 | 16498601 | 23214680 | 18790709 |
| RP_POS_579_4317a11_163 | RP | POS | 11.16 | 579.4317 | | | | 9.40E-01 | 0.01 | 0.71 | 2.75E-01 | 0.23 | 0.87 | 5.85E-01 | 0.13 | 0.62 | 6.03E-01 | -0.10 | 0.65 | 360587 | 112488 | 362387 | 133266 | 347748 | 101614 | 377026 | 158492 |
| RP_POS_305_24518a11_254 | RP | POS | 11.25 | 305.2452 | | | | 1.69E-04 | 0.65 | 1.43 | 2.75E-01 | 0.23 | 0.81 | 4.30E-04 | 0.76 | 1.39 | 3.50E-03 | 0.54 | 1.40 | 6039101 | 2024758 | 7857317 | 2872254 | 7542027 | 2451177 | 8172607 | 3233714 |
| RP_POS_621_39728a10_461 | RP | POS | 10.46 | 621.3973 | | | | 2.67E-01 | 0.20 | 0.53 | 2.75E-01 | 0.23 | 0.67 | 1.47E-01 | 0.32 | 0.56 | 6.62E-01 | 0.09 | 0.46 | 1293196 | 333599 | 1374022 | 416109 | 1328371 | 383549 | 1419674 | 445493 |
| RP_NEG_893_19366a9_609 | RP | NEG | 9.61 | 893.1937 | | | | 7.26E-01 | 0.06 | 0.41 | 2.75E-01 | -0.23 | 0.68 | 8.04E-01 | -0.05 | 0.21 | 3.86E-01 | 0.18 | 0.39 | 4413301 | 266351 | 4433782 | 342419 | 4471342 | 332655 | 4396221 | 351221 |
| HILIC_NEG_158_93901a4_724 | HILIC | NEG | 4.724 | 158.9390 | | | | 4.53E-03 | 0.47 | 0.92 | 2.75E-01 | 0.23 | 0.96 | 5.08E-03 | 0.59 | 0.94 | 5.18E-02 | 0.36 | 0.68 | 384086 | 104174 | 454904 | 157580 | 437617 | 140644 | 472191 | 172566 |
| HILIC_POS_150_05836a4_625 | HILIC | POS | 4.625 | 150.0584 | | | | 5.17E-01 | -0.09 | 0.63 | 2.75E-01 | 0.25 | 0.73 | 8.85E-01 | 0.03 | 0.10 | 5.35E-02 | -0.21 | 1.01 | 16788197 | 3829498 | 16041205 | 9282394 | 15020105 | 4199033 | 17062306 | 12422736 |
| RP_POS_744_58899a14_249 | RP | POS | 14.25 | 744.5890 | | | | 6.06E-01 | 0.08 | 0.30 | 2.75E-01 | 0.24 | 0.87 | 3.39E-01 | 0.20 | 0.47 | 7.77E-01 | -0.05 | 0.63 | 1211415 | 950749 | 1343410 | 1922176 | 1132588 | 1563676 | 1554233 | 2220165 |
| HILIC_NEG_369_17435a0_555 | HILIC | NEG | 0.555 | 369.1744 | | | | 7.84E-07 | 0.65 | 1.18 | 2.75E-01 | 0.24 | 0.62 | 1.69E-04 | 0.77 | 1.26 | 1.72E-04 | 0.53 | 1.27 | 162204 | 172140 | 476383 | 523124 | 419019 | 404359 | 533747 | 618612 |
| HILIC_POS_630_41815a0_563 | HILIC | POS | 0.563 | 630.4182 | | | | 1.30E-06 | 0.61 | 1.31 | 2.76E-01 | -0.24 | 0.93 | 7.21E-04 | 0.49 | 1.48 | 2.05E-04 | 0.73 | 1.48 | 84499 | 280380 | 703338 | 1111912 | 825188 | 1270958 | 581487 | 923198 |
| RP_POS_163_97667a15_627 | RP | POS | 15.63 | 163.9767 | | | | 7.32E-02 | 0.38 | 0.98 | 2.76E-01 | -0.21 | 0.79 | 2.37E-01 | 0.27 | 0.63 | 3.89E-02 | 0.49 | 1.23 | 182742344 | 70554324 | 208637375 | 66874731 | 215960302 | 68029777 | 201314449 | 65560959 |
| RP_NEG_813_53955a12_329 | RP | NEG | 12.33 | 813.5396 | | | | 3.98E-05 | -0.89 | 1.57 | 2.76E-01 | -0.20 | 0.67 | 3.62E-05 | -0.99 | 1.56 | 3.94E-04 | -0.79 | 1.56 | 2208645 | 435291 | 1817240 | 397918 | 1860807 | 361476 | 1773674 | 430546 |
| RP_POS_513_84253a15_153 | RP | POS | 15.15 | 513.8425 | | | | 9.71E-02 | 0.25 | 0.57 | 2.76E-01 | -0.24 | 0.82 | 3.83E-01 | 0.12 | 0.46 | 9.62E-02 | 0.37 | 0.66 | 143736 | 305534 | 281094 | 617905 | 348827 | 770359 | 213360 | 410597 |
| HILIC_POS_630_75275a0_563 | HILIC | POS | 0.563 | 630.7528 | | | | 1.33E-06 | 0.61 | 1.30 | 2.76E-01 | -0.24 | 0.93 | 7.08E-04 | 0.49 | 1.48 | 2.11E-04 | 0.73 | 1.47 | 87571 | 284809 | 715987 | 1130278 | 839717 | 1293855 | 592256 | 935854 |
| RP_POS_397_22189a9_004 | RP | POS | 9.00 | 397.2219 | | | | 4.32E-03 | 0.33 | 0.68 | 2.76E-01 | 0.25 | 0.75 | 3.48E-02 | 0.46 | 0.70 | 1.89E-02 | 0.21 | 0.84 | 18613 | 24123 | 1069134 | 3595727 | 674672 | 1910082 | 1463596 | 4707059 |
| RP_POS_809_4859a10_617 | RP | POS | 10.62 | 809.4859 | | | | 2.98E-02 | 0.48 | 1.19 | 2.76E-01 | -0.21 | 0.83 | 1.34E-01 | 0.37 | 0.92 | 1.07E-02 | 0.58 | 1.26 | 816969 | 388142 | 988708 | 342159 | 1026152 | 276339 | 951264 | 396623 |
| RP_POS_152_96854a0_252 | RP | POS | 0.25 | 152.9685 | | | | 2.51E-01 | 0.24 | 0.63 | 2.77E-01 | 0.22 | 0.66 | 1.26E-01 | 0.35 | 0.58 | 5.74E-01 | 0.13 | 0.39 | 12391810 | 1874662 | 12830650 | 1810489 | 12632765 | 1900171 | 13028535 | 1712329 |
| RP_POS_793_54272a12_766 | RP | POS | 12.77 | 793.5427 | | | | 1.51E-01 | -0.27 | 0.79 | 2.77E-01 | 0.23 | 0.68 | 4.71E-01 | -0.15 | 0.46 | 7.25E-02 | -0.38 | 0.82 | 1152355 | 301436 | 1058841 | 361086 | 1019377 | 352685 | 1098305 | 368593 |
| RP_POS_358_27371a10_661 | RP | POS | 10.66 | 358.2737 | | | | 2.83E-03 | 0.44 | 0.75 | 2.77E-01 | -0.24 | 0.87 | 1.93E-02 | 0.32 | 0.89 | 1.26E-02 | 0.56 | 0.82 | 201090 | 169087 | 342518 | 353816 | 381268 | 449682 | 303768 | 218283 |
| RP_POS_330_89917a15_378 | RP | POS | 15.38 | 330.8992 | | | | 7.87E-01 | 0.05 | 0.44 | 2.77E-01 | -0.22 | 0.78 | 7.85E-01 | -0.06 | 0.36 | 4.51E-01 | 0.16 | 0.41 | 1435979 | 64881 | 1439657 | 72332 | 1447558 | 71351 | 1431755 | 73160 |
| RP_POS_1039_6687a10_499 | RP | POS | 10.50 | 1039.6687 | | | | 1.16E-02 | 0.50 | 1.00 | 2.77E-01 | 0.22 | 0.67 | 7.89E-03 | 0.61 | 1.13 | 6.46E-02 | 0.39 | 0.91 | 656682 | 419869 | 883024 | 448613 | 834031 | 411684 | 932017 | 481905 |
| RP_POS_581_23883a11_804 | RP | POS | 11.80 | 581.2388 | | | | 2.70E-02 | -0.43 | 1.03 | 2.77E-01 | 0.22 | 1.26 | 1.99E-01 | -0.32 | 0.61 | 3.78E-03 | -0.54 | 1.37 | 1188999 | 803885 | 808109 | 893782 | 710267 | 465293 | 905952 | 1173865 |
| RP_POS_481_26181a4_885 | RP | POS | 4.89 | 481.2618 | Unknown | 481.2611(100) | 4 | 4.38E-01 | 0.18 | 1.21 | 2.77E-01 | -0.20 | 1.23 | 7.44E-01 | 0.08 | 0.99 | 2.79E-01 | 0.28 | 1.13 | 872391 | 2775348 | 1292657 | 2177564 | 1530416 | 2466795 | 1054897 | 1838450 |
| HILIC_POS_287_19641a3_509 | HILIC | POS | 3.509 | 287.1964 | | | | 5.02E-03 | 0.40 | 0.76 | 2.77E-01 | 0.24 | 0.79 | 4.70E-03 | 0.52 | 1.03 | 1.23E-01 | 0.28 | 0.65 | 608796 | 891956 | 1352615 | 2069173 | 1126836 | 2066022 | 1578393 | 2068297 |
| RP_POS_475_41516a15_1 | RP | POS | 15.10 | 475.4152 | | | | 6.64E-01 | -0.08 | 0.27 | 2.78E-01 | 0.23 | 0.69 | 8.59E-01 | 0.04 | 0.30 | 3.48E-01 | -0.19 | 0.51 | 126642300 | 8654067 | 125813539 | 11247358 | 124586592 | 10973776 | 127040487 | 11493120 |
| HILIC_POS_432_28073a0_616 | HILIC | POS | 0.616 | 432.2807 | | | | 9.11E-01 | -0.03 | 1.07 | 2.78E-01 | -0.17 | 0.95 | 6.85E-01 | -0.12 | 0.81 | 8.60E-01 | 0.05 | 1.03 | 2901037 | 8396778 | 2726715 | 4263452 | 3191978 | 4868243 | 2261452 | 3547314 |
| RP_POS_363_22897a10_661 | RP | POS | 10.66 | 363.2290 | | | | 2.09E-03 | 0.44 | 0.75 | 2.78E-01 | -0.24 | 0.87 | 1.88E-02 | 0.32 | 0.86 | 1.04E-02 | 0.56 | 0.83 | 206282 | 158664 | 347673 | 350747 | 385952 | 438787 | 309394 | 230290 |
| RP_POS_185_08087a6_347 | RP | POS | 6.35 | 185.0809 | | | | 1.73E-03 | 0.52 | 0.87 | 2.78E-01 | -0.23 | 0.69 | 4.28E-02 | 0.41 | 0.82 | 9.59E-04 | 0.64 | 1.12 | 267404 | 239489 | 448189 | 362633 | 487697 | 341378 | 408681 | 382054 |
| RP_POS_584_25861a11_804 | RP | POS | 11.80 | 584.2586 | | | | 2.42E-02 | -0.45 | 1.11 | 2.79E-01 | 0.22 | 1.30 | 1.70E-01 | -0.34 | 0.70 | 4.48E-03 | -0.56 | 1.30 | 11009756 | 5898109 | 8183894 | 6328078 | 7493964 | 3895386 | 8873824 | 8047416 |
| RP_POS_701_40918a13_574 | RP | POS | 13.57 | 701.4092 | | | | 3.17E-01 | -0.20 | 0.88 | 2.79E-01 | -0.22 | 0.66 | 1.86E-01 | -0.31 | 0.97 | 6.65E-01 | -0.09 | 0.46 | 12983111 | 3625547 | 12234346 | 3716764 | 12638584 | 3298976 | 11830108 | 4085812 |
| RP_POS_323_25592a10_222 | RP | POS | 10.22 | 323.2559 | | | | 4.23E-01 | 0.14 | 0.92 | 2.79E-01 | 0.23 | 0.94 | 2.19E-01 | 0.26 | 0.81 | 9.00E-01 | 0.03 | 0.83 | 18358052 | 2328659 | 18776104 | 3148017 | 18433887 | 3072224 | 19118322 | 3216318 |
| HILIC_POS_144_10199a0_55 | HILIC | POS | 0.55 | 144.1020 | | | | 9.44E-02 | -0.37 | 1.07 | 2.79E-01 | -0.21 | 0.93 | 5.33E-02 | -0.48 | 0.81 | 2.55E-01 | -0.27 | 0.56 | 1444181 | 941566 | 1128694 | 802063 | 1215879 | 763252 | 1041509 | 837659 |
| RP_POS_472_133a6_5 | RP | POS | 6.50 | 472.1330 | | | | 6.27E-02 | 0.38 | 0.72 | 2.79E-01 | -0.22 | 0.68 | 2.29E-01 | 0.27 | 0.62 | 3.08E-02 | 0.49 | 0.88 | 314682 | 227857 | 402734 | 228196 | 427534 | 222503 | 377933 | 233339 |
| RP_NEG_558_33215a10_935 | RP | NEG | 10.94 | 558.3322 | | | | 7.40E-02 | -0.41 | 1.23 | 2.79E-01 | -0.20 | 1.62 | 4.43E-02 | -0.51 | 1.14 | 2.00E-01 | -0.30 | 1.02 | 3461543 | 696853 | 3213050 | 573603 | 3275387 | 520479 | 3150713 | 621229 |
| RP_POS_148_97342a0_256 | RP | POS | 0.26 | 148.9734 | | | | 4.98E-01 | 0.12 | 0.49 | 2.80E-01 | -0.23 | 0.81 | 9.69E-01 | 0.01 | 0.27 | 2.63E-01 | 0.24 | 0.44 | 3669786 | 349662 | 3721863 | 448247 | 3770562 | 456417 | 3673164 | 439085 |
| RP_POS_405_12567a0_709 | RP | POS | 0.71 | 405.1257 | | | | 4.76E-03 | 0.36 | 0.57 | 2.80E-01 | 0.24 | 0.84 | 1.94E-02 | 0.49 | 0.77 | 6.29E-02 | 0.24 | 0.69 | 404648 | 93855 | 507449 | 317050 | 472977 | 227133 | 541920 | 386106 |
| HILIC_NEG_150_88943a2_956 | HILIC | NEG | 2.956 | 150.8894 | | | | 7.86E-01 | 0.05 | 0.63 | 2.80E-01 | -0.22 | 0.72 | 7.96E-01 | -0.06 | 0.24 | 4.71E-01 | 0.16 | 0.27 | 724259 | 296133 | 740884 | 313617 | 774931 | 327832 | 706837 | 298125 |
| RP_POS_476_30658a4_885 | RP | POS | 4.89 | 476.3066 | | | | 4.64E-01 | 0.17 | 1.19 | 2.80E-01 | -0.20 | 1.23 | 7.63E-01 | 0.07 | 0.98 | 3.00E-01 | 0.27 | 1.11 | 1532137 | 4804360 | 2215519 | 3638103 | 2610645 | 4139022 | 1820394 | 3048209 |
| HILIC_POS_152_05397a4_625 | HILIC | POS | 4.625 | 152.0540 | | | | 6.26E-01 | -0.06 | 0.52 | 2.80E-01 | 0.25 | 0.67 | 8.01E-01 | 0.06 | 0.19 | 3.33E-02 | -0.19 | 1.19 | 680362 | 127403 | 659375 | 365495 | 619539 | 116832 | 699210 | 503002 |
| RP_POS_660_37354a9_783 | RP | POS | 9.78 | 660.3735 | | | | 8.77E-05 | 0.51 | 1.13 | 2.80E-01 | 0.24 | 0.68 | 1.47E-03 | 0.63 | 1.20 | 8.99E-03 | 0.39 | 0.88 | 454675 | 489587 | 1215908 | 1668538 | 1034781 | 1395378 | 1397036 | 1900215 |
| RP_POS_952_15094a0_485 | RP | POS | 0.49 | 952.1509 | | | | 3.31E-08 | 0.68 | 1.22 | 2.80E-01 | -0.24 | 1.08 | 7.09E-04 | 0.56 | 1.16 | 8.16E-06 | 0.80 | 1.46 | 164552 | 48361 | 347368 | 296151 | 379486 | 301508 | 315249 | 290153 |
| RP_NEG_246_87759a15_577 | RP | NEG | 15.58 | 246.8776 | | | | 1.14E-01 | -0.35 | 0.81 | 2.81E-01 | -0.21 | 0.86 | 6.06E-02 | -0.46 | 0.76 | 3.01E-01 | -0.25 | 0.79 | 1107433 | 48825 | 1091973 | 41781 | 1096502 | 41871 | 1087444 | 41617 |
| RP_POS_1070_16028a10_617 | RP | POS | 10.62 | 1070.1603 | | | | 5.38E-03 | 0.58 | 1.28 | 2.81E-01 | -0.21 | 0.91 | 4.27E-02 | 0.47 | 0.99 | 2.14E-03 | 0.68 | 1.37 | 597416 | 372235 | 816353 | 366852 | 856119 | 329761 | 776587 | 399922 |
| HILIC_NEG_413_20108a0_612 | HILIC | NEG | 0.612 | 413.2011 | | | | 2.13E-01 | 0.23 | 0.42 | 2.81E-01 | -0.22 | 0.67 | 5.52E-01 | 0.12 | 0.23 | 1.22E-01 | 0.35 | 0.65 | 119738 | 73448 | 139379 | 86811 | 148786 | 93754 | 129971 | 79094 |
| RP_NEG_468_89688a0_497 | RP | NEG | 0.50 | 468.8969 | | | | 2.23E-09 | -0.74 | 1.37 | 2.81E-01 | 0.24 | 1.23 | 1.44E-04 | -0.62 | 1.37 | 2.63E-06 | -0.85 | 1.54 | 27259313 | 1164110 | 22644310 | 6803539 | 21907224 | 7046197 | 23381395 | 6539364 |

**Supplementary table 1: Metabolomics results** (in asceding order according to p-value from progressive ALD vs non-progressive ALD comparison, with molecular features with ID_level of 1-2 first)

| | | | | | | | | all_ALD_vs_control | | | Prog_ALD_vs_non-prog_ALD | | | Prog_ALD_vs_control_ALD | | | Non-prog_ALD_vs_control | | | Healthy_control | | all_ALD | | Non-prog_ALD | | Progressive_ALD | |
|---|---|---|---|---|---|---|---|---|---|---|---|---|---|---|---|---|---|---|---|---|---|---|---|---|---|---|---|
| | | | | | | | | t-test | Cohen's | PLS-DA | t-test | Cohen's | PLS-DA | t-test | Cohen's | PLS-DA | t-test | Cohen's | PLS-DA | | | | | | | | |
| Feature_ID | RP_HILIC | POS_NEG | Average.Rt | Average.Mz | Metabolite.name | curated_MSMS | ID_level | p | d | VIP | p | d | VIP | p | d | VIP | p | d | VIP | Mean | SD | Mean | SD | Mean | SD | Mean | SD |
| HILIC_NEG_132_92361a5_059 | HILIC | NEG | 5.059 | 132.9236 | | | | 1.86E-01 | 0.27 | 0.95 | 2.81E-01 | 0.22 | 1.16 | 9.42E-02 | 0.38 | 0.80 | 4.77E-01 | 0.16 | 0.39 | 4431598 | 1946750 | 4962663 | 1958205 | 4750542 | 2001079 | 5174784 | 1910822 |
| RP_NEG_189_0022a4_572 | RP | NEG | 4.57 | 189.0022 | | | | 7.81E-03 | -0.57 | 0.90 | 2.81E-01 | -0.21 | 0.73 | 6.16E-03 | -0.67 | 1.00 | 3.44E-02 | -0.46 | 0.81 | 14792443 | 10018793 | 9233360 | 9397618 | 10251207 | 7890881 | 8215513 | 10679535 |
| RP_POS_328_23019a10_649 | RP | POS | 10.65 | 328.2302 | | | | 5.42E-01 | -0.08 | 0.48 | 2.82E-01 | 0.24 | 0.67 | 8.57E-01 | 0.04 | 0.40 | 5.94E-02 | -0.20 | 0.74 | 3793398 | 375275 | 3718305 | 1032181 | 3606330 | 507999 | 3830280 | 1367072 |
| RP_POS_199_01088a15_388 | RP | POS | 15.39 | 199.0109 | | | | 1.80E-01 | -0.31 | 1.05 | 2.82E-01 | 0.20 | 0.82 | 3.93E-01 | -0.21 | 0.65 | 1.05E-01 | -0.41 | 0.89 | 13579272 | 5564387 | 12107891 | 4456268 | 11625975 | 4698044 | 12589807 | 4192415 |
| RP_POS_541_32935a10_617 | RP | POS | 10.62 | 541.3294 | | | | 3.99E-02 | 0.48 | 1.24 | 2.82E-01 | -0.20 | 1.06 | 1.41E-01 | 0.38 | 0.95 | 1.63E-02 | 0.58 | 1.29 | 644336 | 232351 | 739416 | 181161 | 759005 | 146779 | 719827 | 209717 |
| RP_POS_497_39636a14_164 | RP | POS | 14.16 | 497.3964 | | | | 3.89E-01 | -0.15 | 0.50 | 2.82E-01 | 0.23 | 0.66 | 8.47E-01 | -0.04 | 0.48 | 1.89E-01 | -0.27 | 0.50 | 5858589 | 2063556 | 5466880 | 2668249 | 5178546 | 2555655 | 5755215 | 2771888 |
| RP_POS_299_87692a15_557 | RP | POS | 15.56 | 299.8769 | | | | 6.27E-01 | 0.08 | 0.67 | 2.83E-01 | 0.23 | 0.87 | 3.34E-01 | 0.20 | 0.49 | 8.81E-01 | -0.03 | 0.79 | 5604776 | 189287 | 5625450 | 260528 | 5597338 | 256329 | 5653561 | 264231 |
| RP_POS_1056_72791a0_494 | RP | POS | 0.49 | 1056.7279 | | | | 4.06E-03 | -0.61 | 1.18 | 2.83E-01 | 0.21 | 0.65 | 2.69E-02 | -0.51 | 1.07 | 2.26E-03 | -0.71 | 1.16 | 696197 | 91324 | 641021 | 86547 | 631683 | 86653 | 650358 | 86295 |
| RP_POS_153_0405a0_71 | RP | POS | 0.71 | 153.0405 | | | | 9.77E-05 | 0.46 | 1.07 | 2.83E-01 | 0.24 | 0.96 | 2.60E-03 | 0.58 | 1.29 | 1.38E-02 | 0.34 | 0.98 | 466901 | 137646 | 2147184 | 4131245 | 1701272 | 3414223 | 2593095 | 4734973 |
| HILIC_NEG_134_89481a6_094 | HILIC | NEG | 6.094 | 134.8948 | | | | 8.65E-01 | -0.03 | 0.51 | 2.83E-01 | -0.22 | 0.79 | 4.91E-01 | -0.14 | 0.46 | 7.37E-01 | 0.08 | 0.65 | 16411299 | 8231395 | 16118769 | 9071617 | 17097525 | 10093399 | 15140012 | 7901659 |
| RP_NEG_241_88274a15_787 | RP | NEG | 15.79 | 241.8827 | | | | 5.73E-01 | -0.13 | 0.32 | 2.83E-01 | -0.20 | 0.72 | 3.62E-01 | -0.23 | 0.45 | 9.15E-01 | -0.03 | 0.23 | 4413221 | 2229062 | 4166507 | 1831506 | 4364074 | 1657849 | 3968941 | 1987251 |
| RP_POS_338_2326a9_441 | RP | POS | 9.44 | 338.2326 | | | | 4.68E-01 | -0.14 | 0.32 | 2.83E-01 | -0.22 | 1.00 | 2.77E-01 | -0.25 | 0.47 | 8.81E-01 | -0.03 | 0.32 | 5424028 | 355954 | 5369852 | 391804 | 5412104 | 341563 | 5327599 | 435712 |
| RP_NEG_992_73187a0_497 | RP | NEG | 0.50 | 992.7319 | | | | 9.80E-01 | 0.00 | 0.80 | 2.84E-01 | -0.22 | 1.16 | 5.94E-01 | -0.11 | 0.52 | 6.44E-01 | 0.11 | 0.85 | 1635281 | 291125 | 1633776 | 315052 | 1667715 | 334209 | 1599836 | 294099 |
| RP_POS_546_3537a10_934 | RP | POS | 10.93 | 546.3537 | | | | 3.41E-01 | -0.22 | 0.88 | 2.84E-01 | -0.20 | 1.35 | 2.03E-01 | -0.32 | 0.67 | 6.32E-01 | -0.12 | 0.78 | 124594877 | 29488688 | 119085380 | 23740978 | 121642029 | 22891328 | 116528732 | 24523830 |
| RP_POS_436_37802a12_243 | RP | POS | 12.24 | 436.3780 | | | | 6.82E-01 | -0.07 | 0.36 | 2.84E-01 | 0.23 | 0.69 | 8.49E-01 | 0.04 | 0.29 | 3.92E-01 | -0.19 | 0.57 | 1894481 | 179252 | 1878464 | 225024 | 1854228 | 243617 | 1902700 | 204345 |
| RP_POS_177_99225a15_425 | RP | POS | 15.43 | 177.9923 | | | | 7.50E-01 | -0.07 | 0.53 | 2.84E-01 | 0.21 | 0.63 | 8.97E-01 | 0.03 | 0.29 | 4.80E-01 | -0.17 | 0.61 | 8404975 | 987683 | 8343104 | 826835 | 8254116 | 852275 | 8432092 | 799207 |
| HILIC_POS_388_25433a0_616 | HILIC | POS | 0.616 | 388.2543 | | | | 7.08E-01 | -0.11 | 1.05 | 2.84E-01 | -0.15 | 0.93 | 5.41E-01 | -0.19 | 0.78 | 9.01E-01 | -0.04 | 0.97 | 4040543 | 12094460 | 3212323 | 5027059 | 3753533 | 5748979 | 2671113 | 4172511 |
| RP_NEG_311_14078a4_717 | RP | NEG | 4.72 | 311.1408 | | | | 4.56E-06 | 0.54 | 1.29 | 2.84E-01 | 0.24 | 1.20 | 7.13E-04 | 0.66 | 1.37 | 1.75E-03 | 0.42 | 1.38 | 71070 | 27630 | 7048569 | 14380514 | 5500865 | 11597653 | 8596273 | 16686062 |
| RP_POS_154_99043a0_761 | RP | POS | 0.76 | 154.9904 | | | | 7.12E-01 | -0.07 | 0.67 | 2.84E-01 | 0.23 | 0.74 | 8.38E-01 | 0.04 | 0.36 | 3.79E-01 | -0.18 | 0.89 | 1453946 | 623404 | 1404107 | 767578 | 1321557 | 713431 | 1486658 | 816998 |
| RP_NEG_416_62344a15_584 | RP | NEG | 15.58 | 416.6234 | | | | 7.69E-01 | 0.06 | 0.90 | 2.85E-01 | -0.22 | 0.64 | 8.32E-01 | -0.05 | 0.63 | 4.70E-01 | 0.17 | 0.64 | 764478 | 44643 | 767149 | 44532 | 771937 | 46329 | 762360 | 42585 |
| RP_POS_315_25241a10_628 | RP | POS | 10.63 | 315.2524 | | | | 2.97E-04 | -0.76 | 1.49 | 2.85E-01 | 0.20 | 0.90 | 5.27E-03 | -0.66 | 1.22 | 8.94E-05 | -0.86 | 1.60 | 6568168 | 676228 | 6039467 | 649900 | 5969556 | 536922 | 6109378 | 745051 |
| RP_POS_350_2121a10_65 | RP | POS | 10.65 | 350.2121 | | | | 4.30E-01 | -0.12 | 0.64 | 2.85E-01 | 0.24 | 0.69 | 9.98E-01 | 0.00 | 0.51 | 7.03E-02 | -0.24 | 0.86 | 10293450 | 1610146 | 9959081 | 3118172 | 9622888 | 1609730 | 10295274 | 4101527 |
| HILIC_NEG_176_83629a3_029 | HILIC | NEG | 3.029 | 176.8363 | | | | 7.15E-01 | -0.06 | 0.90 | 2.85E-01 | -0.23 | 0.93 | 3.55E-01 | -0.18 | 0.42 | 7.95E-01 | 0.05 | 0.22 | 733473 | 84896 | 726297 | 125632 | 739791 | 134439 | 712803 | 115945 |
| HILIC_NEG_162_83916a2_962 | HILIC | NEG | 2.962 | 162.8392 | | | | 3.02E-01 | 0.20 | 0.91 | 2.85E-01 | 0.22 | 1.31 | 1.90E-01 | 0.31 | 0.48 | 6.55E-01 | 0.09 | 0.76 | 40219423 | 14847426 | 43453346 | 16633875 | 41665674 | 13244773 | 45241017 | 19418348 |
| RP_POS_1065_45972a0_484 | RP | POS | 0.48 | 1065.4597 | | | | 5.87E-07 | 0.60 | 1.18 | 2.85E-01 | -0.24 | 1.05 | 3.37E-03 | 0.48 | 1.07 | 4.12E-05 | 0.72 | 1.43 | 135135 | 20116 | 246986 | 207112 | 269223 | 209515 | 224750 | 204354 |
| HILIC_NEG_136_89172a7_391 | HILIC | NEG | 7.391 | 136.8917 | | | | 6.73E-01 | -0.09 | 0.27 | 2.86E-01 | 0.21 | 1.04 | 9.34E-01 | 0.02 | 0.04 | 3.88E-01 | -0.19 | 0.44 | 3552603 | 173237 | 3537745 | 169090 | 3519603 | 158325 | 3555888 | 178962 |
| HILIC_POS_145_0972a0_825 | HILIC | POS | 0.825 | 145.0972 | | | | 2.15E-01 | 0.15 | 0.41 | 2.86E-01 | 0.25 | 0.97 | 2.40E-01 | 0.27 | 0.36 | 5.92E-01 | 0.02 | 0.49 | 391085 | 143952 | 506338 | 888902 | 410504 | 181285 | 602172 | 1242904 |
| RP_POS_616_25061a7_158 | RP | POS | 7.16 | 616.2506 | | | | 6.55E-01 | -0.08 | 0.81 | 2.86E-01 | 0.23 | 1.15 | 8.91E-01 | 0.03 | 0.42 | 3.09E-01 | -0.19 | 0.88 | 613866 | 274340 | 587193 | 344077 | 550292 | 273458 | 624094 | 402039 |
| RP_POS_786_50458a12_951 | RP | POS | 12.95 | 786.5046 | | | | 9.72E-01 | 0.01 | 0.62 | 2.86E-01 | 0.22 | 0.65 | 6.32E-01 | 0.12 | 0.53 | 5.90E-01 | -0.10 | 0.32 | 1477602 | 928505 | 1484383 | 1033914 | 1373477 | 702794 | 1595289 | 1280918 |
| RP_POS_192_00853a15_423 | RP | POS | 15.42 | 192.0085 | | | | 4.69E-01 | -0.16 | 0.75 | 2.87E-01 | -0.21 | 0.78 | 2.81E-01 | -0.26 | 0.53 | 8.16E-01 | -0.05 | 0.57 | 67503044 | 12583349 | 65686633 | 11111095 | 66876122 | 10578877 | 64497144 | 11603113 |
| RP_NEG_276_8754a15_502 | RP | NEG | 15.50 | 276.8754 | | | | 6.24E-01 | -0.10 | 0.61 | 2.87E-01 | -0.21 | 0.67 | 3.60E-01 | -0.21 | 0.47 | 9.89E-01 | 0.00 | 0.43 | 1902240 | 58616 | 1896434 | 55990 | 1902427 | 58623 | 1890441 | 53140 |
| RP_POS_457_27142a11_836 | RP | POS | 11.84 | 457.2714 | | | | 8.39E-01 | 0.04 | 0.52 | 2.87E-01 | -0.21 | 0.73 | 7.82E-01 | -0.06 | 0.60 | 5.53E-01 | 0.15 | 0.66 | 22984509 | 15273211 | 23604806 | 13911694 | 25093381 | 16184894 | 22116232 | 11160072 |
| RP_POS_303_09714a7_093 | RP | POS | 7.09 | 303.0971 | | | | 8.62E-01 | 0.03 | 0.54 | 2.87E-01 | 0.22 | 0.80 | 5.00E-01 | 0.14 | 0.55 | 7.23E-01 | -0.08 | 0.58 | 2089724 | 130317 | 2094524 | 150970 | 2078386 | 155823 | 2110662 | 145729 |
| HILIC_POS_218_09216a4_125 | HILIC | POS | 4.125 | 218.0922 | | | | 4.71E-02 | -0.34 | 1.05 | 2.87E-01 | -0.23 | 1.35 | 3.32E-02 | -0.45 | 1.04 | 2.33E-01 | -0.23 | 0.97 | 1182625 | 288583 | 1050010 | 414170 | 1094286 | 373627 | 1005734 | 450529 |
| HILIC_POS_601_40167a0_553 | HILIC | POS | 0.553 | 601.4017 | | | | 6.85E-07 | 0.60 | 1.32 | 2.88E-01 | -0.24 | 0.94 | 1.00E-03 | 0.48 | 1.50 | 1.68E-04 | 0.72 | 1.53 | 71213 | 140532 | 650083 | 1069240 | 764325 | 1193838 | 535840 | 926281 |
| RP_POS_558_5083a13_618 | RP | POS | 13.62 | 558.5083 | | | | 7.61E-01 | 0.04 | 0.41 | 2.88E-01 | 0.24 | 0.72 | 4.74E-01 | 0.16 | 0.37 | 4.69E-01 | -0.08 | 0.38 | 786552 | 90896 | 795270 | 236429 | 769955 | 114607 | 820584 | 313839 |
| HILIC_NEG_183_93417a0_933 | HILIC | NEG | 0.933 | 183.9342 | | | | 5.80E-01 | 0.11 | 0.98 | 2.88E-01 | -0.22 | 0.90 | 9.98E-01 | 0.00 | 0.20 | 3.33E-01 | 0.22 | 0.44 | 2036152 | 370407 | 2079012 | 403894 | 2122109 | 417010 | 2035915 | 389747 |
| RP_POS_231_14429a0_553 | RP | POS | 0.55 | 231.1443 | | | | 6.55E-01 | 0.08 | 0.29 | 2.88E-01 | 0.22 | 0.66 | 3.73E-01 | 0.19 | 0.65 | 8.94E-01 | -0.03 | 0.45 | 3219607 | 657719 | 3282771 | 789270 | 3198559 | 751694 | 3366983 | 824069 |
| HILIC_NEG_129_01941a1_95 | HILIC | NEG | 1.95 | 129.0194 | | | | 9.73E-03 | 0.31 | 0.75 | 2.88E-01 | 0.24 | 0.94 | 5.77E-02 | 0.43 | 0.58 | 5.59E-03 | 0.19 | 0.88 | 230584 | 97889 | 423893 | 714021 | 347423 | 261372 | 500363 | 974580 |
| RP_NEG_824_80914a0_493 | RP | NEG | 0.49 | 824.8091 | | | | 1.35E-09 | -0.74 | 1.39 | 2.88E-01 | 0.23 | 1.24 | 1.28E-04 | -0.63 | 1.40 | 1.96E-06 | -0.86 | 1.57 | 7104532 | 405429 | 5268768 | 2682094 | 4982679 | 2756956 | 5554857 | 2601198 |
| HILIC_POS_174_14867a0_728 | HILIC | POS | 0.728 | 174.1487 | | | | 7.21E-06 | -0.73 | 1.21 | 2.89E-01 | -0.22 | 1.10 | 1.99E-05 | -0.84 | 1.47 | 1.20E-03 | -0.62 | 1.13 | 908916 | 88416 | 807122 | 144996 | 822584 | 144220 | 791660 | 145565 |
| RP_NEG_510_25348a10_52 | RP | NEG | 10.52 | 510.2535 | | | | 1.35E-02 | 0.32 | 0.68 | 2.89E-01 | 0.24 | 0.67 | 1.28E-02 | 0.44 | 0.79 | 2.46E-01 | 0.20 | 1.01 | 380733 | 670756 | 1033265 | 2318918 | 786020 | 2299567 | 1280510 | 2334896 |
| RP_POS_309_90543a15_528 | RP | POS | 15.53 | 309.9054 | | | | 1.15E-01 | -0.34 | 0.68 | 2.89E-01 | 0.21 | 0.91 | 2.96E-01 | -0.24 | 0.63 | 7.10E-02 | -0.44 | 0.69 | 2028364 | 262133 | 1944587 | 240104 | 1918986 | 265245 | 1970188 | 211623 |
| RP_POS_582_29559a10_306 | RP | POS | 10.31 | 582.2956 | | | | 9.43E-02 | 0.35 | 0.91 | 2.89E-01 | -0.21 | 1.17 | 3.04E-01 | 0.25 | 0.61 | 4.12E-02 | 0.45 | 0.99 | 515598 | 221630 | 591703 | 213842 | 614500 | 188429 | 568907 | 236270 |
| RP_NEG_187_00662a4_573 | RP | NEG | 4.57 | 187.0066 | | | | 7.42E-03 | -0.57 | 0.91 | 2.89E-01 | -0.20 | 0.72 | 6.03E-03 | -0.67 | 1.00 | 3.20E-02 | -0.47 | 0.82 | 286359818 | 195825419 | 177033456 | 182936609 | 196538117 | 152867067 | 157528795 | 208494244 |
| RP_POS_231_15944a6_819 | RP | POS | 6.82 | 231.1594 | | | | 1.87E-01 | 0.28 | 0.61 | 2.89E-01 | 0.21 | 0.69 | 1.11E-01 | 0.38 | 0.67 | 4.37E-01 | 0.17 | 0.50 | 990399 | 195624 | 1042740 | 183775 | 1023162 | 167348 | 1062318 | 198616 |
| RP_POS_129_05473a0_615 | RP | POS | 0.62 | 129.0547 | | | | 1.03E-01 | 0.32 | 0.62 | 2.89E-01 | -0.22 | 0.92 | 3.58E-01 | 0.21 | 0.57 | 4.27E-02 | 0.43 | 0.79 | 1999115 | 508243 | 2174063 | 555940 | 2233288 | 487815 | 2114837 | 615894 |
| RP_POS_305_15695a3_646 | RP | POS | 3.65 | 305.1570 | | | | 5.53E-01 | 0.14 | 1.11 | 2.89E-01 | -0.20 | 1.20 | 8.65E-01 | 0.04 | 0.96 | 3.66E-01 | 0.24 | 1.01 | 758111 | 1925593 | 979575 | 1464392 | 1135592 | 1671618 | 823557 | 1220109 |
| RP_POS_575_41132a10_66 | RP | POS | 10.66 | 575.4113 | | | | 8.39E-01 | -0.05 | 0.34 | 2.89E-01 | 0.20 | 0.63 | 8.18E-01 | 0.06 | 0.24 | 5.58E-01 | -0.15 | 0.29 | 24419619 | 1764021 | 24349186 | 1452315 | 24194532 | 1560760 | 24503839 | 1332929 |
| HILIC_POS_652_41119a0_623 | HILIC | POS | 0.623 | 652.4112 | | | | 1.65E-06 | 0.57 | 1.27 | 2.89E-01 | -0.24 | 0.97 | 1.78E-03 | 0.45 | 1.43 | 2.89E-04 | 0.69 | 1.48 | 118176 | 148098 | 1598015 | 2893457 | 1906189 | 3234774 | 1289841 | 2501492 |
| HILIC_POS_665_24457a0_562 | HILIC | POS | 0.562 | 665.2446 | | | | 1.16E-02 | -0.42 | 0.93 | 2.89E-01 | 0.23 | 0.79 | 1.69E-01 | -0.31 | 0.46 | 1.49E-03 | -0.54 | 1.24 | 394082 | 179947 | 286217 | 270203 | 257407 | 185982 | 315026 | 333506 |
| RP_POS_499_82712a6_958 | RP | POS | 6.96 | 499.8271 | | | | 2.45E-01 | 0.22 | 0.48 | 2.90E-01 | -0.22 | 0.69 | 6.05E-01 | 0.10 | 0.31 | 1.43E-01 | 0.33 | 0.52 | 1489032 | 2879808 | 2213982 | 3505467 | 2587115 | 3822965 | 1840849 | 3150902 |
| RP_NEG_243_19698a9_919 | RP | NEG | 9.92 | 243.1970 | | | | 8.14E-05 | 0.63 | 1.56 | 2.90E-01 | 0.22 | 1.20 | 1.39E-04 | 0.75 | 1.53 | 6.06E-03 | 0.52 | 1.29 | 1762540 | 517509 | 2280772 | 857569 | 2189514 | 853318 | 2372029 | 860645 |
| RP_NEG_915_12726a9_373 | RP | NEG | 9.37 | 915.1273 | | | | 7.73E-01 | -0.05 | 0.22 | 2.90E-01 | -0.23 | 0.76 | 4.31E-01 | -0.16 | 0.35 | 7.63E-01 | 0.06 | 0.32 | 3098493 | 200305 | 3085641 | 266566 | 3114007 | 262218 | 3057275 | 270490 |
| RP_POS_166_98474a0_253 | RP | POS | 0.25 | 166.9847 | | | | 5.05E-01 | 0.14 | 0.51 | 2.90E-01 | -0.21 | 0.80 | 8.84E-01 | 0.03 | 0.18 | 2.86E-01 | 0.25 | 0.59 | 4109341 | 750370 | 4210755 | 721258 | 4287476 | 700726 | 4134034 | 740279 |
| RP_POS_609_48181a11_254 | RP | POS | 11.25 | 609.4818 | | | | 9.83E-05 | 0.64 | 1.61 | 2.90E-01 | 0.22 | 0.98 | 3.12E-04 | 0.75 | 1.52 | 3.75E-03 | 0.53 | 1.53 | 443656 | 273154 | 709986 | 437213 | 663503 | 392852 | 756469 | 476938 |
| HILIC_POS_472_2753a0_622 | HILIC | POS | 0.622 | 472.2753 | | | | 8.21E-06 | 0.53 | 1.19 | 2.91E-01 | -0.24 | 0.99 | 3.84E-03 | 0.42 | 1.34 | 6.30E-04 | 0.65 | 1.37 | 73643 | 155534 | 742674 | 1400195 | 891388 | 1573998 | 593961 | 1199375 |
| RP_POS_643_52637a11_609 | RP | POS | 11.61 | 643.5264 | | | | 9.52E-01 | 0.01 | 0.31 | 2.91E-01 | -0.23 | 0.74 | 5.34E-01 | -0.10 | 0.50 | 5.83E-01 | 0.12 | 0.52 | 1277626 | 1724728 | 1301699 | 2576173 | 1575448 | 3150327 | 1027950 | 1825180 |
| RP_POS_497_34442a11_439 | RP | POS | 11.44 | 497.3444 | | | | 9.77E-01 | 0.00 | 0.50 | 2.91E-01 | -0.24 | 0.87 | 2.76E-01 | -0.11 | 0.53 | 6.05E-01 | 0.12 | 0.54 | 883918 | 1522696 | 895941 | 3257124 | 1243099 | 4493942 | 548784 | 996370 |
| RP_NEG_746_51477a13_263 | RP | NEG | 13.26 | 746.5148 | | | | 3.97E-01 | -0.14 | 0.86 | 2.91E-01 | 0.23 | 0.67 | 9.23E-01 | -0.02 | 0.57 | 7.34E-02 | -0.25 | 0.77 | 1960546 | 1108378 | 1736504 | 1752908 | 1549872 | 776853 | 1923135 | 2352339 |
| RP_NEG_434_8743a15_35 | RP | NEG | 15.35 | 434.8743 | | | | 8.86E-01 | -0.03 | 0.80 | 2.91E-01 | -0.22 | 0.66 | 5.37E-01 | -0.14 | 0.59 | 7.27E-01 | 0.08 | 0.42 | 1263246 | 63644 | 1261360 | 67154 | 1268482 | 69276 | 1254238 | 64874 |
| RP_POS_825_03937a13_059 | RP | POS | 13.06 | 825.0394 | | | | 6.32E-01 | -0.11 | 0.56 | 2.92E-01 | -0.20 | 0.66 | 4.07E-01 | -0.21 | 0.65 | 9.77E-01 | -0.01 | 0.22 | 745284 | 314020 | 715656 | 261843 | 743403 | 234771 | 687908 | 286067 |
| HILIC_NEG_144_92349a7_354 | HILIC | NEG | 7.354 | 144.9235 | | | | 1.78E-01 | 0.24 | 0.46 | 2.92E-01 | -0.22 | 0.95 | 5.36E-01 | 0.13 | 0.42 | 8.46E-02 | 0.35 | 0.61 | 65578647 | 6796807 | 67607538 | 8808803 | 68540382 | 8453347 | 66674694 | 9139946 |
| RP_POS_346_27386a10_907 | RP | POS | 10.91 | 346.2739 | | | | 3.28E-02 | 0.30 | 1.42 | 2.92E-01 | 0.23 | 0.92 | 3.94E-02 | 0.42 | 1.12 | 2.28E-01 | 0.19 | 1.21 | 4359728 | 1855413 | 5503915 | 4157212 | 5063530 | 3379899 | 5944299 | 4806040 |
| RP_NEG_399_18478a11_611 | RP | NEG | 11.61 | 399.1848 | | | | 1.94E-01 | 0.29 | 0.69 | 2.92E-01 | 0.20 | 1.03 | 1.09E-01 | 0.39 | 0.79 | 4.31E-01 | 0.19 | 0.47 | 4431094 | 909857 | 4666183 | 768996 | 4584772 | 765939 | 4747593 | 771096 |
| HILIC_POS_524_37213a1_213 | HILIC | POS | 1.213 | 524.3721 | | | | 4.15E-01 | -0.18 | 0.83 | 2.92E-01 | -0.21 | 1.00 | 2.39E-01 | -0.28 | 0.57 | 7.57E-01 | -0.07 | 0.20 | 96686972 | 39647625 | 90224717 | 35647973 | 93998555 | 35825626 | 86450880 | 35425269 |
| HILIC_POS_60_05658a2_199 | HILIC | POS | 2.199 | 60.0566 | | | | 1.56E-01 | 0.23 | 0.53 | 2.92E-01 | 0.23 | 0.74 | 1.14E-01 | 0.35 | 0.66 | 4.93E-01 | 0.12 | 0.26 | 2728454 | 1737552 | 3321631 | 2776460 | 3027610 | 2171158 | 3615653 | 3268704 |
| RP_NEG_307_85614a15_653 | RP | NEG | 15.65 | 307.8561 | | | | 8.66E-01 | 0.05 | 0.34 | 2.92E-01 | -0.17 | 0.76 | 8.92E-01 | -0.04 | 0.20 | 6.53E-01 | 0.13 | 0.59 | 13118936 | 2060301 | 13183692 | 1113887 | 13301585 | 1231833 | 13065800 | 980322 |
| RP_NEG_226_0179a5_127 | RP | NEG | 5.13 | 226.0179 | | | | 2.66E-02 | -0.56 | 0.86 | 2.92E-01 | -0.18 | 0.73 | 8.56E-03 | -0.65 | 1.26 | 9.99E-02 | -0.47 | 0.62 | 2229501 | 3466674 | 720138 | 2328659 | 967327 | 3125082 | 472949 | 1032056 |
| RP_NEG_654_31287a10_935 | RP | NEG | 10.94 | 654.3129 | | | | 6.43E-02 | -0.46 | 1.17 | 2.93E-01 | -0.19 | 1.72 | 4.12E-02 | -0.55 | 1.11 | 1.51E-01 | -0.36 | 0.97 | 1531919 | 345533 | 1407077 | 241312 | 1432607 | 210385 | 1381548 | 268439 |
| RP_POS_485_81046a0_249 | RP | POS | 0.25 | 485.8105 | | | | 5.15E-01 | 0.13 | 0.54 | 2.93E-01 | -0.21 | 0.84 | 9.16E-01 | 0.02 | 0.33 | 2.98E-01 | 0.24 | 0.50 | 426420 | 86352 | 438058 | 90455 | 447623 | 94007 | 428492 | 86643 |
| RP_NEG_514_28558a11_611 | RP | NEG | 11.61 | 514.2856 | Taurine conjugated sterol (BA 24:1;O4;T) | 514.285(100) | 3 | 9.41E-01 | -0.01 | 0.64 | 2.93E-01 | 0.24 | 1.06 | 6.29E-01 | 0.11 | 0.36 | 1.25E-01 | -0.13 | 0.70 | 71490334 | 52454357 | 70068203 | 168510691 | 52177520 | 58784236 | 87958886 | 230786501 |
| RP_NEG_426_17337a11_254 | RP | NEG | 11.25 | 426.1734 | | | | 4.95E-07 | 0.92 | 1.80 | 2.93E-01 | 0.21 | 1.15 | 2.61E-06 | 1.02 | 1.77 | 2.44E-05 | 0.81 | 1.71 | 968163 | 358246 | 1404778 | 462860 | 1355852 | 411055 | 1453704 | 508920 |
| RP_NEG_309_17471a9_728 | RP | NEG | 9.73 | 309.1747 | | | | 5.27E-01 | 0.08 | 0.37 | 2.93E-01 | -0.24 | 0.66 | 3.97E-01 | -0.04 | 0.45 | 3.92E-01 | 0.20 | 0.37 | 92722 | 82454 | 120713 | 416521 | 164945 | 582206 | 76482 | 86975 |
| RP_POS_120_97947a15_395 | RP | POS | 15.40 | 120.9795 | | | | 1.58E-01 | -0.34 | 0.70 | 2.93E-01 | -0.19 | 0.67 | 9.54E-02 | -0.43 | 0.69 | 3.36E-01 | -0.24 | 0.81 | 18656339 | 9120721 | 16136524 | 6860028 | 16860725 | 6417416 | 15412323 | 7268204 |
| HILIC_POS_164_07384a0_686 | HILIC | POS | 0.686 | 164.0738 | | | | 4.92E-02 | -0.54 | 1.07 | 2.94E-01 | -0.17 | 0.82 | 2.89E-02 | -0.62 | 1.00 | 1.09E-01 | -0.45 | 1.04 | 569518 | 477142 | 389758 | 264409 | 417665 | 271930 | 361852 | 256350 |
| RP_POS_317_18521a11_161 | RP | POS | 11.16 | 317.1852 | | | | 2.79E-01 | 0.19 | 1.00 | 2.94E-01 | 0.22 | 0.96 | 1.66E-01 | 0.30 | 0.91 | 6.86E-01 | 0.08 | 1.02 | 1286867 | 293606 | 1358415 | 400881 | 1316111 | 353020 | 1400719 | 443188 |
| RP_NEG_391_177a8_105 | RP | NEG | 8.11 | 391.1770 | | | | 1.79E-07 | 0.67 | 1.09 | 2.94E-01 | 0.23 | 0.86 | 8.93E-05 | 0.79 | 1.20 | 1.13E-04 | 0.56 | 1.22 | 802057 | 624926 | 2163460 | 2205205 | 1930703 | 1779792 | 2396218 | 2558687 |
| RP_POS_1038_7074a0_497 | RP | POS | 0.50 | 1038.7074 | | | | 7.28E-01 | -0.07 | 0.76 | 2.94E-01 | -0.21 | 1.14 | 4.45E-01 | -0.18 | 0.54 | 8.87E-01 | 0.03 | 0.72 | 950384 | 226534 | 934404 | 219974 | 957598 | 216884 | 911209 | 222774 |
| RP_POS_285_24225a9_662 | RP | POS | 9.66 | 285.2423 | | | | 2.34E-04 | 0.47 | 0.87 | 2.94E-01 | -0.23 | 0.70 | 7.76E-03 | 0.35 | 0.92 | 4.19E-03 | 0.59 | 1.03 | 1980557 | 240178 | 2335260 | 835369 | 2423427 | 1006019 | 2247093 | 618051 |
| RP_POS_283_17508a3_646 | RP | POS | 3.65 | 283.1751 | | | | 6.48E-01 | 0.11 | 1.11 | 2.94E-01 | -0.19 | 1.19 | 9.52E-01 | 0.01 | 0.94 | 4.41E-01 | 0.21 | 1.01 | 731663 | 1838059 | 893308 | 1328400 | 1033335 | 1515057 | 753282 | 1109005 |
| RP_POS_168_97989a10_442 | RP | POS | 10.44 | 168.9799 | | | | 6.54E-01 | -0.08 | 0.68 | 2.94E-01 | -0.22 | 0.82 | 3.67E-01 | -0.19 | 0.83 | 9.11E-01 | 0.02 | 0.45 | 4784737 | 1839261 | 4609556 | 2135015 | 4834497 | 2159011 | 4384614 | 2108319 |
| RP_POS_213_18478a8_146 | RP | POS | 8.15 | 213.1848 | Unknown | 213.1843(100) | 4 | 8.03E-01 | -0.05 | 0.27 | 2.95E-01 | 0.21 | 1.07 | 8.11E-01 | 0.06 | 0.30 | 4.69E-01 | -0.16 | 0.30 | 927242 | 82891 | 923000 | 85313 | 914016 | 75825 | 931984 | 93760 |
| RP_POS_794_56537a13_194 | RP | POS | 13.19 | 794.5654 | | | | 1.06E-03 | 0.62 | 1.09 | 2.95E-01 | 0.21 | 0.84 | 1.54E-03 | 0.72 | 1.16 | 8.80E-03 | 0.51 | 1.20 | 5328774 | 980661 | 6046503 | 1162890 | 5924104 | 962654 | 6168901 | 1332272 |
| RP_POS_357_98346a0_708 | RP | POS | 0.71 | 357.9835 | | | | 2.50E-01 | -0.16 | 0.96 | 2.95E-01 | -0.24 | 0.85 | 1.50E-01 | -0.27 | 0.98 | 8.17E-01 | -0.04 | 0.63 | 453015 | 72786 | 426070 | 194476 | 446530 | 175402 | 405609 | 211645 |
| RP_POS_280_26352a9_982 | RP | POS | 9.98 | 280.2635 | | | | 3.43E-01 | -0.27 | 0.93 | 2.95E-01 | -0.16 | 0.73 | 2.35E-01 | -0.35 | 0.76 | 5.18E-01 | -0.19 | 0.64 | 3241208 | 5397621 | 2290669 | 2645329 | 2568860 | 2755833 | 2012478 | 2527011 |
| RP_POS_379_28146a10_809 | RP | POS | 10.81 | 379.2815 | | | | 1.73E-01 | -0.27 | 0.62 | 2.95E-01 | 0.21 | 0.76 | 4.59E-01 | -0.16 | 0.60 | 9.28E-02 | -0.38 | 0.71 | 1341896 | 319243 | 1250707 | 342836 | 1214664 | 345633 | 1286750 | 339643 |

**Supplementary table 1: Metabolomics results** (in asceding order according to p-value from progressive ALD vs  non-progressive ALD comparison, with molecular features with ID_level of 1-2 first)

| | | | | | | | | all_ALD_vs_control | | | Prog_ALD_vs_non-prog_ALD | | | Prog_ALD_vs_control_ALD | | | Non-prog_ALD_vs_control | | | Healthy_control | | all_ALD | | Non-prog_ALD | | Progressive_ALD | |
|---|---|---|---|---|---|---|---|---|---|---|---|---|---|---|---|---|---|---|---|---|---|---|---|---|---|---|---|
| | | | | | | | | t-test | Cohen's | PLS-DA | t-test | Cohen's | PLS-DA | t-test | Cohen's | PLS-DA | t-test | Cohen's | PLS-DA | | | | | | | | |
| Feature_ID | RP_HILIC | POS_NEG | Average.Rt | Average.Mz | Metabolite.name | curated_MSMS | ID_level | p | d | VIP | p | d | VIP | p | d | VIP | p | d | VIP | Mean | SD | Mean | SD | Mean | SD | Mean | SD |
| RP_POS_343_20035a11_253 | RP | POS | 11.25 | 343.2004 | | | | 8.57E-06 | 0.76 | 1.71 | 2.95E-01 | 0.21 | 0.96 | 3.48E-05 | 0.86 | 1.64 | 5.20E-04 | 0.65 | 1.65 | 2397454 | 1143517 | 3652171 | 1684726 | 3475034 | 1544564 | 3829308 | 1812406 |
| HILIC_NEG_116_9285a6_274 | HILIC | NEG | 6.274 | 116.9285 | | | | 1.51E-02 | -0.50 | 0.82 | 2.96E-01 | -0.21 | 0.73 | 9.71E-03 | -0.60 | 0.94 | 7.33E-02 | -0.39 | 0.86 | 20866262 | 7714338 | 16919155 | 7816611 | 17740838 | 7367448 | 16097473 | 8233381 |
| RP_POS_255_23166a10_135 | RP | POS | 10.14 | 255.2317 | | | | 4.78E-01 | 0.13 | 0.48 | 2.96E-01 | -0.22 | 0.81 | 9.20E-01 | 0.02 | 0.39 | 2.50E-01 | 0.24 | 0.82 | 3495172 | 344173 | 3547970 | 419870 | 3592070 | 404280 | 3503870 | 434475 |
| RP_POS_388_30533a8_356 | RP | POS | 8.36 | 388.3053 | | | | 3.18E-08 | 0.87 | 1.44 | 2.96E-01 | 0.21 | 0.71 | 1.84E-06 | 0.97 | 1.56 | 1.19E-05 | 0.76 | 1.52 | 502599 | 199343 | 803238 | 352849 | 766192 | 311524 | 840284 | 389484 |
| RP_POS_499_82727a4_275 | RP | POS | 4.28 | 499.8273 | | | | 6.69E-03 | 0.43 | 0.86 | 2.96E-01 | -0.22 | 0.75 | 8.19E-02 | 0.32 | 0.67 | 6.65E-03 | 0.55 | 1.08 | 1537956 | 2070261 | 2933673 | 3444578 | 3295248 | 3629766 | 2572098 | 3245053 |
| RP_POS_219_1738a11_631 | RP | POS | 11.63 | 219.1738 | | | | 1.68E-01 | 0.22 | 0.45 | 2.96E-01 | 0.23 | 0.74 | 1.12E-01 | 0.34 | 0.79 | 5.33E-01 | 0.11 | 0.79 | 1344116 | 250541 | 1426950 | 399152 | 1385041 | 340059 | 1468858 | 450191 |
| HILIC_NEG_285_70767a1_362 | HILIC | NEG | 1.362 | 285.7077 | | | | 2.21E-10 | 0.86 | 1.58 | 2.96E-01 | -0.22 | 0.58 | 1.02E-04 | 0.75 | 1.35 | 2.93E-09 | 0.97 | 2.15 | 1569135 | 218244 | 2063103 | 603802 | 2126495 | 513718 | 1999710 | 681532 |
| HILIC_NEG_132_92357a7_947 | HILIC | NEG | 7.947 | 132.9236 | | | | 6.98E-04 | -0.69 | 1.12 | 2.96E-01 | 0.20 | 0.74 | 7.48E-03 | -0.59 | 1.01 | 5.00E-04 | -0.79 | 1.24 | 2518491 | 857702 | 1886068 | 890306 | 1792670 | 907668 | 1979466 | 871666 |
| RP_POS_145_96626a0_254 | RP | POS | 0.25 | 145.9663 | | | | 9.19E-01 | 0.02 | 0.31 | 2.97E-01 | -0.20 | 0.77 | 7.44E-01 | -0.08 | 0.27 | 6.01E-01 | 0.12 | 0.70 | 931766 | 171625 | 935238 | 150581 | 951021 | 145496 | 919455 | 155352 |
| HILIC_POS_155_1179a0_509 | HILIC | POS | 0.509 | 155.1179 | | | | 3.75E-01 | 0.12 | 0.55 | 2.97E-01 | 0.24 | 0.73 | 2.78E-01 | 0.24 | 0.36 | 9.88E-01 | 0.00 | 0.79 | 3269715 | 767147 | 3488737 | 2054867 | 3272919 | 1212389 | 3704555 | 2639353 |
| HILIC_POS_302_23184a0_782 | HILIC | POS | 0.782 | 302.2318 | | | | 8.83E-04 | -0.63 | 1.12 | 2.97E-01 | -0.21 | 0.66 | 3.51E-04 | -0.74 | 1.40 | 1.71E-02 | -0.53 | 0.99 | 4079151 | 1801492 | 2742413 | 2111349 | 2963714 | 2325728 | 2521112 | 1870189 |
| HILIC_NEG_335_05005a4_723 | HILIC | NEG | 4.723 | 335.0501 | | | | 3.24E-04 | 0.57 | 0.99 | 2.97E-01 | 0.22 | 1.09 | 1.60E-03 | 0.68 | 1.02 | 5.48E-03 | 0.46 | 0.95 | 573128 | 293875 | 843976 | 505473 | 790966 | 394708 | 896986 | 595559 |
| RP_NEG_327_12787a10_705 | RP | NEG | 10.71 | 327.1279 | | | | 3.73E-05 | 0.67 | 1.53 | 2.98E-01 | 0.22 | 1.05 | 2.19E-04 | 0.78 | 1.47 | 1.44E-03 | 0.56 | 1.49 | 591380 | 265300 | 868665 | 432002 | 823486 | 370027 | 913844 | 485772 |
| RP_POS_408_78662a0_247 | RP | POS | 0.25 | 408.7866 | | | | 1.52E-01 | 0.29 | 0.74 | 2.98E-01 | -0.21 | 1.01 | 4.04E-01 | 0.19 | 0.49 | 8.35E-02 | 0.40 | 0.90 | 870139 | 116487 | 904714 | 118836 | 917136 | 120757 | 892291 | 116769 |
| HILIC_NEG_100_93336a3_013 | HILIC | NEG | 3.013 | 100.9334 | | | | 8.62E-01 | 0.03 | 0.75 | 2.99E-01 | 0.22 | 0.83 | 5.42E-01 | 0.14 | 0.54 | 7.16E-01 | -0.07 | 0.35 | 14962814 | 1932130 | 15033536 | 2180222 | 14805685 | 1852528 | 15261387 | 2462912 |
| HILIC_POS_357_23569a0_626 | HILIC | POS | 0.626 | 357.2357 | | | | 5.96E-07 | 0.60 | 1.30 | 2.99E-01 | -0.23 | 0.96 | 1.05E-03 | 0.48 | 1.47 | 1.69E-04 | 0.71 | 1.51 | 34881 | 67440 | 577302 | 1010881 | 682898 | 1122787 | 471707 | 883861 |
| RP_POS_956_79181a0_494 | RP | POS | 0.49 | 956.7918 | | | | 1.80E-03 | 0.41 | 1.04 | 2.99E-01 | 0.23 | 1.22 | 8.00E-03 | 0.52 | 1.11 | 4.95E-02 | 0.29 | 1.13 | 297991 | 145541 | 466653 | 462551 | 418333 | 384610 | 514973 | 528756 |
| RP_POS_464_37384a11_924 | RP | POS | 11.92 | 464.3738 | | | | 3.88E-01 | -0.19 | 0.57 | 2.99E-01 | 0.20 | 0.92 | 7.07E-01 | -0.09 | 0.82 | 2.20E-01 | -0.29 | 0.70 | 14263605 | 3682074 | 13634971 | 3119134 | 13309270 | 2903044 | 13960672 | 3318491 |
| HILIC_POS_205_09724a4_123 | HILIC | POS | 4.123 | 205.0972 | | | | 9.14E-03 | -0.44 | 1.10 | 2.99E-01 | -0.22 | 1.40 | 8.85E-03 | -0.55 | 1.11 | 7.69E-02 | -0.33 | 0.99 | 59863888 | 12115387 | 52441111 | 17642531 | 54282521 | 15978957 | 50599700 | 19147401 |
| RP_POS_985_58789a10_833 | RP | POS | 10.83 | 985.5879 | | | | 5.36E-03 | 0.40 | 0.90 | 2.99E-01 | -0.23 | 0.78 | 1.09E-01 | 0.28 | 0.83 | 6.34E-03 | 0.51 | 0.94 | 527895 | 102548 | 611251 | 231147 | 635366 | 237938 | 587136 | 223936 |
| HILIC_POS_136_04829a1_231 | HILIC | POS | 1.231 | 136.0483 | | | | 4.45E-07 | -1.12 | 2.01 | 2.99E-01 | -0.18 | 0.68 | 1.01E-06 | -1.21 | 1.86 | 2.43E-06 | -1.03 | 2.26 | 11797176 | 2504055 | 8906274 | 2192958 | 9135288 | 1585136 | 8677260 | 2663941 |
| RP_POS_168_98042a10_573 | RP | POS | 10.57 | 168.9804 | | | | 2.45E-01 | 0.25 | 0.50 | 3.00E-01 | -0.20 | 0.72 | 5.40E-01 | 0.15 | 0.64 | 1.31E-01 | 0.35 | 0.89 | 4994556 | 1497949 | 5346559 | 1393189 | 5491801 | 1326207 | 5201317 | 1455915 |
| RP_NEG_330_8735a15_437 | RP | NEG | 15.44 | 330.8735 | | | | 2.09E-01 | 0.25 | 1.11 | 3.00E-01 | -0.21 | 0.66 | 4.96E-01 | 0.15 | 0.58 | 1.23E-01 | 0.36 | 0.89 | 5236428 | 181482 | 5283623 | 187420 | 5303159 | 199976 | 5264087 | 173782 |
| RP_NEG_187_13371a8_491 | RP | NEG | 8.49 | 187.1337 | | | | 8.65E-01 | -0.03 | 0.31 | 3.00E-01 | 0.22 | 0.68 | 7.41E-01 | 0.08 | 0.55 | 4.29E-01 | -0.14 | 0.52 | 2206998 | 991002 | 2170164 | 1248571 | 2039888 | 818224 | 2300440 | 1563825 |
| HILIC_POS_148_0966a5_642 | HILIC | POS | 5.642 | 148.0966 | | | | 8.89E-01 | -0.03 | 0.38 | 3.00E-01 | -0.21 | 0.93 | 5.45E-01 | -0.13 | 0.22 | 7.14E-01 | 0.08 | 0.29 | 6044489 | 821804 | 6020454 | 912194 | 6115463 | 893384 | 5925444 | 929848 |
| RP_POS_389_33871a10_269 | RP | POS | 10.27 | 389.3387 | | | | 4.76E-01 | -0.13 | 0.74 | 3.00E-01 | 0.22 | 0.81 | 9.41E-01 | -0.02 | 0.57 | 1.90E-01 | -0.24 | 0.66 | 1742021 | 138079 | 1720247 | 180530 | 1701424 | 130782 | 1739071 | 219136 |
| RP_POS_451_22906a9_064 | RP | POS | 9.06 | 451.2291 | | | | 4.13E-02 | 0.35 | 0.61 | 3.00E-01 | 0.22 | 0.61 | 2.21E-02 | 0.46 | 0.90 | 2.31E-01 | 0.24 | 0.39 | 248330 | 152273 | 319604 | 212799 | 297461 | 216168 | 341748 | 209193 |
| HILIC_POS_696_43726a0_625 | HILIC | POS | 0.625 | 696.4373 | | | | 1.15E-06 | 0.59 | 1.30 | 3.00E-01 | -0.23 | 0.96 | 1.26E-03 | 0.48 | 1.47 | 2.10E-04 | 0.71 | 1.49 | 91861 | 284711 | 1073699 | 1840704 | 1265275 | 2050506 | 882123 | 1601891 |
| RP_POS_181_07217a2_184 | RP | POS | 2.18 | 181.0722 | | | | 5.37E-04 | -0.99 | 1.96 | 3.01E-01 | 0.14 | 0.64 | 1.70E-03 | -0.92 | 1.47 | 2.64E-04 | -1.06 | 1.94 | 36654466 | 33362472 | 13413503 | 15922434 | 11755866 | 12843110 | 15071139 | 18484281 |
| RP_POS_346_17944a10_704 | RP | POS | 10.70 | 346.1794 | | | | 7.01E-03 | 0.55 | 1.30 | 3.01E-01 | 0.20 | 1.14 | 7.19E-03 | 0.65 | 1.24 | 2.97E-02 | 0.45 | 1.18 | 347350 | 84906 | 395909 | 86204 | 386941 | 66763 | 404877 | 101944 |
| RP_POS_300_20166a3_646 | RP | POS | 3.65 | 300.2017 | | | | 5.73E-01 | 0.13 | 1.11 | 3.01E-01 | -0.19 | 1.19 | 8.77E-01 | 0.04 | 0.96 | 3.85E-01 | 0.23 | 1.01 | 1048455 | 2587250 | 1330937 | 1947753 | 1533307 | 2205367 | 1128567 | 1648539 |
| RP_POS_436_85858a0_512 | RP | POS | 0.51 | 436.8586 | | | | 1.68E-03 | -0.57 | 0.91 | 3.01E-01 | 0.21 | 0.73 | 3.54E-02 | -0.46 | 0.73 | 5.10E-04 | -0.68 | 1.17 | 537387 | 136249 | 440059 | 174120 | 421971 | 145183 | 458148 | 198767 |
| HILIC_POS_371_23999a5_476 | HILIC | POS | 5.476 | 371.2400 | | | | 2.33E-02 | 0.43 | 0.80 | 3.02E-01 | -0.21 | 1.14 | 1.30E-01 | 0.33 | 0.65 | 1.26E-02 | 0.54 | 1.06 | 8788594 | 1505346 | 9529598 | 1744992 | 9710766 | 1717142 | 9348431 | 1771039 |
| RP_POS_327_23157a10_512 | RP | POS | 10.51 | 327.2316 | | | | 9.48E-01 | 0.01 | 0.33 | 3.02E-01 | -0.22 | 0.80 | 5.70E-01 | -0.10 | 0.31 | 5.77E-01 | 0.12 | 0.44 | 285038 | 465782 | 292039 | 691107 | 363834 | 806129 | 220244 | 551945 |
| HILIC_NEG_155_86568a0_55 | HILIC | NEG | 0.55 | 155.8657 | | | | 1.66E-03 | -0.44 | 0.75 | 3.02E-01 | 0.23 | 0.68 | 9.60E-02 | -0.33 | 0.57 | 5.65E-04 | -0.56 | 1.53 | 531076 | 64321 | 470476 | 150127 | 454884 | 126304 | 486068 | 170550 |
| RP_POS_439_33917a11_029 | RP | POS | 11.03 | 439.3392 | | | | 9.14E-01 | 0.02 | 0.75 | 3.02E-01 | 0.22 | 0.86 | 5.30E-01 | 0.13 | 0.56 | 6.54E-01 | -0.09 | 0.71 | 1172449 | 166816 | 1176475 | 227112 | 1152905 | 225778 | 1200045 | 228260 |
| RP_POS_414_18463a10_907 | RP | POS | 10.91 | 414.1846 | | | | 5.11E-02 | 0.29 | 1.36 | 3.02E-01 | 0.23 | 0.93 | 4.48E-02 | 0.40 | 1.10 | 3.00E-01 | 0.17 | 1.13 | 676704 | 305198 | 837991 | 614246 | 774238 | 540167 | 901743 | 679868 |
| RP_POS_262_01343a0_734 | RP | POS | 0.73 | 262.0134 | | | | 1.64E-10 | 0.81 | 1.32 | 3.02E-01 | 0.22 | 0.70 | 1.74E-06 | 0.92 | 1.54 | 7.36E-06 | 0.70 | 1.35 | 419971 | 702232 | 2655820 | 2958214 | 2348902 | 2633011 | 2962737 | 3248948 |
| RP_POS_351_18411a10_704 | RP | POS | 10.70 | 351.1841 | | | | 8.07E-03 | 0.50 | 1.42 | 3.02E-01 | 0.21 | 1.26 | 8.27E-03 | 0.60 | 1.35 | 4.60E-02 | 0.39 | 1.24 | 1859945 | 328776 | 2051985 | 392612 | 2011247 | 328604 | 2092723 | 447288 |
| RP_POS_615_49524a14_758 | RP | POS | 14.76 | 615.4952 | | | | 6.27E-01 | 0.06 | 0.67 | 3.02E-01 | 0.24 | 0.64 | 4.31E-01 | 0.18 | 0.51 | 5.42E-01 | -0.05 | 0.41 | 2529641 | 1601048 | 2797725 | 4713439 | 2306908 | 1610890 | 3288542 | 6465277 |
| RP_POS_382_203a10_864 | RP | POS | 10.86 | 382.2030 | | | | 1.40E-02 | -0.52 | 1.06 | 3.02E-01 | -0.20 | 0.68 | 1.06E-02 | -0.62 | 1.09 | 5.56E-02 | -0.42 | 0.83 | 895418 | 358207 | 712383 | 339326 | 747580 | 292439 | 677187 | 380245 |
| HILIC_POS_212_99937a4_797 | HILIC | POS | 4.797 | 212.9994 | | | | 6.46E-01 | 0.08 | 0.25 | 3.02E-01 | -0.22 | 0.92 | 8.92E-01 | -0.03 | 0.13 | 3.26E-01 | 0.19 | 0.51 | 735406 | 91286 | 744762 | 122866 | 757505 | 109566 | 732020 | 134774 |
| RP_POS_547_35443a10_933 | RP | POS | 10.93 | 547.3544 | | | | 3.00E-01 | -0.24 | 0.88 | 3.02E-01 | -0.20 | 1.31 | 1.82E-01 | -0.33 | 0.66 | 5.69E-01 | -0.14 | 0.78 | 37404844 | 8342901 | 35703754 | 6783383 | 36407090 | 6529454 | 35000417 | 7023126 |
| HILIC_NEG_227_96104a4_083 | HILIC | NEG | 4.083 | 227.9610 | | | | 1.98E-08 | 0.83 | 1.29 | 3.03E-01 | 0.21 | 0.75 | 9.67E-06 | 0.94 | 1.39 | 2.12E-06 | 0.73 | 1.59 | 223837 | 69010 | 336245 | 139441 | 321789 | 104416 | 350702 | 167198 |
| RP_POS_282_27908a9_95 | RP | POS | 9.95 | 282.2791 | | | | 8.10E-02 | 0.37 | 0.68 | 3.03E-01 | -0.20 | 0.72 | 2.16E-01 | 0.27 | 0.68 | 5.75E-02 | 0.47 | 0.79 | 994951 | 1164145 | 1410731 | 1103041 | 1525030 | 1284949 | 1296432 | 883444 |
| HILIC_POS_675_54468a0_973 | HILIC | POS | 0.973 | 675.5447 | | | | 2.75E-01 | -0.21 | 0.40 | 3.03E-01 | 0.21 | 0.88 | 6.43E-01 | -0.10 | 0.25 | 1.36E-01 | -0.32 | 0.73 | 1906876 | 456087 | 1801466 | 514828 | 1748173 | 479141 | 1854758 | 547845 |
| RP_POS_485_36041a11_953 | RP | POS | 11.95 | 485.3604 | | | | 5.78E-01 | -0.12 | 0.79 | 3.03E-01 | -0.20 | 0.79 | 3.44E-01 | -0.22 | 0.75 | 9.42E-01 | -0.02 | 0.37 | 1226047 | 514914 | 1168507 | 475615 | 1217721 | 490863 | 1119293 | 459506 |
| HILIC_NEG_259_83914a7_667 | HILIC | NEG | 7.667 | 259.8391 | | | | 2.23E-01 | 0.21 | 0.80 | 3.03E-01 | 0.22 | 0.90 | 1.20E-01 | 0.32 | 0.61 | 6.12E-01 | 0.10 | 0.55 | 16907338 | 999653 | 17182764 | 1379533 | 17040031 | 1351350 | 17325497 | 1406179 |
| HILIC_POS_86_06067a0_846 | HILIC | POS | 0.846 | 86.0607 | | | | 9.82E-02 | 0.30 | 0.69 | 3.03E-01 | 0.22 | 0.87 | 6.79E-02 | 0.41 | 0.70 | 3.16E-01 | 0.20 | 0.37 | 1226379 | 479405 | 1400119 | 594856 | 1338554 | 508207 | 1461685 | 670015 |
| HILIC_NEG_134_89478a2_172 | HILIC | NEG | 2.172 | 134.8948 | | | | 3.26E-01 | 0.19 | 0.71 | 3.03E-01 | -0.21 | 0.86 | 7.04E-01 | 0.08 | 0.14 | 1.79E-01 | 0.29 | 0.49 | 41380174 | 8919999 | 43240109 | 10166663 | 44291521 | 10311338 | 42188698 | 10012580 |
| RP_POS_349_05902a9_047 | RP | POS | 9.05 | 349.0590 | | | | 4.08E-04 | -0.73 | 1.17 | 3.04E-01 | -0.20 | 0.78 | 1.26E-04 | -0.83 | 1.46 | 7.27E-03 | -0.63 | 0.97 | 4829366 | 284132 | 4611447 | 283883 | 4640814 | 326936 | 4582081 | 232759 |
| RP_NEG_1010_75519a0_495 | RP | NEG | 0.50 | 1010.7552 | | | | 1.10E-02 | -0.61 | 1.04 | 3.04E-01 | -0.18 | 0.89 | 5.64E-03 | -0.70 | 1.11 | 4.27E-02 | -0.51 | 0.86 | 958220 | 119289 | 897300 | 89503 | 906553 | 93668 | 888046 | 85068 |
| HILIC_POS_377_23315a0_631 | HILIC | POS | 0.631 | 377.2332 | | | | 1.21E-06 | 0.58 | 1.26 | 3.04E-01 | -0.23 | 0.95 | 1.34E-03 | 0.46 | 1.43 | 2.83E-04 | 0.69 | 1.47 | 39732 | 38516 | 1152518 | 2150814 | 1374906 | 2413383 | 930130 | 1849611 |
| HILIC_NEG_100_93346a5_429 | HILIC | NEG | 5.429 | 100.9335 | | | | 4.20E-01 | 0.15 | 0.41 | 3.04E-01 | -0.22 | 0.74 | 8.34E-01 | 0.04 | 0.09 | 2.47E-01 | 0.26 | 0.44 | 57875088 | 5266951 | 58788230 | 6320484 | 59441498 | 6849085 | 58134962 | 5738589 |
| RP_POS_766_53705a12_414 | RP | POS | 12.41 | 766.5371 | | | | 1.21E-02 | -0.48 | 1.13 | 3.05E-01 | -0.21 | 0.77 | 7.53E-03 | -0.58 | 1.10 | 8.04E-02 | -0.37 | 0.72 | 1281220 | 594426 | 955175 | 697305 | 1027119 | 689375 | 883232 | 704692 |
| RP_POS_553_46045a14_159 | RP | POS | 14.16 | 553.4605 | | | | 6.67E-01 | -0.09 | 0.71 | 3.05E-01 | 0.20 | 0.66 | 9.63E-01 | 0.01 | 0.67 | 4.02E-01 | -0.19 | 0.49 | 18095576 | 8680458 | 17342052 | 8177348 | 16498481 | 7844041 | 18185623 | 8492436 |
| RP_POS_231_92145a15_584 | RP | POS | 15.58 | 231.9215 | | | | 8.17E-02 | -0.36 | 0.63 | 3.05E-01 | 0.20 | 0.80 | 2.69E-01 | -0.26 | 0.46 | 3.96E-02 | -0.46 | 0.94 | 1435525 | 72288 | 1409523 | 71599 | 1402138 | 66715 | 1416908 | 76126 |
| HILIC_POS_64_01662a5_463 | HILIC | POS | 5.463 | 64.0166 | | | | 6.52E-01 | -0.08 | 0.52 | 3.05E-01 | 0.21 | 0.78 | 9.20E-01 | 0.02 | 0.07 | 3.55E-01 | -0.19 | 0.72 | 7856481 | 737740 | 7785386 | 870920 | 7695584 | 801353 | 7875188 | 934804 |
| RP_POS_294_11188a8_017 | RP | POS | 8.02 | 294.1119 | | | | 2.46E-01 | 0.13 | 0.64 | 3.05E-01 | -0.24 | 0.63 | 2.59E-02 | 0.02 | 0.85 | 2.76E-01 | 0.25 | 0.68 | 373575 | 51618 | 856920 | 4138351 | 1285568 | 5848232 | 428271 | 156881 |
| RP_POS_250_21394a10_188 | RP | POS | 10.19 | 250.2139 | | | | 7.99E-03 | 0.32 | 0.70 | 3.05E-01 | 0.23 | 0.90 | 5.60E-02 | 0.44 | 0.79 | 8.17E-04 | 0.21 | 1.20 | 1021630 | 296998 | 1447656 | 1489291 | 1293514 | 408814 | 1601799 | 2065338 |
| RP_NEG_291_08377a1_096 | RP | NEG | 1.10 | 291.0838 | | | | 2.57E-01 | -0.19 | 0.62 | 3.05E-01 | 0.22 | 0.74 | 7.00E-01 | -0.08 | 0.48 | 1.01E-01 | -0.30 | 0.82 | 5584995 | 4298072 | 4469558 | 6136559 | 3836974 | 5142981 | 5102142 | 6986868 |
| RP_POS_166_08627a1_451 | RP | POS | 1.45 | 166.0863 | | 120.0814(100), 166.0854(5) | | 1.24E-02 | 0.41 | 0.78 | 3.05E-01 | 0.22 | 0.74 | 1.91E-02 | 0.52 | 0.89 | 6.88E-02 | 0.30 | 0.87 | 223131323 | 45762898 | 250530596 | 70309489 | 243278780 | 51699440 | 257782412 | 84897458 |
| RP_POS_513_84259a13_501 | RP | POS | 13.50 | 513.8426 | | | | 3.31E-01 | -0.32 | 1.14 | 3.06E-01 | -0.10 | 1.05 | 2.63E-01 | -0.37 | 0.93 | 4.21E-01 | -0.27 | 0.87 | 329834 | 885536 | 173403 | 245269 | 198662 | 263462 | 148144 | 225449 |
| RP_NEG_224_06241a6_687 | RP | NEG | 6.69 | 224.0624 | | | | 2.62E-01 | -0.22 | 0.57 | 3.06E-01 | -0.21 | 0.74 | 1.35E-01 | -0.33 | 0.59 | 5.99E-01 | -0.12 | 0.57 | 43447679 | 47223729 | 32443507 | 49654946 | 37552673 | 52424272 | 27334341 | 46690138 |
| RP_POS_520_33258a5_108 | RP | POS | 5.11 | 520.3326 | | | | 9.06E-02 | 0.32 | 1.17 | 3.06E-01 | -0.21 | 1.21 | 2.90E-01 | 0.21 | 1.06 | 6.32E-02 | 0.42 | 1.19 | 1102384 | 3321698 | 2321378 | 3975916 | 2730546 | 4479529 | 1912211 | 3395670 |
| HILIC_NEG_112_98571a0_699 | HILIC | NEG | 0.699 | 112.9857 | | | | 5.23E-03 | 0.51 | 0.93 | 3.06E-01 | -0.21 | 0.71 | 4.39E-02 | 0.41 | 0.78 | 4.50E-03 | 0.62 | 1.06 | 2215024 | 880774 | 2767025 | 1106644 | 2880876 | 1173672 | 2653174 | 1034580 |
| RP_NEG_494_95416a11_391 | RP | NEG | 11.39 | 494.9542 | | | | 9.67E-02 | -0.44 | 0.77 | 3.06E-01 | -0.17 | 0.69 | 5.30E-02 | -0.53 | 0.91 | 2.07E-01 | -0.36 | 0.55 | 498497 | 540630 | 326953 | 317621 | 359639 | 365276 | 294267 | 261186 |
| HILIC_POS_240_11488a0_537 | HILIC | POS | 0.537 | 240.1149 | | | | 2.57E-01 | 0.13 | 0.36 | 3.06E-01 | -0.24 | 0.65 | 6.61E-01 | 0.02 | 0.24 | 2.76E-01 | 0.25 | 0.46 | 222785 | 675327 | 794891 | 4875252 | 1298648 | 6856430 | 291134 | 701920 |
| RP_NEG_326_98907a8_022 | RP | NEG | 8.02 | 326.9891 | | | | 8.37E-01 | -0.04 | 0.34 | 3.06E-01 | 0.21 | 0.93 | 7.64E-01 | 0.07 | 0.44 | 5.10E-01 | -0.15 | 0.32 | 7674438 | 432180 | 7655767 | 479089 | 7606516 | 483468 | 7705019 | 474388 |
| HILIC_NEG_118_92428a1_049 | HILIC | NEG | 1.049 | 118.9243 | | | | 4.58E-02 | 0.37 | 0.94 | 3.07E-01 | 0.21 | 0.85 | 3.00E-02 | 0.47 | 1.11 | 2.00E-01 | 0.26 | 0.58 | 1241464 | 298683 | 1372916 | 371213 | 1334766 | 347422 | 1411066 | 393368 |
| RP_POS_293_2085a9_962 | RP | POS | 9.96 | 293.2085 | | | | 5.38E-01 | 0.10 | 0.82 | 3.07E-01 | 0.22 | 0.86 | 3.24E-01 | 0.21 | 0.73 | 9.60E-01 | -0.01 | 0.84 | 4050410 | 512919 | 4124653 | 785476 | 4043916 | 651814 | 4205390 | 899095 |
| RP_POS_251_12733a8_624 | RP | POS | 8.62 | 251.1273 | | | | 2.82E-01 | -0.21 | 0.56 | 3.07E-01 | 0.21 | 0.75 | 6.41E-01 | -0.10 | 0.45 | 1.47E-01 | -0.31 | 0.81 | 1508601 | 102044 | 1485446 | 113617 | 1473781 | 109364 | 1497110 | 117656 |
| RP_POS_529_42969a14_264 | RP | POS | 14.26 | 529.4297 | | | | 4.98E-01 | 0.11 | 0.79 | 3.07E-01 | 0.22 | 0.71 | 3.47E-01 | 0.22 | 0.63 | 9.67E-01 | -0.01 | 0.47 | 1319481 | 973830 | 1482657 | 1670351 | 1310728 | 894578 | 1654585 | 2185518 |
| RP_POS_809_52618a13_068 | RP | POS | 13.07 | 809.5262 | | | | 5.78E-01 | -0.11 | 0.41 | 3.07E-01 | -0.21 | 0.76 | 3.37E-01 | -0.21 | 0.74 | 9.99E-01 | 0.00 | 0.45 | 3850035 | 831532 | 3752048 | 955720 | 3850162 | 921814 | 3653934 | 987955 |
| RP_POS_295_15152a8_666 | RP | POS | 8.67 | 295.1515 | | | | 5.91E-01 | 0.09 | 0.57 | 3.07E-01 | -0.22 | 0.77 | 9.28E-01 | -0.02 | 0.23 | 3.71E-01 | 0.20 | 0.65 | 535638 | 270959 | 568412 | 374248 | 606842 | 445665 | 529983 | 285219 |
| RP_POS_646_50574a13_498 | RP | POS | 13.50 | 646.5057 | | | | 7.04E-01 | 0.05 | 0.30 | 3.08E-01 | 0.23 | 0.70 | 4.56E-01 | 0.17 | 0.37 | 5.87E-01 | -0.06 | 0.43 | 1362345 | 175387 | 1382571 | 431301 | 1338222 | 222851 | 1426920 | 567592 |
| RP_POS_320_25583a9_549 | RP | POS | 9.55 | 320.2558 | | | | 3.47E-01 | -0.25 | 0.85 | 3.08E-01 | -0.17 | 0.73 | 2.23E-01 | -0.34 | 0.74 | 5.55E-01 | -0.17 | 0.50 | 12733378 | 18478700 | 9462041 | 10678342 | 10557130 | 11742655 | 8366952 | 9489137 |
| HILIC_NEG_200_85883a4_752 | HILIC | NEG | 4.752 | 200.8588 | | | | 5.65E-01 | -0.09 | 0.70 | 3.08E-01 | -0.23 | 1.28 | 1.81E-01 | -0.20 | 0.47 | 9.02E-01 | 0.03 | 0.48 | 2222442 | 475320 | 2150681 | 917898 | 2244904 | 1132601 | 2056459 | 633534 |
| RP_POS_599_45178a15_291 | RP | POS | 15.29 | 599.4518 | | | | 7.04E-01 | -0.07 | 0.55 | 3.08E-01 | 0.21 | 0.84 | 8.64E-01 | 0.04 | 0.51 | 3.98E-01 | -0.18 | 0.58 | 1320400 | 264768 | 1298659 | 323805 | 1265495 | 314164 | 1331824 | 333010 |
| HILIC_NEG_201_80212a0_553 | HILIC | NEG | 0.553 | 201.8021 | | | | 5.56E-01 | 0.12 | 0.44 | 3.08E-01 | -0.21 | 0.79 | 9.35E-01 | 0.02 | 0.06 | 3.48E-01 | 0.22 | 0.55 | 167655 | 76838 | 176904 | 76890 | 184781 | 84764 | 169028 | 68069 |
| RP_POS_199_01123a10_571 | RP | POS | 10.57 | 199.0112 | | | | 1.80E-02 | -0.46 | 0.84 | 3.08E-01 | 0.21 | 0.66 | 9.43E-02 | -0.36 | 0.69 | 1.11E-02 | -0.57 | 0.88 | 3746455 | 1015468 | 3231919 | 1112036 | 3118082 | 1137214 | 3345757 | 1085718 |
| HILIC_POS_371_23969a2_454 | HILIC | POS | 2.454 | 371.2397 | | | | 8.93E-12 | 0.89 | 1.68 | 3.09E-01 | -0.21 | 0.63 | 3.31E-06 | 0.78 | 1.62 | 6.85E-08 | 1.00 | 1.85 | 1777068 | 866726 | 4199206 | 2843454 | 4489852 | 2934870 | 3908559 | 2747654 |
| RP_POS_355_26318a9_325 | RP | POS | 9.33 | 355.2632 | | | | 2.67E-01 | -0.32 | 0.63 | 3.09E-01 | 0.15 | 0.62 | 4.19E-01 | -0.24 | 0.40 | 1.78E-01 | -0.39 | 0.60 | 1390023 | 2710964 | 829593 | 1320139 | 694673 | 1176509 | 964512 | 1449061 |
| HILIC_POS_678_5061a0_621 | HILIC | POS | 0.621 | 678.5061 | | | | 1.16E-01 | 0.33 | 0.72 | 3.09E-01 | 0.20 | 0.62 | 6.91E-02 | 0.43 | 0.76 | 3.15E-01 | 0.22 | 0.56 | 309194 | 328616 | 415462 | 322838 | 382484 | 304882 | 448439 | 339711 |
| RP_NEG_404_16357a11_612 | RP | NEG | 11.61 | 404.1636 | | | | 1.41E-01 | -0.30 | 0.66 | 3.10E-01 | 0.20 | 0.80 | 3.54E-01 | -0.20 | 0.60 | 9.04E-02 | -0.40 | 0.64 | 1057256 | 135243 | 1016269 | 134507 | 1002536 | 149063 | 1030002 | 118105 |
| RP_NEG_297_15421a14_115 | RP | NEG | 14.12 | 297.1542 | | | | 6.18E-01 | 0.10 | 0.77 | 3.10E-01 | -0.21 | 0.61 | 9.71E-01 | -0.01 | 0.34 | 3.80E-01 | 0.20 | 0.79 | 3865716 | 3080697 | 4187289 | 3400573 | 4534015 | 3713974 | 3840563 | 3053660 |
| HILIC_POS_379_24896a0_627 | HILIC | POS | 0.627 | 379.2490 | | | | 7.95E-07 | 0.59 | 1.28 | 3.11E-01 | -0.23 | 0.95 | 1.37E-03 | 0.48 | 1.45 | 1.75E-04 | 0.70 | 1.49 | 107340 | 265999 | 1827398 | 3237860 | 2157421 | 3558338 | 1497374 | 2880517 |
| RP_POS_159_07649a0_548 | RP | POS | 0.55 | 159.0765 | | | | 7.47E-08 | 0.80 | 1.46 | 3.11E-01 | 0.21 | 1.24 | 1.31E-06 | 0.91 | 1.71 | 1.51E-04 | 0.69 | 1.31 | 523431 | 112199 | 697331 | 226357 | 674276 | 227657 | 720386 | 224962 |

**Supplementary table 1: Metabolomics results** (in asceding order according to p-value from progressive ALD vs  non-progressive ALD comparison, with molecular features with ID_level of 1-2 first)

| | | | | | | | | all_ALD_vs_control | | | Prog_ALD_vs_non-prog_ALD | | | Prog_ALD_vs_control_ALD | | | Non-prog_ALD_vs_control | | | Healthy_control | | all_ALD | | Non-prog_ALD | | Progressive_ALD | |
|---|---|---|---|---|---|---|---|---|---|---|---|---|---|---|---|---|---|---|---|---|---|---|---|---|---|---|---|
| | | | | | | | | t-test | Cohen's | PLS-DA | t-test | Cohen's | PLS-DA | t-test | Cohen's | PLS-DA | t-test | Cohen's | PLS-DA | | | | | | | | |
| Feature_ID | RP_HILIC | POS_NEG | Average.Rt | Average.Mz | Metabolite.name | curated_MSMS | ID_level | p | d | VIP | p | d | VIP | p | d | VIP | p | d | VIP | Mean | SD | Mean | SD | Mean | SD | Mean | SD |
| HILIC_NEG_202_92894a4_723 | HILIC | NEG | 4.723 | 202.9289 | | | | 2.78E-03 | 0.50 | 0.95 | 3.11E-01 | 0.21 | 0.93 | 3.98E-03 | 0.61 | 0.98 | 3.20E-02 | 0.40 | 0.74 | 776072 | 201482 | 920131 | 301223 | 889454 | 267044 | 950808 | 331797 |
| RP_POS_306_89014a15_428 | RP | POS | 15.43 | 306.8901 | | | | 7.30E-01 | -0.06 | 0.50 | 3.11E-01 | 0.21 | 0.95 | 8.21E-01 | 0.04 | 0.33 | 4.42E-01 | -0.17 | 0.38 | 1427678 | 262184 | 1407923 | 333278 | 1373988 | 365515 | 1441859 | 297428 |
| RP_POS_553_4566a14_506 | RP | POS | 14.51 | 553.4566 | | | | 1.07E-01 | -0.31 | 0.91 | 3.11E-01 | 0.21 | 1.11 | 3.56E-01 | -0.20 | 0.81 | 5.31E-02 | -0.41 | 0.77 | 48825141 | 9486894 | 45557893 | 10868619 | 44451576 | 10319505 | 46664209 | 11387471 |
| RP_POS_168_00809a15_535 | RP | POS | 15.54 | 168.0081 | | | | 1.42E-01 | -0.22 | 0.69 | 3.11E-01 | -0.22 | 0.63 | 1.49E-01 | -0.34 | 0.73 | 3.85E-01 | -0.11 | 0.40 | 15293475 | 401970 | 15141319 | 741064 | 15216973 | 360160 | 15065666 | 983954 |
| HILIC_POS_616_07721a0_558 | HILIC | POS | 0.558 | 616.0772 | | | | 2.20E-06 | 0.61 | 1.32 | 3.12E-01 | -0.22 | 0.92 | 1.01E-03 | 0.50 | 1.46 | 2.04E-04 | 0.72 | 1.48 | 112703 | 395621 | 865655 | 1348583 | 1002858 | 1510408 | 728452 | 1163924 |
| RP_POS_764_55566a13_498 | RP | POS | 13.50 | 764.5557 | | | | 2.75E-03 | -0.72 | 1.13 | 3.12E-01 | -0.18 | 0.69 | 1.67E-03 | -0.81 | 1.26 | 1.16E-02 | -0.63 | 0.94 | 8036668 | 3251149 | 6054464 | 2413342 | 6299856 | 2347402 | 5809071 | 2476714 |
| RP_NEG_325_18509a15_122 | RP | NEG | 15.12 | 325.1851 | | | | 1.97E-01 | -0.29 | 0.72 | 3.12E-01 | -0.19 | 0.88 | 9.15E-02 | -0.39 | 0.81 | 4.47E-01 | -0.20 | 0.68 | 12919409 | 12908775 | 9632116 | 10484607 | 10699066 | 12702568 | 8565167 | 7643225 |
| RP_POS_556_44165a10_542 | RP | POS | 10.54 | 556.4417 | | | | 2.93E-01 | -0.34 | 0.55 | 3.12E-01 | -0.12 | 0.85 | 2.24E-01 | -0.39 | 0.61 | 3.95E-01 | -0.28 | 0.46 | 1948360 | 939418 | 1767925 | 315437 | 1799965 | 326314 | 1735885 | 304066 |
| RP_POS_640_31744a7_257 | RP | POS | 7.26 | 640.3174 | | | | 2.29E-01 | -0.23 | 0.76 | 3.12E-01 | 0.21 | 0.88 | 5.72E-01 | -0.12 | 0.64 | 1.23E-01 | -0.33 | 0.83 | 2675866 | 157177 | 2635226 | 185896 | 2616354 | 184419 | 2654097 | 187305 |
| HILIC_NEG_257_98438a0_836 | HILIC | NEG | 0.836 | 257.9844 | | | | 5.53E-01 | -0.11 | 0.87 | 3.12E-01 | -0.21 | 0.95 | 3.31E-01 | -0.22 | 0.48 | 9.85E-01 | 0.00 | 0.34 | 362291 | 96867 | 349916 | 117574 | 361853 | 107150 | 337979 | 127112 |
| RP_NEG_347_22183a10_979 | RP | NEG | 10.98 | 347.2218 | | | | 2.91E-02 | 0.40 | 1.41 | 3.13E-01 | -0.21 | 1.38 | 1.73E-01 | 0.29 | 1.16 | 1.41E-02 | 0.50 | 1.33 | 1723361 | 565129 | 1997709 | 717011 | 2070492 | 674194 | 1924925 | 757200 |
| HILIC_POS_128_08195a1_176 | HILIC | POS | 1.176 | 128.0820 | | | | 7.07E-03 | -0.59 | 1.07 | 3.13E-01 | 0.19 | 0.85 | 4.52E-02 | -0.50 | 0.74 | 2.47E-03 | -0.69 | 1.40 | 493914 | 185809 | 390677 | 162653 | 374162 | 128704 | 407192 | 190605 |
| RP_POS_368_21869a10_271 | RP | POS | 10.27 | 368.2187 | | | | 3.99E-01 | -0.14 | 0.55 | 3.13E-01 | 0.22 | 0.70 | 8.76E-01 | -0.03 | 0.34 | 1.88E-01 | -0.25 | 0.58 | 803711 | 79619 | 788115 | 118377 | 776108 | 107919 | 800122 | 127950 |
| HILIC_POS_145_10857a3_119 | HILIC | POS | 3.119 | 145.1086 | | | | 3.49E-01 | 0.14 | 0.34 | 3.13E-01 | 0.22 | 0.77 | 2.48E-01 | 0.25 | 0.58 | 8.61E-01 | 0.03 | 0.20 | 44780 | 135668 | 79116 | 275331 | 51162 | 193741 | 107070 | 337685 |
| RP_NEG_503_13342a8_946 | RP | NEG | 8.95 | 503.1334 | | | | 9.97E-02 | 0.24 | 0.54 | 3.13E-01 | -0.22 | 0.67 | 3.64E-01 | 0.13 | 0.49 | 1.04E-01 | 0.36 | 0.73 | 892004 | 1647038 | 1624337 | 3312249 | 1960455 | 4095884 | 1288219 | 2271354 |
| RP_NEG_195_81081a0_705 | RP | NEG | 0.71 | 195.8108 | | | | 9.24E-01 | 0.02 | 0.18 | 3.14E-01 | -0.21 | 0.63 | 6.73E-01 | -0.09 | 0.56 | 5.37E-01 | 0.12 | 0.46 | 11294700 | 6551197 | 11432137 | 8593553 | 12302522 | 8060052 | 10561753 | 9093676 |
| RP_NEG_339_68536a15_599 | RP | NEG | 15.60 | 339.6854 | | | | 2.12E-01 | -0.26 | 0.61 | 3.14E-01 | 0.20 | 0.68 | 4.98E-01 | -0.16 | 0.35 | 1.14E-01 | -0.36 | 0.70 | 1106465 | 58805 | 1091410 | 58251 | 1085511 | 56190 | 1097308 | 60227 |
| HILIC_POS_158_04041a3_695 | HILIC | POS | 3.695 | 158.0404 | | | | 2.49E-02 | -0.38 | 0.87 | 3.14E-01 | 0.21 | 0.87 | 1.91E-01 | -0.28 | 0.90 | 1.04E-02 | -0.49 | 0.97 | 1419441 | 445660 | 1186710 | 643274 | 1121563 | 576221 | 1251858 | 703814 |
| HILIC_POS_202_14378a0_507 | HILIC | POS | 0.507 | 202.1438 | | | | 1.53E-02 | 0.41 | 0.90 | 3.14E-01 | -0.21 | 0.91 | 1.30E-01 | 0.30 | 0.91 | 1.03E-02 | 0.51 | 1.03 | 354152 | 368130 | 566174 | 554819 | 622283 | 555417 | 510065 | 554091 |
| RP_POS_365_23407a12_207 | RP | POS | 12.21 | 365.2341 | | | | 2.39E-02 | -0.43 | 0.94 | 3.14E-01 | 0.21 | 0.70 | 1.24E-01 | -0.33 | 0.77 | 1.39E-02 | -0.54 | 0.96 | 795978 | 250946 | 673613 | 286208 | 644671 | 285714 | 702556 | 286635 |
| RP_NEG_287_77679a15_612 | RP | NEG | 15.61 | 287.7768 | | | | 2.72E-01 | -0.24 | 0.64 | 3.14E-01 | -0.19 | 0.92 | 1.60E-01 | -0.34 | 0.68 | 5.44E-01 | -0.15 | 0.48 | 804361 | 42323 | 795102 | 36668 | 798810 | 36585 | 791395 | 36744 |
| RP_POS_501_37546a10_88 | RP | POS | 10.88 | 501.3755 | | | | 1.48E-02 | -0.45 | 0.86 | 3.15E-01 | 0.21 | 0.94 | 8.42E-02 | -0.35 | 0.82 | 1.23E-02 | -0.55 | 0.96 | 35382046 | 1689049 | 34469542 | 2087119 | 34258618 | 2267891 | 34680467 | 1888626 |
| RP_POS_367_20316a10_908 | RP | POS | 10.91 | 367.2032 | | | | 2.56E-03 | 0.40 | 1.41 | 3.15E-01 | 0.23 | 0.76 | 1.43E-02 | 0.51 | 1.11 | 3.23E-02 | 0.29 | 1.38 | 558731 | 298576 | 874222 | 879549 | 785249 | 632458 | 963195 | 1070907 |
| RP_NEG_567_53644a11_61 | RP | NEG | 11.61 | 567.5364 | | | | 9.22E-02 | -0.33 | 0.86 | 3.15E-01 | -0.21 | 1.03 | 3.53E-02 | -0.43 | 0.90 | 3.35E-01 | -0.23 | 0.77 | 1987478 | 722046 | 1729746 | 796987 | 1810319 | 922099 | 1649174 | 647937 |
| RP_POS_514_28741a6_081 | RP | POS | 6.08 | 514.2874 | | | | 9.10E-01 | -0.02 | 0.37 | 3.15E-01 | 0.21 | 0.67 | 7.11E-01 | 0.08 | 0.37 | 5.19E-01 | -0.13 | 0.44 | 6643007 | 384299 | 6633675 | 465777 | 6586602 | 385463 | 6680748 | 534060 |
| HILIC_NEG_312_8367a4_109 | HILIC | NEG | 4.109 | 312.8367 | | | | 3.16E-01 | -0.24 | 0.42 | 3.15E-01 | 0.18 | 0.93 | 5.56E-01 | -0.15 | 0.24 | 1.95E-01 | -0.33 | 0.62 | 1309384 | 653151 | 1182948 | 466760 | 1135817 | 454551 | 1230079 | 478568 |
| RP_POS_256_89368a15_369 | RP | POS | 15.37 | 256.8937 | | | | 4.20E-02 | -0.30 | 0.56 | 3.16E-01 | 0.22 | 0.60 | 2.07E-01 | -0.18 | 0.61 | 5.82E-02 | -0.41 | 0.80 | 1443884 | 87488 | 1394252 | 185198 | 1375554 | 225741 | 1412950 | 132756 |
| RP_NEG_164_83542a15_575 | RP | NEG | 15.58 | 164.8354 | | | | 1.84E-01 | -0.25 | 0.69 | 3.16E-01 | -0.21 | 0.74 | 7.30E-02 | -0.36 | 0.79 | 5.23E-01 | -0.15 | 0.37 | 1002364 | 39939 | 991010 | 46386 | 995691 | 54115 | 986330 | 37068 |
| HILIC_NEG_389_66833a1_364 | HILIC | NEG | 1.364 | 389.6683 | | | | 7.95E-06 | 0.73 | 1.15 | 3.16E-01 | -0.21 | 0.67 | 2.52E-03 | 0.63 | 1.01 | 3.75E-06 | 0.83 | 1.58 | 397473 | 166445 | 586339 | 266461 | 613213 | 225021 | 559465 | 302231 |
| RP_POS_504_84506a0_512 | RP | POS | 0.51 | 504.8451 | | | | 6.89E-03 | -0.54 | 0.86 | 3.16E-01 | 0.20 | 0.72 | 5.79E-02 | -0.44 | 0.69 | 2.47E-03 | -0.64 | 1.07 | 362962 | 115921 | 295996 | 121942 | 283701 | 102232 | 308290 | 138864 |
| RP_POS_1078_62793a10_315 | RP | POS | 10.32 | 1078.6279 | | | | 1.15E-01 | -0.34 | 0.90 | 3.16E-01 | -0.19 | 1.24 | 6.14E-02 | -0.44 | 0.85 | 3.05E-01 | -0.25 | 0.70 | 279830 | 176807 | 223583 | 157831 | 239477 | 164671 | 207690 | 150658 |
| RP_POS_468_38907a10_454 | RP | POS | 10.45 | 468.3891 | | | | 1.30E-01 | -0.32 | 0.72 | 3.17E-01 | 0.20 | 0.78 | 3.43E-01 | -0.22 | 0.56 | 7.19E-02 | -0.42 | 0.73 | 5938201 | 449365 | 5799612 | 425560 | 5756787 | 418866 | 5842437 | 432096 |
| RP_POS_633_45349a10_925 | RP | POS | 10.93 | 633.4535 | | | | 7.57E-01 | -0.06 | 0.68 | 3.17E-01 | 0.21 | 0.75 | 8.59E-01 | 0.04 | 0.63 | 4.19E-01 | -0.16 | 0.66 | 10173631 | 1278169 | 10091257 | 1391697 | 9951163 | 1090947 | 10231352 | 1637971 |
| RP_NEG_231_9321a13_392 | RP | NEG | 13.39 | 231.9321 | | | | 4.66E-01 | 0.16 | 0.34 | 3.17E-01 | -0.20 | 1.05 | 8.06E-01 | 0.06 | 0.27 | 2.72E-01 | 0.26 | 0.45 | 4703084 | 2417512 | 5056291 | 2193848 | 5276838 | 2055607 | 4835744 | 2323656 |
| RP_POS_822_48035a11_627 | RP | POS | 11.63 | 822.4804 | | | | 6.23E-01 | 0.08 | 0.37 | 3.17E-01 | 0.22 | 0.60 | 3.78E-01 | 0.19 | 0.60 | 8.98E-01 | -0.02 | 0.48 | 1903409 | 488069 | 1958131 | 697984 | 1887946 | 596144 | 2028317 | 786680 |
| RP_POS_482_32376a10_824 | RP | POS | 10.82 | 482.3238 | | | | 8.80E-02 | 0.33 | 1.09 | 3.17E-01 | 0.21 | 1.03 | 6.60E-02 | 0.43 | 0.87 | 2.61E-01 | 0.22 | 0.95 | 1649781 | 661959 | 1891799 | 759765 | 1815408 | 617255 | 1968190 | 879405 |
| RP_POS_868_73798a0_498 | RP | POS | 0.50 | 868.7380 | | | | 8.48E-01 | -0.04 | 0.84 | 3.17E-01 | -0.20 | 1.21 | 5.38E-01 | -0.14 | 0.71 | 7.95E-01 | 0.06 | 0.79 | 421896 | 211170 | 413653 | 206707 | 434422 | 212674 | 392884 | 200542 |
| RP_POS_1024_77905a0_495 | RP | POS | 0.50 | 1024.7791 | | | | 6.54E-05 | 0.51 | 1.12 | 3.18E-01 | 0.22 | 1.21 | 1.64E-03 | 0.62 | 1.22 | 6.41E-03 | 0.40 | 1.20 | 135812 | 101785 | 307752 | 375613 | 269999 | 311609 | 345505 | 430164 |
| HILIC_POS_315_0802a7_651 | HILIC | POS | 7.651 | 315.0802 | | | | 4.58E-01 | -0.12 | 0.67 | 3.18E-01 | -0.22 | 0.79 | 2.33E-01 | -0.23 | 0.57 | 9.74E-01 | -0.01 | 0.52 | 1308620 | 132963 | 1284060 | 231057 | 1307272 | 235205 | 1260848 | 226810 |
| RP_POS_289_84821a15_571 | RP | POS | 15.57 | 289.8482 | | | | 1.39E-01 | -0.29 | 0.56 | 3.18E-01 | 0.20 | 0.60 | 4.06E-01 | -0.19 | 0.57 | 6.60E-02 | -0.39 | 0.78 | 1550015 | 61933 | 1530727 | 67053 | 1523990 | 60749 | 1537464 | 72808 |
| HILIC_POS_402_23318a1_841 | HILIC | POS | 1.841 | 402.2332 | | | | 8.16E-01 | 0.05 | 1.03 | 3.18E-01 | -0.19 | 0.82 | 8.66E-01 | -0.04 | 0.87 | 5.76E-01 | 0.15 | 1.02 | 911225 | 2908824 | 1043114 | 2274474 | 1271748 | 2710602 | 814480 | 1731472 |
| RP_POS_185_12868a2_461 | RP | POS | 2.46 | 185.1287 | | | | 6.89E-03 | 0.46 | 0.78 | 3.18E-01 | 0.21 | 0.60 | 1.20E-02 | 0.57 | 1.01 | 3.87E-02 | 0.36 | 0.99 | 5189311 | 2205195 | 6584921 | 3167099 | 6266636 | 2337935 | 6903206 | 3820086 |
| RP_POS_449_35907a11_735 | RP | POS | 11.74 | 449.3591 | | | | 1.45E-01 | -0.24 | 0.83 | 3.18E-01 | 0.22 | 0.97 | 4.84E-01 | -0.13 | 0.82 | 9.06E-02 | -0.35 | 0.80 | 835676 | 161298 | 779626 | 252166 | 754313 | 268667 | 804939 | 234485 |
| RP_POS_335_21921a10_71 | RP | POS | 10.71 | 335.2192 | | | | 2.15E-05 | 0.50 | 0.96 | 3.18E-01 | -0.23 | 0.93 | 5.55E-03 | 0.39 | 0.99 | 1.31E-03 | 0.61 | 1.13 | 56340 | 36739 | 428434 | 831976 | 511975 | 944217 | 344893 | 701931 |
| RP_POS_405_25052a11_609 | RP | POS | 11.61 | 405.2505 | | | | 6.30E-03 | -0.58 | 1.12 | 3.18E-01 | 0.19 | 1.06 | 3.14E-02 | -0.49 | 1.07 | 4.39E-03 | -0.68 | 1.06 | 15418712 | 1748978 | 14420808 | 1635606 | 14256735 | 1723692 | 14584881 | 1542386 |
| RP_POS_160_13333a2_502 | RP | POS | 2.50 | 160.1333 | Isomer of pregabalin | 142.122(100), 160.1333(35), 118.0564(65), 97.1012(35) | 3 | 4.07E-02 | 0.24 | 0.57 | 3.18E-01 | 0.23 | 0.78 | 1.16E-01 | 0.36 | 0.62 | 3.07E-02 | 0.13 | 0.71 | 2967642 | 4363342 | 9767942 | 31919587 | 6553358 | 10125879 | 12982526 | 43987393 |
| RP_POS_572_36804a11_004 | RP | POS | 11.00 | 572.3680 | LPC 20:1 [M+Na]+ | 104.1073(100), 513.2985(50), 572.3693(25), 86.0964(14), 146.982(20) | | 6.66E-01 | -0.09 | 0.70 | 3.19E-01 | 0.20 | 1.16 | 9.81E-01 | 0.01 | 0.45 | 4.01E-01 | -0.19 | 0.78 | 1627412 | 739051 | 1563512 | 678124 | 1495534 | 600995 | 1631490 | 747303 |
| RP_POS_780_55072a13_286 | RP | POS | 13.29 | 780.5507 | | | | 5.17E-01 | -0.16 | 0.64 | 3.19E-01 | -0.18 | 0.68 | 3.05E-01 | -0.25 | 0.62 | 8.00E-01 | -0.07 | 0.80 | 26423790 | 37649344 | 21750102 | 25938187 | 24354069 | 33278139 | 19146135 | 15428445 |
| HILIC_POS_87_09223a3_609 | HILIC | POS | 3.609 | 87.0922 | | | | 1.11E-02 | 0.48 | 1.23 | 3.19E-01 | 0.20 | 0.68 | 8.02E-03 | 0.58 | 1.11 | 6.91E-02 | 0.38 | 0.91 | 7004067 | 3420421 | 8902125 | 3997668 | 8501725 | 3826190 | 9302525 | 4161963 |
| RP_POS_320_25598a9_387 | RP | POS | 9.39 | 320.2560 | | | | 3.02E-01 | -0.28 | 0.90 | 3.19E-01 | -0.16 | 0.70 | 1.96E-01 | -0.36 | 0.76 | 4.87E-01 | -0.20 | 0.54 | 5958485 | 9220387 | 4167163 | 5179436 | 4685915 | 5630438 | 3648411 | 4685036 |
| RP_POS_401_26797a10_444 | RP | POS | 10.44 | 401.2680 | | | | 2.25E-01 | 0.23 | 0.58 | 3.19E-01 | 0.20 | 0.60 | 1.30E-01 | 0.34 | 0.61 | 5.39E-01 | 0.13 | 0.50 | 887168 | 260385 | 953945 | 291364 | 924772 | 282295 | 983119 | 300151 |
| RP_POS_749_53638a13_57 | RP | POS | 13.57 | 749.5364 | | | | 9.78E-01 | 0.01 | 0.34 | 3.19E-01 | 0.21 | 0.72 | 6.07E-01 | 0.11 | 0.23 | 6.53E-01 | -0.10 | 0.46 | 6352534 | 546255 | 6355687 | 636954 | 6291923 | 660862 | 6419451 | 612098 |
| HILIC_POS_200_12828a1_024 | HILIC | POS | 1.024 | 200.1283 | | | | 3.18E-01 | 0.12 | 0.47 | 3.20E-01 | -0.23 | 0.55 | 6.69E-01 | 0.00 | 0.17 | 3.20E-01 | 0.23 | 0.51 | 12002 | 21981 | 2199058 | 21771948 | 4388166 | 30788454 | 9950 | 19609 |
| RP_NEG_241_75818a15_587 | RP | NEG | 15.59 | 241.7582 | | | | 1.03E-01 | -0.31 | 0.66 | 3.20E-01 | -0.21 | 0.70 | 4.91E-02 | -0.41 | 0.77 | 3.51E-01 | -0.21 | 0.39 | 16182151 | 509474 | 16003768 | 591284 | 16062908 | 633717 | 15944627 | 545558 |
| HILIC_NEG_162_83911a3_004 | HILIC | NEG | 3.004 | 162.8391 | | | | 9.26E-01 | -0.02 | 0.93 | 3.20E-01 | 0.21 | 1.12 | 7.11E-01 | 0.09 | 0.36 | 5.34E-01 | -0.12 | 0.30 | 53873330 | 11249888 | 53650459 | 13279140 | 52322403 | 10488805 | 54978514 | 15577398 |
| RP_POS_679_43933a10_703 | RP | POS | 10.70 | 679.4393 | | | | 2.84E-01 | -0.19 | 0.71 | 3.20E-01 | 0.21 | 0.66 | 6.90E-01 | -0.08 | 0.42 | 1.58E-01 | -0.29 | 0.61 | 2398591 | 444075 | 2291103 | 613419 | 2229810 | 627843 | 2352395 | 598658 |
| RP_POS_265_25256a10_652 | RP | POS | 10.65 | 265.2526 | | | | 2.72E-03 | -0.58 | 1.04 | 3.20E-01 | 0.20 | 0.86 | 2.26E-02 | -0.48 | 0.99 | 2.40E-03 | -0.68 | 1.09 | 875806 | 121295 | 795842 | 139147 | 781941 | 147003 | 809744 | 130822 |
| RP_POS_872_82965a0_492 | RP | POS | 0.49 | 872.8297 | | | | 1.73E-08 | 0.68 | 1.33 | 3.20E-01 | -0.22 | 1.20 | 2.14E-04 | 0.57 | 1.35 | 2.45E-05 | 0.79 | 1.49 | 51818 | 56305 | 1751620 | 2767651 | 2028079 | 2997991 | 1475160 | 2516382 |
| RP_POS_413_32385a13_01 | RP | POS | 13.01 | 413.3239 | | | | 1.02E-01 | -0.33 | 0.77 | 3.20E-01 | -0.20 | 0.98 | 5.48E-02 | -0.43 | 1.09 | 3.09E-01 | -0.23 | 0.49 | 2492759 | 862201 | 2199429 | 888750 | 2288182 | 912164 | 2110676 | 864727 |
| HILIC_POS_401_26199a0_63 | HILIC | POS | 0.63 | 401.2620 | | | | 4.18E-07 | 0.60 | 1.29 | 3.21E-01 | -0.22 | 0.93 | 8.80E-04 | 0.49 | 1.44 | 1.51E-04 | 0.71 | 1.51 | 53209 | 46685 | 1774020 | 3173415 | 2090889 | 3506165 | 1457152 | 2801525 |
| HILIC_POS_375_32144a0_797 | HILIC | POS | 0.797 | 375.3214 | | | | 6.88E-02 | 0.37 | 0.63 | 3.21E-01 | 0.20 | 0.72 | 4.27E-02 | 0.47 | 0.79 | 2.21E-01 | 0.27 | 0.58 | 4114822 | 1698331 | 4760234 | 1751381 | 4585405 | 1656896 | 4935064 | 1841082 |
| RP_POS_259_13058a8_457 | RP | POS | 8.46 | 259.1306 | Unknown | 259.1289(100) | 4 | 7.49E-01 | 0.07 | 0.51 | 3.21E-01 | -0.20 | 0.65 | 8.77E-01 | -0.03 | 0.34 | 4.79E-01 | 0.17 | 0.53 | 3283847 | 314546 | 3304418 | 314285 | 3335778 | 334419 | 3273059 | 292785 |
| RP_POS_374_15546a4_997 | RP | POS | 5.00 | 374.1555 | | | | 3.22E-03 | 0.43 | 0.90 | 3.21E-01 | 0.22 | 0.78 | 7.04E-03 | 0.54 | 1.05 | 5.16E-02 | 0.32 | 0.65 | 11740831 | 8739604 | 18878557 | 17964085 | 17087351 | 15705645 | 20669762 | 19969664 |
| RP_NEG_338_7814a15_4 | RP | NEG | 15.40 | 338.7814 | | | | 3.59E-01 | 0.25 | 1.03 | 3.21E-01 | -0.16 | 0.60 | 5.33E-01 | 0.17 | 0.52 | 2.59E-01 | 0.33 | 0.74 | 800222 | 84279 | 814697 | 45405 | 819225 | 51949 | 810170 | 37748 |
| RP_POS_398_75006a13_491 | RP | POS | 13.49 | 398.7501 | | | | 2.32E-02 | -0.47 | 0.81 | 3.21E-01 | -0.20 | 0.66 | 1.28E-02 | -0.56 | 1.00 | 1.05E-01 | -0.37 | 0.57 | 763900 | 314374 | 614524 | 315023 | 645916 | 321343 | 583132 | 308596 |
| RP_POS_741_52795a12_763 | RP | POS | 12.76 | 741.5280 | | | | 1.01E-02 | -0.52 | 0.95 | 3.22E-01 | 0.20 | 0.60 | 6.27E-02 | -0.42 | 0.76 | 5.30E-03 | -0.62 | 1.01 | 1045317 | 400414 | 826089 | 417828 | 784464 | 398291 | 867714 | 436523 |
| RP_POS_406_16394a4_275 | RP | POS | 4.28 | 406.1639 | | | | 3.55E-01 | -0.12 | 0.51 | 3.22E-01 | 0.22 | 0.91 | 9.62E-01 | -0.01 | 0.65 | 7.32E-02 | -0.23 | 1.09 | 10015070 | 1503626 | 9539208 | 4387842 | 9101834 | 3017479 | 9976582 | 5422513 |
| RP_POS_166_98486a10_671 | RP | POS | 10.67 | 166.9849 | | | | 5.13E-01 | 0.15 | 0.46 | 3.22E-01 | -0.19 | 1.02 | 8.38E-01 | 0.05 | 0.30 | 3.13E-01 | 0.24 | 0.51 | 11827814 | 3009170 | 12217619 | 2569826 | 12473430 | 2436743 | 11961809 | 2696576 |
| HILIC_POS_446_25934a1_81 | HILIC | POS | 1.81 | 446.2593 | | | | 4.89E-01 | 0.14 | 1.01 | 3.22E-01 | -0.20 | 0.80 | 8.61E-01 | 0.03 | 0.93 | 3.28E-01 | 0.24 | 1.05 | 487544 | 1516476 | 706085 | 1634250 | 868928 | 1978324 | 543241 | 1195061 |
| RP_POS_279_19138a0_543 | RP | POS | 0.54 | 279.1914 | | | | 1.50E-06 | 0.71 | 1.25 | 3.22E-01 | 0.21 | 0.63 | 6.82E-05 | 0.81 | 1.40 | 2.09E-04 | 0.60 | 1.27 | 457517 | 150421 | 665593 | 310463 | 634680 | 261738 | 696506 | 352541 |
| HILIC_POS_160_13326a1_725 | HILIC | POS | 1.725 | 160.1333 | | | | 3.22E-01 | 0.11 | 0.74 | 3.22E-01 | 0.23 | 0.83 | 3.23E-01 | 0.23 | 0.59 | 1.87E-02 | 0.00 | 1.03 | 106422 | 40865 | 5277106 | 51935307 | 81943 | 50791 | 10472270 | 73447362 |
| RP_POS_223_09433a15_227 | RP | POS | 15.23 | 223.0943 | | | | 2.67E-01 | -0.22 | 0.45 | 3.22E-01 | -0.20 | 0.72 | 1.37E-01 | -0.32 | 0.60 | 5.94E-01 | -0.12 | 0.50 | 19516373 | 8720914 | 17518760 | 8941550 | 18408634 | 9761004 | 16628887 | 8039965 |
| RP_POS_139_12296a0_71 | RP | POS | 0.71 | 139.1230 | | | | 1.15E-01 | 0.29 | 0.70 | 3.22E-01 | 0.21 | 0.64 | 6.67E-02 | 0.39 | 0.81 | 3.79E-01 | 0.18 | 0.31 | 340208 | 26126 | 349287 | 33241 | 345980 | 32540 | 352593 | 33931 |
| RP_POS_158_96167a0_808 | RP | POS | 0.81 | 158.9617 | | | | 8.64E-01 | 0.03 | 0.28 | 3.22E-01 | 0.20 | 0.83 | 5.37E-01 | 0.13 | 0.28 | 7.83E-01 | -0.07 | 0.27 | 3943593 | 1568852 | 3998357 | 1581817 | 3840994 | 1736732 | 4155721 | 1410170 |
| RP_NEG_414_20816a11_611 | RP | NEG | 11.61 | 414.2082 | | | | 3.64E-01 | -0.17 | 0.60 | 3.22E-01 | 0.20 | 0.86 | 7.28E-01 | -0.07 | 0.56 | 2.23E-01 | -0.28 | 0.79 | 512618 | 74407 | 498339 | 83968 | 489987 | 91355 | 506692 | 75875 |
| RP_POS_441_29758a12_243 | RP | POS | 12.24 | 441.2976 | | | | 9.85E-01 | 0.00 | 0.51 | 3.23E-01 | 0.20 | 0.78 | 6.91E-01 | 0.10 | 0.43 | 6.42E-01 | -0.10 | 0.25 | 24240382 | 11358774 | 24197671 | 11021875 | 23102060 | 9777012 | 25293282 | 12140963 |
| HILIC_NEG_157_90117a3_943 | HILIC | NEG | 3.943 | 157.9012 | | | | 5.11E-01 | 0.12 | 0.41 | 3.23E-01 | -0.21 | 0.58 | 9.24E-01 | 0.02 | 0.16 | 2.89E-01 | 0.23 | 0.70 | 1500579 | 454936 | 1564590 | 537357 | 1617971 | 530069 | 1511210 | 544618 |
| RP_POS_253_09706a5_577 | RP | POS | 5.58 | 253.0971 | Isomer of Carbamazepine-10,11-epoxide | 180.0811(100), 210.092(95), 253.0961(35), 182.0972(20) | 3 | 3.21E-01 | 0.11 | 0.75 | 3.23E-01 | -0.23 | 0.78 | 7.38E-01 | 0.00 | 0.62 | 3.23E-01 | 0.23 | 0.83 | 15296 | 9290 | 1080549 | 10677040 | 2146509 | 15099863 | 14590 | 9290 |
| HILIC_POS_139_123a0_555 | HILIC | POS | 0.555 | 139.1230 | | | | 1.22E-02 | -0.54 | 1.41 | 3.23E-01 | 0.19 | 0.71 | 5.54E-02 | -0.45 | 0.79 | 7.15E-03 | -0.64 | 1.27 | 9208154 | 2908793 | 7702753 | 2644977 | 7440061 | 2639786 | 7965445 | 2650524 |
| RP_POS_770_50848a13_248 | RP | POS | 13.25 | 770.5085 | | | | 8.79E-01 | 0.03 | 0.93 | 3.23E-01 | 0.21 | 0.61 | 5.67E-01 | 0.13 | 0.70 | 6.25E-01 | -0.08 | 0.45 | 1151829 | 393749 | 1165580 | 571765 | 1108706 | 378224 | 1222453 | 714736 |
| RP_POS_362_3053a10_423 | RP | POS | 10.42 | 362.3053 | | | | 9.82E-01 | 0.00 | 0.36 | 3.23E-01 | -0.22 | 0.76 | 5.17E-01 | -0.11 | 0.30 | 6.34E-01 | 0.10 | 0.44 | 985226 | 1271596 | 978299 | 1999698 | 1176982 | 2351013 | 779615 | 1572057 |
| RP_POS_303_23187a10_423 | RP | POS | 10.42 | 303.2319 | | | | 5.84E-01 | 0.09 | 0.40 | 3.23E-01 | -0.22 | 0.77 | 8.85E-01 | -0.02 | 0.26 | 3.74E-01 | 0.19 | 0.52 | 2063618 | 3036834 | 2477401 | 5281206 | 3002229 | 6377067 | 1952573 | 3888749 |
| RP_POS_130_96298a15_441 | RP | POS | 15.44 | 130.9630 | | | | 4.76E-01 | -0.14 | 0.46 | 3.23E-01 | 0.20 | 0.59 | 8.71E-01 | -0.04 | 0.34 | 2.50E-01 | -0.24 | 0.59 | 13152164 | 2240326 | 12817394 | 2464870 | 12572715 | 2150617 | 13062073 | 2743689 |
| HILIC_POS_133_09724a7_152 | HILIC | POS | 7.152 | 133.0972 | | | | 1.38E-05 | 0.56 | 1.07 | 3.24E-01 | 0.22 | 0.83 | 9.53E-04 | 0.67 | 1.15 | 1.13E-03 | 0.46 | 1.16 | 2275957 | 1130709 | 4184652 | 3720640 | 3815254 | 2874278 | 4554050 | 4407821 |
| RP_NEG_876_82288a0_486 | RP | NEG | 0.49 | 876.8229 | | | | 6.29E-03 | 0.36 | 1.07 | 3.24E-01 | 0.22 | 1.26 | 1.21E-02 | 0.47 | 1.12 | 1.19E-01 | 0.25 | 1.13 | 2978744 | 521078 | 3473876 | 1525995 | 3322474 | 1396292 | 3625277 | 1645732 |
| RP_POS_513_84302a11_759 | RP | POS | 11.76 | 513.8430 | | | | 3.35E-01 | 0.11 | 0.38 | 3.24E-01 | -0.23 | 0.75 | 9.26E-01 | 0.00 | 0.48 | 3.27E-01 | 0.23 | 0.44 | 120824 | 75876 | 236275 | 1184469 | 354308 | 1666407 | 118241 | 170936 |

**Supplementary table 1: Metabolomics results** (in asceding order according to p-value from progressive ALD vs  non-progressive ALD comparison, with molecular features with ID_level of 1-2 first)

| | | | | | | | | all_ALD_vs_control | | | Prog_ALD_vs_non-prog_ALD | | | Prog_ALD_vs_control_ALD | | | Non-prog_ALD_vs_control | | | Healthy_control | | all_ALD | | Non-prog_ALD | | Progressive_ALD | |
|---|---|---|---|---|---|---|---|---|---|---|---|---|---|---|---|---|---|---|---|---|---|---|---|---|---|---|---|
| | | | | | | | | t-test | Cohen's | PLS-DA | t-test | Cohen's | PLS-DA | t-test | Cohen's | PLS-DA | t-test | Cohen's | PLS-DA | | | | | | | | |
| Feature_ID | RP_HILIC | POS_NEG | Average.Rt | Average.Mz | Metabolite.name | curated_MSMS | ID_level | p | d | VIP | p | d | VIP | p | d | VIP | p | d | VIP | Mean | SD | Mean | SD | Mean | SD | Mean | SD |
| RP_POS_760_57599a10_906 | RP | POS | 10.91 | 760.5760 | | | | 9.63E-03 | -0.55 | 1.10 | 3.24E-01 | 0.19 | 1.17 | 5.84E-02 | -0.45 | 0.82 | 4.06E-03 | -0.64 | 1.13 | 821463 | 234389 | 694846 | 222587 | 672765 | 193462 | 716927 | 248353 |
| RP_POS_594_5993a11_168 | RP | POS | 11.17 | 594.5993 | | | | 3.34E-07 | 0.83 | 1.63 | 3.24E-01 | -0.20 | 1.02 | 1.96E-04 | 0.73 | 1.40 | 1.07E-06 | 0.93 | 1.73 | 995419 | 275750 | 1362556 | 451008 | 1407283 | 430226 | 1317828 | 470947 |
| RP_POS_414_30035a11_183 | RP | POS | 11.18 | 414.3004 | | | | 2.97E-01 | 0.19 | 0.84 | 3.24E-01 | 0.21 | 0.86 | 1.76E-01 | 0.29 | 0.71 | 6.67E-01 | 0.09 | 0.53 | 573976 | 787096 | 752477 | 974186 | 655909 | 913985 | 849046 | 1031041 |
| HILIC_POS_194_02217a4_624 | HILIC | POS | 4.624 | 194.0222 | | | | 5.36E-01 | -0.08 | 0.61 | 3.24E-01 | 0.22 | 0.66 | 9.05E-01 | 0.03 | 0.26 | 5.33E-02 | -0.20 | 1.34 | 701766 | 118164 | 678785 | 306020 | 648358 | 122711 | 709211 | 415042 |
| HILIC_NEG_99_92564a7_352 | HILIC | NEG | 7.352 | 99.9256 | | | | 1.83E-03 | -0.48 | 0.74 | 3.25E-01 | -0.21 | 0.63 | 3.81E-03 | -0.59 | 0.94 | 3.06E-02 | -0.38 | 0.76 | 2845993 | 312809 | 2594241 | 557568 | 2649436 | 495671 | 2539046 | 613353 |
| RP_POS_445_29245a10_448 | RP | POS | 10.45 | 445.2925 | | | | 1.48E-01 | 0.29 | 0.62 | 3.25E-01 | -0.20 | 0.73 | 3.94E-01 | 0.19 | 0.59 | 7.99E-02 | 0.39 | 0.82 | 1253569 | 442019 | 1386394 | 454907 | 1431411 | 441309 | 1341377 | 468204 |
| RP_POS_317_91235a15_381 | RP | POS | 15.38 | 317.9124 | | | | 6.05E-01 | 0.11 | 0.32 | 3.25E-01 | 0.20 | 0.70 | 3.48E-01 | 0.21 | 0.53 | 9.79E-01 | 0.01 | 0.31 | 1519909 | 210055 | 1542167 | 211872 | 1521198 | 233172 | 1563135 | 188226 |
| RP_POS_351_22906a10_907 | RP | POS | 10.91 | 351.2291 | | | | 1.14E-02 | 0.35 | 1.43 | 3.25E-01 | 0.22 | 0.87 | 2.22E-02 | 0.46 | 1.15 | 1.13E-01 | 0.24 | 1.25 | 24316962 | 9844275 | 31811351 | 23410049 | 29494786 | 19215358 | 34127915 | 26964109 |
| RP_POS_337_1821a11_243 | RP | POS | 11.24 | 337.1821 | | | | 1.59E-03 | 0.57 | 1.34 | 3.25E-01 | 0.20 | 1.03 | 2.73E-03 | 0.67 | 1.33 | 1.37E-02 | 0.46 | 1.18 | 1062878 | 280616 | 1266301 | 369172 | 1229774 | 307864 | 1302828 | 421727 |
| RP_POS_266_17313a11_608 | RP | POS | 11.61 | 266.1731 | | | | 4.61E-02 | -0.37 | 0.86 | 3.25E-01 | 0.20 | 0.78 | 1.71E-01 | -0.27 | 0.65 | 3.62E-02 | -0.48 | 0.93 | 1038459 | 115026 | 988574 | 136731 | 975051 | 153516 | 1002097 | 117608 |
| RP_NEG_563_505a11_254 | RP | NEG | 11.25 | 563.5050 | | | | 5.36E-07 | 0.74 | 1.61 | 3.25E-01 | 0.21 | 1.00 | 1.20E-05 | 0.84 | 1.61 | 3.40E-04 | 0.63 | 1.53 | 615472 | 525315 | 1379439 | 1093283 | 1271355 | 1047337 | 1487522 | 1137617 |
| HILIC_NEG_524_27826a1_681 | HILIC | NEG | 1.681 | 524.2783 | | | | 1.69E-02 | 0.39 | 0.73 | 3.25E-01 | -0.21 | 0.74 | 1.39E-01 | 0.29 | 0.51 | 1.25E-02 | 0.50 | 0.93 | 368779 | 135812 | 447223 | 214035 | 468382 | 217136 | 426064 | 210938 |
| RP_NEG_194_08148a8_528 | RP | NEG | 8.53 | 194.0815 | | | | 4.72E-01 | -0.18 | 0.63 | 3.25E-01 | -0.17 | 1.03 | 3.07E-01 | -0.27 | 0.65 | 7.31E-01 | -0.09 | 0.31 | 940301 | 55991 | 932617 | 37758 | 936349 | 40656 | 928884 | 34629 |
| RP_POS_265_2532a11_254 | RP | POS | 11.25 | 265.2532 | | | | 5.20E-06 | 0.84 | 1.74 | 3.25E-01 | 0.19 | 1.11 | 1.78E-05 | 0.94 | 1.70 | 1.49E-04 | 0.74 | 1.65 | 1130437 | 499262 | 1668336 | 627500 | 1606306 | 562037 | 1730366 | 686885 |
| RP_POS_553_45984a11_162 | RP | POS | 11.16 | 553.4598 | | | | 2.32E-01 | -0.22 | 0.63 | 3.26E-01 | 0.21 | 0.67 | 5.72E-01 | -0.12 | 0.52 | 1.38E-01 | -0.32 | 0.58 | 16752261 | 2299267 | 16153222 | 2859754 | 15870696 | 3007816 | 16435747 | 2704336 |
| RP_NEG_158_9778a15_298 | RP | NEG | 15.30 | 158.9778 | | | | 7.68E-01 | -0.05 | 0.61 | 3.26E-01 | -0.21 | 0.61 | 4.35E-01 | -0.16 | 0.52 | 8.23E-01 | 0.05 | 0.34 | 16106937 | 445085 | 16078494 | 551229 | 16132931 | 600635 | 16024056 | 497089 |
| HILIC_NEG_195_741a3_034 | HILIC | NEG | 3.034 | 195.7410 | | | | 4.83E-02 | 0.25 | 0.88 | 3.26E-01 | 0.22 | 0.79 | 2.38E-02 | 0.36 | 1.14 | 4.42E-01 | 0.14 | 0.79 | 564134 | 33568 | 590594 | 118692 | 578879 | 127956 | 602309 | 108675 |
| RP_POS_386_15237a9_047 | RP | POS | 9.05 | 386.1524 | | | | 5.66E-04 | -0.74 | 1.15 | 3.26E-01 | -0.18 | 0.81 | 2.48E-04 | -0.83 | 1.38 | 6.21E-03 | -0.65 | 0.96 | 5526114 | 352143 | 5267055 | 325605 | 5299181 | 358818 | 5234929 | 288694 |
| HILIC_POS_399_24649a0_634 | HILIC | POS | 0.634 | 399.2465 | | | | 3.16E-06 | 0.57 | 1.29 | 3.27E-01 | -0.22 | 0.94 | 1.88E-03 | 0.46 | 1.46 | 3.82E-04 | 0.68 | 1.47 | 275972 | 752339 | 2558223 | 4462126 | 2998264 | 4981767 | 2118182 | 3874828 |
| RP_POS_478_38898a11_841 | RP | POS | 11.84 | 478.3890 | | | | 7.94E-01 | 0.06 | 0.40 | 3.27E-01 | 0.19 | 0.69 | 5.45E-01 | 0.15 | 0.40 | 8.90E-01 | -0.03 | 0.69 | 100295351 | 48353140 | 102763084 | 39176963 | 98902968 | 36396108 | 106623201 | 41784043 |
| HILIC_NEG_266_91565a0_882 | HILIC | NEG | 0.882 | 266.9157 | | | | 1.11E-01 | 0.30 | 0.98 | 3.27E-01 | 0.20 | 0.89 | 7.18E-02 | 0.40 | 0.72 | 3.30E-01 | 0.20 | 0.50 | 597745 | 143978 | 647112 | 167957 | 630564 | 153873 | 663661 | 180994 |
| RP_NEG_327_25452a11_254 | RP | NEG | 11.25 | 327.2545 | | | | 4.19E-07 | 0.76 | 1.65 | 3.27E-01 | 0.21 | 0.99 | 9.62E-06 | 0.86 | 1.63 | 2.15E-04 | 0.65 | 1.58 | 1313397 | 713322 | 2334412 | 1409898 | 2195529 | 1336591 | 2473295 | 1479983 |
| RP_NEG_283_70932a15_59 | RP | NEG | 15.59 | 283.7093 | | | | 6.08E-01 | 0.10 | 1.36 | 3.27E-01 | -0.20 | 0.66 | 9.86E-01 | 0.00 | 0.71 | 3.61E-01 | 0.20 | 1.23 | 1108952 | 46577 | 1114019 | 53458 | 1119284 | 54113 | 1108754 | 52811 |
| RP_NEG_236_84775a15_584 | RP | NEG | 15.58 | 236.8478 | | | | 2.37E-03 | -0.60 | 1.17 | 3.27E-01 | -0.19 | 0.60 | 1.62E-03 | -0.70 | 1.17 | 2.05E-02 | -0.51 | 1.08 | 811981 | 33912 | 789639 | 36443 | 793228 | 36479 | 786051 | 36419 |
| RP_POS_376_29688a11_67 | RP | POS | 11.67 | 376.2969 | | | | 9.90E-01 | 0.00 | 0.64 | 3.27E-01 | -0.20 | 0.61 | 6.56E-01 | -0.10 | 0.42 | 6.72E-01 | 0.10 | 0.36 | 13177311 | 7518375 | 13157454 | 7562627 | 13901924 | 7560889 | 12412983 | 7566751 |
| RP_POS_303_22903a10_978 | RP | POS | 10.98 | 303.2290 | | | | 1.59E-02 | 0.41 | 1.29 | 3.27E-01 | -0.21 | 1.38 | 1.26E-01 | 0.30 | 1.07 | 1.07E-02 | 0.51 | 1.22 | 15885900 | 6220578 | 19406387 | 9102218 | 20302344 | 9065929 | 18510431 | 9141238 |
| RP_POS_327_19391a15_431 | RP | POS | 15.43 | 327.1939 | | | | 6.57E-01 | -0.10 | 1.12 | 3.28E-01 | 0.19 | 0.75 | 9.76E-01 | -0.01 | 0.55 | 4.29E-01 | -0.19 | 1.00 | 3726197 | 1527680 | 3593091 | 1251160 | 3469956 | 1239492 | 3716227 | 1263118 |
| HILIC_POS_586_72632a0_549 | HILIC | POS | 0.549 | 586.7263 | | | | 1.22E-06 | 0.62 | 1.34 | 3.28E-01 | -0.22 | 0.92 | 7.73E-04 | 0.51 | 1.49 | 1.58E-04 | 0.73 | 1.51 | 51994 | 192930 | 443592 | 689094 | 511425 | 766966 | 375758 | 601469 |
| HILIC_NEG_331_65601a1_363 | HILIC | NEG | 1.363 | 331.6560 | | | | 1.03E-07 | 0.85 | 1.36 | 3.28E-01 | -0.20 | 0.67 | 2.84E-04 | 0.75 | 1.20 | 5.59E-08 | 0.95 | 1.86 | 3690301 | 966896 | 5060858 | 1640536 | 5222326 | 1339627 | 4899391 | 1894686 |
| RP_NEG_243_06291a1_341 | RP | NEG | 1.34 | 243.0629 | | | | 9.64E-01 | -0.01 | 0.60 | 3.28E-01 | -0.21 | 0.85 | 5.87E-01 | -0.11 | 0.57 | 6.39E-01 | 0.10 | 0.67 | 630810 | 207656 | 628725 | 277394 | 656011 | 275700 | 601438 | 279169 |
| HILIC_POS_181_05247a1_514 | HILIC | POS | 1.514 | 181.0525 | | | | 7.59E-01 | 0.06 | 1.00 | 3.28E-01 | 0.20 | 0.99 | 4.90E-01 | 0.16 | 0.26 | 8.25E-01 | -0.04 | 0.62 | 439946 | 122061 | 447916 | 142575 | 433897 | 117335 | 461936 | 164016 |
| RP_POS_200_0401a0_507 | RP | POS | 0.51 | 200.0401 | | | | 5.95E-05 | 0.51 | 1.18 | 3.28E-01 | -0.22 | 1.25 | 7.05E-03 | 0.40 | 1.23 | 1.38E-03 | 0.62 | 1.30 | 369954 | 88360 | 518725 | 322411 | 550423 | 363080 | 487026 | 275938 |
| RP_POS_481_81564a0_246 | RP | POS | 0.25 | 481.8156 | | | | 9.46E-02 | 0.28 | 0.61 | 3.29E-01 | 0.21 | 0.67 | 7.87E-02 | 0.39 | 0.87 | 3.16E-01 | 0.18 | 0.70 | 360651 | 161201 | 423163 | 235020 | 400094 | 188863 | 446233 | 273570 |
| RP_POS_1090_71948a0_495 | RP | POS | 0.50 | 1090.7195 | | | | 2.34E-06 | -0.61 | 1.29 | 3.29E-01 | 0.21 | 1.20 | 4.34E-03 | -0.51 | 1.26 | 2.88E-05 | -0.72 | 1.44 | 975786 | 96328 | 798201 | 316465 | 767164 | 304105 | 829237 | 328480 |
| RP_NEG_369_17453a8_265 | RP | NEG | 8.27 | 369.1745 | | | | 2.20E-06 | 0.60 | 1.19 | 3.29E-01 | 0.22 | 0.89 | 5.28E-04 | 0.71 | 1.17 | 3.20E-04 | 0.49 | 1.14 | 6204431 | 5410227 | 18006186 | 21767943 | 15869603 | 16636370 | 20142769 | 25908915 |
| HILIC_POS_136_07585a5_119 | HILIC | POS | 5.119 | 136.0759 | | | | 4.50E-01 | 0.14 | 0.91 | 3.30E-01 | 0.21 | 1.13 | 2.80E-01 | 0.24 | 0.42 | 8.69E-01 | 0.03 | 0.41 | 1024089 | 328094 | 1078783 | 426094 | 1037015 | 372180 | 1120552 | 474066 |
| RP_POS_412_77649a13_998 | RP | POS | 14.00 | 412.7765 | | | | 7.32E-01 | 0.08 | 0.52 | 3.30E-01 | 0.19 | 0.73 | 4.86E-01 | 0.17 | 0.51 | 9.40E-01 | -0.02 | 0.35 | 7984122 | 2201254 | 8132935 | 1880596 | 7948657 | 1834160 | 8317213 | 1926659 |
| RP_NEG_375_13141a10_705 | RP | NEG | 10.71 | 375.1314 | | | | 4.45E-03 | 0.51 | 1.29 | 3.30E-01 | 0.20 | 1.01 | 5.70E-03 | 0.61 | 1.28 | 3.19E-02 | 0.41 | 1.11 | 611554 | 78494 | 662289 | 102251 | 652267 | 87650 | 672311 | 115050 |
| HILIC_NEG_100_9334a4_163 | HILIC | NEG | 4.163 | 100.9334 | | | | 9.82E-02 | 0.36 | 1.03 | 3.30E-01 | 0.19 | 1.24 | 4.94E-02 | 0.46 | 1.03 | 2.75E-01 | 0.27 | 0.64 | 30886573 | 8241287 | 33636684 | 7316750 | 32919861 | 8025474 | 34353507 | 6534930 |
| RP_POS_635_41174a10_704 | RP | POS | 10.70 | 635.4117 | | | | 1.02E-01 | -0.31 | 0.65 | 3.30E-01 | 0.20 | 0.58 | 3.33E-01 | -0.21 | 0.41 | 5.33E-02 | -0.41 | 0.76 | 3441277 | 731524 | 3186075 | 832084 | 3104628 | 794430 | 3267522 | 868447 |
| HILIC_NEG_144_92345a6_105 | HILIC | NEG | 6.105 | 144.9235 | | | | 3.11E-01 | 0.22 | 0.88 | 3.30E-01 | 0.19 | 0.78 | 1.76E-01 | 0.31 | 0.89 | 6.08E-01 | 0.12 | 0.64 | 33409287 | 11600986 | 35763732 | 10422230 | 34743913 | 11151485 | 36783550 | 9642843 |
| HILIC_NEG_249_04369a0_928 | HILIC | NEG | 0.928 | 249.0437 | | | | 1.10E-05 | 0.61 | 0.97 | 3.31E-01 | -0.21 | 1.23 | 6.42E-03 | 0.50 | 0.86 | 4.52E-05 | 0.71 | 1.29 | 128933 | 128624 | 310565 | 324566 | 342313 | 307626 | 278817 | 340802 |
| HILIC_NEG_115_0207a1_299 | HILIC | NEG | 1.299 | 115.0207 | | | | 3.39E-08 | 0.87 | 1.53 | 3.31E-01 | 0.20 | 0.84 | 1.13E-06 | 0.97 | 1.57 | 2.13E-05 | 0.77 | 1.43 | 2376320 | 696803 | 3424191 | 1232072 | 3303689 | 1160807 | 3544693 | 1299951 |
| RP_NEG_457_59796a15_583 | RP | NEG | 15.58 | 457.5980 | | | | 1.35E-02 | -0.49 | 0.97 | 3.31E-01 | -0.19 | 0.65 | 7.50E-03 | -0.59 | 1.00 | 7.63E-02 | -0.40 | 0.86 | 953162 | 39982 | 932130 | 42305 | 936265 | 43732 | 927995 | 40848 |
| RP_POS_217_10445a13_011 | RP | POS | 13.01 | 217.1045 | | | | 2.00E-01 | -0.25 | 0.44 | 3.31E-01 | -0.20 | 0.59 | 1.11E-01 | -0.35 | 0.61 | 4.93E-01 | -0.15 | 0.64 | 3545696 | 959569 | 3287750 | 1042013 | 3389558 | 1060614 | 3185943 | 1023567 |
| HILIC_POS_368_27975a0_643 | HILIC | POS | 0.643 | 368.2798 | | | | 6.85E-01 | -0.07 | 0.69 | 3.31E-01 | -0.21 | 1.05 | 3.29E-01 | -0.18 | 0.37 | 8.90E-01 | 0.03 | 0.44 | 4305974 | 2180921 | 4109357 | 2906060 | 4393538 | 3521941 | 3825175 | 2119868 |
| RP_NEG_413_65027a15_582 | RP | NEG | 15.58 | 413.6503 | | | | 1.43E-01 | -0.32 | 0.63 | 3.31E-01 | -0.19 | 0.66 | 7.79E-02 | -0.42 | 0.78 | 3.49E-01 | -0.23 | 0.54 | 902561 | 43816 | 889697 | 38364 | 893444 | 40836 | 885951 | 35741 |
| RP_POS_457_34958a10_941 | RP | POS | 10.94 | 457.3496 | | | | 7.61E-01 | -0.06 | 0.48 | 3.32E-01 | 0.19 | 0.63 | 8.94E-01 | 0.03 | 0.21 | 4.85E-01 | -0.16 | 0.58 | 37691034 | 6894199 | 37270225 | 6438854 | 36641673 | 6088736 | 37898777 | 6773832 |
| HILIC_NEG_234_82024a5_49 | HILIC | NEG | 5.49 | 234.8202 | | | | 7.77E-01 | -0.06 | 0.19 | 3.32E-01 | 0.19 | 0.85 | 8.97E-01 | 0.03 | 0.20 | 5.17E-01 | -0.16 | 0.25 | 995247 | 381658 | 973888 | 328162 | 941856 | 327313 | 1005920 | 329166 |
| RP_POS_832_81879a0_472 | RP | POS | 0.47 | 832.8188 | | | | 2.10E-09 | -0.80 | 1.41 | 3.32E-01 | 0.21 | 1.17 | 8.57E-05 | -0.70 | 1.42 | 3.46E-07 | -0.91 | 1.58 | 374691 | 56000 | 253427 | 159968 | 237815 | 155947 | 269039 | 163967 |
| RP_POS_300_20163a3_417 | RP | POS | 3.42 | 300.2016 | | | | 5.33E-01 | 0.14 | 1.13 | 3.32E-01 | -0.18 | 1.17 | 8.26E-01 | 0.05 | 0.98 | 3.62E-01 | 0.24 | 1.03 | 1272570 | 3157781 | 1656291 | 2486877 | 1899026 | 2779140 | 1413557 | 2156726 |
| RP_POS_564_18536a2_724 | RP | POS | 2.72 | 564.1854 | | | | 9.71E-01 | 0.01 | 0.64 | 3.32E-01 | 0.22 | 0.70 | 6.06E-01 | 0.11 | 0.75 | 4.59E-01 | -0.10 | 0.31 | 2597582 | 621137 | 2603716 | 1276617 | 2478951 | 818071 | 2728482 | 1609893 |
| RP_NEG_858_8031a0_492 | RP | NEG | 0.49 | 858.8031 | | | | 1.92E-09 | -0.76 | 1.40 | 3.32E-01 | 0.21 | 1.22 | 1.49E-04 | -0.66 | 1.39 | 7.71E-07 | -0.87 | 1.59 | 2042237 | 215060 | 1443516 | 843226 | 1361263 | 828123 | 1525768 | 858474 |
| RP_NEG_207_10272a7_77 | RP | NEG | 7.77 | 207.1027 | | | | 9.16E-02 | -0.35 | 0.82 | 3.32E-01 | 0.19 | 0.64 | 2.52E-01 | -0.25 | 0.49 | 6.04E-02 | -0.45 | 0.99 | 3118403 | 139285 | 3069911 | 137602 | 3056491 | 149249 | 3083331 | 124949 |
| RP_POS_561_81549a10_932 | RP | POS | 10.93 | 561.8155 | | | | 1.81E-03 | -0.69 | 1.38 | 3.32E-01 | -0.18 | 1.05 | 9.53E-04 | -0.78 | 1.41 | 1.20E-02 | -0.60 | 1.29 | 401387 | 165319 | 293759 | 142931 | 307692 | 151100 | 279826 | 134344 |
| RP_NEG_300_99033a7_983 | RP | NEG | 7.98 | 300.9903 | | | | 1.21E-01 | -0.32 | 0.70 | 3.32E-01 | -0.19 | 0.61 | 6.35E-02 | -0.42 | 0.71 | 3.37E-01 | -0.22 | 0.58 | 2758449 | 164911 | 2705958 | 161695 | 2721712 | 172018 | 2690205 | 150755 |
| RP_POS_261_14609a9_687 | RP | POS | 9.69 | 261.1461 | | | | 7.05E-01 | -0.06 | 0.92 | 3.33E-01 | 0.21 | 0.61 | 8.56E-01 | 0.04 | 0.55 | 2.68E-01 | -0.17 | 0.96 | 1522653 | 731212 | 1457688 | 1119732 | 1348348 | 615085 | 1567027 | 1459609 |
| HILIC_POS_336_05252a3_077 | HILIC | POS | 3.077 | 336.0525 | | | | 3.40E-01 | 0.13 | 1.21 | 3.33E-01 | -0.22 | 0.98 | 9.13E-01 | 0.02 | 1.22 | 1.85E-01 | 0.24 | 1.32 | 846890 | 51577 | 862979 | 140914 | 876705 | 143745 | 849253 | 138099 |
| HILIC_NEG_393_664a1_364 | HILIC | NEG | 1.364 | 393.6640 | | | | 5.17E-05 | 0.66 | 1.04 | 3.33E-01 | -0.20 | 0.66 | 7.46E-03 | 0.56 | 0.89 | 1.94E-05 | 0.76 | 1.46 | 138553 | 57918 | 197377 | 92365 | 206377 | 76552 | 188377 | 105883 |
| RP_POS_499_82706a4_05 | RP | POS | 4.05 | 499.8271 | | | | 7.33E-01 | -0.08 | 0.36 | 3.33E-01 | 0.18 | 0.65 | 9.66E-01 | 0.01 | 0.23 | 4.84E-01 | -0.17 | 0.32 | 2389612 | 2254460 | 2240524 | 1742035 | 2070814 | 1502396 | 2410234 | 1953287 |
| RP_NEG_519_40698a13_286 | RP | NEG | 13.29 | 519.4070 | | | | 2.56E-01 | -0.22 | 0.75 | 3.33E-01 | 0.20 | 0.60 | 6.03E-01 | -0.12 | 0.60 | 1.21E-01 | -0.32 | 0.67 | 9940443 | 3036539 | 9213031 | 3380984 | 8883735 | 2857407 | 9542326 | 3835274 |
| RP_POS_383_16611a10_907 | RP | POS | 10.91 | 383.1661 | | | | 1.93E-01 | 0.19 | 1.35 | 3.33E-01 | 0.22 | 0.93 | 1.31E-01 | 0.30 | 1.04 | 6.25E-01 | 0.08 | 1.11 | 269289 | 143151 | 320044 | 293637 | 291458 | 264133 | 348631 | 320579 |
| RP_POS_585_33899a14_949 | RP | POS | 14.95 | 585.3390 | | | | 6.29E-03 | 0.42 | 0.76 | 3.33E-01 | 0.21 | 0.59 | 1.64E-02 | 0.53 | 0.82 | 3.83E-02 | 0.32 | 0.72 | 1574142 | 354467 | 1822851 | 632141 | 1761267 | 444715 | 1884435 | 775789 |
| RP_POS_392_7319a12_532 | RP | POS | 12.53 | 392.7319 | | | | 5.76E-01 | 0.11 | 0.74 | 3.33E-01 | 0.20 | 0.58 | 3.68E-01 | 0.21 | 0.75 | 9.55E-01 | 0.01 | 0.15 | 1365859 | 606190 | 1436285 | 646190 | 1373401 | 572833 | 1499169 | 712346 |
| RP_NEG_428_98193a7_73 | RP | NEG | 7.73 | 428.9819 | | | | 6.37E-01 | -0.10 | 0.33 | 3.34E-01 | 0.19 | 0.59 | 9.95E-01 | 0.00 | 0.61 | 4.17E-01 | -0.19 | 0.36 | 3564715 | 399051 | 3526448 | 388647 | 3488683 | 429227 | 3564212 | 343554 |
| RP_POS_357_27859a9_205 | RP | POS | 9.21 | 357.2786 | | | | 3.23E-01 | 0.17 | 0.77 | 3.34E-01 | 0.21 | 0.61 | 2.35E-01 | 0.28 | 0.66 | 6.92E-01 | 0.07 | 0.80 | 321338 | 328986 | 394793 | 455274 | 350507 | 315010 | 439078 | 561734 |
| HILIC_POS_616_41132a0_558 | HILIC | POS | 0.558 | 616.4113 | | | | 1.88E-06 | 0.62 | 1.32 | 3.34E-01 | -0.21 | 0.90 | 8.57E-04 | 0.51 | 1.48 | 2.04E-04 | 0.72 | 1.49 | 64024 | 227343 | 501257 | 778031 | 576846 | 870151 | 425668 | 673940 |
| HILIC_NEG_134_89471a4_164 | HILIC | NEG | 4.164 | 134.8947 | | | | 8.76E-01 | 0.03 | 0.47 | 3.34E-01 | 0.19 | 1.05 | 6.00E-01 | 0.13 | 0.59 | 7.91E-01 | -0.06 | 0.10 | 15674454 | 8198277 | 15929230 | 7368484 | 15213953 | 6674571 | 16644507 | 8006444 |
| RP_POS_369_19876a11_161 | RP | POS | 11.16 | 369.1988 | | | | 7.03E-01 | 0.07 | 0.70 | 3.35E-01 | 0.20 | 0.81 | 4.43E-01 | 0.17 | 0.64 | 8.70E-01 | -0.03 | 0.67 | 2024818 | 338334 | 2052856 | 423419 | 2011767 | 368973 | 2093945 | 471848 |
| RP_NEG_473_40158a13_286 | RP | NEG | 13.29 | 473.4016 | | | | 1.28E-01 | -0.29 | 0.80 | 3.35E-01 | 0.20 | 0.60 | 3.97E-01 | -0.19 | 0.65 | 5.86E-02 | -0.39 | 0.78 | 8303750 | 2414554 | 7521820 | 2736230 | 7256319 | 2386443 | 7787321 | 3047578 |
| HILIC_NEG_327_65942a1_363 | HILIC | NEG | 1.363 | 327.6594 | | | | 5.24E-08 | 0.86 | 1.38 | 3.35E-01 | -0.20 | 0.67 | 2.11E-04 | 0.76 | 1.22 | 3.03E-08 | 0.96 | 1.89 | 1038917 | 266597 | 1429415 | 459916 | 1474046 | 373520 | 1384784 | 532711 |
| RP_POS_270_31525a9_493 | RP | POS | 9.49 | 270.3153 | | | | 2.38E-02 | -0.39 | 0.63 | 3.35E-01 | 0.20 | 0.81 | 1.45E-01 | -0.29 | 0.55 | 1.66E-02 | -0.50 | 0.86 | 4543385 | 139763 | 4470995 | 192972 | 4452281 | 196800 | 4489709 | 189185 |
| RP_POS_816_58881a13_364 | RP | POS | 13.36 | 816.5888 | | | | 5.75E-06 | -1.06 | 1.92 | 3.35E-01 | 0.16 | 0.77 | 3.88E-05 | -0.98 | 1.72 | 4.73E-06 | -1.14 | 1.73 | 1724399 | 714124 | 1011929 | 560797 | 957543 | 596192 | 1066315 | 523379 |
| RP_NEG_325_1853a13_304 | RP | NEG | 13.30 | 325.1853 | | | | 8.25E-02 | -0.37 | 0.85 | 3.35E-01 | -0.19 | 0.79 | 4.43E-02 | -0.46 | 0.99 | 2.45E-01 | -0.27 | 0.76 | 3320122 | 1607737 | 2750150 | 1504858 | 2895997 | 1581560 | 2604303 | 1425015 |
| RP_POS_276_22968a10_361 | RP | POS | 10.36 | 276.2297 | | | | 9.01E-04 | 0.44 | 0.82 | 3.35E-01 | 0.22 | 0.80 | 1.50E-02 | 0.55 | 0.87 | 1.36E-03 | 0.34 | 1.19 | 4358031 | 1868580 | 6481515 | 5303065 | 5966096 | 2502410 | 6996934 | 7072108 |
| RP_NEG_211_91734a14_271 | RP | NEG | 14.27 | 211.9173 | | | | 4.70E-01 | 0.11 | 0.34 | 3.35E-01 | 0.22 | 0.60 | 3.50E-01 | 0.21 | 0.33 | 9.93E-01 | 0.00 | 0.22 | 3070320 | 342154 | 3137077 | 694526 | 3069600 | 363928 | 3204553 | 912601 |
| HILIC_POS_421_25961a0_637 | HILIC | POS | 0.637 | 421.2596 | | | | 3.33E-06 | 0.56 | 1.26 | 3.35E-01 | -0.22 | 0.92 | 2.38E-03 | 0.45 | 1.41 | 4.26E-04 | 0.66 | 1.47 | 217463 | 495500 | 2876585 | 5339902 | 3394146 | 5919659 | 2359025 | 4692760 |
| RP_NEG_371_15314a11_165 | RP | NEG | 11.17 | 371.1531 | | | | 5.90E-02 | 0.37 | 0.96 | 3.35E-01 | 0.20 | 0.81 | 2.97E-02 | 0.47 | 1.04 | 2.25E-01 | 0.27 | 0.78 | 2159901 | 190959 | 2236239 | 207316 | 2216153 | 221021 | 2256325 | 192778 |
| HILIC_POS_150_11253a2_738 | HILIC | POS | 2.738 | 150.1125 | | | | 4.49E-01 | 0.09 | 0.39 | 3.35E-01 | -0.22 | 0.59 | 1.49E-01 | -0.02 | 0.62 | 3.89E-01 | 0.20 | 0.46 | 1098098 | 1223094 | 2406489 | 17068293 | 4068025 | 24133212 | 744954 | 743416 |
| RP_NEG_484_79187a15_482 | RP | NEG | 15.48 | 484.7919 | | | | 7.22E-01 | 0.08 | 0.29 | 3.35E-01 | -0.19 | 0.72 | 9.42E-01 | -0.02 | 0.51 | 4.91E-01 | 0.17 | 0.36 | 1066176 | 36179 | 1068741 | 32084 | 1071850 | 36189 | 1065632 | 27394 |
| RP_POS_246_94275a15_334 | RP | POS | 15.33 | 246.9428 | | | | 5.08E-01 | -0.14 | 0.30 | 3.35E-01 | -0.19 | 0.59 | 2.96E-01 | -0.23 | 0.51 | 8.60E-01 | -0.04 | 0.17 | 5323682 | 262283 | 5288404 | 253866 | 5312987 | 274104 | 5263820 | 232045 |
| RP_POS_336_25073a8_177 | RP | POS | 8.18 | 336.2507 | | | | 2.70E-01 | -0.32 | 0.89 | 3.35E-01 | -0.14 | 0.68 | 1.86E-01 | -0.39 | 0.79 | 4.06E-01 | -0.25 | 0.57 | 1383834 | 3565404 | 654282 | 1616159 | 810845 | 1864837 | 497719 | 1322727 |
| RP_POS_940_81738a0_492 | RP | POS | 0.49 | 940.8174 | | | | 1.74E-08 | 0.68 | 1.33 | 3.36E-01 | -0.21 | 1.19 | 2.31E-04 | 0.57 | 1.33 | 2.30E-05 | 0.78 | 1.47 | 136366 | 88113 | 1687426 | 2523809 | 1931492 | 2711549 | 1443359 | 2322747 |
| RP_POS_512_26837a10_18 | RP | POS | 10.18 | 512.2684 | | | | 7.99E-01 | 0.05 | 0.64 | 3.36E-01 | 0.19 | 0.68 | 5.59E-01 | 0.15 | 0.96 | 8.32E-01 | -0.04 | 0.18 | 500786 | 631030 | 533326 | 609927 | 474254 | 386223 | 592398 | 771572 |
| HILIC_POS_358_20712a1_886 | HILIC | POS | 1.886 | 358.2071 | | | | 8.56E-01 | 0.04 | 1.03 | 3.36E-01 | -0.18 | 0.82 | 8.61E-01 | -0.04 | 0.84 | 6.24E-01 | 0.13 | 1.02 | 1151390 | 3853231 | 1285586 | 2760679 | 1552593 | 3159680 | 1018578 | 2295466 |
| RP_POS_305_15692a3_416 | RP | POS | 3.42 | 305.1569 | | | | 5.06E-01 | 0.15 | 1.13 | 3.36E-01 | -0.18 | 1.17 | 8.00E-01 | 0.06 | 0.98 | 3.42E-01 | 0.24 | 1.03 | 849850 | 2124908 | 1126792 | 1729185 | 1293943 | 1924938 | 959640 | 1509561 |
| RP_POS_348_28711a10_813 | RP | POS | 10.81 | 348.2871 | | | | 6.36E-16 | 1.11 | 1.93 | 3.37E-01 | 0.19 | 1.12 | 4.69E-11 | 1.20 | 2.09 | 1.16E-08 | 1.01 | 1.93 | 449351 | 209055 | 1060366 | 545344 | 1007694 | 549556 | 1113039 | 541475 |
| RP_POS_239_16339a8_915 | RP | POS | 8.92 | 239.1634 | | | | 3.77E-01 | 0.16 | 0.83 | 3.37E-01 | -0.20 | 0.80 | 7.83E-01 | 0.06 | 0.73 | 2.11E-01 | 0.26 | 0.79 | 1436749 | 63126 | 1448999 | 80868 | 1456807 | 80169 | 1441191 | 81617 |

**Supplementary table 1: Metabolomics results** (in asceding order according to p-value from progressive ALD vs  non-progressive ALD comparison, with molecular features with ID_level of 1-2 first)

| | | | | | | | | all_ALD_vs_control | | | Prog_ALD_vs_non-prog_ALD | | | Prog_ALD_vs_control_ALD | | | Non-prog_ALD_vs_control | | | Healthy_control | | all_ALD | | Non-prog_ALD | | Progressive_ALD | |
|---|---|---|---|---|---|---|---|---|---|---|---|---|---|---|---|---|---|---|---|---|---|---|---|---|---|---|---|
| | | | | | | | | t-test | Cohen's | PLS-DA | t-test | Cohen's | PLS-DA | t-test | Cohen's | PLS-DA | t-test | Cohen's | PLS-DA | | | | | | | | |
| Feature_ID | RP_HILIC | POS_NEG | Average.Rt | Average.Mz | Metabolite.name | curated_MSMS | ID_level | p | d | VIP | p | d | VIP | p | d | VIP | p | d | VIP | Mean | SD | Mean | SD | Mean | SD | Mean | SD |
| HILIC_POS_188_07077a4_123 | HILIC | POS | 4.123 | 188.0708 | | | | 1.44E-02 | -0.42 | 1.08 | 3.37E-01 | -0.20 | 1.38 | 1.33E-02 | -0.52 | 1.04 | 9.56E-02 | -0.32 | 0.94 | 26792506 | 6022675 | 23374865 | 8520295 | 24197467 | 7848557 | 22552262 | 9148311 |
| HILIC_POS_604_35382a0_624 | HILIC | POS | 0.624 | 604.3538 | | | | 3.88E-06 | 0.56 | 1.26 | 3.37E-01 | -0.21 | 0.94 | 2.19E-03 | 0.45 | 1.43 | 4.39E-04 | 0.67 | 1.44 | 196267 | 567447 | 2054840 | 3692037 | 2411123 | 4109836 | 1698557 | 3223455 |
| HILIC_NEG_329_65723a1_363 | HILIC | NEG | 1.363 | 329.6572 | | | | 5.90E-08 | 0.86 | 1.38 | 3.37E-01 | -0.20 | 0.67 | 2.16E-04 | 0.76 | 1.22 | 3.57E-08 | 0.96 | 1.88 | 3243892 | 843056 | 4471389 | 1449083 | 4611248 | 1181190 | 4331531 | 1675533 |
| RP_POS_283_17505a3_416 | RP | POS | 3.42 | 283.1751 | | | | 5.47E-01 | 0.14 | 1.13 | 3.37E-01 | -0.18 | 1.17 | 8.37E-01 | 0.05 | 0.98 | 3.74E-01 | 0.23 | 1.03 | 873987 | 2181736 | 1130273 | 1714320 | 1295628 | 1910406 | 964917 | 1494093 |
| RP_POS_441_29712a10_979 | RP | POS | 10.98 | 441.2971 | | | | 1.80E-01 | 0.26 | 1.19 | 3.37E-01 | 0.20 | 0.96 | 1.06E-01 | 0.36 | 1.06 | 4.48E-01 | 0.17 | 1.10 | 5749136 | 1046442 | 6043449 | 1131027 | 5934390 | 1113011 | 6152507 | 1149559 |
| RP_NEG_910_81549a0_492 | RP | NEG | 0.49 | 910.8155 | | | | 2.46E-03 | 0.40 | 1.05 | 3.38E-01 | 0.21 | 1.22 | 8.00E-03 | 0.51 | 1.13 | 6.35E-02 | 0.29 | 1.12 | 623598 | 231093 | 870404 | 686604 | 804208 | 612097 | 936601 | 754231 |
| HILIC_NEG_266_94656a4_723 | HILIC | NEG | 4.723 | 266.9466 | | | | 2.98E-04 | 0.61 | 1.02 | 3.38E-01 | 0.20 | 1.02 | 1.01E-03 | 0.71 | 1.09 | 4.39E-03 | 0.51 | 1.00 | 880323 | 314143 | 1154929 | 466349 | 1109974 | 389318 | 1199884 | 532642 |
| RP_POS_784_58264a14_255 | RP | POS | 14.26 | 784.5826 | | | | 4.16E-02 | 0.38 | 0.72 | 3.38E-01 | 0.20 | 0.60 | 2.72E-02 | 0.47 | 0.86 | 1.83E-01 | 0.28 | 0.53 | 976841 | 734213 | 1306169 | 907876 | 1218698 | 882013 | 1393640 | 933670 |
| RP_POS_912_76477a0_496 | RP | POS | 0.50 | 912.7648 | | | | 4.40E-10 | -0.77 | 1.41 | 3.38E-01 | 0.21 | 1.21 | 6.87E-05 | -0.66 | 1.45 | 1.09E-06 | -0.87 | 1.58 | 1441379 | 95038 | 1023037 | 588792 | 966314 | 598024 | 1079759 | 579852 |
| RP_POS_388_26947a10_24 | RP | POS | 10.24 | 388.2695 | | | | 1.30E-06 | 0.57 | 1.14 | 3.38E-01 | -0.21 | 1.05 | 3.07E-03 | 0.47 | 1.09 | 1.39E-04 | 0.68 | 1.34 | 14234 | 10316 | 244928 | 447261 | 287981 | 468060 | 201876 | 425798 |
| RP_NEG_466_81677a15_466 | RP | NEG | 15.47 | 466.8168 | | | | 1.91E-01 | 0.25 | 0.96 | 3.38E-01 | -0.20 | 0.67 | 4.86E-01 | 0.15 | 0.46 | 1.09E-01 | 0.35 | 0.86 | 3349992 | 83279 | 3373248 | 95976 | 3382485 | 96170 | 3364011 | 95854 |
| RP_POS_261_1308a3_194 | RP | POS | 3.19 | 261.1308 | | | | 4.23E-02 | 0.32 | 1.11 | 3.38E-01 | -0.21 | 1.13 | 2.21E-01 | 0.21 | 1.12 | 4.20E-02 | 0.42 | 1.12 | 519602 | 556322 | 805657 | 981998 | 900196 | 1099660 | 711119 | 849016 |
| RP_POS_499_12787a10_7 | RP | POS | 10.70 | 499.1279 | | | | 3.46E-01 | 0.21 | 0.68 | 3.38E-01 | -0.19 | 0.83 | 6.31E-01 | 0.11 | 0.47 | 2.17E-01 | 0.30 | 0.68 | 759582 | 198253 | 796926 | 175563 | 813823 | 182199 | 780029 | 168804 |
| RP_POS_432_2804a4_635 | RP | POS | 4.64 | 432.2804 | | | | 8.82E-01 | 0.04 | 1.18 | 3.39E-01 | -0.16 | 1.18 | 8.93E-01 | -0.04 | 0.92 | 6.83E-01 | 0.12 | 1.03 | 2095189 | 6498818 | 2274467 | 3603006 | 2621146 | 4017775 | 1927787 | 3136948 |
| RP_NEG_458_1452a11_604 | RP | NEG | 11.60 | 458.1452 | | | | 1.88E-01 | -0.26 | 0.90 | 3.39E-01 | 0.19 | 0.74 | 4.36E-01 | -0.16 | 0.70 | 1.26E-01 | -0.36 | 0.81 | 570461 | 68824 | 551540 | 73218 | 544496 | 81551 | 558584 | 63871 |
| HILIC_NEG_158_8468a2_961 | HILIC | NEG | 2.961 | 158.8468 | | | | 3.37E-03 | 0.48 | 0.92 | 3.39E-01 | -0.20 | 0.61 | 1.76E-02 | 0.38 | 0.91 | 1.02E-02 | 0.58 | 0.87 | 2114957 | 451000 | 2437128 | 709811 | 2505460 | 884104 | 2368796 | 476212 |
| RP_NEG_362_9422a15_284 | RP | NEG | 15.28 | 362.9422 | | | | 7.76E-01 | -0.06 | 0.54 | 3.39E-01 | -0.19 | 0.68 | 4.84E-01 | -0.15 | 0.44 | 8.68E-01 | 0.04 | 0.41 | 2236432 | 115073 | 2229741 | 116223 | 2240912 | 124630 | 2218569 | 107258 |
| RP_POS_492_21271a11_804 | RP | POS | 11.80 | 492.2127 | | | | 1.24E-02 | -0.49 | 1.20 | 3.39E-01 | 0.19 | 1.31 | 1.07E-01 | -0.39 | 0.79 | 2.59E-03 | -0.58 | 1.41 | 865896 | 411292 | 643924 | 458627 | 599775 | 298261 | 688072 | 576223 |
| RP_POS_419_73749a12_695 | RP | POS | 12.70 | 419.7375 | | | | 1.83E-02 | 0.49 | 0.95 | 3.39E-01 | -0.19 | 0.70 | 9.15E-02 | 0.39 | 0.83 | 8.98E-03 | 0.58 | 0.99 | 1040611 | 560830 | 1317691 | 556926 | 1371185 | 508814 | 1264196 | 601608 |
| RP_POS_546_45386a14_267 | RP | POS | 14.27 | 546.4539 | | | | 9.52E-01 | -0.01 | 0.10 | 3.40E-01 | 0.19 | 0.67 | 7.08E-01 | 0.08 | 0.23 | 6.63E-01 | -0.11 | 0.27 | 8845072 | 1684714 | 8824738 | 1580311 | 8672943 | 1821904 | 8976532 | 1295908 |
| RP_POS_708_51147a13_57 | RP | POS | 13.57 | 708.5115 | | | | 6.19E-01 | 0.09 | 0.57 | 3.40E-01 | 0.20 | 0.75 | 3.65E-01 | 0.19 | 0.49 | 9.76E-01 | -0.01 | 0.49 | 15397712 | 976833 | 15501536 | 1156716 | 15390502 | 1181554 | 15612571 | 1132281 |
| RP_POS_318_96899a0_538 | RP | POS | 0.54 | 318.9690 | | | | 1.66E-01 | 0.20 | 1.03 | 3.40E-01 | -0.21 | 1.02 | 5.63E-01 | 0.10 | 0.80 | 1.19E-01 | 0.31 | 1.21 | 901786 | 311940 | 1018695 | 631787 | 1079337 | 693707 | 958054 | 563669 |
| RP_POS_413_2868a10_591 | RP | POS | 10.59 | 413.2868 | | | | 2.05E-02 | -0.51 | 1.27 | 3.40E-01 | 0.18 | 0.72 | 8.77E-02 | -0.42 | 0.89 | 9.70E-03 | -0.60 | 1.25 | 801094 | 133291 | 737979 | 117618 | 726690 | 103721 | 749267 | 130124 |
| RP_NEG_243_82039a0_769 | RP | NEG | 0.77 | 243.8204 | | | | 2.65E-08 | 0.90 | 1.42 | 3.40E-01 | -0.19 | 0.65 | 7.22E-05 | 0.81 | 1.27 | 3.23E-08 | 1.00 | 1.80 | 2638166 | 1808750 | 5300353 | 2950159 | 5583510 | 2534711 | 5017195 | 3316037 |
| RP_POS_404_7883a13_996 | RP | POS | 14.00 | 404.7883 | | | | 1.80E-01 | -0.30 | 0.56 | 3.40E-01 | 0.18 | 0.90 | 4.09E-01 | -0.21 | 0.50 | 9.57E-02 | -0.39 | 0.68 | 2998319 | 924680 | 2750625 | 797363 | 2674120 | 700530 | 2827129 | 884230 |
| HILIC_POS_648_37988a0_625 | HILIC | POS | 0.625 | 648.3799 | | | | 6.64E-06 | 0.55 | 1.27 | 3.40E-01 | -0.21 | 0.94 | 2.99E-03 | 0.45 | 1.43 | 5.01E-04 | 0.66 | 1.44 | 216672 | 597348 | 1816356 | 3221992 | 2125255 | 3563507 | 1507456 | 2842711 |
| RP_POS_840_77875a11_247 | RP | POS | 11.25 | 840.7788 | | | | 2.61E-02 | 0.36 | 1.30 | 3.40E-01 | -0.21 | 0.94 | 1.93E-01 | 0.26 | 0.91 | 1.44E-02 | 0.47 | 1.42 | 407869 | 201493 | 515785 | 316548 | 546126 | 298871 | 485445 | 333537 |
| HILIC_POS_293_09729a7_328 | HILIC | POS | 7.328 | 293.0973 | | | | 7.36E-01 | -0.07 | 0.44 | 3.41E-01 | -0.19 | 0.71 | 4.45E-01 | -0.17 | 0.31 | 9.20E-01 | 0.02 | 0.63 | 1253348 | 169252 | 1241816 | 162422 | 1257388 | 188492 | 1226245 | 131439 |
| HILIC_POS_224_12793a5_92 | HILIC | POS | 5.92 | 224.1279 | | | | 1.88E-01 | -0.24 | 0.84 | 3.41E-01 | 0.20 | 0.75 | 5.01E-01 | -0.15 | 0.67 | 1.01E-01 | -0.34 | 0.86 | 6085658 | 1207008 | 5741688 | 1457172 | 5602277 | 1395333 | 5881100 | 1517702 |
| HILIC_POS_338_04959a3_077 | HILIC | POS | 3.077 | 338.0496 | | | | 5.12E-01 | 0.09 | 1.25 | 3.41E-01 | -0.22 | 1.00 | 9.08E-01 | -0.02 | 1.29 | 2.73E-01 | 0.20 | 1.36 | 1650713 | 99334 | 1672165 | 275152 | 1698488 | 279438 | 1645843 | 271041 |
| HILIC_POS_465_28586a0_645 | HILIC | POS | 0.645 | 465.2859 | | | | 1.53E-06 | 0.59 | 1.29 | 3.41E-01 | -0.21 | 0.93 | 1.37E-03 | 0.48 | 1.47 | 2.58E-04 | 0.69 | 1.48 | 181331 | 548544 | 1978690 | 3405720 | 2304553 | 3766781 | 1652826 | 3004865 |
| RP_POS_329_00464a8_121 | RP | POS | 8.12 | 329.0046 | | | | 4.07E-01 | -0.18 | 0.44 | 3.41E-01 | 0.19 | 0.99 | 7.19E-01 | -0.08 | 0.36 | 2.57E-01 | -0.27 | 0.63 | 2610845 | 556389 | 2517955 | 514887 | 2468702 | 536504 | 2567209 | 492787 |
| RP_NEG_231_93253a13_214 | RP | NEG | 13.21 | 231.9325 | | | | 5.52E-01 | 0.10 | 0.58 | 3.41E-01 | -0.20 | 0.68 | 9.98E-01 | 0.00 | 0.60 | 2.83E-01 | 0.20 | 0.64 | 4997471 | 569758 | 5074874 | 812811 | 5152621 | 723082 | 4997127 | 894216 |
| RP_POS_207_11032a2_819 | RP | POS | 2.82 | 207.1103 | | | | 1.23E-02 | 0.43 | 0.71 | 3.42E-01 | 0.20 | 0.59 | 1.83E-02 | 0.53 | 0.94 | 5.85E-02 | 0.33 | 0.97 | 855318 | 377594 | 1074305 | 530754 | 1023518 | 399142 | 1125093 | 636058 |
| RP_POS_344_22794a4_023 | RP | POS | 4.02 | 344.2279 | | | | 9.43E-01 | -0.02 | 1.13 | 3.42E-01 | -0.14 | 1.16 | 7.52E-01 | -0.09 | 0.88 | 8.65E-01 | 0.05 | 0.94 | 2393705 | 7272461 | 2297301 | 3510994 | 2633049 | 3924846 | 1961554 | 3044875 |
| RP_POS_144_94791a15_379 | RP | POS | 15.38 | 144.9479 | | | | 8.65E-01 | -0.02 | 0.36 | 3.42E-01 | 0.21 | 0.64 | 4.78E-01 | 0.08 | 0.50 | 5.65E-01 | -0.13 | 0.33 | 9936004 | 248222 | 9923819 | 567721 | 9869434 | 752620 | 9978203 | 280568 |
| HILIC_NEG_199_97797a1_299 | HILIC | NEG | 1.299 | 199.9780 | | | | 9.89E-04 | 0.64 | 1.12 | 3.42E-01 | 0.19 | 1.21 | 1.20E-03 | 0.74 | 1.15 | 7.90E-03 | 0.55 | 1.05 | 670042 | 201956 | 816366 | 223999 | 794977 | 200617 | 837754 | 245345 |
| RP_POS_298_64755a10_621 | RP | POS | 10.62 | 298.6476 | | | | 3.01E-02 | 0.50 | 1.32 | 3.43E-01 | -0.18 | 0.84 | 1.03E-01 | 0.41 | 0.99 | 1.54E-02 | 0.59 | 1.36 | 2231465 | 500185 | 2449259 | 404984 | 2487906 | 363722 | 2410612 | 442753 |
| RP_NEG_369_17462a10_264 | RP | NEG | 10.26 | 369.1746 | | | | 5.62E-01 | -0.10 | 0.30 | 3.43E-01 | -0.21 | 0.63 | 2.07E-01 | -0.20 | 0.48 | 9.73E-01 | 0.01 | 0.34 | 37525595 | 41165307 | 31901289 | 63701153 | 37989775 | 80917285 | 25812804 | 39689135 |
| RP_POS_199_13039a13_111 | RP | POS | 13.11 | 199.1304 | | | | 3.20E-01 | 0.20 | 0.61 | 3.43E-01 | 0.19 | 0.63 | 1.97E-01 | 0.29 | 0.62 | 6.46E-01 | 0.10 | 0.51 | 16411721 | 1887142 | 16803827 | 2030290 | 16610204 | 1919960 | 16997450 | 2136709 |
| RP_POS_385_16687a10_907 | RP | POS | 10.91 | 385.1669 | | | | 2.80E-02 | 0.31 | 1.45 | 3.43E-01 | 0.21 | 0.88 | 3.54E-02 | 0.42 | 1.15 | 1.99E-01 | 0.21 | 1.26 | 2702308 | 1095272 | 3391866 | 2418902 | 3161135 | 2095077 | 3622596 | 2706214 |
| HILIC_POS_72_08161a1_413 | HILIC | POS | 1.413 | 72.0816 | | | | 6.66E-01 | -0.09 | 0.53 | 3.43E-01 | -0.19 | 0.76 | 3.95E-01 | -0.18 | 0.40 | 9.73E-01 | 0.01 | 0.12 | 4748363 | 2121062 | 4560781 | 2144282 | 4765278 | 2395925 | 4356284 | 1861163 |
| RP_NEG_652_29156a10_62 | RP | NEG | 10.62 | 652.2916 | | | | 2.49E-02 | 0.52 | 1.20 | 3.43E-01 | -0.17 | 0.88 | 8.89E-02 | 0.43 | 0.93 | 1.22E-02 | 0.61 | 1.26 | 2139649 | 421652 | 2329188 | 332979 | 2360927 | 288984 | 2297448 | 372083 |
| RP_POS_144_08063a2_893 | RP | POS | 2.89 | 144.0806 | | | | 3.63E-04 | -0.61 | 1.24 | 3.43E-01 | -0.20 | 1.07 | 1.13E-03 | -0.71 | 1.29 | 4.96E-03 | -0.51 | 1.17 | 1798409 | 281614 | 1557180 | 412342 | 1596483 | 345526 | 1517877 | 470087 |
| RP_POS_349_18307a4_023 | RP | POS | 4.02 | 349.1831 | | | | 9.56E-01 | -0.02 | 1.13 | 3.43E-01 | -0.14 | 1.17 | 7.63E-01 | -0.09 | 0.89 | 8.51E-01 | 0.06 | 0.94 | 1158872 | 3632450 | 1122089 | 1775508 | 1291235 | 1978326 | 952944 | 1548233 |
| RP_POS_280_26349a9_535 | RP | POS | 9.54 | 280.2635 | | | | 3.00E-01 | -0.26 | 0.77 | 3.43E-01 | -0.17 | 0.75 | 1.74E-01 | -0.34 | 0.79 | 5.23E-01 | -0.18 | 0.44 | 18839932 | 26869384 | 13519613 | 17704912 | 15207792 | 21714695 | 11831435 | 12489230 |
| RP_POS_395_17786a6_811 | RP | POS | 6.81 | 395.1779 | | | | 3.06E-03 | -0.44 | 0.99 | 3.44E-01 | 0.21 | 0.93 | 7.12E-02 | -0.34 | 0.80 | 3.08E-03 | -0.55 | 1.20 | 1455194 | 70202 | 1398791 | 138544 | 1385600 | 135032 | 1411983 | 142099 |
| HILIC_NEG_215_03293a4_979 | HILIC | NEG | 4.979 | 215.0329 | | | | 4.42E-01 | 0.13 | 0.57 | 3.44E-01 | 0.21 | 0.71 | 2.99E-01 | 0.23 | 0.41 | 8.82E-01 | 0.03 | 0.62 | 13244423 | 7673216 | 14621422 | 11566480 | 13519454 | 8871889 | 15723391 | 13751675 |
| RP_POS_311_18518a8_277 | RP | POS | 8.28 | 311.1852 | | | | 2.18E-01 | -0.29 | 0.55 | 3.44E-01 | -0.18 | 0.62 | 1.19E-01 | -0.38 | 0.65 | 4.44E-01 | -0.20 | 0.45 | 877940 | 114163 | 850355 | 89136 | 858841 | 101931 | 841869 | 74269 |
| HILIC_POS_147_11279a7_097 | HILIC | POS | 7.097 | 147.1128 | | | | 1.97E-02 | 0.31 | 1.00 | 3.44E-01 | -0.21 | 0.88 | 2.28E-01 | 0.20 | 1.06 | 2.39E-02 | 0.41 | 1.07 | 14267022 | 5462863 | 18771378 | 16452010 | 20337036 | 17246561 | 17205720 | 15633767 |
| HILIC_NEG_173_92601a5_428 | HILIC | NEG | 5.428 | 173.9260 | | | | 7.20E-01 | 0.09 | 0.37 | 3.45E-01 | -0.17 | 0.92 | 9.94E-01 | 0.00 | 0.15 | 4.93E-01 | 0.17 | 0.37 | 910305 | 214060 | 925119 | 159248 | 940254 | 149755 | 909984 | 168354 |
| HILIC_NEG_101_02424a1_261 | HILIC | NEG | 1.261 | 101.0242 | | | | 6.76E-01 | -0.07 | 0.37 | 3.45E-01 | 0.20 | 1.28 | 8.89E-01 | 0.03 | 0.32 | 3.06E-01 | -0.17 | 0.78 | 396942 | 243659 | 373441 | 358892 | 339302 | 252397 | 407579 | 440629 |
| RP_POS_499_82724a5_744 | RP | POS | 5.74 | 499.8272 | | | | 3.24E-01 | 0.19 | 0.82 | 3.45E-01 | 0.20 | 0.89 | 2.21E-01 | 0.29 | 0.45 | 6.47E-01 | 0.09 | 0.53 | 1343048 | 1942658 | 1749448 | 2212086 | 1539181 | 1783699 | 1959716 | 2571906 |
| RP_POS_504_30881a10_416 | RP | POS | 10.42 | 504.3088 | | | | 1.53E-05 | 0.73 | 1.35 | 3.45E-01 | -0.19 | 0.59 | 2.62E-03 | 0.63 | 1.18 | 8.28E-06 | 0.82 | 1.54 | 629460 | 245854 | 892653 | 370774 | 927877 | 317053 | 857430 | 417969 |
| HILIC_NEG_144_92343a0_546 | HILIC | NEG | 0.546 | 144.9234 | | | | 2.01E-03 | -0.53 | 1.08 | 3.46E-01 | 0.20 | 0.80 | 6.48E-02 | -0.43 | 0.62 | 1.63E-04 | -0.63 | 1.50 | 7976709 | 1181274 | 7110499 | 1724025 | 6946738 | 1049181 | 7274261 | 2202197 |
| RP_POS_1002_79565a0_489 | RP | POS | 0.49 | 1002.7957 | | | | 3.13E-08 | 0.67 | 1.31 | 3.46E-01 | -0.21 | 1.15 | 3.58E-04 | 0.56 | 1.31 | 2.40E-05 | 0.77 | 1.48 | 100836 | 29160 | 283580 | 301150 | 312122 | 318919 | 255038 | 282598 |
| RP_POS_239_16414a9_687 | RP | POS | 9.69 | 239.1641 | | | | 7.39E-01 | -0.05 | 0.93 | 3.46E-01 | 0.21 | 0.59 | 8.37E-01 | 0.05 | 0.56 | 2.98E-01 | -0.16 | 0.98 | 5916523 | 2889268 | 5688946 | 4507735 | 5260925 | 2548486 | 6116966 | 5846826 |
| HILIC_POS_59_06129a4_279 | HILIC | POS | 4.279 | 59.0613 | | | | 1.77E-02 | -0.41 | 0.68 | 3.46E-01 | 0.20 | 0.77 | 1.18E-01 | -0.31 | 0.75 | 1.46E-02 | -0.51 | 0.82 | 3577961 | 440521 | 3334247 | 634885 | 3274131 | 661128 | 3394364 | 608210 |
| HILIC_POS_195_11279a0_525 | HILIC | POS | 0.525 | 195.1128 | | | | 1.59E-01 | 0.24 | 0.73 | 3.46E-01 | -0.20 | 0.78 | 5.25E-01 | 0.14 | 0.30 | 5.80E-02 | 0.34 | 0.91 | 289874 | 128992 | 331555 | 183555 | 348943 | 144934 | 314167 | 215522 |
| RP_POS_707_49231a10_656 | RP | POS | 10.66 | 707.4923 | | | | 7.41E-01 | 0.06 | 0.44 | 3.46E-01 | 0.19 | 0.55 | 4.58E-01 | 0.16 | 0.44 | 8.87E-01 | -0.03 | 0.26 | 7801772 | 587954 | 7842320 | 639215 | 7781812 | 663698 | 7902827 | 614461 |
| RP_POS_325_21387a10_423 | RP | POS | 10.42 | 325.2139 | | | | 6.57E-01 | 0.07 | 0.39 | 3.47E-01 | -0.21 | 0.74 | 8.44E-01 | -0.03 | 0.26 | 4.23E-01 | 0.17 | 0.51 | 2350974 | 3616247 | 2743473 | 6062134 | 3317654 | 7190184 | 2169292 | 4677323 |
| HILIC_NEG_333_6528a1_364 | HILIC | NEG | 1.364 | 333.6528 | | | | 1.27E-07 | 0.84 | 1.34 | 3.47E-01 | -0.19 | 0.67 | 3.16E-04 | 0.75 | 1.19 | 7.00E-08 | 0.94 | 1.85 | 1704784 | 450149 | 2338569 | 766824 | 2411156 | 622758 | 2265982 | 888516 |
| HILIC_POS_534_29486a1_319 | HILIC | POS | 1.319 | 534.2949 | | | | 1.11E-06 | 0.81 | 1.29 | 3.47E-01 | -0.19 | 0.72 | 8.94E-04 | 0.72 | 1.08 | 1.94E-07 | 0.91 | 2.00 | 4110254 | 421389 | 4629690 | 645522 | 4690799 | 481035 | 4568581 | 776457 |
| RP_NEG_1036_75708a0_493 | RP | NEG | 0.49 | 1036.7571 | | | | 1.05E-02 | -0.44 | 0.81 | 3.47E-01 | 0.20 | 1.13 | 1.12E-01 | -0.34 | 0.75 | 4.29E-03 | -0.54 | 1.17 | 938065 | 114611 | 869751 | 163537 | 854280 | 141202 | 885223 | 183325 |
| RP_NEG_245_00291a7_878 | RP | NEG | 7.88 | 245.0029 | | | | 8.72E-01 | -0.03 | 0.72 | 3.47E-01 | -0.19 | 0.63 | 5.66E-01 | -0.13 | 0.26 | 7.69E-01 | 0.06 | 0.75 | 1782739 | 180236 | 1776700 | 194283 | 1795069 | 191772 | 1758331 | 196968 |
| RP_POS_542_37183a14_267 | RP | POS | 14.27 | 542.3718 | | | | 4.34E-01 | 0.11 | 0.43 | 3.47E-01 | 0.21 | 0.61 | 3.26E-01 | 0.21 | 0.49 | 9.84E-01 | 0.00 | 0.33 | 11582253 | 1610416 | 11962139 | 3919897 | 11591090 | 2462790 | 12333187 | 4969755 |
| HILIC_POS_308_18564a0_952 | HILIC | POS | 0.952 | 308.1856 | | | | 4.82E-01 | 0.09 | 0.57 | 3.47E-01 | -0.21 | 0.71 | 8.78E-01 | -0.01 | 0.31 | 3.84E-01 | 0.20 | 0.48 | 420597 | 1022977 | 667099 | 2987975 | 950392 | 4078919 | 383807 | 1112129 |
| HILIC_NEG_158_93893a5_428 | HILIC | NEG | 5.428 | 158.9389 | | | | 3.26E-02 | 0.38 | 1.03 | 3.48E-01 | 0.20 | 0.64 | 3.07E-02 | 0.48 | 1.05 | 1.39E-01 | 0.28 | 0.82 | 1052471 | 77759 | 1089951 | 103218 | 1080205 | 87975 | 1099697 | 116585 |
| HILIC_POS_159_14922a1_89 | HILIC | POS | 1.89 | 159.1492 | | | | 2.85E-04 | 0.49 | 0.93 | 3.48E-01 | -0.21 | 0.85 | 1.85E-02 | 0.38 | 0.82 | 1.89E-03 | 0.59 | 1.20 | 477780 | 792147 | 1454963 | 2212976 | 1663756 | 2391276 | 1246169 | 2021707 |
| RP_NEG_259_00073a7_439 | RP | NEG | 7.44 | 259.0007 | | | | 5.26E-02 | -0.40 | 0.74 | 3.48E-01 | -0.19 | 0.82 | 3.74E-02 | -0.49 | 0.76 | 1.62E-01 | -0.31 | 0.91 | 2587834 | 347826 | 2447851 | 346677 | 2480542 | 313687 | 2415160 | 377150 |
| HILIC_NEG_348_99936a7_428 | HILIC | NEG | 7.428 | 348.9994 | | | | 7.50E-04 | -0.50 | 1.24 | 3.48E-01 | 0.20 | 0.92 | 2.70E-02 | -0.40 | 1.28 | 1.70E-03 | -0.60 | 1.37 | 1350066 | 55283 | 1298296 | 112767 | 1287664 | 116548 | 1308928 | 108986 |
| RP_POS_327_19333a10_423 | RP | POS | 10.42 | 327.1933 | | | | 9.77E-01 | 0.01 | 0.42 | 3.49E-01 | -0.19 | 0.59 | 6.98E-01 | -0.09 | 0.41 | 6.87E-01 | 0.10 | 0.38 | 3487950 | 1870837 | 3498847 | 1732044 | 3662158 | 1955666 | 3335536 | 1477199 |
| HILIC_NEG_157_90067a2_172 | HILIC | NEG | 2.172 | 157.9007 | | | | 4.43E-01 | 0.15 | 0.60 | 3.49E-01 | -0.19 | 0.68 | 8.05E-01 | 0.06 | 0.12 | 2.62E-01 | 0.25 | 0.56 | 2734750 | 681036 | 2843520 | 732588 | 2912553 | 715468 | 2774487 | 750135 |
| RP_NEG_173_98596a0_708 | RP | NEG | 0.71 | 173.9860 | | | | 4.16E-01 | -0.21 | 0.58 | 3.49E-01 | 0.16 | 0.60 | 6.45E-01 | -0.13 | 0.52 | 2.65E-01 | -0.29 | 0.58 | 1153194 | 2611992 | 750421 | 1622826 | 597346 | 1192254 | 903495 | 1962545 |
| HILIC_POS_284_1644a0_537 | HILIC | POS | 0.537 | 284.1644 | | | | 7.29E-01 | 0.04 | 0.64 | 3.49E-01 | 0.22 | 1.17 | 5.10E-01 | 0.15 | 0.21 | 3.75E-01 | -0.06 | 0.31 | 717727 | 77309 | 729351 | 305968 | 700417 | 97507 | 758285 | 421813 |
| RP_POS_418_20691a4_223 | RP | POS | 4.22 | 418.2069 | | | | 8.52E-01 | -0.04 | 0.84 | 3.49E-01 | 0.18 | 0.91 | 8.43E-01 | 0.05 | 0.56 | 6.00E-01 | -0.13 | 0.60 | 861324 | 529401 | 841924 | 437407 | 800730 | 472991 | 883117 | 399212 |
| RP_POS_434_26779a11_609 | RP | POS | 11.61 | 434.2678 | | | | 2.39E-03 | -0.54 | 1.08 | 3.49E-01 | 0.19 | 0.92 | 2.13E-02 | -0.45 | 1.01 | 3.22E-03 | -0.64 | 1.13 | 7896847 | 614100 | 7470254 | 805667 | 7394446 | 881837 | 7546061 | 722551 |
| RP_POS_722_52661a13_921 | RP | POS | 13.92 | 722.5266 | | | | 6.00E-01 | 0.11 | 0.71 | 3.50E-01 | -0.19 | 0.72 | 9.61E-01 | 0.01 | 0.22 | 3.91E-01 | 0.20 | 0.83 | 3636690 | 1772841 | 3827919 | 1827229 | 3999809 | 1987420 | 3656030 | 1653945 |
| RP_POS_441_29764a10_448 | RP | POS | 10.45 | 441.2976 | | | | 1.36E-01 | 0.28 | 0.53 | 3.50E-01 | 0.19 | 0.55 | 8.93E-02 | 0.38 | 0.67 | 3.69E-01 | 0.19 | 0.54 | 1010321 | 474902 | 1162282 | 551944 | 1110414 | 511196 | 1214150 | 590500 |
| RP_POS_378_24072a9_634 | RP | POS | 9.63 | 378.2407 | | | | 3.36E-01 | 0.18 | 0.81 | 3.51E-01 | -0.20 | 0.84 | 7.16E-01 | 0.08 | 0.60 | 1.68E-01 | 0.28 | 0.57 | 598657 | 243469 | 649083 | 291122 | 676414 | 250655 | 621752 | 326927 |
| RP_POS_654_32996a10_563 | RP | POS | 10.56 | 654.3300 | | | | 1.48E-04 | -0.70 | 1.14 | 3.51E-01 | 0.19 | 0.70 | 8.50E-03 | -0.60 | 0.96 | 2.60E-05 | -0.79 | 1.48 | 645823 | 73195 | 581728 | 92532 | 573035 | 66638 | 590422 | 112713 |
| RP_POS_437_23547a4_635 | RP | POS | 4.64 | 437.2355 | | | | 9.22E-01 | 0.03 | 1.17 | 3.51E-01 | -0.15 | 1.18 | 8.69E-01 | -0.05 | 0.91 | 7.31E-01 | 0.10 | 1.01 | 1090951 | 3540832 | 1154949 | 1860456 | 1329483 | 2062839 | 980414 | 1635774 |
| HILIC_POS_171_18561a5_337 | HILIC | POS | 5.337 | 171.1856 | | | | 9.24E-01 | -0.02 | 0.61 | 3.51E-01 | -0.20 | 0.81 | 5.69E-01 | -0.12 | 0.53 | 6.85E-01 | 0.08 | 0.30 | 124689065 | 30988225 | 124026764 | 42499357 | 128008197 | 42740563 | 120045331 | 42310111 |
| RP_POS_564_18542a3_001 | RP | POS | 3.00 | 564.1854 | | | | 3.87E-01 | 0.11 | 0.37 | 3.52E-01 | 0.21 | 0.73 | 3.11E-01 | 0.22 | 0.65 | 9.46E-01 | 0.01 | 0.85 | 1804749 | 261162 | 1883373 | 779811 | 1810189 | 467815 | 1956556 | 999420 |
| HILIC_POS_350_01508a7_373 | HILIC | POS | 7.373 | 350.0151 | | | | 4.45E-02 | 0.34 | 0.74 | 3.52E-01 | -0.20 | 0.56 | 1.75E-01 | 0.24 | 0.49 | 4.47E-02 | 0.44 | 0.75 | 14093749 | 1244262 | 14672930 | 1786348 | 14840322 | 2069157 | 14505537 | 1452103 |
| RP_NEG_279_03925a0_71 | RP | NEG | 0.71 | 279.0393 | | | | 8.06E-01 | 0.03 | 0.76 | 3.52E-01 | -0.21 | 0.80 | 6.14E-01 | -0.07 | 0.87 | 4.98E-01 | 0.14 | 0.68 | 1963262 | 425043 | 1996741 | 1134731 | 2103094 | 1350109 | 1890387 | 869269 |
| RP_NEG_357_89819a15_4 | RP | NEG | 15.40 | 357.8982 | | | | 5.50E-01 | 0.11 | 0.40 | 3.52E-01 | -0.19 | 0.56 | 9.33E-01 | 0.02 | 0.32 | 3.44E-01 | 0.21 | 0.70 | 840949 | 33136 | 845143 | 37812 | 848683 | 39764 | 841602 | 35805 |
| RP_POS_688_51788a10_457 | RP | POS | 10.46 | 688.5179 | | | | 2.47E-01 | -0.23 | 0.45 | 3.52E-01 | -0.19 | 1.18 | 1.37E-01 | -0.32 | 0.59 | 5.59E-01 | -0.13 | 0.38 | 1951993 | 396841 | 1855395 | 434633 | 1896090 | 455814 | 1814700 | 412942 |

**Supplementary table 1: Metabolomics results** (in asceding order according to p-value from progressive ALD vs  non-progressive ALD comparison, with molecular features with ID_level of 1-2 first)

| | | | | | | | | all_ALD_vs_control | | | Prog_ALD_vs_non-prog_ALD | | | Prog_ALD_vs_control_ALD | | | Non-prog_ALD_vs_control | | | Healthy_control | | all_ALD | | Non-prog_ALD | | Progressive_ALD | |
|---|---|---|---|---|---|---|---|---|---|---|---|---|---|---|---|---|---|---|---|---|---|---|---|---|---|---|---|
| | | | | | | | | t-test | Cohen's | PLS-DA | t-test | Cohen's | PLS-DA | t-test | Cohen's | PLS-DA | t-test | Cohen's | PLS-DA | | | | | | | | |
| Feature_ID | RP_HILIC | POS_NEG | Average.Rt | Average.Mz | Metabolite.name | curated_MSMS | ID_level | p | d | VIP | p | d | VIP | p | d | VIP | p | d | VIP | Mean | SD | Mean | SD | Mean | SD | Mean | SD |
| RP_POS_283_1752a10_66 | RP | POS | 10.66 | 283.1752 | | | | 6.71E-01 | -0.10 | 0.32 | 3.52E-01 | 0.17 | 0.56 | 9.56E-01 | -0.01 | 0.27 | 4.62E-01 | -0.19 | 0.33 | 1216117 | 425769 | 1181041 | 322707 | 1150836 | 323709 | 1211245 | 322111 |
| RP_POS_415_25375a4_635 | RP | POS | 4.64 | 415.2538 | | | | 9.63E-01 | 0.01 | 1.17 | 3.52E-01 | -0.15 | 1.17 | 8.35E-01 | -0.06 | 0.91 | 7.73E-01 | 0.09 | 1.00 | 713349 | 2364871 | 733441 | 1207712 | 846430 | 1339539 | 620451 | 1061359 |
| RP_NEG_241_82358a0_769 | RP | NEG | 0.77 | 241.8236 | | | | 2.33E-08 | 0.91 | 1.43 | 3.52E-01 | -0.19 | 0.64 | 6.70E-05 | 0.81 | 1.27 | 3.07E-08 | 1.00 | 1.81 | 3766699 | 2543991 | 7538179 | 4176664 | 7928941 | 3583711 | 7147417 | 4700048 |
| RP_POS_321_21899a11_252 | RP | POS | 11.25 | 321.2190 | | | | 3.62E-03 | 0.49 | 1.37 | 3.53E-01 | 0.20 | 0.87 | 5.70E-03 | 0.59 | 1.28 | 3.38E-02 | 0.39 | 1.31 | 975896 | 307158 | 1189403 | 460156 | 1146386 | 404945 | 1232419 | 509953 |
| HILIC_POS_220_11758a1_026 | HILIC | POS | 1.026 | 220.1176 | | | | 8.73E-05 | 0.57 | 1.04 | 3.53E-01 | 0.20 | 0.79 | 5.75E-04 | 0.67 | 1.16 | 6.75E-03 | 0.46 | 0.93 | 341051 | 96788 | 451642 | 211198 | 431903 | 196362 | 471381 | 225313 |
| RP_POS_560_32739a5_008 | RP | POS | 5.01 | 560.3274 | | | | 1.11E-06 | 0.58 | 1.27 | 3.53E-01 | -0.21 | 1.21 | 1.11E-03 | 0.48 | 1.37 | 3.06E-04 | 0.68 | 1.36 | 41174 | 88098 | 1020440 | 1880823 | 1196105 | 2099227 | 844775 | 1636292 |
| RP_NEG_312_85175a15_493 | RP | NEG | 15.49 | 312.8518 | | | | 7.45E-01 | 0.08 | 0.73 | 3.53E-01 | -0.16 | 0.55 | 9.99E-01 | 0.00 | 0.32 | 5.46E-01 | 0.16 | 0.68 | 3177622 | 146334 | 3186696 | 97670 | 3195816 | 106438 | 3177576 | 88175 |
| HILIC_NEG_173_92616a0_918 | HILIC | NEG | 0.918 | 173.9262 | | | | 3.00E-07 | -1.05 | 1.76 | 3.53E-01 | -0.17 | 1.13 | 8.21E-07 | -1.14 | 1.79 | 4.00E-06 | -0.97 | 1.80 | 1132842 | 359366 | 703044 | 367462 | 737349 | 319867 | 668740 | 409995 |
| HILIC_POS_615_74255a0_558 | HILIC | POS | 0.558 | 615.7426 | | | | 7.07E-07 | 0.60 | 1.32 | 3.53E-01 | -0.21 | 0.88 | 6.83E-04 | 0.50 | 1.52 | 2.26E-04 | 0.71 | 1.52 | 91643 | 202518 | 824036 | 1346057 | 949683 | 1508973 | 698389 | 1162541 |
| RP_POS_204_9791a1_46 | RP | POS | 1.46 | 204.9791 | | | | 2.67E-11 | 0.89 | 1.48 | 3.53E-01 | 0.19 | 0.59 | 1.74E-07 | 0.99 | 1.71 | 1.81E-06 | 0.80 | 1.46 | 742842 | 696435 | 2454327 | 1996461 | 2268034 | 1870348 | 2640620 | 2117552 |
| RP_NEG_136_90892a15_534 | RP | NEG | 15.53 | 136.9089 | | | | 1.21E-01 | -0.29 | 0.60 | 3.54E-01 | 0.19 | 0.55 | 3.64E-01 | -0.19 | 0.41 | 7.40E-02 | -0.38 | 0.73 | 1996624 | 346646 | 1879904 | 420824 | 1840658 | 427502 | 1919150 | 414606 |
| RP_NEG_327_82883a15_602 | RP | NEG | 15.60 | 327.8288 | | | | 7.00E-02 | -0.36 | 0.67 | 3.54E-01 | 0.19 | 0.60 | 2.25E-01 | -0.27 | 0.64 | 4.20E-02 | -0.46 | 0.90 | 736640 | 29333 | 725523 | 30486 | 722680 | 30383 | 728365 | 30630 |
| RP_POS_586_4201a11_625 | RP | POS | 11.63 | 586.4201 | | | | 1.15E-06 | -1.21 | 1.94 | 3.54E-01 | -0.14 | 0.72 | 5.95E-07 | -1.28 | 1.95 | 6.88E-06 | -1.14 | 1.73 | 395307 | 190258 | 189553 | 127941 | 201479 | 134841 | 177626 | 120828 |
| RP_POS_271_2056a8_793 | RP | POS | 8.79 | 271.2056 | | | | 2.21E-01 | 0.23 | 0.88 | 3.54E-01 | 0.19 | 0.72 | 1.48E-01 | 0.32 | 0.69 | 5.18E-01 | 0.13 | 0.49 | 644003 | 834388 | 865002 | 1011132 | 770739 | 901829 | 959266 | 1110950 |
| HILIC_POS_258_16983a1_179 | HILIC | POS | 1.179 | 258.1698 | | | | 9.91E-03 | 0.44 | 0.87 | 3.54E-01 | 0.20 | 1.00 | 1.24E-02 | 0.54 | 1.06 | 6.57E-02 | 0.34 | 0.65 | 470625 | 236110 | 613578 | 343226 | 581587 | 299150 | 645570 | 382666 |
| RP_POS_821_37671a7_55 | RP | POS | 7.55 | 821.3767 | | | | 8.61E-01 | 0.04 | 0.76 | 3.55E-01 | 0.18 | 0.76 | 5.87E-01 | 0.13 | 0.68 | 8.28E-01 | -0.05 | 0.78 | 4111118 | 304763 | 4121736 | 270006 | 4096608 | 276805 | 4146864 | 263409 |
| HILIC_NEG_155_95197a1_3 | HILIC | NEG | 1.3 | 155.9520 | | | | 6.36E-07 | 0.88 | 1.37 | 3.55E-01 | 0.18 | 0.94 | 2.11E-06 | 0.97 | 1.59 | 6.70E-05 | 0.79 | 1.36 | 1516015 | 280105 | 1857910 | 381095 | 1822452 | 376749 | 1893367 | 385908 |
| HILIC_POS_560_32776a0_623 | HILIC | POS | 0.623 | 560.3278 | | | | 2.51E-06 | 0.57 | 1.25 | 3.55E-01 | -0.21 | 0.93 | 1.74E-03 | 0.46 | 1.43 | 3.78E-04 | 0.67 | 1.42 | 123817 | 410676 | 1663566 | 3013297 | 1943701 | 3341593 | 1383430 | 2649307 |
| RP_POS_672_52209a10_874 | RP | POS | 10.87 | 672.5221 | | | | 3.96E-02 | -0.41 | 0.79 | 3.56E-01 | -0.19 | 1.05 | 2.36E-02 | -0.50 | 0.87 | 1.58E-01 | -0.31 | 0.89 | 2497813 | 350060 | 2344918 | 379132 | 2380133 | 386046 | 2309703 | 372631 |
| HILIC_POS_443_27264a0_64 | HILIC | POS | 0.64 | 443.2726 | | | | 1.43E-06 | 0.59 | 1.29 | 3.56E-01 | -0.21 | 0.92 | 1.27E-03 | 0.48 | 1.48 | 2.55E-04 | 0.69 | 1.47 | 233349 | 792506 | 2771209 | 4786529 | 3215880 | 5282794 | 2326538 | 4240027 |
| RP_NEG_269_87726a15_537 | RP | NEG | 15.54 | 269.8773 | | | | 7.71E-02 | -0.37 | 0.69 | 3.56E-01 | -0.18 | 0.65 | 4.83E-02 | -0.46 | 0.75 | 2.21E-01 | -0.28 | 0.55 | 724025 | 31635 | 712534 | 30467 | 715364 | 29844 | 709704 | 31119 |
| RP_POS_295_927a15_508 | RP | POS | 15.51 | 295.9270 | | | | 2.12E-01 | 0.24 | 0.67 | 3.56E-01 | -0.19 | 0.57 | 5.13E-01 | 0.14 | 0.30 | 1.19E-01 | 0.34 | 0.67 | 30503925 | 2699607 | 31219095 | 3057346 | 31502959 | 2937066 | 30935231 | 3177214 |
| RP_POS_237_22136a9_676 | RP | POS | 9.68 | 237.2214 | | | | 1.57E-03 | 0.54 | 1.13 | 3.56E-01 | 0.19 | 0.74 | 2.84E-03 | 0.64 | 1.16 | 1.72E-02 | 0.44 | 1.20 | 1147946 | 142031 | 1254156 | 204395 | 1235181 | 180506 | 1273132 | 226031 |
| RP_POS_548_50385a14_506 | RP | POS | 14.51 | 548.5039 | | | | 9.63E-02 | -0.30 | 0.59 | 3.56E-01 | 0.19 | 0.91 | 3.27E-01 | -0.21 | 0.41 | 5.70E-02 | -0.40 | 0.78 | 11455996 | 2027344 | 10711833 | 2569768 | 10473397 | 2550240 | 10950269 | 2592765 |
| RP_NEG_146_93753a15_49 | RP | NEG | 15.49 | 146.9375 | | | | 7.28E-01 | -0.07 | 0.40 | 3.56E-01 | -0.18 | 0.60 | 4.60E-01 | -0.16 | 0.36 | 9.33E-01 | 0.02 | 0.22 | 19249309 | 408198 | 19220512 | 396426 | 19257299 | 432220 | 19183726 | 357686 |
| RP_POS_393_20914a4_35 | RP | POS | 4.35 | 393.2091 | | | | 8.56E-01 | -0.05 | 1.16 | 3.56E-01 | -0.13 | 1.16 | 6.95E-01 | -0.12 | 0.88 | 9.73E-01 | 0.01 | 0.96 | 1362662 | 4510098 | 1212557 | 1935835 | 1392198 | 2141211 | 1032917 | 1709019 |
| RP_POS_478_3891a11_092 | RP | POS | 11.09 | 478.3891 | | | | 5.12E-01 | -0.15 | 0.38 | 3.56E-01 | -0.18 | 0.63 | 3.13E-01 | -0.24 | 0.49 | 8.16E-01 | -0.06 | 0.36 | 2546981 | 1402013 | 2366035 | 1165385 | 2474195 | 1331757 | 2257875 | 972886 |
| RP_POS_569_43256a12_896 | RP | POS | 12.90 | 569.4326 | | | | 1.53E-01 | 0.26 | 0.53 | 3.56E-01 | -0.19 | 0.80 | 4.41E-01 | 0.16 | 0.36 | 8.98E-02 | 0.35 | 0.75 | 2784842 | 1131680 | 3142339 | 1454878 | 3277274 | 1451447 | 3007405 | 1460370 |
| RP_NEG_328_88159a15_384 | RP | NEG | 15.38 | 328.8816 | | | | 8.16E-01 | 0.05 | 0.77 | 3.57E-01 | -0.19 | 0.58 | 8.28E-01 | -0.05 | 0.50 | 5.48E-01 | 0.14 | 0.54 | 2098398 | 66858 | 2101607 | 69393 | 2108036 | 76101 | 2095178 | 62074 |
| RP_POS_368_87076a0_514 | RP | POS | 0.51 | 368.8708 | | | | 1.17E-03 | -0.59 | 0.93 | 3.57E-01 | 0.19 | 0.66 | 2.59E-02 | -0.50 | 0.76 | 3.72E-04 | -0.69 | 1.21 | 590464 | 147161 | 481798 | 186604 | 464507 | 150412 | 499089 | 217069 |
| RP_POS_894_74231a0_497 | RP | POS | 0.50 | 894.7423 | | | | 6.07E-01 | -0.10 | 0.84 | 3.57E-01 | -0.19 | 1.15 | 3.62E-01 | -0.19 | 0.67 | 9.97E-01 | 0.00 | 0.86 | 794731 | 163410 | 776655 | 197036 | 794902 | 202732 | 758407 | 191464 |
| RP_NEG_229_054a7_908 | RP | NEG | 7.91 | 229.0540 | | | | 4.78E-01 | -0.23 | 0.59 | 3.57E-01 | -0.11 | 0.60 | 3.79E-01 | -0.28 | 0.53 | 6.02E-01 | -0.17 | 0.60 | 4037867 | 14166722 | 2207799 | 4802103 | 2652885 | 5807574 | 1762713 | 3529701 |
| RP_POS_371_26544a12_763 | RP | POS | 12.76 | 371.2654 | | | | 1.55E-02 | -0.48 | 0.97 | 3.57E-01 | 0.19 | 0.58 | 7.78E-02 | -0.38 | 0.72 | 1.05E-02 | -0.57 | 1.01 | 734181 | 224900 | 617755 | 243954 | 595174 | 246606 | 640335 | 241632 |
| RP_POS_631_46283a11_253 | RP | POS | 11.25 | 631.4628 | | | | 8.80E-06 | 0.67 | 1.63 | 3.58E-01 | 0.20 | 0.79 | 1.26E-04 | 0.77 | 1.55 | 9.29E-04 | 0.57 | 1.51 | 397602 | 286639 | 750389 | 558931 | 698679 | 504455 | 802098 | 609304 |
| RP_NEG_560_3338a10_933 | RP | NEG | 10.93 | 560.3338 | | | | 6.32E-02 | -0.42 | 1.23 | 3.58E-01 | -0.17 | 1.72 | 4.74E-02 | -0.50 | 1.15 | 1.54E-01 | -0.33 | 1.06 | 953715 | 181688 | 885914 | 154005 | 900159 | 129381 | 871668 | 175402 |
| RP_POS_545_34137a10_618 | RP | POS | 10.62 | 545.3414 | | | | 8.03E-03 | 0.59 | 1.36 | 3.58E-01 | -0.17 | 1.00 | 4.32E-02 | 0.50 | 1.06 | 3.43E-03 | 0.68 | 1.43 | 34441648 | 8739047 | 39200571 | 7524687 | 39896638 | 6209811 | 38504503 | 8651404 |
| HILIC_POS_132_10207a3_434 | HILIC | POS | 3.43 | 132.1021 | | 132.1016(100) | 4 | 7.54E-03 | 0.31 | 0.75 | 3.58E-01 | 0.21 | 1.09 | 6.64E-02 | 0.42 | 0.79 | 9.79E-05 | 0.21 | 1.29 | 5190960 | 4076476 | 17045259 | 42901384 | 13063159 | 12387045 | 21027359 | 59437522 |
| RP_POS_209_15349a9_002 | RP | POS | 9.00 | 209.1535 | | | | 1.94E-02 | -0.49 | 0.98 | 3.58E-01 | 0.18 | 0.83 | 8.56E-02 | -0.40 | 0.96 | 1.12E-02 | -0.58 | 1.11 | 1658536 | 99645 | 1609969 | 97307 | 1600973 | 93569 | 1618964 | 101046 |
| RP_POS_920_74915a0_496 | RP | POS | 0.50 | 920.7492 | | | | 2.62E-01 | -0.24 | 0.66 | 3.58E-01 | -0.18 | 0.77 | 1.69E-01 | -0.33 | 0.56 | 5.09E-01 | -0.15 | 0.54 | 1078877 | 107064 | 1054803 | 95985 | 1063676 | 91040 | 1045931 | 100823 |
| RP_POS_581_50049a10_582 | RP | POS | 10.58 | 581.5005 | | | | 9.00E-04 | 0.58 | 0.92 | 3.58E-01 | -0.19 | 0.60 | 2.06E-02 | 0.48 | 0.83 | 5.98E-04 | 0.67 | 1.24 | 1004899 | 676894 | 1532345 | 942386 | 1619443 | 869945 | 1445247 | 1010951 |
| RP_POS_415_15228a9_931 | RP | POS | 9.93 | 415.1523 | | | | 2.01E-01 | -0.42 | 0.69 | 3.59E-01 | 0.10 | 0.83 | 2.66E-01 | -0.37 | 0.52 | 1.56E-01 | -0.47 | 0.65 | 1046204 | 2460353 | 471251 | 704281 | 406180 | 527373 | 536322 | 845803 |
| HILIC_NEG_265_00073a4_789 | HILIC | NEG | 4.789 | 265.0007 | | | | 9.69E-01 | 0.01 | 1.07 | 3.59E-01 | -0.19 | 1.02 | 6.84E-01 | -0.09 | 0.77 | 6.08E-01 | 0.10 | 0.98 | 634219 | 183921 | 635755 | 228790 | 656850 | 208293 | 614660 | 247918 |
| RP_POS_271_22681a10_12 | RP | POS | 10.12 | 271.2268 | | | | 1.86E-02 | -0.42 | 0.80 | 3.59E-01 | -0.19 | 0.91 | 1.15E-02 | -0.52 | 0.99 | 1.18E-01 | -0.33 | 0.86 | 2017122 | 202341 | 1910177 | 262059 | 1934324 | 271028 | 1886030 | 253191 |
| RP_POS_553_45679a15_1 | RP | POS | 15.10 | 553.4568 | | | | 2.17E-01 | 0.24 | 0.54 | 3.59E-01 | -0.19 | 0.55 | 5.10E-01 | 0.14 | 0.28 | 1.31E-01 | 0.33 | 0.75 | 945541564 | 105691166 | 973242877 | 120215347 | 984319139 | 121786788 | 962166615 | 118810758 |
| HILIC_POS_692_40607a0_627 | HILIC | POS | 0.627 | 692.4061 | | | | 2.91E-06 | 0.57 | 1.27 | 3.60E-01 | -0.20 | 0.93 | 2.01E-03 | 0.46 | 1.45 | 3.77E-04 | 0.67 | 1.45 | 129792 | 367949 | 1437295 | 2570890 | 1674156 | 2832655 | 1200435 | 2283728 |
| HILIC_POS_125_98631a7_963 | HILIC | POS | 7.963 | 125.9863 | | | | 1.39E-05 | -0.66 | 1.28 | 3.60E-01 | 0.20 | 0.89 | 2.81E-03 | -0.56 | 1.34 | 5.41E-05 | -0.75 | 1.40 | 7039179 | 1594833 | 5136261 | 3071742 | 4853327 | 3017714 | 5419194 | 3129504 |
| RP_POS_345_1944a11_159 | RP | POS | 11.16 | 345.1944 | | | | 4.91E-02 | -0.35 | 0.83 | 3.60E-01 | 0.19 | 0.90 | 2.15E-01 | -0.26 | 0.69 | 3.11E-02 | -0.45 | 0.88 | 5107982 | 316093 | 4968784 | 412643 | 4930790 | 411560 | 5006777 | 414363 |
| RP_NEG_366_68091a15_577 | RP | NEG | 15.58 | 366.6809 | | | | 3.42E-01 | -0.20 | 0.56 | 3.60E-01 | -0.18 | 0.55 | 2.11E-01 | -0.29 | 0.60 | 6.30E-01 | -0.11 | 0.39 | 1091836 | 52786 | 1081755 | 48303 | 1086200 | 49368 | 1077309 | 47290 |
| RP_NEG_176_81676a15_521 | RP | NEG | 15.52 | 176.8168 | | | | 9.07E-01 | 0.02 | 0.51 | 3.60E-01 | -0.19 | 0.58 | 7.25E-01 | -0.07 | 0.34 | 6.08E-01 | 0.12 | 0.37 | 1582931 | 66770 | 1584582 | 75938 | 1591570 | 84259 | 1577594 | 66721 |
| RP_POS_329_24725a10_907 | RP | POS | 10.91 | 329.2473 | | | | 2.41E-03 | 0.40 | 1.42 | 3.60E-01 | 0.20 | 0.71 | 1.23E-02 | 0.50 | 1.11 | 3.98E-02 | 0.30 | 1.36 | 6981151 | 3109192 | 10297581 | 9199493 | 9450870 | 7377909 | 11144292 | 10728088 |
| RP_NEG_822_62433a10_957 | RP | NEG | 10.96 | 822.6243 | | | | 4.48E-03 | -0.63 | 1.27 | 3.60E-01 | -0.17 | 1.30 | 2.54E-03 | -0.72 | 1.28 | 2.33E-02 | -0.55 | 1.15 | 1228851 | 345253 | 1026047 | 297297 | 1053394 | 312975 | 998699 | 281238 |
| HILIC_POS_175_11884a7_095 | HILIC | POS | 7.095 | 175.1188 | | | | 1.33E-02 | 0.31 | 1.07 | 3.60E-01 | -0.21 | 0.87 | 1.92E-01 | 0.21 | 1.06 | 2.41E-02 | 0.41 | 1.14 | 1089128 | 337624 | 1468772 | 1388042 | 1596456 | 1489167 | 1341088 | 1281308 |
| RP_POS_806_56732a13_58 | RP | POS | 13.58 | 806.5673 | | | | 2.03E-09 | 1.02 | 1.64 | 3.61E-01 | 0.18 | 0.58 | 8.95E-08 | 1.11 | 1.72 | 4.37E-07 | 0.93 | 1.69 | 6305859 | 4776515 | 13787870 | 7098897 | 13135265 | 6404915 | 14440475 | 7741126 |
| RP_NEG_213_99808a3_294 | RP | NEG | 3.29 | 213.9981 | | | | 1.28E-01 | -0.34 | 0.75 | 3.61E-01 | -0.17 | 0.54 | 8.45E-02 | -0.43 | 0.85 | 2.86E-01 | -0.25 | 0.51 | 1189256 | 796658 | 947101 | 676960 | 1009326 | 634812 | 884876 | 717645 |
| RP_NEG_1043_6123a10_313 | RP | NEG | 10.31 | 1043.6123 | | | | 3.87E-03 | 0.48 | 1.10 | 3.61E-01 | -0.19 | 0.61 | 5.00E-02 | 0.38 | 0.84 | 4.11E-03 | 0.57 | 1.11 | 1657101 | 503007 | 2009843 | 785951 | 2082041 | 799406 | 1937645 | 773534 |
| RP_NEG_297_15356a13_293 | RP | NEG | 13.29 | 297.1536 | | | | 8.72E-02 | -0.39 | 0.82 | 3.61E-01 | -0.17 | 0.75 | 4.69E-02 | -0.48 | 0.92 | 2.28E-01 | -0.30 | 0.84 | 8085243 | 6181641 | 5981073 | 5040443 | 6444005 | 5576648 | 5518140 | 4449074 |
| RP_POS_184_98494a0_895 | RP | POS | 0.90 | 184.9849 | | | | 5.05E-01 | 0.14 | 0.40 | 3.61E-01 | -0.18 | 0.58 | 8.43E-01 | 0.04 | 0.60 | 3.27E-01 | 0.23 | 0.37 | 5632422 | 2854956 | 6023003 | 2883349 | 6287768 | 3045716 | 5758238 | 2716177 |
| RP_NEG_203_00211a3_478 | RP | NEG | 3.48 | 203.0021 | | | | 7.96E-01 | 0.05 | 0.44 | 3.61E-01 | 0.19 | 0.84 | 5.47E-01 | 0.14 | 0.57 | 8.48E-01 | -0.04 | 0.46 | 591385 | 525807 | 619214 | 537396 | 569858 | 443632 | 668570 | 617821 |
| RP_POS_249_1824a8_323 | RP | POS | 8.32 | 249.1824 | | | | 7.67E-01 | -0.06 | 0.36 | 3.61E-01 | 0.19 | 0.73 | 8.89E-01 | 0.03 | 0.17 | 4.77E-01 | -0.15 | 0.35 | 1046312 | 57232 | 1042822 | 59000 | 1037404 | 51327 | 1048239 | 65868 |
| RP_POS_604_45929a13_499 | RP | POS | 13.50 | 604.4593 | | | | 9.62E-01 | -0.01 | 0.27 | 3.61E-01 | 0.21 | 0.60 | 6.69E-01 | 0.10 | 0.28 | 3.94E-01 | -0.11 | 0.34 | 1857812 | 226164 | 1854790 | 497995 | 1808993 | 286718 | 1900587 | 643873 |
| RP_NEG_273_82733a15_561 | RP | NEG | 15.56 | 273.8273 | | | | 7.01E-02 | -0.34 | 0.83 | 3.62E-01 | -0.19 | 0.79 | 3.24E-02 | -0.44 | 0.93 | 2.72E-01 | -0.25 | 0.68 | 1126384 | 49729 | 1106906 | 58561 | 1112281 | 65275 | 1101532 | 51079 |
| HILIC_POS_478_29205a1_683 | HILIC | POS | 1.683 | 478.2921 | | | | 4.04E-02 | 0.40 | 0.65 | 3.62E-01 | -0.18 | 0.69 | 1.85E-01 | 0.31 | 0.48 | 1.59E-02 | 0.50 | 0.94 | 429527 | 218170 | 524352 | 235604 | 545980 | 189093 | 502724 | 274664 |
| RP_POS_317_16113a10_704 | RP | POS | 10.70 | 317.1611 | | | | 1.93E-02 | 0.45 | 1.26 | 3.62E-01 | 0.19 | 1.15 | 1.82E-02 | 0.54 | 1.25 | 7.81E-02 | 0.36 | 1.11 | 871834 | 173519 | 959458 | 196365 | 941442 | 168941 | 977473 | 220685 |
| HILIC_NEG_476_27966a1_827 | HILIC | NEG | 1.827 | 476.2797 | | | | 3.92E-02 | 0.41 | 0.66 | 3.62E-01 | -0.18 | 0.69 | 1.79E-01 | 0.32 | 0.49 | 1.57E-02 | 0.50 | 0.94 | 751125 | 371858 | 913311 | 396752 | 949700 | 319748 | 876923 | 461622 |
| RP_NEG_893_19354a9_697 | RP | NEG | 9.70 | 893.1935 | | | | 6.65E-01 | -0.09 | 0.61 | 3.62E-01 | -0.18 | 0.57 | 4.22E-01 | -0.18 | 0.54 | 9.87E-01 | 0.00 | 0.39 | 9315105 | 678664 | 9254932 | 684352 | 9317660 | 707018 | 9192203 | 662063 |
| RP_POS_127_95573a15_424 | RP | POS | 15.42 | 127.9557 | | | | 4.87E-01 | -0.12 | 0.61 | 3.62E-01 | 0.20 | 0.71 | 9.10E-01 | -0.02 | 0.16 | 3.35E-01 | -0.22 | 0.86 | 3511963 | 337285 | 3458136 | 488528 | 3413342 | 582556 | 3502929 | 372456 |
| RP_POS_870_78503a0_496 | RP | POS | 0.50 | 870.7850 | | | | 1.73E-09 | -0.73 | 1.39 | 3.62E-01 | 0.20 | 1.21 | 9.67E-05 | -0.63 | 1.43 | 4.68E-06 | -0.83 | 1.54 | 7117044 | 279013 | 5160484 | 2922236 | 4892782 | 3045733 | 5428187 | 2798296 |
| RP_POS_343_33795a10_978 | RP | POS | 10.98 | 343.3380 | | | | 6.19E-02 | 0.34 | 1.24 | 3.62E-01 | -0.19 | 1.29 | 2.50E-01 | 0.24 | 0.98 | 3.57E-02 | 0.43 | 1.15 | 7367961 | 2381456 | 8352164 | 3021870 | 8628959 | 2919907 | 8075369 | 3125306 |
| RP_POS_341_20868a9_419 | RP | POS | 9.42 | 341.2087 | | | | 6.25E-01 | 0.07 | 0.31 | 3.62E-01 | -0.20 | 0.70 | 8.09E-01 | -0.03 | 0.24 | 4.33E-01 | 0.17 | 0.40 | 446878 | 782097 | 555807 | 1742754 | 715683 | 2200018 | 395931 | 1115404 |
| RP_POS_723_46436a10_697 | RP | POS | 10.70 | 723.4644 | | | | 5.16E-01 | -0.12 | 0.60 | 3.62E-01 | -0.19 | 0.74 | 3.16E-01 | -0.22 | 0.44 | 8.95E-01 | -0.03 | 0.55 | 1459921 | 357227 | 1410590 | 414116 | 1448516 | 412185 | 1372664 | 416716 |
| HILIC_POS_586_39197a0_549 | HILIC | POS | 0.549 | 586.3920 | | | | 1.48E-06 | 0.61 | 1.34 | 3.62E-01 | -0.20 | 0.90 | 9.83E-04 | 0.51 | 1.51 | 1.91E-04 | 0.71 | 1.51 | 64760 | 191302 | 477330 | 743214 | 545399 | 817856 | 409262 | 661560 |
| RP_POS_478_38882a13_804 | RP | POS | 13.80 | 478.3888 | | | | 6.40E-01 | 0.11 | 0.50 | 3.63E-01 | 0.17 | 0.68 | 4.27E-01 | 0.20 | 0.71 | 9.21E-01 | 0.03 | 0.98 | 6981058 | 1835529 | 7147343 | 1388830 | 7020228 | 1557992 | 7274457 | 1198644 |
| HILIC_POS_116_62675a4_87 | HILIC | POS | 4.87 | 116.6268 | | | | 8.26E-01 | -0.06 | 0.57 | 3.63E-01 | -0.15 | 0.68 | 6.29E-01 | -0.13 | 0.60 | 9.46E-01 | 0.02 | 0.36 | 395912 | 421989 | 378434 | 251973 | 401481 | 240018 | 355387 | 263787 |
| RP_POS_504_30576a10_932 | RP | POS | 10.93 | 504.3058 | | | | 4.71E-02 | 0.35 | 1.13 | 3.64E-01 | 0.19 | 1.00 | 4.81E-02 | 0.44 | 0.94 | 1.68E-01 | 0.25 | 0.97 | 2167674 | 545146 | 2413785 | 745185 | 2345666 | 592375 | 2481904 | 872672 |
| RP_POS_281_17172a8_909 | RP | POS | 8.91 | 281.1717 | | | | 1.61E-01 | 0.28 | 0.66 | 3.64E-01 | 0.18 | 0.54 | 1.21E-01 | 0.37 | 0.71 | 3.58E-01 | 0.19 | 0.43 | 2832056 | 472488 | 2969384 | 487706 | 2924806 | 389871 | 3013962 | 569661 |
| RP_POS_149_02342a9_524 | RP | POS | 9.52 | 149.0234 | | | | 4.47E-01 | 0.10 | 0.37 | 3.64E-01 | 0.21 | 0.62 | 3.75E-01 | 0.21 | 0.49 | 9.95E-01 | 0.00 | 0.30 | 2077763 | 89182 | 2099783 | 241759 | 2077629 | 85815 | 2121937 | 331242 |
| RP_POS_161_98148a0_252 | RP | POS | 0.25 | 161.9815 | | | | 2.55E-01 | -0.21 | 0.39 | 3.64E-01 | 0.19 | 0.58 | 5.89E-01 | -0.11 | 0.51 | 1.54E-01 | -0.30 | 0.57 | 1198635 | 167991 | 1156942 | 209115 | 1137854 | 212468 | 1176030 | 206070 |
| RP_NEG_201_0378a0_716 | RP | NEG | 0.72 | 201.0378 | | | | 1.62E-06 | 0.69 | 1.25 | 3.64E-01 | 0.19 | 1.03 | 1.47E-04 | 0.78 | 1.36 | 1.46E-04 | 0.59 | 1.25 | 1420772 | 423937 | 2033624 | 953317 | 1946554 | 765725 | 2120693 | 1111021 |
| HILIC_POS_340_04672a3_077 | HILIC | POS | 3.077 | 340.0467 | | | | 6.38E-01 | 0.06 | 1.23 | 3.64E-01 | -0.21 | 0.96 | 8.27E-01 | -0.04 | 1.26 | 3.51E-01 | 0.17 | 1.31 | 1309586 | 81787 | 1321700 | 212645 | 1341105 | 214192 | 1302295 | 211452 |
| RP_NEG_203_88559a15_54 | RP | NEG | 15.54 | 203.8856 | | | | 2.52E-01 | 0.34 | 0.82 | 3.64E-01 | -0.13 | 0.56 | 3.69E-01 | 0.28 | 0.49 | 1.81E-01 | 0.40 | 0.65 | 1214055 | 248054 | 1266710 | 108252 | 1276586 | 93838 | 1256833 | 121127 |
| RP_NEG_334_8707a15_435 | RP | NEG | 15.44 | 334.8707 | | | | 1.23E-01 | 0.26 | 0.56 | 3.64E-01 | -0.20 | 0.77 | 3.82E-01 | 0.16 | 0.51 | 9.59E-02 | 0.36 | 0.77 | 1626978 | 310500 | 1739481 | 467559 | 1782137 | 522786 | 1696825 | 405791 |
| RP_POS_327_20126a4_023 | RP | POS | 4.02 | 327.2013 | | | | 9.33E-01 | -0.02 | 1.12 | 3.65E-01 | -0.14 | 1.15 | 7.54E-01 | -0.09 | 0.88 | 8.86E-01 | 0.04 | 0.93 | 898870 | 2870324 | 854204 | 1360865 | 978248 | 1509394 | 730160 | 1196674 |
| HILIC_POS_182_0811a5_12 | HILIC | POS | 5.12 | 182.0811 | | | | 3.88E-01 | 0.15 | 0.90 | 3.65E-01 | 0.19 | 1.05 | 2.64E-01 | 0.25 | 0.40 | 7.72E-01 | 0.05 | 0.45 | 9533681 | 2842971 | 10085600 | 3890895 | 9730976 | 3229157 | 10440223 | 4461276 |
| HILIC_POS_418_20605a0_952 | HILIC | POS | 0.952 | 418.2061 | | | | 6.25E-01 | 0.11 | 0.43 | 3.65E-01 | 0.18 | 0.58 | 3.83E-01 | 0.19 | 0.45 | 9.47E-01 | 0.02 | 0.37 | 1067199 | 609187 | 1126725 | 550600 | 1076540 | 639694 | 1176911 | 445152 |
| RP_POS_533_34515a10_456 | RP | POS | 10.46 | 533.3452 | | | | 5.98E-01 | -0.10 | 0.65 | 3.65E-01 | -0.19 | 0.71 | 3.50E-01 | -0.19 | 1.10 | 9.98E-01 | 0.00 | 0.33 | 2405439 | 637367 | 2332300 | 797364 | 2404934 | 842097 | 2259665 | 751440 |
| RP_POS_478_38867a13_234 | RP | POS | 13.23 | 478.3887 | | | | 7.21E-01 | 0.08 | 0.56 | 3.65E-01 | -0.18 | 0.70 | 9.63E-01 | -0.01 | 0.32 | 4.76E-01 | 0.17 | 0.44 | 3445168 | 776846 | 3500517 | 696669 | 3563957 | 654653 | 3437077 | 737440 |
| RP_POS_419_31384a11_88 | RP | POS | 11.88 | 419.3138 | | | | 2.81E-01 | -0.23 | 0.67 | 3.66E-01 | 0.18 | 0.67 | 5.60E-01 | -0.14 | 0.57 | 1.67E-01 | -0.32 | 0.61 | 123651172 | 17731410 | 119767466 | 16887345 | 118231123 | 16155484 | 121303809 | 17617270 |
| RP_POS_369_38385a11_668 | RP | POS | 11.67 | 369.3839 | | | | 8.93E-01 | -0.03 | 0.64 | 3.66E-01 | -0.18 | 0.54 | 6.01E-01 | -0.12 | 0.43 | 7.81E-01 | 0.06 | 0.41 | 961605 | 460588 | 948955 | 461063 | 990878 | 468615 | 907033 | 454214 |

**Supplementary table 1: Metabolomics results** (in asceding order according to p-value from progressive ALD vs  non-progressive ALD comparison, with molecular features with ID_level of 1-2 first)

| | | | | | | | | all_ALD_vs_control | | | Prog_ALD_vs_non-prog_ALD | | | Prog_ALD_vs_control_ALD | | | Non-prog_ALD_vs_control | | | Healthy_control | | all_ALD | | Non-prog_ALD | | Progressive_ALD | |
|---|---|---|---|---|---|---|---|---|---|---|---|---|---|---|---|---|---|---|---|---|---|---|---|---|---|---|---|
| | | | | | | | | t-test | Cohen's | PLS-DA | t-test | Cohen's | PLS-DA | t-test | Cohen's | PLS-DA | t-test | Cohen's | PLS-DA | | | | | | | | |
| Feature_ID | RP_HILIC | POS_NEG | Average.Rt | Average.Mz | Metabolite.name | curated_MSMS | ID_level | p | d | VIP | p | d | VIP | p | d | VIP | p | d | VIP | Mean | SD | Mean | SD | Mean | SD | Mean | SD |
| RP_POS_152_0222a0_55 | RP | POS | 0.55 | 152.0222 | | | | 3.03E-12 | -1.02 | 1.69 | 3.66E-01 | -0.18 | 1.22 | 6.52E-10 | -1.11 | 1.93 | 6.13E-07 | -0.93 | 1.57 | 1046139 | 172718 | 675109 | 361684 | 707979 | 380908 | 642238 | 342060 |
| RP_POS_645_27142a7_257 | RP | POS | 7.26 | 645.2714 | | | | 4.43E-01 | -0.15 | 0.68 | 3.67E-01 | 0.19 | 0.78 | 8.08E-01 | -0.05 | 0.48 | 2.70E-01 | -0.24 | 0.73 | 13524142 | 780964 | 13397150 | 895882 | 13315801 | 898113 | 13478500 | 895252 |
| HILIC_POS_190_14349a0_778 | HILIC | POS | 0.778 | 190.1435 | | | | 7.98E-01 | -0.05 | 0.44 | 3.67E-01 | 0.19 | 0.68 | 8.52E-01 | 0.04 | 0.17 | 4.92E-01 | -0.14 | 0.41 | 728452 | 65523 | 724948 | 72074 | 718402 | 62010 | 731494 | 81011 |
| HILIC_POS_478_29379a1_923 | HILIC | POS | 1.923 | 478.2938 | | | | 4.34E-02 | 0.40 | 0.66 | 3.67E-01 | -0.18 | 0.69 | 1.95E-01 | 0.30 | 0.47 | 1.72E-02 | 0.49 | 0.93 | 3611661 | 1787673 | 4379115 | 1952083 | 4556433 | 1559387 | 4201797 | 2281045 |
| RP_POS_195_11301a2_973 | RP | POS | 2.97 | 195.1130 | | | | 1.45E-01 | 0.24 | 0.55 | 3.67E-01 | -0.19 | 0.60 | 5.10E-01 | 0.14 | 0.39 | 5.18E-02 | 0.34 | 0.83 | 1459927 | 579357 | 1658863 | 882346 | 1738952 | 687028 | 1578775 | 1043005 |
| RP_POS_182_02362a15_525 | RP | POS | 15.53 | 182.0236 | | | | 8.59E-01 | -0.03 | 0.62 | 3.67E-01 | -0.20 | 0.69 | 5.29E-01 | -0.13 | 0.65 | 7.11E-01 | 0.07 | 0.52 | 37098319 | 4194219 | 36919095 | 6766250 | 37532707 | 6379948 | 36305484 | 7143280 |
| RP_POS_996_72632a0_497 | RP | POS | 0.50 | 996.7263 | | | | 7.19E-01 | -0.07 | 0.89 | 3.67E-01 | -0.19 | 1.06 | 4.61E-01 | -0.16 | 0.48 | 8.94E-01 | 0.03 | 0.99 | 672657 | 115033 | 663759 | 138593 | 676323 | 131281 | 651194 | 145777 |
| HILIC_NEG_99_92569a0_927 | HILIC | NEG | 0.927 | 99.9257 | | | | 1.50E-04 | 0.57 | 1.03 | 3.67E-01 | -0.19 | 1.21 | 1.76E-02 | 0.47 | 0.77 | 1.31E-04 | 0.66 | 1.31 | 3109827 | 713741 | 3853462 | 1407940 | 3981078 | 1245452 | 3725845 | 1555846 |
| RP_POS_296_29483a11_056 | RP | POS | 11.06 | 296.2948 | | | | 8.58E-06 | 0.56 | 1.00 | 3.68E-01 | 0.20 | 1.01 | 3.56E-03 | 0.66 | 1.04 | 1.91E-07 | 0.46 | 1.68 | 556433 | 219023 | 992301 | 855981 | 914562 | 349651 | 1070040 | 1160074 |
| RP_POS_315_68723a5_488 | RP | POS | 5.49 | 315.6872 | | | | 1.34E-06 | 0.58 | 1.31 | 3.68E-01 | -0.20 | 1.23 | 1.56E-03 | 0.48 | 1.37 | 2.53E-04 | 0.68 | 1.41 | 93807 | 154215 | 876661 | 1501926 | 1012618 | 1638575 | 740704 | 1354603 |
| RP_NEG_723_08893a9_125 | RP | NEG | 9.13 | 723.0889 | | | | 7.85E-01 | 0.06 | 0.14 | 3.68E-01 | 0.17 | 1.20 | 5.51E-01 | 0.15 | 0.43 | 9.18E-01 | -0.02 | 0.24 | 5074091 | 437122 | 5097582 | 364652 | 5064587 | 347986 | 5130577 | 381235 |
| RP_POS_822_63617a14_714 | RP | POS | 14.71 | 822.6362 | | | | 5.56E-04 | -0.83 | 1.38 | 3.68E-01 | 0.16 | 0.70 | 2.77E-03 | -0.75 | 1.20 | 3.51E-04 | -0.91 | 1.51 | 2313009 | 1319226 | 1368688 | 979579 | 1280073 | 961536 | 1457304 | 999079 |
| RP_POS_564_41315a14_947 | RP | POS | 14.95 | 564.4132 | | | | 1.19E-02 | 0.34 | 0.76 | 3.69E-01 | 0.20 | 0.57 | 4.87E-02 | 0.44 | 0.77 | 3.64E-02 | 0.24 | 0.71 | 2112685 | 235805 | 2308872 | 646239 | 2250313 | 349548 | 2367430 | 845334 |
| RP_POS_181_98769a15_655 | RP | POS | 15.66 | 181.9877 | | | | 3.89E-01 | -0.19 | 0.51 | 3.69E-01 | 0.17 | 0.69 | 6.74E-01 | -0.10 | 0.82 | 2.35E-01 | -0.28 | 0.77 | 44699056 | 24310836 | 40545603 | 20779994 | 38666588 | 18391101 | 42424618 | 22956276 |
| HILIC_POS_445_28815a0_637 | HILIC | POS | 0.637 | 445.2882 | | | | 5.39E-07 | 0.60 | 1.29 | 3.70E-01 | -0.20 | 0.89 | 9.84E-04 | 0.50 | 1.45 | 1.77E-04 | 0.70 | 1.50 | 39187 | 70759 | 997864 | 1784670 | 1158943 | 1949402 | 836786 | 1606853 |
| RP_POS_189_0437a15_341 | RP | POS | 15.34 | 189.0437 | | | | 1.47E-01 | -0.31 | 0.50 | 3.70E-01 | -0.18 | 0.59 | 9.57E-02 | -0.40 | 0.65 | 3.32E-01 | -0.22 | 0.40 | 1834353 | 576377 | 1664958 | 529148 | 1712711 | 505601 | 1617206 | 552651 |
| RP_POS_385_17462a11_161 | RP | POS | 11.16 | 385.1746 | | | | 4.35E-01 | 0.14 | 0.84 | 3.70E-01 | 0.19 | 0.86 | 2.74E-01 | 0.23 | 0.76 | 8.36E-01 | 0.04 | 0.89 | 1133408 | 201575 | 1168708 | 274269 | 1143960 | 256314 | 1193456 | 291609 |
| RP_POS_794_06061a13_275 | RP | POS | 13.28 | 794.0606 | | | | 9.29E-01 | 0.02 | 0.68 | 3.70E-01 | -0.18 | 0.62 | 7.59E-01 | -0.07 | 0.24 | 6.56E-01 | 0.11 | 0.80 | 2881867 | 924184 | 2898307 | 854921 | 2975445 | 924873 | 2821169 | 780488 |
| RP_POS_244_92317a0_497 | RP | POS | 0.50 | 244.9232 | | | | 1.23E-08 | -0.69 | 1.35 | 3.70E-01 | 0.20 | 1.20 | 2.19E-04 | -0.59 | 1.38 | 1.60E-05 | -0.79 | 1.50 | 8774874 | 178586 | 7375669 | 2237170 | 7173865 | 2359240 | 7577474 | 2112552 |
| RP_POS_199_13052a13_33 | RP | POS | 13.33 | 199.1305 | | | | 7.40E-01 | 0.06 | 0.35 | 3.70E-01 | 0.19 | 0.86 | 4.62E-01 | 0.16 | 0.49 | 8.98E-01 | -0.03 | 0.62 | 21467623 | 5849341 | 21875979 | 6566084 | 21284158 | 6960148 | 22467800 | 6160221 |
| RP_POS_380_89728a0_497 | RP | POS | 0.50 | 380.8973 | | | | 1.34E-08 | -0.70 | 1.35 | 3.70E-01 | 0.20 | 1.19 | 1.92E-04 | -0.60 | 1.37 | 1.47E-05 | -0.79 | 1.51 | 2402346 | 98306 | 2002052 | 629612 | 1945318 | 664889 | 2058787 | 593521 |
| RP_POS_401_34161a10_636 | RP | POS | 10.64 | 401.3416 | | | | 7.57E-03 | -0.53 | 0.93 | 3.70E-01 | -0.18 | 1.00 | 5.62E-03 | -0.62 | 1.06 | 4.44E-02 | -0.44 | 0.82 | 601005 | 297203 | 430268 | 322108 | 459281 | 318567 | 401256 | 326224 |
| HILIC_POS_423_27502a0_633 | HILIC | POS | 0.633 | 423.2750 | | | | 4.57E-07 | 0.60 | 1.30 | 3.70E-01 | -0.20 | 0.90 | 8.54E-04 | 0.50 | 1.46 | 1.64E-04 | 0.70 | 1.51 | 69910 | 172716 | 1440487 | 2524328 | 1667877 | 2761723 | 1213097 | 2267570 |
| RP_POS_617_50989a11_165 | RP | POS | 11.17 | 617.5099 | | | | 6.37E-01 | -0.10 | 0.54 | 3.71E-01 | -0.17 | 0.59 | 4.13E-01 | -0.19 | 0.52 | 9.43E-01 | -0.02 | 0.72 | 1178129 | 2206071 | 972081 | 1895385 | 1142796 | 2094136 | 801366 | 1677320 |
| HILIC_POS_342_04358a3_078 | HILIC | POS | 3.078 | 342.0436 | | | | 6.32E-01 | 0.06 | 1.26 | 3.71E-01 | -0.20 | 0.95 | 8.36E-01 | -0.04 | 1.30 | 3.51E-01 | 0.17 | 1.40 | 542570 | 33034 | 547635 | 87883 | 555542 | 88488 | 539729 | 87445 |
| RP_POS_548_36963a10_647 | RP | POS | 10.65 | 548.3696 | | | | 9.27E-02 | 0.37 | 0.89 | 3.71E-01 | -0.17 | 0.74 | 2.53E-01 | 0.28 | 0.65 | 4.91E-02 | 0.45 | 0.96 | 1385468 | 464818 | 1543192 | 412185 | 1580276 | 361698 | 1506109 | 457852 |
| RP_POS_431_27673a9_325 | RP | POS | 9.33 | 431.2767 | Unknown | 431.2795(100) | 4 | 2.59E-01 | -0.33 | 0.65 | 3.71E-01 | 0.13 | 0.57 | 3.86E-01 | -0.26 | 0.43 | 1.84E-01 | -0.39 | 0.59 | 1268391 | 2591023 | 724099 | 1221984 | 614172 | 1098872 | 834026 | 1335958 |
| HILIC_POS_601_0672a0_553 | HILIC | POS | 0.553 | 601.0672 | | | | 8.92E-07 | 0.62 | 1.33 | 3.71E-01 | -0.20 | 0.89 | 6.56E-04 | 0.52 | 1.51 | 1.78E-04 | 0.72 | 1.51 | 101167 | 274338 | 734874 | 1125280 | 836103 | 1248183 | 633645 | 989686 |
| RP_NEG_165_97849a6_183 | RP | NEG | 6.18 | 165.9785 | | | | 4.40E-02 | 0.38 | 0.97 | 3.71E-01 | -0.18 | 0.73 | 1.46E-01 | 0.29 | 0.84 | 3.97E-02 | 0.47 | 0.97 | 1472406 | 1268511 | 2026848 | 1494970 | 2161306 | 1705566 | 1892390 | 1252848 |
| HILIC_POS_467_30136a0_64 | HILIC | POS | 0.64 | 467.3014 | | | | 8.41E-07 | 0.61 | 1.32 | 3.72E-01 | -0.20 | 0.90 | 1.01E-03 | 0.51 | 1.48 | 1.65E-04 | 0.71 | 1.51 | 52250 | 201635 | 630997 | 1053894 | 725647 | 1147798 | 536347 | 953063 |
| RP_POS_326_30536a10_432 | RP | POS | 10.43 | 326.3054 | | | | 4.14E-01 | -0.24 | 0.91 | 3.72E-01 | -0.13 | 0.66 | 3.12E-01 | -0.31 | 0.73 | 5.61E-01 | -0.18 | 0.57 | 1527235 | 3353051 | 1022680 | 1471704 | 1154799 | 1615403 | 890560 | 1315627 |
| RP_POS_223_09459a10_449 | RP | POS | 10.45 | 223.0946 | | | | 8.56E-01 | 0.03 | 0.48 | 3.72E-01 | -0.19 | 0.57 | 7.86E-01 | -0.06 | 0.61 | 5.68E-01 | 0.13 | 0.36 | 6877871 | 2433436 | 6971085 | 2757821 | 7218608 | 2897192 | 6723562 | 2616793 |
| RP_POS_345_2171a8_422 | RP | POS | 8.42 | 345.2171 | | | | 8.46E-01 | 0.04 | 0.35 | 3.72E-01 | 0.18 | 0.68 | 6.01E-01 | 0.13 | 0.45 | 8.41E-01 | -0.05 | 0.42 | 6693103 | 8824097 | 7034989 | 7917610 | 6324431 | 6816223 | 7745547 | 8897481 |
| HILIC_POS_325_19104a0_671 | HILIC | POS | 0.671 | 325.1910 | | | | 4.71E-02 | 0.28 | 0.58 | 3.72E-01 | 0.20 | 0.55 | 6.56E-02 | 0.38 | 0.59 | 2.35E-01 | 0.18 | 0.60 | 2101175 | 2462352 | 3548010 | 5750117 | 3031855 | 4556444 | 4064164 | 6745188 |
| RP_POS_195_12299a2_163 | RP | POS | 2.16 | 195.1230 | | | | 1.29E-01 | 0.22 | 0.92 | 3.72E-01 | -0.20 | 0.91 | 4.94E-01 | 0.12 | 1.01 | 9.91E-02 | 0.32 | 0.93 | 5297949 | 558634 | 5540107 | 1239301 | 5651273 | 1322136 | 5428941 | 1153175 |
| RP_POS_388_25406a4_35 | RP | POS | 4.35 | 388.2541 | | | | 8.46E-01 | -0.06 | 1.16 | 3.73E-01 | -0.12 | 1.15 | 6.92E-01 | -0.12 | 0.88 | 9.89E-01 | 0.00 | 0.96 | 2799419 | 8989331 | 2479782 | 3829582 | 2823230 | 4229584 | 2136335 | 3391251 |
| RP_POS_225_14847a9_418 | RP | POS | 9.42 | 225.1485 | | | | 1.89E-01 | -0.36 | 0.88 | 3.73E-01 | -0.14 | 0.68 | 1.36E-01 | -0.43 | 0.70 | 3.00E-01 | -0.29 | 0.61 | 1128600 | 1454227 | 769142 | 772892 | 838418 | 719267 | 699866 | 824485 |
| RP_POS_765_05273a12_788 | RP | POS | 12.79 | 765.0527 | | | | 2.75E-07 | -1.21 | 1.98 | 3.73E-01 | -0.14 | 0.56 | 2.33E-07 | -1.28 | 1.96 | 1.65E-06 | -1.14 | 1.82 | 787922 | 355373 | 371844 | 271075 | 396112 | 253767 | 347576 | 287861 |
| RP_NEG_216_85312a15_604 | RP | NEG | 15.60 | 216.8531 | | | | 1.24E-01 | -0.33 | 0.79 | 3.74E-01 | -0.18 | 0.89 | 7.64E-02 | -0.41 | 0.72 | 3.07E-01 | -0.24 | 0.68 | 4171689 | 176254 | 4116494 | 165920 | 4131339 | 167721 | 4101649 | 164440 |
| RP_NEG_183_01538a0_715 | RP | NEG | 0.72 | 183.0154 | | | | 1.50E-02 | 0.42 | 0.86 | 3.74E-01 | -0.19 | 0.56 | 1.16E-01 | 0.33 | 0.81 | 9.25E-03 | 0.51 | 0.88 | 1229092 | 284114 | 1389401 | 402145 | 1425382 | 379884 | 1353421 | 424013 |
| RP_POS_659_28522a10_563 | RP | POS | 10.56 | 659.2852 | | | | 2.95E-05 | -0.77 | 1.20 | 3.74E-01 | 0.18 | 0.62 | 2.98E-03 | -0.68 | 1.00 | 5.14E-06 | -0.85 | 1.61 | 4239253 | 334294 | 3912636 | 424328 | 3874634 | 302654 | 3950637 | 518880 |
| RP_POS_198_90514a15_579 | RP | POS | 15.58 | 198.9051 | | | | 2.91E-01 | 0.19 | 0.42 | 3.74E-01 | 0.19 | 1.06 | 1.77E-01 | 0.28 | 0.67 | 6.51E-01 | 0.09 | 0.23 | 2414862 | 71438 | 2431663 | 94896 | 2423175 | 93611 | 2440152 | 96354 |
| RP_NEG_291_16144a9_711 | RP | NEG | 9.71 | 291.1614 | | | | 2.41E-02 | -0.50 | 0.86 | 3.74E-01 | -0.17 | 0.52 | 1.32E-02 | -0.58 | 0.98 | 9.09E-02 | -0.41 | 0.68 | 4859605 | 185452 | 4774244 | 163554 | 4788875 | 176415 | 4759614 | 149955 |
| RP_NEG_325_18533a9_212 | RP | NEG | 9.21 | 325.1853 | | | | 5.74E-01 | 0.12 | 0.65 | 3.74E-01 | -0.18 | 0.57 | 8.98E-01 | 0.03 | 0.43 | 3.84E-01 | 0.21 | 0.57 | 2803658 | 2687851 | 3110185 | 2588699 | 3341527 | 2749334 | 2878843 | 2423103 |
| RP_POS_378_20523a10_704 | RP | POS | 10.70 | 378.2052 | | | | 1.52E-03 | 0.57 | 1.43 | 3.75E-01 | 0.18 | 1.23 | 3.28E-03 | 0.67 | 1.39 | 9.84E-03 | 0.48 | 1.33 | 3550605 | 571112 | 3963713 | 735173 | 3898023 | 587910 | 4029403 | 858804 |
| RP_POS_272_90616a15_366 | RP | POS | 15.37 | 272.9062 | | | | 5.52E-01 | 0.13 | 0.53 | 3.75E-01 | -0.17 | 0.55 | 8.65E-01 | 0.04 | 0.40 | 3.62E-01 | 0.22 | 0.56 | 5350950 | 374051 | 5395498 | 342018 | 5426039 | 339888 | 5364956 | 344836 |
| RP_POS_980_7533a0_494 | RP | POS | 0.49 | 980.7533 | | | | 8.25E-10 | -0.83 | 1.42 | 3.75E-01 | 0.19 | 1.18 | 4.98E-05 | -0.73 | 1.43 | 1.83E-07 | -0.92 | 1.62 | 1044401 | 123932 | 770559 | 349925 | 739322 | 336992 | 801795 | 363097 |
| RP_POS_392_70184a6_811 | RP | POS | 6.81 | 392.7018 | | | | 7.33E-05 | -0.64 | 1.16 | 3.75E-01 | 0.19 | 0.99 | 3.70E-03 | -0.54 | 1.11 | 2.33E-04 | -0.73 | 1.26 | 1060119 | 59158 | 1000414 | 98243 | 991644 | 101508 | 1009183 | 95076 |
| RP_NEG_374_90674a0_511 | RP | NEG | 0.51 | 374.9067 | | | | 8.63E-06 | 0.54 | 1.25 | 3.75E-01 | -0.20 | 1.21 | 4.08E-03 | 0.44 | 1.22 | 5.20E-04 | 0.64 | 1.40 | 592560 | 139054 | 979667 | 793978 | 1050523 | 858722 | 908811 | 725272 |
| RP_POS_358_25861a6_961 | RP | POS | 6.96 | 358.2586 | | | | 3.62E-01 | 0.14 | 0.66 | 3.75E-01 | 0.20 | 0.98 | 2.96E-01 | 0.24 | 0.57 | 7.74E-01 | 0.04 | 0.65 | 593683 | 374876 | 682354 | 705248 | 619348 | 422032 | 745359 | 904814 |
| RP_POS_614_3399a10_82 | RP | POS | 10.82 | 614.3399 | | | | 2.18E-03 | -0.73 | 1.30 | 3.76E-01 | -0.16 | 1.19 | 1.50E-03 | -0.81 | 1.28 | 8.61E-03 | -0.65 | 1.22 | 612442 | 237384 | 463839 | 177155 | 479619 | 173987 | 448059 | 180633 |
| RP_POS_758_77081a0_498 | RP | POS | 0.50 | 758.7708 | | | | 3.60E-01 | -0.15 | 1.02 | 3.76E-01 | -0.19 | 1.25 | 1.93E-01 | -0.25 | 0.90 | 7.83E-01 | -0.06 | 1.07 | 904416 | 238762 | 853237 | 360930 | 885385 | 385305 | 821089 | 335606 |
| RP_POS_199_01111a10_782 | RP | POS | 10.78 | 199.0111 | | | | 2.93E-01 | 0.21 | 0.59 | 3.76E-01 | 0.18 | 0.77 | 1.96E-01 | 0.30 | 0.77 | 5.82E-01 | 0.12 | 0.35 | 3372214 | 1225740 | 3640897 | 1311215 | 3524169 | 1194861 | 3757626 | 1420618 |
| HILIC_POS_394_31628a1_392 | HILIC | POS | 1.392 | 394.3163 | | | | 4.67E-10 | -0.95 | 1.59 | 3.76E-01 | 0.18 | 0.74 | 6.49E-06 | -0.86 | 1.48 | 1.49E-08 | -1.04 | 1.96 | 3084092 | 547042 | 2102444 | 1046154 | 2009350 | 989986 | 2195537 | 1101569 |
| HILIC_POS_115_51926a4_869 | HILIC | POS | 4.869 | 115.5193 | | | | 7.05E-01 | -0.10 | 0.68 | 3.76E-01 | -0.14 | 0.71 | 5.41E-01 | -0.18 | 0.69 | 9.10E-01 | -0.03 | 0.42 | 411404 | 491250 | 376659 | 269424 | 400630 | 262587 | 352688 | 276651 |
| RP_POS_171_03413a15_643 | RP | POS | 15.64 | 171.0341 | | | | 8.05E-01 | -0.05 | 0.45 | 3.76E-01 | 0.18 | 0.58 | 8.55E-01 | 0.04 | 0.26 | 5.40E-01 | -0.14 | 0.53 | 9373077 | 3953912 | 9171926 | 4110599 | 8806259 | 4235159 | 9537592 | 3991168 |
| HILIC_POS_359_00687a7_33 | HILIC | POS | 7.33 | 359.0069 | | | | 1.87E-01 | -0.26 | 0.48 | 3.77E-01 | 0.18 | 0.61 | 4.19E-01 | -0.17 | 0.32 | 1.33E-01 | -0.35 | 0.72 | 1892525 | 60172 | 1875820 | 65697 | 1869980 | 73358 | 1881660 | 57178 |
| RP_POS_304_21173a4_577 | RP | POS | 4.58 | 304.2117 | | | | 1.92E-01 | 0.23 | 0.60 | 3.77E-01 | -0.19 | 0.86 | 5.11E-01 | 0.14 | 0.45 | 1.09E-01 | 0.33 | 0.85 | 4151538 | 1875113 | 4697311 | 2481721 | 4917775 | 2383595 | 4476847 | 2581251 |
| RP_POS_473_25107a10_168 | RP | POS | 10.17 | 473.2511 | | | | 2.22E-01 | 0.21 | 0.58 | 3.77E-01 | -0.19 | 0.56 | 5.24E-01 | 0.12 | 0.36 | 1.61E-01 | 0.31 | 0.60 | 3884701 | 2837849 | 4660685 | 3817629 | 4999797 | 4303680 | 4321573 | 3269506 |
| HILIC_POS_203_05261a5_066 | HILIC | POS | 5.066 | 203.0526 | | | | 9.92E-03 | 0.39 | 1.01 | 3.77E-01 | 0.19 | 0.65 | 2.02E-02 | 0.49 | 0.98 | 6.92E-02 | 0.30 | 0.65 | 37832831 | 19741937 | 51194848 | 36951906 | 47911842 | 29811059 | 54477853 | 42989039 |
| HILIC_POS_155_15425a0_604 | HILIC | POS | 0.604 | 155.1543 | | | | 7.47E-01 | 0.06 | 0.65 | 3.77E-01 | -0.19 | 0.66 | 8.35E-01 | -0.04 | 0.36 | 5.01E-01 | 0.15 | 0.66 | 2703331 | 1084961 | 2781793 | 1480834 | 2913351 | 1730438 | 2650236 | 1183535 |
| HILIC_POS_164_12817a2_534 | HILIC | POS | 2.534 | 164.1282 | | | | 5.44E-02 | 0.23 | 0.90 | 3.78E-01 | 0.20 | 1.03 | 1.25E-01 | 0.34 | 0.89 | 1.61E-01 | 0.13 | 0.70 | 629493 | 413072 | 1057497 | 2078569 | 872605 | 1096162 | 1242389 | 2730885 |
| RP_POS_1043_60645a10_324 | RP | POS | 10.32 | 1043.6065 | | | | 3.27E-08 | 0.96 | 1.63 | 3.78E-01 | -0.17 | 0.75 | 1.76E-05 | 0.88 | 1.52 | 6.56E-08 | 1.05 | 1.77 | 658682 | 229096 | 978637 | 323026 | 1007303 | 296554 | 949972 | 348138 |
| RP_NEG_240_89978a15_555 | RP | NEG | 15.56 | 240.8998 | | | | 7.61E-01 | -0.06 | 0.49 | 3.78E-01 | -0.18 | 0.78 | 4.77E-01 | -0.15 | 0.49 | 9.02E-01 | 0.03 | 0.60 | 586441 | 30363 | 584534 | 31811 | 587357 | 35802 | 581712 | 27323 |
| RP_POS_393_23022a11_376 | RP | POS | 11.38 | 393.2302 | | | | 3.51E-03 | -0.53 | 1.06 | 3.78E-01 | 0.18 | 1.12 | 2.53E-02 | -0.44 | 0.96 | 4.44E-03 | -0.63 | 1.07 | 1086721 | 160861 | 980990 | 202414 | 963038 | 220694 | 998942 | 182798 |
| RP_NEG_285_70731a15_59 | RP | NEG | 15.59 | 285.7073 | | | | 1.36E-02 | -0.50 | 0.86 | 3.78E-01 | -0.18 | 0.69 | 7.81E-03 | -0.58 | 1.01 | 7.28E-02 | -0.41 | 0.66 | 2876459 | 104040 | 2821867 | 109408 | 2831568 | 116040 | 2812166 | 102603 |
| RP_POS_394_35257a10_656 | RP | POS | 10.66 | 394.3526 | | | | 2.39E-01 | 0.23 | 0.75 | 3.78E-01 | -0.18 | 0.84 | 5.11E-01 | 0.15 | 0.38 | 1.51E-01 | 0.32 | 0.68 | 4635830 | 783611 | 4827987 | 827432 | 4901337 | 845394 | 4754638 | 810919 |
| RP_NEG_592_2666a10_301 | RP | NEG | 10.30 | 592.2666 | | | | 7.90E-02 | 0.32 | 1.10 | 3.78E-01 | 0.18 | 0.68 | 5.05E-02 | 0.42 | 1.01 | 2.70E-01 | 0.23 | 0.81 | 684152 | 303286 | 800742 | 372765 | 767706 | 372795 | 833777 | 373551 |
| RP_POS_767_36609a6_81 | RP | POS | 6.81 | 767.3661 | | | | 5.03E-01 | -0.12 | 0.72 | 3.78E-01 | 0.19 | 0.64 | 9.07E-01 | -0.03 | 0.48 | 2.65E-01 | -0.21 | 0.64 | 829605 | 46160 | 822764 | 60322 | 817418 | 50460 | 828110 | 68900 |
| RP_POS_564_84271a0_496 | RP | POS | 0.50 | 564.8427 | | | | 3.85E-07 | -0.64 | 1.33 | 3.78E-01 | 0.19 | 1.22 | 1.24E-03 | -0.55 | 1.34 | 3.24E-05 | -0.74 | 1.47 | 568662 | 60894 | 428113 | 239248 | 406919 | 242182 | 449307 | 236809 |
| HILIC_POS_189_07329a4_839 | HILIC | POS | 4.839 | 189.0733 | | | | 2.29E-02 | -0.49 | 0.72 | 3.79E-01 | 0.17 | 0.68 | 1.06E-01 | -0.40 | 0.66 | 8.87E-03 | -0.57 | 1.05 | 2032131 | 451692 | 1820515 | 417103 | 1783553 | 312516 | 1857476 | 501045 |
| RP_POS_613_33984a5_488 | RP | POS | 5.49 | 613.3398 | | | | 1.72E-06 | 0.57 | 1.30 | 3.79E-01 | -0.20 | 1.23 | 1.80E-03 | 0.47 | 1.36 | 2.89E-04 | 0.67 | 1.40 | 133953 | 210521 | 1217521 | 2104804 | 1403892 | 2289617 | 1031151 | 1907244 |
| HILIC_POS_59_06127a0_798 | HILIC | POS | 0.798 | 59.0613 | | | | 3.49E-03 | 0.56 | 1.11 | 3.79E-01 | 0.18 | 0.56 | 3.57E-03 | 0.65 | 1.10 | 2.32E-02 | 0.47 | 1.26 | 2530050 | 1237529 | 3322601 | 1411927 | 3197636 | 1319346 | 3447566 | 1501738 |
| RP_POS_374_22043a11_163 | RP | POS | 11.16 | 374.2204 | | | | 7.88E-01 | 0.04 | 0.73 | 3.79E-01 | -0.19 | 0.90 | 7.71E-01 | -0.06 | 0.58 | 4.68E-01 | 0.14 | 0.71 | 1119852 | 67709 | 1124426 | 120111 | 1135049 | 120643 | 1113803 | 119844 |
| RP_POS_163_11165a15_1 | RP | POS | 15.10 | 163.1117 | | | | 1.97E-01 | -0.24 | 0.49 | 3.79E-01 | 0.18 | 0.57 | 4.58E-01 | -0.15 | 0.41 | 1.37E-01 | -0.33 | 0.65 | 2994978 | 654584 | 2813735 | 770489 | 2745607 | 842245 | 2881863 | 693233 |
| RP_POS_270_90982a15_367 | RP | POS | 15.37 | 270.9098 | | | | 3.91E-01 | -0.18 | 0.49 | 3.80E-01 | -0.17 | 0.57 | 2.46E-01 | -0.27 | 0.54 | 6.87E-01 | -0.09 | 0.25 | 9623866 | 262725 | 9578368 | 246197 | 9600111 | 253829 | 9556625 | 238891 |
| RP_POS_608_38464a5_488 | RP | POS | 5.49 | 608.3846 | | | | 1.61E-06 | 0.57 | 1.30 | 3.80E-01 | -0.20 | 1.22 | 1.64E-03 | 0.48 | 1.36 | 2.92E-04 | 0.67 | 1.39 | 240985 | 348221 | 2015189 | 3433736 | 2318336 | 3747376 | 1712042 | 3096987 |
| RP_NEG_658_39166a10_977 | RP | NEG | 10.98 | 658.3917 | | | | 1.34E-02 | 0.44 | 1.37 | 3.80E-01 | -0.18 | 1.26 | 8.93E-02 | 0.35 | 1.10 | 1.02E-02 | 0.53 | 1.35 | 709373 | 514260 | 997755 | 680872 | 1057826 | 679630 | 937684 | 683648 |
| RP_POS_198_99181a2_815 | RP | POS | 2.82 | 198.9918 | | | | 3.67E-02 | -0.41 | 0.90 | 3.81E-01 | 0.18 | 0.69 | 1.38E-01 | -0.32 | 0.63 | 2.60E-02 | -0.50 | 0.94 | 1383362 | 107136 | 1335649 | 117684 | 1325271 | 121398 | 1346027 | 114124 |
| RP_NEG_614_34729a10_544 | RP | NEG | 10.54 | 614.3473 | | | | 1.82E-06 | 0.73 | 1.32 | 3.81E-01 | -0.18 | 0.62 | 1.82E-03 | 0.63 | 1.16 | 1.41E-06 | 0.82 | 1.59 | 1069365 | 359871 | 1541490 | 680344 | 1601510 | 564217 | 1481469 | 780698 |
| RP_POS_285_2428a9_579 | RP | POS | 9.58 | 285.2428 | | | | 8.47E-01 | 0.04 | 0.61 | 3.81E-01 | -0.18 | 0.81 | 8.26E-01 | -0.05 | 0.32 | 5.86E-01 | 0.13 | 0.95 | 9174747 | 1302166 | 9225910 | 1294500 | 9340056 | 1384432 | 9111764 | 1201007 |
| RP_POS_159_09175a2_49 | RP | POS | 2.49 | 159.0918 | | | | 1.00E-03 | -0.57 | 1.16 | 3.81E-01 | -0.18 | 1.09 | 2.69E-03 | -0.66 | 1.22 | 8.38E-03 | -0.48 | 1.09 | 4834970 | 829933 | 4192917 | 1164360 | 4295625 | 950537 | 4090208 | 1346881 |
| RP_POS_875_52356a10_849 | RP | POS | 10.85 | 875.5236 | | | | 6.38E-03 | 0.33 | 1.00 | 3.81E-01 | -0.20 | 1.00 | 9.53E-02 | 0.23 | 0.99 | 2.56E-02 | 0.43 | 1.17 | 1095491 | 221937 | 1422212 | 1109281 | 1520023 | 1277715 | 1324401 | 913259 |
| RP_POS_129_03055a15_433 | RP | POS | 15.43 | 129.0306 | | | | 4.32E-02 | -0.25 | 0.81 | 3.81E-01 | 0.20 | 0.57 | 2.66E-01 | -0.15 | 0.70 | 7.75E-02 | -0.35 | 0.65 | 10747379 | 388698 | 10377168 | 1676963 | 10229363 | 1977161 | 10524973 | 1314552 |
| RP_POS_308_2948a10_438 | RP | POS | 10.44 | 308.2948 | | | | 3.84E-01 | -0.26 | 0.94 | 3.81E-01 | -0.12 | 0.67 | 2.93E-01 | -0.32 | 0.75 | 5.14E-01 | -0.20 | 0.60 | 695596 | 1662815 | 429317 | 684083 | 489581 | 763789 | 369053 | 595571 |
| HILIC_POS_151_09654a0_631 | HILIC | POS | 0.631 | 151.0965 | | | | 2.77E-01 | 0.15 | 1.04 | 3.81E-01 | -0.20 | 0.78 | 7.17E-01 | 0.05 | 1.12 | 2.25E-01 | 0.25 | 1.05 | 2893203 | 413883 | 3026780 | 978718 | 3113004 | 1158119 | 2940556 | 760863 |
| RP_POS_242_92581a0_497 | RP | POS | 0.50 | 242.9258 | | | | 1.44E-08 | -0.68 | 1.35 | 3.81E-01 | 0.19 | 1.20 | 1.97E-04 | -0.59 | 1.38 | 2.15E-05 | -0.78 | 1.50 | 107015456 | 1892214 | 90070285 | 27288855 | 87668177 | 29051033 | 92472392 | 25471995 |
| HILIC_POS_342_26398a0_675 | HILIC | POS | 0.675 | 342.2640 | | | | 1.15E-03 | 0.49 | 0.88 | 3.81E-01 | 0.19 | 0.89 | 1.21E-03 | 0.58 | 1.38 | 4.09E-02 | 0.39 | 0.66 | 9775791 | 5362729 | 14471909 | 10355478 | 13560414 | 10979477 | 15383404 | 9716783 |

**Supplementary table 1: Metabolomics results** (in asceding order according to p-value from progressive ALD vs non-progressive ALD comparison, with molecular features with ID_level of 1-2 first)

| | | | | | | | | all_ALD_vs_control | | | Prog_ALD_vs_non-prog_ALD | | | Prog_ALD_vs_control_ALD | | | Non-prog_ALD_vs_control | | | Healthy_control | | all_ALD | | Non-prog_ALD | | Progressive_ALD | |
|---|---|---|---|---|---|---|---|---|---|---|---|---|---|---|---|---|---|---|---|---|---|---|---|---|---|---|---|
| | | | | | | | | t-test | Cohen's | PLS-DA | t-test | Cohen's | PLS-DA | t-test | Cohen's | PLS-DA | t-test | Cohen's | PLS-DA | | | | | | | | |
| Feature_ID | RP_HILIC | POS_NEG | Average.Rt | Average.Mz | Metabolite.name | curated_MSMS | ID_level | p | d | VIP | p | d | VIP | p | d | VIP | p | d | VIP | Mean | SD | Mean | SD | Mean | SD | Mean | SD |
| RP_POS_499_82715a4_636 | RP | POS | 4.64 | 499.8272 | | | | 1.41E-01 | 0.26 | 0.81 | 3.81E-01 | -0.19 | 0.56 | 4.02E-01 | 0.16 | 0.38 | 1.02E-01 | 0.35 | 1.05 | 2041841 | 2195504 | 2774153 | 3032552 | 3041084 | 3278705 | 2507222 | 2772202 |
| HILIC_NEG_219_94669a0_601 | HILIC | NEG | 0.601 | 219.9467 | | | | 9.41E-02 | 0.20 | 0.98 | 3.82E-01 | -0.20 | 0.60 | 4.89E-01 | 0.09 | 0.55 | 1.17E-01 | 0.30 | 0.47 | 40349 | 43907 | 126086 | 501477 | 170237 | 573964 | 81935 | 417944 |
| RP_POS_592_33734a10_543 | RP | POS | 10.54 | 592.3373 | | | | 1.88E-05 | 0.66 | 1.27 | 3.82E-01 | -0.19 | 0.65 | 5.54E-03 | 0.57 | 1.08 | 1.05E-05 | 0.76 | 1.50 | 449895 | 183222 | 658587 | 331632 | 687781 | 274574 | 629393 | 380885 |
| RP_POS_352_24582a11_161 | RP | POS | 11.16 | 352.2458 | | | | 4.21E-01 | -0.14 | 0.71 | 3.82E-01 | 0.19 | 0.79 | 8.28E-01 | -0.04 | 0.60 | 2.64E-01 | -0.23 | 0.70 | 3130669 | 329156 | 3069834 | 476256 | 3027936 | 497308 | 3111732 | 455381 |
| HILIC_POS_487_29883a0_649 | HILIC | POS | 0.649 | 487.2988 | | | | 2.07E-06 | 0.57 | 1.27 | 3.82E-01 | -0.20 | 0.90 | 2.11E-03 | 0.47 | 1.44 | 3.23E-04 | 0.66 | 1.47 | 87462 | 171106 | 1405922 | 2600957 | 1634707 | 2822458 | 1177137 | 2365360 |
| RP_POS_239_14896a3_195 | RP | POS | 3.20 | 239.1490 | | | | 2.61E-02 | 0.34 | 1.11 | 3.82E-01 | -0.19 | 1.10 | 1.51E-01 | 0.25 | 1.13 | 3.28E-02 | 0.44 | 1.12 | 584605 | 634946 | 947809 | 1150871 | 1049047 | 1285202 | 846571 | 1001688 |
| HILIC_POS_516_3017a0_622 | HILIC | POS | 0.622 | 516.3017 | | | | 4.29E-06 | 0.56 | 1.23 | 3.82E-01 | -0.20 | 0.91 | 2.25E-03 | 0.46 | 1.41 | 4.92E-04 | 0.65 | 1.40 | 93364 | 289640 | 1060144 | 1932928 | 1230081 | 2130413 | 890207 | 1717868 |
| HILIC_POS_130_08641a7_097 | HILIC | POS | 7.097 | 130.0864 | | | | 1.57E-02 | 0.32 | 0.99 | 3.82E-01 | -0.20 | 0.85 | 1.83E-01 | 0.22 | 1.04 | 2.33E-02 | 0.42 | 1.05 | 4470529 | 1532178 | 5766457 | 4548236 | 6166110 | 4787947 | 5366803 | 4306354 |
| RP_POS_371_31427a11_436 | RP | POS | 11.44 | 371.3143 | | | | 3.01E-01 | 0.19 | 0.76 | 3.83E-01 | -0.18 | 0.67 | 6.37E-01 | 0.09 | 0.27 | 2.04E-01 | 0.28 | 0.97 | 2212226 | 366438 | 2296048 | 476200 | 2337866 | 520762 | 2254230 | 428251 |
| RP_POS_257_22641a10_168 | RP | POS | 10.17 | 257.2264 | | | | 2.01E-01 | 0.22 | 0.58 | 3.83E-01 | -0.19 | 0.55 | 4.91E-01 | 0.13 | 0.37 | 1.57E-01 | 0.31 | 0.60 | 2580999 | 1942076 | 3149323 | 2767805 | 3392374 | 3199151 | 2906272 | 2263341 |
| RP_POS_221_9818a1_461 | RP | POS | 1.46 | 221.9818 | | | | 4.30E-12 | 0.93 | 1.51 | 3.83E-01 | 0.18 | 0.55 | 6.84E-08 | 1.02 | 1.75 | 5.25E-07 | 0.84 | 1.51 | 1370598 | 1232794 | 4509008 | 3480095 | 4203592 | 3268464 | 4814424 | 3687283 |
| RP_POS_217_10535a2_162 | RP | POS | 2.16 | 217.1054 | | | | 4.40E-03 | 0.39 | 1.03 | 3.83E-01 | -0.19 | 0.99 | 7.39E-02 | 0.29 | 1.07 | 1.18E-02 | 0.48 | 1.10 | 6769392 | 734236 | 7450259 | 1956204 | 7621908 | 2138661 | 7278609 | 1759990 |
| RP_NEG_209_06641a0_618 | RP | NEG | 0.62 | 209.0664 | | | | 8.24E-02 | 0.34 | 0.57 | 3.83E-01 | -0.18 | 0.67 | 2.51E-01 | 0.25 | 0.51 | 5.56E-02 | 0.43 | 0.74 | 1959333 | 638080 | 2195481 | 716237 | 2258311 | 738238 | 2132652 | 695276 |
| RP_POS_441_29492a11_162 | RP | POS | 11.16 | 441.2949 | | | | 8.76E-01 | -0.03 | 0.69 | 3.83E-01 | 0.19 | 0.83 | 7.57E-01 | 0.06 | 0.65 | 5.59E-01 | -0.12 | 0.76 | 12264324 | 1254280 | 12221011 | 1638110 | 12077350 | 1618885 | 12364673 | 1660923 |
| RP_NEG_344_98062a9_044 | RP | NEG | 9.04 | 344.9806 | | | | 8.52E-01 | 0.03 | 0.58 | 3.83E-01 | -0.19 | 0.56 | 7.20E-01 | -0.06 | 0.80 | 5.67E-01 | 0.13 | 0.64 | 3330433 | 590924 | 3356061 | 887701 | 3433910 | 1036639 | 3278211 | 710719 |
| RP_NEG_144_99081a15_809 | RP | NEG | 15.81 | 144.9908 | | | | 5.76E-01 | 0.12 | 0.51 | 3.84E-01 | 0.17 | 0.91 | 3.81E-01 | 0.20 | 0.79 | 8.94E-01 | 0.03 | 0.28 | 2469556 | 1418739 | 2629762 | 1346082 | 2511794 | 1341123 | 2747731 | 1354194 |
| HILIC_POS_154_15897a5_539 | HILIC | POS | 5.539 | 154.1590 | | | | 6.45E-01 | 0.09 | 0.24 | 3.84E-01 | -0.18 | 0.57 | 9.86E-01 | 0.00 | 0.22 | 4.45E-01 | 0.18 | 0.32 | 9757287 | 3912442 | 10128554 | 4061803 | 10484453 | 4569626 | 9772655 | 3491877 |
| HILIC_NEG_387_67157a1_364 | HILIC | NEG | 1.364 | 387.6716 | | | | 2.16E-05 | 0.69 | 1.09 | 3.84E-01 | -0.18 | 0.61 | 3.55E-03 | 0.60 | 0.97 | 1.53E-05 | 0.78 | 1.48 | 142405 | 59670 | 206481 | 95778 | 214870 | 82413 | 198092 | 107697 |
| RP_POS_654_29541a10_501 | RP | POS | 10.50 | 654.2954 | | | | 2.64E-01 | 0.23 | 0.61 | 3.84E-01 | -0.17 | 0.57 | 5.21E-01 | 0.15 | 0.47 | 1.73E-01 | 0.32 | 0.76 | 360753 | 264334 | 421035 | 257601 | 443590 | 267472 | 398481 | 247975 |
| RP_NEG_583_25793a11_808 | RP | NEG | 11.81 | 583.2579 | | | | 3.52E-02 | -0.46 | 1.24 | 3.84E-01 | 0.17 | 1.08 | 1.44E-01 | -0.37 | 0.83 | 1.29E-02 | -0.54 | 1.26 | 2288042 | 1034923 | 1842659 | 937907 | 1760492 | 639204 | 1924827 | 1164016 |
| RP_POS_121_25214a11_666 | RP | POS | 11.67 | 121.2521 | | | | 9.36E-01 | 0.02 | 0.66 | 3.85E-01 | -0.18 | 0.51 | 7.44E-01 | -0.07 | 0.41 | 6.44E-01 | 0.10 | 0.41 | 310202 | 199039 | 313515 | 208633 | 331756 | 214041 | 295273 | 203596 |
| HILIC_POS_132_10208a5_139 | HILIC | POS | 5.139 | 132.1021 | | | | 2.21E-01 | 0.16 | 0.30 | 3.85E-01 | 0.20 | 0.66 | 2.04E-01 | 0.26 | 0.39 | 6.69E-01 | 0.06 | 0.42 | 1572690 | 1197522 | 2130851 | 4011601 | 1779879 | 3069119 | 2481822 | 4779480 |
| HILIC_NEG_229_9762a0_935 | HILIC | NEG | 0.935 | 229.9762 | | | | 7.86E-02 | 0.26 | 1.09 | 3.85E-01 | -0.19 | 0.55 | 3.92E-01 | 0.17 | 0.28 | 4.46E-02 | 0.36 | 0.95 | 139660 | 64306 | 169976 | 127353 | 181102 | 118956 | 158849 | 135518 |
| RP_NEG_343_995a0_727 | RP | NEG | 0.73 | 343.9950 | | | | 4.23E-03 | -0.51 | 1.04 | 3.85E-01 | 0.18 | 0.99 | 3.91E-02 | -0.42 | 0.96 | 3.99E-03 | -0.60 | 1.10 | 64905340 | 30457928 | 44985010 | 40409038 | 41456549 | 40919664 | 48513472 | 39990972 |
| RP_POS_438_26651a11_668 | RP | POS | 11.67 | 438.2665 | | | | 7.54E-01 | -0.06 | 0.74 | 3.85E-01 | -0.18 | 0.53 | 4.94E-01 | -0.15 | 0.47 | 9.11E-01 | 0.03 | 0.56 | 872603 | 501739 | 840243 | 521607 | 885775 | 546645 | 794711 | 496636 |
| RP_POS_373_25851a10_242 | RP | POS | 10.24 | 373.2585 | | | | 3.47E-05 | 0.49 | 0.97 | 3.86E-01 | -0.20 | 0.89 | 1.17E-02 | 0.39 | 0.95 | 1.10E-03 | 0.59 | 1.13 | 31261 | 41821 | 637087 | 1395010 | 758816 | 1482047 | 515357 | 1305802 |
| RP_POS_848_55518a13_871 | RP | POS | 13.87 | 848.5552 | | | | 7.31E-01 | -0.07 | 0.43 | 3.86E-01 | 0.17 | 0.65 | 9.49E-01 | 0.02 | 0.46 | 4.62E-01 | -0.16 | 0.36 | 4601133 | 1583809 | 4490415 | 1548234 | 4355267 | 1261207 | 4625563 | 1793058 |
| RP_NEG_864_50781a10_374 | RP | NEG | 10.37 | 864.5078 | | | | 4.32E-01 | -0.20 | 0.49 | 3.86E-01 | -0.15 | 0.57 | 3.08E-01 | -0.27 | 0.44 | 6.50E-01 | -0.12 | 0.44 | 573983 | 541223 | 492515 | 370538 | 524833 | 349104 | 460198 | 391656 |
| RP_POS_373_21109a10_905 | RP | POS | 10.91 | 373.2111 | | | | 3.89E-03 | 0.42 | 1.52 | 3.86E-01 | 0.19 | 0.91 | 9.83E-03 | 0.52 | 1.29 | 4.79E-02 | 0.33 | 1.31 | 1386144 | 515825 | 1798108 | 1057571 | 1705877 | 922140 | 1890339 | 1179846 |
| RP_POS_544_33722a10_618 | RP | POS | 10.62 | 544.3372 | | | | 7.37E-03 | 0.60 | 1.37 | 3.86E-01 | -0.16 | 1.00 | 3.90E-02 | 0.52 | 1.07 | 3.28E-03 | 0.68 | 1.44 | 123112936 | 30617396 | 139975438 | 26296057 | 142268080 | 21437014 | 137682795 | 30443465 |
| RP_POS_344_3147a10_174 | RP | POS | 10.17 | 344.3147 | | | | 7.41E-01 | -0.06 | 0.83 | 3.86E-01 | 0.18 | 0.90 | 8.88E-01 | 0.03 | 0.66 | 5.04E-01 | -0.15 | 0.77 | 791305 | 146214 | 780974 | 172229 | 765961 | 194301 | 795986 | 147370 |
| RP_POS_569_31427a5_307 | RP | POS | 5.31 | 569.3143 | | | | 4.93E-05 | 0.52 | 1.28 | 3.87E-01 | -0.19 | 1.21 | 7.79E-03 | 0.42 | 1.31 | 1.04E-03 | 0.61 | 1.37 | 308473 | 728500 | 1551338 | 2663031 | 1783282 | 2880071 | 1319393 | 2433844 |
| RP_POS_232_88356a0_245 | RP | POS | 0.25 | 232.8836 | | | | 3.23E-01 | -0.20 | 0.61 | 3.87E-01 | 0.17 | 0.52 | 6.03E-01 | -0.11 | 0.31 | 2.18E-01 | -0.29 | 0.49 | 1286054 | 198416 | 1245794 | 199614 | 1228413 | 213963 | 1263174 | 184683 |
| RP_POS_697_65625a11_668 | RP | POS | 11.67 | 697.6563 | | | | 9.15E-01 | 0.02 | 0.65 | 3.87E-01 | -0.18 | 0.51 | 7.67E-01 | -0.07 | 0.38 | 6.38E-01 | 0.11 | 0.45 | 8445427 | 7699970 | 8613782 | 7785204 | 9291529 | 8227857 | 7936035 | 7336258 |
| HILIC_POS_342_21198a1_952 | HILIC | POS | 1.952 | 342.2120 | | | | 5.77E-01 | 0.12 | 1.07 | 3.88E-01 | -0.17 | 0.80 | 8.81E-01 | 0.03 | 0.95 | 4.02E-01 | 0.20 | 1.09 | 409437 | 1149912 | 538424 | 1064207 | 630815 | 1176542 | 446033 | 941549 |
| HILIC_POS_314_14551a2_457 | HILIC | POS | 2.457 | 314.1455 | | | | 2.70E-02 | 0.26 | 0.79 | 3.88E-01 | 0.20 | 0.72 | 6.76E-02 | 0.36 | 0.58 | 1.96E-01 | 0.17 | 0.98 | 663980 | 100146 | 814540 | 648063 | 758250 | 494070 | 870831 | 773286 |
| RP_NEG_180_98903a6_159 | RP | NEG | 6.16 | 180.9890 | | | | 4.27E-01 | -0.17 | 0.46 | 3.88E-01 | 0.17 | 0.96 | 7.10E-01 | -0.09 | 0.38 | 2.75E-01 | -0.26 | 0.47 | 2568087 | 1569836 | 2319781 | 1377394 | 2200273 | 1312468 | 2439289 | 1442715 |
| RP_POS_161_98148a15_55 | RP | POS | 15.55 | 161.9815 | | | | 9.70E-01 | 0.01 | 0.68 | 3.89E-01 | -0.18 | 0.51 | 7.11E-01 | -0.08 | 0.27 | 6.79E-01 | 0.10 | 0.55 | 6099346 | 176234 | 6100717 | 185312 | 6116785 | 199152 | 6084649 | 170861 |
| RP_POS_591_35822a5_488 | RP | POS | 5.49 | 591.3582 | | | | 1.85E-06 | 0.57 | 1.30 | 3.89E-01 | -0.19 | 1.22 | 1.78E-03 | 0.48 | 1.36 | 3.11E-04 | 0.67 | 1.39 | 82616 | 148745 | 816352 | 1428572 | 940197 | 1554667 | 692507 | 1294217 |
| HILIC_POS_694_42188a1_064 | HILIC | POS | 1.064 | 694.4219 | | | | 5.07E-06 | 0.55 | 1.25 | 3.89E-01 | -0.19 | 0.88 | 2.71E-03 | 0.45 | 1.41 | 5.52E-04 | 0.64 | 1.43 | 141933 | 414098 | 1879634 | 3541611 | 2186666 | 3886876 | 1572602 | 3168880 |
| RP_POS_809_55432a10_857 | RP | POS | 10.86 | 809.5543 | | | | 2.60E-02 | -0.45 | 0.96 | 3.89E-01 | -0.17 | 0.71 | 1.53E-02 | -0.53 | 0.92 | 1.12E-01 | -0.36 | 0.64 | 1240480 | 409988 | 1047930 | 427587 | 1084989 | 451561 | 1010870 | 403340 |
| HILIC_POS_328_24811a0_735 | HILIC | POS | 0.735 | 328.2481 | | | | 1.80E-01 | -0.26 | 0.55 | 3.89E-01 | -0.18 | 0.69 | 1.26E-01 | -0.35 | 0.55 | 4.20E-01 | -0.17 | 0.39 | 5011964 | 2250409 | 4370724 | 2554391 | 4592116 | 2339891 | 4149331 | 2758236 |
| RP_POS_591_45923a11_254 | RP | POS | 11.25 | 591.4592 | | | | 1.76E-01 | 0.20 | 0.40 | 3.89E-01 | -0.19 | 0.76 | 4.66E-01 | 0.11 | 0.48 | 1.75E-01 | 0.30 | 0.50 | 468318 | 507166 | 650918 | 995435 | 737272 | 1234759 | 564563 | 679821 |
| RP_NEG_213_91519a14_266 | RP | NEG | 14.27 | 213.9152 | | | | 5.38E-01 | 0.12 | 0.37 | 3.89E-01 | 0.18 | 0.53 | 3.71E-01 | 0.21 | 0.40 | 8.79E-01 | 0.03 | 0.26 | 932672 | 203779 | 958902 | 223330 | 939550 | 191544 | 978255 | 251629 |
| RP_POS_185_97462a15_577 | RP | POS | 15.58 | 185.9746 | | | | 7.51E-01 | 0.06 | 0.30 | 3.89E-01 | 0.18 | 0.66 | 4.92E-01 | 0.15 | 0.39 | 8.57E-01 | -0.04 | 0.48 | 6442882 | 236397 | 6459587 | 315387 | 6432278 | 289483 | 6486895 | 340067 |
| HILIC_POS_182_0578a3_508 | HILIC | POS | 3.508 | 182.0578 | | | | 7.13E-01 | 0.06 | 0.53 | 3.90E-01 | 0.19 | 0.65 | 4.55E-01 | 0.16 | 0.56 | 8.80E-01 | -0.03 | 0.49 | 3316569 | 3714409 | 3628531 | 5297071 | 3170172 | 5007140 | 4086890 | 5584831 |
| HILIC_POS_217_15443a5_486 | HILIC | POS | 5.486 | 217.1544 | | | | 2.56E-01 | 0.21 | 0.54 | 3.90E-01 | 0.18 | 0.64 | 1.95E-01 | 0.30 | 0.47 | 5.43E-01 | 0.11 | 0.32 | 442754 | 397742 | 541976 | 506802 | 498099 | 404101 | 585852 | 593053 |
| RP_POS_272_79459a0_522 | RP | POS | 0.52 | 272.7946 | | | | 2.39E-06 | -0.67 | 1.24 | 3.90E-01 | 0.19 | 1.04 | 1.10E-03 | -0.58 | 1.18 | 4.07E-05 | -0.76 | 1.39 | 952906 | 125315 | 773967 | 284611 | 749341 | 289611 | 798593 | 280263 |
| RP_POS_721_50726a10_916 | RP | POS | 10.92 | 721.5073 | | | | 9.13E-01 | -0.02 | 0.47 | 3.90E-01 | 0.17 | 0.56 | 7.88E-01 | 0.06 | 0.34 | 6.14E-01 | -0.11 | 0.67 | 4515185 | 744058 | 4498646 | 743165 | 4434345 | 636846 | 4562948 | 837764 |
| HILIC_POS_313_15457a1_741 | HILIC | POS | 1.741 | 313.1546 | | | | 3.94E-05 | 0.49 | 1.27 | 3.90E-01 | 0.19 | 0.80 | 2.42E-03 | 0.59 | 1.40 | 4.69E-03 | 0.39 | 1.26 | 317630 | 1517230 | 6850919 | 14980073 | 5555309 | 12382939 | 8146528 | 17222715 |
| RP_POS_794_56494a13_421 | RP | POS | 13.42 | 794.5649 | | | | 4.27E-06 | -0.97 | 1.62 | 3.90E-01 | 0.16 | 0.78 | 5.05E-05 | -0.90 | 1.62 | 5.79E-06 | -1.05 | 1.55 | 4917386 | 1450683 | 3413720 | 1392928 | 3293361 | 1463715 | 3534079 | 1322147 |
| HILIC_POS_371_24088a4_125 | HILIC | POS | 4.125 | 371.2409 | | | | 5.13E-02 | -0.35 | 1.03 | 3.90E-01 | -0.18 | 1.21 | 3.97E-02 | -0.44 | 0.82 | 1.92E-01 | -0.26 | 0.74 | 8676778 | 1308859 | 8111091 | 1662743 | 8254730 | 1571336 | 7967452 | 1753468 |
| RP_POS_395_24033a10_242 | RP | POS | 10.24 | 395.2403 | | | | 4.20E-05 | 0.48 | 0.94 | 3.91E-01 | -0.19 | 0.87 | 1.18E-02 | 0.39 | 0.93 | 1.33E-03 | 0.58 | 1.11 | 126714 | 155119 | 2006401 | 4376863 | 2384429 | 4687029 | 1628373 | 4055236 |
| RP_POS_428_31177a11_669 | RP | POS | 11.67 | 428.3118 | | | | 9.67E-01 | -0.01 | 0.75 | 3.91E-01 | -0.17 | 0.52 | 6.75E-01 | -0.10 | 0.45 | 7.31E-01 | 0.08 | 0.48 | 1294721 | 826488 | 1287806 | 833062 | 1359744 | 841716 | 1215869 | 826499 |
| HILIC_POS_141_13866a0_558 | HILIC | POS | 0.558 | 141.1387 | | | | 1.74E-02 | -0.51 | 1.01 | 3.91E-01 | 0.17 | 0.87 | 7.45E-02 | -0.43 | 0.76 | 9.52E-03 | -0.59 | 1.20 | 2605241 | 1134441 | 2047868 | 1050553 | 1957170 | 953036 | 2138567 | 1142269 |
| RP_POS_347_17102a6_444 | RP | POS | 6.44 | 347.1710 | | | | 7.42E-01 | 0.05 | 0.26 | 3.91E-01 | 0.19 | 0.53 | 5.08E-01 | 0.15 | 0.40 | 7.90E-01 | -0.04 | 0.24 | 969401 | 90613 | 976740 | 154374 | 963402 | 111367 | 990079 | 188104 |
| RP_POS_371_22751a4_35 | RP | POS | 4.35 | 371.2275 | | | | 8.41E-01 | -0.06 | 1.16 | 3.91E-01 | -0.12 | 1.14 | 6.94E-01 | -0.12 | 0.88 | 9.98E-01 | 0.00 | 0.95 | 930910 | 3130440 | 815662 | 1318949 | 929513 | 1442956 | 701811 | 1185825 |
| RP_POS_318_16489a5_488 | RP | POS | 5.49 | 318.1649 | | | | 1.44E-06 | 0.58 | 1.31 | 3.91E-01 | -0.19 | 1.23 | 1.76E-03 | 0.48 | 1.37 | 2.48E-04 | 0.67 | 1.41 | 62407 | 110726 | 642034 | 1116684 | 738414 | 1203646 | 545655 | 1025522 |
| HILIC_NEG_141_92368a2_899 | HILIC | NEG | 2.899 | 141.9237 | | | | 3.10E-01 | 0.19 | 1.00 | 3.91E-01 | 0.18 | 0.90 | 2.10E-01 | 0.28 | 0.49 | 6.23E-01 | 0.10 | 0.21 | 2139793 | 447490 | 2237131 | 529142 | 2191473 | 484344 | 2282789 | 571710 |
| RP_POS_837_55939a13_871 | RP | POS | 13.87 | 837.5594 | | | | 3.25E-02 | -0.51 | 0.99 | 3.91E-01 | 0.15 | 0.94 | 9.83E-02 | -0.44 | 0.71 | 1.67E-02 | -0.59 | 1.02 | 1089498 | 833585 | 736411 | 615458 | 683302 | 500790 | 789519 | 713274 |
| HILIC_POS_501_27377a1_309 | HILIC | POS | 1.309 | 501.2738 | | | | 6.61E-01 | 0.10 | 0.19 | 3.91E-01 | -0.17 | 0.58 | 9.57E-01 | 0.01 | 0.02 | 4.60E-01 | 0.18 | 0.29 | 339895 | 270992 | 363461 | 237440 | 383933 | 246665 | 342989 | 228489 |
| RP_POS_551_26941a7_093 | RP | POS | 7.09 | 551.2694 | | | | 5.83E-01 | -0.11 | 0.54 | 3.92E-01 | 0.18 | 0.62 | 9.38E-01 | -0.02 | 0.35 | 3.68E-01 | -0.19 | 0.63 | 48020240 | 2581354 | 47721069 | 2910749 | 47470238 | 2833635 | 47971900 | 2993317 |
| HILIC_POS_165_06361a0_721 | HILIC | POS | 0.721 | 165.0636 | | | | 1.02E-02 | 0.31 | 0.57 | 3.92E-01 | -0.20 | 0.61 | 3.95E-03 | 0.21 | 1.29 | 7.25E-02 | 0.40 | 0.56 | 724697 | 976053 | 2665318 | 7216777 | 3289142 | 9808853 | 2041494 | 2866850 |
| RP_POS_532_89423a0_493 | RP | POS | 0.49 | 532.8942 | | | | 3.74E-08 | 0.66 | 1.33 | 3.92E-01 | -0.19 | 1.20 | 3.09E-04 | 0.56 | 1.35 | 3.87E-05 | 0.75 | 1.47 | 20505 | 11620 | 531698 | 856270 | 605485 | 914305 | 457911 | 796425 |
| HILIC_POS_199_18044a2_224 | HILIC | POS | 2.224 | 199.1804 | | | | 9.47E-01 | -0.01 | 0.75 | 3.92E-01 | 0.18 | 0.81 | 7.41E-01 | 0.08 | 0.12 | 6.25E-01 | -0.10 | 0.51 | 6660003 | 1584603 | 6637899 | 1779813 | 6484593 | 1571452 | 6791205 | 1970454 |
| RP_POS_631_2738a11_805 | RP | POS | 11.81 | 631.2738 | | | | 9.33E-03 | -0.53 | 1.24 | 3.92E-01 | 0.17 | 1.30 | 6.91E-02 | -0.44 | 0.82 | 2.97E-03 | -0.61 | 1.34 | 1105632 | 490867 | 834351 | 509093 | 790467 | 368227 | 878235 | 619774 |
| HILIC_POS_185_12846a0_617 | HILIC | POS | 0.617 | 185.1285 | | | | 4.26E-01 | 0.15 | 0.84 | 3.92E-01 | 0.18 | 0.80 | 3.16E-01 | 0.24 | 0.44 | 7.34E-01 | 0.07 | 0.34 | 7601618 | 4198668 | 8305292 | 4679431 | 7902083 | 3358432 | 8708502 | 5712282 |
| RP_POS_329_18701a5_652 | RP | POS | 5.65 | 329.1870 | Tiapride OR propentofylline [M+Na]+ | 329.1549(100) | 3 | 4.19E-07 | 0.60 | 1.31 | 3.93E-01 | -0.19 | 1.22 | 8.32E-04 | 0.51 | 1.38 | 1.62E-04 | 0.70 | 1.41 | 24547 | 36226 | 488581 | 854334 | 562063 | 929484 | 415099 | 774409 |
| RP_POS_319_22433a9_813 | RP | POS | 9.81 | 319.2243 | | | | 1.75E-04 | 0.59 | 1.22 | 3.93E-01 | 0.18 | 1.07 | 4.70E-04 | 0.68 | 1.19 | 8.16E-03 | 0.49 | 1.28 | 1079467 | 489582 | 1558357 | 867879 | 1483735 | 857299 | 1632979 | 880610 |
| RP_POS_170_06018a2_892 | RP | POS | 2.89 | 170.0602 | | | | 8.73E-04 | -0.57 | 1.19 | 3.93E-01 | -0.18 | 1.04 | 2.45E-03 | -0.66 | 1.22 | 8.35E-03 | -0.48 | 1.13 | 863228 | 151376 | 743379 | 218986 | 762211 | 183585 | 724547 | 249922 |
| RP_NEG_637_14545a9_358 | RP | NEG | 9.36 | 637.1455 | | | | 6.53E-01 | -0.09 | 0.52 | 3.93E-01 | -0.18 | 0.60 | 4.33E-01 | -0.17 | 0.46 | 9.89E-01 | 0.00 | 0.36 | 12037800 | 694724 | 11971506 | 798381 | 12040140 | 752501 | 11902871 | 843779 |
| RP_POS_441_33472a12_241 | RP | POS | 12.24 | 441.3347 | | | | 3.16E-01 | -0.18 | 0.46 | 3.93E-01 | 0.18 | 0.52 | 6.36E-01 | -0.09 | 0.34 | 2.22E-01 | -0.27 | 0.67 | 11424113 | 872913 | 11233456 | 1087372 | 11139962 | 1212118 | 11326950 | 949640 |
| HILIC_POS_294_20639a0_882 | HILIC | POS | 0.882 | 294.2064 | | | | 3.41E-01 | 0.13 | 0.40 | 3.93E-01 | -0.19 | 1.18 | 7.42E-01 | 0.03 | 0.19 | 3.24E-01 | 0.23 | 0.49 | 1966910 | 991032 | 2261714 | 2538518 | 2480502 | 3438003 | 2042927 | 1049813 |
| HILIC_NEG_236_81744a7_68 | HILIC | NEG | 7.68 | 236.8174 | | | | 4.95E-01 | -0.14 | 0.44 | 3.93E-01 | -0.17 | 0.67 | 3.00E-01 | -0.22 | 0.40 | 8.36E-01 | -0.05 | 0.26 | 1570791 | 306721 | 1527244 | 327582 | 1555388 | 356494 | 1499101 | 296826 |
| RP_POS_199_13036a12_612 | RP | POS | 12.61 | 199.1304 | | | | 8.49E-01 | -0.04 | 0.26 | 3.93E-01 | 0.17 | 0.54 | 8.47E-01 | 0.05 | 0.33 | 5.59E-01 | -0.13 | 0.42 | 13103385 | 6453003 | 12852375 | 6420033 | 12300933 | 5283418 | 13403816 | 7398630 |
| RP_POS_822_53723a13_399 | RP | POS | 13.40 | 822.5372 | | | | 4.44E-01 | -0.17 | 0.58 | 3.94E-01 | -0.16 | 0.60 | 3.05E-01 | -0.26 | 0.59 | 7.02E-01 | -0.09 | 0.48 | 5648181 | 2001473 | 5347519 | 1624869 | 5486879 | 1584516 | 5208159 | 1668519 |
| RP_NEG_580_28802a9_952 | RP | NEG | 9.95 | 580.2880 | | | | 1.22E-02 | 0.41 | 0.94 | 3.94E-01 | -0.18 | 0.60 | 1.14E-01 | 0.32 | 0.75 | 7.67E-03 | 0.51 | 0.92 | 2131770 | 679259 | 2541058 | 1051391 | 2631122 | 971921 | 2450994 | 1127927 |
| RP_POS_203_1499a0_58 | RP | POS | 0.58 | 203.1499 | | | | 1.41E-04 | 0.53 | 1.03 | 3.95E-01 | 0.19 | 1.09 | 8.36E-04 | 0.63 | 1.21 | 1.14E-02 | 0.44 | 0.99 | 1114551 | 214448 | 1366756 | 517497 | 1322456 | 498510 | 1411055 | 537172 |
| RP_POS_340_17786a5_652 | RP | POS | 5.65 | 340.1779 | | | | 4.69E-07 | 0.60 | 1.31 | 3.95E-01 | -0.19 | 1.22 | 9.42E-04 | 0.51 | 1.38 | 1.63E-04 | 0.70 | 1.41 | 60702 | 57833 | 913789 | 1578996 | 1048951 | 1710138 | 778628 | 1440655 |
| HILIC_POS_161_20377a4_843 | HILIC | POS | 4.843 | 161.2038 | | | | 5.05E-01 | 0.13 | 0.37 | 3.95E-01 | -0.18 | 0.79 | 8.54E-01 | 0.04 | 0.07 | 3.02E-01 | 0.22 | 0.45 | 1407660 | 1045904 | 1553784 | 1152556 | 1652415 | 1027337 | 1455153 | 1268309 |
| RP_POS_121_84098a11_668 | RP | POS | 11.67 | 121.8410 | | | | 9.71E-01 | 0.01 | 0.69 | 3.95E-01 | -0.17 | 0.50 | 7.23E-01 | -0.08 | 0.40 | 6.80E-01 | 0.09 | 0.46 | 1469135 | 865737 | 1475650 | 897151 | 1552406 | 918210 | 1398894 | 878096 |
| RP_NEG_640_2915a10_935 | RP | NEG | 10.94 | 640.2915 | | | | 4.57E-02 | -0.48 | 1.25 | 3.95E-01 | -0.15 | 1.78 | 3.46E-02 | -0.55 | 1.20 | 1.04E-01 | -0.40 | 1.04 | 1545548 | 285551 | 1432776 | 213221 | 1451019 | 188420 | 1414533 | 235952 |
| RP_POS_547_33252a5_307 | RP | POS | 5.31 | 547.3325 | | | | 6.22E-05 | 0.51 | 1.28 | 3.95E-01 | -0.19 | 1.20 | 8.56E-03 | 0.42 | 1.30 | 1.16E-03 | 0.61 | 1.37 | 181608 | 458401 | 932174 | 1621582 | 1070861 | 1749970 | 793487 | 1486967 |
| HILIC_NEG_144_92352a5_425 | HILIC | NEG | 5.425 | 144.9235 | | | | 4.84E-01 | 0.16 | 0.47 | 3.95E-01 | 0.16 | 0.52 | 3.39E-01 | 0.24 | 0.39 | 7.38E-01 | 0.08 | 0.20 | 78702047 | 16832977 | 80999235 | 13209473 | 79870259 | 12667049 | 82128211 | 13765394 |
| RP_POS_840_58612a13_563 | RP | POS | 13.56 | 840.5861 | | | | 2.63E-07 | -1.29 | 2.28 | 3.96E-01 | 0.12 | 0.92 | 1.28E-06 | -1.23 | 1.91 | 1.51E-07 | -1.35 | 2.13 | 3077395 | 1055933 | 1845785 | 696657 | 1786263 | 673903 | 1905307 | 720549 |
| RP_POS_270_79749a0_522 | RP | POS | 0.52 | 270.7975 | | | | 9.93E-06 | -0.64 | 1.21 | 3.96E-01 | 0.18 | 1.01 | 2.30E-03 | -0.55 | 1.13 | 8.55E-05 | -0.73 | 1.37 | 681256 | 97086 | 556351 | 208305 | 538558 | 210196 | 574145 | 206977 |
| RP_POS_668_86829a0_493 | RP | POS | 0.49 | 668.8683 | | | | 4.37E-08 | 0.66 | 1.33 | 3.96E-01 | -0.19 | 1.20 | 3.41E-04 | 0.56 | 1.36 | 4.14E-05 | 0.75 | 1.48 | 103229 | 67913 | 3202947 | 5222909 | 3649065 | 5566211 | 2756830 | 4870873 |

**Supplementary table 1: Metabolomics results** (in asceding order according to p-value from progressive ALD vs  non-progressive ALD comparison, with molecular features with ID_level of 1-2 first)

| | | | | | | | | all_ALD_vs_control | | | Prog_ALD_vs_non-prog_ALD | | | Prog_ALD_vs_control_ALD | | | Non-prog_ALD_vs_control | | | Healthy_control | | all_ALD | | Non-prog_ALD | | Progressive_ALD | |
|---|---|---|---|---|---|---|---|---|---|---|---|---|---|---|---|---|---|---|---|---|---|---|---|---|---|---|---|
| | | | | | | | | t-test | Cohen's | PLS-DA | t-test | Cohen's | PLS-DA | t-test | Cohen's | PLS-DA | t-test | Cohen's | PLS-DA | | | | | | | | |
| Feature_ID | RP_HILIC | POS_NEG | Average.Rt | Average.Mz | Metabolite.name | curated_MSMS | ID_level | p | d | VIP | p | d | VIP | p | d | VIP | p | d | VIP | Mean | SD | Mean | SD | Mean | SD | Mean | SD |
| HILIC_POS_224_1277a4_785 | HILIC | POS | 4.785 | 224.1277 | | | | 1.34E-01 | 0.30 | 0.53 | 3.96E-01 | 0.17 | 0.64 | 1.15E-01 | 0.38 | 0.72 | 2.92E-01 | 0.21 | 0.48 | 5097065 | 2077323 | 5748815 | 2218046 | 5559235 | 1644341 | 5938396 | 2676314 |
| RP_POS_373_2251a11_608 | RP | POS | 11.61 | 373.2251 | | | | 1.33E-02 | -0.50 | 0.93 | 3.96E-01 | 0.17 | 0.88 | 4.67E-02 | -0.41 | 0.87 | 1.45E-02 | -0.58 | 0.96 | 5362390 | 494177 | 5102503 | 518036 | 5058254 | 597144 | 5146753 | 426171 |
| RP_POS_525_3913a11_606 | RP | POS | 11.61 | 525.3913 | | | | 8.74E-02 | -0.34 | 0.72 | 3.96E-01 | -0.17 | 0.79 | 6.75E-02 | -0.43 | 0.78 | 2.31E-01 | -0.25 | 0.69 | 671560 | 430491 | 516933 | 458593 | 556057 | 407723 | 477809 | 505514 |
| RP_POS_159_98203a15_424 | RP | POS | 15.42 | 159.9820 | | | | 8.70E-01 | 0.03 | 0.56 | 3.97E-01 | -0.18 | 0.56 | 7.87E-01 | -0.06 | 0.36 | 5.78E-01 | 0.12 | 0.37 | 52987851 | 1377154 | 53036189 | 1635687 | 53175672 | 1642262 | 52896706 | 1633652 |
| RP_NEG_701_15576a9_437 | RP | NEG | 9.44 | 701.1558 | | | | 3.39E-01 | -0.20 | 0.58 | 3.97E-01 | -0.17 | 0.63 | 2.28E-01 | -0.28 | 0.57 | 6.17E-01 | -0.11 | 0.32 | 11600312 | 713865 | 11460580 | 708278 | 11520968 | 670839 | 11400192 | 745712 |
| RP_POS_310_91199a0_497 | RP | POS | 0.50 | 310.9120 | | | | 1.72E-08 | -0.68 | 1.35 | 3.97E-01 | 0.19 | 1.20 | 2.08E-04 | -0.59 | 1.38 | 2.41E-05 | -0.77 | 1.50 | 22879308 | 474248 | 19090713 | 6132902 | 18567833 | 6518664 | 19613592 | 5739479 |
| RP_POS_570_45563a10_66 | RP | POS | 10.66 | 570.4556 | | | | 2.87E-01 | -0.21 | 0.44 | 3.98E-01 | 0.17 | 0.52 | 5.63E-01 | -0.13 | 0.45 | 1.87E-01 | -0.30 | 0.58 | 8813500 | 635418 | 8673479 | 656879 | 8617595 | 671324 | 8729362 | 644000 |
| HILIC_POS_340_24741a0_705 | HILIC | POS | 0.705 | 340.2474 | | | | 1.40E-01 | -0.28 | 0.87 | 3.98E-01 | 0.17 | 0.76 | 3.90E-01 | -0.20 | 0.32 | 7.25E-02 | -0.37 | 0.99 | 1574065 | 629784 | 1375788 | 716881 | 1314797 | 623795 | 1436779 | 801007 |
| HILIC_POS_274_87381a2_979 | HILIC | POS | 2.979 | 274.8738 | | | | 1.33E-02 | -0.44 | 1.22 | 3.98E-01 | 0.18 | 0.84 | 8.27E-02 | -0.35 | 1.20 | 1.21E-02 | -0.53 | 1.27 | 909655 | 183560 | 805881 | 248006 | 784787 | 255731 | 826974 | 240754 |
| RP_POS_320_25577a9_62 | RP | POS | 9.62 | 320.2558 | | | | 4.65E-01 | -0.19 | 0.78 | 3.98E-01 | -0.14 | 0.59 | 3.35E-01 | -0.26 | 0.66 | 6.66E-01 | -0.12 | 0.46 | 23687273 | 31944474 | 19284390 | 19294343 | 20925207 | 20522920 | 17643573 | 18040400 |
| RP_NEG_437_1618a10_386 | RP | NEG | 10.39 | 437.1618 | | | | 6.87E-01 | -0.06 | 0.52 | 3.98E-01 | 0.19 | 0.52 | 9.09E-01 | 0.03 | 0.20 | 2.33E-01 | -0.16 | 0.64 | 575439 | 658981 | 511634 | 1068366 | 420509 | 378101 | 602759 | 1464991 |
| RP_POS_600_88123a0_493 | RP | POS | 0.49 | 600.8812 | | | | 4.77E-08 | 0.65 | 1.34 | 3.98E-01 | -0.19 | 1.21 | 3.11E-04 | 0.56 | 1.37 | 4.89E-05 | 0.75 | 1.48 | 121910 | 81009 | 2576044 | 4147778 | 2928662 | 4455852 | 2223427 | 3827644 |
| HILIC_POS_156_07663a6_786 | HILIC | POS | 6.786 | 156.0766 | | | | 5.73E-01 | -0.10 | 0.93 | 3.98E-01 | -0.18 | 0.88 | 3.48E-01 | -0.19 | 0.68 | 9.51E-01 | -0.01 | 0.75 | 65527446 | 16948440 | 63445251 | 21305605 | 65256267 | 22574580 | 61634236 | 20020398 |
| RP_POS_321_24002a9_95 | RP | POS | 9.95 | 321.2400 | | | | 2.23E-13 | 0.97 | 1.60 | 3.98E-01 | 0.17 | 1.12 | 2.64E-08 | 1.06 | 1.71 | 8.57E-08 | 0.89 | 1.71 | 1400509 | 560514 | 3006711 | 1694439 | 2862723 | 1569361 | 3150700 | 1815389 |
| RP_NEG_532_30115a10_51 | RP | NEG | 10.51 | 532.3012 | | | | 4.51E-01 | -0.14 | 0.76 | 3.99E-01 | 0.18 | 0.55 | 8.12E-01 | -0.06 | 0.48 | 2.42E-01 | -0.23 | 0.91 | 1476994 | 512321 | 1394894 | 590540 | 1344716 | 466192 | 1445072 | 694347 |
| RP_NEG_306_91946a0_512 | RP | NEG | 0.51 | 306.9195 | | | | 3.02E-04 | 0.47 | 1.20 | 3.99E-01 | -0.19 | 1.17 | 2.21E-02 | 0.37 | 1.13 | 2.36E-03 | 0.56 | 1.35 | 1167266 | 276269 | 1555903 | 925452 | 1634474 | 981748 | 1477332 | 868318 |
| RP_POS_337_06329a0_706 | RP | POS | 0.71 | 337.0633 | | | | 6.27E-02 | 0.29 | 0.62 | 3.99E-01 | 0.18 | 0.54 | 8.13E-02 | 0.38 | 0.75 | 2.06E-01 | 0.20 | 0.56 | 1622814 | 613776 | 1909858 | 1069295 | 1819007 | 771195 | 2000709 | 1303277 |
| RP_NEG_756_82141a0_495 | RP | NEG | 0.50 | 756.8214 | | | | 4.36E-09 | -0.72 | 1.38 | 3.99E-01 | 0.19 | 1.21 | 1.36E-04 | -0.62 | 1.42 | 7.39E-06 | -0.81 | 1.53 | 9705174 | 497250 | 7243781 | 3755315 | 6925099 | 3893851 | 7562463 | 3622602 |
| HILIC_POS_118_12287a0_85 | HILIC | POS | 0.85 | 118.1229 | | | | 4.01E-01 | -0.16 | 0.80 | 4.00E-01 | 0.17 | 0.83 | 7.30E-01 | -0.07 | 0.13 | 2.60E-01 | -0.25 | 0.46 | 779583 | 136283 | 755391 | 154027 | 742341 | 157666 | 768441 | 150755 |
| RP_NEG_584_23071a9_952 | RP | NEG | 9.95 | 584.2307 | | | | 9.71E-03 | 0.42 | 0.91 | 4.00E-01 | -0.18 | 0.59 | 1.08E-01 | 0.32 | 0.75 | 6.22E-03 | 0.51 | 0.93 | 621677 | 195598 | 746594 | 321989 | 773864 | 294699 | 719323 | 348001 |
| RP_POS_467_37024a11_422 | RP | POS | 11.42 | 467.3702 | | | | 7.40E-01 | 0.06 | 0.59 | 4.00E-01 | 0.18 | 0.67 | 4.87E-01 | 0.15 | 0.39 | 8.74E-01 | -0.03 | 0.58 | 1282666 | 170534 | 1295255 | 228869 | 1275874 | 213422 | 1314637 | 243958 |
| HILIC_NEG_183_93422a4_804 | HILIC | NEG | 4.804 | 183.9342 | | | | 7.01E-01 | 0.08 | 0.54 | 4.00E-01 | 0.17 | 0.79 | 4.74E-01 | 0.16 | 0.49 | 9.81E-01 | -0.01 | 0.18 | 1784860 | 624434 | 1833678 | 616585 | 1781477 | 623563 | 1885880 | 611328 |
| RP_POS_163_98772a10_57 | RP | POS | 10.57 | 163.9877 | | | | 2.01E-01 | -0.26 | 0.46 | 4.00E-01 | 0.17 | 0.67 | 4.43E-01 | -0.18 | 0.38 | 1.21E-01 | -0.35 | 0.60 | 1999837 | 565473 | 1851412 | 561883 | 1803841 | 527465 | 1898983 | 595845 |
| RP_NEG_815_5556a12_771 | RP | NEG | 12.77 | 815.5556 | | | | 6.16E-02 | -0.36 | 0.84 | 4.00E-01 | 0.17 | 0.53 | 2.10E-01 | -0.27 | 0.61 | 3.99E-02 | -0.45 | 0.85 | 2047809 | 548500 | 1828301 | 621962 | 1775671 | 612582 | 1880931 | 632975 |
| RP_POS_700_81635a0_495 | RP | POS | 0.50 | 700.8164 | | | | 1.62E-09 | -0.74 | 1.40 | 4.00E-01 | 0.18 | 1.20 | 9.55E-05 | -0.65 | 1.43 | 3.10E-06 | -0.83 | 1.54 | 2919679 | 199753 | 2083849 | 1223297 | 1980347 | 1248478 | 2187351 | 1201205 |
| HILIC_POS_609_40076a1_277 | HILIC | POS | 1.277 | 609.4008 | | | | 8.42E-01 | 0.04 | 0.57 | 4.00E-01 | -0.17 | 0.56 | 8.62E-01 | -0.04 | 0.24 | 5.93E-01 | 0.13 | 0.21 | 587688 | 241343 | 597336 | 223861 | 616273 | 224328 | 578400 | 224044 |
| RP_POS_287_63608a9_952 | RP | POS | 9.95 | 287.6361 | | | | 5.49E-03 | 0.44 | 0.95 | 4.01E-01 | -0.18 | 0.59 | 7.48E-02 | 0.35 | 0.80 | 5.04E-03 | 0.53 | 0.97 | 763114 | 254052 | 941173 | 436155 | 978057 | 420464 | 904290 | 452525 |
| HILIC_POS_310_20111a0_87 | HILIC | POS | 0.87 | 310.2011 | | | | 8.88E-01 | 0.03 | 0.35 | 4.01E-01 | 0.17 | 0.59 | 6.26E-01 | 0.11 | 0.29 | 7.87E-01 | -0.06 | 0.10 | 5735845 | 3940701 | 5851151 | 4161313 | 5499512 | 3687422 | 6202791 | 4597501 |
| RP_POS_605_40082a10_894 | RP | POS | 10.89 | 605.4008 | | | | 8.54E-01 | 0.04 | 0.62 | 4.01E-01 | -0.17 | 0.50 | 8.20E-01 | -0.05 | 0.44 | 5.89E-01 | 0.12 | 0.42 | 1760541 | 598175 | 1783328 | 640286 | 1837385 | 664924 | 1729270 | 616613 |
| RP_POS_335_22266a5_652 | RP | POS | 5.65 | 335.2227 | | | | 4.55E-07 | 0.60 | 1.31 | 4.01E-01 | -0.19 | 1.20 | 8.57E-04 | 0.51 | 1.37 | 1.72E-04 | 0.70 | 1.41 | 52537 | 59483 | 785043 | 1353180 | 899273 | 1470402 | 670812 | 1229166 |
| HILIC_NEG_200_85867a7_393 | HILIC | NEG | 7.393 | 200.8587 | | | | 8.45E-01 | -0.03 | 0.14 | 4.01E-01 | -0.18 | 0.78 | 5.48E-01 | -0.12 | 0.26 | 7.59E-01 | 0.06 | 0.14 | 2288403 | 63362 | 2285518 | 96014 | 2293622 | 90289 | 2277413 | 101683 |
| RP_POS_194_11745a0_71 | RP | POS | 0.71 | 194.1175 | | | | 4.98E-01 | 0.12 | 0.63 | 4.01E-01 | 0.18 | 0.53 | 3.28E-01 | 0.21 | 0.46 | 8.80E-01 | 0.03 | 0.62 | 2735834 | 286458 | 2779134 | 384244 | 2746706 | 359006 | 2811561 | 408985 |
| RP_POS_149_99751a15_465 | RP | POS | 15.47 | 149.9975 | | | | 9.33E-01 | -0.02 | 0.43 | 4.02E-01 | 0.18 | 0.57 | 7.61E-01 | 0.07 | 0.50 | 5.90E-01 | -0.10 | 0.46 | 4235858 | 2028721 | 4199562 | 2380663 | 3998557 | 1782136 | 4400567 | 2862221 |
| RP_POS_199_13007a11_221 | RP | POS | 11.22 | 199.1301 | | | | 4.02E-01 | -0.21 | 0.38 | 4.02E-01 | -0.15 | 0.50 | 2.79E-01 | -0.28 | 0.57 | 6.18E-01 | -0.13 | 0.36 | 3023113 | 1381721 | 2801286 | 953371 | 2881702 | 997820 | 2720869 | 909692 |
| RP_POS_413_22025a10_874 | RP | POS | 10.87 | 413.2203 | | | | 7.52E-02 | 0.32 | 0.69 | 4.02E-01 | 0.18 | 0.64 | 5.51E-02 | 0.41 | 0.83 | 2.49E-01 | 0.24 | 0.63 | 475587 | 221101 | 562449 | 279419 | 538893 | 268742 | 586005 | 290497 |
| RP_POS_308_29471a10_994 | RP | POS | 10.99 | 308.2947 | | | | 2.25E-07 | 0.70 | 1.13 | 4.02E-01 | 0.18 | 1.01 | 2.80E-04 | 0.79 | 1.20 | 2.08E-06 | 0.61 | 1.56 | 963779 | 455970 | 1786217 | 1268631 | 1679157 | 806303 | 1893278 | 1605670 |
| RP_NEG_583_2572a5_909 | RP | NEG | 5.91 | 583.2572 | | | | 4.87E-01 | 0.11 | 0.84 | 4.02E-01 | 0.18 | 1.11 | 3.38E-01 | 0.20 | 0.56 | 9.10E-01 | 0.02 | 1.00 | 813993 | 504715 | 899012 | 829194 | 829134 | 697604 | 968890 | 944743 |
| RP_POS_564_35895a5_307 | RP | POS | 5.31 | 564.3590 | | | | 5.56E-05 | 0.52 | 1.28 | 4.02E-01 | -0.19 | 1.20 | 8.08E-03 | 0.42 | 1.31 | 1.12E-03 | 0.61 | 1.37 | 537874 | 1185658 | 2517612 | 4260139 | 2876455 | 4589932 | 2158769 | 3916334 |
| HILIC_NEG_252_95584a4_801 | HILIC | NEG | 4.801 | 252.9558 | | | | 3.00E-01 | 0.21 | 0.45 | 4.03E-01 | 0.17 | 0.52 | 1.64E-01 | 0.29 | 0.61 | 6.11E-01 | 0.12 | 0.28 | 418551 | 226581 | 467578 | 242385 | 447165 | 276786 | 487992 | 203073 |
| RP_NEG_134_8931a15_597 | RP | NEG | 15.60 | 134.8931 | | | | 7.12E-02 | -0.25 | 0.72 | 4.03E-01 | 0.19 | 0.52 | 3.29E-01 | -0.16 | 0.56 | 7.73E-02 | -0.35 | 0.68 | 2178780 | 96282 | 2126899 | 228681 | 2107644 | 253304 | 2146154 | 201837 |
| HILIC_POS_466_24567a0_8 | HILIC | POS | 0.8 | 466.2457 | | | | 5.98E-01 | -0.12 | 0.66 | 4.03E-01 | 0.16 | 0.56 | 8.65E-01 | -0.04 | 0.37 | 4.28E-01 | -0.20 | 0.70 | 554349 | 335229 | 519763 | 270589 | 496991 | 287718 | 542535 | 253163 |
| RP_POS_298_27411a9_809 | RP | POS | 9.81 | 298.2741 | | | | 4.89E-01 | -0.19 | 0.83 | 4.03E-01 | -0.13 | 0.62 | 3.65E-01 | -0.26 | 0.69 | 6.69E-01 | -0.12 | 0.49 | 5359624 | 9141841 | 4178540 | 4929263 | 4593025 | 5283649 | 3764054 | 4563287 |
| HILIC_NEG_289_06821a1_415 | HILIC | NEG | 1.415 | 289.0682 | | | | 6.84E-01 | 0.07 | 0.80 | 4.03E-01 | -0.18 | 0.68 | 9.37E-01 | -0.02 | 0.64 | 4.33E-01 | 0.16 | 0.91 | 402580 | 125942 | 413930 | 165650 | 427855 | 162839 | 400006 | 168901 |
| RP_POS_320_2561a9_752 | RP | POS | 9.75 | 320.2561 | | | | 1.82E-01 | -0.42 | 1.17 | 4.04E-01 | -0.10 | 0.68 | 1.40E-01 | -0.47 | 0.90 | 2.53E-01 | -0.36 | 0.87 | 8866798 | 14428682 | 5315752 | 5309435 | 5761788 | 5616213 | 4869715 | 5000764 |
| RP_NEG_405_1579a11_168 | RP | NEG | 11.17 | 405.1579 | | | | 5.15E-01 | 0.13 | 0.44 | 4.04E-01 | -0.17 | 0.73 | 8.53E-01 | 0.04 | 0.57 | 3.27E-01 | 0.21 | 0.61 | 818166 | 101213 | 831942 | 110640 | 841232 | 106224 | 822651 | 115206 |
| RP_POS_319_20282a10_978 | RP | POS | 10.98 | 319.2028 | | | | 6.33E-03 | 0.45 | 1.32 | 4.04E-01 | -0.18 | 1.22 | 7.00E-02 | 0.36 | 1.10 | 5.90E-03 | 0.54 | 1.31 | 1893302 | 869571 | 2472156 | 1381864 | 2588106 | 1354666 | 2356206 | 1412625 |
| RP_NEG_437_16223a10_264 | RP | NEG | 10.26 | 437.1622 | | | | 4.50E-01 | -0.20 | 0.48 | 4.05E-01 | -0.14 | 0.56 | 3.34E-01 | -0.27 | 0.39 | 6.42E-01 | -0.13 | 0.38 | 1580309 | 2020108 | 1291881 | 1241793 | 1396001 | 1171385 | 1187760 | 1311985 |
| HILIC_POS_243_18253a1_504 | HILIC | POS | 1.504 | 243.1825 | | | | 4.77E-02 | 0.27 | 0.65 | 4.05E-01 | 0.19 | 0.98 | 8.84E-02 | 0.36 | 0.73 | 1.83E-01 | 0.18 | 0.65 | 186502 | 174864 | 295612 | 449585 | 257894 | 305546 | 333331 | 558674 |
| RP_POS_578_41718a11_375 | RP | POS | 11.38 | 578.4172 | | | | 1.86E-01 | -0.36 | 0.69 | 4.05E-01 | 0.14 | 1.31 | 2.97E-01 | -0.29 | 0.52 | 1.37E-01 | -0.42 | 0.75 | 590542 | 522300 | 459839 | 298597 | 434816 | 321350 | 484861 | 274948 |
| RP_POS_647_45752a14_19 | RP | POS | 14.19 | 647.4575 | | | | 2.87E-01 | 0.16 | 0.40 | 4.05E-01 | -0.18 | 0.50 | 6.93E-01 | 0.07 | 0.49 | 2.00E-01 | 0.25 | 0.49 | 613956 | 332150 | 705005 | 614883 | 756525 | 659303 | 653485 | 569017 |
| RP_POS_812_50983a10_932 | RP | POS | 10.93 | 812.5098 | | | | 2.30E-02 | -0.54 | 1.19 | 4.05E-01 | -0.15 | 1.43 | 1.61E-02 | -0.62 | 1.13 | 6.22E-02 | -0.47 | 1.07 | 524087 | 279490 | 397664 | 208125 | 415098 | 203588 | 380229 | 213185 |
| RP_POS_260_87869a0_251 | RP | POS | 0.25 | 260.8787 | | | | 4.88E-01 | -0.15 | 0.36 | 4.05E-01 | -0.16 | 1.04 | 3.38E-01 | -0.23 | 0.51 | 7.60E-01 | -0.07 | 0.41 | 2401412 | 309481 | 2358773 | 268448 | 2381260 | 257811 | 2336286 | 279465 |
| HILIC_POS_334_08151a5_462 | HILIC | POS | 5.462 | 334.0815 | | | | 2.75E-01 | -0.24 | 0.43 | 4.05E-01 | -0.16 | 0.77 | 1.94E-01 | -0.32 | 0.53 | 4.97E-01 | -0.16 | 0.28 | 1649494 | 280050 | 1588306 | 248626 | 1609128 | 227208 | 1567484 | 269040 |
| HILIC_NEG_311_14035a1_689 | HILIC | NEG | 1.689 | 311.1404 | | | | 5.83E-06 | 0.54 | 1.27 | 4.05E-01 | 0.19 | 0.81 | 1.08E-03 | 0.63 | 1.42 | 1.54E-03 | 0.44 | 1.32 | 14755 | 21704 | 919717 | 1888425 | 761571 | 1573423 | 1077864 | 2162955 |
| RP_POS_565_28265a5_011 | RP | POS | 5.01 | 565.2827 | | | | 1.05E-06 | 0.58 | 1.25 | 4.05E-01 | -0.19 | 1.17 | 1.19E-03 | 0.49 | 1.34 | 2.92E-04 | 0.67 | 1.35 | 20801 | 16875 | 1149072 | 2167009 | 1330447 | 2374123 | 967697 | 1945273 |
| RP_POS_324_21762a9_441 | RP | POS | 9.44 | 324.2176 | | | | 2.63E-01 | -0.21 | 0.42 | 4.05E-01 | -0.17 | 0.96 | 1.92E-01 | -0.29 | 0.54 | 5.48E-01 | -0.12 | 0.39 | 1189472 | 79108 | 1170255 | 96977 | 1178371 | 84336 | 1162138 | 108416 |
| RP_POS_265_25317a10_223 | RP | POS | 10.22 | 265.2532 | | | | 5.99E-01 | 0.10 | 0.79 | 4.06E-01 | 0.18 | 0.81 | 3.90E-01 | 0.18 | 0.67 | 9.69E-01 | 0.01 | 0.77 | 1927666 | 258824 | 1957354 | 325330 | 1930133 | 317148 | 1984574 | 334281 |
| RP_POS_522_79742a10_501 | RP | POS | 10.50 | 522.7974 | | | | 2.30E-01 | -0.27 | 0.74 | 4.06E-01 | 0.16 | 0.50 | 4.30E-01 | -0.19 | 0.45 | 1.57E-01 | -0.35 | 0.81 | 5254167 | 1277593 | 4949860 | 1074395 | 4859977 | 1109686 | 5039742 | 1041305 |
| RP_POS_635_38452a5_652 | RP | POS | 5.65 | 635.3845 | | | | 4.26E-07 | 0.60 | 1.31 | 4.06E-01 | -0.19 | 1.21 | 8.68E-04 | 0.51 | 1.37 | 1.60E-04 | 0.70 | 1.41 | 25616 | 40237 | 551214 | 968365 | 632220 | 1047724 | 470208 | 885144 |
| RP_POS_265_25269a10_555 | RP | POS | 10.56 | 265.2527 | | | | 8.25E-04 | -0.61 | 1.13 | 4.06E-01 | 0.17 | 0.88 | 8.46E-03 | -0.53 | 1.04 | 1.30E-03 | -0.70 | 1.21 | 573411 | 78483 | 513633 | 99008 | 505354 | 105736 | 521913 | 92116 |
| HILIC_POS_178_08606a3_645 | HILIC | POS | 3.645 | 178.0861 | | | | 4.87E-01 | -0.14 | 0.58 | 4.06E-01 | -0.17 | 0.65 | 3.10E-01 | -0.22 | 0.39 | 8.13E-01 | -0.05 | 0.50 | 1510811 | 637908 | 1418969 | 672503 | 1475161 | 704175 | 1362776 | 641439 |
| RP_NEG_473_34885a11_551 | RP | NEG | 11.55 | 473.3489 | | | | 9.28E-01 | 0.01 | 0.43 | 4.06E-01 | -0.19 | 0.69 | 2.39E-01 | -0.08 | 0.68 | 6.49E-01 | 0.11 | 0.51 | 1498030 | 2399004 | 1577353 | 7679098 | 2221434 | 10774020 | 933272 | 1488083 |
| RP_POS_342_2637a7_887 | RP | POS | 7.89 | 342.2637 | | | | 1.02E-03 | 0.54 | 1.08 | 4.06E-01 | 0.18 | 0.80 | 2.96E-03 | 0.62 | 1.09 | 1.24E-02 | 0.45 | 1.12 | 2334194 | 1064954 | 3197757 | 1701354 | 3055638 | 1484217 | 3339875 | 1898463 |
| HILIC_POS_338_14975a0_767 | HILIC | POS | 0.767 | 338.1498 | | | | 3.00E-01 | -0.14 | 0.46 | 4.07E-01 | 0.19 | 0.62 | 7.85E-01 | -0.05 | 0.13 | 1.78E-01 | -0.24 | 0.57 | 23502841 | 5253831 | 21829798 | 13114692 | 20735472 | 12806548 | 22924124 | 13455436 |
| HILIC_POS_158_11755a2_288 | HILIC | POS | 2.288 | 158.1176 | | | | 9.93E-05 | 0.56 | 1.01 | 4.07E-01 | 0.18 | 0.75 | 2.10E-03 | 0.65 | 1.10 | 1.59E-03 | 0.47 | 1.08 | 602706 | 193419 | 821938 | 422417 | 786666 | 316021 | 857210 | 508041 |
| RP_POS_790_53522a12_745 | RP | POS | 12.75 | 790.5352 | | | | 3.61E-03 | -0.65 | 1.43 | 4.07E-01 | -0.15 | 0.70 | 3.11E-03 | -0.72 | 1.46 | 1.45E-02 | -0.57 | 1.13 | 2652343 | 1002110 | 2047963 | 866012 | 2120154 | 810070 | 1975772 | 921092 |
| RP_POS_801_55127a11_626 | RP | POS | 11.63 | 801.5513 | | | | 5.85E-01 | 0.09 | 0.24 | 4.07E-01 | 0.18 | 0.60 | 4.09E-01 | 0.18 | 0.44 | 9.75E-01 | 0.01 | 0.60 | 1248305 | 322797 | 1288141 | 449063 | 1250693 | 366411 | 1325588 | 519916 |
| RP_POS_844_77747a0_497 | RP | POS | 0.50 | 844.7775 | | | | 1.17E-09 | -0.74 | 1.41 | 4.08E-01 | 0.18 | 1.21 | 8.64E-05 | -0.65 | 1.45 | 2.84E-06 | -0.83 | 1.55 | 1900977 | 106398 | 1374887 | 768209 | 1310887 | 782921 | 1438887 | 755651 |
| HILIC_POS_139_12299a0_691 | HILIC | POS | 0.691 | 139.1230 | | | | 7.80E-01 | 0.05 | 0.28 | 4.08E-01 | 0.17 | 0.69 | 5.13E-01 | 0.14 | 0.25 | 8.95E-01 | -0.03 | 0.57 | 10813460 | 4101948 | 11052672 | 4450385 | 10681908 | 4808706 | 11423435 | 4075764 |
| RP_NEG_502_27188a9_951 | RP | NEG | 9.95 | 502.2719 | | | | 8.86E-03 | 0.42 | 0.92 | 4.08E-01 | -0.18 | 0.58 | 9.99E-02 | 0.33 | 0.75 | 5.73E-03 | 0.51 | 0.94 | 763663 | 250047 | 924277 | 405151 | 958015 | 369549 | 890538 | 439038 |
| HILIC_POS_163_08652a0_637 | HILIC | POS | 0.637 | 163.0865 | | | | 5.87E-06 | 0.74 | 1.41 | 4.08E-01 | 0.17 | 0.93 | 1.05E-04 | 0.83 | 1.31 | 1.52E-04 | 0.66 | 1.62 | 106882 | 229515 | 370693 | 364241 | 340356 | 298958 | 401031 | 420472 |
| RP_POS_382_0553a0_615 | RP | POS | 0.62 | 382.0553 | | | | 2.13E-07 | 0.66 | 1.25 | 4.08E-01 | -0.18 | 1.09 | 1.17E-04 | 0.56 | 1.39 | 1.52E-04 | 0.75 | 1.37 | 121924 | 72534 | 297258 | 292522 | 321617 | 335477 | 272900 | 243164 |
| RP_NEG_552_3316a10_583 | RP | NEG | 10.58 | 552.3316 | | | | 5.31E-01 | -0.13 | 0.81 | 4.08E-01 | -0.17 | 1.03 | 3.53E-01 | -0.21 | 0.62 | 8.51E-01 | -0.04 | 0.44 | 822133 | 339462 | 778345 | 349680 | 807442 | 349540 | 749248 | 350915 |
| RP_POS_326_24545a9_368 | RP | POS | 9.37 | 326.2455 | | | | 4.54E-01 | 0.13 | 0.58 | 4.08E-01 | -0.18 | 0.59 | 8.36E-01 | 0.04 | 0.32 | 3.32E-01 | 0.22 | 0.69 | 481269 | 72452 | 493834 | 107391 | 502774 | 126933 | 484893 | 83821 |
| RP_POS_242_13062a5_147 | RP | POS | 5.15 | 242.1306 | | | | 8.95E-02 | 0.22 | 0.48 | 4.08E-01 | -0.19 | 0.50 | 1.06E-01 | 0.12 | 0.91 | 1.80E-01 | 0.31 | 0.59 | 4313454 | 5782156 | 8298012 | 20925564 | 10044481 | 28938390 | 6551543 | 6406211 |
| RP_POS_810_53583a13_196 | RP | POS | 13.20 | 810.5358 | | | | 2.16E-02 | 0.44 | 0.80 | 4.09E-01 | 0.17 | 0.85 | 1.48E-02 | 0.52 | 0.97 | 1.04E-01 | 0.35 | 0.67 | 902399 | 352399 | 1078214 | 409435 | 1044182 | 421069 | 1112246 | 398790 |
| RP_POS_341_30478a11_172 | RP | POS | 11.17 | 341.3048 | | | | 8.43E-01 | 0.03 | 0.35 | 4.09E-01 | 0.18 | 0.60 | 5.64E-01 | 0.12 | 0.48 | 7.49E-01 | -0.06 | 0.41 | 340018 | 228346 | 350732 | 357924 | 320975 | 308245 | 380489 | 402506 |
| RP_POS_313_20966a5_487 | RP | POS | 5.49 | 313.2097 | | | | 1.22E-06 | 0.58 | 1.31 | 4.09E-01 | -0.18 | 1.21 | 1.45E-03 | 0.49 | 1.37 | 2.49E-04 | 0.67 | 1.41 | 30253 | 65228 | 350655 | 611552 | 401457 | 660822 | 299853 | 560064 |
| HILIC_POS_118_65714a3_687 | HILIC | POS | 3.687 | 118.6571 | | | | 8.11E-02 | 0.32 | 0.60 | 4.09E-01 | 0.17 | 0.93 | 7.92E-02 | 0.40 | 0.73 | 2.19E-01 | 0.23 | 0.56 | 933980 | 729841 | 1215323 | 925795 | 1138375 | 721931 | 1292271 | 1094722 |
| RP_POS_806_50671a11_625 | RP | POS | 11.63 | 806.5067 | | | | 5.61E-01 | 0.10 | 0.24 | 4.09E-01 | 0.18 | 0.56 | 3.93E-01 | 0.19 | 0.40 | 9.54E-01 | 0.01 | 0.60 | 8558296 | 2482543 | 8887983 | 3552612 | 8592993 | 2923583 | 9182973 | 4095699 |
| RP_POS_795_57495a13_395 | RP | POS | 13.40 | 795.5750 | | | | 9.49E-01 | 0.01 | 0.37 | 4.10E-01 | -0.16 | 0.53 | 7.77E-01 | -0.07 | 0.49 | 6.85E-01 | 0.10 | 0.50 | 6249705 | 2125244 | 6276952 | 1942936 | 6438081 | 1895964 | 6115824 | 1994838 |
| HILIC_NEG_153_86877a7_393 | HILIC | NEG | 7.393 | 153.8688 | | | | 8.72E-01 | 0.03 | 0.35 | 4.10E-01 | 0.17 | 0.49 | 5.95E-01 | 0.12 | 0.33 | 7.73E-01 | -0.06 | 0.35 | 512517 | 34526 | 513735 | 43638 | 510117 | 39766 | 517353 | 47322 |
| RP_POS_897_53479a10_842 | RP | POS | 10.84 | 897.5348 | | | | 6.50E-03 | 0.33 | 0.72 | 4.10E-01 | -0.19 | 0.73 | 7.40E-02 | 0.24 | 0.73 | 3.08E-02 | 0.43 | 0.92 | 1493480 | 186034 | 1749307 | 863234 | 1820858 | 1017738 | 1677756 | 677728 |
| RP_NEG_204_06639a4_894 | RP | NEG | 4.89 | 204.0664 | | | | 4.12E-02 | 0.30 | 0.69 | 4.10E-01 | -0.18 | 0.55 | 2.39E-01 | 0.21 | 0.53 | 4.15E-02 | 0.39 | 0.76 | 5045142 | 1622025 | 5952670 | 3327455 | 6228241 | 3487007 | 5677098 | 3171069 |
| HILIC_NEG_512_29919a1_357 | HILIC | NEG | 1.357 | 512.2992 | | | | 7.69E-04 | 0.52 | 0.78 | 4.10E-01 | -0.18 | 0.46 | 2.64E-02 | 0.43 | 0.73 | 1.03E-03 | 0.61 | 1.12 | 292043 | 104089 | 383942 | 190200 | 399693 | 181411 | 368190 | 199184 |
| RP_POS_1003_66943a10_378 | RP | POS | 10.38 | 1003.6694 | | | | 3.98E-03 | 0.50 | 1.13 | 4.10E-01 | -0.17 | 0.79 | 4.26E-02 | 0.41 | 0.93 | 3.82E-03 | 0.58 | 1.15 | 209535 | 182278 | 332446 | 259042 | 353894 | 255608 | 310997 | 263250 |
| RP_POS_337_70026a5_652 | RP | POS | 5.65 | 337.7003 | | | | 4.23E-07 | 0.60 | 1.31 | 4.10E-01 | -0.18 | 1.21 | 8.47E-04 | 0.51 | 1.38 | 1.63E-04 | 0.69 | 1.41 | 116241 | 103518 | 1510693 | 2568714 | 1723386 | 2780029 | 1297999 | 2347415 |
| HILIC_NEG_134_8947a5_136 | HILIC | NEG | 5.136 | 134.8947 | | | | 6.41E-01 | -0.09 | 0.28 | 4.11E-01 | 0.17 | 0.62 | 9.82E-01 | 0.00 | 0.07 | 4.31E-01 | -0.18 | 0.29 | 21413138 | 5229450 | 20901292 | 5840275 | 20417792 | 6023230 | 21384792 | 5670743 |

**Supplementary table 1: Metabolomics results** (in asceding order according to p-value from progressive ALD vs  non-progressive ALD comparison, with molecular features with ID_level of 1-2 first)

| | | | | | | | | all_ALD_vs_control | | | Prog_ALD_vs_non-prog_ALD | | | Prog_ALD_vs_control_ALD | | | Non-prog_ALD_vs_control | | | Healthy_control | | all_ALD | | Non-prog_ALD | | Progressive_ALD | |
|---|---|---|---|---|---|---|---|---|---|---|---|---|---|---|---|---|---|---|---|---|---|---|---|---|---|---|---|
| | | | | | | | | t-test | Cohen's | PLS-DA | t-test | Cohen's | PLS-DA | t-test | Cohen's | PLS-DA | t-test | Cohen's | PLS-DA | | | | | | | | |
| Feature_ID | RP_HILIC | POS_NEG | Average.Rt | Average.Mz | Metabolite.name | curated_MSMS | ID_level | p | d | VIP | p | d | VIP | p | d | VIP | p | d | VIP | Mean | SD | Mean | SD | Mean | SD | Mean | SD |
| HILIC_NEG_132_90036a2_172 | HILIC | NEG | 2.172 | 132.9004 | | | | 8.32E-01 | 0.04 | 0.68 | 4.11E-01 | -0.17 | 0.78 | 8.30E-01 | -0.05 | 0.25 | 5.41E-01 | 0.13 | 0.38 | 1937252 | 416730 | 1956242 | 506534 | 1998178 | 470408 | 1914307 | 541772 |
| RP_POS_303_23172a10_099 | RP | POS | 10.10 | 303.2317 | | | | 1.82E-01 | 0.18 | 0.36 | 4.11E-01 | -0.19 | 0.58 | 4.52E-01 | 0.09 | 0.31 | 2.12E-01 | 0.28 | 0.47 | 274350 | 336741 | 413488 | 847349 | 483749 | 1096251 | 343228 | 488686 |
| HILIC_POS_526_29309a1_766 | HILIC | POS | 1.766 | 526.2931 | | | | 9.28E-03 | 0.41 | 0.76 | 4.11E-01 | -0.18 | 0.65 | 8.56E-02 | 0.32 | 0.58 | 1.08E-02 | 0.50 | 0.92 | 1709479 | 595411 | 2097593 | 1011574 | 2181307 | 1038361 | 2013878 | 987396 |
| RP_NEG_272_79538a0_521 | RP | NEG | 0.52 | 272.7954 | | | | 2.37E-04 | 0.46 | 1.23 | 4.11E-01 | -0.18 | 1.19 | 2.00E-02 | 0.37 | 1.19 | 2.58E-03 | 0.55 | 1.36 | 588669 | 89107 | 734966 | 353086 | 764182 | 377556 | 705750 | 328015 |
| RP_POS_413_26755a11_391 | RP | POS | 11.39 | 413.2676 | | | | 3.25E-01 | -0.20 | 0.42 | 4.11E-01 | 0.17 | 0.63 | 6.28E-01 | -0.12 | 0.32 | 1.78E-01 | -0.28 | 0.57 | 17359831 | 1716024 | 17012375 | 1737848 | 16868572 | 1377687 | 17156178 | 2040011 |
| RP_POS_154_96451a0_252 | RP | POS | 0.25 | 154.9645 | | | | 4.78E-01 | 0.15 | 0.56 | 4.11E-01 | -0.17 | 0.53 | 7.69E-01 | 0.06 | 0.47 | 3.41E-01 | 0.23 | 0.45 | 5229781 | 846198 | 5352132 | 833695 | 5421065 | 933798 | 5283200 | 722914 |
| HILIC_POS_144_10196a3_375 | HILIC | POS | 3.375 | 144.1020 | | | | 2.72E-01 | 0.13 | 0.52 | 4.12E-01 | 0.19 | 1.09 | 3.29E-01 | 0.23 | 0.55 | 4.40E-01 | 0.04 | 0.26 | 2318099 | 1660782 | 3680564 | 11988581 | 2686416 | 2639895 | 4674713 | 16774960 |
| RP_POS_388_35327a12_166 | RP | POS | 12.17 | 388.3533 | | | | 6.05E-01 | -0.11 | 0.63 | 4.12E-01 | -0.16 | 0.50 | 4.04E-01 | -0.19 | 0.44 | 9.14E-01 | -0.03 | 0.40 | 2582677 | 1628830 | 2411418 | 1588491 | 2542481 | 1656686 | 2280355 | 1522642 |
| HILIC_NEG_348_00107a7_352 | HILIC | NEG | 7.352 | 348.0011 | | | | 4.77E-03 | 0.51 | 1.35 | 4.12E-01 | -0.17 | 0.78 | 4.34E-02 | 0.43 | 0.81 | 3.67E-03 | 0.60 | 1.22 | 4680394 | 120731 | 4757456 | 155221 | 4770255 | 148739 | 4744658 | 161930 |
| RP_POS_647_16553a11_289 | RP | POS | 11.29 | 647.1655 | | | | 1.20E-01 | 0.28 | 0.73 | 4.12E-01 | -0.17 | 0.59 | 3.44E-01 | 0.19 | 0.57 | 8.82E-02 | 0.36 | 0.76 | 323768 | 115834 | 364021 | 153088 | 376643 | 161037 | 351399 | 145231 |
| HILIC_NEG_315_04202a7_428 | HILIC | NEG | 7.428 | 315.0420 | | | | 4.38E-01 | 0.16 | 0.64 | 4.12E-01 | 0.16 | 0.66 | 2.78E-01 | 0.25 | 0.92 | 7.31E-01 | 0.08 | 0.43 | 2236241 | 327497 | 2287528 | 308599 | 2262084 | 336249 | 2312973 | 279330 |
| RP_POS_652_41095a5_652 | RP | POS | 5.65 | 652.4110 | | | | 5.03E-07 | 0.60 | 1.31 | 4.12E-01 | -0.18 | 1.20 | 9.41E-04 | 0.51 | 1.37 | 1.75E-04 | 0.69 | 1.41 | 102878 | 103001 | 1414059 | 2432944 | 1614644 | 2629442 | 1213475 | 2227815 |
| HILIC_POS_387_21365a4_123 | HILIC | POS | 4.123 | 387.2137 | | | | 1.71E-04 | -0.73 | 1.27 | 4.13E-01 | -0.16 | 1.16 | 2.13E-04 | -0.81 | 1.30 | 2.29E-03 | -0.65 | 1.08 | 2118595 | 403160 | 1778799 | 455753 | 1816352 | 450055 | 1741246 | 462853 |
| RP_POS_371_24298a10_241 | RP | POS | 10.24 | 371.2430 | | | | 7.78E-07 | 0.59 | 1.17 | 4.13E-01 | -0.18 | 1.05 | 1.93E-03 | 0.50 | 1.16 | 1.34E-04 | 0.68 | 1.35 | 19823 | 20367 | 480736 | 873010 | 552655 | 908606 | 408816 | 838885 |
| RP_POS_419_35239a13_194 | RP | POS | 13.19 | 419.3524 | | | | 3.81E-01 | 0.17 | 0.56 | 4.13E-01 | 0.17 | 0.48 | 2.55E-01 | 0.26 | 0.76 | 6.89E-01 | 0.09 | 0.18 | 2079618 | 375172 | 2148563 | 412597 | 2114583 | 399031 | 2182543 | 427042 |
| RP_POS_430_31641a10_241 | RP | POS | 10.24 | 430.3164 | | | | 4.43E-05 | 0.48 | 0.93 | 4.14E-01 | -0.19 | 0.85 | 1.24E-02 | 0.39 | 0.93 | 1.34E-03 | 0.57 | 1.12 | 60073 | 27101 | 423867 | 850123 | 493784 | 900742 | 353949 | 799284 |
| RP_POS_217_10449a10_574 | RP | POS | 10.57 | 217.1045 | | | | 2.85E-01 | 0.22 | 0.34 | 4.14E-01 | -0.16 | 0.62 | 5.57E-01 | 0.14 | 0.27 | 1.76E-01 | 0.30 | 0.57 | 2447967 | 854992 | 2635486 | 853881 | 2705656 | 791816 | 2565317 | 914375 |
| RP_POS_358_30698a11_37 | RP | POS | 11.37 | 358.3070 | | | | 8.11E-01 | -0.05 | 0.64 | 4.14E-01 | -0.16 | 0.49 | 5.67E-01 | -0.13 | 0.39 | 8.91E-01 | 0.03 | 0.38 | 5874941 | 4038103 | 5679290 | 3896612 | 5999423 | 3976975 | 5359157 | 3827736 |
| HILIC_POS_203_15015a6_49 | HILIC | POS | 6.49 | 203.1502 | | | | 1.17E-02 | 0.40 | 0.96 | 4.14E-01 | 0.18 | 0.91 | 1.40E-02 | 0.49 | 0.83 | 9.03E-02 | 0.32 | 0.97 | 4339940 | 1164806 | 5064532 | 1919528 | 4906879 | 1829473 | 5222185 | 2011634 |
| RP_POS_288_92123a15_367 | RP | POS | 15.37 | 288.9212 | | | | 4.45E-01 | -0.11 | 0.46 | 4.14E-01 | -0.18 | 0.58 | 2.77E-01 | -0.20 | 0.56 | 9.01E-01 | -0.02 | 0.44 | 3677011 | 119596 | 3652756 | 235742 | 3672116 | 233791 | 3633395 | 238453 |
| RP_NEG_217_95235a15_411 | RP | NEG | 15.41 | 217.9524 | | | | 1.63E-01 | -0.32 | 0.58 | 4.14E-01 | -0.15 | 0.67 | 1.06E-01 | -0.40 | 0.65 | 3.24E-01 | -0.25 | 0.40 | 3954239 | 235739 | 3889431 | 185938 | 3904698 | 193271 | 3874164 | 178947 |
| HILIC_POS_502_29175a1_798 | HILIC | POS | 1.798 | 502.2918 | | | | 1.09E-06 | 0.86 | 1.47 | 4.15E-01 | -0.16 | 0.52 | 3.54E-04 | 0.78 | 1.25 | 3.84E-07 | 0.94 | 2.05 | 2472246 | 831657 | 3464108 | 1139951 | 3557723 | 889687 | 3370492 | 1347616 |
| RP_POS_627_26117a7_407 | RP | POS | 7.41 | 627.2612 | | | | 6.08E-01 | -0.10 | 0.44 | 4.15E-01 | -0.17 | 0.71 | 4.11E-01 | -0.18 | 0.39 | 9.51E-01 | -0.01 | 0.46 | 733701 | 80747 | 724909 | 92930 | 732536 | 88266 | 717283 | 97667 |
| RP_POS_278_96878a15_333 | RP | POS | 15.33 | 278.9688 | | | | 2.65E-01 | -0.20 | 0.49 | 4.15E-01 | -0.17 | 0.49 | 1.51E-01 | -0.29 | 0.63 | 5.98E-01 | -0.11 | 0.42 | 3564740 | 156008 | 3526493 | 199809 | 3542887 | 217084 | 3510098 | 181627 |
| RP_NEG_594_37946a11_007 | RP | NEG | 11.01 | 594.3795 | | | | 2.83E-01 | -0.26 | 0.79 | 4.15E-01 | 0.15 | 1.37 | 4.81E-01 | -0.19 | 0.60 | 1.87E-01 | -0.33 | 0.89 | 3339423 | 1508839 | 3026355 | 1088258 | 2937086 | 978263 | 3115625 | 1191435 |
| RP_POS_679_4101a5_803 | RP | POS | 5.80 | 679.4101 | | | | 2.40E-07 | 0.62 | 1.31 | 4.15E-01 | -0.18 | 1.21 | 5.93E-04 | 0.52 | 1.39 | 1.32E-04 | 0.71 | 1.42 | 7439 | 5099 | 304010 | 534419 | 347838 | 579993 | 260181 | 486543 |
| RP_POS_818_56506a13_297 | RP | POS | 13.30 | 818.5651 | | | | 1.83E-02 | -0.52 | 1.18 | 4.15E-01 | -0.16 | 0.58 | 2.03E-02 | -0.59 | 1.28 | 4.38E-02 | -0.44 | 0.90 | 3616139 | 1848442 | 2722022 | 1644407 | 2856938 | 1172545 | 2587105 | 2012789 |
| RP_POS_203_13878a0_684 | RP | POS | 0.68 | 203.1388 | | | | 1.05E-04 | 0.59 | 1.00 | 4.15E-01 | 0.17 | 0.86 | 1.78E-04 | 0.68 | 1.34 | 9.72E-03 | 0.50 | 1.09 | 430713 | 319145 | 764122 | 602502 | 714760 | 643623 | 813484 | 560537 |
| RP_POS_657_36591a5_652 | RP | POS | 5.65 | 657.3659 | | | | 5.08E-07 | 0.60 | 1.31 | 4.16E-01 | -0.18 | 1.21 | 9.32E-04 | 0.51 | 1.38 | 1.79E-04 | 0.69 | 1.41 | 48269 | 62041 | 855449 | 1498507 | 978126 | 1619914 | 732772 | 1371874 |
| RP_POS_778_63098a11_673 | RP | POS | 11.67 | 778.6310 | | | | 9.72E-01 | -0.01 | 0.63 | 4.16E-01 | -0.16 | 0.50 | 6.97E-01 | -0.09 | 0.43 | 7.38E-01 | 0.08 | 0.34 | 1711107 | 776341 | 1705602 | 788285 | 1770036 | 770888 | 1641167 | 807915 |
| RP_NEG_331_95239a6_868 | RP | NEG | 6.87 | 331.9524 | | | | 1.15E-01 | -0.31 | 0.66 | 4.17E-01 | 0.17 | 1.12 | 2.87E-01 | -0.22 | 0.72 | 8.83E-02 | -0.39 | 0.81 | 1009174 | 120201 | 969047 | 133059 | 958172 | 145028 | 979922 | 120400 |
| HILIC_NEG_115_92066a6_088 | HILIC | NEG | 6.088 | 115.9207 | | | | 7.45E-01 | 0.07 | 0.55 | 4.17E-01 | -0.17 | 0.51 | 9.39E-01 | -0.02 | 0.23 | 5.15E-01 | 0.15 | 0.46 | 43726613 | 15548990 | 44767057 | 16067253 | 46078526 | 16386407 | 43455587 | 15797383 |
| RP_POS_410_76077a12_818 | RP | POS | 12.82 | 410.7608 | | | | 9.16E-07 | -1.13 | 1.82 | 4.17E-01 | -0.14 | 0.71 | 6.91E-07 | -1.20 | 1.90 | 8.30E-06 | -1.06 | 1.63 | 1930606 | 694086 | 1162637 | 563565 | 1208602 | 578402 | 1116672 | 550303 |
| RP_POS_287_62479a10_042 | RP | POS | 10.04 | 287.6248 | | | | 8.14E-02 | 0.33 | 0.83 | 4.18E-01 | -0.17 | 0.72 | 2.66E-01 | 0.25 | 0.73 | 4.58E-02 | 0.42 | 0.95 | 813292 | 591854 | 1034362 | 678147 | 1089665 | 612211 | 979060 | 740343 |
| RP_POS_151_00529a10_57 | RP | POS | 10.57 | 151.0053 | | | | 4.08E-01 | -0.18 | 0.28 | 4.18E-01 | 0.16 | 0.54 | 6.78E-01 | -0.10 | 0.31 | 2.68E-01 | -0.26 | 0.50 | 2427937 | 845722 | 2288156 | 754304 | 2226656 | 708368 | 2349656 | 800035 |
| HILIC_POS_158_11763a0_523 | HILIC | POS | 0.523 | 158.1176 | | | | 1.07E-02 | -0.75 | 1.81 | 4.18E-01 | -0.11 | 0.55 | 7.16E-03 | -0.81 | 1.29 | 2.08E-02 | -0.70 | 1.38 | 1075992 | 934789 | 616613 | 408656 | 649934 | 449928 | 583291 | 364290 |
| RP_POS_136_98964a15_443 | RP | POS | 15.44 | 136.9896 | | | | 2.67E-01 | -0.31 | 0.81 | 4.18E-01 | 0.13 | 0.65 | 3.90E-01 | -0.24 | 0.45 | 2.04E-01 | -0.37 | 0.76 | 56498037 | 15830334 | 53199800 | 8513440 | 52505817 | 9376941 | 53893783 | 7584627 |
| RP_POS_178_05885a0_511 | RP | POS | 0.51 | 178.0589 | | | | 4.25E-04 | 0.45 | 1.13 | 4.18E-01 | -0.18 | 1.14 | 1.99E-02 | 0.36 | 1.17 | 4.16E-03 | 0.54 | 1.22 | 551215 | 95271 | 683654 | 325068 | 710142 | 357629 | 657166 | 290111 |
| RP_POS_159_09187a2_894 | RP | POS | 2.89 | 159.0919 | | | | 9.77E-04 | -0.57 | 1.20 | 4.18E-01 | -0.17 | 1.14 | 2.96E-03 | -0.65 | 1.21 | 7.75E-03 | -0.48 | 1.14 | 4293800 | 715548 | 3735947 | 1019954 | 3819065 | 838578 | 3652828 | 1176660 |
| RP_POS_307_17932a5_031 | RP | POS | 5.03 | 307.1793 | | | | 1.11E-04 | -0.68 | 1.17 | 4.18E-01 | 0.17 | 0.96 | 2.68E-03 | -0.60 | 1.16 | 2.30E-04 | -0.76 | 1.28 | 732353 | 223225 | 528090 | 305814 | 503193 | 313536 | 552987 | 298970 |
| RP_POS_186_03168a6_397 | RP | POS | 6.40 | 186.0317 | | | | 2.82E-03 | 0.50 | 1.03 | 4.18E-01 | -0.17 | 0.53 | 2.84E-02 | 0.41 | 0.92 | 5.03E-03 | 0.58 | 1.04 | 1782015 | 1022357 | 2520693 | 1576030 | 2648979 | 1697810 | 2392406 | 1449927 |
| RP_POS_546_35272a11_255 | RP | POS | 11.26 | 546.3527 | | | | 1.02E-01 | 0.29 | 1.09 | 4.18E-01 | -0.17 | 0.77 | 3.46E-01 | 0.20 | 0.77 | 5.51E-02 | 0.38 | 1.15 | 743497 | 386417 | 885078 | 513572 | 926885 | 457619 | 843271 | 565608 |
| RP_POS_318_1962a5_652 | RP | POS | 5.65 | 318.1962 | | | | 4.66E-07 | 0.60 | 1.31 | 4.19E-01 | -0.18 | 1.21 | 9.21E-04 | 0.51 | 1.38 | 1.67E-04 | 0.69 | 1.41 | 31422 | 40038 | 628781 | 1105419 | 718754 | 1191599 | 538808 | 1016077 |
| RP_POS_554_46222a10_89 | RP | POS | 10.89 | 554.4622 | | | | 3.13E-02 | -0.41 | 0.84 | 4.19E-01 | 0.17 | 0.77 | 1.44E-01 | -0.32 | 0.79 | 1.82E-02 | -0.49 | 0.91 | 1278546 | 503005 | 1043257 | 590578 | 995207 | 539228 | 1091308 | 639690 |
| HILIC_POS_314_18082a1_952 | HILIC | POS | 1.952 | 314.1808 | | | | 5.93E-01 | 0.11 | 1.07 | 4.19E-01 | -0.16 | 0.78 | 8.80E-01 | 0.03 | 0.94 | 4.25E-01 | 0.19 | 1.09 | 986896 | 2800652 | 1287326 | 2587377 | 1497821 | 2827024 | 1076831 | 2333061 |
| RP_POS_820_52234a13_07 | RP | POS | 13.07 | 820.5223 | | | | 5.05E-02 | 0.45 | 0.96 | 4.19E-01 | -0.15 | 0.62 | 1.47E-01 | 0.37 | 0.69 | 2.73E-02 | 0.52 | 1.10 | 13444793 | 4231741 | 15102329 | 3475204 | 15385099 | 2896897 | 14819559 | 3980622 |
| RP_POS_349_18936a10_704 | RP | POS | 10.70 | 349.1894 | | | | 1.74E-03 | 0.58 | 1.41 | 4.19E-01 | 0.17 | 1.25 | 3.76E-03 | 0.66 | 1.36 | 9.47E-03 | 0.50 | 1.30 | 5223812 | 849553 | 5823329 | 1053397 | 5737617 | 848829 | 5909041 | 1227369 |
| RP_POS_367_33591a11_469 | RP | POS | 11.47 | 367.3359 | | | | 1.92E-01 | 0.19 | 0.41 | 4.19E-01 | 0.18 | 0.76 | 1.70E-01 | 0.28 | 0.55 | 5.42E-01 | 0.10 | 0.51 | 554999 | 402095 | 702963 | 873613 | 631906 | 730758 | 774019 | 998831 |
| HILIC_POS_389_31216a2_202 | HILIC | POS | 2.202 | 389.3122 | | | | 2.00E-01 | 0.24 | 0.60 | 4.19E-01 | 0.17 | 0.69 | 1.49E-01 | 0.32 | 0.62 | 4.51E-01 | 0.15 | 0.31 | 750926 | 380369 | 857122 | 468813 | 819010 | 421658 | 895235 | 513124 |
| RP_NEG_390_87552a15_346 | RP | NEG | 15.35 | 390.8755 | | | | 2.49E-01 | -0.17 | 0.42 | 4.19E-01 | -0.18 | 0.48 | 1.76E-01 | -0.26 | 0.55 | 6.61E-01 | -0.08 | 0.48 | 985786 | 33432 | 975166 | 70128 | 980866 | 67060 | 969465 | 73300 |
| RP_POS_854_56519a13_087 | RP | POS | 13.09 | 854.5652 | | | | 1.72E-04 | -0.77 | 1.29 | 4.19E-01 | 0.15 | 0.49 | 2.68E-03 | -0.70 | 1.12 | 9.39E-05 | -0.85 | 1.51 | 4277939 | 1715366 | 2865073 | 1736594 | 2723953 | 1514077 | 3006193 | 1939069 |
| RP_POS_211_13051a5_596 | RP | POS | 5.60 | 211.1305 | | | | 3.57E-01 | 0.12 | 0.32 | 4.19E-01 | -0.18 | 0.59 | 7.43E-01 | 0.03 | 0.91 | 3.60E-01 | 0.21 | 0.45 | 501578 | 469594 | 662289 | 1528322 | 786842 | 2104910 | 537736 | 506850 |
| RP_POS_223_0945a0_483 | RP | POS | 0.48 | 223.0945 | | | | 8.61E-05 | 0.50 | 1.10 | 4.19E-01 | -0.18 | 1.09 | 1.29E-02 | 0.41 | 0.96 | 1.13E-03 | 0.59 | 1.34 | 10239488 | 419209 | 10937623 | 1553415 | 11063782 | 1619777 | 10811464 | 1489726 |
| RP_NEG_297_24411a10_172 | RP | NEG | 10.17 | 297.2441 | | | | 3.69E-01 | -0.17 | 0.68 | 4.20E-01 | -0.17 | 0.86 | 2.30E-01 | -0.25 | 0.62 | 6.95E-01 | -0.09 | 0.66 | 7523201 | 1045103 | 7322803 | 1216722 | 7421617 | 1271790 | 7223989 | 1163469 |
| RP_NEG_830_59357a13_934 | RP | NEG | 13.93 | 830.5936 | | | | 1.88E-01 | 0.26 | 0.61 | 4.20E-01 | 0.17 | 0.53 | 1.30E-01 | 0.34 | 0.58 | 4.22E-01 | 0.17 | 0.77 | 33932084 | 36296333 | 44018880 | 40277783 | 40747963 | 38850357 | 47289798 | 41791423 |
| RP_POS_279_15961a9_524 | RP | POS | 9.52 | 279.1596 | | | | 8.96E-01 | 0.02 | 0.48 | 4.20E-01 | 0.17 | 0.58 | 6.19E-01 | 0.11 | 0.39 | 7.53E-01 | -0.06 | 0.63 | 5436020 | 890101 | 5461445 | 1145459 | 5368444 | 1027058 | 5554445 | 1256357 |
| RP_NEG_160_97664a15_333 | RP | NEG | 15.33 | 160.9766 | | | | 7.34E-01 | -0.05 | 0.47 | 4.20E-01 | 0.18 | 0.79 | 8.25E-01 | 0.03 | 0.74 | 5.33E-01 | -0.14 | 0.27 | 5167386 | 205302 | 5150513 | 335854 | 5123222 | 426979 | 5177804 | 209922 |
| RP_POS_548_50323a13_808 | RP | POS | 13.81 | 548.5032 | | | | 9.84E-02 | -0.39 | 0.83 | 4.20E-01 | -0.15 | 0.54 | 6.97E-02 | -0.46 | 0.77 | 2.06E-01 | -0.31 | 0.88 | 3616660 | 1168686 | 3236091 | 899375 | 3309031 | 867141 | 3163150 | 933524 |
| RP_NEG_400_14725a11_254 | RP | NEG | 11.25 | 400.1473 | | | | 4.36E-07 | 0.95 | 1.86 | 4.20E-01 | 0.15 | 1.17 | 2.66E-06 | 1.03 | 1.80 | 1.00E-05 | 0.88 | 1.76 | 1383508 | 874304 | 2438477 | 1050269 | 2353294 | 936526 | 2523659 | 1156180 |
| RP_POS_283_26273a10_55 | RP | POS | 10.55 | 283.2627 | | | | 1.58E-01 | -0.30 | 0.76 | 4.20E-01 | 0.16 | 0.82 | 3.48E-01 | -0.22 | 0.67 | 1.08E-01 | -0.38 | 0.72 | 3986005 | 597238 | 3813953 | 574734 | 3767351 | 583425 | 3860555 | 567947 |
| RP_POS_271_65848a10_326 | RP | POS | 10.33 | 271.6585 | | | | 1.14E-02 | -0.60 | 1.21 | 4.20E-01 | -0.15 | 0.69 | 9.26E-03 | -0.67 | 1.21 | 3.19E-02 | -0.53 | 1.11 | 1301359 | 318745 | 1139315 | 242409 | 1158966 | 222011 | 1119665 | 262006 |
| RP_POS_299_16107a9_095 | RP | POS | 9.10 | 299.1611 | | | | 8.39E-01 | 0.03 | 1.19 | 4.21E-01 | -0.17 | 0.50 | 7.77E-01 | -0.05 | 0.26 | 5.69E-01 | 0.12 | 1.73 | 2097000 | 74843 | 2100519 | 110452 | 2109465 | 122311 | 2091573 | 97595 |
| RP_POS_351_20013a5_802 | RP | POS | 5.80 | 351.2001 | | | | 3.04E-07 | 0.61 | 1.31 | 4.21E-01 | -0.18 | 1.21 | 6.86E-04 | 0.52 | 1.39 | 1.46E-04 | 0.70 | 1.41 | 23678 | 21520 | 567575 | 989323 | 647606 | 1070675 | 487545 | 904476 |
| RP_POS_696_84479a0_471 | RP | POS | 0.47 | 696.8448 | | | | 9.02E-09 | -0.77 | 1.38 | 4.21E-01 | 0.17 | 1.15 | 1.29E-04 | -0.68 | 1.40 | 1.57E-06 | -0.85 | 1.54 | 640156 | 94556 | 441604 | 276293 | 419259 | 271264 | 463949 | 282188 |
| RP_POS_553_46021a11_608 | RP | POS | 11.61 | 553.4602 | | | | 3.34E-02 | -0.43 | 0.89 | 4.22E-01 | 0.16 | 0.56 | 1.18E-01 | -0.35 | 0.68 | 2.29E-02 | -0.51 | 0.90 | 34948724 | 6605442 | 32009001 | 6696254 | 31467794 | 6594837 | 32550207 | 6819464 |
| RP_POS_315_19354a9_788 | RP | POS | 9.79 | 315.1935 | | 315.1942(100) | 4 | 6.07E-03 | -0.48 | 0.82 | 4.22E-01 | 0.17 | 0.70 | 4.84E-02 | -0.40 | 0.73 | 6.30E-03 | -0.57 | 1.06 | 11775237 | 382337 | 11533379 | 523876 | 11491087 | 532930 | 11575672 | 516555 |
| RP_POS_379_17502a7_472 | RP | POS | 7.47 | 379.1750 | | | | 5.02E-09 | 0.72 | 1.33 | 4.22E-01 | 0.18 | 1.00 | 2.35E-05 | 0.81 | 1.45 | 3.12E-05 | 0.63 | 1.31 | 147543 | 219395 | 965287 | 1234314 | 865638 | 1084750 | 1064936 | 1371571 |
| RP_POS_512_26782a10_067 | RP | POS | 10.07 | 512.2678 | | | | 3.34E-02 | 0.27 | 0.61 | 4.22E-01 | 0.18 | 0.61 | 7.25E-02 | 0.36 | 0.67 | 1.85E-01 | 0.18 | 0.48 | 398483 | 332134 | 677434 | 1157191 | 583993 | 888237 | 770874 | 1377943 |
| RP_NEG_341_26999a10_614 | RP | NEG | 10.61 | 341.2700 | | | | 1.11E-02 | -0.57 | 0.94 | 4.23E-01 | 0.15 | 0.50 | 3.87E-02 | -0.49 | 0.80 | 8.25E-03 | -0.64 | 1.06 | 989217 | 303762 | 831617 | 258074 | 810809 | 262224 | 852424 | 254791 |
| HILIC_POS_284_16989a0_779 | HILIC | POS | 0.779 | 284.1699 | | | | 9.86E-01 | 0.00 | 0.69 | 4.23E-01 | 0.16 | 0.91 | 7.03E-01 | 0.09 | 0.19 | 7.17E-01 | -0.08 | 0.70 | 1312448 | 282060 | 1313513 | 306671 | 1288805 | 293958 | 1338220 | 319932 |
| RP_POS_356_26138a8_862 | RP | POS | 8.86 | 356.2614 | | | | 4.67E-01 | -0.15 | 0.74 | 4.23E-01 | 0.16 | 0.50 | 7.83E-01 | -0.06 | 0.54 | 2.94E-01 | -0.23 | 0.60 | 673375 | 335814 | 622853 | 351516 | 594531 | 318047 | 651175 | 383221 |
| RP_POS_348_28723a9_95 | RP | POS | 9.95 | 348.2872 | | | | 4.10E-01 | -0.22 | 0.84 | 4.23E-01 | -0.13 | 0.63 | 2.94E-01 | -0.29 | 0.71 | 5.94E-01 | -0.15 | 0.46 | 2186612 | 3685317 | 1614161 | 2187161 | 1790324 | 2455824 | 1437998 | 1889594 |
| HILIC_POS_371_2402a4_905 | HILIC | POS | 4.905 | 371.2402 | | | | 6.84E-01 | -0.08 | 0.73 | 4.24E-01 | 0.16 | 0.80 | 9.99E-01 | 0.00 | 0.10 | 4.48E-01 | -0.16 | 0.52 | 16179012 | 4475135 | 15803439 | 4645359 | 15429363 | 4101445 | 16177516 | 5147007 |
| HILIC_NEG_225_06306a0_753 | HILIC | NEG | 0.753 | 225.0631 | | | | 4.56E-01 | 0.12 | 0.39 | 4.24E-01 | -0.17 | 0.48 | 8.67E-01 | 0.03 | 0.21 | 2.72E-01 | 0.21 | 0.38 | 217075 | 227964 | 257859 | 365690 | 287302 | 347021 | 228416 | 384701 |
| RP_POS_802_79736a0_496 | RP | POS | 0.50 | 802.7974 | | | | 5.80E-09 | -0.70 | 1.38 | 4.24E-01 | 0.18 | 1.21 | 1.49E-04 | -0.61 | 1.42 | 1.10E-05 | -0.79 | 1.52 | 9102383 | 302261 | 6672755 | 3785922 | 6367965 | 3938693 | 6977544 | 3640883 |
| RP_POS_187_05771a0_611 | RP | POS | 0.61 | 187.0577 | | | | 1.47E-05 | 0.60 | 1.19 | 4.24E-01 | -0.17 | 1.08 | 2.21E-03 | 0.51 | 1.21 | 3.65E-04 | 0.68 | 1.33 | 10114806 | 2764816 | 14000443 | 7092792 | 14570634 | 7643182 | 13430253 | 6523828 |
| RP_NEG_230_99678a4_547 | RP | NEG | 4.55 | 230.9968 | | | | 3.72E-01 | -0.25 | 0.54 | 4.25E-01 | -0.13 | 0.54 | 2.53E-01 | -0.31 | 0.60 | 5.44E-01 | -0.19 | 0.36 | 2228117 | 7869453 | 916326 | 4137257 | 1249654 | 5591436 | 582997 | 1758470 |
| RP_POS_249_15567a0_577 | RP | POS | 0.58 | 249.1557 | | | | 4.49E-03 | 0.45 | 0.72 | 4.25E-01 | -0.17 | 0.84 | 9.51E-02 | 0.36 | 0.65 | 1.05E-03 | 0.53 | 1.12 | 699030 | 362901 | 959680 | 623455 | 1009806 | 458691 | 909555 | 754851 |
| RP_POS_219_08257a0_505 | RP | POS | 0.51 | 219.0826 | | | | 2.13E-06 | 0.57 | 1.26 | 4.25E-01 | -0.18 | 1.18 | 1.67E-03 | 0.48 | 1.28 | 3.93E-04 | 0.66 | 1.38 | 293135 | 76827 | 720527 | 840157 | 788023 | 915900 | 653031 | 760240 |
| HILIC_POS_330_33624a0_527 | HILIC | POS | 0.527 | 330.3362 | | | | 7.72E-03 | -0.51 | 1.20 | 4.25E-01 | 0.16 | 0.63 | 4.70E-02 | -0.43 | 0.76 | 6.03E-03 | -0.59 | 1.13 | 2578483 | 618235 | 2219360 | 707723 | 2162526 | 694924 | 2276193 | 722810 |
| RP_POS_637_30396a10_563 | RP | POS | 10.56 | 637.3040 | | | | 1.37E-03 | -0.56 | 0.90 | 4.25E-01 | 0.17 | 0.53 | 3.19E-02 | -0.47 | 0.74 | 4.88E-04 | -0.64 | 1.20 | 4729034 | 312310 | 4494675 | 436813 | 4459579 | 345289 | 4529770 | 513583 |
| RP_POS_286_09607a0_709 | RP | POS | 0.71 | 286.0961 | | | | 5.21E-12 | 0.85 | 1.57 | 4.25E-01 | 0.17 | 0.63 | 2.46E-06 | 0.94 | 1.56 | 1.44E-07 | 0.76 | 1.75 | 33986 | 39078 | 261600 | 285782 | 238652 | 233825 | 284547 | 330546 |
| RP_POS_609_30902a5_216 | RP | POS | 5.22 | 609.3090 | | | | 1.16E-06 | 0.58 | 1.27 | 4.25E-01 | -0.18 | 1.15 | 1.02E-03 | 0.49 | 1.35 | 3.58E-04 | 0.67 | 1.35 | 49921 | 137935 | 1554405 | 2895771 | 1786868 | 3198497 | 1321943 | 2569327 |
| RP_POS_363_78888a0_248 | RP | POS | 0.25 | 363.7889 | | | | 9.90E-01 | 0.00 | 0.40 | 4.25E-01 | -0.15 | 0.53 | 7.61E-01 | -0.07 | 0.32 | 7.43E-01 | 0.08 | 0.46 | 733346 | 90174 | 733577 | 76131 | 739687 | 76549 | 727468 | 75989 |
| RP_POS_281_24738a10_978 | RP | POS | 10.98 | 281.2474 | | | | 6.87E-03 | 0.45 | 1.37 | 4.25E-01 | -0.17 | 1.31 | 6.37E-02 | 0.36 | 1.11 | 7.44E-03 | 0.53 | 1.35 | 2913320 | 1288584 | 3752262 | 1989551 | 3911869 | 2003967 | 3592655 | 1982305 |
| RP_NEG_212_91109a15_628 | RP | NEG | 15.63 | 212.9111 | | | | 3.26E-02 | -0.45 | 0.99 | 4.26E-01 | -0.16 | 0.61 | 2.82E-02 | -0.53 | 0.90 | 9.26E-02 | -0.37 | 0.90 | 3533319 | 146832 | 3468531 | 139643 | 3479718 | 124246 | 3457343 | 153967 |
| RP_POS_459_14725a4_851 | RP | POS | 4.85 | 459.1473 | | | | 5.68E-01 | 0.10 | 0.54 | 4.26E-01 | -0.17 | 0.56 | 9.38E-01 | 0.02 | 0.45 | 3.88E-01 | 0.19 | 0.55 | 3185617 | 1563567 | 3385611 | 2119312 | 3555288 | 2289887 | 3215933 | 1942238 |
| RP_POS_343_22437a9_959 | RP | POS | 9.96 | 343.2244 | | | | 3.85E-07 | 0.61 | 1.11 | 4.26E-01 | 0.18 | 1.14 | 2.59E-04 | 0.69 | 1.29 | 4.09E-04 | 0.52 | 1.18 | 126002 | 98539 | 1406824 | 2349748 | 1218689 | 2034373 | 1594959 | 2635224 |

**Supplementary table 1: Metabolomics results** (in asceding order according to p-value from progressive ALD vs  non-progressive ALD comparison, with molecular features with ID_level of 1-2 first)

| | | | | | | | | all_ALD_vs_control | | | Prog_ALD_vs_non-prog_ALD | | | Prog_ALD_vs_control_ALD | | | Non-prog_ALD_vs_control | | | Healthy_control | | all_ALD | | Non-prog_ALD | | Progressive_ALD | |
|---|---|---|---|---|---|---|---|---|---|---|---|---|---|---|---|---|---|---|---|---|---|---|---|---|---|---|---|
| | | | | | | | | t-test | Cohen's | PLS-DA | t-test | Cohen's | PLS-DA | t-test | Cohen's | PLS-DA | t-test | Cohen's | PLS-DA | | | | | | | | |
| Feature_ID | RP_HILIC | POS_NEG | Average.Rt | Average.Mz | Metabolite.name | curated_MSMS | ID_level | p | d | VIP | p | d | VIP | p | d | VIP | p | d | VIP | Mean | SD | Mean | SD | Mean | SD | Mean | SD |
| RP_POS_406_74686a15_468 | RP | POS | 15.47 | 406.7469 | | | | 2.25E-01 | -0.27 | 0.51 | 4.27E-01 | -0.15 | 0.55 | 1.44E-01 | -0.35 | 0.62 | 4.29E-01 | -0.20 | 0.48 | 528851 | 292735 | 458414 | 245054 | 478013 | 263741 | 438814 | 225799 |
| RP_POS_320_96732a0_54 | RP | POS | 0.54 | 320.9673 | | | | 6.75E-03 | 0.40 | 1.05 | 4.27E-01 | -0.18 | 0.93 | 8.44E-02 | 0.31 | 0.86 | 1.09E-02 | 0.48 | 1.08 | 485622 | 171043 | 615197 | 358922 | 643900 | 371312 | 586494 | 347456 |
| HILIC_NEG_137_0356a0_698 | HILIC | NEG | 0.698 | 137.0356 | | | | 2.03E-01 | -0.42 | 0.79 | 4.27E-01 | 0.08 | 0.72 | 2.59E-01 | -0.38 | 0.53 | 1.65E-01 | -0.46 | 0.76 | 291395 | 546760 | 164085 | 159951 | 151300 | 139001 | 176871 | 178985 |
| RP_NEG_291_89606a3_706 | RP | NEG | 3.71 | 291.8961 | | | | 1.43E-01 | 0.23 | 0.81 | 4.27E-01 | 0.17 | 0.78 | 1.37E-01 | 0.32 | 0.61 | 3.88E-01 | 0.15 | 0.39 | 973443 | 335971 | 1094567 | 565554 | 1049365 | 453361 | 1139768 | 660700 |
| RP_POS_262_0134a4_851 | RP | POS | 4.85 | 262.0134 | | | | 5.44E-01 | 0.11 | 0.50 | 4.27E-01 | -0.17 | 0.58 | 9.05E-01 | 0.02 | 0.39 | 3.74E-01 | 0.19 | 0.55 | 619442 | 263692 | 654830 | 345575 | 682436 | 373006 | 627224 | 317164 |
| RP_POS_696_43671a5_802 | RP | POS | 5.80 | 696.4367 | | | | 3.31E-07 | 0.61 | 1.31 | 4.27E-01 | -0.18 | 1.20 | 7.35E-04 | 0.52 | 1.38 | 1.49E-04 | 0.70 | 1.41 | 37785 | 32219 | 849197 | 1481211 | 967514 | 1598326 | 730880 | 1359977 |
| RP_POS_217_10542a2_646 | RP | POS | 2.65 | 217.1054 | | | | 2.18E-02 | 0.33 | 0.99 | 4.27E-01 | -0.18 | 0.96 | 1.42E-01 | 0.24 | 1.06 | 3.76E-02 | 0.42 | 1.02 | 3031424 | 392533 | 3292604 | 883365 | 3363150 | 991580 | 3222059 | 763677 |
| RP_POS_362_19077a5_803 | RP | POS | 5.80 | 362.1908 | | 362.1882(100) | 4 | 2.74E-07 | 0.61 | 1.32 | 4.28E-01 | -0.18 | 1.21 | 6.91E-04 | 0.52 | 1.39 | 1.31E-04 | 0.70 | 1.42 | 57988 | 36804 | 1070222 | 1833186 | 1216567 | 1972625 | 923877 | 1689606 |
| RP_POS_415_15228a10_047 | RP | POS | 10.05 | 415.1523 | | | | 6.80E-01 | -0.11 | 0.97 | 4.28E-01 | 0.13 | 0.69 | 8.89E-01 | -0.04 | 0.66 | 4.93E-01 | -0.18 | 0.71 | 1027246 | 2109955 | 863638 | 1265204 | 762424 | 582051 | 964853 | 1695423 |
| RP_POS_521_17102a8_801 | RP | POS | 8.80 | 521.1710 | | | | 6.49E-01 | 0.08 | 0.53 | 4.28E-01 | 0.17 | 1.06 | 4.53E-01 | 0.17 | 0.40 | 9.92E-01 | 0.00 | 0.59 | 530683 | 417700 | 572465 | 536670 | 529650 | 470877 | 615279 | 597027 |
| RP_POS_359_71323a5_802 | RP | POS | 5.80 | 359.7132 | | | | 2.49E-07 | 0.61 | 1.32 | 4.28E-01 | -0.18 | 1.21 | 6.45E-04 | 0.53 | 1.39 | 1.27E-04 | 0.70 | 1.42 | 122139 | 67732 | 1922799 | 3247945 | 2181872 | 3498668 | 1663726 | 2989301 |
| RP_NEG_227_90456a1_2 | RP | NEG | 1.20 | 227.9046 | | | | 6.53E-10 | 0.86 | 1.40 | 4.28E-01 | 0.17 | 0.48 | 4.40E-06 | 0.94 | 1.47 | 3.55E-07 | 0.78 | 1.57 | 1257487 | 637342 | 2555823 | 1575633 | 2430201 | 1256241 | 2681445 | 1845416 |
| HILIC_POS_176_06584a4_005 | HILIC | POS | 4.005 | 176.0658 | | | | 6.06E-01 | 0.10 | 0.86 | 4.29E-01 | -0.17 | 0.99 | 9.54E-01 | 0.01 | 0.37 | 3.78E-01 | 0.18 | 0.49 | 1293195 | 354642 | 1332572 | 427953 | 1366658 | 382557 | 1298486 | 470430 |
| RP_NEG_584_80707a0_5 | RP | NEG | 0.50 | 584.8071 | | | | 7.41E-02 | -0.25 | 1.04 | 4.29E-01 | -0.18 | 1.26 | 3.67E-02 | -0.34 | 1.06 | 4.08E-01 | -0.16 | 1.12 | 1093179 | 280308 | 945643 | 651892 | 997562 | 732062 | 893725 | 563190 |
| RP_POS_570_3551a10_543 | RP | POS | 10.54 | 570.3551 | | | | 1.58E-06 | 0.74 | 1.37 | 4.29E-01 | -0.17 | 0.66 | 1.31E-03 | 0.66 | 1.20 | 1.33E-06 | 0.83 | 1.62 | 2348124 | 874428 | 3481075 | 1586321 | 3607388 | 1305210 | 3354762 | 1829767 |
| HILIC_NEG_112_45235a4_235 | HILIC | NEG | 4.235 | 112.4524 | | | | 6.33E-01 | 0.08 | 0.17 | 4.29E-01 | 0.17 | 0.53 | 4.19E-01 | 0.17 | 0.28 | 9.77E-01 | -0.01 | 0.22 | 195485 | 94159 | 205934 | 140337 | 194770 | 133965 | 217097 | 146936 |
| RP_NEG_876_5777a13_086 | RP | NEG | 13.09 | 876.5777 | | | | 4.98E-03 | -0.56 | 0.97 | 4.30E-01 | 0.16 | 0.56 | 3.93E-02 | -0.49 | 0.75 | 2.19E-03 | -0.64 | 1.30 | 3171060 | 1176866 | 2462746 | 1236157 | 2364472 | 1000675 | 2561021 | 1437461 |
| RP_POS_528_30829a10_353 | RP | POS | 10.35 | 528.3083 | | | | 1.72E-14 | 1.06 | 1.83 | 4.30E-01 | 0.16 | 0.58 | 1.84E-08 | 1.14 | 1.82 | 2.35E-10 | 0.98 | 1.92 | 272476 | 135331 | 632490 | 337662 | 605660 | 274672 | 659321 | 391719 |
| RP_POS_495_29913a11_442 | RP | POS | 11.44 | 495.2991 | | | | 5.00E-01 | -0.11 | 0.49 | 4.30E-01 | 0.17 | 0.58 | 9.14E-01 | -0.02 | 0.34 | 2.59E-01 | -0.20 | 0.51 | 700343 | 95427 | 684944 | 152478 | 672836 | 122784 | 697052 | 177760 |
| RP_POS_340_20917a5_802 | RP | POS | 5.80 | 340.2092 | | | | 3.31E-07 | 0.61 | 1.31 | 4.31E-01 | -0.18 | 1.20 | 7.55E-04 | 0.52 | 1.38 | 1.46E-04 | 0.70 | 1.41 | 62551 | 36430 | 1083096 | 1862977 | 1230812 | 2004881 | 935379 | 1717009 |
| RP_POS_318_11911a0_708 | RP | POS | 0.71 | 318.1191 | | | | 5.22E-12 | 0.84 | 1.52 | 4.31E-01 | 0.17 | 0.94 | 5.53E-06 | 0.93 | 1.59 | 2.27E-08 | 0.76 | 1.76 | 245805 | 193042 | 2064494 | 2306718 | 1881544 | 1733273 | 2247443 | 2770910 |
| RP_POS_173_0784a10_278 | RP | POS | 10.28 | 173.0784 | | | | 1.16E-01 | -0.38 | 0.72 | 4.31E-01 | -0.14 | 0.51 | 7.28E-02 | -0.45 | 0.78 | 2.41E-01 | -0.31 | 0.49 | 2152454 | 936419 | 1864709 | 685118 | 1918997 | 757737 | 1810421 | 606771 |
| RP_NEG_860_84863a0_485 | RP | NEG | 0.49 | 860.8486 | | | | 5.24E-08 | 0.65 | 1.34 | 4.31E-01 | -0.17 | 1.21 | 2.84E-04 | 0.56 | 1.38 | 5.84E-05 | 0.74 | 1.47 | 648428 | 195092 | 4576199 | 6658434 | 5103451 | 7157275 | 4048947 | 6146574 |
| RP_POS_355_31992a10_845 | RP | POS | 10.85 | 355.3199 | | | | 1.51E-03 | -0.67 | 1.17 | 4.31E-01 | 0.15 | 0.86 | 8.12E-03 | -0.60 | 1.08 | 1.45E-03 | -0.75 | 1.23 | 388247 | 187848 | 261782 | 177668 | 247716 | 184647 | 275847 | 171107 |
| RP_POS_401_26208a6_067 | RP | POS | 6.07 | 401.2621 | | | | 1.32E-07 | 0.63 | 1.33 | 4.31E-01 | -0.18 | 1.20 | 4.56E-04 | 0.54 | 1.40 | 9.40E-05 | 0.72 | 1.43 | 50680 | 23815 | 916367 | 1522063 | 1036835 | 1638694 | 795900 | 1402032 |
| HILIC_POS_217_08221a6_558 | HILIC | POS | 6.558 | 217.0822 | | | | 1.09E-05 | -0.63 | 1.22 | 4.32E-01 | 0.17 | 0.78 | 7.95E-04 | -0.55 | 1.30 | 3.49E-04 | -0.72 | 1.30 | 16670473 | 716484 | 15737996 | 1581951 | 15612820 | 1775921 | 15863172 | 1367604 |
| RP_POS_357_23578a5_802 | RP | POS | 5.80 | 357.2358 | | | | 2.56E-07 | 0.61 | 1.32 | 4.32E-01 | -0.18 | 1.20 | 6.33E-04 | 0.53 | 1.39 | 1.33E-04 | 0.70 | 1.42 | 59687 | 37544 | 1047017 | 1783149 | 1188063 | 1923363 | 905970 | 1638354 |
| RP_POS_878_77179a0_495 | RP | POS | 0.50 | 878.7718 | | | | 7.74E-09 | -0.72 | 1.36 | 4.32E-01 | 0.17 | 1.17 | 1.45E-04 | -0.64 | 1.40 | 6.63E-06 | -0.81 | 1.50 | 1503989 | 106843 | 1182227 | 482976 | 1144034 | 494160 | 1220420 | 473415 |
| RP_POS_256_17572a2_88 | RP | POS | 2.88 | 256.1757 | | | | 3.97E-02 | 0.32 | 1.13 | 4.32E-01 | -0.17 | 1.09 | 1.83E-01 | 0.24 | 1.15 | 4.46E-02 | 0.41 | 1.11 | 342573 | 418543 | 557684 | 717838 | 614452 | 783156 | 500916 | 648990 |
| RP_POS_253_21599a10_12 | RP | POS | 10.12 | 253.2160 | | | | 1.38E-02 | -0.46 | 1.05 | 4.32E-01 | -0.16 | 0.87 | 8.32E-03 | -0.54 | 1.08 | 8.33E-02 | -0.38 | 0.91 | 1057294 | 104825 | 1000681 | 124514 | 1010526 | 135494 | 990836 | 112994 |
| RP_POS_701_39203a5_802 | RP | POS | 5.80 | 701.3920 | | | | 3.44E-07 | 0.61 | 1.31 | 4.32E-01 | -0.18 | 1.20 | 7.60E-04 | 0.52 | 1.38 | 1.51E-04 | 0.69 | 1.41 | 16912 | 16432 | 528324 | 935215 | 602215 | 1007096 | 454433 | 861231 |
| RP_POS_722_52863a13_57 | RP | POS | 13.57 | 722.5286 | | | | 6.77E-01 | 0.08 | 0.58 | 4.32E-01 | 0.16 | 0.72 | 4.62E-01 | 0.16 | 0.45 | 9.85E-01 | 0.00 | 0.59 | 115565539 | 6644884 | 116156608 | 7860387 | 115535662 | 7652134 | 116777553 | 8092605 |
| RP_POS_343_33734a0_645 | RP | POS | 0.65 | 343.3373 | | | | 1.80E-02 | 0.39 | 0.66 | 4.32E-01 | 0.17 | 0.56 | 2.19E-02 | 0.48 | 1.02 | 9.73E-02 | 0.31 | 0.61 | 1325969 | 415661 | 1559653 | 629629 | 1509920 | 575278 | 1609385 | 681881 |
| RP_POS_900_57135a13_874 | RP | POS | 13.87 | 900.5714 | | | | 5.66E-01 | -0.12 | 0.50 | 4.33E-01 | -0.16 | 0.50 | 4.12E-01 | -0.20 | 0.79 | 8.45E-01 | -0.04 | 0.34 | 4951605 | 1280559 | 4803260 | 1204316 | 4898266 | 1065532 | 4708255 | 1332882 |
| RP_POS_831_56866a13_209 | RP | POS | 13.21 | 831.5687 | | | | 1.03E-03 | 0.62 | 1.10 | 4.33E-01 | 0.16 | 0.83 | 2.48E-03 | 0.70 | 1.10 | 5.21E-03 | 0.54 | 1.13 | 14229130 | 2595757 | 16133328 | 3076765 | 15890587 | 2438190 | 16376069 | 3614020 |
| RP_POS_531_21985a9_327 | RP | POS | 9.33 | 531.2199 | | | | 1.19E-03 | -0.86 | 1.43 | 4.34E-01 | -0.12 | 0.56 | 6.75E-04 | -0.92 | 1.51 | 4.01E-03 | -0.80 | 1.14 | 682866 | 621129 | 277546 | 364601 | 306273 | 418032 | 248819 | 303554 |
| HILIC_POS_146_08121a0_757 | HILIC | POS | 0.757 | 146.0812 | | | | 3.66E-02 | 0.30 | 0.66 | 4.34E-01 | 0.17 | 0.93 | 8.58E-02 | 0.38 | 0.59 | 9.83E-02 | 0.21 | 1.01 | 3143512 | 646919 | 3537018 | 1468708 | 3421140 | 850222 | 3652896 | 1899460 |
| RP_POS_146_06017a2_893 | RP | POS | 2.89 | 146.0602 | fragment of tryptophan | 118.0653(100), 146.0602(70) | | 9.05E-04 | -0.56 | 1.20 | 4.34E-01 | -0.16 | 1.06 | 2.99E-03 | -0.65 | 1.21 | 7.53E-03 | -0.48 | 1.14 | 3813068 | 629651 | 3313821 | 922995 | 3386534 | 765530 | 3241107 | 1060377 |
| RP_POS_487_35938a13_1 | RP | POS | 13.10 | 487.3594 | | | | 2.58E-01 | -0.23 | 1.02 | 4.34E-01 | -0.16 | 0.76 | 1.98E-01 | -0.31 | 0.92 | 4.85E-01 | -0.15 | 0.47 | 3466741 | 644682 | 3315133 | 678400 | 3368565 | 572683 | 3261700 | 772045 |
| HILIC_NEG_183_93423a2_172 | HILIC | NEG | 2.172 | 183.9342 | | | | 6.33E-01 | 0.09 | 0.74 | 4.34E-01 | -0.16 | 1.00 | 9.74E-01 | 0.01 | 0.31 | 4.11E-01 | 0.17 | 0.30 | 1584656 | 337330 | 1619173 | 402927 | 1650864 | 377644 | 1587481 | 428193 |
| RP_POS_512_41718a10_466 | RP | POS | 10.47 | 512.4172 | | | | 2.35E-02 | -0.41 | 0.74 | 4.35E-01 | 0.16 | 0.55 | 1.10E-01 | -0.33 | 0.68 | 2.06E-02 | -0.49 | 0.82 | 6712401 | 1065194 | 6172413 | 1370912 | 6064640 | 1408727 | 6280185 | 1337512 |
| RP_POS_862_75934a11_249 | RP | POS | 11.25 | 862.7593 | | | | 5.47E-07 | 0.90 | 1.43 | 4.35E-01 | -0.15 | 1.12 | 4.19E-05 | 0.82 | 1.39 | 2.14E-06 | 0.98 | 1.57 | 539317 | 216052 | 803063 | 286316 | 825569 | 288645 | 780557 | 285092 |
| HILIC_POS_330_26425a0_677 | HILIC | POS | 0.677 | 330.2643 | | | | 1.61E-01 | -0.31 | 0.84 | 4.35E-01 | -0.15 | 0.51 | 1.11E-01 | -0.38 | 0.74 | 3.31E-01 | -0.23 | 0.78 | 1180520 | 704051 | 982744 | 618366 | 1031309 | 618154 | 934179 | 620990 |
| RP_POS_201_11195a0_655 | RP | POS | 0.66 | 201.1120 | | | | 6.94E-01 | -0.08 | 0.65 | 4.35E-01 | -0.16 | 0.60 | 4.72E-01 | -0.16 | 0.48 | 9.90E-01 | 0.00 | 0.54 | 5116814 | 2158714 | 4944104 | 2119267 | 5110483 | 2301974 | 4777724 | 1928385 |
| HILIC_POS_120_08116a3_927 | HILIC | POS | 3.927 | 120.0812 | | | | 2.20E-02 | 0.34 | 0.85 | 4.35E-01 | 0.17 | 0.86 | 4.64E-02 | 0.43 | 0.83 | 9.34E-02 | 0.26 | 0.75 | 19270995 | 4758082 | 22192173 | 9330102 | 21459226 | 6901939 | 22925119 | 11275861 |
| RP_POS_302_64917a10_508 | RP | POS | 10.51 | 302.6492 | | | | 3.23E-01 | -0.22 | 0.47 | 4.36E-01 | 0.15 | 0.81 | 5.70E-01 | -0.14 | 0.35 | 2.06E-01 | -0.29 | 0.49 | 1777343 | 563829 | 1666262 | 495137 | 1627415 | 433739 | 1705110 | 551466 |
| RP_NEG_806_78931a0_497 | RP | NEG | 0.50 | 806.7893 | | | | 5.70E-03 | -0.42 | 1.13 | 4.36E-01 | -0.17 | 1.24 | 7.14E-03 | -0.50 | 1.17 | 7.89E-02 | -0.33 | 1.15 | 1245076 | 224160 | 1078812 | 435668 | 1112975 | 443412 | 1044649 | 429523 |
| RP_POS_149_9973a15_526 | RP | POS | 15.53 | 149.9973 | | | | 1.36E-01 | -0.21 | 0.43 | 4.36E-01 | -0.18 | 0.92 | 1.99E-01 | -0.30 | 0.43 | 2.59E-01 | -0.12 | 0.56 | 13467734 | 427905 | 13279173 | 1001367 | 13357867 | 423746 | 13200479 | 1354156 |
| HILIC_POS_59_06128a1_435 | HILIC | POS | 1.435 | 59.0613 | | | | 5.42E-01 | -0.15 | 0.33 | 4.36E-01 | 0.14 | 0.65 | 7.61E-01 | -0.08 | 0.15 | 3.97E-01 | -0.22 | 0.35 | 3094350 | 1683639 | 2897416 | 1174535 | 2805446 | 1136145 | 2989386 | 1216195 |
| RP_POS_736_85577a0_492 | RP | POS | 0.49 | 736.8558 | | | | 4.56E-08 | 0.65 | 1.33 | 4.36E-01 | -0.17 | 1.19 | 3.44E-04 | 0.57 | 1.36 | 4.33E-05 | 0.74 | 1.47 | 47203 | 32873 | 2756798 | 4573230 | 3114847 | 4830395 | 2398749 | 4319879 |
| RP_POS_596_32703a5_034 | RP | POS | 5.03 | 596.3270 | | | | 6.25E-05 | -0.70 | 1.20 | 4.37E-01 | 0.16 | 0.97 | 1.79E-03 | -0.62 | 1.19 | 1.50E-04 | -0.78 | 1.29 | 1037668 | 305983 | 745227 | 425005 | 711972 | 433426 | 778483 | 418126 |
| RP_POS_328_23022a8_387 | RP | POS | 8.39 | 328.2302 | | | | 3.42E-01 | 0.17 | 0.92 | 4.37E-01 | 0.16 | 0.70 | 2.53E-01 | 0.25 | 0.79 | 6.53E-01 | 0.09 | 0.62 | 429070 | 209130 | 473052 | 272875 | 451701 | 238980 | 494403 | 303971 |
| HILIC_POS_216_1707a0_549 | HILIC | POS | 0.549 | 216.1707 | | | | 1.89E-01 | 0.16 | 0.71 | 4.37E-01 | 0.18 | 0.64 | 2.01E-01 | 0.25 | 0.68 | 6.26E-01 | 0.07 | 0.42 | 42336 | 44228 | 84114 | 306595 | 60128 | 250667 | 108100 | 354839 |
| RP_POS_468_29572a10_167 | RP | POS | 10.17 | 468.2957 | | | | 2.12E-01 | 0.22 | 0.57 | 4.37E-01 | -0.17 | 0.48 | 4.72E-01 | 0.13 | 0.38 | 1.76E-01 | 0.30 | 0.58 | 766881 | 595169 | 936342 | 837292 | 1001825 | 962489 | 870859 | 693750 |
| RP_NEG_979_1394a9_433 | RP | NEG | 9.43 | 979.1394 | | | | 3.15E-01 | -0.18 | 0.43 | 4.37E-01 | -0.16 | 0.53 | 1.92E-01 | -0.26 | 0.52 | 6.42E-01 | -0.10 | 0.30 | 2109212 | 179131 | 2069834 | 225434 | 2087446 | 242216 | 2052222 | 208271 |
| RP_NEG_642_39667a10_978 | RP | NEG | 10.98 | 642.3967 | | | | 3.97E-02 | 0.38 | 1.30 | 4.38E-01 | -0.16 | 1.29 | 1.54E-01 | 0.30 | 1.05 | 2.97E-02 | 0.46 | 1.24 | 550387 | 387832 | 725064 | 470342 | 761802 | 468918 | 688326 | 473622 |
| RP_POS_245_22583a10_592 | RP | POS | 10.59 | 245.2258 | | | | 1.61E-03 | 0.54 | 1.09 | 4.38E-01 | -0.16 | 0.50 | 2.41E-02 | 0.46 | 0.84 | 1.74E-03 | 0.62 | 1.33 | 2001641 | 982174 | 2735539 | 1420262 | 2846402 | 1374178 | 2624677 | 1470377 |
| HILIC_POS_242_1306a0_571 | HILIC | POS | 0.571 | 242.1306 | | | | 6.56E-01 | 0.06 | 0.20 | 4.38E-01 | -0.18 | 0.57 | 5.54E-01 | -0.03 | 0.24 | 5.35E-01 | 0.14 | 0.25 | 3821115 | 5819860 | 5013616 | 24628067 | 6942772 | 34558464 | 3084459 | 4855836 |
| HILIC_POS_298_18573a2_143 | HILIC | POS | 2.143 | 298.1857 | | | | 1.29E-01 | 0.24 | 1.07 | 4.38E-01 | -0.17 | 0.77 | 3.79E-01 | 0.16 | 1.09 | 1.14E-01 | 0.33 | 1.16 | 529810 | 1102347 | 940712 | 1837358 | 1084049 | 2025524 | 797375 | 1635844 |
| RP_POS_477_10349a0_714 | RP | POS | 0.71 | 477.1035 | | | | 3.45E-07 | -0.75 | 1.35 | 4.38E-01 | -0.16 | 0.97 | 5.74E-06 | -0.83 | 1.54 | 3.23E-04 | -0.67 | 1.16 | 734475 | 109415 | 572735 | 226679 | 590407 | 232939 | 555063 | 221172 |
| RP_POS_327_20135a3_84 | RP | POS | 3.84 | 327.2014 | | | | 9.79E-01 | -0.01 | 1.17 | 4.38E-01 | -0.12 | 1.12 | 8.13E-01 | -0.07 | 0.92 | 8.56E-01 | 0.05 | 0.97 | 848505 | 2697793 | 835269 | 1390128 | 943643 | 1491603 | 726896 | 1286662 |
| RP_NEG_329_95615a6_867 | RP | NEG | 6.87 | 329.9562 | | | | 7.08E-01 | -0.07 | 0.54 | 4.38E-01 | -0.16 | 0.53 | 4.78E-01 | -0.15 | 0.49 | 9.83E-01 | 0.00 | 0.26 | 1629969 | 180440 | 1615955 | 191811 | 1630907 | 206239 | 1601002 | 177039 |
| HILIC_NEG_125_92897a2_904 | HILIC | NEG | 2.904 | 125.9290 | | | | 8.66E-01 | 0.03 | 0.83 | 4.39E-01 | 0.16 | 0.73 | 6.17E-01 | 0.11 | 0.54 | 8.17E-01 | -0.05 | 0.56 | 353987 | 123609 | 358415 | 141751 | 347367 | 129808 | 369463 | 153289 |
| HILIC_POS_267_14407a0_779 | HILIC | POS | 0.779 | 267.1441 | | | | 9.27E-01 | 0.02 | 0.68 | 4.39E-01 | 0.16 | 0.98 | 6.62E-01 | 0.10 | 0.21 | 7.76E-01 | -0.06 | 0.71 | 707310 | 153860 | 710255 | 169110 | 697092 | 164707 | 723418 | 174063 |
| HILIC_POS_171_18555a7_963 | HILIC | POS | 7.963 | 171.1856 | | | | 3.03E-01 | -0.20 | 0.94 | 4.39E-01 | 0.16 | 0.71 | 5.92E-01 | -0.12 | 0.86 | 1.96E-01 | -0.28 | 0.93 | 12442063 | 5532206 | 11236390 | 6239030 | 10750922 | 6020157 | 11721858 | 6474728 |
| RP_NEG_407_28094a9_323 | RP | NEG | 9.32 | 407.2809 | | | | 2.28E-01 | -0.35 | 0.67 | 4.39E-01 | 0.12 | 0.52 | 3.33E-01 | -0.29 | 0.48 | 1.71E-01 | -0.40 | 0.59 | 1511360 | 2881898 | 863929 | 1387538 | 755987 | 1301040 | 971871 | 1474222 |
| RP_POS_344_22815a3_84 | RP | POS | 3.84 | 344.2282 | | | | 9.92E-01 | 0.00 | 1.16 | 4.39E-01 | -0.12 | 1.12 | 8.26E-01 | -0.06 | 0.92 | 8.44E-01 | 0.06 | 0.97 | 2240728 | 6960080 | 2228276 | 3587470 | 2507367 | 3859707 | 1949185 | 3308509 |
| RP_NEG_400_90952a0_497 | RP | NEG | 0.50 | 400.9095 | | | | 9.32E-09 | -0.70 | 1.37 | 4.40E-01 | 0.17 | 1.21 | 1.31E-04 | -0.61 | 1.41 | 1.74E-05 | -0.78 | 1.49 | 23696054 | 885846 | 19276695 | 6925414 | 18738368 | 7312959 | 19815022 | 6544519 |
| RP_POS_151_00688a10_441 | RP | POS | 10.44 | 151.0069 | | | | 9.83E-01 | 0.00 | 0.20 | 4.40E-01 | -0.16 | 0.91 | 7.10E-01 | -0.08 | 0.46 | 7.41E-01 | 0.07 | 0.39 | 2493847 | 964558 | 2489509 | 1012083 | 2568124 | 1021827 | 2410895 | 1006372 |
| RP_POS_523_42706a10_932 | RP | POS | 10.93 | 523.4271 | | | | 1.30E-03 | 0.58 | 1.27 | 4.40E-01 | -0.16 | 0.82 | 7.51E-03 | 0.50 | 1.16 | 3.40E-03 | 0.66 | 1.24 | 1816740 | 402154 | 2112442 | 520162 | 2152863 | 605678 | 2072022 | 420094 |
| RP_NEG_369_17471a9_317 | RP | NEG | 9.32 | 369.1747 | | | | 6.22E-01 | 0.09 | 0.42 | 4.40E-01 | -0.16 | 0.73 | 9.48E-01 | 0.01 | 0.24 | 4.01E-01 | 0.17 | 0.52 | 21903621 | 20525151 | 24041686 | 23231594 | 25846267 | 20716994 | 22237104 | 25584929 |
| RP_POS_300_63419a10_303 | RP | POS | 10.30 | 300.6342 | | | | 3.50E-03 | 0.62 | 1.07 | 4.40E-01 | -0.15 | 0.75 | 2.11E-02 | 0.55 | 0.88 | 2.06E-03 | 0.70 | 1.16 | 3035176 | 821832 | 3539181 | 767773 | 3598815 | 684447 | 3479546 | 845728 |
| RP_POS_311_21945a15_428 | RP | POS | 15.43 | 311.2195 | | | | 3.39E-01 | 0.21 | 0.44 | 4.40E-01 | -0.15 | 0.55 | 5.48E-01 | 0.13 | 0.40 | 2.65E-01 | 0.28 | 0.49 | 4081353 | 1707244 | 4407108 | 1512634 | 4524653 | 1796823 | 4289564 | 1168779 |
| HILIC_POS_146_11758a0_54 | HILIC | POS | 0.54 | 146.1176 | | | | 7.03E-04 | 0.40 | 0.80 | 4.40E-01 | 0.18 | 0.73 | 1.37E-02 | 0.49 | 0.83 | 1.56E-02 | 0.31 | 1.00 | 126090 | 231931 | 1042774 | 2592072 | 841424 | 2000952 | 1244124 | 3080300 |
| RP_NEG_397_16895a11_254 | RP | NEG | 11.25 | 397.1690 | | | | 5.24E-06 | 0.94 | 1.73 | 4.41E-01 | 0.14 | 0.92 | 1.00E-05 | 1.01 | 1.73 | 6.28E-05 | 0.86 | 1.60 | 2459205 | 762682 | 3247609 | 777900 | 3187296 | 731897 | 3307922 | 824324 |
| RP_NEG_449_25485a10_207 | RP | NEG | 10.21 | 449.2549 | | | | 1.46E-01 | 0.25 | 0.60 | 4.41E-01 | -0.17 | 0.49 | 3.71E-01 | 0.16 | 0.41 | 1.31E-01 | 0.33 | 0.61 | 8069828 | 5812790 | 10033347 | 8555557 | 10696624 | 9765294 | 9370070 | 7185411 |
| RP_POS_235_08502a15_34 | RP | POS | 15.34 | 235.0850 | | | | 4.97E-01 | 0.11 | 0.50 | 4.41E-01 | -0.17 | 0.54 | 8.98E-01 | 0.03 | 0.24 | 2.34E-01 | 0.20 | 0.78 | 11979427 | 681640 | 12088015 | 1037542 | 12168505 | 717632 | 12007526 | 1283253 |
| RP_POS_380_33536a10_442 | RP | POS | 10.44 | 380.3354 | | | | 6.31E-02 | -0.37 | 0.73 | 4.42E-01 | 0.16 | 0.54 | 1.91E-01 | -0.29 | 0.58 | 4.55E-02 | -0.44 | 0.80 | 2378559 | 184206 | 2306094 | 200072 | 2290604 | 200676 | 2321584 | 200285 |
| HILIC_POS_270_15451a2_143 | HILIC | POS | 2.143 | 270.1545 | | | | 1.27E-01 | 0.24 | 1.07 | 4.42E-01 | -0.17 | 0.76 | 3.79E-01 | 0.16 | 1.08 | 1.14E-01 | 0.33 | 1.16 | 334050 | 725220 | 607937 | 1234040 | 703493 | 1359108 | 512381 | 1100445 |
| RP_POS_295_22433a9_732 | RP | POS | 9.73 | 295.2243 | | | | 5.99E-05 | 0.56 | 1.23 | 4.42E-01 | 0.17 | 1.00 | 2.87E-04 | 0.64 | 1.35 | 9.32E-03 | 0.48 | 1.31 | 2743421 | 337578 | 3164977 | 819495 | 3101536 | 847595 | 3228419 | 793863 |
| RP_POS_360_32272a11_668 | RP | POS | 11.67 | 360.3227 | | | | 9.88E-01 | 0.00 | 0.67 | 4.42E-01 | -0.16 | 0.45 | 7.38E-01 | -0.08 | 0.40 | 7.22E-01 | 0.08 | 0.43 | 167119332 | 90839775 | 167391291 | 93145444 | 174601691 | 95184903 | 160180891 | 91449127 |
| RP_POS_604_35376a5_214 | RP | POS | 5.21 | 604.3538 | | | | 1.27E-06 | 0.58 | 1.26 | 4.42E-01 | -0.17 | 1.14 | 1.10E-03 | 0.49 | 1.34 | 3.71E-04 | 0.66 | 1.35 | 60212 | 142813 | 1630994 | 3036159 | 1866005 | 3335282 | 1395984 | 2718057 |
| RP_POS_210_10927a11_237 | RP | POS | 11.24 | 210.1093 | | | | 2.31E-03 | 0.62 | 1.47 | 4.42E-01 | 0.15 | 1.06 | 3.11E-03 | 0.70 | 1.37 | 1.04E-02 | 0.55 | 1.46 | 1211059 | 376221 | 1456977 | 384870 | 1427190 | 335532 | 1486764 | 429976 |
| RP_POS_441_29498a13_284 | RP | POS | 13.28 | 441.2950 | | | | 6.06E-03 | 0.47 | 0.96 | 4.42E-01 | -0.16 | 0.54 | 3.81E-02 | 0.39 | 0.97 | 9.79E-03 | 0.55 | 0.90 | 16740766 | 5414424 | 20213941 | 7716251 | 20811034 | 8512736 | 19616849 | 6863084 |
| RP_NEG_227_90456a1_708 | RP | NEG | 1.71 | 227.9046 | | | | 1.82E-10 | 0.84 | 1.39 | 4.42E-01 | 0.16 | 0.48 | 5.10E-06 | 0.92 | 1.43 | 3.47E-07 | 0.76 | 1.56 | 829499 | 435834 | 1913806 | 1363546 | 1808287 | 1100568 | 2019325 | 1588234 |
| RP_POS_407_24576a11_609 | RP | POS | 11.61 | 407.2458 | | | | 1.24E-03 | -0.65 | 1.20 | 4.42E-01 | 0.15 | 0.90 | 7.09E-03 | -0.58 | 1.20 | 1.63E-03 | -0.73 | 1.12 | 5287001 | 488041 | 4945338 | 510431 | 4905868 | 552696 | 4984807 | 466632 |
| RP_POS_296_25833a8_177 | RP | POS | 8.18 | 296.2583 | Unknown | 296.2584(40), 279.2303(100), 57.0706(65) | 4 | 2.99E-01 | -0.30 | 0.87 | 4.42E-01 | -0.11 | 0.53 | 2.26E-01 | -0.36 | 0.73 | 4.20E-01 | -0.24 | 0.56 | 1928762 | 5205807 | 923300 | 2502143 | 1116739 | 2723839 | 729861 | 2270141 |
| RP_POS_1042_79785a0_491 | RP | POS | 0.49 | 1042.7979 | | | | 5.59E-08 | 0.65 | 1.33 | 4.42E-01 | -0.17 | 1.18 | 2.97E-04 | 0.56 | 1.36 | 5.99E-05 | 0.74 | 1.47 | 177520 | 95906 | 1916221 | 2954153 | 2144571 | 3165372 | 1687871 | 2739735 |

**Supplementary table 1: Metabolomics results** (in asceding order according to p-value from progressive ALD vs  non-progressive ALD comparison, with molecular features with ID_level of 1-2 first)

| | | | | | | | | all_ALD_vs_control | | | Prog_ALD_vs_non-prog_ALD | | | Prog_ALD_vs_control_ALD | | | Non-prog_ALD_vs_control | | | Healthy_control | | all_ALD | | Non-prog_ALD | | Progressive_ALD | |
|---|---|---|---|---|---|---|---|---|---|---|---|---|---|---|---|---|---|---|---|---|---|---|---|---|---|---|---|
| | | | | | | | | t-test | Cohen's | PLS-DA | t-test | Cohen's | PLS-DA | t-test | Cohen's | PLS-DA | t-test | Cohen's | PLS-DA | | | | | | | | |
| Feature_ID | RP_HILIC | POS_NEG | Average.Rt | Average.Mz | Metabolite.name | curated_MSMS | ID_level | p | d | VIP | p | d | VIP | p | d | VIP | p | d | VIP | Mean | SD | Mean | SD | Mean | SD | Mean | SD |
| RP_POS_224_0347a15_418 | RP | POS | 15.42 | 224.0347 | | | | 9.27E-01 | -0.02 | 0.94 | 4.42E-01 | -0.16 | 0.50 | 6.59E-01 | -0.10 | 0.63 | 7.97E-01 | 0.06 | 0.82 | 4611895 | 1761378 | 4578713 | 1821154 | 4719453 | 1949095 | 4437974 | 1691597 |
| RP_POS_492_44131a13_202 | RP | POS | 13.20 | 492.4413 | | | | 8.92E-01 | 0.03 | 0.69 | 4.43E-01 | 0.15 | 0.55 | 6.26E-01 | 0.11 | 0.44 | 8.39E-01 | -0.05 | 0.62 | 11299326 | 2356664 | 11364535 | 2325872 | 11184915 | 2663668 | 11544154 | 1941337 |
| RP_NEG_413_20081a8_909 | RP | NEG | 8.91 | 413.2008 | | | | 5.80E-01 | 0.11 | 0.81 | 4.43E-01 | -0.15 | 0.69 | 8.74E-01 | 0.04 | 0.56 | 4.08E-01 | 0.19 | 0.55 | 11700856 | 8773011 | 12693261 | 8778456 | 13370683 | 8981796 | 12015840 | 8607326 |
| RP_POS_339_3363a10_586 | RP | POS | 10.59 | 339.3363 | | | | 1.93E-02 | 0.53 | 0.93 | 4.43E-01 | -0.14 | 0.49 | 6.20E-02 | 0.46 | 0.73 | 1.30E-02 | 0.60 | 1.15 | 2016425 | 766396 | 2379579 | 638165 | 2428809 | 611774 | 2330349 | 666038 |
| RP_NEG_415_2081a10_979 | RP | NEG | 10.98 | 415.2081 | | | | 1.29E-02 | 0.46 | 1.46 | 4.43E-01 | -0.16 | 1.44 | 7.68E-02 | 0.39 | 1.24 | 8.76E-03 | 0.54 | 1.37 | 1396106 | 454112 | 1644876 | 547239 | 1687091 | 510512 | 1602662 | 583775 |
| HILIC_NEG_315_04208a6_094 | HILIC | NEG | 6.094 | 315.0421 | | | | 3.15E-02 | -0.42 | 0.82 | 4.43E-01 | -0.16 | 0.70 | 2.93E-02 | -0.50 | 0.82 | 1.02E-01 | -0.35 | 0.69 | 5661000 | 1432405 | 5004660 | 1562817 | 5125218 | 1415130 | 4884102 | 1703638 |
| RP_POS_217_14349a9_529 | RP | POS | 9.53 | 217.1435 | | | | 1.50E-01 | -0.32 | 0.58 | 4.44E-01 | -0.15 | 0.58 | 1.08E-01 | -0.40 | 0.64 | 2.98E-01 | -0.25 | 0.53 | 2196728 | 114323 | 2163970 | 96436 | 2171403 | 93278 | 2156537 | 99880 |
| RP_NEG_200_91951a15_4 | RP | NEG | 15.40 | 200.9195 | | | | 1.58E-01 | -0.21 | 0.42 | 4.44E-01 | 0.17 | 0.60 | 3.23E-01 | -0.12 | 0.41 | 2.07E-01 | -0.29 | 0.44 | 1139997 | 42702 | 1123264 | 90334 | 1116285 | 120151 | 1130242 | 44174 |
| RP_POS_348_7225a5_802 | RP | POS | 5.80 | 348.7225 | | | | 2.94E-07 | 0.61 | 1.31 | 4.44E-01 | -0.17 | 1.20 | 6.84E-04 | 0.53 | 1.38 | 1.43E-04 | 0.70 | 1.42 | 25662 | 20091 | 573490 | 995178 | 650172 | 1070195 | 496808 | 918505 |
| HILIC_NEG_132_92345a7_666 | HILIC | NEG | 7.666 | 132.9235 | | | | 3.84E-01 | 0.19 | 0.88 | 4.44E-01 | -0.15 | 0.48 | 6.29E-01 | 0.11 | 0.34 | 2.73E-01 | 0.26 | 0.61 | 1069669 | 192258 | 1103151 | 173025 | 1116481 | 178849 | 1089821 | 167740 |
| RP_NEG_646_42749a11_253 | RP | NEG | 11.25 | 646.4275 | | | | 7.26E-07 | 0.92 | 1.80 | 4.44E-01 | 0.15 | 1.15 | 4.83E-06 | 0.99 | 1.76 | 1.87E-05 | 0.84 | 1.70 | 2452822 | 1083124 | 3739111 | 1350598 | 3635047 | 1220950 | 3843176 | 1473986 |
| RP_POS_481_28397a11_489 | RP | POS | 11.49 | 481.2840 | | | | 5.66E-02 | -0.33 | 0.57 | 4.44E-01 | 0.16 | 0.45 | 2.41E-01 | -0.25 | 0.43 | 3.34E-02 | -0.41 | 0.79 | 526642 | 55868 | 502324 | 77504 | 496357 | 69933 | 508291 | 84693 |
| RP_POS_264_84393a0_515 | RP | POS | 0.52 | 264.8439 | | | | 3.94E-03 | -0.46 | 0.96 | 4.44E-01 | -0.16 | 1.02 | 3.84E-03 | -0.54 | 1.09 | 5.68E-02 | -0.38 | 0.92 | 568118 | 148564 | 461034 | 248843 | 480185 | 262660 | 441883 | 235309 |
| HILIC_NEG_131_08253a6_345 | HILIC | NEG | 6.345 | 131.0825 | | | | 3.62E-04 | 0.57 | 0.99 | 4.44E-01 | 0.16 | 0.80 | 9.34E-04 | 0.65 | 1.11 | 9.57E-03 | 0.49 | 0.88 | 234234 | 60974 | 289276 | 102026 | 281425 | 99928 | 297128 | 104496 |
| HILIC_POS_302_19565a1_102 | HILIC | POS | 1.102 | 302.1957 | | | | 7.49E-01 | 0.06 | 0.56 | 4.44E-01 | -0.16 | 0.77 | 9.17E-01 | -0.02 | 0.24 | 4.96E-01 | 0.14 | 0.25 | 408900 | 166005 | 420478 | 208512 | 436525 | 195991 | 404432 | 221142 |
| RP_POS_362_22229a5_94 | RP | POS | 5.94 | 362.2223 | | | | 2.23E-07 | 0.62 | 1.32 | 4.44E-01 | -0.17 | 1.20 | 5.75E-04 | 0.53 | 1.39 | 1.28E-04 | 0.70 | 1.42 | 69280 | 28292 | 1160883 | 1960790 | 1311760 | 2112011 | 1010006 | 1805771 |
| HILIC_POS_264_19559a0_581 | HILIC | POS | 0.581 | 264.1956 | | | | 2.99E-02 | 0.25 | 0.57 | 4.45E-01 | 0.18 | 0.52 | 5.66E-02 | 0.34 | 0.70 | 2.77E-01 | 0.16 | 0.51 | 14587 | 40521 | 779391 | 3470889 | 512377 | 3199247 | 1046405 | 3736218 |
| RP_POS_303_03918a4_851 | RP | POS | 4.85 | 303.0392 | | | | 8.40E-01 | 0.04 | 0.48 | 4.45E-01 | -0.16 | 0.57 | 8.34E-01 | -0.04 | 0.44 | 5.97E-01 | 0.12 | 0.51 | 1196369 | 355243 | 1211797 | 428635 | 1244771 | 466152 | 1178824 | 389472 |
| RP_NEG_412_19138a11_611 | RP | NEG | 11.61 | 412.1914 | | | | 2.04E-01 | -0.24 | 0.72 | 4.45E-01 | 0.16 | 0.46 | 4.57E-01 | -0.16 | 0.74 | 1.43E-01 | -0.32 | 0.61 | 851755 | 150532 | 810501 | 180677 | 796607 | 184765 | 824396 | 177264 |
| RP_POS_301_14099a9_524 | RP | POS | 9.52 | 301.1410 | | | | 4.51E-02 | -0.35 | 0.75 | 4.45E-01 | 0.16 | 0.72 | 2.39E-01 | -0.27 | 0.68 | 1.41E-02 | -0.43 | 0.87 | 12669261 | 1023583 | 12204709 | 1383175 | 12098229 | 960847 | 12311189 | 1708515 |
| RP_POS_247_96416a15_591 | RP | POS | 15.59 | 247.9642 | | | | 2.45E-01 | -0.22 | 0.72 | 4.45E-01 | 0.16 | 0.48 | 4.87E-01 | -0.14 | 0.43 | 1.84E-01 | -0.30 | 0.68 | 2516660 | 57447 | 2502494 | 65067 | 2497492 | 71123 | 2507495 | 58688 |
| RP_POS_209_0331a15_349 | RP | POS | 15.35 | 209.0331 | | | | 2.77E-01 | 0.20 | 0.47 | 4.45E-01 | 0.16 | 0.82 | 1.82E-01 | 0.28 | 0.51 | 5.83E-01 | 0.12 | 0.58 | 979964 | 336063 | 1060485 | 430845 | 1027381 | 439902 | 1093589 | 423430 |
| RP_POS_120_08125a2_202 | RP | POS | 2.20 | 120.0813 | | 120.0814(100), 103.0546(10) | 4 | 2.47E-01 | 0.18 | 0.69 | 4.45E-01 | 0.17 | 0.78 | 2.25E-01 | 0.26 | 0.71 | 5.46E-01 | 0.10 | 0.86 | 7565021 | 1231404 | 7922595 | 2164019 | 7756229 | 1614883 | 8088960 | 2607144 |
| RP_POS_548_50409a15_099 | RP | POS | 15.10 | 548.5041 | | | | 3.47E-03 | -0.41 | 0.85 | 4.45E-01 | -0.17 | 0.82 | 9.65E-03 | -0.50 | 1.01 | 5.22E-02 | -0.33 | 0.83 | 235363565 | 12925260 | 224169039 | 29776778 | 226456339 | 27537092 | 221881740 | 31975609 |
| RP_POS_197_1004a0_507 | RP | POS | 0.51 | 197.1004 | | | | 1.92E-05 | 0.51 | 1.21 | 4.45E-01 | -0.17 | 1.16 | 5.34E-03 | 0.43 | 1.20 | 1.08E-03 | 0.60 | 1.34 | 256403 | 71286 | 497705 | 525004 | 538011 | 567894 | 457399 | 480691 |
| RP_POS_181_0584a0_705 | RP | POS | 0.71 | 181.0584 | | | | 8.46E-02 | -0.28 | 0.52 | 4.46E-01 | -0.16 | 0.64 | 4.01E-02 | -0.37 | 0.77 | 3.46E-01 | -0.20 | 0.58 | 472384 | 99792 | 431463 | 154311 | 443311 | 177321 | 419614 | 127986 |
| HILIC_NEG_209_00409a0_579 | HILIC | NEG | 0.579 | 209.0041 | | | | 1.09E-02 | -0.60 | 1.23 | 4.46E-01 | -0.14 | 0.63 | 7.26E-03 | -0.67 | 1.12 | 3.48E-02 | -0.54 | 1.16 | 1069671 | 365107 | 882919 | 275559 | 904054 | 290728 | 861785 | 260732 |
| RP_NEG_238_93163a0_513 | RP | NEG | 0.51 | 238.9316 | | | | 3.34E-02 | 0.31 | 1.05 | 4.46E-01 | -0.17 | 1.05 | 2.22E-01 | 0.22 | 0.93 | 3.41E-02 | 0.39 | 1.18 | 1096573 | 231661 | 1236639 | 505520 | 1275378 | 508803 | 1197900 | 504355 |
| RP_POS_290_84647a0_5 | RP | POS | 0.50 | 290.8465 | | | | 9.34E-02 | -0.25 | 1.02 | 4.47E-01 | -0.17 | 1.26 | 5.71E-02 | -0.33 | 1.02 | 4.09E-01 | -0.16 | 1.08 | 1245877 | 390205 | 1065495 | 812378 | 1127725 | 879854 | 1003265 | 742522 |
| RP_POS_373_21341a5_94 | RP | POS | 5.94 | 373.2134 | | | | 1.88E-07 | 0.62 | 1.32 | 4.47E-01 | -0.17 | 1.20 | 5.28E-04 | 0.54 | 1.40 | 1.17E-04 | 0.71 | 1.42 | 19901 | 12865 | 501096 | 858239 | 566800 | 923369 | 435393 | 791681 |
| RP_POS_268_26334a10_621 | RP | POS | 10.62 | 268.2633 | | | | 1.05E-02 | 0.31 | 0.70 | 4.47E-01 | 0.17 | 0.78 | 8.44E-02 | 0.40 | 0.74 | 2.17E-04 | 0.22 | 1.32 | 1972569 | 737353 | 3026071 | 3838067 | 2731276 | 1031849 | 3320866 | 5340420 |
| RP_POS_541_87433a14_263 | RP | POS | 14.26 | 541.8743 | | | | 2.65E-02 | 0.28 | 0.61 | 4.47E-01 | 0.17 | 0.54 | 9.32E-02 | 0.36 | 0.63 | 7.48E-02 | 0.19 | 0.69 | 863594 | 428016 | 1295390 | 1768223 | 1159911 | 1027855 | 1430868 | 2285409 |
| RP_POS_299_25806a10_8 | RP | POS | 10.80 | 299.2581 | | | | 8.55E-02 | -0.31 | 0.96 | 4.47E-01 | 0.16 | 0.98 | 2.64E-01 | -0.23 | 0.82 | 6.84E-02 | -0.39 | 0.97 | 1678991 | 219328 | 1594401 | 291023 | 1572147 | 303379 | 1616656 | 279405 |
| RP_NEG_621_13031a0_621 | RP | NEG | 0.62 | 621.1303 | | | | 8.93E-03 | -0.42 | 1.04 | 4.47E-01 | -0.16 | 1.15 | 6.68E-03 | -0.50 | 1.12 | 9.84E-02 | -0.33 | 1.04 | 1740852 | 505815 | 1410052 | 855895 | 1475500 | 926832 | 1344605 | 782500 |
| RP_POS_521_25671a4_783 | RP | POS | 4.78 | 521.2567 | | | | 4.38E-06 | 0.55 | 1.25 | 4.47E-01 | -0.17 | 1.15 | 2.15E-03 | 0.47 | 1.33 | 6.01E-04 | 0.64 | 1.33 | 42061 | 152277 | 689532 | 1310134 | 789690 | 1431729 | 589375 | 1182205 |
| RP_NEG_258_05649a6_707 | RP | NEG | 6.71 | 258.0565 | | | | 2.14E-01 | -0.41 | 0.73 | 4.47E-01 | -0.08 | 0.50 | 1.77E-01 | -0.45 | 0.68 | 2.68E-01 | -0.37 | 0.57 | 931131 | 2325865 | 402564 | 678632 | 454433 | 712309 | 350695 | 646225 |
| RP_POS_319_01111a4_851 | RP | POS | 4.85 | 319.0111 | | | | 6.54E-01 | 0.08 | 0.51 | 4.48E-01 | -0.16 | 0.58 | 9.85E-01 | 0.00 | 0.41 | 4.66E-01 | 0.16 | 0.55 | 1443364 | 601001 | 1501256 | 722874 | 1556489 | 791555 | 1446023 | 650362 |
| RP_NEG_419_24155a11_61 | RP | NEG | 11.61 | 419.2416 | | | | 3.49E-02 | -0.40 | 0.77 | 4.48E-01 | -0.16 | 0.80 | 2.54E-02 | -0.48 | 0.83 | 1.38E-01 | -0.32 | 0.63 | 1809943 | 181134 | 1726995 | 212803 | 1743241 | 218670 | 1710750 | 207701 |
| RP_POS_211_09361a6_035 | RP | POS | 6.04 | 211.0936 | Sinapyl alcohol | 211.0952(100) | 3 | 2.04E-01 | 0.21 | 0.73 | 4.48E-01 | -0.17 | 0.75 | 5.30E-01 | 0.12 | 0.71 | 1.39E-01 | 0.29 | 0.78 | 1707395 | 247860 | 1783362 | 399990 | 1813888 | 397018 | 1752836 | 404628 |
| RP_POS_369_29678a10_881 | RP | POS | 10.88 | 369.2968 | | | | 1.74E-05 | -0.64 | 1.24 | 4.48E-01 | 0.16 | 1.11 | 1.59E-03 | -0.56 | 1.24 | 2.15E-04 | -0.72 | 1.32 | 22115357 | 948812 | 20972146 | 1908801 | 20826511 | 2019415 | 21117780 | 1800021 |
| RP_POS_381_72653a5_94 | RP | POS | 5.94 | 381.7265 | | | | 1.81E-07 | 0.62 | 1.32 | 4.49E-01 | -0.17 | 1.20 | 5.08E-04 | 0.54 | 1.40 | 1.17E-04 | 0.71 | 1.42 | 138710 | 48554 | 1963179 | 3249256 | 2210725 | 3498567 | 1715633 | 2994288 |
| RP_POS_309_65762a5_03 | RP | POS | 5.03 | 309.6576 | | | | 6.12E-05 | -0.70 | 1.20 | 4.50E-01 | 0.15 | 0.96 | 1.62E-03 | -0.63 | 1.20 | 1.56E-04 | -0.78 | 1.29 | 2097156 | 614675 | 1510234 | 847456 | 1445762 | 868427 | 1574705 | 829681 |
| RP_POS_384_20401a5_94 | RP | POS | 5.94 | 384.2040 | | | | 2.04E-07 | 0.62 | 1.32 | 4.50E-01 | -0.17 | 1.20 | 5.77E-04 | 0.54 | 1.40 | 1.17E-04 | 0.70 | 1.42 | 66714 | 27120 | 1055019 | 1768572 | 1189537 | 1895933 | 920502 | 1639524 |
| RP_POS_189_97673a0_256 | RP | POS | 0.26 | 189.9767 | | | | 6.37E-01 | 0.11 | 0.23 | 4.50E-01 | -0.14 | 0.83 | 8.82E-01 | 0.04 | 0.21 | 4.59E-01 | 0.18 | 0.34 | 746618 | 161743 | 761484 | 127382 | 771168 | 115351 | 751800 | 138875 |
| RP_POS_239_14914a2_881 | RP | POS | 2.88 | 239.1491 | | | | 3.83E-02 | 0.32 | 1.13 | 4.50E-01 | -0.17 | 1.08 | 1.78E-01 | 0.24 | 1.14 | 4.57E-02 | 0.40 | 1.11 | 1063313 | 1137495 | 1661574 | 2015794 | 1814812 | 2195794 | 1508336 | 1827662 |
| RP_POS_249_10088a15_34 | RP | POS | 15.34 | 249.1009 | | | | 6.24E-01 | -0.10 | 0.85 | 4.50E-01 | -0.15 | 0.79 | 4.34E-01 | -0.18 | 0.95 | 9.02E-01 | -0.03 | 0.62 | 1778786 | 467876 | 1732629 | 435957 | 1765766 | 466655 | 1699491 | 404955 |
| RP_POS_348_03409a4_852 | RP | POS | 4.85 | 348.0341 | | | | 7.03E-01 | 0.07 | 0.49 | 4.50E-01 | -0.16 | 0.57 | 9.66E-01 | -0.01 | 0.43 | 4.99E-01 | 0.15 | 0.51 | 770786 | 343310 | 798923 | 415366 | 830491 | 449908 | 767355 | 379643 |
| RP_POS_212_20049a10_847 | RP | POS | 10.85 | 212.2005 | | | | 8.34E-06 | 0.74 | 1.20 | 4.50E-01 | 0.16 | 0.92 | 2.97E-04 | 0.81 | 1.19 | 4.92E-05 | 0.66 | 1.47 | 537510 | 193050 | 754150 | 302074 | 731180 | 204231 | 777120 | 376261 |
| RP_POS_463_23245a9_325 | RP | POS | 9.33 | 463.2325 | | | | 3.95E-03 | -0.78 | 1.26 | 4.50E-01 | -0.12 | 0.51 | 2.43E-03 | -0.84 | 1.32 | 1.08E-02 | -0.72 | 1.01 | 1308208 | 1328933 | 550687 | 751127 | 607736 | 849563 | 493637 | 641480 |
| RP_POS_497_39575a12_129 | RP | POS | 12.13 | 497.3958 | | | | 5.48E-01 | -0.14 | 0.47 | 4.51E-01 | -0.14 | 0.68 | 4.08E-01 | -0.21 | 0.44 | 7.85E-01 | -0.07 | 0.52 | 2629099 | 1232391 | 2484439 | 987203 | 2559386 | 923810 | 2409491 | 1050781 |
| HILIC_POS_371_23969a5_648 | HILIC | POS | 5.648 | 371.2397 | | | | 4.36E-01 | -0.14 | 0.98 | 4.51E-01 | -0.16 | 0.85 | 3.00E-01 | -0.22 | 0.73 | 7.57E-01 | -0.06 | 0.66 | 9874219 | 1921444 | 9550454 | 2343195 | 9728312 | 2288011 | 9372595 | 2406994 |
| RP_POS_245_17462a7_475 | RP | POS | 7.48 | 245.1746 | | | | 8.92E-01 | -0.03 | 0.59 | 4.51E-01 | 0.14 | 0.72 | 8.67E-01 | 0.04 | 0.32 | 6.80E-01 | -0.10 | 0.53 | 1256930 | 271149 | 1249726 | 220262 | 1233009 | 227555 | 1266443 | 213704 |
| RP_POS_429_37354a12_328 | RP | POS | 12.33 | 429.3735 | | | | 9.18E-01 | 0.02 | 0.81 | 4.51E-01 | -0.15 | 0.49 | 8.22E-01 | -0.05 | 0.73 | 6.62E-01 | 0.10 | 0.33 | 2714370 | 715477 | 2729288 | 690776 | 2781719 | 612250 | 2676858 | 763952 |
| RP_POS_417_23962a6_184 | RP | POS | 6.18 | 417.2396 | | | | 2.01E-07 | 0.62 | 1.32 | 4.51E-01 | -0.17 | 1.20 | 5.34E-04 | 0.54 | 1.40 | 1.23E-04 | 0.70 | 1.42 | 9363 | 13867 | 266425 | 459397 | 301273 | 494431 | 231576 | 423624 |
| HILIC_POS_166_08627a3_928 | HILIC | POS | 3.928 | 166.0863 | | | | 3.88E-02 | 0.31 | 0.87 | 4.51E-01 | 0.17 | 0.86 | 6.44E-02 | 0.39 | 0.79 | 1.50E-01 | 0.23 | 0.71 | 108521868 | 26189660 | 122946764 | 51070096 | 119071175 | 39527769 | 126822354 | 60633646 |
| RP_POS_255_23077a9_676 | RP | POS | 9.68 | 255.2308 | | | | 2.41E-03 | 0.52 | 1.10 | 4.51E-01 | 0.16 | 0.70 | 4.73E-03 | 0.60 | 1.10 | 1.88E-02 | 0.45 | 1.15 | 5288328 | 638355 | 5742396 | 901912 | 5674012 | 809267 | 5810779 | 989463 |
| RP_POS_189_12393a0_706 | RP | POS | 0.71 | 189.1239 | | | | 3.60E-02 | 0.35 | 0.86 | 4.51E-01 | 0.16 | 1.04 | 4.45E-02 | 0.43 | 0.87 | 1.35E-01 | 0.27 | 0.74 | 584983 | 117720 | 642981 | 174799 | 629727 | 148693 | 656236 | 198154 |
| RP_POS_478_38843a13_49 | RP | POS | 13.49 | 478.3884 | | | | 2.18E-01 | 0.25 | 1.47 | 4.51E-01 | -0.15 | 0.50 | 4.29E-01 | 0.17 | 0.71 | 1.63E-01 | 0.32 | 1.53 | 7453897 | 2606556 | 8118046 | 2687050 | 8321637 | 2891334 | 7914455 | 2478594 |
| HILIC_NEG_241_75836a1_358 | HILIC | NEG | 1.358 | 241.7584 | | | | 5.97E-06 | 0.56 | 1.04 | 4.52E-01 | 0.17 | 0.52 | 6.35E-04 | 0.65 | 1.11 | 1.66E-03 | 0.48 | 1.28 | 383958 | 65251 | 532532 | 291919 | 510427 | 258607 | 554636 | 322952 |
| RP_NEG_228_9903a5_938 | RP | NEG | 5.94 | 228.9903 | | | | 5.25E-01 | -0.12 | 0.62 | 4.52E-01 | -0.16 | 0.60 | 3.50E-01 | -0.20 | 0.62 | 8.58E-01 | -0.04 | 0.29 | 1919086 | 1004131 | 1781184 | 1225530 | 1873939 | 1260802 | 1688430 | 1194704 |
| RP_POS_765_98492a10_507 | RP | POS | 10.51 | 765.9849 | | | | 1.55E-02 | -0.53 | 1.07 | 4.52E-01 | 0.14 | 0.57 | 5.15E-02 | -0.46 | 0.84 | 1.20E-02 | -0.60 | 1.05 | 4928357 | 1396242 | 4235378 | 1237048 | 4141814 | 1252617 | 4328942 | 1226739 |
| HILIC_NEG_197_80801a0_552 | HILIC | NEG | 0.552 | 197.8080 | | | | 5.76E-01 | 0.12 | 0.41 | 4.52E-01 | -0.15 | 0.72 | 8.50E-01 | 0.04 | 0.07 | 4.26E-01 | 0.19 | 0.56 | 1864056 | 859299 | 1961273 | 823300 | 2023551 | 909037 | 1898994 | 731584 |
| RP_POS_406_21732a6_067 | RP | POS | 6.07 | 406.2173 | | | | 1.66E-07 | 0.62 | 1.33 | 4.52E-01 | -0.17 | 1.21 | 5.20E-04 | 0.54 | 1.41 | 1.06E-04 | 0.71 | 1.43 | 52135 | 22293 | 906659 | 1516616 | 1021358 | 1624333 | 791960 | 1407793 |
| HILIC_POS_253_1279a2_142 | HILIC | POS | 2.142 | 253.1279 | | | | 1.13E-01 | 0.25 | 1.09 | 4.53E-01 | -0.16 | 0.76 | 3.47E-01 | 0.17 | 1.12 | 1.04E-01 | 0.33 | 1.17 | 293683 | 606735 | 532435 | 1034103 | 610541 | 1131016 | 454329 | 932156 |
| HILIC_POS_207_11263a3_929 | HILIC | POS | 3.929 | 207.1126 | | | | 1.99E-02 | 0.33 | 0.88 | 4.53E-01 | 0.17 | 0.85 | 5.38E-02 | 0.41 | 0.89 | 8.60E-02 | 0.24 | 0.82 | 843089 | 164515 | 957852 | 389871 | 928390 | 279390 | 987314 | 476729 |
| HILIC_POS_672_40845a1_048 | HILIC | POS | 1.048 | 672.4085 | | | | 5.94E-06 | 0.55 | 1.28 | 4.53E-01 | -0.17 | 0.94 | 2.17E-03 | 0.47 | 1.47 | 6.26E-04 | 0.64 | 1.43 | 346750 | 1068907 | 3270089 | 5862396 | 3712711 | 6409126 | 2827467 | 5287726 |
| RP_POS_476_27423a10_389 | RP | POS | 10.39 | 476.2742 | | | | 4.77E-01 | 0.13 | 1.00 | 4.53E-01 | -0.16 | 0.75 | 8.26E-01 | 0.05 | 0.71 | 3.08E-01 | 0.21 | 0.80 | 497707 | 155365 | 522152 | 204067 | 537556 | 193877 | 506748 | 214625 |
| HILIC_POS_266_1384a1_438 | HILIC | POS | 1.438 | 266.1384 | | | | 4.88E-01 | -0.12 | 0.54 | 4.53E-01 | 0.16 | 0.62 | 8.44E-01 | -0.04 | 0.20 | 2.95E-01 | -0.20 | 0.52 | 695100 | 388846 | 635129 | 515610 | 596205 | 451338 | 674052 | 574748 |
| RP_NEG_276_99146a7_339 | RP | NEG | 7.34 | 276.9915 | | | | 4.08E-01 | -0.17 | 0.42 | 4.54E-01 | 0.15 | 0.66 | 6.79E-01 | -0.10 | 0.27 | 2.77E-01 | -0.25 | 0.60 | 9810179 | 3447790 | 9229795 | 3362696 | 8976182 | 3214269 | 9483408 | 3519009 |
| RP_POS_185_11465a13_109 | RP | POS | 13.11 | 185.1147 | | | | 4.63E-01 | -0.14 | 0.77 | 4.54E-01 | 0.16 | 0.55 | 7.85E-01 | -0.06 | 0.45 | 3.19E-01 | -0.21 | 0.73 | 5343873 | 548243 | 5257115 | 665136 | 5206958 | 678981 | 5307273 | 653980 |
| HILIC_POS_243_05292a4_123 | HILIC | POS | 4.123 | 243.0529 | | | | 1.29E-02 | -0.44 | 1.02 | 4.54E-01 | -0.16 | 1.27 | 1.60E-02 | -0.52 | 1.10 | 6.50E-02 | -0.36 | 1.01 | 1162776 | 447236 | 908834 | 604775 | 954444 | 553354 | 863224 | 654617 |
| HILIC_POS_499_05829a7_642 | HILIC | POS | 7.642 | 499.0583 | | | | 5.01E-02 | -0.38 | 0.58 | 4.54E-01 | -0.15 | 0.60 | 3.63E-02 | -0.46 | 0.77 | 1.63E-01 | -0.31 | 0.52 | 1037476 | 46351 | 1018178 | 50839 | 1022009 | 51726 | 1014347 | 50165 |
| HILIC_POS_275_10983a2_143 | HILIC | POS | 2.143 | 275.1098 | | | | 1.29E-01 | 0.24 | 1.08 | 4.54E-01 | -0.16 | 0.76 | 3.71E-01 | 0.16 | 1.10 | 1.16E-01 | 0.33 | 1.17 | 424419 | 943200 | 773671 | 1553860 | 890761 | 1705239 | 656580 | 1393705 |
| HILIC_POS_224_12772a5_464 | HILIC | POS | 5.464 | 224.1277 | | | | 1.72E-01 | -0.27 | 0.42 | 4.54E-01 | -0.15 | 0.61 | 1.28E-01 | -0.35 | 0.57 | 3.69E-01 | -0.20 | 0.34 | 20258308 | 2631620 | 19507537 | 2791806 | 19717728 | 2650131 | 19297347 | 2938378 |
| RP_NEG_452_27853a10_513 | RP | NEG | 10.51 | 452.2785 | | | | 3.73E-01 | 0.16 | 1.01 | 4.54E-01 | -0.16 | 0.83 | 7.26E-01 | 0.08 | 0.72 | 2.22E-01 | 0.24 | 0.85 | 10631940 | 3031300 | 11238847 | 4134373 | 11550088 | 3665074 | 10927606 | 4572139 |
| RP_NEG_197_08165a5_049 | RP | NEG | 5.05 | 197.0817 | | | | 5.98E-01 | -0.11 | 0.22 | 4.55E-01 | -0.15 | 0.57 | 4.23E-01 | -0.18 | 0.41 | 8.85E-01 | -0.03 | 0.25 | 864147 | 42465 | 859570 | 42380 | 862759 | 42178 | 856381 | 42767 |
| HILIC_POS_167_01276a7_963 | HILIC | POS | 7.963 | 167.0128 | | | | 1.26E-04 | -0.60 | 1.26 | 4.55E-01 | 0.16 | 0.94 | 6.39E-03 | -0.52 | 1.34 | 3.97E-04 | -0.67 | 1.35 | 35845000 | 8849410 | 26923198 | 15919122 | 25725776 | 15859874 | 28120620 | 16048269 |
| RP_POS_630_78296a0_5 | RP | POS | 0.50 | 630.7830 | | | | 2.85E-02 | -0.31 | 1.09 | 4.55E-01 | -0.17 | 1.27 | 2.10E-02 | -0.40 | 1.12 | 2.29E-01 | -0.23 | 1.13 | 1786139 | 477225 | 1487620 | 1048962 | 1566463 | 1132948 | 1408778 | 962726 |
| HILIC_POS_74_09717a4_078 | HILIC | POS | 4.078 | 74.0972 | | | | 5.50E-01 | 0.13 | 0.45 | 4.55E-01 | -0.15 | 0.70 | 8.15E-01 | 0.06 | 0.15 | 3.84E-01 | 0.20 | 0.40 | 9779003 | 3334255 | 10176539 | 2972453 | 10399921 | 2772694 | 9953157 | 3172002 |
| RP_POS_379_24896a5_939 | RP | POS | 5.94 | 379.2490 | | | | 1.90E-07 | 0.62 | 1.32 | 4.55E-01 | -0.17 | 1.19 | 5.18E-04 | 0.54 | 1.40 | 1.20E-04 | 0.70 | 1.42 | 68439 | 31069 | 1076010 | 1797656 | 1211043 | 1933377 | 940977 | 1659495 |
| RP_POS_240_15735a6_319 | RP | POS | 6.32 | 240.1574 | | | | 5.23E-04 | -0.64 | 1.02 | 4.55E-01 | -0.15 | 0.69 | 1.33E-03 | -0.71 | 1.13 | 4.00E-03 | -0.56 | 1.05 | 3295799 | 387177 | 2989410 | 485490 | 3025875 | 420683 | 2952944 | 544539 |
| RP_NEG_995_61194a10_335 | RP | NEG | 10.34 | 995.6119 | | | | 2.34E-01 | 0.24 | 1.01 | 4.56E-01 | -0.15 | 0.89 | 4.99E-01 | 0.16 | 0.76 | 1.31E-01 | 0.31 | 0.96 | 3739166 | 1688229 | 4157254 | 1779802 | 4290813 | 1423838 | 4023695 | 2082383 |
| HILIC_NEG_134_89473a3_102 | HILIC | NEG | 3.102 | 134.8947 | | | | 3.47E-05 | -0.75 | 1.22 | 4.56E-01 | 0.15 | 0.79 | 1.00E-03 | -0.68 | 1.16 | 6.53E-05 | -0.83 | 1.36 | 117444970 | 37992027 | 80550312 | 48977777 | 76878837 | 48524911 | 84221787 | 49642966 |
| HILIC_NEG_158_93909a2_999 | HILIC | NEG | 2.999 | 158.9391 | | | | 1.36E-01 | -0.29 | 0.91 | 4.56E-01 | 0.15 | 0.83 | 3.18E-01 | -0.21 | 0.46 | 1.04E-01 | -0.37 | 0.69 | 2715904 | 566386 | 2537159 | 627688 | 2490121 | 661013 | 2584196 | 595458 |
| RP_POS_326_37796a8_98 | RP | POS | 8.98 | 326.3780 | | | | 6.01E-01 | -0.08 | 0.54 | 4.57E-01 | -0.16 | 0.75 | 2.88E-01 | -0.17 | 1.06 | 9.87E-01 | 0.00 | 0.24 | 983509 | 702436 | 895582 | 1123134 | 979751 | 1418931 | 811413 | 721649 |
| RP_POS_230_09573a0_616 | RP | POS | 0.62 | 230.0957 | | | | 6.43E-03 | 0.39 | 0.78 | 4.57E-01 | 0.16 | 0.48 | 2.82E-03 | 0.48 | 1.07 | 1.27E-01 | 0.31 | 0.56 | 189282 | 266589 | 396098 | 576786 | 352915 | 671346 | 439282 | 466523 |
| RP_POS_423_27496a6_184 | RP | POS | 6.18 | 423.2750 | | | | 1.58E-07 | 0.63 | 1.32 | 4.57E-01 | -0.17 | 1.19 | 4.92E-04 | 0.54 | 1.39 | 1.06E-04 | 0.71 | 1.43 | 28584 | 17785 | 674595 | 1144186 | 760210 | 1226422 | 588979 | 1061119 |
| HILIC_POS_156_0421a3_694 | HILIC | POS | 3.694 | 156.0421 | | | | 4.49E-01 | -0.14 | 0.98 | 4.57E-01 | 0.16 | 0.71 | 7.83E-01 | -0.06 | 0.97 | 3.10E-01 | -0.21 | 0.73 | 20590395 | 7359201 | 19359781 | 9601094 | 18641504 | 9870659 | 20078059 | 9368055 |

**Supplementary table 1: Metabolomics results** (in asceding order according to p-value from progressive ALD vs  non-progressive ALD comparison, with molecular features with ID_level of 1-2 first)

| Feature_ID | RP_HILIC | POS_NEG | Average.Rt | Average.Mz | Metabolite.name | curated_MSMS | ID_level | all_ALD_vs_control t-test p | all_ALD_vs_control Cohen's d | all_ALD_vs_control PLS-DA VIP | Prog_ALD_vs_non-prog_ALD t-test p | Prog_ALD_vs_non-prog_ALD Cohen's d | Prog_ALD_vs_non-prog_ALD PLS-DA VIP | Prog_ALD_vs_control_ALD t-test p | Prog_ALD_vs_control_ALD Cohen's d | Prog_ALD_vs_control_ALD PLS-DA VIP | Non-prog_ALD_vs_control t-test p | Non-prog_ALD_vs_control Cohen's d | Non-prog_ALD_vs_control PLS-DA VIP | Healthy_control Mean | Healthy_control SD | all_ALD Mean | all_ALD SD | Non-prog_ALD Mean | Non-prog_ALD SD | Progressive_ALD Mean | Progressive_ALD SD |
|---|---|---|---|---|---|---|---|---|---|---|---|---|---|---|---|---|---|---|---|---|---|---|---|---|---|---|---|
| RP_POS_435_34415a11_53 | RP | POS | 11.53 | 435.3442 | | | | 4.21E-02 | -0.34 | 0.95 | 4.57E-01 | 0.16 | 0.80 | 1.66E-01 | -0.26 | 0.89 | 4.32E-02 | -0.42 | 0.85 | 1142587 | 187275 | 1053555 | 275736 | 1032926 | 296192 | 1074184 | 254978 |
| HILIC_NEG_134_89471a1_69 | HILIC | NEG | 1.69 | 134.8947 | | | | 1.32E-03 | -0.63 | 1.14 | 4.58E-01 | 0.15 | 0.67 | 9.19E-03 | -0.56 | 0.96 | 1.55E-03 | -0.71 | 1.29 | 24936513 | 7706106 | 19525402 | 8407677 | 18897258 | 8695960 | 20153546 | 8148359 |
| RP_NEG_818_55737a11_651 | RP | NEG | 11.65 | 818.5574 | | | | 7.78E-01 | 0.05 | 0.35 | 4.58E-01 | -0.16 | 0.61 | 8.42E-01 | -0.04 | 0.32 | 5.51E-01 | 0.13 | 0.43 | 1873608 | 2748162 | 2058727 | 4400868 | 2387415 | 4995102 | 1730039 | 3736177 |
| RP_POS_280_26364a9_712 | RP | POS | 9.71 | 280.2636 | | | | 3.68E-01 | -0.25 | 0.87 | 4.58E-01 | -0.11 | 0.51 | 2.85E-01 | -0.31 | 0.70 | 5.01E-01 | -0.20 | 0.56 | 10703579 | 20182106 | 7324251 | 10106249 | 8078824 | 10626118 | 6569678 | 9606278 |
| RP_POS_497_39651a13_201 | RP | POS | 13.20 | 497.3965 | | | | 7.04E-01 | 0.08 | 0.49 | 4.58E-01 | 0.15 | 0.50 | 5.03E-01 | 0.15 | 0.64 | 9.81E-01 | 0.01 | 0.49 | 37752118 | 9799049 | 38503470 | 9350003 | 37805731 | 9694738 | 39201209 | 9035914 |
| RP_NEG_947_612a10_515 | RP | NEG | 10.52 | 947.6120 | | | | 6.11E-01 | 0.09 | 0.91 | 4.59E-01 | -0.16 | 0.78 | 9.50E-01 | 0.01 | 0.65 | 4.03E-01 | 0.17 | 0.73 | 1245111 | 356988 | 1284804 | 453810 | 1318626 | 427119 | 1250982 | 480962 |
| HILIC_POS_354_01196a7_39 | HILIC | POS | 7.39 | 354.0120 | | | | 7.33E-01 | 0.07 | 0.21 | 4.59E-01 | 0.15 | 0.65 | 5.23E-01 | 0.14 | 0.24 | 9.69E-01 | -0.01 | 0.31 | 2644358 | 107293 | 2651972 | 115065 | 2643401 | 113042 | 2660542 | 117567 |
| HILIC_NEG_134_89474a4_83 | HILIC | NEG | 4.83 | 134.8947 | | | | 2.58E-03 | 0.52 | 0.98 | 4.59E-01 | 0.16 | 0.56 | 2.62E-03 | 0.60 | 1.12 | 3.15E-02 | 0.44 | 0.75 | 30104543 | 13672817 | 39797353 | 19542690 | 38341817 | 20397189 | 41252889 | 18741897 |
| RP_POS_180_96384a0_25 | RP | POS | 0.25 | 180.9638 | | | | 3.11E-01 | 0.19 | 0.57 | 4.59E-01 | -0.15 | 0.44 | 5.94E-01 | 0.11 | 0.29 | 2.21E-01 | 0.27 | 0.57 | 738992 | 105146 | 761854 | 125051 | 771168 | 129042 | 752541 | 121515 |
| RP_NEG_205_83934a1_084 | RP | NEG | 1.08 | 205.8393 | | | | 1.20E-03 | 0.62 | 1.08 | 4.59E-01 | -0.15 | 0.63 | 1.21E-02 | 0.54 | 1.04 | 1.14E-03 | 0.69 | 1.15 | 1929262 | 1514888 | 3021465 | 1773268 | 3153473 | 1715048 | 2889457 | 1837437 |
| RP_POS_322_27151a9_943 | RP | POS | 9.94 | 322.2715 | | | | 7.22E-05 | 0.60 | 0.97 | 4.60E-01 | -0.16 | 0.51 | 2.83E-03 | 0.52 | 1.01 | 6.99E-04 | 0.68 | 1.25 | 384402 | 307456 | 718858 | 599115 | 763446 | 653032 | 674271 | 542859 |
| RP_POS_261_14615a8_915 | RP | POS | 8.92 | 261.1462 | | | | 9.02E-06 | -0.77 | 1.19 | 4.60E-01 | -0.15 | 0.56 | 3.00E-05 | -0.84 | 1.44 | 5.03E-04 | -0.69 | 1.15 | 2248874 | 82889 | 2159011 | 118686 | 2167838 | 119189 | 2150185 | 118724 |
| RP_POS_463_08826a0_694 | RP | POS | 0.69 | 463.0883 | | | | 1.13E-10 | -0.88 | 1.53 | 4.60E-01 | -0.15 | 1.18 | 8.99E-08 | -0.96 | 1.75 | 8.15E-06 | -0.80 | 1.45 | 587439 | 86206 | 394409 | 228361 | 411389 | 233949 | 377430 | 223698 |
| RP_POS_194_15178a8_531 | RP | POS | 8.53 | 194.1518 | | | | 2.52E-01 | 0.26 | 0.61 | 4.60E-01 | -0.14 | 0.51 | 4.47E-01 | 0.19 | 0.57 | 1.67E-01 | 0.33 | 0.57 | 354345 | 234465 | 407198 | 188076 | 421183 | 167327 | 393212 | 207533 |
| HILIC_POS_583_25488a0_633 | HILIC | POS | 0.633 | 583.2549 | | | | 5.48E-01 | 0.12 | 0.66 | 4.60E-01 | 0.15 | 0.97 | 4.09E-01 | 0.19 | 0.31 | 8.40E-01 | 0.04 | 0.09 | 1128454 | 581053 | 1201265 | 631050 | 1154346 | 534730 | 1248185 | 717039 |
| RP_POS_312_15924a9_005 | RP | POS | 9.01 | 312.1592 | | | | 2.96E-01 | 0.18 | 0.60 | 4.60E-01 | -0.16 | 0.46 | 5.61E-01 | 0.10 | 0.57 | 2.52E-01 | 0.26 | 0.57 | 2012784 | 2829832 | 2679409 | 3873140 | 2967458 | 4659197 | 2391360 | 2903640 |
| RP_POS_157_98714a15_426 | RP | POS | 15.43 | 157.9871 | | | | 1.19E-01 | -0.26 | 1.42 | 4.60E-01 | 0.16 | 0.62 | 3.14E-01 | -0.18 | 0.68 | 1.18E-01 | -0.34 | 1.51 | 1929854 | 260120 | 1834238 | 395793 | 1804827 | 455107 | 1863650 | 328044 |
| RP_POS_419_75543a13_398 | RP | POS | 13.40 | 419.7554 | | | | 4.21E-01 | 0.17 | 0.42 | 4.60E-01 | -0.15 | 0.56 | 6.68E-01 | 0.10 | 0.38 | 3.04E-01 | 0.25 | 0.70 | 9768499 | 2427367 | 10161466 | 2232993 | 10327308 | 2306011 | 9995623 | 2168055 |
| RP_POS_280_05966a3_969 | RP | POS | 3.97 | 280.0597 | | | | 7.94E-01 | 0.04 | 0.99 | 4.61E-01 | -0.16 | 0.54 | 8.45E-01 | -0.04 | 0.75 | 4.75E-01 | 0.12 | 0.50 | 2601573 | 592184 | 2640239 | 1046342 | 2717966 | 874237 | 2562512 | 1198076 |
| HILIC_POS_151_14423a1_508 | HILIC | POS | 1.508 | 151.1442 | | | | 2.98E-01 | 0.16 | 0.32 | 4.61E-01 | 0.16 | 0.95 | 2.53E-01 | 0.24 | 0.39 | 6.33E-01 | 0.08 | 0.19 | 33213452 | 32178680 | 41715353 | 58058530 | 37406252 | 46916722 | 46024454 | 67611419 |
| HILIC_POS_233_18591a0_55 | HILIC | POS | 0.55 | 233.1859 | | | | 1.45E-01 | -0.34 | 0.77 | 4.61E-01 | -0.14 | 0.65 | 1.08E-01 | -0.41 | 0.81 | 2.74E-01 | -0.27 | 0.55 | 938808 | 808504 | 706365 | 642108 | 753980 | 615877 | 658750 | 670140 |
| RP_NEG_225_89645a15_575 | RP | NEG | 15.58 | 225.8965 | | | | 8.13E-01 | 0.05 | 0.27 | 4.61E-01 | -0.15 | 0.59 | 9.25E-01 | -0.02 | 0.45 | 5.95E-01 | 0.12 | 0.62 | 2934385 | 137770 | 2940937 | 127811 | 2950408 | 124023 | 2931466 | 132061 |
| RP_POS_336_05029a4_851 | RP | POS | 4.85 | 336.0503 | | | | 6.32E-01 | 0.09 | 0.51 | 4.62E-01 | -0.16 | 0.55 | 9.67E-01 | 0.01 | 0.45 | 4.51E-01 | 0.16 | 0.52 | 1673822 | 731159 | 1750565 | 936556 | 1819929 | 1010333 | 1681202 | 861153 |
| RP_POS_403_73975a6_067 | RP | POS | 6.07 | 403.7398 | | | | 1.44E-07 | 0.63 | 1.33 | 4.62E-01 | -0.16 | 1.20 | 4.78E-04 | 0.55 | 1.41 | 9.92E-05 | 0.71 | 1.43 | 118152 | 37932 | 1684692 | 2763989 | 1889382 | 2954951 | 1480001 | 2572536 |
| RP_POS_396_2417a10_142 | RP | POS | 10.14 | 396.2417 | | | | 5.37E-01 | -0.18 | 0.78 | 4.62E-01 | -0.11 | 0.56 | 4.32E-01 | -0.23 | 0.52 | 6.76E-01 | -0.13 | 0.66 | 1194794 | 3397901 | 808448 | 1541129 | 922566 | 1715048 | 694331 | 1353020 |
| HILIC_POS_64_01662a5_648 | HILIC | POS | 5.648 | 64.0166 | | | | 7.98E-01 | 0.05 | 0.54 | 4.62E-01 | -0.15 | 0.64 | 9.17E-01 | -0.02 | 0.23 | 5.58E-01 | 0.13 | 0.33 | 2697284 | 617779 | 2730038 | 653201 | 2778363 | 591285 | 2681713 | 712507 |
| RP_POS_740_46301a5_94 | RP | POS | 5.94 | 740.4630 | | | | 2.12E-07 | 0.62 | 1.32 | 4.62E-01 | -0.16 | 1.19 | 5.30E-04 | 0.54 | 1.40 | 1.32E-04 | 0.70 | 1.42 | 12849 | 9167 | 453099 | 789145 | 511471 | 849400 | 394727 | 727856 |
| RP_POS_473_34268a10_457 | RP | POS | 10.46 | 473.3427 | | | | 8.32E-01 | -0.04 | 0.71 | 4.62E-01 | 0.15 | 0.79 | 8.84E-01 | 0.03 | 0.56 | 6.11E-01 | -0.12 | 0.52 | 26358867 | 1578366 | 26289855 | 1636263 | 26168836 | 1738535 | 26410874 | 1535239 |
| RP_POS_392_74878a6_067 | RP | POS | 6.07 | 392.7488 | | | | 1.48E-07 | 0.63 | 1.33 | 4.63E-01 | -0.16 | 1.20 | 4.79E-04 | 0.55 | 1.40 | 1.02E-04 | 0.71 | 1.43 | 19773 | 13312 | 507347 | 861200 | 571005 | 921291 | 443688 | 800887 |
| HILIC_NEG_351_99756a7_368 | HILIC | NEG | 7.368 | 351.9976 | | | | 9.57E-02 | 0.35 | 0.70 | 4.63E-01 | -0.14 | 0.58 | 2.42E-01 | 0.28 | 0.60 | 5.95E-02 | 0.43 | 0.78 | 1084401 | 40248 | 1098090 | 37871 | 1100887 | 33847 | 1095294 | 41667 |
| HILIC_POS_171_18555a5_121 | HILIC | POS | 5.121 | 171.1856 | | | | 9.27E-01 | 0.02 | 0.59 | 4.63E-01 | -0.15 | 0.76 | 7.95E-01 | -0.06 | 0.20 | 6.34E-01 | 0.09 | 0.18 | 286906319 | 47999507 | 287842267 | 57079472 | 292057655 | 46933743 | 283626879 | 65905865 |
| HILIC_NEG_98_93896a5_606 | HILIC | NEG | 5.606 | 98.9390 | | | | 2.33E-01 | -0.28 | 0.81 | 4.63E-01 | 0.14 | 0.73 | 4.02E-01 | -0.22 | 0.35 | 1.62E-01 | -0.35 | 0.76 | 1503638 | 241294 | 1447581 | 180717 | 1434243 | 169132 | 1460919 | 192393 |
| RP_POS_235_16945a9_111 | RP | POS | 9.11 | 235.1695 | | | | 6.24E-01 | -0.09 | 0.62 | 4.63E-01 | 0.15 | 0.77 | 9.38E-01 | -0.02 | 0.64 | 4.51E-01 | -0.17 | 0.60 | 9590029 | 366438 | 9551793 | 427615 | 9520242 | 463121 | 9583343 | 391061 |
| RP_POS_326_30518a10_81 | RP | POS | 10.81 | 326.3052 | | | | 1.41E-18 | 1.18 | 1.94 | 4.64E-01 | 0.14 | 0.83 | 1.21E-12 | 1.25 | 2.14 | 1.27E-09 | 1.11 | 1.95 | 523001 | 197199 | 1203839 | 559827 | 1162558 | 588885 | 1245119 | 531925 |
| RP_POS_280_05978a3_712 | RP | POS | 3.71 | 280.0598 | | | | 2.12E-01 | 0.22 | 0.75 | 4.64E-01 | -0.16 | 0.54 | 5.22E-01 | 0.14 | 0.77 | 1.20E-01 | 0.30 | 0.58 | 1235242 | 412503 | 1351781 | 568482 | 1393698 | 491701 | 1309864 | 638423 |
| RP_POS_263_00732a0_549 | RP | POS | 0.55 | 263.0073 | | | | 5.82E-04 | 0.43 | 1.19 | 4.64E-01 | -0.17 | 1.19 | 3.26E-02 | 0.34 | 1.15 | 4.81E-03 | 0.51 | 1.32 | 918470 | 90603 | 1073541 | 408789 | 1103674 | 431157 | 1043408 | 387140 |
| RP_POS_440_26535a11_668 | RP | POS | 11.67 | 440.2654 | | | | 7.19E-01 | -0.07 | 0.75 | 4.64E-01 | -0.15 | 0.48 | 5.19E-01 | -0.15 | 0.54 | 9.97E-01 | 0.00 | 0.51 | 1246252 | 724402 | 1193232 | 710918 | 1245626 | 715900 | 1140839 | 709230 |
| RP_POS_795_54419a10_663 | RP | POS | 10.66 | 795.5442 | | | | 3.10E-01 | 0.17 | 0.74 | 4.64E-01 | -0.16 | 0.44 | 6.77E-01 | 0.09 | 0.43 | 1.68E-01 | 0.25 | 0.71 | 2388762 | 204374 | 2437361 | 308637 | 2460115 | 256973 | 2414608 | 354071 |
| RP_POS_820_58289a13_191 | RP | POS | 13.19 | 820.5829 | | | | 1.11E-09 | -1.01 | 1.93 | 4.64E-01 | -0.14 | 0.56 | 6.54E-08 | -1.09 | 2.04 | 4.66E-07 | -0.94 | 1.72 | 2128772 | 584102 | 1183128 | 908758 | 1250088 | 865114 | 1116167 | 954443 |
| RP_NEG_453_28665a9_323 | RP | NEG | 9.32 | 453.2867 | | | | 2.50E-01 | -0.33 | 0.66 | 4.64E-01 | 0.11 | 0.49 | 3.57E-01 | -0.27 | 0.46 | 1.91E-01 | -0.38 | 0.58 | 1792878 | 3562296 | 1028731 | 1743963 | 900266 | 1638076 | 1157195 | 1851461 |
| HILIC_POS_460_27512a0_545 | HILIC | POS | 0.545 | 460.2751 | | | | 3.45E-01 | -0.19 | 0.55 | 4.65E-01 | 0.15 | 0.68 | 6.14E-01 | -0.11 | 0.31 | 2.34E-01 | -0.26 | 0.44 | 970751 | 139054 | 943552 | 145304 | 932867 | 139568 | 954238 | 151477 |
| RP_POS_132_0808a2_894 | RP | POS | 2.89 | 132.0808 | | | | 7.60E-04 | -0.57 | 1.21 | 4.65E-01 | -0.15 | 1.03 | 3.16E-03 | -0.65 | 1.21 | 5.50E-03 | -0.49 | 1.17 | 1130002 | 192156 | 974690 | 284441 | 995618 | 229558 | 953763 | 331472 |
| RP_POS_425_21384a9_946 | RP | POS | 9.95 | 425.2138 | | | | 1.66E-04 | -0.69 | 1.18 | 4.65E-01 | 0.15 | 0.89 | 2.21E-03 | -0.61 | 1.21 | 4.05E-04 | -0.76 | 1.16 | 1067149 | 53795 | 1020279 | 68851 | 1015216 | 73177 | 1025342 | 64579 |
| RP_NEG_394_90125a15_433 | RP | NEG | 15.43 | 394.9013 | | | | 5.82E-02 | -0.41 | 0.77 | 4.65E-01 | -0.14 | 0.47 | 3.91E-02 | -0.48 | 0.83 | 1.55E-01 | -0.34 | 0.65 | 1015618 | 68612 | 989244 | 61297 | 993751 | 64818 | 984737 | 57866 |
| RP_POS_814_55811a11_168 | RP | POS | 11.17 | 814.5581 | | | | 3.25E-02 | 0.27 | 0.57 | 4.65E-01 | -0.17 | 0.61 | 1.60E-01 | 0.19 | 0.56 | 8.24E-02 | 0.35 | 0.63 | 1203344 | 721633 | 1858267 | 2748513 | 2060353 | 3305061 | 1656181 | 2063019 |
| HILIC_NEG_134_89471a7_332 | HILIC | NEG | 7.332 | 134.8947 | | | | 8.26E-02 | -0.33 | 0.87 | 4.66E-01 | 0.15 | 0.77 | 2.14E-01 | -0.26 | 0.70 | 7.07E-02 | -0.41 | 0.67 | 7836164 | 521722 | 7642986 | 588084 | 7599795 | 637144 | 7686178 | 537573 |
| RP_POS_663_45435a13_57 | RP | POS | 13.57 | 663.4544 | CE(16:0) [M+K]+ | 495.2669(100), 493.2017(60), 551.3235(50), 607.388(40), 381.1398(35), | | 3.84E-01 | 0.16 | 0.55 | 4.66E-01 | 0.15 | 0.76 | 2.45E-01 | 0.24 | 0.52 | 7.02E-01 | 0.08 | 0.35 | 61142289 | 4887120 | 62065156 | 5999226 | 61624739 | 6470495 | 62505572 | 5518404 |
| RP_POS_127_03481a15_448 | RP | POS | 15.45 | 127.0348 | | | | 9.76E-01 | 0.00 | 0.18 | 4.66E-01 | 0.16 | 0.59 | 6.12E-01 | 0.07 | 0.46 | 7.18E-01 | -0.08 | 0.32 | 22271329 | 569072 | 22267247 | 907194 | 22200538 | 1185875 | 22333955 | 497408 |
| RP_POS_556_4425a10_461 | RP | POS | 10.46 | 556.4425 | | | | 3.10E-01 | -0.20 | 0.48 | 4.66E-01 | 0.15 | 0.52 | 5.82E-01 | -0.13 | 0.42 | 1.94E-01 | -0.28 | 0.59 | 5177492 | 402656 | 5092629 | 421900 | 5061659 | 368802 | 5123599 | 470807 |
| RP_POS_377_32785a10_705 | RP | POS | 10.71 | 377.3279 | | | | 1.80E-01 | -0.31 | 1.03 | 4.66E-01 | -0.14 | 0.49 | 1.37E-01 | -0.38 | 0.92 | 3.21E-01 | -0.24 | 0.56 | 7768763 | 745848 | 7571429 | 597007 | 7615204 | 552399 | 7527654 | 641134 |
| RP_POS_263_23676a10_977 | RP | POS | 10.98 | 263.2368 | | | | 5.37E-03 | 0.47 | 1.39 | 4.67E-01 | -0.15 | 1.28 | 4.87E-02 | 0.39 | 1.12 | 6.19E-03 | 0.55 | 1.35 | 931261 | 457624 | 1233907 | 679841 | 1283722 | 676781 | 1184092 | 686079 |
| RP_POS_804_84253a0_493 | RP | POS | 0.49 | 804.8425 | | | | 8.07E-08 | 0.64 | 1.33 | 4.67E-01 | -0.16 | 1.17 | 4.38E-04 | 0.56 | 1.39 | 6.10E-05 | 0.72 | 1.44 | 27856 | 15831 | 2245001 | 3824801 | 2525235 | 4025600 | 1964767 | 3631947 |
| RP_POS_278_24786a9_21 | RP | POS | 9.21 | 278.2479 | | | | 2.45E-01 | -0.32 | 0.94 | 4.67E-01 | -0.11 | 0.54 | 1.86E-01 | -0.38 | 0.73 | 3.57E-01 | -0.27 | 0.62 | 9142800 | 17141774 | 5404557 | 8996095 | 6063534 | 9359395 | 4745581 | 8661787 |
| RP_POS_241_15463a4_67 | RP | POS | 4.67 | 241.1546 | | | | 1.06E-11 | 0.87 | 1.44 | 4.67E-01 | -0.15 | 0.52 | 1.21E-05 | 0.79 | 1.48 | 2.77E-08 | 0.95 | 1.62 | 352772 | 209291 | 1007259 | 792822 | 1065305 | 745195 | 949214 | 841292 |
| RP_NEG_266_80435a0_521 | RP | NEG | 0.52 | 266.8044 | | | | 2.46E-04 | 0.46 | 1.23 | 4.67E-01 | -0.16 | 1.17 | 1.74E-02 | 0.38 | 1.20 | 3.10E-03 | 0.54 | 1.34 | 1821411 | 261620 | 2247025 | 1028531 | 2322319 | 1099136 | 2171731 | 957952 |
| RP_POS_418_31619a10_242 | RP | POS | 10.24 | 418.3162 | | | | 4.60E-08 | 0.65 | 1.32 | 4.67E-01 | -0.16 | 1.17 | 3.57E-04 | 0.57 | 1.34 | 4.14E-05 | 0.74 | 1.46 | 19246 | 15254 | 266016 | 416079 | 296439 | 434911 | 235593 | 398420 |
| RP_POS_785_41309a5_862 | RP | POS | 5.86 | 785.4131 | | | | 9.42E-07 | 0.59 | 1.27 | 4.68E-01 | -0.16 | 1.16 | 1.02E-03 | 0.50 | 1.38 | 2.91E-04 | 0.67 | 1.35 | 41497 | 58282 | 466068 | 806725 | 525055 | 874050 | 407081 | 737448 |
| HILIC_POS_116_07091a7_152 | HILIC | POS | 7.152 | 116.0709 | | | | 2.48E-03 | 0.47 | 0.98 | 4.68E-01 | 0.16 | 0.83 | 1.16E-02 | 0.54 | 0.93 | 1.47E-02 | 0.39 | 0.94 | 4156254 | 1157035 | 5069682 | 2111765 | 4915188 | 1591799 | 5224175 | 2535267 |
| RP_POS_159_06534a4_852 | RP | POS | 4.85 | 159.0653 | | | | 5.57E-01 | 0.10 | 0.50 | 4.68E-01 | -0.16 | 0.56 | 8.96E-01 | 0.03 | 0.43 | 4.00E-01 | 0.18 | 0.58 | 97355935 | 43035917 | 103003818 | 58109051 | 107251278 | 62782000 | 98756359 | 53327642 |
| RP_POS_388_25415a4_201 | RP | POS | 4.20 | 388.2542 | | | | 9.86E-01 | 0.00 | 1.20 | 4.68E-01 | -0.11 | 1.10 | 8.30E-01 | -0.06 | 0.93 | 8.59E-01 | 0.05 | 1.01 | 1900595 | 6008143 | 1880970 | 3102293 | 2107722 | 3317780 | 1654217 | 2886566 |
| RP_POS_188_07039a2_893 | RP | POS | 2.89 | 188.0704 | fragment of tryptophan (desethyl atrazine) | 146.0602(100), 188.0693(45) | | 1.02E-03 | -0.56 | 1.20 | 4.68E-01 | -0.15 | 1.04 | 3.44E-03 | -0.64 | 1.21 | 7.16E-03 | -0.49 | 1.16 | 129950787 | 22068613 | 112793151 | 31569521 | 115100147 | 26074177 | 110486154 | 36371478 |
| RP_NEG_792_86108a0_482 | RP | NEG | 0.48 | 792.8611 | | | | 6.26E-08 | 0.65 | 1.34 | 4.68E-01 | -0.16 | 1.20 | 2.87E-04 | 0.57 | 1.39 | 6.91E-05 | 0.73 | 1.46 | 1464144 | 370472 | 6965787 | 9382684 | 7650981 | 10053007 | 6280593 | 8708906 |
| RP_POS_334_31085a11_149 | RP | POS | 11.15 | 334.3109 | | | | 7.11E-01 | -0.08 | 0.60 | 4.68E-01 | -0.15 | 0.43 | 5.10E-01 | -0.15 | 0.38 | 9.92E-01 | 0.00 | 0.36 | 2207633 | 1506077 | 2093529 | 1515115 | 2204126 | 1547980 | 1982931 | 1488888 |
| RP_POS_792_76532a0_501 | RP | POS | 0.50 | 792.7653 | | | | 3.93E-03 | -0.40 | 1.14 | 4.68E-01 | -0.16 | 1.32 | 5.62E-03 | -0.48 | 1.18 | 8.26E-02 | -0.32 | 1.18 | 1193012 | 189731 | 1026527 | 456301 | 1059820 | 479323 | 993234 | 434348 |
| HILIC_NEG_85_00388a3_061 | HILIC | NEG | 3.061 | 85.0039 | | | | 9.43E-02 | -0.39 | 0.79 | 4.69E-01 | 0.13 | 0.54 | 1.86E-01 | -0.32 | 0.55 | 7.39E-02 | -0.46 | 0.98 | 9448347 | 3850087 | 8176463 | 2998711 | 7957719 | 3220479 | 8395207 | 2774693 |
| RP_POS_304_09793a7_093 | RP | POS | 7.09 | 304.0979 | | | | 3.48E-01 | -0.18 | 0.53 | 4.69E-01 | 0.15 | 0.55 | 6.34E-01 | -0.10 | 0.34 | 2.48E-01 | -0.25 | 0.59 | 2922297 | 178860 | 2886338 | 211462 | 2870914 | 217776 | 2901762 | 205993 |
| RP_POS_370_73569a5_94 | RP | POS | 5.94 | 370.7357 | | | | 1.90E-07 | 0.62 | 1.32 | 4.69E-01 | -0.16 | 1.19 | 5.48E-04 | 0.54 | 1.39 | 1.15E-04 | 0.70 | 1.42 | 26217 | 17559 | 601142 | 1025753 | 675918 | 1095614 | 526365 | 956049 |
| RP_POS_613_47931a13_873 | RP | POS | 13.87 | 613.4793 | | | | 3.14E-01 | 0.16 | 0.66 | 4.69E-01 | 0.16 | 0.43 | 3.02E-01 | 0.24 | 0.47 | 5.67E-01 | 0.08 | 0.41 | 517395 | 648886 | 673761 | 1035498 | 598174 | 569671 | 749347 | 1352854 |
| HILIC_NEG_141_92378a4_274 | HILIC | NEG | 4.274 | 141.9238 | | | | 5.28E-01 | -0.12 | 0.63 | 4.69E-01 | -0.15 | 0.62 | 3.55E-01 | -0.20 | 0.48 | 8.32E-01 | -0.05 | 0.15 | 2842854 | 787452 | 2738635 | 868906 | 2801928 | 931829 | 2675342 | 805557 |
| RP_NEG_394_89191a14_25 | RP | NEG | 14.25 | 394.8919 | | | | 7.49E-01 | 0.06 | 0.24 | 4.69E-01 | 0.15 | 0.64 | 5.28E-01 | 0.13 | 0.37 | 9.20E-01 | -0.02 | 0.39 | 2286814 | 797340 | 2343349 | 1052066 | 2266739 | 1004738 | 2419960 | 1102178 |
| HILIC_NEG_287_70486a1_362 | HILIC | NEG | 1.362 | 287.7049 | | | | 3.74E-11 | 0.90 | 1.62 | 4.70E-01 | -0.15 | 0.42 | 2.00E-05 | 0.83 | 1.45 | 2.41E-09 | 0.98 | 2.16 | 1337033 | 189052 | 1775952 | 505483 | 1812743 | 433598 | 1739161 | 570500 |
| RP_POS_261_14444a3_445 | RP | POS | 3.45 | 261.1444 | | | | 3.86E-03 | 0.44 | 0.83 | 4.70E-01 | 0.16 | 1.05 | 1.57E-02 | 0.52 | 0.91 | 2.04E-02 | 0.37 | 0.84 | 979300 | 398203 | 1279057 | 727833 | 1226084 | 544114 | 1332031 | 876644 |
| RP_POS_599_45172a15_1 | RP | POS | 15.10 | 599.4517 | | | | 4.08E-01 | 0.18 | 0.43 | 4.70E-01 | -0.14 | 0.51 | 6.45E-01 | 0.11 | 0.69 | 2.97E-01 | 0.25 | 0.48 | 2297570 | 786359 | 2427553 | 703355 | 2478697 | 716687 | 2376408 | 693206 |
| RP_NEG_826_5979a10_94 | RP | NEG | 10.94 | 826.5979 | | | | 5.07E-08 | 0.92 | 1.63 | 4.70E-01 | 0.14 | 0.44 | 1.18E-06 | 1.00 | 1.66 | 6.05E-06 | 0.85 | 1.64 | 1133810 | 458084 | 1772865 | 683729 | 1723190 | 640288 | 1822541 | 727673 |
| HILIC_POS_125_02105a4_861 | HILIC | POS | 4.861 | 125.0211 | | | | 1.39E-02 | 0.38 | 0.87 | 4.70E-01 | 0.16 | 0.75 | 1.65E-02 | 0.46 | 0.81 | 1.05E-01 | 0.31 | 0.66 | 1094940 | 249711 | 1250885 | 438249 | 1219050 | 435324 | 1282720 | 443249 |
| RP_POS_342_18396a5_683 | RP | POS | 5.68 | 342.1840 | | | | 8.70E-01 | 0.03 | 0.67 | 4.70E-01 | 0.15 | 0.58 | 5.89E-01 | 0.11 | 0.59 | 8.57E-01 | -0.04 | 0.47 | 3323361 | 1560416 | 3377011 | 1749029 | 3249866 | 2100598 | 3504156 | 1317246 |
| RP_POS_553_45905a13_932 | RP | POS | 13.93 | 553.4591 | | | | 4.09E-01 | -0.18 | 0.39 | 4.71E-01 | -0.14 | 0.61 | 2.54E-01 | -0.25 | 0.51 | 6.76E-01 | -0.11 | 0.48 | 12919109 | 2669627 | 12476017 | 2460289 | 12654805 | 2960008 | 12297229 | 1844639 |
| RP_NEG_829_18097a9_373 | RP | NEG | 9.37 | 829.1810 | | | | 3.78E-01 | 0.15 | 0.51 | 4.71E-01 | -0.16 | 0.52 | 7.19E-01 | 0.07 | 0.25 | 2.56E-01 | 0.23 | 0.51 | 5223433 | 326360 | 5289130 | 465805 | 5322940 | 459460 | 5255320 | 474280 |
| HILIC_NEG_353_01108a6_639 | HILIC | NEG | 6.639 | 353.0111 | | | | 3.15E-01 | -0.20 | 0.57 | 4.71E-01 | 0.15 | 0.64 | 5.47E-01 | -0.13 | 0.64 | 2.38E-01 | -0.28 | 0.57 | 1850947 | 252397 | 1798657 | 257157 | 1780005 | 279213 | 1817309 | 234392 |
| HILIC_POS_84_9119a2_83 | HILIC | POS | 2.83 | 84.9119 | | | | 8.13E-06 | 0.56 | 1.41 | 4.71E-01 | -0.16 | 0.64 | 2.80E-03 | 0.48 | 1.17 | 5.49E-04 | 0.64 | 1.86 | 4731689 | 700217 | 6287600 | 3102178 | 6512508 | 3319727 | 6062693 | 2884371 |
| RP_NEG_260_98041a0_552 | RP | NEG | 0.55 | 260.9804 | | | | 3.38E-03 | 0.41 | 0.76 | 4.71E-01 | -0.16 | 0.83 | 8.38E-02 | 0.33 | 0.77 | 3.24E-03 | 0.49 | 0.95 | 3012302 | 667082 | 3592027 | 1537942 | 3703537 | 1373942 | 3480518 | 1692841 |
| HILIC_NEG_297_73328a0_552 | HILIC | NEG | 0.552 | 297.7333 | | | | 3.71E-01 | -0.19 | 0.37 | 4.71E-01 | -0.14 | 0.75 | 2.65E-01 | -0.26 | 0.43 | 6.10E-01 | -0.12 | 0.27 | 186839 | 110346 | 166948 | 102353 | 174366 | 103178 | 159529 | 102020 |
| RP_NEG_327_25543a10_401 | RP | NEG | 10.40 | 327.2554 | FA 19:1;2O | 327.2529(100), 265.2538(60), 309.2448(35) | 3 | 1.28E-01 | -0.33 | 0.57 | 4.71E-01 | 0.14 | 0.45 | 2.60E-01 | -0.26 | 0.51 | 1.00E-01 | -0.40 | 0.71 | 1372103 | 392429 | 1251466 | 353949 | 1225811 | 379384 | 1277120 | 328392 |
| RP_POS_565_42505a11_156 | RP | POS | 11.16 | 565.4251 | | | | 7.93E-01 | -0.06 | 0.74 | 4.71E-01 | 0.14 | 0.48 | 9.76E-01 | 0.01 | 0.55 | 6.11E-01 | -0.13 | 0.44 | 2096937 | 1806928 | 2004495 | 1431724 | 1900727 | 1497191 | 2108262 | 1370381 |
| RP_POS_384_2355a6_067 | RP | POS | 6.07 | 384.2355 | | | | 1.71E-07 | 0.62 | 1.32 | 4.72E-01 | -0.16 | 1.19 | 5.31E-04 | 0.54 | 1.40 | 1.07E-04 | 0.70 | 1.43 | 61595 | 25553 | 997595 | 1662867 | 1118018 | 1772016 | 877173 | 1554687 |
| RP_POS_540_44635a10_878 | RP | POS | 10.88 | 540.4464 | | | | 1.14E-03 | -0.74 | 1.43 | 4.72E-01 | 0.13 | 0.66 | 4.73E-03 | -0.67 | 1.09 | 1.09E-03 | -0.80 | 1.41 | 7614113 | 1108684 | 6861564 | 925390 | 6794568 | 963103 | 6928560 | 890754 |

**Supplementary table 1: Metabolomics results** (in asceding order according to p-value from progressive ALD vs non-progressive ALD comparison, with molecular features with ID_level of 1-2 first)

| | | | | | | | | all_ALD_vs_control | | | Prog_ALD_vs_non-prog_ALD | | | Prog_ALD_vs_control_ALD | | | Non-prog_ALD_vs_control | | | Healthy_control | | all_ALD | | Non-prog_ALD | | Progressive_ALD | |
|---|---|---|---|---|---|---|---|---|---|---|---|---|---|---|---|---|---|---|---|---|---|---|---|---|---|---|---|
| | | | | | | | | t-test | Cohen's | PLS-DA | t-test | Cohen's | PLS-DA | t-test | Cohen's | PLS-DA | t-test | Cohen's | PLS-DA | | | | | | | | |
| Feature_ID | RP_HILIC | POS_NEG | Average.Rt | Average.Mz | Metabolite.name | curated_MSMS | ID_level | p | d | VIP | p | d | VIP | p | d | VIP | p | d | VIP | Mean | SD | Mean | SD | Mean | SD | Mean | SD |
| RP_POS_605_27509a10_331 | RP | POS | 10.33 | 605.2751 | | | | 2.96E-02 | -0.50 | 1.08 | 4.72E-01 | 0.13 | 0.47 | 8.06E-02 | -0.43 | 0.87 | 2.10E-02 | -0.57 | 1.12 | 640391 | 309688 | 505074 | 250210 | 486961 | 242018 | 523187 | 259318 |
| RP_POS_263_99258a0_614 | RP | POS | 0.61 | 263.9926 | | | | 2.87E-07 | 0.66 | 1.30 | 4.72E-01 | -0.16 | 1.15 | 4.17E-04 | 0.58 | 1.35 | 6.84E-05 | 0.74 | 1.43 | 634410 | 194243 | 1060895 | 708340 | 1112166 | 749871 | 1009625 | 667878 |
| HILIC_POS_146_09219a3_841 | HILIC | POS | 3.841 | 146.0922 | | | | 5.41E-04 | 0.43 | 0.69 | 4.72E-01 | 0.16 | 0.69 | 5.65E-03 | 0.51 | 1.02 | 2.50E-02 | 0.35 | 0.70 | 288702 | 312206 | 799003 | 1326514 | 703018 | 1212445 | 894989 | 1437485 |
| RP_NEG_127_869a15_551 | RP | NEG | 15.55 | 127.8690 | | | | 3.44E-01 | 0.19 | 0.66 | 4.72E-01 | -0.14 | 0.52 | 5.98E-01 | 0.12 | 0.32 | 2.36E-01 | 0.27 | 0.48 | 695174 | 153520 | 724911 | 152408 | 735938 | 145621 | 713885 | 159614 |
| RP_NEG_329_26956a11_611 | RP | NEG | 11.61 | 329.2696 | | | | 8.99E-02 | -0.33 | 0.86 | 4.72E-01 | -0.15 | 0.96 | 5.15E-02 | -0.40 | 0.86 | 2.72E-01 | -0.25 | 0.80 | 3062551 | 882284 | 2742985 | 1001291 | 2815439 | 1130569 | 2670531 | 858330 |
| RP_NEG_426_9682a11_253 | RP | NEG | 11.25 | 426.9682 | | | | 7.71E-01 | 0.07 | 0.67 | 4.72E-01 | 0.13 | 0.43 | 6.32E-01 | 0.14 | 0.68 | 9.63E-01 | 0.01 | 0.50 | 23948551 | 15276997 | 24790893 | 9824100 | 24079268 | 6065949 | 25502517 | 12536598 |
| RP_POS_567_44666a10_881 | RP | POS | 10.88 | 567.4467 | | | | 4.06E-02 | -0.40 | 0.70 | 4.72E-01 | 0.15 | 0.51 | 1.23E-01 | -0.33 | 0.64 | 3.62E-02 | -0.48 | 0.73 | 1585470 | 272150 | 1467252 | 294506 | 1445957 | 312917 | 1488546 | 276398 |
| RP_POS_745_41852a5_94 | RP | POS | 5.94 | 745.4185 | | | | 2.04E-07 | 0.62 | 1.32 | 4.73E-01 | -0.16 | 1.19 | 5.38E-04 | 0.54 | 1.40 | 1.26E-04 | 0.70 | 1.42 | 7364 | 4036 | 291299 | 508186 | 328040 | 544417 | 254558 | 471839 |
| RP_NEG_153_86746a15_592 | RP | NEG | 15.59 | 153.8675 | | | | 9.55E-01 | -0.01 | 0.29 | 4.73E-01 | -0.15 | 0.43 | 7.00E-01 | -0.08 | 0.30 | 7.95E-01 | 0.06 | 0.20 | 818908 | 42151 | 818419 | 42567 | 821495 | 46535 | 815343 | 38420 |
| RP_POS_1074_18921a10_932 | RP | POS | 10.93 | 1074.1892 | | | | 1.51E-03 | -0.76 | 1.39 | 4.73E-01 | -0.13 | 1.23 | 1.38E-03 | -0.82 | 1.33 | 4.85E-03 | -0.70 | 1.25 | 399005 | 218190 | 257081 | 162015 | 268783 | 152338 | 245379 | 171892 |
| RP_POS_432_33209a10_242 | RP | POS | 10.24 | 432.3321 | | | | 3.81E-07 | 0.61 | 1.24 | 4.73E-01 | -0.16 | 1.11 | 8.11E-04 | 0.53 | 1.28 | 1.56E-04 | 0.69 | 1.36 | 99582 | 83342 | 1155792 | 1936500 | 1295503 | 2061391 | 1016081 | 1813128 |
| HILIC_NEG_251_86798a7_667 | HILIC | NEG | 7.667 | 251.8680 | | | | 3.35E-01 | 0.17 | 0.73 | 4.73E-01 | 0.15 | 0.70 | 2.48E-01 | 0.25 | 0.64 | 6.32E-01 | 0.10 | 0.27 | 361822 | 30859 | 368428 | 40571 | 365501 | 37924 | 371355 | 43242 |
| RP_NEG_270_7984a0_521 | RP | NEG | 0.52 | 270.7984 | | | | 4.07E-04 | 0.45 | 1.21 | 4.74E-01 | -0.16 | 1.16 | 2.05E-02 | 0.37 | 1.17 | 3.87E-03 | 0.53 | 1.33 | 1925498 | 312882 | 2365792 | 1079576 | 2443655 | 1154611 | 2287930 | 1004617 |
| RP_POS_294_64435a10_514 | RP | POS | 10.51 | 294.6444 | | | | 4.22E-03 | -0.58 | 1.19 | 4.74E-01 | 0.14 | 0.65 | 2.71E-02 | -0.50 | 1.11 | 2.96E-03 | -0.65 | 1.13 | 1964642 | 425733 | 1703225 | 447113 | 1670986 | 410701 | 1735464 | 482807 |
| HILIC_POS_581_23901a0_633 | HILIC | POS | 0.633 | 581.2390 | | | | 8.69E-01 | 0.03 | 0.58 | 4.74E-01 | 0.15 | 0.88 | 6.55E-01 | 0.11 | 0.19 | 8.48E-01 | -0.04 | 0.08 | 782004 | 435521 | 796811 | 457368 | 763826 | 386484 | 829796 | 520624 |
| RP_POS_350_02948a4_851 | RP | POS | 4.85 | 350.0295 | | | | 5.87E-01 | 0.10 | 0.51 | 4.74E-01 | -0.15 | 0.56 | 9.10E-01 | 0.02 | 0.42 | 4.25E-01 | 0.17 | 0.55 | 13342225 | 5727633 | 14022135 | 7262224 | 14545610 | 7835477 | 13498660 | 6678487 |
| RP_POS_362_33905a12_101 | RP | POS | 12.10 | 362.3391 | | | | 8.10E-01 | -0.05 | 0.59 | 4.74E-01 | -0.15 | 0.44 | 5.65E-01 | -0.12 | 0.35 | 8.99E-01 | 0.03 | 0.36 | 1733474 | 1158178 | 1674406 | 1327800 | 1770089 | 1436534 | 1578724 | 1216459 |
| HILIC_NEG_171_02756a0_93 | HILIC | NEG | 0.93 | 171.0276 | | | | 1.76E-05 | 0.67 | 1.16 | 4.75E-01 | -0.15 | 1.23 | 3.38E-03 | 0.59 | 0.97 | 2.77E-05 | 0.74 | 1.40 | 246286 | 105855 | 367207 | 191294 | 380973 | 168376 | 353440 | 212595 |
| RP_POS_275_9928a4_852 | RP | POS | 4.85 | 275.9928 | | | | 6.03E-01 | 0.09 | 0.48 | 4.75E-01 | -0.15 | 0.54 | 9.26E-01 | 0.02 | 0.43 | 4.37E-01 | 0.17 | 0.56 | 647019 | 282577 | 679162 | 357866 | 704912 | 386960 | 653413 | 328113 |
| RP_POS_564_79413a0_5 | RP | POS | 0.50 | 564.7941 | | | | 4.50E-02 | -0.34 | 1.01 | 4.75E-01 | -0.15 | 1.21 | 3.65E-02 | -0.41 | 0.99 | 1.96E-01 | -0.26 | 1.00 | 494613 | 197538 | 401567 | 294197 | 422727 | 301245 | 380407 | 288457 |
| RP_POS_357_34729a9_476 | RP | POS | 9.48 | 357.3473 | | | | 3.87E-01 | -0.23 | 0.83 | 4.75E-01 | -0.12 | 0.55 | 2.93E-01 | -0.29 | 0.67 | 5.44E-01 | -0.17 | 0.51 | 1957290 | 3090697 | 1453554 | 1805111 | 1583342 | 1941512 | 1323765 | 1667184 |
| RP_NEG_432_85736a0_499 | RP | NEG | 0.50 | 432.8574 | | | | 2.28E-02 | -0.32 | 1.11 | 4.75E-01 | -0.16 | 1.29 | 1.93E-02 | -0.40 | 1.17 | 1.99E-01 | -0.25 | 1.15 | 1895665 | 414747 | 1622410 | 930326 | 1689253 | 998783 | 1555566 | 861390 |
| RP_POS_301_03748a0_258 | RP | POS | 0.26 | 301.0375 | | | | 4.33E-01 | -0.12 | 0.48 | 4.75E-01 | 0.16 | 0.52 | 7.99E-01 | -0.04 | 0.32 | 3.52E-01 | -0.20 | 0.54 | 3418144 | 201483 | 3377562 | 372101 | 3350814 | 441029 | 3404311 | 289442 |
| RP_POS_296_17584a6_887 | RP | POS | 6.89 | 296.1758 | | | | 3.63E-02 | -0.30 | 0.76 | 4.75E-01 | -0.16 | 0.50 | 2.61E-03 | -0.38 | 1.07 | 3.25E-01 | -0.22 | 0.71 | 2475284 | 686021 | 2067856 | 1492266 | 2175264 | 1961481 | 1960449 | 792599 |
| RP_NEG_190_92766a15_653 | RP | NEG | 15.65 | 190.9277 | | | | 8.88E-02 | 0.36 | 0.69 | 4.75E-01 | -0.14 | 0.61 | 2.02E-01 | 0.29 | 0.62 | 6.85E-02 | 0.43 | 0.82 | 12094770 | 2671444 | 13019454 | 2463035 | 13196372 | 2554350 | 12842535 | 2380821 |
| HILIC_POS_146_08133a4_885 | HILIC | POS | 4.885 | 146.0813 | | | | 5.12E-01 | -0.11 | 0.37 | 4.75E-01 | 0.15 | 1.31 | 8.83E-01 | -0.03 | 0.24 | 2.66E-01 | -0.19 | 0.72 | 4742644 | 3708508 | 4182834 | 5420947 | 3793163 | 3783322 | 4572505 | 6689518 |
| RP_POS_180_13617a7_649 | RP | POS | 7.65 | 180.1362 | | | | 7.04E-03 | -0.51 | 0.84 | 4.76E-01 | -0.15 | 0.68 | 1.09E-02 | -0.58 | 0.93 | 2.82E-02 | -0.43 | 0.84 | 8540728 | 372773 | 8319269 | 443161 | 8351101 | 374095 | 8287437 | 504753 |
| RP_POS_131_96158a15_405 | RP | POS | 15.41 | 131.9616 | | | | 2.07E-01 | 0.25 | 0.46 | 4.76E-01 | -0.15 | 0.44 | 4.26E-01 | 0.18 | 0.36 | 1.47E-01 | 0.32 | 0.70 | 28631879 | 8532322 | 30886596 | 9160612 | 31544329 | 9124436 | 30228862 | 9241646 |
| RP_POS_158_15396a7_911 | RP | POS | 7.91 | 158.1540 | | | | 6.32E-01 | 0.11 | 0.83 | 4.76E-01 | -0.14 | 0.45 | 8.87E-01 | 0.04 | 0.65 | 4.45E-01 | 0.17 | 0.62 | 3060001 | 632388 | 3120102 | 552326 | 3159758 | 462638 | 3080446 | 631752 |
| HILIC_NEG_127_87013a3_059 | HILIC | NEG | 3.059 | 127.8701 | | | | 3.90E-02 | 0.34 | 1.18 | 4.76E-01 | 0.15 | 0.85 | 2.96E-02 | 0.42 | 1.23 | 1.92E-01 | 0.26 | 1.23 | 476047 | 66991 | 508991 | 103158 | 501589 | 108713 | 516394 | 97826 |
| HILIC_POS_387_21368a5_921 | HILIC | POS | 5.921 | 387.2137 | | | | 3.79E-01 | -0.17 | 0.99 | 4.76E-01 | 0.15 | 0.82 | 6.62E-01 | -0.09 | 0.69 | 2.70E-01 | -0.24 | 0.94 | 1473286 | 333440 | 1411011 | 382116 | 1383591 | 389680 | 1438431 | 376325 |
| RP_POS_176_936a0_497 | RP | POS | 0.50 | 176.9360 | | | | 7.12E-07 | -0.61 | 1.31 | 4.76E-01 | 0.16 | 1.18 | 7.39E-04 | -0.53 | 1.35 | 2.16E-04 | -0.68 | 1.43 | 827364 | 48180 | 670508 | 286434 | 649959 | 309815 | 691057 | 262520 |
| RP_POS_177_00838a15_337 | RP | POS | 15.34 | 177.0084 | | | | 7.37E-01 | 0.07 | 0.78 | 4.76E-01 | 0.14 | 0.81 | 5.34E-01 | 0.14 | 0.42 | 9.95E-01 | 0.00 | 1.14 | 6641843 | 2540607 | 6814907 | 2461514 | 6638332 | 2575936 | 6991481 | 2354290 |
| HILIC_NEG_176_99812a0_7 | HILIC | NEG | 0.7 | 176.9981 | | | | 2.69E-04 | 0.59 | 1.04 | 4.76E-01 | -0.15 | 0.83 | 9.84E-03 | 0.52 | 0.99 | 4.00E-04 | 0.67 | 1.21 | 600823 | 167182 | 752937 | 269726 | 772281 | 252846 | 733592 | 286870 |
| HILIC_NEG_136_89169a1_409 | HILIC | NEG | 1.409 | 136.8917 | | | | 7.39E-01 | -0.05 | 0.86 | 4.76E-01 | 0.16 | 0.79 | 8.58E-01 | 0.03 | 0.14 | 4.60E-01 | -0.13 | 0.32 | 4794878 | 278782 | 4767340 | 661385 | 4719911 | 622379 | 4814769 | 701314 |
| RP_POS_406_74786a13_153 | RP | POS | 13.15 | 406.7479 | | | | 6.89E-02 | 0.42 | 0.72 | 4.76E-01 | 0.13 | 0.56 | 6.57E-02 | 0.49 | 0.73 | 1.24E-01 | 0.36 | 0.75 | 18305811 | 5993300 | 20468800 | 4717584 | 20130373 | 3412804 | 20807227 | 5751906 |
| RP_POS_419_31503a13_796 | RP | POS | 13.80 | 419.3150 | | | | 1.04E-01 | 0.37 | 0.89 | 4.77E-01 | 0.14 | 0.49 | 7.33E-02 | 0.43 | 0.83 | 2.24E-01 | 0.30 | 0.78 | 5327371 | 2142371 | 6022213 | 1789924 | 5894035 | 1855330 | 6150391 | 1731261 |
| HILIC_NEG_335_65012a1_364 | HILIC | NEG | 1.364 | 335.6501 | | | | 6.45E-07 | 0.77 | 1.30 | 4.77E-01 | -0.15 | 0.70 | 5.00E-04 | 0.70 | 1.16 | 1.79E-06 | 0.85 | 1.60 | 267517 | 71186 | 363595 | 129179 | 372844 | 113823 | 354346 | 143474 |
| RP_POS_820_54364a11_616 | RP | POS | 11.62 | 820.5436 | | | | 6.56E-02 | 0.25 | 0.61 | 4.77E-01 | -0.16 | 0.62 | 3.08E-01 | 0.17 | 0.45 | 7.57E-02 | 0.33 | 0.78 | 641466 | 731765 | 1063202 | 1865963 | 1196765 | 1975427 | 929638 | 1759538 |
| RP_NEG_207_96753a13_291 | RP | NEG | 13.29 | 207.9675 | | | | 2.26E-01 | -0.25 | 0.84 | 4.77E-01 | 0.14 | 0.81 | 4.42E-01 | -0.18 | 0.74 | 1.51E-01 | -0.32 | 0.62 | 4237802 | 794105 | 4041444 | 769855 | 3986338 | 715529 | 4096549 | 824178 |
| RP_POS_426_82114a0_5 | RP | POS | 0.50 | 426.8211 | | | | 1.09E-01 | -0.23 | 1.05 | 4.77E-01 | -0.16 | 1.25 | 7.17E-02 | -0.31 | 1.03 | 4.30E-01 | -0.15 | 1.10 | 1410826 | 417150 | 1223274 | 895830 | 1287365 | 967279 | 1159183 | 823037 |
| RP_NEG_718_85681a0_465 | RP | NEG | 0.47 | 718.8568 | | | | 1.23E-09 | -0.80 | 1.42 | 4.77E-01 | 0.15 | 1.14 | 4.72E-05 | -0.72 | 1.44 | 7.75E-07 | -0.87 | 1.57 | 523643 | 70776 | 354734 | 225575 | 338598 | 221597 | 370871 | 230585 |
| RP_POS_445_3678a11_953 | RP | POS | 11.95 | 445.3678 | | | | 9.30E-01 | -0.02 | 0.85 | 4.77E-01 | -0.14 | 0.69 | 7.08E-01 | -0.09 | 0.73 | 8.28E-01 | 0.05 | 0.35 | 874796 | 359704 | 868465 | 327926 | 891920 | 323899 | 845010 | 333514 |
| HILIC_POS_509_31198a0_653 | HILIC | POS | 0.653 | 509.3120 | | | | 1.63E-06 | 0.59 | 1.31 | 4.77E-01 | -0.16 | 0.86 | 1.25E-03 | 0.51 | 1.48 | 2.59E-04 | 0.67 | 1.47 | 77594 | 298956 | 875240 | 1489893 | 981774 | 1592099 | 768706 | 1388136 |
| RP_POS_203_1209a6_498 | RP | POS | 6.50 | 203.1209 | | | | 6.60E-01 | 0.09 | 0.42 | 4.78E-01 | -0.14 | 0.61 | 9.28E-01 | 0.02 | 0.33 | 4.91E-01 | 0.16 | 0.62 | 772477 | 314762 | 800522 | 304533 | 822292 | 322548 | 778753 | 287000 |
| RP_POS_263_23691a9_476 | RP | POS | 9.48 | 263.2369 | | | | 3.41E-01 | -0.27 | 0.88 | 4.78E-01 | -0.11 | 0.49 | 2.66E-01 | -0.32 | 0.70 | 4.69E-01 | -0.21 | 0.57 | 2581497 | 4730017 | 1741486 | 2440069 | 1915886 | 2548818 | 1567086 | 2338983 |
| RP_POS_432_28046a4_512 | RP | POS | 4.51 | 432.2805 | | | | 9.11E-01 | 0.03 | 1.22 | 4.78E-01 | -0.12 | 1.10 | 9.23E-01 | -0.03 | 0.94 | 7.58E-01 | 0.09 | 1.04 | 1200787 | 3872448 | 1281767 | 2146327 | 1435171 | 2276010 | 1128363 | 2019705 |
| RP_POS_848_53931a13_163 | RP | POS | 13.16 | 848.5393 | | | | 6.89E-01 | -0.08 | 0.30 | 4.78E-01 | -0.14 | 0.47 | 5.06E-01 | -0.15 | 0.47 | 9.54E-01 | -0.01 | 0.48 | 10209642 | 3763924 | 9906062 | 3572494 | 10161337 | 3598685 | 9650788 | 3564039 |
| RP_POS_312_17068a5_873 | RP | POS | 5.87 | 312.1707 | | | | 1.81E-01 | 0.16 | 0.63 | 4.78E-01 | -0.16 | 0.63 | 1.50E-01 | 0.08 | 0.64 | 2.89E-01 | 0.25 | 0.72 | 539994 | 701750 | 1008641 | 3251566 | 1241570 | 4550934 | 775712 | 734445 |
| RP_POS_675_67456a11_668 | RP | POS | 11.67 | 675.6746 | 2 M 13-Docosenamide | 338.3418(100), 321.3158(20), 303.3019(14) | | 8.58E-01 | 0.04 | 0.69 | 4.79E-01 | -0.15 | 0.42 | 8.63E-01 | -0.04 | 0.38 | 6.32E-01 | 0.11 | 0.46 | 26166921 | 24082533 | 27063619 | 26052133 | 28921377 | 27029650 | 25205861 | 25172072 |
| RP_POS_469_36386a11_237 | RP | POS | 11.24 | 469.3639 | | | | 4.45E-01 | -0.13 | 0.71 | 4.79E-01 | -0.15 | 0.64 | 3.74E-01 | -0.21 | 0.60 | 7.42E-01 | -0.06 | 0.31 | 2031387 | 864769 | 1881288 | 1221116 | 1968457 | 799624 | 1794120 | 1535504 |
| RP_POS_780_55035a11_611 | RP | POS | 11.61 | 780.5504 | | | | 7.59E-01 | -0.06 | 0.54 | 4.79E-01 | 0.14 | 0.72 | 9.67E-01 | 0.01 | 0.35 | 5.43E-01 | -0.13 | 0.65 | 964988 | 254277 | 949001 | 258794 | 930565 | 240159 | 967437 | 277391 |
| RP_POS_277_89148a15_437 | RP | POS | 15.44 | 277.8915 | | | | 7.31E-02 | -0.25 | 0.55 | 4.79E-01 | 0.16 | 0.55 | 2.81E-01 | -0.17 | 0.49 | 9.93E-02 | -0.33 | 0.60 | 3314774 | 171378 | 3221545 | 417314 | 3191811 | 474062 | 3251279 | 354001 |
| RP_POS_624_63947a15_221 | RP | POS | 15.22 | 624.6395 | | | | 4.61E-02 | 0.36 | 0.74 | 4.79E-01 | 0.15 | 0.42 | 3.17E-02 | 0.43 | 0.81 | 1.88E-01 | 0.28 | 0.62 | 1690176 | 543996 | 1933886 | 717153 | 1882802 | 767505 | 1984969 | 666877 |
| RP_NEG_268_80139a0_521 | RP | NEG | 0.52 | 268.8014 | | | | 4.74E-04 | 0.44 | 1.21 | 4.79E-01 | -0.16 | 1.16 | 2.25E-02 | 0.36 | 1.17 | 4.57E-03 | 0.52 | 1.33 | 3018220 | 451933 | 3678067 | 1657005 | 3796078 | 1777177 | 3560056 | 1536419 |
| RP_POS_250_17767a7_801 | RP | POS | 7.80 | 250.1777 | | | | 9.73E-02 | 0.29 | 0.67 | 4.79E-01 | -0.15 | 0.44 | 2.58E-01 | 0.22 | 0.50 | 9.26E-02 | 0.37 | 0.65 | 519611 | 371610 | 658488 | 501156 | 694179 | 556917 | 622796 | 441232 |
| RP_POS_313_27325a10_541 | RP | POS | 10.54 | 313.2733 | | | | 4.65E-02 | -0.38 | 0.88 | 4.79E-01 | 0.15 | 0.80 | 1.47E-01 | -0.30 | 0.75 | 4.17E-02 | -0.45 | 0.89 | 917097 | 130604 | 860753 | 153570 | 849819 | 161462 | 871687 | 146064 |
| RP_POS_536_3703a10_823 | RP | POS | 10.82 | 536.3703 | | | | 6.58E-01 | 0.09 | 0.75 | 4.79E-01 | 0.15 | 0.90 | 5.04E-01 | 0.16 | 0.44 | 9.44E-01 | 0.01 | 0.79 | 1271907 | 601560 | 1327370 | 651249 | 1280984 | 519620 | 1373755 | 763223 |
| RP_POS_172_98991a0_249 | RP | POS | 0.25 | 172.9899 | | | | 4.16E-01 | -0.17 | 0.46 | 4.79E-01 | -0.14 | 0.42 | 2.74E-01 | -0.24 | 0.57 | 6.83E-01 | -0.10 | 0.24 | 1299551 | 254806 | 1257436 | 246789 | 1275009 | 278623 | 1239864 | 211641 |
| RP_NEG_554_72217a15_49 | RP | NEG | 15.49 | 554.7222 | | | | 1.60E-01 | 0.30 | 1.02 | 4.79E-01 | -0.14 | 0.42 | 3.30E-01 | 0.22 | 0.59 | 1.16E-01 | 0.37 | 0.86 | 1919414 | 66072 | 1938382 | 63348 | 1942891 | 63692 | 1933874 | 63321 |
| RP_POS_806_56842a12_799 | RP | POS | 12.80 | 806.5684 | | | | 8.31E-03 | -0.70 | 1.31 | 4.79E-01 | -0.11 | 0.57 | 5.69E-03 | -0.76 | 1.38 | 2.01E-02 | -0.65 | 0.92 | 42559261 | 24722840 | 29744044 | 14424277 | 30770610 | 15632789 | 28717477 | 13184493 |
| RP_POS_291_1944a9_075 | RP | POS | 9.08 | 291.1944 | | | | 4.35E-02 | -0.46 | 0.94 | 4.80E-01 | 0.13 | 0.95 | 1.15E-01 | -0.39 | 0.93 | 2.92E-02 | -0.53 | 0.86 | 2182178 | 74137 | 2152167 | 60894 | 2147835 | 56168 | 2156499 | 65566 |
| RP_NEG_470_19827a11_61 | RP | NEG | 11.61 | 470.1983 | | | | 3.13E-02 | -0.41 | 1.08 | 4.80E-01 | -0.14 | 0.93 | 2.08E-02 | -0.49 | 1.04 | 1.27E-01 | -0.34 | 0.93 | 2024174 | 229173 | 1917894 | 260677 | 1936421 | 281997 | 1899366 | 238890 |
| HILIC_POS_296_17023a0_554 | HILIC | POS | 0.554 | 296.1702 | | | | 7.08E-05 | 0.50 | 1.00 | 4.80E-01 | -0.16 | 0.64 | 7.55E-03 | 0.42 | 1.13 | 1.96E-03 | 0.58 | 1.04 | 468955 | 33609 | 531692 | 140560 | 541682 | 152648 | 521703 | 128110 |
| RP_POS_144_08049a2_488 | RP | POS | 2.49 | 144.0805 | | | | 5.12E-03 | -0.50 | 1.10 | 4.80E-01 | -0.15 | 0.92 | 9.82E-03 | -0.57 | 1.12 | 2.45E-02 | -0.43 | 0.99 | 1938832 | 343688 | 1719300 | 455005 | 1751636 | 384003 | 1686965 | 518356 |
| RP_NEG_125_87193a15_551 | RP | NEG | 15.55 | 125.8719 | | | | 9.66E-01 | -0.01 | 0.23 | 4.81E-01 | 0.14 | 0.48 | 7.73E-01 | 0.06 | 0.32 | 7.27E-01 | -0.08 | 0.34 | 1152022 | 242266 | 1149890 | 252537 | 1131965 | 267355 | 1167814 | 238154 |
| RP_NEG_482_79044a15_487 | RP | NEG | 15.49 | 482.7904 | | | | 4.24E-02 | 0.44 | 0.69 | 4.81E-01 | 0.14 | 0.70 | 3.25E-02 | 0.51 | 0.86 | 1.14E-01 | 0.37 | 0.74 | 2791665 | 86087 | 2827312 | 77951 | 2821785 | 78042 | 2832839 | 78253 |
| RP_NEG_944_80957a0_486 | RP | NEG | 0.49 | 944.8096 | | | | 3.74E-04 | 0.45 | 1.16 | 4.81E-01 | 0.16 | 1.25 | 3.42E-03 | 0.53 | 1.23 | 2.44E-02 | 0.37 | 1.23 | 1681255 | 341079 | 2219919 | 1344421 | 2124629 | 1288234 | 2315209 | 1404901 |
| RP_NEG_164_07066a2_198 | RP | NEG | 2.20 | 164.0707 | | | | 8.87E-01 | -0.03 | 0.69 | 4.82E-01 | 0.14 | 0.78 | 8.78E-01 | 0.04 | 0.52 | 6.51E-01 | -0.10 | 0.70 | 765120 | 239601 | 758290 | 217286 | 742895 | 172702 | 773686 | 255109 |
| RP_NEG_190_92766a13_28 | RP | NEG | 13.28 | 190.9277 | | | | 6.26E-01 | -0.09 | 0.72 | 4.82E-01 | 0.15 | 0.51 | 9.36E-01 | -0.02 | 0.52 | 4.28E-01 | -0.16 | 0.51 | 3098190 | 465230 | 3049656 | 552798 | 3010511 | 517005 | 3088801 | 589055 |
| HILIC_NEG_132_92337a4_215 | HILIC | NEG | 4.215 | 132.9234 | | | | 3.93E-01 | -0.16 | 0.85 | 4.82E-01 | 0.15 | 0.88 | 6.75E-01 | -0.09 | 0.44 | 2.93E-01 | -0.23 | 0.37 | 7854647 | 1488360 | 7581694 | 1771618 | 7456244 | 1903519 | 7707143 | 1638845 |
| RP_POS_628_85773a0_471 | RP | POS | 0.47 | 628.8577 | | | | 1.52E-09 | -0.78 | 1.41 | 4.82E-01 | 0.15 | 1.15 | 6.31E-05 | -0.70 | 1.44 | 1.25E-06 | -0.85 | 1.57 | 696222 | 83877 | 477697 | 301038 | 456394 | 296955 | 499000 | 306577 |
| RP_POS_410_75156a13_369 | RP | POS | 13.37 | 410.7516 | | | | 5.09E-01 | -0.13 | 0.50 | 4.82E-01 | -0.15 | 0.60 | 3.40E-01 | -0.20 | 0.42 | 8.14E-01 | -0.05 | 0.27 | 1004966 | 357617 | 954909 | 409911 | 983917 | 445378 | 925901 | 373369 |
| HILIC_NEG_165_04167a0_858 | HILIC | NEG | 0.858 | 165.0417 | | | | 1.06E-03 | -0.88 | 1.80 | 4.82E-01 | 0.11 | 0.47 | 3.49E-03 | -0.82 | 1.32 | 5.97E-04 | -0.93 | 1.88 | 469459 | 401502 | 204891 | 229272 | 188669 | 172627 | 221113 | 275439 |
| RP_POS_797_0882a13_822 | RP | POS | 13.82 | 797.0882 | | | | 2.35E-01 | 0.20 | 0.42 | 4.82E-01 | -0.15 | 0.59 | 5.51E-01 | 0.12 | 0.82 | 1.59E-01 | 0.27 | 0.63 | 757110 | 465457 | 887549 | 714243 | 938040 | 687578 | 837057 | 743459 |
| RP_POS_269_08798a1_786 | RP | POS | 1.79 | 269.0880 | Unknown | 137.0454(100) | 4 | 4.23E-09 | 0.72 | 1.34 | 4.82E-01 | 0.15 | 0.83 | 5.57E-05 | 0.80 | 1.42 | 6.62E-06 | 0.64 | 1.48 | 420806 | 972288 | 4589482 | 6311468 | 4143171 | 5135645 | 5035792 | 7328085 |
| RP_NEG_476_27832a10_336 | RP | NEG | 10.34 | 476.2783 | | | | 6.32E-02 | 0.36 | 1.05 | 4.83E-01 | -0.14 | 0.70 | 2.15E-01 | 0.29 | 0.81 | 3.37E-02 | 0.43 | 1.05 | 34795843 | 14013464 | 40330931 | 15488624 | 41424538 | 12584242 | 39237324 | 17996858 |
| RP_POS_360_17526a5_57 | RP | POS | 5.57 | 360.1753 | | | | 7.79E-07 | 0.59 | 1.30 | 4.83E-01 | -0.16 | 1.17 | 9.97E-04 | 0.51 | 1.38 | 2.58E-04 | 0.67 | 1.38 | 55538 | 134275 | 1423897 | 2585494 | 1606332 | 2779095 | 1241462 | 2390541 |
| RP_NEG_866_78992a0_495 | RP | NEG | 0.50 | 866.7899 | | | | 2.30E-08 | -0.69 | 1.37 | 4.83E-01 | 0.15 | 1.22 | 2.40E-04 | -0.61 | 1.41 | 1.95E-05 | -0.76 | 1.49 | 1948247 | 133334 | 1442695 | 807800 | 1385718 | 831166 | 1499673 | 787995 |
| RP_POS_818_52887a11_617 | RP | POS | 11.62 | 818.5289 | | | | 4.53E-02 | 0.26 | 0.63 | 4.83E-01 | -0.16 | 0.60 | 2.77E-01 | 0.18 | 0.45 | 6.12E-02 | 0.34 | 0.79 | 978793 | 1182903 | 1857909 | 3814683 | 2126963 | 3992207 | 1588856 | 3648991 |
| RP_POS_303_23178a9_959 | RP | POS | 9.96 | 303.2318 | | | | 3.47E-06 | 0.55 | 1.06 | 4.84E-01 | 0.16 | 1.10 | 7.00E-04 | 0.63 | 1.24 | 1.65E-03 | 0.47 | 1.12 | 146768 | 120962 | 1763590 | 3281949 | 1532287 | 2937407 | 1994894 | 3608967 |
| RP_POS_820_52399a12_827 | RP | POS | 12.83 | 820.5240 | | | | 2.28E-04 | -0.92 | 1.47 | 4.84E-01 | -0.11 | 0.63 | 1.80E-04 | -0.98 | 1.51 | 8.07E-04 | -0.87 | 1.30 | 3734327 | 1832902 | 2331441 | 1230161 | 2418075 | 1243516 | 2244806 | 1223040 |
| RP_POS_329_21463a14_268 | RP | POS | 14.27 | 329.2146 | | | | 3.79E-01 | 0.14 | 0.40 | 4.84E-01 | 0.15 | 0.47 | 3.17E-01 | 0.21 | 0.50 | 7.17E-01 | 0.06 | 0.39 | 2542444 | 454203 | 2642724 | 801581 | 2586251 | 635407 | 2699196 | 942299 |
| RP_NEG_187_09654a6_033 | RP | NEG | 6.03 | 187.0965 | | | | 1.92E-01 | 0.20 | 0.72 | 4.84E-01 | -0.15 | 0.79 | 5.20E-01 | 0.12 | 0.78 | 1.38E-01 | 0.28 | 0.72 | 3304337 | 427545 | 3446573 | 776693 | 3501257 | 760705 | 3391888 | 796266 |
| RP_NEG_201_11285a6_815 | RP | NEG | 6.82 | 201.1129 | | | | 1.07E-01 | 0.33 | 0.76 | 4.84E-01 | 0.14 | 0.62 | 8.63E-02 | 0.40 | 0.76 | 2.46E-01 | 0.26 | 0.60 | 2667750 | 514843 | 2840290 | 530571 | 2802944 | 502449 | 2877636 | 559871 |
| HILIC_NEG_239_76093a1_358 | HILIC | NEG | 1.358 | 239.7609 | | | | 1.40E-06 | 0.60 | 1.15 | 4.85E-01 | 0.16 | 0.44 | 2.80E-04 | 0.68 | 1.20 | 8.77E-04 | 0.52 | 1.38 | 212805 | 33273 | 296772 | 154604 | 285897 | 141187 | 307647 | 167682 |
| RP_POS_321_01077a4_851 | RP | POS | 4.85 | 321.0108 | | | | 7.61E-01 | 0.06 | 0.49 | 4.85E-01 | -0.15 | 0.59 | 9.46E-01 | -0.01 | 0.38 | 5.60E-01 | 0.13 | 0.51 | 2143389 | 885551 | 2200247 | 1009028 | 2271183 | 1073902 | 2129311 | 945260 |
| RP_POS_371_22757a4_2 | RP | POS | 4.20 | 371.2276 | | | | 9.55E-01 | -0.02 | 1.20 | 4.85E-01 | -0.11 | 1.10 | 8.10E-01 | -0.07 | 0.93 | 8.99E-01 | 0.04 | 0.99 | 644359 | 2125314 | 622026 | 1062081 | 696679 | 1130097 | 547372 | 995268 |

**Supplementary table 1: Metabolomics results** (in asceding order according to p-value from progressive ALD vs non-progressive ALD comparison, with molecular features with ID_level of 1-2 first)

| | | | | | | | | all_ALD_vs_control | | | Prog_ALD_vs_non-prog_ALD | | | Prog_ALD_vs_control_ALD | | | Non-prog_ALD_vs_control | | | Healthy_control | | all_ALD | | Non-prog_ALD | | Progressive_ALD | |
|---|---|---|---|---|---|---|---|---|---|---|---|---|---|---|---|---|---|---|---|---|---|---|---|---|---|---|---|
| | | | | | | | | t-test | Cohen's | PLS-DA | t-test | Cohen's | PLS-DA | t-test | Cohen's | PLS-DA | t-test | Cohen's | PLS-DA | | | | | | | | |
| Feature_ID | RP_HILIC | POS_NEG | Average.Rt | Average.Mz | Metabolite.name | curated_MSMS | ID_level | p | d | VIP | p | d | VIP | p | d | VIP | p | d | VIP | Mean | SD | Mean | SD | Mean | SD | Mean | SD |
| RP_NEG_290_85306a15_622 | RP | NEG | 15.62 | 290.8531 | | | | 6.82E-01 | -0.08 | 0.51 | 4.85E-01 | 0.14 | 0.50 | 9.54E-01 | -0.01 | 0.51 | 5.12E-01 | -0.15 | 0.57 | 1905816 | 76006 | 1899432 | 77032 | 1894017 | 83521 | 1904846 | 70380 |
| RP_POS_130_9626a0_253 | RP | POS | 0.25 | 130.9626 | | | | 2.48E-01 | 0.25 | 0.55 | 4.85E-01 | -0.14 | 0.56 | 4.41E-01 | 0.19 | 0.35 | 1.78E-01 | 0.32 | 0.51 | 4342712 | 513266 | 4461362 | 452857 | 4493186 | 444978 | 4429538 | 462894 |
| RP_NEG_201_14943a8_502 | RP | NEG | 8.50 | 201.1494 | | | | 1.61E-02 | 0.30 | 1.03 | 4.85E-01 | 0.16 | 0.45 | 9.12E-02 | 0.38 | 0.75 | 1.29E-02 | 0.22 | 0.94 | 604520 | 303181 | 946015 | 1292506 | 855034 | 583791 | 1036996 | 1737105 |
| RP_POS_335_68472a5_403 | RP | POS | 5.40 | 335.6847 | | | | 7.77E-07 | 0.59 | 1.29 | 4.86E-01 | -0.16 | 1.16 | 9.93E-04 | 0.51 | 1.38 | 2.56E-04 | 0.67 | 1.38 | 40647 | 126918 | 1222847 | 2232179 | 1379530 | 2397936 | 1066163 | 2065608 |
| RP_POS_793_62634a11_129 | RP | POS | 11.13 | 793.6263 | | | | 3.99E-03 | 0.38 | 0.97 | 4.86E-01 | -0.16 | 0.46 | 2.39E-02 | 0.30 | 0.86 | 2.86E-02 | 0.46 | 0.90 | 906339 | 1273177 | 2206954 | 3823750 | 2475544 | 4681557 | 1938364 | 2734285 |
| RP_POS_499_8273a4_412 | RP | POS | 4.41 | 499.8273 | | | | 2.60E-01 | -0.26 | 0.48 | 4.86E-01 | -0.13 | 0.46 | 1.63E-01 | -0.33 | 0.87 | 4.61E-01 | -0.20 | 0.48 | 2225081 | 2427710 | 1689179 | 1918926 | 1823939 | 2330290 | 1554418 | 1404419 |
| RP_POS_391_77905a13_718 | RP | POS | 13.72 | 391.7791 | | | | 2.51E-03 | 0.58 | 1.10 | 4.86E-01 | -0.14 | 0.41 | 1.88E-02 | 0.51 | 0.94 | 2.41E-03 | 0.65 | 1.13 | 2628410 | 848686 | 3192875 | 973023 | 3261146 | 947166 | 3124603 | 1003112 |
| RP_NEG_632_32196a10_336 | RP | NEG | 10.34 | 632.3220 | | | | 8.42E-02 | -0.45 | 1.14 | 4.86E-01 | -0.12 | 0.45 | 6.62E-02 | -0.50 | 0.99 | 1.48E-01 | -0.39 | 1.06 | 10448740 | 1904039 | 9815539 | 1200813 | 9899731 | 1129621 | 9731347 | 1273899 |
| HILIC_NEG_297_73337a3_058 | HILIC | NEG | 3.058 | 297.7334 | | | | 2.56E-01 | -0.14 | 0.63 | 4.86E-01 | -0.16 | 0.92 | 1.24E-01 | -0.22 | 0.54 | 7.71E-01 | -0.06 | 0.17 | 2280977 | 181177 | 2142366 | 1172061 | 2224541 | 1347842 | 2060191 | 972127 |
| RP_NEG_790_81543a0_494 | RP | NEG | 0.49 | 790.8154 | | | | 2.22E-09 | -0.74 | 1.39 | 4.86E-01 | 0.15 | 1.19 | 9.23E-05 | -0.66 | 1.43 | 4.07E-06 | -0.81 | 1.53 | 2420347 | 179897 | 1726601 | 1020543 | 1655080 | 1030131 | 1798121 | 1016200 |
| RP_NEG_1044_74609a0_495 | RP | NEG | 0.50 | 1044.7461 | | | | 3.92E-09 | -0.73 | 1.39 | 4.86E-01 | 0.15 | 1.18 | 1.17E-04 | -0.66 | 1.44 | 4.55E-06 | -0.81 | 1.52 | 2127941 | 162050 | 1591897 | 793478 | 1536309 | 797398 | 1647485 | 793672 |
| RP_POS_971_76349a15_104 | RP | POS | 15.10 | 971.7635 | | | | 2.42E-02 | 0.44 | 0.70 | 4.86E-01 | 0.14 | 0.44 | 2.24E-02 | 0.51 | 0.82 | 8.54E-02 | 0.37 | 0.71 | 2897966 | 1086367 | 3422132 | 1199113 | 3338142 | 1155844 | 3506122 | 1246883 |
| RP_NEG_341_68329a15_599 | RP | NEG | 15.60 | 341.6833 | | | | 1.33E-01 | -0.32 | 0.89 | 4.87E-01 | -0.14 | 0.46 | 1.07E-01 | -0.39 | 0.72 | 2.67E-01 | -0.25 | 0.78 | 2057180 | 98106 | 2027262 | 90805 | 2033616 | 83890 | 2020907 | 97671 |
| RP_POS_406_24872a6_184 | RP | POS | 6.18 | 406.2487 | | | | 1.92E-07 | 0.62 | 1.32 | 4.87E-01 | -0.15 | 1.18 | 5.50E-04 | 0.54 | 1.39 | 1.17E-04 | 0.70 | 1.42 | 27369 | 17058 | 691761 | 1186039 | 774664 | 1261687 | 608857 | 1111848 |
| RP_POS_282_27905a9_87 | RP | POS | 9.87 | 282.2791 | | | | 2.60E-02 | 0.44 | 0.73 | 4.87E-01 | -0.14 | 0.47 | 8.02E-02 | 0.36 | 0.79 | 2.80E-02 | 0.51 | 0.84 | 657702 | 544937 | 917297 | 601351 | 959332 | 664437 | 875261 | 534362 |
| RP_NEG_512_30011a9_952 | RP | NEG | 9.95 | 512.3001 | | | | 5.05E-03 | 0.44 | 0.94 | 4.87E-01 | -0.15 | 0.52 | 6.70E-02 | 0.37 | 0.80 | 4.66E-03 | 0.52 | 0.95 | 22071746 | 7173047 | 27150443 | 12317365 | 28011381 | 11300018 | 26289506 | 13316461 |
| RP_POS_414_76196a6_184 | RP | POS | 6.18 | 414.7620 | | | | 1.48E-07 | 0.63 | 1.33 | 4.88E-01 | -0.15 | 1.18 | 4.73E-04 | 0.55 | 1.40 | 1.04E-04 | 0.70 | 1.43 | 10123 | 7920 | 358423 | 615362 | 401395 | 655201 | 315450 | 576205 |
| HILIC_POS_584_26245a0_559 | HILIC | POS | 0.559 | 584.2625 | | | | 4.32E-03 | -0.57 | 1.19 | 4.88E-01 | 0.14 | 0.67 | 3.92E-02 | -0.50 | 0.76 | 1.38E-03 | -0.64 | 1.53 | 1080383 | 604919 | 709765 | 636982 | 665241 | 423096 | 754288 | 797945 |
| RP_POS_174_93758a0_498 | RP | POS | 0.50 | 174.9376 | | | | 7.32E-08 | -0.65 | 1.34 | 4.88E-01 | 0.15 | 1.20 | 3.00E-04 | -0.57 | 1.39 | 7.03E-05 | -0.73 | 1.47 | 11667067 | 327217 | 9609689 | 3502600 | 9365168 | 3733091 | 9854209 | 3275484 |
| RP_POS_310_91449a15_459 | RP | POS | 15.46 | 310.9145 | | | | 7.03E-01 | 0.09 | 0.52 | 4.88E-01 | -0.13 | 0.68 | 9.32E-01 | 0.02 | 0.48 | 5.30E-01 | 0.15 | 0.43 | 27334795 | 6686112 | 27832726 | 5377651 | 28207769 | 5069327 | 27457683 | 5695903 |
| RP_POS_242_24785a10_476 | RP | POS | 10.48 | 242.2479 | | | | 3.45E-04 | 0.49 | 0.92 | 4.88E-01 | 0.15 | 0.90 | 1.19E-02 | 0.56 | 0.96 | 4.19E-04 | 0.41 | 1.23 | 1037218 | 347806 | 1446663 | 927898 | 1381851 | 498831 | 1511475 | 1217438 |
| HILIC_NEG_195_81093a0_551 | HILIC | NEG | 0.551 | 195.8109 | | | | 6.42E-01 | 0.10 | 0.42 | 4.89E-01 | -0.14 | 0.71 | 9.07E-01 | 0.03 | 0.05 | 4.89E-01 | 0.17 | 0.64 | 1459577 | 650395 | 1521341 | 650314 | 1566685 | 724629 | 1475996 | 570232 |
| RP_POS_167_99368a6_185 | RP | POS | 6.19 | 167.9937 | | | | 3.31E-02 | 0.41 | 1.01 | 4.89E-01 | -0.14 | 0.64 | 9.86E-02 | 0.34 | 0.88 | 3.56E-02 | 0.48 | 1.02 | 2386255 | 1783360 | 3204954 | 2032392 | 3346544 | 2259903 | 3063364 | 1788144 |
| HILIC_POS_170_09256a6_294 | HILIC | POS | 6.294 | 170.0926 | | | | 7.77E-01 | -0.05 | 0.64 | 4.89E-01 | -0.15 | 0.74 | 5.69E-01 | -0.12 | 0.56 | 8.92E-01 | 0.03 | 0.25 | 25388252 | 8477029 | 24844047 | 11915096 | 25674018 | 10423178 | 24014075 | 13296161 |
| RP_POS_516_46149a13_614 | RP | POS | 13.61 | 516.4615 | | | | 9.61E-01 | 0.01 | 0.19 | 4.89E-01 | 0.16 | 0.59 | 7.05E-01 | 0.09 | 0.23 | 5.25E-01 | -0.07 | 0.35 | 790017 | 90907 | 791408 | 234219 | 775067 | 120218 | 807749 | 309579 |
| HILIC_NEG_234_8201a4_112 | HILIC | NEG | 4.112 | 234.8201 | | | | 2.42E-01 | -0.27 | 0.85 | 4.89E-01 | -0.13 | 0.51 | 1.75E-01 | -0.34 | 0.62 | 4.10E-01 | -0.21 | 0.87 | 717558 | 311807 | 646153 | 242168 | 663011 | 252102 | 629295 | 233134 |
| HILIC_POS_368_21854a5_647 | HILIC | POS | 5.647 | 368.2185 | | | | 1.59E-01 | -0.27 | 1.02 | 4.89E-01 | -0.14 | 0.81 | 1.17E-01 | -0.34 | 0.78 | 3.58E-01 | -0.20 | 0.66 | 727127 | 134411 | 686994 | 150879 | 697495 | 151783 | 676493 | 150767 |
| RP_POS_464_19113a6_782 | RP | POS | 6.78 | 464.1911 | | | | 4.37E-04 | 0.53 | 1.45 | 4.89E-01 | -0.15 | 0.45 | 1.46E-02 | 0.46 | 1.33 | 1.25E-03 | 0.61 | 1.17 | 242306 | 305496 | 532297 | 587262 | 573158 | 584773 | 491436 | 592811 |
| HILIC_NEG_114_98878a7_351 | HILIC | NEG | 7.351 | 114.9888 | | | | 3.87E-03 | -0.48 | 0.89 | 4.90E-01 | 0.15 | 0.82 | 3.19E-02 | -0.41 | 1.09 | 6.74E-03 | -0.56 | 0.91 | 695072 | 72548 | 644842 | 109730 | 637212 | 115804 | 652473 | 103912 |
| RP_POS_453_24539a3_808 | RP | POS | 3.81 | 453.2454 | | | | 7.06E-06 | 0.53 | 1.10 | 4.90E-01 | 0.16 | 1.04 | 2.93E-04 | 0.61 | 1.33 | 6.79E-03 | 0.46 | 1.05 | 244803 | 149913 | 1296572 | 2204704 | 1143311 | 2241242 | 1449833 | 2179344 |
| RP_POS_393_19839a10_978 | RP | POS | 10.98 | 393.1984 | | | | 4.78E-03 | 0.50 | 1.46 | 4.90E-01 | -0.14 | 1.36 | 4.31E-02 | 0.42 | 1.24 | 4.36E-03 | 0.57 | 1.40 | 971186 | 360237 | 1205565 | 491789 | 1239741 | 463654 | 1171389 | 520856 |
| RP_POS_357_69766a5_569 | RP | POS | 5.57 | 357.6977 | | | | 5.68E-07 | 0.60 | 1.30 | 4.90E-01 | -0.15 | 1.16 | 8.21E-04 | 0.52 | 1.38 | 2.30E-04 | 0.67 | 1.38 | 66536 | 139468 | 2104746 | 3804003 | 2368883 | 4091666 | 1840610 | 3514553 |
| HILIC_NEG_312_83664a4_182 | HILIC | NEG | 4.182 | 312.8366 | | | | 3.84E-01 | 0.13 | 1.24 | 4.90E-01 | 0.15 | 0.86 | 2.48E-01 | 0.20 | 1.16 | 7.97E-01 | 0.05 | 0.96 | 725480 | 60799 | 740131 | 128860 | 731184 | 136541 | 749078 | 121417 |
| HILIC_POS_276_18036a2_383 | HILIC | POS | 2.383 | 276.1804 | | | | 2.91E-05 | 0.69 | 1.26 | 4.90E-01 | 0.14 | 1.02 | 1.41E-04 | 0.76 | 1.38 | 1.29E-03 | 0.61 | 1.16 | 393265 | 272192 | 678887 | 430850 | 648979 | 421672 | 708795 | 442063 |
| RP_POS_413_13654a8_7 | RP | POS | 8.70 | 413.1365 | | | | 2.48E-01 | 0.15 | 0.49 | 4.91E-01 | 0.16 | 0.60 | 3.03E-01 | 0.23 | 0.38 | 4.66E-01 | 0.08 | 0.30 | 1054298 | 493705 | 1244402 | 1385561 | 1148072 | 662354 | 1340732 | 1849619 |
| HILIC_NEG_144_92351a1_419 | HILIC | NEG | 1.419 | 144.9235 | | | | 5.01E-02 | -0.39 | 0.71 | 4.91E-01 | -0.14 | 0.82 | 6.25E-02 | -0.46 | 0.71 | 1.03E-01 | -0.32 | 0.67 | 18726211 | 3078648 | 17451827 | 3296037 | 17680717 | 2239054 | 17222936 | 4102335 |
| RP_POS_298_2738a9_745 | RP | POS | 9.75 | 298.2738 | | | | 9.97E-01 | 0.00 | 0.56 | 4.91E-01 | -0.13 | 0.58 | 7.81E-01 | -0.07 | 0.53 | 8.04E-01 | 0.06 | 0.29 | 3485260 | 5827809 | 3481267 | 4649553 | 3803883 | 5338180 | 3158652 | 3869019 |
| HILIC_POS_596_32812a0_588 | HILIC | POS | 0.588 | 596.3281 | | | | 5.95E-04 | -0.73 | 1.17 | 4.91E-01 | 0.13 | 0.77 | 3.96E-03 | -0.66 | 1.13 | 5.56E-04 | -0.79 | 1.27 | 779155 | 340895 | 528385 | 323724 | 505952 | 316690 | 550818 | 332286 |
| RP_POS_395_22656a6_067 | RP | POS | 6.07 | 395.2266 | | | | 1.36E-07 | 0.63 | 1.33 | 4.92E-01 | -0.15 | 1.19 | 4.70E-04 | 0.55 | 1.41 | 9.62E-05 | 0.71 | 1.43 | 13323 | 9367 | 394282 | 670633 | 440677 | 711334 | 347887 | 631100 |
| RP_NEG_320_88718a15_46 | RP | NEG | 15.46 | 320.8872 | | | | 3.12E-01 | -0.13 | 0.55 | 4.92E-01 | -0.16 | 0.42 | 2.67E-01 | -0.21 | 0.56 | 7.52E-01 | -0.05 | 0.44 | 11182162 | 246392 | 11078144 | 927080 | 11142270 | 834092 | 11014019 | 1016067 |
| HILIC_NEG_174_08821a6_345 | HILIC | NEG | 6.345 | 174.0882 | | | | 1.70E-04 | 0.62 | 1.01 | 4.92E-01 | 0.14 | 0.71 | 7.18E-04 | 0.69 | 1.13 | 3.39E-03 | 0.55 | 1.01 | 441664 | 117585 | 551003 | 184005 | 538281 | 171854 | 563725 | 196322 |
| RP_POS_264_97281a0_523 | RP | POS | 0.52 | 264.9728 | | | | 8.64E-07 | -0.71 | 1.29 | 4.92E-01 | -0.15 | 1.18 | 3.68E-05 | -0.78 | 1.38 | 3.33E-04 | -0.63 | 1.36 | 419153 | 71053 | 314234 | 157840 | 325147 | 152692 | 303322 | 163638 |
| RP_POS_464_3739a11_841 | RP | POS | 11.84 | 464.3739 | | | | 6.52E-01 | 0.10 | 0.48 | 4.92E-01 | 0.14 | 0.50 | 5.01E-01 | 0.16 | 0.52 | 8.96E-01 | 0.03 | 0.44 | 18357124 | 9283296 | 19195115 | 8423452 | 18612917 | 7543696 | 19777312 | 9260564 |
| RP_POS_425_75281a6_184 | RP | POS | 6.18 | 425.7528 | | | | 1.46E-07 | 0.63 | 1.33 | 4.92E-01 | -0.15 | 1.19 | 4.74E-04 | 0.55 | 1.40 | 1.03E-04 | 0.70 | 1.43 | 67542 | 30078 | 1228897 | 2050387 | 1370560 | 2179138 | 1087234 | 1924675 |
| RP_POS_860_53925a11_159 | RP | POS | 11.16 | 860.5393 | | | | 3.54E-01 | 0.12 | 0.38 | 4.93E-01 | -0.16 | 0.52 | 6.96E-01 | 0.04 | 0.27 | 3.75E-01 | 0.20 | 0.47 | 932070 | 557140 | 1142075 | 2028935 | 1282470 | 2681765 | 1001680 | 1041672 |
| RP_POS_419_31323a11_165 | RP | POS | 11.17 | 419.3132 | | | | 2.21E-01 | 0.23 | 0.65 | 4.93E-01 | -0.14 | 0.43 | 4.87E-01 | 0.16 | 0.53 | 1.36E-01 | 0.30 | 0.53 | 5799380 | 666940 | 5975495 | 798658 | 6030633 | 692849 | 5920356 | 895814 |
| RP_POS_338_16214a5_403 | RP | POS | 5.40 | 338.1621 | | 338.1592(100) | 4 | 8.47E-07 | 0.59 | 1.29 | 4.93E-01 | -0.15 | 1.16 | 1.07E-03 | 0.51 | 1.38 | 2.63E-04 | 0.66 | 1.38 | 31095 | 89672 | 1001260 | 1840656 | 1128293 | 1969892 | 874228 | 1712240 |
| RP_POS_193_09694a0_709 | RP | POS | 0.71 | 193.0969 | | | | 6.46E-05 | 0.60 | 0.97 | 4.93E-01 | 0.15 | 0.48 | 5.81E-04 | 0.67 | 1.10 | 3.08E-03 | 0.52 | 1.03 | 1435989 | 707337 | 2215608 | 1394392 | 2119428 | 1311769 | 2311788 | 1479433 |
| RP_NEG_582_88123a0_465 | RP | NEG | 0.47 | 582.8812 | | | | 1.18E-09 | -0.77 | 1.41 | 4.94E-01 | 0.15 | 1.18 | 6.82E-05 | -0.69 | 1.44 | 1.54E-06 | -0.84 | 1.56 | 1061116 | 108966 | 730012 | 464825 | 697982 | 459264 | 762042 | 472775 |
| RP_POS_475_41324a13_195 | RP | POS | 13.20 | 475.4132 | | | | 4.59E-01 | 0.14 | 0.40 | 4.94E-01 | 0.14 | 0.43 | 3.25E-01 | 0.21 | 0.56 | 7.57E-01 | 0.07 | 0.27 | 9924451 | 1391759 | 10146330 | 1672867 | 10031058 | 1694542 | 10261601 | 1659967 |
| RP_POS_205_08577a9_46 | RP | POS | 9.46 | 205.0858 | | | | 5.90E-01 | 0.08 | 0.34 | 4.94E-01 | 0.15 | 0.78 | 4.98E-01 | 0.16 | 0.57 | 9.82E-01 | 0.00 | 0.14 | 1019289 | 61004 | 1028210 | 124771 | 1019599 | 63874 | 1036822 | 164991 |
| HILIC_POS_121_0399a0_701 | HILIC | POS | 0.701 | 121.0399 | | | | 2.17E-01 | -0.41 | 0.80 | 4.94E-01 | 0.06 | 0.69 | 2.60E-01 | -0.38 | 0.54 | 1.87E-01 | -0.45 | 0.72 | 591366 | 1255151 | 309201 | 319851 | 287182 | 294038 | 331220 | 345325 |
| RP_NEG_688_83386a0_496 | RP | NEG | 0.50 | 688.8339 | | | | 1.36E-08 | -0.69 | 1.37 | 4.94E-01 | 0.15 | 1.21 | 1.56E-04 | -0.62 | 1.42 | 2.13E-05 | -0.77 | 1.49 | 11403035 | 590494 | 8562792 | 4504745 | 8252876 | 4702829 | 8872708 | 4322843 |
| RP_POS_800_51849a12_25 | RP | POS | 12.25 | 800.5185 | | | | 3.32E-08 | 0.83 | 1.48 | 4.94E-01 | 0.14 | 0.49 | 6.31E-06 | 0.90 | 1.53 | 1.08E-05 | 0.76 | 1.41 | 428963 | 355654 | 990850 | 701284 | 942618 | 632062 | 1039083 | 767714 |
| RP_POS_590_54895a15_102 | RP | POS | 15.10 | 590.5490 | | | | 7.18E-01 | 0.07 | 0.32 | 4.94E-01 | 0.14 | 0.53 | 5.09E-01 | 0.14 | 0.37 | 9.88E-01 | 0.00 | 0.37 | 11779843 | 3839532 | 12065318 | 3943053 | 11794142 | 4384812 | 12336495 | 3469291 |
| HILIC_POS_210_04996a3_928 | HILIC | POS | 3.928 | 210.0500 | | | | 4.23E-02 | 0.30 | 0.89 | 4.95E-01 | 0.15 | 0.84 | 7.33E-02 | 0.38 | 0.82 | 1.47E-01 | 0.23 | 0.78 | 1410508 | 359929 | 1604840 | 699282 | 1556742 | 544051 | 1652939 | 829009 |
| RP_POS_151_00481a15_442 | RP | POS | 15.44 | 151.0048 | | | | 1.78E-01 | -0.29 | 0.82 | 4.95E-01 | -0.13 | 0.40 | 9.54E-02 | -0.35 | 0.77 | 3.90E-01 | -0.22 | 0.61 | 156481390 | 39153444 | 145807813 | 36380171 | 148308912 | 45399960 | 143306714 | 24497377 |
| RP_POS_451_36273a10_544 | RP | POS | 10.54 | 451.3627 | | | | 1.20E-01 | 0.31 | 0.91 | 4.95E-01 | 0.14 | 0.67 | 7.48E-02 | 0.38 | 0.95 | 3.05E-01 | 0.24 | 0.79 | 924032 | 179878 | 982730 | 192628 | 969504 | 215331 | 995957 | 168060 |
| RP_POS_496_80624a0_501 | RP | POS | 0.50 | 496.8062 | | | | 8.20E-02 | -0.27 | 1.06 | 4.95E-01 | -0.15 | 1.25 | 5.37E-02 | -0.34 | 1.06 | 3.37E-01 | -0.19 | 1.07 | 677761 | 224905 | 577736 | 407201 | 605684 | 445888 | 549787 | 366878 |
| RP_POS_974_81061a0_489 | RP | POS | 0.49 | 974.8106 | | | | 6.17E-08 | 0.65 | 1.34 | 4.96E-01 | -0.15 | 1.20 | 2.88E-04 | 0.57 | 1.39 | 6.82E-05 | 0.72 | 1.47 | 699395 | 209241 | 3373676 | 4555731 | 3685818 | 4847023 | 3061534 | 4270886 |
| RP_POS_404_20126a5_862 | RP | POS | 5.86 | 404.2013 | | | | 7.71E-07 | 0.59 | 1.30 | 4.96E-01 | -0.15 | 1.17 | 9.15E-04 | 0.51 | 1.39 | 2.75E-04 | 0.67 | 1.38 | 80660 | 187429 | 1852335 | 3344424 | 2081334 | 3603339 | 1623336 | 3083429 |
| HILIC_POS_114_60739a1_231 | HILIC | POS | 1.231 | 114.6074 | | | | 1.86E-01 | -0.34 | 0.70 | 4.96E-01 | -0.12 | 0.63 | 1.46E-01 | -0.40 | 0.62 | 2.94E-01 | -0.28 | 0.46 | 1152415 | 851042 | 936954 | 549853 | 974599 | 523693 | 899309 | 577668 |
| RP_POS_499_82712a3_398 | RP | POS | 3.40 | 499.8271 | | | | 1.23E-01 | 0.24 | 0.59 | 4.96E-01 | -0.15 | 0.53 | 3.37E-01 | 0.17 | 0.60 | 1.30E-01 | 0.31 | 0.70 | 2059895 | 1727923 | 2732568 | 3057462 | 2941901 | 3457573 | 2523236 | 2615852 |
| RP_POS_323_00494a4_851 | RP | POS | 4.85 | 323.0049 | | | | 7.11E-01 | 0.07 | 0.50 | 4.97E-01 | -0.14 | 0.58 | 9.96E-01 | 0.00 | 0.40 | 5.31E-01 | 0.14 | 0.53 | 775517 | 327613 | 801042 | 367043 | 826163 | 395792 | 775922 | 338011 |
| RP_NEG_724_87354a0_482 | RP | NEG | 0.48 | 724.8735 | | | | 6.26E-08 | 0.65 | 1.34 | 4.97E-01 | -0.15 | 1.21 | 2.51E-04 | 0.57 | 1.40 | 7.84E-05 | 0.72 | 1.46 | 2772608 | 520702 | 10081246 | 12462209 | 10933934 | 13378025 | 9228558 | 11546750 |
| HILIC_POS_346_02039a7_803 | HILIC | POS | 7.803 | 346.0204 | | | | 7.02E-01 | -0.08 | 0.91 | 4.97E-01 | 0.14 | 0.62 | 9.78E-01 | -0.01 | 0.55 | 5.35E-01 | -0.15 | 1.07 | 1581375 | 145876 | 1569775 | 156678 | 1559056 | 175659 | 1580494 | 136038 |
| RP_POS_249_10747a0_608 | RP | POS | 0.61 | 249.1075 | | | | 1.99E-02 | -0.37 | 1.03 | 4.97E-01 | 0.15 | 1.02 | 1.07E-01 | -0.30 | 0.88 | 2.68E-02 | -0.45 | 1.13 | 1064212 | 302400 | 890735 | 499737 | 856554 | 529737 | 924915 | 470708 |
| RP_POS_445_39969a11_038 | RP | POS | 11.04 | 445.3997 | | | | 3.48E-02 | 0.34 | 0.85 | 4.97E-01 | -0.15 | 0.45 | 1.30E-01 | 0.27 | 0.64 | 5.07E-02 | 0.41 | 0.78 | 2899098 | 3075763 | 4488681 | 5041968 | 4833568 | 5721113 | 4143794 | 4288099 |
| RP_POS_419_31677a12_884 | RP | POS | 12.88 | 419.3168 | | | | 1.53E-01 | -0.28 | 0.64 | 4.97E-01 | 0.14 | 0.57 | 3.38E-01 | -0.21 | 0.58 | 1.13E-01 | -0.35 | 0.66 | 7053205 | 2743014 | 6231942 | 2947881 | 6030378 | 2925296 | 6433506 | 2986148 |
| RP_POS_185_11508a15_208 | RP | POS | 15.21 | 185.1151 | | | | 7.43E-01 | -0.07 | 0.32 | 4.97E-01 | -0.14 | 0.41 | 5.56E-01 | -0.14 | 0.23 | 9.91E-01 | 0.00 | 0.59 | 8622353 | 5493465 | 8252266 | 5613018 | 8635746 | 5426710 | 7868785 | 5822854 |
| RP_POS_548_50446a11_608 | RP | POS | 11.61 | 548.5045 | | | | 1.22E-01 | -0.32 | 0.81 | 4.97E-01 | 0.14 | 0.47 | 2.61E-01 | -0.25 | 0.62 | 9.70E-02 | -0.39 | 0.70 | 4276298 | 822037 | 4015009 | 805877 | 3959979 | 843163 | 4070040 | 771379 |
| HILIC_POS_133_0607a6_235 | HILIC | POS | 6.235 | 133.0607 | | | | 7.44E-01 | -0.05 | 1.14 | 4.98E-01 | 0.15 | 0.81 | 9.28E-01 | 0.02 | 0.90 | 5.04E-01 | -0.13 | 1.00 | 2405604 | 600041 | 2360060 | 896267 | 2298860 | 837300 | 2421260 | 956174 |
| RP_NEG_242_94438a15_333 | RP | NEG | 15.33 | 242.9444 | | | | 6.43E-01 | -0.09 | 0.54 | 4.98E-01 | -0.14 | 0.41 | 4.37E-01 | -0.16 | 0.53 | 9.36E-01 | -0.02 | 0.36 | 5386279 | 152846 | 5371339 | 173547 | 5383179 | 195751 | 5359499 | 149156 |
| RP_POS_149_96051a0_252 | RP | POS | 0.25 | 149.9605 | | | | 1.96E-01 | -0.34 | 1.17 | 4.98E-01 | 0.11 | 0.41 | 3.14E-01 | -0.28 | 0.82 | 1.42E-01 | -0.40 | 0.97 | 9284930 | 3514801 | 8421195 | 2123386 | 8276405 | 1829412 | 8565985 | 2391655 |
| RP_POS_478_38898a14_21 | RP | POS | 14.21 | 478.3890 | | | | 1.66E-01 | -0.30 | 0.92 | 4.98E-01 | -0.13 | 0.61 | 1.27E-01 | -0.37 | 1.02 | 3.17E-01 | -0.24 | 0.66 | 6938590 | 2268103 | 6306549 | 2030216 | 6444881 | 1982723 | 6168217 | 2087446 |
| HILIC_POS_327_07944a0_852 | HILIC | POS | 0.852 | 327.0794 | | | | 8.12E-01 | 0.04 | 0.81 | 4.99E-01 | -0.14 | 0.93 | 8.91E-01 | -0.03 | 0.24 | 5.45E-01 | 0.11 | 0.26 | 453762 | 145866 | 461508 | 196397 | 474890 | 164872 | 448126 | 224461 |
| RP_POS_290_63864a10_617 | RP | POS | 10.62 | 290.6386 | | | | 2.77E-01 | 0.25 | 1.00 | 4.99E-01 | -0.13 | 0.97 | 4.54E-01 | 0.19 | 0.75 | 2.01E-01 | 0.32 | 0.99 | 3799708 | 733859 | 3955559 | 569381 | 3994343 | 534768 | 3916776 | 604945 |
| RP_POS_478_38849a11_966 | RP | POS | 11.97 | 478.3885 | | | | 5.39E-01 | -0.14 | 0.41 | 4.99E-01 | 0.13 | 0.46 | 7.80E-01 | -0.07 | 0.23 | 3.85E-01 | -0.20 | 0.53 | 53664252 | 4627471 | 53101264 | 3998694 | 52828962 | 3461589 | 53373565 | 4491270 |
| RP_POS_382_18832a5_722 | RP | POS | 5.72 | 382.1883 | | | | 5.10E-07 | 0.60 | 1.30 | 4.99E-01 | -0.15 | 1.16 | 7.24E-04 | 0.52 | 1.39 | 2.35E-04 | 0.67 | 1.38 | 52414 | 69038 | 1794366 | 3239087 | 2014880 | 3494344 | 1573852 | 2981243 |
| RP_POS_134_95897a0_252 | RP | POS | 0.25 | 134.9590 | | | | 2.14E-01 | 0.29 | 0.63 | 4.99E-01 | -0.13 | 0.60 | 3.68E-01 | 0.22 | 0.43 | 1.62E-01 | 0.35 | 0.71 | 6857128 | 984267 | 7097916 | 790991 | 7151741 | 804484 | 7044092 | 781661 |
| HILIC_POS_227_07893a4_123 | HILIC | POS | 4.123 | 227.0789 | | | | 1.95E-01 | -0.21 | 1.00 | 5.00E-01 | -0.15 | 1.17 | 1.57E-01 | -0.28 | 1.04 | 4.77E-01 | -0.14 | 0.97 | 442210 | 164169 | 390762 | 266703 | 408888 | 258410 | 372637 | 276163 |
| RP_POS_355_28162a10_646 | RP | POS | 10.65 | 355.2816 | | | | 3.24E-03 | -0.65 | 1.24 | 5.00E-01 | 0.13 | 1.01 | 1.44E-02 | -0.59 | 1.08 | 2.54E-03 | -0.71 | 1.23 | 14109629 | 1757398 | 13033890 | 1542749 | 12929042 | 1474297 | 13138739 | 1616396 |
| RP_NEG_171_06586a5_276 | RP | NEG | 5.28 | 171.0659 | | | | 4.65E-02 | -0.56 | 1.08 | 5.00E-01 | -0.10 | 0.92 | 3.60E-02 | -0.61 | 0.95 | 7.81E-02 | -0.51 | 0.84 | 942264 | 454791 | 769983 | 228563 | 785511 | 230542 | 754455 | 227831 |
| RP_NEG_287_92508a15_598 | RP | NEG | 15.60 | 287.9251 | | | | 2.55E-02 | 0.33 | 0.76 | 5.00E-01 | -0.15 | 0.43 | 1.52E-01 | 0.26 | 0.59 | 3.39E-02 | 0.41 | 0.81 | 827217 | 92748 | 883076 | 184090 | 895582 | 191433 | 870570 | 177494 |
| RP_POS_382_2196a5_862 | RP | POS | 5.86 | 382.2196 | | | | 4.49E-07 | 0.60 | 1.29 | 5.00E-01 | -0.15 | 1.16 | 6.73E-04 | 0.53 | 1.39 | 2.22E-04 | 0.68 | 1.38 | 18218 | 21329 | 1387218 | 2532748 | 1559081 | 2732573 | 1215355 | 2330945 |
| RP_NEG_197_04227a3_963 | RP | NEG | 3.96 | 197.0423 | | | | 5.41E-01 | 0.10 | 1.09 | 5.00E-01 | -0.15 | 0.41 | 8.97E-01 | 0.03 | 0.82 | 3.40E-01 | 0.17 | 0.70 | 2350135 | 543540 | 2428552 | 841672 | 2485653 | 731255 | 2371452 | 943341 |
| HILIC_POS_280_15424a1_325 | HILIC | POS | 1.325 | 280.1542 | | | | 4.59E-01 | -0.14 | 0.28 | 5.00E-01 | 0.14 | 0.59 | 7.58E-01 | -0.07 | 0.15 | 2.80E-01 | -0.21 | 0.45 | 1983936 | 1893154 | 1689290 | 2101797 | 1546648 | 1564792 | 1831932 | 2536762 |
| RP_NEG_829_18103a9_654 | RP | NEG | 9.65 | 829.1810 | | | | 6.80E-01 | 0.08 | 0.26 | 5.01E-01 | 0.14 | 0.62 | 4.88E-01 | 0.15 | 0.43 | 9.52E-01 | 0.01 | 0.27 | 9207804 | 1251669 | 9314413 | 1308766 | 9225706 | 1404408 | 9403120 | 1213373 |

**Supplementary table 1: Metabolomics results** (in asceding order according to p-value from progressive ALD vs  non-progressive ALD comparison, with molecular features with ID_level of 1-2 first)

| | | | | | | | | all_ALD_vs_control | | | Prog_ALD_vs_non-prog_ALD | | | Prog_ALD_vs_control_ALD | | | Non-prog_ALD_vs_control | | | Healthy_control | | all_ALD | | Non-prog_ALD | | Progressive_ALD | |
|---|---|---|---|---|---|---|---|---|---|---|---|---|---|---|---|---|---|---|---|---|---|---|---|---|---|---|---|
| | | | | | | | | t-test | Cohen's | PLS-DA | t-test | Cohen's | PLS-DA | t-test | Cohen's | PLS-DA | t-test | Cohen's | PLS-DA | | | | | | | | |
| Feature_ID | RP_HILIC | POS_NEG | Average.Rt | Average.Mz | Metabolite.name | curated_MSMS | ID_level | p | d | VIP | p | d | VIP | p | d | VIP | p | d | VIP | Mean | SD | Mean | SD | Mean | SD | Mean | SD |
| RP_POS_617_52228a9_476 | RP | POS | 9.48 | 617.5223 | | | | 3.20E-01 | -0.28 | 0.88 | 5.01E-01 | -0.10 | 0.46 | 2.51E-01 | -0.33 | 0.70 | 4.38E-01 | -0.23 | 0.58 | 1482486 | 4025718 | 734613 | 2064929 | 874556 | 2204561 | 594670 | 1927389 |
| RP_NEG_404_84625a15_462 | RP | NEG | 15.46 | 404.8463 | | | | 8.15E-01 | 0.04 | 0.77 | 5.01E-01 | 0.15 | 0.48 | 5.50E-01 | 0.11 | 0.61 | 8.35E-01 | -0.04 | 0.73 | 938206 | 39655 | 940637 | 76503 | 935454 | 78639 | 945819 | 74740 |
| HILIC_POS_168_12306a0_631 | HILIC | POS | 0.631 | 168.1231 | | | | 4.40E-01 | 0.11 | 1.11 | 5.01E-01 | -0.15 | 0.76 | 8.35E-01 | 0.03 | 1.26 | 3.74E-01 | 0.18 | 1.06 | 829101 | 114178 | 856110 | 283899 | 875350 | 336467 | 836869 | 221079 |
| RP_POS_323_25562a10_774 | RP | POS | 10.77 | 323.2556 | | | | 3.75E-04 | -0.57 | 1.15 | 5.01E-01 | 0.14 | 1.07 | 4.94E-03 | -0.50 | 1.15 | 2.12E-03 | -0.64 | 1.23 | 4788848 | 383646 | 4442189 | 647428 | 4398337 | 724203 | 4486041 | 564346 |
| RP_POS_379_71094a5_722 | RP | POS | 5.72 | 379.7109 | | | | 5.64E-07 | 0.60 | 1.30 | 5.01E-01 | -0.15 | 1.17 | 8.02E-04 | 0.52 | 1.39 | 2.33E-04 | 0.67 | 1.39 | 107752 | 200253 | 2778581 | 4981643 | 3115867 | 5351068 | 2441295 | 4612363 |
| RP_POS_653_3349a5_399 | RP | POS | 5.40 | 653.3349 | | | | 1.30E-06 | 0.58 | 1.27 | 5.01E-01 | -0.15 | 1.15 | 1.18E-03 | 0.50 | 1.37 | 3.68E-04 | 0.65 | 1.35 | 49437 | 125971 | 1420414 | 2652805 | 1599930 | 2861847 | 1240899 | 2441809 |
| RP_POS_368_20035a11_251 | RP | POS | 11.25 | 368.2004 | | | | 3.16E-04 | 0.70 | 1.55 | 5.01E-01 | 0.13 | 1.07 | 7.24E-04 | 0.76 | 1.49 | 2.19E-03 | 0.63 | 1.41 | 6023199 | 1226313 | 7010620 | 1399958 | 6915882 | 1255164 | 7105359 | 1538184 |
| RP_NEG_258_91592a15_47 | RP | NEG | 15.47 | 258.9159 | | | | 8.35E-01 | 0.04 | 0.87 | 5.02E-01 | 0.14 | 0.47 | 6.02E-01 | 0.11 | 0.58 | 8.80E-01 | -0.03 | 0.66 | 2077258 | 403446 | 2095408 | 496150 | 2061881 | 513618 | 2128935 | 480891 |
| RP_POS_697_36084a5_568 | RP | POS | 5.57 | 697.3608 | | | | 1.97E-06 | 0.57 | 1.26 | 5.02E-01 | -0.15 | 1.14 | 1.30E-03 | 0.49 | 1.36 | 4.93E-04 | 0.64 | 1.34 | 71027 | 122491 | 1097384 | 2021163 | 1233946 | 2198835 | 960821 | 1838648 |
| RP_POS_328_24808a7_219 | RP | POS | 7.22 | 328.2481 | | | | 1.18E-01 | -0.29 | 0.83 | 5.03E-01 | -0.14 | 0.45 | 1.10E-01 | -0.36 | 0.80 | 2.69E-01 | -0.22 | 0.66 | 4779753 | 1925568 | 4127578 | 2317241 | 4284000 | 2024630 | 3971156 | 2588385 |
| RP_POS_360_2066a5_722 | RP | POS | 5.72 | 360.2066 | | | | 5.44E-07 | 0.60 | 1.29 | 5.03E-01 | -0.15 | 1.16 | 7.85E-04 | 0.52 | 1.38 | 2.34E-04 | 0.67 | 1.38 | 13601 | 15157 | 1247217 | 2301287 | 1402538 | 2473212 | 1091895 | 2129298 |
| RP_POS_594_3526a10_678 | RP | POS | 10.68 | 594.3526 | | | | 7.48E-07 | 0.89 | 1.66 | 5.03E-01 | -0.13 | 0.73 | 1.26E-04 | 0.82 | 1.47 | 7.37E-07 | 0.96 | 1.80 | 650943 | 388976 | 1118797 | 514797 | 1153545 | 438725 | 1084049 | 583521 |
| RP_POS_490_29013a9_797 | RP | POS | 9.80 | 490.2901 | | | | 2.89E-03 | 0.45 | 0.95 | 5.03E-01 | -0.15 | 0.47 | 5.12E-02 | 0.38 | 0.84 | 4.00E-03 | 0.52 | 0.96 | 682788 | 240021 | 874201 | 461723 | 905355 | 437705 | 843047 | 486992 |
| HILIC_POS_424_34186a0_613 | HILIC | POS | 0.613 | 424.3419 | | | | 2.44E-03 | 0.51 | 1.09 | 5.03E-01 | -0.14 | 0.67 | 1.52E-02 | 0.44 | 0.91 | 6.88E-03 | 0.58 | 1.21 | 662679 | 250188 | 843506 | 370729 | 868520 | 421162 | 818491 | 314691 |
| RP_POS_468_38968a10_535 | RP | POS | 10.54 | 468.3897 | | | | 3.27E-01 | 0.19 | 0.43 | 5.03E-01 | 0.14 | 0.54 | 2.67E-01 | 0.26 | 0.45 | 5.48E-01 | 0.12 | 0.43 | 1909427 | 684028 | 2049356 | 736701 | 1999662 | 622242 | 2099050 | 839231 |
| HILIC_POS_143_34151a3_5 | HILIC | POS | 3.5 | 143.3415 | | | | 9.29E-02 | 0.24 | 0.53 | 5.03E-01 | -0.15 | 0.57 | 1.95E-01 | 0.17 | 0.48 | 1.57E-01 | 0.32 | 0.51 | 385977 | 543904 | 641808 | 1163763 | 720388 | 1505232 | 563227 | 676761 |
| HILIC_POS_626_30249a1_719 | HILIC | POS | 1.719 | 626.3025 | | | | 6.03E-01 | -0.09 | 1.20 | 5.03E-01 | 0.15 | 0.76 | 9.53E-01 | -0.01 | 1.06 | 3.70E-01 | -0.16 | 1.41 | 571205 | 166384 | 550836 | 256891 | 533512 | 209981 | 568160 | 297705 |
| RP_NEG_426_96704a11_378 | RP | NEG | 11.38 | 426.9670 | | | | 3.71E-01 | -0.19 | 0.50 | 5.03E-01 | -0.13 | 0.55 | 2.93E-01 | -0.26 | 0.51 | 5.74E-01 | -0.13 | 0.40 | 4253470 | 2132643 | 3871366 | 1915607 | 4000486 | 1695947 | 3742245 | 2122198 |
| RP_POS_449_284a11_263 | RP | POS | 11.26 | 449.2840 | | | | 2.85E-01 | 0.19 | 0.58 | 5.03E-01 | 0.14 | 0.53 | 2.00E-01 | 0.26 | 0.68 | 5.67E-01 | 0.12 | 0.52 | 235834 | 371852 | 323594 | 479430 | 291286 | 497987 | 355902 | 462899 |
| RP_POS_261_1445a3_084 | RP | POS | 3.08 | 261.1445 | | | | 1.07E-02 | 0.40 | 0.75 | 5.03E-01 | 0.15 | 0.96 | 3.39E-02 | 0.47 | 0.82 | 3.36E-02 | 0.33 | 0.76 | 1225510 | 489911 | 1543157 | 857581 | 1485349 | 587822 | 1600965 | 1064681 |
| HILIC_POS_59_06133a3_028 | HILIC | POS | 3.028 | 59.0613 | | | | 5.32E-04 | 0.46 | 1.24 | 5.04E-01 | -0.15 | 0.83 | 2.50E-02 | 0.38 | 1.27 | 3.20E-03 | 0.53 | 1.49 | 1791188 | 148550 | 1974395 | 443750 | 2004263 | 455293 | 1944526 | 434437 |
| RP_POS_376_34183a11_265 | RP | POS | 11.27 | 376.3418 | | | | 7.94E-01 | 0.04 | 0.31 | 5.04E-01 | 0.14 | 0.59 | 5.97E-01 | 0.12 | 0.52 | 8.75E-01 | -0.03 | 0.39 | 225939 | 258231 | 241442 | 378007 | 215990 | 305439 | 266894 | 440546 |
| RP_POS_551_41003a14_889 | RP | POS | 14.89 | 551.4100 | | | | 6.33E-01 | 0.07 | 0.23 | 5.04E-01 | 0.15 | 0.47 | 5.30E-01 | 0.14 | 0.31 | 9.55E-01 | -0.01 | 0.33 | 4707952 | 591524 | 4788378 | 1313430 | 4699914 | 662497 | 4876841 | 1740841 |
| RP_POS_308_29477a9_951 | RP | POS | 9.95 | 308.2948 | | | | 3.84E-01 | -0.25 | 0.90 | 5.04E-01 | -0.10 | 0.50 | 3.07E-01 | -0.30 | 0.70 | 5.06E-01 | -0.20 | 0.56 | 3679898 | 7473133 | 2469454 | 3711971 | 2718902 | 3928541 | 2220007 | 3504032 |
| RP_NEG_166_9935a15_459 | RP | NEG | 15.46 | 166.9935 | | | | 9.45E-01 | 0.02 | 0.84 | 5.04E-01 | -0.13 | 0.42 | 8.44E-01 | -0.05 | 0.45 | 7.53E-01 | 0.08 | 0.67 | 2105003 | 123514 | 2106682 | 100091 | 2113408 | 107244 | 2099957 | 92991 |
| RP_POS_299_12872a10_301 | RP | POS | 10.30 | 299.1287 | | | | 1.93E-02 | 0.53 | 1.03 | 5.05E-01 | -0.12 | 0.69 | 6.31E-02 | 0.47 | 0.71 | 1.17E-02 | 0.60 | 1.11 | 557330 | 187945 | 646100 | 153268 | 656394 | 128154 | 635806 | 175561 |
| HILIC_NEG_182_82494a1_362 | HILIC | NEG | 1.362 | 182.8249 | | | | 2.74E-11 | 0.86 | 1.38 | 5.05E-01 | -0.14 | 0.84 | 4.33E-05 | 0.79 | 1.26 | 2.66E-09 | 0.93 | 1.98 | 119612 | 24852 | 189901 | 85831 | 195663 | 71636 | 184139 | 98411 |
| RP_NEG_296_89056a0_515 | RP | NEG | 0.52 | 296.8906 | | | | 6.15E-04 | 0.46 | 1.09 | 5.05E-01 | -0.15 | 1.03 | 2.35E-02 | 0.38 | 1.06 | 3.74E-03 | 0.53 | 1.24 | 433942 | 103939 | 555429 | 293155 | 575093 | 305305 | 535765 | 282195 |
| HILIC_NEG_236_96881a5_607 | HILIC | NEG | 5.607 | 236.9688 | | | | 3.09E-01 | -0.20 | 1.05 | 5.05E-01 | -0.14 | 0.59 | 2.42E-01 | -0.27 | 1.07 | 5.38E-01 | -0.13 | 1.02 | 234156 | 68614 | 219532 | 74574 | 224533 | 68546 | 214531 | 80539 |
| RP_POS_401_72394a5_863 | RP | POS | 5.86 | 401.7239 | | | | 4.66E-07 | 0.60 | 1.30 | 5.05E-01 | -0.15 | 1.16 | 6.70E-04 | 0.53 | 1.40 | 2.30E-04 | 0.67 | 1.38 | 94802 | 119317 | 2973467 | 5331625 | 3330875 | 5753820 | 2616060 | 4905654 |
| RP_POS_569_36292a12_46 | RP | POS | 12.46 | 569.3629 | | | | 2.15E-01 | 0.18 | 0.50 | 5.05E-01 | 0.15 | 0.53 | 2.48E-01 | 0.25 | 0.44 | 4.50E-01 | 0.10 | 0.71 | 862321 | 99949 | 897253 | 217291 | 882672 | 142774 | 911833 | 273086 |
| RP_POS_428_2305a6_184 | RP | POS | 6.18 | 428.2305 | | | | 1.38E-07 | 0.63 | 1.33 | 5.06E-01 | -0.15 | 1.18 | 4.54E-04 | 0.55 | 1.40 | 1.01E-04 | 0.70 | 1.43 | 23768 | 17660 | 656499 | 1114486 | 731180 | 1181848 | 581818 | 1049457 |
| HILIC_POS_140_14337a0_731 | HILIC | POS | 0.731 | 140.1434 | | | | 7.33E-05 | -0.64 | 1.10 | 5.06E-01 | -0.14 | 0.91 | 2.99E-04 | -0.71 | 1.26 | 2.71E-03 | -0.57 | 1.18 | 1990524 | 174312 | 1815881 | 284315 | 1834929 | 280879 | 1796832 | 289286 |
| RP_POS_1067_60181a10_312 | RP | POS | 10.31 | 1067.6018 | | | | 4.90E-03 | 0.49 | 1.07 | 5.06E-01 | -0.14 | 0.54 | 2.87E-02 | 0.42 | 0.95 | 9.06E-03 | 0.56 | 1.06 | 285212 | 172715 | 398278 | 242153 | 414501 | 264914 | 382054 | 218531 |
| HILIC_NEG_347_00293a7_428 | HILIC | NEG | 7.428 | 347.0029 | | | | 1.62E-04 | -0.59 | 1.21 | 5.06E-01 | 0.14 | 0.80 | 7.30E-03 | -0.52 | 1.28 | 4.49E-04 | -0.66 | 1.30 | 4001900 | 138462 | 3865400 | 246525 | 3848903 | 239385 | 3881897 | 254810 |
| HILIC_NEG_100_93346a7_355 | HILIC | NEG | 7.355 | 100.9335 | | | | 8.38E-02 | 0.35 | 0.91 | 5.06E-01 | -0.13 | 0.58 | 2.12E-01 | 0.29 | 0.61 | 6.08E-02 | 0.42 | 1.18 | 49329297 | 3916977 | 50733769 | 3945615 | 50997681 | 3774456 | 50469856 | 4130957 |
| RP_POS_261_91946a15_348 | RP | POS | 15.35 | 261.9195 | | | | 9.74E-01 | -0.01 | 0.60 | 5.06E-01 | -0.14 | 0.41 | 6.87E-01 | -0.08 | 0.32 | 7.62E-01 | 0.07 | 0.91 | 2372931 | 311574 | 2370646 | 429580 | 2399381 | 477596 | 2341910 | 378235 |
| RP_NEG_581_52936a14 | RP | NEG | 14.00 | 581.5294 | | | | 1.44E-01 | -0.39 | 0.74 | 5.07E-01 | 0.11 | 0.50 | 2.25E-01 | -0.34 | 0.59 | 1.14E-01 | -0.44 | 0.67 | 13824138 | 1730508 | 13343516 | 1008008 | 13276145 | 1043855 | 13410886 | 976729 |
| RP_POS_313_27371a9_607 | RP | POS | 9.61 | 313.2737 | | | | 2.28E-06 | 0.68 | 1.21 | 5.07E-01 | 0.14 | 1.06 | 1.27E-04 | 0.75 | 1.28 | 3.51E-04 | 0.60 | 1.25 | 575499 | 83643 | 694461 | 188001 | 681900 | 171632 | 707022 | 204036 |
| RP_NEG_319_2287a9_957 | RP | NEG | 9.96 | 319.2287 | | | | 5.60E-06 | 0.54 | 1.04 | 5.07E-01 | 0.15 | 1.07 | 8.05E-04 | 0.61 | 1.23 | 2.45E-03 | 0.46 | 1.09 | 71463 | 76371 | 1250998 | 2453871 | 1087082 | 2246521 | 1414914 | 2657850 |
| RP_POS_296_21942a8_755 | RP | POS | 8.76 | 296.2194 | | | | 7.11E-01 | -0.07 | 0.77 | 5.07E-01 | -0.14 | 0.55 | 5.55E-01 | -0.14 | 0.55 | 9.94E-01 | 0.00 | 0.44 | 1213887 | 129404 | 1203562 | 157792 | 1214108 | 119554 | 1193017 | 189169 |
| HILIC_POS_310_17575a1_904 | HILIC | POS | 1.904 | 310.1758 | | | | 4.09E-01 | -0.14 | 0.37 | 5.07E-01 | 0.14 | 0.58 | 7.75E-01 | -0.07 | 0.10 | 1.71E-01 | -0.21 | 0.77 | 757316 | 572856 | 648652 | 830061 | 593145 | 430584 | 704160 | 1095615 |
| RP_POS_196_00319a15_624 | RP | POS | 15.62 | 196.0032 | | | | 7.53E-01 | 0.06 | 0.29 | 5.07E-01 | -0.14 | 0.43 | 9.81E-01 | -0.01 | 0.23 | 5.68E-01 | 0.13 | 0.35 | 88045062 | 32250189 | 90150079 | 34169767 | 92429726 | 35955743 | 87870431 | 32489272 |
| RP_POS_393_2247a10_241 | RP | POS | 10.24 | 393.2247 | | | | 3.25E-06 | 0.55 | 1.08 | 5.08E-01 | -0.15 | 0.93 | 2.73E-03 | 0.48 | 1.09 | 4.20E-04 | 0.63 | 1.24 | 52461 | 56434 | 1106312 | 2133823 | 1248602 | 2234181 | 964022 | 2041186 |
| RP_POS_450_24313a6_292 | RP | POS | 6.29 | 450.2431 | | | | 1.29E-07 | 0.63 | 1.33 | 5.08E-01 | -0.15 | 1.18 | 4.23E-04 | 0.56 | 1.41 | 1.02E-04 | 0.70 | 1.43 | 12733 | 11710 | 443769 | 757254 | 494263 | 804687 | 393274 | 711229 |
| RP_POS_257_0426a4_852 | RP | POS | 4.85 | 257.0426 | | | | 3.06E-01 | -0.17 | 0.77 | 5.08E-01 | -0.14 | 0.92 | 2.22E-01 | -0.25 | 0.69 | 6.09E-01 | -0.10 | 0.87 | 3082601 | 903150 | 2870705 | 1295385 | 2957075 | 1308410 | 2784334 | 1289624 |
| RP_POS_465_28571a6_222 | RP | POS | 6.22 | 465.2857 | | | | 4.52E-07 | 0.60 | 1.30 | 5.08E-01 | -0.15 | 1.16 | 5.68E-04 | 0.53 | 1.40 | 2.52E-04 | 0.68 | 1.38 | 30518 | 60352 | 783848 | 1391320 | 876591 | 1514081 | 691105 | 1265328 |
| RP_POS_404_23285a5_992 | RP | POS | 5.99 | 404.2329 | | | | 4.10E-07 | 0.60 | 1.30 | 5.08E-01 | -0.15 | 1.16 | 6.36E-04 | 0.53 | 1.39 | 2.15E-04 | 0.68 | 1.38 | 15693 | 14572 | 1265352 | 2303142 | 1418770 | 2481818 | 1111934 | 2123611 |
| HILIC_POS_550_38513a1_176 | HILIC | POS | 1.176 | 550.3851 | | | | 6.17E-01 | -0.12 | 0.68 | 5.08E-01 | 0.12 | 0.96 | 8.23E-01 | -0.06 | 0.13 | 4.66E-01 | -0.18 | 0.45 | 1536729 | 767647 | 1462593 | 563635 | 1425049 | 483173 | 1500138 | 636804 |
| RP_NEG_170_87065a15_565 | RP | NEG | 15.57 | 170.8707 | | | | 1.36E-01 | -0.32 | 0.74 | 5.08E-01 | -0.13 | 0.41 | 9.48E-02 | -0.39 | 0.85 | 2.87E-01 | -0.26 | 0.57 | 1459924 | 60321 | 1441740 | 54890 | 1445395 | 58967 | 1438086 | 50823 |
| HILIC_NEG_112_98557a0_571 | HILIC | NEG | 0.571 | 112.9856 | | | | 2.69E-02 | 0.28 | 0.77 | 5.09E-01 | -0.15 | 0.56 | 1.51E-02 | 0.21 | 1.08 | 1.18E-01 | 0.36 | 0.73 | 2812829 | 765851 | 3502664 | 2768542 | 3687099 | 3776560 | 3318228 | 1074357 |
| RP_POS_548_5022a13_64 | RP | POS | 13.64 | 548.5022 | | | | 2.23E-01 | -0.26 | 0.61 | 5.10E-01 | -0.13 | 0.42 | 1.72E-01 | -0.33 | 0.83 | 3.95E-01 | -0.20 | 0.44 | 3841838 | 1151138 | 3559744 | 1042093 | 3628925 | 1007703 | 3490563 | 1081132 |
| RP_POS_663_45312a11_909 | RP | POS | 11.91 | 663.4531 | | | | 7.94E-01 | -0.05 | 0.42 | 5.10E-01 | 0.14 | 0.46 | 9.29E-01 | 0.02 | 0.34 | 5.87E-01 | -0.12 | 0.60 | 3470806 | 599210 | 3437643 | 694119 | 3391592 | 700310 | 3483695 | 691861 |
| RP_POS_412_74606a5_992 | RP | POS | 5.99 | 412.7461 | | | | 4.86E-07 | 0.60 | 1.30 | 5.10E-01 | -0.15 | 1.16 | 6.47E-04 | 0.53 | 1.39 | 2.43E-04 | 0.67 | 1.38 | 16547 | 40186 | 534219 | 959151 | 597848 | 1037362 | 470590 | 879964 |
| RP_POS_833_58527a13_877 | RP | POS | 13.88 | 833.5853 | | | | 3.03E-01 | -0.23 | 0.60 | 5.10E-01 | 0.13 | 0.78 | 5.20E-01 | -0.16 | 0.56 | 2.02E-01 | -0.29 | 0.54 | 36522166 | 10490852 | 34364354 | 9234253 | 33752107 | 7594037 | 34976601 | 10669991 |
| RP_POS_383_29425a11_157 | RP | POS | 11.16 | 383.2943 | | | | 4.77E-01 | -0.13 | 0.70 | 5.10E-01 | 0.14 | 0.69 | 7.56E-01 | -0.07 | 0.51 | 3.57E-01 | -0.20 | 0.83 | 1768686 | 358501 | 1714222 | 421122 | 1686323 | 440385 | 1742120 | 403458 |
| RP_POS_137_06573a15_299 | RP | POS | 15.30 | 137.0657 | | | | 1.31E-01 | 0.28 | 0.60 | 5.10E-01 | -0.14 | 0.65 | 3.04E-01 | 0.22 | 0.58 | 1.08E-01 | 0.35 | 0.73 | 3109380 | 901417 | 3401851 | 1057821 | 3471929 | 1102767 | 3331773 | 1017197 |
| RP_POS_297_18558a11_136 | RP | POS | 11.14 | 297.1856 | | | | 4.73E-02 | -0.36 | 0.81 | 5.11E-01 | 0.14 | 0.59 | 2.02E-01 | -0.29 | 0.57 | 2.56E-02 | -0.42 | 0.96 | 642143 | 138586 | 580568 | 181273 | 568558 | 148372 | 592577 | 209958 |
| RP_POS_447_76569a6_292 | RP | POS | 6.29 | 447.7657 | | | | 1.46E-07 | 0.63 | 1.33 | 5.11E-01 | -0.15 | 1.18 | 4.61E-04 | 0.55 | 1.40 | 1.06E-04 | 0.70 | 1.43 | 37596 | 21661 | 818853 | 1379152 | 910154 | 1462308 | 727551 | 1299069 |
| RP_POS_631_35333a5_401 | RP | POS | 5.40 | 631.3533 | | | | 4.92E-06 | 0.55 | 1.27 | 5.11E-01 | -0.15 | 1.14 | 2.21E-03 | 0.48 | 1.35 | 6.44E-04 | 0.62 | 1.34 | 51911 | 138298 | 576421 | 1063128 | 646786 | 1144059 | 506055 | 982126 |
| HILIC_NEG_153_86861a0_55 | HILIC | NEG | 0.55 | 153.8686 | | | | 3.38E-03 | -0.48 | 0.81 | 5.11E-01 | 0.14 | 0.57 | 5.40E-02 | -0.41 | 0.64 | 2.19E-03 | -0.55 | 1.47 | 864522 | 147372 | 758126 | 237944 | 742377 | 201074 | 773875 | 271023 |
| HILIC_POS_371_23965a6_159 | HILIC | POS | 6.159 | 371.2397 | | | | 5.37E-01 | -0.10 | 0.95 | 5.11E-01 | -0.14 | 0.79 | 4.12E-01 | -0.17 | 0.76 | 8.63E-01 | -0.03 | 0.58 | 2511925 | 443682 | 2448237 | 663951 | 2492170 | 585651 | 2404304 | 737382 |
| RP_POS_135_89145a11_667 | RP | POS | 11.67 | 135.8915 | | | | 9.25E-01 | -0.02 | 0.69 | 5.11E-01 | -0.13 | 0.39 | 7.07E-01 | -0.09 | 0.38 | 8.39E-01 | 0.05 | 0.45 | 526610 | 319519 | 520461 | 316665 | 541400 | 321813 | 499522 | 313276 |
| RP_POS_423_19977a6_582 | RP | POS | 6.58 | 423.1998 | | | | 2.74E-03 | -0.50 | 1.06 | 5.11E-01 | 0.14 | 0.93 | 3.26E-02 | -0.43 | 0.99 | 3.59E-03 | -0.57 | 1.19 | 1318735 | 53683 | 1279876 | 82496 | 1274425 | 79998 | 1285328 | 85381 |
| RP_POS_423_73691a5_992 | RP | POS | 5.99 | 423.7369 | | | | 3.14E-07 | 0.61 | 1.31 | 5.12E-01 | -0.15 | 1.17 | 6.21E-04 | 0.54 | 1.40 | 1.70E-04 | 0.68 | 1.40 | 77285 | 85737 | 2752049 | 4872755 | 3073953 | 5203792 | 2430146 | 4547650 |
| RP_POS_282_20383a7_8 | RP | POS | 7.80 | 282.2038 | | | | 1.34E-01 | 0.27 | 0.64 | 5.12E-01 | -0.14 | 0.42 | 3.01E-01 | 0.20 | 0.49 | 1.26E-01 | 0.34 | 0.60 | 662756 | 506934 | 831172 | 652945 | 874310 | 731177 | 788034 | 568293 |
| HILIC_NEG_151_87399a3_022 | HILIC | NEG | 3.022 | 151.8740 | | | | 1.28E-01 | -0.31 | 0.99 | 5.12E-01 | -0.13 | 0.85 | 9.48E-02 | -0.38 | 0.63 | 2.84E-01 | -0.25 | 0.45 | 1698262 | 334504 | 1592841 | 338180 | 1615173 | 347664 | 1570510 | 330419 |
| RP_POS_381_29681a11_263 | RP | POS | 11.26 | 381.2968 | | | | 1.17E-01 | 0.23 | 0.54 | 5.12E-01 | 0.15 | 0.50 | 1.33E-01 | 0.31 | 0.70 | 3.40E-01 | 0.16 | 0.57 | 10813376 | 9189852 | 14676012 | 18306126 | 13467288 | 15810719 | 15884735 | 20593860 |
| RP_POS_772_85352a0_494 | RP | POS | 0.49 | 772.8535 | | | | 1.39E-07 | 0.63 | 1.34 | 5.12E-01 | -0.15 | 1.21 | 2.90E-04 | 0.56 | 1.40 | 1.32E-04 | 0.71 | 1.46 | 91907 | 57168 | 424998 | 580857 | 463343 | 630177 | 386653 | 530615 |
| RP_POS_346_70688a5_569 | RP | POS | 5.57 | 346.7069 | | | | 1.98E-06 | 0.57 | 1.28 | 5.12E-01 | -0.15 | 1.15 | 1.49E-03 | 0.50 | 1.37 | 4.19E-04 | 0.64 | 1.36 | 39117 | 55634 | 352285 | 613547 | 392787 | 658039 | 311784 | 569386 |
| RP_POS_820_5448a11_69 | RP | POS | 11.69 | 820.5448 | | | | 5.71E-02 | 0.24 | 0.61 | 5.12E-01 | -0.15 | 0.58 | 2.82E-01 | 0.17 | 0.46 | 9.02E-02 | 0.32 | 0.73 | 458443 | 554438 | 881207 | 1972366 | 1011367 | 2160726 | 751046 | 1776665 |
| RP_POS_615_54913a11_254 | RP | POS | 11.25 | 615.5491 | | | | 4.47E-02 | 0.40 | 1.24 | 5.12E-01 | 0.13 | 1.08 | 4.81E-02 | 0.46 | 1.08 | 1.11E-01 | 0.33 | 1.29 | 459683 | 296608 | 585757 | 318977 | 564706 | 271953 | 606808 | 361534 |
| RP_NEG_326_76001a0_521 | RP | NEG | 0.52 | 326.7600 | | | | 2.06E-05 | 0.52 | 1.25 | 5.12E-01 | -0.15 | 1.16 | 3.63E-03 | 0.45 | 1.25 | 1.40E-03 | 0.59 | 1.37 | 633628 | 109110 | 912550 | 597946 | 951980 | 653679 | 873120 | 540284 |
| HILIC_POS_254_15956a2_368 | HILIC | POS | 2.368 | 254.1596 | | | | 1.05E-02 | 0.34 | 1.05 | 5.12E-01 | -0.15 | 0.74 | 9.02E-02 | 0.27 | 1.15 | 3.15E-02 | 0.41 | 1.14 | 405683 | 336548 | 699904 | 963954 | 763471 | 1070225 | 636338 | 850736 |
| RP_POS_675_37909a5_567 | RP | POS | 5.57 | 675.3791 | | | | 1.90E-06 | 0.57 | 1.27 | 5.13E-01 | -0.15 | 1.14 | 1.24E-03 | 0.50 | 1.37 | 4.73E-04 | 0.64 | 1.35 | 55898 | 66003 | 435816 | 743315 | 484757 | 806484 | 386875 | 678973 |
| RP_POS_226_1794a7_715 | RP | POS | 7.72 | 226.1794 | | | | 6.48E-01 | -0.10 | 0.55 | 5.13E-01 | 0.13 | 0.40 | 8.90E-01 | -0.03 | 0.41 | 4.63E-01 | -0.16 | 0.43 | 3502144 | 625366 | 3445314 | 558723 | 3408520 | 437561 | 3482108 | 660674 |
| RP_POS_301_65186a10_515 | RP | POS | 10.52 | 301.6519 | | | | 5.79E-01 | -0.12 | 0.74 | 5.13E-01 | 0.13 | 0.50 | 8.23E-01 | -0.05 | 0.54 | 4.25E-01 | -0.18 | 0.82 | 5863099 | 1600603 | 5684500 | 1483874 | 5586861 | 1384958 | 5782138 | 1584657 |
| RP_POS_341_26578a10_433 | RP | POS | 10.43 | 341.2658 | | | | 8.52E-01 | 0.03 | 1.20 | 5.13E-01 | -0.14 | 0.53 | 8.47E-01 | -0.04 | 0.70 | 6.36E-01 | 0.10 | 1.01 | 8654524 | 1345948 | 8710658 | 1827418 | 8830913 | 2015792 | 8590404 | 1629151 |
| RP_NEG_184_95595a15_665 | RP | NEG | 15.67 | 184.9560 | | | | 5.18E-01 | -0.12 | 0.95 | 5.13E-01 | -0.14 | 0.59 | 3.82E-01 | -0.19 | 0.54 | 8.04E-01 | -0.05 | 0.72 | 1916127 | 462326 | 1852084 | 550145 | 1888275 | 541676 | 1815893 | 561614 |
| RP_POS_368_72009a5_721 | RP | POS | 5.72 | 368.7201 | | | | 1.10E-06 | 0.58 | 1.29 | 5.13E-01 | -0.15 | 1.15 | 1.02E-03 | 0.51 | 1.38 | 3.46E-04 | 0.65 | 1.37 | 31151 | 65499 | 531151 | 957170 | 594119 | 1032444 | 468184 | 881484 |
| RP_POS_355_22006a5_569 | RP | POS | 5.57 | 355.2201 | | | | 7.75E-07 | 0.59 | 1.29 | 5.13E-01 | -0.15 | 1.14 | 9.05E-04 | 0.51 | 1.38 | 2.86E-04 | 0.66 | 1.37 | 32925 | 68145 | 957350 | 1748717 | 1072377 | 1879721 | 842323 | 1618047 |
| RP_POS_302_19531a2_424 | RP | POS | 2.42 | 302.1953 | | | | 6.18E-02 | -0.39 | 0.72 | 5.14E-01 | 0.13 | 0.75 | 1.78E-01 | -0.33 | 0.58 | 3.78E-02 | -0.45 | 0.82 | 493805 | 218684 | 409622 | 211717 | 395694 | 177871 | 423550 | 241928 |
| HILIC_NEG_145_05064a0_658 | HILIC | NEG | 0.658 | 145.0506 | | | | 8.36E-01 | -0.04 | 0.68 | 5.14E-01 | 0.14 | 0.81 | 8.98E-01 | 0.03 | 0.15 | 6.17E-01 | -0.11 | 0.67 | 368007 | 85874 | 364246 | 98113 | 357795 | 95798 | 370698 | 100929 |
| RP_POS_277_13989a7_727 | RP | POS | 7.73 | 277.1399 | | | | 2.04E-02 | -0.41 | 0.70 | 5.14E-01 | -0.14 | 0.61 | 2.74E-02 | -0.48 | 0.98 | 7.83E-02 | -0.34 | 0.76 | 20202048 | 1153824 | 19593392 | 1555275 | 19695650 | 1394097 | 19491135 | 1709468 |
| RP_POS_500_27454a10_214 | RP | POS | 10.21 | 500.2745 | | | | 1.21E-01 | 0.30 | 1.04 | 5.14E-01 | -0.13 | 0.80 | 3.21E-01 | 0.23 | 0.77 | 6.97E-02 | 0.37 | 1.01 | 1667417 | 758494 | 1918069 | 856319 | 1974378 | 695905 | 1861759 | 995376 |
| RP_POS_338_19336a5_569 | RP | POS | 5.57 | 338.1934 | | | | 5.06E-07 | 0.60 | 1.29 | 5.14E-01 | -0.15 | 1.15 | 7.80E-04 | 0.52 | 1.38 | 2.21E-04 | 0.67 | 1.38 | 7537 | 8293 | 727056 | 1338061 | 814986 | 1431120 | 639125 | 1246383 |
| HILIC_NEG_262_98499a4_801 | HILIC | NEG | 4.801 | 262.9850 | | | | 4.17E-01 | 0.16 | 0.46 | 5.14E-01 | 0.13 | 0.49 | 2.84E-01 | 0.23 | 0.49 | 6.83E-01 | 0.10 | 0.31 | 405559 | 207754 | 440480 | 217723 | 426172 | 242562 | 454788 | 191103 |
| RP_POS_399_24637a5_862 | RP | POS | 5.86 | 399.2464 | | | | 3.91E-07 | 0.60 | 1.30 | 5.15E-01 | -0.15 | 1.16 | 5.99E-04 | 0.53 | 1.40 | 2.16E-04 | 0.68 | 1.38 | 36851 | 49560 | 1438988 | 2577890 | 1607986 | 2779424 | 1269991 | 2375537 |

**Supplementary table 1: Metabolomics results** (in asceding order according to p-value from progressive ALD vs non-progressive ALD comparison, with molecular features with ID_level of 1-2 first)

| | | | | | | | | all_ALD_vs_control | | | Prog_ALD_vs_non-prog_ALD | | | Prog_ALD_vs_control_ALD | | | Non-prog_ALD_vs_control | | | Healthy_control | | all_ALD | | Non-prog_ALD | | Progressive_ALD | |
|---|---|---|---|---|---|---|---|---|---|---|---|---|---|---|---|---|---|---|---|---|---|---|---|---|---|---|---|
| | | | | | | | | t-test | Cohen's | PLS-DA | t-test | Cohen's | PLS-DA | t-test | Cohen's | PLS-DA | t-test | Cohen's | PLS-DA | | | | | | | | |
| Feature_ID | RP_HILIC | POS_NEG | Average.Rt | Average.Mz | Metabolite.name | curated_MSMS | ID_level | p | d | VIP | p | d | VIP | p | d | VIP | p | d | VIP | Mean | SD | Mean | SD | Mean | SD | Mean | SD |
| RP_POS_441_29718a13_89 | RP | POS | 13.89 | 441.2972 | | | | 5.44E-01 | -0.11 | 0.51 | 5.15E-01 | -0.14 | 0.61 | 3.91E-01 | -0.18 | 0.65 | 8.41E-01 | -0.04 | 0.46 | 16155114 | 6188433 | 15340922 | 7646994 | 15841765 | 7838478 | 14840079 | 7496183 |
| RP_POS_341_18451a10_978 | RP | POS | 10.98 | 341.1845 | | | | 4.80E-03 | 0.47 | 1.36 | 5.15E-01 | -0.14 | 1.21 | 4.62E-02 | 0.40 | 1.13 | 5.29E-03 | 0.54 | 1.36 | 1638115 | 730419 | 2129259 | 1093349 | 2200865 | 1044668 | 2057653 | 1146047 |
| RP_POS_780_45776a5_862 | RP | POS | 5.86 | 780.4578 | | | | 5.91E-07 | 0.59 | 1.28 | 5.15E-01 | -0.15 | 1.14 | 6.49E-04 | 0.52 | 1.39 | 2.93E-04 | 0.67 | 1.36 | 21565 | 31219 | 500750 | 895998 | 559434 | 974680 | 442066 | 815462 |
| RP_POS_393_21054a5_862 | RP | POS | 5.86 | 393.2105 | | | | 2.89E-07 | 0.61 | 1.31 | 5.15E-01 | -0.15 | 1.17 | 5.69E-04 | 0.54 | 1.40 | 1.70E-04 | 0.68 | 1.40 | 18354 | 15835 | 594667 | 1046238 | 663183 | 1119645 | 526151 | 973848 |
| RP_POS_494_22888a11_804 | RP | POS | 11.80 | 494.2289 | | | | 4.81E-03 | -0.57 | 1.28 | 5.15E-01 | 0.13 | 1.33 | 3.60E-02 | -0.51 | 0.93 | 2.07E-03 | -0.64 | 1.36 | 3131408 | 1157487 | 2433920 | 1198432 | 2355382 | 882250 | 2512457 | 1452872 |
| RP_POS_199_01114a10_445 | RP | POS | 10.45 | 199.0111 | | | | 4.17E-01 | -0.20 | 1.09 | 5.15E-01 | 0.12 | 0.70 | 6.09E-01 | -0.14 | 0.91 | 2.98E-01 | -0.26 | 0.50 | 3290890 | 1512297 | 3054611 | 1069276 | 2984545 | 810024 | 3124677 | 1282138 |
| RP_POS_494_80829a0_5 | RP | POS | 0.50 | 494.8083 | | | | 1.13E-01 | -0.23 | 1.06 | 5.15E-01 | -0.15 | 1.24 | 8.05E-02 | -0.30 | 1.06 | 4.19E-01 | -0.16 | 1.12 | 2272249 | 667349 | 1974439 | 1443230 | 2068942 | 1559600 | 1879936 | 1325805 |
| RP_POS_226_18044a11_042 | RP | POS | 11.04 | 226.1804 | | | | 1.82E-02 | 0.40 | 0.77 | 5.15E-01 | -0.14 | 0.42 | 7.75E-02 | 0.33 | 0.66 | 2.79E-02 | 0.47 | 0.80 | 356742 | 363727 | 559371 | 540851 | 594786 | 598774 | 523957 | 479500 |
| RP_POS_276_65277a10_508 | RP | POS | 10.51 | 276.6528 | | | | 5.56E-01 | -0.13 | 0.55 | 5.16E-01 | 0.13 | 0.43 | 7.87E-01 | -0.07 | 0.49 | 4.09E-01 | -0.19 | 0.53 | 552092 | 215582 | 526905 | 186442 | 514700 | 168077 | 539111 | 204148 |
| RP_POS_650_474a11_821 | RP | POS | 11.82 | 650.4740 | | | | 4.78E-01 | 0.12 | 0.70 | 5.16E-01 | 0.14 | 0.61 | 4.32E-01 | 0.19 | 0.51 | 7.21E-01 | 0.05 | 0.31 | 588026 | 980578 | 743926 | 1328380 | 656817 | 568625 | 831035 | 1796212 |
| RP_NEG_241_14413a8_771 | RP | NEG | 8.77 | 241.1441 | | | | 4.25E-01 | -0.15 | 0.40 | 5.16E-01 | -0.14 | 0.90 | 2.95E-01 | -0.21 | 0.54 | 7.24E-01 | -0.08 | 0.36 | 1934062 | 115908 | 1913824 | 146527 | 1923406 | 155805 | 1904242 | 137534 |
| RP_POS_403_2493a11_61 | RP | POS | 11.61 | 403.2493 | | | | 8.69E-03 | -0.54 | 0.97 | 5.16E-01 | 0.13 | 0.73 | 2.69E-02 | -0.48 | 0.91 | 1.11E-02 | -0.60 | 0.96 | 6804001 | 817909 | 6351294 | 816280 | 6297910 | 914716 | 6404677 | 709721 |
| HILIC_NEG_113_92627a5_606 | HILIC | NEG | 5.606 | 113.9263 | | | | 6.91E-01 | -0.08 | 0.66 | 5.16E-01 | -0.13 | 0.64 | 4.92E-01 | -0.14 | 0.27 | 9.65E-01 | -0.01 | 0.23 | 2354692 | 403032 | 2321061 | 447036 | 2350290 | 491830 | 2291833 | 400151 |
| RP_POS_822_56299a11_684 | RP | POS | 11.68 | 822.5630 | | | | 6.88E-01 | 0.06 | 0.35 | 5.16E-01 | -0.14 | 0.53 | 9.46E-01 | -0.01 | 0.20 | 5.38E-01 | 0.13 | 0.42 | 2748001 | 3752521 | 3134094 | 6933489 | 3587639 | 8381761 | 2680550 | 5143337 |
| HILIC_POS_349_01785a7_45 | HILIC | POS | 7.45 | 349.0179 | | | | 8.40E-01 | -0.03 | 0.95 | 5.16E-01 | -0.14 | 0.62 | 5.58E-01 | -0.10 | 0.76 | 8.66E-01 | 0.04 | 0.93 | 14109634 | 1010380 | 14061955 | 1526658 | 14161762 | 1772789 | 13962147 | 1243311 |
| RP_POS_394_2597a11_668 | RP | POS | 11.67 | 394.2597 | | | | 6.89E-01 | -0.08 | 0.82 | 5.16E-01 | -0.13 | 0.43 | 5.12E-01 | -0.15 | 0.56 | 9.60E-01 | -0.01 | 0.60 | 567087 | 291103 | 542659 | 320689 | 563614 | 320020 | 521704 | 323222 |
| RP_POS_263_23682a9_617 | RP | POS | 9.62 | 263.2368 | | | | 3.82E-01 | -0.24 | 0.85 | 5.16E-01 | -0.10 | 0.44 | 3.07E-01 | -0.29 | 0.67 | 5.08E-01 | -0.19 | 0.56 | 2271563 | 4049339 | 1610535 | 2142773 | 1750537 | 2204047 | 1470533 | 2092566 |
| RP_POS_419_31509a14_258 | RP | POS | 14.26 | 419.3151 | | | | 8.21E-03 | 0.44 | 0.87 | 5.17E-01 | -0.14 | 0.53 | 6.70E-02 | 0.38 | 0.65 | 7.90E-03 | 0.51 | 1.03 | 4202253 | 1980780 | 5445829 | 2960161 | 5638964 | 2786279 | 5252695 | 3140772 |
| RP_POS_161_13269a8_506 | RP | POS | 8.51 | 161.1327 | | | | 4.15E-01 | -0.13 | 0.66 | 5.17E-01 | 0.14 | 0.70 | 7.83E-01 | -0.06 | 0.59 | 2.64E-01 | -0.20 | 0.71 | 11411112 | 194639 | 11371525 | 339180 | 11349394 | 302495 | 11393657 | 374074 |
| RP_POS_277_06094a3_314 | RP | POS | 3.31 | 277.0609 | | | | 7.99E-04 | -0.53 | 1.19 | 5.17E-01 | 0.14 | 1.13 | 1.44E-02 | -0.46 | 1.15 | 2.04E-03 | -0.60 | 1.27 | 1444916 | 355635 | 1142832 | 602055 | 1103579 | 612132 | 1182085 | 595389 |
| RP_POS_791_52747a12_325 | RP | POS | 12.33 | 791.5275 | | | | 1.58E-06 | -0.97 | 1.64 | 5.18E-01 | -0.12 | 0.47 | 4.09E-06 | -1.03 | 1.65 | 2.09E-05 | -0.91 | 1.61 | 1304874 | 266342 | 1010058 | 279257 | 1028246 | 267239 | 991871 | 292349 |
| RP_POS_517_83667a15_512 | RP | POS | 15.51 | 517.8367 | | | | 3.46E-02 | -0.33 | 0.88 | 5.18E-01 | 0.14 | 0.60 | 1.87E-01 | -0.25 | 0.56 | 3.33E-02 | -0.40 | 1.07 | 1410028 | 147510 | 1329965 | 267429 | 1312550 | 259237 | 1347380 | 276900 |
| HILIC_POS_113_52961a1_232 | HILIC | POS | 1.232 | 113.5296 | | | | 1.91E-01 | -0.34 | 0.68 | 5.18E-01 | -0.11 | 0.63 | 1.55E-01 | -0.39 | 0.60 | 2.89E-01 | -0.28 | 0.48 | 1184281 | 840354 | 974328 | 530341 | 1008865 | 485055 | 939792 | 574935 |
| RP_POS_556_85919a0_503 | RP | POS | 0.50 | 556.8592 | | | | 1.58E-01 | 0.20 | 0.96 | 5.18E-01 | 0.15 | 1.03 | 1.29E-01 | 0.27 | 0.81 | 4.92E-01 | 0.13 | 1.14 | 789245 | 124445 | 841163 | 292317 | 822139 | 298743 | 860188 | 287500 |
| HILIC_POS_128_10716a0_538 | HILIC | POS | 0.538 | 128.1072 | | | | 3.53E-03 | 0.34 | 0.69 | 5.18E-01 | 0.15 | 0.65 | 3.89E-02 | 0.42 | 0.75 | 2.52E-02 | 0.27 | 0.93 | 600399 | 992309 | 3539173 | 9687347 | 2908273 | 6973409 | 4170074 | 11839081 |
| HILIC_NEG_174_95583a8_032 | HILIC | NEG | 8.032 | 174.9558 | | | | 1.27E-03 | 0.39 | 0.65 | 5.18E-01 | 0.15 | 0.64 | 1.00E-02 | 0.47 | 0.79 | 4.19E-02 | 0.32 | 0.98 | 34683799 | 11855512 | 56544693 | 62767594 | 52460391 | 58497416 | 60628995 | 67111502 |
| HILIC_NEG_176_84979a1_366 | HILIC | NEG | 1.366 | 176.8498 | | | | 7.23E-01 | -0.05 | 0.76 | 5.18E-01 | -0.14 | 0.74 | 5.15E-01 | -0.13 | 0.25 | 9.19E-01 | 0.02 | 0.30 | 566246 | 66196 | 560103 | 127316 | 568382 | 122957 | 551824 | 132254 |
| HILIC_NEG_341_9996a6_271 | HILIC | NEG | 6.271 | 341.9996 | | | | 1.03E-01 | -0.38 | 0.72 | 5.18E-01 | -0.12 | 0.61 | 8.32E-02 | -0.44 | 0.70 | 1.94E-01 | -0.32 | 0.85 | 1031110 | 442372 | 888536 | 347714 | 911142 | 331972 | 865929 | 364731 |
| RP_POS_468_30856a10_733 | RP | POS | 10.73 | 468.3086 | | | | 5.36E-05 | -0.85 | 1.54 | 5.19E-01 | -0.12 | 0.94 | 6.99E-05 | -0.91 | 1.50 | 4.62E-04 | -0.79 | 1.43 | 408103 | 183603 | 244889 | 177924 | 256452 | 178290 | 233327 | 178605 |
| RP_POS_293_20706a9_581 | RP | POS | 9.58 | 293.2071 | | | | 9.85E-01 | 0.00 | 0.63 | 5.19E-01 | -0.13 | 0.79 | 7.65E-01 | -0.07 | 0.43 | 7.99E-01 | 0.06 | 0.76 | 11699448 | 1537346 | 11693451 | 1458193 | 11788197 | 1528263 | 11598706 | 1393607 |
| RP_POS_494_35941a10_841 | RP | POS | 10.84 | 494.3594 | | | | 5.24E-04 | -0.73 | 1.25 | 5.19E-01 | -0.12 | 1.02 | 4.95E-04 | -0.80 | 1.31 | 3.59E-03 | -0.67 | 1.15 | 667814 | 335088 | 418248 | 320250 | 439048 | 333848 | 397447 | 308020 |
| RP_POS_273_20584a9_88 | RP | POS | 9.88 | 273.2058 | | | | 2.33E-01 | 0.21 | 0.52 | 5.19E-01 | -0.14 | 0.42 | 4.70E-01 | 0.14 | 0.40 | 1.95E-01 | 0.28 | 0.57 | 1374945 | 581143 | 1529901 | 777077 | 1580375 | 840253 | 1479427 | 713300 |
| RP_NEG_655_474a10_907 | RP | NEG | 10.91 | 655.4740 | | | | 3.95E-04 | 0.45 | 1.36 | 5.19E-01 | 0.15 | 0.57 | 9.55E-03 | 0.52 | 1.05 | 7.21E-03 | 0.37 | 1.39 | 435553 | 334235 | 967116 | 1335158 | 880368 | 1052705 | 1053865 | 1574207 |
| RP_POS_509_33554a10_318 | RP | POS | 10.32 | 509.3355 | | | | 8.73E-02 | 0.27 | 0.70 | 5.19E-01 | 0.14 | 0.61 | 9.16E-02 | 0.34 | 0.74 | 2.72E-01 | 0.20 | 0.50 | 477976 | 1450572 | 1089979 | 2441417 | 931460 | 2259028 | 1248498 | 2624536 |
| RP_NEG_313_23868a11_337 | RP | NEG | 11.34 | 313.2387 | | | | 2.50E-01 | -0.23 | 0.77 | 5.19E-01 | -0.13 | 0.67 | 1.67E-01 | -0.30 | 0.65 | 4.83E-01 | -0.16 | 0.61 | 2145790 | 164810 | 2106291 | 174100 | 2117594 | 193667 | 2094987 | 153207 |
| RP_POS_172_16966a8_53 | RP | POS | 8.53 | 172.1697 | | | | 2.65E-01 | 0.26 | 0.65 | 5.19E-01 | -0.12 | 0.47 | 4.37E-01 | 0.20 | 0.61 | 1.91E-01 | 0.32 | 0.54 | 3025434 | 1844090 | 3427872 | 1438193 | 3521221 | 1292836 | 3334524 | 1577911 |
| RP_NEG_285_24506a10_947 | RP | NEG | 10.95 | 285.2451 | | | | 3.43E-02 | -0.35 | 0.91 | 5.19E-01 | 0.14 | 0.88 | 1.31E-01 | -0.28 | 0.85 | 4.55E-02 | -0.42 | 0.88 | 771863 | 111439 | 715199 | 174633 | 703867 | 191301 | 726531 | 157345 |
| RP_POS_283_00754a4_77 | RP | POS | 4.77 | 283.0075 | | | | 6.29E-04 | -0.70 | 1.22 | 5.19E-01 | -0.13 | 0.51 | 8.36E-04 | -0.76 | 1.29 | 3.96E-03 | -0.63 | 1.32 | 1659658 | 574296 | 1233259 | 589157 | 1271483 | 573996 | 1195036 | 607324 |
| RP_POS_368_38901a12_71 | RP | POS | 12.71 | 368.3890 | | | | 1.46E-01 | -0.29 | 0.75 | 5.20E-01 | 0.13 | 0.43 | 3.25E-01 | -0.22 | 0.51 | 1.08E-01 | -0.35 | 0.86 | 190002 | 140323 | 147250 | 150675 | 137483 | 145278 | 157017 | 156745 |
| RP_POS_339_02252a4_855 | RP | POS | 4.86 | 339.0225 | | | | 7.36E-01 | 0.06 | 0.51 | 5.20E-01 | -0.13 | 0.59 | 9.93E-01 | 0.00 | 0.41 | 5.57E-01 | 0.13 | 0.50 | 477980 | 202594 | 492393 | 229066 | 507239 | 242973 | 477548 | 215703 |
| RP_NEG_311_16949a10_264 | RP | NEG | 10.26 | 311.1695 | | | | 4.24E-01 | -0.16 | 0.61 | 5.20E-01 | -0.13 | 0.48 | 2.95E-01 | -0.22 | 0.57 | 6.88E-01 | -0.09 | 0.81 | 12020661 | 5524202 | 11102345 | 5870869 | 11482810 | 6440404 | 10721879 | 5278579 |
| RP_POS_333_20703a5_403 | RP | POS | 5.40 | 333.2070 | | | | 6.10E-07 | 0.59 | 1.30 | 5.20E-01 | -0.14 | 1.15 | 7.89E-04 | 0.52 | 1.38 | 2.54E-04 | 0.67 | 1.38 | 25456 | 41257 | 477484 | 845248 | 532235 | 907338 | 422733 | 783638 |
| RP_POS_371_23257a9_006 | RP | POS | 9.01 | 371.2326 | | | | 3.62E-01 | 0.16 | 0.58 | 5.20E-01 | -0.14 | 0.42 | 6.09E-01 | 0.10 | 0.57 | 3.10E-01 | 0.23 | 0.57 | 676146 | 976026 | 872216 | 1260336 | 953886 | 1487595 | 790546 | 991342 |
| RP_POS_740_52185a13_063 | RP | POS | 13.06 | 740.5219 | | | | 1.79E-02 | 0.47 | 0.80 | 5.20E-01 | 0.13 | 0.83 | 1.56E-02 | 0.54 | 0.95 | 6.79E-02 | 0.41 | 0.79 | 2227124 | 1228821 | 2846077 | 1311224 | 2761199 | 1336861 | 2930954 | 1292984 |
| RP_NEG_163_97694a15_452 | RP | NEG | 15.45 | 163.9769 | | | | 8.52E-01 | 0.04 | 0.28 | 5.20E-01 | -0.12 | 0.49 | 9.50E-01 | -0.02 | 0.24 | 6.81E-01 | 0.10 | 0.48 | 2311168 | 225723 | 2319342 | 172013 | 2330468 | 171910 | 2308216 | 173134 |
| RP_POS_820_50549a12_531 | RP | POS | 12.53 | 820.5055 | | | | 6.16E-01 | 0.10 | 0.80 | 5.21E-01 | 0.13 | 0.38 | 4.75E-01 | 0.17 | 0.84 | 8.63E-01 | 0.04 | 0.16 | 1026120 | 535501 | 1081049 | 534555 | 1046485 | 495755 | 1115613 | 573688 |
| HILIC_POS_139_05022a0_701 | HILIC | POS | 0.701 | 139.0502 | | | | 2.26E-01 | -0.40 | 0.78 | 5.21E-01 | 0.06 | 0.65 | 2.70E-01 | -0.37 | 0.53 | 1.95E-01 | -0.43 | 0.71 | 1481998 | 3015196 | 815956 | 813386 | 763369 | 737861 | 868543 | 886912 |
| RP_POS_615_38635a11_897 | RP | POS | 11.90 | 615.3864 | | | | 1.34E-01 | 0.25 | 0.69 | 5.21E-01 | 0.14 | 0.56 | 1.25E-01 | 0.32 | 0.62 | 3.32E-01 | 0.18 | 0.46 | 1020371 | 2195729 | 1784724 | 3220514 | 1576602 | 2947829 | 1992847 | 3489554 |
| RP_POS_136_65872a11_668 | RP | POS | 11.67 | 136.6587 | | | | 9.17E-01 | -0.02 | 0.68 | 5.21E-01 | -0.13 | 0.38 | 7.02E-01 | -0.09 | 0.39 | 8.47E-01 | 0.04 | 0.44 | 2326330 | 1250905 | 2299715 | 1276585 | 2382177 | 1306464 | 2217253 | 1253742 |
| RP_POS_338_21585a6_399 | RP | POS | 6.40 | 338.2159 | | | | 5.59E-02 | -0.37 | 0.65 | 5.21E-01 | 0.13 | 0.60 | 1.62E-01 | -0.30 | 0.55 | 4.74E-02 | -0.43 | 0.77 | 790457 | 49297 | 770299 | 55537 | 766713 | 55925 | 773886 | 55478 |
| HILIC_POS_87_09225a0_798 | HILIC | POS | 0.798 | 87.0923 | | | | 1.39E-03 | 0.64 | 1.33 | 5.21E-01 | -0.13 | 0.89 | 1.09E-02 | 0.58 | 1.04 | 1.18E-03 | 0.70 | 1.56 | 5816366 | 2427873 | 7503271 | 2583598 | 7670138 | 2394606 | 7336405 | 2774025 |
| HILIC_POS_130_08646a3_688 | HILIC | POS | 3.688 | 130.0865 | | | | 8.18E-01 | -0.04 | 0.27 | 5.21E-01 | -0.14 | 0.64 | 5.16E-01 | -0.11 | 0.45 | 8.90E-01 | 0.03 | 0.31 | 16286094 | 19751203 | 15226110 | 29910965 | 17159526 | 36853654 | 13292695 | 21018317 |
| RP_POS_426_21432a5_992 | RP | POS | 5.99 | 426.2143 | | | | 3.00E-07 | 0.61 | 1.31 | 5.21E-01 | -0.14 | 1.16 | 5.13E-04 | 0.54 | 1.41 | 1.92E-04 | 0.68 | 1.39 | 52030 | 63845 | 1738714 | 3066230 | 1936713 | 3303303 | 1540714 | 2829061 |
| RP_POS_445_28796a6_292 | RP | POS | 6.29 | 445.2880 | | | | 1.55E-07 | 0.63 | 1.32 | 5.21E-01 | -0.14 | 1.18 | 4.38E-04 | 0.55 | 1.41 | 1.17E-04 | 0.70 | 1.42 | 15654 | 16169 | 438219 | 747577 | 486472 | 795052 | 389966 | 701632 |
| RP_POS_526_39819a14_267 | RP | POS | 14.27 | 526.3982 | | | | 2.16E-01 | 0.19 | 0.43 | 5.22E-01 | 0.14 | 0.45 | 2.56E-01 | 0.26 | 0.44 | 3.98E-01 | 0.12 | 0.70 | 2648294 | 483508 | 2803332 | 905555 | 2744839 | 527491 | 2861824 | 1171138 |
| RP_NEG_197_80806a2_461 | RP | NEG | 2.46 | 197.8081 | | | | 1.93E-01 | 0.24 | 0.54 | 5.22E-01 | 0.13 | 0.56 | 1.39E-01 | 0.31 | 0.53 | 4.20E-01 | 0.18 | 0.44 | 9365583 | 4244557 | 10562863 | 5130320 | 10232087 | 5388312 | 10893639 | 4890841 |
| HILIC_NEG_257_84482a7_667 | HILIC | NEG | 7.667 | 257.8448 | | | | 2.27E-01 | 0.22 | 0.75 | 5.22E-01 | 0.13 | 0.80 | 1.69E-01 | 0.29 | 0.61 | 4.68E-01 | 0.15 | 0.42 | 791569 | 53781 | 805737 | 66706 | 801436 | 68105 | 810038 | 65683 |
| HILIC_NEG_167_02104a4_723 | HILIC | NEG | 4.723 | 167.0210 | | | | 1.57E-03 | 0.54 | 1.01 | 5.22E-01 | 0.13 | 1.04 | 5.22E-03 | 0.61 | 0.94 | 8.96E-03 | 0.48 | 0.92 | 13169652 | 3506493 | 15783231 | 4997149 | 15461173 | 4150119 | 16105289 | 5746081 |
| RP_POS_453_2933a10_657 | RP | POS | 10.66 | 453.2933 | | | | 4.92E-01 | 0.13 | 0.52 | 5.23E-01 | 0.13 | 1.13 | 3.74E-01 | 0.20 | 0.47 | 7.59E-01 | 0.07 | 0.30 | 1235967 | 502417 | 1308786 | 568097 | 1272227 | 544051 | 1345345 | 594420 |
| HILIC_POS_565_56647a10_709 | HILIC | POS | 10.709 | 565.5665 | | | | 4.71E-01 | -0.13 | 0.39 | 5.23E-01 | -0.14 | 0.66 | 3.17E-01 | -0.20 | 0.41 | 7.77E-01 | -0.06 | 0.26 | 3008509 | 109159 | 2991219 | 139761 | 3000205 | 154694 | 2982232 | 123976 |
| RP_NEG_800_54645a12_766 | RP | NEG | 12.77 | 800.5465 | Unknown | 279.2347(100), 253.2187(40), 305.2496(24), 740.5323(23) | 4 | 2.48E-03 | 0.54 | 0.97 | 5.23E-01 | -0.13 | 0.57 | 1.50E-02 | 0.47 | 0.91 | 5.24E-03 | 0.60 | 1.02 | 10928001 | 5325781 | 14630994 | 7096303 | 15087177 | 7784820 | 14174811 | 6380799 |
| RP_POS_736_43164a5_721 | RP | POS | 5.72 | 736.4316 | | | | 8.07E-07 | 0.59 | 1.28 | 5.23E-01 | -0.14 | 1.14 | 7.98E-04 | 0.52 | 1.38 | 3.33E-04 | 0.66 | 1.35 | 28099 | 53540 | 848153 | 1554652 | 948088 | 1684592 | 748218 | 1423019 |
| RP_POS_448_25909a6_222 | RP | POS | 6.22 | 448.2591 | | | | 3.54E-07 | 0.61 | 1.30 | 5.23E-01 | -0.14 | 1.16 | 5.43E-04 | 0.54 | 1.40 | 2.14E-04 | 0.68 | 1.38 | 10874 | 22962 | 712456 | 1284466 | 795022 | 1386301 | 629891 | 1182217 |
| HILIC_NEG_158_93893a2_946 | HILIC | NEG | 2.946 | 158.9389 | | | | 4.06E-01 | -0.15 | 0.94 | 5.23E-01 | -0.14 | 0.72 | 2.64E-01 | -0.22 | 0.60 | 7.17E-01 | -0.08 | 0.53 | 4871355 | 950923 | 4696157 | 1249787 | 4776482 | 1407668 | 4615833 | 1077549 |
| HILIC_NEG_164_83592a2_962 | HILIC | NEG | 2.962 | 164.8359 | | | | 8.51E-01 | 0.04 | 0.44 | 5.23E-01 | -0.12 | 0.49 | 9.31E-01 | -0.02 | 0.15 | 6.68E-01 | 0.10 | 0.39 | 17467964 | 9450793 | 17820237 | 8235710 | 18349236 | 8364825 | 17291238 | 8154547 |
| RP_POS_284_12802a8_701 | RP | POS | 8.70 | 284.1280 | | | | 3.84E-01 | 0.15 | 0.47 | 5.24E-01 | -0.14 | 0.46 | 6.34E-01 | 0.09 | 0.38 | 3.31E-01 | 0.22 | 0.56 | 638743 | 998691 | 831635 | 1314615 | 916094 | 1569563 | 747175 | 1006783 |
| RP_POS_458_2742a11_862 | RP | POS | 11.86 | 458.2742 | | | | 9.54E-02 | 0.30 | 0.83 | 5.24E-01 | -0.13 | 0.53 | 2.78E-01 | 0.23 | 0.64 | 7.24E-02 | 0.36 | 0.69 | 4592043 | 2063303 | 5362449 | 2729928 | 5537730 | 2612316 | 5187168 | 2858357 |
| RP_POS_308_29486a10_059 | RP | POS | 10.06 | 308.2949 | | | | 4.47E-01 | -0.19 | 0.80 | 5.24E-01 | -0.11 | 0.49 | 3.50E-01 | -0.25 | 0.61 | 6.15E-01 | -0.13 | 0.44 | 9481289 | 16460729 | 7095853 | 10937401 | 7797754 | 11408807 | 6393951 | 10513254 |
| RP_POS_415_22379a5_991 | RP | POS | 5.99 | 415.2238 | | | | 6.64E-07 | 0.59 | 1.30 | 5.24E-01 | -0.14 | 1.16 | 7.61E-04 | 0.52 | 1.40 | 2.74E-04 | 0.67 | 1.38 | 28515 | 61925 | 478078 | 840845 | 532031 | 905605 | 424124 | 776150 |
| RP_NEG_313_23868a10_16 | RP | NEG | 10.16 | 313.2387 | FA 18:1;2O | 313.2389(100), 251.2392(60), 295.2254(37) | 3 | 3.01E-01 | -0.23 | 0.51 | 5.24E-01 | 0.12 | 0.38 | 4.73E-01 | -0.17 | 0.34 | 2.37E-01 | -0.29 | 0.65 | 1941430 | 446964 | 1849874 | 374439 | 1825854 | 395688 | 1873895 | 354293 |
| RP_POS_142_94946a15_381 | RP | POS | 15.38 | 142.9495 | | | | 7.91E-01 | -0.04 | 0.27 | 5.24E-01 | 0.14 | 0.44 | 7.05E-01 | 0.04 | 0.27 | 6.40E-01 | -0.11 | 0.43 | 15036932 | 365362 | 15006070 | 962980 | 14944207 | 1319719 | 15067933 | 352304 |
| RP_POS_421_2594a5_992 | RP | POS | 5.99 | 421.2594 | | | | 4.16E-07 | 0.60 | 1.31 | 5.25E-01 | -0.14 | 1.16 | 6.23E-04 | 0.53 | 1.40 | 2.18E-04 | 0.67 | 1.39 | 44589 | 101340 | 1337015 | 2378705 | 1489267 | 2556375 | 1184763 | 2202179 |
| RP_POS_381_21259a10_705 | RP | POS | 10.71 | 381.2126 | | | | 4.42E-03 | 0.53 | 1.39 | 5.25E-01 | 0.13 | 1.22 | 1.07E-02 | 0.59 | 1.36 | 1.44E-02 | 0.46 | 1.27 | 899741 | 158494 | 1000701 | 196336 | 988129 | 149909 | 1013274 | 234707 |
| RP_POS_171_11656a7_342 | RP | POS | 7.34 | 171.1166 | | | | 6.69E-03 | -0.53 | 0.87 | 5.25E-01 | 0.13 | 0.57 | 3.15E-02 | -0.47 | 0.84 | 6.92E-03 | -0.60 | 1.00 | 1023397 | 82356 | 975194 | 90098 | 969430 | 90669 | 980958 | 90068 |
| RP_POS_663_46381a10_658 | RP | POS | 10.66 | 663.4638 | | | | 6.71E-01 | 0.09 | 0.24 | 5.25E-01 | 0.12 | 0.38 | 5.19E-01 | 0.16 | 0.48 | 8.97E-01 | 0.03 | 0.26 | 12024864 | 937961 | 12104025 | 825661 | 12051209 | 823089 | 12156841 | 833172 |
| RP_POS_371_19769a5_722 | RP | POS | 5.72 | 371.1977 | | | | 5.95E-07 | 0.60 | 1.29 | 5.25E-01 | -0.14 | 1.15 | 7.23E-04 | 0.52 | 1.39 | 2.66E-04 | 0.67 | 1.37 | 21556 | 56243 | 588570 | 1058574 | 656277 | 1140439 | 520862 | 976766 |
| RP_POS_187_97154a15_578 | RP | POS | 15.58 | 187.9715 | | | | 2.90E-01 | -0.18 | 0.58 | 5.25E-01 | 0.14 | 0.81 | 5.79E-01 | -0.11 | 0.69 | 2.21E-01 | -0.25 | 0.50 | 1962458 | 66434 | 1946222 | 97107 | 1940012 | 98394 | 1952432 | 96393 |
| RP_POS_343_28375a10_16 | RP | POS | 10.16 | 343.2838 | | | | 2.91E-01 | -0.23 | 0.47 | 5.25E-01 | 0.12 | 0.39 | 4.81E-01 | -0.17 | 0.39 | 2.21E-01 | -0.29 | 0.67 | 657937 | 169266 | 622107 | 151861 | 612400 | 152201 | 631814 | 152437 |
| RP_POS_216_15952a6_464 | RP | POS | 6.46 | 216.1595 | | | | 6.67E-01 | -0.11 | 0.65 | 5.26E-01 | -0.11 | 0.42 | 5.56E-01 | -0.17 | 0.61 | 8.33E-01 | -0.05 | 0.46 | 674484 | 838664 | 605958 | 542195 | 640616 | 421591 | 571299 | 643242 |
| RP_POS_648_48578a11_246 | RP | POS | 11.25 | 648.4858 | | | | 5.70E-10 | 0.90 | 1.40 | 5.26E-01 | 0.13 | 0.84 | 5.48E-06 | 0.96 | 1.44 | 3.34E-08 | 0.83 | 1.72 | 261561 | 155822 | 558357 | 340756 | 536575 | 251325 | 580139 | 412875 |
| HILIC_POS_353_00952a7_45 | HILIC | POS | 7.45 | 353.0095 | | | | 9.05E-01 | -0.02 | 0.99 | 5.26E-01 | -0.14 | 0.55 | 6.11E-01 | -0.09 | 0.83 | 8.20E-01 | 0.05 | 0.94 | 592478 | 46858 | 591156 | 73062 | 595823 | 85454 | 586488 | 58636 |
| RP_POS_835_66479a11_612 | RP | POS | 11.61 | 835.6648 | | | | 4.29E-01 | -0.17 | 0.37 | 5.26E-01 | 0.13 | 0.41 | 6.33E-01 | -0.11 | 0.44 | 3.48E-01 | -0.23 | 0.49 | 602026 | 627679 | 502178 | 579080 | 465195 | 657568 | 539162 | 492263 |
| HILIC_POS_372_31027a0_627 | HILIC | POS | 0.627 | 372.3103 | | | | 1.02E-03 | 0.57 | 1.02 | 5.26E-01 | -0.13 | 0.85 | 7.28E-03 | 0.50 | 1.08 | 3.33E-03 | 0.63 | 0.98 | 1253724 | 642623 | 1751722 | 910953 | 1809897 | 1021762 | 1693547 | 791013 |
| RP_NEG_463_20804a10_905 | RP | NEG | 10.91 | 463.2080 | | | | 2.05E-03 | 0.47 | 1.57 | 5.26E-01 | 0.14 | 0.86 | 7.40E-03 | 0.54 | 1.32 | 2.02E-02 | 0.41 | 1.36 | 856911 | 299233 | 1097723 | 545912 | 1062890 | 487781 | 1132556 | 601428 |
| HILIC_NEG_92_92761a3_016 | HILIC | NEG | 3.016 | 92.9276 | | | | 7.13E-01 | 0.07 | 0.69 | 5.26E-01 | -0.13 | 0.84 | 9.79E-01 | 0.01 | 0.21 | 5.37E-01 | 0.14 | 0.43 | 1081133 | 313740 | 1105390 | 349094 | 1127661 | 356691 | 1083120 | 343482 |
| RP_POS_766_75732a0_499 | RP | POS | 0.50 | 766.7573 | | | | 2.83E-02 | -0.31 | 1.11 | 5.27E-01 | -0.14 | 1.26 | 2.74E-02 | -0.38 | 1.13 | 2.02E-01 | -0.24 | 1.15 | 2287596 | 580913 | 1919536 | 1300641 | 2002446 | 1385958 | 1836626 | 1217763 |
| RP_POS_776_79108a0_498 | RP | POS | 0.50 | 776.7911 | | | | 7.89E-10 | -0.75 | 1.42 | 5.27E-01 | 0.14 | 1.19 | 3.62E-05 | -0.68 | 1.49 | 5.10E-06 | -0.82 | 1.54 | 2468516 | 130401 | 1782353 | 991850 | 1719187 | 1027755 | 1845520 | 960827 |
| RP_POS_349_18442a5_569 | RP | POS | 5.57 | 349.1844 | | | | 4.92E-07 | 0.60 | 1.30 | 5.27E-01 | -0.14 | 1.16 | 8.02E-04 | 0.53 | 1.39 | 2.09E-04 | 0.67 | 1.39 | 32407 | 21902 | 529538 | 922830 | 588305 | 980757 | 470772 | 866993 |

**Supplementary table 1: Metabolomics results** (in asceding order according to p-value from progressive ALD vs non-progressive ALD comparison, with molecular features with ID_level of 1-2 first)

| | | | | | | | | all_ALD_vs_control | | | Prog_ALD_vs_non-prog_ALD | | | Prog_ALD_vs_control_ALD | | | Non-prog_ALD_vs_control | | | Healthy_control | | all_ALD | | Non-prog_ALD | | Progressive_ALD | |
|---|---|---|---|---|---|---|---|---|---|---|---|---|---|---|---|---|---|---|---|---|---|---|---|---|---|---|---|
| | | | | | | | | t-test | Cohen's | PLS-DA | t-test | Cohen's | PLS-DA | t-test | Cohen's | PLS-DA | t-test | Cohen's | PLS-DA | | | | | | | | |
| Feature_ID | RP_HILIC | POS_NEG | Average.Rt | Average.Mz | Metabolite.name | curated_MSMS | ID_level | p | d | VIP | p | d | VIP | p | d | VIP | p | d | VIP | Mean | SD | Mean | SD | Mean | SD | Mean | SD |
| RP_NEG_167_02026a0_709 | RP | NEG | 0.71 | 167.0203 | | | | 3.70E-02 | 0.37 | 0.84 | 5.27E-01 | -0.13 | 0.45 | 1.52E-01 | 0.30 | 0.85 | 3.07E-02 | 0.43 | 0.80 | 117860014 | 20001848 | 127334562 | 27120022 | 129061538 | 25879092 | 125607586 | 28463788 |
| RP_NEG_369_17401a11_379 | RP | NEG | 11.38 | 369.1740 | | | | 2.27E-01 | -0.23 | 0.89 | 5.27E-01 | -0.13 | 1.02 | 1.55E-01 | -0.30 | 0.81 | 4.65E-01 | -0.16 | 0.91 | 1076763 | 291126 | 1001737 | 335702 | 1023112 | 366168 | 980362 | 304435 |
| RP_POS_182_02362a10_568 | RP | POS | 10.57 | 182.0236 | | | | 9.84E-01 | 0.00 | 0.21 | 5.27E-01 | -0.13 | 0.43 | 7.81E-01 | -0.07 | 0.37 | 7.95E-01 | 0.06 | 0.81 | 1911944 | 616246 | 1909443 | 580796 | 1946410 | 524178 | 1872475 | 635595 |
| RP_POS_741_38696a5_722 | RP | POS | 5.72 | 741.3870 | | | | 3.79E-06 | 0.55 | 1.27 | 5.27E-01 | -0.14 | 1.14 | 1.82E-03 | 0.48 | 1.37 | 6.48E-04 | 0.62 | 1.34 | 60949 | 137220 | 748624 | 1387840 | 836948 | 1498722 | 660299 | 1276486 |
| RP_POS_377_2334a5_721 | RP | POS | 5.72 | 377.2334 | | | | 5.80E-07 | 0.60 | 1.30 | 5.27E-01 | -0.14 | 1.15 | 7.39E-04 | 0.52 | 1.39 | 2.58E-04 | 0.67 | 1.38 | 43445 | 108847 | 1340555 | 2421111 | 1494617 | 2601827 | 1186493 | 2241706 |
| HILIC_POS_186_18518a0_973 | HILIC | POS | 0.973 | 186.1852 | | | | 2.32E-01 | -0.20 | 0.53 | 5.27E-01 | 0.14 | 0.84 | 5.51E-01 | -0.13 | 0.40 | 1.31E-01 | -0.27 | 0.57 | 481222 | 255446 | 409672 | 386694 | 385060 | 310478 | 434284 | 452198 |
| RP_POS_295_88986a0_254 | RP | POS | 0.25 | 295.8899 | | | | 9.12E-01 | -0.02 | 0.30 | 5.28E-01 | -0.13 | 0.63 | 7.05E-01 | -0.09 | 0.26 | 8.52E-01 | 0.04 | 0.14 | 3475569 | 393688 | 3466648 | 402929 | 3492262 | 395880 | 3441034 | 412258 |
| RP_POS_195_12234a2_646 | RP | POS | 2.65 | 195.1223 | | | | 1.21E-01 | 0.23 | 0.93 | 5.28E-01 | -0.14 | 0.86 | 3.36E-01 | 0.17 | 0.99 | 1.34E-01 | 0.30 | 0.91 | 2199735 | 278534 | 2312228 | 525188 | 2345617 | 585282 | 2278838 | 460916 |
| RP_NEG_544_26617a10_344 | RP | NEG | 10.34 | 544.2662 | | | | 7.14E-06 | 0.75 | 1.26 | 5.28E-01 | 0.13 | 0.58 | 9.11E-05 | 0.81 | 1.37 | 2.25E-04 | 0.68 | 1.23 | 1214318 | 288346 | 1539667 | 447561 | 1511233 | 405985 | 1568101 | 488095 |
| RP_POS_656_79065a0_504 | RP | POS | 0.50 | 656.7907 | | | | 7.41E-04 | -0.53 | 1.15 | 5.28E-01 | -0.14 | 1.22 | 1.05E-03 | -0.60 | 1.25 | 2.00E-02 | -0.46 | 1.14 | 769168 | 207174 | 590597 | 357047 | 613276 | 385282 | 567917 | 328747 |
| RP_POS_906_82343a0_489 | RP | POS | 0.49 | 906.8234 | | | | 6.51E-08 | 0.65 | 1.35 | 5.28E-01 | -0.14 | 1.20 | 2.45E-04 | 0.58 | 1.41 | 8.33E-05 | 0.72 | 1.47 | 1685175 | 341025 | 5835279 | 7082695 | 6285054 | 7571012 | 5385504 | 6604530 |
| RP_POS_428_81912a0_501 | RP | POS | 0.50 | 428.8191 | | | | 2.73E-01 | -0.18 | 0.99 | 5.29E-01 | -0.14 | 1.10 | 1.96E-01 | -0.25 | 0.90 | 5.75E-01 | -0.11 | 1.04 | 436335 | 182031 | 389355 | 278045 | 406996 | 290409 | 371714 | 266877 |
| RP_NEG_453_78607a15_515 | RP | NEG | 15.52 | 453.7861 | | | | 8.75E-01 | -0.04 | 0.40 | 5.29E-01 | 0.12 | 0.53 | 9.19E-01 | 0.03 | 0.23 | 6.87E-01 | -0.10 | 0.55 | 3634703 | 116610 | 3631082 | 97929 | 3624869 | 90924 | 3637295 | 105025 |
| RP_NEG_376_98389a9_269 | RP | NEG | 9.27 | 376.9839 | | | | 2.97E-01 | 0.21 | 0.62 | 5.29E-01 | 0.13 | 0.70 | 2.52E-01 | 0.27 | 0.59 | 4.96E-01 | 0.14 | 0.63 | 2854097 | 1219467 | 3119828 | 1312782 | 3036545 | 1105472 | 3203112 | 1498579 |
| RP_POS_411_18686a11_253 | RP | POS | 11.25 | 411.1869 | | | | 2.65E-06 | 0.81 | 1.66 | 5.29E-01 | 0.13 | 0.83 | 3.77E-05 | 0.87 | 1.61 | 6.38E-05 | 0.75 | 1.69 | 1070627 | 397748 | 1530579 | 569696 | 1494444 | 504241 | 1566713 | 631511 |
| RP_POS_294_91995a0_256 | RP | POS | 0.26 | 294.9200 | | | | 7.88E-01 | 0.06 | 0.34 | 5.29E-01 | -0.12 | 0.46 | 9.98E-01 | 0.00 | 0.34 | 6.21E-01 | 0.12 | 0.61 | 722014 | 202603 | 732696 | 166581 | 743257 | 164589 | 722136 | 169550 |
| HILIC_POS_650_39532a1_037 | HILIC | POS | 1.037 | 650.3953 | | | | 1.18E-05 | 0.55 | 1.25 | 5.29E-01 | -0.14 | 0.82 | 2.89E-03 | 0.48 | 1.41 | 7.54E-04 | 0.62 | 1.39 | 373359 | 1231835 | 2969876 | 5252597 | 3302868 | 5604696 | 2636884 | 4909552 |
| HILIC_POS_174_12364a1_87 | HILIC | POS | 1.87 | 174.1236 | | | | 1.31E-01 | 0.19 | 0.94 | 5.29E-01 | 0.14 | 0.55 | 1.33E-01 | 0.27 | 0.75 | 4.66E-01 | 0.12 | 0.51 | 466149 | 256281 | 612000 | 846728 | 558350 | 828059 | 665650 | 870041 |
| RP_POS_261_09985a15_341 | RP | POS | 15.34 | 261.0999 | | | | 4.17E-02 | -0.34 | 0.65 | 5.29E-01 | -0.13 | 0.55 | 5.00E-02 | -0.41 | 0.69 | 1.45E-01 | -0.28 | 0.68 | 7042701 | 845607 | 6639728 | 1245232 | 6718622 | 1143327 | 6560834 | 1346459 |
| HILIC_POS_182_07881a4_596 | HILIC | POS | 4.596 | 182.0788 | | | | 4.42E-01 | 0.14 | 0.34 | 5.29E-01 | -0.13 | 0.83 | 3.17E-01 | 0.20 | 0.41 | 7.40E-01 | 0.07 | 0.31 | 494473 | 1168544 | 693183 | 1530906 | 596227 | 1596872 | 790138 | 1471731 |
| RP_POS_200_1644a10_779 | RP | POS | 10.78 | 200.1644 | | | | 3.09E-02 | 0.39 | 0.79 | 5.29E-01 | -0.13 | 0.40 | 1.61E-01 | 0.33 | 0.61 | 1.23E-02 | 0.46 | 0.95 | 320535 | 169545 | 401727 | 213485 | 415257 | 149892 | 388197 | 263135 |
| RP_POS_438_3783a10_656 | RP | POS | 10.66 | 438.3783 | | | | 1.40E-01 | -0.29 | 0.51 | 5.29E-01 | -0.13 | 0.46 | 2.84E-01 | -0.23 | 0.59 | 1.23E-01 | -0.36 | 0.61 | 5904709 | 475258 | 5757903 | 505783 | 5725876 | 549888 | 5789930 | 460850 |
| HILIC_NEG_136_89172a2_172 | HILIC | NEG | 2.172 | 136.8917 | | | | 4.09E-01 | 0.15 | 0.68 | 5.30E-01 | -0.13 | 0.78 | 6.85E-01 | 0.09 | 0.19 | 2.96E-01 | 0.22 | 0.44 | 12829942 | 2684322 | 13306313 | 3212688 | 13509400 | 3093948 | 13103227 | 3346145 |
| RP_NEG_341_27017a11_868 | RP | NEG | 11.87 | 341.2702 | | | | 1.57E-01 | -0.27 | 0.67 | 5.30E-01 | 0.13 | 0.92 | 3.35E-01 | -0.21 | 0.53 | 1.26E-01 | -0.34 | 0.72 | 2711646 | 224983 | 2643918 | 254330 | 2627846 | 260494 | 2659990 | 249607 |
| RP_POS_402_25949a10_449 | RP | POS | 10.45 | 402.2595 | | | | 8.62E-01 | -0.03 | 0.65 | 5.30E-01 | -0.14 | 0.49 | 5.66E-01 | -0.10 | 0.32 | 8.72E-01 | 0.04 | 0.69 | 1270884 | 166049 | 1264368 | 231739 | 1279023 | 289539 | 1249713 | 155662 |
| RP_POS_207_01419a0_523 | RP | POS | 0.52 | 207.0142 | | | | 6.73E-07 | -0.71 | 1.29 | 5.31E-01 | -0.13 | 1.17 | 3.61E-05 | -0.78 | 1.39 | 2.80E-04 | -0.64 | 1.37 | 1891396 | 324661 | 1401376 | 735080 | 1447786 | 714751 | 1354966 | 759242 |
| RP_POS_692_40546a5_567 | RP | POS | 5.57 | 692.4055 | | | | 1.43E-06 | 0.57 | 1.27 | 5.31E-01 | -0.14 | 1.13 | 1.13E-03 | 0.50 | 1.37 | 4.10E-04 | 0.65 | 1.35 | 52062 | 160154 | 1252645 | 2326326 | 1399435 | 2506445 | 1105856 | 2146576 |
| RP_POS_277_17917a8_349 | RP | POS | 8.35 | 277.1792 | | | | 6.11E-01 | 0.09 | 0.65 | 5.31E-01 | 0.13 | 0.87 | 4.46E-01 | 0.16 | 0.69 | 9.02E-01 | 0.03 | 0.57 | 1136641 | 121161 | 1150103 | 153342 | 1140431 | 156779 | 1159776 | 150788 |
| RP_POS_537_39355a14_268 | RP | POS | 14.27 | 537.3936 | | | | 2.43E-01 | 0.24 | 0.46 | 5.31E-01 | 0.13 | 0.39 | 2.05E-01 | 0.30 | 0.57 | 4.23E-01 | 0.17 | 0.48 | 1153377848 | 128353274 | ######### | 132381118 | 1176040838 | 116871264 | 1192737596 | 146989934 |
| RP_NEG_372_90237a15_34 | RP | NEG | 15.34 | 372.9024 | | | | 5.14E-01 | -0.11 | 0.77 | 5.31E-01 | -0.13 | 0.50 | 3.67E-01 | -0.18 | 0.83 | 8.19E-01 | -0.05 | 0.30 | 2764766 | 77678 | 2753439 | 104537 | 2760029 | 109211 | 2746848 | 100316 |
| RP_POS_962_73065a0_496 | RP | POS | 0.50 | 962.7307 | | | | 1.10E-01 | -0.26 | 1.02 | 5.31E-01 | -0.14 | 1.13 | 9.72E-02 | -0.33 | 0.99 | 3.23E-01 | -0.19 | 1.04 | 673028 | 126357 | 624439 | 201812 | 637161 | 200427 | 611718 | 204415 |
| HILIC_POS_182_10345a1_386 | HILIC | POS | 1.386 | 182.1035 | | | | 3.58E-18 | 1.11 | 1.87 | 5.31E-01 | -0.13 | 0.61 | 8.33E-09 | 1.05 | 1.81 | 1.64E-11 | 1.17 | 2.31 | 1813495 | 1093728 | 6843739 | 4531189 | 7129201 | 4301974 | 6558187 | 4775875 |
| RP_POS_784_57294a13_72 | RP | POS | 13.72 | 784.5729 | | | | 9.31E-07 | 0.87 | 1.40 | 5.32E-01 | -0.12 | 0.60 | 8.25E-05 | 0.81 | 1.40 | 2.94E-06 | 0.93 | 1.58 | 13818388 | 3990833 | 18599603 | 5412855 | 18940538 | 5162573 | 18258667 | 5683885 |
| RP_POS_560_87079a0_471 | RP | POS | 0.47 | 560.8708 | | | | 2.63E-09 | -0.76 | 1.39 | 5.32E-01 | 0.14 | 1.16 | 7.84E-05 | -0.69 | 1.43 | 2.72E-06 | -0.82 | 1.54 | 802060 | 88333 | 555017 | 352299 | 532832 | 349338 | 577201 | 357380 |
| HILIC_POS_628_38232a1_029 | HILIC | POS | 1.029 | 628.3823 | | | | 6.20E-06 | 0.55 | 1.27 | 5.32E-01 | -0.14 | 0.83 | 2.09E-03 | 0.48 | 1.36 | 7.01E-04 | 0.62 | 1.40 | 362289 | 1037887 | 3214985 | 5737159 | 3576249 | 6180481 | 2853722 | 5295001 |
| RP_POS_690_59918a11_258 | RP | POS | 11.26 | 690.5992 | | | | 4.05E-03 | 0.38 | 0.72 | 5.32E-01 | 0.14 | 0.40 | 1.61E-02 | 0.45 | 0.89 | 6.01E-02 | 0.31 | 0.67 | 186522 | 169960 | 361918 | 518706 | 329259 | 482857 | 394577 | 555224 |
| RP_POS_448_22775a6_11 | RP | POS | 6.11 | 448.2278 | | 448.2265(100) | 4 | 3.39E-07 | 0.61 | 1.31 | 5.32E-01 | -0.14 | 1.16 | 5.93E-04 | 0.54 | 1.41 | 1.92E-04 | 0.68 | 1.39 | 39362 | 49251 | 1388932 | 2466225 | 1544032 | 2637152 | 1233833 | 2298912 |
| RP_POS_323_25565a10_653 | RP | POS | 10.65 | 323.2557 | | | | 8.27E-03 | -0.49 | 0.98 | 5.32E-01 | 0.13 | 0.82 | 4.58E-02 | -0.42 | 0.91 | 8.48E-03 | -0.55 | 1.01 | 6373859 | 892273 | 5849896 | 1098397 | 5780823 | 1077722 | 5918968 | 1125323 |
| HILIC_NEG_200_8586a5_573 | HILIC | NEG | 5.573 | 200.8586 | | | | 3.78E-01 | -0.16 | 0.46 | 5.32E-01 | -0.13 | 0.79 | 3.09E-01 | -0.22 | 0.45 | 6.38E-01 | -0.09 | 0.51 | 3098301 | 292756 | 3041409 | 378129 | 3065179 | 334217 | 3017638 | 419555 |
| RP_NEG_277_89676a15_584 | RP | NEG | 15.58 | 277.8968 | | | | 6.56E-01 | 0.09 | 0.68 | 5.33E-01 | -0.12 | 0.39 | 8.90E-01 | 0.03 | 0.42 | 5.20E-01 | 0.16 | 0.36 | 1106688 | 45111 | 1110751 | 43439 | 1113481 | 48293 | 1108021 | 38276 |
| RP_POS_390_28519a10_242 | RP | POS | 10.24 | 390.2852 | | | | 2.22E-06 | 0.56 | 1.09 | 5.33E-01 | -0.14 | 0.96 | 1.86E-03 | 0.49 | 1.14 | 4.19E-04 | 0.63 | 1.21 | 22414 | 20299 | 309190 | 569623 | 344976 | 602110 | 273403 | 538900 |
| HILIC_POS_315_08008a7_811 | HILIC | POS | 7.811 | 315.0801 | | | | 2.70E-01 | -0.19 | 0.57 | 5.33E-01 | -0.13 | 0.86 | 2.15E-01 | -0.26 | 0.59 | 5.27E-01 | -0.13 | 0.50 | 1660724 | 743522 | 1476688 | 1005814 | 1539864 | 970128 | 1413513 | 1046267 |
| RP_POS_443_27267a6_111 | RP | POS | 6.11 | 443.2727 | | | | 2.38E-07 | 0.62 | 1.31 | 5.33E-01 | -0.14 | 1.15 | 4.28E-04 | 0.55 | 1.41 | 1.81E-04 | 0.69 | 1.39 | 26155 | 30967 | 1083101 | 1903297 | 1202638 | 2052876 | 963565 | 1753725 |
| HILIC_POS_145_13342a1_695 | HILIC | POS | 1.695 | 145.1334 | | | | 9.56E-02 | -0.35 | 1.09 | 5.33E-01 | 0.12 | 0.79 | 2.14E-01 | -0.29 | 1.11 | 7.65E-02 | -0.41 | 1.00 | 666749 | 398478 | 530275 | 386948 | 505980 | 388195 | 554570 | 388089 |
| RP_POS_467_76337a6_222 | RP | POS | 6.22 | 467.7634 | | | | 2.40E-07 | 0.62 | 1.31 | 5.33E-01 | -0.14 | 1.16 | 4.79E-04 | 0.55 | 1.41 | 1.66E-04 | 0.69 | 1.40 | 35554 | 39604 | 1634019 | 2879898 | 1814811 | 3084751 | 1453228 | 2678524 |
| RP_POS_648_37976a5_399 | RP | POS | 5.40 | 648.3798 | | | | 7.58E-07 | 0.59 | 1.28 | 5.34E-01 | -0.14 | 1.14 | 8.52E-04 | 0.52 | 1.38 | 2.98E-04 | 0.66 | 1.36 | 42955 | 111170 | 1581382 | 2907517 | 1763586 | 3121616 | 1399179 | 2695848 |
| RP_POS_296_25818a9_691 | RP | POS | 9.69 | 296.2582 | | | | 2.07E-01 | -0.38 | 0.90 | 5.34E-01 | -0.08 | 0.41 | 1.76E-01 | -0.43 | 0.82 | 2.65E-01 | -0.34 | 0.69 | 2576098 | 4512832 | 1522688 | 1828244 | 1637268 | 1619020 | 1408109 | 2026116 |
| RP_POS_424_2717a10_458 | RP | POS | 10.46 | 424.2717 | | | | 1.52E-01 | -0.26 | 0.99 | 5.34E-01 | 0.13 | 0.39 | 2.99E-01 | -0.19 | 0.56 | 1.58E-01 | -0.33 | 1.13 | 1239014 | 158673 | 1188878 | 202283 | 1176200 | 240392 | 1201555 | 156701 |
| HILIC_POS_287_09995a1_904 | HILIC | POS | 1.904 | 287.1000 | | | | 3.46E-01 | -0.17 | 0.38 | 5.34E-01 | 0.13 | 0.57 | 6.82E-01 | -0.10 | 0.15 | 1.53E-01 | -0.23 | 0.82 | 1157257 | 895907 | 968111 | 1211687 | 892085 | 643147 | 1044137 | 1594020 |
| RP_NEG_656_88599a0_484 | RP | NEG | 0.48 | 656.8860 | | | | 7.49E-08 | 0.64 | 1.34 | 5.34E-01 | -0.14 | 1.21 | 2.34E-04 | 0.58 | 1.41 | 9.83E-05 | 0.71 | 1.45 | 4840282 | 791438 | 13295353 | 14497392 | 14203149 | 15590954 | 12387556 | 13411874 |
| HILIC_NEG_173_92604a4_284 | HILIC | NEG | 4.284 | 173.9260 | | | | 9.15E-01 | 0.02 | 0.65 | 5.34E-01 | -0.12 | 0.63 | 8.67E-01 | -0.04 | 0.12 | 7.20E-01 | 0.08 | 0.53 | 1223530 | 358802 | 1231255 | 334961 | 1252226 | 341608 | 1210283 | 330293 |
| RP_POS_432_76474a13_872 | RP | POS | 13.87 | 432.7647 | | | | 5.18E-01 | -0.12 | 0.59 | 5.34E-01 | 0.13 | 0.86 | 8.08E-01 | -0.06 | 0.54 | 3.47E-01 | -0.19 | 0.47 | 8220679 | 1722767 | 7982522 | 2042353 | 7854652 | 1680529 | 8110391 | 2360083 |
| RP_NEG_214_14482a6_461 | RP | NEG | 6.46 | 214.1448 | | | | 7.50E-01 | -0.08 | 0.64 | 5.34E-01 | -0.11 | 0.42 | 6.25E-01 | -0.13 | 0.60 | 9.27E-01 | -0.02 | 0.46 | 949037 | 1094815 | 882413 | 753805 | 929618 | 578193 | 835209 | 899547 |
| RP_POS_295_22446a10_282 | RP | POS | 10.28 | 295.2245 | | | | 9.74E-01 | -0.01 | 0.92 | 5.34E-01 | 0.13 | 1.09 | 7.96E-01 | 0.06 | 0.85 | 7.58E-01 | -0.07 | 0.74 | 1611456 | 325334 | 1609300 | 343228 | 1587824 | 354848 | 1630776 | 333403 |
| RP_NEG_900_78381a0_495 | RP | NEG | 0.50 | 900.7838 | | | | 6.92E-08 | -0.69 | 1.34 | 5.35E-01 | 0.14 | 1.16 | 3.16E-04 | -0.62 | 1.43 | 1.98E-05 | -0.76 | 1.45 | 1680511 | 132467 | 1364468 | 499592 | 1333235 | 502608 | 1395701 | 499671 |
| RP_POS_221_98215a0_74 | RP | POS | 0.74 | 221.9822 | | | | 4.02E-07 | 0.77 | 1.26 | 5.35E-01 | -0.13 | 0.66 | 8.76E-05 | 0.71 | 1.36 | 1.72E-05 | 0.84 | 1.38 | 419915 | 314599 | 861748 | 595659 | 898977 | 624768 | 824519 | 568943 |
| HILIC_NEG_134_89481a3_667 | HILIC | NEG | 3.667 | 134.8948 | | | | 1.16E-01 | 0.32 | 0.67 | 5.35E-01 | 0.12 | 0.72 | 8.63E-02 | 0.39 | 0.69 | 2.60E-01 | 0.26 | 0.42 | 15969885 | 7625871 | 18441918 | 7593986 | 17967326 | 7990907 | 18916510 | 7224950 |
| HILIC_POS_224_12819a7_69 | HILIC | POS | 7.69 | 224.1282 | | | | 2.13E-01 | -0.27 | 0.73 | 5.35E-01 | 0.12 | 0.45 | 3.76E-01 | -0.21 | 0.51 | 1.70E-01 | -0.33 | 0.82 | 8129550 | 2819328 | 7420793 | 2599253 | 7258385 | 2690558 | 7583201 | 2521345 |
| HILIC_POS_199_18045a4_245 | HILIC | POS | 4.245 | 199.1805 | | | | 1.22E-02 | 0.48 | 1.10 | 5.35E-01 | 0.13 | 0.80 | 1.07E-02 | 0.54 | 0.96 | 5.74E-02 | 0.42 | 0.71 | 7658052 | 1430630 | 8437894 | 1642123 | 8335295 | 1720384 | 8540492 | 1570657 |
| RP_POS_467_3013a6_393 | RP | POS | 6.39 | 467.3013 | | | | 1.33E-07 | 0.63 | 1.33 | 5.35E-01 | -0.14 | 1.17 | 4.52E-04 | 0.56 | 1.40 | 9.84E-05 | 0.70 | 1.43 | 8540 | 6488 | 257398 | 437666 | 284742 | 460515 | 230055 | 416427 |
| HILIC_NEG_103_03991a0_656 | HILIC | NEG | 0.656 | 103.0399 | | | | 6.81E-02 | 0.29 | 1.01 | 5.35E-01 | 0.13 | 1.01 | 9.71E-02 | 0.36 | 0.56 | 1.79E-01 | 0.23 | 0.69 | 318726 | 127283 | 375316 | 207790 | 362331 | 162461 | 388300 | 245960 |
| RP_POS_548_50305a13_015 | RP | POS | 13.02 | 548.5031 | | | | 1.65E-01 | -0.33 | 0.80 | 5.35E-01 | 0.11 | 0.47 | 2.87E-01 | -0.28 | 0.81 | 1.22E-01 | -0.39 | 0.65 | 1639275 | 467379 | 1513057 | 340493 | 1491794 | 314284 | 1534320 | 366797 |
| RP_POS_442_33694a12_243 | RP | POS | 12.24 | 442.3369 | | | | 1.06E-01 | -0.30 | 0.63 | 5.36E-01 | 0.13 | 0.47 | 2.45E-01 | -0.24 | 0.46 | 9.85E-02 | -0.37 | 0.71 | 3348164 | 211379 | 3274218 | 251618 | 3258522 | 271646 | 3289913 | 231563 |
| RP_POS_277_17874a9_207 | RP | POS | 9.21 | 277.1787 | | | | 5.37E-03 | -0.53 | 0.82 | 5.36E-01 | 0.13 | 0.45 | 2.78E-02 | -0.47 | 0.87 | 6.49E-03 | -0.59 | 0.91 | 1462641 | 94168 | 1405010 | 110028 | 1398148 | 112463 | 1411873 | 108237 |
| RP_NEG_353_30853a11_037 | RP | NEG | 11.04 | 353.3085 | | | | 9.69E-03 | -0.48 | 0.97 | 5.36E-01 | -0.13 | 0.95 | 6.08E-03 | -0.54 | 1.09 | 6.58E-02 | -0.41 | 0.91 | 706956 | 133913 | 629778 | 166614 | 640163 | 189908 | 619393 | 140719 |
| RP_NEG_174_95599a15_344 | RP | NEG | 15.34 | 174.9560 | | | | 5.11E-01 | -0.15 | 0.49 | 5.36E-01 | 0.12 | 0.57 | 7.08E-01 | -0.09 | 0.44 | 4.17E-01 | -0.21 | 0.48 | 40859881 | 2587557 | 40523705 | 2207932 | 40386110 | 2518587 | 40661301 | 1862121 |
| RP_POS_429_22702a9_327 | RP | POS | 9.33 | 429.2270 | | | | 2.66E-05 | 0.54 | 1.15 | 5.36E-01 | 0.14 | 0.50 | 5.27E-04 | 0.61 | 1.19 | 5.86E-03 | 0.48 | 1.21 | 264847 | 223139 | 630586 | 740971 | 584424 | 740887 | 676748 | 745666 |
| HILIC_NEG_131_03481a0_81 | HILIC | NEG | 0.81 | 131.0348 | | | | 6.81E-01 | -0.08 | 0.76 | 5.36E-01 | 0.13 | 0.78 | 9.49E-01 | -0.01 | 0.03 | 5.20E-01 | -0.14 | 0.73 | 656535 | 163789 | 642251 | 189399 | 630454 | 198964 | 654049 | 180569 |
| HILIC_NEG_202_92863a2_946 | HILIC | NEG | 2.946 | 202.9286 | | | | 3.56E-01 | -0.17 | 0.99 | 5.36E-01 | -0.13 | 0.75 | 2.57E-01 | -0.23 | 0.72 | 6.28E-01 | -0.10 | 0.49 | 8907884 | 1785752 | 8549484 | 2205546 | 8686845 | 2312064 | 8412123 | 2108079 |
| RP_POS_281_91193a15_635 | RP | POS | 15.64 | 281.9119 | | | | 4.59E-01 | 0.18 | 0.76 | 5.36E-01 | 0.11 | 1.27 | 3.59E-01 | 0.23 | 0.78 | 6.41E-01 | 0.12 | 0.61 | 5418304 | 1415126 | 5621335 | 1059243 | 5555375 | 1074639 | 5687296 | 1050315 |
| RP_POS_256_22693a11_018 | RP | POS | 11.02 | 256.2269 | | | | 9.48E-03 | 0.41 | 0.84 | 5.36E-01 | -0.13 | 0.40 | 4.91E-02 | 0.34 | 0.71 | 2.19E-02 | 0.48 | 0.82 | 351764 | 471809 | 658132 | 799994 | 707945 | 901011 | 608318 | 690041 |
| RP_POS_130_06549a5_353 | RP | POS | 5.35 | 130.0655 | Fragment of 3-indoleacetic acid | 130.0655(100), 103.0546(1) | | 5.00E-01 | -0.14 | 0.41 | 5.36E-01 | 0.12 | 0.56 | 7.38E-01 | -0.08 | 0.42 | 3.59E-01 | -0.20 | 0.66 | 2948812 | 1284983 | 2773957 | 1204254 | 2698974 | 1031129 | 2848941 | 1362113 |
| HILIC_NEG_477_52896a1_365 | HILIC | NEG | 1.365 | 477.5290 | | | | 5.43E-01 | -0.09 | 0.33 | 5.36E-01 | -0.14 | 0.44 | 4.32E-01 | -0.16 | 0.35 | 8.90E-01 | -0.02 | 0.38 | 195210 | 68128 | 184460 | 128578 | 192465 | 110894 | 176454 | 144823 |
| RP_POS_765_45886a10_893 | RP | POS | 10.89 | 765.4589 | | | | 6.26E-03 | 0.38 | 1.09 | 5.37E-01 | -0.14 | 0.94 | 3.57E-02 | 0.31 | 1.06 | 3.08E-02 | 0.45 | 1.07 | 1231379 | 562438 | 1710348 | 1406174 | 1797887 | 1672757 | 1622808 | 1086852 |
| RP_POS_323_21442a9_889 | RP | POS | 9.89 | 323.2144 | | | | 8.40E-01 | 0.04 | 0.53 | 5.37E-01 | 0.12 | 0.45 | 6.59E-01 | 0.10 | 0.30 | 9.42E-01 | -0.02 | 0.55 | 1653258 | 293894 | 1665109 | 265574 | 1648589 | 269419 | 1681629 | 263353 |
| RP_POS_852_53381a11_254 | RP | POS | 11.25 | 852.5338 | | | | 1.47E-02 | 0.30 | 0.60 | 5.37E-01 | -0.14 | 0.51 | 4.38E-02 | 0.23 | 0.72 | 7.96E-02 | 0.37 | 0.66 | 475013 | 286524 | 834137 | 1358857 | 918658 | 1718370 | 749617 | 873668 |
| RP_NEG_410_1748a11_262 | RP | NEG | 11.26 | 410.1748 | | | | 1.30E-06 | 0.89 | 1.74 | 5.37E-01 | 0.12 | 1.06 | 9.87E-06 | 0.95 | 1.69 | 2.59E-05 | 0.83 | 1.68 | 572448 | 128595 | 721079 | 161069 | 711074 | 147562 | 731083 | 174546 |
| RP_POS_734_8103a0_497 | RP | POS | 0.50 | 734.8103 | | | | 1.31E-08 | -0.68 | 1.37 | 5.37E-01 | 0.14 | 1.19 | 1.42E-04 | -0.62 | 1.43 | 2.81E-05 | -0.75 | 1.49 | 11629346 | 314934 | 8596331 | 4871218 | 8293848 | 5096077 | 8898814 | 4667446 |
| RP_POS_166_98494a10_441 | RP | POS | 10.44 | 166.9849 | | | | 1.20E-01 | -0.31 | 0.57 | 5.38E-01 | -0.12 | 1.04 | 7.77E-02 | -0.37 | 0.83 | 2.90E-01 | -0.25 | 0.41 | 12422318 | 4958054 | 10811350 | 5190931 | 11133585 | 5885023 | 10489115 | 4426740 |
| RP_POS_198_14888a6_462 | RP | POS | 6.46 | 198.1489 | | | | 6.90E-01 | -0.10 | 0.63 | 5.38E-01 | -0.11 | 0.40 | 5.76E-01 | -0.16 | 0.60 | 8.57E-01 | -0.05 | 0.43 | 1239230 | 1099589 | 1155697 | 728923 | 1200955 | 582422 | 1110439 | 854461 |
| HILIC_POS_171_18561a5_784 | HILIC | POS | 5.784 | 171.1856 | | | | 6.07E-01 | -0.11 | 0.72 | 5.38E-01 | -0.12 | 0.84 | 4.81E-01 | -0.17 | 0.47 | 8.27E-01 | -0.05 | 0.22 | 74955656 | 16139278 | 73297213 | 14609877 | 74202851 | 13512695 | 72391575 | 15715798 |
| RP_POS_551_2757a10_311 | RP | POS | 10.31 | 551.2757 | | | | 1.25E-02 | 0.44 | 1.19 | 5.38E-01 | 0.13 | 0.53 | 1.47E-02 | 0.51 | 1.08 | 6.35E-02 | 0.38 | 1.00 | 269862 | 131419 | 344643 | 176334 | 333714 | 174755 | 355572 | 178995 |
| RP_NEG_316_94928a12_881 | RP | NEG | 12.88 | 316.9493 | | | | 7.86E-01 | -0.05 | 0.36 | 5.38E-01 | 0.13 | 0.42 | 9.70E-01 | 0.01 | 0.29 | 5.89E-01 | -0.12 | 0.33 | 3648149 | 937707 | 3595699 | 980736 | 3534925 | 895233 | 3656473 | 1065052 |
| HILIC_POS_343_29504a0_891 | HILIC | POS | 0.891 | 343.2950 | | | | 6.25E-01 | 0.07 | 0.15 | 5.38E-01 | -0.14 | 0.49 | 5.13E-01 | 0.14 | 0.38 | 9.73E-01 | 0.01 | 0.22 | 182727 | 352219 | 227869 | 677409 | 185865 | 490150 | 269872 | 826609 |
| RP_POS_445_75043a6_111 | RP | POS | 6.11 | 445.7504 | | | | 3.17E-07 | 0.61 | 1.31 | 5.38E-01 | -0.14 | 1.16 | 5.59E-04 | 0.54 | 1.41 | 1.89E-04 | 0.68 | 1.40 | 69159 | 107627 | 2216110 | 3910617 | 2458325 | 4181568 | 1973895 | 3645917 |
| HILIC_POS_202_18036a1_345 | HILIC | POS | 1.345 | 202.1804 | | | | 9.97E-02 | -0.36 | 0.59 | 5.39E-01 | -0.12 | 0.46 | 6.86E-02 | -0.42 | 0.75 | 2.22E-01 | -0.30 | 0.47 | 17118349 | 9713392 | 13897331 | 8546580 | 14426413 | 9526911 | 13368250 | 7499697 |
| RP_POS_426_24576a6_11 | RP | POS | 6.11 | 426.2458 | | | | 3.57E-07 | 0.61 | 1.31 | 5.39E-01 | -0.14 | 1.15 | 5.50E-04 | 0.54 | 1.40 | 2.13E-04 | 0.68 | 1.38 | 21051 | 56894 | 1008470 | 1807095 | 1120314 | 1942092 | 896626 | 1673493 |
| RP_POS_285_00549a4_771 | RP | POS | 4.77 | 285.0055 | | | | 5.98E-04 | -0.70 | 1.23 | 5.39E-01 | -0.12 | 0.53 | 8.44E-04 | -0.76 | 1.29 | 3.58E-03 | -0.64 | 1.34 | 1626548 | 563591 | 1206370 | 576860 | 1242064 | 559680 | 1170676 | 597062 |

**Supplementary table 1: Metabolomics results** (in asceding order according to p-value from progressive ALD vs  non-progressive ALD comparison, with molecular features with ID_level of 1-2 first)

| | | | | | | | | all_ALD_vs_control | | | Prog_ALD_vs_non-prog_ALD | | | Prog_ALD_vs_control_ALD | | | Non-prog_ALD_vs_control | | | Healthy_control | | all_ALD | | Non-prog_ALD | | Progressive_ALD | |
|---|---|---|---|---|---|---|---|---|---|---|---|---|---|---|---|---|---|---|---|---|---|---|---|---|---|---|---|
| | | | | | | | | t-test | Cohen's | PLS-DA | t-test | Cohen's | PLS-DA | t-test | Cohen's | PLS-DA | t-test | Cohen's | PLS-DA | | | | | | | | |
| Feature_ID | RP_HILIC | POS_NEG | Average.Rt | Average.Mz | Metabolite.name | curated_MSMS | ID_level | p | d | VIP | p | d | VIP | p | d | VIP | p | d | VIP | Mean | SD | Mean | SD | Mean | SD | Mean | SD |
| RP_POS_284_67712a10_505 | RP | POS | 10.51 | 284.6771 | | | | 1.45E-01 | -0.33 | 0.87 | 5.39E-01 | 0.12 | 0.81 | 2.77E-01 | -0.27 | 0.61 | 1.05E-01 | -0.39 | 0.90 | 1554806 | 677320 | 1359456 | 551964 | 1325301 | 500287 | 1393611 | 602394 |
| RP_POS_218_91927a0_515 | RP | POS | 0.52 | 218.9193 | | | | 4.87E-06 | 0.56 | 1.27 | 5.39E-01 | -0.14 | 1.15 | 1.84E-03 | 0.49 | 1.29 | 6.11E-04 | 0.63 | 1.40 | 508953 | 128256 | 861417 | 699501 | 904688 | 751339 | 818147 | 648272 |
| RP_POS_159_02777a0_709 | RP | POS | 0.71 | 159.0278 | | | | 1.40E-14 | 0.95 | 1.56 | 5.39E-01 | 0.13 | 0.90 | 3.54E-07 | 1.01 | 1.68 | 2.22E-09 | 0.88 | 1.78 | 56392 | 49287 | 888420 | 919639 | 831560 | 749749 | 945279 | 1067713 |
| HILIC_NEG_259_8161a2_946 | HILIC | NEG | 2.946 | 259.8161 | | | | 2.17E-06 | 0.59 | 1.30 | 5.39E-01 | -0.14 | 0.82 | 1.43E-03 | 0.53 | 1.42 | 2.38E-04 | 0.66 | 1.45 | 1241931 | 80231 | 1423339 | 336377 | 1444123 | 350367 | 1402554 | 323990 |
| RP_POS_617_51111a11_379 | RP | POS | 11.38 | 617.5111 | | | | 2.67E-01 | -0.36 | 0.79 | 5.39E-01 | -0.07 | 0.38 | 2.33E-01 | -0.40 | 0.63 | 3.19E-01 | -0.33 | 0.74 | 2633919 | 5453216 | 1531374 | 1612531 | 1630992 | 1652442 | 1431755 | 1582011 |
| HILIC_POS_199_18042a4_123 | HILIC | POS | 4.123 | 199.1804 | | | | 7.90E-03 | -0.52 | 1.23 | 5.39E-01 | -0.12 | 1.04 | 8.11E-03 | -0.58 | 1.06 | 3.65E-02 | -0.46 | 1.06 | 6667328 | 891981 | 6154833 | 989941 | 6215989 | 999307 | 6093678 | 986772 |
| RP_POS_617_52216a9_616 | RP | POS | 9.62 | 617.5222 | | | | 3.32E-01 | -0.27 | 0.88 | 5.40E-01 | -0.09 | 0.41 | 2.70E-01 | -0.32 | 0.69 | 4.41E-01 | -0.23 | 0.59 | 1647425 | 4520409 | 831319 | 2272552 | 971677 | 2414019 | 690961 | 2136981 |
| RP_POS_703_57404a12_661 | RP | POS | 12.66 | 703.5740 | | | | 1.20E-06 | -1.17 | 1.97 | 5.40E-01 | -0.10 | 0.56 | 8.76E-07 | -1.22 | 1.97 | 7.16E-06 | -1.12 | 1.75 | 1607127 | 608513 | 947797 | 445683 | 975302 | 484079 | 920291 | 406748 |
| RP_POS_390_73294a5_862 | RP | POS | 5.86 | 390.7329 | | | | 4.27E-07 | 0.60 | 1.30 | 5.40E-01 | -0.14 | 1.15 | 6.13E-04 | 0.53 | 1.40 | 2.32E-04 | 0.67 | 1.38 | 16273 | 25182 | 585821 | 1050814 | 650629 | 1128419 | 521013 | 974193 |
| HILIC_POS_635_41479a1_24 | HILIC | POS | 1.24 | 635.4148 | | | | 1.90E-04 | 0.71 | 1.17 | 5.40E-01 | -0.12 | 0.72 | 3.75E-03 | 0.65 | 1.05 | 1.34E-04 | 0.77 | 1.49 | 3638293 | 1619796 | 5001383 | 1884100 | 5117594 | 1594639 | 4885173 | 2145153 |
| HILIC_NEG_142_92892a5_425 | HILIC | NEG | 5.425 | 142.9289 | | | | 8.81E-02 | -0.33 | 0.54 | 5.40E-01 | -0.13 | 0.49 | 5.86E-02 | -0.39 | 0.73 | 2.49E-01 | -0.26 | 0.41 | 3057233 | 539292 | 2859877 | 621546 | 2898190 | 694235 | 2821563 | 543660 |
| HILIC_NEG_315_04199a5_882 | HILIC | NEG | 5.882 | 315.0420 | | | | 9.79E-01 | 0.01 | 0.60 | 5.40E-01 | 0.12 | 0.64 | 7.79E-01 | 0.07 | 0.19 | 8.11E-01 | -0.06 | 0.52 | 4646198 | 1129348 | 4652139 | 1061482 | 4586726 | 1031663 | 4717553 | 1097005 |
| RP_POS_553_36963a14_24 | RP | POS | 14.24 | 553.3696 | | | | 1.88E-01 | -0.24 | 0.68 | 5.41E-01 | -0.13 | 0.62 | 1.62E-01 | -0.31 | 0.82 | 3.85E-01 | -0.18 | 0.59 | 165747176 | 48042245 | 151966956 | 59389763 | 155623246 | 55633551 | 148310665 | 63276346 |
| HILIC_POS_184_16968a0_777 | HILIC | POS | 0.777 | 184.1697 | | | | 9.18E-01 | 0.01 | 0.22 | 5.41E-01 | -0.14 | 0.37 | 5.76E-01 | -0.06 | 0.20 | 7.12E-01 | 0.08 | 0.21 | 1783827 | 243126 | 1792554 | 729104 | 1837531 | 976852 | 1747577 | 340095 |
| RP_POS_335_16562a10_979 | RP | POS | 10.98 | 335.1656 | | | | 5.49E-02 | 0.36 | 1.45 | 5.41E-01 | -0.13 | 1.33 | 1.70E-01 | 0.30 | 1.27 | 4.35E-02 | 0.43 | 1.29 | 2592129 | 648385 | 2861072 | 756920 | 2907658 | 721729 | 2814486 | 795127 |
| RP_POS_424_36359a10_45 | RP | POS | 10.45 | 424.3636 | | | | 3.28E-02 | -0.45 | 0.86 | 5.41E-01 | -0.12 | 1.19 | 3.25E-02 | -0.51 | 0.80 | 8.00E-02 | -0.40 | 0.88 | 3369059 | 278132 | 3246902 | 259992 | 3262892 | 238231 | 3230912 | 281595 |
| RP_POS_506_36023a10_795 | RP | POS | 10.80 | 506.3602 | | | | 1.32E-01 | -0.37 | 0.89 | 5.41E-01 | -0.11 | 1.00 | 1.00E-01 | -0.43 | 0.88 | 2.28E-01 | -0.32 | 0.74 | 1953814 | 890992 | 1693872 | 612359 | 1731523 | 647686 | 1656222 | 578991 |
| RP_POS_336_32626a11_37 | RP | POS | 11.37 | 336.3263 | | | | 8.82E-01 | -0.03 | 0.62 | 5.41E-01 | -0.12 | 0.36 | 6.86E-01 | -0.09 | 0.35 | 8.91E-01 | 0.03 | 0.38 | 8229848 | 5460619 | 8065167 | 5450028 | 8400204 | 5534572 | 7730129 | 5399186 |
| RP_POS_357_34729a9_619 | RP | POS | 9.62 | 357.3473 | | | | 5.14E-01 | -0.17 | 0.75 | 5.42E-01 | -0.10 | 0.44 | 4.13E-01 | -0.22 | 0.61 | 6.71E-01 | -0.12 | 0.45 | 1464877 | 2287346 | 1182685 | 1417001 | 1269747 | 1491679 | 1095624 | 1347659 |
| HILIC_POS_351_01309a7_45 | HILIC | POS | 7.45 | 351.0131 | | | | 4.91E-01 | -0.11 | 1.01 | 5.42E-01 | -0.13 | 0.63 | 3.01E-01 | -0.17 | 0.97 | 8.50E-01 | -0.04 | 0.96 | 4816100 | 320042 | 4761001 | 561729 | 4795515 | 651784 | 4726486 | 458547 |
| HILIC_NEG_129_9364a0_596 | HILIC | NEG | 0.596 | 129.9364 | | | | 3.92E-01 | -0.15 | 0.85 | 5.42E-01 | -0.13 | 0.71 | 2.89E-01 | -0.21 | 0.38 | 6.86E-01 | -0.08 | 0.65 | 759871 | 133407 | 734122 | 183900 | 745415 | 188761 | 722829 | 180103 |
| HILIC_POS_118_0865a4_057 | HILIC | POS | 4.057 | 118.0865 | | | | 3.03E-01 | 0.14 | 0.53 | 5.42E-01 | 0.14 | 0.97 | 3.23E-01 | 0.21 | 0.32 | 5.76E-01 | 0.08 | 0.19 | 9188401 | 3603219 | 10285605 | 8468114 | 9765040 | 5701543 | 10806170 | 10574532 |
| RP_POS_238_14136a6_464 | RP | POS | 6.46 | 238.1414 | | 238.1423(100) | 4 | 6.22E-01 | -0.13 | 0.65 | 5.42E-01 | -0.10 | 0.39 | 5.24E-01 | -0.18 | 0.61 | 7.74E-01 | -0.08 | 0.46 | 626108 | 794916 | 552237 | 490505 | 582346 | 393897 | 522127 | 573668 |
| RP_POS_485_11307a10_133 | RP | POS | 10.13 | 485.1131 | | | | 8.58E-01 | 0.04 | 0.59 | 5.43E-01 | -0.12 | 0.54 | 9.42E-01 | -0.02 | 0.53 | 6.74E-01 | 0.10 | 0.49 | 1514348 | 288230 | 1524470 | 241695 | 1539284 | 210734 | 1509655 | 270496 |
| RP_POS_834_58771a13_869 | RP | POS | 13.87 | 834.5877 | | | | 2.31E-01 | -0.26 | 0.65 | 5.43E-01 | 0.12 | 0.83 | 4.18E-01 | -0.21 | 0.60 | 1.55E-01 | -0.32 | 0.59 | 9947147 | 2776863 | 9283961 | 2408965 | 9136295 | 1932119 | 9431627 | 2819056 |
| RP_POS_304_26056a11_252 | RP | POS | 11.25 | 304.2606 | | | | 3.57E-11 | 1.00 | 1.52 | 5.43E-01 | -0.12 | 0.42 | 1.09E-06 | 0.94 | 1.51 | 3.09E-09 | 1.06 | 1.76 | 10981931 | 4178411 | 19101752 | 8095723 | 19597651 | 7479935 | 18605854 | 8715947 |
| RP_POS_627_46881a10_458 | RP | POS | 10.46 | 627.4688 | | | | 4.72E-01 | -0.13 | 0.63 | 5.43E-01 | -0.13 | 0.88 | 3.51E-01 | -0.20 | 0.91 | 7.47E-01 | -0.07 | 0.14 | 964824 | 133213 | 944134 | 162352 | 954074 | 166126 | 934194 | 159546 |
| RP_POS_328_8989a15_377 | RP | POS | 15.38 | 328.8989 | | | | 3.09E-01 | 0.23 | 0.61 | 5.43E-01 | -0.12 | 0.59 | 4.87E-01 | 0.17 | 0.48 | 2.34E-01 | 0.29 | 0.53 | 1771652 | 115245 | 1794725 | 94158 | 1800489 | 88952 | 1788960 | 99660 |
| RP_POS_499_8262a3_786 | RP | POS | 3.79 | 499.8262 | | | | 1.18E-01 | 0.26 | 0.62 | 5.43E-01 | -0.13 | 0.42 | 3.72E-01 | 0.19 | 0.69 | 6.96E-02 | 0.32 | 0.89 | 1668517 | 1552783 | 2246962 | 2415909 | 2394887 | 2005739 | 2099037 | 2779335 |
| RP_POS_540_36578a9_53 | RP | POS | 9.53 | 540.3658 | | | | 7.14E-05 | -0.76 | 1.36 | 5.44E-01 | 0.12 | 0.84 | 3.71E-03 | -0.70 | 1.29 | 1.27E-05 | -0.82 | 1.73 | 1398332 | 559869 | 891434 | 659185 | 851062 | 383367 | 931806 | 853007 |
| RP_POS_343_33157a9_476 | RP | POS | 9.48 | 343.3316 | | | | 4.51E-01 | -0.20 | 0.90 | 5.44E-01 | -0.10 | 0.44 | 3.57E-01 | -0.25 | 0.66 | 6.03E-01 | -0.15 | 0.53 | 1442446 | 1742953 | 1194074 | 1068019 | 1259389 | 1174115 | 1128759 | 957788 |
| RP_NEG_702_32214a10_63 | RP | NEG | 10.63 | 702.3221 | | | | 1.61E-02 | -0.48 | 1.13 | 5.44E-01 | 0.12 | 0.43 | 6.02E-02 | -0.42 | 0.90 | 1.46E-02 | -0.54 | 1.23 | 2319722 | 339550 | 2146036 | 359054 | 2124082 | 352491 | 2167990 | 367746 |
| RP_POS_236_11311a4_851 | RP | POS | 4.85 | 236.1131 | | | | 6.25E-01 | 0.09 | 0.52 | 5.44E-01 | -0.13 | 0.54 | 9.02E-01 | 0.03 | 0.43 | 4.83E-01 | 0.15 | 0.55 | 66501938 | 19729879 | 68603452 | 24739091 | 70115511 | 26555631 | 67091392 | 22949247 |
| RP_POS_344_66132a5_682 | RP | POS | 5.68 | 344.6613 | | | | 8.89E-01 | 0.03 | 0.68 | 5.44E-01 | 0.13 | 0.52 | 6.48E-01 | 0.09 | 0.62 | 8.83E-01 | -0.04 | 0.46 | 9879189 | 4601675 | 10014170 | 5076679 | 9703740 | 6143822 | 10324599 | 3758737 |
| RP_POS_332_15396a10_73 | RP | POS | 10.73 | 332.1540 | | | | 2.88E-03 | 0.48 | 1.39 | 5.44E-01 | -0.13 | 1.26 | 2.96E-02 | 0.42 | 1.20 | 5.42E-03 | 0.55 | 1.38 | 498233 | 250298 | 682152 | 404182 | 706833 | 410288 | 657471 | 400601 |
| RP_POS_295_1879a9_88 | RP | POS | 9.88 | 295.1879 | Unknown | 295.1868(100) | 4 | 1.76E-01 | 0.24 | 0.52 | 5.44E-01 | -0.13 | 0.39 | 3.74E-01 | 0.17 | 0.43 | 1.60E-01 | 0.30 | 0.56 | 3957647 | 1889536 | 4530188 | 2532136 | 4684763 | 2754115 | 4375612 | 2306590 |
| HILIC_POS_312_21677a0_819 | HILIC | POS | 0.819 | 312.2168 | | | | 4.60E-01 | -0.13 | 0.29 | 5.45E-01 | 0.13 | 0.68 | 7.89E-01 | -0.06 | 0.26 | 2.67E-01 | -0.19 | 0.69 | 2573286 | 1249999 | 2362799 | 1779207 | 2254204 | 1271856 | 2471393 | 2180385 |
| RP_POS_371_0199a0_666 | RP | POS | 0.67 | 371.0199 | | | | 6.04E-08 | -0.66 | 1.35 | 5.45E-01 | 0.13 | 1.19 | 3.71E-04 | -0.59 | 1.40 | 4.13E-05 | -0.72 | 1.48 | 1489861 | 74927 | 1139019 | 587996 | 1103164 | 602491 | 1174874 | 576990 |
| RP_NEG_305_86047a15_522 | RP | NEG | 15.52 | 305.8605 | | | | 1.45E-01 | 0.41 | 0.70 | 5.45E-01 | -0.09 | 0.63 | 2.15E-01 | 0.36 | 0.56 | 1.15E-01 | 0.46 | 0.73 | 1240571 | 103841 | 1269026 | 53359 | 1272278 | 50171 | 1265774 | 56690 |
| RP_POS_482_4054a10_709 | RP | POS | 10.71 | 482.4054 | | | | 9.66E-01 | -0.01 | 0.54 | 5.45E-01 | 0.12 | 0.44 | 8.19E-01 | 0.05 | 0.42 | 7.65E-01 | -0.07 | 0.38 | 10666799 | 1100052 | 10657379 | 1075857 | 10591831 | 1107599 | 10722928 | 1050246 |
| RP_NEG_325_18567a11_611 | RP | NEG | 11.61 | 325.1857 | | | | 8.45E-02 | -0.33 | 0.83 | 5.46E-01 | 0.12 | 0.56 | 2.06E-01 | -0.26 | 0.69 | 7.89E-02 | -0.39 | 0.85 | 3107807 | 546122 | 2904939 | 636693 | 2866221 | 673581 | 2943658 | 601879 |
| RP_POS_428_26151a6_293 | RP | POS | 6.29 | 428.2615 | | | | 1.44E-07 | 0.63 | 1.33 | 5.46E-01 | -0.13 | 1.17 | 4.63E-04 | 0.56 | 1.40 | 1.05E-04 | 0.69 | 1.43 | 12651 | 9811 | 446717 | 765938 | 493279 | 804934 | 400156 | 730037 |
| RP_POS_227_12578a7_012 | RP | POS | 7.01 | 227.1258 | | | | 5.67E-02 | -0.36 | 0.75 | 5.46E-01 | 0.12 | 0.75 | 1.56E-01 | -0.30 | 0.66 | 5.29E-02 | -0.43 | 0.77 | 1256773 | 115972 | 1209234 | 133369 | 1201130 | 137748 | 1217338 | 129727 |
| HILIC_POS_161_20473a4_817 | HILIC | POS | 4.817 | 161.2047 | | | | 2.30E-02 | 0.40 | 0.90 | 5.46E-01 | -0.13 | 0.53 | 9.69E-02 | 0.34 | 0.58 | 2.54E-02 | 0.46 | 1.23 | 1165276 | 848816 | 1605266 | 1153505 | 1675344 | 1174955 | 1535187 | 1139193 |
| RP_NEG_374_13452a11_162 | RP | NEG | 11.16 | 374.1345 | | | | 3.67E-03 | 0.52 | 1.31 | 5.46E-01 | -0.13 | 1.31 | 2.70E-02 | 0.46 | 1.24 | 4.73E-03 | 0.58 | 1.34 | 2280482 | 249041 | 2445668 | 327904 | 2465586 | 324899 | 2425750 | 332966 |
| RP_POS_472_25647a6_394 | RP | POS | 6.39 | 472.2565 | | | | 1.32E-07 | 0.63 | 1.33 | 5.46E-01 | -0.13 | 1.17 | 4.06E-04 | 0.56 | 1.41 | 1.08E-04 | 0.70 | 1.43 | 10090 | 9709 | 279848 | 474169 | 308649 | 501151 | 251047 | 448793 |
| RP_NEG_267_07404a1_789 | RP | NEG | 1.79 | 267.0740 | | | | 2.30E-09 | 0.73 | 1.36 | 5.46E-01 | 0.13 | 0.77 | 4.70E-05 | 0.80 | 1.43 | 4.44E-06 | 0.67 | 1.51 | 261595 | 1049863 | 4888310 | 6870853 | 4470916 | 5684297 | 5305705 | 7919212 |
| RP_POS_376_3187a10_453 | RP | POS | 10.45 | 376.3187 | | | | 8.71E-01 | -0.03 | 0.76 | 5.46E-01 | -0.12 | 0.48 | 6.69E-01 | -0.09 | 0.79 | 9.05E-01 | 0.03 | 0.57 | 696173 | 207760 | 689275 | 210374 | 702050 | 227824 | 676500 | 192824 |
| RP_POS_207_39899a10_591 | RP | POS | 10.59 | 207.3990 | | | | 1.09E-03 | 0.57 | 1.02 | 5.46E-01 | -0.13 | 0.45 | 1.29E-02 | 0.51 | 0.88 | 1.65E-03 | 0.64 | 1.30 | 1114629 | 485755 | 1484936 | 667415 | 1525460 | 652354 | 1444411 | 686337 |
| RP_POS_492_8812a0_471 | RP | POS | 0.47 | 492.8812 | | | | 4.39E-09 | -0.74 | 1.38 | 5.47E-01 | 0.13 | 1.15 | 9.98E-05 | -0.67 | 1.43 | 4.75E-06 | -0.80 | 1.53 | 1016655 | 103457 | 709413 | 450716 | 682073 | 449610 | 736754 | 454709 |
| HILIC_NEG_132_92363a5_428 | HILIC | NEG | 5.428 | 132.9236 | | | | 4.75E-02 | -0.46 | 1.00 | 5.47E-01 | -0.11 | 0.39 | 3.99E-02 | -0.51 | 0.97 | 1.02E-01 | -0.40 | 0.83 | 5324126 | 619174 | 5079315 | 494098 | 5109247 | 487039 | 5049383 | 504196 |
| HILIC_NEG_115_92069a4_16 | HILIC | NEG | 4.16 | 115.9207 | | | | 4.64E-01 | 0.12 | 0.66 | 5.48E-01 | -0.13 | 0.61 | 7.70E-01 | 0.06 | 0.38 | 3.47E-01 | 0.19 | 0.51 | 41602743 | 8486526 | 43039966 | 12489476 | 43795814 | 12493834 | 42284118 | 12565652 |
| RP_NEG_852_57843a13_375 | RP | NEG | 13.38 | 852.5784 | Unknown | 301.2182(100), 283.2661(85), 792.5403(60) | 4 | 1.42E-02 | 0.51 | 1.07 | 5.48E-01 | -0.12 | 0.41 | 4.35E-02 | 0.46 | 0.85 | 1.46E-02 | 0.57 | 1.00 | 11748149 | 4150887 | 13872194 | 4008337 | 14114759 | 4192455 | 13629630 | 3842482 |
| RP_POS_621_44519a15_102 | RP | POS | 15.10 | 621.4452 | | | | 3.91E-01 | 0.18 | 0.57 | 5.48E-01 | 0.12 | 0.52 | 2.88E-01 | 0.24 | 0.43 | 6.14E-01 | 0.12 | 0.73 | 7951055 | 2169081 | 8328253 | 2074000 | 8202775 | 2239635 | 8453731 | 1908572 |
| HILIC_POS_512_33557a1_847 | HILIC | POS | 1.847 | 512.3356 | | | | 8.35E-02 | -0.30 | 0.93 | 5.48E-01 | -0.13 | 0.74 | 1.01E-01 | -0.36 | 0.55 | 1.99E-01 | -0.23 | 0.48 | 2823726 | 738238 | 2531307 | 1036077 | 2593971 | 848231 | 2468643 | 1200546 |
| RP_POS_531_31696a10_323 | RP | POS | 10.32 | 531.3170 | | | | 4.55E-02 | -0.44 | 0.87 | 5.48E-01 | -0.12 | 0.54 | 4.05E-02 | -0.50 | 0.87 | 1.05E-01 | -0.38 | 0.63 | 644118 | 346414 | 503466 | 306621 | 522004 | 295474 | 484928 | 319288 |
| HILIC_NEG_191_81656a3_005 | HILIC | NEG | 3.005 | 191.8166 | | | | 1.81E-01 | -0.27 | 0.48 | 5.48E-01 | -0.12 | 0.50 | 1.19E-01 | -0.33 | 0.63 | 3.85E-01 | -0.21 | 0.56 | 550726 | 85216 | 526900 | 90503 | 532373 | 103511 | 521427 | 75981 |
| HILIC_POS_86_90928a2_83 | HILIC | POS | 2.83 | 86.9093 | | | | 5.54E-05 | 0.50 | 1.38 | 5.48E-01 | -0.13 | 0.65 | 5.51E-03 | 0.44 | 1.13 | 2.03E-03 | 0.57 | 1.68 | 1748844 | 284205 | 2284172 | 1179647 | 2355469 | 1277011 | 2212875 | 1081851 |
| RP_NEG_722_82727a0_495 | RP | NEG | 0.50 | 722.8273 | | | | 2.90E-08 | -0.69 | 1.36 | 5.48E-01 | 0.13 | 1.19 | 2.33E-04 | -0.62 | 1.40 | 2.18E-05 | -0.75 | 1.49 | 2108931 | 169838 | 1544540 | 900599 | 1490122 | 916792 | 1598958 | 890032 |
| RP_POS_811_54382a13_387 | RP | POS | 13.39 | 811.5438 | | | | 4.86E-01 | -0.15 | 0.52 | 5.49E-01 | -0.12 | 0.45 | 3.89E-01 | -0.21 | 0.55 | 7.00E-01 | -0.09 | 0.50 | 1261113 | 572121 | 1180660 | 541153 | 1213351 | 498897 | 1147969 | 583601 |
| RP_NEG_319_88931a15_356 | RP | NEG | 15.36 | 319.8893 | | | | 6.11E-01 | -0.10 | 0.70 | 5.49E-01 | -0.12 | 0.41 | 4.61E-01 | -0.16 | 0.57 | 8.56E-01 | -0.04 | 0.49 | 2340206 | 83086 | 2331517 | 85352 | 2336671 | 89764 | 2326362 | 81282 |
| RP_NEG_226_97752a15_725 | RP | NEG | 15.73 | 226.9775 | | | | 3.57E-01 | -0.13 | 0.59 | 5.49E-01 | 0.14 | 0.39 | 5.68E-01 | -0.06 | 0.39 | 3.96E-01 | -0.20 | 0.63 | 7940261 | 229828 | 7877028 | 550317 | 7843743 | 745616 | 7910314 | 231674 |
| RP_POS_207_96658a0_917 | RP | POS | 0.92 | 207.9666 | | | | 6.23E-13 | 0.94 | 1.46 | 5.49E-01 | 0.13 | 0.87 | 2.49E-07 | 1.00 | 1.61 | 3.60E-08 | 0.88 | 1.57 | 1352257 | 302400 | 2257534 | 998327 | 2197292 | 878977 | 2317776 | 1110696 |
| RP_POS_153_07024a15_462 | RP | POS | 15.46 | 153.0702 | | | | 9.19E-01 | 0.02 | 0.47 | 5.50E-01 | 0.12 | 0.70 | 7.22E-01 | 0.08 | 0.28 | 8.83E-01 | -0.04 | 0.60 | 7177170 | 678890 | 7190963 | 608119 | 7154324 | 685406 | 7227602 | 524065 |
| RP_POS_169_09721a2_321 | RP | POS | 2.32 | 169.0972 | | | | 1.15E-07 | 0.71 | 1.33 | 5.50E-01 | 0.13 | 0.39 | 9.65E-05 | 0.77 | 1.34 | 3.17E-05 | 0.64 | 1.39 | 516433 | 208737 | 914172 | 604620 | 877762 | 511657 | 950581 | 688543 |
| HILIC_POS_186_11261a4_033 | HILIC | POS | 4.033 | 186.1126 | | | | 1.95E-02 | 0.44 | 0.89 | 5.50E-01 | -0.12 | 0.69 | 6.08E-02 | 0.38 | 0.71 | 2.68E-02 | 0.50 | 0.82 | 5636863 | 2699278 | 7019060 | 3231854 | 7213585 | 3607038 | 6824535 | 2831072 |
| RP_NEG_365_2326a10_804 | RP | NEG | 10.80 | 365.2326 | | | | 9.66E-01 | -0.01 | 0.78 | 5.50E-01 | 0.13 | 0.80 | 7.93E-01 | 0.06 | 0.66 | 7.40E-01 | -0.07 | 0.75 | 737641 | 108223 | 736657 | 133379 | 728631 | 135670 | 744683 | 131928 |
| RP_NEG_207_83691a3_108 | RP | NEG | 3.11 | 207.8369 | | | | 3.65E-02 | 0.38 | 0.92 | 5.51E-01 | -0.13 | 0.42 | 1.21E-01 | 0.31 | 1.06 | 4.04E-02 | 0.44 | 0.70 | 1819660 | 1220973 | 2391923 | 1591671 | 2487557 | 1675705 | 2296290 | 1513896 |
| RP_NEG_360_23993a7_233 | RP | NEG | 7.23 | 360.2399 | | | | 4.92E-01 | -0.11 | 0.78 | 5.51E-01 | -0.13 | 1.04 | 3.04E-01 | -0.18 | 0.58 | 8.23E-01 | -0.05 | 0.71 | 607477 | 420254 | 539847 | 638606 | 578234 | 756550 | 501460 | 498580 |
| RP_NEG_210_84201a0_523 | RP | NEG | 0.52 | 210.8420 | | | | 7.32E-03 | 0.37 | 1.15 | 5.51E-01 | -0.13 | 1.11 | 7.57E-02 | 0.30 | 1.07 | 2.02E-02 | 0.43 | 1.27 | 1197474 | 174323 | 1344821 | 443982 | 1371475 | 469235 | 1318167 | 420275 |
| RP_POS_198_18532a8_862 | RP | POS | 8.86 | 198.1853 | | | | 2.54E-01 | 0.17 | 0.67 | 5.51E-01 | 0.13 | 0.60 | 2.27E-01 | 0.24 | 0.44 | 5.48E-01 | 0.11 | 0.50 | 612076 | 280394 | 695361 | 529610 | 663598 | 492231 | 727124 | 567756 |
| HILIC_POS_105_04266a5_463 | HILIC | POS | 5.463 | 105.0427 | | | | 9.98E-01 | 0.00 | 0.35 | 5.51E-01 | 0.12 | 0.66 | 7.95E-01 | 0.06 | 0.44 | 7.91E-01 | -0.06 | 0.11 | 1855478 | 248738 | 1855366 | 246774 | 1840571 | 246805 | 1870161 | 248352 |
| RP_POS_325_21078a10_979 | RP | POS | 10.98 | 325.2108 | | | | 1.16E-02 | 0.45 | 1.45 | 5.52E-01 | -0.12 | 1.41 | 6.65E-02 | 0.39 | 1.22 | 1.12E-02 | 0.51 | 1.39 | 7604494 | 2742510 | 9177553 | 3645771 | 9396105 | 3466957 | 8959001 | 3838916 |
| RP_POS_221_98225a1_709 | RP | POS | 1.71 | 221.9823 | | | | 2.17E-12 | 0.92 | 1.48 | 5.52E-01 | 0.12 | 0.48 | 1.54E-06 | 0.98 | 1.49 | 3.55E-09 | 0.86 | 1.73 | 1187991 | 363418 | 2221846 | 1166737 | 2151874 | 901483 | 2291818 | 1388404 |
| RP_POS_249_88596a0_246 | RP | POS | 0.25 | 249.8860 | | | | 8.88E-01 | -0.03 | 0.73 | 5.52E-01 | -0.12 | 0.46 | 6.88E-01 | -0.09 | 0.34 | 8.76E-01 | 0.03 | 1.20 | 697306 | 76428 | 695051 | 84443 | 700112 | 83606 | 689989 | 85817 |
| RP_POS_561_37604a10_896 | RP | POS | 10.90 | 561.3760 | | | | 7.39E-01 | -0.06 | 0.53 | 5.52E-01 | 0.12 | 0.39 | 9.88E-01 | 0.00 | 0.62 | 5.44E-01 | -0.13 | 0.44 | 2448631 | 865139 | 2388397 | 952154 | 2331327 | 825861 | 2445468 | 1069114 |
| RP_NEG_665_43024a11_253 | RP | NEG | 11.25 | 665.4302 | | | | 4.52E-06 | 0.85 | 1.68 | 5.52E-01 | 0.12 | 1.04 | 2.68E-05 | 0.90 | 1.64 | 8.02E-05 | 0.79 | 1.61 | 992290 | 272832 | 1288498 | 343052 | 1267940 | 318330 | 1309056 | 368204 |
| RP_POS_649_42542a10_902 | RP | POS | 10.90 | 649.4254 | | | | 4.96E-01 | 0.12 | 0.41 | 5.52E-01 | -0.13 | 0.40 | 7.81E-01 | 0.06 | 0.40 | 3.82E-01 | 0.18 | 0.64 | 1240513 | 456373 | 1309785 | 612658 | 1346425 | 631683 | 1273145 | 597157 |
| RP_POS_372_12274a7_727 | RP | POS | 7.73 | 372.1227 | | | | 2.29E-01 | -0.21 | 0.43 | 5.53E-01 | -0.13 | 0.46 | 2.46E-01 | -0.28 | 0.62 | 3.78E-01 | -0.15 | 0.48 | 891991 | 57634 | 876510 | 76863 | 881109 | 48125 | 871912 | 97864 |
| RP_NEG_1061_68005a10_502 | RP | NEG | 10.50 | 1061.6801 | | | | 1.01E-03 | 0.66 | 1.44 | 5.53E-01 | 0.12 | 0.88 | 2.51E-03 | 0.72 | 1.30 | 3.30E-03 | 0.60 | 1.38 | 3174651 | 1091945 | 3954307 | 1145372 | 3885894 | 908848 | 4022719 | 1347209 |
| HILIC_NEG_234_82008a6_89 | HILIC | NEG | 6.89 | 234.8201 | | | | 5.05E-01 | 0.14 | 0.76 | 5.53E-01 | -0.12 | 0.40 | 7.32E-01 | 0.08 | 0.46 | 3.80E-01 | 0.20 | 0.74 | 1034830 | 473161 | 1098496 | 445740 | 1125109 | 414156 | 1071884 | 477966 |
| HILIC_NEG_134_89476a0_824 | HILIC | NEG | 0.824 | 134.8948 | | | | 9.58E-01 | 0.01 | 0.53 | 5.54E-01 | -0.11 | 0.86 | 8.51E-01 | -0.05 | 0.20 | 7.76E-01 | 0.07 | 0.11 | 19809288 | 3471933 | 19844956 | 2954259 | 20020928 | 2947987 | 19668985 | 2979863 |
| RP_POS_896_54144a12_957 | RP | POS | 12.96 | 896.5414 | | | | 1.08E-05 | -1.05 | 1.65 | 5.54E-01 | -0.10 | 0.61 | 1.00E-05 | -1.10 | 1.74 | 5.31E-05 | -1.00 | 1.51 | 6030919 | 1888653 | 4222735 | 1418286 | 4307184 | 1463889 | 4138287 | 1380797 |
| RP_POS_177_10202a0_71 | RP | POS | 0.71 | 177.1020 | | | | 1.57E-06 | 0.84 | 1.46 | 5.54E-01 | 0.12 | 0.48 | 2.97E-05 | 0.90 | 1.39 | 2.74E-05 | 0.78 | 1.64 | 2262403 | 4376048 | 7416711 | 6109719 | 7052884 | 5240371 | 7780538 | 6905615 |
| HILIC_NEG_116_92861a7_66 | HILIC | NEG | 7.66 | 116.9286 | | | | 2.86E-01 | 0.19 | 0.33 | 5.54E-01 | -0.12 | 0.42 | 5.13E-01 | 0.13 | 0.26 | 2.41E-01 | 0.26 | 0.47 | 15046410 | 2776462 | 15694721 | 3496529 | 15902869 | 3778448 | 15486572 | 3214941 |
| RP_POS_756_55267a11_642 | RP | POS | 11.64 | 756.5527 | | | | 6.61E-01 | 0.07 | 0.41 | 5.54E-01 | -0.13 | 0.51 | 9.59E-01 | 0.01 | 0.20 | 5.18E-01 | 0.14 | 0.48 | 414220 | 652551 | 480763 | 973942 | 538742 | 1082729 | 422783 | 858675 |
| HILIC_NEG_397_88855a5_427 | HILIC | NEG | 5.427 | 397.8886 | | | | 9.24E-01 | -0.02 | 0.55 | 5.54E-01 | -0.12 | 0.57 | 7.36E-01 | -0.08 | 0.18 | 8.66E-01 | 0.04 | 0.79 | 540769 | 52642 | 539760 | 50065 | 542739 | 49167 | 536781 | 51271 |
| HILIC_POS_118_12292a1_142 | HILIC | POS | 1.142 | 118.1229 | | | | 1.46E-02 | -0.53 | 1.14 | 5.55E-01 | 0.11 | 0.77 | 4.11E-02 | -0.48 | 1.07 | 1.41E-02 | -0.59 | 1.04 | 2799169 | 1436055 | 2078430 | 1279496 | 2002365 | 1323485 | 2154496 | 1242664 |

**Supplementary table 1: Metabolomics results** (in asceding order according to p-value from progressive ALD vs  non-progressive ALD comparison, with molecular features with ID_level of 1-2 first)

| Feature_ID | RP_HILIC | POS_NEG | Average.Rt | Average.Mz | Metabolite.name | curated_MSMS | ID_level | all_ALD_vs_control t-test p | all_ALD_vs_control Cohen's d | all_ALD_vs_control PLS-DA VIP | Prog_ALD_vs_non-prog_ALD t-test p | Prog_ALD_vs_non-prog_ALD Cohen's d | Prog_ALD_vs_non-prog_ALD PLS-DA VIP | Prog_ALD_vs_control_ALD t-test p | Prog_ALD_vs_control_ALD Cohen's d | Prog_ALD_vs_control_ALD PLS-DA VIP | Non-prog_ALD_vs_control t-test p | Non-prog_ALD_vs_control Cohen's d | Non-prog_ALD_vs_control PLS-DA VIP |
|---|---|---|---|---|---|---|---|---|---|---|---|---|---|---|---|---|---|---|---|
| RP_NEG_1087_6958a10_619 | RP | NEG | 10.62 | 1087.6958 | | | | 1.72E-03 | 0.66 | 1.42 | 5.55E-01 | -0.11 | 0.86 | 1.18E-02 | 0.60 | 1.18 | 1.09E-03 | 0.72 | 1.45 |
| HILIC_NEG_200_85867a6_161 | HILIC | NEG | 6.161 | 200.8587 | | | | 5.03E-01 | -0.10 | 0.81 | 5.55E-01 | -0.13 | 0.73 | 4.29E-01 | -0.17 | 0.69 | 8.24E-01 | -0.04 | 0.64 |
| RP_POS_513_84283a11_328 | RP | POS | 11.33 | 513.8428 | | | | 3.00E-01 | 0.15 | 0.37 | 5.55E-01 | -0.13 | 0.41 | 5.37E-01 | 0.08 | 0.40 | 3.29E-01 | 0.22 | 0.44 |
| RP_POS_309_2034a9_825 | RP | POS | 9.83 | 309.2034 | | | | 1.54E-02 | -0.52 | 0.84 | 5.55E-01 | 0.11 | 0.78 | 4.94E-02 | -0.46 | 0.80 | 1.32E-02 | -0.58 | 0.90 |
| RP_POS_406_24927a13_155 | RP | POS | 13.16 | 406.2493 | | | | 7.02E-01 | 0.08 | 0.44 | 5.55E-01 | -0.12 | 0.55 | 9.17E-01 | 0.02 | 0.31 | 5.48E-01 | 0.14 | 0.69 |
| HILIC_POS_330_33643a3_028 | HILIC | POS | 3.028 | 330.3364 | | | | 2.10E-08 | 0.70 | 1.33 | 5.55E-01 | -0.13 | 0.78 | 7.65E-05 | 0.64 | 1.38 | 3.76E-05 | 0.76 | 1.41 |
| RP_NEG_650_86877a0_465 | RP | NEG | 0.47 | 650.8688 | | | | 6.96E-10 | -0.80 | 1.43 | 5.56E-01 | 0.13 | 1.15 | 2.89E-05 | -0.74 | 1.47 | 1.05E-06 | -0.86 | 1.57 |
| RP_POS_140_06854a0_46 | RP | POS | 0.46 | 140.0685 | | | | 1.56E-06 | 0.58 | 1.31 | 5.56E-01 | -0.13 | 1.18 | 1.04E-03 | 0.52 | 1.36 | 3.90E-04 | 0.65 | 1.41 |
| RP_POS_698_77014a0_5 | RP | POS | 0.50 | 698.7701 | | | | 2.98E-02 | -0.31 | 1.09 | 5.56E-01 | -0.13 | 1.24 | 2.74E-02 | -0.38 | 1.10 | 2.00E-01 | -0.25 | 1.14 |
| RP_POS_169_0972a1_766 | RP | POS | 1.77 | 169.0972 | | | | 2.38E-07 | 0.68 | 1.30 | 5.57E-01 | 0.13 | 0.38 | 2.94E-04 | 0.74 | 1.27 | 1.72E-05 | 0.61 | 1.38 |
| RP_POS_805_55762a13_207 | RP | POS | 13.21 | 805.5576 | | | | 6.33E-02 | 0.39 | 0.84 | 5.57E-01 | 0.12 | 0.67 | 6.09E-02 | 0.44 | 0.79 | 1.40E-01 | 0.33 | 0.81 |
| RP_POS_497_39621a12_891 | RP | POS | 12.89 | 497.3962 | | | | 4.39E-01 | 0.15 | 0.62 | 5.57E-01 | -0.12 | 0.44 | 7.01E-01 | 0.09 | 0.66 | 3.10E-01 | 0.21 | 0.56 |
| RP_POS_254_24792a10_421 | RP | POS | 10.42 | 254.2479 | | | | 1.25E-05 | 0.70 | 1.15 | 5.57E-01 | -0.12 | 0.73 | 1.78E-03 | 0.64 | 1.09 | 2.03E-05 | 0.77 | 1.40 |
| HILIC_POS_173_12859a4_008 | HILIC | POS | 4.008 | 173.1286 | | | | 3.87E-01 | 0.15 | 0.87 | 5.58E-01 | -0.13 | 0.96 | 6.88E-01 | 0.09 | 0.53 | 2.70E-01 | 0.21 | 0.57 |
| RP_POS_790_53485a13_852 | RP | POS | 13.85 | 790.5349 | | | | 3.76E-01 | 0.19 | 0.52 | 5.58E-01 | 0.12 | 0.60 | 3.13E-01 | 0.25 | 0.59 | 5.59E-01 | 0.13 | 0.44 |
| HILIC_POS_90_09186a1_814 | HILIC | POS | 1.814 | 90.0919 | | | | 1.94E-05 | -0.63 | 1.33 | 5.58E-01 | 0.13 | 0.81 | 1.92E-03 | -0.56 | 1.42 | 2.34E-04 | -0.69 | 1.43 |
| HILIC_NEG_68_99498a0_572 | HILIC | NEG | 0.572 | 68.9950 | | | | 1.17E-01 | 0.19 | 0.49 | 5.58E-01 | -0.14 | 0.52 | 5.60E-02 | 0.12 | 0.65 | 2.64E-01 | 0.25 | 0.58 |
| RP_POS_443_33432a10_709 | RP | POS | 10.71 | 443.3343 | | | | 9.59E-02 | -0.38 | 0.65 | 5.58E-01 | 0.11 | 0.39 | 1.96E-01 | -0.32 | 0.52 | 7.30E-02 | -0.43 | 0.70 |
| RP_POS_497_39526a11_257 | RP | POS | 11.26 | 497.3953 | | | | 4.51E-02 | 0.35 | 1.34 | 5.58E-01 | 0.12 | 1.30 | 6.32E-02 | 0.41 | 1.23 | 1.19E-01 | 0.29 | 1.12 |
| RP_POS_344_85773a0_5 | RP | POS | 0.50 | 344.8577 | | | | 1.33E-02 | -0.36 | 1.11 | 5.59E-01 | -0.13 | 1.23 | 1.60E-02 | -0.42 | 1.15 | 1.26E-01 | -0.29 | 1.13 |
| RP_POS_497_25983a6_08 | RP | POS | 6.08 | 497.2598 | | | | 9.08E-01 | 0.02 | 0.49 | 5.59E-01 | 0.12 | 0.56 | 7.15E-01 | 0.08 | 0.51 | 8.43E-01 | -0.04 | 0.44 |
| RP_NEG_316_77795a15_615 | RP | NEG | 15.62 | 316.7780 | | | | 2.15E-01 | -0.27 | 0.77 | 5.59E-01 | -0.11 | 0.64 | 1.74E-01 | -0.33 | 0.59 | 3.64E-01 | -0.21 | 0.72 |
| HILIC_NEG_173_92599a7_355 | HILIC | NEG | 7.355 | 173.9260 | | | | 9.70E-01 | -0.01 | 0.18 | 5.59E-01 | -0.11 | 0.76 | 7.88E-01 | -0.07 | 0.15 | 8.36E-01 | 0.05 | 0.08 |
| RP_POS_553_45697a11_256 | RP | POS | 11.26 | 553.4570 | | | | 2.63E-02 | 0.41 | 1.13 | 5.59E-01 | 0.12 | 1.14 | 3.31E-02 | 0.47 | 1.12 | 8.30E-02 | 0.35 | 0.96 |
| RP_POS_199_13043a11_65 | RP | POS | 11.65 | 199.1304 | | | | 5.44E-01 | 0.11 | 0.56 | 5.60E-01 | 0.12 | 0.36 | 4.79E-01 | 0.17 | 0.48 | 7.75E-01 | 0.05 | 0.50 |
| RP_POS_434_75906a6_111 | RP | POS | 6.11 | 434.7591 | | | | 1.26E-06 | 0.58 | 1.30 | 5.60E-01 | -0.13 | 1.15 | 1.10E-03 | 0.51 | 1.39 | 3.60E-04 | 0.65 | 1.38 |
| RP_POS_181_0493a7_494 | RP | POS | 7.49 | 181.0493 | fragment of Diethyl-phthalate | 149.0228(100), 163.0386(45), 84.9603(70) | 3 | 7.22E-01 | 0.07 | 0.43 | 5.60E-01 | 0.12 | 1.03 | 5.55E-01 | 0.13 | 0.45 | 9.87E-01 | 0.00 | 0.44 |
| HILIC_POS_138_05505a4_254 | HILIC | POS | 4.254 | 138.0551 | | | | 7.03E-01 | -0.07 | 0.31 | 5.60E-01 | -0.12 | 0.88 | 5.32E-01 | -0.13 | 0.26 | 9.55E-01 | -0.01 | 0.18 |
| RP_NEG_299_25934a10_219 | RP | NEG | 10.22 | 299.2593 | | | | 3.80E-01 | 0.16 | 0.79 | 5.60E-01 | 0.12 | 0.69 | 3.06E-01 | 0.22 | 0.69 | 6.32E-01 | 0.10 | 0.73 |
| HILIC_NEG_200_85867a4_112 | HILIC | NEG | 4.112 | 200.8587 | | | | 6.95E-02 | -0.38 | 0.67 | 5.61E-01 | 0.12 | 0.75 | 1.71E-01 | -0.32 | 0.55 | 5.44E-02 | -0.43 | 0.79 |
| RP_NEG_379_15878a10_978 | RP | NEG | 10.98 | 379.1588 | | | | 1.34E-02 | 0.42 | 1.37 | 5.61E-01 | -0.12 | 1.24 | 7.71E-02 | 0.35 | 1.12 | 1.61E-02 | 0.48 | 1.32 |
| RP_POS_517_37115a10_546 | RP | POS | 10.55 | 517.3712 | | | | 5.28E-01 | 0.14 | 0.37 | 5.61E-01 | -0.11 | 0.38 | 7.43E-01 | 0.08 | 0.30 | 4.09E-01 | 0.19 | 0.89 |
| RP_POS_702_86194a0_491 | RP | POS | 0.49 | 702.8619 | | | | 7.64E-08 | 0.64 | 1.35 | 5.61E-01 | -0.13 | 1.20 | 2.36E-04 | 0.58 | 1.41 | 1.00E-04 | 0.71 | 1.46 |
| RP_POS_353_03723a4_852 | RP | POS | 4.85 | 353.0372 | | | | 6.79E-01 | 0.08 | 0.50 | 5.61E-01 | -0.12 | 0.59 | 9.24E-01 | 0.02 | 0.37 | 5.32E-01 | 0.14 | 0.52 |
| RP_POS_216_92223a0_516 | RP | POS | 0.52 | 216.9222 | | | | 9.29E-06 | 0.54 | 1.26 | 5.61E-01 | -0.13 | 1.15 | 2.82E-03 | 0.48 | 1.27 | 8.13E-04 | 0.61 | 1.39 |
| RP_POS_437_23682a6_111 | RP | POS | 6.11 | 437.2368 | | | | 2.63E-07 | 0.61 | 1.32 | 5.61E-01 | -0.13 | 1.16 | 5.18E-04 | 0.55 | 1.41 | 1.71E-04 | 0.68 | 1.40 |
| HILIC_NEG_117_91911a7_351 | HILIC | NEG | 7.351 | 117.9191 | | | | 3.09E-01 | -0.17 | 0.60 | 5.61E-01 | -0.12 | 0.59 | 2.65E-01 | -0.24 | 0.47 | 5.59E-01 | -0.11 | 0.21 |
| RP_POS_431_38412a11_038 | RP | POS | 11.04 | 431.3841 | | | | 4.51E-02 | 0.33 | 0.90 | 5.61E-01 | -0.13 | 0.43 | 1.47E-01 | 0.26 | 0.68 | 6.34E-02 | 0.39 | 0.76 |
| RP_POS_224_86421a0_519 | RP | POS | 0.52 | 224.8642 | | | | 2.17E-05 | -0.72 | 1.29 | 5.62E-01 | 0.12 | 1.06 | 6.28E-04 | -0.66 | 1.25 | 1.38E-04 | -0.78 | 1.34 |
| RP_POS_575_33826a15_435 | RP | POS | 15.44 | 575.3383 | | | | 8.30E-01 | -0.04 | 0.76 | 5.62E-01 | -0.12 | 0.37 | 6.48E-01 | -0.10 | 0.36 | 9.35E-01 | 0.02 | 0.67 |
| RP_POS_173_96153a0_251 | RP | POS | 0.25 | 173.9615 | | | | 8.81E-01 | 0.03 | 0.22 | 5.62E-01 | -0.11 | 0.47 | 9.17E-01 | -0.03 | 0.32 | 7.00E-01 | 0.09 | 0.63 |
| HILIC_NEG_157_86234a3_023 | HILIC | NEG | 3.023 | 157.8623 | | | | 2.74E-01 | -0.22 | 0.98 | 5.62E-01 | -0.12 | 0.83 | 2.16E-01 | -0.27 | 0.47 | 4.83E-01 | -0.16 | 0.26 |
| RP_NEG_321_92273a6_867 | RP | NEG | 6.87 | 321.9227 | | | | 1.89E-01 | -0.24 | 0.54 | 5.63E-01 | 0.12 | 0.95 | 3.59E-01 | -0.18 | 0.36 | 1.82E-01 | -0.30 | 0.51 |
| RP_POS_325_32242a10_585 | RP | POS | 10.59 | 325.3224 | | | | 1.67E-01 | 0.30 | 0.63 | 5.63E-01 | -0.11 | 0.40 | 3.16E-01 | 0.25 | 0.43 | 1.23E-01 | 0.36 | 0.84 |
| RP_POS_237_14659a7_809 | RP | POS | 7.81 | 237.1466 | | | | 7.79E-01 | 0.06 | 0.59 | 5.63E-01 | 0.11 | 0.73 | 6.31E-01 | 0.12 | 0.52 | 9.87E-01 | 0.00 | 0.40 |
| RP_POS_770_84924a0_49 | RP | POS | 0.49 | 770.8492 | | | | 8.04E-08 | 0.64 | 1.35 | 5.64E-01 | -0.13 | 1.20 | 2.48E-04 | 0.58 | 1.41 | 1.01E-04 | 0.71 | 1.46 |
| HILIC_NEG_194_92735a7_667 | HILIC | NEG | 7.667 | 194.9274 | | | | 3.87E-01 | 0.17 | 0.72 | 5.64E-01 | 0.12 | 0.79 | 3.01E-01 | 0.23 | 0.45 | 6.23E-01 | 0.11 | 0.49 |
| RP_POS_419_70483a7_55 | RP | POS | 7.55 | 419.7048 | | | | 5.92E-01 | -0.11 | 0.90 | 5.64E-01 | 0.12 | 0.81 | 8.30E-01 | -0.05 | 0.77 | 4.65E-01 | -0.17 | 0.84 |
| HILIC_NEG_134_89476a1_408 | HILIC | NEG | 1.408 | 134.8948 | | | | 8.95E-01 | -0.02 | 0.73 | 5.64E-01 | 0.13 | 0.86 | 8.38E-01 | 0.04 | 0.23 | 6.03E-01 | -0.08 | 0.19 |
| RP_POS_532_43884a14_268 | RP | POS | 14.27 | 532.4388 | | | | 3.06E-01 | 0.18 | 0.36 | 5.64E-01 | 0.12 | 0.37 | 2.96E-01 | 0.24 | 0.47 | 4.97E-01 | 0.12 | 0.45 |
| RP_POS_172_97864a15_38 | RP | POS | 15.38 | 172.9786 | | | | 9.18E-01 | -0.02 | 0.42 | 5.64E-01 | 0.12 | 0.39 | 8.55E-01 | 0.04 | 0.32 | 7.30E-01 | -0.08 | 0.45 |
| RP_POS_680_38324a11_426 | RP | POS | 11.43 | 680.3832 | | | | 9.35E-01 | -0.02 | 0.23 | 5.65E-01 | 0.12 | 0.74 | 8.48E-01 | 0.04 | 0.54 | 7.38E-01 | -0.08 | 0.26 |
| HILIC_POS_137_04587a2_457 | HILIC | POS | 2.457 | 137.0459 | | | | 1.03E-09 | 0.74 | 1.25 | 5.65E-01 | 0.13 | 0.63 | 5.17E-05 | 0.80 | 1.26 | 1.87E-06 | 0.67 | 1.72 |
| RP_POS_184_17029a2_197 | RP | POS | 2.20 | 184.1703 | | | | 2.65E-02 | 0.29 | 0.50 | 5.65E-01 | -0.13 | 0.34 | 3.22E-02 | 0.22 | 0.79 | 1.12E-01 | 0.36 | 0.64 |
| RP_POS_838_83618a0_49 | RP | POS | 0.49 | 838.8362 | | | | 7.21E-08 | 0.65 | 1.35 | 5.65E-01 | -0.13 | 1.20 | 2.29E-04 | 0.58 | 1.41 | 9.78E-05 | 0.71 | 1.46 |
| RP_POS_524_27405a10_326 | RP | POS | 10.33 | 524.2741 | | | | 1.51E-08 | 1.10 | 1.82 | 5.66E-01 | 0.10 | 0.45 | 1.88E-07 | 1.16 | 1.77 | 1.83E-07 | 1.05 | 1.84 |
| RP_POS_265_17914a8_529 | RP | POS | 8.53 | 265.1791 | 1S,2R,9R,10S,12S)-12-hydroxy-7,15-diazatetracyclo[7.7.1.0^{2,7}.0^{10,15}]heptadecan-6-one | 265.1796(100), 57.0705(45) | 3 | 4.64E-01 | 0.14 | 0.54 | 5.66E-01 | 0.12 | 0.51 | 3.76E-01 | 0.20 | 0.56 | 7.14E-01 | 0.07 | 0.45 |
| RP_POS_634_87494a0_493 | RP | POS | 0.49 | 634.8749 | | | | 1.11E-07 | 0.64 | 1.34 | 5.66E-01 | -0.13 | 1.20 | 2.78E-04 | 0.57 | 1.40 | 1.23E-04 | 0.70 | 1.45 |
| RP_POS_185_11479a13_013 | RP | POS | 13.01 | 185.1148 | | | | 3.45E-01 | 0.20 | 0.74 | 5.66E-01 | 0.11 | 0.40 | 2.74E-01 | 0.25 | 0.78 | 5.44E-01 | 0.14 | 0.41 |
| RP_POS_636_55511a14_987 | RP | POS | 14.99 | 636.5551 | | | | 2.44E-02 | -0.32 | 0.81 | 5.66E-01 | 0.13 | 0.36 | 2.56E-01 | -0.26 | 0.56 | 1.65E-03 | -0.39 | 1.07 |
| HILIC_POS_440_18939a0_947 | HILIC | POS | 0.947 | 440.1894 | | | | 7.07E-01 | -0.09 | 0.51 | 5.66E-01 | -0.11 | 0.38 | 5.46E-01 | -0.14 | 0.55 | 8.99E-01 | -0.03 | 0.38 |
| RP_POS_223_09395a11_629 | RP | POS | 11.63 | 223.0940 | | | | 3.21E-01 | -0.20 | 0.38 | 5.66E-01 | 0.12 | 0.42 | 5.75E-01 | -0.14 | 0.38 | 1.87E-01 | -0.26 | 0.72 |
| RP_NEG_397_00366a4_57 | RP | NEG | 4.57 | 397.0037 | | | | 1.03E-02 | -0.56 | 0.90 | 5.67E-01 | -0.11 | 0.40 | 1.20E-02 | -0.62 | 0.94 | 2.64E-02 | -0.51 | 0.84 |
| RP_POS_570_35291a10_753 | RP | POS | 10.75 | 570.3529 | | | | 1.33E-01 | 0.30 | 1.03 | 5.67E-01 | -0.12 | 0.81 | 3.04E-01 | 0.25 | 0.75 | 8.68E-02 | 0.36 | 1.00 |
| RP_POS_562_79602a0_5 | RP | POS | 0.50 | 562.7960 | | | | 2.83E-02 | -0.32 | 1.09 | 5.67E-01 | -0.13 | 1.23 | 2.66E-02 | -0.39 | 1.10 | 1.82E-01 | -0.26 | 1.12 |
| RP_POS_285_13071a8_608 | RP | POS | 8.61 | 285.1307 | | | | 4.41E-02 | 0.39 | 0.84 | 5.67E-01 | -0.12 | 0.38 | 1.34E-01 | 0.34 | 0.61 | 3.62E-02 | 0.45 | 0.96 |
| RP_NEG_197_80769a0_702 | RP | NEG | 0.70 | 197.8077 | | | | 8.71E-02 | 0.26 | 0.60 | 5.67E-01 | -0.13 | 0.56 | 2.99E-01 | 0.20 | 0.36 | 8.28E-02 | 0.32 | 0.88 |
| HILIC_NEG_136_89166a4_83 | HILIC | NEG | 4.83 | 136.8917 | | | | 3.88E-02 | 0.42 | 0.80 | 5.68E-01 | 0.11 | 0.52 | 3.44E-02 | 0.48 | 0.89 | 1.07E-01 | 0.37 | 0.59 |
| RP_NEG_588_8985a0_49 | RP | NEG | 0.49 | 588.8985 | | | | 1.46E-07 | 0.63 | 1.34 | 5.68E-01 | -0.13 | 1.20 | 2.93E-04 | 0.57 | 1.40 | 1.50E-04 | 0.69 | 1.44 |
| RP_POS_844_54449a11_619 | RP | POS | 11.62 | 844.5445 | | | | 6.90E-01 | 0.06 | 0.64 | 5.68E-01 | -0.13 | 0.59 | 9.99E-01 | 0.00 | 0.38 | 5.43E-01 | 0.13 | 0.63 |
| HILIC_POS_134_11755a1_649 | HILIC | POS | 1.649 | 134.1176 | | | | 1.99E-04 | -0.67 | 1.30 | 5.68E-01 | 0.12 | 0.81 | 3.03E-03 | -0.61 | 1.31 | 4.45E-04 | -0.72 | 1.35 |
| RP_POS_170_1176a4_742 | RP | POS | 4.74 | 170.1176 | | | | 9.76E-01 | 0.00 | 0.49 | 5.68E-01 | 0.13 | 0.54 | 8.05E-01 | 0.06 | 0.55 | 6.44E-01 | -0.07 | 0.57 |
| RP_POS_181_03862a15_342 | RP | POS | 15.34 | 181.0386 | | | | 8.48E-01 | 0.04 | 0.69 | 5.68E-01 | -0.11 | 0.40 | 9.39E-01 | -0.02 | 0.28 | 6.78E-01 | 0.10 | 1.02 |
| RP_POS_443_33426a10_657 | RP | POS | 10.66 | 443.3343 | | | | 5.84E-01 | -0.11 | 0.72 | 5.68E-01 | 0.11 | 0.67 | 8.02E-01 | -0.06 | 0.37 | 4.64E-01 | -0.17 | 0.68 |
| RP_POS_229_1192a7_341 | RP | POS | 7.34 | 229.1192 | | | | 1.51E-02 | -0.48 | 0.78 | 5.69E-01 | -0.11 | 0.38 | 1.62E-02 | -0.54 | 0.88 | 5.22E-02 | -0.42 | 0.71 |
| RP_NEG_514_8938a0_465 | RP | NEG | 0.47 | 514.8938 | | | | 6.48E-10 | -0.77 | 1.42 | 5.69E-01 | 0.12 | 1.17 | 3.77E-05 | -0.71 | 1.47 | 2.10E-06 | -0.83 | 1.55 |
| RP_POS_266_04352a3_97 | RP | POS | 3.97 | 266.0435 | | | | 4.96E-01 | 0.11 | 0.83 | 5.69E-01 | -0.12 | 0.35 | 8.16E-01 | 0.05 | 0.59 | 3.22E-01 | 0.17 | 0.42 |
| HILIC_POS_374_15552a0_552 | HILIC | POS | 0.552 | 374.1555 | | | | 6.45E-04 | 0.51 | 0.93 | 5.69E-01 | 0.12 | 0.92 | 4.86E-03 | 0.57 | 1.09 | 7.40E-03 | 0.45 | 1.03 |
| HILIC_NEG_180_84201a1_366 | HILIC | NEG | 1.366 | 180.8420 | | | | 8.76E-01 | -0.03 | 0.89 | 5.69E-01 | -0.12 | 0.81 | 6.80E-01 | -0.09 | 0.14 | 8.61E-01 | 0.03 | 0.19 |
| RP_POS_470_24081a6_222 | RP | POS | 6.22 | 470.2408 | | | | 2.59E-07 | 0.61 | 1.31 | 5.69E-01 | -0.13 | 1.16 | 4.79E-04 | 0.55 | 1.41 | 1.81E-04 | 0.68 | 1.40 |
| HILIC_POS_138_09137a0_752 | HILIC | POS | 0.752 | 138.0914 | | | | 1.26E-07 | -1.04 | 1.55 | 5.69E-01 | -0.11 | 0.75 | 2.29E-07 | -1.09 | 1.81 | 5.32E-06 | -0.99 | 1.55 |
| RP_POS_441_29675a11_254 | RP | POS | 11.25 | 441.2968 | | | | 6.23E-06 | 0.85 | 1.63 | 5.70E-01 | 0.11 | 1.18 | 3.30E-05 | 0.91 | 1.74 | 8.11E-05 | 0.80 | 1.49 |
| RP_NEG_319_98462a3_961 | RP | NEG | 3.96 | 319.9846 | | | | 6.88E-01 | 0.07 | 1.01 | 5.70E-01 | -0.12 | 0.41 | 9.71E-01 | 0.01 | 0.85 | 4.76E-01 | 0.13 | 0.50 |
| RP_POS_355_36877a11_247 | RP | POS | 11.25 | 355.3688 | | | | 1.08E-11 | 0.99 | 1.52 | 5.71E-01 | 0.12 | 0.90 | 5.36E-07 | 1.04 | 1.59 | 2.28E-09 | 0.93 | 1.77 |
| RP_NEG_344_85251a15_438 | RP | NEG | 15.44 | 344.8525 | | | | 3.27E-01 | 0.27 | 0.80 | 5.71E-01 | 0.09 | 0.66 | 2.49E-01 | 0.31 | 0.69 | 4.55E-01 | 0.22 | 0.44 |

| Feature_ID | Healthy_control Mean | Healthy_control SD | all_ALD Mean | all_ALD SD | Non-prog_ALD Mean | Non-prog_ALD SD | Progressive_ALD Mean | Progressive_ALD SD |
|---|---|---|---|---|---|---|---|---|
| RP_NEG_1087_6958a10_619 | 15206392 | 6827963 | 19754223 | 6553637 | 20143851 | 5458158 | 19364594 | 7528317 |
| HILIC_NEG_200_85867a6_161 | 3945014 | 354665 | 3883340 | 669959 | 3923176 | 533011 | 3843505 | 787091 |
| RP_POS_513_84283a11_328 | 125704 | 132904 | 164391 | 287425 | 181489 | 365203 | 147293 | 181499 |
| RP_POS_309_2034a9_825 | 1677871 | 132365 | 1611537 | 122514 | 1604261 | 118945 | 1618814 | 126766 |
| RP_POS_406_24927a13_155 | 1976085 | 780307 | 2035752 | 702396 | 2077454 | 677207 | 1994050 | 731185 |
| HILIC_POS_330_33643a3_028 | 1102221 | 70847 | 1311655 | 325951 | 1331004 | 349756 | 1292305 | 302594 |
| RP_NEG_650_86877a0_465 | 774924 | 96909 | 526014 | 331938 | 506326 | 328654 | 545701 | 337356 |
| RP_POS_140_06854a0_46 | 751748 | 39749 | 895975 | 275252 | 912299 | 295113 | 879650 | 255809 |
| RP_POS_698_77014a0_5 | 1884815 | 507056 | 1574321 | 1090073 | 1638878 | 1184836 | 1509764 | 994195 |
| RP_POS_169_0972a1_766 | 979255 | 397926 | 1773162 | 1273906 | 1697710 | 981786 | 1848613 | 1517652 |
| RP_POS_805_55762a13_207 | 8216729 | 1443937 | 8770406 | 1412812 | 8686777 | 1297431 | 8854035 | 1528140 |
| RP_POS_497_39621a12_891 | 3088501 | 726615 | 3207020 | 816412 | 3255268 | 708172 | 3158771 | 916734 |
| RP_POS_254_24792a10_421 | 11613463 | 4160029 | 16271308 | 6858163 | 16676485 | 5945087 | 15866131 | 7703868 |
| HILIC_POS_173_12859a4_008 | 2375820 | 759047 | 2525080 | 1069608 | 2588236 | 963023 | 2461924 | 1172995 |
| RP_POS_790_53485a13_852 | 921805 | 666865 | 1040366 | 610432 | 1004331 | 538279 | 1076402 | 678576 |
| HILIC_POS_90_09186a1_814 | 1970592 | 408760 | 1472059 | 850960 | 1421851 | 863934 | 1522267 | 843515 |
| HILIC_NEG_68_99498a0_572 | 256951 | 56689 | 322276 | 400657 | 345979 | 552486 | 298573 | 133942 |
| RP_POS_443_33432a10_709 | 81138979 | 9329636 | 78040223 | 7757519 | 77582568 | 7298971 | 78497878 | 8239171 |
| RP_POS_497_39526a11_257 | 3460326 | 987811 | 3909986 | 1345797 | 3830680 | 1108349 | 3989293 | 1555001 |
| RP_POS_344_85773a0_5 | 892871 | 168461 | 773823 | 368045 | 795487 | 392298 | 752158 | 344708 |
| RP_POS_497_25983a6_08 | 3992866 | 239921 | 3998835 | 288161 | 3981882 | 248517 | 4015787 | 324685 |
| RP_NEG_316_77795a15_615 | 1494821 | 74031 | 1476434 | 65366 | 1480277 | 63989 | 1472592 | 67140 |
| HILIC_NEG_173_92599a7_355 | 2143285 | 282663 | 2141205 | 248504 | 2155809 | 240000 | 2126600 | 258328 |
| RP_POS_553_45697a11_256 | 18551932 | 4900896 | 20953882 | 6040895 | 20598875 | 5499826 | 21308889 | 6574517 |
| RP_POS_199_13043a11_65 | 4919353 | 1505190 | 5117495 | 1869575 | 5007500 | 1089336 | 5227490 | 2418804 |
| RP_POS_434_75906a6_111 | 28295 | 65070 | 412491 | 737427 | 455814 | 785472 | 369168 | 691302 |
| RP_POS_181_0493a7_494 | 1304624 | 110737 | 1313187 | 138169 | 1305076 | 134015 | 1321298 | 143097 |
| HILIC_POS_138_05505a4_254 | 51938899 | 63714489 | 46815563 | 72224917 | 51052654 | 75804338 | 42578472 | 68966651 |
| RP_NEG_299_25934a10_219 | 15168146 | 2069509 | 15565370 | 2613663 | 15412113 | 2486231 | 15718627 | 2751836 |
| HILIC_NEG_200_85867a4_112 | 3558995 | 1309972 | 3067811 | 1287088 | 2992410 | 1223548 | 3143211 | 1355849 |
| RP_NEG_379_15878a10_978 | 4906409 | 2539076 | 6393134 | 3792508 | 6615245 | 3753966 | 6171023 | 3855752 |
| RP_POS_517_37115a10_546 | 7360722 | 1349076 | 7531241 | 1229827 | 7603264 | 1189298 | 7459219 | 1277028 |
| RP_POS_702_86194a0_491 | 6308905 | 1056056 | 17136482 | 18574252 | 18224240 | 19864592 | 16048723 | 17321368 |
| RP_POS_353_03723a4_852 | 1041733 | 423963 | 1078451 | 467143 | 1105804 | 490559 | 1051098 | 445784 |
| RP_POS_216_92223a0_516 | 1667280 | 388166 | 2724190 | 2172857 | 2851373 | 2307586 | 2597007 | 2044751 |
| RP_POS_437_23682a6_111 | 7558 | 6840 | 372166 | 659437 | 410763 | 700678 | 333569 | 620160 |
| HILIC_NEG_117_91911a7_351 | 2466663 | 277909 | 2402132 | 394287 | 2425187 | 359623 | 2379078 | 428584 |
| RP_POS_431_38412a11_038 | 1037789 | 999942 | 1523238 | 1603584 | 1616995 | 1780484 | 1429480 | 1416820 |
| RP_POS_224_86421a0_519 | 348547 | 69398 | 275947 | 103820 | 269878 | 108505 | 282016 | 99642 |
| RP_POS_575_33826a15_435 | 632521 | 228879 | 622229 | 250387 | 636858 | 245340 | 607599 | 256980 |
| RP_POS_173_96153a0_251 | 2095788 | 355059 | 2106427 | 325382 | 2125402 | 307729 | 2087451 | 344202 |
| HILIC_NEG_157_86234a3_023 | 3745986 | 700625 | 3585681 | 758662 | 3629919 | 764903 | 3541444 | 757504 |
| RP_NEG_321_92273a6_867 | 1675872 | 184518 | 1622957 | 229531 | 1609577 | 260316 | 1636336 | 195741 |
| RP_POS_325_32242a10_585 | 2275185 | 855043 | 2512438 | 756777 | 2556532 | 685350 | 2468344 | 826704 |
| RP_POS_237_14659a7_809 | 2255941 | 255695 | 2270241 | 230239 | 2256848 | 210386 | 2283634 | 249946 |
| RP_POS_770_84924a0_49 | 4458927 | 822041 | 12700429 | 14163835 | 13524025 | 15117619 | 11876834 | 13243712 |
| HILIC_NEG_194_92735a7_667 | 6859004 | 525390 | 6954675 | 585753 | 6920631 | 588522 | 6988719 | 586935 |
| RP_POS_419_70483a7_55 | 4055644 | 261397 | 4026531 | 278444 | 4010354 | 288522 | 4042707 | 269936 |
| HILIC_NEG_134_89476a1_408 | 15687884 | 1006878 | 15654052 | 1837554 | 15547241 | 1431419 | 15760863 | 2179422 |
| RP_POS_532_43884a14_268 | 270642793 | 27892354 | 276967697 | 36543701 | 274842960 | 25958194 | 279092433 | 44889831 |
| RP_POS_172_97864a15_38 | 85174751 | 11391055 | 84930867 | 12297078 | 84217229 | 13373295 | 85644506 | 11208835 |
| RP_POS_680_38324a11_426 | 968570 | 162928 | 965811 | 172198 | 955823 | 174340 | 975799 | 171205 |
| HILIC_POS_137_04587a2_457 | 14241 | 17329 | 10443340 | 15455827 | 9546692 | 12458282 | 11339989 | 18049675 |
| RP_POS_184_17029a2_197 | 810627 | 47081 | 848387 | 146264 | 856884 | 193726 | 839890 | 74471 |
| RP_POS_838_83618a0_49 | 3706745 | 701748 | 11051508 | 12573514 | 11779673 | 13436109 | 10323343 | 11738901 |
| RP_POS_524_27405a10_326 | 3275951 | 1068304 | 4748244 | 1208978 | 4678291 | 1051323 | 4818198 | 1355662 |
| RP_POS_265_17914a8_529 | 984148 | 49602 | 992006 | 60761 | 988491 | 55713 | 995521 | 65803 |
| RP_POS_634_87494a0_493 | 6369906 | 1037929 | 15663044 | 16160683 | 16597890 | 17299621 | 14728197 | 15053400 |
| RP_POS_185_11479a13_013 | 6961375 | 1553001 | 7259318 | 1500142 | 7172569 | 1491641 | 7346067 | 1518694 |
| RP_POS_636_55511a14_987 | 1696044 | 912849 | 1105203 | 2025915 | 987837 | 1021008 | 1222569 | 2687351 |
| HILIC_POS_440_18939a0_947 | 1091552 | 694945 | 1040733 | 538530 | 1071859 | 658653 | 1009608 | 387488 |
| RP_POS_223_09395a11_629 | 8703874 | 1770887 | 8340180 | 1839208 | 8233883 | 1125936 | 8446478 | 2354489 |
| RP_NEG_397_00366a4_57 | 1917037 | 1535440 | 1105745 | 1360258 | 1184225 | 1215946 | 1027264 | 1499089 |
| RP_POS_570_35291a10_753 | 1744363 | 825079 | 2002284 | 852789 | 2051478 | 700645 | 1953091 | 986660 |
| RP_POS_562_79602a0_5 | 1669684 | 482712 | 1381970 | 972894 | 1438032 | 1054771 | 1325908 | 890733 |
| RP_POS_285_13071a8_608 | 686983 | 274590 | 804672 | 301661 | 822048 | 286221 | 787297 | 318305 |
| RP_NEG_197_80769a0_702 | 12736704 | 7004521 | 15837829 | 12988373 | 16585528 | 12778246 | 15090129 | 13282093 |
| HILIC_NEG_136_89166a4_83 | 10231134 | 4697818 | 12257703 | 4745125 | 11984586 | 4812022 | 12530820 | 4710005 |
| RP_NEG_588_8985a0_49 | 9671097 | 1425574 | 21219558 | 20283232 | 22387044 | 21824667 | 20052071 | 18764790 |
| RP_POS_844_54449a11_619 | 934572 | 883483 | 1021185 | 1498137 | 1107415 | 1665062 | 934956 | 1321752 |
| HILIC_POS_134_11755a1_649 | 1522440 | 469013 | 1115106 | 621951 | 1079340 | 621016 | 1150872 | 627111 |
| RP_POS_170_1176a4_742 | 1968681 | 1836273 | 1955641 | 2987549 | 1783692 | 1625072 | 2127590 | 3915590 |
| RP_POS_181_03862a15_342 | 18904576 | 6757305 | 19166422 | 6545889 | 19542477 | 6763263 | 18790366 | 6367218 |
| RP_POS_443_33426a10_657 | 56606156 | 4033035 | 56158072 | 3889488 | 55934704 | 4022390 | 56381441 | 3779357 |
| RP_POS_229_1192a7_341 | 2352458 | 196791 | 2250409 | 211269 | 2262538 | 207542 | 2238281 | 216347 |
| RP_NEG_514_8938a0_465 | 1423254 | 132382 | 975633 | 624269 | 939799 | 624224 | 1011467 | 628570 |
| RP_POS_266_04352a3_97 | 1351729 | 335159 | 1405448 | 514705 | 1434998 | 416302 | 1375898 | 600132 |
| HILIC_POS_374_15552a0_552 | 23386514 | 8167724 | 30946165 | 15908362 | 30033137 | 13717466 | 31859194 | 17928933 |
| HILIC_NEG_180_84201a1_366 | 11986967 | 2331203 | 11905017 | 3222172 | 12089789 | 2940764 | 11720244 | 3501272 |
| RP_POS_470_24081a6_222 | 30663 | 39546 | 1035930 | 1816398 | 1140058 | 1935555 | 931803 | 1702185 |
| HILIC_POS_138_09137a0_752 | 653445 | 86150 | 545090 | 95434 | 550558 | 101818 | 539623 | 89294 |
| RP_POS_441_29675a11_254 | 12368503 | 2810771 | 15336349 | 3379491 | 15143148 | 3107128 | 15529550 | 3653023 |
| RP_NEG_319_98462a3_961 | 1776171 | 448885 | 1816919 | 628365 | 1852833 | 507878 | 1781004 | 732919 |
| RP_POS_355_36877a11_247 | 506875 | 238373 | 1010732 | 515548 | 981281 | 398875 | 1040182 | 613297 |
| RP_NEG_344_85251a15_438 | 1149606 | 141453 | 1175623 | 78296 | 1171149 | 98415 | 1180097 | 51570 |

**Supplementary table 1: Metabolomics results** (in asceding order according to p-value from progressive ALD vs  non-progressive ALD comparison, with molecular features with ID_level of 1-2 first)

| | | | | | | | | all_ALD_vs_control | | | Prog_ALD_vs_non-prog_ALD | | | Prog_ALD_vs_control_ALD | | | Non-prog_ALD_vs_control | | | Healthy_control | | all_ALD | | Non-prog_ALD | | Progressive_ALD | |
|---|---|---|---|---|---|---|---|---|---|---|---|---|---|---|---|---|---|---|---|---|---|---|---|---|---|---|---|
| | | | | | | | | t-test | Cohen's | PLS-DA | t-test | Cohen's | PLS-DA | t-test | Cohen's | PLS-DA | t-test | Cohen's | PLS-DA | | | | | | | | |
| Feature_ID | RP_HILIC | POS_NEG | Average.Rt | Average.Mz | Metabolite.name | curated_MSMS | ID_level | p | d | VIP | p | d | VIP | p | d | VIP | p | d | VIP | Mean | SD | Mean | SD | Mean | SD | Mean | SD |
| RP_POS_198_18518a11_128 | RP | POS | 11.13 | 198.1852 | | | | 4.42E-02 | 0.34 | 0.72 | 5.71E-01 | -0.12 | 0.39 | 1.43E-01 | 0.28 | 0.60 | 5.48E-02 | 0.40 | 0.71 | 363434 | 304069 | 505993 | 443349 | 531298 | 476006 | 480687 | 411388 |
| HILIC_NEG_153_86867a3_022 | HILIC | NEG | 3.022 | 153.8687 | | | | 6.32E-01 | -0.09 | 0.95 | 5.71E-01 | -0.12 | 0.84 | 4.90E-01 | -0.15 | 0.26 | 8.82E-01 | -0.03 | 0.32 | 37369172 | 6825190 | 36675570 | 7865375 | 37123848 | 7883898 | 36227293 | 7900938 |
| RP_POS_511_78964a6_421 | RP | POS | 6.42 | 511.7896 | | | | 2.44E-07 | 0.62 | 1.31 | 5.72E-01 | -0.13 | 1.15 | 4.23E-04 | 0.55 | 1.42 | 1.90E-04 | 0.68 | 1.39 | 21964 | 30249 | 670328 | 1168394 | 736887 | 1251807 | 603770 | 1087238 |
| HILIC_POS_171_18555a6_962 | HILIC | POS | 6.962 | 171.1856 | | | | 7.46E-01 | 0.05 | 1.10 | 5.72E-01 | -0.13 | 0.75 | 9.35E-01 | -0.02 | 1.08 | 5.40E-01 | 0.11 | 1.07 | 6222887 | 1146858 | 6322859 | 2323186 | 6455146 | 2252066 | 6190572 | 2407700 |
| RP_POS_173_98438a0_253 | RP | POS | 0.25 | 173.9844 | | | | 7.97E-01 | 0.05 | 0.69 | 5.72E-01 | 0.11 | 0.38 | 6.29E-01 | 0.11 | 0.34 | 9.81E-01 | -0.01 | 0.45 | 873158 | 90690 | 877955 | 93082 | 872656 | 93423 | 883254 | 93382 |
| RP_NEG_524_27899a10_307 | RP | NEG | 10.31 | 524.2790 | | | | 1.90E-02 | 0.37 | 1.12 | 5.72E-01 | -0.12 | 0.36 | 9.89E-02 | 0.31 | 0.84 | 2.96E-02 | 0.43 | 1.01 | 18489676 | 5965638 | 22026643 | 10402387 | 22618638 | 10863424 | 21434647 | 9994922 |
| RP_POS_498_25931a10_052 | RP | POS | 10.05 | 498.2593 | | | | 1.66E-02 | 0.40 | 0.93 | 5.72E-01 | 0.12 | 0.92 | 3.60E-02 | 0.46 | 0.98 | 5.07E-02 | 0.34 | 0.96 | 342112 | 241276 | 478563 | 358610 | 458165 | 282273 | 498961 | 423437 |
| RP_NEG_690_83386a0_495 | RP | NEG | 0.50 | 690.8339 | | | | 1.27E-07 | -0.64 | 1.34 | 5.73E-01 | 0.13 | 1.19 | 3.97E-04 | -0.58 | 1.38 | 7.73E-05 | -0.71 | 1.45 | 1708447 | 107479 | 1285926 | 723924 | 1244811 | 752014 | 1327041 | 699894 |
| RP_POS_265_17734a9_625 | RP | POS | 9.63 | 265.1773 | | | | 8.93E-03 | 0.40 | 1.37 | 5.73E-01 | 0.12 | 1.46 | 9.22E-03 | 0.46 | 1.32 | 9.02E-02 | 0.34 | 1.12 | 1805381 | 630777 | 2236007 | 1167320 | 2169735 | 1277595 | 2302279 | 1054452 |
| HILIC_POS_332_09671a2_458 | HILIC | POS | 2.458 | 332.0967 | | | | 1.72E-05 | 0.52 | 1.04 | 5.73E-01 | 0.13 | 0.61 | 2.66E-03 | 0.58 | 0.97 | 1.35E-03 | 0.45 | 1.41 | 315173 | 60942 | 519873 | 442331 | 494757 | 367618 | 544989 | 508798 |
| HILIC_NEG_243_75554a1_358 | HILIC | NEG | 1.358 | 243.7555 | | | | 3.97E-07 | 0.63 | 1.16 | 5.73E-01 | 0.12 | 0.37 | 1.69E-04 | 0.69 | 1.21 | 4.18E-04 | 0.57 | 1.48 | 257098 | 32987 | 349144 | 161302 | 339989 | 150697 | 358300 | 172298 |
| RP_POS_468_724a6_453 | RP | POS | 6.45 | 468.7240 | | | | 9.92E-01 | 0.00 | 0.61 | 5.73E-01 | 0.12 | 0.44 | 7.36E-01 | 0.06 | 0.56 | 8.08E-01 | -0.06 | 0.44 | 498018 | 242670 | 498526 | 300607 | 481452 | 372988 | 515600 | 207023 |
| HILIC_POS_160_06046a7_687 | HILIC | POS | 7.687 | 160.0605 | | | | 8.10E-01 | -0.05 | 0.47 | 5.73E-01 | 0.12 | 0.49 | 9.53E-01 | 0.01 | 0.22 | 6.47E-01 | -0.10 | 0.20 | 1046809 | 66400 | 1043455 | 74847 | 1039210 | 82326 | 1047700 | 67113 |
| HILIC_POS_141_13869a3_025 | HILIC | POS | 3.025 | 141.1387 | | | | 9.01E-03 | 0.35 | 1.02 | 5.74E-01 | 0.13 | 0.73 | 1.98E-02 | 0.42 | 0.99 | 9.96E-02 | 0.29 | 1.07 | 3949093 | 263984 | 4175033 | 712586 | 4134680 | 713381 | 4215385 | 716718 |
| RP_POS_645_49579a11_466 | RP | POS | 11.47 | 645.4958 | | | | 1.46E-04 | -0.89 | 1.54 | 5.74E-01 | -0.10 | 0.44 | 1.28E-04 | -0.94 | 1.51 | 7.07E-04 | -0.84 | 1.34 | 626217 | 321420 | 367774 | 251011 | 381988 | 266874 | 353560 | 235933 |
| RP_POS_469_77896a6_393 | RP | POS | 6.39 | 469.7790 | | | | 1.54E-07 | 0.63 | 1.32 | 5.74E-01 | -0.13 | 1.16 | 5.01E-04 | 0.56 | 1.40 | 1.04E-04 | 0.69 | 1.43 | 20544 | 14363 | 494587 | 838685 | 542071 | 872953 | 447103 | 809007 |
| RP_POS_199_1304a14_168 | RP | POS | 14.17 | 199.1304 | | | | 7.29E-02 | 0.34 | 0.68 | 5.74E-01 | 0.12 | 0.37 | 6.76E-02 | 0.40 | 0.78 | 1.83E-01 | 0.29 | 0.86 | 28200890 | 14074557 | 33609745 | 16065183 | 32700715 | 15796919 | 34518775 | 16438319 |
| HILIC_POS_356_27933a0_654 | HILIC | POS | 0.654 | 356.2793 | | | | 7.38E-02 | -0.37 | 0.75 | 5.74E-01 | -0.11 | 0.71 | 7.79E-02 | -0.42 | 0.65 | 1.46E-01 | -0.31 | 0.80 | 2472368 | 1173895 | 2037996 | 1165782 | 2103970 | 988477 | 1972021 | 1326598 |
| RP_POS_531_38605a10_661 | RP | POS | 10.66 | 531.3861 | | | | 8.13E-01 | 0.05 | 0.32 | 5.75E-01 | 0.11 | 0.42 | 6.43E-01 | 0.11 | 0.28 | 9.89E-01 | 0.00 | 0.29 | 31384161 | 2322532 | 31493148 | 2061102 | 31376620 | 2297349 | 31609677 | 1810373 |
| RP_NEG_218_98579a6_853 | RP | NEG | 6.85 | 218.9858 | | | | 3.35E-01 | -0.14 | 0.44 | 5.75E-01 | -0.13 | 0.38 | 3.30E-01 | -0.20 | 0.35 | 6.16E-01 | -0.08 | 0.45 | 3153723 | 170561 | 3108714 | 353388 | 3128698 | 278798 | 3088730 | 416859 |
| HILIC_POS_87_09232a3_031 | HILIC | POS | 3.031 | 87.0923 | | | | 3.80E-03 | 0.45 | 1.30 | 5.75E-01 | -0.12 | 0.79 | 3.29E-02 | 0.39 | 1.38 | 9.40E-03 | 0.51 | 1.45 | 2876984 | 569890 | 3297044 | 993723 | 3353195 | 1044885 | 3240893 | 947051 |
| RP_POS_199_13075a13_943 | RP | POS | 13.94 | 199.1308 | | | | 2.39E-01 | 0.24 | 0.57 | 5.75E-01 | -0.11 | 0.50 | 4.20E-01 | 0.18 | 0.34 | 1.93E-01 | 0.30 | 0.70 | 22453238 | 9939232 | 24872537 | 10189187 | 25447754 | 10274161 | 24297320 | 10174683 |
| HILIC_POS_157_09726a4_871 | HILIC | POS | 4.871 | 157.0973 | | | | 5.30E-01 | 0.12 | 0.49 | 5.76E-01 | -0.12 | 0.57 | 3.81E-01 | 0.18 | 0.34 | 7.87E-01 | 0.06 | 0.14 | 2162162 | 909538 | 2282952 | 1032711 | 2224738 | 1174610 | 2341167 | 876426 |
| RP_NEG_499_08923a8_858 | RP | NEG | 8.86 | 499.0892 | | | | 7.02E-01 | -0.07 | 0.52 | 5.76E-01 | -0.12 | 0.34 | 5.60E-01 | -0.13 | 0.65 | 9.59E-01 | -0.01 | 0.28 | 983604 | 56683 | 978870 | 71502 | 982899 | 64027 | 974841 | 78720 |
| RP_NEG_311_16937a10_556 | RP | NEG | 10.56 | 311.1694 | | | | 2.25E-01 | 0.23 | 0.49 | 5.76E-01 | 0.12 | 0.41 | 1.95E-01 | 0.28 | 0.50 | 4.19E-01 | 0.17 | 0.56 | 6991018 | 4902196 | 8275774 | 5897764 | 7943604 | 5574798 | 8607944 | 6242881 |
| RP_POS_294_24265a9_231 | RP | POS | 9.23 | 294.2427 | Unknown | 294.2417(25), 71.0859(100), 99.0803(85), 179.1436(85), 236.8788(80), 250.8921(60) | 4 | 5.48E-01 | -0.12 | 0.84 | 5.76E-01 | 0.11 | 0.80 | 7.97E-01 | -0.06 | 0.55 | 3.91E-01 | -0.18 | 0.97 | 1099273 | 974143 | 977602 | 1043338 | 918817 | 837888 | 1036387 | 1220744 |
| RP_NEG_173_08138a5_102 | RP | NEG | 5.10 | 173.0814 | | | | 5.22E-01 | 0.13 | 0.68 | 5.77E-01 | 0.11 | 0.61 | 4.18E-01 | 0.19 | 0.52 | 7.44E-01 | 0.07 | 0.55 | 1598085 | 285502 | 1635670 | 293196 | 1619187 | 283726 | 1652153 | 304348 |
| RP_POS_163_03891a7_494 | RP | POS | 7.49 | 163.0389 | Unknown | 163.0379(100), 105.9346(55), 123.9451(40), 55.935(20) | 4 | 3.42E-01 | 0.19 | 0.59 | 5.77E-01 | 0.11 | 0.99 | 2.76E-01 | 0.25 | 0.72 | 5.44E-01 | 0.14 | 0.54 | 1209459 | 116562 | 1232298 | 119444 | 1225584 | 117122 | 1239012 | 122537 |
| RP_POS_375_28922a9_305 | RP | POS | 9.31 | 375.2892 | | | | 2.29E-01 | 0.21 | 0.91 | 5.77E-01 | -0.12 | 0.34 | 4.47E-01 | 0.15 | 0.58 | 2.03E-01 | 0.27 | 0.92 | 290903 | 224673 | 352112 | 310353 | 369549 | 329769 | 334675 | 291952 |
| RP_NEG_394_97269a10_108 | RP | NEG | 10.11 | 394.9727 | | | | 9.63E-02 | 0.28 | 0.77 | 5.77E-01 | -0.12 | 0.39 | 2.38E-01 | 0.22 | 0.56 | 1.06E-01 | 0.34 | 0.86 | 1111679 | 697062 | 1378922 | 1000033 | 1435111 | 1093977 | 1322732 | 904054 |
| RP_NEG_652_79449a0_499 | RP | NEG | 0.50 | 652.7945 | | | | 2.05E-02 | -0.33 | 1.12 | 5.77E-01 | -0.12 | 1.24 | 2.05E-02 | -0.39 | 1.16 | 1.71E-01 | -0.26 | 1.15 | 1704524 | 408295 | 1424283 | 950133 | 1477658 | 1042141 | 1370909 | 855603 |
| RP_POS_366_37207a12_166 | RP | POS | 12.17 | 366.3721 | | | | 6.49E-01 | -0.09 | 0.61 | 5.77E-01 | -0.11 | 0.34 | 5.08E-01 | -0.15 | 0.37 | 8.72E-01 | -0.04 | 0.42 | 5340150 | 3224378 | 5040471 | 3206482 | 5220573 | 3320617 | 4860368 | 3111301 |
| RP_NEG_297_24426a10_289 | RP | NEG | 10.29 | 297.2443 | | | | 8.48E-01 | 0.04 | 0.56 | 5.77E-01 | 0.12 | 0.61 | 6.67E-01 | 0.09 | 0.58 | 9.24E-01 | -0.02 | 0.49 | 1903439 | 319949 | 1916348 | 358622 | 1896207 | 354495 | 1936489 | 365171 |
| RP_POS_515_31146a10_501 | RP | POS | 10.50 | 515.3115 | | | | 5.55E-02 | -0.42 | 0.78 | 5.77E-01 | -0.11 | 1.16 | 4.54E-02 | -0.47 | 1.03 | 1.28E-01 | -0.37 | 0.68 | 1039505 | 363171 | 898682 | 319805 | 916638 | 332509 | 880725 | 308897 |
| HILIC_POS_446_0531a3_076 | HILIC | POS | 3.076 | 446.0531 | | | | 1.65E-02 | 0.32 | 1.20 | 5.77E-01 | -0.13 | 0.93 | 1.18E-01 | 0.25 | 1.28 | 4.37E-02 | 0.38 | 1.34 | 1208941 | 72984 | 1270928 | 220202 | 1283289 | 238438 | 1258567 | 201998 |
| RP_POS_487_36093a10_659 | RP | POS | 10.66 | 487.3609 | | | | 2.61E-01 | 0.24 | 0.63 | 5.78E-01 | 0.11 | 0.42 | 1.98E-01 | 0.29 | 0.48 | 4.49E-01 | 0.18 | 0.49 | 45198061 | 3372786 | 45969470 | 3245835 | 45787545 | 3489638 | 46151396 | 3006789 |
| RP_POS_199_01112a15_468 | RP | POS | 15.47 | 199.0111 | | | | 7.23E-01 | 0.07 | 0.39 | 5.78E-01 | 0.12 | 0.35 | 5.57E-01 | 0.13 | 0.34 | 9.64E-01 | 0.01 | 0.27 | 8592059 | 3005462 | 8816778 | 3417260 | 8625514 | 3576901 | 9008041 | 3274870 |
| HILIC_POS_387_21371a5_649 | HILIC | POS | 5.649 | 387.2137 | | | | 1.74E-01 | -0.26 | 1.06 | 5.79E-01 | -0.11 | 0.78 | 1.49E-01 | -0.32 | 0.79 | 3.40E-01 | -0.21 | 0.80 | 1664000 | 331141 | 1568655 | 369893 | 1589323 | 361283 | 1547986 | 380837 |
| RP_POS_153_00526a15_348 | RP | POS | 15.35 | 153.0053 | | | | 4.74E-01 | -0.15 | 0.31 | 5.79E-01 | 0.11 | 0.39 | 6.79E-01 | -0.10 | 0.35 | 3.72E-01 | -0.21 | 0.47 | 7499742 | 2759101 | 7103625 | 2531540 | 6962297 | 2446301 | 7244954 | 2631165 |
| RP_POS_280_88818a0_249 | RP | POS | 0.25 | 280.8882 | | | | 9.44E-01 | 0.01 | 0.40 | 5.79E-01 | 0.11 | 0.36 | 8.48E-01 | -0.04 | 0.30 | 7.77E-01 | 0.07 | 0.17 | 806290 | 84436 | 807502 | 87949 | 812411 | 90348 | 802594 | 86117 |
| RP_POS_263_23636a10_59 | RP | POS | 10.59 | 263.2364 | | | | 1.60E-04 | 0.63 | 1.08 | 5.80E-01 | -0.12 | 0.36 | 4.17E-03 | 0.57 | 0.99 | 4.02E-04 | 0.69 | 1.33 | 13996563 | 6486763 | 19981819 | 9831847 | 20530290 | 9518367 | 19433347 | 10202449 |
| RP_POS_146_97417a15_439 | RP | POS | 15.44 | 146.9742 | | | | 8.60E-01 | -0.03 | 0.40 | 5.80E-01 | -0.12 | 0.45 | 6.85E-01 | -0.09 | 0.35 | 9.08E-01 | 0.02 | 0.39 | 6665600 | 1761642 | 6599928 | 2029981 | 6713062 | 1879003 | 6486795 | 2183797 |
| HILIC_POS_184_09442a4_861 | HILIC | POS | 4.861 | 184.0944 | | | | 8.92E-03 | 0.41 | 0.88 | 5.80E-01 | 0.12 | 0.67 | 1.74E-02 | 0.47 | 0.79 | 6.04E-02 | 0.35 | 0.76 | 41411166 | 7916968 | 46705970 | 14046150 | 45923304 | 13513161 | 47488635 | 14654672 |
| RP_POS_228_06311a4_896 | RP | POS | 4.90 | 228.0631 | | | | 2.14E-02 | 0.33 | 0.69 | 5.80E-01 | 0.12 | 0.44 | 1.26E-01 | 0.27 | 0.61 | 3.68E-02 | 0.39 | 0.74 | 1065592 | 361592 | 1299666 | 773219 | 1342732 | 803102 | 1256601 | 747782 |
| RP_POS_666_82306a0_497 | RP | POS | 0.50 | 666.8231 | | | | 1.10E-08 | -0.69 | 1.38 | 5.80E-01 | 0.12 | 1.19 | 1.05E-04 | -0.63 | 1.45 | 3.02E-05 | -0.75 | 1.49 | 11571250 | 432372 | 8509257 | 4870118 | 8238142 | 5108459 | 8780372 | 4655510 |
| RP_POS_375_21887a11_608 | RP | POS | 11.61 | 375.2189 | | | | 9.56E-02 | -0.32 | 0.82 | 5.81E-01 | 0.11 | 0.76 | 1.80E-01 | -0.26 | 0.69 | 1.15E-01 | -0.37 | 0.90 | 1875975 | 158683 | 1819070 | 185076 | 1808765 | 222332 | 1829374 | 139847 |
| RP_POS_348_99115a8_122 | RP | POS | 8.12 | 348.9912 | | | | 8.05E-01 | -0.05 | 0.54 | 5.81E-01 | -0.11 | 0.34 | 6.48E-01 | -0.11 | 0.57 | 9.92E-01 | 0.00 | 0.61 | 1734422 | 523344 | 1708368 | 490625 | 1735651 | 485980 | 1681085 | 498641 |
| HILIC_POS_144_86832a3_498 | HILIC | POS | 3.498 | 144.8683 | | | | 9.72E-02 | 0.24 | 0.51 | 5.81E-01 | -0.12 | 0.54 | 1.84E-01 | 0.18 | 0.50 | 1.73E-01 | 0.30 | 0.50 | 385521 | 531363 | 629370 | 1115112 | 691390 | 1423887 | 567351 | 690668 |
| HILIC_POS_371_23972a5_785 | HILIC | POS | 5.785 | 371.2397 | | | | 5.56E-01 | -0.11 | 0.92 | 5.81E-01 | -0.12 | 0.88 | 4.46E-01 | -0.17 | 0.72 | 8.00E-01 | -0.05 | 0.55 | 8030481 | 1712581 | 7814928 | 2021646 | 7927224 | 1914884 | 7702632 | 2136585 |
| RP_POS_407_24023a10_377 | RP | POS | 10.38 | 407.2402 | | | | 5.13E-08 | 0.65 | 1.29 | 5.81E-01 | -0.12 | 1.09 | 5.99E-04 | 0.59 | 1.28 | 2.31E-05 | 0.71 | 1.48 | 111028 | 105758 | 1945281 | 3106033 | 2117759 | 3031737 | 1772804 | 3199942 |
| RP_POS_478_38864a13_012 | RP | POS | 13.01 | 478.3886 | | | | 3.68E-02 | -0.47 | 1.03 | 5.81E-01 | 0.10 | 0.35 | 9.29E-02 | -0.42 | 0.98 | 2.76E-02 | -0.52 | 0.90 | 4772160 | 1032742 | 4338228 | 858968 | 4290524 | 769667 | 4385932 | 945346 |
| RP_NEG_397_33371a11_345 | RP | NEG | 11.35 | 397.3337 | | | | 6.06E-02 | -0.34 | 0.84 | 5.81E-01 | 0.12 | 0.67 | 1.78E-01 | -0.28 | 0.82 | 5.92E-02 | -0.39 | 0.70 | 1642422 | 227166 | 1547160 | 296656 | 1530689 | 299321 | 1563630 | 296073 |
| RP_POS_419_35181a15_099 | RP | POS | 15.10 | 419.3518 | | | | 9.82E-01 | 0.00 | 0.57 | 5.82E-01 | 0.11 | 0.48 | 8.02E-01 | 0.06 | 0.38 | 8.39E-01 | -0.05 | 0.56 | 28669739 | 4207242 | 28688491 | 3742512 | 28480814 | 3928275 | 28896168 | 3574811 |
| RP_NEG_934_77313a0_494 | RP | NEG | 0.49 | 934.7731 | | | | 2.45E-09 | -0.74 | 1.40 | 5.82E-01 | 0.12 | 1.17 | 5.80E-05 | -0.68 | 1.46 | 7.13E-06 | -0.80 | 1.53 | 1540347 | 118628 | 1106741 | 637303 | 1071386 | 650517 | 1142097 | 628392 |
| RP_POS_422_88062a0_513 | RP | POS | 0.51 | 422.8806 | | | | 1.87E-06 | 0.58 | 1.28 | 5.82E-01 | -0.12 | 1.12 | 1.48E-03 | 0.52 | 1.26 | 3.25E-04 | 0.64 | 1.40 | 115228 | 41854 | 257569 | 273045 | 272716 | 284603 | 242422 | 262986 |
| RP_NEG_402_16299a11_611 | RP | NEG | 11.61 | 402.1630 | | | | 3.92E-02 | -0.39 | 0.78 | 5.82E-01 | 0.11 | 0.57 | 1.03E-01 | -0.33 | 0.73 | 4.87E-02 | -0.44 | 0.75 | 3403792 | 338328 | 3251320 | 407363 | 3228720 | 451013 | 3273920 | 361690 |
| RP_POS_536_36597a11_821 | RP | POS | 11.82 | 536.3660 | | | | 3.35E-01 | -0.17 | 0.46 | 5.82E-01 | 0.12 | 0.73 | 5.99E-01 | -0.11 | 0.28 | 2.69E-01 | -0.22 | 0.72 | 677630 | 53041 | 665973 | 75212 | 661804 | 75686 | 670143 | 75268 |
| RP_POS_540_3656a10_589 | RP | POS | 10.59 | 540.3656 | | | | 3.69E-01 | 0.16 | 1.03 | 5.82E-01 | -0.12 | 0.54 | 6.44E-01 | 0.10 | 0.74 | 2.58E-01 | 0.22 | 1.40 | 989355 | 362492 | 1061384 | 471750 | 1087538 | 406988 | 1035230 | 531603 |
| RP_NEG_328_75708a0_521 | RP | NEG | 0.52 | 328.7571 | | | | 1.10E-05 | 0.54 | 1.27 | 5.83E-01 | -0.12 | 1.16 | 2.48E-03 | 0.48 | 1.29 | 1.05E-03 | 0.60 | 1.38 | 500560 | 86870 | 727362 | 469032 | 753315 | 504273 | 701409 | 434518 |
| RP_POS_363_25272a11_149 | RP | POS | 11.15 | 363.2527 | | | | 9.22E-01 | -0.02 | 0.60 | 5.83E-01 | -0.12 | 0.73 | 6.99E-01 | -0.08 | 0.51 | 8.45E-01 | 0.04 | 0.66 | 2236029 | 77547 | 2234337 | 105482 | 2240172 | 113919 | 2228501 | 97125 |
| HILIC_NEG_99_9257a5_424 | HILIC | NEG | 5.424 | 99.9257 | | | | 2.27E-01 | -0.26 | 0.83 | 5.83E-01 | 0.11 | 0.42 | 3.77E-01 | -0.21 | 0.35 | 1.84E-01 | -0.32 | 0.85 | 4156026 | 821422 | 3957174 | 725630 | 3917040 | 724383 | 3997308 | 731987 |
| RP_POS_272_94431a0_469 | RP | POS | 0.47 | 272.9443 | | | | 4.50E-04 | -0.60 | 1.10 | 5.83E-01 | -0.11 | 0.99 | 1.28E-03 | -0.66 | 1.18 | 5.55E-03 | -0.55 | 1.15 | 570335 | 100573 | 486240 | 144475 | 494230 | 140775 | 478249 | 149077 |
| HILIC_NEG_283_70966a1_362 | HILIC | NEG | 1.362 | 283.7097 | | | | 1.88E-11 | 0.90 | 1.62 | 5.83E-01 | -0.12 | 0.31 | 1.55E-05 | 0.84 | 1.47 | 3.24E-09 | 0.95 | 2.16 | 627668 | 82836 | 836957 | 243727 | 850433 | 208018 | 823481 | 276363 |
| RP_NEG_308_99936a7_015 | RP | NEG | 7.02 | 308.9994 | | | | 1.14E-01 | 0.35 | 0.54 | 5.83E-01 | -0.11 | 0.39 | 2.17E-01 | 0.29 | 0.49 | 9.66E-02 | 0.40 | 0.66 | 4805170 | 2425411 | 5577887 | 2150962 | 5696664 | 2183214 | 5459110 | 2133654 |
| RP_POS_381_29709a11_171 | RP | POS | 11.17 | 381.2971 | | | | 4.63E-01 | 0.12 | 0.42 | 5.84E-01 | -0.12 | 0.46 | 3.91E-01 | 0.18 | 0.53 | 7.51E-01 | 0.06 | 0.45 | 1036952 | 775469 | 1175915 | 1291326 | 1104652 | 1152464 | 1247178 | 1424971 |
| HILIC_POS_302_19629a1_251 | HILIC | POS | 1.251 | 302.1963 | | | | 8.87E-03 | -0.59 | 1.00 | 5.84E-01 | 0.10 | 0.44 | 2.19E-02 | -0.54 | 0.93 | 1.04E-02 | -0.64 | 1.00 | 1452643 | 693892 | 1080874 | 588218 | 1048419 | 645756 | 1113329 | 529066 |
| RP_POS_497_3959a11_161 | RP | POS | 11.16 | 497.3959 | | | | 3.01E-01 | -0.19 | 0.57 | 5.84E-01 | 0.11 | 0.45 | 4.99E-01 | -0.14 | 0.45 | 2.68E-01 | -0.25 | 0.52 | 3571222 | 521585 | 3455237 | 621217 | 3420960 | 693668 | 3489514 | 544172 |
| RP_POS_666_33282a5_684 | RP | POS | 5.68 | 666.3328 | | | | 8.38E-01 | 0.04 | 0.66 | 5.85E-01 | 0.11 | 0.50 | 6.19E-01 | 0.10 | 0.61 | 9.42E-01 | -0.02 | 0.45 | 2438446 | 1194261 | 2490083 | 1352871 | 2415591 | 1651402 | 2564574 | 979500 |
| RP_POS_407_75516a13_72 | RP | POS | 13.72 | 407.7552 | | | | 5.08E-09 | 1.07 | 1.70 | 5.85E-01 | 0.10 | 0.44 | 1.60E-07 | 1.12 | 1.74 | 1.45E-07 | 1.02 | 1.77 | 13386265 | 3016294 | 17809433 | 3871477 | 17596421 | 3450120 | 18022444 | 4276287 |
| HILIC_POS_188_12817a0_549 | HILIC | POS | 0.549 | 188.1282 | | | | 4.17E-01 | -0.19 | 0.59 | 5.85E-01 | -0.10 | 0.60 | 3.51E-01 | -0.24 | 0.57 | 5.77E-01 | -0.13 | 0.37 | 1612557 | 1155986 | 1428765 | 929530 | 1479897 | 843065 | 1377633 | 1014687 |
| HILIC_NEG_84_9902a4_234 | HILIC | NEG | 4.234 | 84.9902 | | | | 2.39E-01 | -0.23 | 0.82 | 5.85E-01 | 0.11 | 0.77 | 4.33E-01 | -0.17 | 0.31 | 1.99E-01 | -0.28 | 0.74 | 1506168 | 397468 | 1406552 | 454494 | 1381567 | 464681 | 1431537 | 447373 |
| RP_POS_406_32831a11_061 | RP | POS | 11.06 | 406.3283 | | | | 9.79E-01 | 0.00 | 0.86 | 5.85E-01 | 0.12 | 0.82 | 7.79E-01 | 0.06 | 0.62 | 7.35E-01 | -0.06 | 0.82 | 3564864 | 309584 | 3562905 | 512428 | 3534751 | 494570 | 3591059 | 533203 |
| RP_NEG_402_14639a11_253 | RP | NEG | 11.25 | 402.1464 | | | | 8.49E-07 | 0.94 | 1.85 | 5.86E-01 | 0.10 | 1.20 | 6.62E-06 | 0.99 | 1.78 | 1.18E-05 | 0.88 | 1.77 | 428739 | 279842 | 755270 | 332513 | 737010 | 301545 | 773531 | 363011 |
| HILIC_NEG_197_80797a4_081 | HILIC | NEG | 4.081 | 197.8080 | | | | 5.27E-01 | 0.10 | 0.33 | 5.86E-01 | 0.12 | 0.87 | 3.85E-01 | 0.16 | 0.30 | 8.29E-01 | 0.04 | 0.61 | 413887 | 415662 | 476204 | 648870 | 440584 | 700059 | 511823 | 598319 |
| HILIC_POS_74_09716a4_186 | HILIC | POS | 4.186 | 74.0972 | | | | 9.88E-02 | 0.31 | 0.53 | 5.86E-01 | 0.11 | 0.60 | 1.06E-01 | 0.37 | 0.58 | 2.11E-01 | 0.25 | 0.55 | 7419324 | 3175911 | 8556680 | 3811610 | 8347489 | 3347452 | 8765870 | 4249530 |
| RP_POS_397_23096a11_609 | RP | POS | 11.61 | 397.2310 | | | | 4.95E-01 | -0.13 | 0.54 | 5.86E-01 | 0.11 | 0.59 | 7.19E-01 | -0.08 | 0.42 | 4.10E-01 | -0.19 | 0.64 | 2029740 | 359664 | 1977930 | 404732 | 1955730 | 444044 | 2000129 | 364387 |
| RP_NEG_207_96751a15_449 | RP | NEG | 15.45 | 207.9675 | | | | 7.40E-01 | -0.07 | 0.37 | 5.86E-01 | -0.11 | 0.46 | 5.81E-01 | -0.12 | 0.32 | 9.52E-01 | -0.01 | 0.36 | 8831198 | 297610 | 8811128 | 290492 | 8827058 | 313653 | 8795199 | 267578 |
| RP_NEG_620_84607a0_497 | RP | NEG | 0.50 | 620.8461 | | | | 5.63E-08 | -0.66 | 1.35 | 5.86E-01 | 0.12 | 1.20 | 2.02E-04 | -0.60 | 1.42 | 6.43E-05 | -0.72 | 1.46 | 13433106 | 827876 | 10139333 | 5464075 | 9839835 | 5753371 | 10438831 | 5199732 |
| RP_POS_247_2415a10_848 | RP | POS | 10.85 | 247.2415 | | | | 1.50E-08 | 0.88 | 1.37 | 5.86E-01 | -0.11 | 0.55 | 2.85E-05 | 0.82 | 1.35 | 2.23E-07 | 0.93 | 1.65 | 2631168 | 814379 | 3904857 | 1473745 | 3985623 | 1349034 | 3824090 | 1598426 |
| RP_POS_302_24524a11_252 | RP | POS | 11.25 | 302.2452 | | | | 1.41E-08 | 0.91 | 1.41 | 5.86E-01 | -0.11 | 0.59 | 1.75E-05 | 0.86 | 1.38 | 1.25E-07 | 0.97 | 1.65 | 1751283 | 870280 | 3070767 | 1446009 | 3149990 | 1311492 | 2991543 | 1578524 |
| RP_NEG_287_70569a15_59 | RP | NEG | 15.59 | 287.7057 | | | | 4.11E-01 | -0.15 | 0.60 | 5.86E-01 | -0.11 | 0.36 | 2.93E-01 | -0.21 | 0.53 | 6.74E-01 | -0.09 | 0.19 | 2436119 | 84759 | 2421020 | 103745 | 2426703 | 116965 | 2415336 | 89438 |
| HILIC_POS_371_23972a5_921 | HILIC | POS | 5.921 | 371.2397 | | | | 8.03E-01 | -0.05 | 0.83 | 5.87E-01 | 0.11 | 0.78 | 9.68E-01 | 0.01 | 0.47 | 6.26E-01 | -0.10 | 0.72 | 7339882 | 1823071 | 7243586 | 2091572 | 7129092 | 2018566 | 7358079 | 2176526 |
| RP_NEG_520_91077a0_492 | RP | NEG | 0.49 | 520.9108 | | | | 1.99E-07 | 0.62 | 1.34 | 5.87E-01 | -0.12 | 1.21 | 3.07E-04 | 0.56 | 1.41 | 1.90E-04 | 0.68 | 1.44 | 13551579 | 1769262 | 25704305 | 21571129 | 26882881 | 23277859 | 24525729 | 19885421 |
| RP_NEG_395_22052a10_907 | RP | NEG | 10.91 | 395.2205 | | | | 1.74E-03 | 0.45 | 1.52 | 5.87E-01 | 0.12 | 0.72 | 1.02E-02 | 0.51 | 1.22 | 1.86E-02 | 0.39 | 1.37 | 3651303 | 1409692 | 4928005 | 3104852 | 4758354 | 2738189 | 5097657 | 3452613 |
| HILIC_POS_176_12822a4_238 | HILIC | POS | 4.238 | 176.1282 | | | | 2.17E-02 | 0.39 | 0.61 | 5.88E-01 | -0.12 | 1.10 | 3.15E-02 | 0.45 | 0.75 | 8.54E-02 | 0.33 | 0.59 | 5040916 | 7287771 | 8960489 | 10658023 | 8378603 | 10025429 | 9542375 | 11327175 |
| RP_POS_842_56537a13_287 | RP | POS | 13.29 | 842.5654 | | | | 1.26E-08 | -1.37 | 2.59 | 5.88E-01 | -0.08 | 0.55 | 1.13E-08 | -1.41 | 2.41 | 4.75E-08 | -1.33 | 2.23 | 1011145 | 503429 | 333407 | 364333 | 353278 | 366794 | 313536 | 364477 |
| HILIC_POS_175_1189a6_962 | HILIC | POS | 6.962 | 175.1189 | | | | 5.51E-02 | 0.25 | 1.10 | 5.88E-01 | -0.12 | 0.80 | 2.69E-01 | 0.19 | 1.03 | 7.65E-02 | 0.31 | 1.18 | 26703129 | 8000501 | 32128600 | 24208206 | 33448651 | 24529786 | 30808549 | 24057595 |

**Supplementary table 1: Metabolomics results** (in asceding order according to p-value from progressive ALD vs  non-progressive ALD comparison, with molecular features with ID_level of 1-2 first)

| | | | | | | | | all_ALD_vs_control | | | Prog_ALD_vs_non-prog_ALD | | | Prog_ALD_vs_control_ALD | | | Non-prog_ALD_vs_control | | | Healthy_control | | all_ALD | | Non-prog_ALD | | Progressive_ALD | |
|---|---|---|---|---|---|---|---|---|---|---|---|---|---|---|---|---|---|---|---|---|---|---|---|---|---|---|---|
| | | | | | | | | t-test | Cohen's | PLS-DA | t-test | Cohen's | PLS-DA | t-test | Cohen's | PLS-DA | t-test | Cohen's | PLS-DA | | | | | | | | |
| Feature_ID | RP_HILIC | POS_NEG | Average.Rt | Average.Mz | Metabolite.name | curated_MSMS | ID_level | p | d | VIP | p | d | VIP | p | d | VIP | p | d | VIP | Mean | SD | Mean | SD | Mean | SD | Mean | SD |
| RP_NEG_873_56793a13_746 | RP | NEG | 13.75 | 873.5679 | | | | 8.04E-01 | 0.05 | 0.48 | 5.88E-01 | 0.11 | 0.38 | 6.48E-01 | 0.11 | 0.43 | 9.87E-01 | 0.00 | 0.53 | 3945867 | 1545194 | 4023969 | 1537510 | 3940145 | 1502817 | 4107793 | 1582188 |
| RP_POS_609_41461a13_499 | RP | POS | 13.50 | 609.4146 | | | | 8.08E-01 | 0.04 | 0.25 | 5.88E-01 | 0.12 | 0.36 | 6.62E-01 | 0.10 | 0.26 | 9.01E-01 | -0.02 | 0.19 | 13563357 | 2259044 | 13694512 | 3624942 | 13496714 | 2531100 | 13892310 | 4479092 |
| RP_POS_198_05247a5_353 | RP | POS | 5.35 | 198.0525 | | | | 5.98E-01 | -0.11 | 0.38 | 5.88E-01 | 0.11 | 0.51 | 8.14E-01 | -0.06 | 0.40 | 4.57E-01 | -0.17 | 0.63 | 2507312 | 1225451 | 2377166 | 1155161 | 2314183 | 992295 | 2440148 | 1305098 |
| RP_NEG_787_10187a9_549 | RP | NEG | 9.55 | 787.1019 | | | | 3.27E-01 | -0.21 | 0.53 | 5.88E-01 | 0.11 | 0.39 | 4.97E-01 | -0.16 | 0.37 | 2.73E-01 | -0.26 | 0.61 | 4528675 | 544336 | 4421214 | 501369 | 4393883 | 529817 | 4448544 | 475017 |
| HILIC_POS_394_31534a7_72 | HILIC | POS | 7.72 | 394.3153 | | | | 1.41E-01 | 0.26 | 0.66 | 5.89E-01 | 0.11 | 0.60 | 1.37E-01 | 0.32 | 0.59 | 3.02E-01 | 0.21 | 0.56 | 4053552 | 757978 | 4301863 | 992094 | 4247855 | 922686 | 4355870 | 1063620 |
| RP_POS_223_09409a10_573 | RP | POS | 10.57 | 223.0941 | | | | 4.94E-03 | -0.55 | 1.28 | 5.89E-01 | -0.11 | 0.56 | 9.13E-03 | -0.60 | 1.18 | 1.62E-02 | -0.49 | 1.01 | 8508296 | 1662367 | 7492250 | 1856459 | 7593164 | 1595150 | 7391336 | 2097124 |
| RP_POS_326_90259a15_377 | RP | POS | 15.38 | 326.9026 | | | | 9.77E-01 | -0.01 | 0.23 | 5.89E-01 | 0.09 | 0.36 | 9.01E-01 | 0.04 | 0.13 | 8.57E-01 | -0.05 | 0.37 | 1593448 | 143739 | 1592685 | 78183 | 1588437 | 76731 | 1596933 | 80159 |
| RP_POS_539_41333a10_453 | RP | POS | 10.45 | 539.4133 | | | | 8.18E-01 | -0.04 | 0.38 | 5.90E-01 | -0.12 | 0.63 | 5.82E-01 | -0.10 | 0.39 | 9.39E-01 | 0.02 | 0.59 | 1881371 | 184152 | 1871873 | 250628 | 1885492 | 303861 | 1858255 | 184934 |
| RP_POS_840_84283a0_494 | RP | POS | 0.49 | 840.8428 | | | | 4.44E-08 | 0.66 | 1.34 | 5.90E-01 | -0.12 | 1.17 | 1.55E-04 | 0.60 | 1.40 | 8.22E-05 | 0.72 | 1.44 | 65057 | 49991 | 419899 | 593851 | 452123 | 635140 | 387676 | 554069 |
| RP_POS_513_84259a14_974 | RP | POS | 14.97 | 513.8426 | | | | 5.44E-01 | -0.13 | 0.35 | 5.90E-01 | 0.11 | 0.39 | 7.52E-01 | -0.07 | 0.67 | 4.30E-01 | -0.18 | 0.32 | 220253 | 206511 | 194951 | 194662 | 184390 | 187828 | 205512 | 202616 |
| RP_POS_621_43829a11_254 | RP | POS | 11.25 | 621.4383 | | | | 3.62E-01 | 0.17 | 0.97 | 5.91E-01 | 0.11 | 0.75 | 3.16E-01 | 0.23 | 0.87 | 5.64E-01 | 0.12 | 1.02 | 748760 | 304954 | 807754 | 349203 | 788831 | 305796 | 826677 | 390042 |
| RP_POS_491_79153a6_487 | RP | POS | 6.49 | 491.7915 | | | | 9.68E-08 | 0.64 | 1.33 | 5.91E-01 | -0.12 | 1.17 | 3.58E-04 | 0.58 | 1.41 | 9.10E-05 | 0.70 | 1.43 | 10032 | 6432 | 282997 | 474152 | 308681 | 495125 | 257313 | 455777 |
| RP_POS_235_99771a1_711 | RP | POS | 1.71 | 235.9977 | | | | 1.96E-11 | 0.87 | 1.42 | 5.91E-01 | 0.11 | 0.40 | 5.41E-06 | 0.93 | 1.41 | 1.54E-08 | 0.82 | 1.67 | 1484441 | 554547 | 3037901 | 1871667 | 2936504 | 1442789 | 3139299 | 2230503 |
| RP_POS_830_56561a13_37 | RP | POS | 13.37 | 830.5656 | | | | 2.24E-02 | 0.46 | 1.05 | 5.91E-01 | -0.11 | 0.39 | 7.03E-02 | 0.41 | 0.77 | 2.14E-02 | 0.52 | 1.02 | 17187292 | 6402812 | 20270952 | 6629987 | 20629735 | 6539858 | 19912170 | 6765950 |
| RP_POS_329_26761a9_889 | RP | POS | 9.89 | 329.2676 | | | | 7.88E-01 | -0.06 | 0.51 | 5.91E-01 | 0.11 | 0.35 | 9.88E-01 | 0.00 | 0.18 | 6.42E-01 | -0.11 | 0.58 | 779023 | 146179 | 771100 | 137744 | 763647 | 143680 | 778553 | 132577 |
| HILIC_POS_606_36926a1_017 | HILIC | POS | 1.017 | 606.3693 | | | | 5.46E-06 | 0.55 | 1.23 | 5.91E-01 | -0.12 | 0.77 | 1.66E-03 | 0.49 | 1.42 | 8.82E-04 | 0.61 | 1.36 | 316899 | 938227 | 3478405 | 6405940 | 3824959 | 6928480 | 3131852 | 5887331 |
| RP_POS_312_15933a9_128 | RP | POS | 9.13 | 312.1593 | | | | 1.65E-01 | 0.24 | 0.55 | 5.92E-01 | -0.12 | 0.38 | 3.12E-01 | 0.18 | 0.66 | 1.89E-01 | 0.29 | 0.59 | 681543 | 1073363 | 1025518 | 1553701 | 1109520 | 1845904 | 941516 | 1206432 |
| RP_POS_170_02113a3_945 | RP | POS | 3.95 | 170.0211 | | | | 1.38E-02 | -0.63 | 1.14 | 5.92E-01 | 0.09 | 0.71 | 3.31E-02 | -0.59 | 0.85 | 9.72E-03 | -0.68 | 1.35 | 2442502 | 1900681 | 1521818 | 1213244 | 1456275 | 989874 | 1587361 | 1409023 |
| RP_POS_764_83142a0_472 | RP | POS | 0.47 | 764.8314 | | | | 3.72E-09 | -0.78 | 1.40 | 5.92E-01 | 0.11 | 1.12 | 4.09E-05 | -0.72 | 1.46 | 2.71E-06 | -0.84 | 1.54 | 501624 | 71076 | 344103 | 214903 | 332515 | 214675 | 355691 | 216678 |
| HILIC_NEG_199_80495a0_552 | HILIC | NEG | 0.552 | 199.8050 | | | | 5.19E-01 | 0.13 | 0.43 | 5.92E-01 | -0.11 | 0.68 | 7.23E-01 | 0.08 | 0.14 | 4.35E-01 | 0.19 | 0.62 | 832676 | 377707 | 882626 | 383672 | 903314 | 425935 | 861937 | 339292 |
| RP_POS_457_2706a15_421 | RP | POS | 15.42 | 457.2706 | | | | 3.48E-02 | 0.40 | 1.02 | 5.92E-01 | 0.11 | 0.39 | 4.07E-02 | 0.46 | 0.84 | 9.65E-02 | 0.35 | 1.16 | 2922001 | 1099370 | 3424782 | 1281955 | 3355668 | 1193913 | 3493896 | 1373018 |
| RP_POS_559_51898a10_589 | RP | POS | 10.59 | 559.5190 | | | | 8.96E-04 | 0.56 | 0.91 | 5.92E-01 | -0.11 | 0.45 | 1.54E-02 | 0.50 | 0.86 | 1.24E-03 | 0.61 | 1.15 | 4407983 | 3760631 | 7427097 | 5685866 | 7733561 | 5214479 | 7120632 | 6159176 |
| RP_NEG_326_98917a7_73 | RP | NEG | 7.73 | 326.9892 | | | | 1.96E-01 | -0.22 | 0.55 | 5.93E-01 | -0.12 | 0.36 | 1.53E-01 | -0.28 | 0.69 | 4.39E-01 | -0.16 | 0.43 | 7004525 | 1493678 | 6557326 | 2190101 | 6675361 | 2337356 | 6439292 | 2049221 |
| RP_NEG_297_24445a10_802 | RP | NEG | 10.80 | 297.2445 | | | | 5.12E-01 | -0.12 | 0.79 | 5.93E-01 | 0.11 | 0.83 | 7.64E-01 | -0.06 | 0.66 | 4.09E-01 | -0.18 | 0.81 | 5475935 | 695266 | 5376496 | 876832 | 5329285 | 898587 | 5423707 | 861019 |
| RP_POS_796_54559a11_628 | RP | POS | 11.63 | 796.5456 | | | | 4.18E-01 | 0.13 | 0.45 | 5.93E-01 | -0.12 | 0.53 | 7.15E-01 | 0.07 | 0.33 | 3.55E-01 | 0.18 | 0.58 | 5896671 | 8297575 | 7599254 | 14923870 | 8401840 | 15947977 | 6796667 | 13940465 |
| RP_POS_132_95819a0_253 | RP | POS | 0.25 | 132.9582 | | | | 4.59E-01 | 0.16 | 0.46 | 5.94E-01 | -0.11 | 0.56 | 6.56E-01 | 0.10 | 0.29 | 3.72E-01 | 0.21 | 0.37 | 74694239 | 7577325 | 75825763 | 7139470 | 76209483 | 7238250 | 75442043 | 7091654 |
| RP_POS_199_98743a11_166 | RP | POS | 11.17 | 199.9874 | | | | 4.51E-11 | 0.97 | 1.61 | 5.94E-01 | -0.11 | 0.85 | 4.06E-06 | 0.91 | 1.53 | 1.46E-09 | 1.02 | 1.87 | 316953 | 117161 | 551010 | 244211 | 564126 | 208964 | 537894 | 276546 |
| RP_POS_456_77225a6_222 | RP | POS | 6.22 | 456.7723 | | | | 2.30E-07 | 0.62 | 1.31 | 5.94E-01 | -0.12 | 1.14 | 3.64E-04 | 0.56 | 1.42 | 2.06E-04 | 0.68 | 1.38 | 8860 | 7204 | 294888 | 514459 | 322513 | 552901 | 267263 | 476950 |
| RP_POS_299_63199a10_302 | RP | POS | 10.30 | 299.6320 | | | | 4.59E-02 | 0.46 | 0.82 | 5.94E-01 | -0.10 | 0.75 | 1.09E-01 | 0.41 | 0.63 | 3.41E-02 | 0.51 | 0.84 | 500182 | 178355 | 571450 | 145260 | 579243 | 127224 | 563656 | 162238 |
| RP_POS_214_18015a10_278 | RP | POS | 10.28 | 214.1802 | | | | 1.31E-01 | 0.28 | 0.68 | 5.94E-01 | -0.11 | 0.47 | 3.37E-01 | 0.22 | 0.53 | 8.21E-02 | 0.33 | 0.70 | 445125 | 317039 | 550377 | 398811 | 571765 | 316528 | 528990 | 469279 |
| HILIC_NEG_157_96739a0_7 | HILIC | NEG | 0.7 | 157.9674 | | | | 2.84E-06 | 0.82 | 1.43 | 5.95E-01 | 0.11 | 0.87 | 1.84E-05 | 0.87 | 1.67 | 1.19E-04 | 0.76 | 1.36 | 63277 | 44443 | 114118 | 62094 | 110793 | 61621 | 117443 | 63010 |
| RP_NEG_500_27875a10_323 | RP | NEG | 10.32 | 500.2788 | | | | 3.83E-08 | 0.94 | 1.66 | 5.96E-01 | -0.11 | 0.54 | 2.98E-05 | 0.89 | 1.45 | 5.17E-08 | 0.99 | 1.84 | 20599978 | 5827792 | 28778251 | 8550104 | 29235431 | 6959608 | 28321071 | 9941720 |
| RP_POS_810_7843a0_496 | RP | POS | 0.50 | 810.7843 | | | | 4.62E-09 | -0.72 | 1.39 | 5.96E-01 | 0.12 | 1.18 | 7.83E-05 | -0.66 | 1.46 | 1.23E-05 | -0.78 | 1.51 | 1971551 | 124236 | 1456548 | 780603 | 1414835 | 800462 | 1498261 | 766046 |
| RP_NEG_270_9043a15_623 | RP | NEG | 15.62 | 270.9043 | | | | 3.28E-01 | -0.21 | 0.96 | 5.96E-01 | -0.10 | 0.38 | 2.69E-01 | -0.26 | 0.58 | 4.98E-01 | -0.16 | 0.65 | 904391 | 42924 | 895994 | 38297 | 898039 | 38208 | 893948 | 38663 |
| RP_POS_497_39651a11_607 | RP | POS | 11.61 | 497.3965 | | | | 1.30E-01 | -0.30 | 0.70 | 5.97E-01 | 0.11 | 0.37 | 2.51E-01 | -0.24 | 0.54 | 1.27E-01 | -0.35 | 0.61 | 7740263 | 1363592 | 7304255 | 1496259 | 7224544 | 1636212 | 7383967 | 1353925 |
| RP_POS_826_75793a0_498 | RP | POS | 0.50 | 826.7579 | | | | 5.88E-02 | -0.29 | 1.08 | 5.97E-01 | -0.12 | 1.17 | 5.50E-02 | -0.35 | 1.02 | 2.44E-01 | -0.23 | 1.09 | 896914 | 177065 | 809677 | 332182 | 827349 | 355234 | 792005 | 310022 |
| RP_POS_406_164a4_143 | RP | POS | 4.14 | 406.1640 | | | | 7.35E-05 | 0.69 | 1.09 | 5.97E-01 | 0.11 | 0.46 | 5.64E-05 | 0.74 | 1.49 | 3.58E-03 | 0.64 | 1.01 | 5305838 | 2072826 | 7272405 | 2910258 | 7117587 | 3390390 | 7427222 | 2359745 |
| RP_POS_487_2988a6_325 | RP | POS | 6.33 | 487.2988 | | | | 2.42E-06 | 0.57 | 1.29 | 5.98E-01 | -0.12 | 1.14 | 1.25E-03 | 0.51 | 1.38 | 5.63E-04 | 0.63 | 1.36 | 57341 | 109910 | 525307 | 918953 | 574154 | 983400 | 476460 | 856876 |
| RP_POS_721_50757a10_864 | RP | POS | 10.86 | 721.5076 | | | | 2.15E-01 | -0.22 | 0.54 | 5.98E-01 | 0.11 | 0.65 | 4.73E-01 | -0.16 | 0.47 | 1.37E-01 | -0.27 | 0.64 | 5168901 | 494071 | 5031472 | 667066 | 4996022 | 527169 | 5066921 | 786488 |
| RP_POS_172_77734a10_583 | RP | POS | 10.58 | 172.7773 | | | | 2.32E-03 | 0.56 | 0.90 | 5.98E-01 | -0.11 | 0.43 | 1.64E-02 | 0.51 | 0.86 | 3.31E-03 | 0.61 | 1.11 | 585977 | 280816 | 778766 | 350967 | 797394 | 345865 | 760139 | 358523 |
| RP_POS_548_50397a13_291 | RP | POS | 13.29 | 548.5040 | | | | 6.11E-04 | 0.65 | 1.13 | 5.99E-01 | -0.11 | 0.46 | 2.86E-03 | 0.60 | 1.13 | 1.80E-03 | 0.71 | 1.25 | 2101259 | 1075004 | 2924319 | 1251944 | 2990711 | 1406304 | 2857926 | 1086291 |
| RP_POS_355_65216a5_683 | RP | POS | 5.68 | 355.6522 | | | | 8.50E-01 | 0.04 | 0.68 | 5.99E-01 | 0.11 | 0.48 | 6.41E-01 | 0.09 | 0.62 | 9.39E-01 | -0.02 | 0.46 | 2434725 | 1149779 | 2480649 | 1302901 | 2411549 | 1574742 | 2549750 | 969634 |
| RP_POS_293_03442a5_353 | RP | POS | 5.35 | 293.0344 | | | | 6.78E-01 | -0.09 | 0.35 | 5.99E-01 | 0.10 | 0.52 | 8.77E-01 | -0.04 | 0.38 | 5.33E-01 | -0.14 | 0.60 | 564355 | 323881 | 537586 | 289007 | 522269 | 247528 | 552902 | 327117 |
| RP_POS_501_94785a6_786 | RP | POS | 6.79 | 501.9479 | | | | 1.37E-07 | 0.63 | 1.33 | 5.99E-01 | -0.12 | 1.17 | 3.59E-04 | 0.57 | 1.45 | 1.28E-04 | 0.69 | 1.41 | 10848 | 14432 | 1058558 | 1845199 | 1156308 | 1947257 | 960808 | 1751483 |
| HILIC_POS_147_14914a5_424 | HILIC | POS | 5.424 | 147.1491 | | | | 1.05E-02 | 0.48 | 0.99 | 5.99E-01 | 0.11 | 0.54 | 1.73E-02 | 0.53 | 0.92 | 3.59E-02 | 0.42 | 0.96 | 57882396 | 20403124 | 69423765 | 24811771 | 68109252 | 22199938 | 70738279 | 27339496 |
| RP_POS_412_84506a0_499 | RP | POS | 0.50 | 412.8451 | | | | 6.53E-03 | -0.37 | 1.15 | 5.99E-01 | -0.12 | 1.23 | 1.06E-02 | -0.43 | 1.19 | 9.75E-02 | -0.31 | 1.20 | 1722720 | 305323 | 1453623 | 809498 | 1496499 | 871772 | 1410747 | 748470 |
| RP_POS_752_04541a12_764 | RP | POS | 12.76 | 752.0454 | | | | 3.66E-02 | 0.41 | 0.70 | 5.99E-01 | 0.11 | 0.33 | 3.81E-02 | 0.46 | 0.81 | 1.01E-01 | 0.36 | 0.66 | 902369 | 442004 | 1099291 | 485164 | 1073613 | 473508 | 1124969 | 500017 |
| RP_POS_130_06499a4_897 | RP | POS | 4.90 | 130.0650 | | | | 1.26E-01 | 0.23 | 0.60 | 5.99E-01 | -0.12 | 0.43 | 3.45E-01 | 0.18 | 0.48 | 1.28E-01 | 0.29 | 0.59 | 1222512 | 311207 | 1345301 | 574685 | 1375696 | 586497 | 1314906 | 566922 |
| RP_POS_765_53284a10_863 | RP | POS | 10.86 | 765.5328 | | | | 1.66E-03 | -0.62 | 0.98 | 6.00E-01 | 0.10 | 0.37 | 1.14E-02 | -0.57 | 0.89 | 1.67E-03 | -0.67 | 1.07 | 3068932 | 439432 | 2767860 | 476149 | 2742686 | 439802 | 2793035 | 513170 |
| RP_POS_564_43835a11_638 | RP | POS | 11.64 | 564.4384 | | | | 6.58E-05 | -1.05 | 1.75 | 6.00E-01 | -0.08 | 0.63 | 5.62E-05 | -1.09 | 1.66 | 1.79E-04 | -1.01 | 1.56 | 875530 | 509682 | 449718 | 303324 | 465738 | 316058 | 433698 | 292357 |
| RP_POS_446_28693a10_457 | RP | POS | 10.46 | 446.2869 | | | | 1.75E-01 | -0.30 | 0.60 | 6.00E-01 | 0.10 | 0.36 | 2.81E-01 | -0.25 | 0.46 | 1.60E-01 | -0.35 | 0.62 | 937401 | 217670 | 878380 | 187981 | 868457 | 208312 | 888304 | 166734 |
| RP_POS_776_55725a14_095 | RP | POS | 14.10 | 776.5573 | | | | 1.64E-02 | -0.51 | 1.17 | 6.00E-01 | -0.10 | 0.32 | 2.11E-02 | -0.57 | 1.05 | 3.70E-02 | -0.46 | 0.91 | 2019683 | 1008365 | 1520115 | 930759 | 1569244 | 795095 | 1470986 | 1055084 |
| RP_POS_441_29727a10_854 | RP | POS | 10.85 | 441.2973 | | | | 5.65E-01 | -0.11 | 0.78 | 6.01E-01 | 0.11 | 0.77 | 8.01E-01 | -0.06 | 0.60 | 4.43E-01 | -0.16 | 0.79 | 3420967 | 1500264 | 3238608 | 1705530 | 3148677 | 1644589 | 3328538 | 1776472 |
| RP_POS_362_30292a11_128 | RP | POS | 11.13 | 362.3029 | | | | 1.43E-02 | 0.39 | 0.88 | 6.01E-01 | -0.11 | 0.40 | 5.66E-02 | 0.34 | 0.75 | 3.25E-02 | 0.45 | 0.84 | 295128 | 359714 | 513087 | 596385 | 544536 | 673934 | 481639 | 512253 |
| HILIC_POS_490_29001a1_416 | HILIC | POS | 1.416 | 490.2900 | | | | 5.38E-06 | 0.67 | 1.03 | 6.01E-01 | -0.11 | 0.33 | 1.02E-03 | 0.61 | 1.05 | 7.15E-05 | 0.73 | 1.32 | 321566 | 126337 | 485626 | 260117 | 499318 | 254135 | 471935 | 267836 |
| RP_POS_283_26297a10_648 | RP | POS | 10.65 | 283.2630 | | | | 8.29E-04 | -0.57 | 1.04 | 6.01E-01 | 0.11 | 0.71 | 7.32E-03 | -0.52 | 1.06 | 2.56E-03 | -0.63 | 1.05 | 5979848 | 656402 | 5458669 | 944858 | 5408935 | 1003372 | 5508403 | 889903 |
| HILIC_NEG_134_89473a4_671 | HILIC | NEG | 4.671 | 134.8947 | | | | 4.19E-01 | 0.17 | 0.53 | 6.01E-01 | 0.10 | 0.74 | 3.66E-01 | 0.22 | 0.78 | 5.93E-01 | 0.12 | 0.25 | 23673825 | 8660682 | 25099334 | 8434405 | 24655579 | 7059201 | 25543089 | 9669333 |
| RP_POS_489_77646a6_325 | RP | POS | 6.33 | 489.7765 | | | | 2.85E-07 | 0.61 | 1.31 | 6.02E-01 | -0.12 | 1.15 | 5.10E-04 | 0.55 | 1.41 | 1.87E-04 | 0.67 | 1.40 | 39205 | 73037 | 1090821 | 1905621 | 1190967 | 2013727 | 990675 | 1805914 |
| RP_POS_982_79895a0_494 | RP | POS | 0.49 | 982.7990 | | | | 5.46E-08 | 0.65 | 1.33 | 6.02E-01 | -0.12 | 1.13 | 2.33E-04 | 0.59 | 1.40 | 7.67E-05 | 0.71 | 1.44 | 55565 | 37662 | 725054 | 1136396 | 784758 | 1193756 | 665350 | 1084813 |
| RP_POS_198_86171a0_521 | RP | POS | 0.52 | 198.8617 | | | | 1.95E-05 | 0.53 | 1.24 | 6.02E-01 | -0.12 | 1.13 | 3.08E-03 | 0.47 | 1.24 | 1.42E-03 | 0.59 | 1.35 | 342690 | 64578 | 491839 | 315315 | 508402 | 339049 | 475275 | 292167 |
| RP_NEG_157_08632a7_16 | RP | NEG | 7.16 | 157.0863 | | | | 2.71E-02 | -0.57 | 0.96 | 6.02E-01 | -0.09 | 0.33 | 2.18E-02 | -0.62 | 0.97 | 5.30E-02 | -0.53 | 0.84 | 2856616 | 2092604 | 1954909 | 1313225 | 2023872 | 1405712 | 1885946 | 1224166 |
| RP_POS_301_21347a10_73 | RP | POS | 10.73 | 301.2135 | | | | 3.71E-03 | 0.46 | 1.31 | 6.02E-01 | -0.11 | 1.08 | 2.29E-02 | 0.41 | 1.14 | 1.18E-02 | 0.52 | 1.29 | 5945185 | 2807265 | 7976949 | 4670920 | 8222148 | 5161415 | 7731751 | 4161411 |
| RP_POS_342_85999a0_499 | RP | POS | 0.50 | 342.8600 | | | | 6.03E-03 | -0.37 | 1.17 | 6.02E-01 | -0.12 | 1.25 | 1.13E-02 | -0.42 | 1.22 | 9.52E-02 | -0.31 | 1.21 | 4156491 | 770815 | 3436117 | 2190842 | 3551039 | 2336896 | 3321196 | 2051705 |
| RP_POS_553_45856a12_131 | RP | POS | 12.13 | 553.4586 | | | | 4.89E-01 | 0.13 | 0.64 | 6.03E-01 | -0.11 | 0.34 | 7.11E-01 | 0.08 | 0.55 | 3.97E-01 | 0.19 | 0.53 | 12034239 | 5153059 | 12781988 | 5689688 | 13080313 | 5814754 | 12483663 | 5604708 |
| RP_POS_321_3158a11_668 | RP | POS | 11.67 | 321.3158 | | | | 9.41E-01 | -0.01 | 0.68 | 6.03E-01 | -0.11 | 0.31 | 7.63E-01 | -0.07 | 0.38 | 8.66E-01 | 0.04 | 0.44 | 1373520 | 807212 | 1361240 | 835492 | 1405038 | 845917 | 1317441 | 831173 |
| RP_POS_514_26715a6_421 | RP | POS | 6.42 | 514.2672 | | | | 1.09E-06 | 0.58 | 1.29 | 6.03E-01 | -0.12 | 1.14 | 8.82E-04 | 0.52 | 1.39 | 4.15E-04 | 0.64 | 1.37 | 50445 | 37192 | 447562 | 762216 | 487438 | 814610 | 407686 | 712018 |
| RP_POS_270_2063a7_55 | RP | POS | 7.55 | 270.2063 | | | | 7.03E-06 | 0.53 | 1.16 | 6.04E-01 | -0.12 | 1.01 | 3.05E-03 | 0.47 | 1.20 | 8.39E-04 | 0.59 | 1.24 | 36433 | 20858 | 394955 | 754902 | 434409 | 790458 | 355500 | 723453 |
| RP_POS_615_33655a14_265 | RP | POS | 14.27 | 615.3366 | | | | 4.22E-01 | 0.18 | 0.79 | 6.04E-01 | -0.10 | 0.31 | 5.97E-01 | 0.13 | 0.38 | 3.42E-01 | 0.23 | 0.91 | 11815813 | 2872649 | 12271432 | 2424792 | 12398020 | 2359035 | 12144843 | 2506287 |
| RP_NEG_720_78223a0_499 | RP | NEG | 0.50 | 720.7822 | | | | 1.23E-02 | -0.34 | 1.14 | 6.05E-01 | -0.12 | 1.25 | 1.64E-02 | -0.40 | 1.17 | 1.33E-01 | -0.28 | 1.18 | 2087241 | 446425 | 1732664 | 1151844 | 1792739 | 1249173 | 1672590 | 1054878 |
| RP_NEG_383_19125a11_612 | RP | NEG | 11.61 | 383.1913 | | | | 8.66E-02 | -0.29 | 0.85 | 6.05E-01 | -0.11 | 0.81 | 8.90E-02 | -0.34 | 0.74 | 2.43E-01 | -0.23 | 0.75 | 23305066 | 1662934 | 22633578 | 2517047 | 22764806 | 2492957 | 22502350 | 2559369 |
| RP_POS_704_86676a0_495 | RP | POS | 0.50 | 704.8668 | | | | 8.03E-08 | 0.65 | 1.34 | 6.05E-01 | -0.11 | 1.19 | 1.77E-04 | 0.59 | 1.41 | 1.25E-04 | 0.70 | 1.45 | 145209 | 62366 | 532930 | 662321 | 567439 | 713700 | 498420 | 611958 |
| RP_POS_211_16925a11_038 | RP | POS | 11.04 | 211.1693 | | | | 1.32E-02 | 0.39 | 0.79 | 6.05E-01 | -0.11 | 0.35 | 5.59E-02 | 0.34 | 0.70 | 3.03E-02 | 0.45 | 0.77 | 484715 | 536579 | 815379 | 900663 | 862308 | 1007338 | 768449 | 787222 |
| HILIC_POS_265_11816a1_903 | HILIC | POS | 1.903 | 265.1182 | | | | 2.54E-01 | -0.21 | 0.36 | 6.05E-01 | 0.11 | 0.61 | 5.29E-01 | -0.15 | 0.24 | 1.28E-01 | -0.26 | 0.81 | 7112752 | 5311000 | 5786036 | 6681329 | 5437458 | 3814962 | 6134615 | 8682709 |
| RP_POS_496_26852a9_952 | RP | POS | 9.95 | 496.2685 | | | | 2.60E-04 | 0.49 | 0.96 | 6.05E-01 | -0.11 | 0.34 | 1.10E-02 | 0.44 | 0.96 | 2.67E-03 | 0.55 | 0.97 | 197551 | 113030 | 334567 | 305000 | 350455 | 316357 | 318680 | 295553 |
| RP_POS_354_28528a9_039 | RP | POS | 9.04 | 354.2853 | | | | 5.45E-02 | -0.38 | 0.93 | 6.05E-01 | 0.10 | 0.96 | 1.26E-01 | -0.33 | 1.03 | 5.71E-02 | -0.44 | 0.79 | 1058843 | 55443 | 1036409 | 58590 | 1033358 | 62335 | 1039460 | 55054 |
| RP_NEG_159_10202a7_085 | RP | NEG | 7.09 | 159.1020 | | | | 2.07E-02 | -0.73 | 1.53 | 6.05E-01 | 0.06 | 0.31 | 2.75E-02 | -0.70 | 1.24 | 1.84E-02 | -0.76 | 1.17 | 1601622 | 1361355 | 1006437 | 466521 | 982163 | 505391 | 1030711 | 427904 |
| HILIC_POS_143_08165a5_339 | HILIC | POS | 5.339 | 143.0817 | | | | 9.09E-01 | -0.03 | 0.37 | 6.06E-01 | -0.09 | 0.55 | 7.68E-01 | -0.07 | 0.14 | 9.53E-01 | 0.02 | 0.51 | 2065161 | 3580388 | 1987687 | 2354765 | 2110194 | 2922693 | 1865181 | 1621801 |
| HILIC_POS_508_33984a1_286 | HILIC | POS | 1.286 | 508.3398 | | | | 1.03E-05 | 0.71 | 1.15 | 6.06E-01 | 0.11 | 0.64 | 2.19E-04 | 0.77 | 1.25 | 2.26E-04 | 0.66 | 1.35 | 1581778 | 470294 | 2113629 | 773961 | 2073430 | 680850 | 2153828 | 862208 |
| RP_POS_793_62677a11_038 | RP | POS | 11.04 | 793.6268 | | | | 1.15E-03 | 0.42 | 0.96 | 6.06E-01 | -0.12 | 0.35 | 7.89E-03 | 0.37 | 0.95 | 1.88E-02 | 0.48 | 0.86 | 915496 | 1240069 | 2413733 | 3935900 | 2618248 | 4735204 | 2209218 | 2965003 |
| RP_POS_478_29263a10_337 | RP | POS | 10.34 | 478.2926 | | | | 1.48E-02 | 0.45 | 1.10 | 6.06E-01 | -0.11 | 0.61 | 8.18E-02 | 0.39 | 0.91 | 9.69E-03 | 0.50 | 1.15 | 57548987 | 20008618 | 68396081 | 24968439 | 69692494 | 20352992 | 67099668 | 29015420 |
| RP_POS_440_27701a9_247 | RP | POS | 9.25 | 440.2770 | | | | 4.87E-01 | 0.11 | 0.58 | 6.06E-01 | 0.11 | 0.73 | 4.63E-01 | 0.17 | 0.46 | 7.24E-01 | 0.05 | 0.32 | 532320 | 604313 | 635360 | 1018219 | 582455 | 654327 | 688265 | 1288736 |
| RP_POS_244_19102a7_716 | RP | POS | 7.72 | 244.1910 | | | | 7.77E-01 | -0.06 | 0.45 | 6.07E-01 | 0.10 | 0.33 | 9.59E-01 | -0.01 | 0.35 | 6.25E-01 | -0.11 | 0.36 | 4214896 | 790111 | 4170589 | 678864 | 4135377 | 578992 | 4205802 | 770296 |
| RP_NEG_293_17679a8_346 | RP | NEG | 8.35 | 293.1768 | | | | 1.32E-01 | 0.24 | 0.73 | 6.07E-01 | -0.11 | 0.47 | 3.21E-01 | 0.18 | 0.58 | 1.47E-01 | 0.29 | 0.79 | 2008795 | 234632 | 2097012 | 406080 | 2118069 | 438593 | 2075956 | 374029 |
| HILIC_POS_116_07094a4_87 | HILIC | POS | 4.87 | 116.0709 | | | | 5.07E-01 | 0.13 | 0.45 | 6.07E-01 | 0.11 | 0.52 | 3.68E-01 | 0.18 | 0.37 | 7.53E-01 | 0.07 | 0.14 | 379179022 | 187232457 | 405424864 | 212704227 | 394392676 | 248136515 | 416457052 | 172015664 |
| RP_POS_120_94345a0_251 | RP | POS | 0.25 | 120.9435 | | | | 6.40E-01 | 0.10 | 0.38 | 6.07E-01 | 0.10 | 0.45 | 5.21E-01 | 0.15 | 0.47 | 8.25E-01 | 0.05 | 0.35 | 1166894 | 277492 | 1192565 | 237163 | 1180270 | 247085 | 1204861 | 228651 |
| RP_NEG_369_30145a10_991 | RP | NEG | 10.99 | 369.3015 | FA 22:1;2O | 369.2983(100), 307.2993(60), 351.2933(25) | 3 | 5.20E-02 | -0.39 | 0.77 | 6.07E-01 | 0.10 | 0.46 | 1.23E-01 | -0.34 | 0.71 | 5.22E-02 | -0.44 | 0.84 | 1645342 | 338486 | 1507626 | 349554 | 1489519 | 363424 | 1525733 | 337822 |
| RP_POS_553_45874a13_104 | RP | POS | 13.10 | 553.4587 | | | | 8.31E-01 | -0.04 | 0.53 | 6.07E-01 | 0.10 | 0.38 | 9.78E-01 | 0.01 | 0.54 | 6.73E-01 | -0.10 | 0.38 | 2868308 | 634297 | 2840989 | 604238 | 2809695 | 570737 | 2872284 | 640249 |
| RP_POS_130_08649a0_611 | RP | POS | 0.61 | 130.0865 | | | | 4.20E-01 | -0.14 | 0.92 | 6.07E-01 | -0.11 | 0.68 | 2.97E-01 | -0.20 | 0.82 | 6.98E-01 | -0.09 | 0.74 | 1094333 | 941865 | 923531 | 1290964 | 990328 | 1479660 | 856733 | 1081059 |

**Supplementary table 1: Metabolomics results** (in asceding order according to p-value from progressive ALD vs  non-progressive ALD comparison, with molecular features with ID_level of 1-2 first)

| | | | | | | | | all_ALD_vs_control | | | Prog_ALD_vs_non-prog_ALD | | | Prog_ALD_vs_control_ALD | | | Non-prog_ALD_vs_control | | | Healthy_control | | all_ALD | | Non-prog_ALD | | Progressive_ALD | |
|---|---|---|---|---|---|---|---|---|---|---|---|---|---|---|---|---|---|---|---|---|---|---|---|---|---|---|---|
| | | | | | | | | t-test | Cohen's | PLS-DA | t-test | Cohen's | PLS-DA | t-test | Cohen's | PLS-DA | t-test | Cohen's | PLS-DA | | | | | | | | |
| Feature_ID | RP_HILIC | POS_NEG | Average.Rt | Average.Mz | Metabolite.name | curated_MSMS | ID_level | p | d | VIP | p | d | VIP | p | d | VIP | p | d | VIP | Mean | SD | Mean | SD | Mean | SD | Mean | SD |
| RP_POS_569_36511a14_948 | RP | POS | 14.95 | 569.3651 | | | | 2.79E-02 | 0.30 | 0.71 | 6.08E-01 | 0.12 | 0.32 | 9.81E-02 | 0.36 | 0.63 | 5.86E-02 | 0.24 | 0.65 | 17373309 | 1891997 | 18678974 | 4822613 | 18429279 | 3090985 | 18928668 | 6108062 |
| RP_NEG_151_02534a0_71 | RP | NEG | 0.71 | 151.0253 | | | | 4.62E-03 | 0.33 | 1.04 | 6.08E-01 | 0.12 | 0.59 | 2.90E-02 | 0.39 | 1.11 | 7.58E-02 | 0.27 | 0.94 | 244908 | 82217 | 2663192 | 8343172 | 2231871 | 7745452 | 3094514 | 8959125 |
| RP_POS_199_08235a0_708 | RP | POS | 0.71 | 199.0824 | | | | 1.35E-03 | -0.73 | 1.33 | 6.08E-01 | -0.09 | 0.34 | 1.58E-03 | -0.77 | 1.41 | 4.55E-03 | -0.68 | 1.21 | 760859 | 528806 | 407677 | 442547 | 430543 | 433346 | 384811 | 454793 |
| RP_POS_566_88757a0_494 | RP | POS | 0.49 | 566.8876 | | | | 1.23E-07 | 0.63 | 1.34 | 6.08E-01 | -0.11 | 1.20 | 2.66E-04 | 0.58 | 1.41 | 1.41E-04 | 0.69 | 1.45 | 9134206 | 1328967 | 19764425 | 18540474 | 20722223 | 19795459 | 18806627 | 17343047 |
| HILIC_POS_173_12871a4_25 | HILIC | POS | 4.25 | 173.1287 | | | | 3.63E-01 | 0.15 | 0.96 | 6.08E-01 | 0.11 | 0.85 | 3.27E-01 | 0.21 | 0.52 | 6.01E-01 | 0.10 | 0.45 | 798350 | 249526 | 850867 | 368005 | 831862 | 326896 | 869872 | 407456 |
| RP_POS_304_26038a10_973 | RP | POS | 10.97 | 304.2604 | | | | 5.93E-03 | 0.34 | 0.66 | 6.08E-01 | -0.12 | 0.44 | 6.19E-02 | 0.28 | 0.65 | 3.39E-02 | 0.40 | 0.70 | 19136723 | 9121301 | 31463683 | 40961585 | 33578745 | 45519135 | 29348621 | 36177667 |
| RP_POS_509_31195a6_42 | RP | POS | 6.42 | 509.3120 | | | | 4.47E-07 | 0.60 | 1.30 | 6.08E-01 | -0.11 | 1.14 | 5.31E-04 | 0.54 | 1.40 | 2.75E-04 | 0.66 | 1.38 | 27760 | 24577 | 326936 | 552178 | 355439 | 590532 | 298432 | 515400 |
| HILIC_NEG_134_89481a1_329 | HILIC | NEG | 1.329 | 134.8948 | | | | 4.42E-06 | -0.82 | 1.24 | 6.08E-01 | 0.10 | 0.60 | 2.63E-04 | -0.77 | 1.23 | 9.80E-06 | -0.87 | 1.53 | 15716542 | 1978632 | 13531160 | 2648338 | 13394448 | 2422745 | 13667872 | 2874490 |
| RP_POS_278_24765a9_3 | RP | POS | 9.30 | 278.2477 | | | | 7.01E-01 | -0.10 | 0.70 | 6.08E-01 | -0.09 | 0.33 | 5.98E-01 | -0.14 | 0.48 | 8.46E-01 | -0.05 | 0.38 | 9543874 | 9451729 | 8852638 | 6292559 | 9177328 | 5980811 | 8527949 | 6634412 |
| RP_POS_302_245a10_586 | RP | POS | 10.59 | 302.2450 | | | | 1.10E-03 | 0.60 | 0.99 | 6.09E-01 | -0.10 | 0.45 | 1.04E-02 | 0.55 | 0.92 | 1.51E-03 | 0.65 | 1.20 | 112029017 | 47252342 | 147012075 | 59287858 | 150068412 | 56354523 | 143955738 | 62505520 |
| HILIC_POS_199_18039a0_854 | HILIC | POS | 0.854 | 199.1804 | | | | 7.14E-01 | -0.07 | 0.44 | 6.09E-01 | 0.11 | 0.51 | 9.49E-01 | -0.01 | 0.14 | 5.53E-01 | -0.12 | 0.31 | 4716494 | 1438295 | 4602679 | 1752145 | 4512366 | 1621915 | 4692992 | 1885582 |
| RP_POS_195_13745a7_726 | RP | POS | 7.73 | 195.1375 | | | | 3.39E-01 | -0.23 | 0.44 | 6.09E-01 | -0.09 | 0.59 | 3.02E-01 | -0.28 | 0.61 | 4.52E-01 | -0.19 | 0.54 | 1549831 | 144770 | 1523160 | 102661 | 1528451 | 82928 | 1517868 | 119831 |
| RP_NEG_500_84528a0_499 | RP | NEG | 0.50 | 500.8453 | | | | 2.77E-03 | -0.41 | 1.18 | 6.09E-01 | -0.11 | 1.26 | 6.09E-03 | -0.47 | 1.24 | 5.80E-02 | -0.35 | 1.21 | 3767178 | 724400 | 3081625 | 1845072 | 3176677 | 1966899 | 2986574 | 1729429 |
| RP_POS_601_26544a5_446 | RP | POS | 5.45 | 601.2654 | | | | 6.18E-01 | 0.08 | 0.64 | 6.09E-01 | 0.11 | 0.91 | 5.29E-01 | 0.13 | 0.45 | 9.10E-01 | 0.02 | 0.68 | 779186 | 412866 | 832824 | 787053 | 792268 | 635652 | 873380 | 918769 |
| HILIC_POS_258_1098a6_241 | HILIC | POS | 6.241 | 258.1098 | | | | 1.02E-09 | 0.83 | 1.33 | 6.09E-01 | -0.11 | 0.72 | 3.26E-05 | 0.78 | 1.29 | 2.53E-07 | 0.88 | 1.59 | 4251236 | 1575594 | 7605977 | 4254829 | 7825015 | 3960056 | 7386938 | 4560334 |
| RP_POS_233_11482a7_367 | RP | POS | 7.37 | 233.1148 | | | | 3.23E-01 | 0.18 | 0.43 | 6.09E-01 | 0.11 | 0.59 | 2.86E-01 | 0.24 | 0.39 | 5.21E-01 | 0.13 | 0.51 | 764970 | 881609 | 952786 | 1057436 | 898354 | 963130 | 1007218 | 1151301 |
| RP_NEG_229_14487a8_023 | RP | NEG | 8.02 | 229.1449 | | | | 2.63E-01 | 0.23 | 0.62 | 6.09E-01 | 0.10 | 0.40 | 2.19E-01 | 0.29 | 0.54 | 4.27E-01 | 0.18 | 0.59 | 2841291 | 528378 | 2961691 | 507886 | 2935552 | 507865 | 2987831 | 511703 |
| HILIC_NEG_141_92368a2_928 | HILIC | NEG | 2.928 | 141.9237 | | | | 3.10E-03 | 0.47 | 1.10 | 6.09E-01 | 0.11 | 0.83 | 9.26E-03 | 0.53 | 0.92 | 2.27E-02 | 0.42 | 0.90 | 1541555 | 320527 | 1778534 | 534903 | 1751006 | 495663 | 1806061 | 575198 |
| HILIC_NEG_134_89468a7_915 | HILIC | NEG | 7.915 | 134.8947 | | | | 6.93E-01 | 0.08 | 0.54 | 6.10E-01 | -0.10 | 0.70 | 8.97E-01 | 0.03 | 0.05 | 5.68E-01 | 0.13 | 0.65 | 19458662 | 6119782 | 19952342 | 6134226 | 20267667 | 6388465 | 19637017 | 5916925 |
| RP_POS_352_16562a4_669 | RP | POS | 4.67 | 352.1656 | | | | 4.10E-10 | 0.77 | 1.46 | 6.10E-01 | -0.11 | 1.08 | 1.24E-05 | 0.71 | 1.50 | 7.15E-06 | 0.82 | 1.54 | 1283192 | 319195 | 2909930 | 2301021 | 3028085 | 2438348 | 2791775 | 2173343 |
| HILIC_NEG_183_93419a5_882 | HILIC | NEG | 5.882 | 183.9342 | | | | 7.60E-01 | -0.06 | 0.68 | 6.10E-01 | 0.10 | 0.73 | 9.73E-01 | -0.01 | 0.31 | 5.80E-01 | -0.11 | 0.46 | 862116 | 166468 | 851530 | 178550 | 842358 | 140216 | 860701 | 211137 |
| RP_NEG_431_1955a11_616 | RP | NEG | 11.62 | 431.1955 | | | | 9.33E-01 | -0.02 | 0.32 | 6.10E-01 | 0.10 | 0.40 | 9.03E-01 | 0.03 | 0.32 | 7.68E-01 | -0.07 | 0.54 | 1196036 | 581355 | 1186339 | 499403 | 1160697 | 428512 | 1211982 | 564741 |
| RP_POS_476_27493a10_521 | RP | POS | 10.52 | 476.2749 | | | | 5.69E-01 | -0.10 | 0.90 | 6.10E-01 | 0.11 | 0.45 | 8.22E-01 | -0.05 | 0.75 | 4.50E-01 | -0.16 | 0.55 | 1498270 | 350908 | 1453913 | 464183 | 1430090 | 459905 | 1477736 | 471866 |
| HILIC_POS_262_16467a3_481 | HILIC | POS | 3.481 | 262.1647 | | | | 2.06E-04 | 0.47 | 0.89 | 6.10E-01 | 0.11 | 0.71 | 1.68E-02 | 0.53 | 0.86 | 7.35E-05 | 0.41 | 1.26 | 714073 | 287749 | 1169961 | 1078827 | 1114512 | 571712 | 1225409 | 1420689 |
| RP_POS_163_97687a15_549 | RP | POS | 15.55 | 163.9769 | | | | 8.61E-01 | -0.03 | 0.22 | 6.11E-01 | -0.10 | 0.46 | 6.88E-01 | -0.09 | 0.21 | 9.38E-01 | 0.02 | 0.49 | 96538501 | 1990901 | 96466084 | 2144931 | 96576034 | 2282280 | 96356134 | 2015410 |
| RP_POS_796_58258a13_312 | RP | POS | 13.31 | 796.5826 | | | | 9.59E-07 | -1.01 | 1.81 | 6.11E-01 | 0.09 | 0.61 | 3.85E-05 | -0.96 | 1.68 | 5.40E-07 | -1.06 | 1.85 | 7222814 | 2049384 | 4908546 | 2074045 | 4802222 | 1582827 | 5014871 | 2482479 |
| RP_POS_568_33716a10_59 | RP | POS | 10.59 | 568.3372 | | | | 3.20E-07 | 0.77 | 1.44 | 6.11E-01 | 0.11 | 0.71 | 2.25E-04 | 0.82 | 1.34 | 6.16E-07 | 0.71 | 1.72 | 839151 | 564307 | 1653708 | 1108897 | 1596839 | 684512 | 1710576 | 1417478 |
| RP_NEG_355_28546a10_81 | RP | NEG | 10.81 | 355.2855 | FA 21:1;2O | 355.2854(100), 293.2865(55), 337.2757(30) | 3 | 1.59E-01 | -0.30 | 0.55 | 6.11E-01 | 0.10 | 0.38 | 2.77E-01 | -0.25 | 0.56 | 1.45E-01 | -0.35 | 0.62 | 1566142 | 394419 | 1452985 | 374932 | 1433782 | 399174 | 1472189 | 352030 |
| RP_POS_273_20596a9_812 | RP | POS | 9.81 | 273.2060 | | | | 1.03E-02 | -0.57 | 0.99 | 6.11E-01 | -0.10 | 0.47 | 1.01E-02 | -0.61 | 1.08 | 2.99E-02 | -0.52 | 0.78 | 1016898 | 40468 | 995535 | 35556 | 997355 | 36115 | 993715 | 35260 |
| RP_NEG_221_94637a13_292 | RP | NEG | 13.29 | 221.9464 | | | | 1.73E-01 | -0.31 | 0.65 | 6.11E-01 | 0.10 | 0.74 | 3.01E-01 | -0.26 | 0.55 | 1.34E-01 | -0.36 | 0.64 | 12654162 | 1989084 | 12117018 | 1636456 | 12033272 | 1458127 | 12200763 | 1808365 |
| RP_NEG_442_16678a11_165 | RP | NEG | 11.17 | 442.1668 | | | | 3.98E-02 | 0.36 | 1.07 | 6.11E-01 | -0.11 | 1.11 | 1.21E-01 | 0.31 | 0.97 | 5.01E-02 | 0.42 | 1.16 | 1007507 | 92593 | 1050741 | 125737 | 1057175 | 133302 | 1044308 | 118695 |
| RP_POS_508_33963a10_584 | RP | POS | 10.58 | 508.3396 | | | | 8.80E-01 | -0.03 | 0.79 | 6.12E-01 | -0.10 | 1.10 | 7.18E-01 | -0.08 | 0.55 | 9.23E-01 | 0.02 | 0.48 | 1809066 | 789199 | 1784611 | 819519 | 1826524 | 807020 | 1742699 | 837892 |
| RP_POS_391_31787a11_229 | RP | POS | 11.23 | 391.3179 | | | | 8.41E-01 | 0.04 | 1.01 | 6.12E-01 | 0.11 | 1.03 | 6.62E-01 | 0.09 | 0.88 | 9.38E-01 | -0.02 | 0.95 | 1563997 | 428814 | 1582598 | 529043 | 1555541 | 556098 | 1609655 | 504725 |
| RP_NEG_502_85986a15_342 | RP | NEG | 15.34 | 502.8599 | | | | 5.07E-01 | -0.12 | 0.74 | 6.12E-01 | -0.11 | 0.43 | 3.97E-01 | -0.18 | 0.66 | 7.45E-01 | -0.07 | 0.44 | 1031735 | 36961 | 1026484 | 43794 | 1028722 | 46186 | 1024247 | 41613 |
| RP_POS_470_27228a6_325 | RP | POS | 6.33 | 470.2723 | | | | 1.47E-07 | 0.63 | 1.32 | 6.12E-01 | -0.11 | 1.14 | 2.97E-04 | 0.57 | 1.42 | 1.62E-04 | 0.68 | 1.39 | 7536 | 10822 | 465866 | 809507 | 507170 | 864604 | 424562 | 756937 |
| RP_NEG_353_89877a15_487 | RP | NEG | 15.49 | 353.8988 | | | | 4.41E-01 | 0.18 | 0.49 | 6.13E-01 | 0.09 | 0.62 | 3.50E-01 | 0.23 | 0.60 | 6.11E-01 | 0.13 | 0.41 | 1946614 | 235603 | 1982021 | 184486 | 1972631 | 206663 | 1991411 | 160859 |
| HILIC_POS_171_14915a0_543 | HILIC | POS | 0.543 | 171.1492 | | | | 3.45E-01 | -0.18 | 0.73 | 6.13E-01 | -0.10 | 0.66 | 2.86E-01 | -0.23 | 0.55 | 5.54E-01 | -0.13 | 0.53 | 4269476 | 1000956 | 4068895 | 1142501 | 4127020 | 1139283 | 4010770 | 1154295 |
| HILIC_POS_368_22012a5_92 | HILIC | POS | 5.92 | 368.2201 | | | | 1.54E-01 | -0.28 | 1.20 | 6.13E-01 | 0.10 | 0.78 | 3.11E-01 | -0.23 | 0.82 | 1.29E-01 | -0.33 | 1.29 | 617004 | 158501 | 569253 | 175307 | 560336 | 169755 | 578171 | 181969 |
| RP_POS_332_24313a6_463 | RP | POS | 6.46 | 332.2431 | | | | 6.22E-01 | 0.09 | 0.73 | 6.14E-01 | -0.11 | 0.80 | 8.63E-01 | 0.04 | 0.51 | 4.75E-01 | 0.14 | 0.86 | 1037636 | 490553 | 1089740 | 593170 | 1119905 | 530206 | 1059574 | 654145 |
| RP_POS_567_29852a6_22 | RP | POS | 6.22 | 567.2985 | | | | 5.26E-06 | 0.54 | 1.23 | 6.14E-01 | -0.11 | 1.09 | 1.65E-03 | 0.49 | 1.35 | 9.80E-04 | 0.60 | 1.28 | 46216 | 186094 | 937381 | 1826959 | 1030110 | 1973521 | 844653 | 1682660 |
| HILIC_POS_231_08374a2_368 | HILIC | POS | 2.368 | 231.0837 | | | | 8.69E-03 | 0.35 | 1.06 | 6.15E-01 | -0.11 | 0.70 | 7.50E-02 | 0.29 | 1.16 | 3.07E-02 | 0.40 | 1.16 | 461785 | 446948 | 868370 | 1305475 | 934577 | 1405462 | 802163 | 1207908 |
| RP_POS_174_9756a15_38 | RP | POS | 15.38 | 174.9756 | | | | 6.45E-01 | -0.08 | 0.30 | 6.15E-01 | 0.11 | 0.32 | 8.79E-01 | -0.03 | 0.17 | 5.30E-01 | -0.14 | 0.47 | 49702075 | 4943309 | 49207367 | 6163208 | 48894972 | 6607913 | 49519762 | 5734266 |
| RP_POS_798_56329a11_694 | RP | POS | 11.69 | 798.5633 | | | | 1.62E-01 | 0.19 | 0.57 | 6.15E-01 | -0.11 | 0.51 | 4.37E-01 | 0.13 | 0.38 | 1.78E-01 | 0.25 | 0.67 | 1281868 | 1703193 | 2035095 | 4430951 | 2259664 | 4611815 | 1810527 | 4277333 |
| RP_POS_211_1329a8_837 | RP | POS | 8.84 | 211.1329 | | | | 2.13E-01 | -0.29 | 0.87 | 6.15E-01 | -0.09 | 0.37 | 1.98E-01 | -0.33 | 0.56 | 3.13E-01 | -0.24 | 0.75 | 773618 | 796014 | 578596 | 637569 | 610896 | 530192 | 546296 | 733520 |
| RP_NEG_266_82684a0_771 | RP | NEG | 0.77 | 266.8268 | | | | 4.54E-10 | 0.93 | 1.44 | 6.15E-01 | -0.10 | 0.46 | 8.28E-06 | 0.88 | 1.38 | 1.40E-08 | 0.98 | 1.80 | 660192 | 962941 | 2426317 | 1928139 | 2523948 | 1699858 | 2328686 | 2145307 |
| RP_POS_774_56384a11_642 | RP | POS | 11.64 | 774.5638 | | | | 8.01E-01 | 0.04 | 0.36 | 6.15E-01 | -0.11 | 0.49 | 9.47E-01 | -0.01 | 0.24 | 6.50E-01 | 0.10 | 0.41 | 7535358 | 11004340 | 8181012 | 16502700 | 9015839 | 18388064 | 7346184 | 14515265 |
| RP_POS_301_23709a10_082 | RP | POS | 10.08 | 301.2371 | | | | 3.43E-05 | -0.95 | 1.48 | 6.16E-01 | -0.09 | 0.88 | 4.65E-05 | -0.99 | 1.53 | 1.49E-04 | -0.91 | 1.39 | 922090 | 91971 | 839695 | 75060 | 843491 | 73670 | 835899 | 76982 |
| RP_POS_174_14874a0_71 | RP | POS | 0.71 | 174.1487 | | | | 3.53E-01 | -0.19 | 0.66 | 6.16E-01 | 0.10 | 0.43 | 5.53E-01 | -0.14 | 0.59 | 2.85E-01 | -0.24 | 0.45 | 1921334 | 254309 | 1872501 | 264460 | 1859128 | 255397 | 1885874 | 275160 |
| RP_POS_294_11258a8_874 | RP | POS | 8.87 | 294.1126 | | | | 5.37E-01 | -0.13 | 0.54 | 6.16E-01 | -0.10 | 0.37 | 4.45E-01 | -0.18 | 0.47 | 7.38E-01 | -0.07 | 0.27 | 619737 | 108317 | 606021 | 109757 | 611569 | 105615 | 600473 | 114551 |
| HILIC_POS_138_05255a4_873 | HILIC | POS | 4.873 | 138.0526 | | | | 9.16E-01 | 0.02 | 0.37 | 6.16E-01 | 0.10 | 0.50 | 7.16E-01 | 0.07 | 0.20 | 9.05E-01 | -0.03 | 0.10 | 817489 | 500864 | 828317 | 514307 | 802295 | 645119 | 854339 | 341850 |
| RP_POS_141_50729a11_249 | RP | POS | 11.25 | 141.5073 | | | | 3.94E-15 | 1.18 | 1.80 | 6.16E-01 | 0.10 | 0.61 | 3.26E-10 | 1.23 | 1.90 | 5.65E-11 | 1.14 | 1.93 | 757130 | 376026 | 1666912 | 730218 | 1630023 | 664651 | 1703800 | 795474 |
| RP_POS_356_90875a0_47 | RP | POS | 0.47 | 356.9088 | | | | 2.26E-09 | -0.74 | 1.40 | 6.16E-01 | 0.11 | 1.16 | 6.42E-05 | -0.68 | 1.45 | 5.80E-06 | -0.79 | 1.53 | 1686251 | 137793 | 1178315 | 744981 | 1140688 | 747752 | 1215941 | 747862 |
| HILIC_POS_64_01668a4_872 | HILIC | POS | 4.872 | 64.0167 | | | | 9.97E-01 | 0.00 | 0.72 | 6.17E-01 | 0.11 | 0.71 | 8.11E-01 | 0.05 | 0.12 | 7.98E-01 | -0.05 | 0.35 | 7487108 | 1533147 | 7488458 | 1860259 | 7394709 | 1664844 | 7582207 | 2049905 |
| RP_NEG_370_6756a15_577 | RP | NEG | 15.58 | 370.6756 | | | | 2.10E-01 | -0.25 | 0.82 | 6.17E-01 | -0.10 | 0.35 | 1.74E-01 | -0.30 | 0.80 | 3.75E-01 | -0.20 | 0.55 | 1509347 | 58205 | 1494247 | 60024 | 1497271 | 61994 | 1491223 | 58457 |
| RP_NEG_506_32407a10_62 | RP | NEG | 10.62 | 506.3241 | | | | 6.83E-03 | 0.60 | 1.34 | 6.17E-01 | -0.09 | 1.00 | 2.62E-02 | 0.56 | 1.11 | 4.77E-03 | 0.65 | 1.35 | 3105040 | 921605 | 3617746 | 790445 | 3657548 | 651341 | 3577944 | 913718 |
| RP_POS_431_1785a12_339 | RP | POS | 12.34 | 431.1785 | | | | 2.34E-01 | 0.19 | 0.50 | 6.18E-01 | 0.11 | 0.34 | 2.56E-01 | 0.25 | 0.52 | 4.17E-01 | 0.14 | 0.48 | 8778741 | 1207309 | 9124040 | 1936563 | 9026610 | 1528701 | 9221470 | 2284904 |
| HILIC_NEG_178_84396a1_366 | HILIC | NEG | 1.366 | 178.8440 | | | | 8.70E-01 | -0.03 | 0.89 | 6.18E-01 | -0.11 | 0.80 | 7.02E-01 | -0.08 | 0.13 | 8.96E-01 | 0.03 | 0.18 | 12442341 | 2419229 | 12353282 | 3351196 | 12521767 | 3058330 | 12184797 | 3644025 |
| RP_POS_533_80267a6_511 | RP | POS | 6.51 | 533.8027 | | | | 2.11E-07 | 0.62 | 1.32 | 6.18E-01 | -0.11 | 1.16 | 3.79E-04 | 0.56 | 1.42 | 1.78E-04 | 0.68 | 1.40 | 25923 | 38142 | 401344 | 670503 | 435031 | 712014 | 367658 | 631699 |
| HILIC_POS_212_14926a0_619 | HILIC | POS | 0.619 | 212.1493 | | | | 7.34E-01 | -0.06 | 1.03 | 6.18E-01 | -0.10 | 0.89 | 5.83E-01 | -0.12 | 0.49 | 9.56E-01 | -0.01 | 0.22 | 1278313 | 652719 | 1231077 | 763460 | 1269418 | 797152 | 1192735 | 734294 |
| RP_POS_567_58173a11_166 | RP | POS | 11.17 | 567.5817 | | | | 2.80E-07 | 0.64 | 1.08 | 6.18E-01 | 0.11 | 0.90 | 9.81E-04 | 0.69 | 1.12 | 2.36E-06 | 0.58 | 1.38 | 515508 | 444276 | 1753503 | 2134529 | 1646221 | 1434842 | 1860785 | 2668923 |
| RP_POS_490_29028a9_952 | RP | POS | 9.95 | 490.2903 | LPC 14:0 [M+Na+] | 104.1076(100), 431.2133(60), 490.2904(30), 86.097(17), 285.2434(12) | | 4.49E-04 | 0.50 | 1.00 | 6.18E-01 | -0.11 | 0.39 | 1.62E-02 | 0.45 | 0.94 | 1.77E-03 | 0.56 | 1.01 | 6905894 | 2576494 | 9540075 | 5691611 | 9825850 | 5492603 | 9254300 | 5925760 |
| HILIC_POS_201_15968a0_528 | HILIC | POS | 0.528 | 201.1597 | | | | 3.37E-02 | 0.36 | 0.58 | 6.19E-01 | -0.11 | 0.39 | 1.10E-01 | 0.31 | 0.57 | 4.70E-02 | 0.42 | 0.69 | 237988 | 347356 | 409567 | 502302 | 434726 | 535702 | 384408 | 470633 |
| RP_POS_248_16789a6_845 | RP | POS | 6.85 | 248.1679 | | | | 2.24E-03 | 0.59 | 1.39 | 6.19E-01 | 0.10 | 0.90 | 5.06E-03 | 0.64 | 1.25 | 8.18E-03 | 0.54 | 1.24 | 514786 | 270607 | 696526 | 307377 | 681146 | 267661 | 711905 | 344621 |
| RP_POS_792_08875a14_251 | RP | POS | 14.25 | 792.0888 | | | | 1.51E-01 | 0.18 | 0.54 | 6.20E-01 | 0.11 | 0.44 | 2.42E-01 | 0.24 | 0.51 | 3.41E-01 | 0.13 | 0.50 | 908739 | 688418 | 1312869 | 2518580 | 1186971 | 1861226 | 1438768 | 3052775 |
| RP_POS_128_95074a0_813 | RP | POS | 0.81 | 128.9507 | | | | 8.40E-03 | 0.47 | 0.85 | 6.20E-01 | 0.10 | 0.34 | 1.42E-02 | 0.52 | 0.94 | 3.71E-02 | 0.42 | 0.89 | 4320639 | 2556042 | 5857353 | 3415780 | 5686757 | 3243644 | 6027949 | 3604526 |
| RP_NEG_798_80322a0_497 | RP | NEG | 0.50 | 798.8032 | | | | 5.92E-08 | -0.68 | 1.36 | 6.20E-01 | 0.11 | 1.19 | 1.62E-04 | -0.63 | 1.43 | 4.97E-05 | -0.73 | 1.45 | 2157117 | 200027 | 1614403 | 871073 | 1570902 | 909385 | 1657905 | 837961 |
| HILIC_NEG_225_06168a4_984 | HILIC | NEG | 4.984 | 225.0617 | | | | 1.29E-01 | -0.26 | 0.74 | 6.20E-01 | 0.11 | 0.49 | 3.23E-01 | -0.21 | 0.47 | 1.01E-01 | -0.32 | 1.09 | 9524290 | 3958605 | 8162660 | 5440183 | 7891302 | 4900988 | 8434018 | 5968687 |
| HILIC_POS_113_55412a0_505 | HILIC | POS | 0.505 | 113.5541 | | | | 6.41E-01 | -0.08 | 0.84 | 6.20E-01 | -0.11 | 0.50 | 4.86E-01 | -0.14 | 0.67 | 8.87E-01 | -0.03 | 0.87 | 291542 | 217114 | 269345 | 278389 | 283231 | 311740 | 255459 | 242914 |
| RP_POS_286_22876a10_857 | RP | POS | 10.86 | 286.2288 | | | | 7.95E-04 | -0.85 | 1.34 | 6.20E-01 | 0.08 | 0.47 | 2.79E-03 | -0.81 | 1.15 | 5.40E-04 | -0.89 | 1.37 | 794495 | 603962 | 380835 | 397956 | 360982 | 314879 | 400687 | 469058 |
| RP_POS_336_32669a10_453 | RP | POS | 10.45 | 336.3267 | | | | 9.55E-01 | -0.01 | 0.67 | 6.21E-01 | -0.10 | 0.45 | 7.91E-01 | -0.06 | 0.63 | 8.76E-01 | 0.04 | 0.58 | 1061209 | 347481 | 1057304 | 323734 | 1073438 | 339853 | 1041170 | 309378 |
| RP_NEG_512_26947a8_188 | RP | NEG | 8.19 | 512.2695 | | | | 2.62E-01 | -0.31 | 0.99 | 6.21E-01 | 0.08 | 0.38 | 3.49E-01 | -0.27 | 0.68 | 2.22E-01 | -0.35 | 0.91 | 1024342 | 1654092 | 675719 | 899842 | 630917 | 872643 | 720521 | 932928 |
| RP_POS_593_56909a11_262 | RP | POS | 11.26 | 593.5691 | | | | 3.88E-02 | -0.46 | 0.88 | 6.21E-01 | 0.09 | 0.35 | 8.97E-02 | -0.41 | 0.72 | 3.35E-02 | -0.51 | 0.96 | 361349 | 199907 | 277895 | 170484 | 269408 | 162872 | 286382 | 179020 |
| RP_POS_261_01031a0_548 | RP | POS | 0.55 | 261.0103 | | | | 2.20E-04 | 0.46 | 1.22 | 6.21E-01 | -0.11 | 1.17 | 1.34E-02 | 0.40 | 1.21 | 4.06E-03 | 0.51 | 1.33 | 2802336 | 277694 | 3304525 | 1224306 | 3365469 | 1282680 | 3243580 | 1172857 |
| RP_NEG_170_83275a15_349 | RP | NEG | 15.35 | 170.8328 | | | | 7.30E-01 | -0.08 | 0.34 | 6.21E-01 | 0.09 | 0.45 | 8.86E-01 | -0.04 | 0.24 | 6.16E-01 | -0.13 | 0.37 | 1084362 | 152308 | 1074211 | 113172 | 1068579 | 113170 | 1079842 | 114040 |
| RP_POS_276_22971a10_422 | RP | POS | 10.42 | 276.2297 | | | | 4.55E-05 | 0.66 | 1.11 | 6.21E-01 | -0.10 | 0.67 | 3.37E-03 | 0.61 | 1.04 | 7.60E-05 | 0.71 | 1.35 | 4099628 | 1483438 | 5636274 | 2436308 | 5757432 | 2108757 | 5515117 | 2741443 |
| RP_POS_577_13269a9_666 | RP | POS | 9.67 | 577.1327 | | | | 2.26E-02 | -0.43 | 0.71 | 6.22E-01 | 0.10 | 0.38 | 8.92E-02 | -0.38 | 0.63 | 1.89E-02 | -0.48 | 0.84 | 2997883 | 109638 | 2943426 | 128758 | 2937026 | 115019 | 2949826 | 142066 |
| RP_NEG_350_01294a6_867 | RP | NEG | 6.87 | 350.0129 | | | | 7.33E-01 | -0.07 | 0.36 | 6.22E-01 | -0.10 | 0.30 | 5.92E-01 | -0.12 | 0.28 | 9.39E-01 | -0.02 | 0.18 | 1320051 | 114818 | 1311913 | 122430 | 1317992 | 126121 | 1305835 | 119591 |
| RP_NEG_264_83023a0_771 | RP | NEG | 0.77 | 264.8302 | | | | 8.12E-10 | 0.93 | 1.44 | 6.22E-01 | -0.10 | 0.45 | 9.97E-06 | 0.88 | 1.37 | 1.87E-08 | 0.98 | 1.80 | 1558664 | 2232963 | 5534600 | 4357910 | 5750833 | 3828845 | 5318367 | 4859513 |
| RP_NEG_306_00504a3_953 | RP | NEG | 3.95 | 306.0050 | | | | 5.31E-01 | -0.11 | 0.77 | 6.22E-01 | -0.11 | 0.35 | 4.44E-01 | -0.16 | 0.67 | 7.85E-01 | -0.05 | 0.32 | 944337 | 233915 | 910606 | 341582 | 927552 | 321375 | 893660 | 363138 |
| RP_POS_696_37738a10_562 | RP | POS | 10.56 | 696.3774 | | | | 1.33E-03 | -0.60 | 0.95 | 6.22E-01 | 0.10 | 0.60 | 1.49E-02 | -0.55 | 0.83 | 1.05E-03 | -0.65 | 1.16 | 1335942 | 161801 | 1219235 | 197424 | 1209438 | 165125 | 1229032 | 226463 |
| RP_POS_219_03145a15_441 | RP | POS | 15.44 | 219.0315 | | | | 8.00E-01 | 0.05 | 0.37 | 6.23E-01 | 0.10 | 0.63 | 6.53E-01 | 0.10 | 0.38 | 9.90E-01 | 0.00 | 0.52 | 5594468 | 1489991 | 5671019 | 1457602 | 5598768 | 1524355 | 5743269 | 1399377 |
| RP_POS_195_13803a8_816 | RP | POS | 8.82 | 195.1380 | | | | 2.35E-01 | 0.18 | 0.79 | 6.23E-01 | 0.11 | 0.62 | 2.67E-01 | 0.23 | 0.67 | 4.39E-01 | 0.13 | 0.61 | 1436885 | 754358 | 1671174 | 1435466 | 1600053 | 1146407 | 1742295 | 1684817 |
| RP_POS_333_14929a10_73 | RP | POS | 10.73 | 333.1493 | | | | 1.48E-03 | 0.51 | 1.40 | 6.23E-01 | -0.11 | 1.19 | 1.35E-02 | 0.46 | 1.23 | 5.12E-03 | 0.56 | 1.36 | 634515 | 291850 | 865918 | 480087 | 889688 | 509427 | 842148 | 452772 |
| RP_POS_362_26541a12_326 | RP | POS | 12.33 | 362.2654 | | | | 1.57E-04 | -0.73 | 1.27 | 6.23E-01 | 0.10 | 0.50 | 2.34E-03 | -0.68 | 1.11 | 1.58E-04 | -0.78 | 1.46 | 752577 | 171426 | 607063 | 194398 | 597443 | 170236 | 616683 | 217216 |
| RP_POS_798_54126a13_716 | RP | POS | 13.72 | 798.5413 | Unknown | 184.0732(100), 798.5428(25), 162.955(57), 86.097(55), 739.4587(33) | 4 | 2.50E-05 | 0.71 | 1.20 | 6.23E-01 | 0.10 | 0.39 | 1.25E-04 | 0.76 | 1.29 | 8.28E-04 | 0.66 | 1.19 | 7743364 | 2320690 | 10151280 | 3473461 | 9979512 | 3505237 | 10323049 | 3468312 |
| RP_NEG_201_88861a15_537 | RP | NEG | 15.54 | 201.8886 | | | | 8.07E-01 | -0.06 | 0.51 | 6.24E-01 | -0.09 | 0.33 | 7.08E-01 | -0.10 | 0.40 | 9.45E-01 | -0.02 | 0.26 | 1969501 | 266916 | 1957086 | 180170 | 1965995 | 127638 | 1948177 | 221656 |

**Supplementary table 1: Metabolomics results** (in asceding order according to p-value from progressive ALD vs  non-progressive ALD comparison, with molecular features with ID_level of 1-2 first)

| | | | | | | | | all_ALD_vs_control | | | Prog_ALD_vs_non-prog_ALD | | | Prog_ALD_vs_control_ALD | | | Non-prog_ALD_vs_control | | | Healthy_control | | all_ALD | | Non-prog_ALD | | Progressive_ALD | |
|---|---|---|---|---|---|---|---|---|---|---|---|---|---|---|---|---|---|---|---|---|---|---|---|---|---|---|---|
| | | | | | | | | t-test | Cohen's | PLS-DA | t-test | Cohen's | PLS-DA | t-test | Cohen's | PLS-DA | t-test | Cohen's | PLS-DA | | | | | | | | |
| Feature_ID | RP_HILIC | POS_NEG | Average.Rt | Average.Mz | Metabolite.name | curated_MSMS | ID_level | p | d | VIP | p | d | VIP | p | d | VIP | p | d | VIP | Mean | SD | Mean | SD | Mean | SD | Mean | SD |
| RP_NEG_484_80441a15_5 | RP | NEG | 15.50 | 484.8044 | | | | 9.72E-01 | -0.01 | 0.64 | 6.24E-01 | -0.10 | 0.49 | 8.01E-01 | -0.06 | 0.51 | 8.49E-01 | 0.04 | 0.48 | 1099428 | 370043 | 1096729 | 383327 | 1115656 | 380276 | 1077801 | 389277 |
| RP_POS_253_14134a8_027 | RP | POS | 8.03 | 253.1413 | | | | 8.56E-03 | 0.51 | 0.96 | 6.24E-01 | -0.10 | 0.36 | 3.65E-02 | 0.46 | 0.80 | 9.64E-03 | 0.56 | 1.05 | 2144950 | 363855 | 2352659 | 411053 | 2372945 | 400354 | 2332373 | 424563 |
| RP_NEG_788_76935a0_499 | RP | NEG | 0.50 | 788.7694 | | | | 5.49E-03 | -0.38 | 1.16 | 6.24E-01 | -0.11 | 1.24 | 9.10E-03 | -0.44 | 1.22 | 8.23E-02 | -0.33 | 1.19 | 2388550 | 513419 | 1947847 | 1267546 | 2010401 | 1373741 | 1885294 | 1162319 |
| RP_POS_510_75928a4_629 | RP | POS | 4.63 | 510.7593 | | | | 1.48E-05 | 0.51 | 1.06 | 6.24E-01 | 0.11 | 1.08 | 1.72E-03 | 0.57 | 1.20 | 2.98E-03 | 0.46 | 1.15 | 84618 | 57821 | 495286 | 896356 | 451058 | 826341 | 539515 | 967715 |
| RP_POS_450_35675a11_568 | RP | POS | 11.57 | 450.3568 | | | | 1.01E-01 | -0.33 | 0.63 | 6.24E-01 | 0.10 | 0.37 | 2.01E-01 | -0.28 | 0.58 | 1.01E-01 | -0.38 | 0.69 | 2123119 | 147579 | 2072406 | 156438 | 2064689 | 167226 | 2080124 | 146147 |
| RP_NEG_213_91505a13_287 | RP | NEG | 13.29 | 213.9151 | | | | 2.96E-01 | 0.20 | 0.40 | 6.24E-01 | -0.10 | 0.32 | 4.96E-01 | 0.15 | 0.30 | 2.51E-01 | 0.25 | 0.53 | 625624 | 196693 | 669710 | 231768 | 681138 | 234269 | 658282 | 231042 |
| RP_POS_242_21132a11_127 | RP | POS | 11.13 | 242.2113 | | | | 2.13E-02 | 0.39 | 0.76 | 6.25E-01 | -0.10 | 0.34 | 7.34E-02 | 0.34 | 0.68 | 3.73E-02 | 0.44 | 0.76 | 1361093 | 2153225 | 2528669 | 3194520 | 2686110 | 3509729 | 2371228 | 2872177 |
| RP_POS_256_22696a11_1 | RP | POS | 11.10 | 256.2270 | | | | 1.50E-02 | 0.39 | 0.82 | 6.25E-01 | -0.11 | 0.34 | 5.95E-02 | 0.34 | 0.69 | 3.18E-02 | 0.45 | 0.78 | 398645 | 534350 | 715969 | 861541 | 758408 | 953153 | 673530 | 766472 |
| RP_POS_548_27448a10_212 | RP | POS | 10.21 | 548.2745 | | | | 5.78E-03 | 0.38 | 1.33 | 6.25E-01 | 0.11 | 0.39 | 3.48E-02 | 0.44 | 1.19 | 2.50E-02 | 0.33 | 1.02 | 824306 | 263547 | 1046816 | 640474 | 1015256 | 490489 | 1078376 | 765620 |
| RP_NEG_957_20636a9_484 | RP | NEG | 9.48 | 957.2064 | | | | 2.60E-01 | 0.21 | 0.94 | 6.25E-01 | 0.10 | 0.29 | 2.30E-01 | 0.26 | 0.80 | 4.47E-01 | 0.16 | 0.54 | 1668139 | 260827 | 1731333 | 310674 | 1716034 | 300344 | 1746632 | 322991 |
| RP_NEG_627_11353a9_125 | RP | NEG | 9.13 | 627.1135 | | | | 7.16E-01 | 0.08 | 0.59 | 6.25E-01 | -0.10 | 0.62 | 9.13E-01 | 0.03 | 0.30 | 5.74E-01 | 0.12 | 0.49 | 1812835 | 162470 | 1824845 | 159424 | 1832694 | 142861 | 1816996 | 175543 |
| RP_POS_486_86682a0_466 | RP | POS | 0.47 | 486.8668 | | | | 2.97E-07 | -0.69 | 1.32 | 6.25E-01 | 0.11 | 1.12 | 2.97E-04 | -0.63 | 1.36 | 4.05E-05 | -0.74 | 1.44 | 337408 | 55318 | 237549 | 156564 | 229846 | 158391 | 245253 | 155936 |
| RP_POS_485_28665a6_728 | RP | POS | 6.73 | 485.2867 | | | | 1.59E-07 | 0.63 | 1.33 | 6.26E-01 | -0.11 | 1.17 | 4.32E-04 | 0.57 | 1.43 | 1.25E-04 | 0.68 | 1.42 | 24078 | 33852 | 899441 | 1550383 | 975569 | 1614078 | 823312 | 1496448 |
| RP_POS_424_89758a0_47 | RP | POS | 0.47 | 424.8976 | | | | 2.28E-09 | -0.75 | 1.40 | 6.26E-01 | 0.11 | 1.15 | 5.02E-05 | -0.70 | 1.46 | 5.31E-06 | -0.80 | 1.53 | 1653698 | 163096 | 1151094 | 723568 | 1115597 | 728532 | 1186592 | 724194 |
| RP_POS_948_80518a0_494 | RP | POS | 0.49 | 948.8052 | | | | 1.71E-08 | 0.68 | 1.38 | 6.26E-01 | -0.11 | 1.20 | 1.27E-04 | 0.62 | 1.46 | 4.35E-05 | 0.73 | 1.49 | 70432 | 46347 | 857640 | 1280182 | 920433 | 1338130 | 794847 | 1229853 |
| RP_POS_312_2168a6_293 | RP | POS | 6.29 | 312.2168 | | | | 3.67E-01 | -0.16 | 0.45 | 6.26E-01 | 0.10 | 0.39 | 6.52E-01 | -0.11 | 0.68 | 2.10E-01 | -0.21 | 0.83 | 939302 | 453126 | 848114 | 604708 | 818432 | 366517 | 877797 | 776320 |
| RP_POS_255_23178a9_753 | RP | POS | 9.75 | 255.2318 | | | | 1.80E-04 | 0.61 | 1.27 | 6.27E-01 | -0.10 | 1.02 | 3.10E-03 | 0.56 | 1.20 | 9.31E-04 | 0.66 | 1.35 | 1253455 | 368681 | 1597427 | 587810 | 1626206 | 612438 | 1568648 | 566852 |
| RP_POS_453_24554a3_475 | RP | POS | 3.48 | 453.2455 | | | | 2.28E-05 | 0.50 | 1.09 | 6.27E-01 | 0.11 | 1.00 | 9.09E-04 | 0.56 | 1.28 | 8.15E-03 | 0.45 | 1.06 | 254136 | 165198 | 1483183 | 2751395 | 1348657 | 2799752 | 1617708 | 2723839 |
| RP_POS_430_91287a0_495 | RP | POS | 0.50 | 430.9129 | | | | 1.68E-07 | 0.63 | 1.34 | 6.27E-01 | -0.11 | 1.20 | 2.90E-04 | 0.57 | 1.41 | 1.73E-04 | 0.68 | 1.44 | 21498991 | 2596943 | 39770270 | 32216641 | 41344683 | 34414891 | 38195857 | 30125775 |
| HILIC_NEG_303_78622a1_363 | HILIC | NEG | 1.363 | 303.7862 | | | | 2.98E-09 | 0.75 | 1.18 | 6.28E-01 | 0.11 | 0.77 | 5.43E-05 | 0.80 | 1.24 | 2.14E-06 | 0.69 | 1.46 | 163678 | 88374 | 424409 | 376730 | 405998 | 307952 | 442820 | 437288 |
| RP_POS_561_39844a10_464 | RP | POS | 10.46 | 561.3984 | | | | 1.61E-01 | -0.37 | 0.70 | 6.28E-01 | 0.08 | 0.44 | 2.20E-01 | -0.33 | 0.56 | 1.48E-01 | -0.41 | 0.65 | 15907580 | 1726297 | 15447872 | 1017980 | 15398184 | 1155636 | 15497559 | 867860 |
| RP_NEG_256_90634a15_545 | RP | NEG | 15.55 | 256.9063 | | | | 3.79E-01 | -0.18 | 0.41 | 6.28E-01 | -0.10 | 0.36 | 3.15E-01 | -0.23 | 0.50 | 5.74E-01 | -0.13 | 0.39 | 1510604 | 46267 | 1502167 | 48608 | 1504538 | 49147 | 1499795 | 48444 |
| RP_POS_211_16916a11_127 | RP | POS | 11.13 | 211.1692 | | | | 1.41E-02 | 0.39 | 0.79 | 6.28E-01 | -0.10 | 0.33 | 5.73E-02 | 0.34 | 0.70 | 3.28E-02 | 0.44 | 0.77 | 371415 | 419186 | 627606 | 706327 | 662061 | 787100 | 593152 | 621311 |
| HILIC_NEG_144_92345a5_743 | HILIC | NEG | 5.743 | 144.9235 | | | | 4.45E-02 | -0.39 | 1.28 | 6.28E-01 | 0.10 | 0.66 | 1.29E-01 | -0.34 | 0.60 | 4.10E-02 | -0.44 | 1.15 | 41128479 | 7529319 | 37874724 | 8596747 | 37455551 | 8190706 | 38293897 | 9048210 |
| RP_NEG_681_29761a10_563 | RP | NEG | 10.56 | 681.2976 | | | | 7.74E-03 | -0.54 | 1.01 | 6.29E-01 | 0.10 | 0.31 | 2.87E-02 | -0.49 | 0.87 | 8.87E-03 | -0.59 | 1.12 | 1246851 | 107795 | 1185697 | 112058 | 1180240 | 111327 | 1191154 | 113647 |
| RP_POS_397_31015a11_71 | RP | POS | 11.71 | 397.3102 | | | | 4.86E-01 | -0.14 | 0.37 | 6.29E-01 | -0.10 | 0.41 | 4.04E-01 | -0.19 | 0.38 | 6.75E-01 | -0.10 | 0.32 | 2774262 | 1376737 | 2579384 | 1338147 | 2644527 | 1333666 | 2514241 | 1352958 |
| RP_NEG_590_34747a10_589 | RP | NEG | 10.59 | 590.3475 | | | | 3.11E-08 | 0.85 | 1.54 | 6.29E-01 | 0.10 | 0.70 | 4.88E-05 | 0.90 | 1.45 | 8.92E-08 | 0.80 | 1.82 | 1792455 | 1166710 | 3594154 | 2179141 | 3487967 | 1401597 | 3700341 | 2758032 |
| RP_NEG_578_30469a10_302 | RP | NEG | 10.30 | 578.3047 | | | | 4.75E-04 | 0.72 | 1.27 | 6.29E-01 | -0.09 | 0.79 | 3.61E-03 | 0.68 | 1.12 | 4.52E-04 | 0.77 | 1.32 | 789780 | 221260 | 957110 | 219862 | 967812 | 196530 | 946408 | 242501 |
| RP_POS_242_21133a11_038 | RP | POS | 11.04 | 242.2113 | | | | 2.81E-02 | 0.38 | 0.74 | 6.29E-01 | -0.10 | 0.35 | 8.44E-02 | 0.33 | 0.66 | 4.59E-02 | 0.43 | 0.73 | 1324592 | 2018991 | 2351788 | 2879123 | 2491913 | 3194594 | 2211664 | 2549974 |
| HILIC_POS_132_10211a4_005 | HILIC | POS | 4.005 | 132.1021 | | | | 4.12E-01 | 0.14 | 0.87 | 6.29E-01 | -0.10 | 0.96 | 6.76E-01 | 0.09 | 0.48 | 3.11E-01 | 0.19 | 0.56 | 182530633 | 58623901 | 193420833 | 81701969 | 197396466 | 72325048 | 189445200 | 90683423 |
| RP_POS_217_12216a8_264 | RP | POS | 8.26 | 217.1222 | | | | 1.05E-01 | -0.36 | 0.69 | 6.29E-01 | -0.09 | 0.52 | 8.19E-02 | -0.41 | 0.90 | 2.08E-01 | -0.31 | 0.63 | 1234006 | 86657 | 1205767 | 75194 | 1209425 | 83348 | 1202109 | 66705 |
| RP_POS_405_74149a12_757 | RP | POS | 12.76 | 405.7415 | | | | 2.15E-03 | 0.56 | 0.98 | 6.29E-01 | 0.10 | 0.37 | 3.63E-03 | 0.61 | 1.05 | 1.43E-02 | 0.51 | 0.92 | 1540944 | 1138920 | 2333006 | 1446411 | 2262662 | 1454405 | 2403349 | 1449659 |
| RP_POS_488_8714a0_512 | RP | POS | 0.51 | 488.8714 | | | | 2.88E-06 | 0.56 | 1.26 | 6.30E-01 | -0.11 | 1.11 | 1.53E-03 | 0.51 | 1.26 | 5.65E-04 | 0.62 | 1.38 | 219161 | 67090 | 505346 | 566951 | 532863 | 597080 | 477830 | 539793 |
| RP_POS_321_23898a11_254 | RP | POS | 11.25 | 321.2390 | | | | 9.80E-08 | 0.92 | 1.54 | 6.30E-01 | -0.10 | 0.91 | 1.25E-05 | 0.87 | 1.55 | 9.44E-07 | 0.97 | 1.67 | 1508722 | 507697 | 2190010 | 726005 | 2225240 | 710106 | 2154780 | 747088 |
| HILIC_POS_191_00827a0_849 | HILIC | POS | 0.849 | 191.0083 | | | | 3.42E-01 | 0.15 | 0.83 | 6.30E-01 | -0.11 | 0.95 | 6.43E-01 | 0.10 | 0.39 | 2.23E-01 | 0.21 | 0.45 | 575057 | 135741 | 605944 | 217033 | 616481 | 167210 | 595408 | 258809 |
| RP_POS_403_32703a11_61 | RP | POS | 11.61 | 403.3270 | | | | 5.31E-03 | -0.53 | 1.13 | 6.30E-01 | 0.10 | 0.75 | 2.03E-02 | -0.48 | 1.06 | 9.46E-03 | -0.58 | 1.05 | 1606066 | 124966 | 1529210 | 147905 | 1522033 | 159448 | 1536387 | 136636 |
| RP_POS_763_3775a13_569 | RP | POS | 13.57 | 763.3775 | | | | 6.60E-01 | 0.09 | 0.45 | 6.30E-01 | 0.10 | 0.37 | 5.40E-01 | 0.14 | 0.53 | 8.52E-01 | 0.04 | 0.26 | 981877 | 214055 | 1000941 | 205773 | 990960 | 214500 | 1010923 | 198332 |
| RP_POS_393_33304a11_265 | RP | POS | 11.27 | 393.3330 | | | | 9.07E-01 | -0.02 | 0.94 | 6.30E-01 | 0.11 | 0.90 | 8.64E-01 | 0.03 | 0.83 | 7.08E-01 | -0.07 | 0.88 | 447202 | 118106 | 443880 | 192861 | 434532 | 186642 | 453229 | 200341 |
| HILIC_POS_70_06599a4_871 | HILIC | POS | 4.871 | 70.0660 | | | | 5.82E-01 | 0.10 | 0.43 | 6.30E-01 | 0.10 | 0.49 | 4.31E-01 | 0.15 | 0.33 | 8.17E-01 | 0.05 | 0.11 | 19307025 | 9509762 | 20420468 | 11013443 | 19886561 | 13045514 | 20954375 | 8619751 |
| HILIC_NEG_279_01562a4_159 | HILIC | NEG | 4.159 | 279.0156 | | | | 1.69E-01 | 0.21 | 0.94 | 6.30E-01 | 0.11 | 0.83 | 2.00E-01 | 0.26 | 0.67 | 3.66E-01 | 0.15 | 0.55 | 199893 | 51015 | 218345 | 97852 | 213603 | 85447 | 223087 | 109537 |
| RP_POS_322_24326a10_427 | RP | POS | 10.43 | 322.2433 | | | | 3.12E-02 | 0.34 | 0.78 | 6.31E-01 | -0.10 | 0.71 | 1.51E-01 | 0.29 | 0.89 | 3.12E-02 | 0.40 | 0.73 | 1785371 | 217782 | 1901497 | 364789 | 1919163 | 334525 | 1883831 | 395366 |
| RP_POS_724_7782a0_502 | RP | POS | 0.50 | 724.7782 | | | | 7.97E-04 | -0.47 | 1.17 | 6.31E-01 | -0.11 | 1.25 | 2.57E-03 | -0.52 | 1.24 | 2.62E-02 | -0.42 | 1.22 | 1137539 | 225857 | 907272 | 537108 | 933255 | 569506 | 881289 | 507093 |
| RP_POS_553_45837a12_602 | RP | POS | 12.60 | 553.4584 | | | | 3.97E-01 | -0.20 | 0.73 | 6.31E-01 | -0.09 | 0.43 | 3.37E-01 | -0.25 | 0.57 | 5.35E-01 | -0.16 | 0.41 | 15115686 | 5666196 | 14186342 | 4210746 | 14389932 | 4184055 | 13982751 | 4269860 |
| RP_NEG_207_93056a15_702 | RP | NEG | 15.70 | 207.9306 | | | | 8.69E-01 | -0.04 | 0.65 | 6.32E-01 | -0.10 | 0.38 | 7.19E-01 | -0.08 | 0.27 | 9.58E-01 | 0.01 | 0.66 | 11008952 | 5404686 | 10829942 | 5028717 | 11072644 | 5323661 | 10587241 | 4757151 |
| HILIC_NEG_214_96556a0_999 | HILIC | NEG | 0.999 | 214.9656 | | | | 1.28E-01 | 0.22 | 0.73 | 6.32E-01 | -0.11 | 1.11 | 3.46E-01 | 0.17 | 0.31 | 1.44E-01 | 0.28 | 0.67 | 1776343 | 378521 | 1934206 | 781711 | 1971925 | 807393 | 1896486 | 761465 |
| RP_NEG_446_37741a12_334 | RP | NEG | 12.33 | 446.3774 | | | | 8.20E-01 | -0.05 | 0.64 | 6.32E-01 | -0.09 | 0.40 | 6.94E-01 | -0.10 | 0.55 | 9.95E-01 | 0.00 | 0.31 | 549091 | 202819 | 539837 | 186606 | 548839 | 166134 | 530835 | 206369 |
| RP_POS_409_1734a10_977 | RP | POS | 10.98 | 409.1734 | | | | 5.82E-03 | 0.47 | 1.35 | 6.32E-01 | -0.10 | 1.16 | 4.28E-02 | 0.42 | 1.15 | 6.97E-03 | 0.52 | 1.32 | 515528 | 211229 | 651890 | 302006 | 666457 | 280208 | 637323 | 324541 |
| RP_POS_171_09929a4_112 | RP | POS | 4.11 | 171.0993 | | | | 9.63E-01 | 0.01 | 0.29 | 6.32E-01 | 0.10 | 0.66 | 8.06E-01 | 0.06 | 0.54 | 8.56E-01 | -0.04 | 0.49 | 1971471 | 1454229 | 1985259 | 1488383 | 1913499 | 1333326 | 2057019 | 1639363 |
| HILIC_POS_307_04355a2_456 | HILIC | POS | 2.456 | 307.0436 | | | | 1.83E-05 | 0.53 | 1.03 | 6.33E-01 | 0.11 | 0.63 | 3.04E-03 | 0.58 | 0.96 | 7.58E-04 | 0.48 | 1.45 | 925607 | 317231 | 1638696 | 1496239 | 1566623 | 1212675 | 1710770 | 1744125 |
| RP_POS_545_64026a6_946 | RP | POS | 6.95 | 545.6403 | | | | 2.37E-07 | 0.62 | 1.30 | 6.33E-01 | -0.11 | 1.12 | 3.54E-04 | 0.56 | 1.39 | 2.20E-04 | 0.67 | 1.38 | 17502 | 30230 | 983296 | 1738945 | 1067011 | 1859347 | 899580 | 1624222 |
| RP_POS_331_65222a5_615 | RP | POS | 5.62 | 331.6522 | | | | 1.78E-01 | -0.27 | 0.99 | 6.33E-01 | -0.10 | 0.76 | 1.77E-01 | -0.32 | 0.89 | 3.01E-01 | -0.22 | 0.92 | 671879 | 201326 | 615236 | 214355 | 625552 | 187821 | 604919 | 239458 |
| RP_POS_337_14667a10_731 | RP | POS | 10.73 | 337.1467 | | | | 2.76E-03 | 0.49 | 1.37 | 6.33E-01 | -0.10 | 1.14 | 2.03E-02 | 0.44 | 1.20 | 7.83E-03 | 0.54 | 1.34 | 2037062 | 930970 | 2722419 | 1494283 | 2794326 | 1580349 | 2650513 | 1415372 |
| RP_NEG_411_34738a11_534 | RP | NEG | 11.53 | 411.3474 | | | | 4.47E-02 | -0.33 | 0.83 | 6.33E-01 | 0.10 | 0.69 | 1.42E-01 | -0.28 | 0.81 | 5.88E-02 | -0.39 | 0.73 | 1356704 | 194322 | 1264382 | 294914 | 1250199 | 308667 | 1278564 | 282910 |
| RP_POS_834_09357a14_024 | RP | POS | 14.02 | 834.0936 | | | | 1.37E-05 | 0.67 | 1.12 | 6.33E-01 | 0.10 | 0.76 | 8.41E-04 | 0.72 | 1.12 | 1.00E-04 | 0.62 | 1.41 | 845334 | 535275 | 1473359 | 993489 | 1425556 | 744833 | 1521162 | 1197808 |
| RP_POS_516_62372a6_84 | RP | POS | 6.84 | 516.6237 | | | | 2.22E-07 | 0.62 | 1.32 | 6.34E-01 | -0.11 | 1.16 | 3.76E-04 | 0.56 | 1.43 | 1.96E-04 | 0.67 | 1.39 | 17390 | 30622 | 1012804 | 1787295 | 1098590 | 1898668 | 927017 | 1683472 |
| RP_POS_175_14812a11_038 | RP | POS | 11.04 | 175.1481 | | | | 8.23E-03 | 0.42 | 0.82 | 6.34E-01 | -0.10 | 0.34 | 3.69E-02 | 0.37 | 0.75 | 2.31E-02 | 0.47 | 0.80 | 269883 | 366110 | 510847 | 613186 | 540282 | 686195 | 481412 | 535757 |
| RP_POS_398_76251a13_154 | RP | POS | 13.15 | 398.7625 | | | | 2.13E-01 | -0.27 | 0.44 | 6.34E-01 | 0.09 | 0.30 | 3.68E-01 | -0.22 | 0.50 | 1.67E-01 | -0.31 | 0.62 | 6157125 | 2044605 | 5641056 | 1908949 | 5549468 | 1706188 | 5732644 | 2105789 |
| RP_NEG_265_14896a13_621 | RP | NEG | 13.62 | 265.1490 | | | | 8.79E-01 | 0.03 | 0.58 | 6.34E-01 | -0.10 | 0.51 | 9.28E-01 | -0.02 | 0.37 | 6.79E-01 | 0.08 | 0.64 | 346940 | 210169 | 353789 | 250104 | 365792 | 184459 | 341786 | 303416 |
| RP_NEG_452_92313a0_494 | RP | NEG | 0.49 | 452.9231 | | | | 3.70E-07 | 0.61 | 1.33 | 6.34E-01 | -0.11 | 1.20 | 3.58E-04 | 0.56 | 1.40 | 2.87E-04 | 0.66 | 1.42 | 22255410 | 2429329 | 35288296 | 23630810 | 36421507 | 25514222 | 34155086 | 21786012 |
| RP_POS_619_43866a11_253 | RP | POS | 11.25 | 619.4387 | | | | 1.77E-02 | 0.48 | 1.02 | 6.34E-01 | 0.09 | 0.56 | 1.40E-02 | 0.53 | 1.08 | 6.36E-02 | 0.44 | 0.79 | 2745870 | 505055 | 2997673 | 511159 | 2973181 | 573892 | 3022166 | 444210 |
| HILIC_NEG_115_92065a5_425 | HILIC | NEG | 5.425 | 115.9207 | | | | 9.49E-01 | 0.01 | 0.14 | 6.34E-01 | -0.09 | 0.51 | 8.91E-01 | -0.03 | 0.22 | 8.02E-01 | 0.06 | 0.13 | 92181680 | 15912576 | 92385342 | 14048629 | 93058166 | 14551808 | 91712517 | 13640997 |
| RP_POS_199_01102a11_21 | RP | POS | 11.21 | 199.0110 | | | | 2.55E-01 | -0.25 | 0.47 | 6.35E-01 | -0.09 | 0.29 | 2.10E-01 | -0.30 | 0.53 | 4.00E-01 | -0.21 | 0.55 | 3740312 | 862914 | 3544612 | 746801 | 3580364 | 786347 | 3508860 | 711235 |
| RP_POS_158_11772a3_361 | RP | POS | 3.36 | 158.1177 | Unknown | 112.1118(100), 158.0968(17), 95.0867(14) | 4 | 1.99E-01 | -0.20 | 0.65 | 6.35E-01 | 0.11 | 0.48 | 5.41E-01 | -0.14 | 0.71 | 5.59E-02 | -0.25 | 1.05 | 1074535 | 639027 | 862038 | 1194087 | 804786 | 564695 | 919290 | 1598504 |
| RP_POS_422_18185a7_55 | RP | POS | 7.55 | 422.1819 | | | | 9.57E-01 | 0.01 | 0.85 | 6.35E-01 | 0.10 | 0.69 | 7.99E-01 | 0.06 | 0.67 | 8.76E-01 | -0.04 | 0.88 | 6459456 | 443877 | 6464269 | 426497 | 6443861 | 434538 | 6484677 | 421709 |
| RP_POS_189_13455a0_543 | RP | POS | 0.54 | 189.1346 | | | | 5.29E-01 | -0.11 | 0.60 | 6.35E-01 | -0.10 | 0.56 | 4.62E-01 | -0.16 | 0.55 | 7.72E-01 | -0.05 | 0.32 | 386827 | 138646 | 366548 | 207230 | 376462 | 182261 | 356633 | 230966 |
| RP_POS_265_1767a8_795 | RP | POS | 8.80 | 265.1767 | Unknown | 265.1756(100) | 4 | 8.17E-01 | 0.05 | 0.59 | 6.35E-01 | 0.10 | 0.59 | 6.89E-01 | 0.10 | 0.44 | 9.97E-01 | 0.00 | 0.58 | 3816970 | 587522 | 3844665 | 587862 | 3816547 | 521606 | 3872784 | 651559 |
| RP_NEG_336_85922a15_49 | RP | NEG | 15.49 | 336.8592 | | | | 2.48E-01 | 0.23 | 0.56 | 6.35E-01 | -0.10 | 0.43 | 4.07E-01 | 0.18 | 0.40 | 2.17E-01 | 0.28 | 0.62 | 14188123 | 278649 | 14254852 | 288689 | 14268647 | 296617 | 14241056 | 282866 |
| RP_POS_307_26004a11_609 | RP | POS | 11.61 | 307.2600 | | | | 9.23E-01 | -0.02 | 0.57 | 6.35E-01 | 0.10 | 0.60 | 8.67E-01 | 0.03 | 0.46 | 7.57E-01 | -0.07 | 0.78 | 1246923 | 233076 | 1241957 | 301542 | 1227557 | 331724 | 1256356 | 270641 |
| RP_POS_492_25397a6_325 | RP | POS | 6.33 | 492.2540 | | | | 2.80E-07 | 0.61 | 1.31 | 6.36E-01 | -0.11 | 1.14 | 4.52E-04 | 0.56 | 1.42 | 2.09E-04 | 0.67 | 1.39 | 17561 | 29042 | 689099 | 1217118 | 747106 | 1287018 | 631092 | 1153128 |
| RP_POS_473_34482a10_543 | RP | POS | 10.54 | 473.3448 | | | | 6.80E-01 | 0.09 | 0.34 | 6.36E-01 | -0.09 | 0.36 | 8.58E-01 | 0.04 | 0.26 | 5.80E-01 | 0.13 | 0.47 | 11332899 | 1604567 | 11465932 | 1501372 | 11537448 | 1655693 | 11394417 | 1342564 |
| RP_POS_149_0229a11_911 | RP | POS | 11.91 | 149.0229 | | | | 8.22E-02 | -0.38 | 0.67 | 6.36E-01 | -0.09 | 0.61 | 5.64E-02 | -0.43 | 1.19 | 1.87E-01 | -0.33 | 0.55 | 4062985 | 1227873 | 3631517 | 1092645 | 3683578 | 1303656 | 3579457 | 840869 |
| RP_POS_158_11748a0_708 | RP | POS | 0.71 | 158.1175 | | | | 2.98E-01 | 0.17 | 0.43 | 6.36E-01 | -0.10 | 0.31 | 5.05E-01 | 0.12 | 0.48 | 3.00E-01 | 0.22 | 0.75 | 703716 | 737528 | 886762 | 1164117 | 942187 | 1328203 | 831338 | 983667 |
| HILIC_NEG_185_96262a0_655 | HILIC | NEG | 0.655 | 185.9626 | | | | 3.08E-02 | 0.37 | 0.91 | 6.37E-01 | 0.10 | 1.08 | 5.37E-02 | 0.42 | 0.66 | 8.70E-02 | 0.31 | 0.71 | 298363 | 99823 | 348861 | 146794 | 341874 | 126169 | 355849 | 165887 |
| HILIC_NEG_115_92071a7_955 | HILIC | NEG | 7.955 | 115.9207 | | | | 9.77E-01 | -0.01 | 0.44 | 6.37E-01 | 0.10 | 0.40 | 8.54E-01 | 0.04 | 0.30 | 8.10E-01 | -0.05 | 0.55 | 45580926 | 26897351 | 45419439 | 27388454 | 44116263 | 26832366 | 46722616 | 28144658 |
| RP_POS_342_26382a7_82 | RP | POS | 7.82 | 342.2638 | | | | 3.04E-01 | 0.20 | 0.64 | 6.37E-01 | 0.10 | 0.48 | 2.77E-01 | 0.25 | 0.50 | 4.73E-01 | 0.15 | 0.71 | 2845241 | 1272322 | 3119675 | 1396197 | 3053316 | 1273006 | 3186033 | 1519534 |
| RP_POS_738_50677a12_693 | RP | POS | 12.69 | 738.5068 | | | | 5.05E-04 | 0.59 | 0.97 | 6.37E-01 | -0.10 | 0.73 | 5.01E-03 | 0.54 | 0.96 | 2.01E-03 | 0.64 | 1.06 | 362578 | 316903 | 628345 | 471578 | 650738 | 499956 | 605952 | 445344 |
| RP_POS_617_51056a11_257 | RP | POS | 11.26 | 617.5106 | | | | 3.23E-01 | -0.33 | 1.03 | 6.37E-01 | -0.04 | 0.47 | 2.95E-01 | -0.36 | 0.77 | 3.61E-01 | -0.31 | 0.76 | 3803917 | 10228973 | 1971626 | 2464551 | 2088695 | 2876204 | 1854556 | 1992853 |
| RP_POS_499_82703a4_778 | RP | POS | 4.78 | 499.8270 | | | | 3.43E-01 | -0.22 | 0.57 | 6.38E-01 | -0.09 | 0.76 | 3.06E-01 | -0.27 | 0.61 | 4.62E-01 | -0.18 | 0.63 | 2013119 | 1747877 | 1691012 | 1321361 | 1753575 | 1165364 | 1628449 | 1470226 |
| HILIC_POS_110_06044a1_505 | HILIC | POS | 1.505 | 110.0604 | | | | 6.93E-01 | 0.07 | 0.27 | 6.39E-01 | 0.10 | 0.89 | 5.96E-01 | 0.12 | 0.28 | 9.23E-01 | 0.02 | 0.12 | 730884 | 1472822 | 862618 | 2068274 | 764721 | 1651134 | 960514 | 2428377 |
| RP_POS_470_61133a6_669 | RP | POS | 6.67 | 470.6113 | | | | 2.40E-07 | 0.62 | 1.32 | 6.39E-01 | -0.10 | 1.13 | 3.92E-04 | 0.56 | 1.40 | 2.02E-04 | 0.67 | 1.40 | 23304 | 55931 | 840053 | 1469450 | 909558 | 1558768 | 770548 | 1386730 |
| RP_POS_399_30771a13_003 | RP | POS | 13.00 | 399.3077 | | | | 3.24E-01 | -0.22 | 0.46 | 6.39E-01 | 0.09 | 0.34 | 4.74E-01 | -0.17 | 0.42 | 2.77E-01 | -0.26 | 0.44 | 4436218 | 1845879 | 4073113 | 1620883 | 3996463 | 1645636 | 4149763 | 1608736 |
| RP_POS_499_9624a6_788 | RP | POS | 6.79 | 499.9624 | | | | 2.13E-07 | 0.62 | 1.31 | 6.39E-01 | -0.10 | 1.14 | 3.75E-04 | 0.57 | 1.41 | 1.91E-04 | 0.67 | 1.39 | 19991 | 26359 | 970398 | 1703887 | 1050964 | 1806264 | 889832 | 1609309 |
| RP_POS_332_29169a11_249 | RP | POS | 11.25 | 332.2917 | | | | 2.57E-15 | 1.19 | 1.81 | 6.39E-01 | 0.09 | 0.61 | 3.40E-10 | 1.23 | 1.90 | 3.42E-11 | 1.14 | 1.95 | 101588884 | 49080475 | 221789828 | 96083065 | 217247999 | 86799635 | 226331658 | 105242782 |
| RP_POS_460_27957a10_706 | RP | POS | 10.71 | 460.2796 | | | | 2.59E-01 | 0.20 | 0.76 | 6.39E-01 | -0.10 | 0.92 | 4.58E-01 | 0.15 | 0.94 | 2.44E-01 | 0.25 | 0.65 | 518767 | 166491 | 561301 | 230149 | 572167 | 244969 | 550435 | 216257 |
| HILIC_POS_134_11755a1_716 | HILIC | POS | 1.716 | 134.1176 | | | | 3.71E-05 | -0.62 | 1.33 | 6.39E-01 | 0.10 | 0.82 | 2.00E-03 | -0.57 | 1.44 | 4.02E-04 | -0.67 | 1.42 | 1256086 | 304656 | 910068 | 595363 | 881973 | 604746 | 938162 | 590610 |
| RP_POS_126_02209a0_531 | RP | POS | 0.53 | 126.0221 | | | | 4.20E-06 | 0.56 | 1.16 | 6.39E-01 | 0.10 | 1.12 | 6.05E-04 | 0.62 | 1.28 | 1.59E-03 | 0.51 | 1.18 | 280830 | 63309 | 453852 | 340430 | 437791 | 324494 | 469913 | 358227 |
| RP_NEG_976_75726a0_495 | RP | NEG | 0.50 | 976.7573 | | | | 6.63E-08 | -0.66 | 1.35 | 6.40E-01 | 0.10 | 1.19 | 2.74E-04 | -0.61 | 1.41 | 5.17E-05 | -0.71 | 1.46 | 2892014 | 205753 | 2148663 | 1233843 | 2090462 | 1260599 | 2206865 | 1216464 |
| HILIC_NEG_114_94934a2_945 | HILIC | NEG | 2.945 | 114.9493 | | | | 2.16E-02 | -0.44 | 1.00 | 6.40E-01 | -0.10 | 0.73 | 2.62E-02 | -0.49 | 0.92 | 6.54E-02 | -0.39 | 0.67 | 1213133 | 266405 | 1081059 | 302283 | 1095316 | 295315 | 1066802 | 311431 |

**Supplementary table 1: Metabolomics results** (in asceding order according to p-value from progressive ALD vs  non-progressive ALD comparison, with molecular features with ID_level of 1-2 first)

| | | | | | | | | all_ALD_vs_control | | | Prog_ALD_vs_non-prog_ALD | | | Prog_ALD_vs_control_ALD | | | Non-prog_ALD_vs_control | | | Healthy_control | | all_ALD | | Non-prog_ALD | | Progressive_ALD | |
|---|---|---|---|---|---|---|---|---|---|---|---|---|---|---|---|---|---|---|---|---|---|---|---|---|---|---|---|
| | | | | | | | | t-test | Cohen's | PLS-DA | t-test | Cohen's | PLS-DA | t-test | Cohen's | PLS-DA | t-test | Cohen's | PLS-DA | | | | | | | | |
| Feature_ID | RP_HILIC | POS_NEG | Average.Rt | Average.Mz | Metabolite.name | curated_MSMS | ID_level | p | d | VIP | p | d | VIP | p | d | VIP | p | d | VIP | Mean | SD | Mean | SD | Mean | SD | Mean | SD |
| RP_NEG_353_00983a1_093 | RP | NEG | 1.09 | 353.0098 | | | | 1.23E-02 | -0.60 | 1.07 | 6.40E-01 | 0.08 | 0.87 | 2.70E-02 | -0.56 | 0.96 | 1.24E-02 | -0.64 | 1.03 | 2700540 | 2159689 | 1617127 | 1613178 | 1541068 | 1655540 | 1693185 | 1582776 |
| RP_NEG_446_90628a0_464 | RP | NEG | 0.46 | 446.9063 | | | | 6.99E-10 | -0.77 | 1.42 | 6.40E-01 | 0.10 | 1.17 | 2.81E-05 | -0.72 | 1.49 | 3.68E-06 | -0.82 | 1.54 | 1713518 | 149001 | 1178233 | 753085 | 1142742 | 761462 | 1213724 | 750635 |
| RP_POS_254_82314a0_521 | RP | POS | 0.52 | 254.8231 | | | | 5.02E-06 | 0.56 | 1.25 | 6.40E-01 | -0.10 | 1.12 | 1.80E-03 | 0.51 | 1.25 | 6.70E-04 | 0.61 | 1.37 | 710570 | 120622 | 1048332 | 672519 | 1080013 | 707183 | 1016650 | 641591 |
| HILIC_NEG_97_93105a2_956 | HILIC | NEG | 2.956 | 97.9311 | | | | 8.88E-01 | 0.03 | 0.56 | 6.40E-01 | -0.10 | 0.43 | 9.23E-01 | -0.02 | 0.25 | 7.34E-01 | 0.08 | 0.29 | 231357 | 96239 | 234193 | 106882 | 239226 | 110017 | 229160 | 104524 |
| RP_POS_266_69232a10_731 | RP | POS | 10.73 | 266.6923 | | | | 3.09E-06 | 0.72 | 1.22 | 6.40E-01 | -0.10 | 0.81 | 1.12E-03 | 0.68 | 1.16 | 5.46E-06 | 0.77 | 1.47 | 392681 | 134259 | 560721 | 242246 | 572127 | 199008 | 549316 | 280524 |
| HILIC_POS_314_23267a0_776 | HILIC | POS | 0.776 | 314.2327 | | | | 1.61E-01 | -0.23 | 0.62 | 6.41E-01 | -0.10 | 0.78 | 1.05E-01 | -0.28 | 0.60 | 4.04E-01 | -0.18 | 0.61 | 20141733 | 11475282 | 16377158 | 17215408 | 17186746 | 20350087 | 15567569 | 13539767 |
| RP_POS_617_27515a7_187 | RP | POS | 7.19 | 617.2752 | | | | 1.49E-01 | -0.26 | 0.76 | 6.41E-01 | 0.10 | 0.68 | 2.87E-01 | -0.21 | 0.64 | 1.57E-01 | -0.31 | 0.89 | 1407363 | 80937 | 1381586 | 103219 | 1376735 | 112773 | 1386438 | 93594 |
| HILIC_POS_491_47672a1_308 | HILIC | POS | 1.308 | 491.4767 | | | | 3.35E-01 | 0.18 | 0.41 | 6.41E-01 | -0.10 | 0.43 | 5.54E-01 | 0.13 | 0.24 | 2.80E-01 | 0.22 | 0.50 | 292062 | 227136 | 339877 | 285656 | 353296 | 278529 | 326458 | 294818 |
| RP_POS_192_13849a2_004 | RP | POS | 2.00 | 192.1385 | Unknown | 192.1387(100), 133.0653 | 4 | 1.84E-01 | 0.24 | 0.49 | 6.41E-01 | -0.10 | 0.38 | 3.84E-01 | 0.19 | 0.39 | 1.47E-01 | 0.29 | 0.63 | 21233534 | 7679721 | 23485443 | 9869579 | 23948938 | 8935978 | 23021948 | 10794210 |
| RP_NEG_241_18117a9_624 | RP | NEG | 9.62 | 241.1812 | | | | 1.39E-02 | 0.39 | 1.42 | 6.41E-01 | 0.10 | 1.48 | 1.57E-02 | 0.44 | 1.31 | 9.18E-02 | 0.34 | 1.16 | 1774422 | 1160521 | 2481357 | 1932110 | 2390682 | 2101098 | 2572033 | 1763771 |
| HILIC_NEG_305_78415a1_363 | HILIC | NEG | 1.363 | 305.7842 | | | | 4.32E-09 | 0.75 | 1.14 | 6.41E-01 | 0.10 | 0.74 | 8.32E-05 | 0.80 | 1.21 | 9.64E-07 | 0.69 | 1.52 | 78857 | 46212 | 205294 | 182988 | 196704 | 142389 | 213885 | 217319 |
| HILIC_NEG_100_93341a7_676 | HILIC | NEG | 7.676 | 100.9334 | | | | 6.61E-01 | -0.09 | 0.65 | 6.41E-01 | -0.10 | 1.11 | 5.37E-01 | -0.13 | 0.29 | 8.69E-01 | -0.04 | 0.84 | 33808830 | 4097458 | 33431116 | 4562506 | 33645189 | 4766171 | 33217043 | 4387237 |
| HILIC_POS_387_21344a2_224 | HILIC | POS | 2.224 | 387.2134 | | | | 7.85E-01 | 0.05 | 0.73 | 6.41E-01 | -0.10 | 0.72 | 9.89E-01 | 0.00 | 0.20 | 6.39E-01 | 0.10 | 0.34 | 2148433 | 555232 | 2180591 | 646245 | 2210904 | 629810 | 2150278 | 667272 |
| RP_POS_239_16177a9_162 | RP | POS | 9.16 | 239.1618 | | | | 2.09E-03 | 0.49 | 1.46 | 6.42E-01 | 0.10 | 1.37 | 2.64E-03 | 0.54 | 1.42 | 3.30E-02 | 0.44 | 1.17 | 1112629 | 459292 | 1470358 | 784858 | 1433573 | 873991 | 1507143 | 691341 |
| HILIC_POS_244_15462a1_49 | HILIC | POS | 1.49 | 244.1546 | | | | 2.14E-03 | 0.43 | 0.82 | 6.42E-01 | -0.10 | 1.04 | 5.46E-02 | 0.38 | 0.67 | 2.66E-03 | 0.49 | 1.04 | 913590 | 360149 | 1242296 | 831253 | 1281258 | 705826 | 1203333 | 945925 |
| RP_POS_228_19574a11_127 | RP | POS | 11.13 | 228.1957 | | | | 1.58E-02 | 0.40 | 0.77 | 6.42E-01 | -0.10 | 0.33 | 6.18E-02 | 0.35 | 0.69 | 3.15E-02 | 0.45 | 0.77 | 14227900 | 20313494 | 26080169 | 31988950 | 27579294 | 34849107 | 24581045 | 29127696 |
| RP_POS_591_38672a10_704 | RP | POS | 10.70 | 591.3867 | | | | 4.38E-01 | 0.13 | 0.35 | 6.42E-01 | 0.10 | 0.53 | 4.17E-01 | 0.18 | 0.45 | 6.48E-01 | 0.08 | 0.49 | 4070225 | 872720 | 4226846 | 1290261 | 4166356 | 1003469 | 4287336 | 1532678 |
| HILIC_NEG_259_81641a3_027 | HILIC | NEG | 3.027 | 259.8164 | | | | 1.12E-07 | -0.74 | 1.29 | 6.42E-01 | 0.10 | 0.74 | 1.13E-04 | -0.69 | 1.40 | 1.36E-05 | -0.79 | 1.51 | 3668473 | 150385 | 3415807 | 360289 | 3398927 | 361658 | 3432687 | 361784 |
| HILIC_NEG_315_04202a5_743 | HILIC | NEG | 5.743 | 315.0420 | | | | 4.01E-01 | -0.18 | 0.67 | 6.42E-01 | -0.09 | 0.90 | 3.47E-01 | -0.23 | 0.40 | 5.60E-01 | -0.14 | 0.40 | 5096951 | 1065107 | 4918036 | 959465 | 4962988 | 913971 | 4873083 | 1010181 |
| RP_POS_374_28027a11_369 | RP | POS | 11.37 | 374.2803 | | | | 8.03E-01 | -0.05 | 0.64 | 6.42E-01 | -0.09 | 0.29 | 6.69E-01 | -0.10 | 0.40 | 9.89E-01 | 0.00 | 0.37 | 318436 | 253384 | 305473 | 259235 | 317617 | 254357 | 293330 | 266043 |
| RP_POS_427_28256a11_608 | RP | POS | 11.61 | 427.2826 | | | | 1.86E-01 | -0.26 | 0.71 | 6.42E-01 | 0.09 | 0.37 | 3.17E-01 | -0.22 | 0.56 | 1.79E-01 | -0.31 | 0.61 | 2129315 | 301133 | 2046317 | 318641 | 2031393 | 343854 | 2061241 | 294018 |
| RP_POS_739_51318a12_325 | RP | POS | 12.33 | 739.5132 | | | | 5.41E-06 | -1.00 | 1.69 | 6.42E-01 | 0.08 | 0.57 | 3.60E-05 | -0.96 | 1.54 | 8.04E-06 | -1.05 | 1.69 | 782107 | 218344 | 561053 | 192745 | 552031 | 190474 | 570074 | 196501 |
| RP_NEG_235_92618a15_681 | RP | NEG | 15.68 | 235.9262 | | | | 8.03E-01 | -0.05 | 0.27 | 6.42E-01 | 0.10 | 0.30 | 9.87E-01 | 0.00 | 0.40 | 6.28E-01 | -0.09 | 0.46 | 23564748 | 5897808 | 23243127 | 7439154 | 22894875 | 6368966 | 23591379 | 8426162 |
| HILIC_NEG_221_84235a2_947 | HILIC | NEG | 2.947 | 221.8424 | | | | 1.69E-04 | 0.61 | 1.20 | 6.42E-01 | -0.10 | 0.70 | 4.67E-03 | 0.56 | 1.02 | 5.13E-04 | 0.66 | 1.21 | 459937 | 41369 | 498904 | 66765 | 502029 | 63885 | 495780 | 70035 |
| HILIC_POS_175_0343a0_852 | HILIC | POS | 0.852 | 175.0343 | | | | 4.57E-01 | 0.13 | 0.77 | 6.42E-01 | -0.10 | 0.93 | 7.07E-01 | 0.09 | 0.22 | 3.36E-01 | 0.18 | 0.37 | 2310159 | 671979 | 2419524 | 854252 | 2459511 | 693085 | 2379537 | 995367 |
| HILIC_POS_163_02809a4_818 | HILIC | POS | 4.818 | 163.0281 | | | | 5.98E-03 | 0.47 | 1.14 | 6.42E-01 | 0.10 | 0.35 | 1.16E-02 | 0.52 | 0.91 | 3.36E-02 | 0.42 | 1.45 | 1098466 | 818089 | 1630069 | 1201500 | 1573864 | 1170728 | 1686275 | 1240812 |
| HILIC_POS_105_04279a4_324 | HILIC | POS | 4.324 | 105.0428 | | | | 5.68E-01 | 0.11 | 0.86 | 6.43E-01 | 0.10 | 0.78 | 4.61E-01 | 0.15 | 0.27 | 7.93E-01 | 0.06 | 0.19 | 55054853 | 11728059 | 56496980 | 14225894 | 55831923 | 14807569 | 57162037 | 13737375 |
| RP_NEG_332_92242a0_497 | RP | NEG | 0.50 | 332.9224 | | | | 2.22E-07 | -0.63 | 1.33 | 6.43E-01 | 0.10 | 1.20 | 3.07E-04 | -0.58 | 1.40 | 1.71E-04 | -0.68 | 1.43 | 24224868 | 1275741 | 19405347 | 8434331 | 19011065 | 8963880 | 19799630 | 7940985 |
| RP_NEG_615_14105a8_509 | RP | NEG | 8.51 | 615.1411 | | | | 3.31E-01 | -0.21 | 0.48 | 6.43E-01 | 0.09 | 0.95 | 4.93E-01 | -0.16 | 0.38 | 2.84E-01 | -0.25 | 0.50 | 916186 | 63176 | 903758 | 59776 | 900965 | 60617 | 906551 | 59405 |
| HILIC_NEG_115_92072a0_677 | HILIC | NEG | 0.677 | 115.9207 | | | | 1.58E-02 | 0.58 | 1.13 | 6.43E-01 | 0.08 | 0.61 | 1.59E-02 | 0.62 | 1.08 | 3.32E-02 | 0.54 | 0.95 | 14896241 | 3587257 | 16625474 | 2669640 | 16500759 | 2601582 | 16750190 | 2756706 |
| RP_NEG_178_97713a15_515 | RP | NEG | 15.52 | 178.9771 | | | | 2.33E-02 | -0.31 | 0.86 | 6.43E-01 | 0.10 | 0.53 | 6.15E-02 | -0.26 | 0.83 | 8.39E-02 | -0.37 | 0.90 | 1357390 | 52471 | 1320671 | 130137 | 1314593 | 159367 | 1326750 | 93507 |
| RP_NEG_637_14539a9_125 | RP | NEG | 9.13 | 637.1454 | | | | 5.07E-01 | 0.15 | 0.72 | 6.43E-01 | -0.09 | 0.64 | 6.72E-01 | 0.10 | 0.39 | 4.32E-01 | 0.19 | 0.71 | 16633073 | 1370287 | 16813812 | 1197437 | 16869629 | 1236663 | 16757995 | 1166739 |
| HILIC_POS_371_23959a2_224 | HILIC | POS | 2.224 | 371.2396 | | | | 4.66E-01 | 0.14 | 0.67 | 6.44E-01 | -0.10 | 0.80 | 6.73E-01 | 0.10 | 0.20 | 3.80E-01 | 0.19 | 0.38 | 16118929 | 4770413 | 16847000 | 5215023 | 17090025 | 4994833 | 16603975 | 5466175 |
| HILIC_POS_286_20126a0_914 | HILIC | POS | 0.914 | 286.2013 | | | | 7.95E-01 | 0.04 | 0.35 | 6.44E-01 | 0.10 | 0.75 | 6.59E-01 | 0.09 | 0.31 | 9.79E-01 | -0.01 | 0.60 | 2844890 | 1681264 | 2944259 | 2374302 | 2833728 | 2141185 | 3054791 | 2603855 |
| HILIC_NEG_214_85135a7_668 | HILIC | NEG | 7.668 | 214.8514 | | | | 3.78E-01 | -0.18 | 0.47 | 6.45E-01 | 0.09 | 0.42 | 5.63E-01 | -0.13 | 0.22 | 3.19E-01 | -0.22 | 0.38 | 325393 | 18743 | 321982 | 19350 | 321084 | 19278 | 322881 | 19575 |
| RP_POS_612_323a10_619 | RP | POS | 10.62 | 612.3230 | | | | 1.50E-03 | 0.60 | 1.34 | 6.45E-01 | -0.09 | 0.75 | 1.32E-02 | 0.56 | 1.11 | 1.41E-03 | 0.65 | 1.41 | 2497434 | 534497 | 2874649 | 627443 | 2903757 | 545773 | 2845540 | 704135 |
| RP_POS_498_90024a0_495 | RP | POS | 0.50 | 498.9002 | | | | 1.87E-07 | 0.63 | 1.34 | 6.45E-01 | -0.10 | 1.20 | 2.97E-04 | 0.57 | 1.41 | 1.88E-04 | 0.68 | 1.44 | 12585656 | 1653792 | 23696776 | 19665018 | 24608471 | 20972319 | 22785081 | 18433216 |
| RP_POS_352_24579a10_713 | RP | POS | 10.71 | 352.2458 | | | | 4.65E-03 | 0.57 | 1.42 | 6.45E-01 | 0.09 | 1.30 | 4.56E-03 | 0.62 | 1.37 | 2.15E-02 | 0.53 | 1.28 | 2438963 | 759720 | 2898124 | 782619 | 2861845 | 846936 | 2934402 | 719372 |
| RP_POS_364_83392a15_454 | RP | POS | 15.45 | 364.8339 | | | | 5.09E-01 | -0.12 | 0.61 | 6.45E-01 | -0.10 | 0.37 | 3.79E-01 | -0.17 | 0.71 | 7.49E-01 | -0.07 | 0.89 | 2342040 | 126991 | 2323904 | 154919 | 2331087 | 181110 | 2316722 | 124834 |
| HILIC_NEG_100_93347a2_175 | HILIC | NEG | 2.175 | 100.9335 | | | | 5.98E-01 | 0.10 | 0.67 | 6.46E-01 | 0.10 | 0.62 | 5.09E-01 | 0.15 | 0.25 | 8.08E-01 | 0.05 | 0.23 | 22387901 | 3911007 | 22832182 | 4722815 | 22613434 | 4360713 | 23050930 | 5094281 |
| RP_POS_635_49567a11_61 | RP | POS | 11.61 | 635.4957 | | | | 5.07E-01 | -0.13 | 0.66 | 6.46E-01 | 0.10 | 0.68 | 6.96E-01 | -0.08 | 0.58 | 4.55E-01 | -0.17 | 0.69 | 418505 | 124448 | 400876 | 145741 | 394127 | 168748 | 407625 | 119773 |
| RP_POS_441_2981a11_831 | RP | POS | 11.83 | 441.2981 | | | | 8.08E-01 | -0.05 | 0.71 | 6.46E-01 | 0.09 | 0.95 | 9.99E-01 | 0.00 | 0.86 | 6.58E-01 | -0.09 | 0.41 | 306171074 | 74419220 | 302387454 | 82090488 | 298587802 | 76527451 | 306187107 | 87916490 |
| RP_POS_880_81769a0_495 | RP | POS | 0.50 | 880.8177 | | | | 2.97E-08 | 0.67 | 1.37 | 6.46E-01 | -0.10 | 1.20 | 1.55E-04 | 0.61 | 1.44 | 6.10E-05 | 0.72 | 1.47 | 144068 | 91467 | 1186491 | 1726697 | 1266409 | 1806920 | 1106573 | 1657051 |
| RP_POS_209_11487a7_603 | RP | POS | 7.60 | 209.1149 | Unknown | 209.1136(100) | 4 | 3.30E-01 | -0.23 | 0.78 | 6.46E-01 | -0.08 | 1.35 | 2.76E-01 | -0.27 | 1.09 | 4.63E-01 | -0.19 | 0.41 | 1789156 | 232625 | 1745208 | 173289 | 1753228 | 183919 | 1737188 | 163442 |
| RP_NEG_1085_23169a9_788 | RP | NEG | 9.79 | 1085.2317 | | | | 4.37E-01 | -0.17 | 0.54 | 6.46E-01 | 0.09 | 0.68 | 6.01E-01 | -0.12 | 0.27 | 3.81E-01 | -0.21 | 0.76 | 3572312 | 332298 | 3520324 | 309718 | 3505992 | 331267 | 3534657 | 289224 |
| RP_POS_503_26837a15_341 | RP | POS | 15.34 | 503.2684 | | | | 2.49E-01 | -0.22 | 0.94 | 6.46E-01 | -0.10 | 0.37 | 2.06E-01 | -0.27 | 0.53 | 4.38E-01 | -0.17 | 0.80 | 1116871 | 335859 | 1034408 | 386359 | 1052265 | 409577 | 1016551 | 364950 |
| RP_POS_375_21933a11_164 | RP | POS | 11.16 | 375.2193 | | | | 5.48E-01 | -0.10 | 0.55 | 6.47E-01 | -0.10 | 0.61 | 4.22E-01 | -0.15 | 0.48 | 8.05E-01 | -0.05 | 0.62 | 3105787 | 188818 | 3079864 | 271066 | 3092384 | 301150 | 3067344 | 239670 |
| RP_POS_362_9256a0_495 | RP | POS | 0.50 | 362.9256 | | | | 2.01E-07 | 0.62 | 1.34 | 6.47E-01 | -0.10 | 1.20 | 3.07E-04 | 0.57 | 1.40 | 1.93E-04 | 0.68 | 1.44 | 24186674 | 2652053 | 41048777 | 29899711 | 42429396 | 31877786 | 39668159 | 28038312 |
| RP_NEG_161_00838a0_617 | RP | NEG | 0.62 | 161.0084 | | | | 2.02E-03 | -0.61 | 1.02 | 6.47E-01 | -0.09 | 0.56 | 2.92E-03 | -0.65 | 1.10 | 1.02E-02 | -0.56 | 0.92 | 659379 | 421566 | 375926 | 459448 | 397139 | 463823 | 354712 | 458740 |
| RP_POS_421_32843a11_612 | RP | POS | 11.61 | 421.3284 | | | | 7.23E-03 | -0.64 | 1.03 | 6.47E-01 | -0.08 | 0.57 | 4.92E-03 | -0.68 | 1.25 | 2.34E-02 | -0.59 | 0.86 | 1944137 | 466495 | 1690960 | 354981 | 1707357 | 422933 | 1674564 | 274178 |
| RP_POS_196_00262a3_885 | RP | POS | 3.89 | 196.0026 | | | | 9.65E-01 | 0.01 | 0.45 | 6.47E-01 | 0.09 | 0.42 | 8.10E-01 | 0.06 | 0.16 | 8.68E-01 | -0.04 | 0.53 | 12954072 | 5736628 | 13005046 | 5766109 | 12738954 | 5650305 | 13271138 | 5924872 |
| RP_POS_413_26593a11_578 | RP | POS | 11.58 | 413.2659 | | | | 2.30E-02 | -0.43 | 0.69 | 6.47E-01 | -0.09 | 0.43 | 3.27E-02 | -0.48 | 0.77 | 6.17E-02 | -0.39 | 0.74 | 10723974 | 945355 | 10257537 | 1097113 | 10308151 | 999940 | 10206923 | 1194475 |
| RP_NEG_283_85654a15_723 | RP | NEG | 15.72 | 283.8565 | | | | 2.25E-01 | -0.25 | 0.48 | 6.47E-01 | -0.09 | 0.87 | 1.80E-01 | -0.30 | 0.64 | 3.86E-01 | -0.21 | 0.39 | 525587 | 239146 | 466195 | 234084 | 476985 | 256904 | 455405 | 210883 |
| RP_POS_133_03459a0_45 | RP | POS | 0.45 | 133.0346 | | | | 8.73E-06 | -0.64 | 1.30 | 6.47E-01 | -0.10 | 1.15 | 1.11E-04 | -0.69 | 1.39 | 1.70E-03 | -0.59 | 1.32 | 1333642 | 231007 | 1028598 | 512660 | 1052218 | 537616 | 1004978 | 490750 |
| RP_NEG_129_0546a3_713 | RP | NEG | 3.71 | 129.0546 | | | | 1.62E-01 | 0.23 | 0.87 | 6.47E-01 | 0.10 | 0.72 | 1.96E-01 | 0.28 | 0.65 | 3.03E-01 | 0.18 | 0.43 | 25745544 | 6857317 | 28012748 | 10532066 | 27527394 | 8597686 | 28498102 | 12235671 |
| HILIC_POS_185_02325a7_963 | HILIC | POS | 7.963 | 185.0233 | | | | 7.94E-04 | -0.52 | 1.21 | 6.47E-01 | 0.10 | 0.82 | 1.14E-02 | -0.47 | 1.30 | 3.18E-03 | -0.57 | 1.27 | 3968033 | 1057545 | 3049474 | 1878013 | 2962977 | 1925162 | 3135972 | 1845081 |
| HILIC_POS_560_31213a1_255 | HILIC | POS | 1.255 | 560.3121 | | | | 6.47E-07 | 0.86 | 1.53 | 6.48E-01 | -0.09 | 0.66 | 1.04E-04 | 0.82 | 1.38 | 1.50E-06 | 0.91 | 1.88 | 770362 | 230512 | 1054825 | 327176 | 1069882 | 285184 | 1039768 | 366715 |
| RP_POS_613_56274a11_25 | RP | POS | 11.25 | 613.5627 | | | | 3.86E-07 | 0.68 | 1.19 | 6.48E-01 | 0.10 | 0.69 | 2.78E-04 | 0.73 | 1.20 | 2.90E-05 | 0.63 | 1.30 | 122202 | 167782 | 420217 | 471253 | 398544 | 384267 | 441889 | 547788 |
| RP_POS_120_32705a0_496 | RP | POS | 0.50 | 120.3271 | | | | 3.71E-07 | 0.61 | 1.33 | 6.48E-01 | -0.10 | 1.20 | 4.59E-04 | 0.56 | 1.39 | 2.41E-04 | 0.66 | 1.43 | 196464 | 32946 | 386171 | 344544 | 402009 | 365253 | 370333 | 325457 |
| RP_POS_196_00294a10_58 | RP | POS | 10.58 | 196.0029 | | | | 4.09E-01 | -0.18 | 0.49 | 6.49E-01 | 0.09 | 0.46 | 5.72E-01 | -0.13 | 0.32 | 3.47E-01 | -0.22 | 0.54 | 2685209 | 1038762 | 2513971 | 927876 | 2471408 | 924539 | 2556535 | 938620 |
| RP_POS_295_22516a9_676 | RP | POS | 9.68 | 295.2252 | | | | 6.03E-03 | 0.49 | 1.07 | 6.49E-01 | 0.10 | 0.63 | 1.22E-02 | 0.54 | 1.03 | 2.57E-02 | 0.44 | 1.17 | 7180572 | 931393 | 7764200 | 1237242 | 7707443 | 1150762 | 7820957 | 1327384 |
| RP_POS_350_1748a10_13 | RP | POS | 10.13 | 350.1748 | | | | 1.45E-01 | 0.22 | 0.99 | 6.49E-01 | 0.10 | 0.76 | 2.24E-01 | 0.27 | 0.80 | 2.49E-01 | 0.17 | 1.00 | 1113885 | 384766 | 1262781 | 752194 | 1228249 | 504273 | 1297313 | 941496 |
| RP_POS_375_28937a9_926 | RP | POS | 9.93 | 375.2894 | | | | 2.71E-02 | 0.34 | 1.25 | 6.49E-01 | 0.10 | 0.35 | 2.46E-02 | 0.39 | 0.96 | 1.57E-01 | 0.29 | 1.18 | 681729 | 645728 | 1050421 | 1181777 | 996264 | 1328450 | 1104579 | 1025150 |
| RP_POS_435_36612a10_881 | RP | POS | 10.88 | 435.3661 | | | | 5.66E-01 | 0.09 | 0.44 | 6.49E-01 | -0.10 | 0.30 | 8.55E-01 | 0.04 | 0.26 | 3.82E-01 | 0.14 | 0.72 | 3911055 | 226882 | 3943268 | 389604 | 3961126 | 285855 | 3925410 | 473620 |
| RP_POS_278_24503a10_731 | RP | POS | 10.73 | 278.2450 | | | | 1.17E-06 | 0.78 | 1.24 | 6.49E-01 | -0.09 | 0.71 | 4.14E-04 | 0.73 | 1.21 | 2.50E-06 | 0.83 | 1.49 | 54972853 | 15651035 | 74816924 | 26066867 | 76010452 | 21811754 | 73623396 | 29902680 |
| HILIC_POS_86_09712a4_005 | HILIC | POS | 4.005 | 86.0971 | | | | 4.35E-01 | 0.14 | 0.88 | 6.50E-01 | -0.10 | 0.95 | 6.86E-01 | 0.09 | 0.46 | 3.37E-01 | 0.18 | 0.55 | 26868440 | 8319300 | 28330771 | 11431987 | 28854033 | 10146236 | 27807509 | 12670552 |
| RP_NEG_208_84506a0_522 | RP | NEG | 0.52 | 208.8451 | | | | 7.39E-04 | 0.45 | 1.19 | 6.50E-01 | -0.10 | 1.09 | 1.89E-02 | 0.40 | 1.16 | 6.39E-03 | 0.50 | 1.31 | 852571 | 118524 | 992285 | 345572 | 1008093 | 360749 | 976478 | 332607 |
| RP_NEG_411_09113a0_708 | RP | NEG | 0.71 | 411.0911 | | | | 8.66E-01 | -0.03 | 0.43 | 6.50E-01 | -0.10 | 0.62 | 7.15E-01 | -0.08 | 0.79 | 9.38E-01 | 0.02 | 0.73 | 1071880 | 213558 | 1064170 | 256753 | 1075908 | 250887 | 1052431 | 264507 |
| RP_NEG_957_20624a9_894 | RP | NEG | 9.89 | 957.2062 | | | | 5.97E-01 | -0.11 | 0.41 | 6.50E-01 | 0.09 | 0.52 | 7.84E-01 | -0.06 | 0.28 | 4.98E-01 | -0.15 | 0.41 | 3944714 | 275351 | 3915063 | 272151 | 3902621 | 268735 | 3927505 | 277686 |
| RP_POS_188_12819a4_741 | RP | POS | 4.74 | 188.1282 | | | | 8.36E-01 | -0.03 | 0.48 | 6.50E-01 | 0.10 | 0.49 | 9.50E-01 | 0.01 | 0.51 | 5.87E-01 | -0.08 | 0.68 | 1258792 | 1126796 | 1204303 | 1697472 | 1126590 | 968642 | 1282015 | 2207043 |
| RP_POS_583_44031a10_461 | RP | POS | 10.46 | 583.4403 | | | | 1.62E-01 | -0.29 | 0.62 | 6.50E-01 | 0.09 | 0.69 | 2.93E-01 | -0.24 | 0.60 | 1.44E-01 | -0.33 | 0.57 | 1367848 | 163154 | 1320741 | 164941 | 1313212 | 163541 | 1328269 | 167644 |
| RP_POS_373_20407a11_254 | RP | POS | 11.25 | 373.2041 | | | | 2.61E-04 | 0.69 | 1.52 | 6.51E-01 | 0.09 | 1.10 | 8.81E-04 | 0.73 | 1.47 | 1.58E-03 | 0.64 | 1.39 | 7164229 | 1409404 | 8329603 | 1684405 | 8252758 | 1543361 | 8406447 | 1827108 |
| RP_POS_136_07356a3_319 | RP | POS | 3.32 | 136.0736 | | | | 4.87E-05 | -0.63 | 1.26 | 6.51E-01 | 0.10 | 1.12 | 1.56E-03 | -0.58 | 1.27 | 5.06E-04 | -0.68 | 1.31 | 24384568 | 4819241 | 19220905 | 8643208 | 18826656 | 8998457 | 19615154 | 8345372 |
| RP_NEG_411_65292a15_581 | RP | NEG | 15.58 | 411.6529 | | | | 1.60E-01 | -0.28 | 0.73 | 6.51E-01 | -0.09 | 0.30 | 1.33E-01 | -0.32 | 0.64 | 3.12E-01 | -0.23 | 0.59 | 751933 | 37749 | 740815 | 40654 | 742667 | 43761 | 738963 | 37646 |
| RP_POS_644_83203a0_472 | RP | POS | 0.47 | 644.8320 | | | | 1.08E-07 | -0.67 | 1.34 | 6.51E-01 | 0.10 | 1.17 | 2.95E-04 | -0.62 | 1.38 | 4.80E-05 | -0.72 | 1.46 | 640626 | 65571 | 470323 | 278707 | 457634 | 282394 | 483011 | 277245 |
| HILIC_POS_323_21759a4_834 | HILIC | POS | 4.834 | 323.2176 | | | | 2.44E-05 | 0.59 | 0.99 | 6.52E-01 | 0.10 | 0.42 | 3.28E-03 | 0.64 | 0.93 | 6.11E-05 | 0.54 | 1.32 | 412440 | 193546 | 667245 | 469062 | 645887 | 305864 | 688604 | 591647 |
| HILIC_POS_267_14359a0_537 | HILIC | POS | 0.537 | 267.1436 | | | | 7.93E-02 | -0.34 | 0.81 | 6.52E-01 | 0.09 | 0.81 | 1.88E-01 | -0.29 | 0.58 | 7.42E-02 | -0.38 | 0.64 | 557021 | 60794 | 534171 | 69435 | 531014 | 67249 | 537329 | 72098 |
| RP_POS_408_30804a11_129 | RP | POS | 11.13 | 408.3080 | | | | 1.95E-02 | 0.38 | 0.82 | 6.52E-01 | -0.10 | 0.35 | 6.78E-02 | 0.33 | 0.70 | 3.98E-02 | 0.43 | 0.79 | 11852717 | 13519131 | 19498316 | 21449375 | 20473592 | 23759444 | 18523040 | 19054878 |
| RP_NEG_583_17444a0_621 | RP | NEG | 0.62 | 583.1744 | | | | 6.93E-02 | -0.31 | 0.89 | 6.52E-01 | -0.10 | 0.92 | 6.84E-02 | -0.36 | 0.89 | 2.03E-01 | -0.26 | 0.93 | 2563952 | 942653 | 2169996 | 1339106 | 2230862 | 1405636 | 2109129 | 1280481 |
| RP_POS_288_92184a0_469 | RP | POS | 0.47 | 288.9218 | | | | 5.35E-09 | -0.72 | 1.39 | 6.52E-01 | 0.10 | 1.16 | 7.94E-05 | -0.68 | 1.45 | 1.05E-05 | -0.77 | 1.52 | 3663335 | 323503 | 2575691 | 1630754 | 2501608 | 1643703 | 2649775 | 1630960 |
| HILIC_NEG_117_91909a5_424 | HILIC | NEG | 5.424 | 117.9191 | | | | 1.78E-01 | 0.27 | 0.64 | 6.52E-01 | -0.09 | 0.62 | 3.02E-01 | 0.23 | 0.51 | 1.69E-01 | 0.32 | 0.68 | 2048147 | 461056 | 2176301 | 466964 | 2197506 | 494361 | 2155096 | 441870 |
| RP_POS_1017_68683a10_502 | RP | POS | 10.50 | 1017.6868 | | | | 2.11E-03 | 0.60 | 1.39 | 6.52E-01 | -0.09 | 0.86 | 1.74E-02 | 0.56 | 1.12 | 1.44E-03 | 0.65 | 1.43 | 15999210 | 7488085 | 21033924 | 8325188 | 21411994 | 6611993 | 20655855 | 9799072 |
| RP_NEG_425_36435a11_739 | RP | NEG | 11.74 | 425.3644 | | | | 2.31E-02 | -0.38 | 0.89 | 6.52E-01 | 0.10 | 0.69 | 9.04E-02 | -0.33 | 0.86 | 3.41E-02 | -0.43 | 0.79 | 980242 | 169759 | 888698 | 256900 | 877041 | 263666 | 900356 | 252082 |
| RP_POS_306_27698a11_164 | RP | POS | 11.16 | 306.2770 | | | | 1.07E-11 | 1.11 | 1.76 | 6.52E-01 | -0.09 | 0.55 | 8.98E-08 | 1.07 | 1.71 | 3.10E-10 | 1.15 | 1.94 | 41281320 | 13479695 | 66642426 | 21814208 | 67631939 | 19679622 | 65652912 | 23919553 |
| RP_POS_914_81097a0_494 | RP | POS | 0.49 | 914.8110 | | | | 2.29E-08 | 0.67 | 1.36 | 6.53E-01 | -0.10 | 1.18 | 1.36E-04 | 0.62 | 1.43 | 5.39E-05 | 0.72 | 1.47 | 99972 | 65687 | 972660 | 1433000 | 1037567 | 1497169 | 907753 | 1377960 |
| RP_POS_788_53479a13_155 | RP | POS | 13.16 | 788.5348 | | | | 3.94E-02 | -0.47 | 0.81 | 6.53E-01 | -0.08 | 0.27 | 3.88E-02 | -0.51 | 0.91 | 7.89E-02 | -0.43 | 0.71 | 1622652 | 555763 | 1392809 | 456210 | 1413466 | 447503 | 1372151 | 468373 |
| HILIC_NEG_136_89166a5_496 | HILIC | NEG | 5.496 | 136.8917 | | | | 3.01E-01 | -0.22 | 0.58 | 6.53E-01 | -0.09 | 0.42 | 2.48E-01 | -0.27 | 0.68 | 4.64E-01 | -0.18 | 0.35 | 4107743 | 1887973 | 3714437 | 1745339 | 3793470 | 1878622 | 3635404 | 1616359 |
| RP_POS_185_11488a11_219 | RP | POS | 11.22 | 185.1149 | | | | 9.06E-03 | -0.75 | 1.63 | 6.53E-01 | -0.06 | 0.38 | 9.25E-03 | -0.78 | 1.20 | 1.28E-02 | -0.72 | 1.47 | 3017490 | 935430 | 2544831 | 444042 | 2564917 | 359549 | 2524744 | 517950 |

**Supplementary table 1: Metabolomics results** (in asceding order according to p-value from progressive ALD vs  non-progressive ALD comparison, with molecular features with ID_level of 1-2 first)

| | | | | | | | | all_ALD_vs_control | | | Prog_ALD_vs_non-prog_ALD | | | Prog_ALD_vs_control_ALD | | | Non-prog_ALD_vs_control | | | Healthy_control | | all_ALD | | Non-prog_ALD | | Progressive_ALD | |
|---|---|---|---|---|---|---|---|---|---|---|---|---|---|---|---|---|---|---|---|---|---|---|---|---|---|---|---|
| | | | | | | | | t-test | Cohen's | PLS-DA | t-test | Cohen's | PLS-DA | t-test | Cohen's | PLS-DA | t-test | Cohen's | PLS-DA | | | | | | | | |
| Feature_ID | RP_HILIC | POS_NEG | Average.Rt | Average.Mz | Metabolite.name | curated_MSMS | ID_level | p | d | VIP | p | d | VIP | p | d | VIP | p | d | VIP | Mean | SD | Mean | SD | Mean | SD | Mean | SD |
| HILIC_NEG_193_8157a2_95 | HILIC | NEG | 2.95 | 193.8157 | | | | 8.12E-07 | 0.61 | 1.31 | 6.54E-01 | -0.10 | 0.78 | 7.47E-04 | 0.56 | 1.42 | 2.28E-04 | 0.66 | 1.51 | 880220 | 90448 | 1143640 | 479567 | 1165316 | 497526 | 1121964 | 464943 |
| RP_NEG_530_27368a13_287 | RP | NEG | 13.29 | 530.2737 | | | | 7.42E-01 | -0.06 | 0.59 | 6.54E-01 | -0.09 | 0.48 | 6.22E-01 | -0.11 | 0.72 | 9.36E-01 | -0.02 | 0.38 | 307355 | 69577 | 302541 | 77469 | 306042 | 74507 | 299039 | 80926 |
| RP_POS_228_1958a11_038 | RP | POS | 11.04 | 228.1958 | | | | 1.31E-02 | 0.40 | 0.80 | 6.54E-01 | -0.10 | 0.33 | 5.09E-02 | 0.35 | 0.71 | 3.11E-02 | 0.45 | 0.77 | 18800639 | 24406067 | 33659706 | 39768792 | 35456924 | 44152619 | 31862489 | 35204328 |
| RP_NEG_882_7652a0_499 | RP | NEG | 0.50 | 882.7652 | | | | 6.50E-03 | -0.40 | 1.14 | 6.54E-01 | -0.10 | 1.23 | 1.38E-02 | -0.45 | 1.16 | 6.10E-02 | -0.35 | 1.19 | 885652 | 201229 | 734517 | 412372 | 753148 | 423602 | 715886 | 404259 |
| RP_POS_756_55237a12_762 | RP | POS | 12.76 | 756.5524 | | | | 4.45E-02 | 0.36 | 0.73 | 6.54E-01 | 0.09 | 0.30 | 4.84E-02 | 0.40 | 0.78 | 1.38E-01 | 0.31 | 0.62 | 47889311 | 24946535 | 59194930 | 33279157 | 57692118 | 34154797 | 60697742 | 32656506 |
| RP_NEG_289_8981a3_705 | RP | NEG | 3.71 | 289.8981 | | | | 1.44E-01 | 0.24 | 0.80 | 6.54E-01 | 0.10 | 0.59 | 1.83E-01 | 0.29 | 0.62 | 2.81E-01 | 0.19 | 0.42 | 1071673 | 402658 | 1213682 | 646844 | 1184474 | 534667 | 1242889 | 746822 |
| HILIC_POS_124_087a6_286 | HILIC | POS | 6.286 | 124.0870 | | | | 3.29E-01 | 0.18 | 0.43 | 6.54E-01 | -0.09 | 0.49 | 5.54E-01 | 0.13 | 0.29 | 2.67E-01 | 0.22 | 0.46 | 1317829 | 746618 | 1478387 | 962247 | 1521818 | 889855 | 1434955 | 1036843 |
| RP_POS_636_87848a0_495 | RP | POS | 0.50 | 636.8785 | | | | 1.14E-07 | 0.65 | 1.34 | 6.54E-01 | -0.10 | 1.19 | 3.06E-04 | 0.60 | 1.40 | 9.09E-05 | 0.70 | 1.45 | 106467 | 72084 | 392745 | 488587 | 414793 | 505897 | 370697 | 474744 |
| RP_POS_318_60794a7_093 | RP | POS | 7.09 | 318.6079 | | | | 5.57E-01 | -0.12 | 0.57 | 6.54E-01 | 0.09 | 0.48 | 7.39E-01 | -0.08 | 0.33 | 4.73E-01 | -0.17 | 0.66 | 3566913 | 233389 | 3538878 | 233430 | 3528349 | 240508 | 3549407 | 228081 |
| HILIC_NEG_301_78824a1_363 | HILIC | NEG | 1.363 | 301.7882 | | | | 4.36E-10 | 0.79 | 1.21 | 6.54E-01 | 0.10 | 0.72 | 2.89E-05 | 0.84 | 1.29 | 3.54E-07 | 0.74 | 1.58 | 81901 | 45613 | 223539 | 192812 | 214844 | 154539 | 232233 | 225997 |
| HILIC_POS_406_16391a0_889 | HILIC | POS | 0.889 | 406.1639 | | | | 1.24E-03 | 0.44 | 0.81 | 6.54E-01 | 0.10 | 0.63 | 2.20E-02 | 0.49 | 0.83 | 3.52E-03 | 0.39 | 1.16 | 4868349 | 1430069 | 6367339 | 3766775 | 6197409 | 2561841 | 6537270 | 4695189 |
| RP_NEG_498_78397a15_518 | RP | NEG | 15.52 | 498.7840 | | | | 2.06E-01 | -0.21 | 0.61 | 6.55E-01 | 0.10 | 0.42 | 3.22E-01 | -0.16 | 0.42 | 2.61E-01 | -0.26 | 0.63 | 1576603 | 43912 | 1563407 | 68503 | 1560317 | 85723 | 1566497 | 45967 |
| RP_POS_848_79364a0_474 | RP | POS | 0.47 | 848.7936 | | | | 2.17E-08 | -0.72 | 1.37 | 6.55E-01 | 0.10 | 1.16 | 9.62E-05 | -0.67 | 1.43 | 1.62E-05 | -0.77 | 1.50 | 412738 | 50216 | 294745 | 176656 | 286786 | 179308 | 302705 | 175416 |
| HILIC_POS_76_03998a5_922 | HILIC | POS | 5.922 | 76.0400 | | | | 5.21E-01 | -0.12 | 1.04 | 6.55E-01 | 0.09 | 0.82 | 7.56E-01 | -0.07 | 0.76 | 4.12E-01 | -0.16 | 1.16 | 858655 | 287535 | 817964 | 372270 | 801199 | 336568 | 834730 | 407612 |
| RP_POS_356_29224a11_155 | RP | POS | 11.16 | 356.2922 | | | | 6.48E-01 | -0.09 | 0.57 | 6.55E-01 | -0.09 | 0.30 | 5.46E-01 | -0.14 | 0.35 | 8.29E-01 | -0.05 | 0.31 | 1667789 | 1237837 | 1552972 | 1214314 | 1607631 | 1208140 | 1498312 | 1230249 |
| RP_POS_406_29245a10_818 | RP | POS | 10.82 | 406.2925 | | | | 3.52E-04 | 0.53 | 1.12 | 6.55E-01 | 0.10 | 0.40 | 1.89E-03 | 0.58 | 1.17 | 9.15E-03 | 0.48 | 1.00 | 159920 | 188604 | 345841 | 377257 | 328864 | 379327 | 362817 | 378249 |
| RP_POS_340_3577a12_1 | RP | POS | 12.10 | 340.3577 | | | | 8.30E-01 | -0.04 | 0.59 | 6.55E-01 | -0.09 | 0.27 | 6.80E-01 | -0.09 | 0.31 | 9.82E-01 | 0.01 | 0.38 | 4352368 | 2832729 | 4223994 | 3211938 | 4368482 | 3422113 | 4079506 | 3014990 |
| RP_POS_352_8967a0_514 | RP | POS | 0.51 | 352.8967 | | | | 2.88E-06 | 0.56 | 1.26 | 6.55E-01 | -0.10 | 1.14 | 1.60E-03 | 0.51 | 1.26 | 5.28E-04 | 0.61 | 1.40 | 385225 | 99100 | 784037 | 788284 | 819464 | 820733 | 748611 | 761119 |
| RP_POS_390_77277a13_152 | RP | POS | 13.15 | 390.7728 | | | | 3.83E-01 | 0.18 | 0.85 | 6.56E-01 | -0.09 | 0.85 | 5.61E-01 | 0.14 | 0.88 | 3.13E-01 | 0.23 | 0.60 | 3017047 | 1055490 | 3204167 | 1015638 | 3249736 | 937534 | 3158597 | 1095850 |
| RP_POS_343_33142a9_618 | RP | POS | 9.62 | 343.3314 | | | | 6.64E-01 | -0.11 | 0.85 | 6.56E-01 | -0.08 | 0.28 | 5.75E-01 | -0.15 | 0.64 | 7.93E-01 | -0.07 | 0.56 | 1218716 | 1426185 | 1100824 | 939697 | 1142932 | 967419 | 1058716 | 919001 |
| HILIC_NEG_315_04166a7_071 | HILIC | NEG | 7.071 | 315.0417 | | | | 7.04E-01 | -0.07 | 0.62 | 6.57E-01 | 0.09 | 0.64 | 9.02E-01 | -0.03 | 0.17 | 5.75E-01 | -0.12 | 0.37 | 1765193 | 371170 | 1735588 | 411569 | 1717154 | 385582 | 1754022 | 439168 |
| RP_POS_161_96875a0_25 | RP | POS | 0.25 | 161.9688 | | | | 1.68E-02 | -0.49 | 0.98 | 6.57E-01 | -0.09 | 0.32 | 1.91E-02 | -0.53 | 1.03 | 4.77E-02 | -0.45 | 0.83 | 918743 | 146846 | 844956 | 147835 | 851575 | 147361 | 838337 | 149506 |
| RP_POS_243_19542a11_038 | RP | POS | 11.04 | 243.1954 | | | | 1.17E-02 | 0.40 | 0.81 | 6.57E-01 | -0.10 | 0.33 | 4.66E-02 | 0.35 | 0.72 | 3.06E-02 | 0.45 | 0.77 | 1248629 | 1624708 | 2270395 | 2740219 | 2392981 | 3061383 | 2147810 | 2401689 |
| RP_POS_267_17059a9_425 | RP | POS | 9.43 | 267.1706 | | | | 3.57E-01 | -0.20 | 0.60 | 6.57E-01 | -0.09 | 0.41 | 2.63E-01 | -0.25 | 0.63 | 5.40E-01 | -0.16 | 0.32 | 777092 | 105162 | 757786 | 92463 | 761922 | 114458 | 753650 | 64326 |
| HILIC_POS_189_07312a5_648 | HILIC | POS | 5.648 | 189.0731 | | | | 2.73E-01 | -0.21 | 1.00 | 6.57E-01 | -0.09 | 0.76 | 2.47E-01 | -0.26 | 0.61 | 4.41E-01 | -0.17 | 0.85 | 5096605 | 985944 | 4868476 | 1103698 | 4917791 | 1071836 | 4819160 | 1143400 |
| RP_POS_316_11163a0_551 | RP | POS | 0.55 | 316.1116 | | | | 9.34E-01 | -0.01 | 1.04 | 6.57E-01 | -0.10 | 0.85 | 7.59E-01 | -0.06 | 0.97 | 8.50E-01 | 0.04 | 1.05 | 820399 | 203718 | 816333 | 334389 | 831273 | 316876 | 801393 | 353621 |
| HILIC_NEG_206_10359a4_741 | HILIC | NEG | 4.741 | 206.1036 | | | | 4.32E-04 | 0.56 | 1.00 | 6.57E-01 | -0.09 | 0.37 | 1.76E-02 | 0.51 | 0.78 | 2.93E-04 | 0.60 | 1.24 | 983014 | 224661 | 1185309 | 386057 | 1202562 | 297753 | 1168056 | 460280 |
| RP_POS_647_26917a11_805 | RP | POS | 11.81 | 647.2692 | | | | 4.41E-03 | -0.57 | 1.25 | 6.58E-01 | 0.09 | 1.36 | 2.85E-02 | -0.52 | 0.93 | 2.78E-03 | -0.61 | 1.32 | 1106870 | 417729 | 850323 | 449357 | 830253 | 344187 | 870393 | 537286 |
| RP_POS_530_32397a10_684 | RP | POS | 10.68 | 530.3240 | | | | 4.18E-15 | 1.04 | 1.84 | 6.58E-01 | 0.09 | 0.30 | 2.18E-07 | 1.09 | 1.74 | 1.53E-12 | 1.00 | 2.13 | 817850 | 388562 | 2034673 | 1182134 | 1981897 | 839530 | 2087449 | 1453585 |
| HILIC_POS_319_16495a1_44 | HILIC | POS | 1.44 | 319.1650 | | | | 5.51E-03 | 0.41 | 0.71 | 6.58E-01 | -0.10 | 0.44 | 6.97E-02 | 0.36 | 0.65 | 6.98E-03 | 0.46 | 0.88 | 937915 | 575240 | 1375068 | 1160451 | 1426844 | 1020017 | 1323293 | 1294167 |
| RP_POS_383_35159a11_227 | RP | POS | 11.23 | 383.3516 | | | | 6.52E-02 | -0.35 | 0.95 | 6.58E-01 | 0.09 | 0.96 | 1.43E-01 | -0.31 | 0.89 | 7.48E-02 | -0.40 | 0.90 | 574227 | 142962 | 517726 | 162891 | 510461 | 174193 | 524991 | 152177 |
| HILIC_NEG_207_83672a2_943 | HILIC | NEG | 2.943 | 207.8367 | | | | 3.83E-03 | 0.47 | 1.08 | 6.58E-01 | -0.09 | 0.80 | 2.66E-02 | 0.42 | 0.83 | 9.41E-03 | 0.52 | 1.00 | 432072 | 86608 | 493470 | 138493 | 499643 | 143245 | 487298 | 134741 |
| HILIC_NEG_136_89162a2_952 | HILIC | NEG | 2.952 | 136.8916 | | | | 3.89E-03 | 0.43 | 1.11 | 6.58E-01 | -0.10 | 0.81 | 3.52E-02 | 0.39 | 0.91 | 1.16E-02 | 0.48 | 1.10 | 51911865 | 8092531 | 58176821 | 15661951 | 58874319 | 16117077 | 57479324 | 15324701 |
| RP_POS_1083_92676a15_101 | RP | POS | 15.10 | 1083.9268 | | | | 6.30E-01 | -0.10 | 0.21 | 6.58E-01 | -0.09 | 0.27 | 5.31E-01 | -0.14 | 0.31 | 8.15E-01 | -0.05 | 0.29 | 7407742 | 1021631 | 7306568 | 1042025 | 7352960 | 1038748 | 7260177 | 1053757 |
| RP_POS_780_8056a0_472 | RP | POS | 0.47 | 780.8056 | | | | 3.60E-08 | -0.68 | 1.35 | 6.58E-01 | 0.10 | 1.16 | 1.79E-04 | -0.63 | 1.41 | 3.37E-05 | -0.73 | 1.47 | 510273 | 45011 | 369581 | 225109 | 359560 | 229086 | 379602 | 222927 |
| RP_POS_459_35532a9_778 | RP | POS | 9.78 | 459.3553 | | | | 4.11E-02 | -0.38 | 0.64 | 6.59E-01 | 0.09 | 0.32 | 1.39E-01 | -0.33 | 0.57 | 3.29E-02 | -0.43 | 0.90 | 795777 | 58630 | 769549 | 71269 | 766378 | 61143 | 772721 | 80643 |
| RP_POS_688_31476a5_683 | RP | POS | 5.68 | 688.3148 | | | | 6.91E-01 | 0.07 | 0.64 | 6.59E-01 | 0.09 | 0.47 | 5.15E-01 | 0.12 | 0.65 | 9.09E-01 | 0.03 | 0.45 | 951069 | 481278 | 992324 | 585504 | 966281 | 724700 | 1018367 | 407494 |
| HILIC_NEG_473_53348a1_365 | HILIC | NEG | 1.365 | 473.5335 | | | | 3.84E-01 | -0.14 | 0.44 | 6.59E-01 | -0.10 | 0.37 | 3.70E-01 | -0.18 | 0.39 | 6.22E-01 | -0.09 | 0.47 | 224698 | 78938 | 207439 | 139808 | 213652 | 123408 | 201225 | 155509 |
| RP_POS_371_2092a11_254 | RP | POS | 11.25 | 371.2092 | | | | 2.22E-04 | 0.70 | 1.50 | 6.59E-01 | 0.09 | 1.04 | 7.87E-04 | 0.74 | 1.45 | 1.34E-03 | 0.65 | 1.40 | 19857360 | 3855359 | 23081083 | 4587961 | 22877195 | 4196359 | 23284972 | 4983381 |
| HILIC_NEG_183_9342a5_607 | HILIC | NEG | 5.607 | 183.9342 | | | | 1.45E-01 | -0.26 | 1.01 | 6.59E-01 | 0.09 | 0.71 | 3.27E-01 | -0.21 | 0.41 | 1.25E-01 | -0.30 | 1.09 | 1001381 | 123662 | 960777 | 167848 | 953319 | 155432 | 968234 | 180688 |
| RP_POS_338_26651a9_152 | RP | POS | 9.15 | 338.2665 | | | | 8.07E-02 | 0.31 | 0.55 | 6.59E-01 | 0.09 | 0.43 | 1.25E-01 | 0.36 | 0.66 | 1.37E-01 | 0.26 | 0.61 | 985827 | 1469854 | 1561057 | 1943265 | 1474721 | 1371548 | 1647394 | 2394424 |
| RP_POS_381_98285a0_627 | RP | POS | 0.63 | 381.9829 | | | | 3.66E-10 | -0.77 | 1.43 | 6.60E-01 | 0.10 | 1.17 | 9.29E-06 | -0.72 | 1.54 | 7.53E-06 | -0.82 | 1.52 | 1756713 | 93317 | 1336415 | 588934 | 1310303 | 624240 | 1362527 | 556506 |
| RP_POS_598_83228a0_497 | RP | POS | 0.50 | 598.8323 | | | | 3.21E-08 | -0.67 | 1.37 | 6.60E-01 | 0.10 | 1.20 | 1.27E-04 | -0.62 | 1.45 | 6.70E-05 | -0.72 | 1.46 | 13043307 | 645209 | 9684501 | 5514469 | 9440411 | 5809520 | 9928591 | 5250449 |
| RP_POS_168_98062a15_4 | RP | POS | 15.40 | 168.9806 | | | | 9.29E-01 | 0.02 | 0.51 | 6.61E-01 | 0.09 | 0.35 | 7.92E-01 | 0.06 | 0.64 | 9.25E-01 | -0.02 | 0.38 | 42250512 | 20555838 | 42612191 | 17888748 | 41821020 | 19044880 | 43403363 | 16809535 |
| RP_POS_350_89117a0_465 | RP | POS | 0.47 | 350.8912 | | | | 1.90E-06 | -0.63 | 1.29 | 6.61E-01 | 0.10 | 1.14 | 8.27E-04 | -0.58 | 1.32 | 1.64E-04 | -0.68 | 1.40 | 580556 | 93139 | 416847 | 283802 | 404300 | 288409 | 429394 | 281478 |
| RP_POS_800_57819a11_745 | RP | POS | 11.75 | 800.5782 | | | | 5.22E-01 | 0.09 | 0.42 | 6.61E-01 | -0.10 | 0.42 | 7.98E-01 | 0.05 | 0.22 | 4.56E-01 | 0.14 | 0.47 | 1443999 | 1973788 | 1782840 | 3961564 | 1957982 | 4181854 | 1607699 | 3762680 |
| RP_POS_244_19063a11_127 | RP | POS | 11.13 | 244.1906 | | | | 1.78E-02 | 0.39 | 0.76 | 6.61E-01 | -0.09 | 0.31 | 6.60E-02 | 0.34 | 0.69 | 3.45E-02 | 0.44 | 0.76 | 3273657 | 4497028 | 5833289 | 6991408 | 6142076 | 7568391 | 5524502 | 6425106 |
| RP_POS_256_22693a11_476 | RP | POS | 11.48 | 256.2269 | | | | 8.19E-03 | 0.42 | 0.82 | 6.61E-01 | -0.10 | 0.32 | 3.63E-02 | 0.37 | 0.74 | 2.45E-02 | 0.46 | 0.80 | 243683 | 340600 | 470764 | 585881 | 496638 | 653523 | 444891 | 514840 |
| RP_POS_687_54352a12_091 | RP | POS | 12.09 | 687.5435 | | | | 3.33E-08 | -1.15 | 1.98 | 6.61E-01 | -0.08 | 0.36 | 5.01E-08 | -1.19 | 2.06 | 7.65E-07 | -1.11 | 1.76 | 779781 | 281665 | 409577 | 280815 | 421969 | 305108 | 397185 | 256747 |
| RP_POS_219_09738a0_706 | RP | POS | 0.71 | 219.0974 | | | | 2.92E-01 | 0.18 | 0.70 | 6.61E-01 | -0.09 | 0.68 | 5.19E-01 | 0.13 | 0.69 | 2.47E-01 | 0.23 | 0.71 | 303680 | 168766 | 344018 | 236037 | 354429 | 224101 | 333607 | 249241 |
| RP_POS_245_03481a3_316 | RP | POS | 3.32 | 245.0348 | | | | 1.41E-05 | -0.66 | 1.27 | 6.61E-01 | 0.09 | 1.09 | 7.24E-04 | -0.61 | 1.31 | 2.79E-04 | -0.70 | 1.30 | 3495153 | 748113 | 2606705 | 1429404 | 2543662 | 1497991 | 2669748 | 1369672 |
| RP_POS_298_27408a9_153 | RP | POS | 9.15 | 298.2741 | | | | 2.23E-01 | 0.22 | 0.46 | 6.62E-01 | 0.09 | 0.44 | 2.63E-01 | 0.27 | 0.58 | 3.19E-01 | 0.18 | 0.47 | 360896 | 640259 | 530974 | 794640 | 495935 | 511634 | 566014 | 1005742 |
| RP_POS_485_6207a6_73 | RP | POS | 6.73 | 485.6207 | | | | 7.86E-07 | 0.59 | 1.32 | 6.62E-01 | -0.10 | 1.14 | 8.63E-04 | 0.54 | 1.38 | 2.82E-04 | 0.64 | 1.40 | 44625 | 109565 | 647138 | 1130084 | 696906 | 1173296 | 597371 | 1094790 |
| RP_POS_286_20114a5_285 | RP | POS | 5.29 | 286.2011 | | | | 3.14E-01 | -0.20 | 0.56 | 6.62E-01 | -0.09 | 0.54 | 3.06E-01 | -0.24 | 0.53 | 4.60E-01 | -0.15 | 0.63 | 2077422 | 1218104 | 1818804 | 1356243 | 1878547 | 1121829 | 1759061 | 1565422 |
| HILIC_NEG_146_08212a4_741 | HILIC | NEG | 4.741 | 146.0821 | | | | 5.44E-05 | 0.63 | 1.19 | 6.62E-01 | -0.09 | 0.50 | 5.70E-03 | 0.59 | 0.90 | 4.59E-05 | 0.68 | 1.40 | 412391 | 115911 | 534303 | 202597 | 543225 | 157965 | 525380 | 240443 |
| RP_POS_632_82983a0_496 | RP | POS | 0.50 | 632.8298 | | | | 1.14E-09 | -0.74 | 1.41 | 6.62E-01 | 0.10 | 1.17 | 2.82E-05 | -0.69 | 1.50 | 1.04E-05 | -0.79 | 1.51 | 2402902 | 116072 | 1712157 | 1014178 | 1667512 | 1052894 | 1756801 | 982574 |
| HILIC_POS_352_24512a5_648 | HILIC | POS | 5.648 | 352.2451 | | | | 9.78E-01 | -0.01 | 0.83 | 6.62E-01 | -0.09 | 0.75 | 8.09E-01 | -0.05 | 0.36 | 8.43E-01 | 0.04 | 0.46 | 946317 | 177564 | 945230 | 224987 | 955132 | 220915 | 935327 | 230798 |
| HILIC_NEG_144_92352a4_796 | HILIC | NEG | 4.796 | 144.9235 | | | | 2.71E-01 | -0.23 | 0.76 | 6.62E-01 | -0.09 | 0.51 | 2.36E-01 | -0.27 | 0.51 | 4.25E-01 | -0.18 | 0.54 | 61419338 | 19732658 | 56979822 | 19385465 | 57832484 | 19718957 | 56127161 | 19207831 |
| RP_POS_898_55426a13_362 | RP | POS | 13.36 | 898.5543 | | | | 6.76E-03 | 0.58 | 1.07 | 6.63E-01 | 0.08 | 0.32 | 7.36E-03 | 0.62 | 1.08 | 2.20E-02 | 0.54 | 0.99 | 700320 | 494743 | 980003 | 462632 | 959671 | 477745 | 1000335 | 450933 |
| RP_NEG_271_19229a9_269 | RP | NEG | 9.27 | 271.1923 | Unknown | 271.1903(100), 253.1806(55), 209.191(45) | 4 | 8.22E-01 | -0.05 | 0.46 | 6.63E-01 | -0.09 | 0.33 | 6.96E-01 | -0.09 | 0.28 | 9.83E-01 | -0.01 | 0.41 | 2574871 | 438604 | 2555082 | 402822 | 2572785 | 415921 | 2537380 | 392700 |
| HILIC_NEG_98_93899a5_43 | HILIC | NEG | 5.43 | 98.9390 | | | | 9.74E-01 | -0.01 | 0.33 | 6.63E-01 | -0.09 | 0.98 | 8.17E-01 | -0.05 | 0.35 | 8.62E-01 | 0.04 | 0.33 | 2537595 | 361715 | 2535176 | 395159 | 2552519 | 399153 | 2517834 | 394399 |
| RP_POS_407_07483a8_977 | RP | POS | 8.98 | 407.0748 | | | | 1.78E-01 | -0.30 | 0.75 | 6.63E-01 | 0.08 | 0.47 | 2.85E-01 | -0.25 | 0.69 | 1.63E-01 | -0.34 | 0.76 | 1752155 | 158671 | 1709310 | 139683 | 1703180 | 144610 | 1715440 | 135766 |
| RP_POS_210_11011a4_742 | RP | POS | 4.74 | 210.1101 | | | | 9.68E-01 | -0.01 | 0.51 | 6.63E-01 | 0.10 | 0.50 | 8.61E-01 | 0.04 | 0.56 | 7.20E-01 | -0.05 | 0.54 | 1682986 | 1665953 | 1667281 | 2596907 | 1553180 | 1462592 | 1781383 | 3385224 |
| RP_POS_239_06834a0_494 | RP | POS | 0.49 | 239.0683 | | | | 1.39E-04 | -0.48 | 1.23 | 6.63E-01 | 0.10 | 1.17 | 6.92E-03 | -0.43 | 1.27 | 3.91E-03 | -0.53 | 1.31 | 1875740 | 209932 | 1532017 | 792154 | 1497268 | 850715 | 1566766 | 735964 |
| HILIC_POS_210_88203a2_992 | HILIC | POS | 2.992 | 210.8820 | | | | 3.08E-05 | -0.58 | 1.34 | 6.63E-01 | 0.10 | 0.79 | 2.19E-03 | -0.53 | 1.40 | 8.46E-04 | -0.62 | 1.43 | 944911 | 166356 | 724091 | 417091 | 705800 | 437276 | 742382 | 399484 |
| RP_POS_259_19016a11_128 | RP | POS | 11.13 | 259.1902 | | | | 1.03E-02 | 0.41 | 0.87 | 6.63E-01 | -0.09 | 0.28 | 4.71E-02 | 0.36 | 0.73 | 2.64E-02 | 0.45 | 0.80 | 585880 | 347525 | 809931 | 595622 | 836045 | 649980 | 783816 | 541208 |
| RP_POS_226_95065a0_495 | RP | POS | 0.50 | 226.9507 | | | | 2.62E-07 | 0.62 | 1.33 | 6.64E-01 | -0.10 | 1.20 | 3.18E-04 | 0.57 | 1.40 | 2.37E-04 | 0.67 | 1.43 | 109842252 | 9429737 | 164844432 | 98459454 | 169157261 | 105286768 | 160531604 | 91995145 |
| HILIC_NEG_144_92349a8_036 | HILIC | NEG | 8.036 | 144.9235 | | | | 3.35E-02 | -0.40 | 0.83 | 6.64E-01 | -0.09 | 1.27 | 4.87E-02 | -0.44 | 0.69 | 8.18E-02 | -0.35 | 0.68 | 35256866 | 12104407 | 29612319 | 14702590 | 30256327 | 13113102 | 28968310 | 16246353 |
| RP_POS_321_15692a10_13 | RP | POS | 10.13 | 321.1569 | | | | 3.97E-01 | 0.13 | 0.95 | 6.64E-01 | 0.10 | 0.75 | 4.31E-01 | 0.18 | 0.73 | 5.83E-01 | 0.08 | 0.94 | 955648 | 334427 | 1030479 | 651405 | 1001942 | 422327 | 1059015 | 822979 |
| RP_POS_227_17459a3_318 | RP | POS | 3.32 | 227.1746 | | | | 5.55E-05 | -0.71 | 1.29 | 6.64E-01 | 0.09 | 1.11 | 8.24E-04 | -0.67 | 1.29 | 2.51E-04 | -0.76 | 1.32 | 49982486 | 15772854 | 34911987 | 21336106 | 33978276 | 21952636 | 35845698 | 20881855 |
| RP_POS_316_28476a9_152 | RP | POS | 9.15 | 316.2848 | | | | 2.14E-01 | 0.23 | 0.47 | 6.64E-01 | 0.09 | 0.44 | 2.55E-01 | 0.27 | 0.59 | 3.05E-01 | 0.18 | 0.48 | 933714 | 1636469 | 1378525 | 2041526 | 1289120 | 1310440 | 1467929 | 2585952 |
| HILIC_POS_330_33633a0_692 | HILIC | POS | 0.692 | 330.3363 | | | | 5.45E-02 | -0.42 | 0.72 | 6.64E-01 | -0.08 | 0.47 | 4.36E-02 | -0.46 | 0.85 | 1.26E-01 | -0.38 | 0.57 | 3297863 | 924268 | 2937025 | 825001 | 2973101 | 928468 | 2900949 | 714446 |
| RP_POS_277_1795a9_095 | RP | POS | 9.10 | 277.1795 | | | | 1.99E-02 | 0.43 | 0.71 | 6.64E-01 | 0.09 | 0.51 | 2.86E-02 | 0.48 | 0.80 | 6.10E-02 | 0.39 | 0.84 | 3448858 | 115111 | 3507994 | 141178 | 3501822 | 134346 | 3514166 | 148805 |
| RP_NEG_267_07391a0_711 | RP | NEG | 0.71 | 267.0739 | | | | 5.62E-09 | 0.74 | 1.37 | 6.64E-01 | 0.09 | 0.66 | 4.80E-05 | 0.79 | 1.42 | 6.07E-06 | 0.69 | 1.50 | 799629 | 2373825 | 7132080 | 9214887 | 6729208 | 7935741 | 7534953 | 10404518 |
| RP_POS_348_26596a11_248 | RP | POS | 11.25 | 348.2660 | | | | 6.35E-14 | 1.06 | 1.61 | 6.65E-01 | 0.09 | 0.65 | 7.48E-08 | 1.10 | 1.65 | 7.15E-11 | 1.01 | 1.90 | 4175735 | 1759101 | 8577319 | 4147503 | 8396231 | 3288242 | 8758406 | 4886222 |
| RP_POS_617_56555a11_608 | RP | POS | 11.61 | 617.5656 | | | | 2.08E-01 | -0.23 | 0.97 | 6.65E-01 | -0.09 | 0.58 | 1.34E-01 | -0.27 | 0.83 | 4.35E-01 | -0.18 | 0.76 | 1402363 | 468416 | 1272259 | 599308 | 1298419 | 732309 | 1246098 | 433584 |
| RP_NEG_459_59665a15_583 | RP | NEG | 15.58 | 459.5967 | | | | 5.75E-01 | -0.12 | 0.64 | 6.65E-01 | -0.09 | 0.33 | 4.84E-01 | -0.16 | 0.39 | 7.50E-01 | -0.07 | 0.73 | 698514 | 41684 | 693791 | 39591 | 695517 | 41101 | 692064 | 38361 |
| RP_NEG_249_7896a15_551 | RP | NEG | 15.55 | 249.7896 | | | | 1.31E-02 | -0.58 | 1.15 | 6.66E-01 | -0.08 | 0.39 | 1.53E-02 | -0.62 | 1.03 | 2.66E-02 | -0.54 | 1.03 | 1215132 | 89042 | 1170621 | 69802 | 1173658 | 64091 | 1167585 | 75615 |
| RP_POS_252_23283a10_08 | RP | POS | 10.08 | 252.2328 | (2E,4E)-N-(2-methylpropyl)dodeca-2,4-dienamide | 252.1301(100), 95.0861(45), 81.0702(40), 109.1017(30), 69.0704(30), 55.0551(15) | 3 | 4.26E-04 | 0.63 | 1.00 | 6.66E-01 | -0.09 | 0.45 | 7.02E-03 | 0.59 | 0.95 | 4.97E-04 | 0.67 | 1.22 | 1248598 | 580173 | 1722923 | 767184 | 1756298 | 665907 | 1689547 | 862234 |
| RP_POS_465_37708a10_659 | RP | POS | 10.66 | 465.3771 | | | | 4.33E-01 | -0.16 | 0.84 | 6.66E-01 | 0.09 | 0.28 | 5.93E-01 | -0.12 | 0.69 | 3.91E-01 | -0.20 | 0.88 | 5397273 | 428887 | 5328328 | 433114 | 5309489 | 479371 | 5347166 | 385335 |
| HILIC_NEG_80_97431a2_985 | HILIC | NEG | 2.985 | 80.9743 | | | | 7.13E-01 | 0.07 | 0.96 | 6.66E-01 | -0.09 | 0.84 | 9.18E-01 | 0.02 | 0.22 | 5.98E-01 | 0.11 | 0.52 | 5863000 | 1085204 | 5949520 | 1349548 | 6008163 | 1382642 | 5890876 | 1327030 |
| RP_POS_599_11755a9_666 | RP | POS | 9.67 | 599.1176 | | | | 4.00E-02 | -0.38 | 0.75 | 6.66E-01 | -0.09 | 1.10 | 4.59E-02 | -0.42 | 1.00 | 1.16E-01 | -0.33 | 0.77 | 2880866 | 130407 | 2821606 | 163664 | 2828717 | 164725 | 2814494 | 163954 |
| RP_POS_513_36938a13_2 | RP | POS | 13.20 | 513.3694 | | | | 6.90E-01 | 0.08 | 0.50 | 6.67E-01 | -0.09 | 0.55 | 8.63E-01 | 0.04 | 0.50 | 5.82E-01 | 0.13 | 0.32 | 7862057 | 1971409 | 8021099 | 1887661 | 8103010 | 1843494 | 7939188 | 1946043 |
| RP_POS_276_87073a0_499 | RP | POS | 0.50 | 276.8707 | | | | 3.05E-03 | -0.40 | 1.16 | 6.67E-01 | -0.10 | 1.24 | 7.83E-03 | -0.45 | 1.21 | 5.73E-02 | -0.35 | 1.21 | 3428712 | 670989 | 2779659 | 1791738 | 2857284 | 1912291 | 2702034 | 1678395 |
| RP_POS_321_23972a10_173 | RP | POS | 10.17 | 321.2397 | | 321.2402(100) | 4 | 9.86E-01 | 0.00 | 0.93 | 6.67E-01 | 0.09 | 0.84 | 8.09E-01 | 0.05 | 0.70 | 8.48E-01 | -0.04 | 0.88 | 37229135 | 5522419 | 37249625 | 6891094 | 36951319 | 7572012 | 37547931 | 6198911 |
| HILIC_POS_304_21143a1_562 | HILIC | POS | 1.562 | 304.2114 | | | | 2.30E-02 | 0.38 | 0.81 | 6.67E-01 | -0.09 | 0.87 | 9.85E-02 | 0.34 | 0.61 | 2.88E-02 | 0.43 | 0.74 | 4419639 | 2170281 | 5586736 | 3255516 | 5727627 | 3143951 | 5445845 | 3389414 |

**Supplementary table 1: Metabolomics results** (in asceding order according to p-value from progressive ALD vs  non-progressive ALD comparison, with molecular features with ID_level of 1-2 first)

| | | | | | | | | all_ALD_vs_control | | | Prog_ALD_vs_non-prog_ALD | | | Prog_ALD_vs_control_ALD | | | Non-prog_ALD_vs_control | | | Healthy_control | | all_ALD | | Non-prog_ALD | | Progressive_ALD | |
|---|---|---|---|---|---|---|---|---|---|---|---|---|---|---|---|---|---|---|---|---|---|---|---|---|---|---|---|
| | | | | | | | | t-test | Cohen's | PLS-DA | t-test | Cohen's | PLS-DA | t-test | Cohen's | PLS-DA | t-test | Cohen's | PLS-DA | | | | | | | | |
| Feature_ID | RP_HILIC | POS_NEG | Average.Rt | Average.Mz | Metabolite.name | curated_MSMS | ID_level | p | d | VIP | p | d | VIP | p | d | VIP | p | d | VIP | Mean | SD | Mean | SD | Mean | SD | Mean | SD |
| RP_POS_233_9818a0_706 | RP | POS | 0.71 | 233.9818 | | | | 1.22E-01 | 0.22 | 0.72 | 6.68E-01 | 0.10 | 0.79 | 1.96E-01 | 0.27 | 0.82 | 2.68E-01 | 0.17 | 0.52 | 1027757 | 279315 | 1155657 | 656760 | 1127240 | 525142 | 1184074 | 770745 |
| RP_POS_431_28098a12_126 | RP | POS | 12.13 | 431.2810 | | | | 1.05E-01 | -0.29 | 0.83 | 6.68E-01 | 0.09 | 0.72 | 2.13E-01 | -0.25 | 0.75 | 1.23E-01 | -0.34 | 0.78 | 1065701 | 183900 | 999748 | 235649 | 989565 | 257847 | 1009932 | 213301 |
| RP_POS_418_87854a0_465 | RP | POS | 0.47 | 418.8785 | | | | 3.79E-07 | -0.66 | 1.31 | 6.68E-01 | 0.09 | 1.14 | 3.74E-04 | -0.62 | 1.36 | 6.80E-05 | -0.71 | 1.43 | 447902 | 67000 | 317347 | 213184 | 308136 | 215881 | 326558 | 212237 |
| HILIC_POS_518_3172a0_985 | HILIC | POS | 0.985 | 518.3172 | | | | 6.82E-05 | 0.49 | 1.15 | 6.68E-01 | -0.10 | 0.77 | 7.13E-03 | 0.44 | 1.29 | 2.41E-03 | 0.54 | 1.29 | 162332 | 616730 | 1497924 | 3047010 | 1629355 | 3166908 | 1366492 | 2948466 |
| RP_NEG_459_14697a11_611 | RP | NEG | 11.61 | 459.1470 | | | | 6.71E-01 | -0.09 | 0.55 | 6.69E-01 | 0.09 | 0.61 | 8.38E-01 | -0.05 | 0.41 | 5.80E-01 | -0.13 | 0.53 | 578950 | 70568 | 572900 | 67248 | 570002 | 71666 | 575798 | 63117 |
| RP_NEG_924_74475a0_498 | RP | NEG | 0.50 | 924.7448 | | | | 1.42E-02 | -0.35 | 1.14 | 6.69E-01 | -0.09 | 1.24 | 2.24E-02 | -0.40 | 1.16 | 1.09E-01 | -0.31 | 1.17 | 1941405 | 352022 | 1694762 | 770471 | 1727960 | 818801 | 1661565 | 725728 |
| RP_POS_818_58881a11_999 | RP | POS | 12.00 | 818.5888 | | | | 4.72E-01 | 0.10 | 0.36 | 6.69E-01 | -0.10 | 0.35 | 7.10E-01 | 0.05 | 0.19 | 4.97E-01 | 0.15 | 0.40 | 879076 | 848788 | 1080299 | 2350211 | 1181589 | 2947789 | 979009 | 1565027 |
| RP_POS_193_15875a11_038 | RP | POS | 11.04 | 193.1588 | | | | 1.51E-02 | 0.39 | 0.79 | 6.69E-01 | -0.09 | 0.32 | 5.32E-02 | 0.35 | 0.71 | 3.54E-02 | 0.44 | 0.75 | 492334 | 604441 | 850269 | 970494 | 892041 | 1082954 | 808496 | 852401 |
| HILIC_NEG_257_83884a1_362 | HILIC | NEG | 1.362 | 257.8388 | | | | 5.23E-15 | 1.03 | 1.62 | 6.69E-01 | 0.09 | 0.67 | 6.21E-08 | 1.07 | 1.68 | 2.90E-10 | 0.99 | 2.01 | 94874 | 35967 | 210744 | 114301 | 205824 | 96196 | 215664 | 130741 |
| RP_NEG_258_98245a0_553 | RP | NEG | 0.55 | 258.9825 | | | | 7.68E-04 | 0.48 | 0.84 | 6.70E-01 | -0.09 | 0.80 | 2.99E-02 | 0.43 | 0.86 | 1.25E-03 | 0.53 | 1.03 | 2382398 | 622595 | 2998342 | 1402562 | 3058642 | 1196677 | 2938042 | 1592185 |
| RP_NEG_262_99207a8_627 | RP | NEG | 8.63 | 262.9921 | | | | 8.66E-02 | -0.35 | 0.79 | 6.70E-01 | -0.09 | 0.36 | 8.55E-02 | -0.39 | 0.73 | 1.72E-01 | -0.31 | 0.63 | 4728174 | 288731 | 4625797 | 288290 | 4638184 | 285931 | 4613410 | 292999 |
| RP_POS_156_07649a0_514 | RP | POS | 0.51 | 156.0765 | | | | 1.95E-02 | 0.34 | 0.89 | 6.71E-01 | -0.09 | 0.89 | 7.62E-02 | 0.30 | 0.87 | 5.22E-02 | 0.39 | 0.99 | 1867713 | 279860 | 2046735 | 570597 | 2071187 | 639499 | 2022284 | 497621 |
| RP_POS_250_1774a11_129 | RP | POS | 11.13 | 250.1774 | | | | 1.45E-02 | 0.39 | 0.83 | 6.71E-01 | -0.09 | 0.32 | 5.34E-02 | 0.35 | 0.72 | 3.48E-02 | 0.44 | 0.80 | 1422751 | 1803388 | 2511149 | 2976974 | 2638669 | 3308612 | 2383629 | 2631638 |
| RP_POS_791_58746a14_267 | RP | POS | 14.27 | 791.5875 | | | | 2.94E-01 | 0.15 | 0.35 | 6.71E-01 | 0.09 | 0.34 | 3.70E-01 | 0.20 | 0.36 | 4.23E-01 | 0.11 | 0.53 | 1147642 | 460226 | 1278756 | 939184 | 1238506 | 552199 | 1319007 | 1214046 |
| RP_POS_603_52252a15_221 | RP | POS | 15.22 | 603.5225 | | | | 8.17E-01 | 0.05 | 0.31 | 6.71E-01 | 0.08 | 0.82 | 6.89E-01 | 0.09 | 0.50 | 9.79E-01 | 0.01 | 0.58 | 1700305 | 561997 | 1726631 | 535780 | 1703711 | 568378 | 1749551 | 505820 |
| RP_NEG_480_30984a10_932 | RP | NEG | 10.93 | 480.3098 | | | | 1.14E-01 | 0.29 | 1.09 | 6.71E-01 | 0.09 | 0.93 | 1.41E-01 | 0.34 | 0.82 | 2.00E-01 | 0.25 | 0.92 | 8343076 | 2752910 | 9287041 | 3315081 | 9145331 | 2712418 | 9428751 | 3847806 |
| HILIC_NEG_115_92072a0_547 | HILIC | NEG | 0.547 | 115.9207 | | | | 2.26E-04 | -0.57 | 1.04 | 6.71E-01 | 0.09 | 0.67 | 4.70E-03 | -0.52 | 0.94 | 1.50E-03 | -0.61 | 1.03 | 8867036 | 754102 | 8133382 | 1374855 | 8074638 | 1417820 | 8192127 | 1342301 |
| RP_NEG_666_45483a11_612 | RP | NEG | 11.61 | 666.4548 | | | | 8.18E-02 | -0.33 | 0.87 | 6.71E-01 | 0.09 | 0.75 | 1.70E-01 | -0.28 | 0.78 | 9.43E-02 | -0.37 | 0.73 | 6699177 | 579358 | 6481469 | 681219 | 6452361 | 732563 | 6510577 | 631832 |
| RP_NEG_150_88582a0_524 | RP | NEG | 0.52 | 150.8858 | | | | 5.58E-02 | 0.29 | 0.84 | 6.72E-01 | -0.09 | 0.82 | 1.82E-01 | 0.24 | 0.87 | 8.16E-02 | 0.34 | 1.00 | 1808800 | 529101 | 2072507 | 990826 | 2114819 | 1032607 | 2030196 | 955812 |
| RP_POS_478_38892a12_018 | RP | POS | 12.02 | 478.3889 | | | | 9.94E-01 | 0.00 | 0.45 | 6.72E-01 | 0.09 | 0.45 | 8.55E-01 | 0.04 | 0.65 | 8.48E-01 | -0.04 | 0.42 | 43388616 | 15947151 | 43362931 | 15890136 | 42684656 | 16495159 | 44041207 | 15398593 |
| HILIC_NEG_115_92072a5_607 | HILIC | NEG | 5.607 | 115.9207 | | | | 5.58E-01 | -0.11 | 0.72 | 6.72E-01 | 0.09 | 0.75 | 7.75E-01 | -0.06 | 0.17 | 4.67E-01 | -0.15 | 0.49 | 50522328 | 6873398 | 49635397 | 8893444 | 49255973 | 8736442 | 50014820 | 9120397 |
| RP_POS_384_18402a10_585 | RP | POS | 10.59 | 384.1840 | | | | 2.03E-03 | 0.56 | 0.99 | 6.72E-01 | -0.09 | 0.41 | 1.43E-02 | 0.52 | 0.89 | 3.30E-03 | 0.60 | 1.20 | 582918 | 279865 | 779287 | 358866 | 794596 | 347704 | 763978 | 372588 |
| RP_POS_352_35684a11_904 | RP | POS | 11.90 | 352.3568 | | | | 9.23E-01 | -0.02 | 0.67 | 6.72E-01 | -0.08 | 0.28 | 7.85E-01 | -0.06 | 0.32 | 9.26E-01 | 0.02 | 0.49 | 215722 | 183686 | 212149 | 174992 | 219613 | 182555 | 204684 | 168610 |
| RP_POS_722_52521a11_912 | RP | POS | 11.91 | 722.5252 | | | | 9.11E-01 | 0.02 | 0.73 | 6.72E-01 | 0.09 | 0.68 | 7.65E-01 | 0.07 | 0.88 | 9.18E-01 | -0.02 | 0.49 | 6884225 | 841441 | 6904071 | 952423 | 6863450 | 953986 | 6944692 | 958787 |
| RP_POS_259_18988a9_303 | RP | POS | 9.30 | 259.1899 | | | | 4.65E-03 | -0.44 | 1.01 | 6.72E-01 | 0.09 | 1.01 | 3.38E-02 | -0.39 | 1.10 | 1.28E-02 | -0.49 | 1.04 | 1167571 | 123518 | 1077684 | 220218 | 1068302 | 228350 | 1087066 | 213679 |
| RP_POS_375_21915a15_44 | RP | POS | 15.44 | 375.2192 | | | | 6.69E-01 | 0.08 | 0.37 | 6.72E-01 | 0.09 | 0.34 | 5.54E-01 | 0.12 | 0.40 | 8.73E-01 | 0.03 | 0.87 | 1083990 | 231713 | 1105293 | 280479 | 1093345 | 293979 | 1117241 | 268752 |
| RP_NEG_426_96716a10_998 | RP | NEG | 11.00 | 426.9672 | | | | 8.53E-01 | 0.04 | 0.37 | 6.72E-01 | 0.08 | 0.45 | 7.52E-01 | 0.08 | 0.42 | 9.85E-01 | 0.00 | 0.22 | 1472347 | 2400739 | 1558558 | 1809918 | 1481447 | 1539099 | 1635669 | 2058518 |
| HILIC_POS_498_90005a4_324 | HILIC | POS | 4.324 | 498.9001 | | | | 1.22E-01 | 0.24 | 1.05 | 6.73E-01 | -0.09 | 0.91 | 3.19E-01 | 0.19 | 0.65 | 1.31E-01 | 0.28 | 1.04 | 13690295 | 2046823 | 14504670 | 3766495 | 14665025 | 3727058 | 14344315 | 3836541 |
| HILIC_POS_430_38919a0_517 | HILIC | POS | 0.517 | 430.3892 | | | | 3.75E-01 | 0.14 | 0.64 | 6.73E-01 | -0.09 | 0.80 | 5.95E-01 | 0.09 | 0.52 | 3.67E-01 | 0.19 | 0.44 | 84541 | 99602 | 106221 | 167524 | 113350 | 186735 | 99093 | 147403 |
| RP_POS_196_8647a0_521 | RP | POS | 0.52 | 196.8647 | | | | 8.59E-06 | 0.54 | 1.25 | 6.73E-01 | -0.09 | 1.12 | 2.19E-03 | 0.50 | 1.24 | 9.46E-04 | 0.59 | 1.36 | 333643 | 57957 | 487898 | 315080 | 501295 | 330894 | 474501 | 301198 |
| RP_POS_221_17502a11_039 | RP | POS | 11.04 | 221.1750 | | | | 7.53E-03 | 0.41 | 0.90 | 6.73E-01 | -0.09 | 0.34 | 3.60E-02 | 0.36 | 0.80 | 2.58E-02 | 0.45 | 0.81 | 214318 | 233153 | 377643 | 434138 | 396102 | 484050 | 359183 | 381836 |
| HILIC_NEG_214_91925a2_911 | HILIC | NEG | 2.911 | 214.9193 | | | | 1.50E-06 | -0.62 | 1.36 | 6.73E-01 | 0.09 | 0.81 | 4.29E-04 | -0.58 | 1.49 | 3.28E-04 | -0.67 | 1.47 | 1228457 | 141019 | 955967 | 479089 | 935605 | 513819 | 976330 | 445961 |
| RP_POS_313_14249a9_475 | RP | POS | 9.48 | 313.1425 | | | | 9.51E-02 | -0.40 | 0.73 | 6.73E-01 | 0.08 | 0.42 | 1.48E-01 | -0.36 | 0.68 | 9.27E-02 | -0.44 | 0.65 | 774189 | 60701 | 754360 | 44396 | 752474 | 47895 | 756246 | 41000 |
| RP_POS_846_82361a0_493 | RP | POS | 0.49 | 846.8236 | | | | 4.17E-08 | 0.66 | 1.37 | 6.73E-01 | -0.09 | 1.20 | 1.71E-04 | 0.61 | 1.44 | 7.72E-05 | 0.70 | 1.47 | 207948 | 106592 | 1302099 | 1834109 | 1379997 | 1917618 | 1224200 | 1762645 |
| RP_POS_499_27466a11_821 | RP | POS | 11.82 | 499.2747 | | | | 5.63E-01 | 0.11 | 0.51 | 6.73E-01 | -0.09 | 0.49 | 7.64E-01 | 0.07 | 0.42 | 4.63E-01 | 0.16 | 0.47 | 828302 | 64381 | 836144 | 72258 | 839213 | 66698 | 833075 | 77982 |
| RP_POS_666_43732a10_903 | RP | POS | 10.90 | 666.4373 | | | | 1.01E-06 | 0.78 | 1.55 | 6.73E-01 | 0.09 | 0.52 | 8.37E-06 | 0.83 | 1.57 | 2.01E-04 | 0.74 | 1.46 | 268963 | 165790 | 481629 | 279665 | 469751 | 299259 | 493507 | 261108 |
| RP_POS_266_17258a11_038 | RP | POS | 11.04 | 266.1726 | | | | 7.87E-03 | 0.43 | 0.85 | 6.73E-01 | -0.09 | 0.32 | 3.50E-02 | 0.38 | 0.79 | 2.16E-02 | 0.47 | 0.83 | 563685 | 415195 | 835311 | 676759 | 864042 | 742801 | 806580 | 609847 |
| RP_POS_168_98027a15_484 | RP | POS | 15.48 | 168.9803 | | | | 3.30E-01 | 0.19 | 0.74 | 6.74E-01 | 0.09 | 0.41 | 2.82E-01 | 0.23 | 0.40 | 5.14E-01 | 0.15 | 1.05 | 20507439 | 3096451 | 21141968 | 3423780 | 20997012 | 3581163 | 21286924 | 3288794 |
| RP_POS_708_80408a0_498 | RP | POS | 0.50 | 708.8041 | | | | 7.67E-09 | -0.70 | 1.39 | 6.74E-01 | 0.09 | 1.20 | 6.52E-05 | -0.66 | 1.48 | 2.93E-05 | -0.75 | 1.48 | 2755230 | 145438 | 2027142 | 1133255 | 1979197 | 1182492 | 2075088 | 1091679 |
| RP_POS_419_3143a11_253 | RP | POS | 11.25 | 419.3143 | | | | 2.18E-04 | 0.69 | 1.23 | 6.75E-01 | -0.08 | 1.00 | 2.83E-03 | 0.65 | 1.21 | 3.44E-04 | 0.73 | 1.32 | 6057999 | 1051659 | 6943957 | 1280568 | 6998119 | 1182190 | 6889795 | 1381887 |
| RP_NEG_893_1936a9_433 | RP | NEG | 9.43 | 893.1936 | | | | 8.30E-02 | -0.31 | 0.72 | 6.75E-01 | 0.09 | 0.70 | 1.85E-01 | -0.26 | 0.59 | 1.01E-01 | -0.35 | 0.78 | 3305518 | 278467 | 3197084 | 371204 | 3181385 | 398235 | 3212784 | 345403 |
| RP_NEG_378_91846a0_463 | RP | NEG | 0.46 | 378.9185 | | | | 1.92E-09 | -0.75 | 1.41 | 6.75E-01 | 0.09 | 1.18 | 4.00E-05 | -0.70 | 1.48 | 7.55E-06 | -0.79 | 1.52 | 2736057 | 238555 | 1897745 | 1217557 | 1846269 | 1236095 | 1949222 | 1209052 |
| RP_POS_408_30829a11_038 | RP | POS | 11.04 | 408.3083 | | | | 1.11E-02 | 0.42 | 0.83 | 6.75E-01 | -0.09 | 0.34 | 4.36E-02 | 0.37 | 0.76 | 2.66E-02 | 0.46 | 0.80 | 14658051 | 16155458 | 24595437 | 25446372 | 25671238 | 27934846 | 23519636 | 22924503 |
| RP_POS_132_95834a15_441 | RP | POS | 15.44 | 132.9583 | | | | 1.44E-01 | -0.41 | 0.75 | 6.75E-01 | -0.07 | 0.29 | 1.23E-01 | -0.44 | 0.85 | 2.01E-01 | -0.37 | 0.54 | 225801875 | 61974127 | 208754489 | 32570816 | 210130940 | 36016910 | 207378039 | 29022093 |
| RP_NEG_182_89629a15_534 | RP | NEG | 15.53 | 182.8963 | | | | 1.52E-01 | -0.26 | 0.78 | 6.75E-01 | -0.09 | 0.36 | 1.38E-01 | -0.30 | 0.93 | 3.15E-01 | -0.22 | 0.71 | 1502828 | 336119 | 1396794 | 427706 | 1414865 | 450001 | 1378724 | 407958 |
| RP_NEG_137_02351a5_647 | RP | NEG | 5.65 | 137.0235 | | | | 7.80E-02 | 0.20 | 0.92 | 6.75E-01 | -0.10 | 0.37 | 3.08E-01 | 0.16 | 0.86 | 1.51E-01 | 0.25 | 0.63 | 1192298 | 590242 | 3454774 | 12665000 | 3989608 | 13538845 | 2919940 | 11840351 |
| HILIC_NEG_279_01553a3_869 | HILIC | NEG | 3.869 | 279.0155 | | | | 2.30E-01 | 0.22 | 0.89 | 6.75E-01 | -0.09 | 0.92 | 4.35E-01 | 0.17 | 0.45 | 1.85E-01 | 0.26 | 0.61 | 297730 | 105769 | 325635 | 134926 | 331332 | 118761 | 319939 | 150369 |
| RP_NEG_227_90472a1_469 | RP | NEG | 1.47 | 227.9047 | | | | 2.83E-10 | 0.88 | 1.48 | 6.75E-01 | 0.09 | 0.59 | 1.06E-06 | 0.93 | 1.63 | 1.18E-06 | 0.84 | 1.50 | 1924792 | 1476796 | 4976075 | 3590225 | 4824564 | 3396126 | 5127587 | 3802890 |
| RP_POS_420_88394a0_512 | RP | POS | 0.51 | 420.8839 | | | | 1.69E-06 | 0.58 | 1.27 | 6.75E-01 | -0.09 | 1.14 | 1.05E-03 | 0.53 | 1.29 | 4.74E-04 | 0.62 | 1.39 | 322461 | 92946 | 723793 | 774733 | 756479 | 813297 | 691107 | 740971 |
| RP_POS_810_60004a13_653 | RP | POS | 13.65 | 810.6000 | | | | 3.89E-06 | 1.01 | 1.73 | 6.76E-01 | 0.07 | 0.36 | 1.69E-05 | 1.05 | 1.53 | 1.05E-05 | 0.97 | 1.78 | 15455077 | 6669710 | 22349722 | 6003746 | 22096587 | 4779300 | 22602857 | 7060670 |
| RP_NEG_381_17526a11_254 | RP | NEG | 11.25 | 381.1753 | | | | 2.02E-06 | 0.93 | 1.79 | 6.76E-01 | 0.08 | 1.07 | 1.28E-05 | 0.97 | 1.74 | 1.86E-05 | 0.89 | 1.72 | 16529704 | 6050189 | 23225172 | 6829963 | 22937501 | 6293321 | 23512843 | 7380652 |
| RP_NEG_517_24243a10_209 | RP | NEG | 10.21 | 517.2424 | | | | 6.97E-02 | 0.30 | 0.69 | 6.76E-01 | -0.09 | 0.26 | 1.69E-01 | 0.25 | 0.57 | 1.00E-01 | 0.34 | 0.64 | 717608 | 515236 | 940224 | 798166 | 973816 | 879635 | 906631 | 714897 |
| RP_POS_247_99725a0_727 | RP | POS | 0.73 | 247.9973 | | | | 1.47E-09 | 0.82 | 1.38 | 6.76E-01 | -0.09 | 0.82 | 1.50E-05 | 0.78 | 1.42 | 1.25E-06 | 0.87 | 1.54 | 358312 | 255063 | 892291 | 683099 | 921029 | 676225 | 863553 | 695561 |
| RP_POS_413_32361a10_881 | RP | POS | 10.88 | 413.3236 | | | | 4.74E-06 | -0.71 | 1.26 | 6.76E-01 | 0.09 | 0.98 | 2.58E-04 | -0.67 | 1.38 | 1.23E-04 | -0.75 | 1.28 | 40685384 | 1476126 | 38877770 | 2660948 | 38765916 | 2803790 | 38989625 | 2533579 |
| HILIC_POS_192_06172a7_377 | HILIC | POS | 7.377 | 192.0617 | | | | 3.36E-04 | -0.59 | 1.19 | 6.77E-01 | -0.09 | 0.83 | 1.06E-03 | -0.64 | 1.24 | 5.66E-03 | -0.55 | 1.26 | 4772073 | 592020 | 4250650 | 919393 | 4289261 | 945270 | 4212038 | 900689 |
| RP_NEG_920_56598a13_215 | RP | NEG | 13.22 | 920.5660 | | | | 1.52E-04 | 0.69 | 1.14 | 6.77E-01 | 0.08 | 0.53 | 1.52E-03 | 0.73 | 1.14 | 4.22E-04 | 0.64 | 1.30 | 1558087 | 303222 | 1825056 | 393296 | 1808529 | 287857 | 1841583 | 478645 |
| RP_NEG_164_92662a0_444 | RP | NEG | 0.44 | 164.9266 | | | | 1.10E-08 | -0.70 | 1.38 | 6.77E-01 | 0.09 | 1.20 | 1.20E-04 | -0.65 | 1.45 | 2.26E-05 | -0.74 | 1.49 | 1336791 | 76910 | 962897 | 588769 | 938176 | 596970 | 987618 | 585451 |
| RP_NEG_383_31604a11_164 | RP | NEG | 11.16 | 383.3160 | | | | 5.29E-02 | 0.31 | 0.91 | 6.77E-01 | 0.09 | 0.65 | 6.05E-02 | 0.36 | 0.98 | 1.78E-01 | 0.27 | 0.83 | 1430109 | 256710 | 1550556 | 413087 | 1533225 | 430374 | 1567887 | 398660 |
| RP_NEG_397_20599a9_913 | RP | NEG | 9.91 | 397.2060 | | | | 8.98E-01 | -0.02 | 0.90 | 6.77E-01 | -0.09 | 0.75 | 7.62E-01 | -0.07 | 0.55 | 9.32E-01 | 0.02 | 0.48 | 23343468 | 14361885 | 22956416 | 16057323 | 23629685 | 15215329 | 22283147 | 16985462 |
| RP_POS_478_38867a11_211 | RP | POS | 11.21 | 478.3887 | | | | 2.11E-07 | -0.97 | 1.61 | 6.77E-01 | 0.08 | 0.69 | 8.79E-06 | -0.93 | 1.59 | 9.17E-07 | -1.01 | 1.62 | 1965748 | 190717 | 1727289 | 232186 | 1717556 | 224489 | 1737022 | 241521 |
| RP_POS_519_23914a6_08 | RP | POS | 6.08 | 519.2391 | | | | 7.18E-01 | 0.07 | 0.55 | 6.77E-01 | 0.09 | 0.54 | 6.25E-01 | 0.11 | 0.58 | 9.10E-01 | 0.02 | 0.37 | 18437571 | 1111152 | 18524700 | 1377211 | 18466957 | 1183657 | 18582443 | 1557010 |
| RP_POS_192_97971a15_442 | RP | POS | 15.44 | 192.9797 | | | | 8.60E-02 | -0.35 | 0.80 | 6.78E-01 | 0.08 | 0.52 | 1.69E-01 | -0.31 | 0.58 | 8.89E-02 | -0.39 | 0.74 | 18912704 | 6072505 | 16739305 | 6259835 | 16477229 | 6482235 | 17001381 | 6083686 |
| RP_POS_250_17754a11_039 | RP | POS | 11.04 | 250.1775 | | | | 9.90E-03 | 0.41 | 0.84 | 6.78E-01 | -0.09 | 0.32 | 4.14E-02 | 0.37 | 0.76 | 2.59E-02 | 0.46 | 0.81 | 853444 | 978431 | 1476375 | 1609168 | 1543728 | 1768253 | 1409022 | 1447658 |
| RP_POS_256_82013a0_521 | RP | POS | 0.52 | 256.8201 | | | | 2.27E-06 | 0.57 | 1.26 | 6.78E-01 | -0.09 | 1.11 | 1.11E-03 | 0.53 | 1.26 | 5.47E-04 | 0.62 | 1.38 | 890546 | 129514 | 1344872 | 881709 | 1381773 | 928652 | 1307971 | 839948 |
| HILIC_POS_114_63306a0_505 | HILIC | POS | 0.505 | 114.6331 | | | | 7.05E-01 | -0.07 | 0.85 | 6.78E-01 | -0.09 | 0.48 | 5.64E-01 | -0.11 | 0.64 | 9.15E-01 | -0.02 | 0.89 | 282542 | 212482 | 264886 | 274369 | 276370 | 309721 | 253401 | 236424 |
| HILIC_POS_640_31641a1_343 | HILIC | POS | 1.343 | 640.3164 | | | | 3.73E-02 | -0.41 | 1.32 | 6.78E-01 | -0.08 | 0.50 | 3.84E-02 | -0.45 | 1.23 | 1.02E-01 | -0.36 | 1.43 | 385091 | 137070 | 324117 | 151762 | 330458 | 158003 | 317777 | 146584 |
| RP_POS_429_2699a10_614 | RP | POS | 10.61 | 429.2699 | | | | 8.02E-02 | 0.33 | 0.83 | 6.78E-01 | 0.09 | 0.46 | 9.27E-02 | 0.37 | 0.69 | 1.66E-01 | 0.29 | 0.83 | 837914 | 497019 | 1025428 | 581644 | 1001136 | 542659 | 1049720 | 622769 |
| RP_POS_395_81531a15_502 | RP | POS | 15.50 | 395.8153 | | | | 1.36E-01 | -0.24 | 0.43 | 6.79E-01 | -0.09 | 0.49 | 1.50E-01 | -0.29 | 0.54 | 3.03E-01 | -0.20 | 0.42 | 1603000 | 123231 | 1558969 | 195010 | 1567106 | 189980 | 1550832 | 201510 |
| RP_POS_516_28906a6_842 | RP | POS | 6.84 | 516.2891 | | | | 1.95E-07 | 0.62 | 1.33 | 6.79E-01 | -0.09 | 1.15 | 4.04E-04 | 0.57 | 1.43 | 1.65E-04 | 0.67 | 1.41 | 20990 | 48717 | 1347997 | 2370018 | 1446883 | 2470149 | 1249111 | 2286266 |
| RP_POS_379_24234a12_325 | RP | POS | 12.33 | 379.2423 | | | | 1.29E-04 | -0.78 | 1.33 | 6.79E-01 | 0.08 | 0.62 | 1.48E-03 | -0.74 | 1.19 | 1.20E-04 | -0.82 | 1.50 | 3071418 | 652614 | 2520189 | 673097 | 2492126 | 570199 | 2548252 | 767224 |
| RP_POS_296_89896a0_257 | RP | POS | 0.26 | 296.8990 | | | | 2.88E-01 | 0.21 | 0.61 | 6.80E-01 | -0.08 | 0.29 | 4.65E-01 | 0.17 | 0.35 | 2.37E-01 | 0.25 | 0.54 | 1869069 | 355958 | 1947738 | 375289 | 1963353 | 337749 | 1932124 | 412294 |
| RP_NEG_1012_79669a0_489 | RP | NEG | 0.49 | 1012.7967 | | | | 1.22E-05 | 0.54 | 1.23 | 6.80E-01 | 0.09 | 1.21 | 9.75E-04 | 0.59 | 1.33 | 2.58E-03 | 0.50 | 1.29 | 814481 | 260165 | 1395122 | 1187357 | 1345728 | 1145786 | 1444515 | 1237173 |
| RP_NEG_158_97772a0_449 | RP | NEG | 0.45 | 158.9777 | | | | 6.64E-07 | -0.67 | 1.31 | 6.80E-01 | -0.09 | 1.18 | 4.06E-05 | -0.72 | 1.44 | 5.75E-04 | -0.63 | 1.35 | 3369289 | 448709 | 2602605 | 1231816 | 2653847 | 1280351 | 2551363 | 1192079 |
| RP_POS_451_30252a11_047 | RP | POS | 11.05 | 451.3025 | | | | 7.57E-05 | 0.47 | 0.89 | 6.80E-01 | 0.09 | 0.80 | 6.86E-03 | 0.51 | 1.00 | 3.11E-03 | 0.42 | 1.24 | 27642 | 28671 | 253260 | 544273 | 230630 | 460327 | 275890 | 620941 |
| RP_POS_940_52722a13_071 | RP | POS | 13.07 | 940.5272 | | | | 6.58E-01 | -0.09 | 0.59 | 6.80E-01 | 0.08 | 0.31 | 8.27E-01 | -0.05 | 0.41 | 5.66E-01 | -0.13 | 0.68 | 738746 | 447035 | 698442 | 442063 | 680080 | 453319 | 716804 | 434328 |
| RP_POS_337_05789a0_616 | RP | POS | 0.62 | 337.0579 | | | | 3.99E-05 | -0.58 | 0.96 | 6.80E-01 | -0.09 | 0.44 | 2.41E-04 | -0.63 | 1.20 | 5.19E-03 | -0.54 | 1.08 | 2060199 | 251604 | 1747864 | 581200 | 1771999 | 632244 | 1723730 | 530622 |
| RP_POS_178_99969a15_442 | RP | POS | 15.44 | 178.9997 | | | | 6.06E-01 | -0.10 | 0.44 | 6.80E-01 | 0.09 | 0.43 | 7.90E-01 | -0.06 | 0.49 | 5.23E-01 | -0.14 | 0.40 | 4648426 | 1271435 | 4510841 | 1409002 | 4452349 | 1466307 | 4569333 | 1361634 |
| RP_NEG_509_11502a8_859 | RP | NEG | 8.86 | 509.1150 | | | | 6.32E-01 | -0.09 | 0.58 | 6.80E-01 | -0.09 | 0.45 | 5.39E-01 | -0.13 | 0.44 | 8.25E-01 | -0.05 | 0.26 | 9786508 | 520251 | 9733329 | 610075 | 9758653 | 605171 | 9708005 | 620030 |
| RP_POS_182_99545a15_45 | RP | POS | 15.45 | 182.9955 | | | | 6.04E-01 | 0.09 | 0.57 | 6.80E-01 | 0.09 | 0.33 | 5.44E-01 | 0.13 | 0.46 | 8.05E-01 | 0.05 | 0.18 | 7937083 | 1471219 | 8108192 | 2012486 | 8024636 | 1684802 | 8191748 | 2308689 |
| HILIC_NEG_257_81924a2_946 | HILIC | NEG | 2.946 | 257.8192 | | | | 6.97E-04 | 0.51 | 1.14 | 6.80E-01 | -0.09 | 0.80 | 1.10E-02 | 0.46 | 0.95 | 3.87E-03 | 0.55 | 1.15 | 3582915 | 695911 | 4225400 | 1366108 | 4282097 | 1412857 | 4168703 | 1329609 |
| HILIC_POS_236_0713a4_324 | HILIC | POS | 4.324 | 236.0713 | | | | 7.67E-01 | -0.05 | 0.84 | 6.80E-01 | 0.09 | 0.69 | 9.64E-01 | -0.01 | 0.34 | 6.47E-01 | -0.10 | 0.44 | 1405531 | 348271 | 1382781 | 452970 | 1363982 | 467644 | 1401579 | 441746 |
| HILIC_POS_291_06946a2_456 | HILIC | POS | 2.456 | 291.0695 | | | | 2.49E-09 | 0.73 | 1.25 | 6.80E-01 | 0.09 | 0.62 | 5.23E-05 | 0.77 | 1.30 | 6.57E-06 | 0.68 | 1.64 | 655687 | 645206 | 3839903 | 4766542 | 3642109 | 4140156 | 4037697 | 5355630 |
| RP_POS_441_29742a11_934 | RP | POS | 11.93 | 441.2974 | | | | 9.40E-01 | -0.01 | 0.39 | 6.80E-01 | -0.09 | 0.36 | 7.83E-01 | -0.06 | 0.28 | 8.93E-01 | 0.03 | 0.45 | 144239553 | 37678800 | 143627130 | 45734757 | 145524099 | 48048772 | 141730161 | 43703486 |
| RP_NEG_417_22507a11_255 | RP | NEG | 11.26 | 417.2251 | | | | 4.71E-04 | 0.68 | 1.51 | 6.80E-01 | 0.08 | 0.96 | 1.24E-03 | 0.72 | 1.42 | 2.40E-03 | 0.64 | 1.38 | 2272908 | 529472 | 2684408 | 597688 | 2659617 | 560829 | 2709199 | 637157 |
| RP_NEG_131_07033a4_556 | RP | NEG | 4.56 | 131.0703 | | | | 1.06E-04 | 0.53 | 0.91 | 6.81E-01 | 0.09 | 0.40 | 3.22E-04 | 0.57 | 1.15 | 1.40E-02 | 0.48 | 0.76 | 1151013 | 361072 | 1614856 | 967582 | 1574733 | 1096537 | 1654978 | 828165 |
| RP_NEG_530_86743a0_466 | RP | NEG | 0.47 | 530.8674 | | | | 4.89E-08 | -0.68 | 1.36 | 6.81E-01 | 0.09 | 1.23 | 1.96E-04 | -0.63 | 1.41 | 4.13E-05 | -0.72 | 1.46 | 895985 | 84690 | 638560 | 415757 | 621325 | 422646 | 655795 | 412310 |
| RP_NEG_156_92947a15_399 | RP | NEG | 15.40 | 156.9295 | | | | 5.77E-01 | 0.15 | 0.46 | 6.81E-01 | 0.07 | 0.26 | 4.88E-01 | 0.19 | 0.32 | 6.94E-01 | 0.12 | 0.42 | 905915 | 126999 | 919156 | 70876 | 916214 | 93017 | 922099 | 38464 |
| HILIC_NEG_202_9292a4_277 | HILIC | NEG | 4.277 | 202.9292 | | | | 4.98E-01 | 0.12 | 0.93 | 6.81E-01 | -0.09 | 0.78 | 6.97E-01 | 0.08 | 0.15 | 4.34E-01 | 0.17 | 0.34 | 12268307 | 2297215 | 12605736 | 2830203 | 12722946 | 2911442 | 12488527 | 2771107 |

**Supplementary table 1: Metabolomics results** (in asceding order according to p-value from progressive ALD vs non-progressive ALD comparison, with molecular features with ID_level of 1-2 first)

| Feature_ID | RP_HILIC | POS_NEG | Average.Rt | Average.Mz | Metabolite.name | curated_MSMS | ID_level | all_ALD_vs_control t-test p | all_ALD_vs_control Cohen's d | all_ALD_vs_control PLS-DA VIP | Prog_ALD_vs_non-prog_ALD t-test p | Prog_ALD_vs_non-prog_ALD Cohen's d | Prog_ALD_vs_non-prog_ALD PLS-DA VIP | Prog_ALD_vs_control_ALD t-test p | Prog_ALD_vs_control_ALD Cohen's d | Prog_ALD_vs_control_ALD PLS-DA VIP | Non-prog_ALD_vs_control t-test p | Non-prog_ALD_vs_control Cohen's d | Non-prog_ALD_vs_control PLS-DA VIP | Healthy_control Mean | Healthy_control SD | all_ALD Mean | all_ALD SD | Non-prog_ALD Mean | Non-prog_ALD SD | Progressive_ALD Mean | Progressive_ALD SD |
|---|---|---|---|---|---|---|---|---|---|---|---|---|---|---|---|---|---|---|---|---|---|---|---|---|---|---|---|
| HILIC_POS_160_03697a4_251 | HILIC | POS | 4.251 | 160.0370 | | | | 9.94E-01 | 0.00 | 0.33 | 6.81E-01 | -0.09 | 0.82 | 8.28E-01 | -0.05 | 0.34 | 8.40E-01 | 0.04 | 0.28 | 2416353 | 2787981 | 2411541 | 3609413 | 2561018 | 3642688 | 2262064 | 3606499 |
| RP_POS_220_93414a0_468 | RP | POS | 0.47 | 220.9341 | | | | 6.41E-09 | -0.72 | 1.39 | 6.81E-01 | 0.09 | 1.18 | 7.83E-05 | -0.67 | 1.45 | 1.49E-05 | -0.76 | 1.51 | 2876373 | 231817 | 2033507 | 1280842 | 1980518 | 1298394 | 2086495 | 1273985 |
| RP_POS_364_19754a6_444 | RP | POS | 6.44 | 364.1975 | | | | 9.66E-02 | -0.31 | 0.56 | 6.81E-01 | 0.09 | 0.58 | 2.09E-01 | -0.26 | 0.49 | 1.02E-01 | -0.35 | 0.60 | 2644815 | 219570 | 2565191 | 268600 | 2554080 | 273199 | 2576302 | 266222 |
| RP_POS_499_82596a5_14 | RP | POS | 5.14 | 499.8260 | | | | 4.69E-01 | 0.14 | 0.44 | 6.81E-01 | -0.08 | 0.49 | 6.45E-01 | 0.10 | 0.36 | 4.21E-01 | 0.18 | 0.57 | 1553367 | 1602769 | 1797108 | 1782730 | 1870827 | 1919706 | 1723390 | 1650682 |
| RP_POS_134_99664a15_34 | RP | POS | 15.34 | 134.9966 | | | | 1.43E-01 | -0.32 | 1.06 | 6.81E-01 | -0.08 | 0.27 | 1.30E-01 | -0.36 | 0.92 | 2.37E-01 | -0.28 | 0.99 | 34451877 | 12433617 | 30803565 | 10756227 | 31248289 | 10900865 | 30358842 | 10701471 |
| RP_POS_820_07513a13_713 | RP | POS | 13.71 | 820.0751 | | | | 5.83E-03 | -0.55 | 0.99 | 6.81E-01 | -0.08 | 0.81 | 8.16E-03 | -0.59 | 1.06 | 2.10E-02 | -0.51 | 0.98 | 3740102 | 1426293 | 2895061 | 1524378 | 2958084 | 1517943 | 2832039 | 1543575 |
| RP_NEG_295_73599a15_599 | RP | NEG | 15.60 | 295.7360 | | | | 3.46E-01 | -0.19 | 0.76 | 6.82E-01 | 0.08 | 0.47 | 3.05E-01 | -0.23 | 0.53 | 5.13E-01 | -0.15 | 0.66 | 2048918 | 94063 | 2030533 | 98613 | 2034605 | 99373 | 2026460 | 98685 |
| RP_NEG_816_57745a13_558 | RP | NEG | 13.56 | 816.5775 | | | | 1.17E-07 | -1.42 | 2.29 | 6.82E-01 | -0.05 | 0.75 | 9.30E-08 | -1.45 | 2.18 | 2.39E-07 | -1.39 | 1.97 | 4730108 | 1757716 | 2605128 | 943409 | 2644067 | 1017104 | 2566188 | 872129 |
| HILIC_POS_159_14932a6_167 | HILIC | POS | 6.167 | 159.1493 | | | | 8.94E-05 | -0.77 | 1.30 | 6.82E-01 | 0.08 | 0.52 | 1.37E-03 | -0.73 | 1.12 | 8.91E-05 | -0.81 | 1.58 | 924772 | 299516 | 661744 | 329621 | 648138 | 278989 | 675350 | 375906 |
| RP_NEG_487_22961a11_613 | RP | NEG | 11.61 | 487.2296 | | | | 2.37E-02 | -0.43 | 0.87 | 6.82E-01 | -0.08 | 0.58 | 3.13E-02 | -0.47 | 0.83 | 6.90E-02 | -0.38 | 0.66 | 977432 | 93539 | 931191 | 111239 | 935777 | 108671 | 926605 | 114666 |
| RP_POS_454_29236a10_389 | RP | POS | 10.39 | 454.2924 | | | | 1.59E-01 | 0.25 | 1.05 | 6.82E-01 | -0.09 | 0.83 | 3.50E-01 | 0.20 | 0.75 | 1.33E-01 | 0.29 | 0.90 | 3134937 | 953766 | 3438676 | 1310461 | 3492679 | 1165628 | 3384673 | 1450870 |
| HILIC_NEG_201_95781a1_275 | HILIC | NEG | 1.275 | 201.9578 | | | | 1.65E-01 | -0.28 | 0.96 | 6.82E-01 | -0.08 | 0.87 | 1.53E-01 | -0.33 | 0.55 | 2.87E-01 | -0.24 | 0.40 | 744190 | 152772 | 700518 | 153177 | 706824 | 155343 | 694211 | 152290 |
| RP_POS_345_23718a10_962 | RP | POS | 10.96 | 345.2372 | | | | 6.98E-02 | -0.31 | 0.93 | 6.83E-01 | 0.09 | 0.99 | 1.74E-01 | -0.27 | 0.88 | 8.65E-02 | -0.36 | 0.88 | 504125 | 108504 | 458959 | 153197 | 452656 | 159957 | 465262 | 147479 |
| RP_POS_167_07028a8_51 | RP | POS | 8.51 | 167.0703 | | | | 4.95E-01 | -0.11 | 0.52 | 6.83E-01 | -0.09 | 0.44 | 4.71E-01 | -0.16 | 0.45 | 6.96E-01 | -0.07 | 0.76 | 2933422 | 79788 | 2920678 | 121300 | 2925669 | 97958 | 2915687 | 141707 |
| RP_NEG_498_78275a15_66 | RP | NEG | 15.66 | 498.7828 | | | | 1.07E-01 | -0.26 | 0.78 | 6.83E-01 | 0.09 | 0.28 | 2.73E-01 | -0.22 | 0.55 | 1.09E-01 | -0.31 | 1.32 | 1797121 | 209655 | 1716409 | 327720 | 1702931 | 316125 | 1729888 | 341594 |
| RP_POS_859_60461a11_96 | RP | POS | 11.96 | 859.6046 | | | | 5.33E-01 | 0.13 | 0.64 | 6.83E-01 | -0.08 | 0.79 | 7.00E-01 | 0.09 | 0.64 | 4.60E-01 | 0.17 | 0.62 | 9119084 | 3540002 | 9566459 | 3415337 | 9706672 | 3408580 | 9426247 | 3450878 |
| RP_POS_377_32623a10_652 | RP | POS | 10.65 | 377.3262 | Unknown | 377.2632(70), 89.0598(100), 195.1234(65), 133.0858(45) | 4 | 2.66E-01 | -0.27 | 0.50 | 6.84E-01 | -0.07 | 0.31 | 2.37E-01 | -0.31 | 0.53 | 3.68E-01 | -0.23 | 0.37 | 5440177 | 442581 | 5344838 | 321442 | 5358033 | 321476 | 5331643 | 324123 |
| RP_NEG_322_88095a15_472 | RP | NEG | 15.47 | 322.8810 | | | | 8.50E-01 | 0.04 | 0.51 | 6.84E-01 | -0.08 | 0.47 | 9.93E-01 | 0.00 | 0.31 | 7.35E-01 | 0.08 | 0.52 | 23880221 | 744638 | 23908803 | 730891 | 23938803 | 789014 | 23878804 | 674481 |
| HILIC_POS_132_1021a4_248 | HILIC | POS | 4.248 | 132.1021 | | | | 3.98E-01 | 0.14 | 0.94 | 6.84E-01 | 0.09 | 0.86 | 3.75E-01 | 0.19 | 0.51 | 6.03E-01 | 0.10 | 0.48 | 76127022 | 25881370 | 81165445 | 37694249 | 79618539 | 34444183 | 82712351 | 40980784 |
| RP_POS_294_93909a0_494 | RP | POS | 0.49 | 294.9391 | | | | 2.43E-07 | 0.62 | 1.33 | 6.84E-01 | -0.09 | 1.20 | 2.96E-04 | 0.58 | 1.41 | 2.36E-04 | 0.67 | 1.43 | 13699544 | 1228411 | 20773495 | 12620212 | 21291319 | 13468273 | 20255671 | 11825747 |
| HILIC_POS_111_09208a3_029 | HILIC | POS | 3.029 | 111.0921 | | | | 1.74E-03 | 0.51 | 1.10 | 6.84E-01 | -0.09 | 0.72 | 1.30E-02 | 0.46 | 1.00 | 6.65E-03 | 0.55 | 1.13 | 1886544 | 224255 | 2061053 | 366558 | 2076093 | 390711 | 2046013 | 344028 |
| HILIC_POS_87_09229a4_073 | HILIC | POS | 4.073 | 87.0923 | | | | 2.71E-01 | -0.21 | 0.80 | 6.84E-01 | 0.08 | 0.43 | 4.53E-01 | -0.17 | 0.46 | 2.33E-01 | -0.25 | 0.58 | 10381220 | 3075277 | 9660162 | 3551707 | 9514436 | 3343932 | 9805887 | 3776436 |
| HILIC_NEG_115_92066a4_303 | HILIC | NEG | 4.303 | 115.9207 | | | | 8.31E-02 | 0.26 | 0.77 | 6.84E-01 | -0.09 | 0.88 | 2.93E-01 | 0.21 | 0.38 | 7.16E-02 | 0.30 | 0.79 | 37390369 | 2405080 | 38503410 | 4732561 | 38697380 | 4070697 | 38309441 | 5348270 |
| HILIC_POS_144_10202a4_148 | HILIC | POS | 4.148 | 144.1020 | | | | 4.69E-01 | 0.14 | 0.49 | 6.84E-01 | 0.08 | 0.42 | 3.95E-01 | 0.18 | 0.52 | 6.64E-01 | 0.10 | 0.21 | 7711013 | 6330150 | 8678584 | 7128385 | 8386518 | 7551704 | 8970650 | 6742584 |
| RP_POS_526_29254a10_307 | RP | POS | 10.31 | 526.2925 | | | | 1.04E-02 | 0.41 | 1.11 | 6.84E-01 | -0.09 | 0.28 | 4.99E-02 | 0.36 | 0.91 | 2.41E-02 | 0.45 | 1.01 | 28599698 | 9508912 | 34656506 | 15918695 | 35308323 | 16851059 | 34004690 | 15071695 |
| RP_POS_758_5686a15_466 | RP | POS | 15.47 | 758.5686 | | | | 1.77E-01 | -0.34 | 0.57 | 6.85E-01 | -0.07 | 0.30 | 1.55E-01 | -0.38 | 0.63 | 2.54E-01 | -0.31 | 0.46 | 364013 | 259992 | 296648 | 168849 | 303554 | 177313 | 289742 | 161445 |
| RP_POS_515_37292a13_136 | RP | POS | 13.14 | 515.3729 | | | | 7.28E-01 | -0.08 | 0.62 | 6.85E-01 | -0.08 | 0.46 | 6.34E-01 | -0.12 | 0.69 | 8.80E-01 | -0.04 | 0.50 | 481757 | 310201 | 460226 | 280206 | 471685 | 266274 | 448767 | 295743 |
| RP_POS_836_54022a11_165 | RP | POS | 11.17 | 836.5402 | | | | 2.35E-02 | 0.28 | 0.59 | 6.85E-01 | -0.09 | 0.39 | 5.46E-02 | 0.24 | 0.69 | 1.14E-01 | 0.33 | 0.60 | 924211 | 467540 | 1414251 | 1969845 | 1494843 | 2445549 | 1333658 | 1358603 |
| RP_POS_413_13568a8_81 | RP | POS | 8.81 | 413.1357 | | | | 1.66E-02 | 0.34 | 0.85 | 6.85E-01 | -0.09 | 0.51 | 1.06E-01 | 0.29 | 0.63 | 3.36E-02 | 0.38 | 0.86 | 932225 | 707302 | 1428237 | 1613188 | 1494145 | 1607527 | 1362328 | 1632437 |
| RP_NEG_691_50562a11_756 | RP | NEG | 11.76 | 691.5056 | | | | 5.29E-04 | -0.76 | 1.41 | 6.85E-01 | -0.08 | 0.24 | 4.56E-04 | -0.79 | 1.41 | 3.14E-03 | -0.72 | 1.18 | 1140772 | 428267 | 825199 | 385243 | 840937 | 438653 | 809461 | 327004 |
| RP_POS_282_90402a0_464 | RP | POS | 0.46 | 282.9040 | | | | 1.16E-06 | -0.65 | 1.31 | 6.85E-01 | 0.09 | 1.15 | 5.13E-04 | -0.61 | 1.35 | 1.22E-04 | -0.69 | 1.41 | 510801 | 86134 | 362663 | 247023 | 352574 | 251595 | 372752 | 244496 |
| HILIC_POS_218_12877a0_542 | HILIC | POS | 0.542 | 218.1288 | | | | 6.90E-05 | 0.69 | 1.40 | 6.85E-01 | -0.08 | 0.65 | 2.28E-03 | 0.65 | 1.03 | 1.14E-04 | 0.73 | 1.75 | 164446 | 167416 | 324060 | 235592 | 333682 | 206382 | 314438 | 263358 |
| HILIC_NEG_195_81091a2_951 | HILIC | NEG | 2.951 | 195.8109 | | | | 5.83E-05 | 0.53 | 1.22 | 6.85E-01 | -0.09 | 0.79 | 4.27E-03 | 0.48 | 1.19 | 1.77E-03 | 0.57 | 1.37 | 16758683 | 3318756 | 21766128 | 10523901 | 22195579 | 11011713 | 21336677 | 10105990 |
| RP_POS_321_2392a11_61 | RP | POS | 11.61 | 321.2392 | | | | 2.43E-03 | 0.53 | 0.88 | 6.86E-01 | 0.08 | 0.36 | 6.23E-03 | 0.57 | 1.09 | 1.42E-02 | 0.48 | 1.16 | 1955490 | 436805 | 2264881 | 611339 | 2239951 | 586814 | 2289811 | 639906 |
| RP_POS_137_02957a0_454 | RP | POS | 0.45 | 137.0296 | | | | 4.53E-06 | -0.64 | 1.29 | 6.86E-01 | -0.09 | 1.13 | 1.19E-04 | -0.68 | 1.38 | 1.15E-03 | -0.60 | 1.34 | 480599 | 73871 | 374214 | 179036 | 381508 | 184906 | 366919 | 174538 |
| RP_POS_249_15706a3_319 | RP | POS | 3.32 | 249.1571 | | | | 1.29E-04 | -0.61 | 1.22 | 6.86E-01 | 0.09 | 1.11 | 2.69E-03 | -0.57 | 1.22 | 8.08E-04 | -0.66 | 1.28 | 17006370 | 4625852 | 12504967 | 7714106 | 12191000 | 7881568 | 12818934 | 7609798 |
| RP_POS_510_75894a4_431 | RP | POS | 4.43 | 510.7589 | | | | 9.83E-05 | 0.46 | 1.02 | 6.86E-01 | -0.09 | 1.06 | 5.07E-03 | 0.51 | 1.14 | 7.06E-03 | 0.41 | 1.11 | 130344 | 63157 | 471540 | 834208 | 437594 | 769241 | 505485 | 901068 |
| RP_POS_176_12825a0_623 | RP | POS | 0.62 | 176.1283 | | | | 1.06E-01 | 0.31 | 0.53 | 6.86E-01 | 0.08 | 0.32 | 1.15E-01 | 0.35 | 0.69 | 2.08E-01 | 0.27 | 0.48 | 1630353 | 1754582 | 2239191 | 2036185 | 2156353 | 1939157 | 2322029 | 2145314 |
| RP_NEG_196_07428a0_538 | RP | NEG | 0.54 | 196.0743 | | | | 2.98E-05 | 0.65 | 1.22 | 6.87E-01 | -0.09 | 0.36 | 5.07E-03 | 0.60 | 0.97 | 1.75E-05 | 0.69 | 1.65 | 647048 | 156112 | 820516 | 283908 | 832064 | 208826 | 808967 | 344924 |
| HILIC_POS_371_23962a4_249 | HILIC | POS | 4.249 | 371.2396 | | | | 1.58E-01 | 0.26 | 0.92 | 6.87E-01 | 0.08 | 0.86 | 1.65E-01 | 0.30 | 0.49 | 2.89E-01 | 0.22 | 0.39 | 9985025 | 1919974 | 10575755 | 2354675 | 10480064 | 2229813 | 10671446 | 2492265 |
| RP_POS_482_40634a10_658 | RP | POS | 10.66 | 482.4063 | | | | 1.89E-01 | -0.28 | 0.49 | 6.87E-01 | -0.08 | 0.39 | 1.66E-01 | -0.32 | 0.59 | 3.14E-01 | -0.24 | 0.41 | 10739357 | 798656 | 10526941 | 745046 | 10557199 | 784343 | 10496682 | 710248 |
| RP_NEG_418_89471a0_448 | RP | NEG | 0.45 | 418.8947 | | | | 1.55E-04 | -0.53 | 1.17 | 6.87E-01 | -0.09 | 1.11 | 8.98E-04 | -0.58 | 1.25 | 9.50E-03 | -0.49 | 1.23 | 1744850 | 368314 | 1321357 | 866299 | 1356498 | 920914 | 1286217 | 815879 |
| RP_POS_374_2103a11_159 | RP | POS | 11.16 | 374.2103 | | | | 5.36E-03 | -0.55 | 0.93 | 6.87E-01 | 0.08 | 0.72 | 2.02E-02 | -0.51 | 0.84 | 7.81E-03 | -0.59 | 1.01 | 912877 | 89498 | 858977 | 97995 | 855003 | 99149 | 862952 | 97668 |
| HILIC_NEG_209_00395a0_7 | HILIC | NEG | 0.7 | 209.0040 | | | | 1.24E-04 | 0.65 | 1.10 | 6.87E-01 | 0.08 | 0.80 | 7.16E-04 | 0.69 | 1.31 | 1.58E-03 | 0.61 | 1.08 | 458936 | 161134 | 608619 | 236505 | 599029 | 225836 | 618209 | 248638 |
| RP_NEG_310_93149a0_462 | RP | NEG | 0.46 | 310.9315 | | | | 2.54E-09 | -0.74 | 1.40 | 6.87E-01 | 0.09 | 1.19 | 4.36E-05 | -0.70 | 1.48 | 9.36E-06 | -0.78 | 1.51 | 2244372 | 195200 | 1561398 | 1001162 | 1520808 | 1017848 | 1601987 | 992840 |
| RP_POS_499_82712a6_021 | RP | POS | 6.02 | 499.8271 | | | | 5.87E-01 | 0.09 | 0.62 | 6.87E-01 | 0.09 | 0.31 | 5.11E-01 | 0.13 | 0.51 | 8.07E-01 | 0.05 | 0.50 | 1553502 | 1330635 | 1720995 | 1974441 | 1640978 | 1901097 | 1801011 | 2061349 |
| HILIC_NEG_171_94711a1_273 | HILIC | NEG | 1.273 | 171.9471 | | | | 1.35E-01 | 0.28 | 1.06 | 6.87E-01 | 0.08 | 0.86 | 1.40E-01 | 0.32 | 0.59 | 2.57E-01 | 0.24 | 0.44 | 889034 | 183566 | 948124 | 217022 | 939331 | 210515 | 956918 | 225131 |
| RP_NEG_898_58087a14_004 | RP | NEG | 14.00 | 898.5809 | | | | 5.11E-02 | -0.45 | 0.78 | 6.88E-01 | 0.08 | 0.37 | 1.02E-01 | -0.41 | 0.71 | 4.65E-02 | -0.48 | 0.79 | 11340482 | 2346527 | 10424940 | 1914389 | 10347421 | 1807930 | 10502459 | 2030686 |
| RP_POS_838_55621a11_176 | RP | POS | 11.18 | 838.5562 | | | | 7.66E-01 | -0.04 | 0.44 | 6.88E-01 | -0.09 | 0.40 | 4.19E-01 | -0.08 | 0.49 | 9.71E-01 | 0.01 | 0.33 | 2084202 | 1042551 | 1964077 | 3581430 | 2109303 | 4716020 | 1818851 | 1905559 |
| HILIC_POS_189_07359a5_461 | HILIC | POS | 5.461 | 189.0736 | | | | 3.38E-01 | -0.21 | 0.42 | 6.88E-01 | 0.08 | 0.58 | 3.05E-01 | -0.25 | 0.47 | 4.70E-01 | -0.17 | 0.29 | 6914480 | 961735 | 6730716 | 845724 | 6764941 | 816487 | 6696491 | 880940 |
| RP_POS_781_52612a10_46 | RP | POS | 10.46 | 781.5261 | | | | 4.21E-01 | 0.13 | 0.35 | 6.88E-01 | 0.09 | 0.49 | 3.82E-01 | 0.17 | 0.57 | 6.54E-01 | 0.09 | 0.20 | 1558280 | 170890 | 1591501 | 278502 | 1580238 | 271626 | 1602765 | 287523 |
| RP_POS_586_83374a6_307 | RP | POS | 6.31 | 586.8337 | | | | 2.96E-06 | 0.56 | 1.27 | 6.88E-01 | -0.09 | 1.11 | 1.66E-03 | 0.52 | 1.36 | 5.39E-04 | 0.61 | 1.34 | 70155 | 310198 | 1410592 | 2659985 | 1518129 | 2744456 | 1303055 | 2596113 |
| RP_POS_200_90366a15_579 | RP | POS | 15.58 | 200.9037 | | | | 2.84E-01 | -0.18 | 0.44 | 6.89E-01 | -0.09 | 0.25 | 3.19E-01 | -0.23 | 0.51 | 4.18E-01 | -0.14 | 0.52 | 2331444 | 77084 | 2312669 | 108283 | 2317042 | 79540 | 2308296 | 131621 |
| RP_POS_407_33618a10_462 | RP | POS | 10.46 | 407.3362 | | | | 2.22E-03 | -0.54 | 1.09 | 6.89E-01 | 0.08 | 0.52 | 1.21E-02 | -0.50 | 1.05 | 5.65E-03 | -0.58 | 1.04 | 3322453 | 569914 | 2919998 | 768147 | 2889018 | 808135 | 2950979 | 732871 |
| RP_NEG_384_93573a0_495 | RP | NEG | 0.50 | 384.9357 | | | | 9.31E-07 | 0.59 | 1.32 | 6.89E-01 | -0.09 | 1.20 | 5.02E-04 | 0.55 | 1.39 | 4.96E-04 | 0.64 | 1.41 | 28614564 | 2554439 | 39790474 | 20985686 | 40636482 | 22619455 | 38944467 | 19408384 |
| RP_POS_475_41519a11_159 | RP | POS | 11.16 | 475.4152 | | | | 9.02E-02 | -0.28 | 0.88 | 6.89E-01 | 0.09 | 0.55 | 2.42E-01 | -0.23 | 0.60 | 9.83E-02 | -0.32 | 1.13 | 5245027 | 545894 | 5022601 | 867381 | 4987655 | 847293 | 5057548 | 894234 |
| RP_POS_278_2482a10_381 | RP | POS | 10.38 | 278.2482 | | | | 4.51E-04 | 0.55 | 1.00 | 6.89E-01 | -0.09 | 0.35 | 8.21E-03 | 0.50 | 0.95 | 1.95E-03 | 0.59 | 1.20 | 3416419 | 2052970 | 5287176 | 3648862 | 5434108 | 3638621 | 5140243 | 3690052 |
| RP_POS_237_22119a10_135 | RP | POS | 10.14 | 237.2212 | | | | 6.78E-03 | 0.49 | 0.92 | 6.90E-01 | -0.08 | 0.34 | 3.04E-02 | 0.45 | 0.77 | 1.06E-02 | 0.54 | 1.16 | 819273 | 78809 | 867122 | 99366 | 871121 | 99297 | 863123 | 100281 |
| RP_NEG_342_73383a0_522 | RP | NEG | 0.52 | 342.7338 | | | | 1.85E-07 | -0.80 | 1.38 | 6.90E-01 | -0.08 | 1.06 | 3.79E-05 | -0.76 | 1.40 | 1.12E-05 | -0.84 | 1.46 | 645499 | 102045 | 499180 | 188362 | 491600 | 193366 | 506759 | 184869 |
| RP_POS_244_99725a0_74 | RP | POS | 0.74 | 244.9973 | | | | 2.80E-07 | 0.79 | 1.31 | 6.90E-01 | -0.08 | 0.73 | 4.92E-05 | 0.75 | 1.42 | 1.54E-05 | 0.83 | 1.39 | 706468 | 599345 | 1551450 | 1106987 | 1595981 | 1139049 | 1506920 | 1083684 |
| RP_NEG_548_85266a0_448 | RP | NEG | 0.45 | 548.8527 | | | | 1.24E-03 | -0.47 | 1.11 | 6.90E-01 | -0.09 | 1.08 | 3.53E-03 | -0.51 | 1.16 | 2.69E-02 | -0.42 | 1.17 | 695204 | 177254 | 531960 | 380448 | 547249 | 406524 | 516671 | 355945 |
| RP_POS_834_74548a0_499 | RP | POS | 0.50 | 834.7455 | | | | 1.43E-02 | -0.34 | 1.14 | 6.90E-01 | -0.09 | 1.23 | 2.39E-02 | -0.39 | 1.15 | 1.16E-01 | -0.30 | 1.18 | 2163862 | 491790 | 1796414 | 1195197 | 1844441 | 1279799 | 1748388 | 1115147 |
| RP_POS_196_00307a0_67 | RP | POS | 0.67 | 196.0031 | | | | 3.85E-05 | 0.53 | 1.20 | 6.90E-01 | -0.09 | 1.11 | 3.92E-03 | 0.48 | 1.21 | 1.50E-03 | 0.57 | 1.31 | 916157 | 121413 | 1120755 | 429128 | 1137996 | 446316 | 1103515 | 415036 |
| RP_NEG_338_87381a15_658 | RP | NEG | 15.66 | 338.8738 | | | | 7.17E-02 | -0.35 | 0.77 | 6.90E-01 | 0.08 | 0.42 | 8.49E-02 | -0.39 | 0.68 | 1.42E-01 | -0.31 | 0.75 | 3466570 | 371005 | 3324684 | 409363 | 3341128 | 376876 | 3308240 | 442706 |
| RP_POS_221_17497a11_128 | RP | POS | 11.13 | 221.1750 | | | | 1.56E-02 | 0.39 | 0.84 | 6.90E-01 | -0.09 | 0.34 | 5.48E-02 | 0.35 | 0.73 | 3.71E-02 | 0.43 | 0.80 | 444109 | 541135 | 764883 | 881537 | 800279 | 976472 | 729486 | 783593 |
| HILIC_NEG_172_8423a2_946 | HILIC | NEG | 2.946 | 172.8423 | | | | 1.68E-06 | 0.58 | 1.30 | 6.90E-01 | -0.09 | 0.77 | 8.36E-04 | 0.54 | 1.39 | 4.91E-04 | 0.63 | 1.50 | 319857 | 42749 | 458167 | 263072 | 468728 | 278150 | 447605 | 249461 |
| HILIC_POS_448_05026a3_077 | HILIC | POS | 3.077 | 448.0503 | | | | 2.56E-02 | 0.29 | 1.20 | 6.90E-01 | -0.09 | 0.85 | 1.19E-01 | 0.25 | 1.22 | 7.35E-02 | 0.34 | 1.38 | 770415 | 45683 | 806421 | 137471 | 811939 | 150856 | 800904 | 123945 |
| RP_POS_391_23505a11_31 | RP | POS | 11.31 | 391.2351 | | | | 1.83E-02 | -0.47 | 0.90 | 6.90E-01 | 0.08 | 0.75 | 4.57E-02 | -0.43 | 0.83 | 2.61E-02 | -0.51 | 0.92 | 1614261 | 227514 | 1499989 | 242904 | 1490243 | 261225 | 1509736 | 225336 |
| RP_POS_438_37848a10_708 | RP | POS | 10.71 | 438.3785 | | | | 8.07E-01 | -0.05 | 0.35 | 6.90E-01 | -0.08 | 0.65 | 6.93E-01 | -0.09 | 0.26 | 9.65E-01 | -0.01 | 0.25 | 8064370 | 842830 | 8022366 | 840018 | 8056067 | 842619 | 7988665 | 844592 |
| RP_POS_158_96429a0_495 | RP | POS | 0.50 | 158.9643 | | | | 2.60E-07 | 0.62 | 1.33 | 6.91E-01 | -0.09 | 1.20 | 2.98E-04 | 0.58 | 1.41 | 2.43E-04 | 0.66 | 1.43 | 20323041 | 1468471 | 27981966 | 13668832 | 28529982 | 14578315 | 27433950 | 12819781 |
| RP_NEG_210_90118a15_597 | RP | NEG | 15.60 | 210.9012 | | | | 1.12E-01 | -0.37 | 0.81 | 6.91E-01 | -0.07 | 0.33 | 1.08E-01 | -0.41 | 0.73 | 1.76E-01 | -0.33 | 0.86 | 1552417 | 65092 | 1531989 | 50896 | 1534029 | 48862 | 1529949 | 53271 |
| RP_NEG_894_54956a13_073 | RP | NEG | 13.07 | 894.5496 | | | | 1.00E-01 | -0.34 | 0.75 | 6.91E-01 | -0.08 | 0.28 | 8.07E-02 | -0.38 | 0.75 | 2.15E-01 | -0.30 | 0.80 | 6986497 | 2110441 | 6268439 | 2123341 | 6353551 | 2417523 | 6183326 | 1802767 |
| RP_NEG_246_07559a5_074 | RP | NEG | 5.07 | 246.0756 | | | | 1.69E-01 | -0.49 | 0.93 | 6.91E-01 | -0.01 | 0.29 | 1.63E-01 | -0.50 | 0.70 | 1.76E-01 | -0.49 | 0.70 | 1557754 | 4730449 | 378297 | 428341 | 395460 | 458039 | 361134 | 400370 |
| RP_POS_489_31497a11_476 | RP | POS | 11.48 | 489.3150 | | | | 7.94E-01 | 0.05 | 0.40 | 6.91E-01 | 0.08 | 0.77 | 6.83E-01 | 0.09 | 0.43 | 9.52E-01 | 0.01 | 0.46 | 1666406 | 180315 | 1675988 | 177509 | 1668877 | 178287 | 1683099 | 178249 |
| RP_POS_304_24869a9_527 | RP | POS | 9.53 | 304.2487 | | | | 5.21E-02 | -0.39 | 0.98 | 6.91E-01 | 0.08 | 0.69 | 1.19E-01 | -0.35 | 0.84 | 5.60E-02 | -0.43 | 0.92 | 881837 | 51127 | 860906 | 54290 | 858732 | 54599 | 863080 | 54445 |
| RP_POS_197_01042a15_45 | RP | POS | 15.45 | 197.0104 | | | | 5.69E-02 | -0.36 | 1.07 | 6.92E-01 | -0.08 | 0.27 | 5.40E-02 | -0.40 | 0.83 | 1.49E-01 | -0.32 | 1.54 | 15510403 | 3269517 | 14168117 | 3794746 | 14319653 | 4087928 | 14016582 | 3512271 |
| RP_POS_337_23535a10_63 | RP | POS | 10.63 | 337.2354 | | | | 3.46E-03 | -0.64 | 1.34 | 6.92E-01 | 0.08 | 0.91 | 1.29E-02 | -0.60 | 1.16 | 3.47E-03 | -0.67 | 1.34 | 7957339 | 1603526 | 6978201 | 1450812 | 6920276 | 1328652 | 7036126 | 1574961 |
| RP_POS_150_96906a15_627 | RP | POS | 15.63 | 150.9691 | | | | 5.60E-01 | 0.13 | 1.26 | 6.92E-01 | -0.08 | 0.44 | 7.16E-01 | 0.09 | 0.76 | 4.80E-01 | 0.17 | 1.07 | 26583079 | 10748014 | 27827447 | 9458977 | 28205053 | 8942989 | 27449841 | 10025088 |
| HILIC_POS_175_10774a5_847 | HILIC | POS | 5.847 | 175.1077 | | | | 2.72E-02 | 0.36 | 0.70 | 6.92E-01 | 0.09 | 0.66 | 6.12E-02 | 0.41 | 0.62 | 6.65E-02 | 0.32 | 0.79 | 1321815 | 736407 | 1711652 | 1144712 | 1665964 | 928381 | 1757340 | 1334661 |
| RP_POS_196_00238a15_547 | RP | POS | 15.55 | 196.0024 | | | | 6.81E-01 | -0.09 | 0.38 | 6.92E-01 | -0.08 | 0.61 | 5.94E-01 | -0.13 | 0.34 | 8.27E-01 | -0.05 | 0.53 | 83666123 | 8528295 | 82971162 | 7353798 | 83264546 | 7284466 | 82677777 | 7484732 |
| HILIC_NEG_112_89616a2_948 | HILIC | NEG | 2.948 | 112.8962 | | | | 1.02E-05 | -0.58 | 1.34 | 6.92E-01 | 0.09 | 0.79 | 1.09E-03 | -0.54 | 1.45 | 8.25E-04 | -0.63 | 1.42 | 3825882 | 430106 | 3131844 | 1305646 | 3079761 | 1401278 | 3183927 | 1214535 |
| RP_POS_957_2063a9_727 | RP | POS | 9.73 | 957.2063 | | | | 3.75E-01 | 0.18 | 0.51 | 6.92E-01 | -0.08 | 0.26 | 5.36E-01 | 0.14 | 0.36 | 3.35E-01 | 0.22 | 0.44 | 7258769 | 511520 | 7352321 | 525450 | 7373279 | 534271 | 7331363 | 521046 |
| RP_NEG_666_84265a0_466 | RP | NEG | 0.47 | 666.8427 | | | | 5.71E-09 | -0.73 | 1.39 | 6.92E-01 | 0.09 | 1.20 | 5.11E-05 | -0.68 | 1.47 | 1.56E-05 | -0.77 | 1.49 | 789525 | 76909 | 550397 | 356323 | 536192 | 365940 | 564601 | 349571 |
| RP_POS_802_53479a12_466 | RP | POS | 12.47 | 802.5348 | | | | 3.09E-03 | -0.69 | 1.17 | 6.92E-01 | -0.07 | 0.58 | 4.48E-03 | -0.73 | 1.22 | 6.38E-03 | -0.66 | 1.07 | 2730910 | 1564888 | 1784190 | 1217721 | 1832739 | 1069719 | 1735641 | 1358985 |
| RP_POS_579_29224a9_44 | RP | POS | 9.44 | 579.2922 | | | | 6.11E-02 | 0.30 | 0.57 | 6.92E-01 | -0.09 | 0.29 | 1.63E-01 | 0.26 | 0.68 | 9.31E-02 | 0.34 | 0.62 | 445636 | 61434 | 473948 | 102405 | 478028 | 110739 | 469868 | 94290 |
| RP_POS_782_56866a14_258 | RP | POS | 14.26 | 782.5687 | | | | 1.65E-03 | 0.42 | 0.82 | 6.92E-01 | 0.09 | 0.51 | 2.95E-02 | 0.46 | 0.81 | 4.20E-03 | 0.37 | 0.95 | 1781865 | 897580 | 2770196 | 2632613 | 2665234 | 1794275 | 2875158 | 3280372 |
| HILIC_POS_230_13853a2_074 | HILIC | POS | 2.074 | 230.1385 | | | | 4.45E-04 | 0.51 | 1.17 | 6.93E-01 | 0.09 | 0.32 | 4.50E-03 | 0.56 | 1.15 | 6.41E-03 | 0.47 | 1.21 | 366443 | 614229 | 977013 | 1290581 | 925758 | 1184300 | 1028268 | 1399023 |
| RP_POS_410_84726a0_499 | RP | POS | 0.50 | 410.8473 | | | | 1.75E-03 | -0.42 | 1.19 | 6.94E-01 | -0.09 | 1.24 | 5.83E-03 | -0.46 | 1.24 | 4.36E-02 | -0.37 | 1.23 | 8416853 | 1533671 | 6771052 | 4376696 | 6944753 | 4679653 | 6597352 | 4091593 |

**Supplementary table 1: Metabolomics results** (in asceding order according to p-value from progressive ALD vs  non-progressive ALD comparison, with molecular features with ID_level of 1-2 first)

| | | | | | | | | all_ALD_vs_control | | | Prog_ALD_vs_non-prog_ALD | | | Prog_ALD_vs_control_ALD | | | Non-prog_ALD_vs_control | | | Healthy_control | | all_ALD | | Non-prog_ALD | | Progressive_ALD | |
|---|---|---|---|---|---|---|---|---|---|---|---|---|---|---|---|---|---|---|---|---|---|---|---|---|---|---|---|
| | | | | | | | | t-test | Cohen's | PLS-DA | t-test | Cohen's | PLS-DA | t-test | Cohen's | PLS-DA | t-test | Cohen's | PLS-DA | | | | | | | | |
| Feature_ID | RP_HILIC | POS_NEG | Average.Rt | Average.Mz | Metabolite.name | curated_MSMS | ID_level | p | d | VIP | p | d | VIP | p | d | VIP | p | d | VIP | Mean | SD | Mean | SD | Mean | SD | Mean | SD |
| HILIC_POS_86_09711a4_248 | HILIC | POS | 4.248 | 86.0971 | | | | 3.84E-01 | 0.15 | 0.91 | 6.94E-01 | 0.08 | 0.87 | 3.67E-01 | 0.19 | 0.48 | 5.81E-01 | 0.11 | 0.45 | 13694825 | 4441624 | 14579554 | 6372540 | 14326800 | 5839266 | 14832308 | 6915193 |
| RP_POS_452_83173a0_512 | RP | POS | 0.51 | 452.8317 | | | | 1.23E-05 | -0.71 | 1.22 | 6.94E-01 | -0.08 | 0.98 | 7.69E-05 | -0.75 | 1.36 | 7.70E-04 | -0.67 | 1.20 | 375889 | 100302 | 263610 | 164893 | 270147 | 173084 | 257073 | 157762 |
| RP_POS_345_21573a11_609 | RP | POS | 11.61 | 345.2157 | | | | 6.85E-01 | -0.07 | 0.65 | 6.94E-01 | 0.08 | 0.72 | 8.74E-01 | -0.03 | 0.53 | 6.05E-01 | -0.11 | 0.81 | 1552061 | 366642 | 1519400 | 473390 | 1500630 | 530249 | 1538170 | 413384 |
| RP_NEG_242_94353a0_46 | RP | NEG | 0.46 | 242.9435 | | | | 1.38E-08 | -0.71 | 1.38 | 6.94E-01 | 0.09 | 1.19 | 9.68E-05 | -0.66 | 1.45 | 2.28E-05 | -0.75 | 1.49 | 3256898 | 298077 | 2303624 | 1472663 | 2245253 | 1499255 | 2361995 | 1458429 |
| RP_NEG_294_95297a15_288 | RP | NEG | 15.29 | 294.9530 | | | | 4.77E-01 | -0.15 | 0.51 | 6.94E-01 | -0.08 | 0.26 | 3.82E-01 | -0.19 | 0.46 | 6.64E-01 | -0.11 | 0.40 | 2132944 | 90597 | 2119723 | 91396 | 2123344 | 106755 | 2116101 | 73848 |
| HILIC_POS_195_12273a0_615 | HILIC | POS | 0.615 | 195.1227 | | | | 8.54E-01 | -0.03 | 1.04 | 6.94E-01 | -0.08 | 0.88 | 7.12E-01 | -0.07 | 0.48 | 9.63E-01 | 0.01 | 0.26 | 8138216 | 4377566 | 7957538 | 5939822 | 8192658 | 6185381 | 7722418 | 5736831 |
| RP_POS_232_89619a0_515 | RP | POS | 0.52 | 232.8962 | | | | 2.04E-04 | -0.67 | 1.10 | 6.94E-01 | 0.08 | 0.65 | 3.37E-03 | -0.63 | 1.02 | 3.49E-04 | -0.71 | 1.22 | 1917297 | 410031 | 1562865 | 537999 | 1541570 | 489329 | 1584160 | 586878 |
| RP_POS_156_0768a0_708 | RP | POS | 0.71 | 156.0768 | | | | 7.12E-02 | -0.63 | 1.15 | 6.94E-01 | 0.02 | 0.38 | 7.75E-02 | -0.62 | 0.86 | 6.72E-02 | -0.64 | 0.87 | 1237109 | 2740062 | 329126 | 433625 | 311966 | 432761 | 346287 | 438196 |
| RP_NEG_385_63336a15_599 | RP | NEG | 15.60 | 385.6334 | | | | 7.30E-01 | -0.07 | 0.80 | 6.94E-01 | 0.08 | 0.42 | 9.14E-01 | -0.02 | 0.30 | 6.07E-01 | -0.11 | 0.69 | 613053 | 31295 | 610747 | 36704 | 609295 | 33455 | 612199 | 39979 |
| HILIC_NEG_197_80795a3_94 | HILIC | NEG | 3.94 | 197.8080 | | | | 6.53E-01 | 0.07 | 0.56 | 6.94E-01 | 0.09 | 0.51 | 5.80E-01 | 0.12 | 0.40 | 8.66E-01 | 0.03 | 0.21 | 507084 | 556783 | 566536 | 874287 | 531939 | 768519 | 601134 | 975341 |
| HILIC_NEG_226_94025a5_606 | HILIC | NEG | 5.606 | 226.9403 | | | | 2.54E-01 | -0.23 | 1.07 | 6.95E-01 | -0.08 | 0.51 | 2.42E-01 | -0.27 | 1.02 | 3.94E-01 | -0.19 | 1.14 | 186491 | 61795 | 171735 | 66498 | 174365 | 63308 | 169106 | 70088 |
| RP_POS_198_08513a0_549 | RP | POS | 0.55 | 198.0851 | | | | 2.24E-07 | 0.73 | 1.37 | 6.95E-01 | 0.08 | 1.19 | 1.88E-05 | 0.77 | 1.53 | 1.66E-04 | 0.69 | 1.30 | 447741 | 111675 | 628399 | 263007 | 617999 | 268575 | 638799 | 259621 |
| HILIC_NEG_173_92632a2_947 | HILIC | NEG | 2.947 | 173.9263 | | | | 6.00E-01 | -0.10 | 0.70 | 6.95E-01 | 0.08 | 0.63 | 7.93E-01 | -0.06 | 0.68 | 4.98E-01 | -0.14 | 0.32 | 951293 | 252259 | 923268 | 288575 | 911857 | 260758 | 934680 | 316213 |
| RP_POS_441_29733a13_475 | RP | POS | 13.48 | 441.2973 | | | | 8.36E-01 | -0.04 | 0.67 | 6.95E-01 | -0.08 | 0.48 | 7.32E-01 | -0.08 | 0.27 | 9.81E-01 | -0.01 | 0.73 | 13716108 | 6115164 | 13462894 | 5609903 | 13684682 | 5168957 | 13241106 | 6063480 |
| RP_POS_696_89886a6_668 | RP | POS | 6.67 | 696.8989 | | | | 4.62E-07 | 0.60 | 1.30 | 6.95E-01 | -0.09 | 1.12 | 4.99E-04 | 0.56 | 1.42 | 3.14E-04 | 0.64 | 1.36 | 26737 | 59480 | 1275463 | 2311537 | 1366829 | 2442611 | 1184097 | 2193643 |
| RP_POS_846_5401a13_2 | RP | POS | 13.20 | 846.5401 | | | | 2.56E-03 | 0.64 | 1.16 | 6.95E-01 | 0.08 | 0.63 | 6.71E-03 | 0.68 | 1.01 | 4.46E-03 | 0.60 | 1.32 | 2520142 | 795625 | 3027274 | 757316 | 2997353 | 541472 | 3057195 | 929376 |
| RP_POS_322_95074a15_429 | RP | POS | 15.43 | 322.9507 | | | | 4.50E-01 | -0.17 | 0.32 | 6.95E-01 | 0.08 | 0.31 | 5.57E-01 | -0.13 | 0.44 | 4.34E-01 | -0.20 | 0.47 | 15654954 | 1385422 | 15446245 | 1223287 | 15397913 | 1529309 | 15494577 | 824530 |
| HILIC_NEG_151_87407a2_949 | HILIC | NEG | 2.949 | 151.8741 | | | | 5.70E-03 | 0.40 | 1.12 | 6.95E-01 | -0.09 | 0.78 | 4.43E-02 | 0.36 | 0.94 | 1.91E-02 | 0.44 | 1.20 | 663435 | 128180 | 763741 | 274395 | 774569 | 286129 | 752913 | 264600 |
| HILIC_POS_154_1591a5_647 | HILIC | POS | 5.647 | 154.1591 | | | | 2.79E-01 | -0.19 | 0.90 | 6.95E-01 | 0.08 | 0.85 | 4.59E-01 | -0.14 | 0.70 | 2.80E-01 | -0.23 | 0.95 | 19215010 | 1519907 | 18839296 | 2155566 | 18754286 | 2313257 | 18924307 | 2005455 |
| RP_POS_475_30539a10_662 | RP | POS | 10.66 | 475.3054 | | | | 1.88E-01 | -0.28 | 0.84 | 6.96E-01 | -0.08 | 0.42 | 1.90E-01 | -0.32 | 0.74 | 2.81E-01 | -0.24 | 0.59 | 908139 | 271375 | 835823 | 250852 | 845709 | 222634 | 825937 | 278158 |
| RP_NEG_335_04987a0_708 | RP | NEG | 0.71 | 335.0499 | | | | 3.89E-02 | 0.35 | 0.85 | 6.96E-01 | -0.08 | 0.34 | 1.33E-01 | 0.31 | 0.83 | 4.40E-02 | 0.39 | 0.78 | 51450870 | 16779293 | 59487702 | 24141193 | 60438461 | 22896527 | 58536943 | 25522392 |
| RP_NEG_134_89345a11_619 | RP | NEG | 11.62 | 134.8935 | | | | 9.77E-01 | -0.01 | 0.56 | 6.96E-01 | 0.08 | 0.50 | 8.89E-01 | 0.03 | 0.48 | 8.45E-01 | -0.05 | 0.84 | 253678 | 88643 | 253157 | 84368 | 249835 | 82204 | 256479 | 87185 |
| RP_NEG_409_31317a11_586 | RP | NEG | 11.59 | 409.3132 | FA 28:7 | 409.3094(100) | 3 | 2.12E-01 | 0.23 | 0.81 | 6.96E-01 | 0.08 | 0.81 | 1.89E-01 | 0.27 | 0.81 | 3.85E-01 | 0.19 | 0.77 | 860769 | 75247 | 881249 | 92963 | 877594 | 98370 | 884904 | 88071 |
| HILIC_POS_199_18037a5_921 | HILIC | POS | 5.921 | 199.1804 | | | | 9.97E-01 | 0.00 | 0.65 | 6.96E-01 | -0.08 | 0.57 | 8.52E-01 | -0.04 | 0.45 | 8.52E-01 | 0.04 | 0.29 | 3533077 | 921384 | 3532452 | 1053737 | 3573859 | 1019429 | 3491045 | 1095742 |
| RP_POS_289_21606a8_256 | RP | POS | 8.26 | 289.2161 | | | | 1.83E-01 | 0.27 | 0.80 | 6.97E-01 | -0.08 | 0.63 | 3.00E-01 | 0.23 | 0.67 | 1.75E-01 | 0.31 | 0.72 | 414867 | 431123 | 533116 | 431494 | 550067 | 443700 | 516166 | 422750 |
| RP_POS_531_29901a6_896 | RP | POS | 6.90 | 531.2990 | | | | 8.91E-08 | 0.64 | 1.35 | 6.97E-01 | -0.09 | 1.17 | 2.29E-04 | 0.60 | 1.46 | 1.30E-04 | 0.68 | 1.42 | 19954 | 24020 | 934793 | 1583866 | 997008 | 1662606 | 872578 | 1515354 |
| RP_POS_198_18491a10_856 | RP | POS | 10.86 | 198.1849 | | | | 7.10E-04 | 0.54 | 0.87 | 6.97E-01 | 0.08 | 0.69 | 6.53E-03 | 0.59 | 0.99 | 3.10E-03 | 0.50 | 1.03 | 597020 | 197241 | 764786 | 327079 | 751937 | 260724 | 777634 | 384487 |
| RP_POS_444_33182a10_379 | RP | POS | 10.38 | 444.3318 | | | | 3.03E-05 | 0.49 | 0.94 | 6.97E-01 | -0.09 | 0.75 | 9.04E-03 | 0.45 | 0.97 | 1.05E-03 | 0.54 | 1.09 | 58949 | 55974 | 472460 | 941260 | 509419 | 912191 | 435502 | 977315 |
| RP_NEG_129_05458a3_967 | RP | NEG | 3.97 | 129.0546 | | | | 4.95E-01 | 0.11 | 0.87 | 6.97E-01 | -0.09 | 0.29 | 7.49E-01 | 0.07 | 0.61 | 3.83E-01 | 0.15 | 0.46 | 48727724 | 12351919 | 50736165 | 19541701 | 51503306 | 16199988 | 49969023 | 22536835 |
| HILIC_POS_468_30801a1_413 | HILIC | POS | 1.413 | 468.3080 | | | | 6.47E-06 | 0.65 | 1.01 | 6.97E-01 | -0.08 | 0.28 | 1.08E-03 | 0.61 | 1.05 | 1.08E-04 | 0.70 | 1.30 | 10824075 | 3829640 | 15865261 | 8224537 | 16187415 | 7950478 | 15543107 | 8558305 |
| HILIC_POS_154_15919a5_12 | HILIC | POS | 5.12 | 154.1592 | | | | 6.08E-01 | -0.10 | 0.32 | 6.97E-01 | 0.08 | 0.77 | 8.05E-01 | -0.06 | 0.31 | 4.94E-01 | -0.14 | 0.45 | 15984985 | 5177610 | 15422590 | 5957693 | 15189249 | 5002749 | 15655932 | 6824511 |
| RP_POS_509_40408a10_663 | RP | POS | 10.66 | 509.4041 | | | | 3.50E-01 | -0.18 | 0.37 | 6.98E-01 | -0.08 | 0.36 | 2.84E-01 | -0.22 | 0.55 | 5.37E-01 | -0.15 | 0.38 | 4111834 | 362239 | 4041330 | 387599 | 4056500 | 440220 | 4026161 | 330558 |
| HILIC_NEG_115_92064a7_922 | HILIC | NEG | 7.922 | 115.9206 | | | | 2.18E-01 | -0.25 | 0.44 | 6.98E-01 | 0.08 | 0.36 | 3.59E-01 | -0.21 | 0.38 | 1.92E-01 | -0.29 | 0.54 | 60022275 | 16681380 | 55781017 | 16899193 | 55120565 | 16048774 | 56441469 | 17847698 |
| HILIC_NEG_184_99104a0_83 | HILIC | NEG | 0.83 | 184.9910 | | | | 3.53E-01 | 0.16 | 0.84 | 6.98E-01 | -0.08 | 0.92 | 5.92E-01 | 0.12 | 0.32 | 2.78E-01 | 0.20 | 0.46 | 501324 | 166777 | 536825 | 238931 | 546161 | 201653 | 527490 | 272945 |
| RP_POS_356_27936a9_705 | RP | POS | 9.71 | 356.2794 | | | | 5.37E-01 | 0.11 | 0.60 | 6.98E-01 | 0.08 | 0.38 | 3.75E-01 | 0.15 | 0.59 | 7.81E-01 | 0.07 | 0.51 | 366693 | 208701 | 395876 | 291446 | 384482 | 369039 | 407269 | 187512 |
| RP_POS_798_59875a13_662 | RP | POS | 13.66 | 798.5988 | | | | 1.05E-05 | -1.04 | 1.75 | 6.98E-01 | 0.07 | 0.40 | 3.81E-05 | -1.00 | 1.72 | 1.52E-05 | -1.07 | 1.59 | 2230874 | 929283 | 1337245 | 720620 | 1309104 | 732950 | 1365385 | 714391 |
| RP_POS_569_43127a15_414 | RP | POS | 15.41 | 569.4313 | | | | 3.21E-01 | 0.20 | 0.39 | 6.98E-01 | -0.08 | 0.36 | 4.89E-01 | 0.16 | 0.41 | 2.69E-01 | 0.24 | 0.45 | 14222673 | 6444285 | 15539706 | 6545902 | 15795273 | 5882370 | 15284139 | 7199761 |
| RP_POS_281_91116a15_524 | RP | POS | 15.52 | 281.9112 | | | | 6.66E-01 | -0.08 | 0.31 | 6.98E-01 | 0.08 | 0.30 | 8.42E-01 | -0.04 | 0.47 | 5.85E-01 | -0.12 | 0.77 | 9339471 | 402149 | 9302553 | 466038 | 9284368 | 502994 | 9320738 | 430275 |
| RP_POS_674_88623a6_603 | RP | POS | 6.60 | 674.8862 | | | | 3.33E-07 | 0.61 | 1.31 | 6.99E-01 | -0.09 | 1.14 | 5.22E-04 | 0.56 | 1.41 | 2.19E-04 | 0.65 | 1.38 | 32873 | 74311 | 1442399 | 2572566 | 1542706 | 2672887 | 1342092 | 2491264 |
| RP_POS_197_12842a3_729 | RP | POS | 3.73 | 197.1284 | | | | 2.42E-01 | 0.21 | 0.95 | 6.99E-01 | -0.08 | 0.41 | 4.19E-01 | 0.17 | 0.58 | 2.30E-01 | 0.25 | 1.15 | 4664156 | 2443595 | 5300495 | 3224360 | 5426214 | 3243080 | 5174776 | 3233471 |
| RP_POS_798_56317a11_911 | RP | POS | 11.91 | 798.5632 | | | | 3.09E-01 | 0.15 | 0.55 | 6.99E-01 | -0.09 | 0.44 | 5.72E-01 | 0.10 | 0.32 | 3.05E-01 | 0.19 | 0.60 | 1428847 | 1776616 | 1941512 | 3918409 | 2094257 | 3971647 | 1788767 | 3898667 |
| HILIC_NEG_115_92075a2_169 | HILIC | NEG | 2.169 | 115.9208 | | | | 7.53E-01 | 0.06 | 0.58 | 7.00E-01 | -0.08 | 0.53 | 9.27E-01 | 0.02 | 0.20 | 6.27E-01 | 0.10 | 0.20 | 33941859 | 7007344 | 34405735 | 7815494 | 34709618 | 6871466 | 34101851 | 8718081 |
| RP_NEG_264_9888a0_708 | RP | NEG | 0.71 | 264.9888 | | | | 1.76E-02 | 0.37 | 0.96 | 7.00E-01 | -0.08 | 0.33 | 6.92E-02 | 0.33 | 0.93 | 3.85E-02 | 0.42 | 0.80 | 1849732 | 501470 | 2148546 | 861758 | 2181986 | 923483 | 2115107 | 803310 |
| RP_POS_271_18756a8_222 | RP | POS | 8.22 | 271.1876 | | | | 6.33E-02 | 0.40 | 0.75 | 7.00E-01 | -0.08 | 0.33 | 1.20E-01 | 0.36 | 0.64 | 6.69E-02 | 0.44 | 0.92 | 2220004 | 1275731 | 2703002 | 1172616 | 2748491 | 1214166 | 2657513 | 1140041 |
| HILIC_POS_162_11247a5_457 | HILIC | POS | 5.457 | 162.1125 | | | | 3.62E-03 | 0.47 | 1.04 | 7.01E-01 | -0.08 | 0.56 | 4.41E-02 | 0.43 | 0.69 | 3.62E-03 | 0.51 | 1.01 | 1712260 | 453029 | 2040313 | 749924 | 2069357 | 622716 | 2011269 | 864148 |
| RP_NEG_360_27634a9_149 | RP | NEG | 9.15 | 360.2763 | | | | 1.70E-01 | 0.24 | 0.48 | 7.01E-01 | 0.08 | 0.42 | 2.30E-01 | 0.28 | 0.59 | 2.37E-01 | 0.20 | 0.51 | 453845 | 821269 | 706047 | 1092389 | 663718 | 701126 | 748377 | 1384105 |
| RP_POS_243_19553a8_795 | RP | POS | 8.80 | 243.1955 | | | | 5.48E-01 | -0.12 | 0.63 | 7.01E-01 | 0.08 | 0.46 | 7.20E-01 | -0.08 | 0.47 | 4.82E-01 | -0.16 | 0.39 | 1146694 | 123672 | 1131231 | 133413 | 1126068 | 136802 | 1136394 | 131119 |
| RP_POS_341_30481a10_643 | RP | POS | 10.64 | 341.3048 | | | | 1.87E-03 | -0.56 | 1.09 | 7.01E-01 | 0.08 | 0.88 | 1.02E-02 | -0.52 | 1.08 | 4.43E-03 | -0.60 | 1.06 | 1275934 | 225422 | 1116201 | 290330 | 1104966 | 301223 | 1127436 | 281620 |
| RP_POS_217_10706a4_736 | RP | POS | 4.74 | 217.1071 | | | | 3.19E-01 | -0.18 | 0.56 | 7.01E-01 | 0.08 | 0.53 | 5.18E-01 | -0.14 | 0.51 | 2.82E-01 | -0.22 | 0.40 | 1959981 | 204520 | 1914913 | 266437 | 1904614 | 255399 | 1925211 | 279250 |
| HILIC_POS_236_16452a0_819 | HILIC | POS | 0.819 | 236.1645 | | | | 1.31E-01 | 0.17 | 0.47 | 7.02E-01 | -0.09 | 0.54 | 1.39E-01 | 0.13 | 0.60 | 3.10E-01 | 0.22 | 0.47 | 11267 | 7309 | 386164 | 2462495 | 481288 | 3241046 | 291040 | 1314791 |
| RP_POS_166_08409a0_607 | RP | POS | 0.61 | 166.0841 | | | | 3.39E-05 | 0.59 | 1.21 | 7.02E-01 | -0.08 | 0.89 | 1.26E-03 | 0.55 | 1.19 | 1.05E-03 | 0.63 | 1.28 | 250341 | 135321 | 417021 | 303552 | 428729 | 327907 | 405312 | 279939 |
| HILIC_NEG_295_73621a0_552 | HILIC | NEG | 0.552 | 295.7362 | | | | 5.15E-01 | -0.14 | 0.31 | 7.02E-01 | -0.08 | 0.75 | 4.52E-01 | -0.18 | 0.29 | 6.67E-01 | -0.10 | 0.33 | 147441 | 91034 | 135528 | 84161 | 138769 | 84790 | 132287 | 84262 |
| RP_POS_519_23932a15_438 | RP | POS | 15.44 | 519.2393 | | | | 5.40E-01 | -0.12 | 0.63 | 7.02E-01 | -0.08 | 0.27 | 4.74E-01 | -0.16 | 0.45 | 7.24E-01 | -0.08 | 0.81 | 1974088 | 224424 | 1944671 | 264726 | 1954860 | 261337 | 1934482 | 270335 |
| HILIC_NEG_201_80215a3_033 | HILIC | NEG | 3.033 | 201.8022 | | | | 3.14E-06 | 0.57 | 1.32 | 7.02E-01 | -0.09 | 0.82 | 9.50E-04 | 0.53 | 1.44 | 7.11E-04 | 0.62 | 1.49 | 15466803 | 501456 | 16828848 | 2636872 | 16930318 | 2811972 | 16727377 | 2473842 |
| RP_POS_902_73163a0_499 | RP | POS | 0.50 | 902.7316 | | | | 7.97E-03 | -0.39 | 1.14 | 7.02E-01 | -0.08 | 1.22 | 1.42E-02 | -0.43 | 1.14 | 7.40E-02 | -0.35 | 1.17 | 1850468 | 433928 | 1528771 | 910508 | 1563806 | 977722 | 1493735 | 846476 |
| RP_NEG_379_17865a11_254 | RP | NEG | 11.25 | 379.1787 | | | | 2.39E-06 | 0.92 | 1.78 | 7.03E-01 | 0.07 | 0.99 | 1.60E-05 | 0.95 | 1.72 | 2.15E-05 | 0.88 | 1.72 | 793269 | 302033 | 1125205 | 344331 | 1111957 | 318703 | 1138453 | 370967 |
| RP_POS_248_03445a3_97 | RP | POS | 3.97 | 248.0345 | | | | 3.12E-01 | 0.16 | 1.00 | 7.03E-01 | -0.08 | 0.36 | 5.83E-01 | 0.12 | 0.74 | 2.32E-01 | 0.20 | 0.51 | 3924188 | 847978 | 4136801 | 1455825 | 4192769 | 1165580 | 4080834 | 1707964 |
| RP_NEG_765_16833a9_503 | RP | NEG | 9.50 | 765.1683 | | | | 9.91E-01 | 0.00 | 0.51 | 7.03E-01 | -0.08 | 0.37 | 8.57E-01 | -0.04 | 0.28 | 8.35E-01 | 0.04 | 0.36 | 8930190 | 479947 | 8931357 | 620083 | 8955166 | 596359 | 8907549 | 648114 |
| RP_POS_285_06393a0_446 | RP | POS | 0.45 | 285.0639 | | | | 1.09E-07 | -0.67 | 1.35 | 7.04E-01 | 0.08 | 1.18 | 2.39E-04 | -0.62 | 1.40 | 6.26E-05 | -0.71 | 1.46 | 853694 | 92182 | 612705 | 395001 | 597563 | 402676 | 627847 | 390665 |
| HILIC_NEG_226_98643a0_831 | HILIC | NEG | 0.831 | 226.9864 | | | | 2.42E-01 | 0.20 | 0.84 | 7.04E-01 | -0.08 | 0.93 | 4.80E-01 | 0.16 | 0.41 | 1.76E-01 | 0.24 | 0.56 | 384568 | 120642 | 417205 | 176353 | 423964 | 137321 | 410445 | 209488 |
| RP_POS_643_29187a11_426 | RP | POS | 11.43 | 643.2919 | | | | 9.27E-01 | -0.02 | 0.18 | 7.04E-01 | 0.08 | 0.51 | 9.29E-01 | 0.02 | 0.33 | 7.99E-01 | -0.06 | 0.33 | 885335 | 145913 | 882581 | 150596 | 876813 | 148627 | 888349 | 153829 |
| HILIC_POS_96_0449a0_795 | HILIC | POS | 0.795 | 96.0449 | | | | 2.46E-01 | 0.17 | 0.57 | 7.04E-01 | 0.09 | 0.73 | 3.58E-01 | 0.21 | 0.46 | 3.21E-01 | 0.12 | 0.42 | 2784613 | 2537531 | 3628432 | 5683598 | 3410599 | 3090451 | 3846265 | 7457754 |
| HILIC_POS_146_98026a7_399 | HILIC | POS | 7.399 | 146.9803 | | | | 6.52E-01 | -0.09 | 0.28 | 7.04E-01 | -0.08 | 0.81 | 5.63E-01 | -0.13 | 0.23 | 8.13E-01 | -0.06 | 0.13 | 1478191 | 213977 | 1458586 | 209327 | 1466591 | 217200 | 1450581 | 203034 |
| RP_POS_245_18599a3_965 | RP | POS | 3.97 | 245.1860 | | | | 5.26E-05 | 0.48 | 1.02 | 7.04E-01 | 0.09 | 1.09 | 1.42E-03 | 0.52 | 1.26 | 1.21E-02 | 0.43 | 1.00 | 89553 | 29309 | 1164103 | 2541040 | 1066992 | 2650943 | 1261213 | 2449243 |
| RP_POS_341_35315a11_248 | RP | POS | 11.25 | 341.3532 | | | | 1.89E-13 | 1.05 | 1.59 | 7.05E-01 | 0.08 | 0.66 | 5.68E-08 | 1.08 | 1.67 | 6.72E-10 | 1.01 | 1.79 | 204842 | 109941 | 469772 | 253063 | 460113 | 215977 | 479432 | 287319 |
| HILIC_POS_197_08411a0_734 | HILIC | POS | 0.734 | 197.0841 | | | | 8.51E-03 | 0.33 | 1.08 | 7.05E-01 | 0.09 | 0.87 | 6.87E-02 | 0.37 | 0.64 | 2.61E-02 | 0.29 | 0.95 | 640621 | 672475 | 1415549 | 2644010 | 1314590 | 1917819 | 1516508 | 3228842 |
| HILIC_NEG_132_90034a1_409 | HILIC | NEG | 1.409 | 132.9003 | | | | 7.57E-01 | 0.06 | 0.64 | 7.05E-01 | -0.08 | 0.91 | 9.17E-01 | 0.03 | 0.10 | 6.19E-01 | 0.10 | 0.39 | 730873 | 97712 | 737023 | 95476 | 740663 | 65065 | 733383 | 118982 |
| RP_POS_176_97488a15_38 | RP | POS | 15.38 | 176.9749 | | | | 7.10E-01 | 0.08 | 0.56 | 7.05E-01 | -0.07 | 0.33 | 8.52E-01 | 0.04 | 0.26 | 6.24E-01 | 0.12 | 0.53 | 33177518 | 4462664 | 33506935 | 3924705 | 33656423 | 4004237 | 33357447 | 3878333 |
| RP_POS_132_10551a1_368 | RP | POS | 1.37 | 132.1055 | | | | 2.39E-01 | 0.19 | 0.64 | 7.06E-01 | -0.08 | 0.45 | 4.45E-01 | 0.14 | 0.49 | 2.48E-01 | 0.23 | 0.48 | 25958858 | 6404977 | 27826394 | 10978381 | 28244217 | 11342518 | 27408570 | 10700487 |
| RP_POS_284_29446a11_166 | RP | POS | 11.17 | 284.2945 | | | | 1.09E-10 | 0.93 | 1.56 | 7.06E-01 | -0.08 | 0.85 | 7.08E-06 | 0.89 | 1.51 | 6.80E-09 | 0.97 | 1.77 | 75308554 | 24393651 | 124564001 | 54379187 | 126633194 | 46672939 | 122494809 | 61542179 |
| RP_NEG_248_96021a14_262 | RP | NEG | 14.26 | 248.9602 | | | | 2.60E-01 | -0.21 | 0.45 | 7.06E-01 | 0.08 | 0.30 | 4.32E-01 | -0.17 | 0.47 | 2.31E-01 | -0.25 | 0.73 | 6432277 | 2531324 | 5822836 | 2957064 | 5710386 | 2798712 | 5935287 | 3131823 |
| RP_POS_302_21472a8_219 | RP | POS | 8.22 | 302.2147 | | | | 2.80E-02 | 0.43 | 1.11 | 7.06E-01 | -0.08 | 1.21 | 6.55E-02 | 0.39 | 0.96 | 3.99E-02 | 0.47 | 0.96 | 6418905 | 3131622 | 7894522 | 3495197 | 8027199 | 3774108 | 7761845 | 3225259 |
| RP_POS_365_24689a11_14 | RP | POS | 11.14 | 365.2469 | | | | 8.12E-02 | -0.26 | 0.95 | 7.06E-01 | 0.08 | 1.06 | 2.38E-01 | -0.22 | 0.86 | 1.07E-01 | -0.30 | 1.00 | 1223459 | 83201 | 1184905 | 162233 | 1178747 | 163725 | 1191063 | 162152 |
| HILIC_POS_176_01083a4_25 | HILIC | POS | 4.25 | 176.0108 | | | | 6.15E-01 | -0.10 | 0.37 | 7.06E-01 | -0.08 | 0.98 | 5.40E-01 | -0.14 | 0.34 | 7.78E-01 | -0.06 | 0.23 | 999948 | 1079338 | 887947 | 1118372 | 930394 | 1096202 | 845500 | 1149653 |
| RP_POS_572_83289a0_508 | RP | POS | 0.51 | 572.8329 | | | | 1.28E-06 | -0.66 | 1.28 | 7.06E-01 | -0.08 | 1.16 | 4.72E-05 | -0.70 | 1.42 | 8.02E-04 | -0.62 | 1.33 | 683372 | 94672 | 531342 | 247560 | 540736 | 261640 | 521948 | 234919 |
| HILIC_NEG_290_84866a2_926 | HILIC | NEG | 2.926 | 290.8487 | | | | 2.35E-06 | -0.59 | 1.33 | 7.06E-01 | 0.08 | 0.78 | 8.12E-04 | -0.54 | 1.45 | 5.33E-04 | -0.63 | 1.51 | 298141 | 25514 | 235567 | 117928 | 231093 | 124862 | 240042 | 111653 |
| RP_NEG_372_90158a0_456 | RP | NEG | 0.46 | 372.9016 | | | | 1.62E-06 | -0.63 | 1.29 | 7.07E-01 | 0.08 | 1.14 | 6.42E-04 | -0.59 | 1.33 | 1.92E-04 | -0.67 | 1.39 | 1121752 | 176354 | 804274 | 549278 | 783443 | 562043 | 825106 | 541101 |
| RP_POS_323_19537a10_73 | RP | POS | 10.73 | 323.1954 | | | | 1.46E-03 | 0.52 | 1.44 | 7.07E-01 | -0.08 | 1.15 | 1.09E-02 | 0.48 | 1.28 | 5.49E-03 | 0.56 | 1.40 | 1691645 | 733750 | 2264253 | 1158730 | 2308183 | 1221234 | 2220323 | 1103340 |
| RP_POS_343_17175a6_399 | RP | POS | 6.40 | 343.1718 | Magnoflorine | 343.1749(100) | 3 | 3.03E-01 | -0.19 | 0.40 | 7.07E-01 | 0.08 | 0.38 | 4.98E-01 | -0.15 | 0.33 | 2.65E-01 | -0.23 | 0.57 | 1197745 | 64242 | 1183201 | 82069 | 1180090 | 76933 | 1186311 | 87576 |
| RP_NEG_257_17599a8_906 | RP | NEG | 8.91 | 257.1760 | Unknown | 257.1742(100), 239.1642(60), 195.1735(40) | 4 | 1.45E-01 | 0.29 | 0.78 | 7.07E-01 | 0.08 | 0.42 | 1.66E-01 | 0.33 | 0.69 | 2.27E-01 | 0.25 | 0.50 | 2645225 | 517570 | 2802939 | 547688 | 2782190 | 459743 | 2823688 | 627538 |
| RP_POS_712_8197a0_472 | RP | POS | 0.47 | 712.8197 | | | | 1.32E-07 | -0.65 | 1.34 | 7.07E-01 | 0.08 | 1.17 | 2.84E-04 | -0.61 | 1.39 | 8.43E-05 | -0.69 | 1.46 | 604151 | 52639 | 446550 | 264948 | 436517 | 271110 | 456582 | 261000 |
| RP_POS_316_12134a12_117 | RP | POS | 12.12 | 316.1213 | | | | 2.96E-01 | 0.20 | 0.49 | 7.07E-01 | 0.08 | 0.34 | 2.94E-01 | 0.24 | 0.57 | 4.41E-01 | 0.16 | 0.45 | 398362 | 133932 | 428330 | 156942 | 422388 | 141204 | 434271 | 172492 |
| HILIC_NEG_199_80498a2_952 | HILIC | NEG | 2.952 | 199.8050 | | | | 8.00E-05 | 0.52 | 1.22 | 7.07E-01 | -0.08 | 0.79 | 4.47E-03 | 0.48 | 1.19 | 2.32E-03 | 0.56 | 1.36 | 9496132 | 1849069 | 12186924 | 5739676 | 12404153 | 6045606 | 11969694 | 5469408 |
| RP_NEG_215_86908a15_571 | RP | NEG | 15.57 | 215.8691 | | | | 2.42E-01 | 0.17 | 0.37 | 7.07E-01 | -0.08 | 0.42 | 4.95E-01 | 0.12 | 0.64 | 2.49E-01 | 0.21 | 0.42 | 1229039 | 69735 | 1252396 | 155726 | 1258289 | 155275 | 1246503 | 157528 |
| RP_POS_230_96283a15_587 | RP | POS | 15.59 | 230.9628 | | | | 5.69E-01 | -0.09 | 0.36 | 7.07E-01 | -0.08 | 0.34 | 5.71E-01 | -0.13 | 0.32 | 7.53E-01 | -0.04 | 0.56 | 8107911 | 186596 | 8080294 | 353285 | 8093676 | 217028 | 8066911 | 452439 |
| RP_NEG_190_9277a15_488 | RP | NEG | 15.49 | 190.9277 | | | | 8.31E-01 | 0.04 | 0.37 | 7.08E-01 | -0.08 | 0.39 | 9.80E-01 | 0.01 | 0.24 | 7.31E-01 | 0.08 | 0.31 | 36526720 | 699642 | 36557184 | 705812 | 36583854 | 775657 | 36530513 | 635148 |
| RP_POS_296_25839a9_398 | RP | POS | 9.40 | 296.2584 | | | | 7.15E-01 | -0.11 | 0.75 | 7.08E-01 | -0.05 | 0.28 | 6.60E-01 | -0.13 | 0.56 | 7.88E-01 | -0.08 | 0.46 | 2515724 | 4761683 | 2196018 | 2118233 | 2275958 | 1925654 | 2116077 | 2311754 |
| HILIC_POS_122_92489a2_992 | HILIC | POS | 2.992 | 122.9249 | | | | 1.54E-05 | -0.59 | 1.35 | 7.08E-01 | 0.08 | 0.80 | 1.18E-03 | -0.55 | 1.44 | 7.99E-04 | -0.63 | 1.42 | 154992135 | 22941962 | 122285874 | 60377874 | 120008170 | 64279332 | 124563578 | 56771057 |

**Supplementary table 1: Metabolomics results** (in asceding order according to p-value from progressive ALD vs  non-progressive ALD comparison, with molecular features with ID_level of 1-2 first)

| | | | | | | | | all_ALD_vs_control | | | Prog_ALD_vs_non-prog_ALD | | | Prog_ALD_vs_control_ALD | | | Non-prog_ALD_vs_control | | | Healthy_control | | all_ALD | | Non-prog_ALD | | Progressive_ALD | |
|---|---|---|---|---|---|---|---|---|---|---|---|---|---|---|---|---|---|---|---|---|---|---|---|---|---|---|---|
| | | | | | | | | t-test | Cohen's | PLS-DA | t-test | Cohen's | PLS-DA | t-test | Cohen's | PLS-DA | t-test | Cohen's | PLS-DA | | | | | | | | |
| Feature_ID | RP_HILIC | POS_NEG | Average.Rt | Average.Mz | Metabolite.name | curated_MSMS | ID_level | p | d | VIP | p | d | VIP | p | d | VIP | p | d | VIP | Mean | SD | Mean | SD | Mean | SD | Mean | SD |
| HILIC_POS_371_2402a4_626 | HILIC | POS | 4.626 | 371.2402 | | | | 6.00E-01 | 0.10 | 0.83 | 7.08E-01 | 0.08 | 0.70 | 5.52E-01 | 0.13 | 0.21 | 7.74E-01 | 0.06 | 0.42 | 6183672 | 1323518 | 6335102 | 1673443 | 6271971 | 1393406 | 6398232 | 1925685 |
| RP_POS_1016_79303a0_493 | RP | POS | 0.49 | 1016.7930 | | | | 1.52E-08 | 0.68 | 1.38 | 7.08E-01 | -0.08 | 1.19 | 1.48E-04 | 0.64 | 1.45 | 3.29E-05 | 0.72 | 1.49 | 41313 | 42918 | 669270 | 1016476 | 707593 | 1029519 | 630947 | 1012239 |
| RP_NEG_128_0343a0_71 | RP | NEG | 0.71 | 128.0343 | | | | 1.53E-07 | 0.72 | 1.12 | 7.08E-01 | 0.08 | 0.70 | 1.61E-04 | 0.76 | 1.23 | 9.16E-06 | 0.68 | 1.36 | 3435480 | 544191 | 4371521 | 1379677 | 4319494 | 1123595 | 4423548 | 1605576 |
| RP_NEG_289_89835a3_965 | RP | NEG | 3.97 | 289.8984 | | | | 3.78E-01 | 0.15 | 0.87 | 7.08E-01 | -0.08 | 0.32 | 6.23E-01 | 0.11 | 0.62 | 3.02E-01 | 0.19 | 0.50 | 1972341 | 669166 | 2110950 | 1024152 | 2149531 | 869557 | 2072369 | 1166197 |
| RP_POS_175_06256a5_353 | RP | POS | 5.35 | 175.0626 | | | | 4.81E-01 | -0.14 | 0.42 | 7.09E-01 | 0.08 | 0.44 | 6.54E-01 | -0.11 | 0.46 | 4.03E-01 | -0.18 | 0.60 | 1023940 | 331579 | 976148 | 330523 | 963702 | 290591 | 988594 | 368730 |
| HILIC_POS_142_08643a1_318 | HILIC | POS | 1.318 | 142.0864 | | | | 4.97E-03 | 0.41 | 0.73 | 7.09E-01 | 0.08 | 0.43 | 9.44E-03 | 0.45 | 0.93 | 5.65E-02 | 0.37 | 0.69 | 10299266 | 9343694 | 17593764 | 19350304 | 16865743 | 20921250 | 18321786 | 17824974 |
| RP_POS_530_32245a10_584 | RP | POS | 10.58 | 530.3225 | | | | 5.45E-03 | 0.48 | 1.08 | 7.09E-01 | -0.08 | 0.75 | 4.08E-02 | 0.44 | 0.89 | 5.55E-03 | 0.52 | 1.08 | 408224 | 168790 | 517039 | 235006 | 525881 | 200774 | 508197 | 266670 |
| HILIC_POS_294_84357a2_992 | HILIC | POS | 2.992 | 294.8436 | | | | 7.60E-06 | -0.57 | 1.35 | 7.09E-01 | 0.08 | 0.78 | 1.34E-03 | -0.53 | 1.47 | 7.65E-04 | -0.61 | 1.51 | 2033095 | 235409 | 1589506 | 854714 | 1557355 | 900844 | 1621657 | 813850 |
| RP_POS_643_52643a11_71 | RP | POS | 11.71 | 643.5264 | | | | 2.01E-01 | -0.25 | 0.64 | 7.09E-01 | -0.08 | 0.85 | 1.69E-01 | -0.29 | 0.79 | 3.60E-01 | -0.21 | 0.45 | 2398575 | 2659781 | 1681898 | 2929570 | 1792109 | 3264992 | 1571686 | 2579720 |
| RP_POS_302_24557a11_161 | RP | POS | 11.16 | 302.2456 | | | | 5.90E-05 | 0.72 | 1.15 | 7.09E-01 | 0.08 | 0.59 | 5.94E-04 | 0.76 | 1.17 | 3.08E-04 | 0.68 | 1.31 | 2372502 | 1099130 | 3411608 | 1454075 | 3356912 | 1215619 | 3466304 | 1669725 |
| HILIC_POS_114_12802a0_868 | HILIC | POS | 0.868 | 114.1280 | | | | 2.05E-03 | -0.52 | 1.13 | 7.09E-01 | -0.08 | 0.67 | 4.21E-03 | -0.56 | 1.20 | 1.86E-02 | -0.48 | 1.05 | 504533 | 151912 | 392014 | 229129 | 400629 | 239789 | 383399 | 220049 |
| RP_POS_346_00757a0_63 | RP | POS | 0.63 | 346.0076 | | | | 5.18E-08 | -0.66 | 1.36 | 7.09E-01 | 0.08 | 1.18 | 2.02E-04 | -0.62 | 1.42 | 5.96E-05 | -0.71 | 1.47 | 30759798 | 2173156 | 22152702 | 14228035 | 21618114 | 14548436 | 22687291 | 14027299 |
| RP_POS_615_43115a15_336 | RP | POS | 15.34 | 615.4312 | | | | 9.14E-01 | 0.02 | 0.85 | 7.09E-01 | 0.08 | 0.39 | 7.89E-01 | 0.06 | 0.59 | 9.41E-01 | -0.02 | 0.64 | 7733531 | 2043600 | 7779277 | 2164485 | 7697961 | 2200590 | 7860592 | 2146976 |
| RP_NEG_650_46106a11_611 | RP | NEG | 11.61 | 650.4611 | | | | 7.01E-02 | -0.36 | 0.82 | 7.09E-01 | 0.08 | 0.56 | 1.27E-01 | -0.32 | 0.70 | 8.88E-02 | -0.40 | 0.70 | 5151818 | 831421 | 4834640 | 888385 | 4801277 | 992834 | 4868002 | 778825 |
| RP_NEG_339_20068a11_613 | RP | NEG | 11.61 | 339.2007 | | | | 3.05E-01 | 0.18 | 0.61 | 7.10E-01 | 0.08 | 0.32 | 2.51E-01 | 0.22 | 0.58 | 5.16E-01 | 0.14 | 0.47 | 1842222 | 743273 | 2011478 | 977588 | 1974794 | 1097780 | 2048162 | 850290 |
| RP_POS_441_297a13_631 | RP | POS | 13.63 | 441.2970 | | | | 6.20E-01 | -0.10 | 0.26 | 7.10E-01 | -0.08 | 0.36 | 5.34E-01 | -0.14 | 0.29 | 7.85E-01 | -0.06 | 0.16 | 20204422 | 6076553 | 19586221 | 6161204 | 19817186 | 6491127 | 19355256 | 5869581 |
| HILIC_POS_468_30814a1_267 | HILIC | POS | 1.267 | 468.3081 | | | | 1.02E-04 | 0.59 | 0.90 | 7.10E-01 | -0.08 | 0.30 | 4.93E-03 | 0.55 | 0.92 | 4.99E-04 | 0.63 | 1.18 | 1543343 | 520557 | 2094292 | 1004717 | 2131892 | 943077 | 2056693 | 1071090 |
| RP_POS_239_1261a7_475 | RP | POS | 7.48 | 239.1261 | | | | 8.77E-01 | -0.04 | 0.61 | 7.10E-01 | 0.07 | 0.48 | 9.99E-01 | 0.00 | 0.30 | 7.77E-01 | -0.07 | 0.60 | 1784200 | 349411 | 1773592 | 285178 | 1762924 | 300158 | 1784259 | 272003 |
| RP_POS_590_48065a12_463 | RP | POS | 12.46 | 590.4807 | | | | 4.37E-01 | 0.13 | 0.62 | 7.10E-01 | 0.08 | 0.30 | 4.29E-01 | 0.17 | 0.39 | 6.25E-01 | 0.09 | 0.35 | 2117548 | 349381 | 2181391 | 533913 | 2161424 | 458242 | 2201358 | 604271 |
| RP_POS_514_63751a6_842 | RP | POS | 6.84 | 514.6375 | | | | 2.83E-07 | 0.61 | 1.32 | 7.11E-01 | -0.08 | 1.13 | 4.58E-04 | 0.57 | 1.41 | 2.08E-04 | 0.65 | 1.40 | 28701 | 73273 | 935920 | 1642392 | 997329 | 1706907 | 874511 | 1590196 |
| HILIC_POS_312_20135a0_552 | HILIC | POS | 0.552 | 312.2014 | | | | 4.93E-01 | 0.11 | 0.31 | 7.11E-01 | 0.08 | 0.63 | 4.84E-01 | 0.15 | 0.25 | 6.91E-01 | 0.07 | 0.35 | 354950 | 46862 | 362848 | 79408 | 359879 | 64941 | 365816 | 92220 |
| RP_POS_444_76266a13_708 | RP | POS | 13.71 | 444.7627 | | | | 3.22E-04 | -0.76 | 1.46 | 7.11E-01 | -0.07 | 0.51 | 7.15E-04 | -0.79 | 1.37 | 1.16E-03 | -0.72 | 1.40 | 1829214 | 864937 | 1157522 | 830119 | 1188537 | 771948 | 1126507 | 891287 |
| RP_POS_480_83252a0_499 | RP | POS | 0.50 | 480.8325 | | | | 1.03E-03 | -0.45 | 1.20 | 7.11E-01 | -0.08 | 1.24 | 4.17E-03 | -0.49 | 1.27 | 2.86E-02 | -0.41 | 1.23 | 2181893 | 396269 | 1762870 | 1031907 | 1801418 | 1095526 | 1724323 | 973749 |
| RP_POS_639_62732a0_208 | RP | POS | 0.21 | 639.6273 | | | | 4.47E-01 | 0.15 | 0.52 | 7.11E-01 | -0.08 | 0.38 | 6.07E-01 | 0.12 | 0.50 | 3.99E-01 | 0.19 | 0.53 | 2775140 | 1142379 | 2954241 | 1175972 | 2998156 | 1188721 | 2910326 | 1173478 |
| RP_POS_300_91559a0_464 | RP | POS | 0.46 | 300.9156 | | | | 2.15E-06 | -0.63 | 1.29 | 7.11E-01 | 0.08 | 1.14 | 6.28E-04 | -0.59 | 1.33 | 2.13E-04 | -0.67 | 1.39 | 322194 | 54082 | 231046 | 156814 | 225196 | 161166 | 236897 | 153751 |
| RP_NEG_437_0553a7_855 | RP | NEG | 7.86 | 437.0553 | | | | 5.14E-01 | -0.14 | 0.60 | 7.11E-01 | -0.07 | 0.82 | 3.83E-01 | -0.18 | 0.49 | 6.93E-01 | -0.11 | 0.50 | 3154236 | 6738239 | 2279781 | 5935410 | 2501534 | 8051242 | 2058028 | 2500861 |
| HILIC_NEG_114_89414a2_951 | HILIC | NEG | 2.951 | 114.8941 | | | | 2.56E-05 | -0.54 | 1.31 | 7.11E-01 | 0.08 | 0.78 | 1.68E-03 | -0.50 | 1.42 | 2.08E-03 | -0.58 | 1.48 | 316720 | 33438 | 258378 | 119778 | 253913 | 131301 | 262843 | 108193 |
| RP_POS_999_63898a10_516 | RP | POS | 10.52 | 999.6390 | | | | 3.83E-05 | 0.63 | 1.30 | 7.11E-01 | -0.08 | 0.28 | 1.05E-03 | 0.59 | 1.26 | 6.77E-04 | 0.67 | 1.25 | 232332 | 158466 | 408363 | 297335 | 419441 | 316485 | 397285 | 279655 |
| RP_POS_274_87259a0_499 | RP | POS | 0.50 | 274.8726 | | | | 1.45E-03 | -0.42 | 1.18 | 7.12E-01 | -0.08 | 1.23 | 5.12E-03 | -0.46 | 1.24 | 4.00E-02 | -0.38 | 1.23 | 15572909 | 2875319 | 12391066 | 8352728 | 12702205 | 8990675 | 12079926 | 7741456 |
| RP_POS_716_54761a10_902 | RP | POS | 10.90 | 716.5476 | | | | 3.27E-01 | -0.22 | 0.52 | 7.12E-01 | -0.07 | 0.40 | 2.96E-01 | -0.26 | 0.42 | 4.43E-01 | -0.19 | 0.48 | 1461624 | 396682 | 1385143 | 321493 | 1397105 | 320745 | 1373180 | 325047 |
| HILIC_POS_171_18558a5_177 | HILIC | POS | 5.177 | 171.1856 | | | | 7.48E-01 | -0.06 | 0.59 | 7.12E-01 | 0.08 | 0.55 | 9.31E-01 | -0.02 | 0.41 | 6.01E-01 | -0.10 | 0.60 | 132639745 | 23478208 | 131014009 | 28637539 | 129948014 | 21196304 | 132080004 | 34718178 |
| RP_POS_254_12431a0_705 | RP | POS | 0.71 | 254.1243 | | | | 2.98E-13 | 0.90 | 1.72 | 7.12E-01 | -0.08 | 0.61 | 1.32E-06 | 0.86 | 1.70 | 5.76E-08 | 0.93 | 1.81 | 78026 | 53982 | 842434 | 905594 | 876110 | 881757 | 808757 | 936550 |
| HILIC_POS_508_37698a1_303 | HILIC | POS | 1.303 | 508.3770 | | | | 1.53E-01 | -0.31 | 0.74 | 7.12E-01 | -0.07 | 0.87 | 1.49E-01 | -0.35 | 0.56 | 2.42E-01 | -0.28 | 0.60 | 3047323 | 980854 | 2765704 | 869408 | 2797990 | 851027 | 2733417 | 894870 |
| RP_POS_357_27856a8_865 | RP | POS | 8.87 | 357.2786 | | | | 6.00E-05 | 0.53 | 1.70 | 7.13E-01 | -0.08 | 0.43 | 6.49E-04 | 0.49 | 1.61 | 4.89E-03 | 0.57 | 1.57 | 243112 | 245965 | 593115 | 722799 | 619956 | 858917 | 566273 | 562432 |
| RP_POS_419_34915a11_62 | RP | POS | 11.62 | 419.3492 | | | | 5.42E-02 | -0.38 | 1.04 | 7.13E-01 | -0.07 | 0.72 | 3.78E-02 | -0.42 | 0.95 | 1.56E-01 | -0.34 | 0.91 | 3309035 | 914110 | 2935906 | 995697 | 2972852 | 1202725 | 2898959 | 744128 |
| RP_POS_794_56805a12_888 | RP | POS | 12.89 | 794.5681 | | | | 1.78E-01 | -0.25 | 0.86 | 7.13E-01 | -0.08 | 0.55 | 1.83E-01 | -0.29 | 0.65 | 3.15E-01 | -0.21 | 0.50 | 3543853 | 1345500 | 3149211 | 1656083 | 3210617 | 1611978 | 3087805 | 1713192 |
| RP_NEG_225_06955a7_846 | RP | NEG | 7.85 | 225.0696 | | | | 7.96E-01 | -0.04 | 0.43 | 7.13E-01 | -0.08 | 0.28 | 7.04E-01 | -0.08 | 0.36 | 9.84E-01 | 0.00 | 0.15 | 2211908 | 2564337 | 2058513 | 3795176 | 2199221 | 3105863 | 1917804 | 4406118 |
| RP_NEG_738_80194a0_498 | RP | NEG | 0.50 | 738.8019 | | | | 3.07E-03 | -0.43 | 1.15 | 7.13E-01 | -0.08 | 1.23 | 7.60E-03 | -0.47 | 1.19 | 4.13E-02 | -0.39 | 1.19 | 867318 | 203694 | 697227 | 431556 | 713219 | 458590 | 681235 | 406762 |
| RP_POS_489_31802a10_455 | RP | POS | 10.46 | 489.3180 | | | | 4.87E-01 | 0.14 | 0.69 | 7.13E-01 | 0.07 | 0.24 | 4.42E-01 | 0.18 | 0.85 | 6.38E-01 | 0.10 | 0.34 | 2269148 | 752942 | 2376469 | 760240 | 2348314 | 717127 | 2404623 | 807363 |
| HILIC_POS_169_0457a7_374 | HILIC | POS | 7.374 | 169.0457 | | | | 7.97E-05 | -0.58 | 1.20 | 7.13E-01 | -0.08 | 0.85 | 7.25E-04 | -0.62 | 1.23 | 3.79E-03 | -0.54 | 1.29 | 1852308 | 185747 | 1644978 | 384689 | 1659221 | 393430 | 1630735 | 379197 |
| RP_NEG_638_27618a10_622 | RP | NEG | 10.62 | 638.2762 | | | | 3.13E-01 | 0.21 | 1.21 | 7.13E-01 | -0.07 | 0.81 | 4.59E-01 | 0.17 | 0.93 | 2.87E-01 | 0.24 | 0.97 | 2585591 | 410339 | 2670733 | 416814 | 2686164 | 418939 | 2655303 | 418351 |
| RP_NEG_180_97238a0_497 | RP | NEG | 0.50 | 180.9724 | | | | 3.30E-03 | 0.41 | 1.15 | 7.13E-01 | -0.08 | 1.09 | 3.33E-02 | 0.37 | 1.13 | 1.66E-02 | 0.45 | 1.24 | 17253729 | 580331 | 17781826 | 1432736 | 17834825 | 1507637 | 17728827 | 1366988 |
| HILIC_POS_396_31082a0_623 | HILIC | POS | 0.623 | 396.3108 | | | | 1.09E-01 | 0.28 | 0.83 | 7.14E-01 | -0.08 | 0.92 | 1.88E-01 | 0.24 | 0.65 | 1.56E-01 | 0.32 | 0.49 | 340941 | 180455 | 406543 | 248484 | 415735 | 292389 | 397350 | 197700 |
| RP_NEG_530_30237a10_508 | RP | NEG | 10.51 | 530.3024 | | | | 1.19E-01 | -0.32 | 0.62 | 7.14E-01 | -0.07 | 0.80 | 1.16E-01 | -0.36 | 0.70 | 2.12E-01 | -0.29 | 0.71 | 5559800 | 1532196 | 5068539 | 1500434 | 5123975 | 1519674 | 5013104 | 1494278 |
| RP_NEG_352_85303a15_533 | RP | NEG | 15.53 | 352.8530 | | | | 5.97E-01 | 0.12 | 0.45 | 7.14E-01 | -0.07 | 0.48 | 7.25E-01 | 0.09 | 0.53 | 5.26E-01 | 0.16 | 0.31 | 2327598 | 257374 | 2353994 | 197084 | 2361272 | 189308 | 2346716 | 206230 |
| RP_NEG_144_92288a15_618 | RP | NEG | 15.62 | 144.9229 | | | | 4.34E-02 | -0.41 | 0.88 | 7.14E-01 | 0.07 | 0.34 | 1.01E-01 | -0.38 | 0.69 | 4.49E-02 | -0.45 | 0.99 | 3054208 | 107582 | 3008836 | 109025 | 3004815 | 105298 | 3012857 | 113556 |
| RP_POS_771_64496a11_039 | RP | POS | 11.04 | 771.6450 | | | | 1.21E-01 | 0.25 | 0.66 | 7.15E-01 | -0.08 | 0.24 | 1.94E-01 | 0.21 | 0.79 | 1.91E-01 | 0.29 | 0.58 | 422405 | 795022 | 718086 | 1256849 | 764408 | 1538297 | 671764 | 906003 |
| RP_NEG_215_16521a9_161 | RP | NEG | 9.16 | 215.1652 | | | | 1.23E-03 | 0.51 | 1.49 | 7.15E-01 | 0.08 | 1.33 | 1.71E-03 | 0.55 | 1.47 | 2.30E-02 | 0.47 | 1.20 | 1599735 | 991340 | 2414875 | 1702457 | 2352302 | 1931162 | 2477448 | 1455503 |
| RP_POS_232_1543a2_226 | RP | POS | 2.23 | 232.1543 | | | | 3.46E-03 | 0.37 | 0.66 | 7.16E-01 | -0.08 | 0.78 | 8.14E-02 | 0.33 | 0.67 | 6.47E-03 | 0.42 | 0.82 | 1553546 | 580897 | 2226626 | 2013095 | 2300539 | 1730751 | 2152712 | 2276216 |
| RP_POS_302_936a15_367 | RP | POS | 15.37 | 302.9360 | | | | 6.19E-02 | -0.24 | 0.49 | 7.17E-01 | -0.08 | 0.25 | 1.09E-01 | -0.28 | 0.63 | 2.45E-01 | -0.20 | 0.52 | 6797762 | 232510 | 6630048 | 790029 | 6658926 | 783993 | 6601171 | 802922 |
| RP_POS_151_144a0_536 | RP | POS | 0.54 | 151.1440 | | | | 6.31E-01 | 0.08 | 0.66 | 7.17E-01 | 0.08 | 0.60 | 5.81E-01 | 0.12 | 0.67 | 8.33E-01 | 0.04 | 0.40 | 2316801 | 2110769 | 2563086 | 3489290 | 2435544 | 2973359 | 2690628 | 3965438 |
| HILIC_NEG_134_89476a0_936 | HILIC | NEG | 0.936 | 134.8948 | | | | 6.55E-03 | 0.47 | 1.29 | 7.17E-01 | -0.08 | 0.64 | 2.43E-02 | 0.43 | 0.92 | 1.68E-02 | 0.51 | 1.32 | 31862186 | 5886746 | 35582384 | 8267754 | 35884368 | 9020002 | 35280400 | 7520636 |
| RP_NEG_368_68005a15_577 | RP | NEG | 15.58 | 368.6801 | | | | 4.14E-02 | -0.40 | 0.87 | 7.17E-01 | -0.07 | 0.24 | 4.66E-02 | -0.44 | 0.85 | 1.02E-01 | -0.36 | 0.76 | 1954541 | 90272 | 1915276 | 99649 | 1918914 | 101664 | 1911638 | 98488 |
| RP_POS_335_15131a10_731 | RP | POS | 10.73 | 335.1513 | | | | 3.22E-03 | 0.48 | 1.37 | 7.18E-01 | -0.08 | 1.10 | 1.87E-02 | 0.44 | 1.19 | 1.10E-02 | 0.52 | 1.34 | 5654861 | 2536158 | 7499032 | 4114220 | 7648864 | 4391820 | 7349200 | 3855536 |
| HILIC_NEG_160_97612a7_352 | HILIC | NEG | 7.352 | 160.9761 | | | | 3.04E-04 | -0.61 | 1.17 | 7.18E-01 | 0.08 | 0.73 | 2.62E-03 | -0.57 | 1.18 | 1.74E-03 | -0.65 | 1.23 | 2736016 | 167306 | 2590143 | 247550 | 2581132 | 265456 | 2599154 | 230609 |
| HILIC_POS_144_98228a7_4 | HILIC | POS | 7.4 | 144.9823 | | | | 1.67E-01 | -0.30 | 0.59 | 7.19E-01 | -0.07 | 0.39 | 1.75E-01 | -0.33 | 0.52 | 2.50E-01 | -0.26 | 0.48 | 3068514 | 393233 | 2958528 | 363636 | 2971733 | 324580 | 2945323 | 401813 |
| RP_POS_804_55054a13_288 | RP | POS | 13.29 | 804.5505 | | | | 6.58E-01 | 0.11 | 0.71 | 7.19E-01 | -0.06 | 0.23 | 7.79E-01 | 0.08 | 0.52 | 5.72E-01 | 0.14 | 0.75 | 5459658 | 4021259 | 5799876 | 2779403 | 5900742 | 2178598 | 5699011 | 3292531 |
| RP_POS_233_11487a7_239 | RP | POS | 7.24 | 233.1149 | | | | 3.79E-01 | 0.17 | 0.38 | 7.19E-01 | 0.07 | 0.55 | 3.59E-01 | 0.21 | 0.37 | 5.35E-01 | 0.13 | 0.49 | 486192 | 493147 | 578289 | 565066 | 557811 | 528166 | 598768 | 604401 |
| RP_POS_375_20288a2_891 | RP | POS | 2.89 | 375.2029 | | | | 2.68E-06 | 0.90 | 1.47 | 7.19E-01 | 0.07 | 0.94 | 1.82E-06 | 0.93 | 1.76 | 1.69E-04 | 0.86 | 1.26 | 18572579 | 5379793 | 24484255 | 6311351 | 24255509 | 7635068 | 24713000 | 4698741 |
| RP_NEG_301_24066a11_16 | RP | NEG | 11.16 | 301.2407 | | | | 1.20E-01 | 0.27 | 1.05 | 7.19E-01 | -0.08 | 1.00 | 2.53E-01 | 0.23 | 0.91 | 1.36E-01 | 0.31 | 1.06 | 757769 | 181504 | 822535 | 257159 | 831848 | 263035 | 813222 | 253469 |
| RP_NEG_201_80171a15_584 | RP | NEG | 15.58 | 201.8017 | | | | 1.75E-01 | -0.25 | 0.54 | 7.19E-01 | -0.08 | 0.29 | 1.55E-01 | -0.29 | 0.56 | 3.32E-01 | -0.22 | 0.37 | 3537935 | 114837 | 3504208 | 138130 | 3509209 | 150904 | 3499207 | 125405 |
| RP_NEG_191_01924a0_708 | RP | NEG | 0.71 | 191.0192 | | | | 6.71E-02 | -0.34 | 0.95 | 7.19E-01 | 0.07 | 0.76 | 1.63E-01 | -0.31 | 0.84 | 6.84E-02 | -0.38 | 0.89 | 2650306 | 1304774 | 2130995 | 1563586 | 2074397 | 1478203 | 2187592 | 1657672 |
| RP_POS_649_4491a10_465 | RP | POS | 10.47 | 649.4491 | | | | 4.40E-01 | -0.20 | 0.68 | 7.19E-01 | -0.06 | 0.29 | 3.80E-01 | -0.23 | 0.45 | 5.54E-01 | -0.16 | 0.59 | 8557513 | 1277960 | 8369686 | 834506 | 8399891 | 970550 | 8339481 | 680574 |
| HILIC_POS_143_34065a3_53 | HILIC | POS | 3.53 | 143.3407 | | | | 2.62E-01 | 0.20 | 0.48 | 7.20E-01 | -0.08 | 0.52 | 3.96E-01 | 0.16 | 0.34 | 2.93E-01 | 0.23 | 0.54 | 356218 | 536271 | 492066 | 737095 | 518739 | 853101 | 465394 | 607020 |
| HILIC_POS_134_11757a1_88 | HILIC | POS | 1.88 | 134.1176 | | | | 4.07E-03 | -0.47 | 1.07 | 7.20E-01 | 0.08 | 0.69 | 3.42E-02 | -0.43 | 1.16 | 7.18E-03 | -0.51 | 1.07 | 523830 | 149146 | 418594 | 239873 | 409920 | 224585 | 427267 | 256243 |
| RP_NEG_222_81096a0_768 | RP | NEG | 0.77 | 222.8110 | | | | 3.51E-04 | -0.77 | 1.48 | 7.20E-01 | -0.07 | 0.23 | 4.64E-04 | -0.81 | 1.44 | 1.70E-03 | -0.74 | 1.27 | 1966105 | 519137 | 1568678 | 476369 | 1585894 | 502883 | 1551461 | 452758 |
| RP_POS_263_16165a9_225 | RP | POS | 9.23 | 263.1617 | | | | 3.76E-01 | 0.14 | 1.23 | 7.20E-01 | -0.08 | 1.45 | 5.60E-01 | 0.10 | 1.01 | 3.89E-01 | 0.18 | 1.04 | 715950 | 364141 | 793959 | 594070 | 815431 | 672149 | 772487 | 510212 |
| RP_POS_137_04562a0_709 | RP | POS | 0.71 | 137.0456 | Hypoxanthine | 137.0455(100), 94.0399(1), 110.0345(1), 119.0361(1) | 3 | 1.10E-12 | 0.87 | 1.64 | 7.20E-01 | 0.08 | 0.78 | 1.95E-06 | 0.91 | 1.67 | 8.69E-08 | 0.83 | 1.78 | 2714190 | 1488139 | 43399016 | 49842283 | 41597840 | 43777837 | 45200191 | 55642478 |
| RP_POS_464_85721a0_498 | RP | POS | 0.50 | 464.8572 | | | | 9.36E-07 | -0.60 | 1.32 | 7.20E-01 | 0.08 | 1.19 | 4.82E-04 | -0.56 | 1.39 | 4.45E-04 | -0.64 | 1.41 | 3304389 | 242779 | 2506813 | 1476253 | 2453488 | 1576590 | 2560137 | 1382635 |
| HILIC_POS_360_21661a0_555 | HILIC | POS | 0.555 | 360.2166 | | | | 1.10E-02 | -0.70 | 1.24 | 7.20E-01 | -0.05 | 0.84 | 1.00E-02 | -0.73 | 1.10 | 1.86E-02 | -0.67 | 0.97 | 37530367 | 20207562 | 27540478 | 10777868 | 27929701 | 11555582 | 27151255 | 10042654 |
| RP_POS_282_27957a11_159 | RP | POS | 11.16 | 282.2796 | | | | 7.41E-08 | 0.79 | 1.27 | 7.20E-01 | -0.08 | 0.65 | 1.45E-04 | 0.75 | 1.25 | 1.37E-06 | 0.82 | 1.54 | 10104724 | 2915843 | 14739949 | 6173663 | 14962714 | 5460318 | 14517184 | 6862194 |
| RP_POS_382_18866a10_584 | RP | POS | 10.58 | 382.1887 | | | | 1.98E-03 | 0.57 | 0.94 | 7.21E-01 | -0.07 | 0.40 | 1.19E-02 | 0.53 | 0.91 | 3.71E-03 | 0.60 | 1.11 | 1795254 | 781916 | 2342239 | 983618 | 2377686 | 967248 | 2306791 | 1008275 |
| HILIC_POS_189_16013a6_797 | HILIC | POS | 6.797 | 189.1601 | | | | 5.03E-02 | 0.29 | 0.63 | 7.21E-01 | 0.08 | 0.58 | 1.09E-01 | 0.33 | 0.55 | 1.21E-01 | 0.25 | 0.68 | 4546322 | 2046985 | 5610660 | 3972449 | 5467566 | 3278658 | 5753753 | 4592534 |
| RP_POS_804_55206a12_491 | RP | POS | 12.49 | 804.5521 | | | | 6.83E-01 | -0.07 | 0.89 | 7.21E-01 | 0.08 | 0.35 | 8.71E-01 | -0.04 | 0.65 | 5.77E-01 | -0.11 | 0.49 | 8195953 | 2890813 | 7936821 | 3724946 | 7802807 | 3396899 | 8070836 | 4056850 |
| RP_NEG_401_95358a0_645 | RP | NEG | 0.65 | 401.9536 | | | | 4.98E-12 | -0.85 | 1.50 | 7.21E-01 | 0.08 | 1.18 | 1.46E-06 | -0.81 | 1.62 | 7.89E-07 | -0.89 | 1.59 | 3389336 | 172469 | 2323502 | 1339280 | 2275328 | 1385062 | 2371675 | 1304153 |
| RP_POS_579_40063a11_598 | RP | POS | 11.60 | 579.4006 | | | | 2.51E-02 | -0.47 | 0.79 | 7.21E-01 | 0.07 | 0.35 | 4.97E-02 | -0.44 | 0.76 | 3.48E-02 | -0.51 | 0.73 | 787277 | 118907 | 732047 | 114201 | 727940 | 125927 | 736154 | 102264 |
| RP_POS_316_77582a0_521 | RP | POS | 0.52 | 316.7758 | | | | 1.43E-06 | 0.59 | 1.27 | 7.21E-01 | -0.08 | 1.11 | 6.14E-04 | 0.55 | 1.30 | 5.51E-04 | 0.63 | 1.38 | 179513 | 42076 | 317039 | 259725 | 326374 | 277274 | 307703 | 243377 |
| RP_POS_820_54456a11_906 | RP | POS | 11.91 | 820.5446 | | | | 3.08E-02 | 0.26 | 0.64 | 7.21E-01 | -0.08 | 0.48 | 1.49E-01 | 0.22 | 0.60 | 9.58E-02 | 0.30 | 0.71 | 856330 | 483451 | 1412349 | 2396848 | 1498490 | 2609315 | 1326208 | 2187084 |
| RP_POS_302_24524a10_641 | RP | POS | 10.64 | 302.2452 | | | | 4.29E-03 | 0.51 | 0.85 | 7.21E-01 | -0.07 | 0.43 | 2.32E-02 | 0.48 | 0.82 | 7.22E-03 | 0.55 | 1.08 | 32915606 | 14995631 | 42656736 | 19589495 | 43360363 | 19088169 | 41953108 | 20247461 |
| RP_POS_185_11501a10_578 | RP | POS | 10.58 | 185.1150 | | | | 9.30E-01 | -0.02 | 0.28 | 7.22E-01 | -0.07 | 0.24 | 8.14E-01 | -0.05 | 0.29 | 9.34E-01 | 0.02 | 0.42 | 2236953 | 685053 | 2224603 | 703268 | 2249845 | 680011 | 2199361 | 731814 |
| RP_POS_399_76999a13_723 | RP | POS | 13.72 | 399.7700 | | | | 5.10E-03 | 0.51 | 1.04 | 7.23E-01 | 0.07 | 0.23 | 1.01E-02 | 0.54 | 1.02 | 2.19E-02 | 0.47 | 1.08 | 6173007 | 1584470 | 7176468 | 2038655 | 7103647 | 1995029 | 7249290 | 2099067 |
| RP_NEG_209_15465a7_723 | RP | NEG | 7.72 | 209.1547 | | | | 2.41E-01 | -0.23 | 0.56 | 7.23E-01 | -0.07 | 0.35 | 2.09E-01 | -0.26 | 0.57 | 4.04E-01 | -0.19 | 0.51 | 2964676 | 147305 | 2928227 | 163927 | 2934074 | 180288 | 2922379 | 147374 |
| RP_NEG_856_75732a0_498 | RP | NEG | 0.50 | 856.7573 | | | | 1.94E-03 | -0.42 | 1.18 | 7.24E-01 | -0.08 | 1.23 | 6.87E-03 | -0.46 | 1.24 | 4.14E-02 | -0.38 | 1.22 | 2258783 | 413101 | 1831467 | 1135834 | 1871928 | 1208873 | 1791006 | 1068575 |
| RP_POS_364_1676a10_731 | RP | POS | 10.73 | 364.1676 | | | | 2.76E-03 | 0.49 | 1.38 | 7.24E-01 | -0.08 | 1.13 | 1.81E-02 | 0.45 | 1.20 | 8.90E-03 | 0.52 | 1.34 | 1844469 | 825961 | 2453164 | 1329336 | 2500420 | 1387551 | 2405909 | 1280812 |
| HILIC_NEG_448_74265a2_929 | HILIC | NEG | 2.929 | 448.7427 | | | | 1.62E-06 | -0.63 | 1.34 | 7.25E-01 | 0.08 | 0.76 | 3.58E-04 | -0.60 | 1.45 | 2.97E-04 | -0.67 | 1.47 | 151963 | 21361 | 114588 | 64054 | 112314 | 68137 | 116862 | 60302 |
| RP_POS_291_64645a10_303 | RP | POS | 10.30 | 291.6465 | | | | 3.85E-02 | 0.46 | 0.81 | 7.25E-01 | -0.07 | 0.49 | 8.76E-02 | 0.43 | 0.69 | 3.34E-02 | 0.49 | 0.86 | 408905 | 127216 | 462187 | 109605 | 466077 | 94731 | 458296 | 123558 |
| RP_POS_292_90692a15_364 | RP | POS | 15.36 | 292.9069 | | | | 6.49E-01 | 0.09 | 0.50 | 7.25E-01 | -0.07 | 0.26 | 8.13E-01 | 0.05 | 0.34 | 5.66E-01 | 0.13 | 0.53 | 1486281 | 278666 | 1512681 | 301671 | 1523388 | 293273 | 1501974 | 312451 |
| RP_POS_325_12433a6_254 | RP | POS | 6.25 | 325.1243 | | | | 2.79E-01 | -0.22 | 0.48 | 7.25E-01 | -0.07 | 0.29 | 2.56E-01 | -0.25 | 0.48 | 4.24E-01 | -0.18 | 0.34 | 54482280 | 5538297 | 53238947 | 5775737 | 53443910 | 5936904 | 53033985 | 5662730 |

**Supplementary table 1: Metabolomics results** (in asceding order according to p-value from progressive ALD vs  non-progressive ALD comparison, with molecular features with ID_level of 1-2 first)

| | | | | | | | | all_ALD_vs_control | | | Prog_ALD_vs_non-prog_ALD | | | Prog_ALD_vs_control_ALD | | | Non-prog_ALD_vs_control | | | Healthy_control | | all_ALD | | Non-prog_ALD | | Progressive_ALD | |
|---|---|---|---|---|---|---|---|---|---|---|---|---|---|---|---|---|---|---|---|---|---|---|---|---|---|---|---|
| | | | | | | | | t-test | Cohen's | PLS-DA | t-test | Cohen's | PLS-DA | t-test | Cohen's | PLS-DA | t-test | Cohen's | PLS-DA | | | | | | | | |
| Feature_ID | RP_HILIC | POS_NEG | Average.Rt | Average.Mz | Metabolite.name | curated_MSMS | ID_level | p | d | VIP | p | d | VIP | p | d | VIP | p | d | VIP | Mean | SD | Mean | SD | Mean | SD | Mean | SD |
| HILIC_NEG_215_97316a0_934 | HILIC | NEG | 0.934 | 215.9732 | | | | 1.02E-08 | 0.85 | 1.47 | 7.25E-01 | 0.07 | 1.16 | 2.36E-05 | 0.89 | 1.39 | 2.33E-07 | 0.81 | 1.77 | 1419033 | 368418 | 2032800 | 743441 | 2006411 | 572178 | 2059190 | 887626 |
| HILIC_NEG_130_99246a0_7 | HILIC | NEG | 0.7 | 130.9925 | | | | 3.20E-04 | 0.66 | 1.11 | 7.25E-01 | 0.07 | 1.08 | 2.00E-03 | 0.69 | 1.12 | 1.15E-03 | 0.62 | 1.21 | 700164 | 259271 | 914433 | 328785 | 902767 | 270571 | 926100 | 380682 |
| RP_POS_744_84302a0_49 | RP | POS | 0.49 | 744.8430 | | | | 4.44E-08 | 0.66 | 1.36 | 7.25E-01 | -0.08 | 1.20 | 1.53E-04 | 0.62 | 1.44 | 9.03E-05 | 0.70 | 1.46 | 500697 | 186756 | 2092436 | 2670228 | 2187134 | 2788272 | 1997739 | 2571572 |
| RP_POS_291_84552a15_571 | RP | POS | 15.57 | 291.8455 | | | | 7.23E-01 | 0.07 | 0.40 | 7.25E-01 | 0.07 | 0.22 | 6.44E-01 | 0.11 | 0.28 | 8.78E-01 | 0.03 | 0.50 | 884364 | 38386 | 887213 | 42380 | 885710 | 38968 | 888716 | 45887 |
| RP_POS_702_53503a10_657 | RP | POS | 10.66 | 702.5350 | | | | 7.36E-01 | -0.07 | 0.27 | 7.25E-01 | -0.07 | 0.28 | 6.46E-01 | -0.11 | 0.36 | 8.78E-01 | -0.04 | 0.25 | 3050330 | 330956 | 3027879 | 311664 | 3038928 | 321840 | 3016829 | 304008 |
| HILIC_POS_356_35199a0_521 | HILIC | POS | 0.521 | 356.3520 | | | | 4.30E-02 | -0.48 | 0.96 | 7.25E-01 | 0.06 | 0.33 | 7.74E-02 | -0.45 | 0.72 | 4.25E-02 | -0.51 | 0.82 | 1891551 | 684374 | 1617073 | 517963 | 1598710 | 510659 | 1635436 | 529703 |
| HILIC_POS_175_14423a6_711 | HILIC | POS | 6.711 | 175.1442 | | | | 5.48E-01 | 0.11 | 0.77 | 7.25E-01 | 0.07 | 0.57 | 4.91E-01 | 0.15 | 0.55 | 7.29E-01 | 0.07 | 0.80 | 1715934 | 982741 | 1844465 | 1229714 | 1800904 | 1211880 | 1888026 | 1258065 |
| RP_POS_406_20081a7_106 | RP | POS | 7.11 | 406.2008 | | | | 1.63E-01 | -0.32 | 1.06 | 7.25E-01 | -0.07 | 0.49 | 1.55E-01 | -0.35 | 0.82 | 2.45E-01 | -0.29 | 1.04 | 5905887 | 567553 | 5749147 | 460424 | 5765452 | 458813 | 5732843 | 466105 |
| HILIC_POS_584_35632a1_005 | HILIC | POS | 1.005 | 584.3563 | | | | 1.36E-05 | 0.53 | 1.25 | 7.25E-01 | -0.08 | 0.78 | 3.12E-03 | 0.49 | 1.42 | 1.15E-03 | 0.57 | 1.38 | 347639 | 923917 | 2923581 | 5425785 | 3115681 | 5576728 | 2731482 | 5320140 |
| RP_POS_184_07391a10_822 | RP | POS | 10.82 | 184.0739 | | | | 7.91E-01 | -0.06 | 0.79 | 7.25E-01 | -0.07 | 0.93 | 7.11E-01 | -0.09 | 0.48 | 9.09E-01 | -0.03 | 0.75 | 576323 | 224999 | 564713 | 179133 | 571047 | 165674 | 558379 | 193138 |
| RP_NEG_1067_61292a10_304 | RP | NEG | 10.30 | 1067.6129 | | | | 2.39E-04 | 0.59 | 1.21 | 7.26E-01 | -0.07 | 0.30 | 6.14E-03 | 0.55 | 1.03 | 6.67E-04 | 0.63 | 1.31 | 506924 | 237343 | 726786 | 391509 | 740626 | 360160 | 712946 | 423769 |
| HILIC_POS_146_08116a0_801 | HILIC | POS | 0.801 | 146.0812 | | | | 9.27E-01 | 0.02 | 0.24 | 7.26E-01 | 0.08 | 0.49 | 7.91E-01 | 0.05 | 0.29 | 9.11E-01 | -0.02 | 0.31 | 4572596 | 2797821 | 4631535 | 4071573 | 4487679 | 4061979 | 4775390 | 4117259 |
| RP_NEG_221_94632a14_268 | RP | NEG | 14.27 | 221.9463 | | | | 7.18E-01 | -0.08 | 0.63 | 7.26E-01 | 0.07 | 0.25 | 8.53E-01 | -0.05 | 0.46 | 6.29E-01 | -0.11 | 0.55 | 5945104 | 1586112 | 5831937 | 1362594 | 5783800 | 1256363 | 5880073 | 1472426 |
| RP_NEG_662_42291a11_254 | RP | NEG | 11.25 | 662.4229 | | | | 5.85E-07 | 0.97 | 1.82 | 7.26E-01 | 0.07 | 1.08 | 5.85E-06 | 1.00 | 1.76 | 4.92E-06 | 0.94 | 1.79 | 3325787 | 1462328 | 5050110 | 1671839 | 4991082 | 1512192 | 5109139 | 1831201 |
| RP_POS_420_78503a0_251 | RP | POS | 0.25 | 420.7850 | | | | 5.51E-01 | -0.13 | 0.48 | 7.26E-01 | 0.07 | 0.46 | 6.93E-01 | -0.10 | 0.25 | 4.84E-01 | -0.16 | 0.58 | 674040 | 73032 | 665383 | 64446 | 663108 | 61041 | 667657 | 68227 |
| HILIC_POS_582_59375a10_679 | HILIC | POS | 10.679 | 582.5938 | | | | 4.35E-01 | -0.14 | 0.51 | 7.26E-01 | 0.07 | 0.51 | 3.90E-01 | -0.18 | 0.60 | 6.23E-01 | -0.10 | 0.27 | 3248155 | 195953 | 3214705 | 248343 | 3223465 | 255258 | 3205946 | 243502 |
| RP_NEG_400_15421a11_162 | RP | NEG | 11.16 | 400.1542 | | | | 2.16E-02 | 0.40 | 1.15 | 7.26E-01 | -0.07 | 1.01 | 7.29E-02 | 0.36 | 1.00 | 3.28E-02 | 0.44 | 1.20 | 1551548 | 154513 | 1632956 | 213267 | 1640472 | 215591 | 1625439 | 212835 |
| HILIC_NEG_354_99301a7_309 | HILIC | NEG | 7.309 | 354.9930 | | | | 2.48E-01 | 0.22 | 1.14 | 7.26E-01 | -0.07 | 0.36 | 3.87E-01 | 0.19 | 0.61 | 2.43E-01 | 0.26 | 1.13 | 967818 | 28559 | 974812 | 32285 | 975950 | 33409 | 973675 | 31418 |
| RP_NEG_195_81001a2_45 | RP | NEG | 2.45 | 195.8100 | | | | 8.93E-01 | 0.03 | 0.59 | 7.27E-01 | 0.07 | 0.38 | 7.74E-01 | 0.06 | 0.24 | 9.67E-01 | -0.01 | 0.72 | 8261354 | 3751121 | 8366298 | 4028588 | 8224592 | 4261808 | 8508005 | 3819198 |
| RP_POS_325_27209a10_866 | RP | POS | 10.87 | 325.2721 | | | | 9.66E-02 | -0.31 | 0.66 | 7.28E-01 | -0.07 | 1.10 | 1.09E-01 | -0.35 | 0.83 | 1.90E-01 | -0.28 | 0.54 | 4791111 | 1127673 | 4387483 | 1321728 | 4433869 | 1286298 | 4341097 | 1367708 |
| HILIC_NEG_198_88084a7_668 | HILIC | NEG | 7.668 | 198.8808 | | | | 3.11E-01 | 0.17 | 0.67 | 7.28E-01 | -0.07 | 0.70 | 5.01E-01 | 0.13 | 0.51 | 3.00E-01 | 0.21 | 0.54 | 333542 | 12656 | 336495 | 18333 | 337137 | 18549 | 335853 | 18280 |
| RP_POS_306_93033a15_367 | RP | POS | 15.37 | 306.9303 | | | | 8.45E-01 | -0.03 | 0.32 | 7.28E-01 | 0.08 | 0.49 | 9.66E-01 | 0.01 | 0.30 | 7.65E-01 | -0.07 | 0.46 | 2303646 | 125655 | 2297781 | 200660 | 2290749 | 260445 | 2304813 | 115836 |
| RP_NEG_552_85883a0_497 | RP | NEG | 0.50 | 552.8588 | | | | 2.07E-07 | -0.64 | 1.34 | 7.28E-01 | 0.08 | 1.20 | 2.25E-04 | -0.60 | 1.42 | 1.98E-04 | -0.67 | 1.42 | 14012282 | 996033 | 10584531 | 5944866 | 10376473 | 6305000 | 10792589 | 5618074 |
| RP_POS_797_58649a13_3 | RP | POS | 13.30 | 797.5865 | | | | 2.28E-05 | -0.90 | 1.71 | 7.28E-01 | 0.06 | 0.58 | 3.67E-04 | -0.87 | 1.59 | 1.38E-05 | -0.93 | 1.72 | 2756416 | 1121746 | 1704835 | 1066759 | 1667491 | 760402 | 1742178 | 1310770 |
| RP_POS_609_30933a10_931 | RP | POS | 10.93 | 609.3093 | | | | 2.73E-04 | -0.85 | 1.39 | 7.29E-01 | -0.06 | 0.52 | 3.95E-04 | -0.88 | 1.39 | 7.84E-04 | -0.82 | 1.35 | 438837 | 168489 | 309934 | 131534 | 314530 | 128833 | 305337 | 135331 |
| RP_POS_277_21381a10_629 | RP | POS | 10.63 | 277.2138 | | | | 4.19E-03 | -0.59 | 1.21 | 7.29E-01 | 0.07 | 0.65 | 1.69E-02 | -0.56 | 1.09 | 4.59E-03 | -0.63 | 1.19 | 742309 | 248068 | 591610 | 246591 | 582996 | 224626 | 600223 | 268789 |
| RP_POS_771_64435a11_126 | RP | POS | 11.13 | 771.6444 | | | | 1.30E-01 | 0.27 | 0.69 | 7.30E-01 | -0.07 | 0.25 | 2.22E-01 | 0.23 | 0.76 | 1.73E-01 | 0.30 | 0.58 | 378494 | 890681 | 682205 | 1205928 | 724194 | 1385301 | 640216 | 1007744 |
| RP_POS_218_98363a0_453 | RP | POS | 0.45 | 218.9836 | | | | 3.74E-04 | -0.54 | 1.22 | 7.30E-01 | -0.07 | 1.05 | 1.67E-03 | -0.58 | 1.19 | 8.71E-03 | -0.50 | 1.27 | 355423 | 103084 | 257350 | 194254 | 264111 | 202302 | 250589 | 187669 |
| RP_POS_797_58752a13_443 | RP | POS | 13.44 | 797.5875 | | | | 1.65E-08 | -1.25 | 2.20 | 7.30E-01 | 0.06 | 0.87 | 2.07E-07 | -1.22 | 2.03 | 1.72E-08 | -1.27 | 2.15 | 3592349 | 1183801 | 2005149 | 1043887 | 1968893 | 900276 | 2041406 | 1178330 |
| HILIC_NEG_110_89904a2_948 | HILIC | NEG | 2.948 | 110.8990 | | | | 1.32E-05 | -0.60 | 1.34 | 7.30E-01 | 0.08 | 0.79 | 8.70E-04 | -0.56 | 1.45 | 8.35E-04 | -0.63 | 1.40 | 26651666 | 3429849 | 21760266 | 8915845 | 21450657 | 9591098 | 22069876 | 8271765 |
| HILIC_NEG_155_86559a3_023 | HILIC | NEG | 3.023 | 155.8656 | | | | 3.96E-01 | -0.16 | 0.95 | 7.31E-01 | -0.07 | 0.80 | 3.62E-01 | -0.20 | 0.83 | 5.57E-01 | -0.13 | 0.35 | 24633995 | 4555649 | 23819020 | 5091948 | 23995490 | 5124281 | 23642551 | 5105212 |
| RP_NEG_304_91385a15_345 | RP | NEG | 15.35 | 304.9139 | | | | 6.04E-01 | -0.10 | 0.57 | 7.31E-01 | -0.07 | 0.39 | 5.12E-01 | -0.14 | 0.64 | 7.77E-01 | -0.07 | 0.24 | 8774077 | 321771 | 8739100 | 354318 | 8751361 | 398592 | 8726838 | 307348 |
| RP_POS_169_03601a0_98 | RP | POS | 0.98 | 169.0360 | Uric acid | 169.0352(100), 141.04(18), 126.0298(5), 152.009(4) | 3 | 4.72E-04 | 0.55 | 1.04 | 7.31E-01 | 0.07 | 0.34 | 4.35E-03 | 0.59 | 1.09 | 3.42E-03 | 0.52 | 0.97 | 14025126 | 3384477 | 17052574 | 5823659 | 16851052 | 5094483 | 17254097 | 6518092 |
| RP_POS_266_15909a4_09 | RP | POS | 4.09 | 266.1591 | | | | 6.56E-01 | 0.09 | 0.43 | 7.31E-01 | 0.07 | 0.36 | 5.93E-01 | 0.13 | 0.33 | 7.94E-01 | 0.06 | 0.59 | 1383288 | 74718 | 1390008 | 71372 | 1387539 | 66205 | 1392477 | 76788 |
| HILIC_NEG_115_92072a5_882 | HILIC | NEG | 5.882 | 115.9207 | | | | 4.81E-02 | -0.42 | 1.10 | 7.32E-01 | 0.07 | 0.78 | 9.84E-02 | -0.38 | 0.71 | 5.20E-02 | -0.45 | 0.96 | 43710194 | 7938631 | 40481156 | 7550031 | 40220342 | 7538951 | 40741970 | 7628541 |
| RP_POS_648_80328a0_499 | RP | POS | 0.50 | 648.8033 | | | | 8.37E-03 | -0.36 | 1.18 | 7.32E-01 | -0.08 | 1.21 | 1.75E-02 | -0.39 | 1.20 | 9.14E-02 | -0.32 | 1.23 | 1056217 | 189726 | 891346 | 516107 | 909162 | 558621 | 873530 | 474831 |
| RP_POS_553_38934a12_462 | RP | POS | 12.46 | 553.3893 | Unknown | 553.454(95), 313.181(100) | 4 | 5.62E-01 | 0.09 | 0.20 | 7.32E-01 | -0.08 | 0.22 | 7.90E-01 | 0.05 | 0.22 | 4.84E-01 | 0.13 | 0.31 | 7150248 | 834736 | 7270533 | 1447388 | 7320486 | 1356083 | 7220581 | 1545499 |
| RP_POS_646_35687a14_267 | RP | POS | 14.27 | 646.3569 | | | | 8.40E-01 | -0.04 | 0.46 | 7.32E-01 | -0.07 | 0.67 | 7.48E-01 | -0.08 | 0.48 | 9.67E-01 | -0.01 | 0.42 | 2528568 | 748060 | 2498243 | 687976 | 2521976 | 649620 | 2474510 | 730156 |
| RP_POS_298_27402a8_967 | RP | POS | 8.97 | 298.2740 | | | | 2.27E-01 | 0.24 | 0.47 | 7.32E-01 | 0.07 | 0.35 | 2.54E-01 | 0.28 | 0.58 | 3.15E-01 | 0.21 | 0.47 | 439159 | 770911 | 633112 | 816754 | 604932 | 641058 | 661292 | 967063 |
| RP_POS_548_50494a14_165 | RP | POS | 14.17 | 548.5049 | | | | 5.16E-01 | -0.16 | 0.49 | 7.32E-01 | 0.06 | 0.22 | 6.18E-01 | -0.13 | 0.83 | 4.72E-01 | -0.19 | 0.48 | 3939421 | 2253249 | 3658813 | 1567368 | 3604789 | 1646675 | 3712837 | 1498641 |
| RP_NEG_734_83112a0_467 | RP | NEG | 0.47 | 734.8311 | | | | 1.51E-09 | -0.76 | 1.41 | 7.33E-01 | 0.07 | 1.17 | 2.39E-05 | -0.72 | 1.50 | 7.90E-06 | -0.80 | 1.51 | 652778 | 64688 | 448138 | 291384 | 438108 | 297577 | 458169 | 287723 |
| RP_POS_400_27829a11_25 | RP | POS | 11.25 | 400.2783 | | | | 2.49E-11 | 1.17 | 1.83 | 7.33E-01 | 0.06 | 0.44 | 3.48E-09 | 1.20 | 1.89 | 3.17E-09 | 1.14 | 1.90 | 1151690 | 490432 | 2022348 | 689189 | 1998624 | 653775 | 2046071 | 728756 |
| RP_NEG_280_90869a0_499 | RP | NEG | 0.50 | 280.9087 | | | | 1.78E-03 | -0.41 | 1.18 | 7.33E-01 | -0.08 | 1.23 | 6.75E-03 | -0.45 | 1.24 | 4.26E-02 | -0.37 | 1.23 | 2147442 | 373593 | 1733131 | 1118233 | 1771581 | 1196180 | 1694680 | 1045249 |
| RP_NEG_355_15854a11_162 | RP | NEG | 11.16 | 355.1585 | | | | 7.54E-02 | 0.31 | 1.07 | 7.33E-01 | 0.07 | 0.90 | 9.36E-02 | 0.35 | 0.98 | 1.71E-01 | 0.28 | 0.96 | 19053108 | 1485623 | 19647519 | 1987104 | 19579197 | 1947951 | 19715842 | 2042965 |
| RP_POS_170_95744a0_468 | RP | POS | 0.47 | 170.9574 | | | | 3.28E-08 | -0.69 | 1.37 | 7.33E-01 | 0.08 | 1.18 | 1.26E-04 | -0.65 | 1.44 | 4.21E-05 | -0.72 | 1.47 | 491213 | 46214 | 350327 | 223980 | 342629 | 228879 | 358024 | 221024 |
| RP_NEG_300_74933a0_522 | RP | NEG | 0.52 | 300.7493 | | | | 7.54E-06 | -0.61 | 1.27 | 7.33E-01 | -0.07 | 1.24 | 1.18E-04 | -0.65 | 1.40 | 2.41E-03 | -0.57 | 1.29 | 653678 | 117434 | 479196 | 310536 | 489868 | 336264 | 468523 | 285531 |
| HILIC_NEG_280_81897a2_932 | HILIC | NEG | 2.932 | 280.8190 | | | | 1.70E-06 | -0.62 | 1.34 | 7.33E-01 | 0.07 | 0.77 | 3.82E-04 | -0.59 | 1.46 | 3.64E-04 | -0.66 | 1.47 | 285891 | 35191 | 221111 | 113208 | 217225 | 120862 | 224996 | 106090 |
| RP_POS_488_58948a6_727 | RP | POS | 6.73 | 488.5895 | | | | 5.28E-07 | 0.60 | 1.30 | 7.34E-01 | -0.08 | 1.12 | 5.63E-04 | 0.56 | 1.41 | 3.18E-04 | 0.64 | 1.36 | 31509 | 79219 | 1007223 | 1814047 | 1069378 | 1892872 | 945067 | 1748669 |
| RP_NEG_376_12936a11_16 | RP | NEG | 11.16 | 376.1294 | | | | 3.08E-03 | 0.53 | 1.30 | 7.34E-01 | -0.07 | 1.17 | 1.60E-02 | 0.50 | 1.24 | 6.43E-03 | 0.57 | 1.29 | 714143 | 83825 | 770712 | 109567 | 774465 | 110512 | 766958 | 109604 |
| RP_POS_796_52307a13_16 | RP | POS | 13.16 | 796.5231 | | | | 2.65E-02 | -0.48 | 0.88 | 7.34E-01 | -0.07 | 0.27 | 3.25E-02 | -0.51 | 0.94 | 5.37E-02 | -0.45 | 0.75 | 23821781 | 8749218 | 19841606 | 7948111 | 20113630 | 7608254 | 19569582 | 8342518 |
| RP_POS_561_39673a10_546 | RP | POS | 10.55 | 561.3967 | | | | 5.31E-01 | -0.21 | 0.47 | 7.34E-01 | 0.03 | 0.23 | 5.68E-01 | -0.19 | 0.34 | 5.04E-01 | -0.23 | 0.32 | 5220838 | 3454511 | 4829585 | 947525 | 4797184 | 1033175 | 4861986 | 862749 |
| HILIC_POS_138_12769a0_668 | HILIC | POS | 0.668 | 138.1277 | | | | 1.07E-01 | -0.33 | 0.96 | 7.34E-01 | -0.07 | 0.81 | 1.12E-01 | -0.36 | 0.61 | 1.92E-01 | -0.29 | 0.51 | 1025863 | 211617 | 955002 | 215937 | 962381 | 214717 | 947623 | 219077 |
| RP_POS_265_90387a0_448 | RP | POS | 0.45 | 265.9039 | | | | 3.63E-08 | -0.68 | 1.36 | 7.35E-01 | 0.07 | 1.17 | 1.30E-04 | -0.64 | 1.43 | 5.02E-05 | -0.72 | 1.47 | 1001682 | 88029 | 718110 | 455246 | 702560 | 467443 | 733661 | 446913 |
| RP_POS_335_12476a9_476 | RP | POS | 9.48 | 335.1248 | | | | 4.66E-02 | -0.45 | 0.80 | 7.35E-01 | -0.06 | 0.50 | 4.13E-02 | -0.49 | 0.84 | 9.54E-02 | -0.42 | 0.81 | 1320110 | 139325 | 1264626 | 113457 | 1268501 | 126947 | 1260751 | 99306 |
| RP_POS_668_82117a0_497 | RP | POS | 0.50 | 668.8212 | | | | 6.15E-08 | -0.66 | 1.36 | 7.35E-01 | 0.08 | 1.19 | 1.93E-04 | -0.62 | 1.43 | 8.02E-05 | -0.70 | 1.46 | 1548718 | 84537 | 1183927 | 609724 | 1163134 | 627769 | 1204720 | 596773 |
| RP_POS_560_31519a6_996 | RP | POS | 7.00 | 560.3152 | | | | 2.26E-07 | 0.62 | 1.33 | 7.35E-01 | -0.08 | 1.12 | 4.27E-04 | 0.58 | 1.41 | 1.83E-04 | 0.65 | 1.41 | 9899 | 8744 | 759496 | 1347311 | 805397 | 1389347 | 713594 | 1316417 |
| RP_POS_497_39578a15_235 | RP | POS | 15.24 | 497.3958 | | | | 5.53E-02 | -0.38 | 0.84 | 7.35E-01 | -0.07 | 0.25 | 5.50E-02 | -0.42 | 0.73 | 1.28E-01 | -0.35 | 0.88 | 7158521 | 3131000 | 5896990 | 3295464 | 6009136 | 3538912 | 5784844 | 3064675 |
| RP_POS_1084_7804a0_494 | RP | POS | 0.49 | 1084.7804 | | | | 6.79E-08 | 0.65 | 1.35 | 7.36E-01 | -0.08 | 1.10 | 3.33E-04 | 0.61 | 1.39 | 6.68E-05 | 0.68 | 1.48 | 31528 | 12209 | 484567 | 776149 | 510954 | 777610 | 458179 | 781672 |
| RP_POS_199_13033a15_211 | RP | POS | 15.21 | 199.1303 | | | | 7.02E-01 | 0.08 | 0.32 | 7.36E-01 | -0.07 | 0.49 | 8.32E-01 | 0.05 | 0.26 | 6.25E-01 | 0.12 | 0.40 | 36673418 | 26183269 | 38660944 | 22835387 | 39437026 | 22522109 | 37884863 | 23346887 |
| RP_POS_820_07532a13_209 | RP | POS | 13.21 | 820.0753 | | | | 1.02E-02 | 0.48 | 0.86 | 7.36E-01 | -0.07 | 0.30 | 3.05E-02 | 0.51 | 0.82 | 1.63E-02 | 0.44 | 1.03 | 387999 | 336010 | 578752 | 406968 | 564924 | 281427 | 592580 | 505010 |
| HILIC_NEG_194_90605a3_007 | HILIC | NEG | 3.007 | 194.9061 | | | | 8.40E-01 | -0.04 | 0.95 | 7.36E-01 | -0.07 | 0.77 | 7.27E-01 | -0.07 | 0.37 | 9.92E-01 | 0.00 | 0.44 | 1108869 | 211625 | 1099742 | 252659 | 1108319 | 266004 | 1091165 | 240972 |
| HILIC_POS_375_32135a3_198 | HILIC | POS | 3.198 | 375.3214 | | | | 9.31E-01 | -0.02 | 0.63 | 7.36E-01 | -0.07 | 0.52 | 8.19E-01 | -0.05 | 0.52 | 9.34E-01 | 0.02 | 0.47 | 8016710 | 2532327 | 7971110 | 2774741 | 8065296 | 2635542 | 7876923 | 2931095 |
| RP_POS_420_25281a11_31 | RP | POS | 11.31 | 420.2528 | | | | 2.26E-02 | -0.42 | 0.88 | 7.36E-01 | 0.07 | 0.77 | 6.08E-02 | -0.39 | 0.82 | 3.57E-02 | -0.46 | 0.96 | 802919 | 97787 | 753664 | 120632 | 749571 | 127251 | 757757 | 114779 |
| RP_POS_457_34787a10_882 | RP | POS | 10.88 | 457.3479 | | | | 5.13E-05 | -0.73 | 1.24 | 7.36E-01 | 0.07 | 0.90 | 7.16E-04 | -0.70 | 1.27 | 1.97E-04 | -0.77 | 1.26 | 50704421 | 2103237 | 48709290 | 2725662 | 48616841 | 2707536 | 48801739 | 2768012 |
| RP_POS_497_39572a13_631 | RP | POS | 13.63 | 497.3957 | | | | 5.33E-01 | -0.13 | 1.07 | 7.36E-01 | 0.07 | 0.87 | 6.54E-01 | -0.10 | 1.14 | 5.01E-01 | -0.17 | 0.66 | 6940597 | 1993979 | 6690761 | 1845071 | 6628180 | 2107106 | 6753342 | 1558897 |
| HILIC_NEG_144_92349a1_049 | HILIC | NEG | 1.049 | 144.9235 | | | | 8.97E-01 | -0.03 | 0.65 | 7.36E-01 | 0.07 | 0.62 | 9.76E-01 | 0.01 | 0.31 | 7.93E-01 | -0.06 | 0.19 | 34120331 | 8304625 | 33901216 | 8118087 | 33626006 | 8264122 | 34176426 | 8043715 |
| RP_POS_396_87027a0_498 | RP | POS | 0.50 | 396.8703 | | | | 1.75E-06 | -0.59 | 1.31 | 7.37E-01 | 0.07 | 1.20 | 5.94E-04 | -0.55 | 1.38 | 5.74E-04 | -0.63 | 1.40 | 3157571 | 284193 | 2399338 | 1421327 | 2351180 | 1517426 | 2447497 | 1332012 |
| RP_NEG_605_40747a12_092 | RP | NEG | 12.09 | 605.4075 | | | | 5.00E-01 | -0.14 | 0.76 | 7.37E-01 | -0.07 | 0.41 | 4.61E-01 | -0.17 | 0.58 | 6.35E-01 | -0.11 | 0.45 | 1250188 | 632442 | 1163791 | 607272 | 1184315 | 574210 | 1143266 | 643824 |
| HILIC_POS_160_13315a1_611 | HILIC | POS | 1.611 | 160.1332 | | | | 5.92E-05 | 0.54 | 1.24 | 7.37E-01 | 0.07 | 0.61 | 5.63E-03 | 0.58 | 1.21 | 2.96E-04 | 0.51 | 1.54 | 473874 | 251537 | 814008 | 687396 | 790765 | 500203 | 837252 | 838667 |
| RP_POS_812_8302a0_492 | RP | POS | 0.49 | 812.8302 | | | | 3.15E-08 | 0.66 | 1.37 | 7.38E-01 | -0.07 | 1.19 | 1.29E-04 | 0.63 | 1.45 | 7.62E-05 | 0.70 | 1.47 | 269797 | 136934 | 1552161 | 2125576 | 1623882 | 2212744 | 1480439 | 2054557 |
| RP_NEG_239_16548a9_224 | RP | NEG | 9.22 | 239.1655 | | | | 3.07E-01 | 0.17 | 1.30 | 7.38E-01 | -0.07 | 1.42 | 4.77E-01 | 0.13 | 1.07 | 3.33E-01 | 0.20 | 1.09 | 950717 | 581761 | 1092671 | 923681 | 1123831 | 1024773 | 1061510 | 819538 |
| RP_NEG_343_99506a0_632 | RP | NEG | 0.63 | 343.9951 | | | | 1.76E-08 | -0.69 | 1.37 | 7.38E-01 | 0.07 | 1.20 | 1.02E-04 | -0.65 | 1.44 | 3.91E-05 | -0.73 | 1.47 | 61261364 | 4285389 | 43580680 | 28109446 | 42633048 | 28825066 | 44528311 | 27634945 |
| HILIC_NEG_115_92065a7_352 | HILIC | NEG | 7.352 | 115.9207 | | | | 2.87E-01 | -0.20 | 0.43 | 7.38E-01 | -0.07 | 0.28 | 2.83E-01 | -0.23 | 0.38 | 4.37E-01 | -0.16 | 0.28 | 83057823 | 5201173 | 81863402 | 6261422 | 82074335 | 6079191 | 81652468 | 6493347 |
| RP_POS_184_99527a15_387 | RP | POS | 15.39 | 184.9953 | | | | 3.73E-02 | 0.39 | 0.76 | 7.38E-01 | -0.07 | 0.22 | 8.40E-02 | 0.36 | 0.72 | 5.51E-02 | 0.42 | 0.76 | 7379569 | 3564028 | 8998175 | 4262201 | 9141760 | 4591811 | 8854590 | 3946727 |
| HILIC_NEG_157_08696a0_635 | HILIC | NEG | 0.635 | 157.0870 | | | | 7.20E-02 | -0.44 | 0.84 | 7.38E-01 | -0.06 | 0.54 | 6.82E-02 | -0.47 | 0.79 | 1.15E-01 | -0.41 | 0.70 | 340926 | 240300 | 256573 | 167832 | 262224 | 174876 | 250921 | 162059 |
| RP_POS_530_84827a0_498 | RP | POS | 0.50 | 530.8483 | | | | 2.06E-07 | -0.63 | 1.34 | 7.39E-01 | 0.07 | 1.20 | 2.30E-04 | -0.60 | 1.42 | 2.11E-04 | -0.67 | 1.43 | 14159491 | 917524 | 10671144 | 6087410 | 10466732 | 6450918 | 10875557 | 5759318 |
| RP_POS_214_95479a15_564 | RP | POS | 15.56 | 214.9548 | | | | 3.38E-01 | -0.22 | 0.48 | 7.39E-01 | 0.06 | 0.29 | 4.54E-01 | -0.19 | 0.49 | 3.04E-01 | -0.25 | 0.50 | 1592844 | 63524 | 1580897 | 50999 | 1579185 | 48401 | 1582609 | 53910 |
| RP_POS_167_10422a5_52 | RP | POS | 5.52 | 167.1042 | | | | 6.05E-01 | -0.11 | 0.40 | 7.39E-01 | -0.07 | 0.26 | 5.55E-01 | -0.14 | 0.49 | 7.44E-01 | -0.07 | 0.33 | 1028686 | 169209 | 1010785 | 170484 | 1016506 | 155635 | 1005064 | 185562 |
| RP_POS_158_15398a7_649 | RP | POS | 7.65 | 158.1540 | | | | 6.06E-02 | -0.36 | 0.67 | 7.39E-01 | 0.07 | 0.34 | 1.54E-01 | -0.33 | 0.55 | 5.26E-02 | -0.40 | 0.89 | 37964479 | 1257513 | 37461652 | 1399263 | 37414692 | 1184813 | 37508612 | 1596105 |
| RP_POS_778_83624a0_491 | RP | POS | 0.49 | 778.8362 | | | | 7.18E-08 | 0.65 | 1.35 | 7.39E-01 | -0.07 | 1.19 | 2.10E-04 | 0.61 | 1.43 | 1.08E-04 | 0.68 | 1.45 | 381334 | 164861 | 1761248 | 2357324 | 1840262 | 2442984 | 1682235 | 2290514 |
| RP_POS_819_57544a13_714 | RP | POS | 13.71 | 819.5754 | | | | 4.19E-05 | -0.77 | 1.34 | 7.40E-01 | -0.07 | 0.93 | 1.63E-04 | -0.81 | 1.40 | 4.04E-04 | -0.74 | 1.31 | 3838571 | 1223378 | 2686933 | 1462871 | 2735911 | 1444564 | 2637955 | 1493987 |
| RP_NEG_235_92618a15_548 | RP | NEG | 15.55 | 235.9262 | | | | 2.68E-01 | 0.21 | 0.45 | 7.40E-01 | -0.07 | 0.30 | 4.28E-01 | 0.17 | 0.48 | 2.54E-01 | 0.24 | 0.62 | 7465800 | 203279 | 7514492 | 246243 | 7522736 | 240915 | 7506248 | 253634 |
| RP_NEG_568_83246a0_499 | RP | NEG | 0.50 | 568.8325 | | | | 4.14E-04 | -0.47 | 1.21 | 7.40E-01 | -0.07 | 1.24 | 2.75E-03 | -0.51 | 1.29 | 1.73E-02 | -0.44 | 1.25 | 5013170 | 868626 | 3971706 | 2427306 | 4052972 | 2558154 | 3890440 | 2312193 |
| HILIC_NEG_475_53125a1_365 | HILIC | NEG | 1.365 | 475.5313 | | | | 4.55E-01 | -0.11 | 0.38 | 7.40E-01 | -0.07 | 0.34 | 4.59E-01 | -0.15 | 0.32 | 6.53E-01 | -0.08 | 0.45 | 309756 | 109820 | 288936 | 198769 | 295591 | 174448 | 282282 | 222040 |
| HILIC_NEG_219_8455a2_947 | HILIC | NEG | 2.947 | 219.8455 | | | | 4.49E-02 | 0.35 | 0.99 | 7.40E-01 | -0.07 | 0.85 | 1.18E-01 | 0.32 | 0.55 | 6.16E-02 | 0.39 | 0.70 | 696002 | 137407 | 758426 | 185697 | 764641 | 189815 | 752210 | 183199 |
| HILIC_POS_256_10974a0_626 | HILIC | POS | 0.626 | 256.1097 | | | | 1.20E-01 | 0.25 | 0.61 | 7.40E-01 | -0.07 | 0.40 | 1.54E-01 | 0.21 | 0.90 | 2.17E-01 | 0.29 | 0.59 | 11772580 | 5267325 | 13771030 | 8692672 | 14061965 | 11214565 | 13480096 | 5169911 |
| RP_NEG_289_70337a15_584 | RP | NEG | 15.58 | 289.7034 | | | | 2.24E-01 | -0.24 | 0.72 | 7.40E-01 | -0.07 | 0.20 | 2.01E-01 | -0.27 | 0.58 | 3.70E-01 | -0.21 | 0.58 | 838362 | 38329 | 828619 | 41172 | 829996 | 44826 | 827243 | 37571 |
| RP_NEG_622_84503a0_497 | RP | NEG | 0.50 | 622.8450 | | | | 3.94E-08 | -0.67 | 1.36 | 7.40E-01 | 0.07 | 1.19 | 1.19E-04 | -0.64 | 1.44 | 6.92E-05 | -0.71 | 1.45 | 2106209 | 143689 | 1584318 | 848819 | 1555989 | 882840 | 1612647 | 821370 |

**Supplementary table 1: Metabolomics results** (in asceding order according to p-value from progressive ALD vs  non-progressive ALD comparison, with molecular features with ID_level of 1-2 first)

| | | | | | | | | all_ALD_vs_control | | | Prog_ALD_vs_non-prog_ALD | | | Prog_ALD_vs_control_ALD | | | Non-prog_ALD_vs_control | | | Healthy_control | | all_ALD | | Non-prog_ALD | | Progressive_ALD | |
|---|---|---|---|---|---|---|---|---|---|---|---|---|---|---|---|---|---|---|---|---|---|---|---|---|---|---|---|
| | | | | | | | | t-test | Cohen's | PLS-DA | t-test | Cohen's | PLS-DA | t-test | Cohen's | PLS-DA | t-test | Cohen's | PLS-DA | | | | | | | | |
| Feature_ID | RP_HILIC | POS_NEG | Average.Rt | Average.Mz | Metabolite.name | curated_MSMS | ID_level | p | d | VIP | p | d | VIP | p | d | VIP | p | d | VIP | Mean | SD | Mean | SD | Mean | SD | Mean | SD |
| RP_POS_225_14679a9_052 | RP | POS | 9.05 | 225.1468 | | | | 2.14E-01 | -0.25 | 0.61 | 7.40E-01 | -0.07 | 0.35 | 2.04E-01 | -0.29 | 0.75 | 3.38E-01 | -0.22 | 0.58 | 1368855 | 188375 | 1320405 | 193278 | 1326856 | 198371 | 1313955 | 189840 |
| RP_NEG_408_78726a15_5 | RP | NEG | 15.50 | 408.7873 | | | | 1.59E-01 | 0.33 | 0.75 | 7.41E-01 | -0.06 | 0.32 | 2.44E-01 | 0.30 | 0.64 | 1.42E-01 | 0.36 | 0.60 | 2144819 | 79583 | 2166832 | 61530 | 2168884 | 55911 | 2164779 | 67190 |
| RP_NEG_605_15643a0_621 | RP | NEG | 0.62 | 605.1564 | | | | 7.75E-04 | -0.55 | 1.09 | 7.41E-01 | -0.07 | 1.01 | 2.27E-03 | -0.58 | 1.20 | 1.02E-02 | -0.51 | 1.10 | 6877914 | 1912199 | 5284977 | 3072991 | 5387413 | 3213285 | 5182540 | 2955107 |
| RP_POS_710_84906a0_49 | RP | POS | 0.49 | 710.8491 | | | | 1.10E-07 | 0.64 | 1.35 | 7.41E-01 | -0.07 | 1.20 | 2.29E-04 | 0.60 | 1.42 | 1.50E-04 | 0.67 | 1.44 | 650267 | 206004 | 2288330 | 2843767 | 2383108 | 2972970 | 2193553 | 2735358 |
| RP_NEG_238_07695a8_055 | RP | NEG | 8.06 | 238.0770 | | | | 2.01E-01 | -0.45 | 0.79 | 7.41E-01 | 0.02 | 0.48 | 2.16E-01 | -0.43 | 0.59 | 1.91E-01 | -0.46 | 0.73 | 3737043 | 9379165 | 1563181 | 1670317 | 1507570 | 1398781 | 1618793 | 1916756 |
| HILIC_NEG_270_00494a0_93 | HILIC | NEG | 0.93 | 270.0049 | | | | 5.01E-09 | 0.82 | 1.32 | 7.41E-01 | -0.07 | 1.18 | 3.76E-05 | 0.78 | 1.32 | 6.74E-07 | 0.85 | 1.58 | 589245 | 363635 | 1274456 | 878041 | 1303682 | 809873 | 1245230 | 948687 |
| HILIC_POS_160_09677a4_594 | HILIC | POS | 4.594 | 160.0968 | | | | 4.98E-01 | 0.12 | 0.31 | 7.41E-01 | 0.07 | 0.76 | 4.23E-01 | 0.16 | 0.34 | 6.97E-01 | 0.09 | 0.36 | 2986890 | 6356738 | 3939619 | 8312810 | 3662921 | 9281385 | 4216317 | 7301747 |
| HILIC_NEG_100_93345a7_941 | HILIC | NEG | 7.941 | 100.9335 | | | | 1.39E-02 | -0.49 | 0.85 | 7.41E-01 | -0.07 | 0.82 | 2.03E-02 | -0.52 | 0.89 | 3.78E-02 | -0.45 | 0.82 | 44668125 | 9594274 | 39623831 | 10349817 | 39968252 | 10117008 | 39279410 | 10669000 |
| RP_POS_332_24304a6_293 | RP | POS | 6.29 | 332.2430 | | | | 5.63E-01 | 0.11 | 0.91 | 7.41E-01 | -0.07 | 0.80 | 7.40E-01 | 0.07 | 0.59 | 4.92E-01 | 0.14 | 0.96 | 2742355 | 1329667 | 2908006 | 1603989 | 2961361 | 1504725 | 2854652 | 1711157 |
| HILIC_NEG_197_80791a2_952 | HILIC | NEG | 2.952 | 197.8079 | | | | 8.78E-05 | 0.52 | 1.21 | 7.41E-01 | -0.07 | 0.78 | 4.29E-03 | 0.48 | 1.17 | 2.62E-03 | 0.55 | 1.36 | 21172374 | 4150375 | 27127685 | 12746727 | 27551674 | 13422828 | 26703696 | 12154621 |
| HILIC_POS_193_09491a0_544 | HILIC | POS | 0.544 | 193.0949 | | | | 2.32E-01 | 0.15 | 0.73 | 7.41E-01 | 0.08 | 0.40 | 3.11E-01 | 0.19 | 0.55 | 4.77E-01 | 0.11 | 0.44 | 291381 | 1170676 | 939012 | 4982672 | 773367 | 4536728 | 1104657 | 5433490 |
| HILIC_NEG_132_92345a4_723 | HILIC | NEG | 4.723 | 132.9235 | | | | 1.64E-02 | 0.42 | 0.81 | 7.41E-01 | 0.07 | 0.94 | 3.99E-02 | 0.46 | 0.70 | 3.68E-02 | 0.39 | 0.77 | 2333718 | 575374 | 2648369 | 776087 | 2622569 | 635759 | 2674168 | 900758 |
| RP_POS_406_23849a11_162 | RP | POS | 11.16 | 406.2385 | | | | 6.57E-01 | 0.08 | 0.76 | 7.42E-01 | -0.07 | 0.74 | 8.20E-01 | 0.05 | 0.64 | 5.96E-01 | 0.12 | 0.77 | 8675611 | 509997 | 8725006 | 641528 | 8746294 | 690292 | 8703719 | 595052 |
| RP_NEG_367_24722a10_962 | RP | NEG | 10.96 | 367.2472 | | | | 2.01E-04 | -0.58 | 1.24 | 7.42E-01 | 0.07 | 0.80 | 2.35E-03 | -0.55 | 1.25 | 2.22E-03 | -0.62 | 1.15 | 3210471 | 364169 | 2858018 | 644471 | 2836640 | 700507 | 2879397 | 589507 |
| RP_NEG_832_79498a0_495 | RP | NEG | 0.50 | 832.7950 | | | | 1.42E-07 | -0.65 | 1.34 | 7.42E-01 | 0.07 | 1.18 | 3.30E-04 | -0.62 | 1.40 | 6.85E-05 | -0.69 | 1.45 | 1920898 | 157166 | 1480287 | 737537 | 1455830 | 738427 | 1504744 | 743323 |
| RP_POS_310_08942a6_862 | RP | POS | 6.86 | 310.0894 | | | | 6.92E-02 | -0.35 | 0.74 | 7.42E-01 | 0.07 | 0.60 | 1.27E-01 | -0.32 | 0.74 | 9.17E-02 | -0.39 | 0.81 | 1952618 | 181141 | 1882389 | 203244 | 1875652 | 224137 | 1889126 | 182015 |
| RP_POS_271_22598a9_193 | RP | POS | 9.19 | 271.2260 | | | | 3.41E-01 | -0.19 | 0.51 | 7.42E-01 | 0.07 | 0.77 | 4.79E-01 | -0.16 | 0.37 | 3.17E-01 | -0.23 | 0.61 | 2999506 | 311528 | 2938565 | 313603 | 2928172 | 313403 | 2948958 | 316636 |
| RP_POS_474_2847a8_659 | RP | POS | 8.66 | 474.2847 | | | | 2.15E-02 | 0.35 | 0.75 | 7.42E-01 | -0.07 | 0.73 | 1.20E-01 | 0.31 | 0.67 | 2.78E-02 | 0.38 | 0.85 | 480404 | 216820 | 612036 | 413230 | 625730 | 369659 | 598341 | 456040 |
| HILIC_NEG_136_89165a5_388 | HILIC | NEG | 5.388 | 136.8917 | | | | 7.95E-01 | 0.05 | 0.90 | 7.43E-01 | 0.07 | 0.31 | 7.05E-01 | 0.09 | 0.61 | 9.32E-01 | 0.02 | 0.31 | 4328242 | 1775842 | 4422996 | 1812095 | 4363049 | 1808608 | 4482944 | 1831936 |
| HILIC_POS_59_06127a0_684 | HILIC | POS | 0.684 | 59.0613 | | | | 1.04E-02 | -0.62 | 1.02 | 7.43E-01 | -0.06 | 0.91 | 1.49E-02 | -0.65 | 0.96 | 1.57E-02 | -0.60 | 1.28 | 2774567 | 563550 | 2486195 | 403127 | 2499536 | 319711 | 2472854 | 475143 |
| RP_NEG_334_91953a0_497 | RP | NEG | 0.50 | 334.9195 | | | | 1.70E-06 | -0.59 | 1.30 | 7.43E-01 | 0.07 | 1.17 | 5.51E-04 | -0.55 | 1.35 | 6.16E-04 | -0.63 | 1.39 | 1560936 | 121617 | 1225212 | 630649 | 1204355 | 676188 | 1246069 | 587737 |
| HILIC_NEG_134_89478a2_953 | HILIC | NEG | 2.953 | 134.8948 | | | | 1.09E-02 | 0.39 | 1.10 | 7.43E-01 | -0.07 | 0.80 | 5.30E-02 | 0.35 | 0.84 | 2.93E-02 | 0.42 | 1.11 | 165544034 | 25840824 | 182757791 | 48053990 | 184346902 | 50417307 | 181168680 | 46026511 |
| RP_POS_1051_79895a14_269 | RP | POS | 14.27 | 1051.7990 | | | | 5.22E-01 | 0.10 | 0.29 | 7.43E-01 | 0.07 | 0.21 | 5.50E-01 | 0.13 | 0.33 | 6.79E-01 | 0.06 | 0.19 | 199456688 | 19203251 | 202662783 | 36623278 | 201450740 | 23987330 | 203874827 | 46168298 |
| RP_POS_724_54303a12_325 | RP | POS | 12.33 | 724.5430 | | | | 1.59E-05 | -0.95 | 1.62 | 7.43E-01 | -0.06 | 0.37 | 3.74E-05 | -0.98 | 1.57 | 6.33E-05 | -0.92 | 1.57 | 7495296 | 1846240 | 5741045 | 1639390 | 5795249 | 1564058 | 5686842 | 1725623 |
| HILIC_POS_129_10237a0_792 | HILIC | POS | 0.792 | 129.1024 | | | | 1.64E-04 | -0.57 | 1.32 | 7.43E-01 | 0.07 | 0.79 | 3.48E-03 | -0.53 | 1.40 | 1.71E-03 | -0.60 | 1.35 | 2810019 | 682120 | 2108898 | 1325887 | 2065063 | 1377056 | 2152732 | 1285157 |
| RP_NEG_294_8855a0_446 | RP | NEG | 0.45 | 294.8855 | | | | 4.61E-07 | -0.64 | 1.32 | 7.43E-01 | 0.07 | 1.18 | 3.08E-04 | -0.61 | 1.38 | 1.68E-04 | -0.68 | 1.41 | 738637 | 92191 | 536283 | 344135 | 524921 | 357750 | 547646 | 333205 |
| HILIC_NEG_202_96536a0_93 | HILIC | NEG | 0.93 | 202.9654 | | | | 5.00E-09 | 0.85 | 1.38 | 7.44E-01 | -0.07 | 1.24 | 3.15E-05 | 0.81 | 1.34 | 2.76E-07 | 0.88 | 1.65 | 3717122 | 2140304 | 7521534 | 4671764 | 7675335 | 4185810 | 7367733 | 5150426 |
| HILIC_NEG_130_95763a2_912 | HILIC | NEG | 2.912 | 130.9576 | | | | 1.06E-04 | -0.58 | 1.33 | 7.44E-01 | 0.07 | 0.80 | 2.46E-03 | -0.54 | 1.37 | 1.43E-03 | -0.62 | 1.35 | 13323078 | 2498775 | 10657062 | 4927177 | 10494952 | 5171849 | 10819172 | 4716773 |
| RP_POS_955_57971a10_339 | RP | POS | 10.34 | 955.5797 | | | | 4.77E-03 | 0.43 | 1.05 | 7.44E-01 | 0.07 | 0.52 | 3.21E-02 | 0.47 | 0.86 | 1.03E-02 | 0.40 | 1.14 | 371900 | 268941 | 570403 | 496488 | 554059 | 356286 | 586748 | 608724 |
| RP_POS_221_98213a1_199 | RP | POS | 1.20 | 221.9821 | | | | 2.29E-12 | 0.92 | 1.46 | 7.44E-01 | 0.07 | 0.28 | 1.61E-06 | 0.96 | 1.48 | 6.67E-09 | 0.89 | 1.69 | 2029890 | 708290 | 4019504 | 2240308 | 3945860 | 1845262 | 4093149 | 2593131 |
| RP_POS_568_89178a0_496 | RP | POS | 0.50 | 568.8918 | | | | 5.04E-07 | 0.61 | 1.32 | 7.44E-01 | -0.07 | 1.15 | 4.28E-04 | 0.57 | 1.39 | 3.21E-04 | 0.64 | 1.41 | 180300 | 73362 | 497987 | 579905 | 517042 | 610318 | 478931 | 553365 |
| RP_POS_359_315a11_264 | RP | POS | 11.26 | 359.3150 | | | | 5.61E-01 | 0.10 | 0.36 | 7.44E-01 | 0.07 | 0.36 | 5.22E-01 | 0.14 | 0.45 | 7.38E-01 | 0.07 | 0.44 | 943178 | 945533 | 1067691 | 1322566 | 1024235 | 1229126 | 1111148 | 1421076 |
| HILIC_NEG_270_79047a2_937 | HILIC | NEG | 2.937 | 270.7905 | | | | 1.13E-04 | -0.58 | 1.33 | 7.45E-01 | 0.07 | 0.78 | 2.59E-03 | -0.54 | 1.39 | 1.54E-03 | -0.61 | 1.37 | 284431 | 63768 | 216410 | 126765 | 212249 | 133225 | 220571 | 121172 |
| RP_POS_236_07108a6_993 | RP | POS | 6.99 | 236.0711 | | | | 2.88E-03 | -0.51 | 1.08 | 7.45E-01 | 0.07 | 0.84 | 1.28E-02 | -0.47 | 0.97 | 9.90E-03 | -0.54 | 1.28 | 2319338 | 108305 | 2243053 | 157059 | 2237899 | 170794 | 2248207 | 143575 |
| RP_NEG_249_15019a10_291 | RP | NEG | 10.29 | 249.1502 | | | | 7.50E-01 | 0.07 | 0.64 | 7.45E-01 | 0.07 | 0.65 | 6.76E-01 | 0.10 | 0.57 | 8.83E-01 | 0.03 | 0.59 | 1371715 | 105708 | 1378594 | 105557 | 1375133 | 97508 | 1382056 | 113928 |
| HILIC_NEG_173_92603a6_275 | HILIC | NEG | 6.275 | 173.9260 | | | | 7.85E-01 | 0.06 | 0.16 | 7.45E-01 | -0.07 | 0.52 | 9.16E-01 | 0.02 | 0.11 | 6.97E-01 | 0.09 | 0.28 | 8108044 | 2764529 | 8260689 | 2642588 | 8347313 | 2589329 | 8174066 | 2718293 |
| RP_POS_350_98758a8_122 | RP | POS | 8.12 | 350.9876 | | | | 1.43E-01 | -0.31 | 0.81 | 7.45E-01 | -0.06 | 0.81 | 1.42E-01 | -0.34 | 0.70 | 2.36E-01 | -0.28 | 0.53 | 1729033 | 538893 | 1567869 | 515891 | 1584773 | 524321 | 1550964 | 512077 |
| RP_POS_296_22165a9_141 | RP | POS | 9.14 | 296.2217 | | | | 9.78E-04 | -0.71 | 1.17 | 7.45E-01 | 0.06 | 0.89 | 4.02E-03 | -0.67 | 1.11 | 1.32E-03 | -0.74 | 1.23 | 2774571 | 158782 | 2663288 | 148273 | 2658431 | 141693 | 2668145 | 155863 |
| HILIC_NEG_118_92398a4_811 | HILIC | NEG | 4.811 | 118.9240 | | | | 6.43E-01 | 0.09 | 0.52 | 7.45E-01 | 0.07 | 0.65 | 5.93E-01 | 0.13 | 0.30 | 7.77E-01 | 0.06 | 0.30 | 2348100 | 785866 | 2422905 | 802780 | 2396606 | 698891 | 2449205 | 901223 |
| HILIC_POS_540_33026a0_99 | HILIC | POS | 0.99 | 540.3303 | | | | 5.40E-05 | 0.50 | 1.21 | 7.45E-01 | -0.07 | 0.76 | 6.40E-03 | 0.46 | 1.35 | 1.92E-03 | 0.54 | 1.33 | 294807 | 910088 | 2189771 | 4231304 | 2328344 | 4267321 | 2051198 | 4233679 |
| RP_NEG_288_93591a15_331 | RP | NEG | 15.33 | 288.9359 | | | | 6.68E-01 | -0.08 | 0.65 | 7.45E-01 | -0.07 | 0.39 | 5.87E-01 | -0.12 | 0.66 | 8.25E-01 | -0.05 | 0.40 | 3832710 | 116206 | 3822302 | 127397 | 3826474 | 134793 | 3818130 | 120775 |
| RP_POS_151_00519a10_666 | RP | POS | 10.67 | 151.0052 | | | | 2.36E-01 | 0.24 | 0.54 | 7.45E-01 | 0.07 | 0.45 | 2.25E-01 | 0.27 | 0.51 | 3.67E-01 | 0.20 | 0.65 | 1939674 | 606708 | 2089017 | 635766 | 2068209 | 653178 | 2109826 | 623795 |
| HILIC_NEG_94_92467a3_017 | HILIC | NEG | 3.017 | 94.9247 | | | | 8.30E-01 | -0.04 | 0.73 | 7.45E-01 | 0.07 | 0.63 | 9.61E-01 | -0.01 | 0.33 | 7.37E-01 | -0.08 | 0.61 | 741962 | 236065 | 731608 | 236726 | 723860 | 238443 | 739356 | 237160 |
| RP_NEG_554_85638a0_497 | RP | NEG | 0.50 | 554.8564 | | | | 5.05E-07 | -0.61 | 1.32 | 7.45E-01 | 0.07 | 1.19 | 3.64E-04 | -0.58 | 1.40 | 3.07E-04 | -0.65 | 1.41 | 2055890 | 153860 | 1555094 | 900073 | 1525640 | 951305 | 1584548 | 854425 |
| RP_POS_433_28128a12_127 | RP | POS | 12.13 | 433.2813 | | | | 7.44E-02 | -0.32 | 0.99 | 7.45E-01 | 0.07 | 0.86 | 1.42E-01 | -0.29 | 0.89 | 1.06E-01 | -0.36 | 0.90 | 1051056 | 192528 | 974889 | 246410 | 966826 | 273928 | 982952 | 217958 |
| RP_POS_166_98492a15_398 | RP | POS | 15.40 | 166.9849 | | | | 5.59E-01 | -0.12 | 0.68 | 7.45E-01 | 0.07 | 0.23 | 7.02E-01 | -0.08 | 0.52 | 5.12E-01 | -0.15 | 0.42 | 105686192 | 38102296 | 101108992 | 39211940 | 99826678 | 41107638 | 102391305 | 37594385 |
| RP_NEG_291_89355a3_965 | RP | NEG | 3.97 | 291.8936 | | | | 3.49E-01 | 0.16 | 0.87 | 7.46E-01 | -0.07 | 0.29 | 5.75E-01 | 0.12 | 0.61 | 2.92E-01 | 0.19 | 0.50 | 1786873 | 625245 | 1924393 | 951418 | 1955508 | 806147 | 1893278 | 1084904 |
| RP_NEG_369_00574a0_678 | RP | NEG | 0.68 | 369.0057 | | | | 3.59E-09 | -0.72 | 1.40 | 7.46E-01 | 0.07 | 1.18 | 5.31E-05 | -0.68 | 1.48 | 1.66E-05 | -0.75 | 1.50 | 4615379 | 263414 | 3285973 | 2016434 | 3220047 | 2050657 | 3351898 | 2000232 |
| HILIC_NEG_132_90038a7_673 | HILIC | NEG | 7.673 | 132.9004 | | | | 4.29E-02 | -0.38 | 0.76 | 7.46E-01 | -0.07 | 0.38 | 4.18E-02 | -0.42 | 0.90 | 1.20E-01 | -0.35 | 0.56 | 1597849 | 296072 | 1468076 | 345876 | 1479362 | 383723 | 1456790 | 306918 |
| RP_POS_979_57941a10_332 | RP | POS | 10.33 | 979.5794 | | | | 4.69E-08 | 0.72 | 1.31 | 7.46E-01 | 0.07 | 0.31 | 6.61E-05 | 0.76 | 1.36 | 2.80E-05 | 0.69 | 1.27 | 208760 | 119977 | 454983 | 367396 | 443012 | 336095 | 466954 | 399327 |
| HILIC_POS_175_10776a4_792 | HILIC | POS | 4.792 | 175.1078 | | | | 1.09E-01 | 0.28 | 0.76 | 7.47E-01 | 0.07 | 0.62 | 1.07E-01 | 0.31 | 0.65 | 2.51E-01 | 0.25 | 0.41 | 958993 | 182367 | 1025189 | 249807 | 1017059 | 271959 | 1033318 | 228001 |
| RP_POS_341_30447a11_264 | RP | POS | 11.26 | 341.3045 | | | | 9.36E-01 | 0.01 | 0.34 | 7.47E-01 | 0.07 | 0.40 | 8.23E-01 | 0.05 | 0.41 | 9.24E-01 | -0.02 | 0.45 | 401205 | 401927 | 408232 | 500271 | 391971 | 464916 | 424494 | 537554 |
| RP_POS_285_06412a15_342 | RP | POS | 15.34 | 285.0641 | | | | 8.34E-01 | 0.04 | 0.36 | 7.47E-01 | 0.07 | 0.27 | 7.38E-01 | 0.08 | 0.37 | 9.66E-01 | 0.01 | 0.32 | 1937354 | 533773 | 1960258 | 542338 | 1942638 | 555573 | 1977877 | 533825 |
| HILIC_POS_375_3212a1_017 | HILIC | POS | 1.017 | 375.3212 | | | | 3.31E-01 | -0.17 | 0.32 | 7.47E-01 | -0.07 | 0.26 | 3.22E-01 | -0.20 | 0.39 | 5.05E-01 | -0.14 | 0.24 | 4282236 | 1098677 | 4042194 | 1494125 | 4090656 | 1482771 | 3993732 | 1518872 |
| RP_POS_160_13335a4_294 | RP | POS | 4.29 | 160.1334 | Unknown | 160.1337(5), 114.1282(100) | 4 | 4.03E-01 | -0.19 | 0.36 | 7.48E-01 | 0.06 | 0.20 | 5.14E-01 | -0.16 | 0.35 | 3.73E-01 | -0.22 | 0.36 | 2372772 | 1832644 | 2072220 | 1483377 | 2024147 | 1513695 | 2120293 | 1466197 |
| RP_POS_751_51599a10_655 | RP | POS | 10.66 | 751.5160 | | | | 3.02E-01 | 0.22 | 0.78 | 7.48E-01 | 0.06 | 0.29 | 2.82E-01 | 0.25 | 0.69 | 4.25E-01 | 0.19 | 0.71 | 4529116 | 386627 | 4609829 | 362467 | 4598092 | 367515 | 4621566 | 360691 |
| HILIC_NEG_119_90752a6_272 | HILIC | NEG | 6.272 | 119.9075 | | | | 2.50E-01 | -0.26 | 0.44 | 7.48E-01 | -0.06 | 0.45 | 2.28E-01 | -0.29 | 0.47 | 3.59E-01 | -0.23 | 0.45 | 1852964 | 822350 | 1665030 | 696315 | 1687568 | 736876 | 1642492 | 659985 |
| HILIC_POS_59_06131a0_934 | HILIC | POS | 0.934 | 59.0613 | | | | 2.69E-01 | -0.25 | 0.47 | 7.49E-01 | -0.06 | 0.69 | 2.51E-01 | -0.28 | 0.47 | 3.71E-01 | -0.22 | 0.35 | 3705000 | 1089321 | 3467314 | 894940 | 3496181 | 910621 | 3438447 | 887271 |
| RP_POS_244_86719a0_246 | RP | POS | 0.25 | 244.8672 | | | | 9.19E-01 | 0.02 | 0.40 | 7.49E-01 | 0.07 | 0.65 | 8.04E-01 | 0.05 | 0.36 | 9.48E-01 | -0.01 | 0.48 | 409356 | 60257 | 410664 | 70686 | 408384 | 73451 | 412944 | 68479 |
| HILIC_NEG_179_8828a7_667 | HILIC | NEG | 7.667 | 179.8828 | | | | 9.45E-02 | 0.28 | 0.78 | 7.49E-01 | 0.07 | 0.79 | 1.12E-01 | 0.32 | 0.61 | 2.19E-01 | 0.25 | 0.49 | 1176403 | 57101 | 1198660 | 84237 | 1195945 | 85624 | 1201375 | 83607 |
| HILIC_POS_292_84528a2_991 | HILIC | POS | 2.991 | 292.8453 | | | | 6.91E-06 | -0.58 | 1.35 | 7.49E-01 | 0.07 | 0.78 | 1.01E-03 | -0.54 | 1.47 | 8.35E-04 | -0.61 | 1.50 | 20010893 | 2373848 | 15637446 | 8332125 | 15368993 | 8839799 | 15905900 | 7872504 |
| RP_POS_369_38422a11_247 | RP | POS | 11.25 | 369.3842 | | | | 5.95E-10 | 0.89 | 1.43 | 7.49E-01 | 0.07 | 0.96 | 1.73E-05 | 0.92 | 1.42 | 4.65E-09 | 0.85 | 1.79 | 486658 | 222687 | 917877 | 502992 | 901682 | 349144 | 934071 | 623480 |
| HILIC_NEG_157_90068a1_328 | HILIC | NEG | 1.328 | 157.9007 | | | | 7.16E-04 | -0.55 | 0.97 | 7.50E-01 | -0.07 | 0.55 | 2.71E-03 | -0.58 | 1.00 | 8.28E-03 | -0.52 | 0.88 | 866494 | 93498 | 787825 | 151379 | 792693 | 153236 | 782956 | 150894 |
| HILIC_POS_666_33441a0_855 | HILIC | POS | 0.855 | 666.3344 | | | | 8.69E-01 | 0.03 | 0.37 | 7.50E-01 | 0.06 | 0.36 | 7.47E-01 | 0.07 | 0.36 | 9.93E-01 | 0.00 | 0.29 | 4931198 | 2827253 | 5025523 | 2738408 | 4937509 | 3431430 | 5113537 | 1833105 |
| HILIC_POS_130_0977a6_968 | HILIC | POS | 6.968 | 130.0977 | | | | 8.44E-02 | 0.23 | 1.07 | 7.50E-01 | -0.07 | 0.76 | 2.64E-01 | 0.20 | 0.95 | 1.29E-01 | 0.27 | 1.14 | 530243 | 165169 | 622153 | 440327 | 636290 | 442482 | 608017 | 442192 |
| RP_NEG_643_11218a0_621 | RP | NEG | 0.62 | 643.1122 | | | | 7.18E-06 | -0.66 | 1.27 | 7.50E-01 | -0.07 | 1.11 | 7.73E-05 | -0.70 | 1.41 | 1.19E-03 | -0.63 | 1.26 | 1092188 | 230648 | 797568 | 473364 | 812755 | 509908 | 782380 | 438455 |
| HILIC_NEG_342_72574a2_938 | HILIC | NEG | 2.938 | 342.7257 | | | | 2.75E-07 | -0.67 | 1.35 | 7.50E-01 | 0.07 | 0.77 | 1.38E-04 | -0.63 | 1.49 | 1.43E-04 | -0.70 | 1.49 | 403637 | 50365 | 301401 | 166130 | 296073 | 176468 | 306729 | 156725 |
| HILIC_NEG_315_04199a5_607 | HILIC | NEG | 5.607 | 315.0420 | | | | 2.30E-01 | -0.25 | 0.81 | 7.50E-01 | -0.06 | 0.67 | 2.35E-01 | -0.28 | 0.47 | 3.34E-01 | -0.22 | 0.61 | 6607011 | 1151307 | 6323398 | 1143155 | 6360050 | 1067955 | 6286746 | 1223522 |
| RP_NEG_268_98413a8_316 | RP | NEG | 8.32 | 268.9841 | | | | 4.67E-02 | -0.41 | 0.85 | 7.50E-01 | 0.06 | 0.39 | 9.58E-02 | -0.38 | 0.66 | 5.30E-02 | -0.44 | 0.78 | 3517898 | 147440 | 3457136 | 144811 | 3452496 | 145374 | 3461777 | 145572 |
| RP_POS_644_49445a10_46 | RP | POS | 10.46 | 644.4945 | | | | 3.93E-01 | -0.22 | 0.49 | 7.50E-01 | 0.05 | 0.69 | 4.76E-01 | -0.20 | 0.37 | 3.60E-01 | -0.25 | 0.52 | 2899895 | 463188 | 2825131 | 279885 | 2816165 | 273193 | 2834097 | 288914 |
| RP_POS_169_9856a0_522 | RP | POS | 0.52 | 169.9856 | | | | 1.09E-06 | 0.59 | 1.22 | 7.51E-01 | 0.07 | 1.12 | 4.34E-04 | 0.62 | 1.32 | 7.43E-04 | 0.55 | 1.27 | 295900 | 65601 | 598248 | 572924 | 579898 | 553487 | 616597 | 596780 |
| RP_POS_147_01398a0_45 | RP | POS | 0.45 | 147.0140 | | | | 2.43E-06 | -0.63 | 1.30 | 7.51E-01 | -0.07 | 1.13 | 1.28E-04 | -0.67 | 1.37 | 1.00E-03 | -0.60 | 1.35 | 598569 | 86435 | 455655 | 246007 | 463530 | 255148 | 447781 | 238845 |
| RP_POS_316_13089a6_074 | RP | POS | 6.07 | 316.1309 | | | | 6.41E-01 | 0.09 | 0.40 | 7.51E-01 | 0.07 | 0.54 | 5.65E-01 | 0.12 | 0.47 | 8.05E-01 | 0.05 | 0.62 | 584786 | 135972 | 598323 | 160467 | 593191 | 169224 | 603455 | 152756 |
| RP_POS_1050_78564a0_493 | RP | POS | 0.49 | 1050.7856 | | | | 1.65E-08 | 0.68 | 1.39 | 7.51E-01 | -0.07 | 1.19 | 1.45E-04 | 0.64 | 1.46 | 3.67E-05 | 0.71 | 1.49 | 37438 | 29279 | 586083 | 891240 | 614580 | 898476 | 557586 | 892139 |
| RP_NEG_381_19635a11_611 | RP | NEG | 11.61 | 381.1964 | | | | 2.45E-01 | -0.22 | 0.54 | 7.51E-01 | -0.07 | 0.91 | 2.49E-01 | -0.25 | 0.62 | 3.85E-01 | -0.18 | 0.45 | 1099176 | 139144 | 1064116 | 169157 | 1069524 | 165032 | 1058708 | 174692 |
| RP_NEG_265_14908a11_966 | RP | NEG | 11.97 | 265.1491 | | | | 1.82E-01 | 0.20 | 0.39 | 7.51E-01 | -0.07 | 0.44 | 3.74E-01 | 0.16 | 0.32 | 2.16E-01 | 0.23 | 0.54 | 257020 | 137264 | 305865 | 271031 | 314514 | 276742 | 297216 | 267725 |
| RP_POS_564_82123a6_221 | RP | POS | 6.22 | 564.8212 | | | | 1.09E-05 | 0.53 | 1.25 | 7.52E-01 | -0.07 | 1.09 | 3.29E-03 | 0.50 | 1.32 | 9.02E-04 | 0.57 | 1.31 | 80288 | 344821 | 1150265 | 2241755 | 1221756 | 2252513 | 1078774 | 2251498 |
| HILIC_POS_90_97712a5_463 | HILIC | POS | 5.463 | 90.9771 | | | | 2.53E-01 | -0.24 | 0.40 | 7.52E-01 | 0.06 | 0.55 | 3.74E-01 | -0.21 | 0.35 | 2.32E-01 | -0.28 | 0.47 | 1365399 | 111163 | 1339803 | 102223 | 1336546 | 96178 | 1343061 | 108814 |
| RP_NEG_318_98199a9_425 | RP | NEG | 9.43 | 318.9820 | | | | 2.72E-01 | 0.17 | 0.63 | 7.52E-01 | -0.07 | 0.31 | 4.46E-01 | 0.14 | 0.57 | 3.05E-01 | 0.21 | 0.75 | 2854597 | 839193 | 3081626 | 1423629 | 3126964 | 1543413 | 3036288 | 1307089 |
| RP_POS_514_97168a6_842 | RP | POS | 6.84 | 514.9717 | | | | 2.99E-07 | 0.61 | 1.32 | 7.52E-01 | -0.07 | 1.12 | 5.15E-04 | 0.58 | 1.41 | 1.99E-04 | 0.65 | 1.41 | 22732 | 33010 | 679656 | 1193579 | 717586 | 1221130 | 641725 | 1176538 |
| RP_POS_226_21646a8_727 | RP | POS | 8.73 | 226.2165 | | | | 6.46E-05 | 0.47 | 0.96 | 7.52E-01 | -0.07 | 0.84 | 1.99E-03 | 0.43 | 1.09 | 8.02E-03 | 0.51 | 1.07 | 53368 | 67941 | 1042654 | 2368095 | 1117908 | 2722000 | 967400 | 1977209 |
| RP_POS_798_56305a11_8 | RP | POS | 11.80 | 798.5631 | | | | 2.70E-01 | 0.16 | 0.55 | 7.53E-01 | -0.07 | 0.45 | 4.98E-01 | 0.13 | 0.35 | 2.79E-01 | 0.20 | 0.64 | 1366940 | 1854506 | 1920470 | 3764633 | 2039752 | 3695625 | 1801188 | 3866197 |
| RP_POS_487_27258a6_73 | RP | POS | 6.73 | 487.2726 | | | | 2.58E-07 | 0.61 | 1.32 | 7.54E-01 | -0.07 | 1.13 | 4.06E-04 | 0.58 | 1.42 | 2.16E-04 | 0.65 | 1.39 | 25462 | 58478 | 925083 | 1623729 | 976422 | 1681666 | 873743 | 1579055 |
| RP_POS_814_56927a13_509 | RP | POS | 13.51 | 814.5693 | | | | 7.59E-01 | -0.06 | 0.51 | 7.54E-01 | 0.07 | 0.70 | 9.14E-01 | -0.02 | 0.46 | 6.77E-01 | -0.09 | 0.47 | 5310910 | 1298252 | 5224073 | 1641032 | 5172189 | 1695739 | 5275957 | 1599971 |
| RP_POS_358_11896a3_317 | RP | POS | 3.32 | 358.1190 | | | | 6.33E-04 | -0.66 | 1.20 | 7.54E-01 | 0.06 | 1.03 | 3.05E-03 | -0.63 | 1.18 | 1.70E-03 | -0.69 | 1.19 | 1380257 | 598983 | 924656 | 687466 | 902924 | 717054 | 946388 | 663124 |
| RP_POS_527_77814a10_339 | RP | POS | 10.34 | 527.7781 | | | | 4.74E-02 | 0.35 | 0.81 | 7.54E-01 | -0.07 | 0.42 | 1.42E-01 | 0.32 | 0.74 | 4.76E-02 | 0.39 | 0.80 | 361023 | 164519 | 434185 | 216041 | 441013 | 191090 | 427357 | 240185 |
| RP_POS_314_77866a0_522 | RP | POS | 0.52 | 314.7787 | | | | 2.47E-06 | 0.57 | 1.26 | 7.54E-01 | -0.07 | 1.10 | 8.99E-04 | 0.54 | 1.27 | 7.13E-04 | 0.61 | 1.37 | 292900 | 62135 | 502547 | 407682 | 515419 | 430337 | 489676 | 387650 |
| RP_POS_522_35571a11_16 | RP | POS | 11.16 | 522.3557 | | | | 1.06E-01 | 0.31 | 0.94 | 7.55E-01 | -0.06 | 0.58 | 2.43E-01 | 0.28 | 0.76 | 7.95E-02 | 0.34 | 1.03 | 954544 | 440619 | 1105983 | 496197 | 1121620 | 366989 | 1090345 | 601887 |

**Supplementary table 1: Metabolomics results** (in asceding order according to p-value from progressive ALD vs non-progressive ALD comparison, with molecular features with ID_level of 1-2 first)

| Feature_ID | RP_HILIC | POS_NEG | Average.Rt | Average.Mz | Metabolite.name | curated_MSMS | ID_level | all_ALD_vs_control | | | Prog_ALD_vs_non-prog_ALD | | | Prog_ALD_vs_control_ALD | | | Non-prog_ALD_vs_control | | | Healthy_control | | all_ALD | | Non-prog_ALD | | Progressive_ALD | |
|---|---|---|---|---|---|---|---|---|---|---|---|---|---|---|---|---|---|---|---|---|---|---|---|---|---|---|---|
| | | | | | | | | t-test | Cohen's | PLS-DA | t-test | Cohen's | PLS-DA | t-test | Cohen's | PLS-DA | t-test | Cohen's | PLS-DA | | | | | | | | |
| | | | | | | | | p | d | VIP | p | d | VIP | p | d | VIP | p | d | VIP | Mean | SD | Mean | SD | Mean | SD | Mean | SD |
| HILIC_POS_375_32129a0_686 | HILIC | POS | 0.686 | 375.3213 | | | | 7.23E-02 | -0.37 | 0.74 | 7.55E-01 | -0.06 | 0.51 | 8.23E-02 | -0.40 | 0.64 | 1.34E-01 | -0.34 | 0.77 | 2956069 | 815355 | 2652345 | 815286 | 2677998 | 797957 | 2626692 | 839565 |
| RP_POS_627_43268a10_978 | RP | POS | 10.98 | 627.4327 | | | | 7.45E-03 | 0.44 | 1.29 | 7.55E-01 | -0.07 | 1.21 | 3.79E-02 | 0.41 | 1.12 | 1.64E-02 | 0.47 | 1.26 | 196195 | 123927 | 276343 | 193220 | 282419 | 194474 | 270267 | 193739 |
| RP_NEG_594_39551a11_163 | RP | NEG | 11.16 | 594.3955 | | | | 5.66E-03 | 0.48 | 1.16 | 7.55E-01 | -0.07 | 1.06 | 2.40E-02 | 0.44 | 1.09 | 1.39E-02 | 0.51 | 1.13 | 3287035 | 311724 | 3488273 | 441595 | 3502159 | 462564 | 3474387 | 423831 |
| HILIC_POS_170_11766a0_549 | HILIC | POS | 0.549 | 170.1177 | | | | 1.84E-01 | -0.30 | 0.74 | 7.55E-01 | -0.06 | 0.55 | 1.92E-01 | -0.33 | 0.66 | 2.56E-01 | -0.27 | 0.56 | 2807184 | 1738102 | 2348078 | 1467680 | 2394194 | 1317273 | 2301963 | 1616349 |
| RP_POS_278_24802a9_417 | RP | POS | 9.42 | 278.2480 | | | | 9.43E-01 | -0.02 | 0.70 | 7.55E-01 | -0.06 | 0.32 | 8.54E-01 | -0.05 | 0.46 | 9.56E-01 | 0.01 | 0.41 | 2469329 | 1938970 | 2442083 | 1582636 | 2491757 | 1507429 | 2392408 | 1668296 |
| RP_POS_234_89365a0_515 | RP | POS | 0.52 | 234.8937 | | | | 3.63E-04 | -0.64 | 1.04 | 7.56E-01 | 0.06 | 0.66 | 4.21E-03 | -0.61 | 0.99 | 7.71E-04 | -0.67 | 1.18 | 689569 | 156456 | 560134 | 206008 | 553671 | 192073 | 566596 | 220833 |
| HILIC_POS_171_18549a7_15 | HILIC | POS | 7.15 | 171.1855 | | | | 3.64E-02 | 0.39 | 0.82 | 7.56E-01 | 0.06 | 0.67 | 6.44E-02 | 0.42 | 0.79 | 6.91E-02 | 0.36 | 0.75 | 4215169 | 1449674 | 4878956 | 1753109 | 4823968 | 1465328 | 4933944 | 2013989 |
| HILIC_NEG_144_92325a2_939 | HILIC | NEG | 2.939 | 144.9233 | | | | 6.67E-07 | 0.64 | 1.32 | 7.56E-01 | -0.07 | 0.77 | 1.39E-04 | 0.60 | 1.49 | 4.02E-04 | 0.67 | 1.44 | 11270424 | 843474 | 13039141 | 3035237 | 13134326 | 3347796 | 12943957 | 2717924 |
| RP_POS_608_84674a6_388 | RP | POS | 6.39 | 608.8467 | | | | 2.89E-06 | 0.56 | 1.27 | 7.56E-01 | -0.07 | 1.10 | 1.64E-03 | 0.53 | 1.36 | 5.45E-04 | 0.60 | 1.34 | 92326 | 324401 | 1613719 | 3023193 | 1708488 | 3069078 | 1518951 | 3004721 |
| RP_POS_385_23587a11_149 | RP | POS | 11.15 | 385.2359 | | | | 5.81E-01 | -0.08 | 1.01 | 7.56E-01 | 0.07 | 1.10 | 7.78E-01 | -0.05 | 0.91 | 5.58E-01 | -0.12 | 1.08 | 4597599 | 212759 | 4567163 | 401410 | 4554583 | 443497 | 4579744 | 358518 |
| RP_POS_738_50494a10_464 | RP | POS | 10.46 | 738.5049 | | | | 2.05E-01 | -0.28 | 0.93 | 7.56E-01 | 0.06 | 0.33 | 2.90E-01 | -0.25 | 0.60 | 2.03E-01 | -0.31 | 0.82 | 1212287 | 283052 | 1141014 | 239455 | 1133513 | 249897 | 1148516 | 230834 |
| RP_POS_155_15419a2_78 | RP | POS | 2.78 | 155.1542 | | | | 1.85E-02 | 0.36 | 0.84 | 7.56E-01 | -0.07 | 0.67 | 5.30E-02 | 0.33 | 0.87 | 5.59E-02 | 0.40 | 0.85 | 2920513 | 1090154 | 3580748 | 1971105 | 3642490 | 2248980 | 3519006 | 1668565 |
| RP_NEG_144_9989a15_302 | RP | NEG | 15.30 | 144.9989 | | | | 1.19E-01 | 0.28 | 0.65 | 7.56E-01 | 0.07 | 0.27 | 1.27E-01 | 0.32 | 0.64 | 2.39E-01 | 0.25 | 0.64 | 1347742 | 201107 | 1416812 | 254654 | 1408838 | 263671 | 1424786 | 247726 |
| RP_NEG_765_1684a9_804 | RP | NEG | 9.80 | 765.1684 | | | | 4.56E-01 | -0.16 | 0.51 | 7.56E-01 | 0.06 | 0.53 | 5.76E-01 | -0.13 | 0.31 | 4.22E-01 | -0.19 | 0.44 | 5349113 | 348428 | 5297453 | 304787 | 5287918 | 311863 | 5306989 | 300403 |
| RP_POS_256_26309a10_731 | RP | POS | 10.73 | 256.2631 | | | | 6.10E-06 | 0.71 | 1.19 | 7.56E-01 | -0.07 | 0.82 | 1.18E-03 | 0.68 | 1.16 | 1.48E-05 | 0.74 | 1.41 | 157953919 | 47916688 | 215405209 | 84813491 | 218057863 | 69911572 | 212752555 | 98140057 |
| RP_POS_532_84973a0_498 | RP | POS | 0.50 | 532.8497 | | | | 1.93E-07 | -0.63 | 1.34 | 7.56E-01 | 0.07 | 1.19 | 2.12E-04 | -0.60 | 1.43 | 2.16E-04 | -0.67 | 1.42 | 1998235 | 125934 | 1509420 | 851694 | 1482792 | 902491 | 1536049 | 805998 |
| RP_NEG_315_86319a15_786 | RP | NEG | 15.79 | 315.8632 | | | | 1.37E-01 | 0.27 | 0.58 | 7.57E-01 | -0.06 | 0.26 | 2.48E-01 | 0.24 | 0.54 | 1.52E-01 | 0.31 | 0.67 | 851108 | 394257 | 978968 | 483264 | 994060 | 496068 | 963876 | 474665 |
| HILIC_POS_282_81635a3_009 | HILIC | POS | 3.009 | 282.8164 | | | | 2.99E-05 | -0.56 | 1.35 | 7.57E-01 | 0.07 | 0.78 | 1.93E-03 | -0.53 | 1.46 | 1.26E-03 | -0.60 | 1.47 | 3722495 | 588210 | 2887417 | 1624651 | 2836733 | 1706717 | 2938100 | 1553938 |
| RP_POS_334_17252a10_979 | RP | POS | 10.98 | 334.1725 | | | | 3.63E-02 | 0.39 | 1.44 | 7.57E-01 | -0.06 | 1.24 | 1.06E-01 | 0.35 | 1.23 | 4.07E-02 | 0.42 | 1.30 | 1577660 | 384123 | 1754909 | 475351 | 1769735 | 441016 | 1740084 | 511454 |
| RP_POS_459_3075a10_705 | RP | POS | 10.71 | 459.3075 | | | | 2.81E-02 | -0.37 | 0.59 | 7.57E-01 | -0.07 | 0.40 | 4.37E-02 | -0.40 | 0.79 | 9.06E-02 | -0.34 | 0.55 | 3416985 | 723988 | 3044940 | 1061493 | 3078041 | 1067098 | 3011840 | 1065643 |
| RP_NEG_801_08136a9_224 | RP | NEG | 9.22 | 801.0814 | | | | 9.09E-01 | 0.02 | 0.75 | 7.57E-01 | 0.07 | 0.46 | 7.84E-01 | 0.05 | 0.68 | 9.41E-01 | -0.01 | 0.36 | 1327618 | 101419 | 1330345 | 157464 | 1325436 | 165985 | 1335254 | 149986 |
| RP_POS_523_37915a13_755 | RP | POS | 13.76 | 523.3792 | | | | 2.47E-03 | -0.58 | 1.00 | 7.57E-01 | -0.06 | 0.37 | 1.01E-02 | -0.61 | 0.90 | 4.12E-03 | -0.55 | 1.08 | 3234633 | 770161 | 2722048 | 878702 | 2749460 | 623316 | 2694636 | 1081636 |
| RP_POS_517_94098a6_841 | RP | POS | 6.84 | 517.9410 | | | | 1.64E-07 | 0.62 | 1.33 | 7.57E-01 | -0.07 | 1.15 | 3.20E-04 | 0.59 | 1.45 | 1.75E-04 | 0.66 | 1.39 | 16563 | 32355 | 962514 | 1677530 | 1014809 | 1737106 | 910218 | 1631725 |
| RP_POS_294_92075a15_626 | RP | POS | 15.63 | 294.9208 | | | | 1.87E-01 | 0.25 | 0.60 | 7.57E-01 | -0.06 | 0.20 | 3.19E-01 | 0.22 | 0.62 | 1.81E-01 | 0.29 | 0.64 | 11296584 | 2794139 | 12077592 | 3141914 | 12175441 | 2991733 | 11979744 | 3312809 |
| RP_POS_800_57812a11_832 | RP | POS | 11.83 | 800.5781 | | | | 7.75E-02 | 0.22 | 0.56 | 7.57E-01 | -0.07 | 0.38 | 2.54E-01 | 0.18 | 0.48 | 1.53E-01 | 0.26 | 0.59 | 1054908 | 1326270 | 2102850 | 5400985 | 2271045 | 5701853 | 1934654 | 5134952 |
| HILIC_POS_201_06108a4_868 | HILIC | POS | 4.868 | 201.0611 | | | | 9.20E-01 | 0.02 | 0.41 | 7.57E-01 | 0.06 | 0.27 | 8.01E-01 | 0.05 | 0.49 | 9.66E-01 | -0.01 | 0.15 | 341952 | 230540 | 346689 | 229398 | 339540 | 282124 | 353837 | 163164 |
| RP_NEG_174_95537a0_448 | RP | NEG | 0.45 | 174.9554 | | | | 4.71E-08 | -0.68 | 1.36 | 7.57E-01 | 0.07 | 1.19 | 1.39E-04 | -0.64 | 1.43 | 5.92E-05 | -0.71 | 1.46 | 27438693 | 2508913 | 19643510 | 12599847 | 19251288 | 12922115 | 20035731 | 12387784 |
| RP_NEG_598_85529a0_466 | RP | NEG | 0.47 | 598.8553 | | | | 1.06E-08 | -0.72 | 1.39 | 7.58E-01 | 0.07 | 1.20 | 5.20E-05 | -0.68 | 1.47 | 2.60E-05 | -0.75 | 1.47 | 877755 | 90865 | 614971 | 397082 | 602627 | 409165 | 627315 | 388379 |
| RP_POS_149_02388a7_493 | RP | POS | 7.49 | 149.0239 | Unknown | 149.0228(70), 121.0287(10), 84.9604(100) | 4 | 7.20E-01 | 0.06 | 0.64 | 7.58E-01 | 0.07 | 0.53 | 6.52E-01 | 0.10 | 0.35 | 8.78E-01 | 0.03 | 0.39 | 3928527 | 289577 | 3951511 | 381269 | 3939674 | 359837 | 3963348 | 404884 |
| HILIC_NEG_272_95865a7_347 | HILIC | NEG | 7.347 | 272.9587 | | | | 2.51E-03 | -0.57 | 1.15 | 7.58E-01 | -0.06 | 0.91 | 5.73E-03 | -0.61 | 0.98 | 9.62E-03 | -0.54 | 0.97 | 627305 | 168296 | 514746 | 197311 | 520871 | 189128 | 508621 | 206910 |
| RP_POS_201_79674a10_848 | RP | POS | 10.85 | 201.7967 | | | | 5.99E-05 | 0.68 | 1.16 | 7.58E-01 | -0.06 | 0.65 | 3.44E-03 | 0.64 | 1.06 | 4.96E-05 | 0.71 | 1.47 | 946277 | 312191 | 1253284 | 468384 | 1267831 | 353724 | 1238736 | 563641 |
| HILIC_NEG_228_98117a0_831 | HILIC | NEG | 0.831 | 228.9812 | | | | 3.64E-01 | 0.15 | 0.86 | 7.58E-01 | -0.07 | 0.92 | 5.86E-01 | 0.12 | 0.37 | 3.04E-01 | 0.19 | 0.47 | 8310802 | 2698346 | 8881984 | 4050393 | 9007623 | 3358823 | 8756345 | 4672501 |
| HILIC_NEG_212_92065a2_91 | HILIC | NEG | 2.91 | 212.9207 | | | | 2.39E-07 | -0.63 | 1.35 | 7.58E-01 | 0.07 | 0.78 | 2.19E-04 | -0.60 | 1.49 | 2.48E-04 | -0.66 | 1.54 | 8575672 | 497138 | 6748024 | 3203332 | 6648717 | 3407681 | 6847330 | 3016566 |
| HILIC_POS_200_05412a1_385 | HILIC | POS | 1.385 | 200.0541 | | | | 1.87E-14 | 0.95 | 1.64 | 7.58E-01 | -0.07 | 0.61 | 4.74E-07 | 0.91 | 1.60 | 8.27E-09 | 0.98 | 2.07 | 127368 | 73304 | 1035006 | 1008170 | 1066259 | 955871 | 1003752 | 1066711 |
| RP_POS_282_9201a15_454 | RP | POS | 15.45 | 282.9201 | | | | 7.20E-01 | -0.08 | 1.03 | 7.58E-01 | 0.06 | 0.34 | 8.41E-01 | -0.05 | 0.82 | 6.53E-01 | -0.11 | 0.72 | 2704329 | 704656 | 2654062 | 631486 | 2634490 | 644865 | 2673634 | 623742 |
| HILIC_POS_160_13338a2_295 | HILIC | POS | 2.295 | 160.1334 | | | | 1.59E-03 | 0.51 | 0.93 | 7.58E-01 | 0.07 | 0.58 | 6.51E-03 | 0.54 | 0.97 | 1.14E-02 | 0.48 | 0.86 | 1722676 | 709280 | 2277612 | 1149969 | 2241993 | 1105657 | 2313231 | 1202822 |
| HILIC_POS_444_05597a3_076 | HILIC | POS | 3.076 | 444.0560 | | | | 1.22E-02 | 0.33 | 1.21 | 7.59E-01 | -0.07 | 0.84 | 7.05E-02 | 0.30 | 1.26 | 4.81E-02 | 0.37 | 1.39 | 1059588 | 66942 | 1116840 | 191674 | 1122774 | 205322 | 1110907 | 178882 |
| RP_POS_363_28598a10_845 | RP | POS | 10.85 | 363.2860 | | | | 1.65E-04 | -0.62 | 1.18 | 7.59E-01 | 0.06 | 0.97 | 2.93E-03 | -0.59 | 1.18 | 7.50E-04 | -0.65 | 1.22 | 1489979 | 347134 | 1166828 | 541461 | 1150080 | 530859 | 1183576 | 556739 |
| RP_NEG_288_93677a0_449 | RP | NEG | 0.45 | 288.9368 | | | | 5.53E-06 | -0.60 | 1.26 | 7.59E-01 | -0.07 | 1.16 | 1.81E-04 | -0.63 | 1.36 | 2.06E-03 | -0.57 | 1.32 | 3909743 | 651909 | 2820238 | 1988701 | 2881707 | 2107962 | 2758768 | 1881319 |
| RP_POS_313_32086a10_848 | RP | POS | 10.85 | 313.3209 | | | | 4.37E-01 | -0.14 | 0.32 | 7.60E-01 | -0.06 | 0.26 | 3.94E-01 | -0.18 | 0.35 | 6.10E-01 | -0.11 | 0.36 | 1724842 | 372052 | 1662280 | 454197 | 1676278 | 483834 | 1648281 | 426971 |
| RP_POS_669_49329a11_752 | RP | POS | 11.75 | 669.4933 | | | | 8.32E-04 | -0.69 | 1.29 | 7.60E-01 | -0.06 | 0.21 | 1.09E-03 | -0.72 | 1.28 | 4.53E-03 | -0.66 | 1.07 | 524714 | 213848 | 370321 | 216295 | 376988 | 235420 | 363655 | 197506 |
| RP_POS_546_82196a0_499 | RP | POS | 0.50 | 546.8220 | | | | 4.80E-04 | -0.46 | 1.22 | 7.60E-01 | -0.07 | 1.23 | 3.02E-03 | -0.49 | 1.29 | 2.16E-02 | -0.42 | 1.26 | 6275813 | 1025702 | 4961859 | 3188557 | 5060100 | 3414393 | 4863619 | 2977163 |
| RP_POS_299_25803a9_865 | RP | POS | 9.87 | 299.2580 | | | | 3.27E-02 | -0.42 | 0.83 | 7.60E-01 | -0.06 | 0.72 | 3.56E-02 | -0.45 | 0.81 | 8.43E-02 | -0.39 | 0.86 | 3427640 | 426311 | 3234660 | 456249 | 3248710 | 488562 | 3220609 | 426004 |
| RP_POS_329_24155a11_609 | RP | POS | 11.61 | 329.2416 | | | | 4.96E-01 | -0.13 | 0.57 | 7.60E-01 | 0.06 | 0.61 | 6.30E-01 | -0.10 | 0.49 | 4.81E-01 | -0.16 | 0.71 | 3718468 | 853925 | 3595248 | 975861 | 3565231 | 1097607 | 3625265 | 847033 |
| RP_POS_166_98578a0_45 | RP | POS | 0.45 | 166.9858 | | | | 2.59E-07 | -0.65 | 1.33 | 7.60E-01 | 0.07 | 1.17 | 2.54E-04 | -0.61 | 1.40 | 1.45E-04 | -0.68 | 1.44 | 4849034 | 475554 | 3655844 | 2021323 | 3593690 | 2096127 | 3717998 | 1962968 |
| HILIC_NEG_128_9597a2_912 | HILIC | NEG | 2.912 | 128.9597 | | | | 1.34E-04 | -0.57 | 1.33 | 7.60E-01 | 0.07 | 0.80 | 2.76E-03 | -0.54 | 1.36 | 1.74E-03 | -0.60 | 1.35 | 160069215 | 29963627 | 128616249 | 59022444 | 126802201 | 61983405 | 130430297 | 56477734 |
| RP_POS_303_14374a6_254 | RP | POS | 6.25 | 303.1437 | | | | 3.56E-01 | -0.19 | 0.43 | 7.60E-01 | -0.06 | 0.28 | 3.31E-01 | -0.22 | 0.39 | 4.95E-01 | -0.16 | 0.31 | 3524320 | 378141 | 3452430 | 386404 | 3464297 | 398075 | 3440563 | 378038 |
| HILIC_POS_634_875a4_325 | HILIC | POS | 4.325 | 634.8750 | | | | 2.61E-03 | 0.40 | 1.23 | 7.61E-01 | -0.07 | 0.91 | 2.35E-02 | 0.37 | 1.28 | 2.12E-02 | 0.44 | 1.41 | 5314370 | 355603 | 5676222 | 996915 | 5706823 | 1087100 | 5645620 | 907889 |
| HILIC_POS_208_88416a2_991 | HILIC | POS | 2.991 | 208.8842 | | | | 8.56E-05 | -0.56 | 1.34 | 7.61E-01 | 0.07 | 0.79 | 2.50E-03 | -0.53 | 1.40 | 1.73E-03 | -0.60 | 1.39 | 12708261 | 2449504 | 9866242 | 5475089 | 9698292 | 5777360 | 10034192 | 5208600 |
| RP_NEG_174_08777a0_548 | RP | NEG | 0.55 | 174.0878 | | | | 2.19E-05 | 0.72 | 1.50 | 7.61E-01 | 0.06 | 0.96 | 1.40E-04 | 0.75 | 1.53 | 5.54E-04 | 0.69 | 1.39 | 748047 | 200129 | 956067 | 294287 | 947060 | 300588 | 965074 | 290617 |
| RP_POS_479_37039a11_166 | RP | POS | 11.17 | 479.3704 | | | | 1.58E-01 | -0.21 | 0.94 | 7.62E-01 | 0.07 | 0.91 | 3.18E-01 | -0.17 | 0.82 | 2.21E-01 | -0.24 | 0.95 | 328808 | 61462 | 304815 | 129107 | 300876 | 140221 | 308754 | 118253 |
| RP_POS_270_27859a10_958 | RP | POS | 10.96 | 270.2786 | | | | 9.83E-09 | 0.78 | 1.27 | 7.62E-01 | 0.06 | 0.84 | 1.01E-04 | 0.81 | 1.30 | 1.63E-07 | 0.75 | 1.58 | 846847 | 249879 | 1337208 | 665435 | 1316899 | 485114 | 1357517 | 811460 |
| RP_NEG_612_26489a10_511 | RP | NEG | 10.51 | 612.2649 | | | | 5.24E-02 | -0.43 | 0.87 | 7.62E-01 | 0.06 | 0.55 | 9.84E-02 | -0.40 | 0.77 | 5.34E-02 | -0.46 | 0.81 | 2365002 | 607725 | 2127607 | 517387 | 2111833 | 495894 | 2143382 | 542608 |
| RP_NEG_325_185a9_979 | RP | NEG | 9.98 | 325.1850 | | | | 8.17E-01 | -0.05 | 0.47 | 7.62E-01 | -0.06 | 0.19 | 7.39E-01 | -0.08 | 0.20 | 9.24E-01 | -0.02 | 0.44 | 1656715 | 1470994 | 1589926 | 1205749 | 1626645 | 1244441 | 1553208 | 1177262 |
| RP_POS_253_11765a0_716 | RP | POS | 0.72 | 253.1177 | | | | 3.58E-08 | 0.66 | 1.28 | 7.63E-01 | 0.07 | 1.14 | 1.40E-04 | 0.69 | 1.39 | 7.33E-05 | 0.63 | 1.41 | 35214 | 26861 | 377189 | 570700 | 359815 | 529311 | 394563 | 614213 |
| RP_POS_462_30243a12_124 | RP | POS | 12.12 | 462.3024 | | | | 2.49E-01 | -0.21 | 1.02 | 7.63E-01 | 0.06 | 0.80 | 3.73E-01 | -0.18 | 0.85 | 2.74E-01 | -0.24 | 0.85 | 630278 | 128393 | 597735 | 163258 | 592767 | 179618 | 602703 | 146756 |
| RP_POS_553_45959a12_89 | RP | POS | 12.89 | 553.4596 | | | | 4.58E-02 | 0.34 | 0.85 | 7.63E-01 | 0.06 | 0.54 | 6.37E-02 | 0.37 | 0.93 | 1.25E-01 | 0.31 | 0.61 | 14218528 | 1775452 | 15038201 | 2537044 | 14961230 | 2559499 | 15115172 | 2537995 |
| RP_POS_181_98769a0_677 | RP | POS | 0.68 | 181.9877 | | | | 8.85E-08 | 0.67 | 1.33 | 7.63E-01 | -0.07 | 1.18 | 1.95E-04 | 0.64 | 1.40 | 6.58E-05 | 0.70 | 1.44 | 2053556 | 246411 | 2722232 | 1091924 | 2755354 | 1108933 | 2689111 | 1084877 |
| RP_POS_172_09679a3_969 | RP | POS | 3.97 | 172.0968 | | | | 1.68E-01 | 0.16 | 0.69 | 7.63E-01 | -0.07 | 0.26 | 3.21E-01 | 0.12 | 0.86 | 3.22E-01 | 0.19 | 0.45 | 7827 | 9238 | 1888247 | 13531850 | 2298811 | 16189246 | 1477684 | 10369339 |
| RP_POS_213_14603a7_439 | RP | POS | 7.44 | 213.1460 | | | | 8.60E-01 | 0.03 | 0.54 | 7.63E-01 | 0.06 | 0.68 | 7.71E-01 | 0.07 | 0.22 | 9.89E-01 | 0.00 | 0.66 | 3801582 | 224250 | 3809879 | 250637 | 3802280 | 239958 | 3817478 | 263101 |
| RP_POS_204_06316a0_704 | RP | POS | 0.70 | 204.0632 | | | | 4.33E-03 | -0.43 | 1.18 | 7.63E-01 | -0.07 | 0.66 | 1.16E-02 | -0.47 | 1.12 | 3.64E-02 | -0.40 | 1.29 | 830110 | 193877 | 683939 | 364969 | 695003 | 377658 | 672875 | 355313 |
| HILIC_POS_278_21115a0_578 | HILIC | POS | 0.578 | 278.2112 | | | | 3.49E-03 | 0.38 | 0.73 | 7.64E-01 | 0.07 | 0.51 | 1.46E-02 | 0.41 | 0.80 | 5.67E-02 | 0.34 | 0.77 | 6712434 | 9527836 | 17652933 | 32676556 | 16662664 | 34201274 | 18643202 | 31393904 |
| RP_POS_173_07843a10_572 | RP | POS | 10.57 | 173.0784 | | | | 3.89E-02 | 0.38 | 0.70 | 7.64E-01 | -0.06 | 0.24 | 1.06E-01 | 0.35 | 0.66 | 4.51E-02 | 0.42 | 0.81 | 1898955 | 741215 | 2234030 | 898502 | 2261257 | 848277 | 2206802 | 953940 |
| RP_NEG_229_9019a1_466 | RP | NEG | 1.47 | 229.9019 | | | | 3.56E-10 | 0.89 | 1.49 | 7.64E-01 | 0.06 | 0.61 | 1.12E-06 | 0.92 | 1.63 | 8.47E-07 | 0.86 | 1.52 | 1202202 | 953261 | 3093669 | 2188566 | 3027352 | 2082796 | 3159986 | 2308763 |
| RP_POS_136_95326a0_255 | RP | POS | 0.26 | 136.9533 | | | | 3.82E-01 | 0.17 | 0.35 | 7.64E-01 | -0.06 | 0.49 | 5.34E-01 | 0.13 | 0.23 | 3.65E-01 | 0.20 | 0.34 | 20888005 | 2004780 | 21262269 | 2347485 | 21333395 | 2379302 | 21191144 | 2337184 |
| HILIC_POS_130_08653a3_843 | HILIC | POS | 3.843 | 130.0865 | | | | 1.01E-03 | -0.87 | 1.92 | 7.64E-01 | 0.05 | 0.49 | 2.17E-03 | -0.85 | 1.42 | 9.68E-04 | -0.89 | 1.72 | 25028932 | 13172289 | 16283625 | 7814072 | 16046892 | 7053498 | 16520358 | 8573176 |
| RP_POS_607_29266a10_619 | RP | POS | 10.62 | 607.2927 | | | | 1.47E-01 | -0.32 | 0.76 | 7.64E-01 | 0.06 | 0.24 | 2.45E-01 | -0.29 | 0.63 | 1.31E-01 | -0.35 | 0.70 | 765736 | 291867 | 680721 | 257026 | 672934 | 223577 | 688508 | 288726 |
| HILIC_NEG_132_92365a5_606 | HILIC | NEG | 5.606 | 132.9237 | | | | 3.97E-01 | -0.19 | 0.81 | 7.64E-01 | -0.06 | 0.74 | 3.63E-01 | -0.22 | 0.42 | 5.20E-01 | -0.16 | 0.52 | 1427666 | 280543 | 1380430 | 245089 | 1387853 | 258004 | 1373007 | 233848 |
| RP_POS_204_95728a0_468 | RP | POS | 0.47 | 204.9573 | | | | 5.98E-04 | -0.59 | 1.20 | 7.64E-01 | -0.06 | 1.01 | 2.06E-03 | -0.62 | 1.16 | 5.50E-03 | -0.56 | 1.25 | 386988 | 73609 | 326776 | 106309 | 329995 | 107086 | 323556 | 106513 |
| RP_NEG_299_22354a9_889 | RP | NEG | 9.89 | 299.2235 | | | | 8.31E-01 | 0.04 | 0.54 | 7.64E-01 | 0.06 | 0.39 | 7.38E-01 | 0.07 | 0.19 | 9.51E-01 | 0.01 | 0.59 | 1734209 | 315843 | 1747801 | 299404 | 1738736 | 331906 | 1756867 | 266057 |
| HILIC_POS_208_03973a7_963 | HILIC | POS | 7.963 | 208.0397 | | | | 1.94E-05 | -0.65 | 1.28 | 7.65E-01 | 0.06 | 0.72 | 8.89E-04 | -0.62 | 1.29 | 3.05E-04 | -0.69 | 1.35 | 12907136 | 3081928 | 9351833 | 5753158 | 9178217 | 5812555 | 9525450 | 5746811 |
| RP_POS_248_89961a15_43 | RP | POS | 15.43 | 248.8996 | | | | 8.04E-01 | -0.04 | 0.22 | 7.65E-01 | -0.07 | 0.57 | 6.99E-01 | -0.07 | 0.32 | 9.67E-01 | -0.01 | 0.30 | 3087109 | 297250 | 3069882 | 450835 | 3083488 | 481833 | 3056277 | 422033 |
| RP_POS_478_83469a0_499 | RP | POS | 0.50 | 478.8347 | | | | 8.87E-04 | -0.44 | 1.20 | 7.65E-01 | -0.07 | 1.23 | 4.60E-03 | -0.47 | 1.26 | 2.79E-02 | -0.41 | 1.25 | 10328994 | 1788429 | 8214084 | 5350339 | 8375380 | 5716173 | 8052789 | 5010861 |
| HILIC_NEG_203_7989a3_032 | HILIC | NEG | 3.032 | 203.7989 | | | | 7.34E-05 | 0.50 | 1.32 | 7.65E-01 | -0.07 | 0.77 | 3.56E-03 | 0.47 | 1.41 | 3.65E-03 | 0.54 | 1.52 | 1235549 | 55490 | 1330681 | 210350 | 1337020 | 226416 | 1324342 | 195067 |
| HILIC_NEG_234_82027a5_575 | HILIC | NEG | 5.575 | 234.8203 | | | | 5.36E-01 | -0.09 | 0.64 | 7.65E-01 | 0.07 | 0.57 | 6.89E-01 | -0.06 | 0.79 | 5.68E-01 | -0.12 | 0.44 | 556655 | 51933 | 547996 | 104891 | 544841 | 130438 | 551150 | 72072 |
| RP_POS_167_05641a1_973 | RP | POS | 1.97 | 167.0564 | | | | 5.01E-04 | -0.97 | 1.77 | 7.66E-01 | -0.04 | 0.42 | 5.26E-04 | -0.99 | 1.55 | 8.51E-04 | -0.94 | 1.66 | 767627 | 662246 | 301594 | 342748 | 311887 | 344554 | 291300 | 344116 |
| RP_NEG_659_07574a9_006 | RP | NEG | 9.01 | 659.0757 | | | | 9.20E-01 | -0.02 | 0.60 | 7.66E-01 | -0.06 | 0.55 | 8.33E-01 | -0.05 | 0.34 | 9.71E-01 | 0.01 | 0.26 | 3303946 | 209148 | 3299704 | 194735 | 3305547 | 175538 | 3293862 | 213855 |
| RP_POS_641_59399a11_248 | RP | POS | 11.25 | 641.5940 | | | | 1.78E-13 | 0.98 | 1.51 | 7.66E-01 | 0.06 | 0.67 | 3.15E-07 | 1.01 | 1.60 | 2.45E-09 | 0.94 | 1.71 | 3189550 | 2255080 | 9741143 | 6898905 | 9534176 | 5897303 | 9948110 | 7829144 |
| RP_POS_370_7402a0_52 | RP | POS | 0.52 | 370.7402 | | | | 5.73E-07 | 0.61 | 1.30 | 7.66E-01 | -0.07 | 1.12 | 5.10E-04 | 0.57 | 1.32 | 2.96E-04 | 0.64 | 1.39 | 99709 | 26708 | 203194 | 189070 | 208858 | 196054 | 197531 | 183636 |
| RP_POS_282_40607a10_845 | RP | POS | 10.85 | 282.4061 | | | | 1.86E-04 | 0.59 | 1.05 | 7.67E-01 | -0.06 | 0.68 | 5.98E-03 | 0.56 | 1.02 | 5.31E-04 | 0.62 | 1.28 | 306954 | 133645 | 435497 | 230371 | 442385 | 206005 | 428609 | 254342 |
| RP_NEG_908_77045a0_496 | RP | NEG | 0.50 | 908.7705 | | | | 7.66E-07 | -0.61 | 1.32 | 7.67E-01 | 0.07 | 1.20 | 5.18E-04 | -0.58 | 1.38 | 3.10E-04 | -0.64 | 1.41 | 3861207 | 325786 | 2909962 | 1727362 | 2858357 | 1793549 | 2961567 | 1675173 |
| RP_POS_295_22427a10_017 | RP | POS | 10.02 | 295.2243 | | | | 6.25E-02 | -0.38 | 0.81 | 7.67E-01 | 0.06 | 0.71 | 1.21E-01 | -0.35 | 0.73 | 7.30E-02 | -0.41 | 0.84 | 2443390 | 246067 | 2347650 | 253218 | 2340087 | 257466 | 2355213 | 251279 |
| HILIC_POS_290_84735a2_99 | HILIC | POS | 2.99 | 290.8474 | | | | 5.07E-06 | -0.59 | 1.36 | 7.67E-01 | 0.07 | 0.77 | 8.18E-04 | -0.55 | 1.47 | 7.29E-04 | -0.62 | 1.50 | 75532028 | 9021591 | 58682730 | 31585652 | 57741085 | 33456394 | 59624375 | 29908648 |
| RP_POS_399_34448a11_162 | RP | POS | 11.16 | 399.3445 | | | | 1.36E-03 | -0.62 | 1.04 | 7.67E-01 | 0.06 | 0.52 | 6.41E-03 | -0.59 | 1.07 | 2.70E-03 | -0.65 | 1.07 | 643015 | 107668 | 567067 | 121379 | 563450 | 121118 | 570683 | 122760 |
| RP_POS_359_24121a9_041 | RP | POS | 9.04 | 359.2412 | | | | 6.29E-03 | -0.51 | 1.19 | 7.68E-01 | -0.06 | 0.54 | 1.20E-02 | -0.54 | 1.14 | 2.18E-02 | -0.48 | 0.98 | 2785349 | 88030 | 2732142 | 105712 | 2735289 | 103467 | 2728995 | 108870 |
| HILIC_POS_113_10762a0_549 | HILIC | POS | 0.549 | 113.1076 | | | | 4.94E-05 | -0.79 | 1.38 | 7.68E-01 | -0.06 | 0.51 | 1.63E-04 | -0.82 | 1.34 | 4.01E-04 | -0.76 | 1.29 | 2671205 | 426389 | 2278599 | 484754 | 2293028 | 484536 | 2264170 | 489457 |
| RP_NEG_428_18729a11_611 | RP | NEG | 11.61 | 428.1873 | | | | 7.26E-02 | -0.33 | 0.97 | 7.68E-01 | -0.06 | 0.71 | 7.76E-02 | -0.36 | 0.84 | 1.67E-01 | -0.30 | 0.76 | 2189833 | 232430 | 2098863 | 281551 | 2107220 | 300863 | 2090506 | 263627 |
| RP_POS_458_3237a9_678 | RP | POS | 9.68 | 458.3237 | | | | 8.57E-01 | -0.05 | 0.33 | 7.68E-01 | -0.05 | 0.25 | 8.02E-01 | -0.07 | 0.35 | 9.28E-01 | -0.03 | 0.19 | 590834 | 844655 | 562519 | 463209 | 576259 | 392350 | 548778 | 528375 |

**Supplementary table 1: Metabolomics results** (in asceding order according to p-value from progressive ALD vs  non-progressive ALD comparison, with molecular features with ID_level of 1-2 first)

| | | | | | | | | all_ALD_vs_control | | | Prog_ALD_vs_non-prog_ALD | | | Prog_ALD_vs_control_ALD | | | Non-prog_ALD_vs_control | | | Healthy_control | | all_ALD | | Non-prog_ALD | | Progressive_ALD | |
|---|---|---|---|---|---|---|---|---|---|---|---|---|---|---|---|---|---|---|---|---|---|---|---|---|---|---|---|
| | | | | | | | | t-test | Cohen's | PLS-DA | t-test | Cohen's | PLS-DA | t-test | Cohen's | PLS-DA | t-test | Cohen's | PLS-DA | | | | | | | | |
| Feature_ID | RP_HILIC | POS_NEG | Average.Rt | Average.Mz | Metabolite.name | curated_MSMS | ID_level | p | d | VIP | p | d | VIP | p | d | VIP | p | d | VIP | Mean | SD | Mean | SD | Mean | SD | Mean | SD |
| RP_POS_954_7467a0_497 | RP | POS | 0.50 | 954.7467 | | | | 3.34E-07 | -0.62 | 1.33 | 7.69E-01 | 0.07 | 1.19 | 3.72E-04 | -0.59 | 1.40 | 2.18E-04 | -0.65 | 1.43 | 2955460 | 206885 | 2204349 | 1334850 | 2164798 | 1383392 | 2243901 | 1297322 |
| RP_POS_548_82056a0_499 | RP | POS | 0.50 | 548.8206 | | | | 4.99E-04 | -0.46 | 1.20 | 7.69E-01 | -0.07 | 1.24 | 3.10E-03 | -0.50 | 1.28 | 1.97E-02 | -0.43 | 1.24 | 1356756 | 216025 | 1096016 | 622191 | 1114423 | 663645 | 1077609 | 583977 |
| RP_POS_140_90327a0_521 | RP | POS | 0.52 | 140.9033 | | | | 2.92E-05 | 0.51 | 1.21 | 7.69E-01 | -0.07 | 1.09 | 3.07E-03 | 0.48 | 1.19 | 2.46E-03 | 0.54 | 1.35 | 234216 | 28250 | 305598 | 156273 | 310220 | 165335 | 300977 | 148195 |
| RP_NEG_316_9483a0_496 | RP | NEG | 0.50 | 316.9483 | | | | 4.19E-06 | 0.56 | 1.30 | 7.69E-01 | -0.07 | 1.20 | 8.68E-04 | 0.53 | 1.37 | 1.19E-03 | 0.59 | 1.38 | 49315114 | 3092171 | 59339887 | 19982300 | 59930419 | 21534790 | 58749355 | 18500664 |
| RP_POS_195_09996a5_581 | RP | POS | 5.58 | 195.1000 | | | | 5.65E-01 | 0.10 | 0.37 | 7.69E-01 | -0.06 | 0.46 | 7.29E-01 | 0.07 | 0.38 | 5.26E-01 | 0.13 | 0.59 | 1146233 | 124689 | 1162175 | 165665 | 1167067 | 171049 | 1157282 | 161689 |
| RP_POS_245_07825a7_493 | RP | POS | 7.49 | 245.0783 | | | | 5.88E-01 | 0.10 | 0.46 | 7.69E-01 | 0.06 | 0.72 | 5.49E-01 | 0.13 | 0.32 | 7.38E-01 | 0.07 | 0.44 | 11972984 | 902824 | 12078560 | 1101194 | 12046048 | 1041875 | 12111071 | 1167195 |
| RP_POS_397_22189a9_072 | RP | POS | 9.07 | 397.2219 | | | | 1.21E-09 | 0.73 | 1.20 | 7.70E-01 | -0.06 | 0.38 | 3.49E-05 | 0.70 | 1.41 | 1.09E-05 | 0.77 | 1.31 | 74246 | 175516 | 2999141 | 4350531 | 3127540 | 4403187 | 2870742 | 4338065 |
| HILIC_POS_387_21359a3_927 | HILIC | POS | 3.927 | 387.2136 | | | | 3.28E-02 | -0.39 | 0.96 | 7.70E-01 | 0.06 | 0.78 | 8.62E-02 | -0.36 | 0.77 | 4.72E-02 | -0.42 | 0.76 | 4472596 | 982600 | 4006201 | 1249140 | 3969354 | 1267040 | 4043047 | 1242734 |
| RP_POS_344_01434a0_631 | RP | POS | 0.63 | 344.0143 | | | | 2.63E-08 | -0.68 | 1.36 | 7.70E-01 | 0.06 | 1.17 | 1.13E-04 | -0.65 | 1.44 | 4.74E-05 | -0.72 | 1.47 | 1828004 | 135700 | 1339406 | 781829 | 1316370 | 799141 | 1362441 | 771543 |
| RP_POS_512_33423a8_761 | RP | POS | 8.76 | 512.3342 | | | | 1.43E-03 | -0.50 | 1.31 | 7.70E-01 | 0.06 | 0.63 | 3.65E-02 | -0.47 | 1.25 | 4.84E-04 | -0.53 | 1.30 | 849368 | 249201 | 646077 | 434105 | 633274 | 278430 | 658879 | 550347 |
| RP_NEG_325_18503a15_464 | RP | NEG | 15.46 | 325.1850 | | | | 7.86E-01 | -0.06 | 0.43 | 7.70E-01 | 0.06 | 0.37 | 8.99E-01 | -0.03 | 0.51 | 7.03E-01 | -0.09 | 0.23 | 7162562 | 2596075 | 7023649 | 2203404 | 6958741 | 1892046 | 7088558 | 2494118 |
| RP_NEG_371_19043a9_565 | RP | NEG | 9.57 | 371.1904 | | | | 1.12E-02 | 0.40 | 0.76 | 7.70E-01 | 0.06 | 0.69 | 3.83E-02 | 0.43 | 0.83 | 3.50E-02 | 0.37 | 0.82 | 1167551 | 1320760 | 2010435 | 2270563 | 1943579 | 1953672 | 2077290 | 2567132 |
| RP_POS_605_45154a10_976 | RP | POS | 10.98 | 605.4515 | | | | 1.09E-02 | 0.43 | 1.33 | 7.70E-01 | -0.06 | 1.17 | 5.44E-02 | 0.40 | 1.14 | 1.72E-02 | 0.46 | 1.27 | 552315 | 268832 | 714943 | 403957 | 726834 | 379663 | 703052 | 430420 |
| RP_POS_478_38873a10_785 | RP | POS | 10.79 | 478.3887 | | | | 6.30E-01 | -0.10 | 1.04 | 7.70E-01 | -0.06 | 0.59 | 5.74E-01 | -0.13 | 0.90 | 7.56E-01 | -0.07 | 0.81 | 1301536 | 460086 | 1257013 | 428574 | 1269617 | 437411 | 1244410 | 423613 |
| RP_POS_472_26309a6_668 | RP | POS | 6.67 | 472.2631 | | | | 2.03E-07 | 0.62 | 1.34 | 7.70E-01 | -0.07 | 1.14 | 4.43E-04 | 0.59 | 1.44 | 1.58E-04 | 0.65 | 1.42 | 17822 | 20337 | 1284906 | 2267663 | 1351578 | 2303321 | 1218234 | 2252814 |
| RP_NEG_326_83194a15_519 | RP | NEG | 15.52 | 326.8319 | | | | 1.67E-01 | 0.32 | 0.56 | 7.70E-01 | 0.05 | 0.38 | 1.40E-01 | 0.35 | 0.62 | 2.68E-01 | 0.29 | 0.47 | 2183056 | 335007 | 2274216 | 266961 | 2266363 | 320019 | 2282069 | 203599 |
| HILIC_POS_274_78516a3_016 | HILIC | POS | 3.016 | 274.7852 | | | | 1.42E-04 | -0.50 | 1.34 | 7.71E-01 | 0.06 | 0.77 | 5.29E-03 | -0.47 | 1.44 | 3.41E-03 | -0.54 | 1.47 | 386654 | 59429 | 306463 | 175459 | 301307 | 183920 | 311619 | 168283 |
| HILIC_NEG_153_86874a0_675 | HILIC | NEG | 0.675 | 153.8687 | | | | 5.27E-04 | -0.71 | 1.13 | 7.71E-01 | 0.06 | 1.47 | 1.80E-03 | -0.69 | 1.26 | 1.32E-03 | -0.74 | 1.18 | 964686 | 168634 | 837976 | 169864 | 832987 | 181507 | 842965 | 159062 |
| RP_POS_293_28351a11_246 | RP | POS | 11.25 | 293.2835 | | | | 9.80E-11 | 0.92 | 1.44 | 7.71E-01 | 0.06 | 0.85 | 7.54E-06 | 0.95 | 1.45 | 1.80E-09 | 0.89 | 1.82 | 858432 | 414138 | 1718766 | 966157 | 1690381 | 694457 | 1747151 | 1184083 |
| HILIC_NEG_298_8804a2_91 | HILIC | NEG | 2.91 | 298.8804 | | | | 3.08E-07 | -0.63 | 1.35 | 7.71E-01 | 0.06 | 0.78 | 2.33E-04 | -0.60 | 1.50 | 2.65E-04 | -0.66 | 1.54 | 4236438 | 296095 | 3302813 | 1636722 | 3254796 | 1737751 | 3350830 | 1545284 |
| RP_POS_684_79498a0_499 | RP | POS | 0.50 | 684.7950 | | | | 1.65E-03 | -0.42 | 1.21 | 7.71E-01 | -0.06 | 1.24 | 7.20E-03 | -0.46 | 1.27 | 3.37E-02 | -0.39 | 1.25 | 1209617 | 197218 | 1003698 | 537301 | 1019457 | 568678 | 987939 | 509289 |
| RP_NEG_314_98926a7_717 | RP | NEG | 7.72 | 314.9893 | | | | 2.20E-01 | -0.28 | 0.60 | 7.71E-01 | -0.06 | 0.17 | 2.14E-01 | -0.30 | 0.58 | 3.09E-01 | -0.25 | 0.41 | 9841051 | 508677 | 9717134 | 428397 | 9729689 | 430413 | 9704579 | 430368 |
| HILIC_POS_129_06601a5_5 | HILIC | POS | 5.5 | 129.0660 | | | | 3.41E-01 | 0.15 | 0.47 | 7.71E-01 | 0.06 | 0.97 | 3.95E-01 | 0.18 | 0.36 | 4.89E-01 | 0.12 | 0.31 | 361763 | 435096 | 465698 | 766848 | 443255 | 624665 | 488142 | 892680 |
| RP_POS_860_75238a0_5 | RP | POS | 0.50 | 860.7524 | | | | 3.43E-04 | -0.49 | 1.22 | 7.72E-01 | -0.06 | 1.22 | 2.19E-03 | -0.52 | 1.27 | 1.40E-02 | -0.46 | 1.26 | 1046151 | 175435 | 845031 | 449720 | 858188 | 478758 | 831873 | 423156 |
| RP_POS_167_716a11_165 | RP | POS | 11.17 | 167.7160 | | | | 4.75E-11 | 1.11 | 1.76 | 7.72E-01 | -0.06 | 0.53 | 7.25E-08 | 1.08 | 1.74 | 1.61E-09 | 1.13 | 1.89 | 220681 | 90099 | 380447 | 136972 | 384452 | 126922 | 376442 | 147525 |
| RP_POS_616_80725a0_499 | RP | POS | 0.50 | 616.8073 | | | | 5.22E-04 | -0.47 | 1.22 | 7.72E-01 | -0.06 | 1.23 | 3.18E-03 | -0.50 | 1.29 | 1.88E-02 | -0.44 | 1.26 | 1299593 | 215316 | 1049072 | 592019 | 1066364 | 629558 | 1031780 | 557829 |
| RP_NEG_405_19357a6_795 | RP | NEG | 6.80 | 405.1936 | | | | 3.22E-02 | 0.37 | 1.39 | 7.72E-01 | 0.06 | 0.33 | 3.78E-02 | 0.40 | 0.97 | 1.09E-01 | 0.34 | 1.34 | 753908 | 160515 | 832885 | 224104 | 826343 | 244497 | 839427 | 203972 |
| HILIC_NEG_115_92069a3_008 | HILIC | NEG | 3.008 | 115.9207 | | | | 8.48E-01 | 0.04 | 0.88 | 7.72E-01 | -0.05 | 0.67 | 9.45E-01 | 0.02 | 0.43 | 7.71E-01 | 0.07 | 0.43 | 17426681 | 2105672 | 17505188 | 1638413 | 17552955 | 1542064 | 17457421 | 1743834 |
| RP_POS_312_91617a15_483 | RP | POS | 15.48 | 312.9162 | | | | 2.07E-01 | -0.23 | 0.78 | 7.72E-01 | 0.06 | 0.31 | 3.53E-01 | -0.20 | 0.56 | 2.04E-01 | -0.26 | 0.71 | 2427593 | 323451 | 2338324 | 403375 | 2326569 | 384068 | 2350080 | 425386 |
| RP_NEG_461_14453a11_611 | RP | NEG | 11.61 | 461.1445 | | | | 5.22E-01 | 0.15 | 0.98 | 7.72E-01 | 0.06 | 0.58 | 4.79E-01 | 0.17 | 0.77 | 6.35E-01 | 0.12 | 0.98 | 818573 | 117410 | 833313 | 95676 | 830525 | 99160 | 836100 | 92985 |
| RP_NEG_508_87665a0_46 | RP | NEG | 0.46 | 508.8767 | | | | 3.05E-07 | -0.67 | 1.32 | 7.73E-01 | 0.06 | 1.14 | 2.12E-04 | -0.64 | 1.38 | 8.65E-05 | -0.71 | 1.41 | 526686 | 81805 | 369570 | 252385 | 362228 | 257598 | 376913 | 249458 |
| HILIC_NEG_316_94769a4_232 | HILIC | NEG | 4.232 | 316.9477 | | | | 1.02E-01 | 0.24 | 1.07 | 7.73E-01 | -0.06 | 0.87 | 2.46E-01 | 0.21 | 0.76 | 1.51E-01 | 0.27 | 1.07 | 46409867 | 6254097 | 49190424 | 12721298 | 49559655 | 13221475 | 48821192 | 12323955 |
| RP_POS_419_31558a12_242 | RP | POS | 12.24 | 419.3156 | | | | 7.32E-01 | 0.07 | 0.32 | 7.73E-01 | -0.06 | 0.68 | 8.64E-01 | 0.04 | 0.58 | 6.59E-01 | 0.10 | 0.65 | 10329837 | 4534451 | 10650744 | 4771535 | 10789232 | 4628615 | 10512255 | 4953435 |
| RP_POS_581_27832a10_517 | RP | POS | 10.52 | 581.2783 | | | | 6.77E-02 | -0.38 | 0.77 | 7.74E-01 | -0.06 | 0.44 | 7.96E-02 | -0.41 | 0.75 | 1.19E-01 | -0.35 | 0.57 | 684772 | 199419 | 609862 | 191743 | 615420 | 184944 | 604304 | 200034 |
| RP_NEG_384_16144a11_162 | RP | NEG | 11.16 | 384.1614 | | | | 2.91E-01 | 0.18 | 0.86 | 7.74E-01 | 0.06 | 0.70 | 2.88E-01 | 0.21 | 0.76 | 4.59E-01 | 0.15 | 0.74 | 836035 | 69768 | 852678 | 96195 | 849895 | 98819 | 855461 | 94418 |
| RP_POS_239_16145a8_561 | RP | POS | 8.56 | 239.1615 | | | | 4.83E-01 | 0.13 | 0.54 | 7.74E-01 | -0.06 | 0.68 | 6.62E-01 | 0.10 | 0.22 | 4.17E-01 | 0.16 | 0.52 | 4663057 | 564749 | 4748763 | 691505 | 4768763 | 583729 | 4728763 | 790291 |
| RP_NEG_440_88895a0_458 | RP | NEG | 0.46 | 440.8890 | | | | 1.73E-06 | -0.63 | 1.28 | 7.74E-01 | 0.06 | 1.13 | 4.91E-04 | -0.60 | 1.34 | 2.38E-04 | -0.66 | 1.37 | 821081 | 135166 | 585729 | 404188 | 574061 | 415501 | 597396 | 396423 |
| RP_POS_249_13313a4_569 | RP | POS | 4.57 | 249.1331 | | | | 2.25E-01 | -0.24 | 0.54 | 7.75E-01 | -0.06 | 0.33 | 2.37E-01 | -0.27 | 0.50 | 3.29E-01 | -0.21 | 0.51 | 1635068 | 116127 | 1605657 | 123434 | 1609217 | 116424 | 1602097 | 131158 |
| RP_POS_297_0293a3_622 | RP | POS | 3.62 | 297.0293 | | | | 1.65E-02 | -0.51 | 0.91 | 7.75E-01 | 0.06 | 0.83 | 4.62E-02 | -0.49 | 0.79 | 1.55E-02 | -0.54 | 1.12 | 1082177 | 919886 | 626542 | 849490 | 602043 | 723928 | 651041 | 965763 |
| RP_NEG_915_1272a9_56 | RP | NEG | 9.56 | 915.1272 | | | | 8.48E-01 | -0.04 | 0.51 | 7.75E-01 | -0.06 | 0.32 | 7.64E-01 | -0.07 | 0.43 | 9.60E-01 | -0.01 | 0.49 | 3350670 | 245336 | 3341216 | 230910 | 3347873 | 243173 | 3334558 | 220235 |
| RP_NEG_211_91736a15_562 | RP | NEG | 15.56 | 211.9174 | | | | 9.14E-04 | -0.63 | 1.61 | 7.75E-01 | -0.06 | 0.27 | 1.03E-03 | -0.66 | 1.61 | 8.30E-03 | -0.60 | 1.12 | 6013615 | 797713 | 5421510 | 947540 | 5448832 | 1086269 | 5394187 | 795287 |
| RP_POS_524_83057a0_474 | RP | POS | 0.47 | 524.8306 | | | | 2.51E-05 | -0.57 | 1.26 | 7.75E-01 | -0.06 | 1.23 | 4.40E-04 | -0.60 | 1.35 | 3.59E-03 | -0.54 | 1.32 | 359408 | 64283 | 268236 | 174760 | 273272 | 184887 | 263200 | 165742 |
| RP_NEG_224_06203a6_51 | RP | NEG | 6.51 | 224.0620 | | | | 3.22E-01 | -0.36 | 0.70 | 7.75E-01 | 0.01 | 0.44 | 3.29E-01 | -0.35 | 0.50 | 3.16E-01 | -0.36 | 0.58 | 95338903 | 443431971 | 16379572 | 34638819 | 15383105 | 25168561 | 17376039 | 42293018 |
| RP_NEG_324_87967a15_474 | RP | NEG | 15.47 | 324.8797 | | | | 9.20E-01 | -0.02 | 0.55 | 7.75E-01 | 0.06 | 0.43 | 9.51E-01 | 0.01 | 0.24 | 8.10E-01 | -0.05 | 0.81 | 4262564 | 559231 | 4250044 | 753130 | 4228395 | 718834 | 4271693 | 792657 |
| RP_NEG_226_96576a15_292 | RP | NEG | 15.29 | 226.9658 | | | | 2.45E-01 | -0.22 | 0.46 | 7.75E-01 | -0.06 | 0.44 | 2.38E-01 | -0.25 | 0.51 | 3.90E-01 | -0.19 | 0.78 | 3813513 | 109774 | 3786150 | 129593 | 3789874 | 136286 | 3782426 | 123810 |
| RP_POS_330_27655a10_985 | RP | POS | 10.99 | 330.2766 | | | | 2.43E-04 | 0.60 | 0.94 | 7.76E-01 | 0.06 | 0.44 | 3.12E-03 | 0.63 | 1.01 | 1.30E-03 | 0.57 | 1.11 | 728943 | 576789 | 1258391 | 933451 | 1231597 | 784830 | 1285184 | 1069121 |
| RP_POS_407_258a13_724 | RP | POS | 13.72 | 407.2580 | | | | 1.46E-03 | 0.64 | 1.04 | 7.76E-01 | 0.06 | 0.37 | 3.45E-03 | 0.67 | 1.06 | 4.47E-03 | 0.62 | 1.02 | 1192904 | 580581 | 1592094 | 603074 | 1574794 | 559511 | 1609394 | 648965 |
| RP_POS_321_06982a8_805 | RP | POS | 8.81 | 321.0698 | | | | 5.54E-02 | 0.32 | 0.81 | 7.76E-01 | 0.06 | 1.11 | 2.41E-02 | 0.35 | 1.05 | 2.20E-01 | 0.29 | 0.81 | 84769674 | 29191663 | 98007419 | 44420146 | 96731982 | 57889577 | 99282857 | 25140486 |
| RP_POS_793_5575a13_277 | RP | POS | 13.28 | 793.5575 | | | | 9.30E-01 | -0.02 | 0.30 | 7.76E-01 | 0.06 | 0.32 | 9.60E-01 | 0.01 | 0.23 | 8.39E-01 | -0.05 | 0.31 | 3172370 | 1022450 | 3153864 | 1049034 | 3123781 | 1105579 | 3183947 | 999625 |
| HILIC_NEG_118_93065a2_931 | HILIC | NEG | 2.931 | 118.9307 | | | | 4.17E-04 | -0.53 | 1.32 | 7.76E-01 | 0.06 | 0.78 | 5.68E-03 | -0.50 | 1.34 | 3.71E-03 | -0.56 | 1.33 | 17692367 | 3362204 | 14466678 | 6551497 | 14278998 | 6875654 | 14654359 | 6274846 |
| HILIC_NEG_100_93346a4_797 | HILIC | NEG | 4.797 | 100.9335 | | | | 2.00E-01 | -0.26 | 0.75 | 7.76E-01 | -0.06 | 0.62 | 2.14E-01 | -0.29 | 0.55 | 2.90E-01 | -0.23 | 0.49 | 52407041 | 11865522 | 49278834 | 11862065 | 49618625 | 11005626 | 48939042 | 12764377 |
| HILIC_NEG_253_79393a0_55 | HILIC | NEG | 0.55 | 253.7939 | | | | 3.45E-03 | -0.55 | 0.94 | 7.76E-01 | 0.06 | 0.89 | 2.21E-02 | -0.52 | 0.79 | 3.54E-03 | -0.58 | 1.08 | 255778 | 74552 | 207350 | 89389 | 204791 | 74334 | 209909 | 102980 |
| RP_NEG_200_8588a15_618 | RP | NEG | 15.62 | 200.8588 | | | | 5.33E-01 | -0.13 | 0.62 | 7.76E-01 | -0.06 | 0.32 | 5.04E-01 | -0.16 | 0.65 | 6.54E-01 | -0.10 | 0.62 | 629171 | 37317 | 624428 | 36807 | 625481 | 34354 | 623375 | 39430 |
| RP_POS_774_56281a11_525 | RP | POS | 11.53 | 774.5628 | | | | 1.69E-01 | 0.19 | 0.55 | 7.76E-01 | -0.06 | 0.61 | 3.50E-01 | 0.16 | 0.54 | 2.43E-01 | 0.22 | 0.57 | 506942 | 368649 | 666458 | 949750 | 693623 | 1021358 | 639293 | 881918 |
| RP_POS_167_97215a0_676 | RP | POS | 0.68 | 167.9722 | | | | 4.94E-07 | 0.62 | 1.30 | 7.77E-01 | -0.06 | 1.16 | 5.83E-04 | 0.59 | 1.35 | 1.56E-04 | 0.65 | 1.42 | 979347 | 120823 | 1316900 | 597662 | 1333964 | 598861 | 1299836 | 602047 |
| RP_POS_530_96454a6_895 | RP | POS | 6.90 | 530.9645 | | | | 1.72E-07 | 0.62 | 1.32 | 7.77E-01 | -0.06 | 1.13 | 3.67E-04 | 0.59 | 1.42 | 1.61E-04 | 0.66 | 1.40 | 13550 | 29404 | 1235927 | 2172459 | 1297851 | 2221323 | 1174004 | 2143215 |
| RP_POS_420_25281a11_613 | RP | POS | 11.61 | 420.2528 | | | | 1.29E-03 | -0.55 | 1.02 | 7.77E-01 | 0.06 | 0.80 | 4.31E-03 | -0.52 | 1.13 | 7.81E-03 | -0.58 | 1.02 | 795281 | 50334 | 756827 | 72938 | 754747 | 84078 | 758906 | 60585 |
| HILIC_NEG_82_97139a2_985 | HILIC | NEG | 2.985 | 82.9714 | | | | 9.35E-01 | 0.02 | 0.97 | 7.77E-01 | -0.06 | 0.83 | 9.45E-01 | -0.01 | 0.24 | 8.34E-01 | 0.04 | 0.57 | 2372768 | 442789 | 2380583 | 538001 | 2395904 | 549313 | 2365262 | 531577 |
| HILIC_POS_562_34326a0_998 | HILIC | POS | 0.998 | 562.3433 | | | | 3.10E-05 | 0.51 | 1.22 | 7.78E-01 | -0.06 | 0.78 | 5.99E-03 | 0.47 | 1.36 | 1.34E-03 | 0.54 | 1.36 | 348062 | 952908 | 2922941 | 5689671 | 3084909 | 5591814 | 2760974 | 5838102 |
| HILIC_POS_113_1076a0_681 | HILIC | POS | 0.681 | 113.1076 | | | | 1.10E-01 | -0.37 | 0.72 | 7.78E-01 | -0.05 | 0.31 | 1.16E-01 | -0.40 | 0.62 | 1.60E-01 | -0.35 | 0.58 | 3135377 | 637136 | 2935125 | 490759 | 2949091 | 469032 | 2921158 | 515961 |
| HILIC_NEG_520_91058a4_232 | HILIC | NEG | 4.232 | 520.9106 | | | | 1.92E-03 | 0.40 | 1.25 | 7.78E-01 | -0.06 | 0.92 | 2.54E-02 | 0.37 | 1.30 | 1.86E-02 | 0.43 | 1.45 | 9951626 | 626547 | 10721705 | 2164828 | 10783249 | 2299710 | 10660161 | 2042597 |
| HILIC_NEG_296_88248a2_91 | HILIC | NEG | 2.91 | 296.8825 | | | | 1.74E-07 | -0.64 | 1.35 | 7.78E-01 | 0.06 | 0.78 | 1.69E-04 | -0.61 | 1.50 | 2.16E-04 | -0.67 | 1.54 | 21439631 | 1383180 | 16640611 | 8265995 | 16405633 | 8787365 | 16875589 | 7792131 |
| RP_NEG_297_82843a15_552 | RP | NEG | 15.55 | 297.8284 | | | | 2.96E-01 | -0.20 | 0.58 | 7.78E-01 | -0.06 | 0.17 | 3.03E-01 | -0.23 | 0.52 | 4.18E-01 | -0.17 | 0.45 | 2559108 | 89652 | 2539356 | 99999 | 2542193 | 94803 | 2536520 | 105828 |
| RP_POS_148_97606a0_45 | RP | POS | 0.45 | 148.9761 | | | | 5.59E-07 | -0.64 | 1.32 | 7.79E-01 | 0.06 | 1.16 | 3.21E-04 | -0.61 | 1.38 | 1.68E-04 | -0.68 | 1.42 | 12100383 | 1639699 | 8713461 | 5737673 | 8550924 | 5908540 | 8875997 | 5616840 |
| HILIC_POS_366_26379a0_665 | HILIC | POS | 0.665 | 366.2638 | | | | 9.91E-01 | 0.00 | 0.50 | 7.79E-01 | -0.06 | 0.67 | 8.83E-01 | -0.03 | 0.16 | 9.02E-01 | 0.03 | 0.41 | 1067331 | 538225 | 1066110 | 619771 | 1083650 | 650119 | 1048570 | 593959 |
| HILIC_NEG_360_78674a2_93 | HILIC | NEG | 2.93 | 360.7867 | | | | 1.14E-06 | -0.62 | 1.34 | 7.79E-01 | 0.06 | 0.77 | 3.32E-04 | -0.59 | 1.47 | 3.54E-04 | -0.66 | 1.50 | 870717 | 98969 | 670275 | 351147 | 660353 | 372346 | 680198 | 332088 |
| RP_NEG_512_26764a7_388 | RP | NEG | 7.39 | 512.2676 | | | | 2.43E-01 | -0.38 | 1.03 | 7.79E-01 | 0.03 | 0.58 | 2.68E-01 | -0.37 | 0.90 | 2.31E-01 | -0.40 | 0.76 | 653756 | 1214368 | 395224 | 357497 | 385122 | 352387 | 405325 | 365831 |
| HILIC_POS_198_85435a3_011 | HILIC | POS | 3.011 | 198.8544 | | | | 1.45E-04 | -0.55 | 1.34 | 7.79E-01 | 0.06 | 0.78 | 3.52E-03 | -0.52 | 1.40 | 2.03E-03 | -0.58 | 1.43 | 531559 | 113019 | 408544 | 240458 | 401750 | 249573 | 415337 | 233323 |
| RP_POS_380_24667a10_455 | RP | POS | 10.46 | 380.2467 | | | | 7.72E-01 | 0.05 | 0.67 | 7.79E-01 | 0.06 | 0.24 | 7.18E-01 | 0.08 | 0.56 | 9.20E-01 | 0.02 | 0.27 | 978830 | 116976 | 986725 | 176430 | 981742 | 144471 | 991708 | 204861 |
| RP_POS_652_87286a6_534 | RP | POS | 6.53 | 652.8729 | | | | 2.95E-07 | 0.61 | 1.33 | 7.79E-01 | -0.06 | 1.14 | 6.22E-04 | 0.58 | 1.41 | 1.57E-04 | 0.64 | 1.41 | 41218 | 114294 | 1653117 | 2924778 | 1735685 | 2922731 | 1570550 | 2954144 |
| RP_NEG_1021_21887a9_76 | RP | NEG | 9.76 | 1021.2189 | | | | 1.63E-01 | 0.24 | 0.43 | 7.79E-01 | 0.06 | 0.42 | 2.07E-01 | 0.27 | 0.49 | 2.78E-01 | 0.21 | 0.50 | 4788232 | 420099 | 4924043 | 614068 | 4906707 | 559139 | 4941379 | 669781 |
| RP_POS_876_56958a14 | RP | POS | 14.00 | 876.5696 | | | | 2.99E-01 | -0.20 | 0.44 | 7.81E-01 | 0.06 | 0.26 | 4.46E-01 | -0.18 | 0.46 | 2.73E-01 | -0.23 | 0.45 | 9928087 | 2900765 | 9298468 | 3129043 | 9210647 | 2817372 | 9386289 | 3439231 |
| RP_POS_294_21915a10_732 | RP | POS | 10.73 | 294.2192 | | | | 5.07E-07 | 0.70 | 1.12 | 7.81E-01 | -0.06 | 0.58 | 9.85E-04 | 0.67 | 1.10 | 2.08E-06 | 0.73 | 1.46 | 1218843 | 302837 | 1701195 | 734627 | 1721809 | 580579 | 1680581 | 867426 |
| RP_POS_130_96547a0_45 | RP | POS | 0.45 | 130.9655 | | | | 1.18E-06 | -0.64 | 1.31 | 7.81E-01 | 0.06 | 1.15 | 4.17E-04 | -0.61 | 1.36 | 2.24E-04 | -0.67 | 1.41 | 8251994 | 1235259 | 5962191 | 3924946 | 5852176 | 4045022 | 6072206 | 3838996 |
| HILIC_POS_209_09174a4_124 | HILIC | POS | 4.124 | 209.0917 | | | | 2.72E-02 | -0.36 | 1.01 | 7.81E-01 | -0.06 | 1.31 | 7.59E-02 | -0.39 | 0.60 | 4.81E-02 | -0.33 | 1.16 | 2306663 | 493977 | 2045292 | 766521 | 2066775 | 575425 | 2023810 | 924686 |
| HILIC_POS_374_80884a2_99 | HILIC | POS | 2.99 | 374.8088 | | | | 1.46E-05 | -0.57 | 1.35 | 7.82E-01 | 0.06 | 0.77 | 1.28E-03 | -0.54 | 1.47 | 1.16E-03 | -0.60 | 1.49 | 8536433 | 1174773 | 6635405 | 3673831 | 6532920 | 3892341 | 6737889 | 3478185 |
| HILIC_NEG_382_84122a2_91 | HILIC | NEG | 2.91 | 382.8412 | | | | 2.95E-07 | -0.63 | 1.35 | 7.82E-01 | 0.06 | 0.78 | 2.14E-04 | -0.60 | 1.50 | 2.66E-04 | -0.66 | 1.54 | 3100347 | 228947 | 2397021 | 1227750 | 2362798 | 1304630 | 2431244 | 1158038 |
| HILIC_NEG_458_77112a2_923 | HILIC | NEG | 2.923 | 458.7711 | | | | 4.58E-07 | -0.65 | 1.34 | 7.82E-01 | 0.06 | 0.76 | 1.83E-04 | -0.62 | 1.47 | 2.23E-04 | -0.68 | 1.50 | 195745 | 23116 | 147781 | 80498 | 145538 | 85559 | 150024 | 75904 |
| RP_POS_462_85864a0_498 | RP | POS | 0.50 | 462.8586 | | | | 5.31E-07 | -0.61 | 1.33 | 7.82E-01 | 0.06 | 1.19 | 3.09E-04 | -0.58 | 1.41 | 3.84E-04 | -0.64 | 1.41 | 24392526 | 1731296 | 18539615 | 10571707 | 18245172 | 11247989 | 18834057 | 9955331 |
| RP_POS_553_45837a13_631 | RP | POS | 13.63 | 553.4584 | | | | 5.69E-02 | -0.40 | 0.74 | 7.82E-01 | -0.05 | 0.70 | 5.93E-02 | -0.42 | 0.90 | 1.16E-01 | -0.37 | 0.61 | 16192373 | 2533630 | 15197210 | 2456814 | 15265546 | 2627112 | 15128874 | 2298654 |
| HILIC_POS_113_10769a3_032 | HILIC | POS | 3.032 | 113.1077 | | | | 4.68E-05 | 0.50 | 1.19 | 7.83E-01 | -0.06 | 0.75 | 2.68E-03 | 0.47 | 1.27 | 3.71E-03 | 0.53 | 1.33 | 2652702 | 136019 | 2938376 | 632301 | 2955942 | 686574 | 2920810 | 579443 |
| RP_POS_955_70135a14_271 | RP | POS | 14.27 | 955.7014 | | | | 6.45E-01 | -0.09 | 0.74 | 7.83E-01 | -0.06 | 0.36 | 6.01E-01 | -0.12 | 0.57 | 7.81E-01 | -0.06 | 0.75 | 2491307 | 859007 | 2406986 | 1002629 | 2434814 | 951493 | 2379158 | 1060248 |
| HILIC_NEG_136_89175a7_674 | HILIC | NEG | 7.674 | 136.8918 | | | | 5.45E-02 | -0.36 | 0.56 | 7.83E-01 | -0.06 | 0.58 | 6.59E-02 | -0.39 | 0.75 | 1.24E-01 | -0.33 | 0.56 | 11600333 | 1903996 | 10805802 | 2258960 | 10868466 | 2329100 | 10743137 | 2208442 |
| RP_POS_1064_7146a0_496 | RP | POS | 0.50 | 1064.7146 | | | | 2.99E-01 | -0.24 | 0.47 | 7.83E-01 | 0.05 | 0.72 | 3.92E-01 | -0.22 | 0.67 | 2.81E-01 | -0.27 | 0.50 | 571025 | 131002 | 544449 | 101476 | 541634 | 98103 | 547264 | 105663 |
| RP_POS_742_79657a0_496 | RP | POS | 0.50 | 742.7966 | | | | 1.71E-07 | -0.65 | 1.35 | 7.83E-01 | 0.06 | 1.17 | 2.61E-04 | -0.62 | 1.42 | 1.23E-04 | -0.68 | 1.44 | 2352111 | 190748 | 1778953 | 974630 | 1751928 | 998181 | 1805978 | 959863 |
| RP_POS_487_36023a10_71 | RP | POS | 10.71 | 487.3602 | | | | 6.85E-03 | -0.57 | 1.00 | 7.84E-01 | 0.05 | 0.20 | 1.84E-02 | -0.54 | 0.88 | 1.01E-02 | -0.59 | 1.09 | 64763919 | 5597341 | 61580950 | 5445274 | 61430389 | 5493371 | 61731512 | 5448280 |
| RP_POS_443_33395a13_099 | RP | POS | 13.10 | 443.3340 | | | | 7.68E-01 | -0.04 | 0.77 | 7.84E-01 | 0.06 | 0.36 | 9.46E-01 | -0.01 | 0.97 | 6.55E-01 | -0.08 | 0.37 | 4527910 | 588557 | 4482780 | 1116980 | 4451892 | 946727 | 4513667 | 1273779 |

**Supplementary table 1: Metabolomics results** (in asceding order according to p-value from progressive ALD vs  non-progressive ALD comparison, with molecular features with ID_level of 1-2 first)

| | | | | | | | | all_ALD_vs_control | | | Prog_ALD_vs_non-prog_ALD | | | Prog_ALD_vs_control_ALD | | | Non-prog_ALD_vs_control | | | Healthy_control | | all_ALD | | Non-prog_ALD | | Progressive_ALD | |
|---|---|---|---|---|---|---|---|---|---|---|---|---|---|---|---|---|---|---|---|---|---|---|---|---|---|---|---|
| | | | | | | | | t-test | Cohen's | PLS-DA | t-test | Cohen's | PLS-DA | t-test | Cohen's | PLS-DA | t-test | Cohen's | PLS-DA | | | | | | | | |
| Feature_ID | RP_HILIC | POS_NEG | Average.Rt | Average.Mz | Metabolite.name | curated_MSMS | ID_level | p | d | VIP | p | d | VIP | p | d | VIP | p | d | VIP | Mean | SD | Mean | SD | Mean | SD | Mean | SD |
| RP_POS_191_08553a9_449 | RP | POS | 9.45 | 191.0855 | 6-methoxy-4-methyl-2H-chromen-2-one | 191.0847(100) | 3 | 9.77E-01 | 0.00 | 0.30 | 7.84E-01 | 0.06 | 0.39 | 9.04E-01 | 0.03 | 0.29 | 8.01E-01 | -0.03 | 0.53 | 2653271 | 238464 | 2651343 | 509036 | 2637263 | 334860 | 2665423 | 641084 |
| RP_POS_120_97961a10_572 | RP | POS | 10.57 | 120.9796 | | | | 4.45E-01 | -0.17 | 0.48 | 7.84E-01 | -0.05 | 0.20 | 4.16E-01 | -0.20 | 0.57 | 5.54E-01 | -0.14 | 0.39 | 2200995 | 891510 | 2066614 | 748914 | 2087289 | 765540 | 2045939 | 739098 |
| RP_POS_228_19649a8_755 | RP | POS | 8.76 | 228.1965 | | | | 3.53E-02 | -0.37 | 0.59 | 7.84E-01 | 0.06 | 0.46 | 8.85E-02 | -0.34 | 0.60 | 5.76E-02 | -0.40 | 0.76 | 1776981 | 105913 | 1726315 | 143891 | 1722346 | 150610 | 1730284 | 138263 |
| RP_POS_716_79022a0_498 | RP | POS | 0.50 | 716.7902 | | | | 6.39E-04 | -0.45 | 1.18 | 7.84E-01 | -0.06 | 1.20 | 4.11E-03 | -0.48 | 1.24 | 2.29E-02 | -0.42 | 1.24 | 1588079 | 223954 | 1309745 | 689819 | 1328769 | 734323 | 1290722 | 649188 |
| HILIC_POS_460_7677a2_992 | HILIC | POS | 2.992 | 460.7677 | | | | 1.18E-05 | -0.58 | 1.35 | 7.85E-01 | 0.06 | 0.77 | 1.11E-03 | -0.55 | 1.47 | 9.90E-04 | -0.61 | 1.49 | 4236226 | 610629 | 3251842 | 1870609 | 3200311 | 1977063 | 3303372 | 1776392 |
| RP_NEG_766_85376a0_49 | RP | NEG | 0.49 | 766.8538 | | | | 6.61E-08 | 0.65 | 1.36 | 7.85E-01 | -0.06 | 1.20 | 1.46E-04 | 0.62 | 1.45 | 1.32E-04 | 0.68 | 1.44 | 630510 | 218047 | 2021897 | 2360008 | 2086860 | 2471698 | 1956933 | 2266063 |
| RP_POS_411_09268a9_625 | RP | POS | 9.63 | 411.0927 | Artemitin | 411.0932(100) | 3 | 2.15E-01 | 0.25 | 0.66 | 7.85E-01 | -0.05 | 0.48 | 3.20E-01 | 0.23 | 0.50 | 2.17E-01 | 0.28 | 0.73 | 924649 | 203975 | 976609 | 203632 | 982213 | 203856 | 971006 | 205321 |
| RP_POS_150_01347a0_518 | RP | POS | 0.52 | 150.0135 | | | | 5.08E-03 | 0.35 | 1.07 | 7.85E-01 | 0.06 | 1.03 | 4.45E-02 | 0.38 | 1.08 | 3.36E-02 | 0.32 | 1.10 | 296097 | 67446 | 380892 | 272335 | 373397 | 236805 | 388387 | 306031 |
| RP_NEG_226_96593a0_462 | RP | NEG | 0.46 | 226.9659 | | | | 1.30E-06 | -0.65 | 1.30 | 7.85E-01 | -0.06 | 1.16 | 8.16E-05 | -0.68 | 1.41 | 7.37E-04 | -0.62 | 1.35 | 956016 | 124289 | 744228 | 354356 | 753971 | 371876 | 734485 | 339430 |
| HILIC_NEG_200_97098a0_93 | HILIC | NEG | 0.93 | 200.9710 | | | | 3.62E-09 | 0.83 | 1.37 | 7.86E-01 | -0.06 | 1.23 | 3.95E-05 | 0.80 | 1.31 | 2.30E-07 | 0.86 | 1.66 | 164656 | 94692 | 341951 | 222318 | 348029 | 194816 | 335873 | 248657 |
| HILIC_NEG_408_62604a2_947 | HILIC | NEG | 2.947 | 408.6260 | | | | 1.28E-05 | -0.60 | 1.31 | 7.86E-01 | 0.06 | 0.76 | 1.01E-03 | -0.57 | 1.43 | 7.37E-04 | -0.63 | 1.45 | 417689 | 67270 | 321377 | 175510 | 316582 | 183720 | 326171 | 168631 |
| RP_POS_241_17737a7_525 | RP | POS | 7.53 | 241.1774 | | | | 8.98E-01 | 0.02 | 0.28 | 7.86E-01 | 0.06 | 0.19 | 8.16E-01 | 0.05 | 0.27 | 9.85E-01 | 0.00 | 0.21 | 3183123 | 473304 | 3196002 | 549391 | 3180998 | 496889 | 3211007 | 602049 |
| RP_NEG_301_88419a15_686 | RP | NEG | 15.69 | 301.8842 | | | | 2.93E-01 | -0.21 | 0.86 | 7.87E-01 | -0.06 | 0.33 | 2.78E-01 | -0.24 | 0.60 | 4.27E-01 | -0.18 | 1.00 | 1267658 | 472940 | 1164095 | 502302 | 1177786 | 532406 | 1150404 | 475316 |
| RP_POS_206_88542a0_5 | RP | POS | 0.50 | 206.8854 | | | | 8.84E-03 | -0.41 | 1.09 | 7.87E-01 | -0.06 | 1.12 | 1.50E-02 | -0.44 | 1.10 | 5.90E-02 | -0.38 | 1.12 | 1556926 | 432075 | 1269521 | 755912 | 1290066 | 823356 | 1248976 | 689704 |
| RP_POS_280_15436a3_352 | RP | POS | 3.35 | 280.1544 | Likely acylcarnitine | 280.1533(65), 221.0797(5), 85.0288(100) | 3 | 7.72E-01 | -0.06 | 0.42 | 7.87E-01 | 0.06 | 0.58 | 9.05E-01 | -0.03 | 0.30 | 6.80E-01 | -0.08 | 0.38 | 780140 | 801104 | 731402 | 898943 | 706974 | 748727 | 755830 | 1034833 |
| RP_POS_323_25598a10_96 | RP | POS | 10.96 | 323.2560 | | | | 1.06E-02 | -0.42 | 1.06 | 7.88E-01 | 0.06 | 0.96 | 3.57E-02 | -0.39 | 1.03 | 2.98E-02 | -0.45 | 0.99 | 12872053 | 1756994 | 11785379 | 2756855 | 11710659 | 2991885 | 11860098 | 2528407 |
| HILIC_POS_553_40698a2_224 | HILIC | POS | 2.224 | 553.4070 | | | | 3.66E-01 | 0.17 | 0.76 | 7.88E-01 | 0.06 | 0.77 | 3.77E-01 | 0.20 | 0.35 | 4.90E-01 | 0.14 | 0.29 | 1111481 | 309919 | 1171522 | 366521 | 1161592 | 333461 | 1181451 | 400025 |
| RP_POS_530_35797a10_868 | RP | POS | 10.87 | 530.3580 | | | | 1.21E-03 | -0.78 | 1.27 | 7.88E-01 | -0.05 | 0.90 | 1.35E-03 | -0.80 | 1.30 | 3.22E-03 | -0.76 | 1.27 | 2319735 | 989904 | 1661691 | 729982 | 1681431 | 784952 | 1641951 | 677995 |
| RP_NEG_691_12634a9_222 | RP | NEG | 9.22 | 691.1263 | | | | 9.45E-02 | 0.29 | 0.57 | 7.88E-01 | -0.06 | 0.44 | 2.41E-01 | 0.26 | 0.41 | 8.60E-02 | 0.31 | 0.66 | 1485949 | 97749 | 1523672 | 140532 | 1527473 | 116361 | 1519871 | 162272 |
| RP_POS_185_1149a5_77 | RP | POS | 5.77 | 185.1149 | | | | 2.37E-02 | -0.79 | 1.70 | 7.88E-01 | -0.02 | 0.58 | 2.28E-02 | -0.79 | 1.29 | 2.54E-02 | -0.78 | 1.14 | 5864384 | 2562392 | 4783568 | 394868 | 4794243 | 399725 | 4772892 | 393715 |
| RP_POS_280_13837a0_709 | RP | POS | 0.71 | 280.1384 | | | | 4.69E-01 | 0.12 | 0.66 | 7.89E-01 | 0.06 | 0.63 | 4.87E-01 | 0.15 | 0.50 | 6.07E-01 | 0.09 | 0.69 | 412154 | 129725 | 433765 | 188516 | 428677 | 157877 | 438854 | 216388 |
| RP_NEG_320_8082a15_378 | RP | NEG | 15.38 | 320.8082 | | | | 5.62E-01 | -0.10 | 0.42 | 7.89E-01 | -0.06 | 0.72 | 4.86E-01 | -0.13 | 0.56 | 7.39E-01 | -0.07 | 0.23 | 818827 | 68515 | 810008 | 90429 | 812448 | 104467 | 807568 | 74809 |
| RP_POS_421_23212a9_679 | RP | POS | 9.68 | 421.2321 | | | | 9.30E-01 | -0.02 | 0.35 | 7.89E-01 | -0.04 | 0.23 | 8.74E-01 | -0.05 | 0.35 | 9.97E-01 | 0.00 | 0.18 | 537932 | 709752 | 526313 | 410726 | 537366 | 344090 | 515260 | 471367 |
| HILIC_POS_372_15857a0_552 | HILIC | POS | 0.552 | 372.1586 | | | | 1.52E-03 | 0.50 | 0.92 | 7.90E-01 | 0.06 | 0.79 | 1.07E-02 | 0.53 | 1.03 | 7.21E-03 | 0.47 | 1.03 | 69598702 | 30637600 | 94326537 | 52825067 | 92906575 | 45872338 | 95746499 | 59409915 |
| HILIC_NEG_99_92558a2_951 | HILIC | NEG | 2.951 | 99.9256 | | | | 8.05E-01 | 0.05 | 0.78 | 7.90E-01 | -0.05 | 0.53 | 9.11E-01 | 0.03 | 0.21 | 7.36E-01 | 0.08 | 0.52 | 6577330 | 2582111 | 6704527 | 2357222 | 6767867 | 2336636 | 6641188 | 2399652 |
| HILIC_NEG_259_83615a1_362 | HILIC | NEG | 1.362 | 259.8362 | | | | 1.08E-15 | 1.05 | 1.66 | 7.90E-01 | 0.05 | 0.64 | 5.25E-08 | 1.08 | 1.69 | 4.73E-11 | 1.03 | 2.10 | 118365 | 44345 | 266909 | 142357 | 263088 | 118491 | 270731 | 163935 |
| HILIC_POS_87_09224a0_684 | HILIC | POS | 0.684 | 87.0922 | | | | 6.13E-03 | -0.55 | 0.93 | 7.90E-01 | 0.05 | 0.52 | 1.52E-02 | -0.52 | 0.90 | 1.25E-02 | -0.57 | 1.04 | 5154371 | 1014235 | 4557373 | 1083525 | 4528295 | 1175514 | 4586450 | 994211 |
| RP_POS_331_05368a0_62 | RP | POS | 0.62 | 331.0537 | | | | 5.96E-06 | -0.61 | 1.26 | 7.90E-01 | -0.06 | 1.18 | 1.62E-04 | -0.64 | 1.38 | 1.91E-03 | -0.58 | 1.30 | 11020357 | 1957834 | 7935362 | 5537912 | 8083841 | 5926782 | 7786883 | 5176011 |
| RP_POS_534_31622a8_913 | RP | POS | 8.91 | 534.3162 | | | | 1.11E-03 | -0.51 | 1.31 | 7.91E-01 | 0.06 | 0.59 | 3.04E-02 | -0.48 | 1.24 | 4.40E-04 | -0.54 | 1.32 | 671304 | 201211 | 503019 | 351188 | 493611 | 230427 | 512426 | 442612 |
| RP_POS_457_58731a6_606 | RP | POS | 6.61 | 457.5873 | | | | 3.90E-07 | 0.60 | 1.31 | 7.91E-01 | -0.06 | 1.13 | 5.31E-04 | 0.57 | 1.40 | 2.54E-04 | 0.63 | 1.38 | 32406 | 54162 | 1024569 | 1822972 | 1073346 | 1865001 | 975791 | 1797552 |
| RP_NEG_458_86832a0_511 | RP | NEG | 0.51 | 458.8683 | | | | 2.08E-03 | -0.57 | 0.98 | 7.91E-01 | -0.05 | 0.68 | 6.89E-03 | -0.59 | 0.93 | 6.97E-03 | -0.54 | 0.99 | 603624 | 145198 | 502918 | 180736 | 507750 | 163074 | 498087 | 198386 |
| HILIC_POS_206_88622a2_991 | HILIC | POS | 2.991 | 206.8862 | | | | 6.65E-05 | -0.57 | 1.34 | 7.91E-01 | 0.06 | 0.78 | 1.95E-03 | -0.54 | 1.41 | 1.58E-03 | -0.60 | 1.40 | 62798179 | 12147094 | 48513284 | 27022969 | 47791261 | 28506175 | 49235306 | 25723892 |
| HILIC_NEG_372_81213a2_924 | HILIC | NEG | 2.924 | 372.8121 | | | | 2.75E-07 | -0.65 | 1.35 | 7.91E-01 | 0.06 | 0.77 | 1.57E-04 | -0.62 | 1.49 | 2.08E-04 | -0.68 | 1.52 | 1380588 | 136034 | 1058395 | 542107 | 1043912 | 576184 | 1072879 | 511216 |
| HILIC_POS_482_32538a1_83 | HILIC | POS | 1.83 | 482.3254 | | | | 5.80E-02 | 0.32 | 0.83 | 7.91E-01 | 0.06 | 0.58 | 1.16E-01 | 0.35 | 0.53 | 9.58E-02 | 0.29 | 0.65 | 675628 | 223211 | 773741 | 322642 | 765124 | 250486 | 782358 | 383959 |
| HILIC_POS_90_05559a5_648 | HILIC | POS | 5.648 | 90.0556 | | | | 3.71E-01 | -0.18 | 1.00 | 7.91E-01 | -0.05 | 0.62 | 3.74E-01 | -0.20 | 0.85 | 4.87E-01 | -0.15 | 0.96 | 15903732 | 6674281 | 14659507 | 7148113 | 14850151 | 6621596 | 14468863 | 7701564 |
| RP_POS_239_08968a4_735 | RP | POS | 4.74 | 239.0897 | | | | 1.11E-01 | -0.25 | 0.85 | 7.91E-01 | -0.06 | 0.55 | 1.89E-01 | -0.28 | 0.82 | 1.97E-01 | -0.22 | 0.54 | 1753365 | 138959 | 1697475 | 246067 | 1704040 | 203920 | 1690911 | 284011 |
| RP_POS_293_89764a0_448 | RP | POS | 0.45 | 293.8976 | | | | 4.72E-08 | -0.68 | 1.35 | 7.92E-01 | 0.06 | 1.17 | 1.22E-04 | -0.65 | 1.43 | 6.20E-05 | -0.71 | 1.46 | 527335 | 49811 | 379842 | 237184 | 373537 | 242963 | 386146 | 233557 |
| RP_NEG_701_15558a9_55 | RP | NEG | 9.55 | 701.1556 | | | | 1.15E-01 | -0.37 | 0.67 | 7.92E-01 | -0.05 | 0.58 | 1.14E-01 | -0.40 | 0.65 | 1.73E-01 | -0.35 | 0.65 | 9156693 | 989139 | 8850267 | 755633 | 8870340 | 789712 | 8830193 | 727425 |
| RP_POS_315_15643a9_707 | RP | POS | 9.71 | 315.1564 | | | | 1.96E-01 | -0.26 | 0.63 | 7.92E-01 | -0.05 | 0.60 | 1.94E-01 | -0.29 | 0.55 | 3.09E-01 | -0.23 | 0.60 | 4136516 | 138762 | 4099218 | 144940 | 4103068 | 152534 | 4095369 | 138373 |
| RP_POS_503_26544a6_787 | RP | POS | 6.79 | 503.2654 | | | | 2.41E-07 | 0.62 | 1.35 | 7.92E-01 | -0.06 | 1.15 | 7.46E-04 | 0.59 | 1.42 | 9.88E-05 | 0.64 | 1.45 | 14037 | 13518 | 995998 | 1769560 | 1042913 | 1716043 | 949084 | 1837741 |
| HILIC_NEG_221_84229a3_02 | HILIC | NEG | 3.02 | 221.8423 | | | | 3.02E-04 | -0.70 | 1.07 | 7.92E-01 | -0.05 | 0.70 | 9.08E-04 | -0.73 | 1.21 | 1.50E-03 | -0.68 | 1.16 | 744270 | 69891 | 688016 | 78337 | 690092 | 76175 | 685939 | 81161 |
| RP_POS_247_08498a15_339 | RP | POS | 15.34 | 247.0850 | | | | 4.86E-01 | -0.14 | 0.43 | 7.93E-01 | -0.05 | 0.42 | 4.61E-01 | -0.17 | 0.41 | 6.11E-01 | -0.12 | 0.68 | 1812144 | 458508 | 1746747 | 462081 | 1758985 | 460777 | 1734509 | 467732 |
| RP_POS_181_00143a0_45 | RP | POS | 0.45 | 181.0014 | | | | 9.60E-08 | -0.67 | 1.35 | 7.93E-01 | 0.06 | 1.18 | 1.51E-04 | -0.64 | 1.42 | 9.38E-05 | -0.70 | 1.45 | 1683236 | 175204 | 1206658 | 781202 | 1185968 | 806241 | 1227347 | 762961 |
| HILIC_POS_148_13315a1_378 | HILIC | POS | 1.378 | 148.1332 | | | | 4.07E-02 | -0.38 | 1.11 | 7.93E-01 | 0.05 | 0.71 | 8.88E-02 | -0.35 | 1.07 | 6.23E-02 | -0.40 | 1.17 | 875238 | 208477 | 781356 | 256970 | 774554 | 271527 | 788159 | 244116 |
| RP_NEG_638_8183a0_498 | RP | NEG | 0.50 | 638.8183 | | | | 2.67E-04 | -0.48 | 1.22 | 7.93E-01 | -0.06 | 1.21 | 2.67E-03 | -0.51 | 1.29 | 1.24E-02 | -0.45 | 1.26 | 1365401 | 200404 | 1107697 | 589172 | 1123264 | 616639 | 1092130 | 566209 |
| RP_NEG_319_98438a3_698 | RP | NEG | 3.70 | 319.9844 | | | | 8.46E-01 | 0.04 | 0.44 | 7.93E-01 | 0.05 | 0.68 | 7.75E-01 | 0.06 | 0.28 | 9.63E-01 | 0.01 | 0.26 | 1127360 | 385679 | 1143279 | 445644 | 1131517 | 411599 | 1155041 | 481200 |
| HILIC_NEG_194_10326a1_446 | HILIC | NEG | 1.446 | 194.1033 | | | | 2.41E-02 | 0.34 | 0.54 | 7.94E-01 | -0.06 | 0.50 | 1.31E-01 | 0.31 | 0.52 | 2.86E-02 | 0.37 | 0.74 | 377934 | 73290 | 422171 | 143372 | 425947 | 121631 | 418394 | 163424 |
| RP_NEG_199_8046a0_723 | RP | NEG | 0.72 | 199.8046 | | | | 4.07E-01 | -0.17 | 0.76 | 7.94E-01 | -0.05 | 0.32 | 3.93E-01 | -0.20 | 0.44 | 5.28E-01 | -0.14 | 0.66 | 5830706 | 2991143 | 5322152 | 3008155 | 5401353 | 2986889 | 5242951 | 3057508 |
| RP_NEG_333_09457a0_71 | RP | NEG | 0.71 | 333.0946 | | | | 2.76E-01 | 0.17 | 0.62 | 7.94E-01 | -0.06 | 0.65 | 4.75E-01 | 0.14 | 0.58 | 2.77E-01 | 0.20 | 0.74 | 880723 | 296136 | 960727 | 508056 | 974080 | 476264 | 947373 | 542512 |
| HILIC_NEG_116_92847a4_727 | HILIC | NEG | 4.727 | 116.9285 | | | | 2.68E-02 | 0.45 | 0.95 | 7.95E-01 | -0.05 | 0.69 | 5.46E-02 | 0.43 | 0.76 | 3.82E-02 | 0.48 | 0.91 | 10456057 | 2040413 | 11402555 | 2063489 | 11456698 | 2170353 | 11348411 | 1971409 |
| RP_POS_271_22568a9_581 | RP | POS | 9.58 | 271.2257 | | | | 6.74E-01 | 0.09 | 0.82 | 7.95E-01 | -0.05 | 0.78 | 7.85E-01 | 0.06 | 0.56 | 6.23E-01 | 0.12 | 0.80 | 4230314 | 762225 | 4294593 | 701617 | 4312995 | 703090 | 4276190 | 706789 |
| RP_POS_642_86206a0_489 | RP | POS | 0.49 | 642.8621 | | | | 1.40E-07 | 0.63 | 1.35 | 7.95E-01 | -0.06 | 1.20 | 2.37E-04 | 0.60 | 1.43 | 1.77E-04 | 0.66 | 1.44 | 836642 | 246998 | 2358610 | 2657526 | 2428304 | 2760869 | 2288917 | 2576182 |
| HILIC_NEG_146_96136a7_948 | HILIC | NEG | 7.948 | 146.9614 | | | | 1.85E-03 | -0.60 | 1.09 | 7.95E-01 | -0.05 | 0.60 | 2.82E-03 | -0.63 | 1.13 | 9.76E-03 | -0.58 | 1.06 | 2918580 | 834281 | 2347419 | 943294 | 2372157 | 1017472 | 2322681 | 872506 |
| HILIC_NEG_384_93555a4_232 | HILIC | NEG | 4.232 | 384.9356 | | | | 1.41E-01 | 0.24 | 1.04 | 7.95E-01 | -0.06 | 0.86 | 2.70E-01 | 0.21 | 0.64 | 1.82E-01 | 0.27 | 0.95 | 23573087 | 4528036 | 25170717 | 7186956 | 25359106 | 7466600 | 24982327 | 6966795 |
| RP_POS_215_16426a7_185 | RP | POS | 7.19 | 215.1643 | | | | 6.95E-01 | 0.09 | 0.65 | 7.95E-01 | 0.05 | 0.73 | 6.46E-01 | 0.12 | 0.61 | 7.88E-01 | 0.07 | 0.47 | 900069 | 134516 | 910311 | 103742 | 907593 | 102818 | 913029 | 105631 |
| RP_POS_309_20279a9_594 | RP | POS | 9.59 | 309.2028 | | | | 3.07E-01 | 0.21 | 0.64 | 7.95E-01 | 0.05 | 0.29 | 3.14E-01 | 0.24 | 0.49 | 4.04E-01 | 0.19 | 0.61 | 2640441 | 384818 | 2720517 | 372457 | 2710759 | 344378 | 2730275 | 401856 |
| HILIC_POS_376_80582a2_992 | HILIC | POS | 2.992 | 376.8058 | | | | 8.63E-06 | -0.57 | 1.35 | 7.95E-01 | 0.06 | 0.77 | 9.46E-04 | -0.55 | 1.47 | 1.05E-03 | -0.60 | 1.49 | 3035698 | 385878 | 2350462 | 1312025 | 2316129 | 1396150 | 2384794 | 1235438 |
| RP_POS_736_80988a0_497 | RP | POS | 0.50 | 736.8099 | | | | 3.25E-08 | -0.67 | 1.37 | 7.95E-01 | 0.06 | 1.17 | 1.28E-04 | -0.64 | 1.44 | 6.20E-05 | -0.70 | 1.48 | 1669395 | 89930 | 1280523 | 634172 | 1263935 | 648045 | 1297110 | 626125 |
| RP_POS_384_84512a0_513 | RP | POS | 0.51 | 384.8451 | | | | 9.85E-06 | -0.71 | 1.22 | 7.95E-01 | -0.05 | 0.95 | 1.00E-04 | -0.74 | 1.34 | 5.07E-04 | -0.68 | 1.22 | 375434 | 99144 | 262409 | 165437 | 266733 | 171969 | 258085 | 160269 |
| RP_NEG_243_16a8_496 | RP | NEG | 8.50 | 243.1600 | | | | 9.06E-01 | 0.03 | 0.57 | 7.95E-01 | 0.05 | 0.47 | 8.27E-01 | 0.05 | 0.27 | 1.00E+00 | 0.00 | 0.55 | 3400792 | 595835 | 3414871 | 540398 | 3400750 | 564122 | 3428992 | 520940 |
| HILIC_POS_370_29559a0_631 | HILIC | POS | 0.631 | 370.2956 | | | | 7.98E-02 | 0.30 | 0.68 | 7.95E-01 | -0.06 | 0.83 | 1.37E-01 | 0.28 | 0.74 | 1.32E-01 | 0.33 | 0.52 | 7113682 | 4414794 | 8874926 | 6112029 | 9034675 | 7010858 | 8715178 | 5125619 |
| RP_NEG_347_89267a15_686 | RP | NEG | 15.69 | 347.8927 | | | | 4.58E-01 | -0.14 | 0.61 | 7.95E-01 | -0.05 | 0.47 | 4.27E-01 | -0.17 | 0.48 | 6.03E-01 | -0.12 | 0.47 | 1053117 | 314320 | 1003905 | 353247 | 1013130 | 373355 | 994680 | 335480 |
| HILIC_POS_154_15901a5_783 | HILIC | POS | 5.783 | 154.1590 | | | | 2.35E-01 | -0.25 | 0.94 | 7.96E-01 | 0.05 | 0.55 | 3.40E-01 | -0.22 | 0.47 | 2.36E-01 | -0.27 | 0.81 | 7705552 | 1790539 | 7270577 | 1759142 | 7224660 | 1749566 | 7316494 | 1785226 |
| RP_POS_413_20303a11_609 | RP | POS | 11.61 | 413.2030 | | | | 9.86E-01 | 0.00 | 0.68 | 7.96E-01 | 0.05 | 0.66 | 8.78E-01 | 0.03 | 0.54 | 9.14E-01 | -0.02 | 0.80 | 1137617 | 213546 | 1138458 | 266287 | 1131538 | 296524 | 1145379 | 235033 |
| RP_POS_500_27444a10_34 | RP | POS | 10.34 | 500.2744 | | | | 1.80E-03 | 0.54 | 1.13 | 7.97E-01 | -0.05 | 0.43 | 1.77E-02 | 0.52 | 1.04 | 2.29E-03 | 0.57 | 1.19 | 6946859 | 1883323 | 8317824 | 2609359 | 8385619 | 2195530 | 8250028 | 2987764 |
| RP_POS_132_95859a0_675 | RP | POS | 0.68 | 132.9586 | | | | 1.96E-02 | 0.38 | 0.91 | 7.97E-01 | 0.06 | 0.56 | 3.93E-02 | 0.41 | 0.96 | 6.64E-02 | 0.36 | 0.71 | 1223629 | 131718 | 1297451 | 204987 | 1292128 | 201581 | 1302774 | 210248 |
| RP_NEG_235_92621a1_04 | RP | NEG | 1.04 | 235.9262 | | | | 1.18E-01 | 0.25 | 0.68 | 7.97E-01 | -0.06 | 0.38 | 2.52E-01 | 0.22 | 0.65 | 1.51E-01 | 0.28 | 0.64 | 3532520 | 1376445 | 4057438 | 2264816 | 4116145 | 2266923 | 3998731 | 2284183 |
| RP_POS_142_0862a0_708 | RP | POS | 0.71 | 142.0862 | | | | 5.59E-04 | 0.49 | 0.75 | 7.97E-01 | 0.06 | 0.20 | 2.20E-03 | 0.52 | 1.03 | 1.70E-02 | 0.46 | 0.72 | 720233 | 637691 | 1371677 | 1449388 | 1334159 | 1586054 | 1409196 | 1313729 |
| HILIC_NEG_234_81987a5_828 | HILIC | NEG | 5.828 | 234.8199 | | | | 2.92E-01 | -0.23 | 0.57 | 7.97E-01 | 0.05 | 0.70 | 3.91E-01 | -0.21 | 0.50 | 2.85E-01 | -0.26 | 0.41 | 1063405 | 403670 | 978431 | 355778 | 969223 | 354589 | 987638 | 360323 |
| RP_POS_281_17181a9_529 | RP | POS | 9.53 | 281.1718 | | | | 2.83E-02 | -0.48 | 0.90 | 7.97E-01 | -0.05 | 0.60 | 3.10E-02 | -0.50 | 0.92 | 6.07E-02 | -0.45 | 0.80 | 27494738 | 1337795 | 26894769 | 1202871 | 26925895 | 1281780 | 26863644 | 1130659 |
| RP_POS_193_09491a3_143 | RP | POS | 3.14 | 193.0949 | | 193.0948(100), 134.0588(5) | 4 | 3.98E-01 | 0.10 | 0.73 | 7.98E-01 | -0.06 | 0.27 | 6.47E-01 | 0.07 | 0.72 | 4.61E-01 | 0.13 | 0.52 | 2734568 | 4213803 | 5031342 | 25999698 | 5703160 | 27764884 | 4359524 | 24371177 |
| HILIC_POS_458_76917a2_991 | HILIC | POS | 2.991 | 458.7692 | | | | 1.41E-05 | -0.57 | 1.36 | 7.98E-01 | 0.06 | 0.77 | 1.21E-03 | -0.54 | 1.46 | 1.11E-03 | -0.60 | 1.48 | 9989565 | 1453523 | 7679310 | 4427030 | 7565200 | 4671208 | 7793420 | 4213123 |
| RP_POS_338_1499a3_701 | RP | POS | 3.70 | 338.1499 | | | | 1.33E-01 | 0.23 | 0.78 | 7.98E-01 | 0.06 | 0.72 | 1.98E-01 | 0.25 | 0.69 | 2.64E-01 | 0.20 | 0.85 | 19494438 | 4671882 | 21347708 | 9026572 | 21115465 | 8343807 | 21579951 | 9741236 |
| RP_POS_419_31564a11_827 | RP | POS | 11.83 | 419.3156 | | | | 7.39E-01 | 0.05 | 0.33 | 7.99E-01 | 0.06 | 0.55 | 7.13E-01 | 0.08 | 0.26 | 8.92E-01 | 0.02 | 0.62 | 201212983 | 15578599 | 202586698 | 30493857 | 201802736 | 23478060 | 203370660 | 36417772 |
| RP_NEG_178_05038a3_625 | RP | NEG | 3.63 | 178.0504 | | | | 1.79E-02 | -0.51 | 0.90 | 7.99E-01 | 0.05 | 0.78 | 4.58E-02 | -0.49 | 0.79 | 1.82E-02 | -0.54 | 1.11 | 18699386 | 15940152 | 10931462 | 14526944 | 10558497 | 12766504 | 11304427 | 16220464 |
| HILIC_POS_90_05548a5_48 | HILIC | POS | 5.48 | 90.0555 | | | | 1.01E-01 | 0.25 | 0.87 | 7.99E-01 | 0.06 | 0.47 | 1.56E-01 | 0.28 | 0.60 | 2.26E-01 | 0.22 | 0.49 | 369952 | 93323 | 410284 | 179298 | 405685 | 171201 | 414883 | 188677 |
| RP_POS_300_2301a10_395 | RP | POS | 10.40 | 300.2301 | | | | 2.72E-02 | 0.45 | 0.81 | 7.99E-01 | -0.05 | 0.32 | 6.15E-02 | 0.43 | 0.68 | 3.36E-02 | 0.48 | 1.00 | 743947 | 463250 | 957611 | 462612 | 969474 | 451705 | 945748 | 477558 |
| RP_POS_335_25574a10_412 | RP | POS | 10.41 | 335.2557 | | | | 2.84E-03 | -0.52 | 0.98 | 7.99E-01 | 0.05 | 0.71 | 1.04E-02 | -0.49 | 0.99 | 1.07E-02 | -0.54 | 0.99 | 2526499 | 302112 | 2315839 | 424191 | 2304973 | 465341 | 2326704 | 383100 |
| RP_NEG_186_11313a4_74 | RP | NEG | 4.74 | 186.1131 | | | | 6.54E-01 | -0.08 | 0.54 | 8.00E-01 | 0.05 | 0.40 | 8.22E-01 | -0.06 | 0.55 | 5.27E-01 | -0.11 | 0.65 | 2214924 | 2257334 | 1994538 | 2821906 | 1922265 | 1612303 | 2066811 | 3671326 |
| RP_POS_422_80295a0_25 | RP | POS | 0.25 | 422.8030 | | | | 8.41E-01 | 0.04 | 0.34 | 8.00E-01 | -0.05 | 0.38 | 9.40E-01 | 0.02 | 0.21 | 7.75E-01 | 0.07 | 0.38 | 571417 | 76064 | 574463 | 69731 | 576247 | 71602 | 572680 | 68488 |
| HILIC_NEG_364_78018a2_931 | HILIC | NEG | 2.931 | 364.7802 | | | | 1.07E-05 | -0.57 | 1.32 | 8.00E-01 | 0.06 | 0.76 | 1.07E-03 | -0.54 | 1.43 | 1.18E-03 | -0.60 | 1.47 | 235447 | 29197 | 184142 | 99357 | 181601 | 105616 | 186683 | 93688 |
| HILIC_NEG_380_84409a2_91 | HILIC | NEG | 2.91 | 380.8441 | | | | 2.20E-07 | -0.64 | 1.35 | 8.00E-01 | 0.06 | 0.78 | 1.77E-04 | -0.61 | 1.50 | 2.49E-04 | -0.66 | 1.54 | 11467523 | 806319 | 8843108 | 4545137 | 8726935 | 4830331 | 8959281 | 4287015 |
| RP_NEG_287_83652a0_772 | RP | NEG | 0.77 | 287.8365 | | | | 3.71E-10 | 0.88 | 1.35 | 8.00E-01 | 0.05 | 0.43 | 5.14E-06 | 0.91 | 1.42 | 1.45E-07 | 0.85 | 1.68 | 456691 | 678284 | 1831956 | 1619143 | 1790567 | 1387775 | 1873345 | 1835031 |
| HILIC_NEG_189_97369a0_933 | HILIC | NEG | 0.933 | 189.9737 | | | | 1.89E-07 | 0.80 | 1.36 | 8.00E-01 | -0.05 | 1.20 | 1.17E-04 | 0.77 | 1.29 | 2.51E-06 | 0.82 | 1.55 | 352742 | 110864 | 512401 | 207065 | 517686 | 183515 | 507115 | 229984 |
| RP_POS_437_19315a8_659 | RP | POS | 8.66 | 437.1932 | | | | 1.28E-02 | 0.37 | 0.77 | 8.00E-01 | -0.06 | 0.74 | 8.35E-02 | 0.35 | 0.71 | 2.03E-02 | 0.40 | 0.87 | 634445 | 285577 | 824035 | 551960 | 838124 | 492126 | 809945 | 610692 |
| RP_NEG_829_18073a9_56 | RP | NEG | 9.56 | 829.1807 | | | | 4.86E-01 | 0.12 | 0.59 | 8.00E-01 | -0.05 | 0.33 | 6.59E-01 | 0.09 | 0.28 | 4.54E-01 | 0.15 | 0.62 | 6370204 | 345219 | 6424940 | 487881 | 6437386 | 459922 | 6412494 | 518718 |
| RP_POS_633_14893a10_813 | RP | POS | 10.81 | 633.1489 | | | | 1.44E-02 | 0.43 | 0.97 | 8.00E-01 | -0.05 | 0.51 | 2.98E-02 | 0.40 | 0.93 | 4.08E-02 | 0.45 | 1.00 | 379305 | 173266 | 476729 | 238633 | 482818 | 277556 | 470640 | 194783 |

**Supplementary table 1: Metabolomics results** (in asceding order according to p-value from progressive ALD vs non-progressive ALD comparison, with molecular features with ID_level of 1-2 first)

| | | | | | | | | all_ALD_vs_control | | | Prog_ALD_vs_non-prog_ALD | | | Prog_ALD_vs_control_ALD | | | Non-prog_ALD_vs_control | | | Healthy_control | | all_ALD | | Non-prog_ALD | | Progressive_ALD | |
|---|---|---|---|---|---|---|---|---|---|---|---|---|---|---|---|---|---|---|---|---|---|---|---|---|---|---|---|
| | | | | | | | | t-test | Cohen's | PLS-DA | t-test | Cohen's | PLS-DA | t-test | Cohen's | PLS-DA | t-test | Cohen's | PLS-DA | | | | | | | | |
| Feature_ID | RP_HILIC | POS_NEG | Average.Rt | Average.Mz | Metabolite.name | curated_MSMS | ID_level | p | d | VIP | p | d | VIP | p | d | VIP | p | d | VIP | Mean | SD | Mean | SD | Mean | SD | Mean | SD |
| RP_POS_168_98029a10_662 | RP | POS | 10.66 | 168.9803 | | | | 3.62E-01 | 0.21 | 0.49 | 8.00E-01 | -0.05 | 0.75 | 4.62E-01 | 0.18 | 0.31 | 3.41E-01 | 0.23 | 0.52 | 4669548 | 1268851 | 4896469 | 1022414 | 4922529 | 971862 | 4870410 | 1079858 |
| RP_POS_309_12933a0_573 | RP | POS | 0.57 | 309.1293 | | | | 1.01E-03 | 0.40 | 0.85 | 8.00E-01 | 0.06 | 0.70 | 4.16E-02 | 0.43 | 0.79 | 1.36E-03 | 0.37 | 1.20 | 362964 | 44081 | 454429 | 259499 | 447810 | 169622 | 461049 | 327403 |
| HILIC_POS_566_8894a4_324 | HILIC | POS | 4.324 | 566.8894 | | | | 2.05E-03 | 0.41 | 1.23 | 8.01E-01 | -0.06 | 0.87 | 2.00E-02 | 0.38 | 1.28 | 1.96E-02 | 0.44 | 1.40 | 9527712 | 617776 | 10187743 | 1790476 | 10233264 | 1934284 | 10142222 | 1652679 |
| HILIC_NEG_374_81015a2_924 | HILIC | NEG | 2.924 | 374.8102 | | | | 4.67E-07 | -0.63 | 1.34 | 8.01E-01 | 0.06 | 0.76 | 1.97E-04 | -0.61 | 1.49 | 3.03E-04 | -0.66 | 1.51 | 261037 | 25492 | 200607 | 104336 | 197961 | 111393 | 203253 | 97834 |
| RP_NEG_319_92706a6_867 | RP | NEG | 6.87 | 319.9271 | | | | 7.47E-02 | -0.36 | 0.65 | 8.01E-01 | 0.05 | 0.73 | 1.28E-01 | -0.33 | 0.62 | 9.43E-02 | -0.39 | 0.59 | 1815137 | 220510 | 1733064 | 227906 | 1727287 | 241273 | 1738842 | 216013 |
| HILIC_NEG_452_92413a4_232 | HILIC | NEG | 4.232 | 452.9241 | | | | 1.32E-01 | 0.24 | 1.04 | 8.02E-01 | -0.05 | 0.86 | 2.56E-01 | 0.22 | 0.65 | 1.76E-01 | 0.27 | 0.98 | 16234313 | 3136986 | 17373652 | 5041972 | 17501294 | 5257320 | 17246011 | 4867145 |
| RP_POS_419_31528a10_573 | RP | POS | 10.57 | 419.3153 | | | | 8.45E-01 | 0.04 | 0.26 | 8.02E-01 | 0.05 | 0.16 | 7.75E-01 | 0.07 | 0.24 | 9.39E-01 | 0.02 | 0.60 | 1082347 | 396551 | 1097665 | 339512 | 1089094 | 366198 | 1106236 | 314067 |
| RP_POS_372_88251a0_471 | RP | POS | 0.47 | 372.8825 | | | | 5.80E-07 | -0.63 | 1.32 | 8.02E-01 | 0.06 | 1.18 | 3.23E-04 | -0.61 | 1.39 | 2.28E-04 | -0.66 | 1.41 | 1054818 | 113846 | 797580 | 445120 | 786361 | 461379 | 808799 | 432641 |
| RP_POS_151_00874a0_45 | RP | POS | 0.45 | 151.0087 | | | | 1.68E-06 | -0.64 | 1.31 | 8.03E-01 | -0.05 | 1.13 | 1.40E-04 | -0.67 | 1.38 | 6.25E-04 | -0.61 | 1.37 | 968916 | 130422 | 750742 | 368376 | 760011 | 377284 | 741472 | 362840 |
| HILIC_NEG_80_91625a1_363 | HILIC | NEG | 1.363 | 80.9163 | | | | 3.77E-06 | 0.62 | 1.01 | 8.03E-01 | 0.05 | 0.70 | 1.30E-03 | 0.64 | 1.00 | 9.91E-05 | 0.59 | 1.22 | 1522265 | 385391 | 2158016 | 1125813 | 2129689 | 922837 | 2186344 | 1306717 |
| RP_POS_193_13132a5_931 | RP | POS | 5.93 | 193.1313 | | | | 2.74E-01 | -0.18 | 1.04 | 8.03E-01 | -0.05 | 1.06 | 2.60E-01 | -0.21 | 0.90 | 4.66E-01 | -0.15 | 1.07 | 2745836 | 733587 | 2556570 | 1128845 | 2584968 | 1253809 | 2528173 | 1000452 |
| RP_POS_387_20505a10_173 | RP | POS | 10.17 | 387.2051 | | | | 6.80E-01 | -0.08 | 0.78 | 8.03E-01 | 0.05 | 0.73 | 8.05E-01 | -0.05 | 0.58 | 6.40E-01 | -0.10 | 0.83 | 1208539 | 201052 | 1190879 | 236224 | 1184940 | 251935 | 1196818 | 221808 |
| HILIC_POS_450_73837a3_007 | HILIC | POS | 3.007 | 450.7384 | | | | 4.07E-05 | -0.54 | 1.36 | 8.03E-01 | 0.06 | 0.79 | 2.17E-03 | -0.52 | 1.48 | 1.91E-03 | -0.57 | 1.46 | 965571 | 151611 | 744193 | 446747 | 732979 | 471082 | 755406 | 425516 |
| HILIC_NEG_144_92349a7_425 | HILIC | NEG | 7.425 | 144.9235 | | | | 3.31E-01 | 0.18 | 0.68 | 8.03E-01 | 0.05 | 0.34 | 3.35E-01 | 0.21 | 0.59 | 4.68E-01 | 0.15 | 0.82 | 34829112 | 8919411 | 36706073 | 10877475 | 36433074 | 10835349 | 36979073 | 11022556 |
| HILIC_POS_124_92298a2_992 | HILIC | POS | 2.992 | 124.9230 | | | | 5.72E-07 | -0.63 | 1.37 | 8.03E-01 | 0.05 | 0.78 | 2.15E-04 | -0.61 | 1.52 | 3.22E-04 | -0.66 | 1.52 | 23623814 | 2200441 | 18611428 | 8680757 | 18393615 | 9261152 | 18829241 | 8147588 |
| RP_POS_416_78769a13_869 | RP | POS | 13.87 | 416.7877 | | | | 1.90E-01 | -0.26 | 0.49 | 8.04E-01 | 0.05 | 0.51 | 3.02E-01 | -0.24 | 0.59 | 1.88E-01 | -0.29 | 0.50 | 2024317 | 597810 | 1861808 | 620253 | 1846259 | 580059 | 1877358 | 663563 |
| HILIC_POS_154_15889a5_921 | HILIC | POS | 5.921 | 154.1589 | | | | 1.42E-02 | -0.53 | 1.22 | 8.04E-01 | -0.05 | 0.70 | 1.70E-02 | -0.55 | 1.17 | 3.45E-02 | -0.50 | 1.16 | 6967396 | 1843255 | 6033652 | 1693886 | 6076096 | 1789463 | 5991207 | 1609721 |
| RP_NEG_146_96024a0_448 | RP | NEG | 0.45 | 146.9602 | | | | 7.30E-08 | -0.67 | 1.35 | 8.04E-01 | 0.05 | 1.18 | 1.40E-04 | -0.64 | 1.43 | 8.30E-05 | -0.70 | 1.45 | 1452960 | 143612 | 1040426 | 673058 | 1023572 | 691698 | 1057280 | 660480 |
| RP_POS_866_526a13_713 | RP | POS | 13.71 | 866.5260 | | | | 8.64E-05 | 0.66 | 1.38 | 8.04E-01 | -0.05 | 0.57 | 7.61E-04 | 0.64 | 1.33 | 8.85E-04 | 0.69 | 1.23 | 726854 | 372533 | 1083100 | 553091 | 1096929 | 597451 | 1069270 | 510613 |
| RP_NEG_425_91077a15_704 | RP | NEG | 15.70 | 425.9108 | | | | 4.85E-01 | -0.14 | 0.79 | 8.04E-01 | -0.05 | 0.37 | 4.73E-01 | -0.16 | 0.46 | 6.05E-01 | -0.11 | 0.82 | 1374021 | 353701 | 1322691 | 377870 | 1332133 | 360201 | 1313250 | 398193 |
| HILIC_POS_159_02786a1_385 | HILIC | POS | 1.385 | 159.0279 | | | | 9.91E-14 | 0.92 | 1.59 | 8.04E-01 | -0.05 | 0.63 | 8.04E-07 | 0.89 | 1.57 | 2.87E-08 | 0.94 | 2.01 | 1035984 | 570334 | 8680159 | 8822465 | 8900517 | 8431090 | 8459801 | 9277818 |
| RP_POS_408_16074a4_144 | RP | POS | 4.14 | 408.1607 | | | | 7.81E-01 | 0.04 | 0.32 | 8.05E-01 | 0.06 | 0.22 | 6.83E-01 | 0.06 | 0.43 | 9.67E-01 | 0.01 | 0.74 | 2007522 | 295231 | 2037214 | 931122 | 2014005 | 1028425 | 2060422 | 832415 |
| RP_POS_390_21472a10_733 | RP | POS | 10.73 | 390.2147 | | | | 1.40E-02 | 0.40 | 1.16 | 8.05E-01 | 0.05 | 1.09 | 4.66E-02 | 0.42 | 1.01 | 3.47E-02 | 0.37 | 1.21 | 414197 | 164121 | 513072 | 267128 | 506413 | 223475 | 519730 | 306823 |
| HILIC_POS_171_18552a5_566 | HILIC | POS | 5.566 | 171.1855 | | | | 4.20E-01 | -0.11 | 0.76 | 8.05E-01 | -0.06 | 0.73 | 5.09E-01 | -0.14 | 0.21 | 5.59E-01 | -0.08 | 0.20 | 98172906 | 4795114 | 96955241 | 12423004 | 97264809 | 9148807 | 96645673 | 15096868 |
| RP_POS_627_26166a7_624 | RP | POS | 7.62 | 627.2617 | | | | 2.47E-01 | -0.22 | 0.46 | 8.05E-01 | -0.05 | 0.68 | 2.62E-01 | -0.25 | 0.48 | 3.65E-01 | -0.19 | 0.40 | 1675612 | 180832 | 1630845 | 210624 | 1636088 | 207161 | 1625602 | 216005 |
| RP_POS_339_16129a10_978 | RP | POS | 10.98 | 339.1613 | | | | 3.26E-02 | 0.40 | 1.44 | 8.05E-01 | -0.05 | 1.16 | 9.32E-02 | 0.37 | 1.23 | 3.85E-02 | 0.42 | 1.29 | 9427085 | 2488253 | 10597519 | 3055429 | 10673566 | 2792333 | 10521471 | 3324589 |
| HILIC_NEG_300_87851a2_911 | HILIC | NEG | 2.911 | 300.8785 | | | | 4.51E-05 | -0.60 | 1.34 | 8.05E-01 | 0.05 | 0.76 | 1.14E-03 | -0.58 | 1.48 | 1.12E-03 | -0.63 | 1.46 | 284220 | 56895 | 219060 | 115602 | 216183 | 122769 | 221937 | 109139 |
| HILIC_NEG_195_81087a4_1 | HILIC | NEG | 4.1 | 195.8109 | | | | 5.49E-01 | -0.14 | 0.71 | 8.05E-01 | 0.05 | 0.66 | 6.45E-01 | -0.12 | 0.72 | 5.14E-01 | -0.16 | 0.58 | 362408 | 445429 | 310419 | 347062 | 301790 | 339601 | 319049 | 357605 |
| RP_POS_367_9902a0_639 | RP | POS | 0.64 | 367.9902 | | | | 3.54E-10 | -0.77 | 1.43 | 8.05E-01 | 0.05 | 1.17 | 1.22E-05 | -0.75 | 1.53 | 5.87E-06 | -0.80 | 1.53 | 4574560 | 314795 | 3175193 | 1957422 | 3126529 | 1994110 | 3223858 | 1939043 |
| HILIC_POS_126_92099a2_993 | HILIC | POS | 2.993 | 126.9210 | | | | 1.33E-06 | -0.61 | 1.36 | 8.05E-01 | 0.05 | 0.79 | 3.54E-04 | -0.59 | 1.52 | 4.58E-04 | -0.64 | 1.52 | 821894 | 84660 | 641674 | 321953 | 633670 | 342200 | 649678 | 303629 |
| HILIC_NEG_130_92897a4_279 | HILIC | NEG | 4.279 | 130.9290 | | | | 9.61E-01 | -0.01 | 0.75 | 8.05E-01 | 0.05 | 0.82 | 9.43E-01 | 0.02 | 0.07 | 8.74E-01 | -0.04 | 0.17 | 1177235 | 284805 | 1174290 | 312796 | 1166515 | 314426 | 1182066 | 314155 |
| RP_POS_223_09406a13_263 | RP | POS | 13.26 | 223.0941 | | | | 6.81E-01 | -0.08 | 0.53 | 8.05E-01 | -0.05 | 0.49 | 6.31E-01 | -0.11 | 0.45 | 7.98E-01 | -0.06 | 0.88 | 13381343 | 4157544 | 13030868 | 4237090 | 13136152 | 4312538 | 12925583 | 4201386 |
| RP_POS_631_40112a15_097 | RP | POS | 15.10 | 631.4011 | | | | 9.53E-01 | 0.01 | 0.33 | 8.05E-01 | -0.05 | 0.33 | 9.53E-01 | -0.01 | 0.42 | 8.69E-01 | 0.04 | 0.48 | 7964920 | 1964061 | 7989039 | 2049098 | 8039935 | 2062678 | 7938142 | 2055082 |
| RP_POS_630_85986a6_463 | RP | POS | 6.46 | 630.8599 | | | | 6.16E-07 | 0.59 | 1.32 | 8.05E-01 | -0.06 | 1.14 | 9.38E-04 | 0.57 | 1.40 | 2.17E-04 | 0.62 | 1.40 | 50652 | 96790 | 1650403 | 2997153 | 1724819 | 2961339 | 1575987 | 3060776 |
| HILIC_POS_402_35745a0_526 | HILIC | POS | 0.526 | 402.3575 | | | | 5.41E-01 | 0.10 | 0.65 | 8.06E-01 | -0.05 | 0.56 | 7.05E-01 | 0.08 | 0.50 | 5.20E-01 | 0.13 | 0.33 | 129932 | 151635 | 151484 | 227197 | 157119 | 229443 | 145848 | 227115 |
| RP_NEG_311_16943a10_051 | RP | NEG | 10.05 | 311.1694 | | | | 6.66E-01 | 0.08 | 0.63 | 8.06E-01 | 0.05 | 0.42 | 6.17E-01 | 0.11 | 0.37 | 7.98E-01 | 0.06 | 0.18 | 12747189 | 12269188 | 13880623 | 14501660 | 13520921 | 14803841 | 14240324 | 14334237 |
| HILIC_POS_542_73163a2_992 | HILIC | POS | 2.992 | 542.7316 | | | | 2.64E-05 | -0.56 | 1.35 | 8.06E-01 | 0.05 | 0.77 | 1.57E-03 | -0.54 | 1.46 | 1.44E-03 | -0.59 | 1.47 | 7695778 | 1218874 | 5918088 | 3461645 | 5832267 | 3651484 | 6003910 | 3295780 |
| HILIC_NEG_108_90194a2_948 | HILIC | NEG | 2.948 | 108.9019 | | | | 5.56E-07 | -0.62 | 1.34 | 8.06E-01 | 0.05 | 0.77 | 2.37E-04 | -0.59 | 1.47 | 3.91E-04 | -0.65 | 1.52 | 28040867 | 1803003 | 23177381 | 8624638 | 22963772 | 9229056 | 23390989 | 8063388 |
| RP_POS_486_13574a8_699 | RP | POS | 8.70 | 486.1357 | | | | 2.25E-01 | 0.19 | 0.87 | 8.06E-01 | -0.05 | 0.24 | 4.25E-01 | 0.16 | 0.56 | 2.19E-01 | 0.22 | 0.72 | 1893763 | 717433 | 2109604 | 1227736 | 2140003 | 1082010 | 2079205 | 1368503 |
| RP_POS_393_29587a10_987 | RP | POS | 10.99 | 393.2959 | | | | 6.41E-01 | -0.09 | 0.58 | 8.06E-01 | 0.05 | 0.60 | 7.58E-01 | -0.07 | 0.60 | 6.03E-01 | -0.12 | 0.54 | 2125465 | 370761 | 2089830 | 385353 | 2080298 | 397503 | 2099361 | 376603 |
| RP_NEG_343_99524a15_554 | RP | NEG | 15.55 | 343.9952 | | | | 8.39E-06 | -0.67 | 1.31 | 8.06E-01 | 0.05 | 1.20 | 4.12E-04 | -0.64 | 1.36 | 2.88E-04 | -0.69 | 1.37 | 1169286 | 252545 | 856237 | 496922 | 843962 | 516131 | 868512 | 481874 |
| RP_POS_229_11835a0_621 | RP | POS | 0.62 | 229.1184 | | | | 9.49E-01 | 0.01 | 0.65 | 8.06E-01 | -0.05 | 0.73 | 9.40E-01 | -0.02 | 0.64 | 8.14E-01 | 0.04 | 0.41 | 382002 | 165418 | 384632 | 282661 | 391613 | 199264 | 377651 | 348739 |
| RP_POS_146_93704a0_251 | RP | POS | 0.25 | 146.9370 | | | | 2.76E-01 | 0.22 | 0.43 | 8.07E-01 | -0.05 | 0.25 | 3.81E-01 | 0.19 | 0.38 | 2.86E-01 | 0.24 | 0.45 | 7206791 | 693870 | 7363790 | 726434 | 7381705 | 757554 | 7345874 | 701169 |
| RP_POS_463_37509a11_958 | RP | POS | 11.96 | 463.3751 | | | | 1.36E-01 | -0.24 | 0.79 | 8.07E-01 | 0.05 | 0.77 | 2.65E-01 | -0.21 | 0.73 | 1.83E-01 | -0.26 | 0.77 | 504546 | 134650 | 455076 | 226609 | 449491 | 235619 | 460662 | 219480 |
| HILIC_NEG_153_86855a2_949 | HILIC | NEG | 2.949 | 153.8686 | | | | 1.64E-03 | 0.45 | 1.14 | 8.07E-01 | -0.05 | 0.79 | 1.63E-02 | 0.42 | 0.98 | 1.12E-02 | 0.48 | 1.20 | 14060125 | 2665180 | 16496966 | 5908285 | 16642502 | 6176781 | 16351431 | 5686142 |
| HILIC_NEG_144_92342a5_882 | HILIC | NEG | 5.882 | 144.9234 | | | | 9.44E-01 | -0.02 | 0.23 | 8.08E-01 | -0.05 | 0.34 | 8.74E-01 | -0.04 | 0.19 | 9.75E-01 | 0.01 | 0.08 | 33691820 | 9659433 | 33559035 | 8036399 | 33755970 | 7862688 | 33362099 | 8281573 |
| RP_NEG_787_10187a9_222 | RP | NEG | 9.22 | 787.1019 | | | | 9.41E-01 | -0.01 | 0.34 | 8.08E-01 | -0.05 | 0.57 | 8.53E-01 | -0.04 | 0.26 | 9.56E-01 | 0.01 | 0.09 | 5132718 | 332834 | 5127403 | 402547 | 5137263 | 408878 | 5117542 | 400021 |
| RP_POS_455_18875a7_265 | RP | POS | 7.27 | 455.1888 | | | | 4.01E-01 | -0.16 | 0.47 | 8.08E-01 | -0.05 | 0.54 | 3.98E-01 | -0.19 | 0.35 | 5.28E-01 | -0.14 | 0.39 | 791807 | 74551 | 778579 | 84176 | 780640 | 82625 | 776519 | 86489 |
| HILIC_NEG_153_86879a6_295 | HILIC | NEG | 6.295 | 153.8688 | | | | 4.60E-03 | -0.66 | 1.21 | 8.08E-01 | -0.04 | 0.19 | 5.73E-03 | -0.68 | 1.14 | 1.06E-02 | -0.63 | 1.26 | 1660276 | 679658 | 1266909 | 540846 | 1280126 | 560100 | 1253692 | 526242 |
| RP_NEG_311_16962a9_368 | RP | NEG | 9.37 | 311.1696 | | | | 6.16E-01 | 0.09 | 0.63 | 8.09E-01 | -0.05 | 0.16 | 7.42E-01 | 0.07 | 0.58 | 5.92E-01 | 0.12 | 0.53 | 2848740 | 3889544 | 3269428 | 4716737 | 3384430 | 5083432 | 3154426 | 4368178 |
| RP_POS_223_20511a8_812 | RP | POS | 8.81 | 223.2051 | | | | 8.66E-01 | 0.03 | 0.39 | 8.09E-01 | 0.05 | 0.56 | 7.89E-01 | 0.06 | 0.31 | 9.76E-01 | 0.01 | 0.34 | 511326 | 234827 | 519805 | 277023 | 513051 | 282955 | 526558 | 273665 |
| RP_POS_418_74771a13_069 | RP | POS | 13.07 | 418.7477 | | | | 8.58E-02 | -0.32 | 1.30 | 8.09E-01 | -0.05 | 0.27 | 1.03E-01 | -0.34 | 0.96 | 1.70E-01 | -0.29 | 1.16 | 16169930 | 2556182 | 15213787 | 3095524 | 15289210 | 3169216 | 15138364 | 3050336 |
| RP_POS_532_61633a6_895 | RP | POS | 6.90 | 532.6163 | | | | 2.08E-07 | 0.62 | 1.32 | 8.09E-01 | -0.05 | 1.14 | 3.85E-04 | 0.59 | 1.43 | 1.86E-04 | 0.65 | 1.39 | 19276 | 24866 | 863408 | 1511628 | 900207 | 1540500 | 826609 | 1496920 |
| RP_POS_469_36566a12_33 | RP | POS | 12.33 | 469.3657 | | | | 6.80E-01 | 0.09 | 0.81 | 8.09E-01 | -0.05 | 0.36 | 7.96E-01 | 0.06 | 0.63 | 6.18E-01 | 0.11 | 0.37 | 2840137 | 866960 | 2912011 | 811186 | 2931738 | 701121 | 2892284 | 914937 |
| HILIC_NEG_588_89764a4_232 | HILIC | NEG | 4.232 | 588.8976 | | | | 8.84E-04 | 0.43 | 1.24 | 8.10E-01 | -0.05 | 0.89 | 1.39E-02 | 0.40 | 1.29 | 1.39E-02 | 0.45 | 1.42 | 5921665 | 367695 | 6414879 | 1294827 | 6446303 | 1390033 | 6383455 | 1205471 |
| RP_POS_405_3335a11_419 | RP | POS | 11.42 | 405.3335 | | | | 4.07E-02 | -0.38 | 0.96 | 8.10E-01 | -0.05 | 0.92 | 3.93E-02 | -0.40 | 0.96 | 1.21E-01 | -0.35 | 0.93 | 1139309 | 243356 | 1029587 | 301263 | 1036896 | 347646 | 1022277 | 249807 |
| RP_POS_930_18707a0_621 | RP | POS | 0.62 | 930.1871 | | | | 3.79E-01 | -0.17 | 0.77 | 8.10E-01 | -0.05 | 0.79 | 3.55E-01 | -0.20 | 0.65 | 5.17E-01 | -0.15 | 0.77 | 421466 | 226254 | 380068 | 241072 | 385913 | 262673 | 374223 | 219887 |
| RP_POS_818_56561a13_395 | RP | POS | 13.40 | 818.5656 | | | | 1.38E-02 | 0.45 | 0.74 | 8.10E-01 | 0.05 | 0.49 | 1.55E-02 | 0.48 | 0.95 | 5.54E-02 | 0.43 | 0.69 | 2933860 | 1196376 | 3588920 | 1488756 | 3552831 | 1682494 | 3625010 | 1282409 |
| RP_POS_279_91782a0_448 | RP | POS | 0.45 | 279.9178 | | | | 3.43E-08 | -0.68 | 1.36 | 8.10E-01 | 0.05 | 1.17 | 9.22E-05 | -0.66 | 1.45 | 6.60E-05 | -0.71 | 1.46 | 529739 | 47364 | 377885 | 243125 | 372001 | 251075 | 383770 | 237311 |
| HILIC_NEG_99_92554a4_207 | HILIC | NEG | 4.207 | 99.9255 | | | | 8.18E-01 | 0.04 | 0.69 | 8.11E-01 | 0.05 | 0.35 | 7.49E-01 | 0.07 | 0.71 | 9.34E-01 | 0.02 | 0.69 | 1025731 | 135724 | 1032383 | 159954 | 1028523 | 164146 | 1036243 | 157222 |
| RP_POS_676_85571a0_489 | RP | POS | 0.49 | 676.8557 | | | | 1.07E-07 | 0.64 | 1.35 | 8.11E-01 | -0.05 | 1.20 | 2.06E-04 | 0.61 | 1.43 | 1.60E-04 | 0.66 | 1.44 | 794072 | 240190 | 2487609 | 2932246 | 2558297 | 3039878 | 2416920 | 2849658 |
| HILIC_NEG_112_98579a5_429 | HILIC | NEG | 5.429 | 112.9858 | | | | 3.24E-01 | -0.20 | 0.63 | 8.11E-01 | 0.05 | 1.05 | 4.49E-01 | -0.18 | 0.30 | 3.10E-01 | -0.22 | 0.92 | 11876436 | 1132912 | 11646349 | 1153223 | 11618568 | 1078433 | 11674131 | 1233854 |
| RP_POS_163_97684a0_251 | RP | POS | 0.25 | 163.9768 | | | | 5.93E-01 | -0.11 | 0.55 | 8.11E-01 | -0.05 | 0.72 | 5.40E-01 | -0.13 | 0.37 | 7.27E-01 | -0.08 | 0.37 | 22973623 | 4609787 | 22461895 | 4912961 | 22580225 | 5446743 | 22343566 | 4367079 |
| RP_NEG_344_86771a15_682 | RP | NEG | 15.68 | 344.8677 | | | | 3.17E-02 | -0.46 | 0.84 | 8.11E-01 | 0.05 | 0.22 | 6.03E-02 | -0.44 | 0.84 | 4.01E-02 | -0.48 | 0.79 | 894560 | 320111 | 753509 | 294136 | 746427 | 299485 | 760592 | 291554 |
| RP_POS_315_25217a9_594 | RP | POS | 9.59 | 315.2522 | | | | 1.86E-01 | 0.25 | 0.72 | 8.11E-01 | 0.05 | 0.36 | 2.14E-01 | 0.28 | 0.55 | 2.81E-01 | 0.23 | 0.70 | 1325446 | 183092 | 1377030 | 209738 | 1371984 | 198090 | 1382076 | 222679 |
| HILIC_NEG_186_82896a2_951 | HILIC | NEG | 2.951 | 186.8290 | | | | 6.46E-07 | -0.64 | 1.33 | 8.11E-01 | 0.05 | 0.77 | 2.11E-04 | -0.62 | 1.45 | 2.65E-04 | -0.67 | 1.51 | 668617 | 71853 | 524393 | 244445 | 518518 | 258682 | 530267 | 231817 |
| HILIC_NEG_278_82208a2_932 | HILIC | NEG | 2.932 | 278.8221 | | | | 6.40E-07 | -0.63 | 1.34 | 8.12E-01 | 0.05 | 0.76 | 2.04E-04 | -0.61 | 1.48 | 3.50E-04 | -0.66 | 1.50 | 1053942 | 107965 | 815798 | 412231 | 805894 | 441406 | 825702 | 385094 |
| RP_POS_1022_73462a0_496 | RP | POS | 0.50 | 1022.7346 | | | | 4.04E-07 | -0.63 | 1.32 | 8.12E-01 | 0.05 | 1.16 | 3.47E-04 | -0.60 | 1.38 | 2.23E-04 | -0.65 | 1.42 | 1928643 | 132041 | 1551172 | 663780 | 1535227 | 683916 | 1567116 | 649569 |
| RP_NEG_215_12943a7_471 | RP | NEG | 7.47 | 215.1294 | | | | 8.47E-01 | -0.04 | 0.58 | 8.12E-01 | 0.05 | 0.47 | 9.34E-01 | -0.02 | 0.33 | 7.90E-01 | -0.07 | 0.47 | 2770098 | 574549 | 2748286 | 488114 | 2736563 | 517721 | 2760010 | 461581 |
| RP_POS_277_88074a0_246 | RP | POS | 0.25 | 277.8807 | | | | 5.37E-01 | 0.13 | 0.33 | 8.12E-01 | -0.05 | 0.15 | 6.41E-01 | 0.11 | 0.30 | 5.14E-01 | 0.16 | 0.63 | 1274463 | 152192 | 1293326 | 140288 | 1296695 | 145957 | 1289957 | 135780 |
| RP_POS_181_04898a9_441 | RP | POS | 9.44 | 181.0490 | fragment fo Di-n-butyl phtalate | 149.0226(100), 163.0377(45), 84.9603(80) | 3 | 6.66E-01 | 0.09 | 0.37 | 8.12E-01 | 0.05 | 0.84 | 6.28E-01 | 0.11 | 0.71 | 7.71E-01 | 0.07 | 0.31 | 1460514 | 126185 | 1471510 | 120673 | 1468613 | 116090 | 1474408 | 126202 |
| HILIC_NEG_255_82204a2_946 | HILIC | NEG | 2.946 | 255.8220 | | | | 1.63E-03 | 0.44 | 1.11 | 8.12E-01 | -0.05 | 0.79 | 1.90E-02 | 0.41 | 0.95 | 1.15E-02 | 0.47 | 1.13 | 3868370 | 603482 | 4449607 | 1454349 | 4484463 | 1499585 | 4414751 | 1422040 |
| RP_NEG_829_18103a9_838 | RP | NEG | 9.84 | 829.1810 | | | | 5.63E-01 | -0.12 | 0.44 | 8.12E-01 | 0.05 | 0.55 | 6.76E-01 | -0.09 | 0.26 | 5.40E-01 | -0.14 | 0.45 | 5252076 | 333291 | 5212435 | 341006 | 5204272 | 357637 | 5220598 | 326962 |
| RP_POS_248_97632a0_453 | RP | POS | 0.45 | 248.9763 | | | | 1.81E-04 | -0.55 | 1.21 | 8.12E-01 | -0.05 | 1.08 | 1.24E-03 | -0.57 | 1.24 | 6.30E-03 | -0.52 | 1.26 | 624932 | 148513 | 467323 | 310529 | 474754 | 328800 | 459892 | 294283 |
| HILIC_POS_247_09232a7_688 | HILIC | POS | 7.688 | 247.0923 | | | | 5.59E-01 | 0.11 | 0.37 | 8.13E-01 | -0.05 | 0.52 | 6.95E-01 | 0.09 | 0.19 | 5.25E-01 | 0.14 | 0.26 | 869122 | 71654 | 877999 | 82594 | 879972 | 80040 | 876026 | 85838 |
| RP_NEG_310_93307a15_326 | RP | NEG | 15.33 | 310.9331 | | | | 7.84E-01 | 0.05 | 0.61 | 8.13E-01 | -0.05 | 0.20 | 9.03E-01 | 0.02 | 0.61 | 7.31E-01 | 0.07 | 0.33 | 2158899 | 69869 | 2163069 | 87977 | 2165170 | 94128 | 2160969 | 82273 |
| HILIC_NEG_262_75821a2_947 | HILIC | NEG | 2.947 | 262.7582 | | | | 7.64E-07 | -0.67 | 1.35 | 8.13E-01 | 0.05 | 0.76 | 1.30E-04 | -0.64 | 1.48 | 2.25E-04 | -0.69 | 1.47 | 296545 | 44288 | 221740 | 120623 | 218861 | 129250 | 224619 | 112580 |
| RP_POS_1047_73303a10_828 | RP | POS | 10.83 | 1047.7330 | | | | 1.81E-03 | 0.56 | 1.21 | 8.13E-01 | 0.05 | 0.91 | 9.25E-03 | 0.59 | 1.00 | 4.22E-03 | 0.54 | 1.27 | 689383 | 554864 | 1084806 | 721241 | 1067597 | 578651 | 1102016 | 845904 |
| HILIC_NEG_120_92785a2_931 | HILIC | NEG | 2.931 | 120.9279 | | | | 1.84E-04 | -0.56 | 1.33 | 8.13E-01 | 0.05 | 0.77 | 2.96E-03 | -0.53 | 1.36 | 2.47E-03 | -0.59 | 1.33 | 5246855 | 1023544 | 4196044 | 2017588 | 4147923 | 2125873 | 4244165 | 1923626 |
| HILIC_POS_522_67371a3_013 | HILIC | POS | 3.013 | 522.6737 | | | | 3.18E-05 | -0.60 | 1.32 | 8.13E-01 | -0.05 | 0.76 | 4.46E-04 | -0.62 | 1.45 | 2.43E-03 | -0.57 | 1.41 | 271642 | 52794 | 206692 | 117401 | 209490 | 123247 | 203894 | 112434 |
| HILIC_NEG_160_84204a3_002 | HILIC | NEG | 3.002 | 160.8420 | | | | 4.84E-01 | 0.14 | 0.50 | 8.13E-01 | 0.05 | 0.64 | 4.43E-01 | 0.16 | 0.35 | 6.29E-01 | 0.11 | 0.19 | 43605422 | 6738492 | 44598781 | 7541300 | 44419349 | 8372559 | 44778213 | 6688617 |
| RP_POS_197_05724a6_418 | RP | POS | 6.42 | 197.0572 | | | | 1.04E-01 | -0.31 | 0.52 | 8.14E-01 | 0.05 | 0.24 | 1.94E-01 | -0.28 | 0.49 | 1.20E-01 | -0.33 | 0.53 | 650345 | 88761 | 619274 | 104194 | 616801 | 102057 | 621746 | 107268 |
| HILIC_NEG_286_85382a2_924 | HILIC | NEG | 2.924 | 286.8538 | | | | 7.42E-07 | -0.62 | 1.35 | 8.14E-01 | 0.05 | 0.77 | 2.55E-04 | -0.59 | 1.49 | 4.18E-04 | -0.65 | 1.52 | 4005809 | 358201 | 3138190 | 1538086 | 3101713 | 1643887 | 3174667 | 1440353 |
| RP_NEG_435_10196a0_709 | RP | NEG | 0.71 | 435.1020 | | | | 8.33E-02 | 0.23 | 0.71 | 8.14E-01 | 0.05 | 0.51 | 1.86E-01 | 0.26 | 0.63 | 1.83E-01 | 0.21 | 1.04 | 1113014 | 355168 | 1315886 | 978257 | 1292690 | 835169 | 1339082 | 1111260 |
| RP_POS_503_59991a6_788 | RP | POS | 6.79 | 503.5999 | | | | 1.92E-07 | 0.62 | 1.33 | 8.14E-01 | -0.05 | 1.13 | 4.38E-04 | 0.59 | 1.42 | 1.49E-04 | 0.65 | 1.41 | 10787 | 15594 | 689660 | 1211906 | 718376 | 1216557 | 660944 | 1218898 |
| RP_NEG_179_05702a2_929 | RP | NEG | 2.93 | 179.0570 | | | | 6.97E-01 | -0.07 | 0.42 | 8.14E-01 | -0.05 | 0.16 | 6.53E-01 | -0.10 | 0.28 | 8.20E-01 | -0.05 | 0.53 | 1455632 | 1386234 | 1340579 | 1634657 | 1379293 | 1610133 | 1301865 | 1674262 |
| RP_POS_248_90034a0_449 | RP | POS | 0.45 | 248.9003 | | | | 2.14E-06 | -0.64 | 1.31 | 8.14E-01 | 0.05 | 1.13 | 4.05E-04 | -0.61 | 1.36 | 2.67E-04 | -0.67 | 1.42 | 1057759 | 181654 | 763854 | 497127 | 752086 | 513022 | 775622 | 485634 |
| HILIC_NEG_464_80627a2_909 | HILIC | NEG | 2.909 | 464.8063 | | | | 2.20E-07 | -0.64 | 1.35 | 8.14E-01 | 0.05 | 0.77 | 1.60E-04 | -0.61 | 1.52 | 2.43E-04 | -0.67 | 1.54 | 5318895 | 422278 | 4065981 | 2151552 | 4015078 | 2285632 | 4116883 | 2030621 |

**Supplementary table 1: Metabolomics results** (in asceding order according to p-value from progressive ALD vs  non-progressive ALD comparison, with molecular features with ID_level of 1-2 first)

| | | | | | | | | all_ALD_vs_control | | | Prog_ALD_vs_non-prog_ALD | | | Prog_ALD_vs_control_ALD | | | Non-prog_ALD_vs_control | | | Healthy_control | | all_ALD | | Non-prog_ALD | | Progressive_ALD | |
|---|---|---|---|---|---|---|---|---|---|---|---|---|---|---|---|---|---|---|---|---|---|---|---|---|---|---|---|
| | | | | | | | | t-test | Cohen's | PLS-DA | t-test | Cohen's | PLS-DA | t-test | Cohen's | PLS-DA | t-test | Cohen's | PLS-DA | | | | | | | | |
| Feature_ID | RP_HILIC | POS_NEG | Average.Rt | Average.Mz | Metabolite.name | curated_MSMS | ID_level | p | d | VIP | p | d | VIP | p | d | VIP | p | d | VIP | Mean | SD | Mean | SD | Mean | SD | Mean | SD |
| RP_POS_794_50836a12_76 | RP | POS | 12.76 | 794.5084 | | | | 1.18E-01 | 0.28 | 0.64 | 8.15E-01 | -0.05 | 0.45 | 1.70E-01 | 0.26 | 0.55 | 1.79E-01 | 0.31 | 0.57 | 995585 | 782041 | 1266536 | 1005871 | 1290317 | 1186754 | 1242755 | 796657 |
| RP_POS_424_77634a13_872 | RP | POS | 13.87 | 424.7763 | | | | 3.96E-01 | -0.17 | 0.56 | 8.15E-01 | 0.05 | 1.00 | 5.32E-01 | -0.15 | 0.57 | 3.63E-01 | -0.20 | 0.42 | 4566870 | 983545 | 4394779 | 1006882 | 4371039 | 877809 | 4418520 | 1129874 |
| RP_POS_257_21057a8_795 | RP | POS | 8.80 | 257.2106 | | | | 7.13E-01 | 0.08 | 0.54 | 8.15E-01 | 0.05 | 0.55 | 6.71E-01 | 0.10 | 0.40 | 8.14E-01 | 0.05 | 0.51 | 2914418 | 345444 | 2940054 | 330161 | 2932273 | 315465 | 2947835 | 347269 |
| RP_POS_319_16531a3_83 | RP | POS | 3.83 | 319.1653 | | | | 4.63E-03 | 0.40 | 0.64 | 8.15E-01 | -0.05 | 0.41 | 5.30E-02 | 0.38 | 0.74 | 1.12E-02 | 0.43 | 0.98 | 626545 | 358616 | 926347 | 822664 | 945728 | 743669 | 906966 | 901971 |
| RP_POS_276_89288a0_248 | RP | POS | 0.25 | 276.8929 | | | | 5.40E-01 | -0.12 | 0.41 | 8.15E-01 | 0.05 | 0.37 | 6.68E-01 | -0.09 | 0.30 | 5.14E-01 | -0.14 | 0.33 | 444182 | 58320 | 436650 | 65406 | 435111 | 65233 | 438188 | 66204 |
| HILIC_NEG_197_80783a3_033 | HILIC | NEG | 3.033 | 197.8078 | | | | 4.62E-02 | 0.30 | 1.06 | 8.16E-01 | -0.05 | 0.81 | 1.34E-01 | 0.28 | 0.69 | 8.78E-02 | 0.33 | 0.95 | 146737461 | 22408641 | 158459372 | 42455359 | 159457432 | 43828057 | 157461311 | 41458033 |
| HILIC_POS_141_13853a1_074 | HILIC | POS | 1.074 | 141.1385 | | | | 8.81E-03 | -0.47 | 1.03 | 8.16E-01 | -0.05 | 0.71 | 1.99E-02 | -0.49 | 0.93 | 2.84E-02 | -0.44 | 0.87 | 6466565 | 1208920 | 5746639 | 1600966 | 5784257 | 1542521 | 5709022 | 1672194 |
| RP_POS_553_4588a10_458 | RP | POS | 10.46 | 553.4588 | | | | 4.57E-02 | -0.45 | 0.93 | 8.16E-01 | 0.04 | 0.30 | 8.44E-02 | -0.42 | 0.77 | 4.92E-02 | -0.47 | 0.82 | 1108136 | 449251 | 926925 | 384833 | 917889 | 364108 | 935960 | 408012 |
| RP_NEG_282_77814a0_522 | RP | NEG | 0.52 | 282.7781 | | | | 1.60E-08 | -0.80 | 1.41 | 8.16E-01 | 0.05 | 1.14 | 9.64E-06 | -0.77 | 1.49 | 1.33E-05 | -0.82 | 1.46 | 1270793 | 172768 | 962702 | 406300 | 953167 | 427840 | 972238 | 387681 |
| RP_NEG_213_02271a6_441 | RP | NEG | 6.44 | 213.0227 | | | | 8.74E-02 | 0.24 | 0.55 | 8.17E-01 | 0.05 | 0.18 | 1.49E-01 | 0.27 | 0.59 | 2.18E-01 | 0.22 | 0.56 | 1097512 | 1170190 | 1669374 | 2593001 | 1608814 | 2514834 | 1729933 | 2693074 |
| RP_NEG_322_89328a0_512 | RP | NEG | 0.51 | 322.8933 | | | | 8.54E-05 | -0.69 | 1.20 | 8.17E-01 | 0.05 | 0.96 | 8.82E-04 | -0.66 | 1.19 | 5.40E-04 | -0.71 | 1.25 | 604584 | 156352 | 458306 | 216907 | 453253 | 223215 | 463358 | 212557 |
| RP_POS_282_88321a0_254 | RP | POS | 0.25 | 282.8832 | | | | 3.89E-01 | 0.18 | 0.57 | 8.17E-01 | -0.05 | 0.18 | 5.14E-01 | 0.15 | 0.35 | 3.68E-01 | 0.20 | 0.44 | 3123147 | 492198 | 3209984 | 493109 | 3221467 | 457352 | 3198501 | 530882 |
| RP_POS_693_45184a10_905 | RP | POS | 10.91 | 693.4518 | | | | 4.95E-01 | 0.12 | 0.60 | 8.17E-01 | -0.05 | 0.60 | 6.32E-01 | 0.09 | 0.44 | 5.02E-01 | 0.14 | 0.81 | 779918 | 350895 | 834168 | 490415 | 845586 | 530475 | 822750 | 451943 |
| HILIC_POS_173_09183a5_188 | HILIC | POS | 5.188 | 173.0918 | | | | 4.33E-07 | -0.76 | 1.39 | 8.17E-01 | 0.05 | 0.76 | 5.68E-05 | -0.73 | 1.51 | 4.07E-05 | -0.78 | 1.46 | 5727339 | 1298625 | 3873785 | 2559421 | 3814228 | 2645797 | 3933342 | 2495492 |
| RP_POS_619_75122a6_197 | RP | POS | 6.20 | 619.7512 | | | | 1.05E-04 | 0.46 | 1.05 | 8.17E-01 | -0.05 | 0.93 | 9.81E-03 | 0.43 | 1.13 | 3.99E-03 | 0.48 | 1.21 | 230666 | 75081 | 663518 | 1063735 | 688270 | 1067417 | 638765 | 1070292 |
| HILIC_NEG_288_85132a2_924 | HILIC | NEG | 2.924 | 288.8513 | | | | 7.72E-07 | -0.62 | 1.34 | 8.17E-01 | 0.05 | 0.77 | 2.69E-04 | -0.59 | 1.48 | 4.39E-04 | -0.64 | 1.52 | 1942485 | 168584 | 1522082 | 749914 | 1504633 | 800970 | 1539532 | 702884 |
| RP_POS_486_93842a6_729 | RP | POS | 6.73 | 486.9384 | | | | 1.65E-07 | 0.62 | 1.33 | 8.17E-01 | -0.05 | 1.14 | 3.58E-04 | 0.60 | 1.42 | 1.58E-04 | 0.65 | 1.41 | 27895 | 46337 | 1424534 | 2477802 | 1482182 | 2511797 | 1366886 | 2467468 |
| RP_POS_550_29034a10_456 | RP | POS | 10.46 | 550.2903 | | | | 1.48E-13 | 1.03 | 1.80 | 8.17E-01 | 0.05 | 0.52 | 1.36E-07 | 1.05 | 1.75 | 3.40E-10 | 1.01 | 1.93 | 644568 | 263786 | 1306889 | 646059 | 1291857 | 541139 | 1321920 | 741627 |
| RP_POS_429_31851a13_016 | RP | POS | 13.02 | 429.3185 | | | | 3.04E-01 | -0.21 | 1.02 | 8.18E-01 | -0.05 | 0.27 | 2.98E-01 | -0.24 | 1.09 | 4.14E-01 | -0.19 | 0.44 | 2778401 | 1089041 | 2550630 | 1050743 | 2575027 | 1095998 | 2526233 | 1014016 |
| HILIC_POS_126_02219a5_325 | HILIC | POS | 5.325 | 126.0222 | | | | 1.39E-04 | 0.49 | 1.14 | 8.18E-01 | 0.05 | 0.69 | 5.03E-03 | 0.51 | 1.26 | 5.01E-03 | 0.46 | 1.27 | 2966642 | 929057 | 4437964 | 3366331 | 4359829 | 3178104 | 4516099 | 3575309 |
| HILIC_NEG_538_7373a2_922 | HILIC | NEG | 2.922 | 538.7373 | | | | 8.77E-07 | -0.62 | 1.34 | 8.18E-01 | 0.05 | 0.77 | 3.07E-04 | -0.59 | 1.48 | 4.24E-04 | -0.64 | 1.51 | 989237 | 95748 | 759197 | 410726 | 749669 | 435973 | 768725 | 388042 |
| HILIC_NEG_656_88708a4_232 | HILIC | NEG | 4.232 | 656.8871 | | | | 6.28E-04 | 0.44 | 1.25 | 8.18E-01 | -0.05 | 0.90 | 1.10E-02 | 0.42 | 1.31 | 1.12E-02 | 0.47 | 1.42 | 2654840 | 171808 | 2882950 | 575694 | 2896278 | 615847 | 2869621 | 538498 |
| RP_POS_800_57666a14_261 | RP | POS | 14.26 | 800.5767 | | | | 3.42E-01 | 0.15 | 0.46 | 8.18E-01 | 0.05 | 0.18 | 4.47E-01 | 0.18 | 0.34 | 3.85E-01 | 0.13 | 1.02 | 414706 | 279924 | 479771 | 467052 | 468947 | 264603 | 490595 | 608666 |
| RP_POS_267_15591a8_499 | RP | POS | 8.50 | 267.1559 | | | | 4.27E-01 | 0.18 | 0.69 | 8.18E-01 | 0.04 | 0.43 | 4.11E-01 | 0.20 | 0.43 | 5.22E-01 | 0.16 | 0.65 | 2754550 | 482254 | 2830208 | 404452 | 2820854 | 408038 | 2839561 | 404757 |
| RP_POS_158_96312a15_379 | RP | POS | 15.38 | 158.9631 | | | | 3.55E-01 | 0.20 | 0.55 | 8.18E-01 | 0.05 | 0.26 | 3.36E-01 | 0.22 | 0.42 | 4.66E-01 | 0.18 | 0.79 | 7287835 | 1160718 | 7502817 | 1047295 | 7478601 | 1130504 | 7527034 | 967885 |
| HILIC_NEG_57_03372a0_927 | HILIC | NEG | 0.927 | 57.0337 | | | | 1.44E-06 | 0.68 | 1.11 | 8.19E-01 | -0.05 | 1.25 | 1.23E-03 | 0.65 | 1.03 | 8.22E-06 | 0.70 | 1.43 | 89170 | 53580 | 169397 | 126667 | 172326 | 103198 | 166468 | 147476 |
| RP_POS_441_29691a13_797 | RP | POS | 13.80 | 441.2969 | | | | 6.39E-01 | 0.10 | 0.24 | 8.19E-01 | -0.04 | 0.49 | 7.29E-01 | 0.08 | 0.29 | 6.11E-01 | 0.13 | 0.37 | 19261710 | 6781479 | 19891355 | 5838171 | 20026252 | 6317300 | 19756459 | 5377608 |
| RP_POS_253_1263a0_551 | RP | POS | 0.55 | 253.1263 | | | | 6.60E-01 | 0.08 | 0.30 | 8.19E-01 | -0.05 | 0.27 | 7.93E-01 | 0.06 | 0.19 | 6.16E-01 | 0.10 | 0.66 | 946326 | 240123 | 969230 | 297663 | 976107 | 290690 | 962353 | 307274 |
| RP_POS_783_0658a13_23 | RP | POS | 13.23 | 783.0658 | | | | 3.48E-01 | 0.17 | 0.45 | 8.19E-01 | -0.05 | 0.30 | 4.67E-01 | 0.15 | 0.34 | 3.73E-01 | 0.20 | 0.50 | 1010626 | 1065102 | 1228958 | 1337879 | 1259858 | 1447213 | 1198058 | 1232881 |
| HILIC_NEG_134_89481a3_128 | HILIC | NEG | 3.128 | 134.8948 | | | | 2.66E-04 | -0.58 | 1.04 | 8.19E-01 | 0.05 | 0.63 | 3.65E-03 | -0.55 | 1.06 | 1.91E-03 | -0.60 | 1.09 | 106519762 | 21149052 | 86767725 | 36236650 | 85931625 | 36840305 | 87603826 | 35977114 |
| RP_POS_553_4585a11_693 | RP | POS | 11.69 | 553.4585 | | | | 6.89E-01 | -0.09 | 0.41 | 8.20E-01 | -0.04 | 0.26 | 6.46E-01 | -0.11 | 0.31 | 7.81E-01 | -0.07 | 0.40 | 11328276 | 5569474 | 10890190 | 4619458 | 10996105 | 4728272 | 10784275 | 4553552 |
| HILIC_POS_146_11775a3_338 | HILIC | POS | 3.338 | 146.1178 | | | | 4.79E-01 | 0.12 | 0.65 | 8.20E-01 | -0.05 | 0.65 | 6.52E-01 | 0.10 | 0.39 | 4.39E-01 | 0.15 | 0.47 | 70867146 | 22140112 | 74415236 | 30891907 | 75123538 | 26986989 | 73706935 | 34623318 |
| RP_NEG_321_13254a7_723 | RP | NEG | 7.72 | 321.1325 | | | | 5.43E-02 | -0.36 | 0.74 | 8.20E-01 | -0.05 | 0.41 | 6.39E-02 | -0.39 | 0.76 | 1.23E-01 | -0.34 | 0.60 | 899143 | 47704 | 879299 | 55783 | 880578 | 59537 | 878020 | 52334 |
| HILIC_NEG_155_86565a2_95 | HILIC | NEG | 2.95 | 155.8657 | | | | 2.19E-03 | 0.44 | 1.15 | 8.20E-01 | -0.05 | 0.79 | 1.91E-02 | 0.41 | 0.99 | 1.37E-02 | 0.46 | 1.20 | 8585850 | 1641784 | 10040724 | 3623448 | 10123774 | 3785031 | 9957674 | 3490869 |
| HILIC_POS_120_06582a5_785 | HILIC | POS | 5.785 | 120.0658 | | | | 2.75E-01 | -0.20 | 0.95 | 8.20E-01 | 0.05 | 0.65 | 4.50E-01 | -0.17 | 0.66 | 2.38E-01 | -0.22 | 0.82 | 7800729 | 2675338 | 7159692 | 3398170 | 7081967 | 2648854 | 7237416 | 4037578 |
| RP_POS_148_03964a3_947 | RP | POS | 3.95 | 148.0396 | | | | 1.41E-02 | -0.64 | 1.23 | 8.21E-01 | 0.04 | 0.88 | 2.57E-02 | -0.62 | 0.94 | 1.29E-02 | -0.66 | 1.36 | 5314290 | 3773505 | 3500202 | 2294646 | 3447752 | 1867937 | 3552652 | 2672724 |
| HILIC_POS_407_16473a5_723 | HILIC | POS | 5.723 | 407.1647 | | | | 1.34E-02 | 0.33 | 0.57 | 8.21E-01 | -0.05 | 0.46 | 6.80E-02 | 0.31 | 0.58 | 5.47E-02 | 0.36 | 0.64 | 193102 | 332843 | 470176 | 935517 | 491519 | 994703 | 448832 | 881975 |
| RP_POS_632_49078a13_505 | RP | POS | 13.51 | 632.4908 | | | | 8.89E-01 | -0.03 | 0.28 | 8.21E-01 | 0.05 | 0.30 | 9.94E-01 | 0.00 | 0.26 | 7.91E-01 | -0.05 | 0.23 | 432506 | 108128 | 429222 | 133964 | 426168 | 100908 | 432276 | 161423 |
| HILIC_NEG_141_92374a0_593 | HILIC | NEG | 0.593 | 141.9237 | | | | 2.46E-01 | 0.24 | 0.80 | 8.21E-01 | -0.05 | 0.73 | 3.33E-01 | 0.22 | 0.42 | 2.60E-01 | 0.26 | 0.63 | 445697 | 154582 | 482335 | 150328 | 485760 | 157842 | 478909 | 143944 |
| RP_POS_629_40137a11_978 | RP | POS | 11.98 | 629.4014 | | | | 7.54E-01 | -0.06 | 0.68 | 8.22E-01 | 0.05 | 0.21 | 8.65E-01 | -0.04 | 0.51 | 6.94E-01 | -0.09 | 0.30 | 1114741 | 528344 | 1080474 | 558982 | 1067801 | 516587 | 1093147 | 603415 |
| RP_POS_517_34979a10_889 | RP | POS | 10.89 | 517.3498 | | | | 4.02E-01 | 0.14 | 0.77 | 8.22E-01 | 0.05 | 0.48 | 4.32E-01 | 0.17 | 0.65 | 5.38E-01 | 0.12 | 0.51 | 2683308 | 707461 | 2820900 | 1048622 | 2797141 | 954491 | 2844659 | 1144312 |
| HILIC_POS_366_77661a3_008 | HILIC | POS | 3.008 | 366.7766 | | | | 1.18E-05 | -0.59 | 1.35 | 8.22E-01 | 0.05 | 0.77 | 9.68E-04 | -0.56 | 1.47 | 9.39E-04 | -0.61 | 1.47 | 711469 | 111677 | 540300 | 319444 | 533073 | 335834 | 547528 | 305418 |
| HILIC_NEG_370_81476a2_923 | HILIC | NEG | 2.923 | 370.8148 | | | | 6.51E-07 | -0.62 | 1.35 | 8.23E-01 | 0.05 | 0.77 | 2.40E-04 | -0.60 | 1.49 | 3.99E-04 | -0.65 | 1.52 | 2417911 | 219509 | 1875493 | 958205 | 1853841 | 1022528 | 1897145 | 899183 |
| HILIC_POS_263_0867a7_689 | HILIC | POS | 7.689 | 263.0867 | | | | 8.54E-03 | -0.50 | 0.90 | 8.23E-01 | 0.05 | 0.63 | 2.48E-02 | -0.48 | 0.83 | 1.66E-02 | -0.52 | 0.90 | 1538949 | 110710 | 1475064 | 130129 | 1472123 | 134208 | 1478004 | 127213 |
| HILIC_POS_362_92596a4_324 | HILIC | POS | 4.324 | 362.9260 | | | | 1.33E-01 | 0.25 | 1.03 | 8.23E-01 | -0.05 | 0.85 | 2.47E-01 | 0.22 | 0.59 | 1.79E-01 | 0.27 | 0.92 | 34527024 | 6625448 | 36885437 | 10236912 | 37116687 | 10676100 | 36654187 | 9881252 |
| RP_POS_394_87268a0_498 | RP | POS | 0.50 | 394.8727 | | | | 1.25E-06 | -0.59 | 1.32 | 8.23E-01 | 0.05 | 1.20 | 4.30E-04 | -0.57 | 1.39 | 6.48E-04 | -0.62 | 1.40 | 24028214 | 1782128 | 18397241 | 10539309 | 18159671 | 11224834 | 18634811 | 9915082 |
| RP_POS_310_2373a9_788 | RP | POS | 9.79 | 310.2373 | | | | 6.23E-03 | -0.47 | 1.14 | 8.23E-01 | 0.05 | 0.51 | 2.76E-02 | -0.44 | 0.85 | 1.45E-02 | -0.49 | 1.30 | 5585408 | 191542 | 5462311 | 276922 | 5456070 | 276933 | 5468553 | 279580 |
| RP_POS_600_83197a0_498 | RP | POS | 0.50 | 600.8320 | | | | 6.76E-08 | -0.66 | 1.36 | 8.23E-01 | 0.05 | 1.18 | 1.42E-04 | -0.64 | 1.45 | 1.06E-04 | -0.68 | 1.45 | 1801587 | 116138 | 1368945 | 720117 | 1352718 | 743278 | 1385172 | 703363 |
| RP_POS_278_88828a0_249 | RP | POS | 0.25 | 278.8883 | | | | 7.80E-01 | -0.06 | 0.54 | 8.23E-01 | 0.04 | 0.24 | 8.77E-01 | -0.04 | 0.42 | 7.28E-01 | -0.08 | 0.28 | 4271961 | 505039 | 4243404 | 488059 | 4232424 | 489548 | 4254385 | 491285 |
| RP_POS_195_10164a8_51 | RP | POS | 8.51 | 195.1016 | | | | 5.33E-01 | -0.11 | 0.54 | 8.23E-01 | 0.05 | 0.42 | 6.93E-01 | -0.09 | 0.46 | 4.78E-01 | -0.14 | 0.70 | 16690327 | 543561 | 16616395 | 686610 | 16600949 | 567200 | 16631840 | 793905 |
| RP_POS_337_16782a10_978 | RP | POS | 10.98 | 337.1678 | | | | 3.04E-02 | 0.40 | 1.44 | 8.24E-01 | -0.05 | 1.15 | 8.73E-02 | 0.37 | 1.23 | 3.77E-02 | 0.42 | 1.28 | 25458620 | 6540283 | 28586585 | 8122823 | 28768900 | 7441095 | 28404270 | 8824359 |
| HILIC_POS_76_07645a1_509 | HILIC | POS | 1.509 | 76.0765 | | | | 7.30E-01 | 0.06 | 0.32 | 8.24E-01 | 0.05 | 0.88 | 7.06E-01 | 0.08 | 0.15 | 8.50E-01 | 0.03 | 0.10 | 121835579 | 76300708 | 127860276 | 109947102 | 125393958 | 91536939 | 130326594 | 126617562 |
| RP_POS_820_58411a13_312 | RP | POS | 13.31 | 820.5841 | | | | 2.27E-11 | -1.42 | 2.49 | 8.24E-01 | -0.04 | 0.84 | 7.78E-11 | -1.44 | 2.39 | 1.33E-10 | -1.40 | 2.26 | 3008226 | 1011724 | 1271217 | 961453 | 1292743 | 949686 | 1249692 | 982242 |
| RP_POS_599_36584a14_266 | RP | POS | 14.27 | 599.3658 | | | | 2.94E-01 | -0.22 | 0.52 | 8.24E-01 | -0.04 | 0.78 | 2.91E-01 | -0.24 | 0.56 | 4.04E-01 | -0.19 | 0.35 | 3088573 | 960576 | 2882271 | 950769 | 2903533 | 991472 | 2861008 | 917842 |
| HILIC_NEG_263_10385a1_832 | HILIC | NEG | 1.832 | 263.1039 | | | | 1.71E-01 | -0.25 | 0.53 | 8.25E-01 | 0.05 | 0.52 | 3.38E-01 | -0.23 | 0.33 | 1.27E-01 | -0.28 | 0.81 | 1057862 | 680141 | 855374 | 830860 | 836839 | 541841 | 873909 | 1049024 |
| RP_NEG_343_68149a15_599 | RP | NEG | 15.60 | 343.6815 | | | | 5.53E-01 | -0.12 | 0.55 | 8.25E-01 | -0.05 | 0.16 | 5.40E-01 | -0.14 | 0.37 | 6.62E-01 | -0.10 | 0.40 | 1301934 | 60403 | 1294492 | 64173 | 1295922 | 60520 | 1293063 | 68217 |
| RP_NEG_218_98579a7_148 | RP | NEG | 7.15 | 218.9858 | | | | 3.35E-01 | -0.22 | 0.53 | 8.25E-01 | 0.04 | 0.22 | 4.12E-01 | -0.20 | 0.36 | 3.41E-01 | -0.24 | 0.86 | 3220331 | 1509276 | 2933109 | 1259372 | 2905060 | 1358977 | 2961158 | 1164464 |
| RP_POS_273_20645a8_499 | RP | POS | 8.50 | 273.2065 | | | | 9.20E-01 | -0.02 | 0.57 | 8.25E-01 | 0.04 | 0.39 | 9.95E-01 | 0.00 | 0.22 | 8.59E-01 | -0.04 | 0.56 | 2007422 | 367892 | 2000190 | 304758 | 1993411 | 312372 | 2006970 | 299967 |
| HILIC_POS_430_91431a4_324 | HILIC | POS | 4.324 | 430.9143 | | | | 1.45E-01 | 0.24 | 1.02 | 8.25E-01 | -0.05 | 0.84 | 2.60E-01 | 0.22 | 0.57 | 1.91E-01 | 0.27 | 0.91 | 25638178 | 4948425 | 27339323 | 7550140 | 27507280 | 7885791 | 27171366 | 7275254 |
| RP_POS_253_88002a0_249 | RP | POS | 0.25 | 253.8800 | | | | 2.63E-01 | 0.22 | 0.41 | 8.26E-01 | 0.04 | 0.45 | 2.61E-01 | 0.25 | 0.68 | 3.78E-01 | 0.20 | 0.39 | 1036690 | 138567 | 1068867 | 144315 | 1065663 | 152577 | 1072072 | 137032 |
| HILIC_POS_106_05019a6_165 | HILIC | POS | 6.165 | 106.0502 | | | | 5.08E-01 | -0.10 | 1.15 | 8.26E-01 | -0.05 | 0.70 | 5.37E-01 | -0.13 | 1.07 | 6.53E-01 | -0.08 | 1.16 | 1543151 | 445283 | 1469226 | 785303 | 1486666 | 687232 | 1451785 | 879249 |
| RP_POS_282_16006a6_888 | RP | POS | 6.89 | 282.1601 | | | | 1.20E-02 | 0.38 | 0.66 | 8.26E-01 | -0.05 | 0.57 | 1.03E-02 | 0.36 | 0.98 | 7.41E-02 | 0.41 | 0.75 | 1231277 | 594956 | 1623825 | 1116453 | 1648617 | 1447804 | 1599032 | 648832 |
| RP_NEG_730_81561a0_499 | RP | NEG | 0.50 | 730.8156 | | | | 4.67E-06 | -0.58 | 1.30 | 8.26E-01 | 0.05 | 1.19 | 5.35E-04 | -0.56 | 1.39 | 1.04E-03 | -0.61 | 1.36 | 1815803 | 190793 | 1444375 | 699503 | 1428862 | 755508 | 1459887 | 645995 |
| RP_NEG_241_75789a0_57 | RP | NEG | 0.57 | 241.7579 | | | | 1.71E-04 | 0.51 | 0.90 | 8.26E-01 | -0.05 | 0.81 | 1.37E-02 | 0.49 | 0.97 | 6.66E-04 | 0.53 | 1.17 | 2164414 | 618545 | 2933805 | 1658015 | 2970509 | 1403914 | 2897101 | 1892195 |
| HILIC_POS_317_056a5_463 | HILIC | POS | 5.463 | 317.0560 | | | | 1.35E-01 | -0.29 | 0.77 | 8.26E-01 | 0.05 | 0.32 | 2.00E-01 | -0.27 | 0.52 | 1.72E-01 | -0.31 | 0.69 | 7838974 | 1059979 | 7504242 | 1166984 | 7478415 | 1287489 | 7530069 | 1045232 |
| RP_POS_496_41922a10_88 | RP | POS | 10.88 | 496.4192 | | | | 2.74E-01 | -0.19 | 0.55 | 8.26E-01 | 0.05 | 0.48 | 3.87E-01 | -0.17 | 0.76 | 3.15E-01 | -0.22 | 0.46 | 7042410 | 581981 | 6901095 | 765987 | 6884155 | 832873 | 6918034 | 700845 |
| RP_POS_177_0546a7_494 | RP | POS | 7.49 | 177.0546 | | | | 5.73E-01 | 0.10 | 0.43 | 8.27E-01 | 0.05 | 0.59 | 5.53E-01 | 0.13 | 0.30 | 7.03E-01 | 0.08 | 0.34 | 4375046 | 373330 | 4420673 | 462326 | 4410465 | 458727 | 4430880 | 470331 |
| RP_POS_182_95851a0_258 | RP | POS | 0.26 | 182.9585 | | | | 2.23E-01 | 0.23 | 0.57 | 8.27E-01 | -0.05 | 0.17 | 3.44E-01 | 0.21 | 0.34 | 2.29E-01 | 0.26 | 0.76 | 1117283 | 285264 | 1191159 | 325096 | 1198327 | 309179 | 1183991 | 343271 |
| RP_POS_171_14877a5_931 | RP | POS | 5.93 | 171.1488 | | | | 3.35E-01 | -0.16 | 1.01 | 8.27E-01 | -0.05 | 1.04 | 3.19E-01 | -0.18 | 0.87 | 5.18E-01 | -0.14 | 1.04 | 16534477 | 4229499 | 15582181 | 6354425 | 15722207 | 7092148 | 15442154 | 5589537 |
| RP_POS_736_54028a13_57 | RP | POS | 13.57 | 736.5403 | | | | 5.93E-01 | 0.13 | 0.62 | 8.27E-01 | -0.04 | 0.30 | 6.75E-01 | 0.11 | 0.46 | 5.56E-01 | 0.15 | 0.65 | 2757166 | 315826 | 2789452 | 218617 | 2794267 | 198570 | 2784637 | 238923 |
| HILIC_NEG_100_93345a5_606 | HILIC | NEG | 5.606 | 100.9335 | | | | 1.77E-01 | -0.22 | 0.75 | 8.27E-01 | -0.05 | 0.65 | 2.24E-01 | -0.24 | 0.50 | 3.11E-01 | -0.19 | 0.48 | 33154881 | 3032277 | 32158908 | 4968913 | 32268274 | 4844901 | 32049542 | 5136788 |
| HILIC_NEG_271_99994a0_93 | HILIC | NEG | 0.93 | 271.9999 | | | | 1.83E-08 | 0.80 | 1.33 | 8.27E-01 | -0.05 | 1.19 | 7.02E-05 | 0.78 | 1.28 | 1.03E-06 | 0.82 | 1.58 | 12471019 | 8109958 | 26706582 | 18717167 | 27118450 | 16631147 | 26294714 | 20757484 |
| HILIC_NEG_426_6882a2_935 | HILIC | NEG | 2.935 | 426.6882 | | | | 5.67E-06 | -0.61 | 1.31 | 8.27E-01 | 0.05 | 0.75 | 5.77E-04 | -0.58 | 1.43 | 6.60E-04 | -0.63 | 1.48 | 142232 | 21820 | 107510 | 62357 | 106138 | 65867 | 108882 | 59278 |
| HILIC_NEG_136_89165a4_165 | HILIC | NEG | 4.165 | 136.8917 | | | | 8.51E-01 | -0.04 | 0.43 | 8.27E-01 | 0.05 | 0.50 | 9.53E-01 | -0.01 | 0.16 | 7.85E-01 | -0.06 | 0.15 | 4626951 | 2215466 | 4539047 | 2537400 | 4483254 | 2469637 | 4594839 | 2627307 |
| HILIC_POS_115_12332a3_024 | HILIC | POS | 3.024 | 115.1233 | | | | 3.00E-07 | 0.66 | 1.30 | 8.27E-01 | 0.05 | 0.80 | 6.98E-05 | 0.68 | 1.47 | 3.90E-04 | 0.63 | 1.44 | 1249437 | 95940 | 1457499 | 345368 | 1449911 | 357402 | 1465088 | 336362 |
| RP_POS_275_07907a5_873 | RP | POS | 5.87 | 275.0791 | Unknown | 275.0778(100) | 4 | 1.59E-01 | -0.20 | 0.46 | 8.28E-01 | -0.05 | 0.32 | 4.33E-02 | -0.22 | 0.95 | 4.54E-01 | -0.17 | 0.48 | 4256796 | 2321578 | 3284517 | 5503152 | 3405511 | 7448823 | 3163524 | 2381716 |
| RP_NEG_299_25934a10_962 | RP | NEG | 10.96 | 299.2593 | | | | 3.98E-03 | -0.49 | 1.09 | 8.28E-01 | 0.05 | 0.84 | 1.38E-02 | -0.46 | 1.09 | 1.54E-02 | -0.51 | 1.02 | 25740713 | 3395215 | 23417210 | 5023390 | 23307027 | 5508293 | 23527393 | 4540683 |
| RP_POS_467_80005a0_245 | RP | POS | 0.25 | 467.8001 | | | | 8.82E-02 | 0.32 | 0.58 | 8.28E-01 | 0.05 | 0.20 | 1.04E-01 | 0.34 | 0.76 | 1.76E-01 | 0.29 | 0.78 | 522692 | 228950 | 607893 | 279597 | 601763 | 292266 | 614023 | 269162 |
| RP_NEG_304_91425a0_448 | RP | NEG | 0.45 | 304.9143 | | | | 9.80E-06 | -0.60 | 1.26 | 8.28E-01 | 0.05 | 1.13 | 9.34E-04 | -0.57 | 1.31 | 6.69E-04 | -0.62 | 1.34 | 10733442 | 2006522 | 7737873 | 5450827 | 7618525 | 5635887 | 7857221 | 5313859 |
| HILIC_POS_429_36749a1_071 | HILIC | POS | 1.071 | 429.3675 | | | | 1.16E-02 | -0.41 | 1.15 | 8.28E-01 | -0.05 | 0.68 | 2.80E-02 | -0.44 | 1.25 | 4.50E-02 | -0.39 | 1.06 | 1321326 | 377657 | 1089726 | 600099 | 1102847 | 593649 | 1076604 | 612222 |
| RP_POS_169_11946a4_931 | RP | POS | 4.93 | 169.1195 | | | | 5.25E-01 | 0.12 | 0.50 | 8.28E-01 | 0.05 | 0.35 | 5.28E-01 | 0.14 | 0.46 | 6.30E-01 | 0.10 | 0.44 | 2601333 | 292714 | 2640727 | 332975 | 2633450 | 294293 | 2648004 | 370529 |
| HILIC_NEG_446_74567a2_929 | HILIC | NEG | 2.929 | 446.7457 | | | | 1.31E-06 | -0.63 | 1.34 | 8.28E-01 | 0.05 | 0.76 | 2.76E-04 | -0.60 | 1.46 | 4.31E-04 | -0.65 | 1.49 | 425019 | 52024 | 324097 | 176323 | 320247 | 187855 | 327947 | 165813 |
| HILIC_NEG_167_8343a3_01 | HILIC | NEG | 3.01 | 167.8343 | | | | 3.40E-01 | 0.18 | 0.96 | 8.29E-01 | -0.05 | 0.77 | 4.67E-01 | 0.15 | 0.66 | 3.55E-01 | 0.20 | 0.53 | 3810572 | 838418 | 3984632 | 1038937 | 4007303 | 1065693 | 3961960 | 1021793 |
| RP_POS_248_86255a0_245 | RP | POS | 0.25 | 248.8626 | | | | 9.27E-01 | 0.02 | 0.30 | 8.29E-01 | -0.05 | 0.29 | 9.81E-01 | -0.01 | 0.19 | 8.57E-01 | 0.04 | 0.66 | 366397 | 50164 | 367376 | 58216 | 368645 | 61421 | 366107 | 55421 |
| RP_POS_524_29944a10_502 | RP | POS | 10.50 | 524.2994 | | | | 1.92E-03 | -0.55 | 1.07 | 8.29E-01 | -0.04 | 0.22 | 4.36E-03 | -0.57 | 1.10 | 1.20E-02 | -0.53 | 1.09 | 12915064 | 2068749 | 11432077 | 2790582 | 11492812 | 2933696 | 11371342 | 2668242 |
| RP_POS_338_26642a8_966 | RP | POS | 8.97 | 338.2664 | | | | 1.59E-01 | 0.28 | 0.52 | 8.29E-01 | 0.04 | 0.26 | 1.93E-01 | 0.31 | 0.61 | 2.22E-01 | 0.26 | 0.53 | 1402781 | 2175552 | 2039440 | 2245367 | 1990628 | 1991177 | 2088251 | 2493309 |
| HILIC_NEG_532_70422a2_927 | HILIC | NEG | 2.927 | 532.7042 | | | | 3.45E-06 | -0.61 | 1.33 | 8.29E-01 | 0.05 | 0.76 | 4.52E-04 | -0.58 | 1.45 | 6.65E-04 | -0.63 | 1.46 | 152919 | 20578 | 116359 | 66005 | 114925 | 70346 | 117792 | 62045 |
| RP_POS_321_2399a10_265 | RP | POS | 10.27 | 321.2399 | | | | 3.13E-01 | -0.21 | 0.71 | 8.29E-01 | 0.04 | 0.46 | 4.05E-01 | -0.19 | 0.55 | 3.21E-01 | -0.23 | 0.66 | 9691333 | 2223015 | 9237585 | 2097110 | 9192054 | 2179854 | 9283116 | 2032118 |

**Supplementary table 1: Metabolomics results** (in asceding order according to p-value from progressive ALD vs  non-progressive ALD comparison, with molecular features with ID_level of 1-2 first)

| | | | | | | | | all_ALD_vs_control | | | Prog_ALD_vs_non-prog_ALD | | | Prog_ALD_vs_control_ALD | | | Non-prog_ALD_vs_control | | | Healthy_control | | all_ALD | | Non-prog_ALD | | Progressive_ALD | |
|---|---|---|---|---|---|---|---|---|---|---|---|---|---|---|---|---|---|---|---|---|---|---|---|---|---|---|---|
| | | | | | | | | t-test | Cohen's | PLS-DA | t-test | Cohen's | PLS-DA | t-test | Cohen's | PLS-DA | t-test | Cohen's | PLS-DA | | | | | | | | |
| Feature_ID | RP_HILIC | POS_NEG | Average.Rt | Average.Mz | Metabolite.name | curated_MSMS | ID_level | p | d | VIP | p | d | VIP | p | d | VIP | p | d | VIP | Mean | SD | Mean | SD | Mean | SD | Mean | SD |
| RP_POS_846_53998a13_373 | RP | POS | 13.37 | 846.5400 | | | | 4.81E-02 | 0.40 | 0.85 | 8.30E-01 | -0.04 | 0.22 | 9.87E-02 | 0.38 | 0.77 | 5.52E-02 | 0.43 | 0.79 | 1517360 | 742983 | 1823328 | 749292 | 1839573 | 705644 | 1807083 | 797409 |
| HILIC_POS_155_0928a1_236 | HILIC | POS | 1.236 | 155.0928 | | | | 5.52E-02 | -0.36 | 0.92 | 8.30E-01 | -0.04 | 0.66 | 8.39E-02 | -0.38 | 0.90 | 1.05E-01 | -0.33 | 1.01 | 1045514 | 112152 | 998544 | 136051 | 1001484 | 128127 | 995605 | 144788 |
| RP_NEG_349_23743a11_255 | RP | NEG | 11.26 | 349.2374 | | | | 1.19E-03 | 0.61 | 1.39 | 8.30E-01 | 0.04 | 0.82 | 3.42E-03 | 0.64 | 1.30 | 4.82E-03 | 0.59 | 1.26 | 2606231 | 730404 | 3134197 | 862363 | 3115568 | 836917 | 3152826 | 895198 |
| RP_POS_577_36945a10_464 | RP | POS | 10.46 | 577.3695 | | | | 1.64E-03 | 0.51 | 0.87 | 8.30E-01 | 0.05 | 0.36 | 7.01E-03 | 0.54 | 1.20 | 1.07E-02 | 0.49 | 1.01 | 1570765 | 383959 | 1869116 | 616846 | 1855791 | 603898 | 1882441 | 635380 |
| HILIC_POS_438_71259a3_013 | HILIC | POS | 3.013 | 438.7126 | | | | 3.34E-04 | -0.50 | 1.33 | 8.30E-01 | -0.05 | 0.76 | 2.37E-03 | -0.53 | 1.43 | 1.09E-02 | -0.48 | 1.44 | 368543 | 70679 | 290953 | 168935 | 294602 | 179366 | 287305 | 159566 |
| RP_POS_293_20853a9_675 | RP | POS | 9.68 | 293.2085 | | | | 3.92E-05 | 0.57 | 1.08 | 8.31E-01 | 0.05 | 0.43 | 2.43E-03 | 0.59 | 1.13 | 8.15E-04 | 0.55 | 1.26 | 3159323 | 415148 | 3700879 | 1035867 | 3678553 | 914467 | 3723205 | 1153547 |
| RP_POS_415_21124a8_658 | RP | POS | 8.66 | 415.2112 | | | | 1.69E-02 | 0.35 | 0.75 | 8.31E-01 | -0.05 | 0.71 | 1.02E-01 | 0.33 | 0.70 | 2.50E-02 | 0.38 | 0.84 | 378910 | 168005 | 488279 | 339160 | 495578 | 293690 | 480979 | 382156 |
| HILIC_NEG_132_90039a1_329 | HILIC | NEG | 1.329 | 132.9004 | | | | 3.56E-06 | -0.85 | 1.31 | 8.31E-01 | -0.04 | 0.57 | 4.48E-05 | -0.87 | 1.38 | 3.67E-05 | -0.83 | 1.45 | 732033 | 102051 | 619367 | 130565 | 622174 | 122556 | 616559 | 139307 |
| RP_POS_140_06808a11_224 | RP | POS | 11.22 | 140.0681 | | | | 9.16E-01 | 0.02 | 0.53 | 8.31E-01 | -0.04 | 0.23 | 9.92E-01 | 0.00 | 0.19 | 8.47E-01 | 0.04 | 0.64 | 1977723 | 803609 | 1995677 | 929153 | 2015630 | 952167 | 1975725 | 914786 |
| HILIC_POS_368_21994a2_934 | HILIC | POS | 2.934 | 368.2199 | | | | 2.74E-01 | -0.22 | 1.00 | 8.31E-01 | -0.04 | 0.83 | 2.85E-01 | -0.24 | 0.48 | 3.82E-01 | -0.19 | 0.34 | 3309356 | 605236 | 3171293 | 647402 | 3185188 | 649880 | 3157399 | 651210 |
| RP_NEG_264_93466a0_498 | RP | NEG | 0.50 | 264.9347 | | | | 1.59E-06 | -0.59 | 1.31 | 8.31E-01 | 0.05 | 1.20 | 3.94E-04 | -0.57 | 1.39 | 7.05E-04 | -0.62 | 1.38 | 14406798 | 1185576 | 11362499 | 5653152 | 11241199 | 6055045 | 11483798 | 5279664 |
| RP_NEG_851_11444a9_605 | RP | NEG | 9.61 | 851.1144 | | | | 1.70E-01 | -0.31 | 0.67 | 8.32E-01 | 0.04 | 0.54 | 2.17E-01 | -0.29 | 0.55 | 1.96E-01 | -0.33 | 0.52 | 5540572 | 409284 | 5428476 | 349634 | 5420991 | 397367 | 5435961 | 298275 |
| RP_POS_452_39294a10_881 | RP | POS | 10.88 | 452.3929 | | | | 6.71E-01 | -0.08 | 0.57 | 8.32E-01 | 0.05 | 0.44 | 7.87E-01 | -0.05 | 0.56 | 6.53E-01 | -0.10 | 0.55 | 4775982 | 436047 | 4734921 | 573765 | 4722645 | 632972 | 4747198 | 513974 |
| HILIC_POS_280_8183a3_007 | HILIC | POS | 3.007 | 280.8183 | | | | 2.39E-05 | -0.56 | 1.35 | 8.32E-01 | 0.05 | 0.77 | 1.42E-03 | -0.54 | 1.46 | 1.58E-03 | -0.58 | 1.47 | 6761346 | 997045 | 5254150 | 2952208 | 5191090 | 3123708 | 5317209 | 2800584 |
| HILIC_NEG_371_61011a1_364 | HILIC | NEG | 1.364 | 371.6101 | | | | 5.58E-06 | 0.63 | 0.96 | 8.32E-01 | -0.05 | 0.57 | 2.48E-03 | 0.60 | 0.95 | 3.77E-05 | 0.65 | 1.30 | 822733 | 398780 | 1406933 | 1008866 | 1428466 | 840773 | 1385401 | 1161269 |
| RP_POS_440_86914a0_471 | RP | POS | 0.47 | 440.8691 | | | | 1.63E-07 | -0.66 | 1.34 | 8.32E-01 | 0.05 | 1.17 | 1.71E-04 | -0.63 | 1.41 | 1.33E-04 | -0.68 | 1.43 | 1318122 | 123175 | 1001999 | 526024 | 990777 | 543079 | 1013221 | 513671 |
| HILIC_POS_642_15289a0_508 | HILIC | POS | 0.508 | 642.1529 | | | | 3.84E-01 | 0.14 | 0.79 | 8.33E-01 | -0.05 | 0.56 | 5.56E-01 | 0.12 | 0.41 | 3.80E-01 | 0.17 | 0.84 | 151300 | 75202 | 166647 | 114543 | 169080 | 106986 | 164213 | 122678 |
| HILIC_POS_371_2395a4_007 | HILIC | POS | 4.007 | 371.2395 | | | | 5.15E-01 | 0.14 | 0.60 | 8.33E-01 | -0.04 | 0.56 | 6.10E-01 | 0.12 | 0.21 | 5.05E-01 | 0.16 | 0.28 | 12209483 | 4167683 | 12755022 | 3884091 | 12837432 | 4086611 | 12672612 | 3710249 |
| HILIC_NEG_278_98145a4_988 | HILIC | NEG | 4.988 | 278.9815 | | | | 5.45E-01 | -0.12 | 0.65 | 8.33E-01 | 0.04 | 0.49 | 6.40E-01 | -0.10 | 0.23 | 5.39E-01 | -0.15 | 0.84 | 29200815 | 17838429 | 27000983 | 17639905 | 26626757 | 19312282 | 27375210 | 15982264 |
| HILIC_NEG_204_88899a2_928 | HILIC | NEG | 2.928 | 204.8890 | | | | 1.60E-06 | -0.60 | 1.33 | 8.34E-01 | 0.05 | 0.76 | 3.83E-04 | -0.58 | 1.46 | 6.38E-04 | -0.62 | 1.51 | 389581 | 34001 | 309574 | 146862 | 306464 | 156771 | 312684 | 137768 |
| RP_POS_181_07216a3_264 | RP | POS | 3.26 | 181.0722 | | | | 9.58E-01 | 0.01 | 0.38 | 8.34E-01 | -0.04 | 0.16 | 9.48E-01 | -0.01 | 0.18 | 8.78E-01 | 0.03 | 0.56 | 3755586 | 3276040 | 3794396 | 4462192 | 3888627 | 4544896 | 3700165 | 4422044 |
| RP_POS_235_99773a1_2 | RP | POS | 1.20 | 235.9977 | | | | 4.35E-10 | 0.83 | 1.33 | 8.34E-01 | 0.04 | 0.20 | 1.76E-05 | 0.85 | 1.34 | 2.02E-07 | 0.81 | 1.53 | 1181008 | 574225 | 2531543 | 1722583 | 2495201 | 1441806 | 2567885 | 1978292 |
| HILIC_NEG_350_75714a2_935 | HILIC | NEG | 2.935 | 350.7571 | | | | 2.64E-06 | -0.61 | 1.33 | 8.34E-01 | 0.05 | 0.76 | 3.85E-04 | -0.58 | 1.48 | 6.56E-04 | -0.63 | 1.48 | 415988 | 51047 | 320319 | 172508 | 316687 | 184596 | 323951 | 161315 |
| HILIC_POS_296_00943a4_863 | HILIC | POS | 4.863 | 296.0094 | | | | 8.98E-01 | -0.03 | 0.83 | 8.35E-01 | 0.04 | 0.32 | 9.68E-01 | -0.01 | 0.61 | 8.47E-01 | -0.05 | 0.47 | 322399 | 220231 | 316904 | 175583 | 313213 | 191759 | 320594 | 159651 |
| RP_POS_442_91208a6_535 | RP | POS | 6.54 | 442.9121 | | | | 6.21E-07 | 0.60 | 1.32 | 8.35E-01 | -0.05 | 1.13 | 8.05E-04 | 0.57 | 1.40 | 2.49E-04 | 0.62 | 1.40 | 37438 | 89624 | 762148 | 1353113 | 790563 | 1344358 | 733732 | 1374863 |
| RP_POS_407_31213a11_162 | RP | POS | 11.16 | 407.3121 | | | | 7.47E-01 | -0.05 | 0.76 | 8.35E-01 | 0.05 | 0.66 | 8.81E-01 | -0.03 | 0.72 | 7.08E-01 | -0.07 | 0.62 | 2958866 | 477233 | 2920941 | 811335 | 2903940 | 843642 | 2937942 | 785902 |
| RP_POS_627_26184a7_514 | RP | POS | 7.51 | 627.2618 | | | | 5.03E-01 | -0.12 | 0.47 | 8.35E-01 | -0.04 | 0.69 | 5.08E-01 | -0.15 | 0.40 | 6.21E-01 | -0.10 | 0.36 | 1473446 | 147873 | 1452053 | 180747 | 1455839 | 169131 | 1448267 | 193317 |
| RP_POS_175_07539a6_419 | RP | POS | 6.42 | 175.0754 | | | | 2.59E-01 | -0.22 | 0.45 | 8.35E-01 | 0.04 | 0.18 | 3.72E-01 | -0.20 | 0.39 | 2.74E-01 | -0.24 | 0.39 | 1012961 | 127048 | 982598 | 143750 | 979588 | 143790 | 985609 | 145107 |
| HILIC_NEG_144_92349a5_607 | HILIC | NEG | 5.607 | 144.9235 | | | | 1.33E-01 | -0.32 | 1.18 | 8.35E-01 | 0.04 | 0.63 | 2.16E-01 | -0.30 | 0.56 | 1.31E-01 | -0.34 | 0.93 | 43376471 | 6809572 | 41298849 | 6307381 | 41166814 | 5601914 | 41430883 | 6997213 |
| RP_NEG_248_9606a0_497 | RP | NEG | 0.50 | 248.9606 | | | | 1.62E-04 | 0.47 | 1.25 | 8.36E-01 | -0.05 | 1.18 | 5.37E-03 | 0.45 | 1.29 | 6.49E-03 | 0.50 | 1.32 | 54342739 | 2285249 | 58322472 | 9399645 | 58518788 | 10048540 | 58126157 | 8801022 |
| HILIC_POS_246_17014a1_31 | HILIC | POS | 1.31 | 246.1701 | | | | 2.44E-01 | 0.19 | 0.42 | 8.36E-01 | -0.05 | 0.69 | 3.76E-01 | 0.17 | 0.40 | 2.99E-01 | 0.21 | 0.35 | 7333424 | 3668341 | 8364608 | 5929834 | 8488394 | 6333441 | 8240822 | 5558753 |
| RP_POS_199_1692a8_616 | RP | POS | 8.62 | 199.1692 | | | | 7.24E-03 | 0.47 | 0.96 | 8.36E-01 | 0.04 | 0.24 | 2.39E-02 | 0.49 | 0.76 | 1.80E-02 | 0.45 | 1.11 | 1234017 | 138068 | 1319357 | 188944 | 1315415 | 163587 | 1323298 | 212921 |
| RP_NEG_369_17432a10_358 | RP | NEG | 10.36 | 369.1743 | | | | 3.99E-02 | 0.26 | 0.52 | 8.36E-01 | -0.05 | 0.28 | 2.04E-01 | 0.24 | 0.42 | 6.83E-02 | 0.28 | 0.81 | 11713452 | 15435175 | 24627107 | 55913649 | 25792688 | 50087079 | 23461525 | 61684576 |
| RP_NEG_696_82062a0_496 | RP | NEG | 0.50 | 696.8206 | | | | 5.12E-04 | -0.54 | 1.21 | 8.36E-01 | -0.04 | 1.21 | 3.35E-03 | -0.56 | 1.21 | 6.33E-03 | -0.52 | 1.21 | 1015919 | 156269 | 873951 | 282061 | 879823 | 281946 | 868080 | 284914 |
| RP_NEG_135_03026a0_71 | RP | NEG | 0.71 | 135.0303 | | | | 7.90E-13 | 0.88 | 1.62 | 8.36E-01 | 0.04 | 0.67 | 2.17E-06 | 0.90 | 1.64 | 5.41E-08 | 0.86 | 1.80 | 168902 | 119293 | 2135807 | 2387408 | 2086121 | 2112809 | 2185494 | 2654572 |
| HILIC_POS_355_01123a7_33 | HILIC | POS | 7.33 | 355.0112 | | | | 3.64E-02 | -0.40 | 0.98 | 8.37E-01 | 0.04 | 0.31 | 7.78E-02 | -0.38 | 0.73 | 5.38E-02 | -0.42 | 0.97 | 5478625 | 138996 | 5415858 | 159999 | 5412534 | 163311 | 5419181 | 158205 |
| HILIC_POS_293_09732a7_687 | HILIC | POS | 7.687 | 293.0973 | | | | 5.15E-01 | -0.12 | 0.46 | 8.37E-01 | 0.04 | 0.46 | 6.21E-01 | -0.10 | 0.19 | 5.25E-01 | -0.14 | 0.23 | 30653375 | 2268801 | 30337660 | 2680823 | 30281967 | 2973381 | 30393353 | 2381798 |
| RP_POS_722_52686a12_991 | RP | POS | 12.99 | 722.5269 | | | | 6.72E-02 | -0.39 | 0.75 | 8.37E-01 | -0.04 | 0.23 | 8.25E-02 | -0.41 | 0.75 | 1.10E-01 | -0.37 | 0.62 | 1495390 | 393846 | 1348060 | 367498 | 1355679 | 359601 | 1340441 | 378728 |
| RP_POS_769_55811a13_157 | RP | POS | 13.16 | 769.5581 | | | | 4.43E-02 | -0.42 | 0.69 | 8.37E-01 | 0.04 | 0.21 | 8.49E-02 | -0.40 | 0.68 | 5.54E-02 | -0.44 | 0.82 | 26314940 | 3502989 | 24852824 | 3459557 | 24781167 | 3426993 | 24924480 | 3525133 |
| RP_POS_778_53552a12_82 | RP | POS | 12.82 | 778.5355 | | | | 1.22E-04 | 0.61 | 1.03 | 8.37E-01 | 0.04 | 0.21 | 1.63E-03 | 0.64 | 1.11 | 1.26E-03 | 0.59 | 1.14 | 14406606 | 7363674 | 21619807 | 12366812 | 21363701 | 11472965 | 21875914 | 13312944 |
| RP_NEG_324_89612a15_46 | RP | NEG | 15.46 | 324.8961 | | | | 3.21E-02 | -0.42 | 0.96 | 8.37E-01 | -0.04 | 0.26 | 3.86E-02 | -0.44 | 0.84 | 7.72E-02 | -0.40 | 0.90 | 3281319 | 241530 | 3171408 | 260378 | 3176800 | 280894 | 3166016 | 240866 |
| RP_NEG_360_27655a8_963 | RP | NEG | 8.96 | 360.2766 | | | | 2.85E-01 | 0.22 | 0.49 | 8.37E-01 | 0.04 | 0.27 | 3.18E-01 | 0.25 | 0.56 | 3.50E-01 | 0.20 | 0.47 | 502981 | 970565 | 713546 | 923992 | 694418 | 769985 | 732674 | 1063636 |
| HILIC_NEG_466_80344a2_91 | HILIC | NEG | 2.91 | 466.8034 | | | | 1.59E-07 | -0.65 | 1.36 | 8.37E-01 | 0.05 | 0.77 | 1.20E-04 | -0.63 | 1.52 | 2.20E-04 | -0.67 | 1.54 | 1873288 | 151390 | 1427452 | 754108 | 1411847 | 801934 | 1443057 | 710893 |
| HILIC_NEG_205_83957a2_943 | HILIC | NEG | 2.943 | 205.8396 | | | | 2.49E-02 | 0.36 | 1.03 | 8.37E-01 | -0.04 | 0.77 | 7.64E-02 | 0.34 | 0.72 | 5.42E-02 | 0.38 | 0.97 | 474898 | 93621 | 526778 | 155882 | 530001 | 161382 | 523554 | 151753 |
| RP_POS_271_11563a4_09 | RP | POS | 4.09 | 271.1156 | | | | 2.82E-01 | 0.20 | 0.49 | 8.38E-01 | -0.04 | 0.32 | 4.21E-01 | 0.18 | 0.45 | 2.72E-01 | 0.22 | 0.94 | 3949534 | 174809 | 3989821 | 205110 | 3994053 | 182263 | 3985589 | 227469 |
| HILIC_POS_74_09716a5_481 | HILIC | POS | 5.481 | 74.0972 | | | | 8.76E-01 | -0.03 | 0.37 | 8.38E-01 | -0.04 | 0.34 | 8.23E-01 | -0.05 | 0.21 | 9.58E-01 | -0.01 | 0.32 | 14482436 | 2651101 | 14398140 | 2612505 | 14451942 | 2424090 | 14344339 | 2812033 |
| RP_NEG_824_54669a12_463 | RP | NEG | 12.46 | 824.5467 | | | | 5.15E-03 | -0.62 | 1.06 | 8.38E-01 | -0.04 | 0.57 | 1.07E-02 | -0.63 | 1.14 | 8.49E-03 | -0.60 | 1.02 | 2345081 | 1247886 | 1621828 | 1101174 | 1644508 | 920290 | 1599148 | 1265675 |
| RP_NEG_356_92645a15_32 | RP | NEG | 15.32 | 356.9265 | | | | 6.69E-01 | -0.08 | 0.74 | 8.38E-01 | -0.04 | 0.43 | 6.33E-01 | -0.10 | 0.69 | 7.89E-01 | -0.06 | 0.46 | 1084718 | 34596 | 1081538 | 41782 | 1082399 | 42912 | 1080678 | 41039 |
| HILIC_NEG_131_91556a2_963 | HILIC | NEG | 2.963 | 131.9156 | | | | 6.68E-01 | -0.10 | 0.40 | 8.38E-01 | -0.04 | 0.72 | 6.41E-01 | -0.12 | 0.29 | 7.50E-01 | -0.08 | 0.16 | 576520 | 193068 | 560148 | 164062 | 563524 | 154535 | 556772 | 174579 |
| RP_POS_501_61377a6_787 | RP | POS | 6.79 | 501.6138 | | | | 2.75E-07 | 0.61 | 1.33 | 8.38E-01 | -0.05 | 1.13 | 5.10E-04 | 0.59 | 1.42 | 1.83E-04 | 0.64 | 1.41 | 26522 | 53022 | 1459222 | 2595044 | 1512592 | 2595349 | 1405851 | 2619978 |
| RP_NEG_239_76036a0_569 | RP | NEG | 0.57 | 239.7604 | | | | 2.77E-04 | 0.50 | 0.88 | 8.38E-01 | -0.05 | 0.85 | 1.48E-02 | 0.48 | 0.99 | 1.11E-03 | 0.52 | 1.09 | 1120013 | 341313 | 1512994 | 859254 | 1530664 | 741720 | 1495323 | 970009 |
| RP_NEG_286_97781a7_698 | RP | NEG | 7.70 | 286.9778 | | | | 9.84E-01 | 0.00 | 0.88 | 8.38E-01 | -0.04 | 0.15 | 9.49E-01 | -0.02 | 0.66 | 9.20E-01 | 0.02 | 0.78 | 2550043 | 206430 | 2550874 | 178025 | 2554532 | 180650 | 2547216 | 177117 |
| RP_POS_274_90555a15_367 | RP | POS | 15.37 | 274.9056 | | | | 1.62E-01 | -0.20 | 0.62 | 8.39E-01 | -0.05 | 0.34 | 2.44E-01 | -0.22 | 0.99 | 3.17E-01 | -0.17 | 0.67 | 3567226 | 115416 | 3518996 | 275280 | 3524642 | 261970 | 3513351 | 290536 |
| RP_POS_553_4585a12_268 | RP | POS | 12.27 | 553.4585 | | | | 5.16E-02 | -0.42 | 0.73 | 8.39E-01 | 0.04 | 0.44 | 9.64E-02 | -0.41 | 0.77 | 5.53E-02 | -0.44 | 0.93 | 13569585 | 5147074 | 11535633 | 4582625 | 11441715 | 4208936 | 11629551 | 4969541 |
| HILIC_POS_294_93857a4_323 | HILIC | POS | 4.323 | 294.9386 | | | | 1.42E-01 | 0.24 | 1.03 | 8.39E-01 | -0.04 | 0.84 | 2.54E-01 | 0.22 | 0.58 | 1.90E-01 | 0.27 | 0.91 | 30103976 | 5735528 | 32097763 | 8817428 | 32278262 | 9151746 | 31917264 | 8559194 |
| HILIC_POS_199_18044a5_783 | HILIC | POS | 5.783 | 199.1804 | | | | 2.99E-01 | -0.22 | 0.71 | 8.39E-01 | 0.04 | 0.55 | 4.05E-01 | -0.20 | 0.37 | 2.93E-01 | -0.24 | 0.52 | 3834276 | 839801 | 3657123 | 802993 | 3640704 | 750530 | 3673541 | 859599 |
| RP_NEG_207_93144a0_104 | RP | NEG | 0.10 | 207.9314 | | | | 4.22E-01 | 0.16 | 0.54 | 8.39E-01 | 0.04 | 0.31 | 4.18E-01 | 0.18 | 0.47 | 5.41E-01 | 0.14 | 0.57 | 3498477 | 1392848 | 3732752 | 1527481 | 3701525 | 1559064 | 3763979 | 1510411 |
| RP_POS_223_0943a7_165 | RP | POS | 7.17 | 223.0943 | Unknown | 223.0946(100) | 4 | 1.05E-01 | -0.36 | 0.64 | 8.39E-01 | 0.04 | 0.32 | 1.45E-01 | -0.34 | 0.62 | 1.29E-01 | -0.38 | 0.61 | 7728072 | 4557994 | 6240204 | 4000028 | 6158499 | 4426670 | 6321910 | 3566224 |
| RP_POS_371_22498a11_608 | RP | POS | 11.61 | 371.2250 | | | | 3.65E-02 | -0.40 | 0.78 | 8.40E-01 | 0.04 | 0.58 | 5.26E-02 | -0.38 | 0.80 | 7.29E-02 | -0.43 | 0.84 | 3716549 | 360815 | 3554809 | 405260 | 3546542 | 483656 | 3563076 | 312666 |
| HILIC_POS_226_95108a4_323 | HILIC | POS | 4.323 | 226.9511 | | | | 1.65E-01 | 0.23 | 1.02 | 8.40E-01 | -0.04 | 0.85 | 2.82E-01 | 0.21 | 0.58 | 2.12E-01 | 0.25 | 0.90 | 324962027 | 61958734 | 345230486 | 94220010 | 347151385 | 97835426 | 343309587 | 91414942 |
| HILIC_POS_357_00858a7_33 | HILIC | POS | 7.33 | 357.0086 | | | | 9.51E-03 | -0.53 | 1.25 | 8.40E-01 | 0.04 | 0.54 | 2.28E-02 | -0.51 | 1.03 | 1.52E-02 | -0.55 | 1.32 | 2935666 | 115069 | 2872760 | 114936 | 2870418 | 116681 | 2875103 | 114299 |
| HILIC_NEG_214_0769a0_929 | HILIC | NEG | 0.929 | 214.0769 | | | | 2.36E-08 | 0.72 | 1.22 | 8.40E-01 | 0.04 | 1.00 | 2.42E-04 | 0.74 | 1.13 | 1.25E-06 | 0.70 | 1.57 | 1179442 | 946347 | 3389754 | 3316441 | 3322149 | 2586499 | 3457359 | 3939889 |
| HILIC_NEG_115_92069a2_939 | HILIC | NEG | 2.939 | 115.9207 | | | | 1.52E-04 | 0.50 | 1.11 | 8.40E-01 | -0.04 | 0.78 | 2.80E-03 | 0.48 | 1.06 | 5.84E-03 | 0.53 | 1.18 | 23305151 | 1550772 | 25364828 | 4507639 | 25456651 | 4965924 | 25273005 | 4046565 |
| RP_POS_226_95076a15_422 | RP | POS | 15.42 | 226.9508 | | | | 3.91E-01 | 0.16 | 0.39 | 8.40E-01 | 0.04 | 0.43 | 4.14E-01 | 0.18 | 0.37 | 5.03E-01 | 0.14 | 0.42 | 2748088 | 1044734 | 2941682 | 1271285 | 2915795 | 1183931 | 2967570 | 1364645 |
| HILIC_NEG_301_68372a1_368 | HILIC | NEG | 1.368 | 301.6837 | | | | 9.96E-11 | -0.90 | 1.70 | 8.40E-01 | -0.04 | 0.45 | 9.47E-07 | -0.92 | 1.70 | 4.67E-07 | -0.88 | 1.92 | 338354 | 39852 | 252382 | 99072 | 254398 | 94746 | 250367 | 104142 |
| HILIC_NEG_201_80197a2_952 | HILIC | NEG | 2.952 | 201.8020 | | | | 1.10E-06 | 0.60 | 1.32 | 8.40E-01 | -0.04 | 0.77 | 4.86E-04 | 0.57 | 1.46 | 4.95E-04 | 0.62 | 1.52 | 1961349 | 180439 | 2501276 | 999683 | 2521586 | 1043846 | 2480965 | 963676 |
| HILIC_POS_448_74155a3_007 | HILIC | POS | 3.007 | 448.7416 | | | | 3.20E-05 | -0.55 | 1.36 | 8.41E-01 | 0.04 | 0.78 | 1.85E-03 | -0.52 | 1.47 | 1.87E-03 | -0.57 | 1.49 | 1368659 | 204412 | 1056609 | 627363 | 1043891 | 659441 | 1069327 | 600006 |
| RP_NEG_294_98242a7_424 | RP | NEG | 7.42 | 294.9824 | | | | 8.59E-03 | -0.49 | 0.91 | 8.41E-01 | 0.04 | 0.21 | 2.85E-02 | -0.47 | 0.78 | 1.53E-02 | -0.51 | 0.93 | 8065436 | 1444065 | 7225993 | 1747782 | 7190564 | 1716681 | 7261422 | 1795069 |
| RP_POS_352_24429a11_609 | RP | POS | 11.61 | 352.2443 | | | | 1.46E-01 | -0.32 | 0.67 | 8.41E-01 | 0.04 | 0.19 | 1.92E-01 | -0.31 | 0.60 | 1.70E-01 | -0.34 | 0.52 | 3958098 | 805257 | 3724799 | 682319 | 3710988 | 757769 | 3738610 | 604992 |
| HILIC_POS_274_20102a0_963 | HILIC | POS | 0.963 | 274.2010 | | | | 7.09E-01 | -0.08 | 0.42 | 8.41E-01 | 0.04 | 0.56 | 8.10E-01 | -0.06 | 0.10 | 6.45E-01 | -0.10 | 0.41 | 746359 | 611251 | 701188 | 533271 | 690395 | 378063 | 711980 | 656802 |
| HILIC_NEG_276_84253a7_667 | HILIC | NEG | 7.667 | 276.8425 | | | | 4.18E-01 | 0.15 | 0.77 | 8.41E-01 | 0.04 | 0.72 | 4.22E-01 | 0.17 | 0.55 | 5.53E-01 | 0.13 | 0.65 | 2570137 | 191341 | 2604146 | 244539 | 2599203 | 248184 | 2609090 | 243256 |
| HILIC_POS_394_31659a3_166 | HILIC | POS | 3.166 | 394.3166 | | | | 2.98E-07 | 0.62 | 1.31 | 8.41E-01 | -0.04 | 0.76 | 3.00E-04 | 0.59 | 1.44 | 2.90E-04 | 0.64 | 1.51 | 1239904 | 421222 | 3474128 | 4012185 | 3555190 | 4171680 | 3393067 | 3886831 |
| RP_POS_543_34448a10_931 | RP | POS | 10.93 | 543.3445 | | | | 7.26E-02 | -0.45 | 0.98 | 8.42E-01 | -0.03 | 1.14 | 8.18E-02 | -0.47 | 0.86 | 9.68E-02 | -0.43 | 0.86 | 424237 | 208326 | 351582 | 140213 | 354406 | 130721 | 348758 | 150388 |
| HILIC_NEG_144_92342a2_172 | HILIC | NEG | 2.172 | 144.9234 | | | | 5.14E-01 | 0.13 | 0.71 | 8.42E-01 | -0.04 | 0.52 | 6.36E-01 | 0.11 | 0.40 | 4.92E-01 | 0.15 | 0.38 | 28479391 | 6517882 | 29367960 | 7088502 | 29510630 | 6710952 | 29225290 | 7512705 |
| HILIC_NEG_118_92426a2_171 | HILIC | NEG | 2.171 | 118.9243 | | | | 4.41E-01 | 0.15 | 0.60 | 8.42E-01 | -0.04 | 0.66 | 5.63E-01 | 0.13 | 0.21 | 4.34E-01 | 0.17 | 0.33 | 2118905 | 528433 | 2204786 | 592873 | 2216701 | 579122 | 2192870 | 611961 |
| RP_POS_508_85791a0_472 | RP | POS | 0.47 | 508.8579 | | | | 1.29E-07 | -0.66 | 1.34 | 8.42E-01 | 0.04 | 1.18 | 1.52E-04 | -0.63 | 1.42 | 1.46E-04 | -0.68 | 1.43 | 849971 | 74028 | 634655 | 360445 | 627414 | 374153 | 641896 | 349850 |
| RP_POS_286_77121a0_52 | RP | POS | 0.52 | 286.7712 | | | | 4.59E-06 | -0.63 | 1.27 | 8.43E-01 | 0.04 | 1.14 | 3.92E-04 | -0.61 | 1.35 | 5.23E-04 | -0.65 | 1.31 | 375640 | 65007 | 277962 | 168399 | 274591 | 178380 | 281333 | 159539 |
| HILIC_POS_524_67102a3_012 | HILIC | POS | 3.012 | 524.6710 | | | | 3.51E-05 | -0.59 | 1.32 | 8.43E-01 | -0.04 | 0.77 | 4.77E-04 | -0.61 | 1.48 | 2.59E-03 | -0.57 | 1.40 | 297437 | 58674 | 225440 | 131218 | 228066 | 138937 | 222815 | 124379 |
| HILIC_POS_240_18048a0_625 | HILIC | POS | 0.625 | 240.1805 | | | | 8.91E-01 | 0.02 | 1.00 | 8.43E-01 | -0.04 | 0.85 | 9.86E-01 | 0.00 | 0.28 | 8.31E-01 | 0.05 | 0.08 | 787281 | 1366961 | 828293 | 1754362 | 863381 | 1841655 | 793205 | 1680529 |
| RP_POS_222_86752a0_519 | RP | POS | 0.52 | 222.8675 | | | | 4.83E-06 | -0.79 | 1.37 | 8.43E-01 | 0.04 | 1.04 | 7.16E-05 | -0.77 | 1.42 | 9.17E-05 | -0.81 | 1.36 | 511287 | 102428 | 396241 | 147497 | 393292 | 156875 | 399190 | 139023 |
| RP_POS_202_04749a3_625 | RP | POS | 3.63 | 202.0475 | | | | 2.12E-02 | -0.50 | 0.89 | 8.43E-01 | 0.04 | 0.78 | 5.02E-02 | -0.48 | 0.77 | 2.23E-02 | -0.52 | 1.07 | 5208975 | 4371076 | 3139687 | 3969802 | 3060352 | 3449522 | 3219023 | 4464104 |
| HILIC_NEG_255_82175a3_026 | HILIC | NEG | 3.026 | 255.8218 | | | | 1.26E-02 | -0.43 | 1.13 | 8.43E-01 | 0.04 | 0.82 | 4.25E-02 | -0.41 | 0.87 | 2.61E-02 | -0.45 | 0.82 | 9198269 | 1404745 | 8386723 | 1976686 | 8347243 | 1990734 | 8426203 | 1981946 |
| RP_POS_295_18796a9_812 | RP | POS | 9.81 | 295.1880 | | | | 3.99E-03 | -0.54 | 0.93 | 8.43E-01 | -0.04 | 0.51 | 8.93E-03 | -0.56 | 1.03 | 1.41E-02 | -0.52 | 0.98 | 1575596 | 59302 | 1537680 | 71337 | 1539103 | 70968 | 1536256 | 72395 |
| RP_POS_765_3783a13_57 | RP | POS | 13.57 | 765.3783 | | | | 4.00E-01 | 0.22 | 0.45 | 8.43E-01 | 0.03 | 0.47 | 3.86E-01 | 0.23 | 0.68 | 4.65E-01 | 0.20 | 0.47 | 1334657 | 294266 | 1381686 | 186180 | 1377975 | 193176 | 1385396 | 180800 |
| RP_NEG_299_25961a10_652 | RP | NEG | 10.65 | 299.2596 | | | | 8.72E-03 | -0.51 | 0.98 | 8.43E-01 | -0.04 | 0.74 | 1.25E-02 | -0.53 | 1.01 | 2.94E-02 | -0.49 | 0.90 | 6898892 | 849525 | 6415620 | 956474 | 6434678 | 1028770 | 6396562 | 888395 |
| RP_POS_415_28122a10_705 | RP | POS | 10.71 | 415.2812 | | | | 3.77E-02 | -0.40 | 0.78 | 8.43E-01 | 0.04 | 0.51 | 9.53E-02 | -0.38 | 0.69 | 4.43E-02 | -0.42 | 0.89 | 2794032 | 765821 | 2449970 | 888055 | 2432280 | 801060 | 2467659 | 975214 |
| RP_POS_658_51111a10_658 | RP | POS | 10.66 | 658.5111 | | | | 3.41E-01 | -0.19 | 0.47 | 8.43E-01 | 0.04 | 0.47 | 4.52E-01 | -0.17 | 0.49 | 3.49E-01 | -0.21 | 0.55 | 4794378 | 380662 | 4718664 | 410828 | 4710487 | 411135 | 4726841 | 414530 |
| RP_POS_276_19904a7_791 | RP | POS | 7.79 | 276.1990 | | | | 2.89E-04 | 0.64 | 1.38 | 8.44E-01 | -0.04 | 1.12 | 1.77E-03 | 0.62 | 1.31 | 1.57E-03 | 0.66 | 1.22 | 694006 | 349059 | 989945 | 469489 | 999278 | 494146 | 980612 | 448309 |

**Supplementary table 1: Metabolomics results** (in asceding order according to p-value from progressive ALD vs non-progressive ALD comparison, with molecular features with ID_level of 1-2 first)

| | | | | | | | | all_ALD_vs_control | | | Prog_ALD_vs_non-prog_ALD | | | Prog_ALD_vs_control_ALD | | | Non-prog_ALD_vs_control | | | Healthy_control | | all_ALD | | Non-prog_ALD | | Progressive_ALD | |
|---|---|---|---|---|---|---|---|---|---|---|---|---|---|---|---|---|---|---|---|---|---|---|---|---|---|---|---|
| | | | | | | | | t-test | Cohen's | PLS-DA | t-test | Cohen's | PLS-DA | t-test | Cohen's | PLS-DA | t-test | Cohen's | PLS-DA | | | | | | | | |
| Feature_ID | RP_HILIC | POS_NEG | Average.Rt | Average.Mz | Metabolite.name | curated_MSMS | ID_level | p | d | VIP | p | d | VIP | p | d | VIP | p | d | VIP | Mean | SD | Mean | SD | Mean | SD | Mean | SD |
| RP_NEG_313_0658a0_622 | RP | NEG | 0.62 | 313.0658 | | | | 1.14E-09 | -0.77 | 1.41 | 8.44E-01 | 0.04 | 1.16 | 1.10E-05 | -0.75 | 1.53 | 1.12E-05 | -0.79 | 1.49 | 24946427 | 2638932 | 16902830 | 11283330 | 16678678 | 11671328 | 17126983 | 10995584 |
| RP_NEG_563_09882a9_006 | RP | NEG | 9.01 | 563.0988 | | | | 4.86E-01 | 0.14 | 0.71 | 8.44E-01 | -0.04 | 0.20 | 6.10E-01 | 0.12 | 0.64 | 4.56E-01 | 0.16 | 0.45 | 1629724 | 116795 | 1646507 | 120905 | 1648904 | 106863 | 1644111 | 134548 |
| RP_POS_252_95801a15_499 | RP | POS | 15.50 | 252.9580 | | | | 3.39E-01 | -0.19 | 0.54 | 8.44E-01 | 0.04 | 0.28 | 4.22E-01 | -0.17 | 0.47 | 3.65E-01 | -0.21 | 0.57 | 2102891 | 85022 | 2086164 | 86547 | 2084449 | 95812 | 2087879 | 77120 |
| RP_POS_165_02408a0_45 | RP | POS | 0.45 | 165.0241 | | | | 1.31E-06 | -0.65 | 1.30 | 8.44E-01 | -0.04 | 1.11 | 1.37E-04 | -0.67 | 1.38 | 4.44E-04 | -0.63 | 1.36 | 690334 | 92100 | 537169 | 253837 | 542187 | 258863 | 532152 | 251236 |
| RP_NEG_367_15869a8_933 | RP | NEG | 8.93 | 367.1587 | | | | 1.31E-01 | 0.25 | 0.89 | 8.45E-01 | 0.04 | 0.67 | 2.13E-01 | 0.28 | 0.58 | 1.87E-01 | 0.23 | 0.68 | 161252875 | 128541918 | 206545925 | 190451670 | 202784833 | 150681620 | 210307016 | 224833492 |
| RP_NEG_319_05618a8_812 | RP | NEG | 8.81 | 319.0562 | | | | 3.51E-02 | 0.29 | 0.61 | 8.45E-01 | 0.04 | 0.64 | 3.54E-02 | 0.31 | 0.77 | 1.93E-01 | 0.27 | 0.56 | 38756826 | 4497943 | 41751171 | 11592359 | 41522283 | 13773870 | 41980058 | 9037791 |
| RP_POS_432_23804a8_659 | RP | POS | 8.66 | 432.2380 | | | | 1.84E-02 | 0.36 | 0.76 | 8.45E-01 | -0.04 | 0.74 | 9.85E-02 | 0.33 | 0.69 | 2.83E-02 | 0.38 | 0.85 | 318485 | 150782 | 412820 | 289821 | 418525 | 254604 | 407114 | 323755 |
| RP_NEG_437_16223a9_327 | RP | NEG | 9.33 | 437.1622 | | | | 6.30E-01 | 0.09 | 0.39 | 8.45E-01 | -0.04 | 0.58 | 7.67E-01 | 0.07 | 0.23 | 5.67E-01 | 0.11 | 0.55 | 1256572 | 1305285 | 1392079 | 1580634 | 1423196 | 1240176 | 1360962 | 1872908 |
| HILIC_NEG_215_07324a0_929 | HILIC | NEG | 0.929 | 215.0732 | | | | 1.96E-08 | 0.72 | 1.22 | 8.45E-01 | 0.04 | 0.99 | 2.72E-04 | 0.74 | 1.12 | 7.27E-07 | 0.70 | 1.61 | 5033655 | 3871106 | 14449342 | 14147218 | 14170820 | 10770367 | 14727864 | 16976836 |
| RP_POS_363_30911a10_535 | RP | POS | 10.54 | 363.3091 | | | | 4.85E-01 | -0.16 | 0.65 | 8.45E-01 | 0.04 | 0.65 | 5.67E-01 | -0.14 | 0.54 | 4.79E-01 | -0.17 | 0.75 | 935159 | 222748 | 904331 | 190812 | 900583 | 201128 | 908079 | 181879 |
| RP_NEG_573_12897a9_006 | RP | NEG | 9.01 | 573.1290 | | | | 9.70E-01 | -0.01 | 0.58 | 8.46E-01 | -0.04 | 0.74 | 9.00E-01 | -0.03 | 0.25 | 9.52E-01 | 0.01 | 0.34 | 16879032 | 951954 | 16871550 | 1090904 | 16892907 | 1089527 | 16850192 | 1102919 |
| HILIC_NEG_248_96017a4_232 | HILIC | NEG | 4.232 | 248.9602 | | | | 1.83E-01 | 0.22 | 1.03 | 8.46E-01 | -0.04 | 0.85 | 3.04E-01 | 0.20 | 0.58 | 2.30E-01 | 0.24 | 0.89 | 69498304 | 13219343 | 73632138 | 20013623 | 74023525 | 20650373 | 73240751 | 19557968 |
| HILIC_NEG_132_92331a4_278 | HILIC | NEG | 4.278 | 132.9233 | | | | 6.50E-01 | 0.09 | 0.94 | 8.47E-01 | -0.04 | 0.80 | 7.62E-01 | 0.06 | 0.12 | 6.26E-01 | 0.11 | 0.37 | 26445327 | 4883974 | 26919169 | 5782963 | 27031869 | 5870640 | 26806469 | 5751304 |
| HILIC_NEG_196_8856a7_667 | HILIC | NEG | 7.667 | 196.8856 | | | | 6.14E-01 | 0.10 | 0.71 | 8.47E-01 | 0.04 | 0.71 | 5.94E-01 | 0.12 | 0.48 | 7.27E-01 | 0.08 | 0.41 | 8160090 | 306648 | 8192908 | 355192 | 8185988 | 355441 | 8199828 | 358415 |
| RP_POS_132_99835a0_45 | RP | POS | 0.45 | 132.9984 | | | | 1.17E-06 | -0.65 | 1.31 | 8.47E-01 | -0.04 | 1.12 | 1.26E-04 | -0.67 | 1.38 | 4.74E-04 | -0.63 | 1.38 | 1378301 | 194429 | 1046032 | 553170 | 1056796 | 567871 | 1035268 | 543622 |
| RP_POS_796_61877a14_278 | RP | POS | 14.28 | 796.6188 | | | | 5.59E-01 | 0.09 | 0.22 | 8.47E-01 | -0.04 | 0.14 | 6.97E-01 | 0.07 | 0.26 | 5.83E-01 | 0.11 | 0.26 | 3996212 | 2692643 | 4394442 | 4844323 | 4488640 | 5337157 | 4300244 | 4348621 |
| RP_POS_262_87753a0_249 | RP | POS | 0.25 | 262.8775 | | | | 8.71E-01 | 0.03 | 0.56 | 8.47E-01 | 0.04 | 0.16 | 8.11E-01 | 0.05 | 0.31 | 9.56E-01 | 0.01 | 0.26 | 1023477 | 109805 | 1027147 | 114392 | 1024929 | 123021 | 1029365 | 106272 |
| RP_POS_138_90623a0_521 | RP | POS | 0.52 | 138.9062 | | | | 5.41E-05 | 0.50 | 1.21 | 8.48E-01 | -0.04 | 1.11 | 4.17E-03 | 0.48 | 1.20 | 3.16E-03 | 0.52 | 1.33 | 342440 | 48648 | 447049 | 234596 | 451591 | 242450 | 442506 | 228843 |
| HILIC_NEG_436_71536a2_933 | HILIC | NEG | 2.933 | 436.7154 | | | | 8.47E-07 | -0.65 | 1.33 | 8.48E-01 | 0.04 | 0.75 | 1.65E-04 | -0.62 | 1.48 | 3.42E-04 | -0.67 | 1.46 | 240532 | 32198 | 180430 | 101266 | 178469 | 108484 | 182390 | 94564 |
| RP_POS_261_10995a7_349 | RP | POS | 7.35 | 261.1100 | | | | 3.74E-01 | 0.16 | 0.49 | 8.48E-01 | -0.04 | 0.32 | 5.01E-01 | 0.14 | 0.36 | 3.97E-01 | 0.18 | 0.52 | 701757 | 809579 | 862609 | 1084878 | 883589 | 1122530 | 841628 | 1056867 |
| HILIC_NEG_550_76477a2_91 | HILIC | NEG | 2.91 | 550.7648 | | | | 3.37E-07 | -0.63 | 1.35 | 8.48E-01 | 0.04 | 0.77 | 1.82E-04 | -0.61 | 1.51 | 3.15E-04 | -0.65 | 1.54 | 1490118 | 125979 | 1136347 | 615696 | 1124454 | 653436 | 1148240 | 581922 |
| RP_POS_284_88412a0_251 | RP | POS | 0.25 | 284.8841 | | | | 3.09E-01 | 0.23 | 0.68 | 8.48E-01 | 0.04 | 0.13 | 3.16E-01 | 0.25 | 0.52 | 3.87E-01 | 0.21 | 0.49 | 374258 | 77074 | 389807 | 65566 | 388543 | 64344 | 391070 | 67395 |
| RP_POS_288_76837a0_522 | RP | POS | 0.52 | 288.7684 | | | | 1.88E-05 | -0.57 | 1.24 | 8.48E-01 | 0.04 | 1.15 | 1.14E-03 | -0.54 | 1.29 | 1.52E-03 | -0.59 | 1.29 | 538562 | 85274 | 406660 | 255755 | 401733 | 271681 | 411587 | 241445 |
| HILIC_POS_462_76495a2_992 | HILIC | POS | 2.992 | 462.7650 | | | | 1.14E-05 | -0.57 | 1.36 | 8.49E-01 | 0.04 | 0.77 | 9.28E-04 | -0.55 | 1.49 | 1.28E-03 | -0.59 | 1.49 | 740096 | 102298 | 568255 | 330094 | 561905 | 350539 | 574604 | 311749 |
| RP_POS_366_1864a10_978 | RP | POS | 10.98 | 366.1864 | | | | 2.60E-02 | 0.41 | 1.47 | 8.49E-01 | -0.04 | 1.18 | 7.51E-02 | 0.39 | 1.27 | 3.41E-02 | 0.43 | 1.31 | 7947614 | 2015894 | 8938305 | 2488838 | 8986038 | 2280846 | 8890572 | 2703357 |
| RP_POS_508_33978a10_261 | RP | POS | 10.26 | 508.3398 | | | | 1.29E-03 | 0.54 | 1.17 | 8.49E-01 | 0.04 | 0.80 | 9.06E-03 | 0.56 | 1.07 | 4.06E-03 | 0.52 | 1.13 | 664955 | 278933 | 880743 | 417782 | 872736 | 352533 | 888750 | 477740 |
| RP_NEG_146_93761a15_662 | RP | NEG | 15.66 | 146.9376 | | | | 8.31E-01 | 0.05 | 0.46 | 8.49E-01 | 0.04 | 0.60 | 7.76E-01 | 0.06 | 0.25 | 9.11E-01 | 0.03 | 0.60 | 6645537 | 1464650 | 6708099 | 1325434 | 6682712 | 1469188 | 6733486 | 1178803 |
| RP_NEG_636_82013a0_498 | RP | NEG | 0.50 | 636.8201 | | | | 6.03E-05 | -0.52 | 1.25 | 8.49E-01 | -0.04 | 1.23 | 1.20E-03 | -0.54 | 1.34 | 6.56E-03 | -0.50 | 1.30 | 6056277 | 835371 | 4716469 | 2872524 | 4771417 | 3051459 | 4661522 | 2711755 |
| RP_NEG_297_24393a10_54 | RP | NEG | 10.54 | 297.2439 | | | | 6.68E-02 | -0.37 | 0.74 | 8.50E-01 | -0.04 | 0.66 | 7.59E-02 | -0.39 | 0.77 | 1.27E-01 | -0.35 | 0.74 | 2183731 | 324499 | 2059691 | 332219 | 2066031 | 355509 | 2053350 | 310682 |
| HILIC_NEG_115_04009a0_999 | HILIC | NEG | 0.999 | 115.0401 | | | | 8.43E-02 | 0.26 | 0.92 | 8.50E-01 | -0.04 | 1.07 | 1.98E-01 | 0.24 | 0.44 | 1.33E-01 | 0.28 | 0.72 | 7342123 | 1902831 | 8191901 | 3525470 | 8259129 | 3543472 | 8124674 | 3542050 |
| RP_POS_534_38629a14_267 | RP | POS | 14.27 | 534.3863 | | | | 7.50E-01 | 0.06 | 0.43 | 8.50E-01 | 0.04 | 0.27 | 7.25E-01 | 0.08 | 0.35 | 8.33E-01 | 0.05 | 0.62 | 2084836 | 609260 | 2124598 | 619519 | 2112800 | 536315 | 2136395 | 698227 |
| RP_POS_784_58496a13_572 | RP | POS | 13.57 | 784.5850 | | | | 5.09E-08 | 0.76 | 1.32 | 8.50E-01 | 0.04 | 0.27 | 1.91E-05 | 0.78 | 1.37 | 4.24E-05 | 0.74 | 1.36 | 15193814 | 11871484 | 36260441 | 29607819 | 35696792 | 29670712 | 36824091 | 29835157 |
| RP_POS_239_06401a0_612 | RP | POS | 0.61 | 239.0640 | | | | 1.46E-07 | -0.71 | 1.35 | 8.50E-01 | 0.04 | 1.14 | 8.39E-05 | -0.68 | 1.42 | 6.65E-05 | -0.73 | 1.42 | 688268 | 111033 | 482025 | 314380 | 476041 | 323534 | 488008 | 308122 |
| HILIC_NEG_213_87982a2_945 | HILIC | NEG | 2.945 | 213.8798 | | | | 4.93E-01 | 0.13 | 0.78 | 8.50E-01 | -0.04 | 0.75 | 6.06E-01 | 0.11 | 0.19 | 4.95E-01 | 0.15 | 0.26 | 272650 | 59796 | 281396 | 69920 | 282726 | 72080 | 280065 | 68397 |
| RP_POS_323_21811a10_083 | RP | POS | 10.08 | 323.2181 | | | | 4.06E-04 | -0.77 | 1.20 | 8.50E-01 | -0.04 | 0.96 | 9.02E-04 | -0.79 | 1.23 | 1.15E-03 | -0.75 | 1.17 | 2454852 | 203033 | 2301559 | 184401 | 2305067 | 178268 | 2298052 | 192085 |
| RP_POS_576_53491a15_1 | RP | POS | 15.10 | 576.5349 | | | | 9.23E-01 | -0.02 | 0.22 | 8.50E-01 | 0.04 | 0.12 | 9.92E-01 | 0.00 | 0.38 | 8.69E-01 | -0.04 | 0.33 | 30219667 | 10392557 | 30021057 | 9254651 | 29845062 | 9308325 | 30197052 | 9291767 |
| HILIC_NEG_260_76074a2_947 | HILIC | NEG | 2.947 | 260.7607 | | | | 7.44E-06 | -0.59 | 1.33 | 8.50E-01 | 0.04 | 0.76 | 6.83E-04 | -0.57 | 1.45 | 8.90E-04 | -0.61 | 1.48 | 1023989 | 136085 | 802313 | 409283 | 794529 | 432763 | 810096 | 388633 |
| HILIC_NEG_528_70941a2_928 | HILIC | NEG | 2.928 | 528.7094 | | | | 1.06E-05 | -0.55 | 1.33 | 8.50E-01 | 0.04 | 0.77 | 1.08E-03 | -0.53 | 1.45 | 1.57E-03 | -0.58 | 1.50 | 367362 | 39869 | 287149 | 159954 | 284109 | 170146 | 290189 | 150745 |
| RP_POS_475_41525a11_263 | RP | POS | 11.26 | 475.4153 | | | | 3.63E-01 | -0.16 | 0.75 | 8.51E-01 | -0.04 | 1.06 | 3.99E-01 | -0.18 | 0.73 | 4.70E-01 | -0.14 | 0.56 | 4283370 | 935324 | 4096586 | 1191202 | 4119201 | 1089758 | 4073971 | 1295479 |
| RP_POS_328_88492a0_498 | RP | POS | 0.50 | 328.8849 | | | | 7.35E-06 | -0.56 | 1.29 | 8.51E-01 | 0.04 | 1.21 | 9.10E-04 | -0.54 | 1.36 | 1.37E-03 | -0.58 | 1.36 | 1885786 | 203293 | 1448476 | 862393 | 1432108 | 917525 | 1464845 | 812541 |
| HILIC_POS_147_14922a4_831 | HILIC | POS | 4.831 | 147.1492 | | | | 5.57E-01 | -0.11 | 0.86 | 8.51E-01 | -0.04 | 0.72 | 5.42E-01 | -0.13 | 0.37 | 6.69E-01 | -0.09 | 0.17 | 17971886 | 2795752 | 17626639 | 3111204 | 17685669 | 3157997 | 17567609 | 3094618 |
| RP_POS_249_1458a8_4 | RP | POS | 8.40 | 249.1458 | Pterosin A | 249.1438(100) | 3 | 7.41E-01 | 0.06 | 0.60 | 8.51E-01 | -0.04 | 0.35 | 8.32E-01 | 0.05 | 0.40 | 7.13E-01 | 0.08 | 0.48 | 685418 | 201476 | 699286 | 218842 | 703436 | 235458 | 695136 | 203188 |
| RP_POS_263_9006a15_512 | RP | POS | 15.51 | 263.9006 | | | | 4.21E-02 | 0.47 | 1.10 | 8.51E-01 | 0.03 | 0.80 | 5.15E-02 | 0.49 | 1.00 | 6.52E-02 | 0.46 | 0.95 | 1436785 | 336622 | 1573105 | 264695 | 1568097 | 257321 | 1578112 | 274392 |
| RP_POS_436_3403a11_391 | RP | POS | 11.39 | 436.3403 | | | | 2.02E-01 | 0.21 | 0.54 | 8.51E-01 | -0.04 | 0.30 | 2.62E-01 | 0.19 | 0.51 | 2.98E-01 | 0.23 | 0.58 | 1668193 | 547738 | 1832038 | 829002 | 1847705 | 999107 | 1816371 | 624337 |
| RP_POS_135_03207a15_303 | RP | POS | 15.30 | 135.0321 | | | | 1.76E-01 | -0.29 | 0.72 | 8.51E-01 | 0.04 | 0.44 | 2.43E-01 | -0.27 | 0.49 | 1.95E-01 | -0.31 | 0.69 | 17600349 | 5908465 | 15988203 | 5383441 | 15886552 | 5569777 | 16089853 | 5245077 |
| HILIC_NEG_182_8345a2_95 | HILIC | NEG | 2.95 | 182.8345 | | | | 8.21E-07 | -0.63 | 1.34 | 8.52E-01 | 0.04 | 0.77 | 1.91E-04 | -0.61 | 1.47 | 4.29E-04 | -0.65 | 1.50 | 1782082 | 174040 | 1416763 | 633991 | 1404818 | 680523 | 1428709 | 590499 |
| RP_POS_271_22675a9_961 | RP | POS | 9.96 | 271.2268 | | | | 8.03E-02 | -0.37 | 0.78 | 8.52E-01 | -0.04 | 0.74 | 8.89E-02 | -0.39 | 0.70 | 1.36E-01 | -0.36 | 0.83 | 1440339 | 162359 | 1382494 | 150114 | 1385320 | 158633 | 1379667 | 142646 |
| RP_POS_631_401a15_431 | RP | POS | 15.43 | 631.4010 | | | | 7.37E-01 | -0.06 | 0.35 | 8.52E-01 | 0.04 | 0.37 | 8.41E-01 | -0.04 | 0.32 | 7.00E-01 | -0.08 | 0.51 | 5501029 | 682970 | 5451921 | 815232 | 5436576 | 809750 | 5467266 | 828610 |
| HILIC_NEG_226_86159a0_837 | HILIC | NEG | 0.837 | 226.8616 | | | | 3.72E-01 | -0.20 | 0.92 | 8.52E-01 | -0.04 | 0.74 | 3.79E-01 | -0.21 | 0.34 | 4.54E-01 | -0.18 | 0.47 | 152378 | 33652 | 146394 | 29609 | 146952 | 28593 | 145837 | 30872 |
| RP_POS_154_08643a2_879 | RP | POS | 2.88 | 154.0864 | | | | 1.62E-01 | 0.16 | 0.72 | 8.52E-01 | -0.04 | 0.21 | 3.23E-01 | 0.14 | 0.86 | 3.22E-01 | 0.18 | 0.45 | 9318 | 9575 | 2298503 | 16263416 | 2604404 | 18358290 | 1992601 | 14041993 |
| RP_NEG_636_08643a0_624 | RP | NEG | 0.62 | 636.0864 | | | | 2.71E-03 | -0.47 | 1.08 | 8.52E-01 | -0.04 | 1.01 | 7.44E-03 | -0.49 | 1.10 | 2.36E-02 | -0.45 | 1.12 | 2992099 | 753253 | 2417059 | 1307621 | 2441615 | 1398795 | 2392503 | 1223430 |
| RP_POS_801_03644a13_152 | RP | POS | 13.15 | 801.0364 | | | | 3.40E-02 | -0.47 | 0.94 | 8.52E-01 | 0.04 | 0.30 | 6.57E-02 | -0.45 | 0.77 | 3.88E-02 | -0.48 | 0.96 | 1107470 | 446741 | 914735 | 394111 | 907339 | 368638 | 922131 | 421677 |
| RP_POS_563_55054a10_848 | RP | POS | 10.85 | 563.5505 | | | | 1.12E-08 | 0.81 | 1.26 | 8.53E-01 | 0.04 | 0.74 | 5.35E-05 | 0.83 | 1.31 | 4.46E-07 | 0.79 | 1.51 | 133071935 | 70183115 | 259530967 | 164535070 | 256451655 | 131778081 | 262610278 | 193161201 |
| RP_POS_184_13326a5_662 | RP | POS | 5.66 | 184.1333 | | | | 5.37E-01 | -0.15 | 0.63 | 8.53E-01 | -0.03 | 0.24 | 5.51E-01 | -0.16 | 0.61 | 5.80E-01 | -0.13 | 0.50 | 485260 | 680041 | 403804 | 516817 | 413449 | 328438 | 394159 | 656955 |
| RP_POS_149_96095a0_678 | RP | POS | 0.68 | 149.9610 | | | | 3.78E-08 | 0.69 | 1.33 | 8.53E-01 | -0.04 | 1.16 | 9.79E-05 | 0.67 | 1.40 | 5.60E-05 | 0.71 | 1.44 | 1914522 | 217750 | 2533947 | 982197 | 2552236 | 997640 | 2515658 | 976290 |
| RP_POS_223_09398a13_11 | RP | POS | 13.11 | 223.0940 | | | | 3.19E-01 | 0.18 | 0.55 | 8.53E-01 | -0.04 | 0.62 | 4.31E-01 | 0.16 | 0.42 | 3.46E-01 | 0.20 | 0.48 | 9387315 | 991686 | 9601207 | 1209163 | 9623706 | 1253282 | 9578708 | 1175686 |
| HILIC_POS_112_89601a3_017 | HILIC | POS | 3.017 | 112.8960 | | | | 1.54E-04 | -0.55 | 1.34 | 8.54E-01 | 0.04 | 0.78 | 2.72E-03 | -0.53 | 1.40 | 2.79E-03 | -0.57 | 1.38 | 41962648 | 8446995 | 32818481 | 17939650 | 32484893 | 18904843 | 33152070 | 17105844 |
| RP_NEG_203_88588a14_274 | RP | NEG | 14.27 | 203.8859 | | | | 2.28E-01 | -0.24 | 0.62 | 8.54E-01 | 0.04 | 0.24 | 3.33E-01 | -0.22 | 0.38 | 2.37E-01 | -0.26 | 0.62 | 883402 | 457233 | 768429 | 486418 | 759388 | 461266 | 777470 | 514879 |
| HILIC_POS_544_73077a2_992 | HILIC | POS | 2.992 | 544.7308 | | | | 2.55E-05 | -0.56 | 1.35 | 8.54E-01 | 0.04 | 0.76 | 1.41E-03 | -0.54 | 1.46 | 1.71E-03 | -0.58 | 1.47 | 3936308 | 603810 | 3027309 | 1786522 | 2994129 | 1887889 | 3060489 | 1697670 |
| RP_POS_289_15405a9_356 | RP | POS | 9.36 | 289.1541 | | | | 4.88E-01 | 0.13 | 0.49 | 8.54E-01 | 0.04 | 0.35 | 4.86E-01 | 0.15 | 0.42 | 6.08E-01 | 0.11 | 0.50 | 476792 | 71814 | 487495 | 86053 | 485897 | 86883 | 489092 | 86067 |
| RP_POS_265_25104a10_848 | RP | POS | 10.85 | 265.2510 | | | | 5.22E-06 | 0.80 | 1.26 | 8.54E-01 | -0.04 | 0.65 | 2.82E-04 | 0.79 | 1.26 | 1.25E-05 | 0.82 | 1.47 | 11241735 | 3645938 | 15256530 | 4981813 | 15348998 | 4194342 | 15164061 | 5703811 |
| RP_POS_165_06042a15_299 | RP | POS | 15.30 | 165.0604 | | | | 5.65E-02 | -0.33 | 0.56 | 8.54E-01 | -0.04 | 0.18 | 8.65E-02 | -0.35 | 0.62 | 1.24E-01 | -0.31 | 0.54 | 29026576 | 6154170 | 26353150 | 8480436 | 26510364 | 8454510 | 26195935 | 8589187 |
| HILIC_POS_336_69052a3_023 | HILIC | POS | 3.023 | 336.6905 | | | | 5.46E-04 | -0.50 | 1.31 | 8.54E-01 | -0.04 | 0.76 | 3.25E-03 | -0.52 | 1.41 | 1.22E-02 | -0.48 | 1.40 | 731590 | 158506 | 572149 | 349904 | 578636 | 370984 | 565663 | 331133 |
| HILIC_POS_421_36954a7_689 | HILIC | POS | 7.689 | 421.3695 | | | | 3.72E-01 | 0.18 | 0.49 | 8.54E-01 | 0.04 | 0.38 | 3.93E-01 | 0.19 | 0.31 | 4.68E-01 | 0.16 | 0.38 | 1006511 | 83260 | 1022070 | 90527 | 1020392 | 84978 | 1023748 | 96594 |
| RP_POS_576_84497a0_472 | RP | POS | 0.47 | 576.8450 | | | | 5.40E-08 | -0.68 | 1.35 | 8.54E-01 | 0.04 | 1.17 | 7.60E-05 | -0.66 | 1.45 | 1.01E-04 | -0.70 | 1.43 | 692652 | 65270 | 509149 | 294837 | 503685 | 308472 | 514613 | 283577 |
| HILIC_NEG_273_01746a0_579 | HILIC | NEG | 0.579 | 273.0175 | | | | 9.52E-01 | 0.01 | 0.60 | 8.54E-01 | 0.04 | 0.58 | 8.83E-01 | 0.03 | 0.18 | 9.71E-01 | -0.01 | 0.11 | 559046 | 126412 | 560687 | 151893 | 557875 | 161953 | 563499 | 142720 |
| RP_POS_441_17322a6_81 | RP | POS | 6.81 | 441.1732 | | | | 6.54E-02 | -0.34 | 0.75 | 8.54E-01 | 0.04 | 0.55 | 1.32E-01 | -0.32 | 0.61 | 8.82E-02 | -0.36 | 0.69 | 1062427 | 78782 | 1030633 | 96763 | 1028843 | 95891 | 1032422 | 98567 |
| RP_POS_512_33459a8_913 | RP | POS | 8.91 | 512.3346 | | | | 6.64E-04 | -0.54 | 1.33 | 8.55E-01 | 0.04 | 0.60 | 1.90E-02 | -0.53 | 1.27 | 3.50E-04 | -0.56 | 1.34 | 7454333 | 2152298 | 5608216 | 3591450 | 5541899 | 2392433 | 5674533 | 4508609 |
| RP_POS_614_80927a0_499 | RP | POS | 0.50 | 614.8093 | | | | 1.97E-04 | -0.49 | 1.23 | 8.55E-01 | -0.04 | 1.22 | 2.28E-03 | -0.51 | 1.30 | 1.09E-02 | -0.47 | 1.28 | 6164065 | 988288 | 4815637 | 3054661 | 4871953 | 3254869 | 4759321 | 2872575 |
| RP_POS_409_18735a2_489 | RP | POS | 2.49 | 409.1874 | | | | 2.09E-03 | -0.64 | 1.23 | 8.55E-01 | 0.04 | 1.10 | 8.74E-03 | -0.62 | 1.14 | 2.69E-03 | -0.65 | 1.28 | 917168 | 404746 | 651205 | 403323 | 643780 | 353343 | 658630 | 451326 |
| RP_NEG_732_86047a0_491 | RP | NEG | 0.49 | 732.8605 | | | | 1.41E-07 | 0.63 | 1.35 | 8.55E-01 | -0.04 | 1.21 | 2.00E-04 | 0.61 | 1.44 | 2.02E-04 | 0.65 | 1.43 | 781470 | 235714 | 2055649 | 2218903 | 2096430 | 2301416 | 2014868 | 2155837 |
| RP_POS_415_28177a9_935 | RP | POS | 9.94 | 415.2818 | | | | 7.80E-02 | 0.28 | 1.09 | 8.55E-01 | -0.04 | 0.36 | 1.47E-01 | 0.26 | 0.64 | 1.51E-01 | 0.30 | 1.22 | 2846335 | 2434954 | 3924154 | 4247772 | 4002222 | 4736837 | 3846086 | 3742367 |
| RP_NEG_462_88031a0_465 | RP | NEG | 0.47 | 462.8803 | | | | 1.30E-07 | -0.67 | 1.35 | 8.55E-01 | 0.04 | 1.20 | 1.20E-04 | -0.65 | 1.43 | 1.16E-04 | -0.69 | 1.43 | 790046 | 95388 | 565336 | 364668 | 558640 | 377630 | 572032 | 354941 |
| RP_POS_408_36716a10_881 | RP | POS | 10.88 | 408.3672 | | | | 1.63E-05 | -0.69 | 1.07 | 8.55E-01 | -0.04 | 0.57 | 1.62E-04 | -0.71 | 1.28 | 6.85E-04 | -0.67 | 1.09 | 4438033 | 420510 | 3970082 | 708358 | 3983088 | 742788 | 3957077 | 679488 |
| RP_POS_279_03922a6_074 | RP | POS | 6.07 | 279.0392 | | | | 7.04E-01 | 0.07 | 0.43 | 8.56E-01 | 0.04 | 0.55 | 6.65E-01 | 0.09 | 0.42 | 8.12E-01 | 0.05 | 0.61 | 622619 | 155087 | 635093 | 178772 | 631814 | 191067 | 638372 | 167459 |
| HILIC_NEG_632_72791a2_91 | HILIC | NEG | 2.91 | 632.7279 | | | | 5.25E-07 | -0.63 | 1.36 | 8.56E-01 | 0.04 | 0.78 | 2.19E-04 | -0.60 | 1.52 | 3.77E-04 | -0.65 | 1.54 | 1712263 | 157048 | 1306159 | 714393 | 1293062 | 757628 | 1319256 | 675855 |
| RP_NEG_172_86778a15_568 | RP | NEG | 15.57 | 172.8678 | | | | 1.96E-02 | -0.43 | 0.77 | 8.56E-01 | -0.04 | 0.44 | 2.70E-02 | -0.45 | 0.80 | 6.00E-02 | -0.41 | 0.88 | 1066936 | 74237 | 1028663 | 91253 | 1030332 | 98910 | 1026995 | 83877 |
| HILIC_NEG_134_89478a3_013 | HILIC | NEG | 3.013 | 134.8948 | | | | 3.85E-01 | 0.16 | 0.96 | 8.56E-01 | -0.04 | 0.80 | 5.05E-01 | 0.14 | 0.25 | 3.98E-01 | 0.18 | 0.33 | 241512667 | 38664308 | 248752196 | 46652711 | 249604657 | 46826528 | 247899735 | 46937852 |
| RP_NEG_296_99902a6_797 | RP | NEG | 6.80 | 296.9990 | | | | 5.47E-01 | 0.18 | 0.89 | 8.56E-01 | -0.03 | 0.58 | 5.86E-01 | 0.17 | 0.78 | 5.30E-01 | 0.19 | 0.57 | 5753301 | 695643 | 5830264 | 298902 | 5835723 | 299824 | 5824804 | 300919 |
| RP_NEG_384_93628a14_265 | RP | NEG | 14.27 | 384.9363 | | | | 5.07E-01 | -0.15 | 0.48 | 8.56E-01 | -0.04 | 0.21 | 4.84E-01 | -0.16 | 0.48 | 6.05E-01 | -0.13 | 0.42 | 3226751 | 1801899 | 2988814 | 1589035 | 3017837 | 1729053 | 2959791 | 1452670 |
| RP_NEG_329_03979a0_621 | RP | NEG | 0.62 | 329.0398 | | | | 2.21E-07 | -0.66 | 1.33 | 8.56E-01 | -0.04 | 1.22 | 4.67E-05 | -0.68 | 1.46 | 4.68E-04 | -0.64 | 1.38 | 6306349 | 757853 | 4500039 | 3011107 | 4555003 | 3199504 | 4445076 | 2841756 |
| RP_POS_617_33704a14_267 | RP | POS | 14.27 | 617.3370 | | | | 6.63E-01 | -0.09 | 0.62 | 8.56E-01 | 0.04 | 0.20 | 7.60E-01 | -0.07 | 0.57 | 6.33E-01 | -0.11 | 0.31 | 9294091 | 2187064 | 9099236 | 2195602 | 9059191 | 2122555 | 9139282 | 2287183 |
| HILIC_POS_116_08198a2_131 | HILIC | POS | 2.131 | 116.0820 | | | | 2.74E-01 | -0.28 | 0.54 | 8.57E-01 | 0.03 | 0.47 | 3.39E-01 | -0.27 | 0.39 | 2.58E-01 | -0.30 | 0.48 | 225424 | 313917 | 160092 | 195669 | 156529 | 162987 | 163655 | 225306 |
| RP_POS_304_26071a11_608 | RP | POS | 11.61 | 304.2607 | | | | 3.75E-06 | 0.75 | 1.20 | 8.57E-01 | 0.04 | 0.48 | 2.84E-04 | 0.77 | 1.21 | 2.58E-05 | 0.73 | 1.47 | 11480856 | 3971554 | 16197236 | 6464308 | 16079627 | 5309382 | 16314845 | 7497306 |
| HILIC_NEG_480_56104a2_946 | HILIC | NEG | 2.946 | 480.5610 | | | | 1.10E-04 | -0.54 | 1.30 | 8.57E-01 | -0.04 | 0.76 | 9.49E-04 | -0.56 | 1.44 | 6.21E-03 | -0.52 | 1.39 | 252709 | 46312 | 197914 | 109875 | 199911 | 118894 | 195917 | 101230 |
| RP_POS_296_25821a9_495 | RP | POS | 9.50 | 296.2582 | | | | 3.37E-01 | -0.28 | 0.86 | 8.57E-01 | 0.03 | 0.45 | 3.85E-01 | -0.27 | 0.66 | 3.17E-01 | -0.30 | 0.74 | 3971033 | 9103691 | 2356831 | 4035616 | 2283491 | 2930695 | 2430172 | 4929992 |
| RP_NEG_197_04224a3_698 | RP | NEG | 3.70 | 197.0422 | | | | 2.98E-01 | 0.19 | 0.67 | 8.57E-01 | 0.04 | 0.80 | 3.48E-01 | 0.21 | 0.55 | 3.85E-01 | 0.17 | 0.37 | 1286983 | 392758 | 1375503 | 480282 | 1366800 | 419075 | 1384206 | 538767 |
| RP_POS_553_45856a13_013 | RP | POS | 13.01 | 553.4586 | | | | 4.83E-02 | -0.43 | 0.98 | 8.58E-01 | 0.03 | 0.16 | 9.03E-02 | -0.41 | 0.99 | 5.38E-02 | -0.44 | 0.77 | 6736800 | 1195390 | 6255625 | 1084234 | 6236053 | 1001599 | 6275197 | 1170954 |

**Supplementary table 1: Metabolomics results** (in asceding order according to p-value from progressive ALD vs  non-progressive ALD comparison, with molecular features with ID_level of 1-2 first)

| | | | | | | | | all_ALD_vs_control | | | Prog_ALD_vs_non-prog_ALD | | | Prog_ALD_vs_control_ALD | | | Non-prog_ALD_vs_control | | | Healthy_control | | all_ALD | | Non-prog_ALD | | Progressive_ALD | |
|---|---|---|---|---|---|---|---|---|---|---|---|---|---|---|---|---|---|---|---|---|---|---|---|---|---|---|---|
| | | | | | | | | t-test | Cohen's | PLS-DA | t-test | Cohen's | PLS-DA | t-test | Cohen's | PLS-DA | t-test | Cohen's | PLS-DA | | | | | | | | |
| Feature_ID | RP_HILIC | POS_NEG | Average.Rt | Average.Mz | Metabolite.name | curated_MSMS | ID_level | p | d | VIP | p | d | VIP | p | d | VIP | p | d | VIP | Mean | SD | Mean | SD | Mean | SD | Mean | SD |
| HILIC_NEG_384_83936a2_911 | HILIC | NEG | 2.911 | 384.8394 | | | | 1.62E-06 | -0.59 | 1.34 | 8.58E-01 | 0.04 | 0.77 | 4.28E-04 | -0.57 | 1.48 | 7.39E-04 | -0.61 | 1.54 | 305350 | 24517 | 238364 | 125396 | 236110 | 133337 | 240619 | 118237 |
| RP_POS_250_89377a0_257 | RP | POS | 0.26 | 250.8938 | | | | 5.80E-01 | 0.11 | 0.58 | 8.58E-01 | 0.04 | 0.34 | 5.72E-01 | 0.13 | 0.47 | 6.79E-01 | 0.09 | 0.58 | 2895412 | 1208738 | 3035009 | 1311301 | 3011442 | 1272924 | 3058575 | 1361119 |
| RP_POS_502_29239a10_324 | RP | POS | 10.32 | 502.2924 | | | | 7.83E-10 | 1.07 | 1.80 | 8.58E-01 | -0.03 | 0.23 | 4.61E-07 | 1.05 | 1.68 | 6.83E-09 | 1.09 | 1.91 | 32604540 | 8682680 | 46484972 | 12334014 | 46706640 | 10749378 | 46263304 | 13845975 |
| HILIC_NEG_180_97305a4_232 | HILIC | NEG | 4.232 | 180.9731 | | | | 2.49E-01 | 0.20 | 1.02 | 8.59E-01 | -0.04 | 0.85 | 3.74E-01 | 0.18 | 0.53 | 2.93E-01 | 0.21 | 0.85 | 39505128 | 7478103 | 41499345 | 10929325 | 41695406 | 11249818 | 41303285 | 10709864 |
| RP_POS_124_92249a0_498 | RP | POS | 0.50 | 124.9225 | | | | 7.00E-05 | -0.52 | 1.25 | 8.59E-01 | -0.04 | 1.22 | 1.11E-03 | -0.54 | 1.32 | 6.40E-03 | -0.50 | 1.30 | 1485627 | 239897 | 1138297 | 731293 | 1151394 | 781998 | 1125200 | 684560 |
| RP_NEG_1043_15271a9_73 | RP | NEG | 9.73 | 1043.1527 | | | | 4.14E-01 | -0.17 | 0.43 | 8.59E-01 | -0.04 | 1.06 | 4.03E-01 | -0.19 | 0.48 | 5.22E-01 | -0.15 | 0.34 | 3957047 | 307694 | 3905722 | 302990 | 3911134 | 326727 | 3900310 | 280481 |
| RP_POS_184_07294a10_618 | RP | POS | 10.62 | 184.0729 | | | | 1.02E-01 | 0.34 | 1.09 | 8.59E-01 | -0.04 | 0.90 | 1.90E-01 | 0.32 | 0.86 | 9.61E-02 | 0.35 | 0.99 | 615375 | 244970 | 698360 | 245866 | 702747 | 198792 | 693973 | 287362 |
| HILIC_POS_158_96407a4_322 | HILIC | POS | 4.322 | 158.9641 | | | | 2.06E-01 | 0.21 | 1.02 | 8.60E-01 | -0.04 | 0.84 | 3.24E-01 | 0.19 | 0.55 | 2.56E-01 | 0.23 | 0.87 | 102545558 | 19500429 | 108282861 | 28914325 | 108797715 | 29958753 | 107768008 | 28125975 |
| RP_NEG_484_87112a0_498 | RP | NEG | 0.50 | 484.8711 | | | | 1.53E-06 | -0.60 | 1.31 | 8.60E-01 | 0.04 | 1.21 | 3.93E-04 | -0.58 | 1.39 | 6.96E-04 | -0.62 | 1.38 | 14054298 | 1305605 | 10678287 | 6262137 | 10566829 | 6662379 | 10789745 | 5900539 |
| HILIC_POS_90_97725a4_322 | HILIC | POS | 4.322 | 90.9773 | | | | 2.49E-01 | 0.20 | 1.01 | 8.60E-01 | -0.04 | 0.84 | 3.71E-01 | 0.18 | 0.50 | 2.93E-01 | 0.22 | 0.81 | 265878856 | 50022827 | 279125642 | 71837018 | 280401710 | 74074487 | 277849574 | 70257234 |
| HILIC_NEG_193_81575a3_034 | HILIC | NEG | 3.034 | 193.8158 | | | | 5.52E-05 | 0.50 | 1.20 | 8.60E-01 | 0.04 | 0.77 | 1.48E-03 | 0.52 | 1.43 | 7.78E-03 | 0.48 | 1.25 | 6251790 | 252621 | 6800273 | 1233148 | 6778369 | 1309886 | 6822177 | 1164285 |
| RP_NEG_701_15546a9_222 | RP | NEG | 9.22 | 701.1555 | | | | 4.39E-01 | 0.13 | 0.71 | 8.60E-01 | -0.04 | 0.52 | 5.73E-01 | 0.11 | 0.39 | 4.54E-01 | 0.15 | 0.57 | 12372954 | 678587 | 12491741 | 946716 | 12508537 | 950247 | 12474945 | 952518 |
| HILIC_POS_448_36249a1_071 | HILIC | POS | 1.071 | 448.3625 | | | | 4.39E-02 | -0.33 | 1.15 | 8.60E-01 | -0.04 | 0.66 | 7.95E-02 | -0.35 | 1.22 | 1.05E-01 | -0.32 | 1.06 | 390753 | 121969 | 332456 | 186086 | 335757 | 181754 | 329156 | 192108 |
| RP_POS_210_10957a11_609 | RP | POS | 11.61 | 210.1096 | | | | 7.51E-02 | -0.34 | 0.79 | 8.60E-01 | 0.04 | 0.43 | 1.12E-01 | -0.32 | 0.67 | 1.22E-01 | -0.36 | 0.73 | 1594943 | 164046 | 1532038 | 191191 | 1528648 | 218208 | 1535427 | 161913 |
| RP_POS_403_78049a13_394 | RP | POS | 13.39 | 403.7805 | | | | 3.78E-01 | 0.21 | 0.60 | 8.60E-01 | 0.03 | 0.21 | 3.66E-01 | 0.22 | 0.60 | 4.59E-01 | 0.19 | 0.65 | 2038442 | 965030 | 2204339 | 745229 | 2191134 | 803613 | 2217544 | 689868 |
| RP_NEG_362_9415a0_465 | RP | NEG | 0.47 | 362.9415 | | | | 5.70E-07 | -0.72 | 1.33 | 8.61E-01 | -0.04 | 1.10 | 4.57E-05 | -0.74 | 1.42 | 1.53E-04 | -0.70 | 1.37 | 1221994 | 183382 | 947353 | 404541 | 954509 | 414208 | 940198 | 398715 |
| RP_POS_589_427a10_875 | RP | POS | 10.88 | 589.4270 | | | | 9.09E-01 | 0.02 | 0.64 | 8.61E-01 | 0.04 | 0.49 | 8.53E-01 | 0.04 | 0.37 | 9.83E-01 | 0.00 | 0.79 | 16406138 | 1011714 | 16430221 | 1078373 | 16411166 | 1116554 | 16449275 | 1049796 |
| RP_POS_248_87036a0_515 | RP | POS | 0.52 | 248.8704 | | | | 7.51E-06 | -0.66 | 1.23 | 8.61E-01 | -0.04 | 1.05 | 1.55E-04 | -0.68 | 1.32 | 7.32E-04 | -0.64 | 1.27 | 1062744 | 245632 | 751012 | 500183 | 759833 | 524698 | 742192 | 479588 |
| RP_POS_313_27362a10_25 | RP | POS | 10.25 | 313.2736 | | | | 9.82E-01 | 0.00 | 0.45 | 8.61E-01 | 0.03 | 0.20 | 9.58E-01 | 0.01 | 0.53 | 9.24E-01 | -0.02 | 0.52 | 5688088 | 985980 | 5683621 | 917172 | 5667454 | 904609 | 5699788 | 938470 |
| RP_POS_255_97752a0_51 | RP | POS | 0.51 | 255.9775 | | | | 1.09E-05 | 0.55 | 1.20 | 8.62E-01 | 0.04 | 1.15 | 1.12E-03 | 0.57 | 1.30 | 2.01E-03 | 0.53 | 1.27 | 236022 | 86342 | 430960 | 396449 | 423991 | 395682 | 437929 | 401109 |
| RP_POS_240_23146a10_846 | RP | POS | 10.85 | 240.2315 | | | | 4.89E-04 | 0.58 | 0.92 | 8.62E-01 | -0.04 | 0.49 | 3.34E-03 | 0.56 | 1.04 | 3.23E-03 | 0.60 | 0.93 | 317940 | 171882 | 464161 | 263825 | 468796 | 277945 | 459526 | 251655 |
| RP_NEG_326_98917a7_342 | RP | NEG | 7.34 | 326.9892 | | | | 6.65E-01 | -0.08 | 0.29 | 8.62E-01 | -0.04 | 0.21 | 6.40E-01 | -0.10 | 0.39 | 7.69E-01 | -0.06 | 0.37 | 5461895 | 1420191 | 5332168 | 1610959 | 5360469 | 1656117 | 5303866 | 1580818 |
| HILIC_POS_196_85735a3_011 | HILIC | POS | 3.011 | 196.8574 | | | | 6.54E-05 | -0.58 | 1.34 | 8.62E-01 | 0.04 | 0.77 | 1.62E-03 | -0.56 | 1.42 | 1.61E-03 | -0.60 | 1.40 | 1225094 | 255781 | 930455 | 547967 | 920832 | 573546 | 940077 | 526801 |
| RP_NEG_271_22867a10_283 | RP | NEG | 10.28 | 271.2287 | | | | 5.15E-01 | -0.12 | 0.59 | 8.62E-01 | 0.04 | 0.53 | 6.30E-01 | -0.10 | 0.46 | 5.29E-01 | -0.13 | 0.72 | 1822764 | 172869 | 1797850 | 227741 | 1793851 | 240274 | 1801848 | 216855 |
| HILIC_POS_354_75095a3_014 | HILIC | POS | 3.014 | 354.7510 | | | | 7.81E-05 | -0.54 | 1.35 | 8.62E-01 | 0.04 | 0.79 | 2.57E-03 | -0.52 | 1.46 | 2.68E-03 | -0.56 | 1.46 | 375830 | 65297 | 290325 | 174720 | 287260 | 183374 | 293390 | 167427 |
| HILIC_POS_375_3212a1_393 | HILIC | POS | 1.393 | 375.3212 | | | | 1.55E-04 | -0.63 | 1.12 | 8.62E-01 | -0.04 | 0.35 | 1.19E-03 | -0.65 | 1.10 | 1.53E-03 | -0.62 | 1.37 | 8542574 | 1545837 | 7114083 | 2335970 | 7155047 | 2281733 | 7073119 | 2411461 |
| HILIC_NEG_128_95969a3_046 | HILIC | NEG | 3.046 | 128.9597 | | | | 7.35E-04 | -0.54 | 1.27 | 8.62E-01 | 0.04 | 0.76 | 5.96E-03 | -0.52 | 1.28 | 4.87E-03 | -0.55 | 1.32 | 4145025 | 1106672 | 3195085 | 1887040 | 3162058 | 1959849 | 3228113 | 1830650 |
| RP_POS_307_22415a9_865 | RP | POS | 9.87 | 307.2242 | | | | 2.40E-01 | -0.21 | 0.74 | 8.62E-01 | 0.04 | 0.73 | 3.42E-01 | -0.19 | 0.56 | 2.84E-01 | -0.23 | 0.89 | 4342464 | 487574 | 4216076 | 627258 | 4205104 | 661564 | 4227049 | 597513 |
| HILIC_POS_145_09697a5_188 | HILIC | POS | 5.188 | 145.0970 | | | | 1.49E-06 | -0.73 | 1.37 | 8.62E-01 | 0.04 | 0.75 | 1.01E-04 | -0.71 | 1.49 | 1.01E-04 | -0.75 | 1.41 | 738249 | 156333 | 529237 | 301484 | 523966 | 313332 | 534509 | 292244 |
| RP_POS_472_59756a6_667 | RP | POS | 6.67 | 472.5976 | | | | 3.37E-07 | 0.61 | 1.31 | 8.62E-01 | -0.04 | 1.11 | 4.42E-04 | 0.59 | 1.40 | 2.65E-04 | 0.63 | 1.38 | 15753 | 29147 | 860567 | 1543411 | 887541 | 1567415 | 833593 | 1534463 |
| RP_POS_185_03215a0_52 | RP | POS | 0.52 | 185.0322 | | | | 1.58E-05 | -0.68 | 1.23 | 8.63E-01 | -0.04 | 1.07 | 4.05E-04 | -0.70 | 1.25 | 3.14E-04 | -0.67 | 1.31 | 1579824 | 368927 | 1165022 | 634454 | 1176094 | 601354 | 1153951 | 671864 |
| HILIC_POS_131_08162a5_648 | HILIC | POS | 5.648 | 131.0816 | | | | 4.79E-01 | -0.14 | 0.98 | 8.63E-01 | -0.04 | 0.62 | 4.98E-01 | -0.16 | 0.80 | 5.69E-01 | -0.12 | 0.92 | 456107 | 166717 | 431387 | 182957 | 434578 | 165018 | 428196 | 200942 |
| RP_POS_230_22221a5_932 | RP | POS | 5.93 | 230.2222 | | | | 2.92E-01 | -0.18 | 1.01 | 8.63E-01 | -0.04 | 1.01 | 2.95E-01 | -0.20 | 0.86 | 4.53E-01 | -0.16 | 1.03 | 663575 | 189926 | 617470 | 275814 | 622267 | 305662 | 612674 | 245404 |
| HILIC_POS_58_06571a4_831 | HILIC | POS | 4.831 | 58.0657 | | | | 1.81E-02 | 0.41 | 0.98 | 8.63E-01 | -0.04 | 0.58 | 7.33E-02 | 0.39 | 0.64 | 2.23E-02 | 0.43 | 0.84 | 2646557 | 645146 | 2999238 | 908621 | 3015029 | 771375 | 2983447 | 1035618 |
| RP_POS_335_24301a10_978 | RP | POS | 10.98 | 335.2430 | | | | 5.49E-02 | 0.36 | 1.41 | 8.63E-01 | -0.04 | 1.21 | 1.26E-01 | 0.34 | 1.21 | 6.52E-02 | 0.38 | 1.22 | 1431330 | 356941 | 1580821 | 431207 | 1588309 | 390099 | 1573333 | 472633 |
| RP_POS_126_0916a2_88 | RP | POS | 2.88 | 126.0916 | | | | 1.59E-01 | 0.16 | 0.72 | 8.63E-01 | -0.04 | 0.21 | 3.19E-01 | 0.14 | 0.86 | 3.20E-01 | 0.18 | 0.45 | 45925 | 51405 | 5107331 | 35681283 | 5726920 | 39975274 | 4487742 | 31200715 |
| HILIC_NEG_169_96764a0_93 | HILIC | NEG | 0.93 | 169.9676 | | | | 1.89E-08 | 0.79 | 1.31 | 8.64E-01 | -0.04 | 1.23 | 5.99E-05 | 0.77 | 1.29 | 1.74E-06 | 0.81 | 1.53 | 351542 | 201096 | 708560 | 473855 | 716743 | 429132 | 700377 | 519007 |
| RP_POS_311_00858a3_622 | RP | POS | 3.62 | 311.0086 | | | | 2.29E-02 | -0.49 | 0.87 | 8.64E-01 | 0.03 | 0.73 | 5.23E-02 | -0.48 | 0.78 | 2.44E-02 | -0.51 | 1.06 | 959636 | 838668 | 568187 | 758205 | 555097 | 653358 | 581276 | 856888 |
| RP_NEG_167_02037a0_982 | RP | NEG | 0.98 | 167.0204 | | | | 2.23E-02 | 0.39 | 0.94 | 8.64E-01 | -0.04 | 0.28 | 7.92E-02 | 0.37 | 0.97 | 3.41E-02 | 0.40 | 0.77 | 15386854 | 3917253 | 17493937 | 5806281 | 17594144 | 5327060 | 17393730 | 6302044 |
| RP_NEG_297_73343a15_599 | RP | NEG | 15.60 | 297.7334 | | | | 3.65E-02 | -0.45 | 0.89 | 8.65E-01 | 0.03 | 0.36 | 6.73E-02 | -0.43 | 0.74 | 4.53E-02 | -0.47 | 0.88 | 2516882 | 125435 | 2463230 | 114308 | 2461267 | 111542 | 2465193 | 118110 |
| HILIC_POS_284_81412a3_009 | HILIC | POS | 3.009 | 284.8141 | | | | 2.15E-05 | -0.58 | 1.35 | 8.65E-01 | 0.04 | 0.77 | 1.04E-03 | -0.56 | 1.46 | 1.33E-03 | -0.60 | 1.46 | 733211 | 124870 | 558424 | 328746 | 552793 | 347099 | 564055 | 312748 |
| RP_POS_309_23941a10_744 | RP | POS | 10.74 | 309.2394 | | | | 4.24E-04 | -0.58 | 1.14 | 8.65E-01 | -0.04 | 1.00 | 1.15E-03 | -0.60 | 1.24 | 7.02E-03 | -0.56 | 1.14 | 1486665 | 166446 | 1341651 | 263612 | 1346163 | 292448 | 1337140 | 234165 |
| RP_POS_285_24237a9_854 | RP | POS | 9.85 | 285.2424 | | | | 8.54E-01 | -0.04 | 0.68 | 8.65E-01 | -0.04 | 0.71 | 8.05E-01 | -0.05 | 0.43 | 9.36E-01 | -0.02 | 0.82 | 2413672 | 274754 | 2403059 | 306233 | 2408290 | 320246 | 2397828 | 294717 |
| RP_POS_682_79657a0_499 | RP | POS | 0.50 | 682.7966 | | | | 1.63E-04 | -0.48 | 1.24 | 8.65E-01 | -0.04 | 1.21 | 2.43E-03 | -0.50 | 1.31 | 1.04E-02 | -0.47 | 1.29 | 6058505 | 856545 | 4763239 | 2971416 | 4813975 | 3148975 | 4712503 | 2813809 |
| RP_POS_752_78241a0_498 | RP | POS | 0.50 | 752.7824 | | | | 9.79E-05 | -0.50 | 1.25 | 8.66E-01 | -0.04 | 1.22 | 1.89E-03 | -0.52 | 1.32 | 7.12E-03 | -0.49 | 1.30 | 1375552 | 185523 | 1096452 | 611839 | 1106892 | 641494 | 1086012 | 587023 |
| RP_POS_293_12561a9_356 | RP | POS | 9.36 | 293.1256 | | | | 3.43E-05 | 0.61 | 1.22 | 8.66E-01 | 0.04 | 0.72 | 1.22E-03 | 0.63 | 1.16 | 7.88E-04 | 0.59 | 1.17 | 514386 | 142550 | 682611 | 296898 | 677561 | 277844 | 687661 | 317563 |
| RP_POS_126_09157a3_143 | RP | POS | 3.14 | 126.0916 | | | | 1.62E-01 | 0.16 | 0.72 | 8.66E-01 | -0.04 | 0.21 | 3.22E-01 | 0.14 | 0.86 | 3.23E-01 | 0.18 | 0.45 | 13910 | 26342 | 6578748 | 46562561 | 7370733 | 52074759 | 5786763 | 40832952 |
| RP_NEG_451_29416a11_612 | RP | NEG | 11.61 | 451.2942 | | | | 5.73E-02 | -0.34 | 0.99 | 8.66E-01 | -0.04 | 0.89 | 6.72E-02 | -0.36 | 0.91 | 1.42E-01 | -0.32 | 0.92 | 841841 | 127061 | 787643 | 167649 | 790494 | 186700 | 784791 | 148030 |
| RP_POS_325_21362a10_704 | RP | POS | 10.70 | 325.2136 | | | | 1.46E-07 | 0.75 | 1.63 | 8.66E-01 | 0.04 | 0.51 | 3.83E-05 | 0.77 | 1.51 | 4.40E-05 | 0.73 | 1.57 | 2464004 | 1325131 | 4608881 | 3036177 | 4557261 | 2975213 | 4660501 | 3125307 |
| HILIC_NEG_159_06624a0_576 | HILIC | NEG | 0.576 | 159.0662 | | | | 8.96E-01 | -0.03 | 0.84 | 8.66E-01 | 0.03 | 0.76 | 9.71E-01 | -0.01 | 0.09 | 8.48E-01 | -0.04 | 0.40 | 252316 | 55696 | 250794 | 61248 | 249754 | 62961 | 251835 | 60107 |
| RP_POS_263_87595a0_242 | RP | POS | 0.24 | 263.8760 | | | | 8.29E-02 | 0.27 | 0.79 | 8.67E-01 | -0.04 | 0.14 | 1.64E-01 | 0.25 | 0.95 | 1.49E-01 | 0.29 | 0.58 | 835327 | 69145 | 865453 | 120791 | 867497 | 130081 | 863408 | 112023 |
| RP_POS_154_95532a15_566 | RP | POS | 15.57 | 154.9553 | | | | 5.04E-01 | -0.13 | 0.60 | 8.67E-01 | 0.03 | 0.51 | 6.06E-01 | -0.11 | 0.33 | 5.07E-01 | -0.15 | 0.61 | 29786675 | 1680985 | 29550495 | 1868685 | 29518893 | 1911896 | 29582097 | 1843325 |
| RP_NEG_551_12665a8_312 | RP | NEG | 8.31 | 551.1267 | | | | 6.82E-01 | -0.08 | 0.36 | 8.67E-01 | -0.03 | 0.50 | 6.53E-01 | -0.10 | 0.30 | 7.83E-01 | -0.06 | 0.30 | 899521 | 51531 | 895071 | 57948 | 896050 | 60891 | 894093 | 55451 |
| RP_NEG_297_1543a15_107 | RP | NEG | 15.11 | 297.1543 | | | | 2.76E-01 | 0.20 | 0.34 | 8.67E-01 | 0.04 | 0.13 | 3.24E-01 | 0.22 | 0.39 | 3.62E-01 | 0.18 | 0.51 | 3203513 | 2604993 | 3817274 | 3152499 | 3764093 | 2839722 | 3870455 | 3465477 |
| RP_POS_308_12863a8_874 | RP | POS | 8.87 | 308.1286 | | | | 8.06E-01 | 0.05 | 0.44 | 8.67E-01 | -0.03 | 0.20 | 8.93E-01 | 0.03 | 0.31 | 7.60E-01 | 0.07 | 0.23 | 2505325 | 399943 | 2525739 | 433351 | 2533045 | 398551 | 2518432 | 469540 |
| RP_POS_322_24994a11_166 | RP | POS | 11.17 | 322.2499 | | | | 4.20E-09 | 1.04 | 1.61 | 8.67E-01 | -0.03 | 0.53 | 9.25E-07 | 1.02 | 1.61 | 4.12E-08 | 1.05 | 1.77 | 1072909 | 366008 | 1621361 | 503989 | 1629847 | 455248 | 1612875 | 552988 |
| RP_POS_309_91843a15_439 | RP | POS | 15.44 | 309.9184 | | | | 9.12E-01 | 0.03 | 1.10 | 8.68E-01 | -0.03 | 0.42 | 9.65E-01 | 0.01 | 0.75 | 8.72E-01 | 0.04 | 0.72 | 3244998 | 712882 | 3260062 | 515378 | 3268719 | 528688 | 3251404 | 506937 |
| RP_POS_152_96909a15_42 | RP | POS | 15.42 | 152.9691 | | | | 8.94E-01 | -0.03 | 0.36 | 8.68E-01 | -0.03 | 0.48 | 8.40E-01 | -0.04 | 0.16 | 9.67E-01 | -0.01 | 0.25 | 5900970 | 2442058 | 5833732 | 2578067 | 5876914 | 2800230 | 5790551 | 2362924 |
| RP_NEG_529_46448a15_123 | RP | NEG | 15.12 | 529.4645 | | | | 1.47E-01 | -0.24 | 0.51 | 8.68E-01 | -0.04 | 0.35 | 2.13E-01 | -0.26 | 0.46 | 2.36E-01 | -0.22 | 0.46 | 16323246 | 2647841 | 15422813 | 3991547 | 15489647 | 3660041 | 15355978 | 4334170 |
| RP_POS_349_2699a10_645 | RP | POS | 10.65 | 349.2699 | | | | 7.56E-02 | -0.34 | 0.98 | 8.68E-01 | 0.03 | 0.92 | 1.35E-01 | -0.32 | 0.88 | 1.02E-01 | -0.36 | 0.85 | 1684815 | 352339 | 1550412 | 405577 | 1543635 | 411930 | 1557189 | 403190 |
| RP_POS_413_30258a11_306 | RP | POS | 11.31 | 413.3026 | | | | 3.33E-02 | -0.40 | 0.73 | 8.68E-01 | -0.03 | 0.68 | 3.96E-02 | -0.42 | 0.87 | 8.67E-02 | -0.39 | 0.63 | 1100159 | 85715 | 1060639 | 99762 | 1062306 | 111003 | 1058973 | 88209 |
| RP_POS_418_24918a13_07 | RP | POS | 13.07 | 418.2492 | | | | 1.61E-01 | -0.29 | 0.61 | 8.68E-01 | -0.03 | 0.22 | 1.81E-01 | -0.31 | 0.66 | 2.30E-01 | -0.28 | 0.58 | 1787006 | 473946 | 1651266 | 454706 | 1658856 | 455036 | 1643675 | 458867 |
| HILIC_NEG_552_76215a2_91 | HILIC | NEG | 2.91 | 552.7622 | | | | 2.40E-07 | -0.64 | 1.35 | 8.68E-01 | 0.04 | 0.76 | 1.22E-04 | -0.63 | 1.51 | 2.87E-04 | -0.66 | 1.52 | 266355 | 24453 | 200667 | 111958 | 198798 | 119491 | 202536 | 105069 |
| HILIC_POS_371_2402a5_16 | HILIC | POS | 5.16 | 371.2402 | | | | 8.32E-01 | -0.05 | 0.58 | 8.68E-01 | 0.03 | 0.47 | 9.04E-01 | -0.03 | 0.12 | 7.89E-01 | -0.06 | 0.36 | 14437567 | 6290894 | 14169844 | 5786278 | 14073278 | 5465984 | 14266410 | 6144045 |
| RP_POS_336_18341a10_581 | RP | POS | 10.58 | 336.1834 | | | | 1.11E-02 | 0.49 | 0.80 | 8.69E-01 | -0.03 | 0.35 | 3.38E-02 | 0.47 | 0.79 | 1.71E-02 | 0.50 | 1.00 | 521068 | 248614 | 658377 | 285065 | 663119 | 269571 | 653635 | 302437 |
| RP_POS_443_33432a13_004 | RP | POS | 13.00 | 443.3343 | | | | 5.22E-01 | 0.13 | 0.43 | 8.69E-01 | 0.03 | 0.31 | 5.22E-01 | 0.15 | 0.56 | 6.15E-01 | 0.11 | 0.40 | 4258277 | 1960646 | 4515913 | 2000107 | 4482752 | 1967553 | 4549074 | 2051582 |
| RP_POS_609_15918a9_666 | RP | POS | 9.67 | 609.1592 | | | | 2.46E-01 | -0.22 | 0.56 | 8.69E-01 | -0.03 | 0.59 | 2.80E-01 | -0.23 | 0.44 | 3.44E-01 | -0.20 | 0.82 | 2213479 | 122663 | 2182874 | 145613 | 2185286 | 142691 | 2180462 | 149887 |
| RP_POS_203_08086a5_411 | RP | POS | 5.41 | 203.0809 | | | | 8.67E-01 | 0.03 | 0.28 | 8.70E-01 | -0.03 | 0.12 | 9.45E-01 | 0.02 | 0.19 | 8.16E-01 | 0.05 | 0.11 | 2025563 | 333760 | 2037259 | 367133 | 2043328 | 337244 | 2031190 | 398140 |
| RP_POS_192_90997a0_498 | RP | POS | 0.50 | 192.9100 | | | | 3.90E-04 | -0.46 | 1.22 | 8.70E-01 | -0.04 | 1.21 | 3.96E-03 | -0.47 | 1.27 | 1.66E-02 | -0.44 | 1.28 | 1130689 | 162633 | 901558 | 559618 | 910808 | 597158 | 892308 | 525323 |
| RP_POS_316_2847a8_965 | RP | POS | 8.97 | 316.2847 | | | | 3.23E-01 | 0.21 | 0.47 | 8.70E-01 | 0.03 | 0.23 | 3.54E-01 | 0.23 | 0.54 | 3.86E-01 | 0.19 | 0.44 | 1050098 | 1854754 | 1420116 | 1724597 | 1391625 | 1502407 | 1448607 | 1936566 |
| RP_POS_542_80774a6_133 | RP | POS | 6.13 | 542.8077 | | | | 1.26E-05 | 0.53 | 1.24 | 8.70E-01 | -0.04 | 1.06 | 3.86E-03 | 0.51 | 1.32 | 8.68E-04 | 0.54 | 1.30 | 69462 | 283183 | 1033303 | 2046497 | 1067003 | 1964472 | 999603 | 2144833 |
| HILIC_POS_178_0722a1_387 | HILIC | POS | 1.387 | 178.0722 | | | | 3.55E-16 | 1.02 | 1.68 | 8.70E-01 | -0.03 | 0.60 | 1.02E-07 | 1.00 | 1.65 | 3.71E-10 | 1.03 | 2.20 | 55380 | 35470 | 313214 | 261478 | 317519 | 236793 | 308909 | 286406 |
| HILIC_POS_532_70203a3_006 | HILIC | POS | 3.006 | 532.7020 | | | | 6.31E-05 | -0.54 | 1.34 | 8.71E-01 | 0.04 | 0.77 | 2.11E-03 | -0.53 | 1.46 | 2.45E-03 | -0.56 | 1.45 | 1010134 | 177919 | 775363 | 471981 | 767617 | 496885 | 783110 | 450623 |
| HILIC_POS_270_78946a3_016 | HILIC | POS | 3.016 | 270.7895 | | | | 7.92E-05 | -0.53 | 1.35 | 8.71E-01 | 0.04 | 0.77 | 2.69E-03 | -0.51 | 1.45 | 3.13E-03 | -0.54 | 1.48 | 1500877 | 238380 | 1172587 | 686114 | 1161330 | 722772 | 1183844 | 654565 |
| HILIC_POS_387_21396a4_248 | HILIC | POS | 4.248 | 387.2140 | | | | 4.14E-01 | -0.14 | 0.93 | 8.71E-01 | -0.03 | 0.88 | 4.39E-01 | -0.16 | 0.67 | 5.36E-01 | -0.13 | 0.38 | 1848017 | 364357 | 1781469 | 491715 | 1789532 | 485245 | 1773405 | 502899 |
| RP_POS_399_30795a10_705 | RP | POS | 10.71 | 399.3080 | | | | 1.28E-01 | -0.31 | 0.70 | 8.71E-01 | -0.03 | 0.55 | 1.61E-01 | -0.33 | 0.60 | 1.82E-01 | -0.30 | 1.07 | 61204383 | 6397368 | 59200357 | 6352272 | 59304483 | 5910906 | 59096231 | 6823876 |
| RP_POS_294_92041a15_445 | RP | POS | 15.45 | 294.9204 | | | | 1.67E-01 | -0.34 | 0.69 | 8.71E-01 | -0.03 | 0.48 | 1.77E-01 | -0.36 | 0.65 | 2.09E-01 | -0.33 | 0.64 | 18658753 | 2199752 | 18071425 | 1514864 | 18096201 | 1487184 | 18046649 | 1556757 |
| RP_POS_313_27353a10_173 | RP | POS | 10.17 | 313.2735 | | | | 7.31E-01 | -0.07 | 0.69 | 8.71E-01 | -0.03 | 0.70 | 6.93E-01 | -0.08 | 0.48 | 8.24E-01 | -0.05 | 0.81 | 27990889 | 4075079 | 27698929 | 4392977 | 27770764 | 4786957 | 27627094 | 4008097 |
| HILIC_NEG_134_89476a0_71 | HILIC | NEG | 0.71 | 134.8948 | | | | 1.78E-01 | 0.28 | 0.75 | 8.71E-01 | -0.03 | 0.57 | 2.74E-01 | 0.26 | 0.88 | 1.76E-01 | 0.30 | 0.55 | 16109788 | 4492462 | 17346618 | 4360370 | 17417900 | 3756053 | 17275336 | 4929033 |
| HILIC_POS_682_32935a1_245 | HILIC | POS | 1.245 | 682.3294 | | | | 8.55E-01 | 0.04 | 0.57 | 8.71E-01 | 0.03 | 0.69 | 8.11E-01 | 0.05 | 0.77 | 9.31E-01 | 0.02 | 0.29 | 476425 | 291912 | 487468 | 310223 | 482402 | 321355 | 492534 | 301861 |
| HILIC_POS_546_729a2_992 | HILIC | POS | 2.992 | 546.7290 | | | | 1.85E-05 | -0.58 | 1.35 | 8.71E-01 | 0.04 | 0.77 | 9.60E-04 | -0.56 | 1.47 | 1.43E-03 | -0.59 | 1.47 | 856215 | 139321 | 651542 | 388532 | 645202 | 412543 | 657881 | 367043 |
| HILIC_NEG_299_68469a1_365 | HILIC | NEG | 1.365 | 299.6847 | | | | 1.12E-02 | -0.33 | 0.94 | 8.72E-01 | 0.04 | 0.40 | 1.05E-01 | -0.31 | 0.67 | 2.22E-02 | -0.35 | 1.12 | 364008 | 39805 | 327809 | 121805 | 325824 | 104074 | 329794 | 138333 |
| HILIC_NEG_296_68744a1_363 | HILIC | NEG | 1.363 | 296.6874 | | | | 3.44E-05 | 0.58 | 0.88 | 8.72E-01 | 0.04 | 0.59 | 3.53E-03 | 0.59 | 0.92 | 2.86E-04 | 0.56 | 1.16 | 105233 | 41429 | 159242 | 102026 | 157583 | 82425 | 160901 | 119295 |
| HILIC_POS_186_82828a3_023 | HILIC | POS | 3.023 | 186.8283 | | | | 5.51E-05 | -0.55 | 1.35 | 8.72E-01 | 0.04 | 0.77 | 1.97E-03 | -0.53 | 1.45 | 2.34E-03 | -0.56 | 1.46 | 1124991 | 182516 | 879249 | 492502 | 871244 | 518876 | 887254 | 469791 |
| RP_POS_341_0549a8_805 | RP | POS | 8.81 | 341.0549 | | | | 4.50E-01 | 0.11 | 0.74 | 8.72E-01 | 0.04 | 1.00 | 3.20E-01 | 0.12 | 1.02 | 6.94E-01 | 0.09 | 0.47 | 26081454 | 8338395 | 27936425 | 19555561 | 27618348 | 25406691 | 28254503 | 11266678 |
| RP_POS_139_05435a15_491 | RP | POS | 15.49 | 139.0544 | | | | 2.73E-03 | -0.49 | 0.83 | 8.72E-01 | 0.03 | 0.22 | 1.69E-02 | -0.47 | 0.82 | 8.73E-03 | -0.51 | 1.01 | 17223043 | 2021932 | 15742022 | 3195435 | 15690125 | 3140803 | 15793919 | 3280223 |
| HILIC_NEG_548_76624a2_909 | HILIC | NEG | 2.909 | 548.7662 | | | | 4.73E-07 | -0.62 | 1.35 | 8.72E-01 | 0.04 | 0.77 | 1.98E-04 | -0.60 | 1.50 | 4.17E-04 | -0.64 | 1.53 | 3455761 | 286785 | 2646830 | 1430835 | 2623650 | 1523212 | 2670010 | 1347253 |
| RP_POS_309_20303a9_912 | RP | POS | 9.91 | 309.2030 | | | | 8.00E-01 | 0.05 | 0.48 | 8.72E-01 | 0.03 | 0.55 | 7.61E-01 | 0.06 | 0.26 | 8.92E-01 | 0.03 | 0.46 | 12339784 | 2495820 | 12478027 | 3170957 | 12426695 | 3275419 | 12529360 | 3095383 |

**Supplementary table 1: Metabolomics results** (in asceding order according to p-value from progressive ALD vs  non-progressive ALD comparison, with molecular features with ID_level of 1-2 first)

| Feature_ID | RP_HILIC | POS_NEG | Average.Rt | Average.Mz | Metabolite.name | curated_MSMS | ID_level | all_ALD_vs_control | | | Prog_ALD_vs_non-prog_ALD | | | Prog_ALD_vs_control_ALD | | | Non-prog_ALD_vs_control | | | Healthy_control | | all_ALD | | Non-prog_ALD | | Progressive_ALD | |
|---|---|---|---|---|---|---|---|---|---|---|---|---|---|---|---|---|---|---|---|---|---|---|---|---|---|---|---|
| | | | | | | | | t-test p | Cohen's d | PLS-DA VIP | t-test p | Cohen's d | PLS-DA VIP | t-test p | Cohen's d | PLS-DA VIP | t-test p | Cohen's d | PLS-DA VIP | Mean | SD | Mean | SD | Mean | SD | Mean | SD |
| RP_POS_397_14792a6_593 | RP | POS | 6.59 | 397.1479 | | | | 1.64E-01 | -0.27 | 0.56 | 8.72E-01 | -0.03 | 0.24 | 1.82E-01 | -0.29 | 0.61 | 2.59E-01 | -0.25 | 0.43 | 16703217 | 1774747 | 16179772 | 1987987 | 16211950 | 2102091 | 16147594 | 1887840 |
| HILIC_NEG_404_63226a2_947 | HILIC | NEG | 2.947 | 404.6323 | | | | 1.22E-05 | -0.61 | 1.30 | 8.72E-01 | 0.03 | 0.74 | 4.81E-04 | -0.59 | 1.44 | 9.71E-04 | -0.63 | 1.41 | 183572 | 33211 | 138343 | 79868 | 137050 | 85919 | 139636 | 74178 |
| RP_NEG_297_15411a11_61 | RP | NEG | 11.61 | 297.1541 | | | | 6.73E-02 | -0.34 | 0.70 | 8.72E-01 | 0.03 | 0.72 | 1.17E-01 | -0.32 | 0.88 | 1.05E-01 | -0.36 | 0.65 | 2173768 | 288031 | 2058541 | 351758 | 2052853 | 376490 | 2064229 | 328911 |
| HILIC_NEG_87_00832a1_688 | HILIC | NEG | 1.688 | 87.0083 | | | | 3.12E-02 | -0.45 | 1.01 | 8.73E-01 | 0.03 | 0.60 | 5.35E-02 | -0.43 | 0.75 | 4.87E-02 | -0.46 | 1.06 | 4954468 | 2532178 | 3819032 | 2497732 | 3778679 | 2671628 | 3859384 | 2337462 |
| RP_POS_511_51904a10_731 | RP | POS | 10.73 | 511.5190 | | | | 1.81E-05 | 0.58 | 1.05 | 8.73E-01 | 0.04 | 0.87 | 3.33E-03 | 0.60 | 1.08 | 1.68E-04 | 0.56 | 1.19 | 2537599 | 1552062 | 4806762 | 4291638 | 4737463 | 3408712 | 4876060 | 5057971 |
| RP_POS_267_15607a9_21 | RP | POS | 9.21 | 267.1561 | | | | 2.82E-01 | 0.17 | 0.45 | 8.73E-01 | 0.03 | 0.36 | 3.20E-01 | 0.19 | 0.51 | 4.29E-01 | 0.16 | 0.66 | 471714 | 157750 | 512561 | 253718 | 508468 | 260847 | 516653 | 248965 |
| HILIC_POS_338_68753a3_023 | HILIC | POS | 3.023 | 338.6875 | | | | 4.27E-04 | -0.50 | 1.31 | 8.73E-01 | -0.04 | 0.76 | 2.90E-03 | -0.52 | 1.40 | 1.04E-02 | -0.49 | 1.40 | 476068 | 102713 | 369905 | 229571 | 373604 | 243548 | 366205 | 217112 |
| RP_POS_310_23422a9_36 | RP | POS | 9.36 | 310.2342 | | | | 7.60E-03 | -0.58 | 0.94 | 8.73E-01 | 0.03 | 0.33 | 1.60E-02 | -0.57 | 1.00 | 1.19E-02 | -0.60 | 0.91 | 3307219 | 291938 | 3146020 | 260999 | 3141829 | 264926 | 3150212 | 259632 |
| RP_POS_266_87213a0_249 | RP | POS | 0.25 | 266.8721 | | | | 5.88E-01 | 0.12 | 0.50 | 8.73E-01 | -0.03 | 0.14 | 6.67E-01 | 0.10 | 0.29 | 5.80E-01 | 0.13 | 0.40 | 760358 | 88938 | 770004 | 80860 | 771302 | 83563 | 768705 | 78890 |
| HILIC_POS_628_69232a2_992 | HILIC | POS | 2.992 | 628.6923 | | | | 5.82E-06 | -0.60 | 1.35 | 8.74E-01 | 0.04 | 0.76 | 5.27E-04 | -0.58 | 1.47 | 8.40E-04 | -0.62 | 1.47 | 1759035 | 265348 | 1323786 | 792090 | 1311095 | 839900 | 1336478 | 749572 |
| HILIC_NEG_157_90071a1_414 | HILIC | NEG | 1.414 | 157.9007 | | | | 2.82E-01 | 0.21 | 0.58 | 8.74E-01 | 0.03 | 0.66 | 3.46E-01 | 0.23 | 0.40 | 3.33E-01 | 0.19 | 0.62 | 1085263 | 134684 | 1115808 | 149770 | 1113416 | 115312 | 1118200 | 178917 |
| RP_POS_988_73529a0_495 | RP | POS | 0.50 | 988.7353 | | | | 4.72E-01 | -0.17 | 0.61 | 8.74E-01 | -0.03 | 0.33 | 4.47E-01 | -0.18 | 0.46 | 5.62E-01 | -0.15 | 0.43 | 864991 | 104794 | 850230 | 84066 | 851569 | 96711 | 848891 | 70156 |
| HILIC_NEG_244_8483a5_465 | HILIC | NEG | 5.465 | 244.8483 | | | | 9.05E-01 | -0.02 | 0.09 | 8.75E-01 | -0.03 | 0.30 | 8.61E-01 | -0.04 | 0.26 | 9.70E-01 | -0.01 | 0.18 | 1317239 | 449316 | 1306258 | 450937 | 1313433 | 442826 | 1299083 | 463291 |
| RP_POS_271_20554a8_691 | RP | POS | 8.69 | 271.2055 | | | | 4.17E-01 | 0.15 | 0.53 | 8.75E-01 | 0.03 | 0.20 | 4.63E-01 | 0.16 | 0.38 | 5.10E-01 | 0.13 | 0.45 | 127226 | 213263 | 165495 | 278203 | 161069 | 244430 | 169922 | 310784 |
| RP_NEG_435_14615a8_933 | RP | NEG | 8.93 | 435.1462 | | | | 1.54E-01 | 0.25 | 0.88 | 8.75E-01 | -0.03 | 0.65 | 2.79E-01 | 0.24 | 0.56 | 1.65E-01 | 0.27 | 0.69 | 7869065 | 5533504 | 9626965 | 7289639 | 9742674 | 6448862 | 9511255 | 8108451 |
| HILIC_NEG_362_7836a2_93 | HILIC | NEG | 2.93 | 362.7836 | | | | 2.15E-06 | -0.60 | 1.34 | 8.75E-01 | 0.03 | 0.76 | 3.48E-04 | -0.58 | 1.48 | 7.75E-04 | -0.62 | 1.49 | 753652 | 79568 | 585235 | 308533 | 580346 | 330528 | 590123 | 288150 |
| HILIC_NEG_336_69138a2_947 | HILIC | NEG | 2.947 | 336.6914 | | | | 1.13E-05 | -0.58 | 1.32 | 8.75E-01 | -0.03 | 0.79 | 2.80E-04 | -0.59 | 1.45 | 2.90E-03 | -0.56 | 1.46 | 527515 | 70797 | 411578 | 220755 | 415065 | 240583 | 408091 | 201383 |
| RP_POS_608_86865a0_489 | RP | POS | 0.49 | 608.8687 | | | | 1.69E-07 | 0.63 | 1.35 | 8.76E-01 | -0.03 | 1.20 | 2.47E-04 | 0.61 | 1.43 | 2.02E-04 | 0.65 | 1.43 | 790724 | 221363 | 2006653 | 2134260 | 2040312 | 2187433 | 1972995 | 2101408 |
| RP_POS_163_12274a0_71 | RP | POS | 0.71 | 163.1227 | | | | 1.35E-08 | 0.75 | 1.49 | 8.76E-01 | -0.03 | 0.30 | 5.95E-05 | 0.73 | 1.40 | 9.16E-06 | 0.76 | 1.62 | 36621 | 116104 | 289783 | 363607 | 295516 | 350999 | 284049 | 379277 |
| RP_NEG_243_75516a0_57 | RP | NEG | 0.57 | 243.7552 | | | | 3.98E-04 | 0.49 | 0.86 | 8.76E-01 | -0.03 | 0.85 | 1.76E-02 | 0.47 | 0.96 | 1.46E-03 | 0.50 | 1.09 | 1452108 | 434912 | 1942141 | 1105232 | 1959551 | 939020 | 1924732 | 1259218 |
| HILIC_NEG_98_93902a7_354 | HILIC | NEG | 7.354 | 98.9390 | | | | 6.14E-01 | 0.08 | 0.46 | 8.76E-01 | 0.03 | 0.50 | 6.30E-01 | 0.10 | 0.32 | 7.19E-01 | 0.07 | 0.58 | 2246957 | 152451 | 2264842 | 227638 | 2261259 | 205590 | 2268424 | 249805 |
| RP_POS_193_09488a2_877 | RP | POS | 2.88 | 193.0949 | | 193.0942(100), 134.0682(8) | 4 | 2.30E-01 | 0.14 | 0.70 | 8.76E-01 | -0.04 | 0.27 | 3.79E-01 | 0.12 | 0.81 | 3.99E-01 | 0.16 | 0.52 | 925915 | 1108964 | 2174456 | 10153545 | 2334214 | 11613176 | 2014697 | 8565920 |
| HILIC_NEG_155_86562a7_678 | HILIC | NEG | 7.678 | 155.8656 | | | | 2.08E-01 | -0.25 | 0.50 | 8.76E-01 | -0.03 | 0.24 | 2.49E-01 | -0.27 | 0.50 | 2.77E-01 | -0.23 | 0.63 | 1014542 | 265048 | 944699 | 283766 | 949159 | 260712 | 940239 | 307698 |
| RP_POS_826_53394a12_487 | RP | POS | 12.49 | 826.5339 | | | | 6.72E-01 | -0.08 | 0.92 | 8.76E-01 | -0.03 | 0.21 | 6.70E-01 | -0.10 | 0.67 | 7.54E-01 | -0.07 | 0.49 | 2511616 | 1130042 | 2410632 | 1280047 | 2430743 | 1138677 | 2390520 | 1418826 |
| RP_POS_318_21945a10_59 | RP | POS | 10.59 | 318.2195 | | | | 5.96E-04 | 0.54 | 0.94 | 8.76E-01 | 0.03 | 0.33 | 7.43E-03 | 0.56 | 0.90 | 2.34E-03 | 0.53 | 1.06 | 3560260 | 1300476 | 4696338 | 2215277 | 4661541 | 1867860 | 4731135 | 2534499 |
| RP_POS_180_02762a15_527 | RP | POS | 15.53 | 180.0276 | | | | 5.72E-01 | -0.12 | 0.50 | 8.77E-01 | 0.03 | 0.13 | 6.68E-01 | -0.10 | 0.41 | 5.53E-01 | -0.13 | 0.33 | 1895248 | 545597 | 1832790 | 527874 | 1824534 | 487871 | 1841047 | 569940 |
| RP_POS_290_8786a15_426 | RP | POS | 15.43 | 290.8786 | | | | 6.75E-01 | -0.07 | 0.49 | 8.77E-01 | 0.03 | 0.35 | 7.84E-01 | -0.05 | 0.56 | 6.79E-01 | -0.09 | 0.51 | 1866349 | 209266 | 1845430 | 332875 | 1840231 | 359937 | 1850629 | 307022 |
| RP_POS_349_01547a0_623 | RP | POS | 0.62 | 349.0155 | | | | 3.86E-03 | -0.44 | 1.04 | 8.77E-01 | 0.03 | 0.98 | 2.22E-02 | -0.42 | 1.02 | 1.72E-02 | -0.46 | 1.06 | 1056836 | 344981 | 794007 | 646168 | 783918 | 665273 | 804096 | 633081 |
| HILIC_NEG_179_05746a0_562 | HILIC | NEG | 0.562 | 179.0575 | | | | 6.41E-01 | -0.09 | 0.43 | 8.77E-01 | -0.03 | 0.40 | 6.35E-01 | -0.11 | 0.29 | 7.32E-01 | -0.07 | 0.19 | 2320705 | 2073626 | 2115802 | 2372806 | 2152835 | 2277297 | 2078770 | 2487255 |
| RP_NEG_242_17572a7_71 | RP | NEG | 7.71 | 242.1757 | | | | 8.02E-01 | 0.06 | 0.37 | 8.77E-01 | -0.03 | 0.18 | 8.64E-01 | 0.04 | 0.39 | 7.65E-01 | 0.07 | 0.35 | 4058594 | 894434 | 4102071 | 680605 | 4112662 | 604295 | 4091479 | 755312 |
| HILIC_POS_189_07315a5_921 | HILIC | POS | 5.921 | 189.0732 | | | | 3.36E-01 | -0.20 | 0.97 | 8.78E-01 | 0.03 | 0.76 | 4.26E-01 | -0.18 | 0.73 | 3.54E-01 | -0.21 | 0.88 | 3955090 | 1051497 | 3746783 | 1070487 | 3730186 | 1084742 | 3763379 | 1066792 |
| RP_POS_370_29483a8_585 | RP | POS | 8.59 | 370.2948 | | | | 5.96E-05 | 0.67 | 1.28 | 8.78E-01 | 0.03 | 0.83 | 5.37E-04 | 0.68 | 1.28 | 8.83E-04 | 0.65 | 1.30 | 1302152 | 613124 | 1910672 | 942585 | 1896079 | 943404 | 1925265 | 951115 |
| HILIC_NEG_340_72769a2_938 | HILIC | NEG | 2.938 | 340.7277 | | | | 6.03E-06 | -0.59 | 1.32 | 8.78E-01 | 0.03 | 0.76 | 4.69E-04 | -0.58 | 1.45 | 1.04E-03 | -0.61 | 1.47 | 260756 | 35984 | 200142 | 111588 | 198415 | 119957 | 201868 | 103741 |
| HILIC_POS_58_06611a1_509 | HILIC | POS | 1.509 | 58.0661 | | | | 9.65E-01 | -0.01 | 0.35 | 8.78E-01 | 0.03 | 0.94 | 9.68E-01 | 0.01 | 0.10 | 8.99E-01 | -0.02 | 0.05 | 2588134 | 1178766 | 2576559 | 1609193 | 2551728 | 1371648 | 2601390 | 1830075 |
| HILIC_NEG_276_8248a2_931 | HILIC | NEG | 2.931 | 276.8248 | | | | 1.30E-06 | -0.61 | 1.34 | 8.78E-01 | 0.03 | 0.76 | 2.83E-04 | -0.59 | 1.47 | 6.11E-04 | -0.63 | 1.50 | 1337358 | 129576 | 1045145 | 526904 | 1037016 | 562405 | 1053274 | 494458 |
| HILIC_POS_102_05539a5_784 | HILIC | POS | 5.784 | 102.0554 | | | | 3.25E-01 | -0.18 | 0.96 | 8.78E-01 | 0.03 | 0.64 | 4.77E-01 | -0.16 | 0.67 | 3.04E-01 | -0.20 | 0.75 | 746947 | 240309 | 695387 | 300191 | 690755 | 238117 | 700019 | 354012 |
| HILIC_NEG_268_79288a2_937 | HILIC | NEG | 2.937 | 268.7929 | | | | 7.06E-06 | -0.56 | 1.33 | 8.79E-01 | 0.03 | 0.76 | 7.02E-04 | -0.55 | 1.45 | 1.58E-03 | -0.58 | 1.49 | 715529 | 70234 | 565748 | 294250 | 561217 | 316557 | 570279 | 273282 |
| RP_POS_162_99109a0_45 | RP | POS | 0.45 | 162.9911 | | | | 6.02E-07 | -0.65 | 1.32 | 8.79E-01 | 0.03 | 1.16 | 2.02E-04 | -0.63 | 1.40 | 2.45E-04 | -0.66 | 1.40 | 2917202 | 405777 | 2116007 | 1346786 | 2095385 | 1402187 | 2136629 | 1302954 |
| RP_POS_240_23224a10_244 | RP | POS | 10.24 | 240.2322 | | | | 3.64E-04 | 0.61 | 0.97 | 8.79E-01 | -0.03 | 0.54 | 7.02E-03 | 0.59 | 0.95 | 5.15E-04 | 0.62 | 1.24 | 491459 | 218225 | 678477 | 320192 | 683373 | 255069 | 673582 | 376868 |
| RP_POS_347_19598a10_704 | RP | POS | 10.70 | 347.1960 | | | | 2.06E-07 | 0.69 | 1.56 | 8.79E-01 | 0.03 | 0.53 | 1.14E-04 | 0.71 | 1.43 | 7.85E-05 | 0.68 | 1.55 | 280466 | 167089 | 592153 | 484987 | 584740 | 466451 | 599565 | 507481 |
| RP_POS_802_5946a12_043 | RP | POS | 12.04 | 802.5946 | | | | 6.37E-01 | 0.07 | 0.36 | 8.80E-01 | -0.03 | 0.29 | 7.67E-01 | 0.06 | 0.24 | 6.38E-01 | 0.09 | 0.39 | 1629580 | 2256588 | 1897601 | 4013989 | 1958915 | 4046494 | 1836287 | 4021349 |
| RP_POS_585_53223a10_848 | RP | POS | 10.85 | 585.5322 | | | | 3.17E-05 | 0.79 | 1.26 | 8.80E-01 | 0.03 | 0.35 | 3.44E-04 | 0.80 | 1.24 | 1.04E-04 | 0.77 | 1.49 | 35180138 | 12954156 | 47576552 | 15399839 | 47341399 | 13118468 | 47811706 | 17519787 |
| RP_POS_199_16925a9_912 | RP | POS | 9.91 | 199.1693 | | | | 6.74E-01 | 0.08 | 0.45 | 8.80E-01 | 0.03 | 0.53 | 6.57E-01 | 0.09 | 0.26 | 7.79E-01 | 0.06 | 0.37 | 2520024 | 511120 | 2567321 | 663341 | 2557201 | 683493 | 2577441 | 649347 |
| RP_POS_191_1799a8_812 | RP | POS | 8.81 | 191.1799 | | | | 9.29E-01 | 0.02 | 0.37 | 8.80E-01 | 0.03 | 0.52 | 8.78E-01 | 0.03 | 0.29 | 9.96E-01 | 0.00 | 0.34 | 585771 | 256973 | 590671 | 301360 | 586074 | 313221 | 595267 | 292127 |
| RP_POS_808_07715a13_68 | RP | POS | 13.68 | 808.0772 | | | | 7.21E-03 | 0.56 | 1.16 | 8.80E-01 | -0.03 | 0.27 | 1.49E-02 | 0.54 | 0.97 | 1.40E-02 | 0.57 | 1.07 | 582053 | 458434 | 841365 | 449203 | 848200 | 477618 | 834529 | 423639 |
| HILIC_NEG_510_64792a2_934 | HILIC | NEG | 2.934 | 510.6479 | | | | 1.11E-05 | -0.57 | 1.32 | 8.80E-01 | -0.03 | 0.77 | 3.01E-04 | -0.59 | 1.47 | 2.94E-03 | -0.56 | 1.47 | 137270 | 19156 | 105015 | 61870 | 105954 | 67319 | 104077 | 56572 |
| RP_NEG_485_20923a11_258 | RP | NEG | 11.26 | 485.2092 | | | | 3.41E-04 | 0.72 | 1.51 | 8.81E-01 | 0.03 | 1.04 | 1.16E-03 | 0.73 | 1.42 | 1.15E-03 | 0.70 | 1.42 | 932249 | 218674 | 1104381 | 231520 | 1100875 | 219907 | 1107887 | 244771 |
| RP_POS_1027_66565a10_932 | RP | POS | 10.93 | 1027.6657 | | | | 1.84E-01 | 0.23 | 1.01 | 8.81E-01 | -0.03 | 0.84 | 3.08E-01 | 0.21 | 0.74 | 2.15E-01 | 0.25 | 0.88 | 291374 | 163882 | 340972 | 230235 | 344444 | 219143 | 337501 | 243001 |
| HILIC_NEG_410_62479a2_947 | HILIC | NEG | 2.947 | 410.6248 | | | | 2.41E-05 | -0.61 | 1.31 | 8.81E-01 | 0.03 | 0.75 | 7.47E-04 | -0.59 | 1.43 | 1.10E-03 | -0.62 | 1.43 | 227035 | 44178 | 171821 | 98173 | 170341 | 104116 | 173301 | 92888 |
| RP_POS_898_55011a13_21 | RP | POS | 13.21 | 898.5501 | | | | 7.17E-04 | 0.65 | 1.07 | 8.81E-01 | 0.03 | 0.29 | 3.77E-03 | 0.67 | 1.12 | 1.48E-03 | 0.64 | 1.25 | 1213465 | 366798 | 1488725 | 416809 | 1482439 | 341856 | 1495011 | 483797 |
| RP_POS_859_60193a11_839 | RP | POS | 11.84 | 859.6019 | | | | 2.89E-01 | 0.23 | 0.49 | 8.81E-01 | -0.03 | 0.20 | 3.76E-01 | 0.22 | 0.38 | 2.88E-01 | 0.25 | 0.62 | 26671717 | 5927337 | 27928472 | 5226997 | 28007031 | 4768699 | 27849913 | 5696270 |
| HILIC_POS_553_40704a5_647 | HILIC | POS | 5.647 | 553.4070 | | | | 3.40E-01 | -0.16 | 1.02 | 8.82E-01 | -0.03 | 0.77 | 3.80E-01 | -0.18 | 0.66 | 4.61E-01 | -0.15 | 0.89 | 586259 | 98246 | 564946 | 138341 | 567024 | 136551 | 562868 | 141463 |
| HILIC_NEG_197_73663a3_033 | HILIC | NEG | 3.033 | 197.7366 | | | | 7.14E-05 | 0.53 | 1.16 | 8.82E-01 | -0.03 | 0.82 | 2.18E-03 | 0.52 | 1.39 | 3.11E-03 | 0.55 | 1.18 | 749307 | 51694 | 819489 | 144493 | 821656 | 153494 | 817322 | 136429 |
| RP_POS_928_73901a0_499 | RP | POS | 0.50 | 928.7390 | | | | 1.93E-05 | -0.57 | 1.26 | 8.82E-01 | -0.03 | 1.18 | 4.90E-04 | -0.59 | 1.34 | 2.69E-03 | -0.56 | 1.31 | 868957 | 124075 | 686267 | 348355 | 691487 | 370884 | 681047 | 327976 |
| HILIC_NEG_99_92547a4_289 | HILIC | NEG | 4.289 | 99.9255 | | | | 9.15E-01 | 0.02 | 0.84 | 8.82E-01 | 0.03 | 0.81 | 8.70E-01 | 0.04 | 0.07 | 9.82E-01 | 0.00 | 0.59 | 2609224 | 446259 | 2619246 | 507862 | 2611650 | 506392 | 2626841 | 514355 |
| RP_POS_307_04355a0_71 | RP | POS | 0.71 | 307.0436 | | | | 1.92E-03 | 0.43 | 1.36 | 8.82E-01 | -0.03 | 0.91 | 2.30E-02 | 0.41 | 1.40 | 1.25E-02 | 0.44 | 1.32 | 292201 | 160613 | 449026 | 404987 | 455082 | 401973 | 442970 | 411969 |
| RP_POS_532_38806a11_442 | RP | POS | 11.44 | 532.3881 | | | | 3.61E-01 | -0.18 | 0.61 | 8.82E-01 | -0.03 | 0.23 | 3.97E-01 | -0.19 | 0.61 | 4.44E-01 | -0.16 | 0.51 | 2425581 | 293269 | 2369217 | 325164 | 2374078 | 298624 | 2364356 | 352699 |
| RP_POS_304_89594a0_47 | RP | POS | 0.47 | 304.8959 | | | | 1.63E-06 | -0.61 | 1.31 | 8.82E-01 | 0.03 | 1.19 | 4.42E-04 | -0.59 | 1.37 | 4.72E-04 | -0.63 | 1.40 | 2014819 | 223300 | 1545570 | 845394 | 1532943 | 876557 | 1558197 | 821760 |
| RP_NEG_285_24496a10_747 | RP | NEG | 10.75 | 285.2450 | | | | 2.33E-02 | -0.39 | 0.99 | 8.82E-01 | -0.03 | 0.93 | 3.50E-02 | -0.41 | 0.98 | 7.64E-02 | -0.38 | 0.97 | 2453381 | 338072 | 2276179 | 477337 | 2283306 | 519712 | 2269053 | 436058 |
| RP_POS_122_92949a15_568 | RP | POS | 15.57 | 122.9295 | | | | 1.05E-01 | 0.27 | 0.54 | 8.82E-01 | -0.03 | 0.09 | 1.38E-01 | 0.26 | 0.56 | 1.98E-01 | 0.29 | 0.51 | 5560573 | 179515 | 5628826 | 268750 | 5632838 | 323586 | 5624813 | 202944 |
| HILIC_NEG_315_04196a7_355 | HILIC | NEG | 7.355 | 315.0420 | | | | 2.11E-01 | 0.18 | 0.35 | 8.82E-01 | -0.03 | 0.42 | 3.52E-01 | 0.16 | 0.31 | 3.06E-01 | 0.19 | 0.45 | 1989682 | 125361 | 2034924 | 282945 | 2039146 | 300644 | 2030702 | 267071 |
| RP_POS_852_76514a0_497 | RP | POS | 0.50 | 852.7651 | | | | 5.52E-04 | -0.49 | 1.21 | 8.82E-01 | -0.03 | 1.14 | 2.88E-03 | -0.51 | 1.25 | 1.40E-02 | -0.47 | 1.26 | 1325478 | 156079 | 1165731 | 355285 | 1171029 | 386720 | 1160434 | 324686 |
| HILIC_POS_345_19946a4_846 | HILIC | POS | 4.846 | 345.1995 | | | | 7.28E-05 | 0.57 | 0.94 | 8.82E-01 | 0.03 | 0.42 | 3.82E-03 | 0.59 | 0.92 | 5.02E-04 | 0.56 | 1.15 | 223982 | 133698 | 377332 | 287721 | 373043 | 237467 | 381621 | 332909 |
| RP_POS_268_14008a0_71 | RP | POS | 0.71 | 268.1401 | | | | 1.87E-12 | 0.86 | 1.65 | 8.83E-01 | -0.03 | 0.66 | 2.04E-06 | 0.85 | 1.64 | 2.43E-07 | 0.88 | 1.76 | 238524 | 154152 | 2127445 | 2339146 | 2162267 | 2267867 | 2092622 | 2430880 |
| RP_POS_222_18231a9_477 | RP | POS | 9.48 | 222.1823 | | | | 1.12E-01 | 0.31 | 0.87 | 8.83E-01 | -0.03 | 0.54 | 2.16E-01 | 0.29 | 0.78 | 1.05E-01 | 0.32 | 0.79 | 1704535 | 228693 | 1781615 | 253578 | 1785380 | 196686 | 1777851 | 301996 |
| RP_POS_181_98721a0_257 | RP | POS | 0.26 | 181.9872 | | | | 7.57E-01 | -0.06 | 0.27 | 8.83E-01 | -0.03 | 0.58 | 7.25E-01 | -0.08 | 0.38 | 8.39E-01 | -0.05 | 0.55 | 4006331 | 849580 | 3951760 | 904032 | 3965123 | 962523 | 3938397 | 851124 |
| RP_POS_478_38901a12_889 | RP | POS | 12.89 | 478.3890 | | | | 8.76E-01 | -0.03 | 0.36 | 8.83E-01 | -0.03 | 0.24 | 8.37E-01 | -0.05 | 0.24 | 9.41E-01 | -0.02 | 0.41 | 7965849 | 2636139 | 7882076 | 2653923 | 7921296 | 2725839 | 7842857 | 2607109 |
| HILIC_POS_271_20557a0_507 | HILIC | POS | 0.507 | 271.2056 | | | | 1.20E-02 | 0.40 | 0.77 | 8.83E-01 | 0.03 | 0.44 | 4.89E-02 | 0.41 | 0.70 | 2.78E-02 | 0.38 | 0.77 | 394798 | 317012 | 594398 | 540189 | 586414 | 456491 | 602382 | 617292 |
| HILIC_NEG_456_77396a2_923 | HILIC | NEG | 2.923 | 456.7740 | | | | 1.26E-06 | -0.60 | 1.35 | 8.83E-01 | 0.03 | 0.77 | 2.90E-04 | -0.59 | 1.50 | 7.14E-04 | -0.62 | 1.52 | 828378 | 72599 | 642352 | 341240 | 637312 | 365645 | 647393 | 318618 |
| RP_POS_540_88171a0_496 | RP | POS | 0.50 | 540.8817 | | | | 3.18E-07 | 0.62 | 1.34 | 8.84E-01 | 0.03 | 1.22 | 1.36E-04 | 0.64 | 1.45 | 5.21E-04 | 0.61 | 1.41 | 106570 | 62729 | 326356 | 388891 | 320631 | 401831 | 332081 | 379505 |
| RP_NEG_656_31952a10_301 | RP | NEG | 10.30 | 656.3195 | | | | 2.60E-03 | 0.66 | 1.17 | 8.84E-01 | 0.03 | 0.28 | 5.28E-03 | 0.68 | 1.10 | 5.18E-03 | 0.65 | 1.12 | 2324921 | 666260 | 2743636 | 589250 | 2734971 | 548121 | 2752301 | 633188 |
| RP_POS_153_00705a0_451 | RP | POS | 0.45 | 153.0071 | | | | 3.00E-07 | -0.66 | 1.34 | 8.85E-01 | 0.03 | 1.17 | 1.54E-04 | -0.64 | 1.42 | 1.91E-04 | -0.67 | 1.42 | 834149 | 105018 | 605696 | 379436 | 600146 | 394518 | 611245 | 367660 |
| RP_POS_677_36371a6_603 | RP | POS | 6.60 | 677.3637 | | | | 7.42E-07 | 0.59 | 1.29 | 8.85E-01 | -0.03 | 1.11 | 8.40E-04 | 0.57 | 1.39 | 2.97E-04 | 0.61 | 1.37 | 29829 | 76222 | 1083514 | 1989456 | 1112491 | 1963578 | 1054536 | 2034524 |
| RP_POS_486_13669a8_805 | RP | POS | 8.81 | 486.1367 | | | | 2.46E-04 | 0.44 | 0.82 | 8.85E-01 | -0.03 | 0.33 | 1.13E-02 | 0.42 | 0.79 | 8.09E-03 | 0.45 | 0.92 | 579989 | 619157 | 2823211 | 5808171 | 2907774 | 5920148 | 2738648 | 5752862 |
| RP_POS_628_49567a10_873 | RP | POS | 10.87 | 628.4957 | | | | 3.25E-03 | -0.52 | 0.95 | 8.85E-01 | -0.03 | 0.79 | 1.25E-02 | -0.54 | 0.94 | 9.46E-03 | -0.51 | 0.92 | 3736473 | 397798 | 3467753 | 531530 | 3475491 | 482388 | 3460014 | 581372 |
| HILIC_NEG_294_68777a1_363 | HILIC | NEG | 1.363 | 294.6878 | | | | 1.36E-05 | 0.62 | 0.95 | 8.85E-01 | -0.03 | 0.61 | 3.29E-03 | 0.61 | 0.93 | 3.87E-05 | 0.64 | 1.33 | 86570 | 39842 | 138162 | 89059 | 139458 | 69830 | 136866 | 105572 |
| RP_POS_304_26126a11_161 | RP | POS | 11.16 | 304.2613 | | | | 6.09E-07 | 0.84 | 1.30 | 8.86E-01 | 0.03 | 0.58 | 9.07E-05 | 0.85 | 1.33 | 2.37E-06 | 0.82 | 1.62 | 15796929 | 6038619 | 23431486 | 9187707 | 23298375 | 7097829 | 23564597 | 10960626 |
| RP_NEG_238_07845a8_301 | RP | NEG | 8.30 | 238.0785 | | | | 1.91E-01 | -0.46 | 0.85 | 8.86E-01 | 0.01 | 0.20 | 1.97E-01 | -0.45 | 0.68 | 1.89E-01 | -0.46 | 0.63 | 14111335 | 39851183 | 4656769 | 6736757 | 4559363 | 7144932 | 4754175 | 6373741 |
| RP_POS_359_15128a10_703 | RP | POS | 10.70 | 359.1513 | | | | 2.42E-07 | 0.73 | 1.58 | 8.86E-01 | -0.03 | 0.54 | 5.67E-05 | 0.71 | 1.49 | 6.68E-05 | 0.74 | 1.53 | 585805 | 317582 | 1099188 | 750809 | 1109999 | 778751 | 1088378 | 729549 |
| RP_POS_372_24884a10_851 | RP | POS | 10.85 | 372.2488 | | | | 4.90E-03 | 0.55 | 0.99 | 8.86E-01 | 0.03 | 0.75 | 8.96E-03 | 0.56 | 1.03 | 1.58E-02 | 0.53 | 1.04 | 4077031 | 1297426 | 4869743 | 1440795 | 4849001 | 1499254 | 4890485 | 1394813 |
| HILIC_POS_169_98592a5_325 | HILIC | POS | 5.325 | 169.9859 | | | | 4.40E-03 | 0.39 | 1.28 | 8.86E-01 | -0.03 | 0.64 | 4.14E-02 | 0.37 | 1.32 | 2.08E-02 | 0.40 | 1.36 | 619156 | 83696 | 696810 | 223167 | 700022 | 217900 | 693599 | 230481 |
| RP_POS_199_13063a14_343 | RP | POS | 14.34 | 199.1306 | | | | 6.17E-01 | -0.11 | 0.78 | 8.87E-01 | -0.03 | 0.38 | 6.09E-01 | -0.12 | 0.50 | 6.89E-01 | -0.10 | 0.67 | 31428861 | 11916218 | 30242796 | 10552355 | 30394120 | 10438315 | 30091472 | 10769017 |
| HILIC_POS_164_02905a5_783 | HILIC | POS | 5.783 | 164.0291 | | | | 1.01E-01 | -0.29 | 1.23 | 8.87E-01 | -0.03 | 0.68 | 1.72E-01 | -0.31 | 1.09 | 1.39E-01 | -0.28 | 1.15 | 540721 | 90385 | 507769 | 117350 | 509451 | 95129 | 506086 | 136995 |
| HILIC_POS_94_06577a1_23 | HILIC | POS | 1.23 | 94.0658 | | | | 1.90E-01 | -0.25 | 0.44 | 8.87E-01 | -0.03 | 0.87 | 2.82E-01 | -0.27 | 0.39 | 1.99E-01 | -0.24 | 0.55 | 4094590 | 5438926 | 2576048 | 6209460 | 2665055 | 3769159 | 2487041 | 7979909 |
| RP_NEG_610_39313a11_163 | RP | NEG | 11.16 | 610.3931 | | | | 1.48E-02 | 0.46 | 1.22 | 8.87E-01 | 0.03 | 0.93 | 2.75E-02 | 0.47 | 1.12 | 3.76E-02 | 0.44 | 1.19 | 4189734 | 356795 | 4380807 | 426928 | 4374701 | 427307 | 4386914 | 430798 |
| RP_NEG_311_16949a11_158 | RP | NEG | 11.16 | 311.1695 | | | | 4.76E-01 | -0.15 | 0.37 | 8.87E-01 | 0.03 | 0.28 | 5.85E-01 | -0.13 | 0.27 | 4.55E-01 | -0.16 | 0.46 | 2117718 | 871313 | 1990751 | 867817 | 1978339 | 727294 | 2003163 | 996148 |
| RP_POS_574_87494a0_492 | RP | POS | 0.49 | 574.8749 | | | | 3.97E-07 | 0.61 | 1.33 | 8.88E-01 | -0.03 | 1.21 | 3.53E-04 | 0.60 | 1.41 | 3.04E-04 | 0.63 | 1.42 | 521896 | 164384 | 1206675 | 1234894 | 1224254 | 1265553 | 1189096 | 1216056 |
| RP_POS_553_45868a10_982 | RP | POS | 10.98 | 553.4587 | | | | 2.83E-02 | 0.43 | 1.23 | 8.88E-01 | -0.03 | 1.23 | 6.59E-02 | 0.41 | 1.09 | 3.85E-02 | 0.44 | 1.06 | 5929858 | 1597235 | 6678174 | 1757767 | 6703190 | 1650673 | 6653159 | 1875246 |

**Supplementary table 1: Metabolomics results** (in asceding order according to p-value from progressive ALD vs non-progressive ALD comparison, with molecular features with ID_level of 1-2 first)

| | | | | | | | | all_ALD_vs_control | | | Prog_ALD_vs_non-prog_ALD | | | Prog_ALD_vs_control_ALD | | | Non-prog_ALD_vs_control | | | Healthy_control | | all_ALD | | Non-prog_ALD | | Progressive_ALD | |
|---|---|---|---|---|---|---|---|---|---|---|---|---|---|---|---|---|---|---|---|---|---|---|---|---|---|---|---|
| | | | | | | | | t-test | Cohen's | PLS-DA | t-test | Cohen's | PLS-DA | t-test | Cohen's | PLS-DA | t-test | Cohen's | PLS-DA | | | | | | | | |
| Feature_ID | RP_HILIC | POS_NEG | Average.Rt | Average.Mz | Metabolite.name | curated_MSMS | ID_level | p | d | VIP | p | d | VIP | p | d | VIP | p | d | VIP | Mean | SD | Mean | SD | Mean | SD | Mean | SD |
| HILIC_POS_136_11208a0_636 | HILIC | POS | 0.636 | 136.1121 | | | | 1.14E-01 | -0.33 | 0.85 | 8.88E-01 | -0.03 | 0.73 | 1.20E-01 | -0.34 | 0.84 | 1.89E-01 | -0.31 | 0.49 | 2166434 | 172222 | 2110365 | 169186 | 2112769 | 188224 | 2107960 | 149639 |
| HILIC_NEG_375_60498a1_363 | HILIC | NEG | 1.363 | 375.6050 | | | | 6.62E-06 | 0.62 | 0.95 | 8.88E-01 | -0.03 | 0.58 | 2.61E-03 | 0.60 | 0.94 | 4.26E-05 | 0.64 | 1.30 | 4371337 | 2160130 | 7523515 | 5504907 | 7601480 | 4506281 | 7445550 | 6395895 |
| HILIC_NEG_257_8194a3_026 | HILIC | NEG | 3.026 | 257.8194 | | | | 9.82E-03 | -0.45 | 1.14 | 8.88E-01 | 0.03 | 0.79 | 3.26E-02 | -0.43 | 0.90 | 2.36E-02 | -0.46 | 0.84 | 9736922 | 1339437 | 8933162 | 1894323 | 8906390 | 1916730 | 8959935 | 1890724 |
| HILIC_POS_371_24091a3_93 | HILIC | POS | 3.93 | 371.2409 | | | | 6.87E-01 | 0.07 | 0.79 | 8.89E-01 | 0.03 | 0.75 | 6.84E-01 | 0.09 | 0.39 | 7.76E-01 | 0.06 | 0.63 | 14466856 | 3297276 | 14756853 | 4184121 | 14697752 | 3980821 | 14815953 | 4417801 |
| RP_POS_785_6521a14_567 | RP | POS | 14.57 | 785.6521 | | | | 4.39E-01 | -0.15 | 0.51 | 8.89E-01 | 0.03 | 0.12 | 5.46E-01 | -0.13 | 0.47 | 4.50E-01 | -0.16 | 0.38 | 1779422 | 1039251 | 1608222 | 1206142 | 1591211 | 1177937 | 1625233 | 1245440 |
| HILIC_NEG_354_75201a2_935 | HILIC | NEG | 2.935 | 354.7520 | | | | 1.50E-06 | -0.63 | 1.34 | 8.89E-01 | 0.03 | 0.76 | 1.98E-04 | -0.61 | 1.49 | 5.50E-04 | -0.64 | 1.50 | 226369 | 29602 | 171859 | 94560 | 170526 | 101748 | 173193 | 87803 |
| RP_NEG_177_95628a15_476 | RP | NEG | 15.48 | 177.9563 | | | | 1.46E-01 | -0.28 | 0.59 | 8.89E-01 | 0.03 | 0.19 | 2.06E-01 | -0.27 | 0.62 | 1.93E-01 | -0.29 | 0.61 | 1772005 | 617203 | 1581613 | 693778 | 1571832 | 753155 | 1591393 | 636430 |
| RP_POS_273_08359a0_709 | RP | POS | 0.71 | 273.0836 | | | | 1.00E-02 | 0.32 | 0.81 | 8.89E-01 | 0.03 | 0.62 | 7.88E-02 | 0.34 | 0.72 | 3.75E-02 | 0.31 | 1.25 | 200808 | 87589 | 301968 | 354644 | 296975 | 300421 | 306961 | 404733 |
| HILIC_NEG_199_73282a3_033 | HILIC | NEG | 3.033 | 199.7328 | | | | 2.21E-01 | 0.24 | 0.59 | 8.89E-01 | 0.03 | 0.82 | 2.38E-01 | 0.26 | 0.63 | 3.18E-01 | 0.23 | 0.51 | 314406 | 65830 | 331269 | 70966 | 330270 | 75355 | 332267 | 67042 |
| RP_POS_663_45386a14_26 | RP | POS | 14.26 | 663.4539 | | | | 8.81E-01 | 0.03 | 0.42 | 8.89E-01 | 0.03 | 0.33 | 8.34E-01 | 0.04 | 0.37 | 9.53E-01 | 0.01 | 0.57 | 2008425 | 1081972 | 2043055 | 1289300 | 2024912 | 1427791 | 2061198 | 1148588 |
| RP_NEG_205_83942a3_116 | RP | NEG | 3.12 | 205.8394 | | | | 4.08E-01 | 0.16 | 0.65 | 8.89E-01 | -0.03 | 0.29 | 4.95E-01 | 0.15 | 0.40 | 4.34E-01 | 0.18 | 0.49 | 2165679 | 1688105 | 2457067 | 1821980 | 2482664 | 1914860 | 2431470 | 1743255 |
| RP_POS_280_65439a10_615 | RP | POS | 10.62 | 280.6544 | | | | 1.38E-02 | -0.49 | 0.79 | 8.89E-01 | 0.03 | 0.62 | 2.97E-02 | -0.48 | 0.80 | 2.53E-02 | -0.50 | 0.81 | 815672 | 173282 | 724650 | 185398 | 722046 | 192531 | 727255 | 179902 |
| RP_NEG_698_86639a0_49 | RP | NEG | 0.49 | 698.8664 | | | | 3.86E-07 | 0.61 | 1.33 | 8.89E-01 | -0.03 | 1.20 | 3.07E-04 | 0.60 | 1.41 | 3.50E-04 | 0.63 | 1.41 | 850228 | 231009 | 1898570 | 1894775 | 1925176 | 1962821 | 1871963 | 1843774 |
| RP_POS_258_89948a0_498 | RP | POS | 0.50 | 258.8995 | | | | 2.64E-06 | -0.58 | 1.31 | 8.90E-01 | 0.03 | 1.20 | 4.73E-04 | -0.56 | 1.39 | 1.06E-03 | -0.59 | 1.38 | 48786385 | 4316532 | 37482694 | 21603062 | 37180323 | 23091127 | 37785065 | 20236653 |
| RP_NEG_196_94128a15_779 | RP | NEG | 15.78 | 196.9413 | | | | 8.00E-01 | 0.05 | 0.36 | 8.90E-01 | 0.03 | 0.75 | 7.80E-01 | 0.07 | 0.23 | 8.66E-01 | 0.04 | 0.18 | 909451 | 357613 | 928118 | 371369 | 922920 | 340350 | 933316 | 403425 |
| RP_NEG_233_01889a8_812 | RP | NEG | 8.81 | 233.0189 | | | | 1.77E-02 | 0.32 | 0.77 | 8.90E-01 | 0.03 | 0.70 | 5.90E-03 | 0.34 | 1.09 | 1.63E-01 | 0.31 | 0.54 | 3820842 | 4052387 | 6940013 | 10825161 | 6788394 | 13983205 | 7091632 | 6417383 |
| RP_POS_322_14294a9_357 | RP | POS | 9.36 | 322.1429 | | | | 4.96E-06 | 0.66 | 1.29 | 8.90E-01 | 0.03 | 0.81 | 4.48E-04 | 0.67 | 1.23 | 2.25E-04 | 0.64 | 1.26 | 934265 | 254196 | 1277286 | 556967 | 1269495 | 522723 | 1285077 | 594469 |
| HILIC_NEG_542_73218a2_922 | HILIC | NEG | 2.922 | 542.7322 | | | | 4.98E-06 | -0.60 | 1.32 | 8.90E-01 | 0.03 | 0.76 | 4.33E-04 | -0.59 | 1.44 | 9.13E-04 | -0.62 | 1.49 | 197243 | 27323 | 150940 | 84323 | 149765 | 90123 | 152116 | 78999 |
| HILIC_POS_387_21368a5_784 | HILIC | POS | 5.784 | 387.2137 | | | | 2.49E-01 | -0.23 | 1.02 | 8.90E-01 | -0.03 | 0.76 | 2.85E-01 | -0.24 | 0.78 | 3.29E-01 | -0.21 | 0.67 | 1534486 | 352518 | 1449198 | 382982 | 1454535 | 369259 | 1443862 | 399917 |
| RP_POS_718_91187a6_73 | RP | POS | 6.73 | 718.9119 | | | | 4.49E-07 | 0.60 | 1.30 | 8.90E-01 | -0.03 | 1.12 | 6.30E-04 | 0.59 | 1.39 | 2.39E-04 | 0.62 | 1.38 | 26317 | 75527 | 1044386 | 1880245 | 1070585 | 1860700 | 1018187 | 1918121 |
| RP_POS_170_20627a10_847 | RP | POS | 10.85 | 170.2063 | | | | 4.58E-07 | 0.92 | 1.49 | 8.90E-01 | -0.03 | 0.14 | 1.47E-05 | 0.91 | 1.46 | 3.38E-06 | 0.94 | 1.74 | 3065324 | 739267 | 3967104 | 943951 | 3980256 | 898116 | 3953952 | 996648 |
| RP_POS_680_47876a13_57 | RP | POS | 13.57 | 680.4788 | | | | 8.18E-01 | -0.05 | 0.43 | 8.90E-01 | -0.03 | 0.25 | 7.83E-01 | -0.06 | 0.44 | 8.88E-01 | -0.03 | 0.36 | 3457934 | 293046 | 3444169 | 293350 | 3448245 | 317005 | 3440092 | 270810 |
| HILIC_POS_171_1857a5_921 | HILIC | POS | 5.921 | 171.1857 | | | | 8.24E-01 | 0.05 | 0.69 | 8.90E-01 | -0.03 | 0.59 | 8.89E-01 | 0.03 | 0.49 | 7.95E-01 | 0.06 | 0.34 | 58608600 | 16086042 | 59336711 | 15859153 | 59556804 | 15998875 | 59116618 | 15877516 |
| RP_POS_255_23184a10_283 | RP | POS | 10.28 | 255.2318 | | | | 2.26E-01 | -0.24 | 0.86 | 8.91E-01 | 0.03 | 0.77 | 3.01E-01 | -0.23 | 0.61 | 2.63E-01 | -0.25 | 0.94 | 1617148 | 163515 | 1575928 | 173220 | 1573526 | 181991 | 1578331 | 165800 |
| RP_POS_267_05847a0_706 | RP | POS | 0.71 | 267.0585 | | | | 5.53E-01 | -0.08 | 1.07 | 8.91E-01 | 0.03 | 0.77 | 7.08E-01 | -0.07 | 0.72 | 5.94E-01 | -0.10 | 1.30 | 772120 | 121692 | 749559 | 312428 | 745226 | 320821 | 753892 | 307003 |
| RP_POS_266_17294a7_715 | RP | POS | 7.72 | 266.1729 | | | | 4.02E-01 | -0.20 | 0.77 | 8.91E-01 | 0.03 | 0.21 | 4.88E-01 | -0.19 | 0.56 | 3.78E-01 | -0.21 | 0.53 | 2688094 | 597835 | 2590702 | 453535 | 2584425 | 345443 | 2596979 | 544219 |
| RP_POS_166_98488a11_672 | RP | POS | 11.67 | 166.9849 | | | | 1.19E-01 | 0.34 | 1.46 | 8.91E-01 | 0.03 | 0.61 | 1.29E-01 | 0.35 | 1.32 | 1.79E-01 | 0.33 | 1.01 | 11519014 | 4573392 | 12952846 | 4031932 | 12897085 | 4339048 | 13008608 | 3743132 |
| RP_NEG_264_99155a6_739 | RP | NEG | 6.74 | 264.9916 | | | | 3.14E-01 | -0.22 | 0.55 | 8.91E-01 | -0.03 | 0.31 | 3.47E-01 | -0.23 | 0.42 | 3.70E-01 | -0.21 | 0.56 | 12922011 | 803318 | 12760204 | 716628 | 12770114 | 636876 | 12750294 | 794846 |
| HILIC_POS_188_82552a3_023 | HILIC | POS | 3.023 | 188.8255 | | | | 1.94E-05 | -0.58 | 1.34 | 8.91E-01 | 0.03 | 0.76 | 8.94E-04 | -0.57 | 1.46 | 1.38E-03 | -0.60 | 1.46 | 837032 | 137145 | 643164 | 363194 | 638160 | 384520 | 648167 | 344391 |
| RP_NEG_231_02188a8_812 | RP | NEG | 8.81 | 231.0219 | | | | 6.45E-03 | 0.33 | 0.72 | 8.91E-01 | -0.03 | 0.85 | 3.02E-04 | 0.31 | 1.36 | 1.20E-01 | 0.34 | 0.51 | 7184529 | 5703142 | 16602470 | 32413009 | 17049278 | 43540565 | 16155662 | 15048555 |
| RP_POS_437_27682a11_612 | RP | POS | 11.61 | 437.2768 | | | | 5.39E-02 | -0.36 | 1.11 | 8.91E-01 | 0.03 | 0.81 | 8.41E-02 | -0.35 | 1.16 | 9.56E-02 | -0.38 | 0.84 | 1594053 | 150277 | 1531342 | 176745 | 1528909 | 198184 | 1533774 | 154358 |
| RP_POS_311_21909a10_825 | RP | POS | 10.83 | 311.2191 | | | | 6.83E-01 | 0.07 | 0.74 | 8.91E-01 | -0.03 | 0.17 | 7.90E-01 | 0.06 | 0.92 | 6.53E-01 | 0.09 | 0.27 | 4481297 | 1829362 | 4644467 | 2324628 | 4676446 | 2030248 | 4612489 | 2606546 |
| HILIC_POS_144_98228a3_101 | HILIC | POS | 3.101 | 144.9823 | | | | 4.92E-01 | 0.12 | 0.95 | 8.92E-01 | -0.03 | 0.83 | 6.11E-01 | 0.10 | 0.57 | 5.13E-01 | 0.13 | 0.75 | 3292171 | 646723 | 3393463 | 917555 | 3406069 | 921434 | 3380857 | 922839 |
| HILIC_POS_147_14917a5_888 | HILIC | POS | 5.888 | 147.1492 | | | | 2.82E-01 | 0.23 | 0.45 | 8.92E-01 | 0.03 | 0.57 | 2.98E-01 | 0.24 | 0.50 | 3.63E-01 | 0.21 | 0.34 | 136504568 | 55962662 | 148732675 | 53217451 | 148002169 | 54552471 | 149463180 | 52392088 |
| RP_POS_462_30725a9_945 | RP | POS | 9.95 | 462.3073 | | | | 1.27E-03 | -0.61 | 1.11 | 8.92E-01 | -0.03 | 0.65 | 3.44E-03 | -0.62 | 1.14 | 5.42E-03 | -0.59 | 1.01 | 654330 | 38592 | 626555 | 45874 | 627184 | 46512 | 625927 | 45690 |
| RP_NEG_272_95886a15_304 | RP | NEG | 15.30 | 272.9589 | | | | 2.10E-01 | -0.24 | 0.53 | 8.92E-01 | -0.03 | 0.13 | 2.12E-01 | -0.25 | 0.52 | 3.27E-01 | -0.23 | 0.47 | 1223351 | 44047 | 1211585 | 50365 | 1212275 | 57273 | 1210895 | 42941 |
| RP_NEG_459_19745a11_166 | RP | NEG | 11.17 | 459.1975 | | | | 4.46E-01 | -0.15 | 0.58 | 8.92E-01 | -0.03 | 0.14 | 4.13E-01 | -0.16 | 0.38 | 5.80E-01 | -0.13 | 0.47 | 715639 | 89965 | 701079 | 103217 | 702492 | 124007 | 699666 | 78378 |
| RP_NEG_296_99893a7_376 | RP | NEG | 7.38 | 296.9989 | | | | 1.95E-01 | 0.28 | 0.71 | 8.92E-01 | 0.03 | 0.27 | 2.23E-01 | 0.29 | 0.59 | 2.57E-01 | 0.26 | 0.58 | 2717355 | 1052012 | 2993436 | 980782 | 2980022 | 951846 | 3006850 | 1018391 |
| RP_NEG_152_978a15_497 | RP | NEG | 15.50 | 152.9780 | | | | 8.97E-01 | -0.03 | 0.30 | 8.92E-01 | 0.03 | 0.62 | 9.53E-01 | -0.01 | 0.36 | 8.62E-01 | -0.04 | 0.35 | 989134 | 36886 | 988163 | 35921 | 987672 | 37301 | 988654 | 34859 |
| RP_POS_296_89929a15_46 | RP | POS | 15.46 | 296.8993 | | | | 2.94E-01 | 0.24 | 0.70 | 8.92E-01 | 0.03 | 0.15 | 3.03E-01 | 0.26 | 0.52 | 3.58E-01 | 0.23 | 0.58 | 13108032 | 3714722 | 13871140 | 2913044 | 13831359 | 2975624 | 13910922 | 2878761 |
| HILIC_POS_396_03836a4_799 | HILIC | POS | 4.799 | 396.0384 | | | | 3.77E-02 | 0.43 | 0.78 | 8.92E-01 | 0.03 | 0.70 | 5.46E-02 | 0.44 | 0.71 | 6.71E-02 | 0.41 | 0.70 | 368530 | 86731 | 406095 | 86789 | 404911 | 85698 | 407278 | 88721 |
| HILIC_POS_96_08138a0_505 | HILIC | POS | 0.505 | 96.0814 | | | | 2.15E-05 | -0.70 | 1.27 | 8.92E-01 | -0.03 | 0.75 | 2.06E-04 | -0.72 | 1.41 | 5.49E-04 | -0.69 | 1.33 | 815376 | 187884 | 615338 | 294685 | 619355 | 305014 | 611321 | 287026 |
| RP_NEG_896_56488a13_369 | RP | NEG | 13.37 | 896.5649 | | | | 1.80E-01 | 0.23 | 0.64 | 8.92E-01 | 0.03 | 0.24 | 2.30E-01 | 0.25 | 0.69 | 2.78E-01 | 0.22 | 0.49 | 4673603 | 1395240 | 5095460 | 1905229 | 5069496 | 1877721 | 5121424 | 1951067 |
| HILIC_POS_137_13249a5_647 | HILIC | POS | 5.647 | 137.1325 | | | | 6.59E-02 | -0.33 | 1.03 | 8.92E-01 | -0.03 | 0.70 | 1.13E-01 | -0.35 | 0.61 | 1.08E-01 | -0.32 | 0.92 | 2619627 | 294590 | 2499792 | 373432 | 2504879 | 336605 | 2494705 | 410356 |
| RP_POS_223_13063a8_072 | RP | POS | 8.07 | 223.1306 | | | | 7.89E-01 | -0.05 | 0.37 | 8.93E-01 | -0.03 | 0.09 | 7.59E-01 | -0.07 | 0.33 | 8.60E-01 | -0.04 | 0.47 | 1258408 | 301024 | 1241909 | 302409 | 1246017 | 319175 | 1237800 | 287857 |
| RP_NEG_851_11438a9_503 | RP | NEG | 9.50 | 851.1144 | | | | 4.63E-01 | -0.14 | 0.46 | 8.93E-01 | -0.03 | 0.27 | 4.75E-01 | -0.15 | 0.31 | 5.65E-01 | -0.13 | 0.33 | 4016287 | 240688 | 3978811 | 276188 | 3982560 | 282370 | 3975062 | 272682 |
| HILIC_NEG_373_6073a1_363 | HILIC | NEG | 1.363 | 373.6073 | | | | 6.97E-06 | 0.62 | 0.95 | 8.93E-01 | -0.03 | 0.59 | 2.62E-03 | 0.60 | 0.94 | 4.60E-05 | 0.63 | 1.29 | 3043629 | 1507664 | 5235433 | 3835418 | 5287437 | 3146083 | 5183428 | 4451710 |
| HILIC_POS_421_37097a3_173 | HILIC | POS | 3.173 | 421.3710 | | | | 5.97E-02 | 0.34 | 0.87 | 8.93E-01 | 0.03 | 0.82 | 7.88E-02 | 0.36 | 0.71 | 1.29E-01 | 0.33 | 0.86 | 1757451 | 701538 | 2049304 | 884331 | 2037326 | 946574 | 2061281 | 826890 |
| RP_POS_327_29028a10_412 | RP | POS | 10.41 | 327.2903 | | | | 1.53E-02 | -0.44 | 0.95 | 8.93E-01 | -0.03 | 0.80 | 2.09E-02 | -0.45 | 0.95 | 5.47E-02 | -0.42 | 0.93 | 1539368 | 226750 | 1415964 | 292064 | 1419918 | 327462 | 1412010 | 255110 |
| RP_POS_473_91483a6_667 | RP | POS | 6.67 | 473.9148 | | | | 4.31E-07 | 0.60 | 1.33 | 8.93E-01 | 0.03 | 1.15 | 7.95E-04 | 0.62 | 1.44 | 1.38E-04 | 0.59 | 1.41 | 23849 | 47667 | 995391 | 1792950 | 971156 | 1618979 | 1019625 | 1967905 |
| HILIC_NEG_530_70685a2_928 | HILIC | NEG | 2.928 | 530.7069 | | | | 9.57E-07 | -0.64 | 1.35 | 8.94E-01 | 0.03 | 0.76 | 1.69E-04 | -0.63 | 1.49 | 4.03E-04 | -0.66 | 1.47 | 382921 | 50642 | 287765 | 161791 | 285582 | 172619 | 289947 | 151920 |
| HILIC_POS_312_20139a0_779 | HILIC | POS | 0.779 | 312.2014 | | | | 4.30E-01 | -0.16 | 0.72 | 8.94E-01 | -0.03 | 0.78 | 4.45E-01 | -0.17 | 0.35 | 5.17E-01 | -0.15 | 0.79 | 752347 | 166955 | 725244 | 170324 | 727540 | 169896 | 722948 | 172446 |
| HILIC_NEG_379_60126a1_363 | HILIC | NEG | 1.363 | 379.6013 | | | | 5.66E-06 | 0.63 | 0.96 | 8.94E-01 | -0.03 | 0.59 | 2.45E-03 | 0.61 | 0.95 | 3.40E-05 | 0.64 | 1.32 | 635650 | 311373 | 1091759 | 788448 | 1102374 | 639628 | 1081145 | 920127 |
| RP_POS_422_32602a8_861 | RP | POS | 8.86 | 422.3260 | | | | 1.91E-03 | 0.49 | 0.88 | 8.94E-01 | -0.03 | 0.11 | 1.04E-02 | 0.47 | 1.07 | 1.15E-02 | 0.50 | 1.04 | 545342 | 219281 | 719816 | 384736 | 724994 | 407253 | 714639 | 364903 |
| RP_NEG_299_92615a3_96 | RP | NEG | 3.96 | 299.9262 | | | | 4.09E-01 | 0.14 | 0.78 | 8.94E-01 | -0.03 | 0.21 | 5.67E-01 | 0.12 | 0.59 | 4.07E-01 | 0.15 | 0.42 | 965037 | 341462 | 1031121 | 520438 | 1038121 | 449597 | 1024121 | 587368 |
| RP_POS_533_50092a10_731 | RP | POS | 10.73 | 533.5009 | | | | 2.61E-06 | 0.68 | 1.11 | 8.94E-01 | -0.03 | 0.71 | 1.07E-03 | 0.67 | 1.13 | 1.34E-05 | 0.70 | 1.35 | 1825820 | 936284 | 3107555 | 1989451 | 3134233 | 1613232 | 3080877 | 2322206 |
| RP_POS_264_8728a0_249 | RP | POS | 0.25 | 264.8728 | | | | 5.29E-01 | 0.13 | 0.56 | 8.95E-01 | 0.03 | 0.11 | 5.21E-01 | 0.14 | 0.36 | 6.22E-01 | 0.12 | 0.29 | 8095653 | 866653 | 8207304 | 869299 | 8195742 | 934819 | 8218866 | 807852 |
| RP_POS_694_47803a10_463 | RP | POS | 10.46 | 694.4780 | | | | 5.11E-01 | -0.15 | 0.49 | 8.95E-01 | 0.03 | 0.44 | 5.91E-01 | -0.14 | 0.36 | 4.96E-01 | -0.16 | 0.86 | 1884835 | 312139 | 1844543 | 255484 | 1841146 | 225083 | 1847941 | 284939 |
| RP_POS_214_91756a0_453 | RP | POS | 0.45 | 214.9176 | | | | 9.94E-04 | -0.51 | 1.16 | 8.95E-01 | 0.03 | 1.06 | 7.58E-03 | -0.49 | 1.15 | 7.10E-03 | -0.52 | 1.22 | 1926212 | 593684 | 1416145 | 1074572 | 1401893 | 1116552 | 1430397 | 1042053 |
| HILIC_NEG_377_60303a1_363 | HILIC | NEG | 1.363 | 377.6030 | | | | 5.83E-06 | 0.62 | 0.95 | 8.95E-01 | -0.03 | 0.59 | 2.47E-03 | 0.61 | 0.95 | 3.94E-05 | 0.64 | 1.30 | 2716405 | 1333104 | 4685304 | 3426137 | 4730730 | 2801409 | 4639878 | 3983000 |
| RP_POS_638_09833a0_624 | RP | POS | 0.62 | 638.0983 | | | | 6.36E-03 | -0.43 | 1.06 | 8.96E-01 | -0.03 | 1.00 | 1.74E-02 | -0.44 | 1.05 | 3.57E-02 | -0.41 | 1.10 | 1761423 | 478912 | 1429429 | 834938 | 1440473 | 877006 | 1418384 | 799426 |
| RP_NEG_266_93259a0_498 | RP | NEG | 0.50 | 266.9326 | | | | 3.25E-06 | -0.58 | 1.30 | 8.96E-01 | 0.03 | 1.19 | 3.91E-04 | -0.57 | 1.39 | 1.16E-03 | -0.60 | 1.36 | 1137467 | 125829 | 861246 | 521282 | 854367 | 563253 | 868124 | 481316 |
| RP_POS_140_94911a0_25 | RP | POS | 0.25 | 140.9491 | | | | 1.49E-01 | 0.26 | 0.57 | 8.96E-01 | 0.03 | 0.11 | 1.86E-01 | 0.28 | 0.52 | 2.38E-01 | 0.25 | 0.41 | 665804 | 170671 | 719578 | 211697 | 716788 | 215484 | 722367 | 209992 |
| RP_POS_322_91418a0_254 | RP | POS | 0.25 | 322.9142 | | | | 7.14E-01 | 0.08 | 0.47 | 8.96E-01 | -0.03 | 0.17 | 7.72E-01 | 0.07 | 0.21 | 7.02E-01 | 0.09 | 0.66 | 764195 | 158579 | 775670 | 134042 | 777434 | 141024 | 773906 | 128091 |
| RP_POS_420_26498a13_99 | RP | POS | 13.99 | 420.2650 | | | | 2.89E-02 | -0.50 | 0.83 | 8.96E-01 | -0.02 | 0.56 | 3.26E-02 | -0.51 | 0.91 | 5.36E-02 | -0.49 | 0.74 | 1463681 | 603288 | 1198525 | 493356 | 1205016 | 542804 | 1192034 | 443897 |
| RP_POS_381_79999a0_254 | RP | POS | 0.25 | 381.8000 | | | | 2.74E-01 | 0.22 | 0.54 | 8.96E-01 | -0.03 | 0.14 | 3.76E-01 | 0.21 | 0.37 | 2.82E-01 | 0.23 | 0.51 | 358912 | 70435 | 374834 | 72757 | 375790 | 65738 | 373877 | 79823 |
| HILIC_POS_126_09152a0_617 | HILIC | POS | 0.617 | 126.0915 | | | | 1.33E-01 | 0.18 | 0.53 | 8.97E-01 | -0.03 | 0.33 | 2.25E-01 | 0.16 | 0.66 | 3.21E-01 | 0.19 | 0.45 | 420748 | 218649 | 693818 | 1763446 | 716920 | 2070702 | 670715 | 1412110 |
| RP_POS_727_57397a12_445 | RP | POS | 12.45 | 727.5740 | | | | 1.43E-05 | -1.04 | 1.69 | 8.97E-01 | -0.02 | 0.48 | 2.28E-05 | -1.05 | 1.68 | 4.13E-05 | -1.03 | 1.52 | 2512257 | 1156107 | 1425150 | 865513 | 1436479 | 907213 | 1413821 | 830793 |
| RP_POS_250_93854a0_454 | RP | POS | 0.45 | 250.9385 | | | | 2.04E-04 | -0.54 | 1.20 | 8.97E-01 | 0.03 | 1.09 | 3.21E-03 | -0.53 | 1.22 | 3.18E-03 | -0.56 | 1.27 | 3161689 | 815617 | 2305855 | 1697262 | 2283695 | 1762542 | 2328015 | 1647006 |
| HILIC_NEG_186_11357a0_546 | HILIC | NEG | 0.546 | 186.1136 | | | | 5.27E-01 | -0.13 | 0.51 | 8.97E-01 | -0.03 | 0.50 | 5.49E-01 | -0.15 | 0.45 | 5.87E-01 | -0.12 | 0.39 | 202238 | 210959 | 175334 | 198656 | 177927 | 171890 | 172742 | 223995 |
| RP_POS_154_08638a3_143 | RP | POS | 3.14 | 154.0864 | | | | 1.60E-01 | 0.16 | 0.74 | 8.97E-01 | -0.03 | 0.20 | 3.22E-01 | 0.15 | 0.86 | 3.22E-01 | 0.18 | 0.45 | 6456 | 10230 | 3184245 | 22446586 | 3477179 | 24539804 | 2891310 | 20386368 |
| RP_POS_316_22458a9_231 | RP | POS | 9.23 | 316.2246 | | | | 3.60E-01 | -0.18 | 0.92 | 8.97E-01 | 0.03 | 0.69 | 4.69E-01 | -0.17 | 0.66 | 3.60E-01 | -0.20 | 1.01 | 831678 | 690550 | 700350 | 731020 | 690812 | 650529 | 709889 | 810132 |
| HILIC_NEG_112_98553a4_233 | HILIC | NEG | 4.233 | 112.9855 | | | | 3.68E-01 | 0.16 | 0.99 | 8.97E-01 | -0.03 | 0.84 | 4.79E-01 | 0.14 | 0.40 | 4.08E-01 | 0.17 | 0.71 | 537457571 | 99738422 | 557568216 | 134754689 | 559321637 | 137685153 | 555814794 | 133134387 |
| HILIC_POS_210_11008a0_549 | HILIC | POS | 0.549 | 210.1101 | | | | 5.85E-01 | -0.12 | 0.52 | 8.97E-01 | -0.03 | 0.56 | 6.06E-01 | -0.13 | 0.44 | 6.30E-01 | -0.11 | 0.41 | 264056 | 321488 | 229067 | 285581 | 232781 | 216464 | 225353 | 343355 |
| RP_POS_380_25436a10_058 | RP | POS | 10.06 | 380.2544 | | | | 3.77E-03 | 0.51 | 0.96 | 8.97E-01 | -0.03 | 0.60 | 1.74E-02 | 0.50 | 0.85 | 9.07E-03 | 0.52 | 1.00 | 928834 | 475644 | 1247112 | 649508 | 1255543 | 625661 | 1238680 | 678773 |
| RP_POS_260_89597a0_498 | RP | POS | 0.50 | 260.8960 | | | | 5.00E-06 | -0.56 | 1.30 | 8.98E-01 | 0.03 | 1.20 | 6.82E-04 | -0.55 | 1.37 | 1.45E-03 | -0.58 | 1.37 | 7325014 | 662763 | 5636894 | 3329565 | 5593679 | 3553813 | 5680109 | 3124877 |
| HILIC_POS_342_18304a0_851 | HILIC | POS | 0.851 | 342.1830 | | | | 8.33E-01 | 0.04 | 0.34 | 8.98E-01 | 0.03 | 0.44 | 7.83E-01 | 0.06 | 0.17 | 9.04E-01 | 0.03 | 0.20 | 2504970 | 1553415 | 2571760 | 1544420 | 2551737 | 1920250 | 2591782 | 1063467 |
| RP_NEG_468_80865a15_474 | RP | NEG | 15.47 | 468.8087 | | | | 2.45E-01 | 0.24 | 0.51 | 8.98E-01 | 0.03 | 0.35 | 2.67E-01 | 0.25 | 0.52 | 3.26E-01 | 0.23 | 0.51 | 3039683 | 109201 | 3065733 | 108794 | 3064327 | 111389 | 3067140 | 107250 |
| RP_NEG_394_89273a0_465 | RP | NEG | 0.47 | 394.8927 | | | | 8.30E-07 | -0.65 | 1.34 | 8.98E-01 | 0.03 | 1.22 | 2.14E-04 | -0.63 | 1.40 | 2.79E-04 | -0.66 | 1.40 | 782688 | 111618 | 573952 | 351512 | 569416 | 365778 | 578488 | 340305 |
| RP_NEG_772_79541a0_497 | RP | NEG | 0.50 | 772.7954 | | | | 4.36E-06 | -0.58 | 1.30 | 8.98E-01 | 0.03 | 1.21 | 6.03E-04 | -0.57 | 1.38 | 1.01E-03 | -0.60 | 1.37 | 6028069 | 716673 | 4568694 | 2763316 | 4533054 | 2916312 | 4604335 | 2630626 |
| RP_POS_391_20697a8_101 | RP | POS | 8.10 | 391.2070 | | | | 9.12E-03 | -0.46 | 0.81 | 8.98E-01 | -0.03 | 0.67 | 2.03E-02 | -0.47 | 0.88 | 3.10E-02 | -0.44 | 0.85 | 3348606 | 108464 | 3283703 | 147756 | 3285604 | 150775 | 3281803 | 146180 |
| HILIC_POS_537_53601a10_681 | HILIC | POS | 10.681 | 537.5360 | | | | 2.06E-01 | -0.22 | 0.60 | 8.98E-01 | 0.03 | 0.44 | 2.89E-01 | -0.21 | 0.47 | 2.73E-01 | -0.23 | 0.61 | 2151099 | 129216 | 2114263 | 176578 | 2111992 | 191328 | 2116533 | 162415 |
| RP_NEG_275_91916a15_699 | RP | NEG | 15.70 | 275.9192 | | | | 4.11E-01 | -0.17 | 0.35 | 8.99E-01 | -0.03 | 0.14 | 4.25E-01 | -0.18 | 0.33 | 4.96E-01 | -0.16 | 0.26 | 793783 | 263893 | 749559 | 259807 | 752892 | 263486 | 746226 | 258706 |
| HILIC_NEG_540_73511a2_922 | HILIC | NEG | 2.922 | 540.7351 | | | | 4.83E-07 | -0.63 | 1.35 | 8.99E-01 | 0.03 | 0.76 | 1.43E-04 | -0.62 | 1.51 | 4.11E-04 | -0.65 | 1.52 | 714102 | 72588 | 542519 | 296601 | 538729 | 317595 | 546308 | 277206 |
| RP_POS_546_35236a10_82 | RP | POS | 10.82 | 546.3524 | | | | 2.81E-01 | -0.24 | 0.85 | 8.99E-01 | -0.02 | 0.95 | 3.08E-01 | -0.26 | 0.59 | 3.32E-01 | -0.23 | 0.87 | 18860671 | 7071908 | 17353663 | 5855086 | 17428444 | 5389623 | 17278881 | 6340682 |
| RP_POS_201_00687a15_383 | RP | POS | 15.38 | 201.0069 | | | | 6.17E-01 | -0.10 | 0.60 | 8.99E-01 | 0.03 | 0.28 | 6.96E-01 | -0.08 | 0.31 | 6.29E-01 | -0.11 | 0.73 | 4886767 | 1746674 | 4701745 | 2004504 | 4676148 | 2156930 | 4727341 | 1861287 |
| RP_NEG_213_91513a15_549 | RP | NEG | 15.55 | 213.9151 | | | | 4.79E-01 | -0.17 | 0.38 | 9.00E-01 | 0.02 | 0.24 | 5.45E-01 | -0.15 | 0.40 | 4.77E-01 | -0.18 | 0.45 | 1391738 | 407016 | 1335728 | 313148 | 1331752 | 302372 | 1339705 | 326594 |
| RP_POS_120_98109a0_451 | RP | POS | 0.45 | 120.9811 | | | | 1.04E-06 | -0.64 | 1.31 | 9.00E-01 | 0.03 | 1.16 | 2.35E-04 | -0.63 | 1.39 | 3.30E-04 | -0.65 | 1.40 | 4716701 | 716957 | 3383354 | 2270462 | 3354525 | 2369716 | 3412183 | 2190423 |

**Supplementary table 1: Metabolomics results** (in asceding order according to p-value from progressive ALD vs  non-progressive ALD comparison, with molecular features with ID_level of 1-2 first)

| | | | | | | | | all_ALD_vs_control | | | Prog_ALD_vs_non-prog_ALD | | | Prog_ALD_vs_control_ALD | | | Non-prog_ALD_vs_control | | | Healthy_control | | all_ALD | | Non-prog_ALD | | Progressive_ALD | |
|---|---|---|---|---|---|---|---|---|---|---|---|---|---|---|---|---|---|---|---|---|---|---|---|---|---|---|---|
| | | | | | | | | t-test | Cohen's | PLS-DA | t-test | Cohen's | PLS-DA | t-test | Cohen's | PLS-DA | t-test | Cohen's | PLS-DA | | | | | | | | |
| Feature_ID | RP_HILIC | POS_NEG | Average.Rt | Average.Mz | Metabolite.name | curated_MSMS | ID_level | p | d | VIP | p | d | VIP | p | d | VIP | p | d | VIP | Mean | SD | Mean | SD | Mean | SD | Mean | SD |
| HILIC_POS_175_00172a1_384 | HILIC | POS | 1.384 | 175.0017 | | | | 1.80E-12 | 0.87 | 1.48 | 9.00E-01 | -0.03 | 0.61 | 2.67E-06 | 0.85 | 1.47 | 1.40E-07 | 0.88 | 1.95 | 228569 | 250422 | 2284638 | 2532816 | 2316774 | 2395557 | 2252502 | 2687087 |
| HILIC_NEG_136_89166a4_671 | HILIC | NEG | 4.671 | 136.8917 | | | | 8.74E-01 | 0.03 | 0.61 | 9.00E-01 | -0.02 | 0.53 | 9.25E-01 | 0.02 | 0.05 | 8.49E-01 | 0.05 | 0.23 | 7265269 | 2667027 | 7349679 | 2438774 | 7380589 | 2670015 | 7318769 | 2210359 |
| RP_POS_320_16986a6_254 | RP | POS | 6.25 | 320.1699 | | | | 1.23E-01 | -0.31 | 0.58 | 9.00E-01 | 0.03 | 0.34 | 1.76E-01 | -0.30 | 0.56 | 1.61E-01 | -0.32 | 0.51 | 15279421 | 1534753 | 14786513 | 1599231 | 14766305 | 1698709 | 14806722 | 1510241 |
| RP_POS_645_49646a10_456 | RP | POS | 10.46 | 645.4965 | | | | 6.28E-01 | -0.11 | 0.33 | 9.01E-01 | 0.02 | 0.08 | 7.01E-01 | -0.09 | 0.22 | 6.13E-01 | -0.12 | 0.36 | 1003233 | 200387 | 983972 | 173702 | 981784 | 161467 | 986161 | 186759 |
| HILIC_POS_81_9885a2_997 | HILIC | POS | 2.997 | 81.9885 | | | | 7.89E-05 | -0.58 | 1.32 | 9.01E-01 | 0.03 | 0.78 | 1.22E-03 | -0.57 | 1.39 | 2.17E-03 | -0.60 | 1.32 | 1459019 | 235571 | 1197298 | 483125 | 1191212 | 518220 | 1203383 | 450506 |
| RP_POS_420_25299a11_379 | RP | POS | 11.38 | 420.2530 | | | | 4.03E-04 | -0.61 | 1.21 | 9.01E-01 | 0.03 | 0.94 | 1.59E-03 | -0.60 | 1.23 | 3.46E-03 | -0.62 | 1.12 | 1581976 | 196469 | 1416650 | 280106 | 1413123 | 311016 | 1420178 | 248524 |
| RP_POS_134_99677a0_451 | RP | POS | 0.45 | 134.9968 | | | | 6.37E-07 | -0.64 | 1.32 | 9.01E-01 | 0.03 | 1.16 | 1.94E-04 | -0.63 | 1.40 | 2.90E-04 | -0.66 | 1.41 | 6808117 | 945035 | 4908402 | 3218635 | 4868032 | 3363921 | 4948771 | 3100218 |
| RP_NEG_180_97295a14_262 | RP | NEG | 14.26 | 180.9730 | | | | 8.71E-01 | -0.03 | 0.39 | 9.01E-01 | -0.03 | 0.15 | 8.42E-01 | -0.05 | 0.14 | 9.29E-01 | -0.02 | 0.53 | 2149402 | 846405 | 2121140 | 880110 | 2132164 | 863962 | 2110115 | 904608 |
| RP_POS_311_21915a10_458 | RP | POS | 10.46 | 311.2192 | | | | 2.78E-01 | 0.21 | 0.38 | 9.01E-01 | -0.03 | 0.18 | 3.45E-01 | 0.20 | 0.47 | 3.26E-01 | 0.23 | 0.42 | 5116620 | 1265559 | 5403610 | 1362428 | 5420642 | 1492961 | 5386577 | 1233198 |
| HILIC_NEG_234_81995a7_394 | HILIC | NEG | 7.394 | 234.8200 | | | | 5.46E-01 | -0.12 | 0.39 | 9.01E-01 | -0.03 | 0.47 | 5.63E-01 | -0.13 | 0.21 | 6.22E-01 | -0.11 | 0.42 | 495386 | 25023 | 492255 | 26479 | 492586 | 24847 | 491925 | 28265 |
| HILIC_NEG_228_00932a0_93 | HILIC | NEG | 0.93 | 228.0093 | | | | 3.80E-08 | 0.78 | 1.30 | 9.02E-01 | -0.03 | 1.19 | 9.19E-05 | 0.76 | 1.26 | 2.48E-06 | 0.79 | 1.52 | 99743 | 63412 | 209579 | 149507 | 211441 | 133056 | 207718 | 165680 |
| HILIC_POS_158_1541a1_247 | HILIC | POS | 1.247 | 158.1541 | | | | 2.04E-01 | -0.27 | 0.92 | 9.02E-01 | -0.02 | 0.43 | 2.08E-01 | -0.29 | 0.83 | 2.86E-01 | -0.26 | 0.88 | 1532847 | 596094 | 1380285 | 541893 | 1387022 | 604379 | 1373548 | 477413 |
| RP_POS_185_11481a13_245 | RP | POS | 13.25 | 185.1148 | | | | 9.31E-01 | 0.02 | 0.25 | 9.02E-01 | -0.02 | 0.30 | 9.80E-01 | 0.01 | 0.37 | 8.95E-01 | 0.03 | 0.55 | 7996239 | 1905791 | 8029594 | 1826057 | 8052287 | 1786115 | 8006900 | 1883019 |
| RP_NEG_218_81709a0_757 | RP | NEG | 0.76 | 218.8171 | | | | 2.11E-04 | -0.79 | 1.48 | 9.02E-01 | -0.02 | 0.14 | 3.51E-04 | -0.80 | 1.48 | 9.96E-04 | -0.78 | 1.27 | 6183019 | 1657790 | 4855367 | 1563033 | 4874738 | 1712392 | 4835996 | 1415257 |
| RP_POS_320_86957a0_472 | RP | POS | 0.47 | 320.8696 | | | | 6.93E-04 | -0.47 | 1.21 | 9.02E-01 | -0.03 | 1.23 | 4.94E-03 | -0.49 | 1.24 | 1.39E-02 | -0.46 | 1.26 | 503877 | 107954 | 391812 | 259163 | 395023 | 273598 | 388601 | 246627 |
| RP_NEG_520_90936a14_241 | RP | NEG | 14.24 | 520.9094 | | | | 9.83E-01 | 0.00 | 0.63 | 9.02E-01 | -0.03 | 0.33 | 9.40E-01 | -0.02 | 0.38 | 9.62E-01 | 0.01 | 0.62 | 1172240 | 528925 | 1169739 | 671878 | 1178060 | 564282 | 1161418 | 770388 |
| HILIC_NEG_352_75458a2_934 | HILIC | NEG | 2.934 | 352.7546 | | | | 2.03E-06 | -0.62 | 1.33 | 9.03E-01 | 0.03 | 0.76 | 2.34E-04 | -0.61 | 1.47 | 6.59E-04 | -0.63 | 1.48 | 496202 | 63895 | 379950 | 204588 | 377440 | 219848 | 382461 | 190318 |
| HILIC_NEG_229_06918a0_93 | HILIC | NEG | 0.93 | 229.0692 | | | | 7.07E-09 | 0.79 | 1.28 | 9.03E-01 | -0.03 | 1.09 | 3.51E-05 | 0.78 | 1.31 | 2.66E-06 | 0.80 | 1.49 | 55296 | 49712 | 153774 | 132077 | 155390 | 123718 | 152157 | 141185 |
| RP_POS_133_03453a13_108 | RP | POS | 13.11 | 133.0345 | | | | 1.57E-01 | -0.30 | 0.52 | 9.03E-01 | -0.02 | 0.18 | 1.90E-01 | -0.32 | 0.55 | 2.04E-01 | -0.29 | 0.61 | 4800346 | 2103943 | 4197714 | 1927239 | 4221299 | 1802778 | 4174128 | 2062319 |
| HILIC_POS_126_10269a4_556 | HILIC | POS | 4.556 | 126.1027 | | | | 9.84E-11 | 0.81 | 1.29 | 9.03E-01 | 0.03 | 0.74 | 2.11E-06 | 0.82 | 1.52 | 6.53E-06 | 0.79 | 1.44 | 284071 | 94651 | 639822 | 472513 | 634047 | 481902 | 645598 | 467757 |
| HILIC_POS_64_0167a4_326 | HILIC | POS | 4.326 | 64.0167 | | | | 4.35E-01 | 0.14 | 0.98 | 9.04E-01 | -0.03 | 0.82 | 5.40E-01 | 0.13 | 0.33 | 4.69E-01 | 0.15 | 0.66 | 247559902 | 45598300 | 255443196 | 59901286 | 256173649 | 61238780 | 254712743 | 59146250 |
| HILIC_NEG_566_34772a1_07 | HILIC | NEG | 1.07 | 566.3477 | | | | 2.83E-05 | 0.78 | 1.28 | 9.04E-01 | -0.02 | 0.65 | 6.62E-04 | 0.77 | 1.22 | 4.36E-05 | 0.79 | 1.62 | 469908 | 192770 | 657216 | 236972 | 660105 | 190192 | 654328 | 277971 |
| RP_POS_410_32654a9_934 | RP | POS | 9.93 | 410.3265 | | | | 6.54E-02 | 0.28 | 1.12 | 9.04E-01 | -0.03 | 0.36 | 1.23E-01 | 0.27 | 0.68 | 1.45E-01 | 0.30 | 1.22 | 647365 | 597316 | 930800 | 1094663 | 944137 | 1214905 | 917462 | 971943 |
| HILIC_POS_364_77948a3_008 | HILIC | POS | 3.008 | 364.7795 | | | | 4.49E-05 | -0.54 | 1.36 | 9.04E-01 | 0.03 | 0.77 | 1.74E-03 | -0.53 | 1.47 | 2.66E-03 | -0.55 | 1.50 | 1102724 | 168531 | 856333 | 501489 | 850224 | 530550 | 862441 | 475978 |
| RP_NEG_192_92888a15_361 | RP | NEG | 15.36 | 192.9289 | | | | 6.51E-01 | 0.09 | 0.58 | 9.04E-01 | 0.03 | 0.37 | 6.51E-01 | 0.10 | 0.42 | 7.31E-01 | 0.07 | 0.37 | 3671795 | 1149312 | 3781535 | 1298151 | 3765732 | 1284874 | 3797337 | 1324151 |
| RP_POS_199_13049a13_242 | RP | POS | 13.24 | 199.1305 | | | | 7.11E-01 | 0.09 | 0.33 | 9.04E-01 | -0.02 | 0.25 | 7.61E-01 | 0.08 | 0.20 | 6.99E-01 | 0.10 | 0.56 | 26867959 | 5075527 | 27231855 | 3798100 | 27277941 | 3908901 | 27185769 | 3723128 |
| HILIC_NEG_566_34814a1_193 | HILIC | NEG | 1.193 | 566.3481 | | | | 2.26E-05 | 0.80 | 1.29 | 9.04E-01 | 0.02 | 0.63 | 3.69E-04 | 0.81 | 1.26 | 5.15E-05 | 0.79 | 1.61 | 3103342 | 1190679 | 4269609 | 1429658 | 4252256 | 1133988 | 4286962 | 1686123 |
| RP_POS_310_31015a11_247 | RP | POS | 11.25 | 310.3102 | | | | 1.53E-11 | 0.97 | 1.50 | 9.04E-01 | 0.02 | 0.82 | 3.23E-06 | 0.98 | 1.50 | 2.57E-10 | 0.95 | 1.90 | 146059397 | 63887519 | 282847841 | 143937542 | 281103536 | 105174581 | 284592146 | 175473493 |
| RP_POS_352_24551a10_98 | RP | POS | 10.98 | 352.2455 | | | | 1.69E-01 | 0.27 | 1.15 | 9.05E-01 | 0.02 | 1.41 | 1.93E-01 | 0.29 | 1.00 | 2.49E-01 | 0.26 | 1.07 | 1847317 | 414370 | 1966283 | 438092 | 1960995 | 457563 | 1971572 | 422310 |
| RP_POS_177_09097a6_349 | RP | POS | 6.35 | 177.0910 | | | | 1.82E-01 | -0.23 | 0.76 | 9.05E-01 | -0.03 | 0.82 | 2.40E-01 | -0.24 | 0.59 | 2.82E-01 | -0.21 | 0.79 | 1025699 | 252203 | 948026 | 365997 | 952434 | 360021 | 943617 | 375482 |
| HILIC_POS_87_09226a0_546 | HILIC | POS | 0.546 | 87.0923 | | | | 1.15E-03 | -0.59 | 1.02 | 9.05E-01 | -0.02 | 0.71 | 4.73E-03 | -0.60 | 1.03 | 4.48E-03 | -0.58 | 1.01 | 4429409 | 947865 | 3724298 | 1225282 | 3739048 | 1171397 | 3709548 | 1288665 |
| HILIC_NEG_130_98364a7_352 | HILIC | NEG | 7.352 | 130.9836 | | | | 8.23E-04 | -0.59 | 1.12 | 9.05E-01 | -0.02 | 0.69 | 2.07E-03 | -0.60 | 1.14 | 6.50E-03 | -0.58 | 1.03 | 2306433 | 154476 | 2186319 | 208481 | 2188814 | 226385 | 2183824 | 191188 |
| RP_POS_278_24539a11_166 | RP | POS | 11.17 | 278.2454 | | | | 4.73E-05 | 0.73 | 1.14 | 9.06E-01 | 0.02 | 0.31 | 3.07E-04 | 0.74 | 1.27 | 4.57E-04 | 0.72 | 1.30 | 1309998 | 270752 | 1569451 | 357026 | 1565184 | 358423 | 1573718 | 359210 |
| HILIC_POS_163_12299a1_306 | HILIC | POS | 1.306 | 163.1230 | | | | 1.45E-04 | 0.54 | 1.08 | 9.06E-01 | 0.03 | 0.73 | 3.42E-03 | 0.56 | 1.09 | 2.46E-03 | 0.53 | 1.01 | 949657 | 858351 | 1913658 | 1928826 | 1890606 | 1831548 | 1936711 | 2039857 |
| RP_NEG_437_29175a9_933 | RP | NEG | 9.93 | 437.2918 | | | | 7.21E-02 | 0.28 | 1.05 | 9.06E-01 | -0.03 | 0.36 | 1.33E-01 | 0.27 | 0.63 | 1.48E-01 | 0.29 | 1.23 | 2103521 | 1951089 | 2984810 | 3402109 | 3025411 | 3736024 | 2944210 | 3069799 |
| RP_NEG_268_98416a8_483 | RP | NEG | 8.48 | 268.9842 | | | | 9.73E-01 | -0.01 | 0.76 | 9.06E-01 | -0.02 | 0.29 | 9.36E-01 | -0.02 | 0.99 | 9.83E-01 | 0.00 | 0.27 | 2435183 | 165574 | 2434071 | 157921 | 2435954 | 158128 | 2432188 | 159296 |
| RP_POS_886_75903a0_497 | RP | POS | 0.50 | 886.7590 | | | | 3.10E-06 | -0.58 | 1.31 | 9.06E-01 | 0.03 | 1.20 | 6.26E-04 | -0.56 | 1.38 | 9.97E-04 | -0.59 | 1.38 | 4499220 | 421140 | 3411170 | 2094216 | 3386199 | 2199878 | 3436140 | 2005057 |
| HILIC_POS_183_11253a0_753 | HILIC | POS | 0.753 | 183.1125 | | | | 1.76E-03 | 0.51 | 1.04 | 9.06E-01 | 0.03 | 0.64 | 1.08E-02 | 0.52 | 1.06 | 7.55E-03 | 0.50 | 0.92 | 2144717 | 740359 | 2716910 | 1194476 | 2702708 | 1102186 | 2731112 | 1291292 |
| HILIC_POS_203_10271a7_689 | HILIC | POS | 7.689 | 203.1027 | | | | 2.70E-01 | 0.20 | 0.62 | 9.06E-01 | 0.02 | 0.52 | 3.29E-01 | 0.22 | 0.50 | 3.46E-01 | 0.19 | 0.51 | 657036 | 51777 | 669375 | 62760 | 668629 | 57337 | 670121 | 68333 |
| HILIC_NEG_406_62949a2_947 | HILIC | NEG | 2.947 | 406.6295 | | | | 2.72E-05 | -0.57 | 1.32 | 9.06E-01 | 0.03 | 0.76 | 9.44E-04 | -0.56 | 1.44 | 1.98E-03 | -0.58 | 1.45 | 424091 | 67030 | 330072 | 180526 | 327926 | 193614 | 332218 | 168372 |
| RP_POS_186_11221a0_704 | RP | POS | 0.70 | 186.1122 | | | | 7.65E-03 | 0.51 | 0.78 | 9.06E-01 | -0.02 | 0.12 | 1.99E-02 | 0.49 | 0.93 | 1.72E-02 | 0.52 | 0.88 | 1163468 | 492730 | 1452110 | 579434 | 1458980 | 597216 | 1445241 | 567077 |
| RP_POS_524_2691a6_992 | RP | POS | 6.99 | 524.2691 | | | | 2.01E-02 | -0.40 | 0.86 | 9.06E-01 | -0.03 | 0.75 | 3.29E-02 | -0.41 | 0.84 | 6.69E-02 | -0.39 | 0.85 | 778172 | 46891 | 752832 | 67097 | 753627 | 72770 | 752036 | 61636 |
| RP_NEG_197_06781a0_709 | RP | NEG | 0.71 | 197.0678 | | | | 1.17E-02 | -0.51 | 1.08 | 9.06E-01 | 0.02 | 0.35 | 4.08E-02 | -0.49 | 1.05 | 1.12E-02 | -0.52 | 1.15 | 709737 | 499728 | 441876 | 523944 | 435666 | 392866 | 448087 | 632625 |
| HILIC_NEG_150_88956a2_989 | HILIC | NEG | 2.989 | 150.8896 | | | | 8.61E-01 | -0.03 | 0.87 | 9.07E-01 | 0.02 | 0.56 | 9.21E-01 | -0.02 | 0.55 | 8.32E-01 | -0.05 | 0.67 | 786414 | 282823 | 776061 | 308450 | 772411 | 302740 | 779710 | 317090 |
| RP_POS_223_09392a13_632 | RP | POS | 13.63 | 223.0939 | | | | 1.19E-01 | 0.32 | 0.78 | 9.07E-01 | 0.02 | 0.15 | 1.38E-01 | 0.33 | 0.65 | 1.83E-01 | 0.31 | 0.58 | 12866275 | 7243561 | 15199465 | 7229429 | 15113957 | 7592940 | 15284973 | 6923024 |
| RP_POS_424_2818a11_127 | RP | POS | 11.13 | 424.2818 | | | | 1.16E-01 | 0.31 | 0.68 | 9.07E-01 | -0.02 | 0.24 | 1.82E-01 | 0.30 | 0.59 | 1.46E-01 | 0.32 | 0.59 | 1080383 | 1993897 | 1740498 | 2168917 | 1766151 | 2154026 | 1714846 | 2205276 |
| RP_POS_173_02884a15_646 | RP | POS | 15.65 | 173.0288 | | | | 6.15E-01 | -0.11 | 0.34 | 9.07E-01 | 0.02 | 0.11 | 6.78E-01 | -0.10 | 0.22 | 6.19E-01 | -0.12 | 0.46 | 95323810 | 37374892 | 91552492 | 34259518 | 91147591 | 36089295 | 91957392 | 32689611 |
| RP_POS_216_95068a0_453 | RP | POS | 0.45 | 216.9507 | | | | 1.73E-04 | -0.54 | 1.20 | 9.07E-01 | -0.03 | 1.07 | 1.79E-03 | -0.55 | 1.22 | 5.40E-03 | -0.53 | 1.27 | 1370573 | 333690 | 1002545 | 742107 | 1011254 | 781006 | 993835 | 708915 |
| RP_POS_148_97328a15_454 | RP | POS | 15.45 | 148.9733 | | | | 6.39E-02 | -0.34 | 0.63 | 9.07E-01 | 0.02 | 0.32 | 1.43E-01 | -0.33 | 0.57 | 7.52E-02 | -0.35 | 0.76 | 3115446 | 543309 | 2893779 | 678563 | 2885822 | 588936 | 2901736 | 763753 |
| RP_POS_149_02361a9_441 | RP | POS | 9.44 | 149.0236 | Unknown | 149.0228(100), 121.0287(8) | 4 | 5.89E-01 | 0.10 | 0.48 | 9.08E-01 | 0.02 | 0.73 | 6.13E-01 | 0.11 | 0.33 | 6.68E-01 | 0.09 | 0.52 | 3824037 | 216923 | 3849449 | 268457 | 3846306 | 246153 | 3852592 | 291544 |
| RP_POS_539_27667a10_326 | RP | POS | 10.33 | 539.2767 | | | | 9.15E-08 | 1.01 | 1.61 | 9.08E-01 | 0.02 | 0.40 | 4.54E-07 | 1.02 | 1.74 | 3.92E-06 | 0.99 | 1.59 | 307505 | 183307 | 544973 | 222579 | 542385 | 243399 | 547561 | 202081 |
| RP_POS_368_27951a8_181 | RP | POS | 8.18 | 368.2795 | | | | 3.46E-02 | 0.35 | 1.00 | 9.08E-01 | 0.02 | 0.99 | 6.94E-02 | 0.36 | 0.85 | 8.77E-02 | 0.33 | 1.02 | 969277 | 419836 | 1182410 | 658141 | 1174769 | 656430 | 1190051 | 666422 |
| HILIC_POS_199_99051a4_327 | HILIC | POS | 4.327 | 199.9905 | | | | 2.00E-01 | 0.22 | 0.99 | 9.08E-01 | -0.02 | 0.84 | 2.98E-01 | 0.21 | 0.52 | 2.61E-01 | 0.23 | 0.80 | 3344872 | 637345 | 3533307 | 922951 | 3544022 | 956235 | 3522593 | 898022 |
| RP_POS_249_18294a8_904 | RP | POS | 8.90 | 249.1829 | | | | 8.08E-01 | 0.04 | 0.42 | 9.09E-01 | -0.02 | 0.39 | 8.78E-01 | 0.03 | 0.32 | 7.92E-01 | 0.06 | 0.41 | 1972868 | 827637 | 2016615 | 1043028 | 2028687 | 1069752 | 2004543 | 1026328 |
| RP_POS_163_97708a0_677 | RP | POS | 0.68 | 163.9771 | | | | 4.76E-06 | 0.63 | 1.28 | 9.09E-01 | -0.03 | 1.15 | 4.95E-04 | 0.62 | 1.32 | 4.09E-04 | 0.64 | 1.36 | 4145148 | 708220 | 5191098 | 1791946 | 5211832 | 1826618 | 5170364 | 1774897 |
| RP_POS_232_92776a0_453 | RP | POS | 0.45 | 232.9278 | | | | 4.50E-04 | -0.53 | 1.18 | 9.09E-01 | 0.02 | 1.07 | 4.74E-03 | -0.51 | 1.19 | 4.87E-03 | -0.54 | 1.25 | 5113572 | 1440792 | 3741207 | 2801594 | 3708798 | 2912776 | 3773617 | 2715075 |
| HILIC_NEG_144_92345a4_164 | HILIC | NEG | 4.164 | 144.9235 | | | | 1.12E-01 | -0.33 | 0.73 | 9.09E-01 | -0.02 | 0.80 | 1.45E-01 | -0.34 | 0.56 | 1.56E-01 | -0.32 | 0.53 | 43023111 | 9370794 | 39967845 | 9075675 | 40072676 | 8617286 | 39863015 | 9598786 |
| RP_POS_359_20868a11_379 | RP | POS | 11.38 | 359.2087 | | | | 1.41E-03 | -0.56 | 1.10 | 9.09E-01 | -0.02 | 0.94 | 2.27E-03 | -0.57 | 1.17 | 1.29E-02 | -0.54 | 1.05 | 1034397 | 134495 | 933859 | 187421 | 936023 | 215757 | 931695 | 156237 |
| HILIC_NEG_132_92368a2_946 | HILIC | NEG | 2.946 | 132.9237 | | | | 5.85E-02 | -0.41 | 1.02 | 9.09E-01 | -0.02 | 0.73 | 8.06E-02 | -0.42 | 0.82 | 8.81E-02 | -0.39 | 0.66 | 25439289 | 5990855 | 23125131 | 5533129 | 23188787 | 5308723 | 23061475 | 5802158 |
| RP_POS_281_17184a9_303 | RP | POS | 9.30 | 281.1718 | | | | 1.31E-05 | -0.65 | 1.15 | 9.09E-01 | 0.02 | 0.98 | 8.69E-04 | -0.63 | 1.22 | 2.74E-04 | -0.66 | 1.24 | 3779797 | 382465 | 3310000 | 774871 | 3301104 | 747701 | 3318896 | 808631 |
| HILIC_POS_440_70877a3_013 | HILIC | POS | 3.013 | 440.7088 | | | | 2.05E-05 | -0.61 | 1.33 | 9.09E-01 | 0.02 | 0.76 | 7.40E-04 | -0.59 | 1.45 | 1.02E-03 | -0.62 | 1.43 | 386671 | 76026 | 289461 | 172930 | 287476 | 181318 | 291446 | 165939 |
| HILIC_POS_338_23248a0_759 | HILIC | POS | 0.759 | 338.2325 | | | | 4.39E-01 | -0.15 | 0.44 | 9.09E-01 | -0.02 | 0.49 | 4.88E-01 | -0.16 | 0.29 | 5.07E-01 | -0.14 | 0.31 | 582312 | 277553 | 536743 | 319005 | 540402 | 277166 | 533085 | 358827 |
| HILIC_NEG_115_92065a1_049 | HILIC | NEG | 1.049 | 115.9207 | | | | 1.03E-01 | 0.31 | 0.91 | 9.10E-01 | 0.02 | 0.50 | 1.33E-01 | 0.32 | 0.67 | 1.73E-01 | 0.30 | 0.57 | 28335843 | 7801372 | 31065098 | 9050898 | 30961475 | 9315375 | 31168721 | 8871933 |
| HILIC_POS_356_00937a7_391 | HILIC | POS | 7.391 | 356.0094 | | | | 8.23E-01 | 0.04 | 0.43 | 9.10E-01 | 0.02 | 0.44 | 8.00E-01 | 0.05 | 0.48 | 8.90E-01 | 0.03 | 0.24 | 1067381 | 41807 | 1069394 | 50492 | 1068817 | 51593 | 1069972 | 49884 |
| RP_NEG_764_80872a0_496 | RP | NEG | 0.50 | 764.8087 | | | | 6.27E-06 | -0.57 | 1.28 | 9.10E-01 | -0.03 | 1.19 | 5.87E-04 | -0.58 | 1.36 | 1.60E-03 | -0.56 | 1.34 | 1841602 | 182800 | 1470486 | 717678 | 1478674 | 740243 | 1462298 | 701812 |
| HILIC_NEG_185_96243a0_699 | HILIC | NEG | 0.699 | 185.9624 | | | | 4.93E-05 | 0.78 | 1.30 | 9.10E-01 | -0.02 | 0.83 | 2.62E-04 | 0.77 | 1.48 | 2.64E-04 | 0.79 | 1.31 | 722935 | 387015 | 1079983 | 443825 | 1085039 | 460736 | 1074927 | 430872 |
| HILIC_NEG_454_77625a2_923 | HILIC | NEG | 2.923 | 454.7763 | | | | 6.71E-07 | -0.62 | 1.35 | 9.10E-01 | 0.03 | 0.76 | 1.76E-04 | -0.61 | 1.49 | 5.50E-04 | -0.63 | 1.52 | 1285180 | 117070 | 990387 | 522945 | 984431 | 561034 | 996342 | 487537 |
| RP_POS_916_52686a13_165 | RP | POS | 13.17 | 916.5269 | | | | 9.40E-01 | 0.02 | 0.55 | 9.10E-01 | -0.02 | 0.30 | 9.83E-01 | 0.01 | 0.57 | 9.08E-01 | 0.03 | 0.44 | 943533 | 571044 | 952057 | 512358 | 957870 | 496412 | 946245 | 532808 |
| RP_POS_279_1088a8_874 | RP | POS | 8.87 | 279.1088 | | | | 8.20E-01 | 0.05 | 0.45 | 9.11E-01 | -0.02 | 0.20 | 8.80E-01 | 0.03 | 0.30 | 7.97E-01 | 0.06 | 0.33 | 1025323 | 179473 | 1033730 | 185193 | 1035827 | 180791 | 1031633 | 191307 |
| RP_NEG_179_05704a3_266 | RP | NEG | 3.27 | 179.0570 | | | | 7.51E-01 | -0.06 | 0.38 | 9.11E-01 | -0.02 | 0.09 | 7.45E-01 | -0.07 | 0.25 | 8.20E-01 | -0.05 | 0.54 | 1505484 | 1485771 | 1405280 | 1738184 | 1424902 | 1672082 | 1385658 | 1818676 |
| RP_POS_235_092a0_629 | RP | POS | 0.63 | 235.0920 | | | | 3.20E-05 | -0.59 | 1.24 | 9.11E-01 | 0.02 | 1.11 | 1.04E-03 | -0.58 | 1.29 | 1.32E-03 | -0.60 | 1.30 | 9991588 | 2245965 | 7178917 | 5150132 | 7120830 | 5382015 | 7237005 | 4961456 |
| HILIC_NEG_195_81091a3_955 | HILIC | NEG | 3.955 | 195.8109 | | | | 1.53E-01 | 0.22 | 0.82 | 9.11E-01 | -0.02 | 0.45 | 3.25E-01 | 0.20 | 0.37 | 1.71E-01 | 0.23 | 0.95 | 364687 | 416723 | 519607 | 785291 | 528463 | 656284 | 510752 | 902818 |
| RP_NEG_872_565a13_73 | RP | NEG | 13.73 | 872.5650 | | | | 3.83E-04 | 0.66 | 1.15 | 9.11E-01 | 0.02 | 0.38 | 1.71E-03 | 0.67 | 1.15 | 1.76E-03 | 0.65 | 1.11 | 7726445 | 3099200 | 10230862 | 3817891 | 10187940 | 3709829 | 10273784 | 3960235 |
| HILIC_POS_162_14861a1_047 | HILIC | POS | 1.047 | 162.1486 | | | | 6.54E-04 | -0.66 | 1.27 | 9.12E-01 | 0.02 | 0.73 | 2.26E-03 | -0.64 | 1.30 | 2.42E-03 | -0.67 | 1.24 | 727385 | 278420 | 516289 | 319238 | 512719 | 335501 | 519859 | 305481 |
| HILIC_NEG_103_03988a0_928 | HILIC | NEG | 0.928 | 103.0399 | | | | 2.21E-06 | 0.66 | 1.14 | 9.12E-01 | -0.02 | 1.26 | 1.44E-03 | 0.65 | 1.02 | 1.08E-05 | 0.68 | 1.43 | 16897165 | 9488159 | 30872499 | 22554166 | 31124064 | 17791731 | 30620934 | 26666222 |
| RP_POS_215_96272a15_558 | RP | POS | 15.56 | 215.9627 | | | | 3.99E-01 | -0.17 | 0.41 | 9.12E-01 | -0.02 | 0.16 | 4.29E-01 | -0.18 | 0.38 | 4.78E-01 | -0.16 | 0.32 | 4799796 | 128314 | 4777352 | 135148 | 4778859 | 131492 | 4775845 | 140029 |
| RP_NEG_1003_67517a10_932 | RP | NEG | 10.93 | 1003.6752 | | | | 3.59E-01 | 0.17 | 1.00 | 9.12E-01 | -0.02 | 0.98 | 4.89E-01 | 0.16 | 0.69 | 3.58E-01 | 0.18 | 0.87 | 862158 | 336105 | 929305 | 417596 | 933949 | 352880 | 924660 | 477246 |
| RP_POS_531_47534a15_099 | RP | POS | 15.10 | 531.4753 | | | | 9.69E-01 | -0.01 | 0.37 | 9.12E-01 | 0.02 | 0.07 | 9.85E-01 | 0.00 | 0.39 | 9.31E-01 | -0.02 | 0.40 | 11120962 | 1002008 | 11112819 | 1152409 | 11100051 | 1156189 | 11125587 | 1160212 |
| RP_POS_528_30829a10_551 | RP | POS | 10.55 | 528.3083 | | | | 2.88E-06 | 0.84 | 1.56 | 9.12E-01 | 0.02 | 0.12 | 6.47E-05 | 0.85 | 1.51 | 2.10E-05 | 0.83 | 1.54 | 1759594 | 973702 | 2854697 | 1282635 | 2840485 | 1160897 | 2868908 | 1405625 |
| HILIC_NEG_219_84549a3_021 | HILIC | NEG | 3.021 | 219.8455 | | | | 5.26E-02 | -0.38 | 1.04 | 9.13E-01 | 0.02 | 0.77 | 9.61E-02 | -0.37 | 0.74 | 7.42E-02 | -0.39 | 0.66 | 1053628 | 205857 | 968927 | 224847 | 966439 | 222425 | 971414 | 229476 |
| RP_NEG_564_83026a0_448 | RP | NEG | 0.45 | 564.8303 | | | | 2.82E-02 | -0.38 | 0.98 | 9.13E-01 | 0.02 | 0.95 | 6.60E-02 | -0.37 | 0.92 | 5.85E-02 | -0.39 | 1.02 | 1267723 | 461735 | 1033592 | 654358 | 1026378 | 676287 | 1040806 | 638465 |
| RP_POS_320_23502a10_849 | RP | POS | 10.85 | 320.2350 | | | | 1.23E-06 | 0.80 | 1.25 | 9.13E-01 | 0.02 | 0.13 | 1.12E-04 | 0.81 | 1.24 | 1.14E-05 | 0.79 | 1.51 | 18439480 | 5267396 | 24979006 | 8346969 | 24886989 | 7154979 | 25071022 | 9463333 |
| RP_NEG_452_92499a13_622 | RP | NEG | 13.62 | 452.9250 | | | | 1.57E-01 | 0.27 | 0.49 | 9.13E-01 | -0.02 | 0.33 | 2.12E-01 | 0.26 | 0.53 | 2.15E-01 | 0.28 | 0.66 | 1489572 | 578953 | 1665087 | 673321 | 1672496 | 740642 | 1657678 | 606062 |
| RP_POS_348_202a6_254 | RP | POS | 6.25 | 348.2020 | | | | 5.61E-01 | -0.12 | 0.39 | 9.13E-01 | -0.02 | 0.27 | 5.55E-01 | -0.13 | 0.38 | 6.40E-01 | -0.11 | 0.28 | 573880 | 72009 | 565453 | 67095 | 566190 | 72556 | 564715 | 61886 |
| HILIC_POS_227_17558a0_505 | HILIC | POS | 0.505 | 227.1756 | | | | 1.06E-04 | -0.68 | 1.23 | 9.13E-01 | 0.02 | 0.72 | 6.87E-04 | -0.66 | 1.35 | 9.92E-04 | -0.69 | 1.29 | 20361493 | 6734193 | 14139079 | 9414757 | 14035595 | 10013654 | 14242563 | 8876323 |
| HILIC_POS_618_66107a3_005 | HILIC | POS | 3.005 | 618.6611 | | | | 3.88E-05 | -0.56 | 1.35 | 9.13E-01 | -0.02 | 0.76 | 8.73E-04 | -0.57 | 1.47 | 2.98E-03 | -0.55 | 1.44 | 456606 | 81004 | 347399 | 212227 | 349725 | 223253 | 345073 | 202848 |
| RP_NEG_128_95856a0_499 | RP | NEG | 0.50 | 128.9586 | | | | 4.10E-05 | -0.52 | 1.26 | 9.13E-01 | 0.02 | 1.21 | 1.84E-03 | -0.50 | 1.31 | 3.92E-03 | -0.53 | 1.32 | 2291985 | 256785 | 1809095 | 1041019 | 1797687 | 1116886 | 1820503 | 970493 |
| RP_NEG_395_19012a11_987 | RP | NEG | 11.99 | 395.1901 | | | | 5.41E-02 | 0.36 | 0.73 | 9.13E-01 | 0.02 | 0.47 | 7.25E-02 | 0.37 | 0.69 | 1.11E-01 | 0.35 | 0.76 | 91437 | 168714 | 161928 | 199613 | 159742 | 213263 | 164113 | 187118 |

**Supplementary table 1: Metabolomics results** (in asceding order according to p-value from progressive ALD vs  non-progressive ALD comparison, with molecular features with ID_level of 1-2 first)

| | | | | | | | | all_ALD_vs_control | | | Prog_ALD_vs_non-prog_ALD | | | Prog_ALD_vs_control_ALD | | | Non-prog_ALD_vs_control | | | Healthy_control | | all_ALD | | Non-prog_ALD | | Progressive_ALD | |
|---|---|---|---|---|---|---|---|---|---|---|---|---|---|---|---|---|---|---|---|---|---|---|---|---|---|---|---|
| | | | | | | | | t-test | Cohen's | PLS-DA | t-test | Cohen's | PLS-DA | t-test | Cohen's | PLS-DA | t-test | Cohen's | PLS-DA | | | | | | | | |
| Feature_ID | RP_HILIC | POS_NEG | Average.Rt | Average.Mz | Metabolite.name | curated_MSMS | ID_level | p | d | VIP | p | d | VIP | p | d | VIP | p | d | VIP | Mean | SD | Mean | SD | Mean | SD | Mean | SD |
| HILIC_POS_344_72208a3_019 | HILIC | POS | 3.019 | 344.7221 | | | | 1.37E-04 | -0.53 | 1.33 | 9.14E-01 | -0.02 | 0.76 | 1.65E-03 | -0.54 | 1.43 | 5.95E-03 | -0.52 | 1.44 | 254416 | 47751 | 197237 | 118203 | 198530 | 125723 | 195944 | 111443 |
| RP_NEG_840_7829a0_496 | RP | NEG | 0.50 | 840.7829 | | | | 1.10E-06 | -0.61 | 1.31 | 9.14E-01 | 0.02 | 1.20 | 3.02E-04 | -0.59 | 1.40 | 6.21E-04 | -0.62 | 1.38 | 5042641 | 493159 | 3779994 | 2300729 | 3754942 | 2431292 | 3805046 | 2186840 |
| RP_POS_162_96411a11_247 | RP | POS | 11.25 | 162.9641 | | | | 1.02E-11 | 0.98 | 1.51 | 9.14E-01 | 0.02 | 0.80 | 2.60E-06 | 0.99 | 1.51 | 1.64E-10 | 0.97 | 1.92 | 875846 | 393320 | 1723010 | 878814 | 1713439 | 641046 | 1732580 | 1072038 |
| HILIC_NEG_482_55823a2_946 | HILIC | NEG | 2.946 | 482.5582 | | | | 2.83E-05 | -0.58 | 1.30 | 9.14E-01 | -0.02 | 0.75 | 4.99E-04 | -0.59 | 1.43 | 2.66E-03 | -0.57 | 1.42 | 303366 | 52034 | 234667 | 128379 | 236064 | 137988 | 233270 | 119391 |
| HILIC_NEG_234_82014a4_68 | HILIC | NEG | 4.68 | 234.8201 | | | | 3.49E-01 | 0.17 | 0.53 | 9.14E-01 | 0.02 | 0.41 | 3.74E-01 | 0.19 | 0.35 | 4.55E-01 | 0.16 | 0.75 | 675330 | 301212 | 736084 | 361100 | 732157 | 379798 | 740010 | 345208 |
| RP_POS_185_11507a12_894 | RP | POS | 12.89 | 185.1151 | | | | 6.90E-01 | -0.08 | 0.34 | 9.15E-01 | 0.02 | 0.28 | 7.77E-01 | -0.07 | 0.18 | 6.63E-01 | -0.09 | 0.40 | 6333405 | 2669203 | 6111305 | 2906438 | 6079884 | 2370535 | 6142726 | 3383151 |
| RP_NEG_348_89621a0_499 | RP | NEG | 0.50 | 348.8962 | | | | 7.48E-05 | -0.51 | 1.25 | 9.15E-01 | -0.02 | 1.22 | 1.45E-03 | -0.52 | 1.32 | 6.64E-03 | -0.50 | 1.30 | 6579386 | 960131 | 5086414 | 3230227 | 5121307 | 3464381 | 5051522 | 3012849 |
| RP_POS_468_29883a10_454 | RP | POS | 10.45 | 468.2988 | | | | 8.84E-01 | 0.03 | 0.72 | 9.15E-01 | 0.02 | 0.18 | 8.57E-01 | 0.04 | 0.69 | 9.44E-01 | 0.01 | 0.54 | 664846 | 160434 | 670034 | 211124 | 667754 | 211129 | 672313 | 213238 |
| HILIC_NEG_134_89478a7_391 | HILIC | NEG | 7.391 | 134.8948 | | | | 8.09E-01 | 0.05 | 0.33 | 9.15E-01 | 0.02 | 1.15 | 7.94E-01 | 0.06 | 0.10 | 8.62E-01 | 0.04 | 0.63 | 11674559 | 520025 | 11699939 | 498772 | 11694554 | 487246 | 11705323 | 514934 |
| HILIC_NEG_136_89168a0_936 | HILIC | NEG | 0.936 | 136.8917 | | | | 3.87E-02 | 0.38 | 1.21 | 9.15E-01 | -0.02 | 0.79 | 6.73E-02 | 0.37 | 0.91 | 7.59E-02 | 0.39 | 1.09 | 9757555 | 1958501 | 10651019 | 2434900 | 10677229 | 2659750 | 10624809 | 2214227 |
| HILIC_POS_228_02203a4_865 | HILIC | POS | 4.865 | 228.0220 | | | | 9.60E-01 | 0.01 | 0.63 | 9.15E-01 | -0.02 | 0.22 | 9.97E-01 | 0.00 | 0.57 | 9.33E-01 | 0.02 | 0.29 | 613445 | 382247 | 617178 | 319343 | 620610 | 367884 | 613746 | 265856 |
| RP_POS_685_43457a13_57 | RP | POS | 13.57 | 685.4346 | | | | 2.56E-01 | 0.21 | 0.93 | 9.15E-01 | -0.02 | 0.22 | 3.27E-01 | 0.19 | 0.65 | 3.25E-01 | 0.22 | 0.70 | 97539709 | 6060561 | 99053686 | 7716602 | 99136288 | 8512604 | 98971084 | 6915835 |
| RP_POS_441_29599a14_018 | RP | POS | 14.02 | 441.2960 | | | | 5.44E-01 | 0.11 | 0.55 | 9.15E-01 | -0.02 | 0.33 | 6.30E-01 | 0.10 | 0.46 | 5.71E-01 | 0.12 | 0.38 | 13883652 | 5871416 | 14654986 | 7245710 | 14732454 | 7563741 | 14577518 | 6989251 |
| HILIC_NEG_115_92067a7_422 | HILIC | NEG | 7.422 | 115.9207 | | | | 8.98E-01 | -0.03 | 0.39 | 9.16E-01 | 0.02 | 0.42 | 9.40E-01 | -0.02 | 0.42 | 8.72E-01 | -0.04 | 0.21 | 53441552 | 8744769 | 53220737 | 7264275 | 53143136 | 7032095 | 53298339 | 7560004 |
| RP_POS_229_18031a7_809 | RP | POS | 7.81 | 229.1803 | | | | 6.83E-01 | 0.09 | 0.42 | 9.16E-01 | -0.02 | 0.46 | 7.44E-01 | 0.08 | 0.48 | 6.73E-01 | 0.10 | 0.43 | 1406630 | 196882 | 1422604 | 171944 | 1424437 | 165364 | 1420771 | 179947 |
| HILIC_POS_246_10959a5_463 | HILIC | POS | 5.463 | 246.1096 | | | | 3.75E-01 | -0.16 | 0.44 | 9.16E-01 | 0.02 | 0.32 | 4.57E-01 | -0.15 | 0.58 | 4.35E-01 | -0.17 | 0.29 | 1492682 | 54597 | 1481974 | 71089 | 1481220 | 77599 | 1482729 | 64712 |
| HILIC_POS_394_31653a7_325 | HILIC | POS | 7.325 | 394.3165 | | | | 9.45E-01 | -0.01 | 0.54 | 9.16E-01 | 0.02 | 0.30 | 9.92E-01 | 0.00 | 0.41 | 9.14E-01 | -0.02 | 0.23 | 3059577 | 509518 | 3052238 | 573681 | 3046155 | 605484 | 3058320 | 546123 |
| HILIC_NEG_215_06737a6_522 | HILIC | NEG | 6.522 | 215.0674 | | | | 4.34E-05 | -0.57 | 1.10 | 9.16E-01 | -0.02 | 0.68 | 2.93E-03 | -0.58 | 1.14 | 6.27E-04 | -0.56 | 1.21 | 3258186 | 186546 | 3022410 | 447300 | 3027146 | 393714 | 3017674 | 499181 |
| RP_POS_264_95483a0_454 | RP | POS | 0.45 | 264.9548 | | | | 1.77E-04 | -0.55 | 1.19 | 9.17E-01 | 0.02 | 1.08 | 2.70E-03 | -0.53 | 1.23 | 3.38E-03 | -0.56 | 1.26 | 730971 | 184830 | 532665 | 393141 | 528534 | 412018 | 536795 | 377466 |
| RP_POS_177_08653a0_63 | RP | POS | 0.63 | 177.0865 | | | | 1.72E-06 | -0.70 | 1.33 | 9.17E-01 | 0.02 | 1.12 | 1.38E-04 | -0.69 | 1.40 | 1.70E-04 | -0.71 | 1.37 | 3252912 | 736423 | 2223710 | 1567573 | 2207261 | 1623007 | 2240159 | 1526438 |
| RP_NEG_128_03394a1_128 | RP | NEG | 1.13 | 128.0339 | | | | 1.55E-09 | 0.82 | 1.40 | 9.17E-01 | 0.02 | 0.88 | 1.73E-05 | 0.84 | 1.43 | 6.61E-07 | 0.81 | 1.56 | 1104775 | 190439 | 1499752 | 504182 | 1494481 | 445517 | 1505022 | 561288 |
| RP_POS_271_90927a15_67 | RP | POS | 15.67 | 271.9093 | | | | 2.02E-01 | -0.23 | 1.00 | 9.18E-01 | 0.02 | 0.49 | 3.23E-01 | -0.21 | 1.10 | 2.27E-01 | -0.24 | 0.64 | 4338566 | 1380497 | 3944015 | 1851795 | 3924689 | 1673266 | 3963341 | 2031672 |
| RP_NEG_434_87195a0_449 | RP | NEG | 0.45 | 434.8720 | | | | 3.77E-04 | -0.53 | 1.17 | 9.18E-01 | 0.02 | 1.08 | 4.55E-03 | -0.52 | 1.19 | 4.38E-03 | -0.54 | 1.23 | 3731206 | 1017331 | 2735305 | 2028962 | 2714142 | 2091400 | 2756468 | 1985579 |
| HILIC_POS_160_03464a4_868 | HILIC | POS | 4.868 | 160.0346 | | | | 7.87E-01 | 0.05 | 0.33 | 9.18E-01 | 0.02 | 0.22 | 7.45E-01 | 0.07 | 0.49 | 8.59E-01 | 0.04 | 0.09 | 7458395 | 5293226 | 7751816 | 5372825 | 7695994 | 6710079 | 7807637 | 3645834 |
| RP_NEG_888_50806a10_328 | RP | NEG | 10.33 | 888.5081 | | | | 7.32E-01 | -0.08 | 0.36 | 9.18E-01 | 0.02 | 0.26 | 7.83E-01 | -0.07 | 0.25 | 7.11E-01 | -0.09 | 0.35 | 842545 | 1088060 | 771045 | 767855 | 763089 | 644424 | 779002 | 880809 |
| RP_POS_800_07922a13_711 | RP | POS | 13.71 | 800.0792 | | | | 1.95E-03 | -0.59 | 1.00 | 9.19E-01 | -0.02 | 0.59 | 4.57E-03 | -0.60 | 1.07 | 7.88E-03 | -0.58 | 0.94 | 1766689 | 675936 | 1301921 | 794674 | 1310094 | 827431 | 1293748 | 768846 |
| HILIC_NEG_202_92915a7_353 | HILIC | NEG | 7.353 | 202.9292 | | | | 1.74E-02 | -0.41 | 0.87 | 9.19E-01 | -0.02 | 0.75 | 4.80E-02 | -0.42 | 0.85 | 3.75E-02 | -0.40 | 1.01 | 621254 | 49290 | 594120 | 69685 | 594836 | 63147 | 593403 | 76305 |
| HILIC_NEG_144_92336a3_015 | HILIC | NEG | 3.015 | 144.9234 | | | | 5.61E-01 | -0.16 | 0.93 | 9.19E-01 | 0.02 | 0.48 | 5.90E-01 | -0.15 | 0.55 | 5.62E-01 | -0.17 | 0.50 | 10757231 | 2166260 | 10522393 | 1147583 | 10510601 | 1258550 | 10534185 | 1037563 |
| HILIC_NEG_187_9781a0_93 | HILIC | NEG | 0.93 | 187.9781 | | | | 2.72E-08 | 0.79 | 1.32 | 9.19E-01 | -0.02 | 1.17 | 8.58E-05 | 0.78 | 1.26 | 1.19E-06 | 0.80 | 1.57 | 459018 | 256401 | 902939 | 591898 | 909007 | 510829 | 896872 | 668442 |
| HILIC_NEG_241_01044a0_577 | HILIC | NEG | 0.577 | 241.0104 | | | | 4.86E-02 | -0.55 | 1.36 | 9.19E-01 | -0.02 | 0.70 | 5.05E-02 | -0.56 | 0.90 | 6.28E-02 | -0.54 | 1.02 | 939203 | 356700 | 805044 | 186180 | 806946 | 202912 | 803142 | 169861 |
| HILIC_POS_144_17468a0_542 | HILIC | POS | 0.542 | 144.1747 | | | | 7.99E-05 | -0.65 | 1.38 | 9.19E-01 | -0.02 | 0.61 | 7.93E-04 | -0.66 | 1.16 | 1.05E-03 | -0.64 | 1.08 | 1539833 | 142983 | 1399424 | 224922 | 1401716 | 224681 | 1397132 | 227421 |
| HILIC_POS_272_78702a3_016 | HILIC | POS | 3.016 | 272.7870 | | | | 3.36E-05 | -0.55 | 1.35 | 9.20E-01 | 0.02 | 0.76 | 1.30E-03 | -0.54 | 1.47 | 2.30E-03 | -0.56 | 1.48 | 1260010 | 199515 | 970588 | 573607 | 964753 | 608331 | 976423 | 542797 |
| RP_POS_166_98494a10_261 | RP | POS | 10.26 | 166.9849 | | | | 7.17E-01 | 0.08 | 0.63 | 9.20E-01 | 0.02 | 0.09 | 7.24E-01 | 0.09 | 0.39 | 7.63E-01 | 0.07 | 0.52 | 10741652 | 4048730 | 11036988 | 3792131 | 10998537 | 3227196 | 11075439 | 4316965 |
| RP_NEG_267_16104a8_332 | RP | NEG | 8.33 | 267.1610 | | | | 8.04E-02 | -0.31 | 0.59 | 9.20E-01 | -0.02 | 0.17 | 1.20E-01 | -0.32 | 0.60 | 1.50E-01 | -0.30 | 0.52 | 1339093 | 78246 | 1308253 | 105328 | 1309317 | 106610 | 1307190 | 105102 |
| RP_POS_185_58588a0_622 | RP | POS | 0.62 | 185.5859 | | | | 2.05E-04 | -0.53 | 1.19 | 9.20E-01 | 0.02 | 1.08 | 3.10E-03 | -0.52 | 1.21 | 4.23E-03 | -0.54 | 1.25 | 1577023 | 349683 | 1193743 | 785820 | 1185815 | 827627 | 1201671 | 750023 |
| HILIC_POS_188_12811a4_027 | HILIC | POS | 4.027 | 188.1281 | | | | 2.50E-01 | 0.20 | 0.46 | 9.21E-01 | -0.02 | 0.29 | 2.85E-01 | 0.19 | 0.43 | 3.50E-01 | 0.22 | 0.32 | 1884698 | 1008653 | 2142689 | 1328303 | 2156034 | 1604833 | 2129344 | 994452 |
| RP_NEG_203_00168a4_112 | RP | NEG | 4.11 | 203.0017 | | | | 9.62E-01 | -0.01 | 0.60 | 9.21E-01 | -0.02 | 0.46 | 9.37E-01 | -0.02 | 0.52 | 9.98E-01 | 0.00 | 0.29 | 3689667 | 5427244 | 3638675 | 4859945 | 3687444 | 3939192 | 3589905 | 5674334 |
| HILIC_NEG_78_91827a1_363 | HILIC | NEG | 1.363 | 78.9183 | | | | 1.44E-05 | 0.58 | 1.03 | 9.21E-01 | 0.02 | 0.69 | 3.05E-03 | 0.60 | 0.93 | 1.51E-04 | 0.57 | 1.19 | 1468739 | 415965 | 2084503 | 1150042 | 2072995 | 933664 | 2096011 | 1341710 |
| HILIC_NEG_614_66974a2_927 | HILIC | NEG | 2.927 | 614.6697 | | | | 1.45E-05 | -0.57 | 1.33 | 9.21E-01 | 0.02 | 0.76 | 7.56E-04 | -0.56 | 1.46 | 1.59E-03 | -0.59 | 1.48 | 218395 | 32357 | 168082 | 95842 | 167125 | 102115 | 169039 | 90164 |
| RP_POS_249_18303a8_812 | RP | POS | 8.81 | 249.1830 | Matrin | 249.1816(100) | 3 | 9.76E-01 | 0.01 | 0.37 | 9.21E-01 | -0.02 | 0.41 | 9.82E-01 | 0.00 | 0.30 | 9.41E-01 | 0.02 | 0.32 | 1414639 | 588187 | 1418405 | 701862 | 1425411 | 713069 | 1411399 | 697643 |
| HILIC_NEG_622_70093a2_921 | HILIC | NEG | 2.921 | 622.7009 | | | | 5.78E-07 | -0.64 | 1.35 | 9.23E-01 | 0.02 | 0.76 | 1.40E-04 | -0.63 | 1.51 | 4.10E-04 | -0.65 | 1.52 | 542716 | 61632 | 410043 | 227306 | 407823 | 242520 | 412263 | 213458 |
| RP_POS_362_88556a0_456 | RP | POS | 0.46 | 362.8856 | | | | 6.64E-02 | -0.33 | 0.93 | 9.23E-01 | -0.02 | 0.89 | 9.61E-02 | -0.34 | 0.85 | 1.33E-01 | -0.32 | 0.95 | 582061 | 214898 | 494005 | 280018 | 496737 | 292175 | 491272 | 270256 |
| HILIC_POS_118_12289a1_071 | HILIC | POS | 1.071 | 118.1229 | | | | 4.18E-02 | -0.48 | 1.04 | 9.23E-01 | 0.02 | 0.65 | 5.75E-02 | -0.47 | 0.96 | 5.66E-02 | -0.49 | 0.87 | 773991 | 497792 | 572628 | 384345 | 568884 | 410133 | 576372 | 360858 |
| RP_POS_443_33423a13_24 | RP | POS | 13.24 | 443.3342 | | | | 1.38E-01 | -0.32 | 0.58 | 9.23E-01 | -0.02 | 0.32 | 1.50E-01 | -0.32 | 0.63 | 2.06E-01 | -0.31 | 0.57 | 5226796 | 2021102 | 4617240 | 1890349 | 4635650 | 2081197 | 4598830 | 1699322 |
| HILIC_POS_145_13358a2_668 | HILIC | POS | 2.668 | 145.1336 | | | | 6.35E-05 | 0.49 | 0.83 | 9.23E-01 | 0.02 | 0.98 | 1.12E-02 | 0.50 | 0.78 | 4.92E-04 | 0.48 | 1.18 | 327912 | 389757 | 1176625 | 1927625 | 1157920 | 1513955 | 1195331 | 2283535 |
| RP_POS_145_06491a6_163 | RP | POS | 6.16 | 145.0649 | | | | 6.29E-01 | -0.10 | 0.30 | 9.24E-01 | 0.02 | 0.26 | 7.08E-01 | -0.09 | 0.26 | 6.28E-01 | -0.10 | 0.19 | 1154076 | 179686 | 1136014 | 193663 | 1134162 | 182160 | 1137866 | 206365 |
| RP_POS_600_46826a10_547 | RP | POS | 10.55 | 600.4683 | | | | 1.57E-01 | 0.36 | 0.97 | 9.25E-01 | 0.02 | 0.36 | 1.69E-01 | 0.37 | 0.79 | 1.89E-01 | 0.35 | 0.91 | 1200092 | 360896 | 1298107 | 231481 | 1295913 | 233946 | 1300301 | 231343 |
| RP_NEG_220_814a0_757 | RP | NEG | 0.76 | 220.8140 | | | | 1.62E-04 | -0.80 | 1.52 | 9.25E-01 | 0.02 | 0.13 | 3.42E-04 | -0.79 | 1.48 | 6.83E-04 | -0.81 | 1.31 | 6000700 | 1614690 | 4676773 | 1550021 | 4662100 | 1736248 | 4691445 | 1356159 |
| HILIC_NEG_634_7251a2_91 | HILIC | NEG | 2.91 | 634.7251 | | | | 6.04E-07 | -0.63 | 1.35 | 9.25E-01 | 0.02 | 0.77 | 1.63E-04 | -0.62 | 1.51 | 4.66E-04 | -0.64 | 1.52 | 883329 | 90233 | 671856 | 369101 | 668371 | 393301 | 675342 | 347190 |
| RP_POS_159_96886a0_254 | RP | POS | 0.25 | 159.9689 | | | | 7.89E-01 | -0.06 | 0.36 | 9.25E-01 | -0.02 | 0.25 | 7.79E-01 | -0.07 | 0.24 | 8.41E-01 | -0.05 | 0.39 | 2493696 | 271065 | 2479010 | 262203 | 2481481 | 261511 | 2476538 | 265526 |
| HILIC_NEG_468_80127a2_911 | HILIC | NEG | 2.911 | 468.8013 | | | | 5.39E-05 | -0.58 | 1.37 | 9.25E-01 | 0.02 | 0.77 | 1.08E-03 | -0.57 | 1.50 | 2.02E-03 | -0.59 | 1.49 | 252377 | 49502 | 193634 | 109334 | 192603 | 116504 | 194664 | 102841 |
| RP_NEG_251_82169a15_599 | RP | NEG | 15.60 | 251.8217 | | | | 2.29E-01 | -0.25 | 0.72 | 9.26E-01 | -0.02 | 0.25 | 2.65E-01 | -0.26 | 0.62 | 2.87E-01 | -0.24 | 0.52 | 1657642 | 62731 | 1642287 | 59851 | 1642849 | 57847 | 1641724 | 62373 |
| HILIC_NEG_484_55756a2_946 | HILIC | NEG | 2.946 | 484.5576 | | | | 1.12E-05 | -0.64 | 1.32 | 9.26E-01 | -0.02 | 0.75 | 2.09E-04 | -0.65 | 1.44 | 1.14E-03 | -0.63 | 1.43 | 202613 | 39775 | 151644 | 85716 | 152449 | 92107 | 150839 | 79746 |
| RP_POS_352_24548a10_568 | RP | POS | 10.57 | 352.2455 | | | | 9.98E-02 | -0.33 | 0.58 | 9.26E-01 | -0.02 | 0.24 | 1.45E-01 | -0.34 | 0.61 | 1.34E-01 | -0.32 | 0.64 | 1819647 | 447605 | 1666532 | 457693 | 1670827 | 407655 | 1662238 | 506973 |
| HILIC_POS_139_12311a3_027 | HILIC | POS | 3.027 | 139.1231 | | | | 7.36E-03 | 0.41 | 1.01 | 9.26E-01 | -0.02 | 0.74 | 2.31E-02 | 0.40 | 0.84 | 3.56E-02 | 0.42 | 0.96 | 10275192 | 1008571 | 10975361 | 1833500 | 10992543 | 2007762 | 10958180 | 1661429 |
| RP_NEG_369_20526a10_703 | RP | NEG | 10.70 | 369.2053 | | | | 1.08E-07 | 0.72 | 1.58 | 9.26E-01 | -0.02 | 0.50 | 6.30E-05 | 0.71 | 1.46 | 5.80E-05 | 0.73 | 1.58 | 371711 | 200910 | 744589 | 558977 | 749793 | 569070 | 739385 | 554429 |
| RP_NEG_338_87787a15_473 | RP | NEG | 15.47 | 338.8779 | | | | 4.93E-01 | 0.14 | 0.33 | 9.27E-01 | 0.02 | 0.37 | 5.02E-01 | 0.15 | 0.30 | 5.77E-01 | 0.13 | 0.30 | 5537264 | 462830 | 5602406 | 471067 | 5598035 | 503230 | 5606777 | 441652 |
| RP_NEG_285_20847a9_593 | RP | NEG | 9.59 | 285.2085 | FA 16:1;2O | 285.2063(100), 223.2058(60), 267.1978(45) | 3 | 7.94E-02 | 0.33 | 0.68 | 9.27E-01 | 0.02 | 0.31 | 1.22E-01 | 0.34 | 0.63 | 1.26E-01 | 0.32 | 0.63 | 2339593 | 328543 | 2464097 | 386933 | 2460515 | 369665 | 2467678 | 407200 |
| RP_POS_777_54163a13_156 | RP | POS | 13.16 | 777.5416 | | | | 8.99E-01 | 0.03 | 0.19 | 9.27E-01 | -0.02 | 0.33 | 9.42E-01 | 0.02 | 0.29 | 8.72E-01 | 0.04 | 0.14 | 958262 | 362697 | 967485 | 337930 | 970613 | 293524 | 964357 | 380194 |
| RP_POS_485_80945a0_407 | RP | POS | 0.41 | 485.8095 | | | | 5.17E-01 | -0.15 | 0.55 | 9.27E-01 | -0.02 | 0.33 | 5.28E-01 | -0.16 | 0.34 | 5.64E-01 | -0.15 | 0.84 | 616149 | 732889 | 524361 | 544388 | 529368 | 526932 | 519354 | 566618 |
| HILIC_NEG_136_89166a1_69 | HILIC | NEG | 1.69 | 136.8917 | | | | 2.26E-02 | -0.43 | 0.67 | 9.28E-01 | 0.02 | 0.51 | 4.64E-02 | -0.42 | 0.75 | 4.34E-02 | -0.44 | 0.75 | 6876242 | 2441011 | 5662718 | 2879978 | 5636393 | 2982834 | 5689043 | 2803409 |
| HILIC_NEG_195_81113a3_033 | HILIC | NEG | 3.033 | 195.8111 | | | | 5.97E-02 | 0.28 | 1.02 | 9.28E-01 | 0.02 | 0.80 | 1.13E-01 | 0.29 | 0.72 | 1.54E-01 | 0.27 | 0.83 | 112650547 | 16705212 | 121088513 | 33070517 | 120787952 | 34108956 | 121389073 | 32342427 |
| RP_POS_332_28049a9_906 | RP | POS | 9.91 | 332.2805 | | | | 8.90E-01 | -0.03 | 0.88 | 9.28E-01 | -0.02 | 0.15 | 8.68E-01 | -0.04 | 0.53 | 9.37E-01 | -0.02 | 0.60 | 640866 | 193127 | 635335 | 208098 | 637222 | 217149 | 633449 | 200829 |
| RP_POS_465_37732a11_158 | RP | POS | 11.16 | 465.3773 | | | | 9.25E-01 | 0.02 | 0.74 | 9.28E-01 | -0.02 | 0.19 | 9.66E-01 | 0.01 | 0.53 | 9.01E-01 | 0.03 | 0.59 | 1333390 | 405902 | 1341288 | 426865 | 1345157 | 429990 | 1337419 | 428048 |
| RP_POS_199_13048a13_807 | RP | POS | 13.81 | 199.1305 | | | | 3.90E-01 | 0.16 | 0.73 | 9.28E-01 | -0.02 | 0.83 | 4.91E-01 | 0.15 | 0.31 | 4.08E-01 | 0.17 | 0.61 | 27705291 | 12973782 | 30073324 | 14892280 | 30208086 | 13775594 | 29938561 | 16071156 |
| RP_POS_223_09421a11_209 | RP | POS | 11.21 | 223.0942 | | | | 7.05E-01 | -0.08 | 0.33 | 9.29E-01 | -0.02 | 0.43 | 6.91E-01 | -0.09 | 0.38 | 7.74E-01 | -0.07 | 0.47 | 8534452 | 2993355 | 8303601 | 2968541 | 8330437 | 3312696 | 8276764 | 2613215 |
| HILIC_NEG_158_97836a7_353 | HILIC | NEG | 7.353 | 158.9784 | | | | 9.43E-05 | -0.63 | 1.18 | 9.29E-01 | 0.02 | 0.78 | 1.15E-03 | -0.62 | 1.24 | 1.22E-03 | -0.64 | 1.24 | 20084260 | 980751 | 19109590 | 1633785 | 19094860 | 1686588 | 19124319 | 1596234 |
| RP_POS_513_84296a11_952 | RP | POS | 11.95 | 513.8430 | | | | 9.25E-01 | 0.02 | 0.34 | 9.29E-01 | 0.02 | 0.30 | 8.98E-01 | 0.03 | 0.29 | 9.70E-01 | 0.01 | 0.31 | 209619 | 398138 | 217531 | 462849 | 213366 | 488523 | 221695 | 440613 |
| RP_POS_167_97151a0_253 | RP | POS | 0.25 | 167.9715 | | | | 6.47E-01 | -0.09 | 0.41 | 9.29E-01 | -0.02 | 0.26 | 6.42E-01 | -0.10 | 0.34 | 7.21E-01 | -0.08 | 0.35 | 2797953 | 1671496 | 2639977 | 1738377 | 2655570 | 1883979 | 2624384 | 1598658 |
| RP_POS_640_81689a0_499 | RP | POS | 0.50 | 640.8169 | | | | 1.86E-08 | -0.69 | 1.37 | 9.29E-01 | 0.02 | 1.19 | 2.80E-05 | -0.68 | 1.50 | 1.15E-04 | -0.70 | 1.44 | 2125859 | 150903 | 1574888 | 871080 | 1567076 | 929614 | 1582699 | 817764 |
| RP_POS_296_25836a9_592 | RP | POS | 9.59 | 296.2584 | Hexadecanedioic acid [M+Na]+ | 296.2584(30), 81.0705(100), 261.2233(70) | 3 | 5.76E-01 | -0.15 | 0.71 | 9.29E-01 | 0.01 | 0.54 | 6.24E-01 | -0.15 | 0.61 | 5.57E-01 | -0.16 | 0.61 | 4957836 | 8048303 | 4117730 | 4430437 | 4077995 | 3031888 | 4157465 | 5519291 |
| RP_NEG_238_84509a15_583 | RP | NEG | 15.58 | 238.8451 | | | | 2.43E-01 | 0.22 | 0.83 | 9.29E-01 | 0.02 | 0.33 | 2.94E-01 | 0.23 | 0.69 | 3.13E-01 | 0.22 | 0.83 | 469433 | 28178 | 476416 | 31904 | 476131 | 30524 | 476700 | 33536 |
| HILIC_NEG_209_83401a3_059 | HILIC | NEG | 3.059 | 209.8340 | | | | 4.27E-03 | 0.37 | 1.25 | 9.30E-01 | 0.02 | 0.75 | 1.65E-02 | 0.38 | 1.34 | 5.74E-02 | 0.36 | 1.40 | 1280241 | 71659 | 1359989 | 243162 | 1357829 | 268543 | 1362148 | 217571 |
| RP_NEG_353_16406a11_162 | RP | NEG | 11.16 | 353.1641 | | | | 3.73E-02 | 0.36 | 1.11 | 9.30E-01 | -0.02 | 1.04 | 7.94E-02 | 0.35 | 1.00 | 7.49E-02 | 0.37 | 1.03 | 916913 | 71276 | 951234 | 101600 | 952132 | 105405 | 950336 | 98712 |
| HILIC_NEG_199_80508a3_033 | HILIC | NEG | 3.033 | 199.8051 | | | | 2.44E-02 | 0.40 | 1.07 | 9.30E-01 | -0.02 | 0.80 | 4.77E-02 | 0.39 | 0.80 | 5.79E-02 | 0.41 | 0.93 | 71432490 | 13257171 | 78200772 | 17768126 | 78357273 | 19307890 | 78044270 | 16279157 |
| HILIC_NEG_253_79361a3_137 | HILIC | NEG | 3.137 | 253.7936 | | | | 2.21E-05 | 0.52 | 1.25 | 9.30E-01 | -0.02 | 0.74 | 2.53E-03 | 0.51 | 1.39 | 2.38E-03 | 0.53 | 1.39 | 314489 | 159721 | 750374 | 943830 | 758683 | 963981 | 742066 | 932959 |
| RP_NEG_253_14417a7_723 | RP | NEG | 7.72 | 253.1442 | | | | 1.75E-01 | -0.23 | 0.65 | 9.31E-01 | -0.02 | 0.21 | 2.04E-01 | -0.24 | 0.59 | 2.95E-01 | -0.23 | 0.54 | 12634647 | 470048 | 12489493 | 658170 | 12495265 | 726527 | 12483721 | 589327 |
| RP_POS_282_27792a10_848 | RP | POS | 10.85 | 282.2779 | | | | 4.75E-06 | 0.76 | 1.20 | 9.31E-01 | 0.02 | 0.69 | 3.58E-04 | 0.77 | 1.24 | 1.91E-05 | 0.75 | 1.48 | 1217452071 | 366404542 | ######### | 570076061 | 1635207843 | 457580860 | 1645199927 | 668711170 |
| RP_POS_234_96066a0_453 | RP | POS | 0.45 | 234.9607 | | | | 7.56E-05 | -0.56 | 1.22 | 9.31E-01 | -0.02 | 1.08 | 1.04E-03 | -0.57 | 1.25 | 3.42E-03 | -0.55 | 1.28 | 2065303 | 456426 | 1527249 | 1033716 | 1536303 | 1095045 | 1518194 | 979620 |
| RP_POS_846_52124a12_756 | RP | POS | 12.76 | 846.5212 | | | | 5.60E-03 | 0.52 | 0.93 | 9.31E-01 | -0.02 | 0.34 | 1.48E-02 | 0.51 | 0.90 | 1.44E-02 | 0.53 | 0.90 | 1257682 | 830384 | 1765730 | 988226 | 1774385 | 1024751 | 1757074 | 960648 |
| RP_POS_122_92456a0_499 | RP | POS | 0.50 | 122.9246 | | | | 5.66E-05 | -0.51 | 1.26 | 9.31E-01 | -0.02 | 1.21 | 1.42E-03 | -0.52 | 1.34 | 6.31E-03 | -0.50 | 1.32 | 8531691 | 1053614 | 6657102 | 4094370 | 6692943 | 4392582 | 6621260 | 3817390 |
| RP_POS_253_14149a8_847 | RP | POS | 8.85 | 253.1415 | | | | 6.60E-01 | 0.08 | 0.29 | 9.31E-01 | 0.02 | 0.43 | 6.71E-01 | 0.09 | 0.41 | 7.41E-01 | 0.07 | 0.59 | 514501 | 268625 | 540544 | 350616 | 537481 | 357613 | 543608 | 347081 |
| RP_POS_166_98502a11_345 | RP | POS | 11.35 | 166.9850 | | | | 1.92E-01 | 0.26 | 0.58 | 9.31E-01 | 0.02 | 0.24 | 2.13E-01 | 0.27 | 0.62 | 2.73E-01 | 0.26 | 0.61 | 13147278 | 5387565 | 14602832 | 5530036 | 14554617 | 5984703 | 14651047 | 5095616 |
| HILIC_NEG_134_89471a5_381 | HILIC | NEG | 5.381 | 134.8947 | | | | 6.63E-01 | 0.09 | 0.83 | 9.31E-01 | -0.02 | 0.54 | 7.24E-01 | 0.08 | 0.48 | 6.68E-01 | 0.10 | 0.32 | 14355448 | 6322766 | 14917655 | 6289938 | 14972481 | 6336914 | 14862829 | 6306461 |
| HILIC_POS_332_24283a1_204 | HILIC | POS | 1.204 | 332.2428 | | | | 4.38E-01 | 0.14 | 0.63 | 9.31E-01 | -0.02 | 0.71 | 5.52E-01 | 0.13 | 0.30 | 4.52E-01 | 0.15 | 0.41 | 3178081 | 1639675 | 3456110 | 2078613 | 3474223 | 1859945 | 3437996 | 2295511 |
| RP_POS_554_46216a13_931 | RP | POS | 13.93 | 554.4622 | | | | 2.36E-01 | -0.25 | 0.52 | 9.31E-01 | 0.02 | 0.55 | 2.78E-01 | -0.25 | 0.46 | 2.83E-01 | -0.26 | 0.62 | 4733514 | 1384423 | 4402681 | 1270757 | 4391624 | 1415885 | 4413738 | 1121437 |
| RP_NEG_339_20209a11_182 | RP | NEG | 11.18 | 339.2021 | | | | 6.06E-01 | 0.10 | 0.49 | 9.31E-01 | 0.02 | 0.19 | 6.19E-01 | 0.11 | 0.36 | 6.71E-01 | 0.10 | 0.69 | 889242 | 419696 | 933748 | 430678 | 930009 | 427004 | 937486 | 438625 |
| RP_POS_369_33496a11_038 | RP | POS | 11.04 | 369.3350 | | | | 1.47E-02 | -0.48 | 1.06 | 9.31E-01 | 0.02 | 0.89 | 2.28E-02 | -0.48 | 1.05 | 3.49E-02 | -0.49 | 0.99 | 681279 | 149662 | 603378 | 160192 | 601988 | 182036 | 604768 | 136768 |

**Supplementary table 1: Metabolomics results** (in asceding order according to p-value from progressive ALD vs non-progressive ALD comparison, with molecular features with ID_level of 1-2 first)

| Feature_ID | RP_HILIC | POS_NEG | Average.Rt | Average.Mz | Metabolite.name | curated_MSMS | ID_level | all_ALD_vs_control | | | Prog_ALD_vs_non-prog_ALD | | | Prog_ALD_vs_control_ALD | | | Non-prog_ALD_vs_control | | | Healthy_control | | all_ALD | | Non-prog_ALD | | Progressive_ALD | |
|---|---|---|---|---|---|---|---|---|---|---|---|---|---|---|---|---|---|---|---|---|---|---|---|---|---|---|---|
| | | | | | | | | t-test | Cohen's | PLS-DA | t-test | Cohen's | PLS-DA | t-test | Cohen's | PLS-DA | t-test | Cohen's | PLS-DA | | | | | | | | |
| | | | | | | | | p | d | VIP | p | d | VIP | p | d | VIP | p | d | VIP | Mean | SD | Mean | SD | Mean | SD | Mean | SD |
| HILIC_POS_334_69373a3_023 | HILIC | POS | 3.023 | 334.6937 | | | | 7.29E-04 | -0.49 | 1.31 | 9.31E-01 | -0.02 | 0.75 | 5.00E-03 | -0.49 | 1.39 | 1.20E-02 | -0.48 | 1.41 | 463668 | 102616 | 363091 | 225885 | 365051 | 238101 | 361132 | 215382 |
| RP_POS_750_78333a0_498 | RP | POS | 0.50 | 750.7833 | | | | 3.87E-05 | -0.52 | 1.27 | 9.31E-01 | -0.02 | 1.21 | 1.32E-03 | -0.53 | 1.34 | 4.49E-03 | -0.51 | 1.33 | 6669530 | 854890 | 5162746 | 3194348 | 5190438 | 3372864 | 5135055 | 3039439 |
| HILIC_POS_114_0666a1_232 | HILIC | POS | 1.232 | 114.0666 | | | | 4.95E-02 | -0.39 | 1.02 | 9.32E-01 | -0.02 | 0.77 | 7.99E-02 | -0.40 | 0.64 | 7.93E-02 | -0.38 | 0.84 | 988070159 | 214702594 | 898908528 | 230417550 | 900888120 | 218269252 | 896928936 | 244169092 |
| RP_POS_250_86765a0_515 | RP | POS | 0.52 | 250.8677 | | | | 1.13E-05 | -0.69 | 1.24 | 9.32E-01 | -0.02 | 1.01 | 1.64E-04 | -0.70 | 1.31 | 5.04E-04 | -0.68 | 1.28 | 461544 | 117166 | 326471 | 204343 | 328223 | 214518 | 324719 | 195807 |
| RP_POS_475_41397a10_975 | RP | POS | 10.98 | 475.4140 | | | | 3.18E-02 | 0.38 | 1.17 | 9.33E-01 | 0.02 | 0.94 | 6.58E-02 | 0.39 | 1.10 | 6.24E-02 | 0.37 | 1.03 | 1517754 | 399128 | 1710656 | 525682 | 1706173 | 497463 | 1715139 | 557505 |
| RP_NEG_344_98062a8_89 | RP | NEG | 8.89 | 344.9806 | | | | 2.91E-01 | 0.22 | 0.60 | 9.33E-01 | -0.02 | 0.37 | 3.63E-01 | 0.21 | 0.55 | 3.23E-01 | 0.22 | 0.50 | 5759404 | 246417 | 5812921 | 248111 | 5815035 | 248040 | 5810806 | 250682 |
| RP_POS_464_37329a11_978 | RP | POS | 11.98 | 464.3733 | | | | 2.84E-01 | -0.18 | 1.02 | 9.33E-01 | 0.02 | 0.48 | 3.80E-01 | -0.17 | 0.58 | 3.49E-01 | -0.19 | 0.87 | 11221172 | 822624 | 11019780 | 1168025 | 11009849 | 1208810 | 11029710 | 1137974 |
| RP_POS_245_11447a8_597 | RP | POS | 8.60 | 245.1145 | Unknown | 245.1135(100) | 4 | 1.16E-03 | -0.56 | 0.97 | 9.33E-01 | -0.02 | 0.44 | 7.11E-03 | -0.56 | 0.98 | 4.30E-03 | -0.55 | 0.99 | 14461618 | 708799 | 13915125 | 1024528 | 13923825 | 941534 | 13906425 | 1110899 |
| RP_POS_160_0369a0_59 | RP | POS | 0.59 | 160.0369 | | | | 4.71E-02 | 0.29 | 0.79 | 9.33E-01 | 0.02 | 0.69 | 1.18E-01 | 0.30 | 0.87 | 1.10E-01 | 0.28 | 0.87 | 478464 | 241139 | 609434 | 493588 | 605266 | 466294 | 613602 | 524179 |
| RP_POS_189_08682a0_625 | RP | POS | 0.63 | 189.0868 | | | | 1.81E-05 | -0.67 | 1.29 | 9.33E-01 | -0.02 | 1.10 | 2.29E-04 | -0.67 | 1.39 | 8.29E-04 | -0.66 | 1.31 | 4626349 | 1268571 | 3180143 | 2289136 | 3199464 | 2428103 | 3160823 | 2165822 |
| RP_POS_318_24042a9_59 | RP | POS | 9.59 | 318.2404 | | 318.2414(100) | 4 | 5.39E-01 | -0.15 | 0.68 | 9.33E-01 | 0.02 | 0.59 | 6.09E-01 | -0.14 | 0.63 | 5.13E-01 | -0.16 | 0.60 | 4338626 | 5865935 | 3647792 | 4102956 | 3613143 | 2547534 | 3682440 | 5245920 |
| RP_NEG_352_85284a15_665 | RP | NEG | 15.67 | 352.8528 | | | | 3.21E-01 | -0.21 | 0.38 | 9.34E-01 | 0.02 | 0.24 | 3.91E-01 | -0.20 | 0.39 | 3.48E-01 | -0.22 | 0.38 | 12742543 | 3451926 | 12049008 | 3264507 | 12021575 | 3231906 | 12076442 | 3329377 |
| RP_POS_266_17236a11_133 | RP | POS | 11.13 | 266.1724 | | | | 2.55E-02 | 0.37 | 0.82 | 9.34E-01 | -0.02 | 0.34 | 5.18E-02 | 0.36 | 0.75 | 7.06E-02 | 0.38 | 0.76 | 1261222 | 713286 | 1642025 | 1100069 | 1651253 | 1212179 | 1632798 | 987648 |
| RP_NEG_473_28503a13_276 | RP | NEG | 13.28 | 473.2850 | | | | 8.99E-01 | 0.03 | 0.38 | 9.34E-01 | 0.02 | 0.17 | 8.80E-01 | 0.03 | 0.38 | 9.39E-01 | 0.02 | 0.33 | 132665 | 80230 | 134771 | 83413 | 134073 | 82981 | 135469 | 84679 |
| RP_NEG_325_27414a10_642 | RP | NEG | 10.64 | 325.2741 | | | | 2.30E-02 | -0.38 | 0.90 | 9.34E-01 | -0.02 | 0.79 | 4.15E-02 | -0.39 | 0.91 | 7.31E-02 | -0.37 | 0.87 | 705088 | 113474 | 643886 | 171712 | 645322 | 184460 | 642450 | 159819 |
| RP_POS_545_40173a10_933 | RP | POS | 10.93 | 545.4017 | | | | 4.96E-01 | -0.13 | 0.54 | 9.34E-01 | 0.02 | 0.57 | 5.80E-01 | -0.12 | 0.49 | 5.14E-01 | -0.14 | 0.64 | 19774666 | 2988905 | 19349847 | 3256056 | 19322701 | 3126622 | 19376993 | 3412169 |
| RP_POS_138_95358a0_253 | RP | POS | 0.25 | 138.9536 | | | | 2.78E-01 | 0.20 | 0.73 | 9.34E-01 | 0.02 | 0.20 | 3.19E-01 | 0.21 | 0.40 | 3.67E-01 | 0.20 | 0.75 | 1750912 | 583989 | 1887034 | 692210 | 1881276 | 706461 | 1892792 | 684787 |
| RP_NEG_187_94193a15_415 | RP | NEG | 15.42 | 187.9419 | | | | 4.66E-01 | -0.14 | 0.66 | 9.34E-01 | 0.02 | 0.07 | 5.33E-01 | -0.13 | 0.71 | 5.17E-01 | -0.15 | 0.35 | 3819038 | 452047 | 3748658 | 532581 | 3744228 | 582689 | 3753088 | 483225 |
| RP_NEG_152_88289a0_524 | RP | NEG | 0.52 | 152.8829 | | | | 3.49E-02 | 0.30 | 0.89 | 9.34E-01 | -0.02 | 0.93 | 1.22E-01 | 0.29 | 0.89 | 7.49E-02 | 0.31 | 0.89 | 2014818 | 566682 | 2355624 | 1243111 | 2365939 | 1178147 | 2345310 | 1316791 |
| HILIC_NEG_129_05566a0_83 | HILIC | NEG | 0.83 | 129.0557 | | | | 2.51E-01 | 0.19 | 0.92 | 9.34E-01 | 0.02 | 0.91 | 3.56E-01 | 0.20 | 0.39 | 3.07E-01 | 0.18 | 0.47 | 50953156 | 16153762 | 55271351 | 24097944 | 55071401 | 19884058 | 55471300 | 27889348 |
| RP_POS_163_07553a6_163 | RP | POS | 6.16 | 163.0755 | | | | 4.78E-01 | -0.14 | 0.42 | 9.34E-01 | 0.02 | 0.18 | 5.52E-01 | -0.14 | 0.38 | 5.01E-01 | -0.15 | 0.30 | 7354823 | 1067392 | 7199142 | 1093671 | 7190079 | 1087139 | 7208206 | 1111126 |
| HILIC_POS_227_12563a1_232 | HILIC | POS | 1.232 | 227.1256 | | | | 3.10E-02 | -0.42 | 1.00 | 9.35E-01 | 0.02 | 0.77 | 7.01E-02 | -0.41 | 0.66 | 4.38E-02 | -0.42 | 0.98 | 165088327 | 37893882 | 147503325 | 42922685 | 147148548 | 39616310 | 147858103 | 46396163 |
| HILIC_POS_302_19592a1_54 | HILIC | POS | 1.54 | 302.1959 | | | | 3.31E-05 | 0.56 | 0.95 | 9.35E-01 | 0.02 | 0.52 | 2.24E-03 | 0.57 | 1.04 | 1.13E-03 | 0.55 | 1.13 | 372006 | 244817 | 715443 | 670442 | 709912 | 632775 | 720973 | 712525 |
| HILIC_POS_170_15401a0_687 | HILIC | POS | 0.687 | 170.1540 | | | | 3.06E-01 | -0.20 | 0.91 | 9.35E-01 | -0.02 | 0.87 | 3.28E-01 | -0.20 | 0.46 | 4.06E-01 | -0.19 | 0.32 | 3481538 | 448469 | 3384254 | 508971 | 3388444 | 554128 | 3380065 | 465076 |
| HILIC_POS_554_39386a1_321 | HILIC | POS | 1.321 | 554.3939 | | | | 1.17E-02 | 0.40 | 1.02 | 9.35E-01 | -0.02 | 0.53 | 4.46E-02 | 0.39 | 0.72 | 3.16E-02 | 0.41 | 1.07 | 495716 | 119747 | 570771 | 200637 | 572417 | 197588 | 569124 | 205634 |
| HILIC_POS_200_06842a4_856 | HILIC | POS | 4.856 | 200.0684 | | | | 2.10E-01 | 0.22 | 0.79 | 9.35E-01 | 0.02 | 0.65 | 2.87E-01 | 0.23 | 0.38 | 2.81E-01 | 0.21 | 0.41 | 3707831 | 897477 | 3961839 | 1228147 | 3951766 | 1125693 | 3971913 | 1334197 |
| RP_POS_170_97743a15_392 | RP | POS | 15.39 | 170.9774 | | | | 4.11E-02 | 0.36 | 0.60 | 9.35E-01 | -0.02 | 0.42 | 8.99E-02 | 0.35 | 0.59 | 6.99E-02 | 0.37 | 0.66 | 5163868 | 2717306 | 6417393 | 3635758 | 6447116 | 3583931 | 6387669 | 3723021 |
| RP_NEG_347_79639a15_613 | RP | NEG | 15.61 | 347.7964 | | | | 3.07E-01 | -0.22 | 0.66 | 9.35E-01 | -0.02 | 0.52 | 3.31E-01 | -0.23 | 0.66 | 3.77E-01 | -0.21 | 0.72 | 847265 | 56821 | 835531 | 53049 | 835964 | 55049 | 835098 | 51527 |
| HILIC_NEG_136_89168a1_488 | HILIC | NEG | 1.488 | 136.8917 | | | | 2.09E-01 | 0.23 | 0.59 | 9.36E-01 | -0.02 | 0.90 | 3.26E-01 | 0.22 | 0.35 | 2.24E-01 | 0.24 | 0.51 | 5434425 | 1329221 | 5799781 | 1663230 | 5813337 | 1420071 | 5786225 | 1890011 |
| RP_POS_299_25797a10_25 | RP | POS | 10.25 | 299.2580 | | | | 6.93E-01 | 0.08 | 0.42 | 9.36E-01 | -0.02 | 0.16 | 7.54E-01 | 0.07 | 0.53 | 6.99E-01 | 0.09 | 0.56 | 3756704 | 578307 | 3804007 | 614982 | 3809013 | 620577 | 3799001 | 615596 |
| RP_POS_326_88602a0_498 | RP | POS | 0.50 | 326.8860 | | | | 5.91E-06 | -0.56 | 1.30 | 9.36E-01 | 0.02 | 1.20 | 6.54E-04 | -0.55 | 1.38 | 1.73E-03 | -0.57 | 1.37 | 14402177 | 1336862 | 11105265 | 6540565 | 11052025 | 6989573 | 11158504 | 6129527 |
| RP_POS_243_19559a8_339 | RP | POS | 8.34 | 243.1956 | | | | 7.73E-01 | 0.07 | 0.52 | 9.36E-01 | 0.02 | 0.43 | 7.71E-01 | 0.07 | 0.49 | 8.11E-01 | 0.06 | 0.35 | 1904149 | 247389 | 1918129 | 202676 | 1916485 | 188169 | 1919773 | 218129 |
| HILIC_POS_158_11752a3_102 | HILIC | POS | 3.102 | 158.1175 | | | | 8.28E-01 | 0.04 | 0.62 | 9.36E-01 | 0.02 | 0.45 | 8.24E-01 | 0.05 | 0.22 | 8.69E-01 | 0.04 | 0.59 | 7376692 | 8839754 | 7770490 | 8992590 | 7697542 | 8112111 | 7843437 | 9877575 |
| HILIC_POS_132_06575a5_65 | HILIC | POS | 5.65 | 132.0658 | | | | 5.33E-01 | 0.10 | 0.72 | 9.36E-01 | 0.02 | 0.70 | 5.96E-01 | 0.11 | 0.78 | 6.09E-01 | 0.09 | 0.41 | 4628137 | 1548961 | 4853781 | 2348061 | 4834828 | 2084875 | 4872733 | 2606120 |
| RP_POS_563_42761a11_158 | RP | POS | 11.16 | 563.4276 | | | | 9.82E-01 | 0.00 | 0.38 | 9.36E-01 | 0.02 | 0.58 | 9.52E-01 | 0.01 | 0.40 | 9.80E-01 | 0.00 | 0.37 | 550440 | 392218 | 552510 | 586812 | 547774 | 552344 | 557245 | 624975 |
| RP_POS_143_99864a15_465 | RP | POS | 15.47 | 143.9986 | | | | 3.38E-01 | -0.20 | 0.52 | 9.36E-01 | 0.02 | 0.30 | 4.08E-01 | -0.19 | 0.43 | 3.68E-01 | -0.21 | 0.68 | 2992321 | 868153 | 2823360 | 838059 | 2816599 | 836896 | 2830122 | 847663 |
| RP_NEG_526_29468a10_551 | RP | NEG | 10.55 | 526.2947 | | | | 1.70E-06 | 0.86 | 1.53 | 9.36E-01 | -0.02 | 0.23 | 5.49E-05 | 0.85 | 1.52 | 1.18E-05 | 0.86 | 1.54 | 1098452 | 584845 | 1776361 | 781848 | 1782659 | 727289 | 1770063 | 840248 |
| HILIC_NEG_173_96242a1_299 | HILIC | NEG | 1.299 | 173.9624 | | | | 7.21E-04 | 0.51 | 0.88 | 9.36E-01 | 0.02 | 0.83 | 2.05E-02 | 0.52 | 0.84 | 4.84E-04 | 0.50 | 1.45 | 1370265 | 171909 | 1527612 | 333830 | 1524929 | 208886 | 1530295 | 426041 |
| HILIC_NEG_334_69348a2_947 | HILIC | NEG | 2.947 | 334.6935 | | | | 7.37E-06 | -0.61 | 1.32 | 9.37E-01 | 0.02 | 0.76 | 4.16E-04 | -0.60 | 1.45 | 1.02E-03 | -0.61 | 1.47 | 1254191 | 184431 | 972866 | 505891 | 968838 | 539351 | 976893 | 475544 |
| RP_NEG_299_2597a10_775 | RP | NEG | 10.78 | 299.2597 | | | | 1.23E-01 | -0.28 | 0.85 | 9.37E-01 | 0.02 | 0.86 | 1.81E-01 | -0.27 | 0.69 | 1.86E-01 | -0.29 | 0.94 | 4964321 | 543547 | 4778446 | 703233 | 4772863 | 753455 | 4784028 | 656817 |
| HILIC_POS_126_09161a0_548 | HILIC | POS | 0.548 | 126.0916 | | | | 1.60E-01 | 0.16 | 0.68 | 9.37E-01 | -0.02 | 0.35 | 3.24E-01 | 0.15 | 0.58 | 3.23E-01 | 0.17 | 0.42 | 424071 | 335239 | 16760474 | 115391565 | 17675412 | 122116945 | 15845536 | 109489310 |
| RP_POS_313_27365a10_663 | RP | POS | 10.66 | 313.2737 | | | | 2.72E-01 | -0.22 | 0.58 | 9.37E-01 | 0.02 | 0.59 | 3.30E-01 | -0.21 | 0.58 | 3.21E-01 | -0.23 | 0.68 | 1591284 | 263212 | 1531707 | 268318 | 1529581 | 287299 | 1533834 | 250815 |
| HILIC_NEG_330_69919a2_946 | HILIC | NEG | 2.946 | 330.6992 | | | | 5.99E-06 | -0.60 | 1.33 | 9.37E-01 | 0.02 | 0.76 | 3.96E-04 | -0.59 | 1.47 | 1.10E-03 | -0.61 | 1.47 | 871617 | 118679 | 676499 | 356059 | 673682 | 381353 | 679316 | 332712 |
| HILIC_POS_346_7193a3_018 | HILIC | POS | 3.018 | 346.7193 | | | | 2.52E-04 | -0.53 | 1.32 | 9.37E-01 | -0.02 | 0.75 | 2.42E-03 | -0.54 | 1.40 | 5.99E-03 | -0.52 | 1.43 | 317240 | 65848 | 247760 | 142278 | 248885 | 149556 | 246635 | 136123 |
| RP_POS_626_29712a10_932 | RP | POS | 10.93 | 626.2971 | | | | 3.70E-04 | -0.82 | 1.42 | 9.38E-01 | -0.01 | 1.11 | 7.25E-04 | -0.83 | 1.53 | 8.23E-04 | -0.82 | 1.25 | 567711 | 224953 | 399369 | 180604 | 400795 | 179545 | 397943 | 183469 |
| RP_POS_806_05994a13_202 | RP | POS | 13.20 | 806.0599 | | | | 6.66E-04 | 0.69 | 1.16 | 9.38E-01 | 0.02 | 0.32 | 3.22E-03 | 0.70 | 1.13 | 1.11E-03 | 0.68 | 1.24 | 5250051 | 800951 | 5843155 | 831470 | 5836656 | 677404 | 5849654 | 968417 |
| HILIC_NEG_184_83141a2_949 | HILIC | NEG | 2.949 | 184.8314 | | | | 1.20E-05 | -0.56 | 1.33 | 9.38E-01 | 0.02 | 0.77 | 7.64E-04 | -0.55 | 1.45 | 2.16E-03 | -0.56 | 1.49 | 1827393 | 176423 | 1489156 | 673339 | 1483899 | 723564 | 1494414 | 626435 |
| RP_NEG_486_86917a0_498 | RP | NEG | 0.50 | 486.8692 | | | | 5.29E-06 | -0.57 | 1.30 | 9.39E-01 | -0.02 | 1.22 | 5.34E-04 | -0.58 | 1.38 | 1.67E-03 | -0.56 | 1.36 | 1945691 | 192688 | 1515406 | 837187 | 1521878 | 875010 | 1508934 | 806437 |
| RP_NEG_922_58124a13_876 | RP | NEG | 13.88 | 922.5812 | | | | 3.95E-01 | -0.19 | 0.54 | 9.39E-01 | 0.01 | 0.64 | 4.78E-01 | -0.18 | 0.55 | 3.90E-01 | -0.20 | 0.43 | 5714177 | 1403583 | 5477537 | 1204213 | 5468229 | 967134 | 5486844 | 1412206 |
| RP_POS_223_09438a13_807 | RP | POS | 13.81 | 223.0944 | | | | 4.78E-01 | 0.15 | 0.59 | 9.39E-01 | -0.02 | 0.23 | 5.59E-01 | 0.14 | 0.36 | 4.88E-01 | 0.15 | 0.38 | 13140349 | 5487383 | 13937375 | 5493083 | 13979804 | 5029498 | 13894945 | 5971950 |
| RP_POS_133_03452a15_301 | RP | POS | 15.30 | 133.0345 | | | | 5.43E-01 | -0.14 | 0.51 | 9.39E-01 | 0.01 | 0.71 | 5.95E-01 | -0.13 | 0.57 | 5.57E-01 | -0.14 | 0.33 | 114964928 | 17646093 | 112857433 | 14562842 | 112744960 | 14768053 | 112969905 | 14503779 |
| HILIC_NEG_434_71805a2_933 | HILIC | NEG | 2.933 | 434.7181 | | | | 2.54E-06 | -0.64 | 1.33 | 9.39E-01 | -0.02 | 0.76 | 1.04E-04 | -0.64 | 1.48 | 9.53E-04 | -0.63 | 1.47 | 188596 | 29232 | 141614 | 80150 | 142233 | 87322 | 140995 | 73165 |
| HILIC_NEG_624_69891a2_921 | HILIC | NEG | 2.921 | 624.6989 | | | | 1.47E-06 | -0.61 | 1.34 | 9.39E-01 | 0.02 | 0.76 | 2.40E-04 | -0.60 | 1.49 | 7.32E-04 | -0.62 | 1.51 | 437486 | 47875 | 334205 | 185958 | 332770 | 198680 | 335641 | 174324 |
| HILIC_POS_522_3562a1_113 | HILIC | POS | 1.113 | 522.3562 | | | | 7.83E-04 | 0.61 | 1.15 | 9.39E-01 | 0.02 | 0.71 | 5.66E-03 | 0.62 | 1.05 | 1.67E-03 | 0.60 | 1.28 | 16564837 | 8073811 | 22769754 | 10335637 | 22690057 | 8572743 | 22849451 | 11930047 |
| RP_POS_399_30847a10_651 | RP | POS | 10.65 | 399.3085 | | | | 9.96E-01 | 0.00 | 0.55 | 9.39E-01 | 0.02 | 0.32 | 9.70E-01 | 0.01 | 0.61 | 9.78E-01 | -0.01 | 0.53 | 32437716 | 3578338 | 32441204 | 3428286 | 32414888 | 3590378 | 32467519 | 3294529 |
| RP_POS_374_26251a11_167 | RP | POS | 11.17 | 374.2625 | | | | 1.50E-08 | 1.02 | 1.87 | 9.39E-01 | -0.01 | 0.85 | 2.70E-06 | 1.01 | 1.66 | 6.85E-08 | 1.02 | 1.99 | 369733 | 126466 | 549296 | 168636 | 550589 | 142093 | 548004 | 193036 |
| RP_NEG_333_87619a15_715 | RP | NEG | 15.72 | 333.8762 | | | | 8.57E-01 | -0.04 | 0.72 | 9.39E-01 | -0.02 | 0.47 | 8.49E-01 | -0.04 | 0.52 | 8.99E-01 | -0.03 | 0.43 | 762006 | 240190 | 753015 | 259131 | 754998 | 246992 | 751032 | 273230 |
| RP_POS_463_20038a11_254 | RP | POS | 11.25 | 463.2004 | | | | 6.47E-03 | 0.47 | 1.40 | 9.40E-01 | -0.02 | 0.82 | 2.87E-02 | 0.47 | 1.21 | 1.26E-02 | 0.48 | 1.45 | 566958 | 246234 | 722215 | 342063 | 724817 | 310227 | 719613 | 374363 |
| RP_POS_383_96194a0_638 | RP | POS | 0.64 | 383.9619 | | | | 6.65E-07 | -0.62 | 1.30 | 9.40E-01 | -0.02 | 1.16 | 1.15E-04 | -0.63 | 1.41 | 7.56E-04 | -0.61 | 1.35 | 1730065 | 148224 | 1364241 | 646798 | 1369153 | 691646 | 1359328 | 605647 |
| RP_POS_255_15912a7_727 | RP | POS | 7.73 | 255.1591 | | | | 7.24E-01 | -0.07 | 0.26 | 9.40E-01 | 0.02 | 0.19 | 7.93E-01 | -0.06 | 0.21 | 7.15E-01 | -0.08 | 0.41 | 29659518 | 1428287 | 29552881 | 1623483 | 29540627 | 1442428 | 29565134 | 1801188 |
| RP_POS_369_1521a6_444 | RP | POS | 6.44 | 369.1521 | | 369.1526(100) | 4 | 2.33E-01 | -0.23 | 0.45 | 9.40E-01 | -0.02 | 0.32 | 2.81E-01 | -0.24 | 0.43 | 3.03E-01 | -0.22 | 0.40 | 8901924 | 776482 | 8706261 | 863678 | 8712760 | 848872 | 8699762 | 886811 |
| RP_NEG_257_2132a10_235 | RP | NEG | 10.24 | 257.2132 | | | | 2.65E-02 | -0.43 | 0.92 | 9.41E-01 | -0.02 | 0.79 | 3.91E-02 | -0.43 | 0.89 | 6.19E-02 | -0.42 | 0.87 | 1078333 | 131314 | 1015431 | 150232 | 1016560 | 161860 | 1014302 | 139279 |
| HILIC_POS_86_07201a1_232 | HILIC | POS | 1.232 | 86.0720 | | | | 1.58E-02 | -0.45 | 1.10 | 9.41E-01 | -0.02 | 0.75 | 3.74E-02 | -0.46 | 0.75 | 3.13E-02 | -0.44 | 1.05 | 4269824 | 761192 | 3865333 | 921306 | 3872256 | 855967 | 3858410 | 991037 |
| RP_POS_229_14093a5_601 | RP | POS | 5.60 | 229.1409 | | | | 9.49E-01 | 0.01 | 0.38 | 9.41E-01 | -0.02 | 0.28 | 9.85E-01 | 0.00 | 0.23 | 9.28E-01 | 0.02 | 0.28 | 1090654 | 476639 | 1097197 | 581394 | 1101551 | 602177 | 1092843 | 565934 |
| RP_POS_731_60516a13_489 | RP | POS | 13.49 | 731.6052 | | | | 7.29E-02 | -0.40 | 0.71 | 9.41E-01 | -0.01 | 0.54 | 8.60E-02 | -0.40 | 0.75 | 1.13E-01 | -0.39 | 0.67 | 2358133 | 1589582 | 1783562 | 1383731 | 1793898 | 1488855 | 1773227 | 1285142 |
| HILIC_POS_132_00327a4_323 | HILIC | POS | 4.323 | 132.0033 | | | | 3.62E-01 | 0.16 | 1.00 | 9.41E-01 | -0.02 | 0.83 | 4.56E-01 | 0.15 | 0.42 | 4.21E-01 | 0.17 | 0.75 | 29892635 | 5568047 | 31038547 | 7674323 | 31095832 | 7878053 | 30981261 | 7544667 |
| RP_NEG_224_06204a6_61 | RP | NEG | 6.61 | 224.0620 | | | | 2.62E-01 | -0.30 | 0.76 | 9.41E-01 | 0.01 | 0.42 | 3.15E-01 | -0.29 | 0.58 | 2.50E-01 | -0.31 | 0.59 | 97020869 | 171848732 | 60580175 | 99988124 | 59835585 | 66301133 | 61324765 | 125707245 |
| RP_POS_269_20935a9_74 | RP | POS | 9.74 | 269.2094 | | | | 5.10E-01 | -0.13 | 0.51 | 9.42E-01 | 0.02 | 0.43 | 5.73E-01 | -0.12 | 0.34 | 5.48E-01 | -0.14 | 0.56 | 1365754 | 86499 | 1353858 | 94181 | 1353162 | 100292 | 1354553 | 88667 |
| RP_POS_824_57849a11_766 | RP | POS | 11.77 | 824.5785 | | | | 3.85E-02 | 0.26 | 0.60 | 9.42E-01 | 0.02 | 0.31 | 1.48E-01 | 0.26 | 0.54 | 1.12E-01 | 0.25 | 0.64 | 1189883 | 1355385 | 2504921 | 5811449 | 2462114 | 5308570 | 2547728 | 6328543 |
| RP_POS_318_24023a9_44 | RP | POS | 9.44 | 318.2402 | | | | 7.16E-01 | -0.10 | 0.66 | 9.42E-01 | -0.01 | 0.36 | 7.20E-01 | -0.11 | 0.52 | 7.34E-01 | -0.09 | 0.46 | 3500683 | 4899942 | 3169008 | 2636539 | 3188422 | 2030162 | 3149594 | 3149954 |
| RP_NEG_336_785a15_4 | RP | NEG | 15.40 | 336.7850 | | | | 4.70E-01 | 0.20 | 0.69 | 9.42E-01 | -0.01 | 0.40 | 4.79E-01 | 0.19 | 0.62 | 4.97E-01 | 0.20 | 0.42 | 1253092 | 165184 | 1275450 | 93043 | 1276134 | 118922 | 1274765 | 57854 |
| HILIC_NEG_158_93895a4_275 | HILIC | NEG | 4.275 | 158.9390 | | | | 5.99E-01 | 0.10 | 0.89 | 9.42E-01 | -0.02 | 0.77 | 6.73E-01 | 0.09 | 0.20 | 6.26E-01 | 0.10 | 0.38 | 6603429 | 1247510 | 6746289 | 1560406 | 6757708 | 1593573 | 6734870 | 1542623 |
| RP_POS_726_54169a13_644 | RP | POS | 13.64 | 726.5417 | | | | 1.68E-02 | -0.55 | 0.98 | 9.42E-01 | 0.01 | 0.25 | 2.96E-02 | -0.55 | 0.95 | 2.17E-02 | -0.56 | 0.97 | 1764845 | 1124559 | 1222599 | 897624 | 1216036 | 847117 | 1229162 | 954048 |
| RP_NEG_294_9538a0_464 | RP | NEG | 0.46 | 294.9538 | | | | 1.17E-06 | -0.68 | 1.31 | 9.42E-01 | -0.02 | 1.13 | 1.19E-04 | -0.69 | 1.38 | 2.50E-04 | -0.67 | 1.38 | 791293 | 115774 | 613487 | 280779 | 615530 | 287654 | 611445 | 276639 |
| HILIC_POS_139_12294a1_385 | HILIC | POS | 1.385 | 139.1229 | | | | 3.27E-03 | 0.58 | 1.22 | 9.42E-01 | -0.01 | 0.54 | 8.61E-03 | 0.58 | 1.03 | 7.85E-03 | 0.59 | 1.20 | 10237668 | 5289308 | 13602248 | 5696905 | 13643675 | 5810313 | 13560822 | 5639908 |
| RP_POS_283_26303a10_386 | RP | POS | 10.39 | 283.2630 | | | | 7.05E-01 | 0.06 | 0.77 | 9.43E-01 | 0.02 | 1.04 | 7.53E-01 | 0.06 | 0.58 | 7.69E-01 | 0.05 | 0.59 | 2034403 | 283551 | 2062513 | 545342 | 2058568 | 460226 | 2066459 | 623720 |
| HILIC_NEG_132_92348a2_998 | HILIC | NEG | 2.998 | 132.9235 | | | | 8.78E-01 | 0.03 | 0.93 | 9.43E-01 | 0.01 | 0.83 | 8.65E-01 | 0.04 | 0.43 | 9.14E-01 | 0.03 | 0.22 | 12891589 | 3117030 | 12987832 | 2903197 | 12966876 | 2949038 | 13008788 | 2886416 |
| RP_POS_388_1694a10_703 | RP | POS | 10.70 | 388.1694 | | | | 1.89E-06 | 0.70 | 1.53 | 9.43E-01 | -0.02 | 0.60 | 4.98E-05 | 0.70 | 1.50 | 3.33E-04 | 0.71 | 1.47 | 165223 | 117447 | 325536 | 241274 | 327276 | 266433 | 323795 | 215920 |
| RP_NEG_286_77243a0_522 | RP | NEG | 0.52 | 286.7724 | | | | 1.62E-08 | -0.79 | 1.39 | 9.43E-01 | 0.02 | 1.13 | 6.41E-06 | -0.78 | 1.51 | 2.56E-05 | -0.79 | 1.44 | 1475494 | 192450 | 1123429 | 472173 | 1120027 | 501946 | 1126831 | 445500 |
| RP_POS_546_245a7_847 | RP | POS | 7.85 | 546.2450 | | | | 8.09E-02 | -0.39 | 0.85 | 9.43E-01 | 0.01 | 0.81 | 1.16E-01 | -0.38 | 0.72 | 9.99E-02 | -0.40 | 0.80 | 633995 | 61246 | 612544 | 52279 | 612167 | 51552 | 612920 | 53517 |
| RP_POS_217_08084a0_618 | RP | POS | 0.62 | 217.0808 | | | | 3.20E-05 | -0.55 | 1.24 | 9.43E-01 | 0.02 | 1.14 | 1.34E-03 | -0.54 | 1.30 | 2.33E-03 | -0.56 | 1.32 | 5440694 | 868536 | 4145421 | 2583188 | 4126906 | 2720000 | 4163935 | 2466334 |
| RP_POS_377_21988a11_161 | RP | POS | 11.16 | 377.2199 | | | | 6.33E-01 | -0.08 | 0.71 | 9.43E-01 | -0.02 | 0.68 | 6.48E-01 | -0.09 | 0.57 | 7.23E-01 | -0.07 | 0.67 | 18094036 | 1005793 | 17984243 | 1437994 | 17994534 | 1527475 | 17973953 | 1358109 |
| RP_POS_602_28308a10_5 | RP | POS | 10.50 | 602.2831 | | | | 4.11E-01 | 0.20 | 0.80 | 9.43E-01 | 0.01 | 0.11 | 4.39E-01 | 0.21 | 0.60 | 4.45E-01 | 0.19 | 0.63 | 478989 | 345391 | 533715 | 249695 | 531928 | 222899 | 535501 | 276184 |
| HILIC_NEG_115_92075a1_415 | HILIC | NEG | 1.415 | 115.9208 | | | | 2.66E-02 | -0.36 | 0.94 | 9.44E-01 | -0.02 | 0.68 | 5.83E-02 | -0.36 | 0.70 | 7.65E-02 | -0.35 | 0.63 | 19093452 | 1594996 | 18224048 | 2629545 | 18242795 | 2693827 | 18205300 | 2590888 |
| RP_POS_818_77045a0_498 | RP | POS | 0.50 | 818.7705 | | | | 1.58E-05 | -0.54 | 1.28 | 9.44E-01 | 0.02 | 1.21 | 1.28E-03 | -0.53 | 1.35 | 2.22E-03 | -0.55 | 1.35 | 5650497 | 631952 | 4356419 | 2654423 | 4337564 | 2786308 | 4375274 | 2543910 |
| RP_POS_476_29556a11_144 | RP | POS | 11.14 | 476.2956 | | | | 1.49E-02 | 0.39 | 0.93 | 9.44E-01 | -0.02 | 0.29 | 3.43E-02 | 0.39 | 0.83 | 5.26E-02 | 0.40 | 0.78 | 152733 | 186709 | 264288 | 304268 | 266434 | 335298 | 262141 | 273154 |
| HILIC_NEG_122_92595a2_933 | HILIC | NEG | 2.933 | 122.9260 | | | | 4.11E-07 | -0.65 | 1.34 | 9.44E-01 | 0.02 | 0.77 | 7.86E-05 | -0.64 | 1.51 | 4.24E-04 | -0.66 | 1.49 | 375332 | 36312 | 297620 | 130662 | 296699 | 141666 | 298541 | 120095 |
| RP_POS_334_14359a9_318 | RP | POS | 9.32 | 334.1436 | | | | 1.14E-01 | 0.23 | 0.98 | 9.44E-01 | -0.02 | 0.44 | 3.20E-01 | 0.22 | 0.74 | 8.68E-02 | 0.23 | 0.79 | 352860 | 165571 | 428117 | 371293 | 430730 | 240848 | 425504 | 469583 |

**Supplementary table 1: Metabolomics results** (in asceding order according to p-value from progressive ALD vs  non-progressive ALD comparison, with molecular features with ID_level of 1-2 first)

| | | | | | | | | all_ALD_vs_control | | | Prog_ALD_vs_non-prog_ALD | | | Prog_ALD_vs_control_ALD | | | Non-prog_ALD_vs_control | | | Healthy_control | | all_ALD | | Non-prog_ALD | | Progressive_ALD | |
|---|---|---|---|---|---|---|---|---|---|---|---|---|---|---|---|---|---|---|---|---|---|---|---|---|---|---|---|
| | | | | | | | | t-test | Cohen's | PLS-DA | t-test | Cohen's | PLS-DA | t-test | Cohen's | PLS-DA | t-test | Cohen's | PLS-DA | | | | | | | | |
| Feature_ID | RP_HILIC | POS_NEG | Average.Rt | Average.Mz | Metabolite.name | curated_MSMS | ID_level | p | d | VIP | p | d | VIP | p | d | VIP | p | d | VIP | Mean | SD | Mean | SD | Mean | SD | Mean | SD |
| RP_POS_357_19379a11_258 | RP | POS | 11.26 | 357.1938 | | | | 4.35E-05 | 0.81 | 1.52 | 9.45E-01 | 0.01 | 0.90 | 2.59E-04 | 0.82 | 1.45 | 1.55E-04 | 0.81 | 1.48 | 1123127 | 249302 | 1352315 | 270071 | 1350425 | 251824 | 1354204 | 289728 |
| RP_NEG_284_7753a0_522 | RP | NEG | 0.52 | 284.7753 | | | | 1.90E-08 | -0.77 | 1.39 | 9.45E-01 | 0.01 | 1.14 | 7.79E-06 | -0.76 | 1.51 | 3.78E-05 | -0.78 | 1.43 | 2172952 | 265548 | 1669347 | 694975 | 1664494 | 743187 | 1674200 | 650740 |
| RP_POS_375_16388a6_593 | RP | POS | 6.59 | 375.1639 | | | | 2.14E-01 | -0.24 | 0.45 | 9.45E-01 | -0.01 | 0.33 | 2.55E-01 | -0.25 | 0.45 | 2.93E-01 | -0.23 | 0.47 | 1640124 | 182216 | 1592088 | 205910 | 1593518 | 211560 | 1590657 | 202241 |
| RP_NEG_378_98596a6_755 | RP | NEG | 6.76 | 378.9860 | | | | 8.04E-01 | -0.05 | 0.48 | 9.46E-01 | -0.01 | 0.35 | 8.04E-01 | -0.06 | 0.29 | 8.44E-01 | -0.04 | 0.44 | 2703241 | 843272 | 2660916 | 813633 | 2666489 | 779735 | 2655342 | 854083 |
| RP_NEG_564_34491a11_625 | RP | NEG | 11.63 | 564.3449 | | | | 1.39E-01 | 0.23 | 0.66 | 9.46E-01 | 0.01 | 0.38 | 2.40E-01 | 0.24 | 0.65 | 2.10E-01 | 0.23 | 0.83 | 4628617 | 816161 | 4930082 | 1412217 | 4920407 | 1274672 | 4939757 | 1550624 |
| RP_POS_155_02971a15_449 | RP | POS | 15.45 | 155.0297 | | | | 6.26E-01 | -0.11 | 0.59 | 9.46E-01 | 0.01 | 0.30 | 6.73E-01 | -0.11 | 0.36 | 6.33E-01 | -0.12 | 0.64 | 16064855 | 701745 | 15998126 | 561391 | 15994302 | 554149 | 16001950 | 574142 |
| RP_POS_304_26099a10_847 | RP | POS | 10.85 | 304.2610 | | | | 5.38E-06 | 0.86 | 1.35 | 9.46E-01 | -0.01 | 0.24 | 7.59E-05 | 0.85 | 1.36 | 2.69E-05 | 0.86 | 1.57 | 525115769 | 132986682 | 666688280 | 160033711 | 667777152 | 151571566 | 665599408 | 169610988 |
| HILIC_NEG_518_67914a2_932 | HILIC | NEG | 2.932 | 518.6791 | | | | 7.68E-07 | -0.66 | 1.32 | 9.46E-01 | 0.01 | 0.75 | 1.13E-04 | -0.65 | 1.46 | 3.58E-04 | -0.66 | 1.48 | 167612 | 24159 | 124280 | 71618 | 123796 | 76349 | 124764 | 67330 |
| HILIC_POS_154_0864a0_546 | HILIC | POS | 0.546 | 154.0864 | | | | 1.57E-01 | 0.16 | 0.69 | 9.47E-01 | -0.02 | 0.35 | 3.20E-01 | 0.16 | 0.58 | 3.19E-01 | 0.17 | 0.42 | 171922 | 113896 | 7091917 | 48468142 | 7418540 | 50944511 | 6765294 | 46375670 |
| RP_POS_273_10992a8_624 | RP | POS | 8.62 | 273.1099 | | | | 7.50E-01 | -0.06 | 0.31 | 9.47E-01 | -0.01 | 0.40 | 7.66E-01 | -0.07 | 0.33 | 7.90E-01 | -0.06 | 0.36 | 3043253 | 208720 | 3029406 | 225716 | 3030925 | 196255 | 3027886 | 253800 |
| RP_POS_780_55298a12_465 | RP | POS | 12.47 | 780.5530 | | | | 5.42E-03 | -0.64 | 1.08 | 9.47E-01 | -0.01 | 0.58 | 1.03E-02 | -0.64 | 1.12 | 8.31E-03 | -0.63 | 1.02 | 8810069 | 5034252 | 5947898 | 4083449 | 5975327 | 3713749 | 5920469 | 4460486 |
| RP_POS_569_43268a11_608 | RP | POS | 11.61 | 569.4327 | | | | 9.51E-01 | 0.01 | 0.84 | 9.47E-01 | 0.01 | 0.67 | 9.29E-01 | 0.02 | 0.51 | 9.82E-01 | 0.01 | 0.72 | 4577811 | 1110052 | 4592053 | 1193305 | 4584055 | 1314680 | 4600052 | 1071681 |
| HILIC_NEG_266_79578a2_936 | HILIC | NEG | 2.936 | 266.7958 | | | | 1.00E-06 | -0.63 | 1.34 | 9.47E-01 | 0.01 | 0.76 | 1.44E-04 | -0.62 | 1.49 | 5.83E-04 | -0.64 | 1.50 | 656150 | 75558 | 505569 | 260925 | 503824 | 280936 | 507315 | 242119 |
| RP_POS_163_97723a7_1 | RP | POS | 7.10 | 163.9772 | | | | 9.13E-01 | -0.02 | 0.37 | 9.47E-01 | -0.01 | 0.14 | 8.97E-01 | -0.03 | 0.44 | 9.46E-01 | -0.02 | 0.67 | 28387358 | 6793591 | 28237057 | 6591851 | 28280956 | 6960867 | 28193158 | 6271697 |
| RP_POS_890_78992a11_672 | RP | POS | 11.67 | 890.7899 | | | | 6.70E-02 | -0.48 | 0.86 | 9.47E-01 | 0.01 | 0.46 | 8.56E-02 | -0.47 | 0.77 | 7.46E-02 | -0.48 | 0.84 | 666808 | 727961 | 410249 | 443619 | 407305 | 426786 | 413193 | 464163 |
| RP_NEG_893_19391a9_868 | RP | NEG | 9.87 | 893.1939 | | | | 2.08E-01 | -0.27 | 0.70 | 9.48E-01 | -0.01 | 0.45 | 2.39E-01 | -0.28 | 0.54 | 2.62E-01 | -0.27 | 0.61 | 4749487 | 341251 | 4662965 | 308696 | 4665012 | 311132 | 4660917 | 309385 |
| RP_POS_282_27994a11_254 | RP | POS | 11.25 | 282.2799 | | | | 4.28E-08 | 0.81 | 1.23 | 9.48E-01 | -0.01 | 0.43 | 9.12E-05 | 0.80 | 1.26 | 5.50E-07 | 0.82 | 1.59 | 7831501 | 2812743 | 12325290 | 5780982 | 12363580 | 4715011 | 12287001 | 6729579 |
| RP_NEG_556_3266a8_911 | RP | NEG | 8.91 | 556.3266 | | | | 9.40E-04 | -0.52 | 1.34 | 9.48E-01 | 0.01 | 0.52 | 2.18E-02 | -0.51 | 1.27 | 6.53E-04 | -0.53 | 1.29 | 3596400 | 1031271 | 2718829 | 1809526 | 2706850 | 1207723 | 2730809 | 2270837 |
| RP_POS_223_13063a7_184 | RP | POS | 7.18 | 223.1306 | | | | 8.29E-01 | 0.05 | 0.57 | 9.48E-01 | -0.01 | 0.63 | 8.68E-01 | 0.04 | 0.41 | 8.17E-01 | 0.05 | 0.59 | 1633155 | 221649 | 1642678 | 195399 | 1643970 | 177500 | 1641386 | 213614 |
| HILIC_NEG_205_83936a3_059 | HILIC | NEG | 3.059 | 205.8394 | | | | 2.01E-01 | 0.21 | 1.05 | 9.48E-01 | -0.01 | 0.82 | 2.93E-01 | 0.20 | 0.61 | 2.79E-01 | 0.22 | 0.95 | 3941947 | 747297 | 4167112 | 1145310 | 4174626 | 1186528 | 4159598 | 1114574 |
| HILIC_NEG_100_93327a2_941 | HILIC | NEG | 2.941 | 100.9333 | | | | 3.35E-03 | 0.44 | 1.13 | 9.49E-01 | -0.01 | 0.79 | 1.52E-02 | 0.43 | 1.01 | 2.31E-02 | 0.44 | 1.11 | 15869858 | 1512956 | 17081302 | 3023184 | 17100970 | 3241969 | 17061635 | 2820400 |
| RP_POS_851_55768a13_718 | RP | POS | 13.72 | 851.5577 | | | | 2.32E-01 | 0.24 | 0.45 | 9.49E-01 | 0.01 | 0.61 | 2.80E-01 | 0.24 | 0.52 | 2.98E-01 | 0.23 | 0.64 | 2970289 | 1293267 | 3294477 | 1400706 | 3285505 | 1378472 | 3303449 | 1436539 |
| HILIC_NEG_250_95161a1_203 | HILIC | NEG | 1.203 | 250.9516 | | | | 7.17E-08 | 0.79 | 1.44 | 9.49E-01 | 0.01 | 0.69 | 8.08E-06 | 0.80 | 1.43 | 4.04E-05 | 0.78 | 1.35 | 904792 | 810718 | 2190115 | 1703757 | 2179229 | 1796466 | 2201002 | 1623972 |
| HILIC_POS_224_12802a5_648 | HILIC | POS | 5.648 | 224.1280 | | | | 8.86E-01 | -0.03 | 0.63 | 9.49E-01 | 0.01 | 0.64 | 9.24E-01 | -0.02 | 0.03 | 8.73E-01 | -0.03 | 0.10 | 6281780 | 1090906 | 6249441 | 1160690 | 6242030 | 1095683 | 6256852 | 1233395 |
| RP_POS_279_19238a9_193 | RP | POS | 9.19 | 279.1924 | Unknown | 279.1907(100) | 4 | 5.76E-01 | -0.11 | 0.51 | 9.50E-01 | 0.01 | 0.67 | 6.40E-01 | -0.10 | 0.33 | 6.07E-01 | -0.11 | 0.66 | 3647778 | 372753 | 3603773 | 422737 | 3601096 | 436628 | 3606449 | 412797 |
| RP_POS_499_82739a5_296 | RP | POS | 5.30 | 499.8274 | | | | 6.57E-02 | 0.28 | 0.80 | 9.50E-01 | 0.01 | 0.62 | 1.65E-01 | 0.28 | 0.49 | 1.10E-01 | 0.27 | 0.79 | 1587808 | 1525868 | 2331310 | 2948963 | 2312641 | 2536807 | 2349979 | 3336786 |
| HILIC_POS_195_11623a0_846 | HILIC | POS | 0.846 | 195.1162 | | | | 5.00E-01 | 0.13 | 0.59 | 9.50E-01 | -0.01 | 0.60 | 5.77E-01 | 0.12 | 0.25 | 5.35E-01 | 0.13 | 0.24 | 1242000 | 342255 | 1291542 | 407745 | 1294122 | 409192 | 1288962 | 410430 |
| RP_POS_167_05643a0_727 | RP | POS | 0.73 | 167.0564 | | | | 4.76E-04 | -0.95 | 1.72 | 9.50E-01 | 0.01 | 0.41 | 7.81E-04 | -0.94 | 1.47 | 5.68E-04 | -0.95 | 1.69 | 1349957 | 1117073 | 557413 | 618431 | 553523 | 570279 | 561304 | 668932 |
| RP_POS_392_1918a6_593 | RP | POS | 6.59 | 392.1918 | | | | 9.69E-02 | -0.32 | 0.65 | 9.50E-01 | -0.01 | 0.56 | 1.24E-01 | -0.32 | 0.65 | 1.64E-01 | -0.31 | 0.65 | 3726086 | 415964 | 3578340 | 476098 | 3581330 | 509000 | 3575350 | 445943 |
| HILIC_NEG_115_92074a5_742 | HILIC | NEG | 5.742 | 115.9207 | | | | 5.51E-01 | -0.11 | 0.49 | 9.51E-01 | 0.01 | 0.48 | 6.49E-01 | -0.10 | 0.28 | 5.61E-01 | -0.11 | 0.41 | 38546348 | 5887877 | 37779216 | 7470965 | 37732777 | 6545924 | 37825655 | 8361590 |
| RP_POS_772_58411a13_557 | RP | POS | 13.56 | 772.5841 | | | | 1.02E-06 | -1.36 | 2.20 | 9.51E-01 | 0.01 | 0.77 | 1.31E-06 | -1.35 | 2.03 | 1.21E-06 | -1.36 | 1.92 | 18093793 | 7552710 | 9947229 | 3672301 | 9924481 | 3730038 | 9969977 | 3651379 |
| RP_POS_223_09416a12_89 | RP | POS | 12.89 | 223.0942 | | | | 9.80E-01 | 0.00 | 0.35 | 9.51E-01 | 0.01 | 0.54 | 9.60E-01 | 0.01 | 0.22 | 9.93E-01 | 0.00 | 0.17 | 12336300 | 4636317 | 12360831 | 5579098 | 12326344 | 5217791 | 12395318 | 5971611 |
| HILIC_NEG_186_93375a1_687 | HILIC | NEG | 1.687 | 186.9338 | | | | 2.54E-02 | -0.47 | 1.05 | 9.51E-01 | 0.01 | 0.62 | 3.91E-02 | -0.47 | 0.82 | 4.43E-02 | -0.48 | 1.06 | 3021863 | 1772249 | 2201934 | 1688297 | 2191559 | 1815498 | 2212310 | 1569270 |
| RP_NEG_966_56793a13_977 | RP | NEG | 13.98 | 966.5679 | | | | 1.43E-02 | 0.43 | 1.12 | 9.51E-01 | 0.01 | 0.63 | 3.00E-02 | 0.44 | 0.97 | 4.19E-02 | 0.42 | 1.10 | 2308770 | 1481707 | 3140221 | 2022931 | 3127803 | 2099941 | 3152639 | 1964166 |
| RP_POS_141_95854a0_25 | RP | POS | 0.25 | 141.9585 | | | | 6.01E-01 | -0.11 | 0.43 | 9.52E-01 | -0.01 | 0.24 | 6.17E-01 | -0.12 | 0.34 | 6.56E-01 | -0.10 | 0.33 | 4492559 | 486269 | 4441118 | 464682 | 4443962 | 470784 | 4438273 | 463261 |
| HILIC_NEG_174_95631a7_95 | HILIC | NEG | 7.95 | 174.9563 | | | | 3.17E-04 | -0.48 | 0.86 | 9.52E-01 | 0.01 | 0.58 | 7.81E-03 | -0.48 | 1.06 | 5.19E-03 | -0.49 | 0.91 | 63431015 | 9425078 | 51918764 | 26264163 | 51758405 | 26012024 | 52079124 | 26777085 |
| HILIC_NEG_332_69598a2_946 | HILIC | NEG | 2.946 | 332.6960 | | | | 1.20E-05 | -0.58 | 1.33 | 9.52E-01 | 0.01 | 0.76 | 5.79E-04 | -0.57 | 1.45 | 1.66E-03 | -0.59 | 1.46 | 1618089 | 220137 | 1269428 | 659504 | 1265411 | 707153 | 1273446 | 615362 |
| RP_NEG_188_98595a2_662 | RP | NEG | 2.66 | 188.9860 | | | | 1.09E-01 | -0.37 | 0.80 | 9.52E-01 | 0.01 | 0.67 | 1.52E-01 | -0.36 | 0.77 | 1.22E-01 | -0.37 | 0.79 | 3773333 | 3081452 | 2793458 | 2481027 | 2778373 | 2282836 | 2808544 | 2687901 |
| RP_POS_159_96918a0_88 | RP | POS | 0.88 | 159.9692 | | | | 2.61E-01 | -0.25 | 0.49 | 9.52E-01 | -0.01 | 0.26 | 2.88E-01 | -0.25 | 0.51 | 3.18E-01 | -0.24 | 0.55 | 6504952 | 3170698 | 5794915 | 2746646 | 5811527 | 2834925 | 5778302 | 2684162 |
| RP_NEG_282_98325a7_564 | RP | NEG | 7.56 | 282.9833 | | | | 7.55E-01 | -0.07 | 0.66 | 9.52E-01 | -0.01 | 0.27 | 7.62E-01 | -0.07 | 0.60 | 7.94E-01 | -0.06 | 0.44 | 4260721 | 988860 | 4198285 | 942438 | 4203960 | 902505 | 4192609 | 989911 |
| RP_POS_497_39612a10_981 | RP | POS | 10.98 | 497.3961 | | | | 1.31E-01 | 0.31 | 0.93 | 9.52E-01 | 0.01 | 1.05 | 1.87E-01 | 0.31 | 0.86 | 1.62E-01 | 0.30 | 0.85 | 1174452 | 397474 | 1298592 | 402402 | 1296176 | 351582 | 1301008 | 451152 |
| RP_POS_424_28171a11_038 | RP | POS | 11.04 | 424.2817 | | | | 7.82E-02 | 0.33 | 0.68 | 9.52E-01 | -0.01 | 0.29 | 1.30E-01 | 0.33 | 0.61 | 1.15E-01 | 0.34 | 0.63 | 1249683 | 2024546 | 2019381 | 2376240 | 2033644 | 2379444 | 2005117 | 2397103 |
| RP_POS_246_8624a0_246 | RP | POS | 0.25 | 246.8624 | | | | 9.73E-01 | 0.01 | 0.30 | 9.53E-01 | 0.01 | 0.44 | 9.52E-01 | 0.01 | 0.25 | 9.99E-01 | 0.00 | 0.53 | 3721339 | 470991 | 3724649 | 539231 | 3721418 | 589365 | 3727879 | 490001 |
| HILIC_POS_356_7482a3_014 | HILIC | POS | 3.014 | 356.7482 | | | | 4.74E-05 | -0.56 | 1.34 | 9.53E-01 | 0.01 | 0.77 | 1.23E-03 | -0.56 | 1.46 | 2.31E-03 | -0.57 | 1.44 | 355214 | 65235 | 271649 | 161109 | 270686 | 170279 | 272611 | 153118 |
| HILIC_NEG_160_842a2_961 | HILIC | NEG | 2.961 | 160.8420 | | | | 6.64E-01 | 0.08 | 1.03 | 9.53E-01 | -0.01 | 0.79 | 7.11E-01 | 0.08 | 0.70 | 6.95E-01 | 0.09 | 0.93 | 39132139 | 8830973 | 39943141 | 10047423 | 40003181 | 11080131 | 39883101 | 9010272 |
| HILIC_POS_330_22736a0_901 | HILIC | POS | 0.901 | 330.2274 | | | | 6.47E-01 | 0.08 | 0.60 | 9.53E-01 | -0.01 | 0.87 | 7.25E-01 | 0.08 | 0.39 | 6.54E-01 | 0.09 | 0.34 | 377228 | 167589 | 393894 | 207890 | 395136 | 188333 | 392652 | 227697 |
| RP_POS_1005_68726a10_931 | RP | POS | 10.93 | 1005.6873 | | | | 8.16E-01 | 0.05 | 0.98 | 9.53E-01 | 0.01 | 1.13 | 8.26E-01 | 0.05 | 0.66 | 8.45E-01 | 0.04 | 0.85 | 1015435 | 604958 | 1044382 | 620158 | 1040681 | 503524 | 1048084 | 723517 |
| HILIC_POS_116_89114a3_019 | HILIC | POS | 3.019 | 116.8911 | | | | 3.20E-06 | -0.62 | 1.37 | 9.53E-01 | 0.01 | 0.78 | 2.47E-04 | -0.61 | 1.52 | 7.69E-04 | -0.63 | 1.49 | 1866747 | 255387 | 1444295 | 740728 | 1439878 | 792158 | 1448711 | 693542 |
| RP_POS_423_32294a11_416 | RP | POS | 11.42 | 423.3229 | | | | 5.12E-01 | 0.11 | 0.78 | 9.53E-01 | -0.01 | 0.78 | 6.04E-01 | 0.10 | 0.63 | 5.60E-01 | 0.12 | 0.63 | 807068 | 667747 | 908491 | 987471 | 914308 | 991896 | 902674 | 993061 |
| RP_POS_523_42212a10_881 | RP | POS | 10.88 | 523.4221 | | | | 4.98E-01 | -0.12 | 0.46 | 9.53E-01 | -0.01 | 0.11 | 5.02E-01 | -0.13 | 0.50 | 6.14E-01 | -0.11 | 0.39 | 2548200 | 286454 | 2504722 | 387853 | 2507004 | 449524 | 2502440 | 319135 |
| RP_POS_389_23569a11_379 | RP | POS | 11.38 | 389.2357 | | | | 7.24E-04 | -0.60 | 1.11 | 9.54E-01 | -0.01 | 0.89 | 1.31E-03 | -0.60 | 1.20 | 7.43E-03 | -0.59 | 1.05 | 914654 | 130291 | 811838 | 177700 | 812874 | 205254 | 810801 | 147198 |
| RP_NEG_244_98633a6_179 | RP | NEG | 6.18 | 244.9863 | | | | 9.54E-01 | -0.01 | 1.05 | 9.54E-01 | -0.01 | 0.52 | 9.41E-01 | -0.02 | 1.16 | 9.75E-01 | -0.01 | 0.67 | 3840908 | 1896159 | 3819705 | 1513127 | 3828521 | 1471252 | 3810890 | 1568787 |
| RP_NEG_164_07065a1_452 | RP | NEG | 1.45 | 164.0707 | | | | 4.62E-02 | -0.47 | 0.97 | 9.54E-01 | -0.01 | 0.52 | 6.46E-02 | -0.48 | 0.80 | 5.94E-02 | -0.47 | 1.03 | 834601 | 474290 | 647161 | 361883 | 649266 | 331623 | 645055 | 393202 |
| HILIC_POS_630_68988a2_992 | HILIC | POS | 2.992 | 630.6899 | | | | 5.81E-06 | -0.61 | 1.35 | 9.54E-01 | -0.01 | 0.76 | 2.35E-04 | -0.62 | 1.48 | 1.26E-03 | -0.61 | 1.47 | 437871 | 63661 | 339329 | 175263 | 340339 | 188390 | 338319 | 162998 |
| RP_POS_261_13071a13_014 | RP | POS | 13.01 | 261.1307 | | | | 6.66E-02 | -0.34 | 0.65 | 9.55E-01 | 0.01 | 0.25 | 1.38E-01 | -0.33 | 0.68 | 8.56E-02 | -0.34 | 0.88 | 2510855 | 439201 | 2333215 | 551134 | 2330057 | 486157 | 2336373 | 614270 |
| RP_POS_353_03558a0_617 | RP | POS | 0.62 | 353.0356 | | | | 4.40E-10 | -0.79 | 1.40 | 9.55E-01 | -0.01 | 1.14 | 1.28E-06 | -0.80 | 1.59 | 2.12E-05 | -0.79 | 1.44 | 1334960 | 140000 | 923194 | 555840 | 926375 | 598343 | 920012 | 515933 |
| RP_POS_377_30374a11_038 | RP | POS | 11.04 | 377.3037 | | | | 1.42E-02 | -0.46 | 1.03 | 9.56E-01 | -0.01 | 0.94 | 2.12E-02 | -0.46 | 1.04 | 4.33E-02 | -0.45 | 0.98 | 1701902 | 322426 | 1528009 | 387467 | 1530179 | 432400 | 1525840 | 341095 |
| HILIC_NEG_181_83746a3_043 | HILIC | NEG | 3.043 | 181.8375 | | | | 4.35E-06 | 0.72 | 1.19 | 9.56E-01 | 0.01 | 0.69 | 1.43E-04 | 0.72 | 1.28 | 1.85E-04 | 0.71 | 1.28 | 324342 | 140642 | 496522 | 250920 | 495119 | 252604 | 497925 | 251781 |
| RP_POS_190_99968a0_257 | RP | POS | 0.26 | 190.9997 | | | | 9.73E-01 | -0.01 | 0.32 | 9.56E-01 | -0.01 | 0.42 | 9.51E-01 | -0.01 | 0.44 | 9.98E-01 | 0.00 | 0.26 | 1326287 | 144762 | 1325219 | 178434 | 1326215 | 200796 | 1324222 | 154940 |
| RP_POS_203_62335a10_849 | RP | POS | 10.85 | 203.6234 | | | | 3.73E-06 | 0.72 | 1.18 | 9.56E-01 | 0.01 | 0.69 | 6.64E-04 | 0.72 | 1.17 | 1.42E-05 | 0.71 | 1.39 | 5673843 | 1704869 | 7790496 | 3085103 | 7773261 | 2397257 | 7807730 | 3671854 |
| RP_POS_229_14133a5_663 | RP | POS | 5.66 | 229.1413 | | | | 8.63E-01 | 0.03 | 0.44 | 9.56E-01 | 0.01 | 0.31 | 8.59E-01 | 0.04 | 0.30 | 8.99E-01 | 0.03 | 0.41 | 924967 | 381245 | 938813 | 424037 | 936452 | 422753 | 941174 | 429597 |
| RP_POS_263_20062a9_307 | RP | POS | 9.31 | 263.2006 | | | | 1.78E-03 | 0.36 | 0.76 | 9.56E-01 | 0.01 | 0.77 | 3.78E-03 | 0.37 | 1.13 | 6.92E-02 | 0.36 | 0.71 | 66349 | 30869 | 1215872 | 3578855 | 1196110 | 4299510 | 1235634 | 2718679 |
| RP_NEG_774_79639a0_496 | RP | NEG | 0.50 | 774.7964 | | | | 3.83E-06 | -0.58 | 1.29 | 9.57E-01 | 0.01 | 1.20 | 6.66E-04 | -0.58 | 1.35 | 8.74E-04 | -0.59 | 1.35 | 1305966 | 144097 | 1002708 | 573456 | 999576 | 590204 | 1005840 | 562187 |
| HILIC_NEG_556_49017a2_945 | HILIC | NEG | 2.945 | 556.4902 | | | | 1.57E-04 | -0.57 | 1.28 | 9.57E-01 | -0.01 | 0.75 | 1.25E-03 | -0.57 | 1.39 | 4.06E-03 | -0.56 | 1.38 | 134968 | 30654 | 103392 | 59516 | 103716 | 63945 | 103069 | 55384 |
| RP_POS_188_12798a0_619 | RP | POS | 0.62 | 188.1280 | | | | 6.58E-01 | 0.09 | 0.21 | 9.57E-01 | -0.01 | 0.10 | 7.04E-01 | 0.09 | 0.20 | 6.73E-01 | 0.10 | 0.19 | 268724 | 219449 | 288203 | 203035 | 289302 | 206769 | 287104 | 201325 |
| RP_NEG_312_97357a8_477 | RP | NEG | 8.48 | 312.9736 | | | | 1.97E-01 | -0.25 | 0.52 | 9.57E-01 | -0.01 | 0.13 | 2.39E-01 | -0.26 | 0.51 | 2.66E-01 | -0.25 | 0.53 | 1754532 | 105536 | 1726028 | 113840 | 1726641 | 116295 | 1725414 | 112509 |
| RP_POS_902_58533a14_328 | RP | POS | 14.33 | 902.5853 | | | | 7.71E-01 | -0.07 | 0.92 | 9.57E-01 | -0.01 | 0.19 | 7.76E-01 | -0.07 | 0.64 | 8.00E-01 | -0.06 | 0.72 | 1259483 | 938262 | 1205888 | 765351 | 1210003 | 719733 | 1201773 | 815739 |
| RP_POS_191_10657a6_585 | RP | POS | 6.59 | 191.1066 | | | | 8.24E-01 | 0.04 | 0.44 | 9.57E-01 | 0.01 | 0.45 | 8.24E-01 | 0.05 | 0.24 | 8.68E-01 | 0.04 | 0.32 | 349738 | 213338 | 359863 | 252309 | 358507 | 258813 | 361219 | 248256 |
| RP_POS_262_91586a15_43 | RP | POS | 15.43 | 262.9159 | | | | 6.41E-01 | 0.11 | 0.26 | 9.58E-01 | 0.01 | 0.13 | 6.36E-01 | 0.11 | 0.24 | 6.91E-01 | 0.10 | 0.28 | 16701248 | 1963444 | 16878675 | 1497329 | 16870653 | 1722020 | 16886698 | 1250702 |
| HILIC_POS_260_18558a1_087 | HILIC | POS | 1.087 | 260.1856 | | | | 2.31E-01 | 0.17 | 0.33 | 9.58E-01 | -0.01 | 0.59 | 3.90E-01 | 0.16 | 0.43 | 3.12E-01 | 0.17 | 0.36 | 9351553 | 7368161 | 11956012 | 17283358 | 12048543 | 16319471 | 11863480 | 18362532 |
| RP_NEG_459_11221a0_707 | RP | NEG | 0.71 | 459.1122 | | | | 7.02E-03 | -0.41 | 1.07 | 9.58E-01 | -0.01 | 1.05 | 2.09E-02 | -0.42 | 1.07 | 3.92E-02 | -0.41 | 1.12 | 1882133 | 500470 | 1527821 | 934794 | 1532816 | 997488 | 1522826 | 877770 |
| HILIC_POS_114_09164a0_505 | HILIC | POS | 0.505 | 114.0916 | | | | 1.17E-04 | -0.64 | 1.25 | 9.58E-01 | -0.01 | 0.73 | 7.27E-04 | -0.64 | 1.36 | 1.79E-03 | -0.63 | 1.31 | 318186070 | 86956286 | 235345726 | 135150859 | 236065908 | 143074384 | 234625545 | 128191038 |
| HILIC_POS_339_0376a5_461 | HILIC | POS | 5.461 | 339.0376 | | | | 4.61E-02 | -0.40 | 0.83 | 9.58E-01 | 0.01 | 0.29 | 6.67E-02 | -0.39 | 0.78 | 8.19E-02 | -0.40 | 0.88 | 1100370 | 128984 | 1046139 | 136574 | 1045411 | 150161 | 1046867 | 123027 |
| RP_POS_156_95285a15_566 | RP | POS | 15.57 | 156.9529 | | | | 6.51E-01 | 0.09 | 0.58 | 9.58E-01 | -0.01 | 0.23 | 7.10E-01 | 0.09 | 0.45 | 6.60E-01 | 0.10 | 0.59 | 11003270 | 1125429 | 11107195 | 1125178 | 11113178 | 1052749 | 11101212 | 1203966 |
| RP_POS_185_05739a6_163 | RP | POS | 6.16 | 185.0574 | | | | 3.93E-01 | -0.17 | 0.36 | 9.58E-01 | 0.01 | 0.16 | 4.73E-01 | -0.16 | 0.33 | 4.27E-01 | -0.17 | 0.29 | 2820773 | 425609 | 2744633 | 466145 | 2742171 | 447102 | 2747094 | 488982 |
| HILIC_NEG_134_89481a7_675 | HILIC | NEG | 7.675 | 134.8948 | | | | 1.57E-03 | -0.57 | 0.95 | 9.58E-01 | -0.01 | 0.27 | 3.72E-03 | -0.58 | 1.09 | 8.94E-03 | -0.56 | 0.92 | 40324318 | 4703700 | 36925768 | 6103619 | 36957987 | 6655215 | 36893549 | 5565485 |
| HILIC_POS_616_66266a3_005 | HILIC | POS | 3.005 | 616.6627 | | | | 7.51E-05 | -0.54 | 1.35 | 9.58E-01 | 0.01 | 0.76 | 1.79E-03 | -0.54 | 1.46 | 3.11E-03 | -0.55 | 1.43 | 528239 | 96241 | 405910 | 245497 | 404621 | 258394 | 407199 | 234514 |
| RP_POS_576_67114a10_876 | RP | POS | 10.88 | 576.6711 | | | | 4.62E-01 | -0.09 | 0.37 | 9.59E-01 | -0.01 | 0.22 | 6.33E-01 | -0.10 | 0.47 | 4.89E-01 | -0.09 | 0.35 | 1172240 | 146803 | 1127715 | 544150 | 1130555 | 381975 | 1124874 | 672548 |
| RP_POS_619_47931a11_387 | RP | POS | 11.39 | 619.4793 | | | | 2.07E-01 | -0.23 | 0.84 | 9.59E-01 | 0.01 | 0.23 | 2.88E-01 | -0.23 | 0.61 | 2.56E-01 | -0.24 | 0.63 | 848951 | 378302 | 745355 | 460880 | 742963 | 453652 | 747746 | 472593 |
| RP_NEG_765_16821a9_606 | RP | NEG | 9.61 | 765.1682 | | | | 1.17E-01 | -0.30 | 0.49 | 9.59E-01 | -0.01 | 0.57 | 1.60E-01 | -0.30 | 0.54 | 1.76E-01 | -0.29 | 0.49 | 10934245 | 932987 | 10621149 | 1074833 | 10626706 | 1081775 | 10615593 | 1078799 |
| RP_NEG_299_25943a11_158 | RP | NEG | 11.16 | 299.2594 | | | | 5.31E-01 | 0.11 | 0.97 | 9.59E-01 | -0.01 | 0.92 | 5.95E-01 | 0.11 | 0.84 | 5.87E-01 | 0.12 | 0.97 | 12797287 | 3547133 | 13289862 | 4674779 | 13313921 | 5029225 | 13265802 | 4342676 |
| RP_POS_530_32129a10_388 | RP | POS | 10.39 | 530.3213 | | | | 1.41E-02 | 0.45 | 1.11 | 9.59E-01 | 0.01 | 0.84 | 4.08E-02 | 0.46 | 0.93 | 2.31E-02 | 0.45 | 1.04 | 1626424 | 598991 | 1951852 | 734137 | 1948086 | 629803 | 1955618 | 832005 |
| HILIC_POS_534_69897a3_006 | HILIC | POS | 3.006 | 534.6990 | | | | 5.40E-05 | -0.55 | 1.35 | 9.59E-01 | 0.01 | 0.77 | 1.53E-03 | -0.54 | 1.47 | 2.73E-03 | -0.56 | 1.46 | 810381 | 143090 | 619940 | 378942 | 617998 | 398745 | 621883 | 362104 |
| RP_POS_164_02917a0_529 | RP | POS | 0.53 | 164.0292 | | | | 2.36E-01 | 0.18 | 1.14 | 9.59E-01 | 0.01 | 0.98 | 3.64E-01 | 0.19 | 1.07 | 2.95E-01 | 0.18 | 1.06 | 433282 | 134331 | 473229 | 236000 | 472021 | 198239 | 474437 | 270605 |
| RP_POS_223_20541a8_904 | RP | POS | 8.90 | 223.2054 | Unknown | 223.0948(100), 135.1169(35), 73.0653(25) | 4 | 7.78E-01 | 0.05 | 0.42 | 9.60E-01 | 0.01 | 0.44 | 7.87E-01 | 0.06 | 0.32 | 8.27E-01 | 0.05 | 0.39 | 654353 | 307462 | 673039 | 374138 | 671127 | 378601 | 674951 | 373456 |
| HILIC_POS_260_76056a3_023 | HILIC | POS | 3.023 | 260.7606 | | | | 3.14E-04 | -0.51 | 1.33 | 9.60E-01 | 0.01 | 0.76 | 4.28E-03 | -0.50 | 1.42 | 6.02E-03 | -0.51 | 1.43 | 1041283 | 207885 | 814452 | 488427 | 811962 | 510070 | 816943 | 470965 |
| HILIC_POS_130_15916a0_576 | HILIC | POS | 0.576 | 130.1592 | | | | 2.07E-01 | -0.20 | 0.69 | 9.60E-01 | 0.01 | 0.46 | 2.85E-01 | -0.19 | 0.37 | 3.20E-01 | -0.20 | 0.38 | 5675973 | 762281 | 5435228 | 1334062 | 5428459 | 1464235 | 5441996 | 1204859 |
| RP_POS_160_05946a0_622 | RP | POS | 0.62 | 160.0595 | | | | 1.02E-04 | -0.56 | 1.21 | 9.60E-01 | 0.01 | 1.09 | 1.73E-03 | -0.55 | 1.26 | 3.03E-03 | -0.56 | 1.28 | 2432339 | 578612 | 1769466 | 1291548 | 1762969 | 1361925 | 1775963 | 1230980 |

**Supplementary table 1: Metabolomics results** (in asceding order according to p-value from progressive ALD vs  non-progressive ALD comparison, with molecular features with ID_level of 1-2 first)

| | | | | | | | | all_ALD_vs_control | | | Prog_ALD_vs_non-prog_ALD | | | Prog_ALD_vs_control_ALD | | | Non-prog_ALD_vs_control | | | Healthy_control | | all_ALD | | Non-prog_ALD | | Progressive_ALD | |
|---|---|---|---|---|---|---|---|---|---|---|---|---|---|---|---|---|---|---|---|---|---|---|---|---|---|---|---|
| | | | | | | | | t-test | Cohen's | PLS-DA | t-test | Cohen's | PLS-DA | t-test | Cohen's | PLS-DA | t-test | Cohen's | PLS-DA | | | | | | | | |
| Feature_ID | RP_HILIC | POS_NEG | Average.Rt | Average.Mz | Metabolite.name | curated_MSMS | ID_level | p | d | VIP | p | d | VIP | p | d | VIP | p | d | VIP | Mean | SD | Mean | SD | Mean | SD | Mean | SD |
| RP_NEG_850_59747a10_918 | RP | NEG | 10.92 | 850.5975 | | | | 5.48E-03 | 0.40 | 1.41 | 9.60E-01 | -0.01 | 0.35 | 3.86E-02 | 0.40 | 1.00 | 1.96E-02 | 0.41 | 1.33 | 1176658 | 497150 | 1566120 | 1057695 | 1571436 | 991842 | 1560805 | 1129806 |
| RP_NEG_280_98236a9_007 | RP | NEG | 9.01 | 280.9824 | | | | 1.97E-01 | -0.18 | 0.41 | 9.60E-01 | -0.01 | 0.30 | 8.79E-02 | -0.19 | 0.77 | 4.45E-01 | -0.18 | 0.66 | 4064893 | 183128 | 3996651 | 414665 | 3998737 | 563444 | 3994566 | 172989 |
| RP_POS_539_35406a9_527 | RP | POS | 9.53 | 539.3541 | | | | 1.73E-02 | -0.44 | 0.86 | 9.61E-01 | 0.01 | 0.58 | 3.43E-02 | -0.43 | 0.81 | 4.09E-02 | -0.44 | 0.88 | 518762 | 25574 | 505272 | 31635 | 505115 | 33647 | 505429 | 29829 |
| RP_POS_190_91154a0_499 | RP | POS | 0.50 | 190.9115 | | | | 5.91E-05 | -0.50 | 1.26 | 9.61E-01 | -0.01 | 1.21 | 1.65E-03 | -0.51 | 1.33 | 6.16E-03 | -0.50 | 1.32 | 7917946 | 942463 | 6190279 | 3807291 | 6209181 | 4080442 | 6171376 | 3554719 |
| RP_NEG_377_20117a10_157 | RP | NEG | 10.16 | 377.2012 | | | | 6.06E-01 | -0.13 | 0.70 | 9.61E-01 | 0.01 | 0.93 | 6.24E-01 | -0.13 | 0.26 | 6.24E-01 | -0.14 | 0.78 | 7032262 | 6225425 | 6427477 | 3793217 | 6408705 | 4387511 | 6446248 | 3133633 |
| RP_NEG_369_00562a1_093 | RP | NEG | 1.09 | 369.0056 | | | | 2.03E-01 | -0.27 | 0.76 | 9.61E-01 | -0.01 | 0.94 | 2.31E-01 | -0.27 | 0.74 | 2.66E-01 | -0.26 | 0.77 | 1438141 | 1154486 | 1138604 | 1102804 | 1144059 | 1165658 | 1133148 | 1048024 |
| RP_POS_589_56335a11_166 | RP | POS | 11.17 | 589.5634 | | | | 1.40E-10 | 0.86 | 1.38 | 9.61E-01 | 0.01 | 0.80 | 1.05E-05 | 0.86 | 1.44 | 7.71E-08 | 0.85 | 1.57 | 740466 | 519832 | 1963209 | 1491985 | 1955833 | 1276001 | 1970584 | 1693864 |
| RP_NEG_349_90378a15_451 | RP | NEG | 15.45 | 349.9038 | | | | 2.88E-01 | -0.18 | 0.38 | 9.61E-01 | 0.01 | 0.47 | 3.69E-01 | -0.18 | 0.60 | 3.64E-01 | -0.19 | 0.47 | 2684483 | 247535 | 2625001 | 342989 | 2623324 | 358960 | 2626678 | 329888 |
| RP_POS_1067_69702a10_617 | RP | POS | 10.62 | 1067.6970 | | | | 1.24E-03 | 0.63 | 1.40 | 9.62E-01 | 0.01 | 0.98 | 7.81E-03 | 0.63 | 1.21 | 1.46E-03 | 0.62 | 1.44 | 1113500 | 588105 | 1531655 | 659572 | 1528458 | 477866 | 1534852 | 806581 |
| RP_POS_232_92702a15_334 | RP | POS | 15.33 | 232.9270 | | | | 5.41E-01 | 0.14 | 0.72 | 9.62E-01 | -0.01 | 0.32 | 5.75E-01 | 0.13 | 0.59 | 5.73E-01 | 0.14 | 0.97 | 1165279 | 82632 | 1175255 | 69728 | 1175592 | 76573 | 1174918 | 62925 |
| HILIC_NEG_100_93346a0_745 | HILIC | NEG | 0.745 | 100.9335 | | | | 6.15E-06 | 0.70 | 1.26 | 9.62E-01 | 0.01 | 0.27 | 1.51E-04 | 0.70 | 1.42 | 3.32E-04 | 0.69 | 1.21 | 6967797 | 2058153 | 9475112 | 3777733 | 9457105 | 3916593 | 9493120 | 3673340 |
| HILIC_POS_375_32132a0_892 | HILIC | POS | 0.892 | 375.3213 | | | | 9.03E-01 | 0.02 | 0.37 | 9.62E-01 | 0.01 | 0.77 | 9.03E-01 | 0.03 | 0.08 | 9.29E-01 | 0.02 | 0.35 | 5306213 | 601418 | 5322063 | 729094 | 5318598 | 624649 | 5325529 | 826919 |
| RP_NEG_351_02167a0_619 | RP | NEG | 0.62 | 351.0217 | | | | 8.19E-08 | -0.74 | 1.34 | 9.63E-01 | 0.01 | 1.13 | 1.62E-05 | -0.73 | 1.48 | 8.65E-05 | -0.74 | 1.37 | 1924883 | 319496 | 1359062 | 816519 | 1355212 | 876981 | 1362912 | 760190 |
| HILIC_NEG_134_89473a7_497 | HILIC | NEG | 7.497 | 134.8947 | | | | 3.21E-02 | -0.49 | 1.00 | 9.63E-01 | -0.01 | 0.66 | 4.47E-02 | -0.49 | 0.85 | 4.84E-02 | -0.48 | 1.07 | 13936830 | 5436584 | 11594222 | 4456305 | 11615080 | 4476172 | 11573364 | 4481695 |
| HILIC_POS_90_09194a1_707 | HILIC | POS | 1.707 | 90.0919 | | | | 2.12E-02 | -0.40 | 1.19 | 9.63E-01 | 0.01 | 0.72 | 5.21E-02 | -0.40 | 1.13 | 4.51E-02 | -0.41 | 1.01 | 2046068 | 398720 | 1836169 | 545024 | 1833642 | 544028 | 1838695 | 551530 |
| RP_POS_589_42773a10_929 | RP | POS | 10.93 | 589.4277 | | | | 7.85E-01 | -0.06 | 1.09 | 9.63E-01 | -0.01 | 0.05 | 7.93E-01 | -0.06 | 0.86 | 8.22E-01 | -0.05 | 1.05 | 14398381 | 1972031 | 14288572 | 1978778 | 14297741 | 1949489 | 14279402 | 2027401 |
| RP_POS_339_25104a10_258 | RP | POS | 10.26 | 339.2510 | | | | 5.71E-02 | -0.38 | 0.84 | 9.64E-01 | -0.01 | 0.76 | 7.79E-02 | -0.38 | 0.74 | 1.01E-01 | -0.37 | 0.94 | 1103232 | 156038 | 1040691 | 165734 | 1041456 | 176237 | 1039927 | 156319 |
| HILIC_POS_151_03519a7_377 | HILIC | POS | 7.377 | 151.0352 | | | | 5.23E-05 | -0.62 | 1.20 | 9.64E-01 | 0.01 | 0.80 | 1.01E-03 | -0.62 | 1.24 | 9.77E-04 | -0.63 | 1.28 | 14317902 | 1526856 | 12671137 | 2804107 | 12658397 | 2843042 | 12683876 | 2793437 |
| RP_POS_221_0696a15_34 | RP | POS | 15.34 | 221.0696 | | | | 1.77E-02 | 0.47 | 1.14 | 9.65E-01 | -0.01 | 0.51 | 3.74E-02 | 0.47 | 1.00 | 3.06E-02 | 0.47 | 1.27 | 3968500 | 1429951 | 4692539 | 1542478 | 4699342 | 1511338 | 4685736 | 1588332 |
| HILIC_NEG_128_92854a5_426 | HILIC | NEG | 5.426 | 128.9285 | | | | 1.93E-02 | -0.52 | 0.84 | 9.66E-01 | 0.01 | 0.55 | 3.53E-02 | -0.51 | 0.83 | 2.67E-02 | -0.52 | 0.93 | 2714127 | 410018 | 2518364 | 357574 | 2516809 | 336895 | 2519918 | 380557 |
| RP_POS_190_01431a15_422 | RP | POS | 15.42 | 190.0143 | | | | 7.74E-01 | -0.06 | 0.67 | 9.66E-01 | -0.01 | 0.39 | 7.79E-01 | -0.06 | 0.37 | 8.15E-01 | -0.05 | 0.33 | 2543533 | 618333 | 2507144 | 629406 | 2509859 | 656181 | 2504429 | 608112 |
| RP_NEG_144_92305a14_262 | RP | NEG | 14.26 | 144.9231 | | | | 2.58E-01 | -0.21 | 0.56 | 9.66E-01 | -0.01 | 0.43 | 3.43E-01 | -0.22 | 0.49 | 2.99E-01 | -0.21 | 0.53 | 1051900 | 322086 | 973646 | 382420 | 975291 | 325908 | 972001 | 435033 |
| RP_POS_469_16885a6_836 | RP | POS | 6.84 | 469.1689 | Unknown | 469.1664(100), 325.125(35), 397.1505(33) | 4 | 2.07E-01 | -0.23 | 0.59 | 9.66E-01 | -0.01 | 0.30 | 2.65E-01 | -0.24 | 0.50 | 2.79E-01 | -0.23 | 0.46 | 1306840 | 126309 | 1272298 | 153429 | 1272954 | 152634 | 1271642 | 155767 |
| RP_POS_400_22595a11_255 | RP | POS | 11.26 | 400.2260 | | | | 4.54E-05 | 0.75 | 1.54 | 9.66E-01 | 0.01 | 1.17 | 5.28E-04 | 0.75 | 1.47 | 1.77E-04 | 0.75 | 1.54 | 3687957 | 729564 | 4379981 | 918095 | 4376056 | 825315 | 4383905 | 1010853 |
| RP_POS_872_55444a13_711 | RP | POS | 13.71 | 872.5544 | | | | 3.24E-03 | -0.70 | 1.28 | 9.67E-01 | -0.01 | 0.59 | 5.62E-03 | -0.71 | 1.14 | 5.15E-03 | -0.70 | 1.31 | 1194190 | 822451 | 701686 | 615544 | 704290 | 589366 | 699083 | 646649 |
| RP_POS_292_9249a15_444 | RP | POS | 15.44 | 292.9249 | | | | 3.79E-02 | -0.47 | 0.84 | 9.67E-01 | 0.01 | 0.27 | 4.51E-02 | -0.46 | 0.86 | 6.47E-02 | -0.47 | 0.84 | 2157352 | 361031 | 2006228 | 304519 | 2004951 | 353876 | 2007506 | 249248 |
| RP_POS_175_10779a0_621 | RP | POS | 0.62 | 175.1078 | | | | 9.42E-03 | 0.46 | 0.98 | 9.67E-01 | 0.01 | 0.26 | 2.70E-02 | 0.46 | 0.80 | 2.41E-02 | 0.45 | 1.32 | 685088 | 551834 | 1012858 | 745387 | 1009735 | 721297 | 1015980 | 776050 |
| RP_NEG_224_06238a6_09 | RP | NEG | 6.09 | 224.0624 | | | | 2.59E-01 | 0.15 | 0.58 | 9.67E-01 | -0.01 | 0.15 | 4.85E-01 | 0.14 | 0.50 | 2.72E-01 | 0.15 | 0.72 | 674323 | 480237 | 873076 | 1533327 | 879490 | 1165902 | 866661 | 1841399 |
| HILIC_NEG_134_89465a7_947 | HILIC | NEG | 7.947 | 134.8947 | | | | 8.74E-01 | -0.03 | 0.43 | 9.67E-01 | -0.01 | 0.58 | 8.72E-01 | -0.04 | 0.09 | 9.03E-01 | -0.03 | 0.72 | 16095041 | 10176427 | 15759270 | 10859777 | 15804669 | 11070512 | 15713872 | 10757132 |
| HILIC_NEG_136_89175a3_013 | HILIC | NEG | 3.013 | 136.8918 | | | | 6.04E-01 | 0.10 | 0.95 | 9.67E-01 | -0.01 | 0.81 | 6.67E-01 | 0.09 | 0.19 | 6.35E-01 | 0.10 | 0.27 | 85136215 | 13769499 | 86667729 | 16399748 | 86736144 | 16362248 | 86599314 | 16602999 |
| HILIC_NEG_280_76764a1_368 | HILIC | NEG | 1.368 | 280.7676 | | | | 3.65E-17 | -1.36 | 2.38 | 9.67E-01 | -0.01 | 0.42 | 8.07E-13 | -1.36 | 2.33 | 2.72E-13 | -1.35 | 2.50 | 237845 | 31930 | 156785 | 52794 | 157002 | 51704 | 156568 | 54387 |
| RP_NEG_196_94653a0_498 | RP | NEG | 0.50 | 196.9465 | | | | 2.36E-05 | -0.53 | 1.26 | 9.68E-01 | -0.01 | 1.21 | 1.07E-03 | -0.53 | 1.34 | 3.87E-03 | -0.52 | 1.33 | 1873751 | 200768 | 1464252 | 861585 | 1467778 | 918829 | 1460727 | 809650 |
| HILIC_NEG_612_66968a2_927 | HILIC | NEG | 2.927 | 612.6697 | | | | 8.27E-07 | -0.64 | 1.34 | 9.68E-01 | -0.01 | 0.77 | 9.46E-05 | -0.64 | 1.49 | 6.64E-04 | -0.63 | 1.48 | 199752 | 24825 | 150048 | 85324 | 150395 | 92047 | 149702 | 78969 |
| HILIC_NEG_299_73022a3_058 | HILIC | NEG | 3.058 | 299.7302 | | | | 5.42E-03 | -0.34 | 0.58 | 9.68E-01 | 0.01 | 0.42 | 4.66E-02 | -0.33 | 0.61 | 4.66E-02 | -0.34 | 0.62 | 1102787 | 72495 | 975720 | 429486 | 973982 | 437706 | 977459 | 425544 |
| HILIC_POS_79_99043a2_995 | HILIC | POS | 2.995 | 79.9904 | | | | 1.35E-04 | -0.59 | 1.33 | 9.68E-01 | 0.01 | 0.79 | 1.29E-03 | -0.59 | 1.36 | 2.41E-03 | -0.60 | 1.31 | 18056817 | 3390141 | 14666671 | 6058617 | 14642183 | 6423599 | 14691160 | 5735760 |
| HILIC_NEG_197_04283a0_831 | HILIC | NEG | 0.831 | 197.0428 | | | | 1.44E-01 | 0.23 | 0.86 | 9.68E-01 | -0.01 | 0.97 | 2.93E-01 | 0.23 | 0.44 | 1.54E-01 | 0.24 | 0.54 | 397843 | 80589 | 426527 | 132851 | 427064 | 102434 | 425991 | 158636 |
| RP_NEG_303_05084a0_712 | RP | NEG | 0.71 | 303.0508 | | | | 1.62E-02 | 0.33 | 0.87 | 9.68E-01 | 0.01 | 0.32 | 9.50E-02 | 0.33 | 0.80 | 3.84E-02 | 0.32 | 1.05 | 1301044 | 570718 | 1747638 | 1530358 | 1741499 | 1292905 | 1753777 | 1749317 |
| HILIC_NEG_96_95986a7_667 | HILIC | NEG | 7.667 | 96.9599 | | | | 6.74E-02 | 0.33 | 0.74 | 9.68E-01 | 0.01 | 0.69 | 1.07E-01 | 0.33 | 0.57 | 1.23E-01 | 0.32 | 0.56 | 10435319 | 412275 | 10603553 | 536733 | 10601410 | 548582 | 10605695 | 530181 |
| RP_POS_529_22766a6_992 | RP | POS | 6.99 | 529.2277 | | | | 1.43E-01 | -0.25 | 0.71 | 9.68E-01 | -0.01 | 0.66 | 2.00E-01 | -0.26 | 0.60 | 2.25E-01 | -0.25 | 0.78 | 2929664 | 172438 | 2872413 | 238089 | 2873363 | 244429 | 2871463 | 234057 |
| HILIC_NEG_202_92914a5_428 | HILIC | NEG | 5.428 | 202.9291 | | | | 9.76E-01 | -0.01 | 0.10 | 9.69E-01 | 0.01 | 0.29 | 9.93E-01 | 0.00 | 0.07 | 9.63E-01 | -0.01 | 0.09 | 2223022 | 233073 | 2221595 | 238122 | 2220650 | 219491 | 2222540 | 257650 |
| RP_POS_288_13885a10_617 | RP | POS | 10.62 | 288.1389 | | | | 5.31E-01 | 0.13 | 0.62 | 9.69E-01 | -0.01 | 0.36 | 5.82E-01 | 0.13 | 0.63 | 5.61E-01 | 0.14 | 0.68 | 2554099 | 489118 | 2615872 | 461800 | 2617703 | 467124 | 2614041 | 461149 |
| RP_POS_562_51727a15_099 | RP | POS | 15.10 | 562.5173 | | | | 5.72E-01 | 0.12 | 0.40 | 9.69E-01 | 0.01 | 0.10 | 6.02E-01 | 0.12 | 0.51 | 6.19E-01 | 0.11 | 0.36 | 6660218 | 2319592 | 6925460 | 2229681 | 6916631 | 2187521 | 6934289 | 2293252 |
| HILIC_NEG_344_72253a2_938 | HILIC | NEG | 2.938 | 344.7225 | | | | 5.98E-06 | -0.59 | 1.31 | 9.69E-01 | 0.01 | 0.75 | 3.83E-04 | -0.58 | 1.43 | 1.43E-03 | -0.59 | 1.50 | 236966 | 30701 | 182588 | 101578 | 182188 | 109366 | 182988 | 94263 |
| RP_POS_257_21094a9_193 | RP | POS | 9.19 | 257.2109 | | | | 6.99E-01 | -0.08 | 0.56 | 9.69E-01 | -0.01 | 0.58 | 7.14E-01 | -0.08 | 0.38 | 7.43E-01 | -0.08 | 0.63 | 984769 | 103427 | 976634 | 101732 | 977031 | 103952 | 976238 | 100518 |
| RP_POS_129_06596a0_569 | RP | POS | 0.57 | 129.0660 | | | | 2.21E-02 | 0.41 | 0.87 | 9.70E-01 | 0.01 | 0.30 | 3.59E-02 | 0.42 | 0.96 | 5.99E-02 | 0.41 | 0.63 | 1411597 | 854171 | 1849246 | 1097995 | 1845079 | 1199412 | 1853413 | 998582 |
| HILIC_NEG_207_83685a3_059 | HILIC | NEG | 3.059 | 207.8369 | | | | 1.88E-01 | 0.22 | 1.05 | 9.70E-01 | -0.01 | 0.82 | 2.71E-01 | 0.21 | 0.61 | 2.71E-01 | 0.22 | 0.94 | 3933990 | 743858 | 4164820 | 1137013 | 4169124 | 1177056 | 4160516 | 1107466 |
| HILIC_NEG_171_94728a1_403 | HILIC | NEG | 1.403 | 171.9473 | | | | 8.62E-01 | 0.03 | 0.51 | 9.70E-01 | 0.01 | 0.65 | 8.66E-01 | 0.04 | 0.49 | 8.91E-01 | 0.03 | 0.12 | 2341527 | 1014224 | 2378669 | 1134408 | 2374387 | 1105511 | 2382951 | 1173811 |
| HILIC_NEG_277_97629a0_972 | HILIC | NEG | 0.972 | 277.9763 | | | | 2.32E-02 | -0.50 | 1.27 | 9.70E-01 | 0.01 | 0.21 | 4.11E-02 | -0.50 | 0.94 | 3.17E-02 | -0.50 | 1.30 | 169313 | 150087 | 99849 | 131178 | 99356 | 123544 | 100343 | 139652 |
| RP_POS_441_29761a13_01 | RP | POS | 13.01 | 441.2976 | | | | 6.23E-03 | -0.59 | 1.02 | 9.70E-01 | 0.01 | 0.19 | 1.07E-02 | -0.59 | 1.09 | 1.30E-02 | -0.59 | 0.99 | 17806759 | 2589940 | 16333239 | 2364367 | 16324424 | 2519908 | 16342054 | 2223612 |
| RP_POS_304_89331a15_431 | RP | POS | 15.43 | 304.8933 | | | | 3.64E-01 | 0.22 | 0.67 | 9.71E-01 | 0.01 | 0.18 | 3.74E-01 | 0.23 | 0.39 | 4.12E-01 | 0.22 | 0.76 | 4241770 | 554382 | 4338278 | 382451 | 4336863 | 426136 | 4339692 | 337530 |
| HILIC_POS_114_89283a3_018 | HILIC | POS | 3.018 | 114.8928 | | | | 4.29E-06 | -0.60 | 1.36 | 9.71E-01 | 0.01 | 0.77 | 3.48E-04 | -0.59 | 1.51 | 1.10E-03 | -0.60 | 1.50 | 12232993 | 1489894 | 9576530 | 4867369 | 9558557 | 5193089 | 9594503 | 4571347 |
| HILIC_POS_171_11293a0_546 | HILIC | POS | 0.546 | 171.1129 | | | | 1.61E-01 | 0.16 | 0.69 | 9.71E-01 | -0.01 | 0.36 | 3.25E-01 | 0.16 | 0.58 | 3.25E-01 | 0.17 | 0.42 | 288962 | 177557 | 8278632 | 56583436 | 8485991 | 58312893 | 8071273 | 55391602 |
| RP_POS_277_11993a9_45 | RP | POS | 9.45 | 277.1199 | | | | 7.01E-01 | -0.06 | 0.36 | 9.71E-01 | 0.01 | 0.38 | 8.13E-01 | -0.05 | 0.37 | 6.63E-01 | -0.06 | 0.46 | 1052774 | 110157 | 1041454 | 220264 | 1040651 | 139298 | 1042256 | 280387 |
| RP_POS_253_21637a9_843 | RP | POS | 9.84 | 253.2164 | | | | 9.36E-02 | 0.31 | 0.91 | 9.71E-01 | 0.01 | 0.62 | 1.43E-01 | 0.32 | 0.68 | 1.42E-01 | 0.31 | 1.04 | 1087606 | 109617 | 1127420 | 130825 | 1126946 | 128005 | 1127893 | 134884 |
| RP_POS_456_84372a0_473 | RP | POS | 0.47 | 456.8437 | | | | 3.23E-05 | -0.55 | 1.27 | 9.72E-01 | -0.01 | 1.21 | 1.10E-03 | -0.55 | 1.33 | 3.05E-03 | -0.54 | 1.34 | 512084 | 80486 | 388795 | 248361 | 389689 | 262070 | 387902 | 236522 |
| RP_POS_248_03436a3_717 | RP | POS | 3.72 | 248.0344 | | | | 1.19E-01 | 0.24 | 0.99 | 9.72E-01 | 0.01 | 0.55 | 2.35E-01 | 0.25 | 0.84 | 1.68E-01 | 0.24 | 0.49 | 2335960 | 543969 | 2549997 | 962462 | 2546540 | 826578 | 2553454 | 1090100 |
| RP_POS_304_2489a9_912 | RP | POS | 9.91 | 304.2489 | | | | 8.18E-01 | -0.04 | 0.43 | 9.72E-01 | -0.01 | 0.54 | 8.24E-01 | -0.05 | 0.22 | 8.59E-01 | -0.04 | 0.35 | 1107045 | 247650 | 1094724 | 305768 | 1095813 | 322635 | 1093635 | 291205 |
| RP_POS_409_18707a2_893 | RP | POS | 2.89 | 409.1871 | | | | 8.33E-04 | -0.67 | 1.29 | 9.72E-01 | -0.01 | 1.04 | 4.05E-03 | -0.67 | 1.21 | 1.48E-03 | -0.66 | 1.32 | 1525008 | 590369 | 1093523 | 634172 | 1095774 | 535774 | 1091272 | 724907 |
| RP_POS_251_16084a8_339 | RP | POS | 8.34 | 251.1608 | | | | 7.30E-01 | 0.08 | 0.67 | 9.73E-01 | -0.01 | 0.53 | 7.63E-01 | 0.08 | 0.46 | 7.33E-01 | 0.08 | 0.62 | 2783629 | 340029 | 2806440 | 261894 | 2807340 | 241090 | 2805540 | 283639 |
| HILIC_NEG_216_85336a7_676 | HILIC | NEG | 7.676 | 216.8534 | | | | 5.86E-01 | -0.11 | 0.63 | 9.73E-01 | -0.01 | 0.72 | 6.11E-01 | -0.11 | 0.24 | 6.41E-01 | -0.11 | 0.47 | 966844 | 215385 | 942817 | 218129 | 943564 | 226347 | 942070 | 211890 |
| RP_POS_605_38263a14_27 | RP | POS | 14.27 | 605.3826 | | | | 7.53E-01 | 0.06 | 0.44 | 9.73E-01 | -0.01 | 0.14 | 7.96E-01 | 0.06 | 0.29 | 7.67E-01 | 0.06 | 0.69 | 5749748 | 1599405 | 5855000 | 1762493 | 5861019 | 1728950 | 5848981 | 1812958 |
| HILIC_NEG_173_92606a0_595 | HILIC | NEG | 0.595 | 173.9261 | | | | 1.17E-01 | -0.29 | 0.89 | 9.73E-01 | -0.01 | 0.69 | 1.61E-01 | -0.30 | 0.58 | 1.78E-01 | -0.29 | 0.67 | 2258722 | 391314 | 2126038 | 464832 | 2127625 | 475043 | 2124451 | 459213 |
| RP_POS_361_14664a10_703 | RP | POS | 10.70 | 361.1466 | | | | 4.06E-07 | 0.73 | 1.57 | 9.73E-01 | 0.01 | 0.50 | 3.08E-05 | 0.73 | 1.54 | 1.39E-04 | 0.72 | 1.48 | 185940 | 126455 | 378369 | 279733 | 377414 | 296812 | 379324 | 264573 |
| HILIC_NEG_352_99701a7_309 | HILIC | NEG | 7.309 | 352.9970 | | | | 2.22E-01 | 0.26 | 1.27 | 9.73E-01 | -0.01 | 0.36 | 2.71E-01 | 0.25 | 0.87 | 2.68E-01 | 0.26 | 0.89 | 1807692 | 53404 | 1821007 | 51681 | 1821182 | 53306 | 1820831 | 50546 |
| RP_POS_419_31531a10_978 | RP | POS | 10.98 | 419.3153 | | | | 8.40E-02 | 0.29 | 1.28 | 9.73E-01 | 0.01 | 1.15 | 1.56E-01 | 0.30 | 1.17 | 1.32E-01 | 0.29 | 1.07 | 2474809 | 421296 | 2643567 | 612104 | 2641488 | 568787 | 2645645 | 658377 |
| RP_POS_660_0816a0_625 | RP | POS | 0.63 | 660.0816 | | | | 3.45E-05 | -0.62 | 1.20 | 9.73E-01 | 0.01 | 1.03 | 5.19E-04 | -0.62 | 1.28 | 1.35E-03 | -0.63 | 1.24 | 590705 | 135742 | 435816 | 265448 | 434921 | 284074 | 436711 | 248322 |
| RP_POS_401_34113a11_416 | RP | POS | 11.42 | 401.3411 | | | | 4.05E-01 | 0.14 | 0.80 | 9.73E-01 | -0.01 | 0.77 | 5.02E-01 | 0.14 | 0.65 | 4.70E-01 | 0.14 | 0.68 | 1087920 | 875826 | 1257941 | 1316751 | 1262372 | 1301491 | 1253510 | 1345039 |
| RP_POS_378_2428a12_326 | RP | POS | 12.33 | 378.2428 | | | | 1.17E-05 | -0.83 | 1.41 | 9.73E-01 | 0.01 | 0.33 | 1.67E-04 | -0.82 | 1.37 | 4.44E-05 | -0.83 | 1.48 | 3644851 | 701829 | 2929192 | 845738 | 2926353 | 769617 | 2932031 | 923481 |
| RP_POS_276_10114a8_873 | RP | POS | 8.87 | 276.1011 | | | | 6.59E-01 | 0.09 | 0.47 | 9.73E-01 | -0.01 | 0.10 | 7.06E-01 | 0.09 | 0.34 | 6.79E-01 | 0.09 | 0.28 | 642169 | 116848 | 652682 | 116473 | 653073 | 113952 | 652292 | 120099 |
| HILIC_POS_392_27908a0_634 | HILIC | POS | 0.634 | 392.2791 | | | | 3.08E-01 | 0.17 | 0.61 | 9.74E-01 | 0.01 | 0.84 | 4.28E-01 | 0.17 | 0.34 | 3.52E-01 | 0.16 | 0.37 | 535810 | 249200 | 595846 | 385122 | 594562 | 316425 | 597130 | 446696 |
| RP_POS_315_15613a9_603 | RP | POS | 9.60 | 315.1561 | | | | 2.82E-02 | -0.52 | 0.93 | 9.74E-01 | 0.01 | 0.43 | 4.03E-02 | -0.51 | 0.96 | 3.94E-02 | -0.52 | 0.88 | 2631313 | 148795 | 2566205 | 114222 | 2565825 | 116388 | 2566585 | 113195 |
| RP_POS_485_19962a6_861 | RP | POS | 6.86 | 485.1996 | | | | 1.55E-01 | -0.27 | 0.57 | 9.74E-01 | 0.01 | 0.51 | 2.22E-01 | -0.27 | 0.54 | 1.97E-01 | -0.28 | 0.60 | 796431 | 62654 | 777517 | 70429 | 777285 | 68174 | 777749 | 73307 |
| RP_POS_169_9772a0_774 | RP | POS | 0.77 | 169.9772 | | | | 1.97E-01 | 0.23 | 0.74 | 9.74E-01 | -0.01 | 0.28 | 2.92E-01 | 0.23 | 0.76 | 2.44E-01 | 0.23 | 0.65 | 1459820 | 624223 | 1638714 | 818696 | 1641391 | 765593 | 1636037 | 876388 |
| HILIC_POS_300_21713a0_84 | HILIC | POS | 0.84 | 300.2171 | | | | 1.44E-01 | -0.25 | 0.58 | 9.74E-01 | -0.01 | 0.46 | 2.32E-01 | -0.26 | 0.42 | 1.93E-01 | -0.25 | 0.63 | 1341851 | 501217 | 1175491 | 694247 | 1177730 | 622206 | 1173252 | 765926 |
| HILIC_POS_189_07329a5_201 | HILIC | POS | 5.201 | 189.0733 | | | | 3.40E-01 | 0.16 | 0.73 | 9.75E-01 | -0.01 | 0.62 | 4.05E-01 | 0.16 | 0.38 | 4.35E-01 | 0.17 | 0.39 | 5303880 | 842352 | 5488430 | 1218065 | 5492350 | 1334786 | 5484510 | 1102706 |
| RP_POS_285_98807a0_512 | RP | POS | 0.51 | 285.9881 | | | | 3.20E-05 | 0.51 | 1.23 | 9.75E-01 | 0.01 | 1.08 | 4.49E-03 | 0.51 | 1.25 | 1.80E-03 | 0.50 | 1.30 | 85583 | 33906 | 175920 | 199811 | 175281 | 188394 | 176559 | 212534 |
| RP_POS_303_19312a9_308 | RP | POS | 9.31 | 303.1931 | | | | 1.66E-03 | 0.37 | 0.77 | 9.75E-01 | 0.01 | 0.79 | 4.27E-03 | 0.37 | 1.13 | 6.38E-02 | 0.36 | 0.72 | 40166 | 27418 | 686492 | 1997787 | 680174 | 2386039 | 692810 | 1539642 |
| RP_NEG_658_06799a0_626 | RP | NEG | 0.63 | 658.0680 | | | | 1.94E-06 | -0.70 | 1.24 | 9.75E-01 | -0.01 | 0.96 | 9.70E-05 | -0.70 | 1.35 | 2.47E-04 | -0.69 | 1.27 | 2349011 | 341133 | 1879114 | 714331 | 1881340 | 744844 | 1876889 | 690035 |
| HILIC_POS_497_06091a3_075 | HILIC | POS | 3.075 | 497.0609 | | | | 3.05E-04 | 0.49 | 1.20 | 9.75E-01 | 0.01 | 0.83 | 2.33E-03 | 0.50 | 1.32 | 1.10E-02 | 0.49 | 1.37 | 538466 | 42272 | 587628 | 109252 | 587287 | 120891 | 587968 | 97472 |
| HILIC_POS_626_69366a2_992 | HILIC | POS | 2.992 | 626.6937 | | | | 1.68E-05 | -0.58 | 1.35 | 9.75E-01 | 0.01 | 0.76 | 6.63E-04 | -0.58 | 1.47 | 1.77E-03 | -0.58 | 1.46 | 3055299 | 499920 | 2317768 | 1392283 | 2313454 | 1481768 | 2322081 | 1311790 |
| RP_POS_302_24512a11_608 | RP | POS | 11.61 | 302.2451 | | | | 1.95E-04 | 0.66 | 1.06 | 9.76E-01 | -0.01 | 0.21 | 2.33E-03 | 0.66 | 1.05 | 5.64E-04 | 0.66 | 1.27 | 1995029 | 847897 | 2736492 | 1143453 | 2740029 | 1006416 | 2732956 | 1276226 |
| RP_POS_150_96881a0_256 | RP | POS | 0.26 | 150.9688 | | | | 7.78E-01 | 0.05 | 0.35 | 9.76E-01 | -0.01 | 0.52 | 8.18E-01 | 0.05 | 0.21 | 7.95E-01 | 0.06 | 0.15 | 69251612 | 5347510 | 69577163 | 6520334 | 69597285 | 6579945 | 69557040 | 6526918 |
| RP_POS_291_06915a0_71 | RP | POS | 0.71 | 291.0692 | | | | 9.19E-03 | 0.33 | 0.71 | 9.76E-01 | -0.01 | 0.31 | 8.29E-02 | 0.32 | 0.72 | 3.32E-02 | 0.33 | 0.94 | 884502 | 253290 | 1176635 | 1009266 | 1179729 | 904109 | 1173540 | 1113819 |
| RP_POS_397_31033a11_161 | RP | POS | 11.16 | 397.3103 | | | | 1.91E-02 | -0.42 | 0.80 | 9.76E-01 | 0.01 | 0.61 | 3.33E-02 | -0.41 | 0.86 | 5.38E-02 | -0.42 | 0.80 | 3463628 | 532351 | 3181320 | 705218 | 3179183 | 783123 | 3183458 | 625717 |
| RP_POS_341_35233a10_861 | RP | POS | 10.86 | 341.3523 | | | | 1.10E-04 | -0.78 | 1.29 | 9.76E-01 | -0.01 | 0.60 | 5.94E-04 | -0.78 | 1.26 | 3.02E-04 | -0.77 | 1.26 | 2369771 | 420096 | 2009114 | 444358 | 2010453 | 408383 | 2007775 | 481826 |
| RP_POS_565_4259a10_979 | RP | POS | 10.98 | 565.4259 | | | | 9.23E-01 | -0.02 | 0.71 | 9.77E-01 | 0.01 | 0.51 | 9.46E-01 | -0.02 | 0.78 | 9.16E-01 | -0.02 | 0.44 | 1157333 | 762112 | 1141614 | 880439 | 1139049 | 756127 | 1144180 | 997209 |
| HILIC_NEG_200_93427a2_944 | HILIC | NEG | 2.944 | 200.9343 | | | | 7.42E-02 | -0.35 | 1.00 | 9.77E-01 | 0.01 | 0.68 | 1.22E-01 | -0.35 | 0.61 | 1.02E-01 | -0.36 | 0.63 | 401113 | 96545 | 364731 | 104137 | 364428 | 99365 | 365034 | 109712 |
| HILIC_NEG_136_89166a1_329 | HILIC | NEG | 1.329 | 136.8917 | | | | 3.97E-06 | -0.85 | 1.29 | 9.78E-01 | -0.01 | 0.54 | 9.16E-05 | -0.86 | 1.32 | 1.54E-05 | -0.85 | 1.54 | 4712670 | 660794 | 3991908 | 823132 | 3994246 | 716656 | 3989571 | 924832 |

**Supplementary table 1: Metabolomics results** (in asceding order according to p-value from progressive ALD vs  non-progressive ALD comparison, with molecular features with ID_level of 1-2 first)

| | | | | | | | | all_ALD_vs_control | | | Prog_ALD_vs_non-prog_ALD | | | Prog_ALD_vs_control_ALD | | | Non-prog_ALD_vs_control | | | Healthy_control | | all_ALD | | Non-prog_ALD | | Progressive_ALD | |
|---|---|---|---|---|---|---|---|---|---|---|---|---|---|---|---|---|---|---|---|---|---|---|---|---|---|---|---|
| | | | | | | | | t-test | Cohen's | PLS-DA | t-test | Cohen's | PLS-DA | t-test | Cohen's | PLS-DA | t-test | Cohen's | PLS-DA | | | | | | | | |
| Feature_ID | RP_HILIC | POS_NEG | Average.Rt | Average.Mz | Metabolite.name | curated_MSMS | ID_level | p | d | VIP | p | d | VIP | p | d | VIP | p | d | VIP | Mean | SD | Mean | SD | Mean | SD | Mean | SD |
| HILIC_POS_371_23956a1_439 | HILIC | POS | 1.439 | 371.2396 | | | | 1.52E-01 | 0.25 | 0.79 | 9.78E-01 | -0.01 | 0.69 | 2.12E-01 | 0.24 | 0.50 | 2.38E-01 | 0.25 | 0.42 | 2328544 | 332748 | 2438025 | 475593 | 2439370 | 510789 | 2436679 | 442815 |
| RP_NEG_208_78639a15_578 | RP | NEG | 15.58 | 208.7864 | | | | 9.83E-01 | 0.00 | 0.85 | 9.78E-01 | -0.01 | 0.15 | 9.74E-01 | -0.01 | 0.69 | 9.95E-01 | 0.00 | 0.66 | 445087 | 25747 | 444974 | 26779 | 445049 | 29631 | 444899 | 23891 |
| RP_POS_246_94331a0_453 | RP | POS | 0.45 | 246.9433 | | | | 6.48E-04 | -0.51 | 1.16 | 9.78E-01 | 0.01 | 1.06 | 4.63E-03 | -0.51 | 1.19 | 7.91E-03 | -0.52 | 1.22 | 661230 | 184290 | 490699 | 359127 | 489710 | 380176 | 491689 | 340646 |
| RP_POS_574_48584a14_268 | RP | POS | 14.27 | 574.4858 | | | | 1.66E-01 | 0.28 | 0.55 | 9.79E-01 | -0.01 | 0.15 | 2.45E-01 | 0.27 | 0.59 | 1.89E-01 | 0.28 | 0.64 | 67249432 | 15182238 | 71634787 | 16036648 | 71677910 | 13952776 | 71591664 | 18025340 |
| HILIC_NEG_200_85867a5_651 | HILIC | NEG | 5.651 | 200.8587 | | | | 9.00E-01 | -0.03 | 0.51 | 9.79E-01 | -0.01 | 0.87 | 9.07E-01 | -0.03 | 0.37 | 9.15E-01 | -0.02 | 0.04 | 3883454 | 881098 | 3860453 | 927850 | 3862906 | 785682 | 3858000 | 1059279 |
| HILIC_POS_262_75803a3_023 | HILIC | POS | 3.023 | 262.7580 | | | | 3.05E-04 | -0.52 | 1.33 | 9.80E-01 | -0.01 | 0.76 | 3.38E-03 | -0.52 | 1.39 | 6.15E-03 | -0.51 | 1.41 | 1199920 | 256953 | 928313 | 570790 | 929777 | 595679 | 926849 | 550831 |
| HILIC_NEG_134_89474a1_487 | HILIC | NEG | 1.487 | 134.8947 | | | | 3.97E-01 | 0.15 | 0.52 | 9.80E-01 | 0.01 | 0.87 | 4.79E-01 | 0.15 | 0.25 | 4.55E-01 | 0.15 | 0.31 | 19013610 | 4383941 | 19837820 | 5780649 | 19822997 | 5289998 | 19852643 | 6287229 |
| RP_POS_419_20367a9_071 | RP | POS | 9.07 | 419.2037 | | | | 5.74E-09 | 0.70 | 1.15 | 9.80E-01 | -0.01 | 0.15 | 1.22E-04 | 0.70 | 1.25 | 1.08E-05 | 0.70 | 1.25 | 130668 | 203460 | 2111952 | 3092676 | 2119784 | 2863254 | 2104120 | 3335587 |
| RP_NEG_627_13837a0_621 | RP | NEG | 0.62 | 627.1384 | | | | 2.09E-06 | -0.73 | 1.30 | 9.80E-01 | -0.01 | 1.02 | 5.62E-05 | -0.74 | 1.43 | 1.80E-04 | -0.73 | 1.32 | 1997540 | 445784 | 1427301 | 809607 | 1429316 | 855352 | 1425285 | 769851 |
| HILIC_POS_491_4957a1_306 | HILIC | POS | 1.306 | 491.4957 | | | | 7.54E-02 | 0.29 | 0.61 | 9.80E-01 | 0.01 | 0.50 | 1.66E-01 | 0.30 | 0.53 | 1.06E-01 | 0.29 | 0.60 | 232335 | 208950 | 320541 | 321497 | 319741 | 273282 | 321340 | 366258 |
| RP_POS_143_95935a0_249 | RP | POS | 0.25 | 143.9594 | | | | 8.31E-01 | -0.04 | 0.29 | 9.80E-01 | -0.01 | 0.18 | 8.36E-01 | -0.05 | 0.19 | 8.61E-01 | -0.04 | 0.36 | 454895 | 53955 | 452530 | 55470 | 452668 | 59059 | 452392 | 52236 |
| RP_POS_299_25711a10_174 | RP | POS | 10.17 | 299.2571 | | | | 6.14E-01 | -0.10 | 0.67 | 9.80E-01 | 0.01 | 0.67 | 6.52E-01 | -0.10 | 0.49 | 6.58E-01 | -0.10 | 0.78 | 15011695 | 2184099 | 14782139 | 2365551 | 14776292 | 2566965 | 14787985 | 2171746 |
| RP_POS_301_23651a9_271 | RP | POS | 9.27 | 301.2365 | | | | 4.96E-01 | -0.14 | 0.59 | 9.80E-01 | 0.00 | 0.27 | 5.30E-01 | -0.15 | 0.30 | 5.46E-01 | -0.14 | 0.58 | 1425345 | 232557 | 1393459 | 218109 | 1393997 | 220816 | 1392922 | 217608 |
| HILIC_NEG_136_89175a4_128 | HILIC | NEG | 4.128 | 136.8918 | | | | 1.18E-01 | 0.32 | 0.75 | 9.81E-01 | 0.00 | 0.51 | 1.61E-01 | 0.31 | 0.66 | 1.61E-01 | 0.32 | 0.73 | 4985446 | 2879805 | 5922150 | 2967218 | 5929440 | 3045791 | 5914860 | 2917447 |
| RP_POS_352_65912a5_606 | RP | POS | 5.61 | 352.6591 | | | | 5.00E-01 | 0.12 | 0.66 | 9.81E-01 | 0.01 | 0.56 | 5.62E-01 | 0.12 | 0.48 | 5.58E-01 | 0.12 | 0.65 | 1340965 | 623518 | 1432987 | 790181 | 1431066 | 752974 | 1434909 | 833389 |
| HILIC_POS_260_14896a2_383 | HILIC | POS | 2.383 | 260.1490 | | | | 8.88E-03 | 0.33 | 1.22 | 9.81E-01 | 0.01 | 0.84 | 6.16E-02 | 0.34 | 0.92 | 4.54E-02 | 0.33 | 0.73 | 503202 | 195848 | 711142 | 701793 | 709459 | 670614 | 712825 | 738477 |
| RP_NEG_429_17966a11_256 | RP | NEG | 11.26 | 429.1797 | | | | 4.63E-02 | 0.35 | 0.97 | 9.81E-01 | 0.01 | 1.02 | 8.95E-02 | 0.35 | 0.75 | 8.70E-02 | 0.35 | 1.03 | 1944850 | 499752 | 2171219 | 681869 | 2169591 | 671169 | 2172848 | 699220 |
| RP_NEG_416_88354a0_498 | RP | NEG | 0.50 | 416.8835 | | | | 9.07E-06 | -0.55 | 1.28 | 9.82E-01 | 0.00 | 1.21 | 6.43E-04 | -0.55 | 1.37 | 2.26E-03 | -0.56 | 1.34 | 9310550 | 1015000 | 7131718 | 4350012 | 7121945 | 4665809 | 7141492 | 4057268 |
| HILIC_POS_100_11257a1_071 | HILIC | POS | 1.071 | 100.1126 | | | | 8.45E-02 | -0.30 | 1.07 | 9.82E-01 | 0.00 | 0.61 | 1.46E-01 | -0.29 | 1.14 | 1.42E-01 | -0.30 | 0.95 | 97774868 | 36957417 | 83071972 | 52945373 | 82953602 | 53580964 | 83190343 | 52845862 |
| RP_NEG_942_76471a0_495 | RP | NEG | 0.50 | 942.7647 | | | | 4.41E-05 | -0.58 | 1.24 | 9.83E-01 | 0.00 | 1.17 | 1.34E-03 | -0.58 | 1.29 | 1.59E-03 | -0.58 | 1.31 | 1172834 | 151799 | 985622 | 350327 | 986389 | 354078 | 984856 | 350129 |
| RP_POS_860_60785a11_974 | RP | POS | 11.97 | 860.6079 | | | | 5.12E-01 | 0.12 | 0.61 | 9.83E-01 | 0.00 | 0.21 | 5.70E-01 | 0.12 | 0.33 | 5.62E-01 | 0.13 | 0.61 | 5120547 | 862824 | 5241110 | 1007084 | 5243245 | 1029712 | 5238976 | 994393 |
| RP_POS_343_3382a11_26 | RP | POS | 11.26 | 343.3382 | | | | 3.29E-01 | -0.17 | 0.37 | 9.83E-01 | 0.00 | 0.25 | 4.02E-01 | -0.17 | 0.34 | 4.03E-01 | -0.18 | 0.33 | 20305401 | 4460991 | 19337922 | 5877616 | 19325605 | 6062428 | 19350239 | 5748428 |
| RP_NEG_979_14014a9_692 | RP | NEG | 9.69 | 979.1401 | | | | 1.63E-01 | -0.28 | 0.62 | 9.83E-01 | 0.00 | 0.46 | 1.95E-01 | -0.28 | 0.55 | 2.34E-01 | -0.27 | 0.58 | 4785640 | 315732 | 4693442 | 338826 | 4694151 | 368088 | 4692732 | 310579 |
| RP_POS_229_18065a8_339 | RP | POS | 8.34 | 229.1807 | | | | 9.50E-01 | -0.01 | 0.55 | 9.83E-01 | 0.00 | 0.57 | 9.48E-01 | -0.02 | 0.46 | 9.60E-01 | -0.01 | 0.39 | 771097 | 93659 | 769960 | 75180 | 770117 | 72881 | 769803 | 78152 |
| RP_POS_808_58344a13_375 | RP | POS | 13.38 | 808.5834 | | | | 7.37E-03 | 0.53 | 1.13 | 9.84E-01 | 0.00 | 0.29 | 1.89E-02 | 0.52 | 0.96 | 1.46E-02 | 0.53 | 1.01 | 57486862 | 18009545 | 67900672 | 19761617 | 67941504 | 19053498 | 67859840 | 20639128 |
| RP_NEG_263_10416a3_746 | RP | NEG | 3.75 | 263.1042 | | | | 9.18E-02 | -0.33 | 0.65 | 9.84E-01 | 0.00 | 0.22 | 1.71E-01 | -0.33 | 0.58 | 9.50E-02 | -0.34 | 0.74 | 8369474 | 5314873 | 6481317 | 5730718 | 6469474 | 4293650 | 6493160 | 6922194 |
| RP_POS_243_09978a8_492 | RP | POS | 8.49 | 243.0998 | | | | 5.45E-01 | 0.11 | 0.42 | 9.84E-01 | 0.00 | 0.43 | 6.05E-01 | 0.11 | 0.24 | 5.91E-01 | 0.12 | 0.31 | 434496 | 249662 | 466851 | 297195 | 467458 | 298304 | 466245 | 299109 |
| RP_POS_499_82697a6_697 | RP | POS | 6.70 | 499.8270 | | | | 5.63E-01 | 0.12 | 0.68 | 9.84E-01 | 0.00 | 0.29 | 6.12E-01 | 0.12 | 0.31 | 5.88E-01 | 0.12 | 0.90 | 888958 | 1289530 | 1039661 | 1221020 | 1037209 | 1048284 | 1042114 | 1383219 |
| RP_POS_355_28217a10_701 | RP | POS | 10.70 | 355.2822 | | | | 8.27E-01 | 0.04 | 0.48 | 9.84E-01 | 0.00 | 0.11 | 8.47E-01 | 0.05 | 0.35 | 8.42E-01 | 0.04 | 0.78 | 29871091 | 3424219 | 30025873 | 3574594 | 30018834 | 3001905 | 30032912 | 4099351 |
| RP_NEG_213_88892a0_73 | RP | NEG | 0.73 | 213.8889 | | | | 8.53E-01 | -0.03 | 0.69 | 9.84E-01 | 0.00 | 0.52 | 8.83E-01 | -0.03 | 0.36 | 8.80E-01 | -0.03 | 0.94 | 1299933 | 231630 | 1288663 | 447536 | 1289539 | 389659 | 1287786 | 502822 |
| RP_POS_304_93228a15_367 | RP | POS | 15.37 | 304.9323 | | | | 9.65E-01 | -0.01 | 0.24 | 9.85E-01 | 0.00 | 0.05 | 9.78E-01 | -0.01 | 0.18 | 9.63E-01 | -0.01 | 0.21 | 3925273 | 500927 | 3920362 | 690263 | 3919062 | 696547 | 3921661 | 690992 |
| HILIC_POS_282_19135a0_578 | HILIC | POS | 0.578 | 282.1914 | | | | 7.65E-04 | -0.58 | 1.13 | 9.85E-01 | 0.00 | 0.66 | 2.90E-03 | -0.58 | 1.26 | 5.30E-03 | -0.58 | 1.14 | 517387 | 124141 | 418563 | 176367 | 418894 | 186921 | 418231 | 167050 |
| RP_POS_343_33759a0_249 | RP | POS | 0.25 | 343.3376 | | | | 1.83E-01 | 0.23 | 0.65 | 9.85E-01 | 0.00 | 0.42 | 2.48E-01 | 0.24 | 0.60 | 2.62E-01 | 0.23 | 0.64 | 2822530 | 2064069 | 3440450 | 2789149 | 3435227 | 2831465 | 3445674 | 2774926 |
| RP_POS_287_22269a9_912 | RP | POS | 9.91 | 287.2227 | | | | 4.64E-01 | -0.14 | 0.46 | 9.85E-01 | 0.00 | 0.58 | 5.20E-01 | -0.14 | 0.30 | 5.25E-01 | -0.14 | 0.41 | 709917 | 143023 | 687518 | 169083 | 687202 | 176969 | 687834 | 162613 |
| RP_POS_589_22803a7_093 | RP | POS | 7.09 | 589.2280 | | | | 1.81E-02 | -0.40 | 1.08 | 9.86E-01 | 0.00 | 0.93 | 3.25E-02 | -0.40 | 1.04 | 5.89E-02 | -0.40 | 1.09 | 2055219 | 185408 | 1952570 | 270233 | 1952082 | 301921 | 1953058 | 237456 |
| HILIC_POS_264_75504a3_023 | HILIC | POS | 3.023 | 264.7550 | | | | 2.33E-04 | -0.52 | 1.33 | 9.86E-01 | 0.00 | 0.76 | 2.84E-03 | -0.52 | 1.42 | 6.24E-03 | -0.51 | 1.43 | 532398 | 107008 | 411968 | 254863 | 412420 | 269378 | 411517 | 242221 |
| RP_POS_152_05392a0_709 | RP | POS | 0.71 | 152.0539 | | | | 4.36E-01 | 0.10 | 0.42 | 9.86E-01 | 0.00 | 0.66 | 6.22E-01 | 0.10 | 0.28 | 4.57E-01 | 0.10 | 0.88 | 1069715 | 157101 | 1117996 | 552103 | 1117042 | 401937 | 1118950 | 674019 |
| RP_POS_181_07205a2_934 | RP | POS | 2.93 | 181.0721 | | | | 5.87E-01 | -0.11 | 0.32 | 9.86E-01 | 0.00 | 0.04 | 6.32E-01 | -0.11 | 0.24 | 6.27E-01 | -0.11 | 0.50 | 6278825 | 5930845 | 5610196 | 6319813 | 5621052 | 5982938 | 5599341 | 6700701 |
| RP_POS_255_23183a10_018 | RP | POS | 10.02 | 255.2318 | | | | 6.85E-02 | -0.37 | 0.77 | 9.87E-01 | 0.00 | 0.65 | 9.58E-02 | -0.37 | 0.72 | 1.09E-01 | -0.37 | 0.78 | 1977572 | 189624 | 1905399 | 195976 | 1905729 | 204257 | 1905068 | 189408 |
| RP_POS_226_21611a10_846 | RP | POS | 10.85 | 226.2161 | | | | 9.54E-06 | 0.81 | 1.29 | 9.87E-01 | 0.00 | 0.56 | 3.11E-04 | 0.81 | 1.25 | 1.87E-05 | 0.81 | 1.53 | 579140 | 245509 | 835952 | 313477 | 836472 | 248282 | 835432 | 369995 |
| RP_POS_814_76312a11_172 | RP | POS | 11.17 | 814.7631 | | | | 5.16E-07 | 0.74 | 1.52 | 9.87E-01 | 0.00 | 1.01 | 1.07E-04 | 0.73 | 1.49 | 3.94E-05 | 0.74 | 1.55 | 217785 | 104527 | 370352 | 218338 | 370709 | 210453 | 369996 | 228090 |
| HILIC_NEG_234_8203a3_136 | HILIC | NEG | 3.136 | 234.8203 | | | | 9.18E-05 | 0.48 | 1.14 | 9.87E-01 | 0.00 | 0.69 | 4.59E-03 | 0.48 | 1.29 | 5.31E-03 | 0.48 | 1.24 | 996727 | 354403 | 1770518 | 1805140 | 1767573 | 1824189 | 1773463 | 1804405 |
| RP_NEG_706_8064a0_497 | RP | NEG | 0.50 | 706.8064 | | | | 6.95E-05 | -0.52 | 1.26 | 9.87E-01 | 0.00 | 1.20 | 2.09E-03 | -0.52 | 1.32 | 4.38E-03 | -0.52 | 1.32 | 1361500 | 181578 | 1087202 | 585030 | 1088140 | 612559 | 1086264 | 562383 |
| RP_POS_413_17972a6_61 | RP | POS | 6.61 | 413.1797 | Unknown | 413.1807(100) | 4 | 1.93E-01 | -0.23 | 0.63 | 9.87E-01 | 0.00 | 0.55 | 2.64E-01 | -0.23 | 0.52 | 2.69E-01 | -0.23 | 0.65 | 864331 | 51572 | 849273 | 69552 | 849383 | 69649 | 849162 | 70161 |
| RP_POS_629_15143a0_619 | RP | POS | 0.62 | 629.1514 | | | | 6.67E-06 | -0.70 | 1.23 | 9.88E-01 | 0.00 | 0.96 | 1.73E-04 | -0.71 | 1.31 | 2.62E-04 | -0.70 | 1.27 | 418554 | 101383 | 297615 | 179286 | 297896 | 183659 | 297333 | 176670 |
| HILIC_POS_293_09732a7_451 | HILIC | POS | 7.451 | 293.0973 | | | | 2.26E-01 | -0.21 | 0.85 | 9.88E-01 | 0.00 | 0.59 | 2.65E-01 | -0.21 | 0.73 | 3.30E-01 | -0.22 | 1.00 | 1166017 | 175460 | 1118545 | 234177 | 1118179 | 266226 | 1118912 | 199802 |
| HILIC_NEG_381_00626a6_639 | HILIC | NEG | 6.639 | 381.0063 | | | | 5.32E-01 | -0.11 | 1.17 | 9.88E-01 | 0.00 | 0.54 | 5.60E-01 | -0.11 | 0.85 | 6.13E-01 | -0.11 | 1.20 | 746883 | 71346 | 737111 | 90803 | 736971 | 105195 | 737251 | 74782 |
| HILIC_NEG_157_86256a2_949 | HILIC | NEG | 2.949 | 157.8626 | | | | 6.35E-03 | 0.40 | 1.10 | 9.88E-01 | 0.00 | 0.79 | 2.84E-02 | 0.40 | 0.92 | 3.07E-02 | 0.41 | 1.13 | 1360654 | 271461 | 1562954 | 545640 | 1563786 | 556928 | 1562121 | 539771 |
| RP_POS_303_23175a10_704 | RP | POS | 10.70 | 303.2318 | | | | 3.12E-07 | 0.72 | 1.59 | 9.89E-01 | 0.00 | 0.50 | 7.11E-05 | 0.71 | 1.47 | 8.33E-05 | 0.72 | 1.54 | 1067691 | 575917 | 2000420 | 1389038 | 2002401 | 1408843 | 1998439 | 1383251 |
| HILIC_POS_295_06619a1_386 | HILIC | POS | 1.386 | 295.0662 | | | | 5.48E-11 | 0.80 | 1.38 | 9.89E-01 | 0.00 | 0.66 | 1.02E-05 | 0.80 | 1.39 | 1.20E-06 | 0.80 | 1.82 | 104858 | 227083 | 1829714 | 2324366 | 1832983 | 2198515 | 1826445 | 2466196 |
| HILIC_POS_129_06612a5_723 | HILIC | POS | 5.723 | 129.0661 | | | | 6.48E-02 | 0.33 | 0.65 | 9.89E-01 | 0.00 | 0.38 | 1.08E-01 | 0.33 | 0.57 | 1.13E-01 | 0.33 | 0.55 | 16187620 | 10767842 | 20610459 | 13872304 | 20590950 | 14013640 | 20629969 | 13871775 |
| RP_POS_1043_70276a10_618 | RP | POS | 10.62 | 1043.7028 | | | | 1.11E-03 | 0.61 | 1.44 | 9.89E-01 | 0.00 | 0.80 | 7.78E-03 | 0.61 | 1.20 | 1.58E-03 | 0.61 | 1.41 | 41265780 | 31352288 | 64185241 | 37643859 | 64133221 | 29346944 | 64237261 | 44741381 |
| RP_NEG_239_12886a8_233 | RP | NEG | 8.23 | 239.1289 | | | | 7.08E-01 | -0.06 | 0.53 | 9.89E-01 | 0.00 | 0.57 | 7.34E-01 | -0.06 | 0.55 | 7.68E-01 | -0.06 | 0.50 | 1088069 | 77290 | 1081371 | 114741 | 1081216 | 132198 | 1081527 | 95516 |
| HILIC_NEG_117_91909a5_606 | HILIC | NEG | 5.606 | 117.9191 | | | | 4.79E-01 | -0.14 | 0.83 | 9.89E-01 | 0.00 | 0.64 | 5.28E-01 | -0.14 | 0.26 | 5.27E-01 | -0.14 | 0.39 | 1526715 | 264213 | 1488252 | 271101 | 1488618 | 265412 | 1487885 | 279369 |
| RP_POS_379_21448a11_161 | RP | POS | 11.16 | 379.2145 | | | | 4.42E-01 | -0.13 | 0.71 | 9.89E-01 | 0.00 | 0.66 | 5.10E-01 | -0.13 | 0.53 | 5.21E-01 | -0.13 | 0.69 | 6546278 | 369163 | 6481315 | 528209 | 6480612 | 553326 | 6482017 | 507478 |
| RP_POS_816_58661a13_708 | RP | POS | 13.71 | 816.5866 | | | | 7.78E-07 | -1.02 | 1.60 | 9.90E-01 | 0.00 | 0.46 | 3.33E-06 | -1.02 | 1.64 | 5.30E-06 | -1.02 | 1.60 | 11970287 | 2799832 | 8776990 | 2823525 | 8780720 | 2931514 | 8773260 | 2741074 |
| RP_POS_163_03865a9_442 | RP | POS | 9.44 | 163.0387 | 4-hydroxycoumarin | 163.038(100) | 3 | 7.63E-01 | -0.07 | 0.33 | 9.90E-01 | 0.00 | 0.73 | 7.92E-01 | -0.06 | 0.60 | 7.75E-01 | -0.07 | 0.20 | 1375320 | 123498 | 1367928 | 108952 | 1367785 | 102422 | 1368071 | 116159 |
| RP_NEG_439_80557a15_501 | RP | NEG | 15.50 | 439.8056 | | | | 9.87E-01 | 0.00 | 0.55 | 9.90E-01 | 0.00 | 0.50 | 9.83E-01 | 0.00 | 0.32 | 9.93E-01 | 0.00 | 0.39 | 1364075 | 62929 | 1363866 | 60958 | 1363946 | 66374 | 1363786 | 55698 |
| HILIC_NEG_258_7644a2_945 | HILIC | NEG | 2.945 | 258.7644 | | | | 3.79E-06 | -0.59 | 1.33 | 9.90E-01 | 0.00 | 0.76 | 2.98E-04 | -0.59 | 1.47 | 1.30E-03 | -0.59 | 1.48 | 1816917 | 203237 | 1426757 | 723318 | 1425816 | 778455 | 1427698 | 671611 |
| HILIC_POS_297_07977a4_868 | HILIC | POS | 4.868 | 297.0798 | | | | 9.49E-01 | 0.01 | 0.32 | 9.90E-01 | 0.00 | 0.23 | 9.43E-01 | 0.01 | 0.47 | 9.63E-01 | 0.01 | 0.04 | 497452 | 568672 | 504836 | 560797 | 504144 | 715935 | 505528 | 350487 |
| RP_POS_805_54333a12_701 | RP | POS | 12.70 | 805.5433 | | | | 8.21E-02 | 0.33 | 0.66 | 9.90E-01 | 0.00 | 0.27 | 9.83E-02 | 0.33 | 0.81 | 1.53E-01 | 0.33 | 0.55 | 1087856 | 568249 | 1300055 | 656412 | 1299246 | 754146 | 1300863 | 549372 |
| HILIC_POS_387_21371a2_925 | HILIC | POS | 2.925 | 387.2137 | | | | 7.81E-01 | -0.05 | 0.90 | 9.90E-01 | 0.00 | 0.84 | 8.07E-01 | -0.05 | 0.43 | 8.12E-01 | -0.05 | 0.30 | 1510759 | 261112 | 1495158 | 316611 | 1495541 | 309500 | 1494775 | 326711 |
| RP_POS_499_82724a5_457 | RP | POS | 5.46 | 499.8272 | | | | 2.57E-01 | -0.25 | 1.43 | 9.90E-01 | 0.00 | 0.68 | 3.21E-01 | -0.24 | 1.20 | 2.81E-01 | -0.25 | 1.11 | 1752168 | 1537474 | 1402529 | 1385201 | 1400866 | 1233893 | 1404192 | 1534349 |
| RP_POS_293_20862a10_121 | RP | POS | 10.12 | 293.2086 | | | | 5.08E-02 | -0.32 | 0.91 | 9.91E-01 | 0.00 | 0.78 | 1.45E-01 | -0.32 | 0.77 | 6.18E-02 | -0.32 | 0.97 | 5629547 | 582872 | 5356305 | 915604 | 5355217 | 718168 | 5357393 | 1085358 |
| RP_POS_239_16162a8_616 | RP | POS | 8.62 | 239.1616 | | | | 2.54E-01 | 0.21 | 0.65 | 9.91E-01 | 0.00 | 0.52 | 3.41E-01 | 0.22 | 0.37 | 2.93E-01 | 0.21 | 0.63 | 4787997 | 594608 | 4933346 | 699811 | 4932548 | 614527 | 4934144 | 782191 |
| RP_POS_391_28409a11_573 | RP | POS | 11.57 | 391.2841 | | | | 5.10E-02 | -0.41 | 1.03 | 9.92E-01 | 0.00 | 0.51 | 7.66E-02 | -0.41 | 0.74 | 7.78E-02 | -0.41 | 1.08 | 3104452 | 228319 | 3012327 | 222887 | 3012088 | 226792 | 3012566 | 221216 |
| HILIC_POS_79_05497a0_505 | HILIC | POS | 0.505 | 79.0550 | | | | 5.25E-05 | -0.67 | 1.26 | 9.92E-01 | 0.00 | 0.74 | 4.30E-04 | -0.68 | 1.38 | 8.64E-04 | -0.67 | 1.33 | 582257 | 162323 | 420105 | 248386 | 420369 | 261093 | 419841 | 237657 |
| RP_POS_431_75708a13_369 | RP | POS | 13.37 | 431.7571 | | | | 1.25E-02 | 0.52 | 1.09 | 9.92E-01 | 0.00 | 0.25 | 2.12E-02 | 0.53 | 0.94 | 2.45E-02 | 0.52 | 0.96 | 1818533 | 765222 | 2217747 | 737645 | 2216967 | 763161 | 2218526 | 718978 |
| HILIC_POS_189_07352a4_923 | HILIC | POS | 4.923 | 189.0735 | | | | 7.64E-01 | -0.06 | 0.46 | 9.92E-01 | 0.00 | 0.64 | 7.80E-01 | -0.07 | 0.11 | 7.89E-01 | -0.06 | 0.29 | 7927622 | 3352268 | 7726243 | 3036228 | 7729330 | 3095597 | 7723156 | 3007117 |
| RP_NEG_373_00531a0_708 | RP | NEG | 0.71 | 373.0053 | | | | 1.83E-03 | -0.50 | 1.08 | 9.92E-01 | 0.00 | 1.03 | 1.02E-02 | -0.50 | 1.10 | 9.99E-03 | -0.50 | 1.13 | 1135725 | 292672 | 906094 | 493070 | 905609 | 496172 | 906578 | 494985 |
| HILIC_POS_499_0563a3_075 | HILIC | POS | 3.075 | 499.0563 | | | | 3.70E-04 | 0.50 | 1.21 | 9.93E-01 | 0.00 | 0.84 | 2.31E-03 | 0.50 | 1.33 | 1.08E-02 | 0.50 | 1.37 | 704019 | 60774 | 769449 | 142574 | 769314 | 158876 | 769583 | 125809 |
| HILIC_NEG_234_82027a7_68 | HILIC | NEG | 7.68 | 234.8203 | | | | 2.23E-01 | -0.25 | 0.59 | 9.93E-01 | 0.00 | 0.60 | 2.67E-01 | -0.25 | 0.60 | 2.80E-01 | -0.25 | 0.48 | 4875609 | 892148 | 4652275 | 890374 | 4653060 | 918507 | 4651491 | 870663 |
| RP_NEG_291_08362a0_622 | RP | NEG | 0.62 | 291.0836 | | | | 7.53E-06 | -0.64 | 1.27 | 9.93E-01 | 0.00 | 1.09 | 2.95E-04 | -0.64 | 1.34 | 6.51E-04 | -0.64 | 1.30 | 197133276 | 45555617 | 136336515 | 101595873 | 136422581 | 106089790 | 136250448 | 97974648 |
| HILIC_NEG_132_92365a7_354 | HILIC | NEG | 7.354 | 132.9237 | | | | 2.44E-01 | -0.18 | 0.68 | 9.93E-01 | 0.00 | 0.59 | 3.65E-01 | -0.18 | 0.30 | 3.16E-01 | -0.18 | 1.11 | 2745350 | 128678 | 2707927 | 223791 | 2707740 | 208961 | 2708114 | 239837 |
| RP_NEG_201_02246a5_964 | RP | NEG | 5.96 | 201.0225 | | | | 2.13E-01 | -0.44 | 0.99 | 9.94E-01 | 0.00 | 0.52 | 2.15E-01 | -0.44 | 0.76 | 2.13E-01 | -0.44 | 0.69 | 3425290 | 9137910 | 1364593 | 1251637 | 1363568 | 1071559 | 1365619 | 1420184 |
| RP_NEG_395_20816a9_14 | RP | NEG | 9.14 | 395.2082 | | | | 4.02E-07 | 0.64 | 1.18 | 9.94E-01 | 0.00 | 0.25 | 1.89E-04 | 0.64 | 1.27 | 2.81E-04 | 0.64 | 1.10 | 1118964 | 1534537 | 4735093 | 6191762 | 4730063 | 6315960 | 4740123 | 6129179 |
| RP_NEG_609_55774a15_218 | RP | NEG | 15.22 | 609.5577 | | | | 5.64E-01 | 0.11 | 0.40 | 9.94E-01 | 0.00 | 0.52 | 6.13E-01 | 0.11 | 0.25 | 6.17E-01 | 0.11 | 0.61 | 5300218 | 286861 | 5335621 | 340756 | 5335348 | 341162 | 5335894 | 343813 |
| RP_POS_214_91718a15_334 | RP | POS | 15.33 | 214.9172 | | | | 5.40E-01 | -0.12 | 0.37 | 9.94E-01 | 0.00 | 0.20 | 5.75E-01 | -0.12 | 0.35 | 6.05E-01 | -0.12 | 0.54 | 2062105 | 83860 | 2051207 | 96417 | 2051133 | 106504 | 2051281 | 86251 |
| HILIC_NEG_113_92621a2_939 | HILIC | NEG | 2.939 | 113.9262 | | | | 1.34E-01 | 0.22 | 0.64 | 9.94E-01 | 0.00 | 0.55 | 2.05E-01 | 0.22 | 0.43 | 2.62E-01 | 0.22 | 0.56 | 1054494 | 107587 | 1098422 | 220586 | 1098257 | 238487 | 1098586 | 203551 |
| HILIC_NEG_180_85939a2_98 | HILIC | NEG | 2.98 | 180.8594 | | | | 7.29E-01 | 0.06 | 0.31 | 9.94E-01 | 0.00 | 0.42 | 7.60E-01 | 0.06 | 0.34 | 7.67E-01 | 0.06 | 0.63 | 1117785 | 298905 | 1140155 | 365614 | 1140410 | 387676 | 1139901 | 346095 |
| HILIC_NEG_256_76682a2_946 | HILIC | NEG | 2.946 | 256.7668 | | | | 3.41E-06 | -0.61 | 1.34 | 9.95E-01 | 0.00 | 0.76 | 2.39E-04 | -0.61 | 1.47 | 1.03E-03 | -0.61 | 1.48 | 1259696 | 159680 | 982477 | 496447 | 982816 | 532021 | 982139 | 463576 |
| HILIC_POS_161_1284a6_821 | HILIC | POS | 6.821 | 161.1284 | | | | 5.14E-01 | 0.12 | 0.70 | 9.95E-01 | 0.00 | 0.38 | 5.73E-01 | 0.12 | 0.54 | 5.79E-01 | 0.12 | 0.58 | 6132524 | 4444728 | 6773631 | 5817380 | 6769806 | 5867690 | 6777456 | 5826204 |
| HILIC_NEG_520_67883a2_932 | HILIC | NEG | 2.932 | 520.6788 | | | | 1.25E-05 | -0.56 | 1.32 | 9.96E-01 | 0.00 | 0.76 | 6.56E-04 | -0.56 | 1.44 | 2.28E-03 | -0.56 | 1.48 | 220751 | 27814 | 170598 | 98781 | 170544 | 105737 | 170652 | 92381 |
| HILIC_POS_252_04523a2_995 | HILIC | POS | 2.995 | 252.0452 | | | | 4.82E-04 | -0.52 | 1.30 | 9.96E-01 | 0.00 | 0.75 | 4.71E-03 | -0.52 | 1.27 | 5.94E-03 | -0.52 | 1.34 | 2398993 | 393169 | 2020441 | 788491 | 2020871 | 806501 | 2020011 | 778254 |
| HILIC_POS_128_10715a1_104 | HILIC | POS | 1.104 | 128.1072 | | | | 8.68E-01 | -0.04 | 0.61 | 9.96E-01 | 0.00 | 0.95 | 8.75E-01 | -0.05 | 0.12 | 8.71E-01 | -0.04 | 0.13 | 513872 | 954497 | 484076 | 548327 | 484365 | 456883 | 483787 | 631441 |
| HILIC_NEG_100_93347a4_301 | HILIC | NEG | 4.301 | 100.9335 | | | | 3.96E-01 | 0.14 | 0.89 | 9.96E-01 | 0.00 | 0.76 | 4.87E-01 | 0.14 | 0.25 | 4.79E-01 | 0.14 | 0.45 | 29919318 | 2759487 | 30482830 | 4458222 | 30485039 | 4449948 | 30480621 | 4511659 |
| RP_POS_191_17972a8_904 | RP | POS | 8.90 | 191.1797 | | | | 8.43E-01 | 0.04 | 0.42 | 9.96E-01 | 0.00 | 0.41 | 8.64E-01 | 0.04 | 0.32 | 8.62E-01 | 0.04 | 0.37 | 760760 | 361981 | 775986 | 424323 | 776188 | 434603 | 775784 | 418205 |
| RP_POS_223_09419a0_672 | RP | POS | 0.67 | 223.0942 | | | | 5.22E-05 | 0.61 | 1.23 | 9.96E-01 | 0.00 | 1.08 | 1.29E-03 | 0.61 | 1.27 | 8.77E-04 | 0.62 | 1.26 | 1281810 | 320627 | 1632923 | 607492 | 1633211 | 595804 | 1632636 | 625014 |

**Supplementary table 1: Metabolomics results** (in asceding order according to p-value from progressive ALD vs  non-progressive ALD comparison, with molecular features with ID_level of 1-2 first)

| Feature_ID | RP_HILIC | POS_NEG | Average.Rt | Average.Mz | Metabolite.name | curated_MSMS | ID_level |
|---|---|---|---|---|---|---|---|
| RP_NEG_418_16086a11_615 | RP | NEG | 11.62 | 418.1609 | | | |
| RP_POS_310_2012a5_931 | RP | POS | 5.93 | 310.2012 | Unknown | 85.0292(100), 310.2007(45) | 4 |
| RP_POS_438_29779a10_706 | RP | POS | 10.71 | 438.2978 | | | |
| RP_POS_331_18817a9_072 | RP | POS | 9.07 | 331.1882 | | | |
| RP_POS_383_29657a11_608 | RP | POS | 11.61 | 383.2966 | | | |
| RP_POS_606_82281a0_498 | RP | POS | 0.50 | 606.8228 | | | |
| RP_POS_379_215a10_142 | RP | POS | 10.14 | 379.2150 | | | |
| RP_POS_756_55353a12_817 | RP | POS | 12.82 | 756.5535 | | | |
| HILIC_NEG_444_74869a2_929 | HILIC | NEG | 2.929 | 444.7487 | | | |
| HILIC_NEG_142_94432a1_687 | HILIC | NEG | 1.687 | 142.9443 | | | |
| RP_NEG_704_8075a0_498 | RP | NEG | 0.50 | 704.8075 | | | |
| RP_NEG_390_12875a11_16 | RP | NEG | 11.16 | 390.1288 | | | |
| HILIC_POS_501_05304a3_077 | HILIC | POS | 3.077 | 501.0530 | | | |
| RP_POS_166_98497a10_575 | RP | POS | 10.58 | 166.9850 | | | |
| RP_POS_674_81097a0_497 | RP | POS | 0.50 | 674.8110 | | | |

| | all_ALD_vs_control | | | Prog_ALD_vs_non-prog_ALD | | | Prog_ALD_vs_control_ALD | | | Non-prog_ALD_vs_control | | |
|---|---|---|---|---|---|---|---|---|---|---|---|---|
| | t-test | Cohen's | PLS-DA | t-test | Cohen's | PLS-DA | t-test | Cohen's | PLS-DA | t-test | Cohen's | PLS-DA |
| Feature_ID | p | d | VIP | p | d | VIP | p | d | VIP | p | d | VIP |
| RP_NEG_418_16086a11_615 | 4.78E-02 | 0.38 | 0.64 | 9.96E-01 | 0.00 | 0.38 | 8.93E-02 | 0.38 | 0.62 | 7.35E-02 | 0.38 | 0.65 |
| RP_POS_310_2012a5_931 | 5.64E-01 | -0.12 | 0.56 | 9.96E-01 | 0.00 | 0.25 | 6.13E-01 | -0.12 | 0.60 | 5.99E-01 | -0.12 | 0.26 |
| RP_POS_438_29779a10_706 | 2.18E-01 | 0.22 | 0.75 | 9.96E-01 | 0.00 | 0.98 | 2.87E-01 | 0.22 | 0.96 | 2.86E-01 | 0.22 | 0.67 |
| RP_POS_331_18817a9_072 | 2.85E-03 | -0.59 | 1.07 | 9.97E-01 | 0.00 | 0.33 | 8.40E-03 | -0.59 | 1.05 | 6.32E-03 | -0.59 | 1.02 |
| RP_POS_383_29657a11_608 | 5.70E-02 | -0.36 | 0.65 | 9.98E-01 | 0.00 | 0.39 | 7.62E-02 | -0.36 | 0.68 | 1.15E-01 | -0.36 | 0.53 |
| RP_POS_606_82281a0_498 | 1.16E-04 | -0.51 | 1.25 | 9.98E-01 | 0.00 | 1.18 | 2.54E-03 | -0.51 | 1.31 | 5.68E-03 | -0.51 | 1.31 |
| RP_POS_379_215a10_142 | 3.95E-01 | -0.24 | 0.95 | 9.99E-01 | 0.00 | 0.87 | 4.05E-01 | -0.24 | 0.52 | 4.17E-01 | -0.24 | 0.93 |
| RP_POS_756_55353a12_817 | 4.56E-02 | 0.35 | 0.78 | 9.99E-01 | 0.00 | 0.22 | 7.73E-02 | 0.35 | 0.70 | 9.52E-02 | 0.35 | 0.68 |
| HILIC_NEG_444_74869a2_929 | 3.18E-06 | -0.61 | 1.33 | 9.99E-01 | 0.00 | 0.76 | 2.22E-04 | -0.61 | 1.48 | 1.15E-03 | -0.61 | 1.48 |
| HILIC_NEG_142_94432a1_687 | 2.78E-02 | -0.46 | 1.04 | 9.99E-01 | 0.00 | 0.62 | 3.98E-02 | -0.46 | 0.85 | 5.31E-02 | -0.46 | 1.04 |
| RP_NEG_704_8075a0_498 | 2.12E-05 | -0.54 | 1.27 | 9.99E-01 | 0.00 | 1.21 | 1.14E-03 | -0.54 | 1.34 | 2.65E-03 | -0.54 | 1.33 |
| RP_NEG_390_12875a11_16 | 1.89E-02 | 0.45 | 0.91 | 9.99E-01 | 0.00 | 0.78 | 4.55E-02 | 0.45 | 0.82 | 3.14E-02 | 0.45 | 1.13 |
| HILIC_POS_501_05304a3_077 | 2.82E-04 | 0.50 | 1.18 | 1.00E+00 | 0.00 | 0.80 | 2.39E-03 | 0.50 | 1.28 | 9.53E-03 | 0.50 | 1.36 |
| RP_POS_166_98497a10_575 | 1.73E-01 | -0.28 | 0.51 | 1.00E+00 | 0.00 | 0.30 | 2.12E-01 | -0.28 | 0.59 | 2.24E-01 | -0.28 | 0.74 |
| RP_POS_674_81097a0_497 | 4.09E-07 | -0.62 | 1.33 | 1.00E+00 | 0.00 | 1.19 | 2.08E-04 | -0.62 | 1.42 | 4.31E-04 | -0.62 | 1.41 |

| | Healthy_control | | all_ALD | | Non-prog_ALD | | Progressive_ALD | |
|---|---|---|---|---|---|---|---|---|
| Feature_ID | Mean | SD | Mean | SD | Mean | SD | Mean | SD |
| RP_NEG_418_16086a11_615 | 557300 | 197411 | 641615 | 228934 | 641510 | 215657 | 641719 | 243686 |
| RP_POS_310_2012a5_931 | 2583146 | 1631462 | 2389660 | 1671431 | 2388911 | 1615891 | 2390409 | 1741629 |
| RP_POS_438_29779a10_706 | 3099329 | 1022873 | 3375737 | 1292022 | 3376309 | 1301495 | 3375165 | 1295692 |
| RP_POS_331_18817a9_072 | 1477890 | 95394 | 1416266 | 102707 | 1416225 | 98593 | 1416307 | 107666 |
| RP_POS_383_29657a11_608 | 1950296 | 311129 | 1821448 | 372717 | 1821565 | 418840 | 1821330 | 324412 |
| RP_POS_606_82281a0_498 | 1058003 | 153800 | 842070 | 470271 | 841947 | 497539 | 842192 | 446406 |
| RP_POS_379_215a10_142 | 5680454 | 7941850 | 4421770 | 4039662 | 4422529 | 4415488 | 4421010 | 3670727 |
| RP_POS_756_55353a12_817 | 47143237 | 25851907 | 58811415 | 34616901 | 58816871 | 36673786 | 58805960 | 32804705 |
| HILIC_NEG_444_74869a2_929 | 448170 | 56927 | 344608 | 187321 | 344586 | 202447 | 344630 | 172943 |
| HILIC_NEG_142_94432a1_687 | 871884 | 472408 | 655409 | 466173 | 655366 | 506638 | 655452 | 427069 |
| RP_NEG_704_8075a0_498 | 6157136 | 796139 | 4738919 | 2887713 | 4739157 | 3035859 | 4738681 | 2762519 |
| RP_NEG_390_12875a11_16 | 554444 | 51862 | 580931 | 60313 | 580936 | 55379 | 580926 | 65441 |
| HILIC_POS_501_05304a3_077 | 531090 | 42644 | 580210 | 107673 | 580213 | 118675 | 580207 | 96641 |
| RP_POS_166_98497a10_575 | 15020383 | 4269496 | 13830989 | 4127457 | 13831061 | 4288756 | 13830917 | 4003254 |
| RP_POS_674_81097a0_497 | 2277676 | 164208 | 1738320 | 960806 | 1738334 | 994581 | 1738305 | 935919 |
